# RNAseq analysis of heart tissue from mice treated with atenolol and isoproterenol reveals a reciprocal transcriptional response

**DOI:** 10.1186/s12864-016-3059-6

**Published:** 2016-09-07

**Authors:** Andrea Prunotto, Brian J. Stevenson, Corinne Berthonneche, Fanny Schüpfer, Jacques S. Beckmann, Fabienne Maurer, Sven Bergmann

**Affiliations:** 1 Department of Medical Genetics, University of Lausanne, Rue du Bugnon 27, 1011 Lausanne, Switzerland; 2 Swiss Institute of Bioinformatics, Lausanne, Switzerland; 3 Service of Medical Genetics, Centre Hospitalier Universitaire Vaudois and University of Lausanne, Rue du Bugnon 27, 1011 Lausanne, Switzerland; 4 Department of Integrative Biomedical Sciences, University of Cape Town, Cape Town, South Africa

**Keywords:** Mouse heart, *β*-adrenergic system, Cardiac hypertrophy, RNAseq, Atenolol, Isoproterenol

## Abstract

**Background:**

The transcriptional response to many widely used drugs and its modulation by genetic variability is poorly understood. Here we present an analysis of RNAseq profiles from heart tissue of 18 inbred mouse strains treated with the *β*-blocker atenolol (ATE) and the *β*-agonist isoproterenol (ISO).

**Results:**

Differential expression analyses revealed a large set of genes responding to ISO (*n* = 1770 at *FDR* = 0.0001) and a comparatively small one responding to ATE (*n* = 23 at *FDR* = 0.0001). At a less stringent definition of differential expression, the transcriptional responses to these two antagonistic drugs are reciprocal for many genes, with an overall anti-correlation of *r* = −0.3. This trend is also observed at the level of most individual strains even though the power to detect differential expression is significantly reduced. The inversely expressed gene sets are enriched with genes annotated for heart-related functions. Modular analysis revealed gene sets that exhibit coherent transcription profiles across some strains and/or treatments. Correlations between these modules and a broad spectrum of cardiovascular traits are stronger than expected by chance. This provides evidence for the overall importance of transcriptional regulation for these organismal responses and explicits links between co-expressed genes and the traits they are associated with. Gene set enrichment analysis of differentially expressed groups of genes pointed to pathways related to heart development and functionality.

**Conclusions:**

Our study provides new insights into the transcriptional response of the heart to perturbations of the *β*-adrenergic system, implicating several new genes that had not been associated to this system previously.

**Electronic supplementary material:**

The online version of this article (doi:10.1186/s12864-016-3059-6) contains supplementary material, which is available to authorized users.

## Background

The effects of many drugs are mediated by transcriptional changes. Variability in drug response can be due to different environment as well as distinct genetic and epigenetic backgrounds. Yet, how and to what extent these variables affect drug response is usually poorly understood (see [1–5] for reviews of recent advances [6]).

The *β*-adrenergic system exerts a tight physiological control on cardiac performance and contractility. However, when placed under conditions of exacerbated and sustained stimulation triggered by persisting cardiac insult or hypertension, its maladaptive response causes cardiac hypertrophy that, if not treated adequately, will ultimately lead to heart failure [7]. A range of *β*-blocking drugs, such as the *β*_1_-selective antagonist atenolol (ATE), have been developed to counter these effects. In the clinic, they are routinely prescribed for the management of heart failure, myocardial infarction, angina, atrial fibrillation and hypertension [8]. Conversely, long-term exposure of cellular or animal models to *β*-agonists such as the non-selective activator isoproterenol (ISO) can be used in the laboratory to mimic and study cardiac hypertrophy, independently of hypertension [9].

Owing to the broad diversity and heterogeneity of clinical presentations and interactions with environmental factors, the genetic etiologies of heart failure and response variability to *β*-blocking therapy remain poorly understood [10]. This complexity is reflected by the very small number of successful genome-wide association studies (GWASs) for heart failure and related phenotypes reported so far, despite meta-analyzing up to tens of thousands of patients [11–16].

Genetic reference populations (GRPs) such as those composed of panels of inbred mouse strains provide attractive alternatives to study complex traits. Not only are such panels genetically stable and environmentally controllable, but they also allow for longitudinal data acquisition, replication in multiple individuals or across laboratories and facilitated access to organs and tissues. Taking advantage of these principles, we recently investigated the organismal response of a population of 22 inbred mouse strains to ATE and ISO [17]. Our phenotypic dataset consisted of 27 highly heritable cardiovascular-related traits, each measured across the various strains and treatment conditions. Subsequently, we screened for genetic variants significantly correlated with these traits by genome-wide association mapping [18]. The most genome-wide significant hits included three candidate loci related to cardiac and body weight, three loci for electrocardiographic (ECG) values, two loci for the susceptibility of atrial weight to ISO, four loci for the susceptibility of systolic blood pressure to perturbations of the *β*-adrenergic system, and one locus for the responsiveness of the ECG QTc interval. These and about 100 additional marginally significant loci for cardiac-related traits were enriched in genes expressed in the heart.

In the present work, we have analyzed the cardiac expression patterns of 160 representative individuals of the same population of mice by RNA sequencing (RNAseq), considering 18 of the initial 22 strains. Our goal was to identify the transcriptional changes induced by ATE and ISO, study their modulation by the genetic background, and investigate their relation with the phenotypic traits measured earlier. We first examined the overall transcriptional response, pooling the data of control animals as well as those of mice treated with ISO and ATE, respectively. We analyzed the distributions of cross-sample correlations and performed principal component as well as clustering analyses. We then identified sets of genes that were either induced or repressed by each drug across all or most strains. Our key observation is that there was a strong enrichment for genes showing reciprocal responses to ATE and ISO, while not a single gene was significantly induced or repressed by both compounds. A finer modular analysis revealed transcription modules representing gene sets that exhibited coherent transcription profiles for some strains and/or treatments. Globally, module gene expression correlates with the phenotypic traits measured earlier [9]. Gene set enrichment analysis of differentially expressed groups of genes revealed pathways related to heart development and functionality. Our study provides a rich resource for the better understanding of how pharmacological perturbations of the *β*-adrenergic system affect the cardiac transcriptome and how these changes impact on heart-related traits within different mouse strains.

## Results

On average, 20 million of sequencing reads were obtained for each sample (Additional file 1: Figure S1). After filtering out 2896 genes with inconsistent evidence of expression (see Methods), read counts of the remaining 16397 genes were normalized with the trimmed mean of M-values (TMM) method [19]. We took a first global look at these data by computing all pairwise Pearson correlations C_ss’_ between samples and across genes. We assigned these correlations into four classes (Additional file 1: Figure S2): (1) 158 correlations between biological replicates (i.e. same strain and same treatment), (2) 474 correlations between samples of the same strains assessed under distinct treatments, (3) 4029 correlations between samples of different strains assessed under the same treatments and (4) 8059 correlations between samples of different strains assessed under distinct treatments. All cross correlations are relatively high (C_min_ = 0.929; Additional file 1: Figure S3), indicating that the expression of the vast majority of genes is neither affected significantly by treatment nor by genetic background. Nevertheless, the highest correlations are seen, as expected, between biological replicates with C_1_ = 0.970 ± 0.003 (mean ± SD), whereas the lowest correlations occur between unrelated samples (C_4_ = 0.950 ± 0.005). Intra-strain (C_2_ = 0.965 ± 0.005) and intra-treatment correlations (C_3_ = 0.955 ± 0.005) are distributed in between, yet the former are significantly higher than the latter (*t*_2,3_ ≈ 49, *p*_2,3_ < 3.9 × 10^−211^). This suggests that, overall, genetic differences between the various strains have a stronger impact on changes in gene expression than pharmacological treatments.

We next performed a cluster analysis of the inter-sample correlation matrix, reordering it in such a way that similar samples tend to be close to each other and using 1-C_ss’_ as similarity measure between the samples (Additional file 1: Figure S4). This clustering reflects the properties of the four categories of correlations described above: almost all samples clustered first by biological replicates and then predominantly by strain, rather than by treatment. Given the high intra-replicate correlations, we computed a new expression matrix averaging across replicates. This matrix had 54 conditions (1 control, 1 ISO and 1 ATE for each of the 18 strains). Clustering the corresponding cross-condition matrix revealed that conditions were grouped predominantly by strain, with the exception of few related strains (C57BL/6J and C57BLKS/J as well as BALB/cByJ and BALB/cJ) that clustered together under ISO (Additional file 1: Figures S5-S8).

**Fig. 1:**
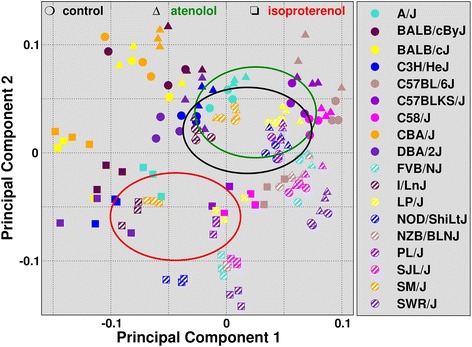
Principal component analysis of the TMM-normalized reads in the 160 samples. The loadings of the first two principal components are plotted against each other for each sample. The type of treatment is indicated by the shape of the markers (circles for CTR, triangles for ATE and squares for ISO), while the filling of the markers refers to the strains (see legend on the right). Biological replicates (same shape and filling) tend to cluster together. For any given strain the markers corresponding to CTR and ATE are usually not far apart, while those for ISO tend to be more distal with smaller loadings on both principal components. This effect is visualized by the ellipsoids, whose centers and major axes reflect the first two moments of the respective distributions (see legend on top)

As a second means to reveal the global structure of the expression data, we performed principal component analysis (PCA). Projection of the data onto the first two principal components reflected the aforementioned correlation structure, placing biological replicates, and to a lesser extent samples from the same strain, close to each other (Fig. 1). The PCA further showed that ISO samples tend to better segregate from the untreated controls (CTR) of the same strains than the ATE samples, revealing a stronger transcriptional response to ISO than ATE.

We next focused on gene expression changes induced by ISO or ATE. For this, we used the edge*R* software [20], delivering the average fold change (FC) and a significance value *p*_*FC*_ for the differential expression of each gene under the two drugs across all strains. At the *FDR* level of 0.0001, there are 1770 differentially expressed (DE) genes (958 up-regulated, 812 down-regulated) for the ISO-CTR comparison and 23 DE genes (12 up-regulated, 11 down-regulated) for the ATE-CTR one, in a total of 15966 genes surviving low-count filtering in both comparisons. The sizable difference in the number of DE genes across the two comparisons corroborates the higher transcriptional impact of ISO than ATE on the *β*-adrenergic system, indicated already by our PCA analysis. This observation holds well at various thresholds of significance (Table 1).

**Table 1:** Differentially expressed (DE) genes

Gene response	*FDR* < 5×10^−2^	*FDR* < 10^−2^	*FDR* < 10^−3^	*FDR* < 10^−4^
up-regulated by ISO (DE^ISO^_CTR_)	2501	1826	1289	958
down-regulated by ISO (DE_ISO_^CTR^)	2402	1669	1132	812
total for ISO	4903	3495	2421	1770
up-regulated by ATE (DE^ATE^_CTR_)	41	28	18	12
down-regulated by ATE (DE_ATE_^CTR^)	42	24	15	11
total for ATE	83	52	33	23

Number of DE genes obtained at four different *FDR* thresholds for samples pooled by treatment

The ATE treatment elicits FCs by a factor of at most four but lower than two for the vast majority of genes, while for ISO we observed a few genes with higher magnitudes. These differences are even more pronounced in terms of significance, with all but one gene with a *p*_*FC*_ > 10^−10^ for ATE, while a sizable number of them had much smaller *p*-values for ISO (Additional file 1: Figure S9a-b). As a consequence we found that a signed significance value, that we termed *Differential Expression Index* (DEI), was the most useful single measure to characterize DE across a range of significance values. The DEI is defined as the product of the sign of the log FC and the log of its *p*-value:

$$ DEI=\mathrm{sign}\left[ \log (FC)\right]\cdot \left[-{ \log}_{10}\left({p}_{FC}\right)\right] $$

It is strongly correlated to the average log FC itself (*r* ≈ 0.609 ± 0.010, *p* < 2.2 × 10^−308^ for ISO and *r* ≈ 0.608 ± 0.022, *p* < 2.2 × 10^−308^ for ATE, as computed at a confidence level of 95 %) but it can only be sizable if the FC is consistent across biological replicates (i.e. if there is statistical evidence for differential expression). This is expected as high FC reflects a substantial difference in read counts, which will lead to a small *p*-value as long as the FC is consistent across biological replicates (i.e. if there is statistical evidence for differential expression).

**Fig. 2:**
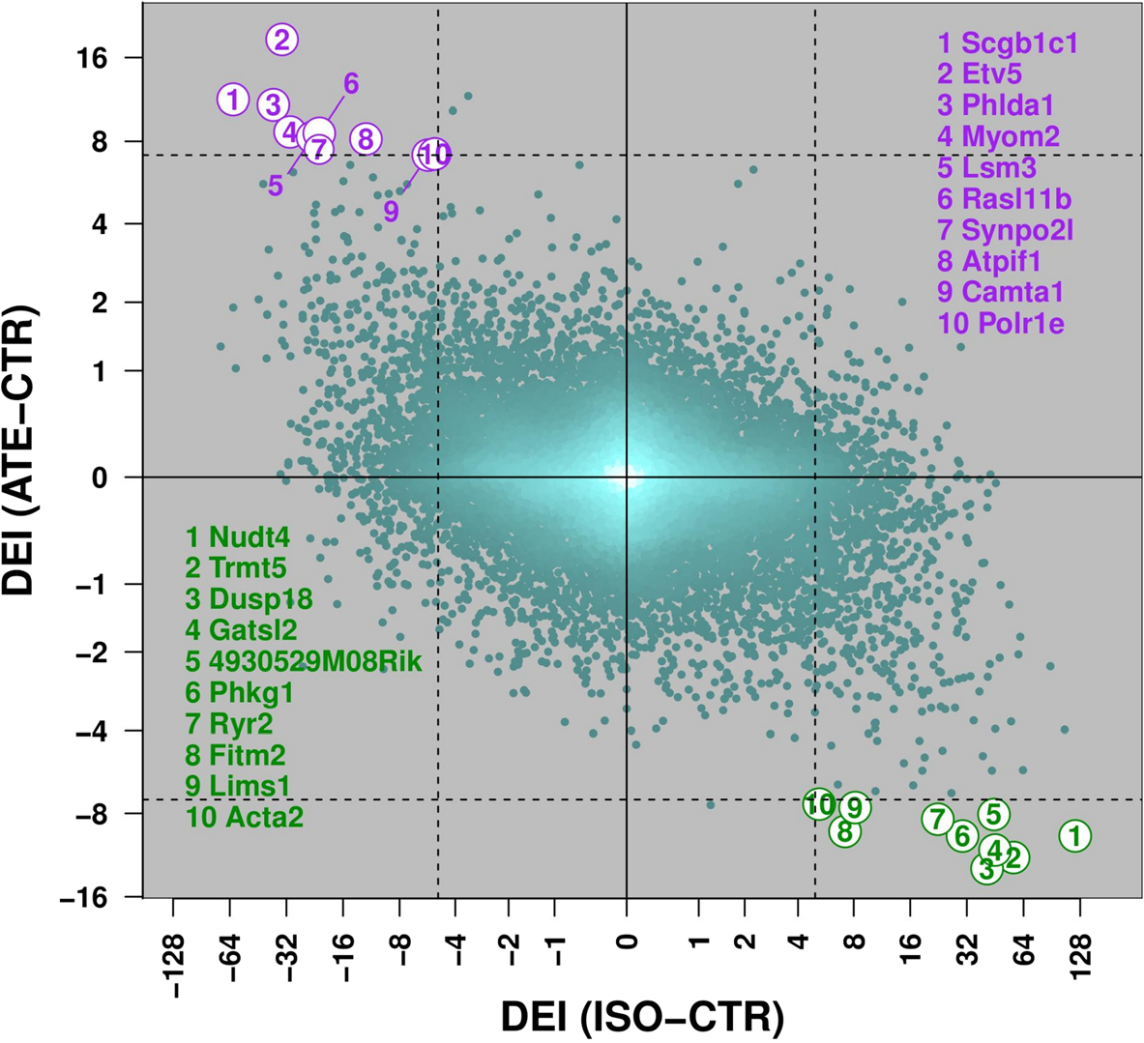
Uncorrected differential expression index (DEI) of the ISO-CTR comparison plotted against that of ATE-CTR. Each dot represents one of the 15966 genes for which it was possible to assign DEI values in both treatment groups. The color of the dots reflects the local gene density (the lighter the denser). The DEIs are anti-correlated (*r* = −0.3). The dashed lines at DEI = ±4.96 for ISO-CTR comparison and DEI = ±7.13 for ATE-CTR correspond to a (corrected) *FDR* of 0.0001. Genes passing both thresholds are observed in the upper-left and lower-right quadrants only, defining two sets of counter-expressed (CE) genes: CE_ISO_^ATE^ genes (shown in purple) and CE^ISO^_ATE_ (in green); the corresponding gene names are shown in the respective lists (10 genes each). The ordinal number preceding the CE genes reflects the gene rank according to the linear combination of the two DEI indexes. Strain-specific versions of this plot can be found in Additional file 1: Figures S20-S21

To compare differential expression under the two treatments, we plotted the DEI values for ISO against those for ATE (Fig. 2, Additional file 1: Figure S9c-d). These DEIs are anti-correlated (*r* ≈ −0.391 ± 0.013, *p* < 2.2 10^−308^), indicating that at least part of the transcriptional responses triggered by ISO and ATE is reciprocal. At a very stringent *FDR* of 0.0001 for Benjamini-Hochberg [21] corrected *p*-values, 10 genes are induced by ATE but repressed by ISO (we refer to these genes as to “counter-expressed” genes CE_ISO_^ATE^), whereas 10 genes are induced by ISO but repressed by ATE (CE^ISO^_ATE_), as highlighted in Fig. 2. The numbers of CE_ISO_^ATE^ and CE^ISO^_ATE_ genes as a function of different *FDR* thresholds are reported in Table 2. Strikingly, there is not a single “co-expressed” gene that is, up to a *FDR* threshold of 0.01, induced or repressed under both treatments (Fig. 2, Additional file 1: Figure S9). It is important to note that the observed anti-correlation between DEI(ATE-CTR) and DEI(ISO-CTR) is strongest for the genes whose DEIs are both significant, but that the trend persists even when considering larger gene sets where either or both DEI are not significant.

**Table 2:** Counter-expressed (CE) and co-expressed (CoE) genes

Gene response	*FDR* < 5×10^−2^	*FDR* < 10^−2^	*FDR* < 10^−3^	*FDR* < 10^−4^
up-regulated by ISO but down-regulated by ATE (CE^ISO^_ATE_)	34	22	14	10
down-regulated by ISO but up-regulated by ATE (CE_ISO_^ATE^)	34	24	15	10
total for counter-expressed (CE)	68	46	29	20
up-regulated by ISO and ATE (CoE^ISO ATE^)	2	0	0	0
down-regulated by ISO and ATE (CoE_ISO ATE_)	0	0	0	0
total for co-expressed (CoE)	2	0	0	0

The number of counter-expressed (CE) and co-expressed (CoE) genes obtained for samples pooled by treatment is presented at four different *FDR* thresholds. CE genes survive stringent *FDR* thresholds while the significance of the few co-expressed genes is much less robust

We verified that the human orthologs of the 20 CE genes are expressed in the heart (according to the MGI database [22], available at http://www.informatics.jax.org/). Most of these genes appear to be relevant in the context of prolonged cardiac excitation or blockade. Specifically, *Lims1* is required for normal development of cranial and cardiac neural crest-derived structures [23] and mice double knockout for *Lims1* and *2* develop dilated cardiomyopathy and die of heart failure within 4 weeks [24]. *Etv5* codes for a transcription factor involved in skeletal muscle acetylcholine-gated channel clustering at neuromuscular junctions; it belongs to the most abundant *Ets* transcripts in the early embryonic mouse heart [25] and gene targeting in zebrafish suggested a role in embryonic cardiac patterning [26]. *Myom2* codes for myomesin, the major component of myofibrillar M bands in vertebrates; human *MYOM2* mutations are associated with hypertrophic cardiomyopathy [27], while overexpression of EH-myomesin, the predominant *MYOM2* isoform in the embryonic heart, has been suggested as a biomarker of dilated cardiomyopathy [28]. *Synpo2l* encodes a cytoskeletal, heart-enriched, actin-associated protein; knock-down in zebrafish causes aberrant cardiac and skeletal muscle development and function [29], whereas human variants are associated with atrial fibrillation [30]. *Ryr2* encodes a ryanodine receptor found in the sarcoplasmic reticulum of cardiac muscle cells. This receptor is a component of a calcium channel that supplies calcium to cardiac muscle. Human *RYR2* mutations are associated with stress-induced polymorphic ventricular tachycardia [31–36] and arrhythmogenic right ventricular dysplasia [37]. *Acta2* codes for a skeletal muscle alpha actin. Defects in this gene lead to a diversity of vascular diseases, including aortic aneurysm [38], premature onset of coronary artery disease and premature ischemic strokes [39]. *Fitm2* codes for an endoplasmic reticulum protein involved in fat storage and homeostasis of cellular triglycerides; in skeletal muscle, it participates to the regulation of energy metabolism [40]. *Phkg1* codes for the broadly expressed phosphorylase kinase gamma, an enzymatic mediator of the neurohormonal regulation of glycogen breakdown; it is further involved in cellular pathways relevant to *β*-adrenergic stimulation such as c-AMP-dependent PKA signaling and Ca^2+^ signaling, and is high in muscle. *Camta1* codes for the calmodulin binding transcription activator 1, an apparent regulator of Ca^2+^-dependent adult stem cell commitment to myocardial lineage [41]. Finally, *Polr1e* codes for a component of the RNA polymerase I complex, that is active in the transcription of ribosomal RNAs and has been involved in the response to mechanical loading in a mouse model of muscle hypertrophy [42].

Reactivation of the fetal transcriptional program is a well-known hallmark feature of cardiac hypertrophy [9]. Therefore, it was expected that among the genes induced by ISO, a subset would relate to early cardiac development. What is interesting here is that even in the absence of heart failure, at least some of these genes can be down-regulated by ATE, suggesting that up to some extent, *β*-adrenergic blockade may not only block but also reverse the process of cardiac remodeling triggered by cardiac insult.

In line with the above, Gene Ontology (GO) analyses of the CE gene sets with DAVID [43] indicate that CE_ISO_^ATE^ set is enriched for genes related to “calmodulin binding”, whereas CE^ISO^_ATE_ genes significantly overlap with the more general classes of “calcium signalling” and “contractile fiber”. Analysis of the combined set further points to “muscle contraction” and related terms, which are all essential features of cardiac function (Additional file 2: Table S1). Owing to the small number of genes taken into consideration, these enrichments are not highly significant.

Relaxing the *FDR* threshold gives rise to larger gene sets but these are enriched for many Gene Ontologies that mostly refer to very general categories (data not shown). We therefore decided to investigate further the sets of CE genes using GOSeq [44] and scanning over several thresholds. Retaining only annotation terms that were enriched across a range of threshold (see Methods and Additional file 3) yielded many terms specific of heart-related features. This is particularly the case for the subset of CE^ISO^_ATE_ genes (Additional file 1: Figures S10-S17), that appears strongly associated to cardiac muscle action potential, contraction and conduction, as well as for the combined list of CE^ISO^_ATE_ and CE_ISO_^ATE^ genes (data not shown). The subset of CE_ISO_^ATE^ genes is overrepresented in more general cellular processes of proliferation and division, although several cellular component (CC) categories include heart-related terms, such as M-band, contractile fiber part and myofibril.

When pooling the data across all strains to compare treated against control samples we achieved excellent power to detect DE genes. In contrast, using edge*R* to compute a strain-specific differential expression index DEI(*s*) by comparing just the three ISO or ATE replicates of a given strain *s* against the corresponding CTR samples, power was significantly reduced. This is reflected by the much lower count of DE genes detected in each strain when compared to the figures discussed above (compare Table 1 with Additional file 2: Table S2). In particular, for many strains we could only detect DE genes under ATE treatment at a relaxed *FDR* and none at all in strains BALB/cByJ, BALB/cJ, I/LnJ and LP/J, even at a *FDR* of 0.05. At the level of individual strains, NODShiLt/J mice are by far the strongest transcriptional responders to ISO, while C57BL6/J animals are the most responsive to ATE (Additional file 2: Table S2 and Additional file 1: Figures S18-S19).

Decreased power is the likely reason why for most strains we did not observe any significant anti-correlation between the DEI(*s*) values for ISO and ATE (Additional file 1: Figures S20-S21). We therefore searched for a more sensitive measure of counter-expression by restricting our analyses to the ISO-responsive genes (since ISO affects many more genes than ATE). We thus defined a *Counter-Expression Index* (CEI):

$$ CEI(t)=\frac{1}{\left|G(t)\right|}{\sum}_{g\in G(t)}z\left({DEI}^{ISO-CTR}\left(\mathsf{g}\right)\right)\cdot z\left({DEI}^{ATE-CTR}\left(\mathsf{g}\right)\right), $$

where *z*(…) indicates a *z*-score normalization with respect to G(*t*) which is the set of genes for which |DEI^ISO-CTR^| = −log_10_(*p*^ISO-CTR^) > *t* and |G(*t*)| is its size. So CEI(*t*) is nothing more than the correlation between two DEIs after removing non-significant genes (under ISO). Using a threshold *t* = 4 we found that 16 out of 18 strains obtained a negative CEI and that this trend changes only at a very low level of significance for DE genes (Additional file 1: Figures S22-S25). Nevertheless, although this confirms that anti-correlated expression between the two treatments is present in the large majority of strains, the individual signals are quite weak (Additional file 1: Figures S22-S25).

To evaluate the extent by which drug-induced expression changes are strain-specific and whether strain specificity differs between treatments, we introduced a *Strain-Specificity Index* (SSI). This index is computed from the strain-specific DEI(*s*). It is defined by analogy with the tissue specificity index introduced in [45] as follows:

$$ SSI=\frac{\sum_{s=1}^{N_s}1-{x}_s}{N_s-1}\mathrm{with}{x}_s=\frac{\left|DEI(s)\right|}{max_{s=1}^{N_s}\left|DEI(s)\right|} $$

The SSI of a given gene and treatment vanishes if its DEI values are identical across all strains and it is small if they are similar. In contrast, the SSI equals one if the gene is differentially expressed in a single strain and is close to one if its expression is specific to relatively few strains. Intermediate values indicate partial strain specificity. Note that the absolute value of the DEI is used, so only the significance but not the direction of the differential expression is relevant here.

**Fig. 3:**
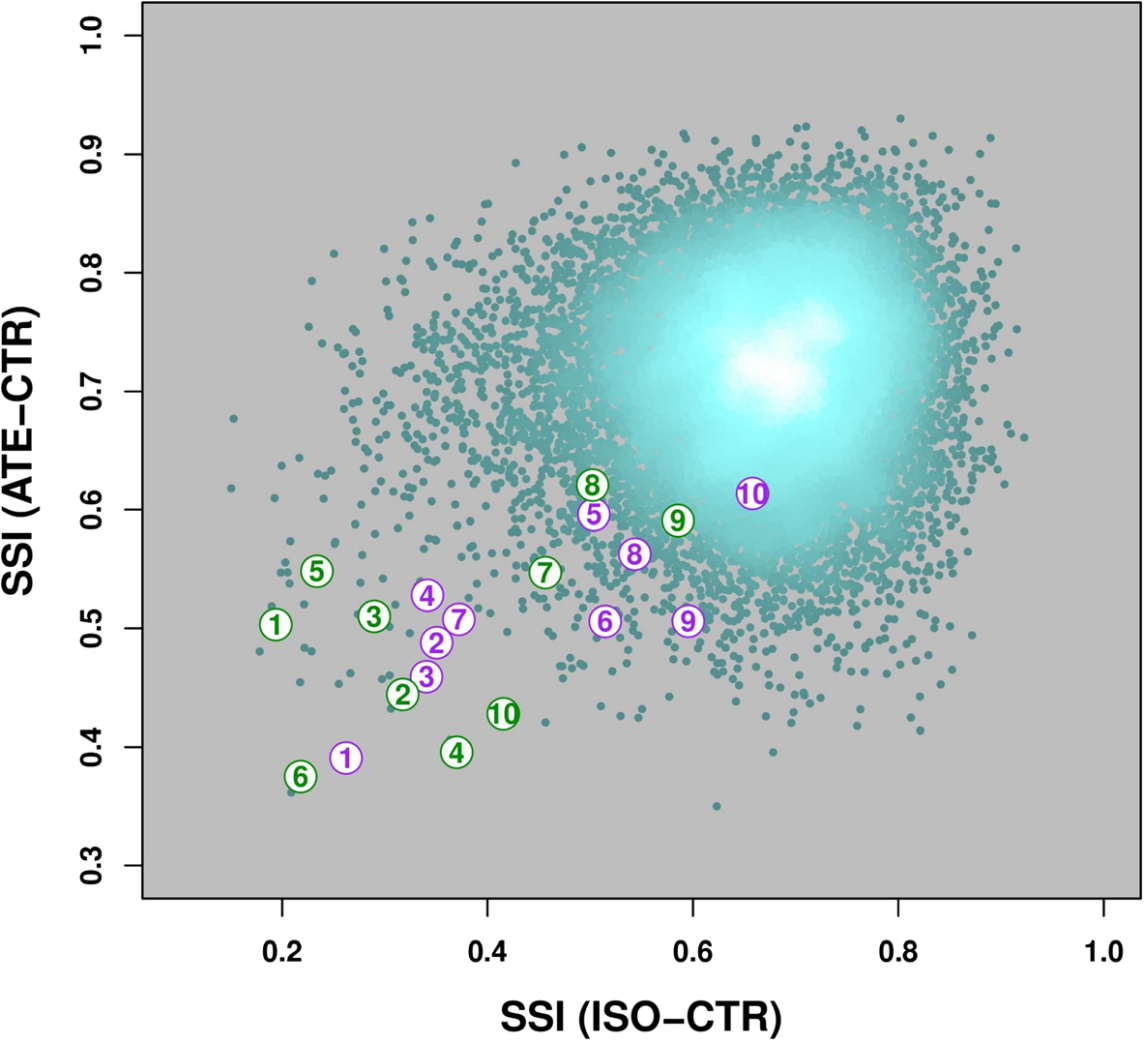
Strain-specificity index (SSI) of the ISO-CTR comparison plotted against that of ATE-CTR. Each dot represents one of the 14537 genes for which it was possible to assign SSI values (i.e. the genes were not filtered out in at least 12 out of the 18 available strains in both the ISO-CTR and the ATE-CTR comparisons). The color of the dots reflects the local gene density (the lighter the denser). The SSIs are slightly correlated with each other (*r* ≈ 0.007, *p* ≈ 9.0 × 10^−21^). The two groups of CE genes, CE_ISO_^ATE^ (in purple) and group CE^ISO^_ATE_ (in green) have relatively low SSIs compared to the bulk of genes and their SSIs are strongly correlated with each other (CE_ISO_^ATE^: *r* ≈ 0.76, *p* ≈ 0.01; CE^ISO^_ATE_: *r* ≈ 0.51, *p* ≈ 0.13). Thus, the expression of genes playing a key role at the transcriptional level appear to be less influenced by the strain (lower SSI than the bulk genes) and the treatment (CE genes fall closer to the diagonal than bulk genes) with respect to the other genes

Plotting the SSI values of ATE-CTR versus those of ISO-CTR (Fig. 3) revealed that most genes exhibit relatively high strain-specificity (the mean ± SD of the respective distributions are 0.71 ± 0.31 for ISO and 0.72 ± 0.32 for ATE, respectively). Also, we observed a very mild global correlation (*r* ≈ 0.078, *p* < 9.0 × 10^−21^), so for the vast majority of genes any SSI value in one treatment is uninformative of its value in the other. This is not unexpected, since most genes are not affected significantly by either treatment. We therefore asked whether CE genes behave differently. At a stringent *FDR* of 0.0001, the respective correlation coefficients (CE_ISO_^ATE^: *r* ≈ 0.76, *p* < 0.01; CE^ISO^_ATE_: *r* ≈ 0.51, *p* < 0.13) are much higher, but not very significant. Yet, genes of both CE_ISO_^ATE^ and CE^ISO^_ATE_ sets tend to have a significantly lower SSI(ISO) (*t* ≈ 5.4, *p* < 0.0004 for CE_ISO_^ATE^ and *t* ≈ 9.6, *p* < 5.1 × 10^−6^ for CE^ISO^_ATE_) and SSI(ATE) (*t* ≈ 7.6, *p* < 3.2 × 10^−5^ for CE_ISO_^ATE^ and *t* ≈ 8.2, *p* < 1.7 × 10^−5^ for CE^ISO^_ATE_). The combined set of CE_ISO_^ATE^ and CE^ISO^_ATE_ genes shows an intermediate correlation (*r* ≈ 0.60, *p* < 0.0047). The shift of the SSI(ISO) distribution towards lower values (i.e. less specificity) is reflected by a *t*-statistic *t* ≈ 8.9, *p* < 3.3 × 10^−8^ while that of the SSI(ATE) has *t* ≈ 10.4, *p* < 2.5 × 10^−9^. These correlations refer to a definition of DE genes at a *FDR* < 0.0001. They hold at less stringent thresholds as well (data not shown). Taken together, these results show that the CE genes are significantly less strain-specific than the other genes.

In the paragraphs above, we saw that the transcriptional response of individual genes under specific environmental conditions and/or genetic backgrounds is often quite weak and can be difficult to detect due to the overall noise when using only a few biological replicates. The power to detect strain-specific regulation can be increased in principle by integrating signals from multiple genes which are expressed differentially only across subsets of strains and a number of methods have been proposed for the unsupervised identification of such subsets. Here we used the *Iterative Signature Algorithm* (ISA) [46, 47], which identifies transcription modules consisting of subsets of genes that are either coherently induced or repressed over some, but usually not all samples. We have previously used the ISA to reveal the modular structure of transcriptomes, extracting robust modular signals [47–52]. One of the advantages of the ISA is that the search for modules can be biased towards modules with a particular signature (i.e. a preselected set of “seed” samples, see [52]).

We applied the ISA to the entire set of the 160 RNAseq profiles using random selections of strains to define the seed scores such that all selected strains were assigned a positive score for ISO and a negative score for ATE, or vice-versa. CTR samples were not included as seeds, but we observed that the ISA often added those samples in the final modules, usually with scores of the same sign as ATE. After filtering out the highly similar ones, the ISA revealed 98 transcription modules involving various subsets of genes that are CE in some but not all of the strains. Our attempt to annotate these modules using standard gene enrichment analysis for GO categories and KEGG pathways revealed a number of highly significant but rather general terms: “immune response” (*p* ≈ 1.5 × 10^−12^) for Biological Process (BP); “proteinaceous extracellular matrix” (*p* ≈ 9.0 × 10^−4^) for Cellular Component (CC), and “tetrapyrrole binding” (*p* ≈ 3.8 × 10^−5^) for Molecular Function (MF). Amongst the KEGG pathways, “PPAR signaling pathway” (*p* ≈ 5.5 × 10^−4^) received the smallest *p*-value (see Additional file 2: Table S3 for all significant GO/KEGG terms). The complete annotation of the 98 modules in terms of genes, strains and GO or KEGG terms is available in HTML format as part of our Additional file 4. This compendium of modules provides a rich resource of co-expression, allowing researchers to investigate their strain or gene of interest and study which other genes or strains responded similarly to the drugs.

**Fig. 4:**
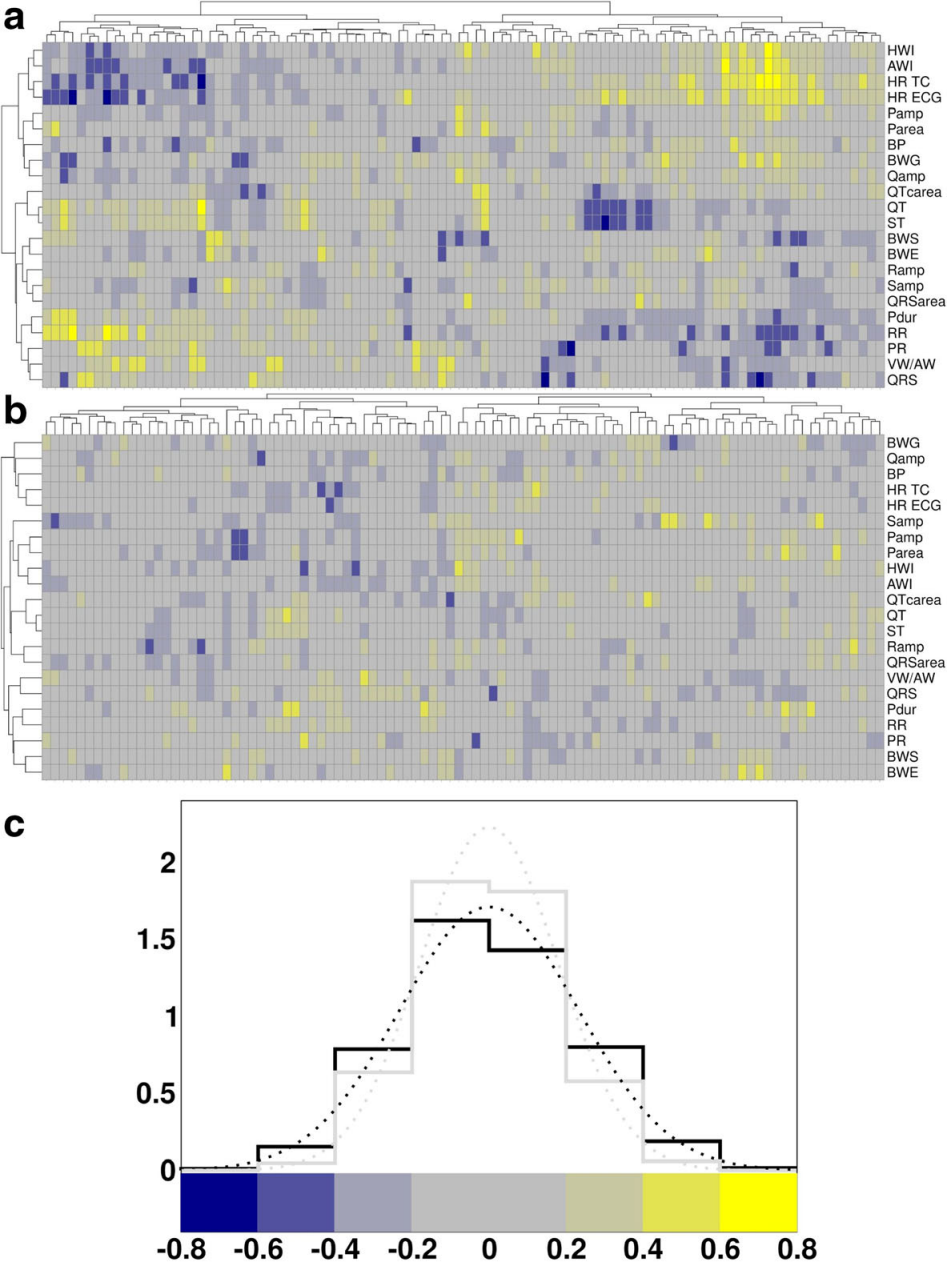
Module-trait correlations are larger than those of randomized controls. **a** Bi-clustered module-phenotype correlation matrix for the real data and (**b**) reshuffled data. The color code and distributions for these correlations are shown in (**c**). The two distributions are significantly different from each other (*F* ≈ 0.52, *p* ≈ 3.3 × 10^−51^) with heavier tails for the real data (Additional file 1: Figure S26). The phenotypes are described in Additional file 2: Table S4 [17]

We next asked whether module gene expression (i.e. the average expression of all genes associated with a module) is correlated with any of the organismal traits that had previously been measured in these mice [17] (Additional file 2: Table S4). Figure 4 shows that indeed on average module-trait correlations tend to be significantly larger than those of randomized controls (Additional file 1: Figure S26 and Methods). Importantly, different modules correlate with different traits, indicating that there might be specific aspects of the transcriptional program that mediate the different organismal responses (Additional file 2: Tables S5-S8).

As the standard enrichment analysis did not reveal very specific terms we sought to further scrutinize a subset of modules with strong (anti-) correlation to any of the phenotypic traits by applying the scanning approach we already used to recover more specific GO terms in relation with the CE genes analysis. Specifically, we progressively excluded genes with low gene scores from the evaluation of the GO term significance (see Methods).

The full list of the GO categories enriched in the core genes of the modules showing highest (anti-) correlation with the phenotypic traits is provided in the supplement. A comprehensive analysis of these results is a major task and we hope that making our transcription modules available to the community will shed new light on genes whose differential expression is in concert with known genes. Here we focus on the results related to heart rate (HR), which was found to be the trait with the strongest (anti-) correlation to a transcriptional module. Additional file 1: Figures S27-S30 summarize the GO analysis performed on the 73 genes of module 38, which was strongly anti-correlated to HR measured by tail-cuff (TC), whereas Additional file 1: Figures S31-S34 show the GO analysis performed on the 211 genes of module 56, whose average expression was strongly correlated to HR measured by electrocardiography (ECG).

Module 38 was the most anti-correlated to HR, yet its ISO samples had predominantly negative scores. Conversely module 56 was correlated to HR, but its ISO samples had positive scores. Thus, in fact the genes of both modules were induced by ISO treatment. Inspection of the core genes of module 38 (Additional file 1: Figures S27-S30) revealed, at the BP level, a very significant enrichment with “Golgi-apparatus” related GO terms, both regarding the number of inherent terms and their significance. Among the significant terms, albeit only for a specific gene threshold, is also “muscle system process”. At the CC level we found 8 out of 19 terms related to the rather unspecific term “organelle”. At the MF level, the highest significance and stability is reached by the term “kinase activity”. The KEGG terms most enriched in this module are dominated by the “fatty acid elongation”. The 211 genes of module 56 are significantly enriched in the BP GO terms “fibroblast” and “cytokine activity”, as well as “fibril/extracellular fibril organization” (Additional file 1: Figure S31). Among the CC terms associated to this module “mitochondrial/respiratory chain” and “fibril components” are among the most significant (Additional file 1: Figure S32). The most significant MF terms are dominated by “transmembrane” (Additional file 1: Figure S33), whereas at the level of KEGG pathways “TGF-beta signaling”, “vascular smooth muscle contraction” and “vasopressin-regulated reabsorption” are among the most strongly enriched pathways associated to the HR TC (Additional file 1: Figure S34). This suggests that this module might be linked to fibrosis, a well-described process that may occur as a result of sustained (ISO-induced) cardiac stimulation.

Our modular analysis generated a compendium of transcriptional modules that differ with respect to their gene and/or strain and/or treatment sets. Yet, distinct gene sets may still share the same functional annotation, so we asked to what extent similarity in gene content goes along similarity in annotation. We used the Jaccard indices S^G^_ij_ and S^A^_ij_ to quantify the relative overlap in genes and annotation terms for any pair of modules (i, j), respectively (Methods). Plotting one against the other (Additional file 1: Figure S35) revealed that these quantities are not very strongly correlated. In particular, some pairs of modules may overlap only mildly with respect to genes (say S^G^_ij_ < 0.3), but have a sizable functional overlap (S^A^_ij_ > 0.5).

In order to further reduce functional complexity and identify the major functional classes of our modules we clustered them according to S^A^_ij_ (Additional file 1: Figures S36-S39). This clustering revealed that the modules segregate into several blocks of functionally highly similar profiles, while the blocks are essentially functionally disjoint. We concentrated on blocks which contain at least 5 modules with high functional coherence, referred as to “macro-modules” in the following. For each of these macro-modules we selected one module (the one with the highest global annotation significance and broadest gene score range) as the representative module of the functional block (Additional file 2: Table S9). Here we highlight three representative modules which had strong enrichment in any of the annotation categories. The three macro-modules were analyzed at the level of BP, CC, MF and KEGG pathways and, for each of these categories, they can be listed (according to the prominently significant categories) as: “fibroblast”, “antigen” and “cardiac” macro-modules for BP (Additional file 1: Figures S40-S42); “cytoskeleton”, “MHC-complex” and “organelle” for (Additional file 1: Figures S43-S45); “ribonuclease”, “hormone” and “pyrimidine” for MF (Additional file 1: Figures S46-S48). Concerning the KEGG pathways, there are only two shared macro-modules, which can be named as “graft” and “kinase” (Additional file 1: Figures S49-S51). However, intermediate macro-modules (i.e. macro-modules with lower similarity among the inner modules) contain “tRNA”, “sodium-potassium channel” and “Golgi-apparatus” terms (data not shown). The complete documentation about the macro-modules is available as part of the supplement.

## Discussion

Treatments with the *β*-blocker atenolol (ATE) and the *β*-agonist isoproterenol (ISO) induce inverse physiological responses, notably heart rate is increased by ISO but reduced by ATE. In our initial study we also observed that while ISO induced cardiac growth, mice treated with ATE often had smaller hearts [17]. How these reciprocal effects are mediated has been poorly understood so far. Our analysis of RNAseq data from heart tissue within a broad panel of genetically diverse mice sheds new light at the transcriptional response induced by these drugs.

First, the treatments altered the expression of many genes for ISO and to much less extent for ATE (Table 1). The stronger transcriptional response detected under ISO reflects what we observed at the physiological level [17], where ISO elicited a stronger effect than ATE on many traits. It should be noted that while the effective drug concentrations cannot be readily compared between ATE and ISO, ATE was used at a dose close to its upper limit of solubility (see Methods).

Second, the transcriptional responses upon treatment with the two drugs are not independent. Remarkably, for the affected genes, and in particular for those whose transcript levels changed most consistently, we observed a reciprocal effect: most genes that are significantly (*FDR* < 0.05) induced in mice treated with ATE are repressed under treatment with ISO, and at the slightly more stringent *FDR* of 0.01 we did not observe a single co-induced or co-repressed gene. This counter-expression is a remarkable result hinting that some processes responsible for cardiac health may be operational at some base level from which they may either be enhanced or repressed, depending on the sensed level of stress. The 20 genes for which the counter-expression was most significant are good candidates for playing important roles in these processes and should be studied further. Importantly, the transcriptional response of the large majority of the CE genes is much less strain-specific than that of most genes, indicating that the processes they are involved in are robustly regulated across strains. However, our modular analysis showed as well that the ISO treatment exerts strain-specific cardiac transcriptional signatures and phenotypic traits. These results are in line with independent evidence showing that some aspects of cardiac remodeling in ISO-induced cardiac dysfunction are strain dependent [53, 54].

It is difficult to annotate these processes in terms of known gene categories. Enrichment analysis of the most significant counter-expressed genes pointed at  “calcium signaling” and “regulation of contractile fiber”, both of which make sense in the context of drugs affecting the *β*-adrenergic system. While this manuscript was in preparation, Rau et al. published an investigation that aimed at mapping genetic contributions to cardiac pathology induced by longer and more pronounced ISO stimulation in female individuals of the hybrid mouse diversity panel (HMDP) [54]. This work studied similar traits as ours but did not address *β*-blockade. Interestingly their analysis of differentially expressed genes, as well as genetic variations, also pointed at genes involved in calcium signaling as important players for cardiomyopathy. Including genes with less significantly altered expression pointed at many, but often rather unspecific categories. It thus seems that the observed changes in gene expression reflect not only the primary transcriptional response, but very likely many secondary effects. This is also reflected in our modular analysis which revealed counter-expressed genes over subsets of strains. One category that is frequently showing up with high significance is “immune response”, which may indicate a general stress response in mice treated with ISO. Also the link to ‘tetrapyrrole binding’ is interesting, since this molecular process is relevant for hemoglobin expression which has been linked to ISO treatment [55].

It is beyond the scope of this paper to investigate in detail the various links generated by our analysis. We hope that with the availability of the raw expression data and our module database our work will provide a valuable resource for experts follow up on these leads. We are also aware that the modular links which are specific to only some species call for further genetic investigations with the aim to identify what might be common in these strains that elicit a similar response. For example our expression data would allow for detecting potential quantitative trait loci for expression (eQTLs) which would be natural candidates. These and further integrated analysis combining the various molecular data and organismal traits will be the subject of further investigations.

## Conclusions

We showed that the inverse physiological responses of mice treated with the antagonistic drugs atenolol and isoproterenol are reflected by a reciprocal transcriptional response of many genes expressed in the heart. Our modular gene expression analysis grouped genes together which exhibit coherent transcription profiles across some strains and/or treatments. Our study provides new insights into the transcriptional response of the heart to perturbations of the *β*-adrenergic system, implicating several new genes that had not been associated to this system previously.

## Methods

### Mice

Animal procedures have been published earlier [17]. Briefly, atenolol (ATE) and isoproterenol (ISO) were administered at 10 mg/kg per day for two weeks through surgically-implanted osmotic minipumps. In accordance with reports showing their effectiveness on mouse heart rate and, at least in some of the strains, normalized heart weight [17], these doses were set so as to trigger as large phenotypic changes as possible while keeping adverse events minimal. More specifically, higher concentrations of ISO induced a number of fatalities, a trend confirmed by the recent work of Rau et al., who reported ca 30 % early death in representatives of 105 inbred mouse strains treated with 30 mg/kg per day of ISO for 3 weeks [54]. In contrast, phenotypic changes induced by ATE below 10 mg/kg per day were hardly detectable when compared to untreated mice, while testing higher concentrations was impractical due to the limited solubility of ATE in water or any vehicle compatible with physiological conditions and delivery through osmotic minipumps for 2 weeks. At the end of the respective treatments, mice were euthanized and hearts were rapidly excised, rinsed in ice-cold phosphate buffered saline (PBS) solution and blotted dry. Cardiac atria and ventricles were dissected, frozen separately in liquid nitrogen and stored at −80 °C. Mouse ID numbers refer to those described in [17]. Corresponding individual phenotypic values, in particular heart rate, systolic blood pressure, electrocardiogaphic measurements and heart weight are available in dataset “maurer1” of the Mouse Phenome Database (http://phenome.jax.org/).

### RNA purification

Total RNA was purified from the cardiac ventricles of untreated (CTR), ATE, and ISO mice of 18 inbred strains (ie A/J, BALB/cByJ, BALB/cJ, C3H/HeJ, C57BL/6J, C57BLKS/J, C58J, CBA/J, DBA/2 J, FVB/NJ, I/LnJ, LP/J, NOD/ShiLtJ, NZB/BLNJ, PL/J, SJL/J, SM/J and SWR/J) following the recommendations of Ambion for the semi-automated MagMax procedure, then stored at −80 °C. RNA quality and integrity (RIN > 8.1) were verified on a Bioanalyzer (Agilent).

### RNA sequencing

Library preparation and RNA sequencing were performed by the Beijing Genomics Institute (BGI, Hong Kong, China), following Illumina’s protocols for poly(A) selection and HiSeq2000 paired-end sequencing (2 × 100 bp). Except for strains BALB/cJ (two CTR samples) and C57BLKS/J (two ISO samples), we had measurements of three biological replicates for each combination of strains and treatments; all data passed quality control. An average of 20 ± 0.8 millions reads were produced for each sample, in accordance with standard requirements for minimal sequencing coverage [56, 57]. The sequencing reads were mapped to a total of 19293 genes using the NCBI37/mm9 *Mus musculus* reference genome.

### Bioinformatics tools

We used the edge*R* Bioconductor software package [20], version 3.2.4, to assess differential gene expression using default parameters. edge*R* is designed to work with count data providing the appropriate statistical framework for digital gene expression data. Specifically, it is based on empirical Bayes estimation and exact tests founded on the negative binomial distribution. Read counts were normalized with the trimmed mean of M-values (TMM) method [19].

### Differential expression (DE) and Gene Ontology (GO) analyses

For each comparison (ISO versus CTR, ATE versus CTR), we filtered out low-count genes, so defined when showing less than 5 reads across all study samples. 16209 genes survived filtering for the ISO-CTR comparison and 16156 for the ATE-CTR one. For CE and CoE we always considered as background a total of 15966 genes surviving low-count filtering in both comparisons, so there was at least some evidence that these genes could be transcribed in heart tissue. The GO categories and KEGG pathways related to different DE genes thresholds were recovered with GOSeq for an easier scripting implementation of the scanning procedure over the *FDR* thresholds. The length of the stability region (along the DE genes definition in units of -log_10_*FDR*) and the GO significance threshold (in units of -log_10_*p*-values) were slightly adjusted through the different categories in order to list around 10 top categories for each group, eventually sorted by the total area. These adjustments follow the intrinsic variability of the maximum value of significance achieved by the different type of categories in the two groups (e.g. *p* ≈ 10^−6^ for Biological Process and *p* ≈ 10^−4^ for KEGG pathways).

### Transcriptional modules

The ISA algorithm was run on the (log_10_) TMM-normalized data after removing genes with low counts across the 160 samples (16397 genes). The data were then z-normalized through the built-in “*ISANormalize*” function. The iterations were initiated with 100 random seeds for each threshold combination (8 different gene thresholds ranging from 0 to 4 and and 8 different samples thresholds ranging from 0 to 1 were tested). For each seed a random selection of strains was chosen with a 50 % chance for each strain to be part of the seed. The seed vector was then formed by assigning “1” to ISO samples, “-1” to ATE samples (or vice-versa) and “0” to CTR samples. Using more seeds (160, 250, and 1000) did not result in additional modules, indicating that module identification had been saturated. The direction of the iteration (within the “*ISAIterate*” function) was set to “*updown*” for the conditions (samples) and to “*up*” for the features (genes). The resulting modules were filtered out in case of redundancy (with the “*ISAUnique*” function) and in case of low robustness (with the “*ISAFilterRobust*” function, carried out with 100 permutations). The modules were finally selected if they contained both ATE and ISO samples.

### GO analysis of the modules and macro-modules definition

The correlations between the module condition scores and 22 organismal phenotypes profiles among each of the 160 samples were explored after averaging the phenotypic values over each set of biological replicates.

For the 98 modules obtained with the ISA algorithm we performed a GO analysis (KEGG pathways, BP, CC and MF) by progressively including in the analysis only the genes with the highest scores, applying a step size of 0.05. The significance patterns of the various GO categories are represented as heatmaps in Additional file 1: Figures S27-S34 for two modules (which had strong (anti-) correlation with HR phenotypes). For the sake of readability, only the top 40 categories (when present in such number or greater) are shown. We only retained categories whose *p*-value was significant in at least 1 % of all enrichment tests across different gene-score cutoffs.

Analyzing the GO categories of the 98 modules we realized that modules with very different gene content frequently shared functional annotations. We therefore evaluated a simple similarity measure between the modules based on the number of shared GO categories and clustered the modules according to these similarity values (see Additional file 1: Figures S36-S39). Specifically, we adopted the Jaccard coefficient as the measure of similarity. This coefficient is defined as J = |A∩B|/|AUB|, where A and B are two intersecting sets. In order to quantitatively define the macro-modules, we ran again the ISA algorithm (implemented in *R* with the “*isa*” function) on the similarity matrices (one for each GO category) and selected the resulting macro-modules if they contained at least 5 modules for an average macro-module similarity of 0.4. The Jaccard coefficient (used to find the macro-modules) was evaluated on the basis of all module categories surviving a *p*-value below 0.01 in at least one score step. For the sake of readability, the heatmaps in Additional file 1: Figures S40-S51 only show up to 40 entries.

## Additional files

Additional file 1: Supplementary figures. (PDF 12000 kb) Additional file 2: Supplementary tables. (PDF 3020 kb) Additional file 3: Supplementary information. (PDF 416 kb) Additional file 4: Supplementary data. Compressed HTML files of 98 expression modules annotated for genes, strains and GO or KEGG terms (see Additional file 3 for navigation details). (GZ 11006 kb)

## Abbreviations

*Acta2*: Alpha actin 2
ATE: Atenolol
BP: Biological process
*Camta1*: Calmodulin binding transcription activator 1
CC: Cellular component
CE: Counter-expressed
CEI: Counter-expression index
CoE: Co-expressed
CTR: Control
DAVID: Database for annotation, visualization and integrated discovery
DE: Differentially expressed
DEI: Differential expression index
ECG: Electrocardiography
eQTL: Expression quantitative trait locus
*Etv5*: ETS variant 5
FC: Fold change
FDR: False discovery rate
*Fitm2*: Fat storage inducing transmembrane protein 2
GEO: Gene expression omnibus
GO: Gene ontology
GRP: Genetic reference population
GWAS: Genome-wide association study
HMDP: Hybrid mouse diversity panel
HR: Heart rate
ISA: Iterative signature algorithm
ISO: Isoproterenol
KEGG: Kyoto encyclopedia of genes and genomes
*Lims1*: LIM zinc finger domain containing 1
MF: Molecular function
MGI: Mouse genome informatics
MHC: Major histocompatibility complex
*Myom2*: Myomesin 2
PBS: Phosphate buffered saline
PCA: Principal component analysis
*Phkg1*: Phosphorylase kinase gamma subunit 1
PKA: Protein kinase A
*Polr1e*: Polymerase (RNA) I subunit E
PPAR: Peroxisome proliferator-activated receptor
QTc: Corrected QT interval, as recorded by ECG
RNAseq: RNA sequencing
*Ryr2*: Ryanodine receptor 2
SD: Standard deviation
SSI: Strain-specific index
*Synpo2l*: Synaptopodin 2 like
TC: Tail-cuff
TG: Transforming growth factor
TMM: Trimmed mean of M-values
