# Supplementary material for: RNAseq analysis of heart tissue from mice treated with atenolol and isoproterenol reveals a reciprocal transcriptional response
Source: BMC Genomics. 2016 Sep 7;17(1):717. doi: 10.1186/s12864-016-3059-6 (PMC5015234; doi:10.1186/s12864-016-3059-6)
Supplement: Additional file 1: — Supplementary figures. (PDF 12000 kb) [file 12864_2016_3059_MOESM1_ESM.pdf]

Supplementary Figures

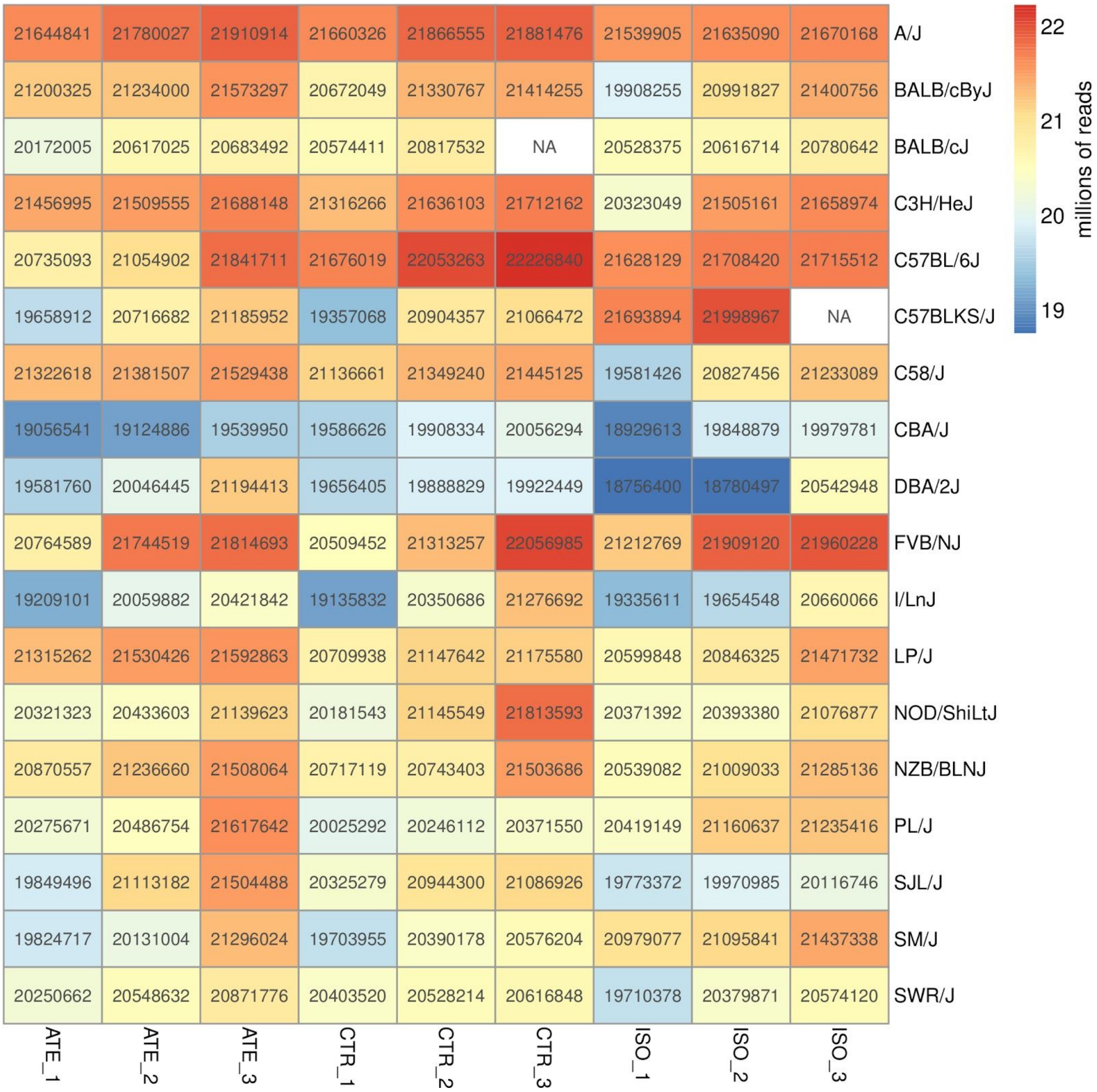

Supplementary Figure 1a: Summary of the number of sequencing reads obtained for each of the 160 samples. The dataset contains 3 CTR, 3 ATE and 3 ISO biological replicates for each strain, except in strains BALB/cJ and C57BLKS/J where one CTR, respectively one ISO samples failed sequencing.

	ATE_1	ATE_2	ATE_3	CTR_1	CTR_2	CTR_3	ISO_1	ISO_2	ISO_3
A/J	13201	13211	13244	13152	13177	13208	13200	13248	13261
BALB/cByJ	13129	13252	13323	13155	13286	13477	13225	13246	13556
BALB/cJ	13190	13383	13488	13367	13406	NA	13252	13320	13483
C3H/HeJ	13427	13432	13468	13357	13445	13455	13388	13562	13602
C57BL/6J	13074	13181	13184	13218	13243	13278	13250	13375	13388
C57BLKS/J	13126	13212	13338	13051	13246	13289	13398	13540	NA
C58/J	13225	13278	13421	13216	13226	13454	13231	13268	13305
CBA/J	12707	12845	12849	12926	13044	13104	12936	12967	13172
DBA/2J	13254	13497	13656	13276	13448	13610	13318	13623	13697
FVB/NJ	13377	13466	13496	13277	13316	13538	13410	13509	13557
I/LnJ	13135	13372	13390	13245	13288	13546	13245	13327	13495
LP/J	13109	13120	13152	13017	13230	13265	13195	13287	13349
NOD/ShiLtJ	13366	13390	13459	13310	13366	13394	13600	13611	13644
NZB/BLNJ	13130	13232	13293	13197	13243	13291	13327	13342	13402
PL/J	13326	13371	13556	13224	13331	13374	13350	13413	13497
SJL/J	13245	13329	13515	13391	13394	13491	13380	13422	13473
SM/J	13068	13084	13263	13014	13235	13336	13160	13249	13279
SWR/J	13270	13390	13471	13349	13385	13388	13374	13480	13550

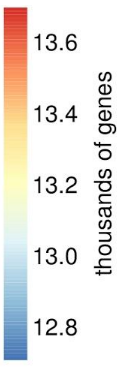

Supplementary Figure 1b: Number of genes mapped in each sample. The values refer to genes with at least 5 reads in each sample. Without applying any such thresholding, the total number of mapped genes is 19293. Upon thresholding, this number is reduced to 16397.

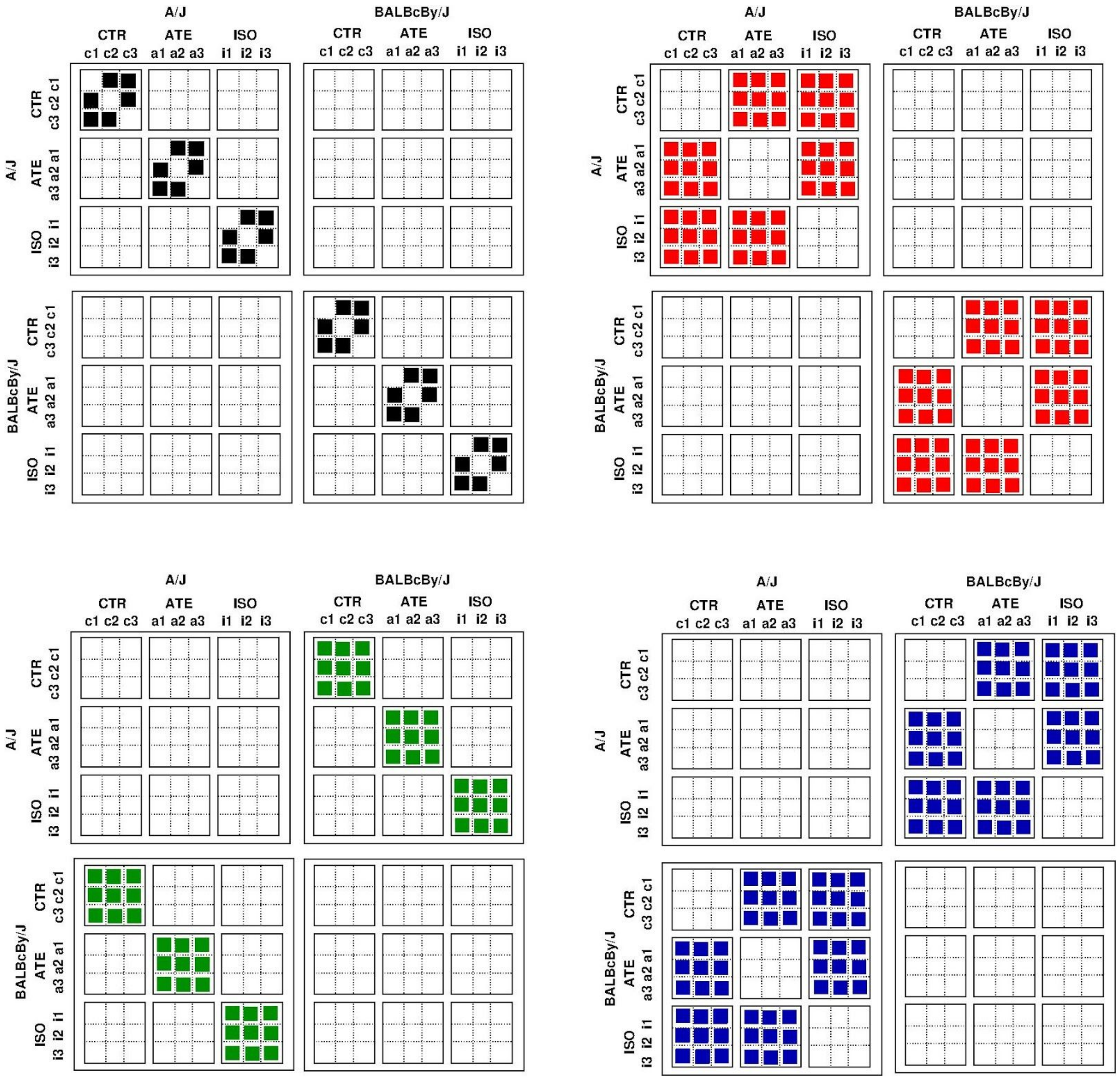

Supplementary Figure 2: Correlations between samples were assigned into four classes: 158 correlations between biological replicates (i.e. same strain and same treatment, indicated in black), (2) 474 between samples from the same strains but under different treatment (red), (3) 4029 between samples under the same treatment but from different strains (green) and (4) 8059 between samples from different strains and under different treatments. For the sake of simplicity, only the first two strains in alphabetical order are shown. See Suppl. Figure 4 for the quantitative results.

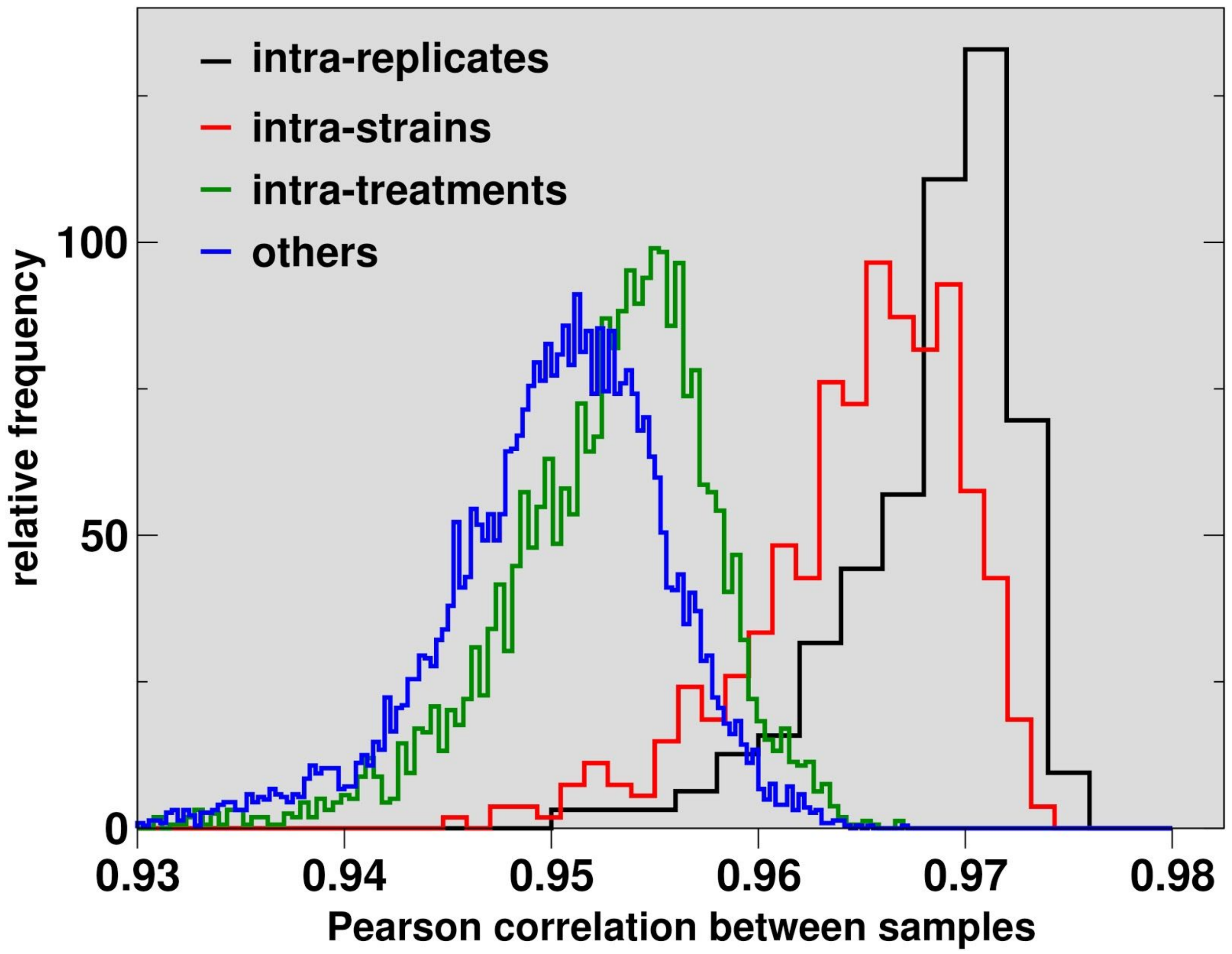

Supplementary Figure 3: Distributions of the four types of cross-sample correlations described in Suppl. Figure 2. As expected, the highest correlations are observed between biological replicates (black line) with C_1=0.97±0.003 (mean ± SD), while the lowest correlations occur between unrelated samples (blue line, C_4=0.950±0.005). The distributions of intra-strain correlations (C_2=0.965±0.005, red line) and intra-treatment correlations (C_3=0.955±0.005, green line) stand in between, yet the former are significantly higher than the latter ($t_{2,3}\approx-49.1$, $p_{23}\approx3.9\times10^{-211}$). The separation of the three intra-groups with respect to the cross-conditions one is statistically highly significant (for intra-replicates: $t\approx51.8$, df=166.625, $p\approx1.3\times10^{-104}$; for intra-strain: $t\approx61.1$, df=537.635, $p\approx3.8\times10^{-244}$; for intra-treatment: $t\approx25.7$, df=8424.173, $p\approx1.7\times10^{-140}$).

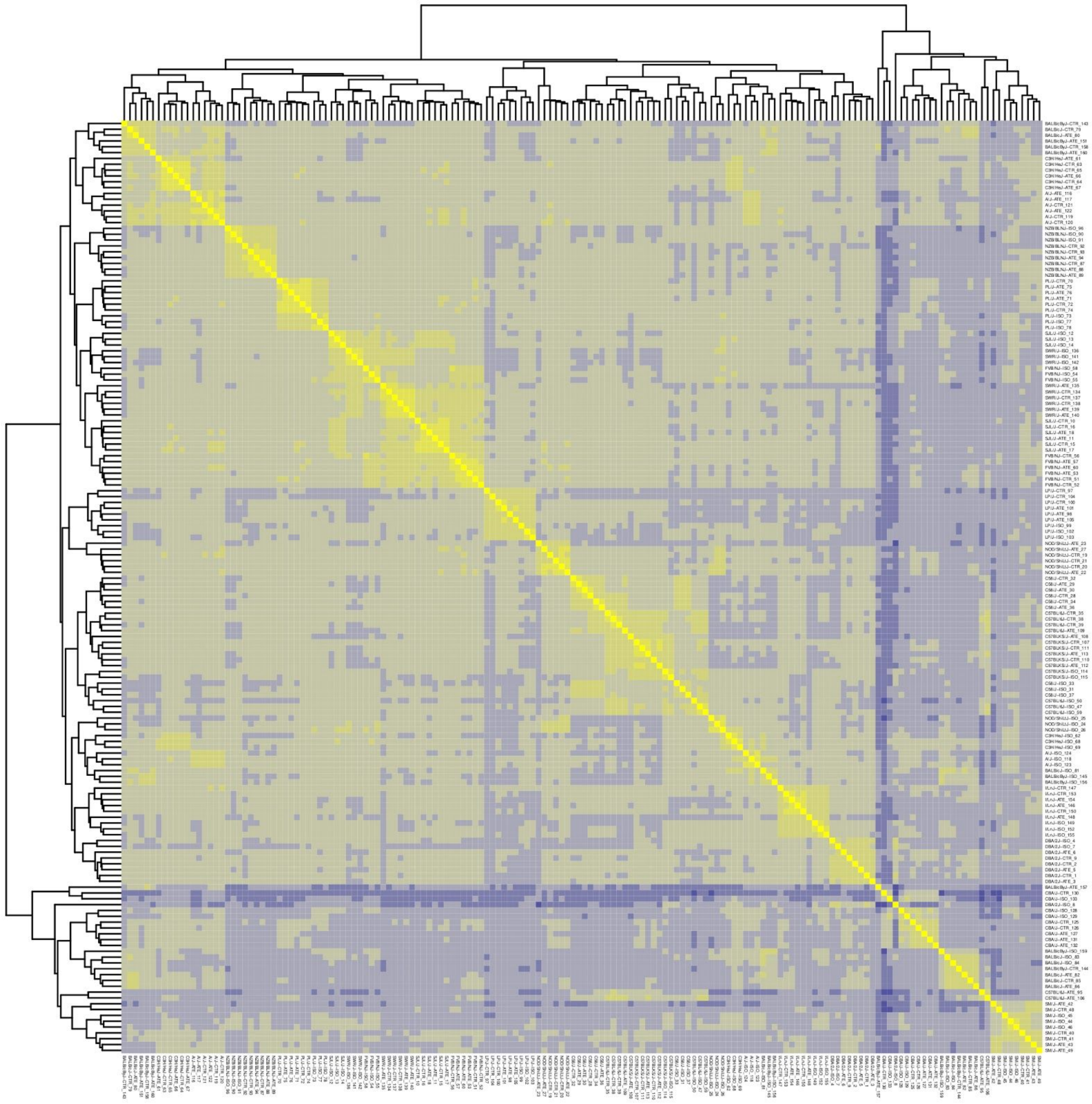

Supplementary Figure 4: Clustered matrix of cross-sample Pearson correlations of the (log_{10}-transformed) TMM-normalized reads computed across the 16397 expressed genes. The color code ranges from dark blue (0.92) to yellow (1.0). Samples cluster first by biological replicates, then predominantly by strain rather than treatment (with very few exceptions, see Suppl. Figures 5-8).

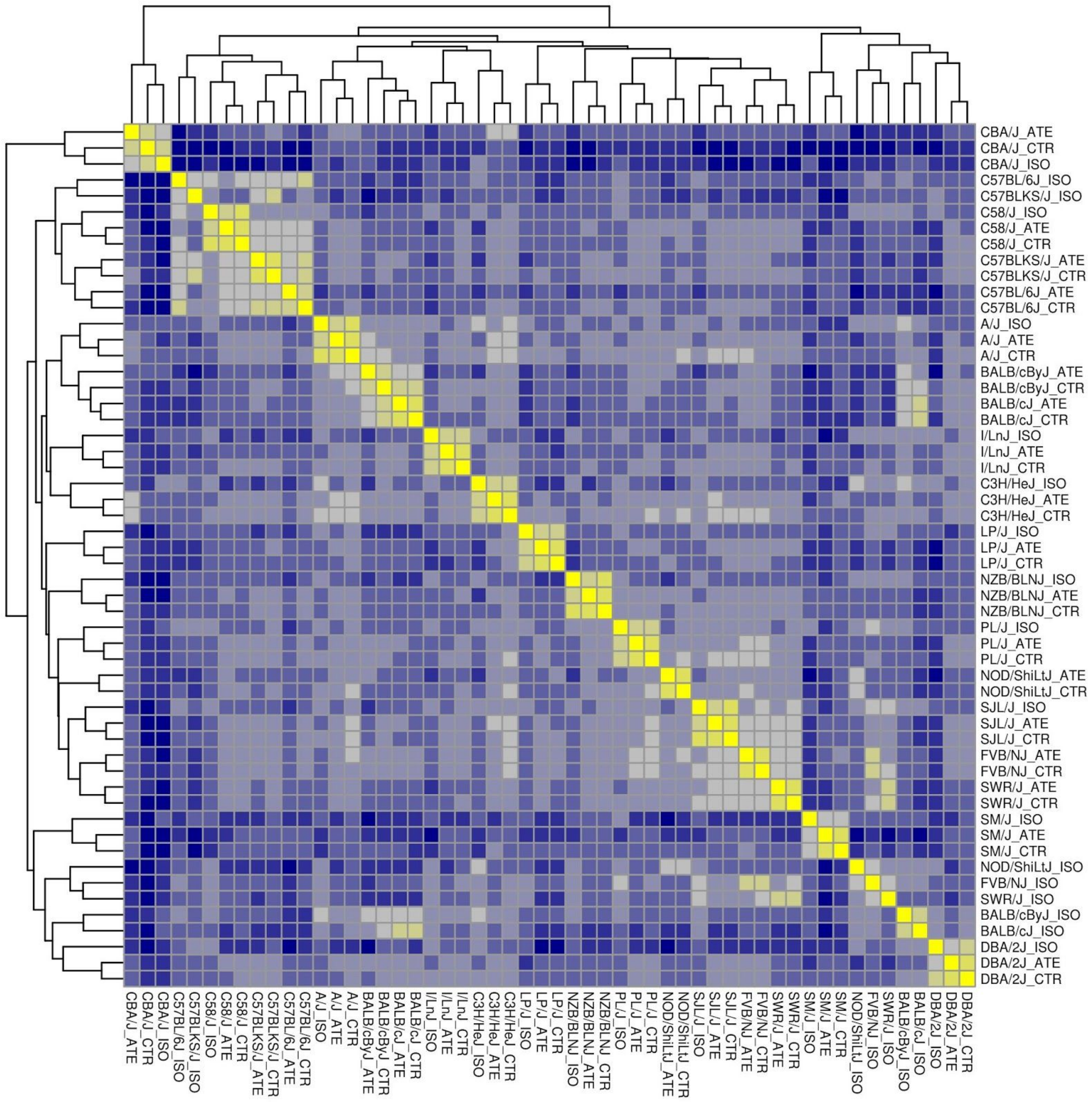

Supplementary Figure 5: Clustered matrix of cross-sample Pearson correlations of the (log_{10}-transformed) TMM-normalized reads averaged over biological replicates. The color code ranges from dark blue (0.92) to yellow (1.0). Samples cluster predominantly by strain; however in some cases the related strains under ISO treatment are grouped together (C57BL/6J and C57BLKS/J as well as BALB/cByJ and BALB/cJ).

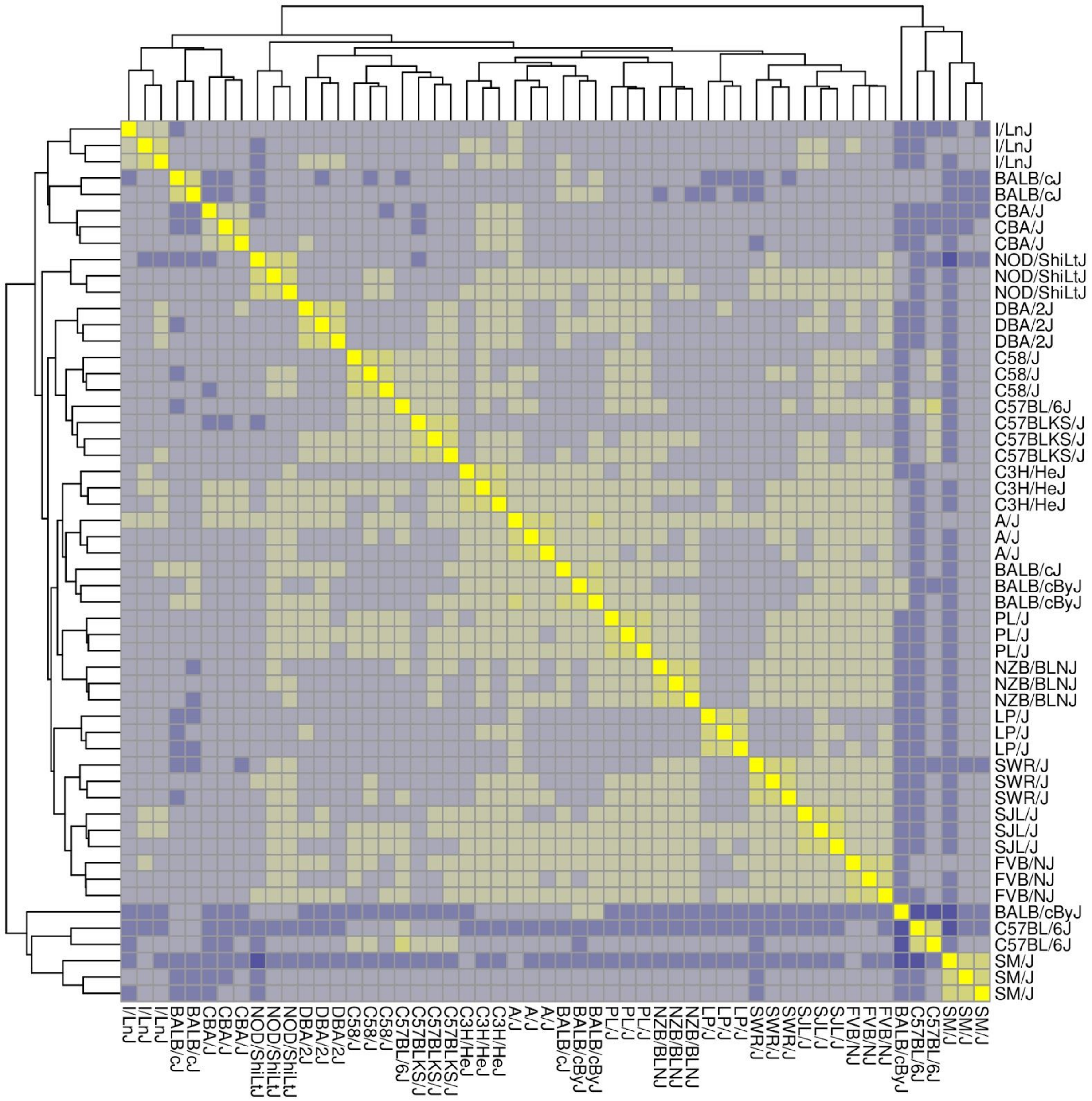

Supplementary Figure 6: Pearson correlation matrix between the (log_{10}-transformed) TMM-normalized reads of the 54 ATE samples. The analysis relates to the genes expressed above 5 reads in all of the selected samples (15814 genes).

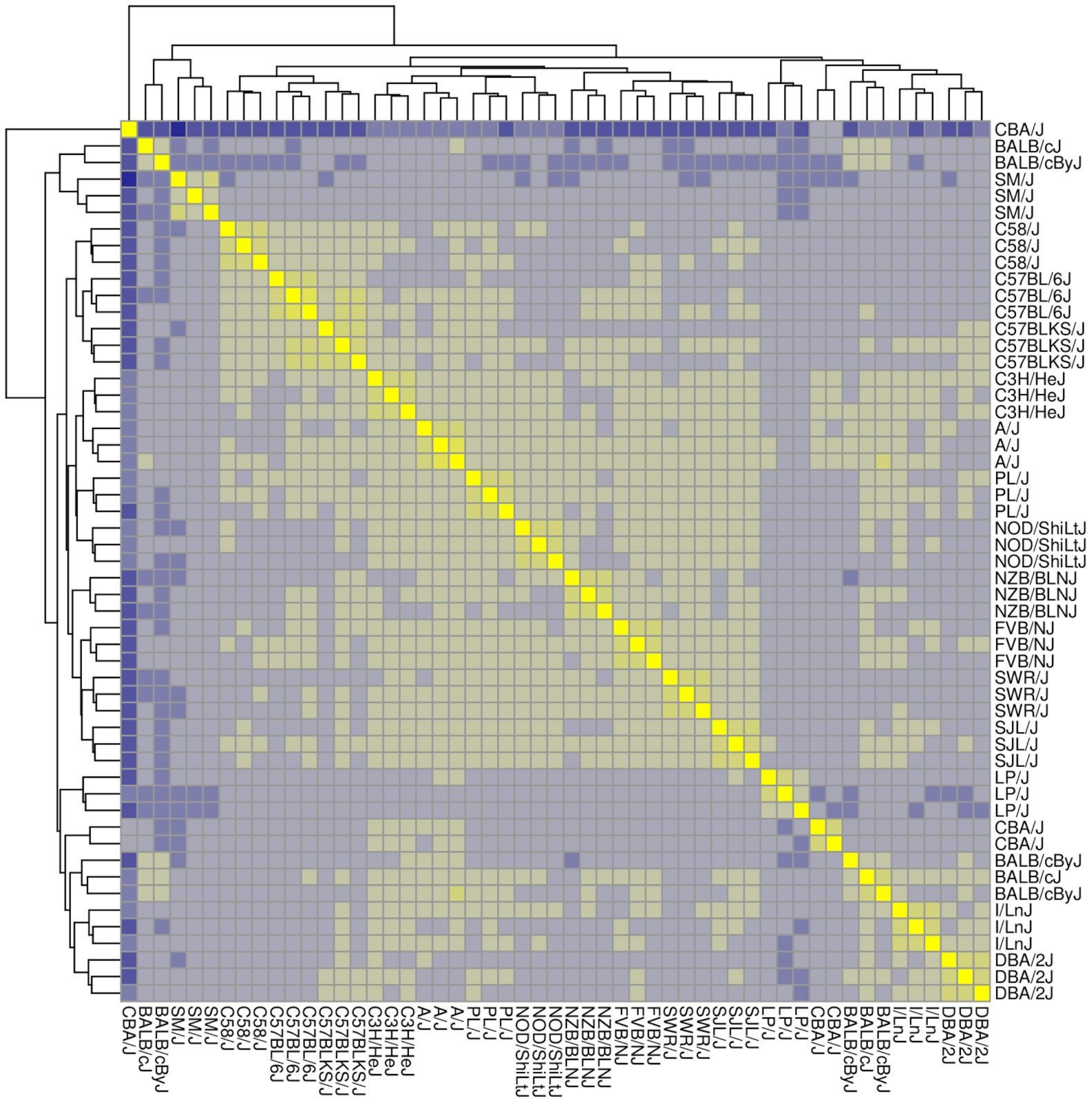

Supplementary Figure 7: Pearson correlation matrix between the (log_{10}-transformed) TMM-normalized reads of the 53 CTR samples. The analysis relates to the genes expressed above 5 reads in all of the selected samples (15821 genes).

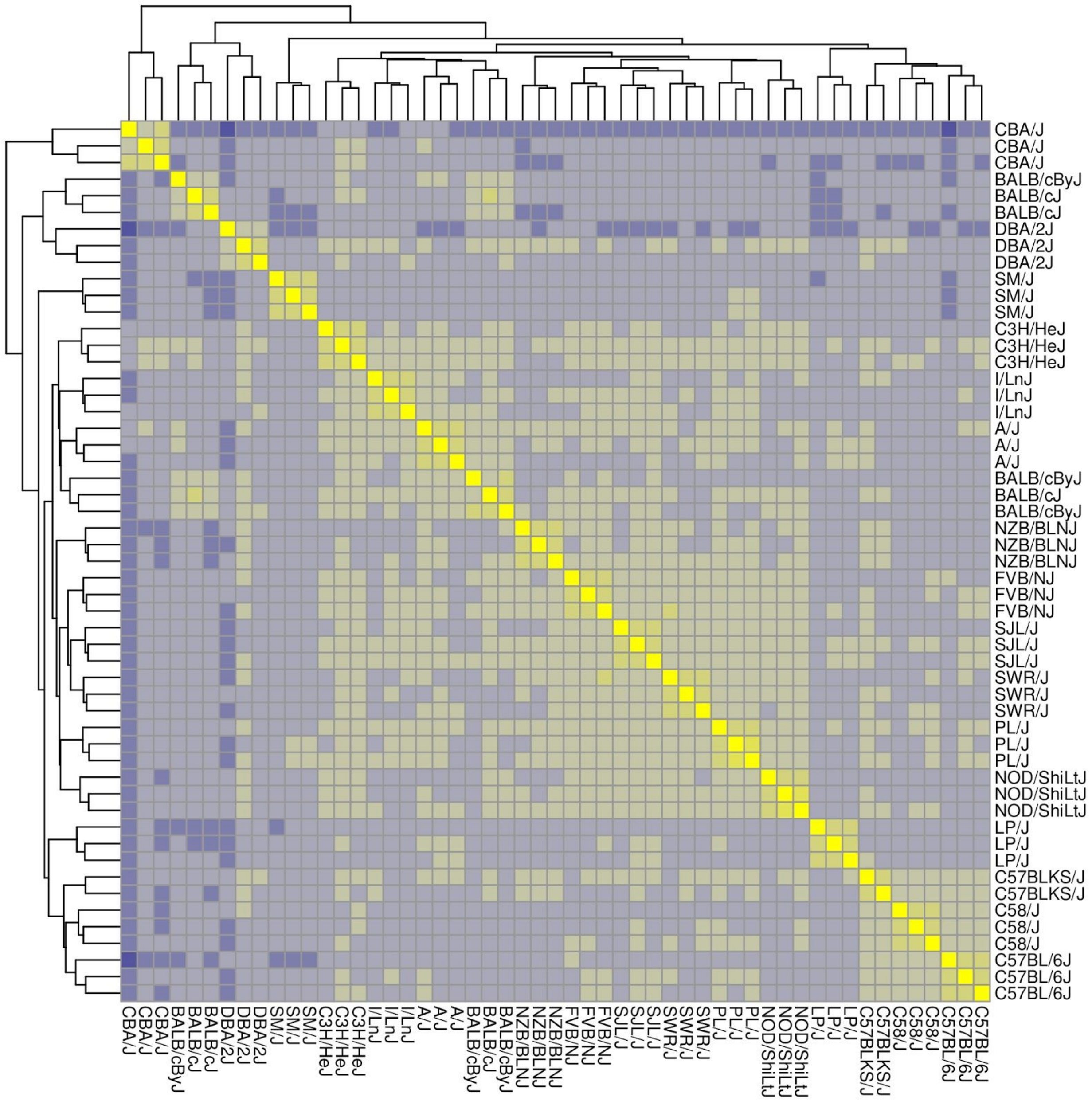

Supplementary Figure 8: Pearson correlation matrix between the (log_{10}-transformed) TMM-normalized reads of the 53 ISO samples. The analysis relates to the genes expressed above 5 reads in all of the selected samples (15857 genes).

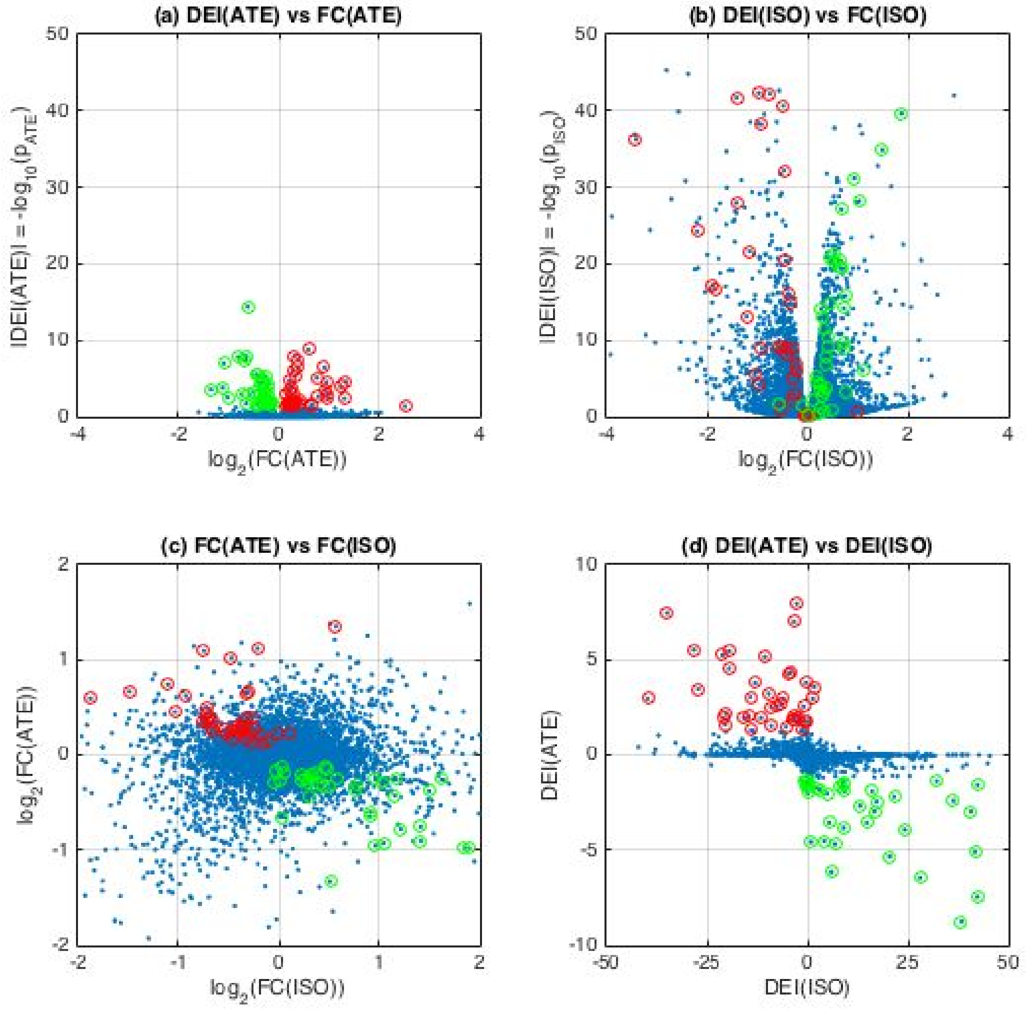

Supplementary Figure 9a-d: Comparison of fold-change (FC) and differential expression index (DEI) under ATE and ISO treatments. The significance values (i.e. $-\log_{10}p$ which is the absolute value of the DEI) are plotted as a function of the $\log_2$FC for ATE (a) and ISO (b) treatment. Green and red circles indicate genes which are significantly (*FDR*<0.05) suppressed and induced, respectively, by ATE treatment. There is a reciprocal behavior of these genes under ISO treatment, which is less pronounced in the comparison of FC (c) than in DEI (d) between the two treatments.

(e)

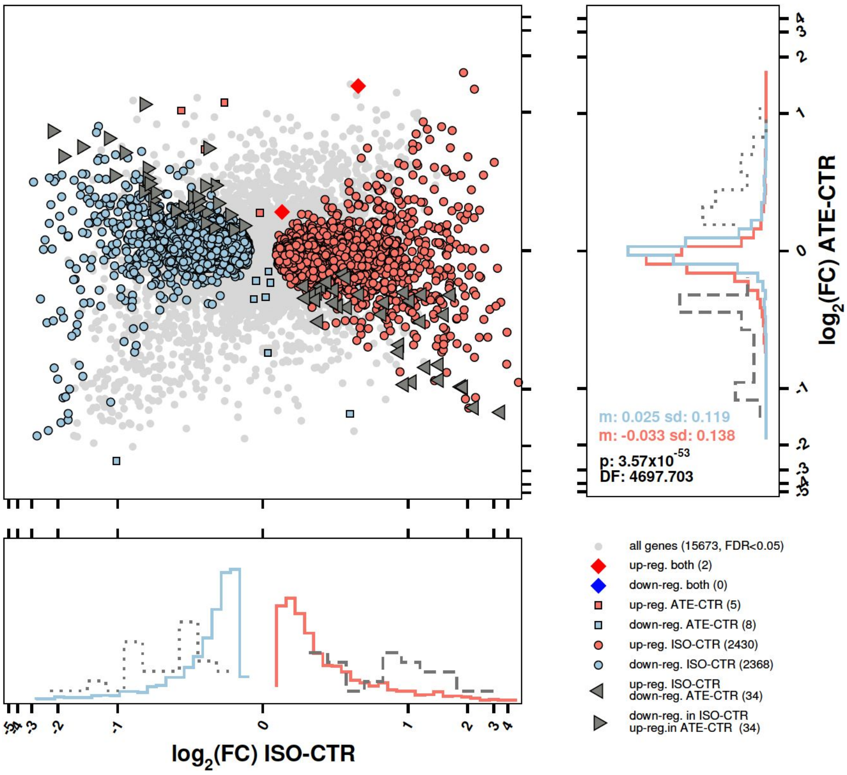

(f)

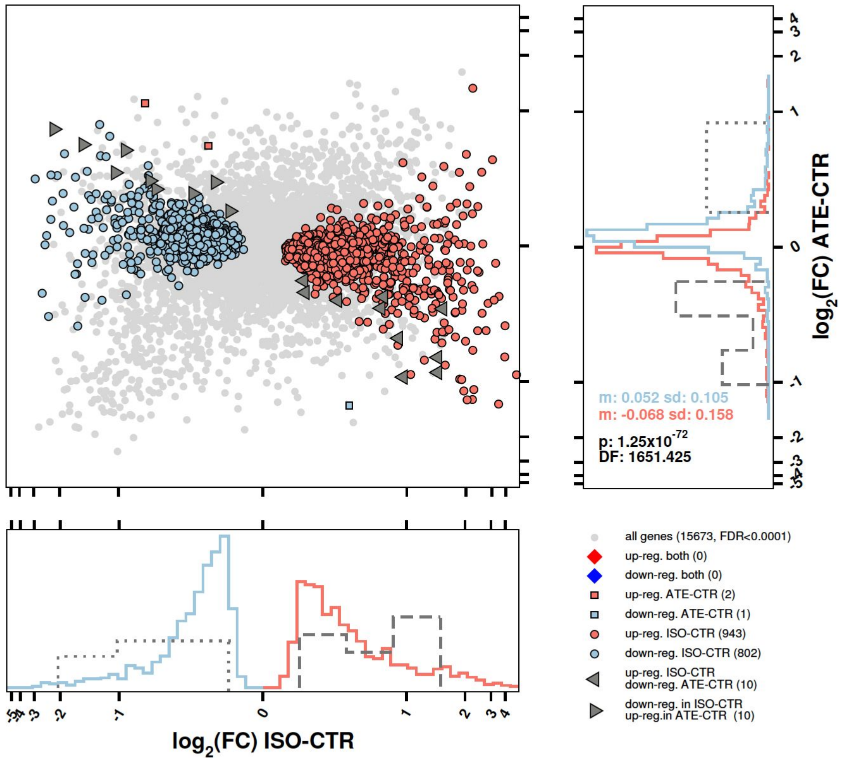

Supplementary Figure 9e-f: Distribution of fold changes (FC) for different FDR thresholds: Genes with a signal for differential expression at FDR = 0.05 (top) and FDR = 0.0001 are highlighted.

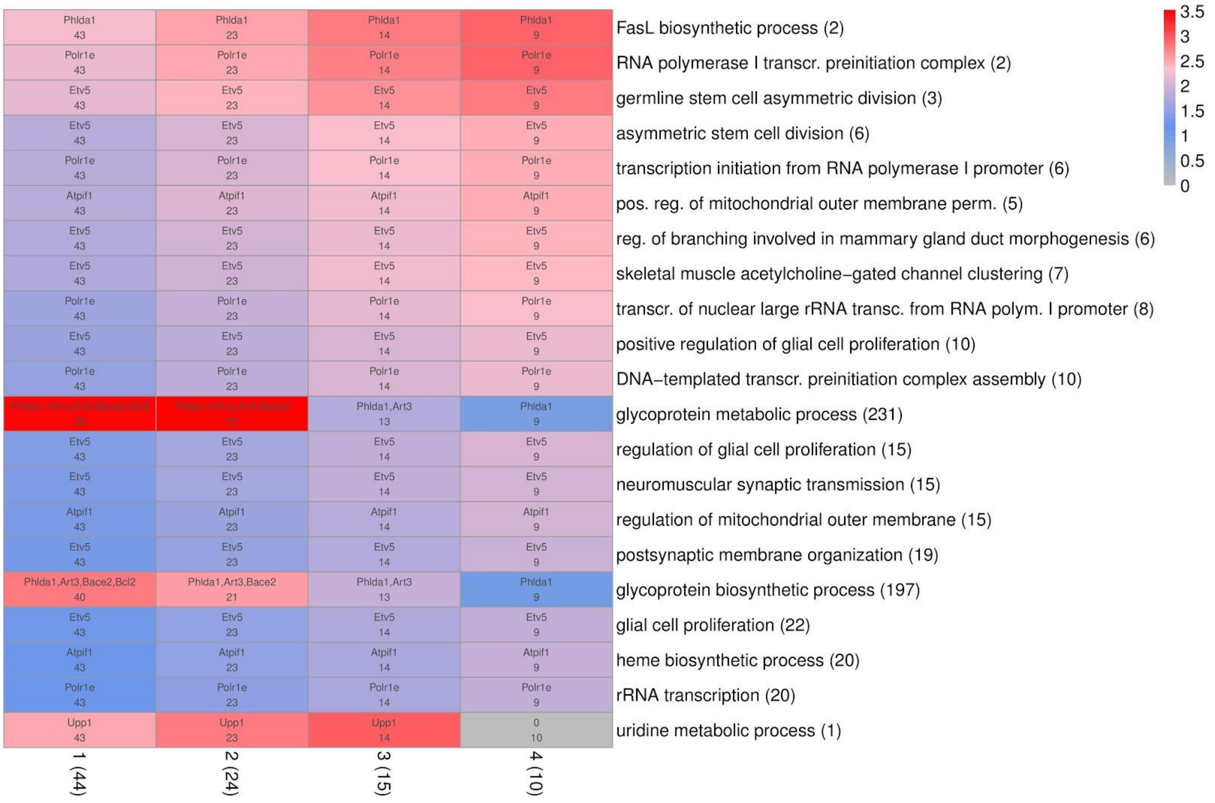

Supplementary Figure 10: Most stable/significant GO BP categories in the *FDR* threshold range for the sets of CE_{ISO}^{ATE} genes. On the *x*-axis, the *FDR* significance and, in brackets, the number of CE genes at that threshold, are reported. On the *y*-axis, the name of the GO category and, in brackets, the number of genes within that category are shown. Within the cells, the number of genes within (first line) or out of (second line) the related category, are shown. Gene names are explicitly shown in case they are less than 5. Cells are colored according to GO significance, as indicated by the color code is on the right bar. See Suppl. Information for further details.

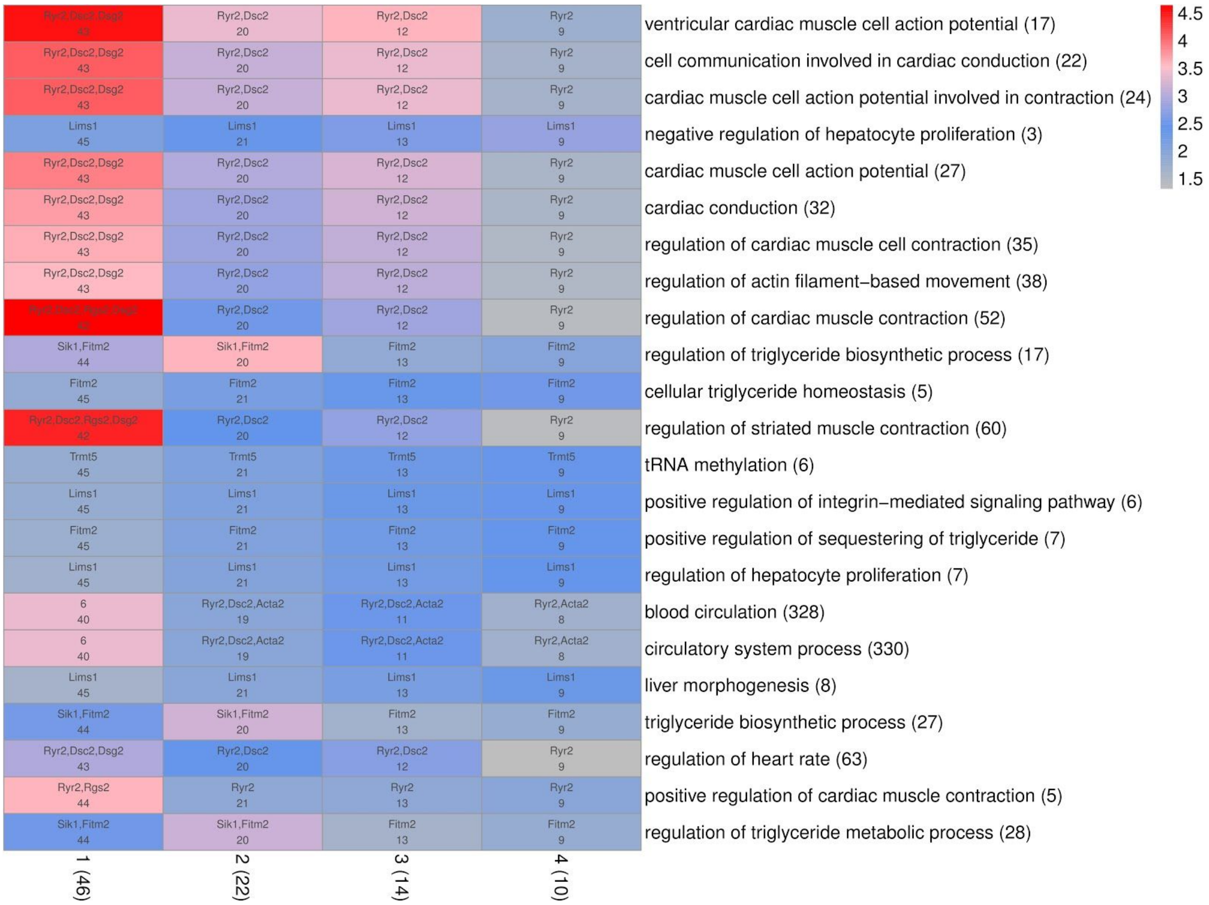

Supplementary Figure 11: Most stable/significant GO BP categories for CE^{ISO}_{ATE} genes.

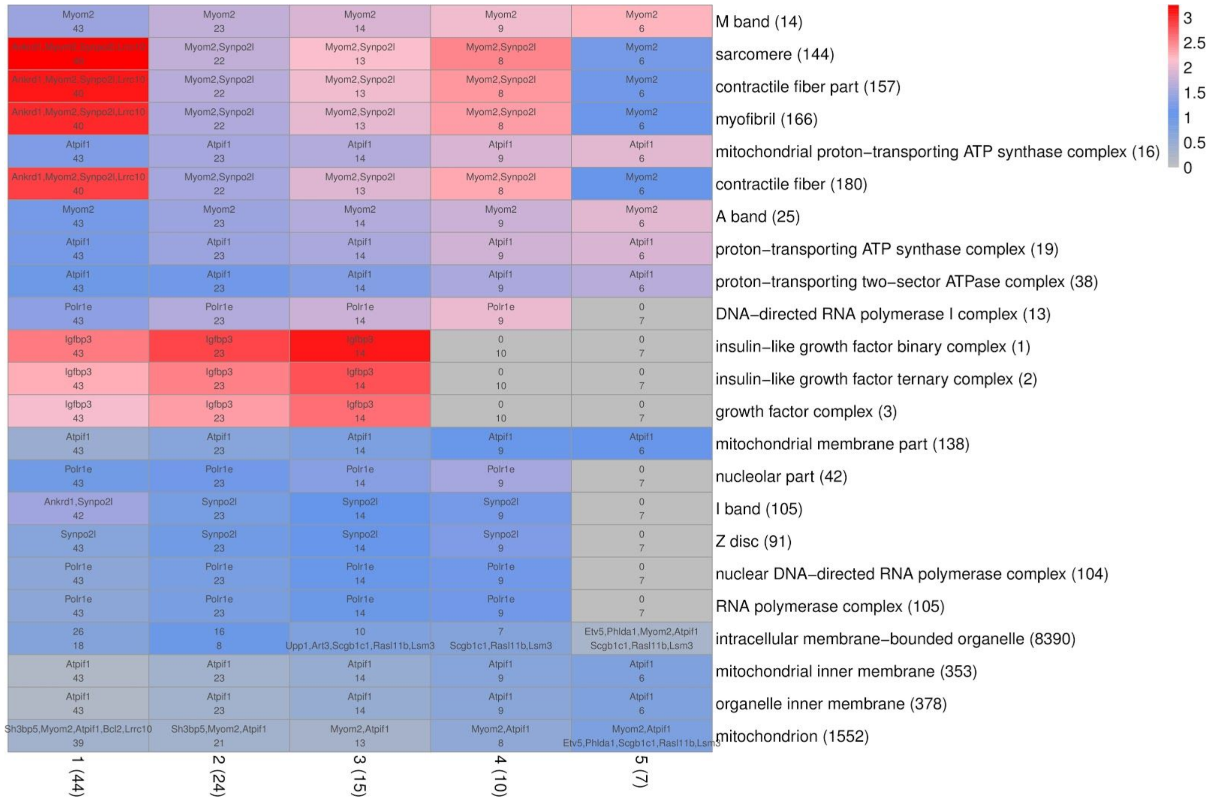

Supplementary Figure 12: Most stable/significant GO CC categories for CE_{ISO}^{ATE} genes.

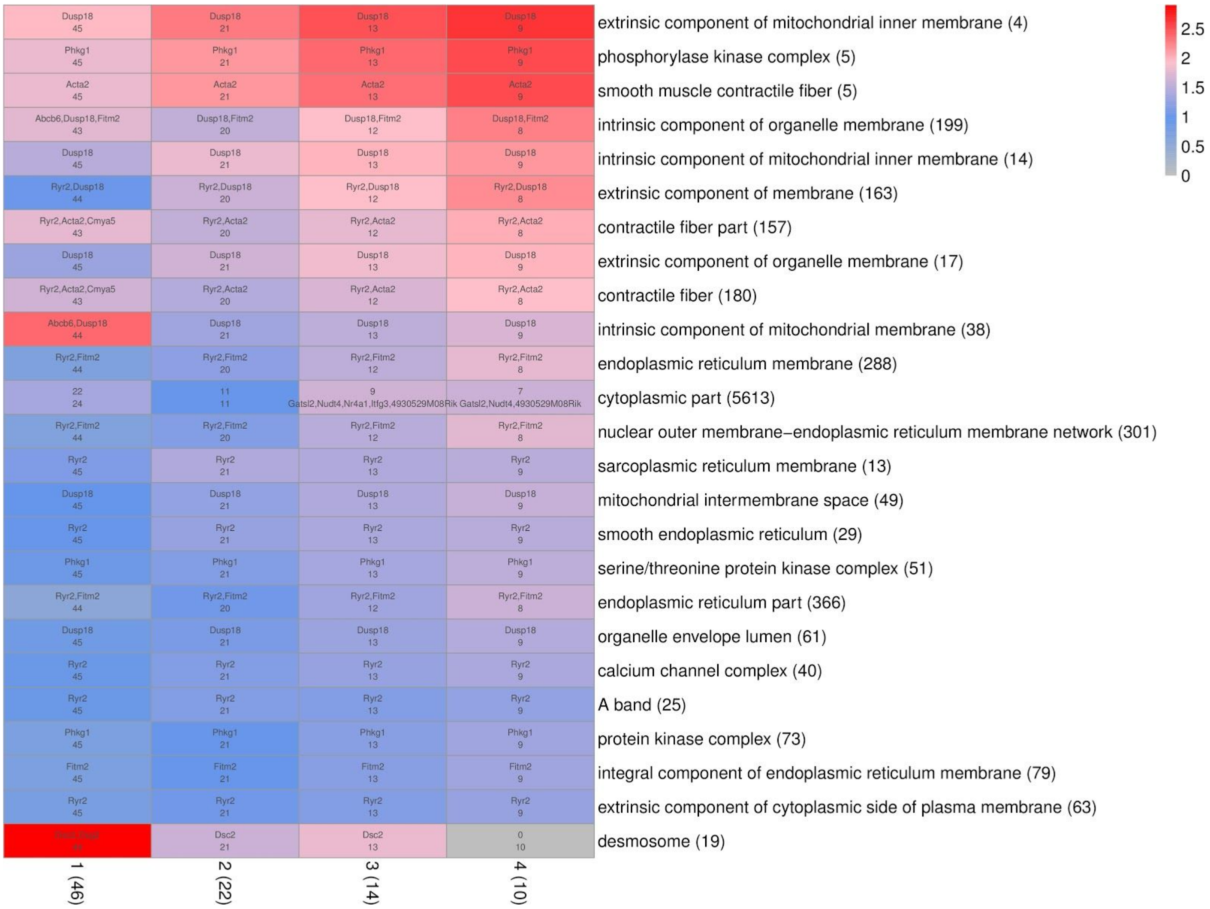

Supplementary Figure 13: Most stable/significant GO CC categories for CE^{ISO}_{ATE} genes.

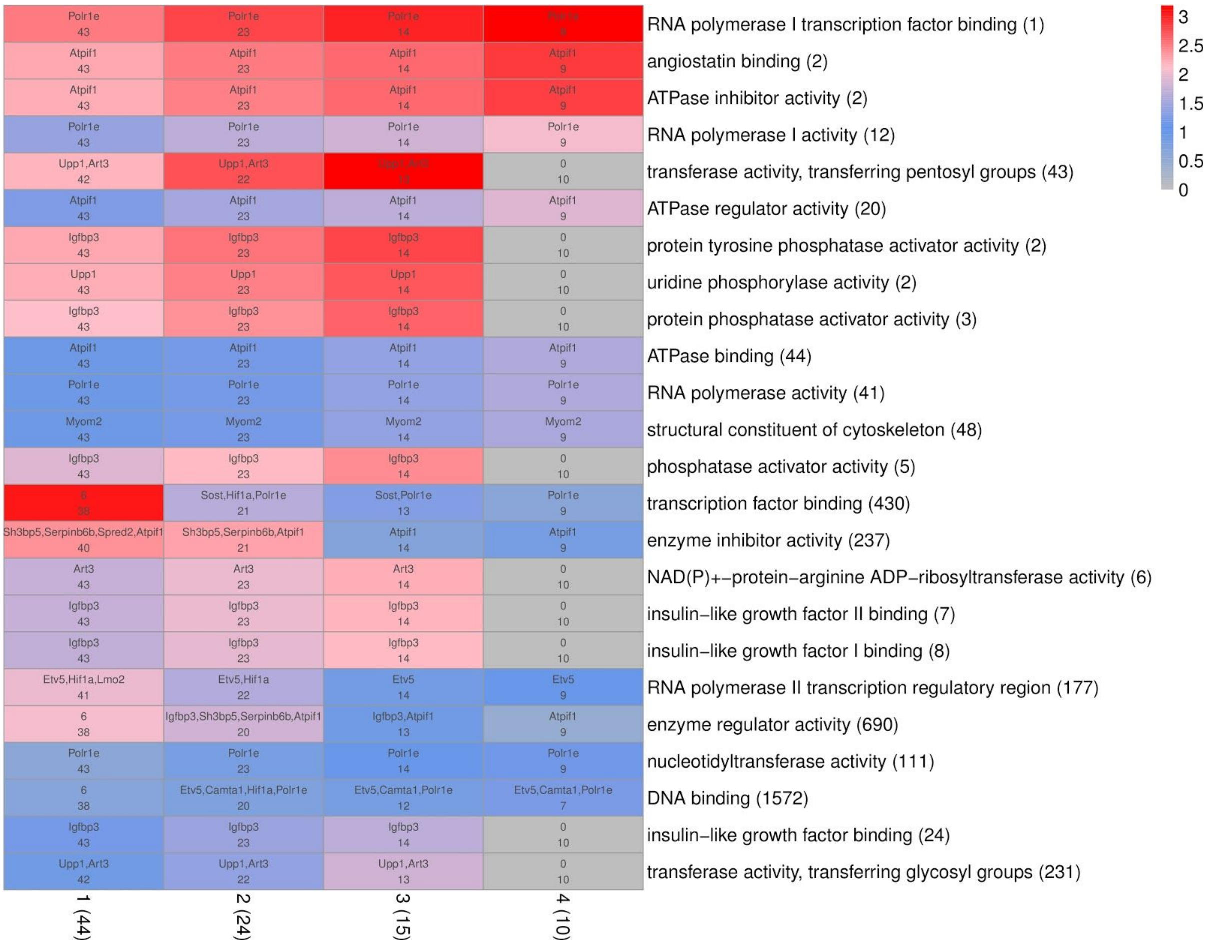

Supplementary Figure 14: Most stable/significant GO MF categories for CE_{ISO}^{ATE} genes.

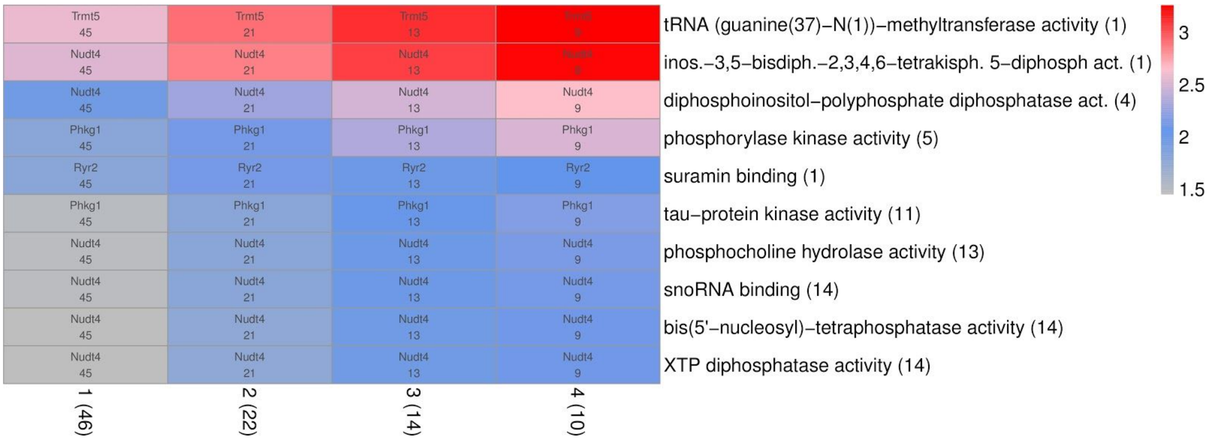

Supplementary Figure 15: Most stable/significant GO MF categories for CE^{ISO}_{ATE} genes.

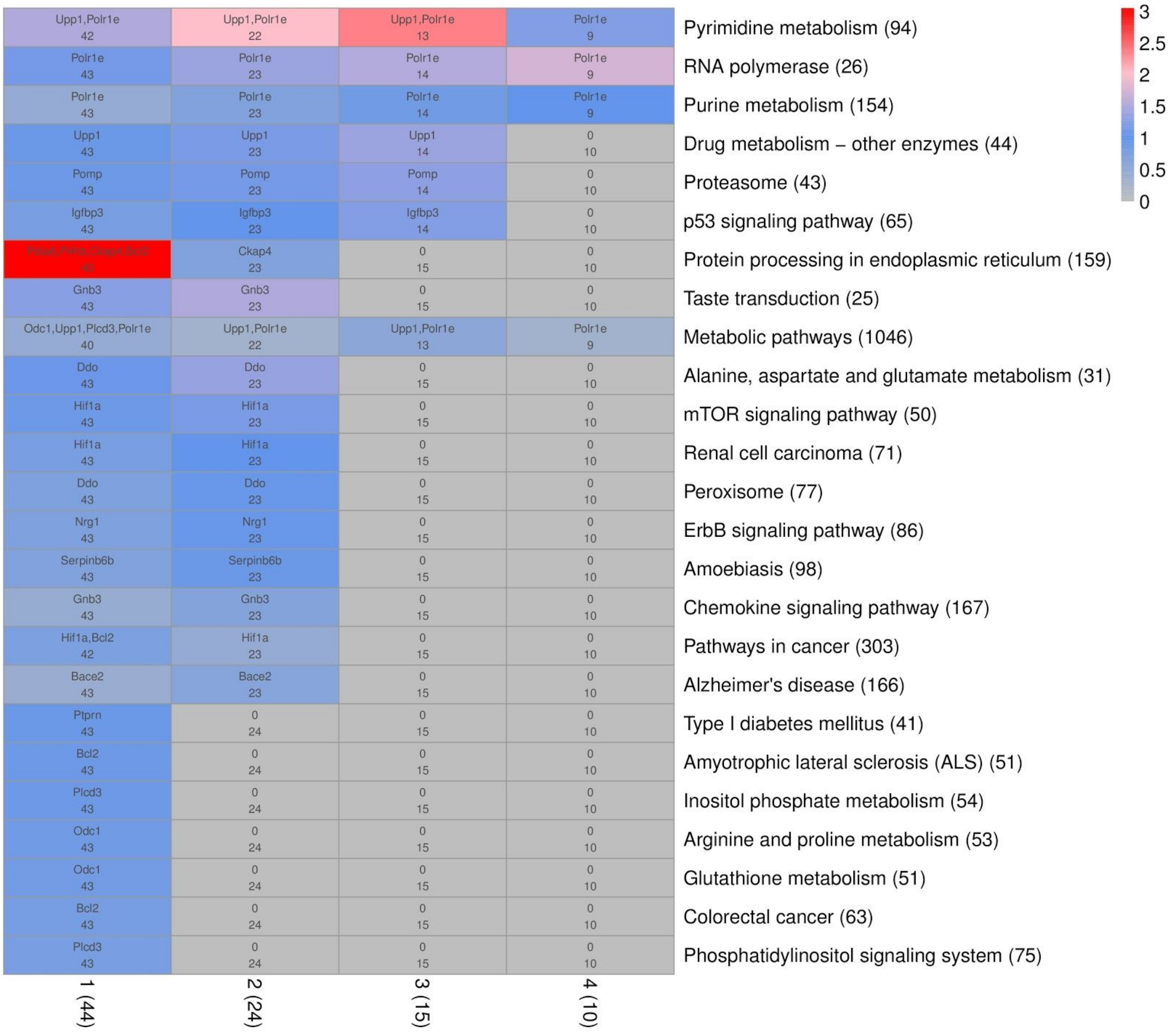

Supplementary Figure 16: Most stable/significant KEGG pathways for CE_{ISO}^{ATE} genes.

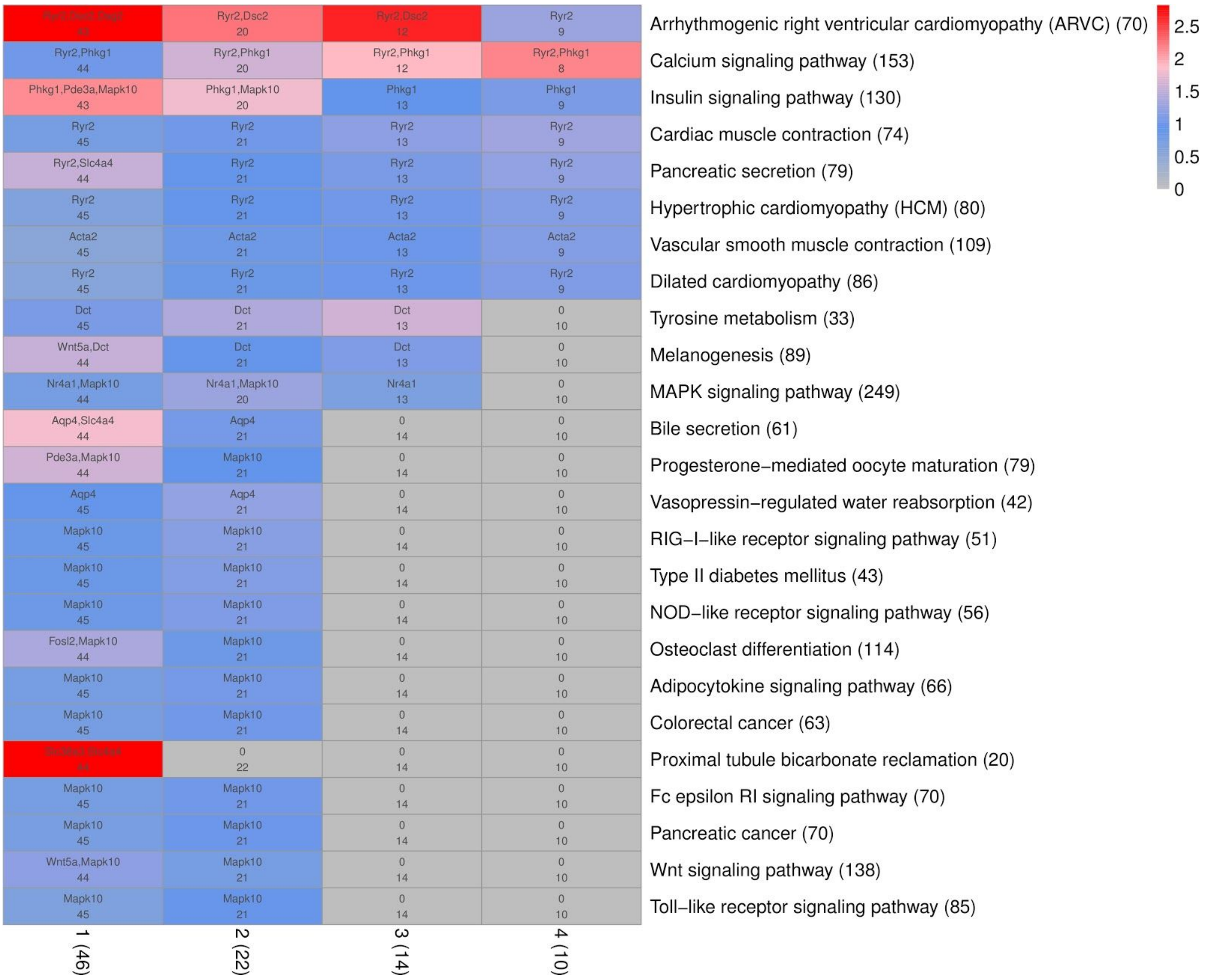

Supplementary Figure 17: Most stable/significant KEGG pathways for CE^{ISO}_{ATE} genes.

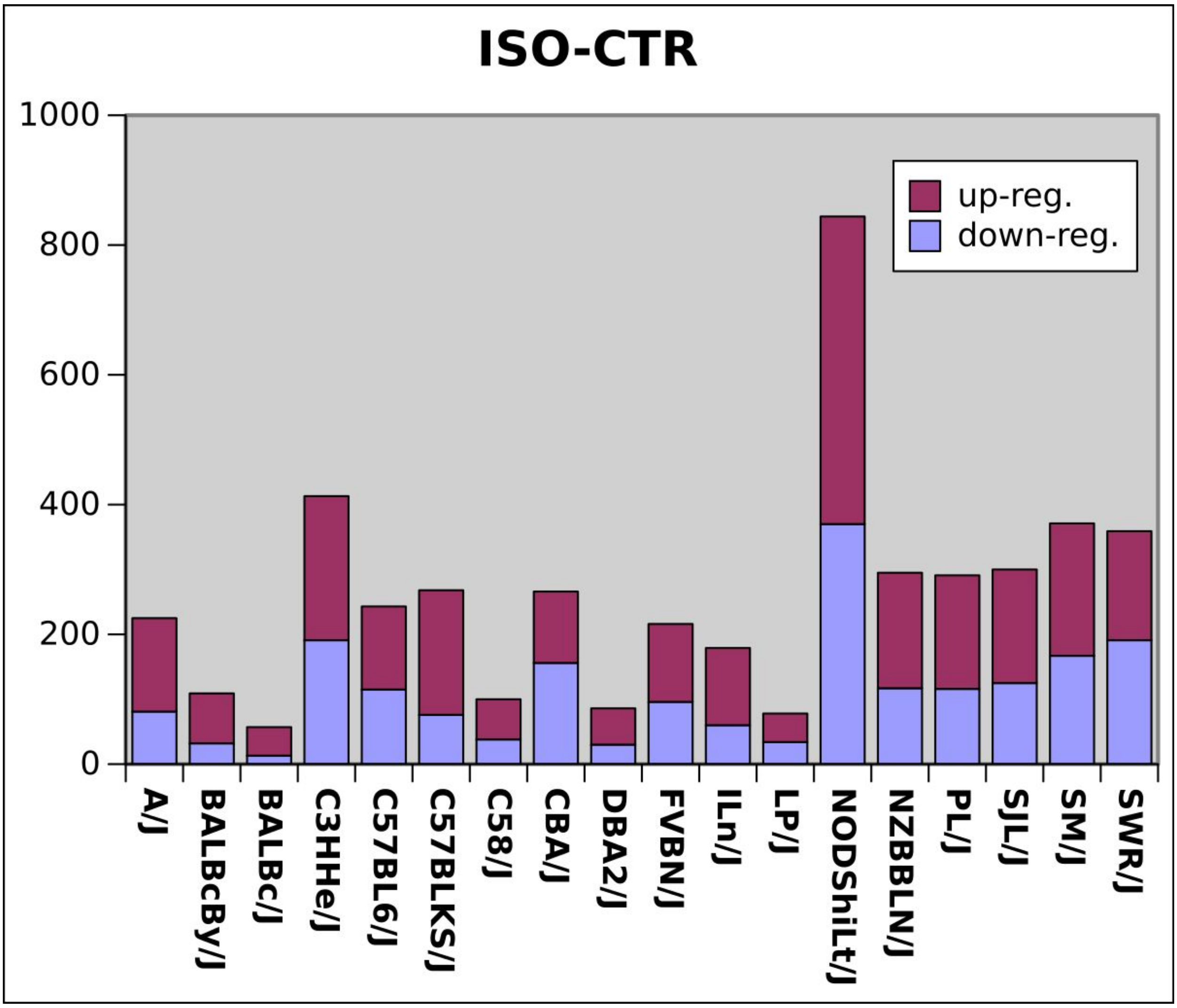

Supplementary Figure 18: ISO *vs* CTR DE analysis in the 18 strains (*FDR*<0.01). See Suppl. Table 2 for further details.

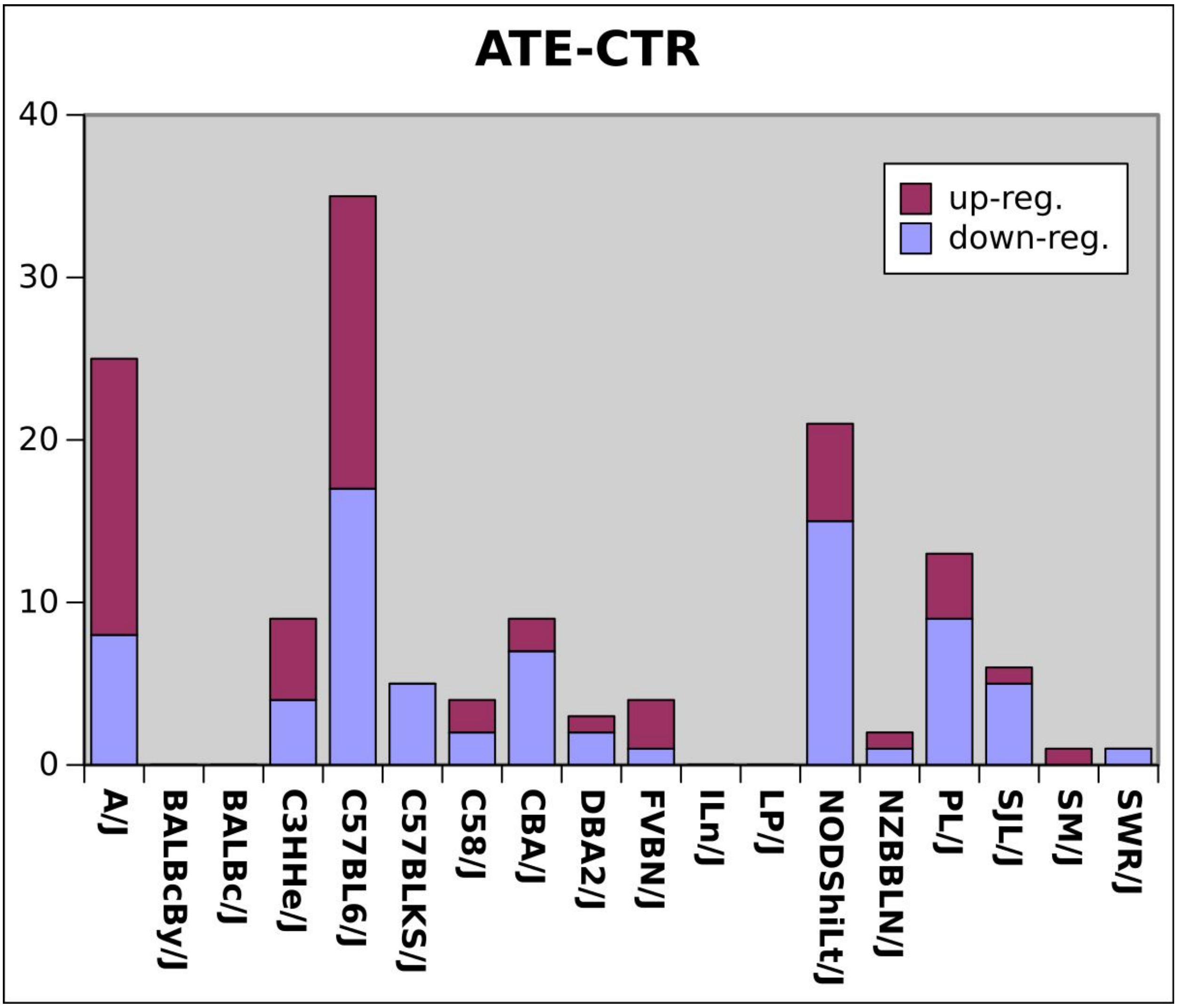

Supplementary Figure 19: ATE *vs* CTR DE analysis in the 18 strains (*FDR*<0.01). See Suppl. Table 2 for further details.

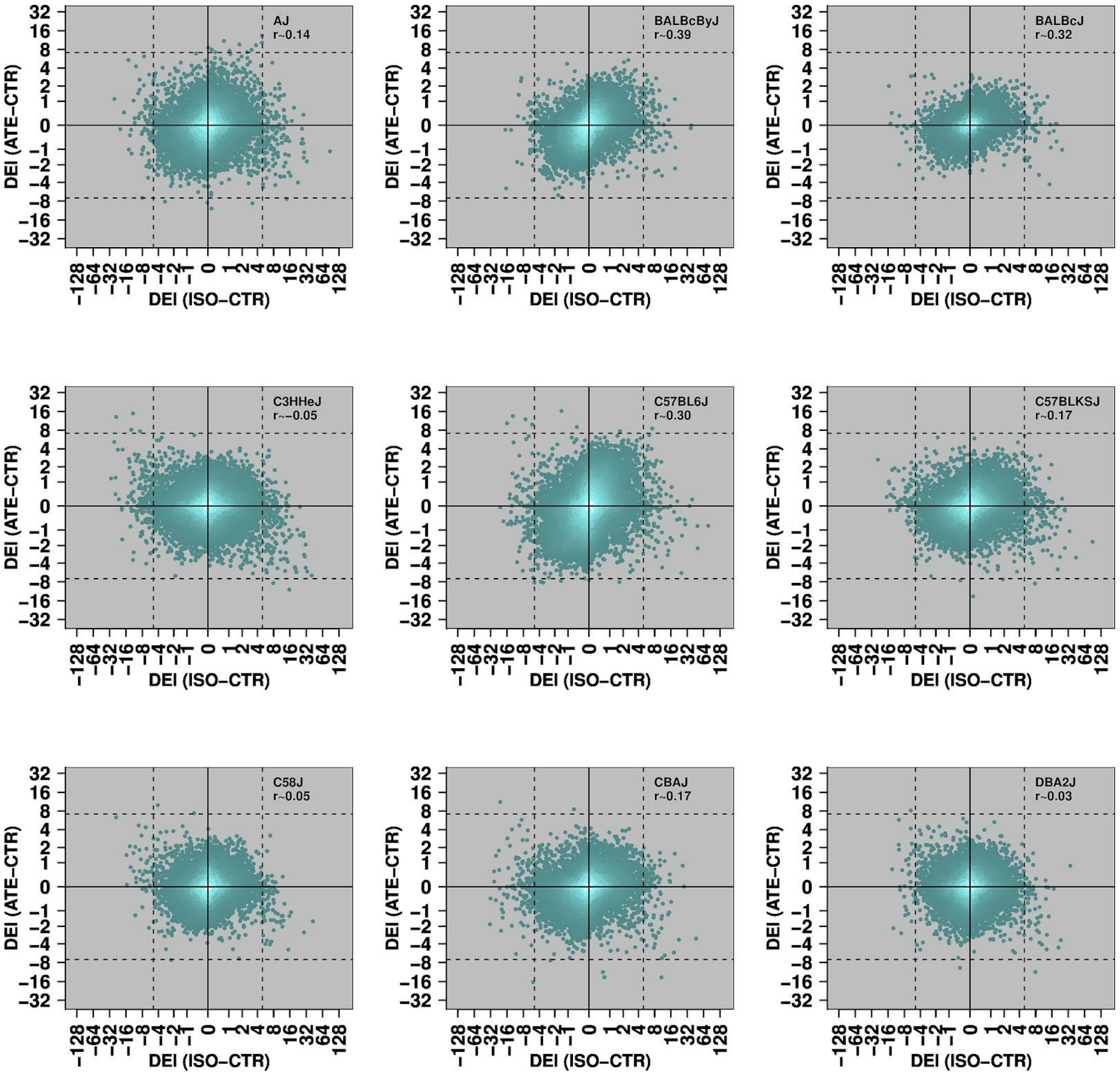

Supplementary Figure 20: Trend of the DE index distribution in each strain. Dashed lines correspond to a *FDR* threshold of 0.0001 (to be compared with Figure 2). At first sight, the counter-expression trend as seen when pooling all strains together (as in Figure 2) seems lost (see also Suppl. Figure 21). However, analysis of the CEI index (obtained using only the significant DE genes, i.e. the ones placed beyond the DEI(ISO-CTR) thresholds, vertical dashed lines) shows that the anti-correlation trend, while considering only these significantly DE genes, is restored. This result holds at various *FDR* thresholds, also in comparison with reshuffled DEI values (see Suppl. Figures 22-25).

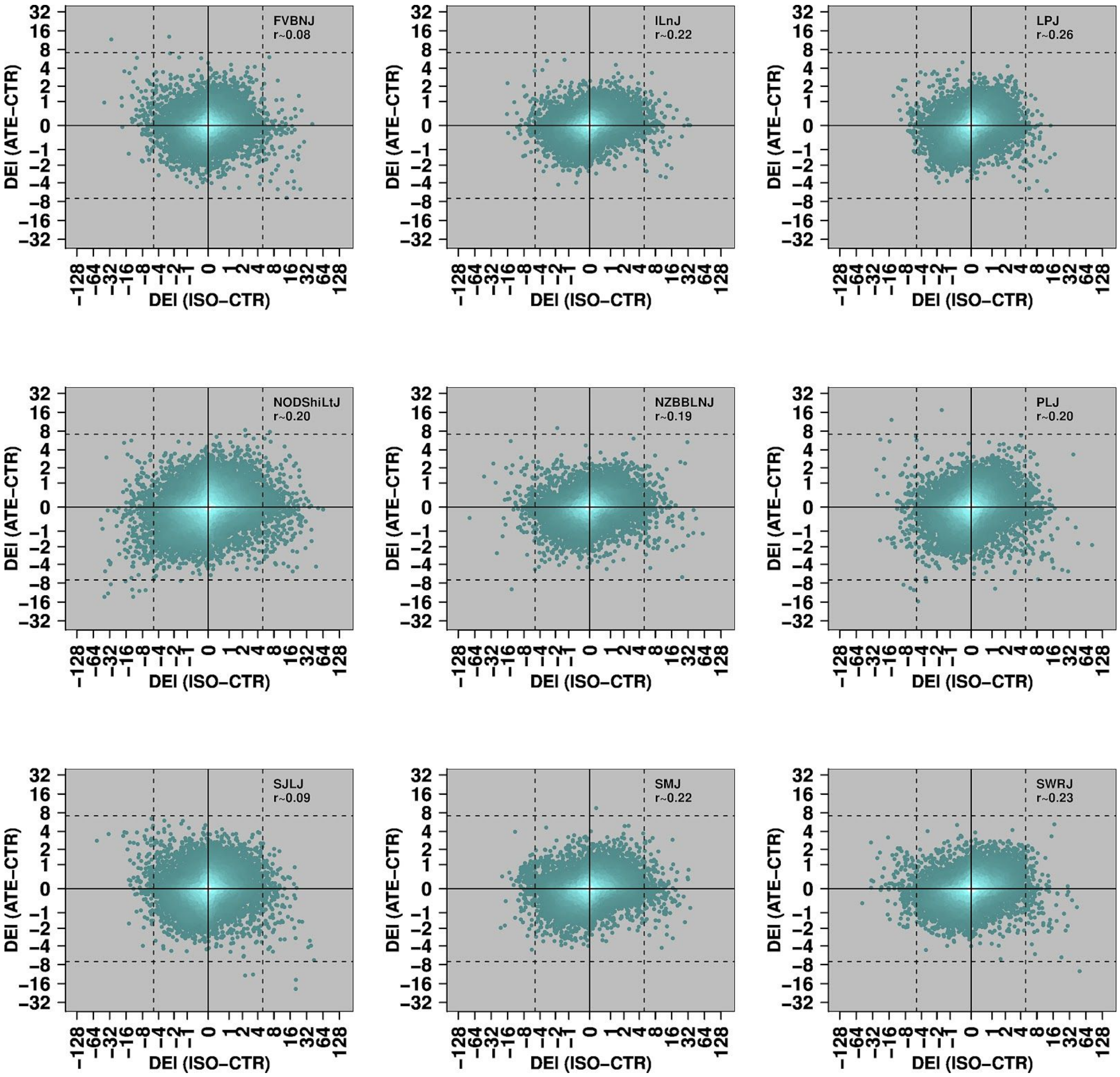

Supplementary Figure 21: Trend of the DE index distribution for each strain (see caption of Suppl. Figure 20).

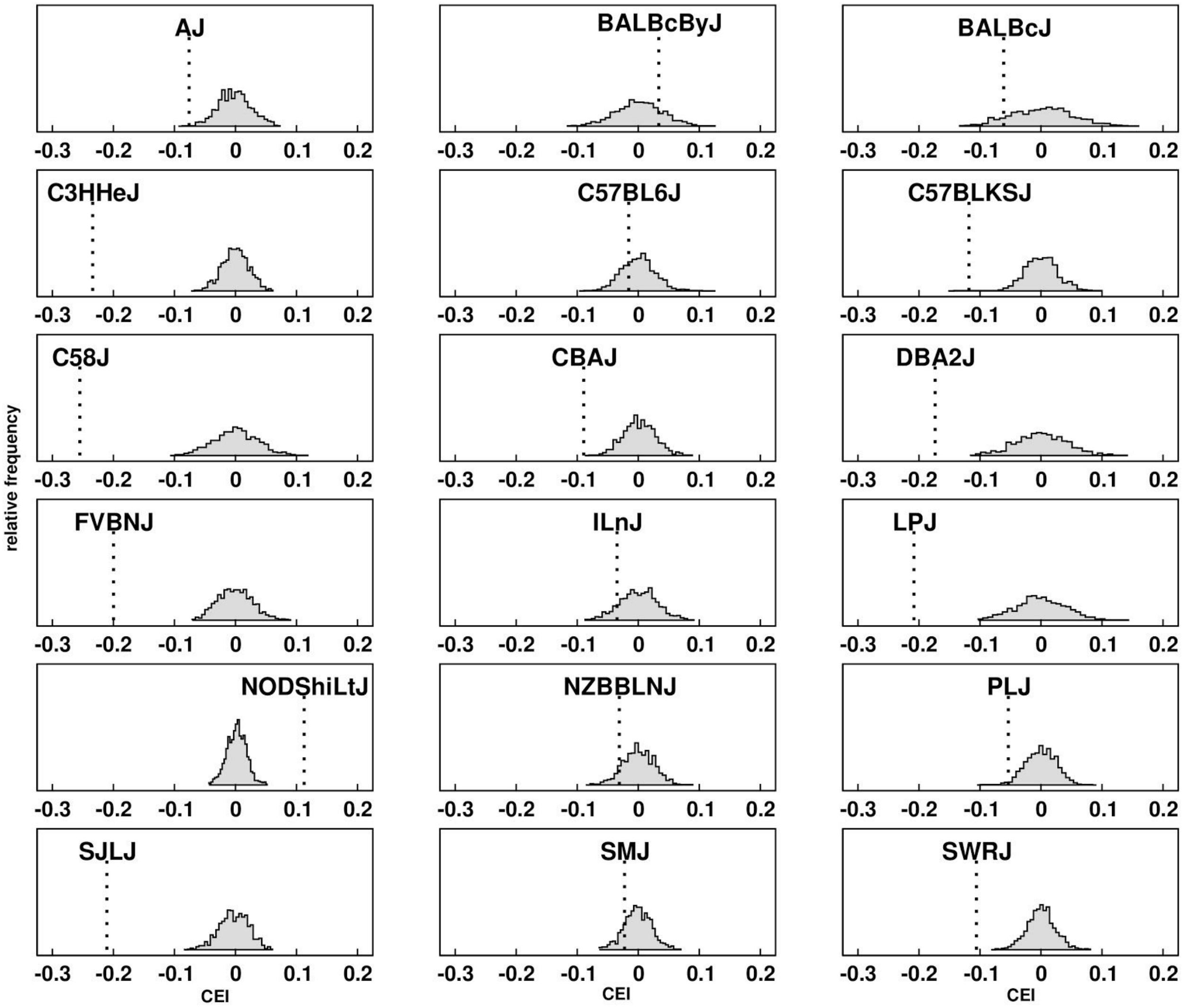

Supplementary Figure 22: CEI(4) values, i.e. counter-expression index at a *FDR* level (for DE genes definition) <0.0001. For each strain, the actual index (dotted line) is shown in comparison with a sample obtained by randomizing the genes. 16 of the 18 strains show a negative CEI at *t*=4, while only two strains (BALBcBy/J and NOD/ShiltJ) show a positive index with respect to their respective controls. This indicates that the anti-correlation trend between DEI(ISO-CTR) and DEI(ATE-CTR) seen in Figure 2 is still substantial (although weaker than when pooling all strains together) if we consider only the significantly DE genes.

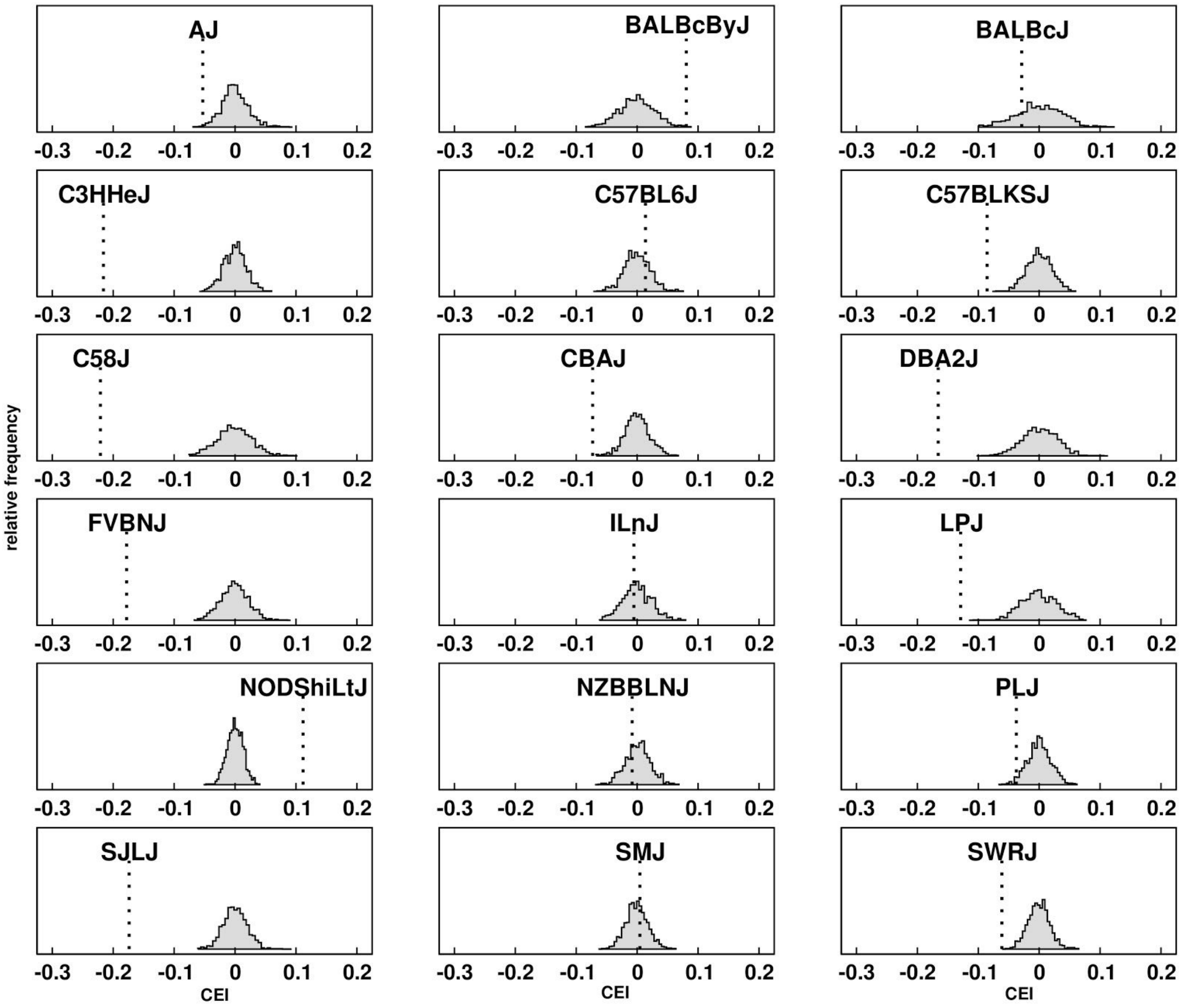

Supplementary Figure 23: CEI(3). Lowering the definition of DE genes to *t*=3, another two strains (C57BL6/J and SM/J) show a positive CEI (with respect to their control), while CEIs remain negative in the other 14 strains.

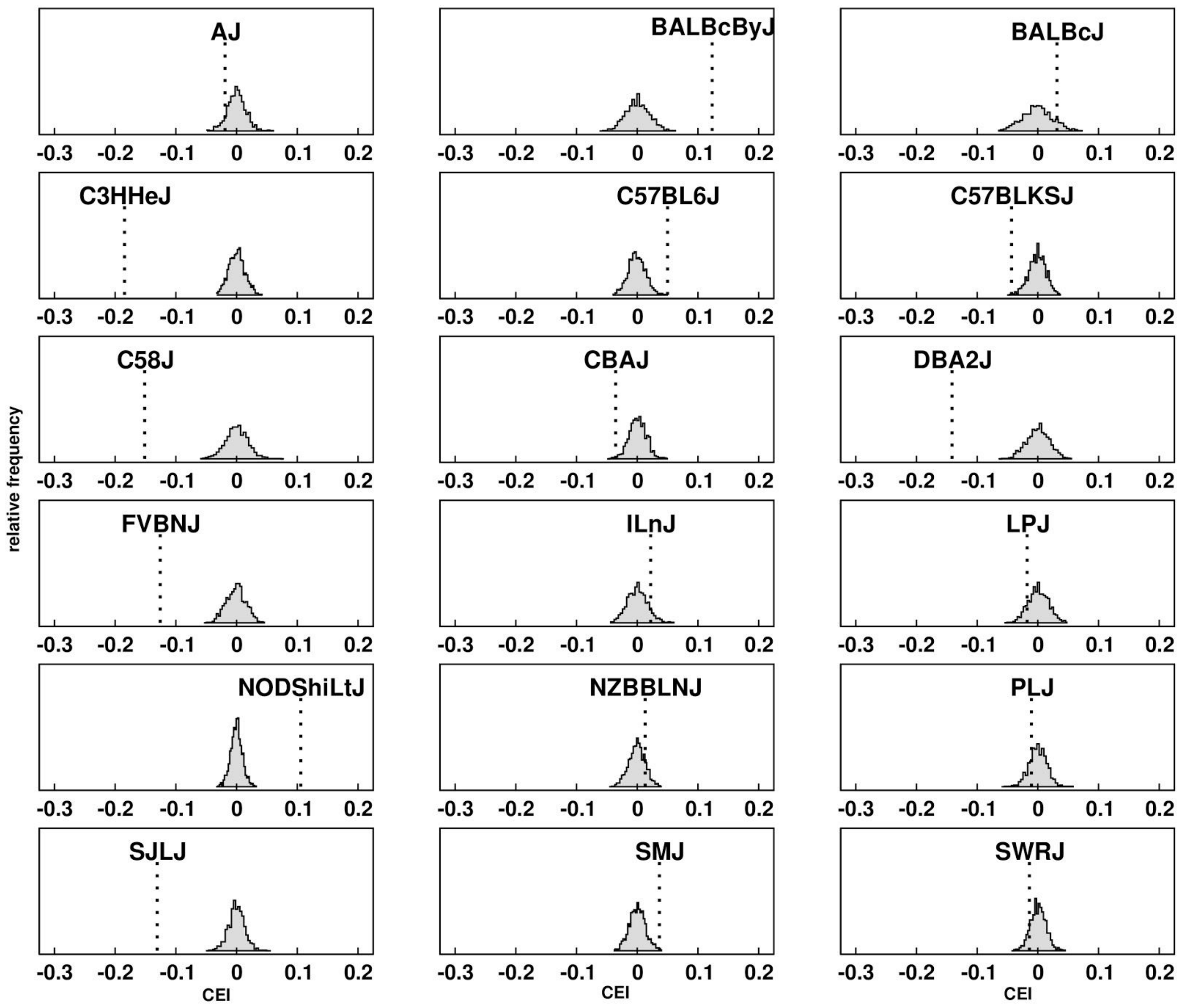

Supplementary Figure 24: CEI(2). Lowering the definition of DE genes further to *t*=2, another three strains (BALBc/J, ILn/J and NZBBLN/J) show a positive CEI (with respect to their control), while CEIs remain negative in the other 11 strains.

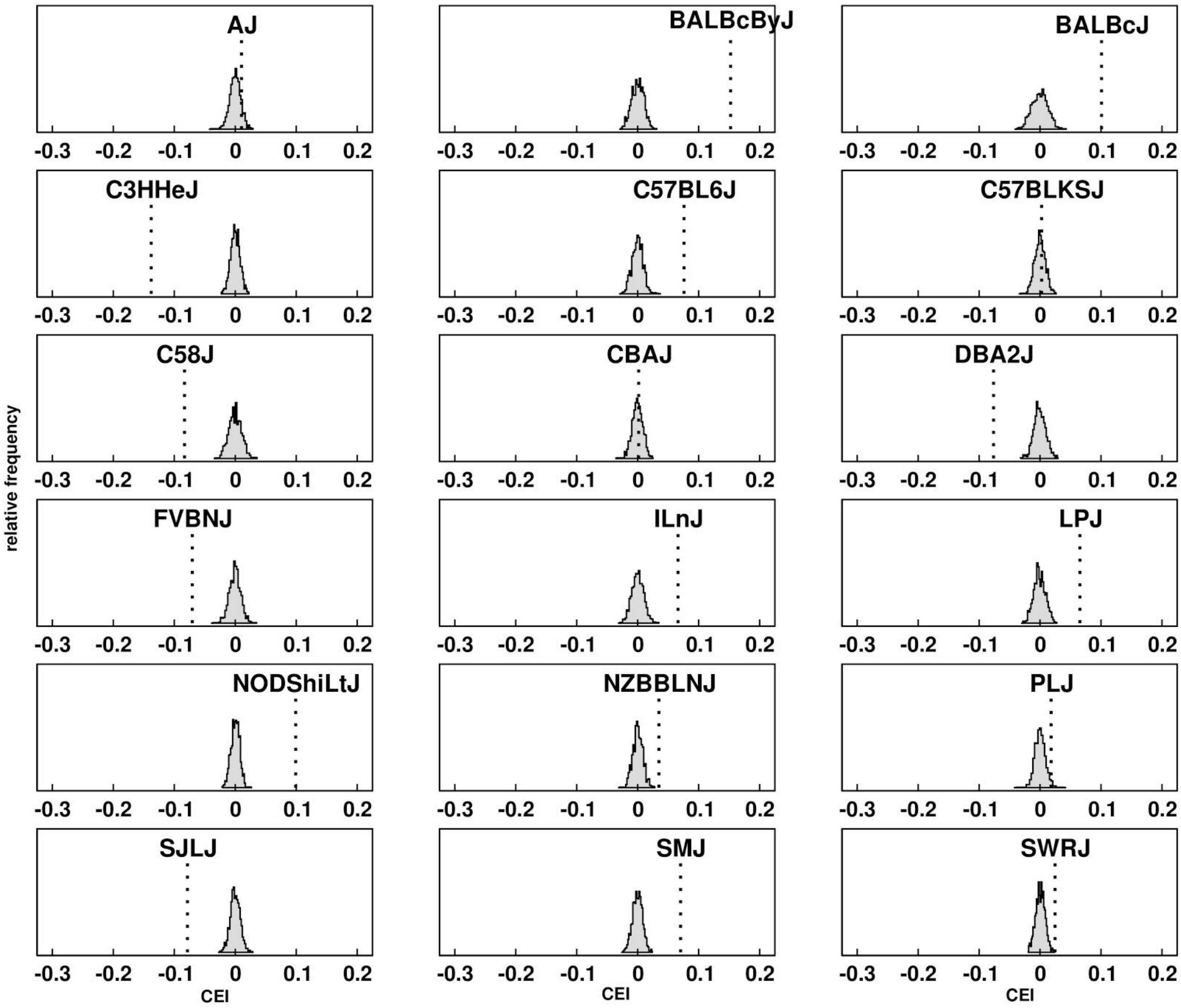

Supplementary Figure 25: CEI(1). 12 of the 18 strains show a negative CEI at *t*=1. However, the DE genes definition at this point is very loose.

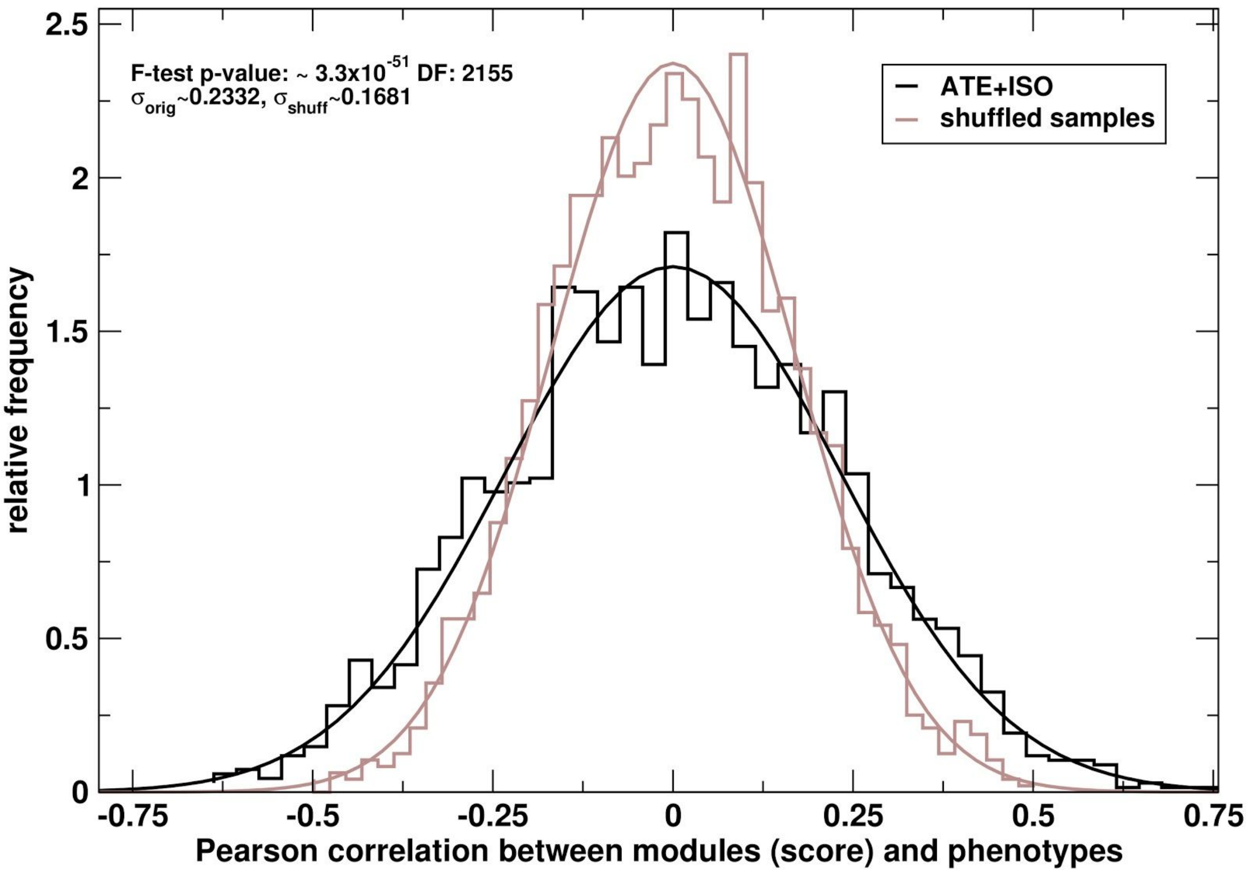

Supplementary Figure 26: Detailed statistics of Figure 4. Black line, distribution of the correlations between the modules (containing both ATE and ISO samples) and the selected phenotypes. Brown line, the same, after reshuffling the names of the phenotypes.

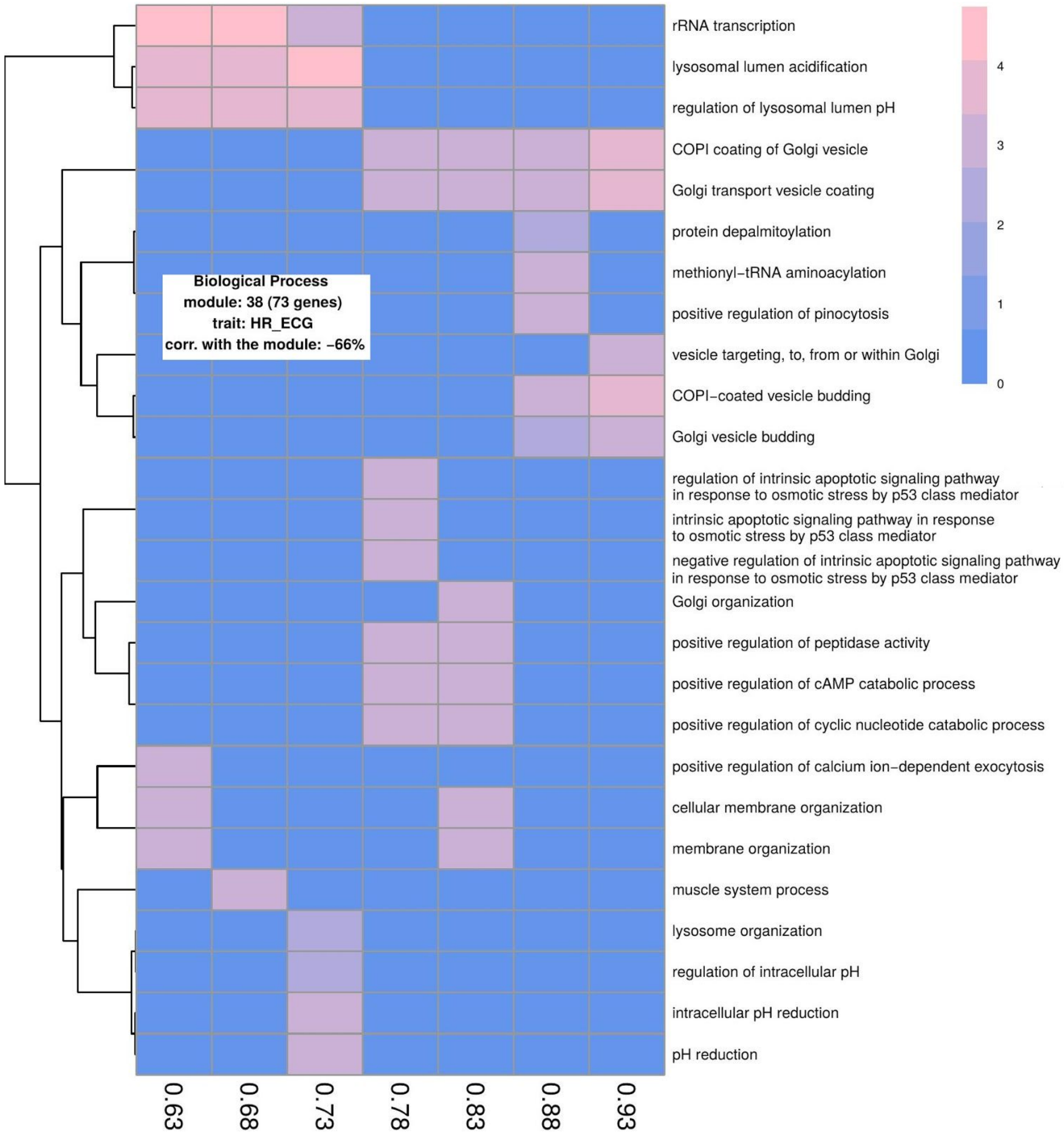

Supplementary Figure 27: BP GO categories of module 38, the module with the strongest anti-correlation to HR ECG. This representation is a combination of a plot similar to the ones presented in Suppl. Figures 10-17 and a heat-map. Along the the *x*-axis (columns), the progressively more stringent gene score threshold is shown (step score: 0.05). The color code indicates the significance ($-\log_{10}p$-value) of the GO categories shown in the *y*-axis, related to the genes of the module with a score greater than or equal to the value indicated in the respective columns. The rows are clustered to underline a similar behavior along the gene score range (which is specific of each module). GO term *p*-values were recovered with GOSeq.

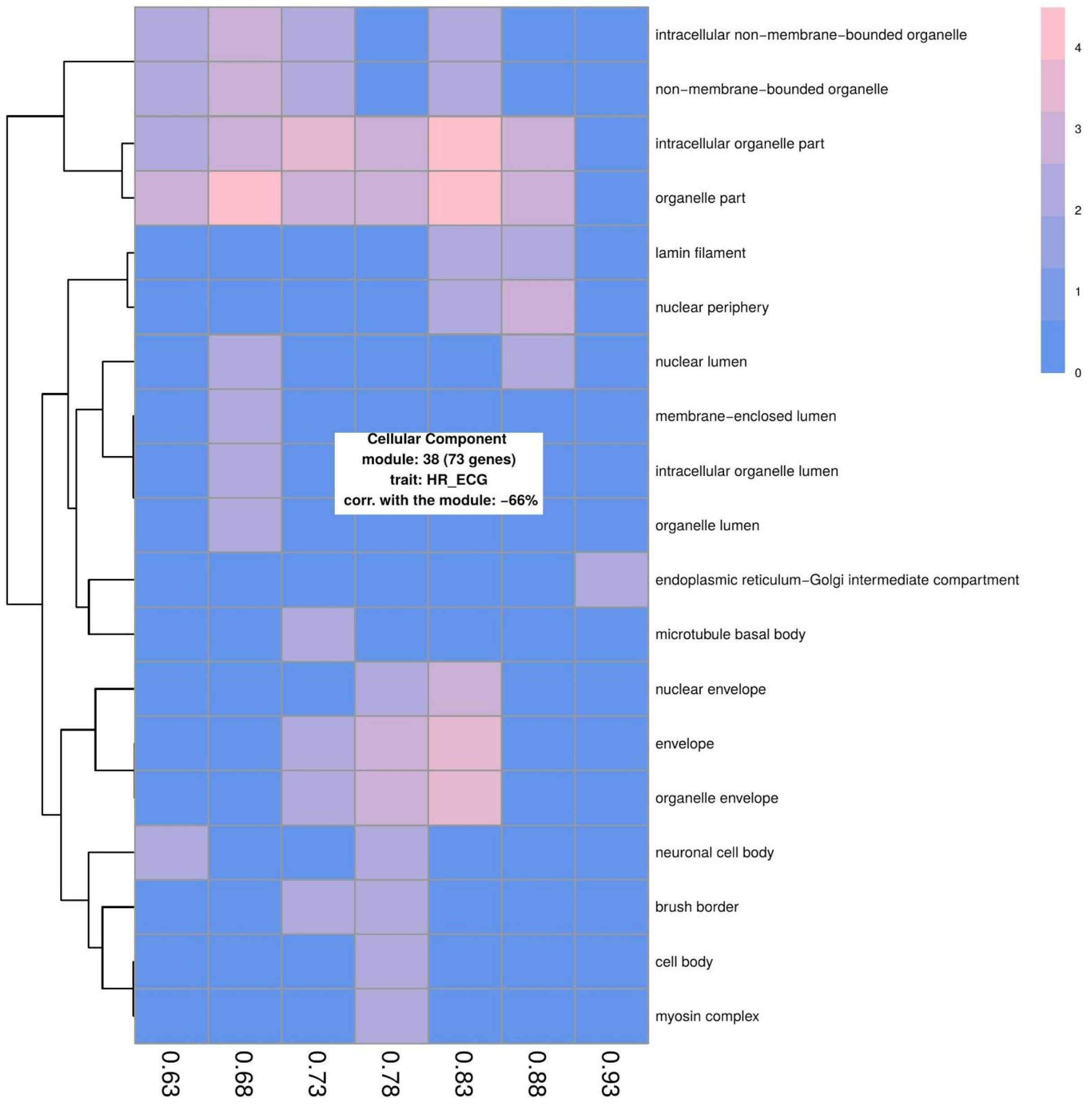

Supplementary Figure 28: CC GO categories of module 38, the module with the strongest anti-correlation to HR ECG.

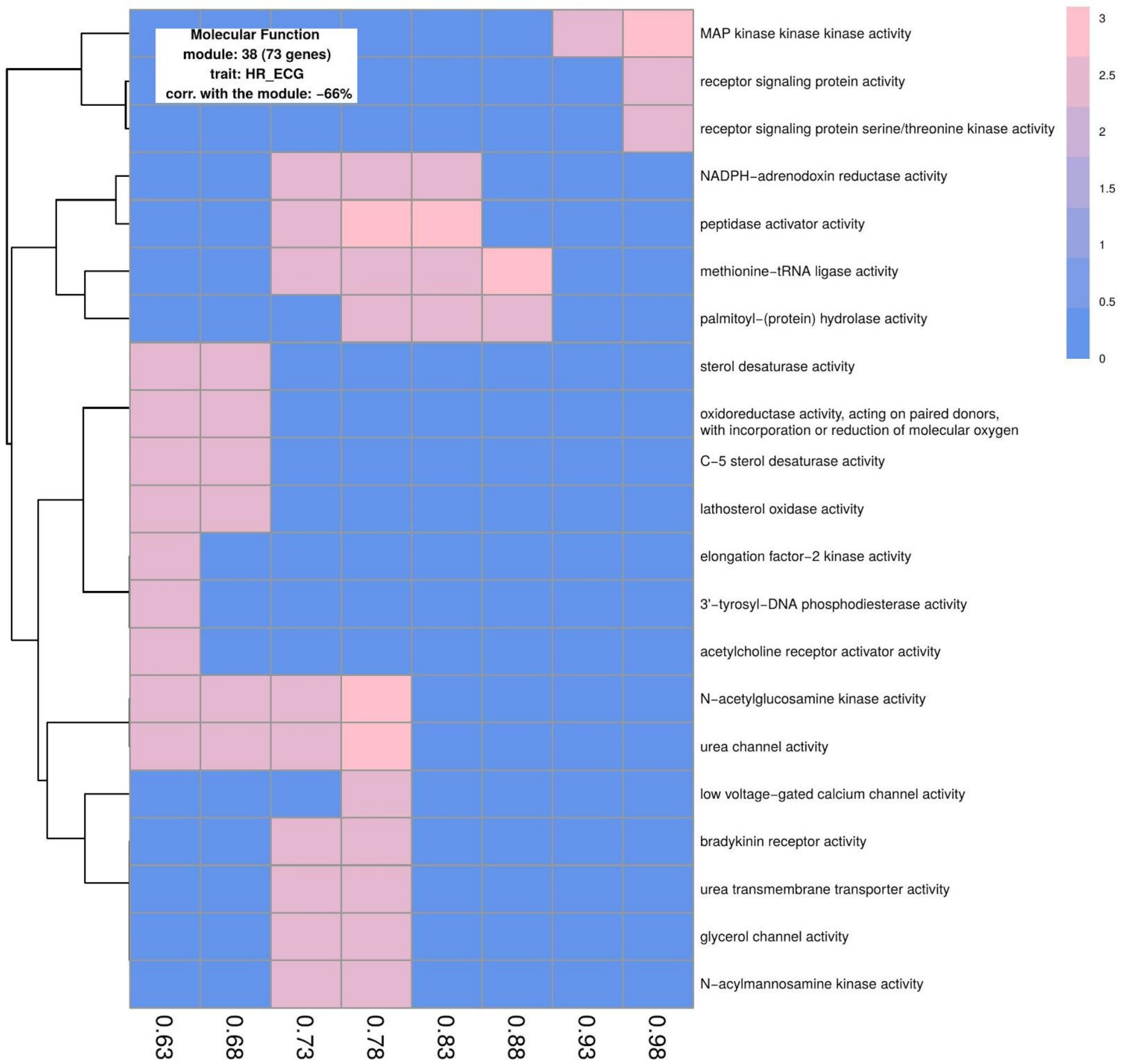

Supplementary Figure 29: MF GO categories of module 38, the module with the strongest anti-correlation to HR ECG.

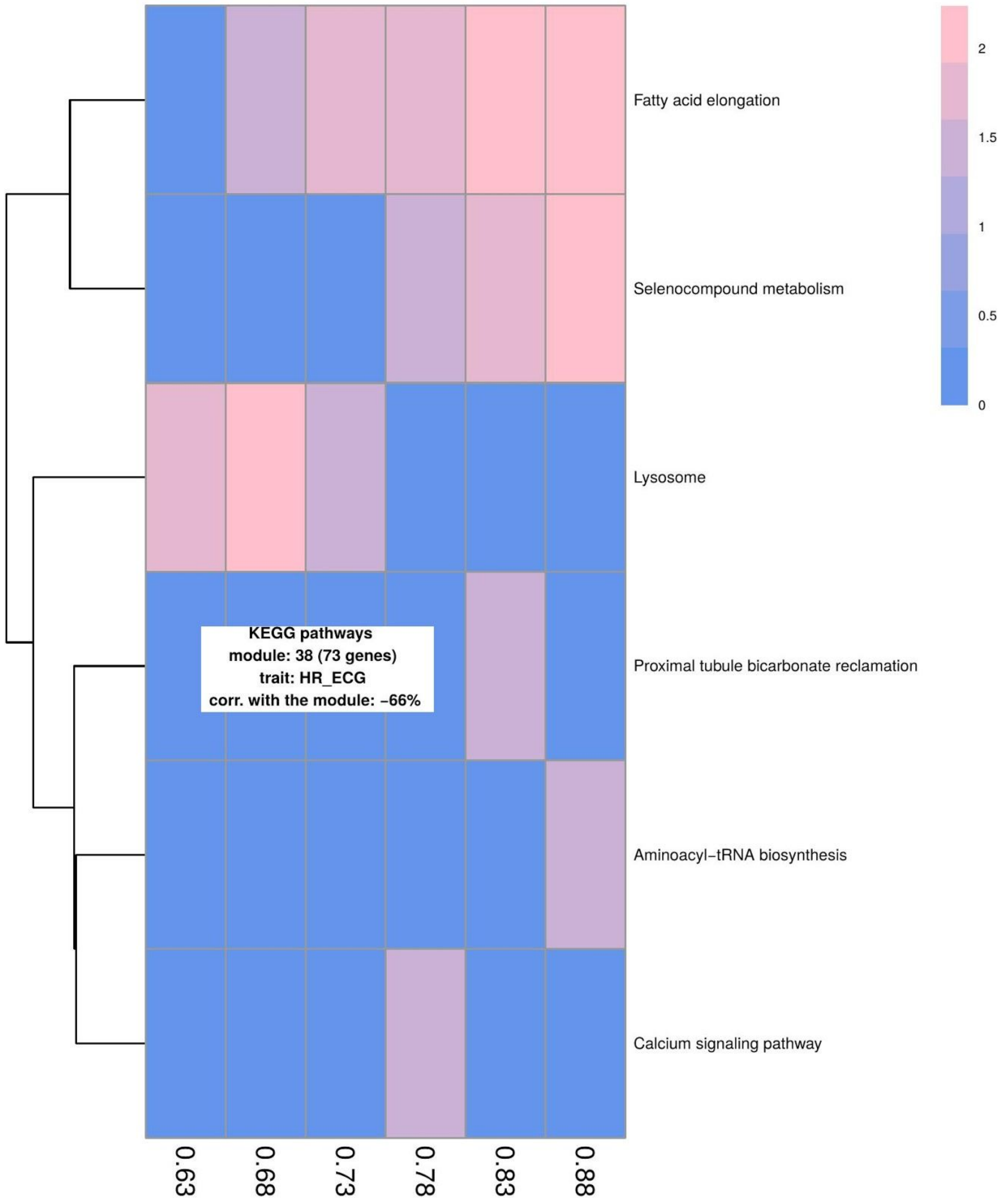

Supplementary Figure 30: KEGG pathways of module 38, the module with the strongest anti-correlation to HR ECG.

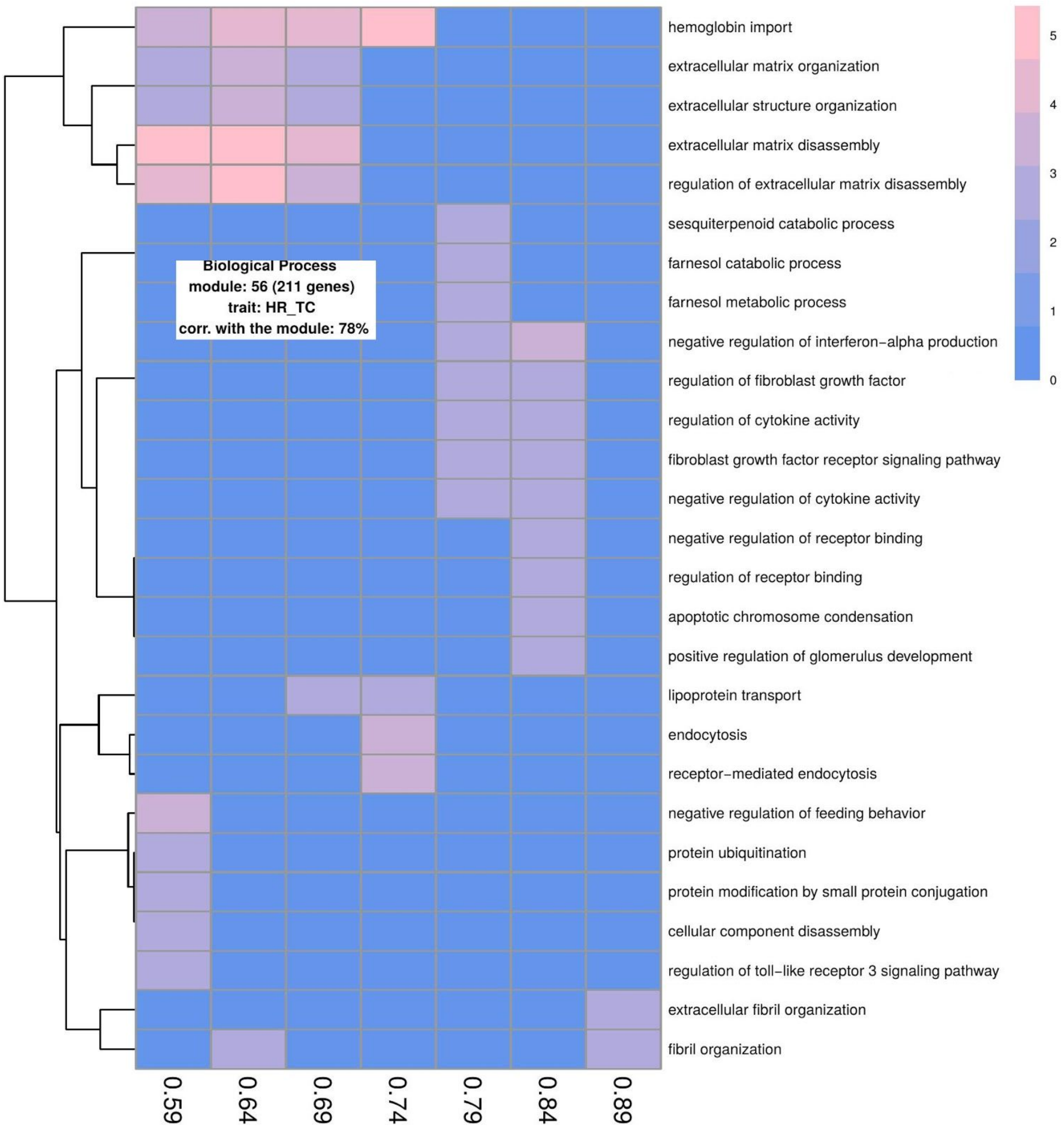

Supplementary Figure 31: BP GO categories of module 56, the module with the best correlation with HR (as measured both by tail-cuff and ECG).

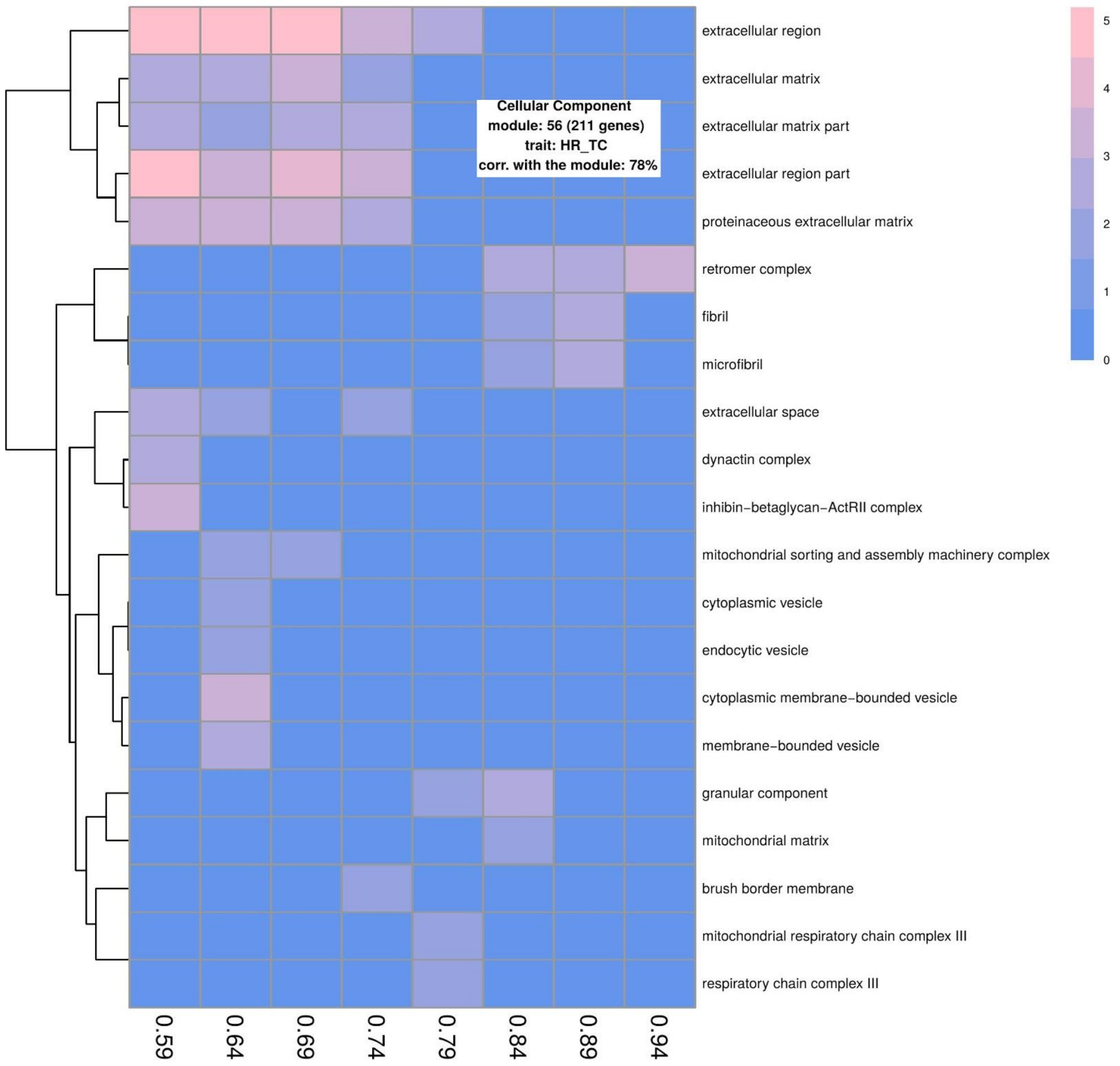

Supplementary Figure 32: CC GO categories of module 56, the module with the best correlation with HR (as measured both by tail-cuff and ECG).

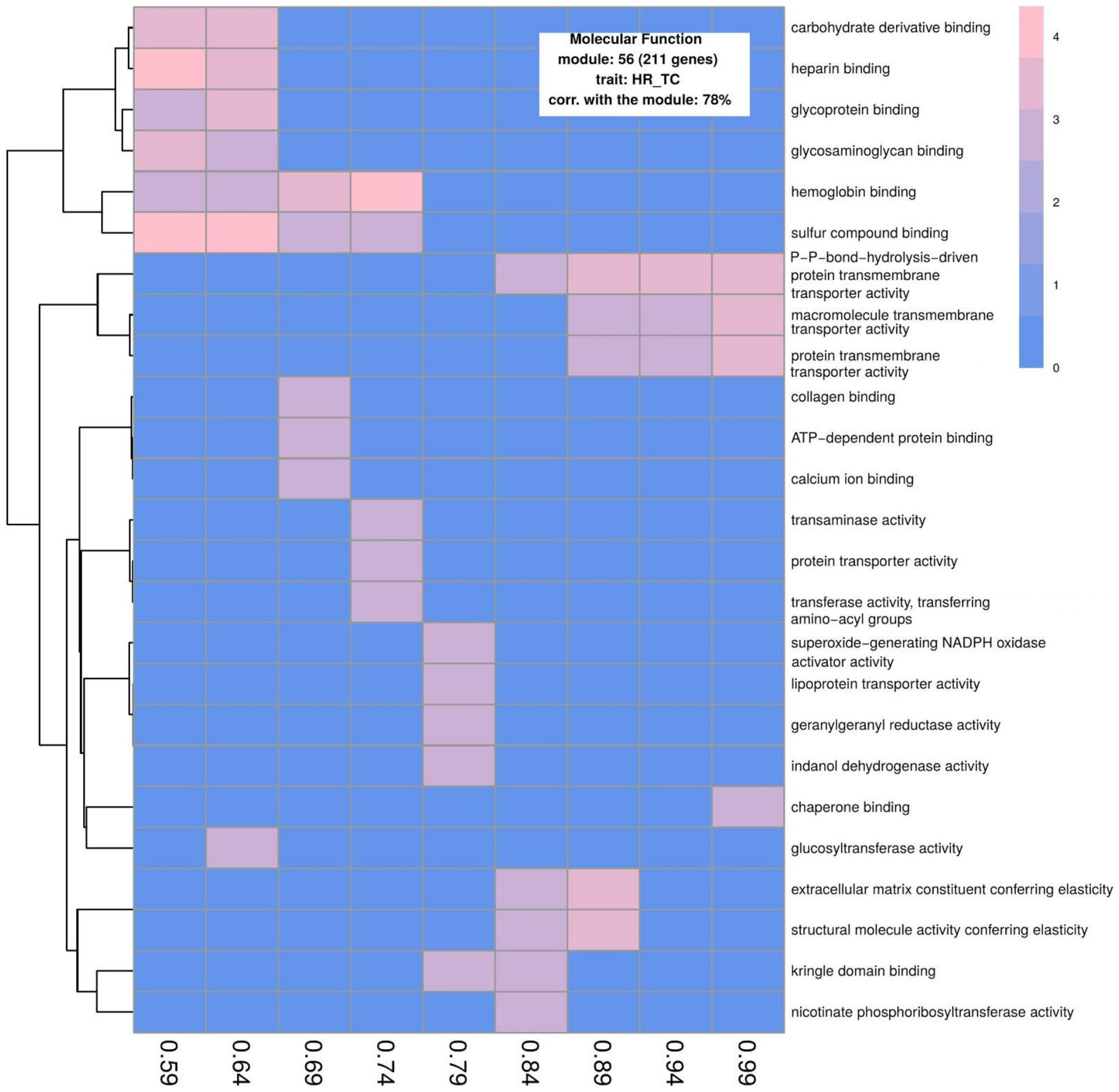

Supplementary Figure 33: MF GO categories of module 56, the module with the best correlation with HR (as measured both by tail-cuff and ECG).

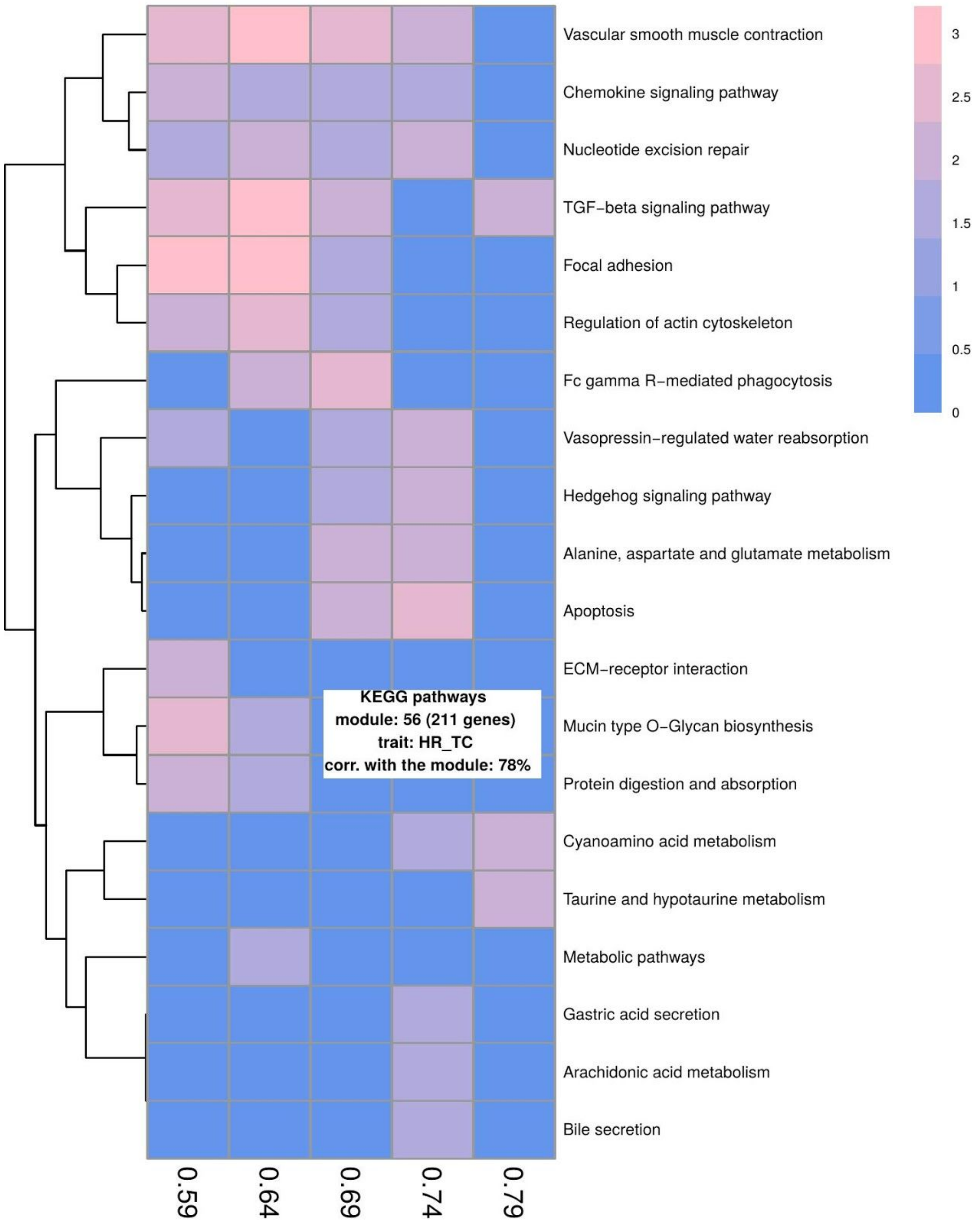

Supplementary Figure 34: KEGG pathways of module 56, the module with the best correlation with HR (as measured both by tail-cuff and ECG).

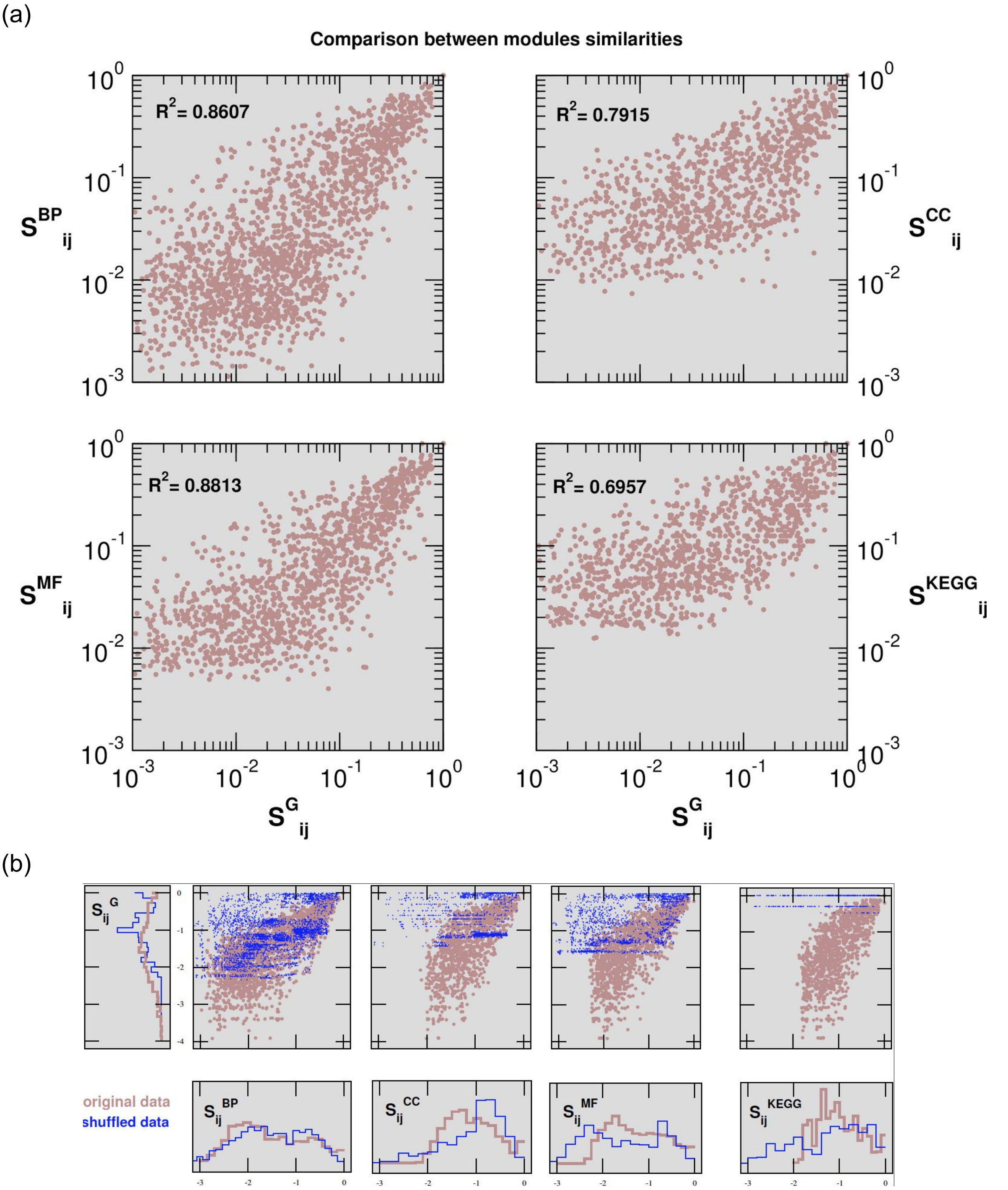

Supplementary Figure 35: Comparison between module similarities based on the Jaccard indices for gene content, S^G_{ij}, and annotation, S^A_{ij} (where A can be either BP, CC, MF or KEGG). (a) Plotting these coefficients for each pair of modules (i,j) reveals that these quantities are not very strongly correlated. In particular, some pairs may overlap only mildly with respect to genes (say S^G_{ij} <0.3), yet display sizable functional overlap (S^A_{ij} >0.5). (b) Yet, the distributions and, in particular, their correlation, are markedly different from what is obtained by a control experiment using reshuffled data (shown in blue).

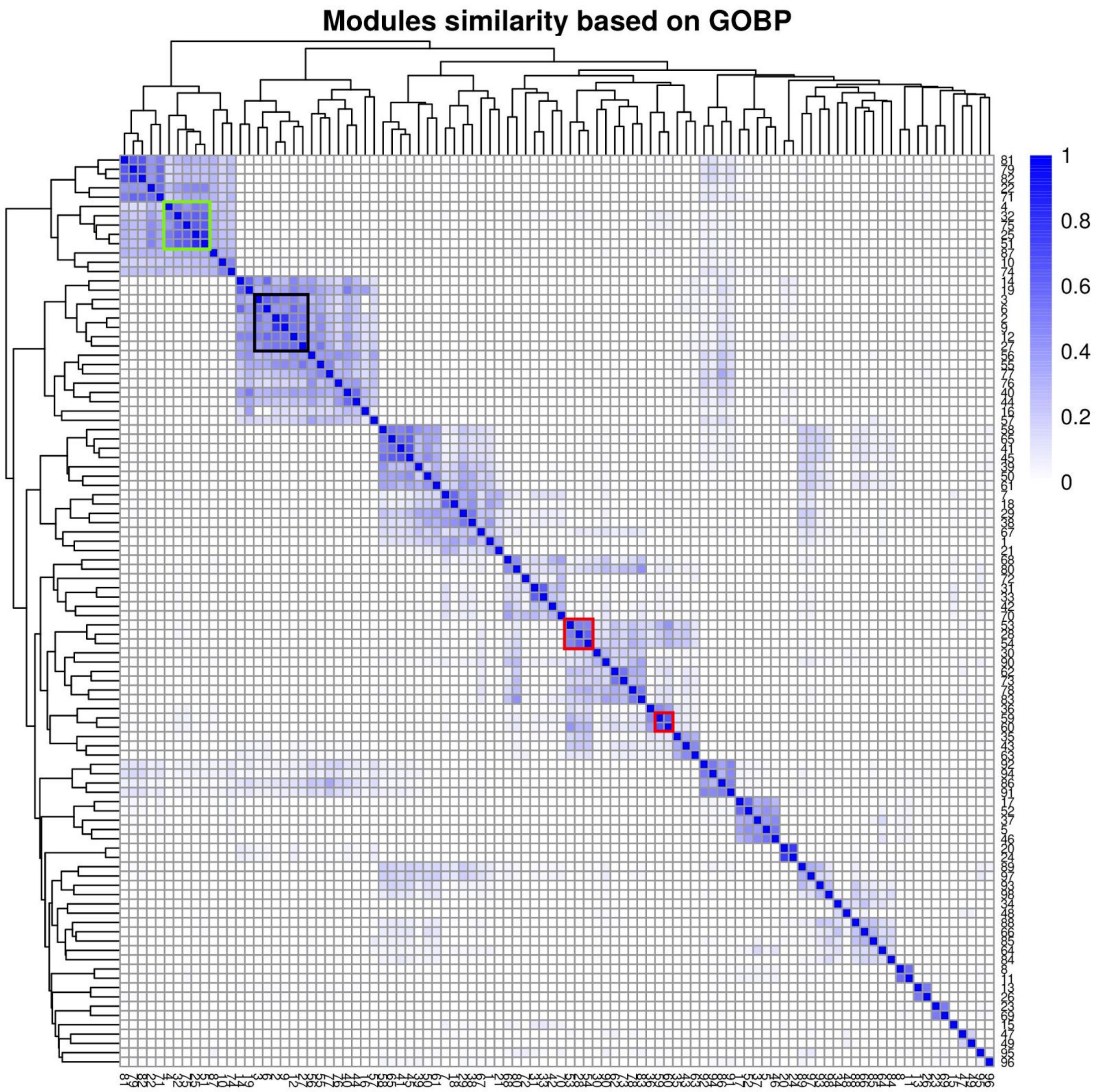

Figure 36: Jaccard similarity among the modules in terms of shared BP GO categories. The presence of a superstructure among the modules is evidenced by clustering the matrix. The 3 macro-modules identified by ISA, shared with the other GO categories, are indicated by the open squares. Macro-module “antigen” (green square, 5 modules) displays an internal average Jaccard similarity of 0.643. For macro-module “fibroblast” (black square, 6 modules), the average similarity is 0.556, whereas for macro-module “cardiac” (red square, 5 modules), it is 0.441. The names of the macro-modules were given after detailed examination of the GO entries reported in Suppl. Figures 39-50.

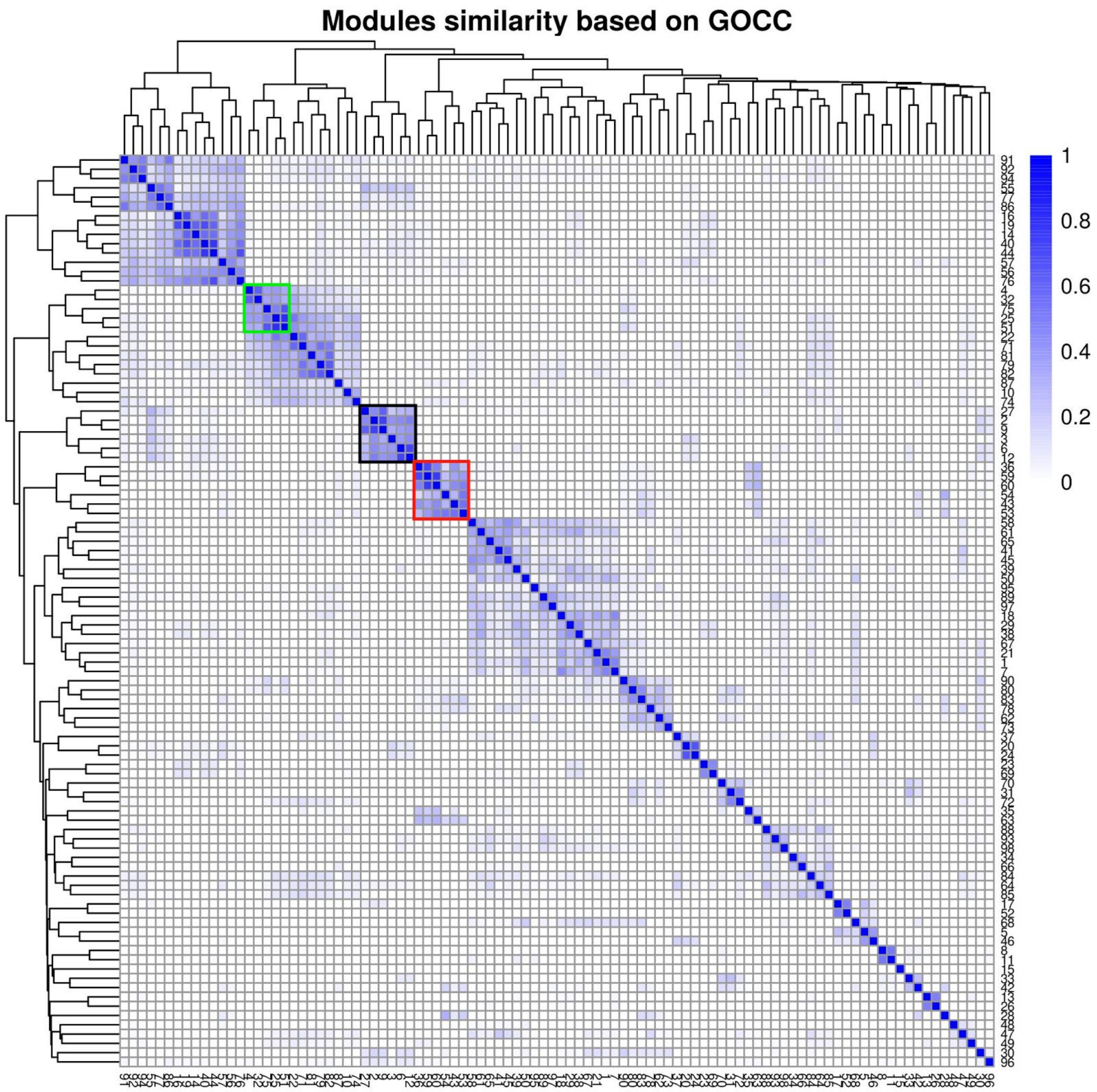

Figure 37: Jaccard similarity among the modules in terms of shared CC GO categories. The presence of a superstructure among the modules is evidenced by clustering the matrix. The 3 macro-modules identified by ISA, shared with the other GO categories, are indicated by the open squares. Macro-module "MHC-complex" (green square, 6 modules) displays an internal average Jaccard similarity of 0.465. For macro-module "cytoskeleton" (black square, 6 modules), the average similarity is 0.481, whereas for macro-module "organelle" (red square, 5 modules), it is 0.548.

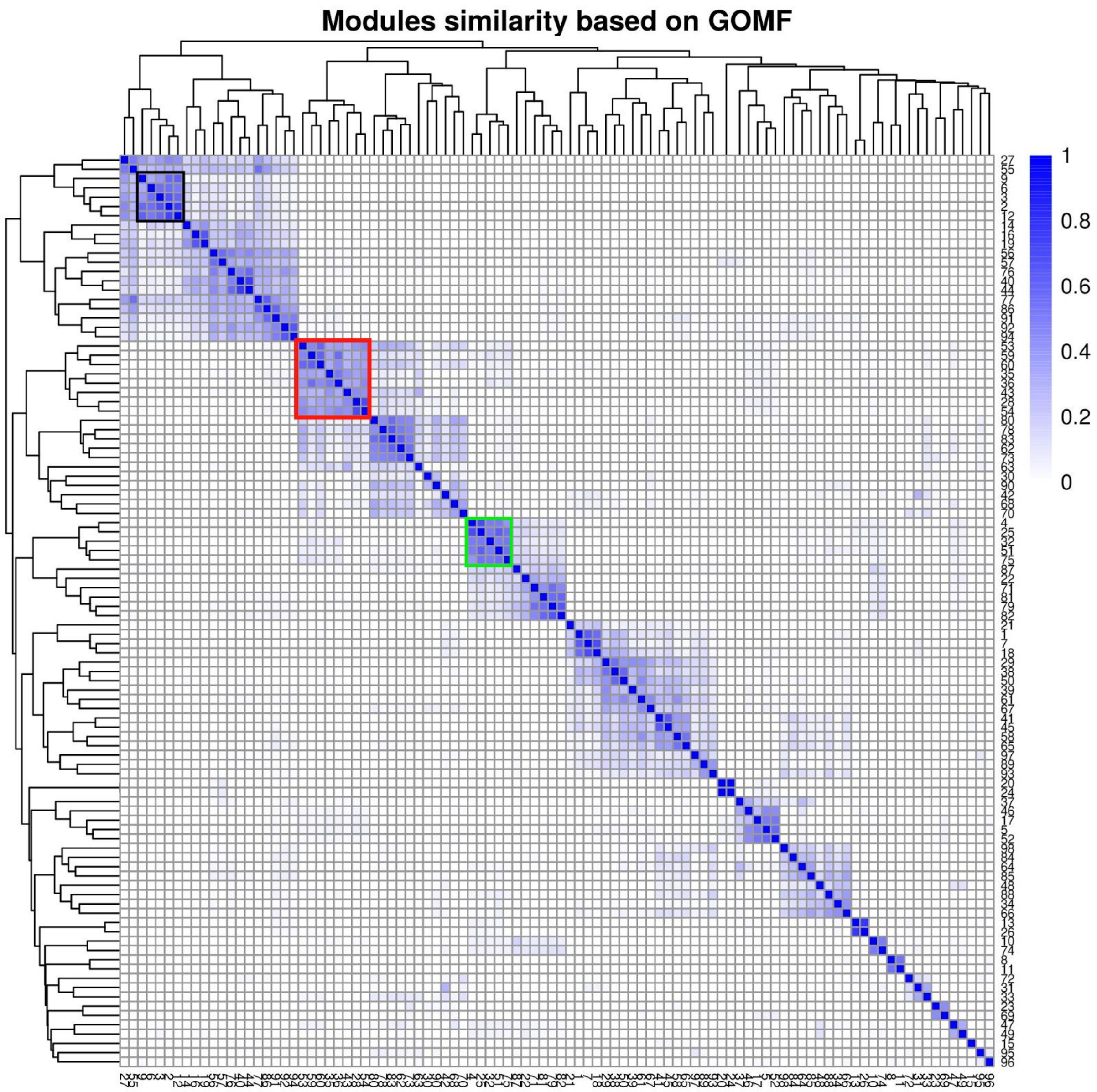

Figure 38: Jaccard similarity among the modules in terms of shared MF GO categories. The presence of a superstructure among the modules is evidenced by clustering the matrix. The 3 macro-modules identified by ISA, shared with the other GO categories, are indicated by the open squares. Macro-module “hormone” (green square, 5 modules) displays an internal average Jaccard similarity of 0.610. For macro-module “ribonuclease” (black square, 5 modules), the average similarity is 0.618, whereas for the macro-module “pyrimidine” (red square, 7 modules), it is 0.466.

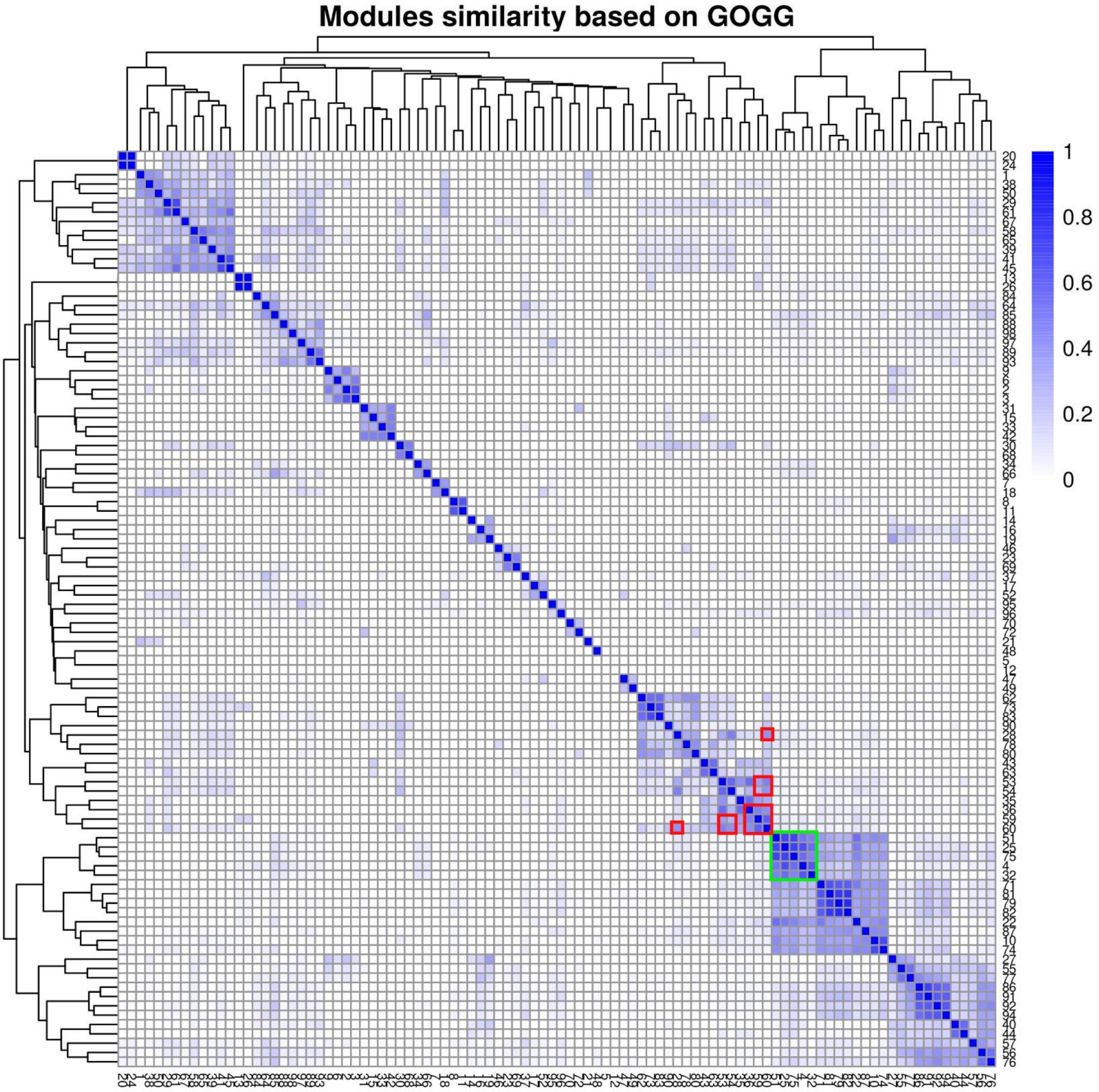

Figure 39: Jaccard similarity among the modules in terms of shared KEGG pathways. The presence of a superstructure among the modules is evidenced by clustering the matrix. The 2 macro-modules identified by ISA, shared with the other GO categories, are indicated by the open squares. Macro-module “Graft” (green square, 5 modules) displays an internal average Jaccard similarity of 0.615, whereas for macro-module “chemokine” (red square, 6 modules), it is 0.430.

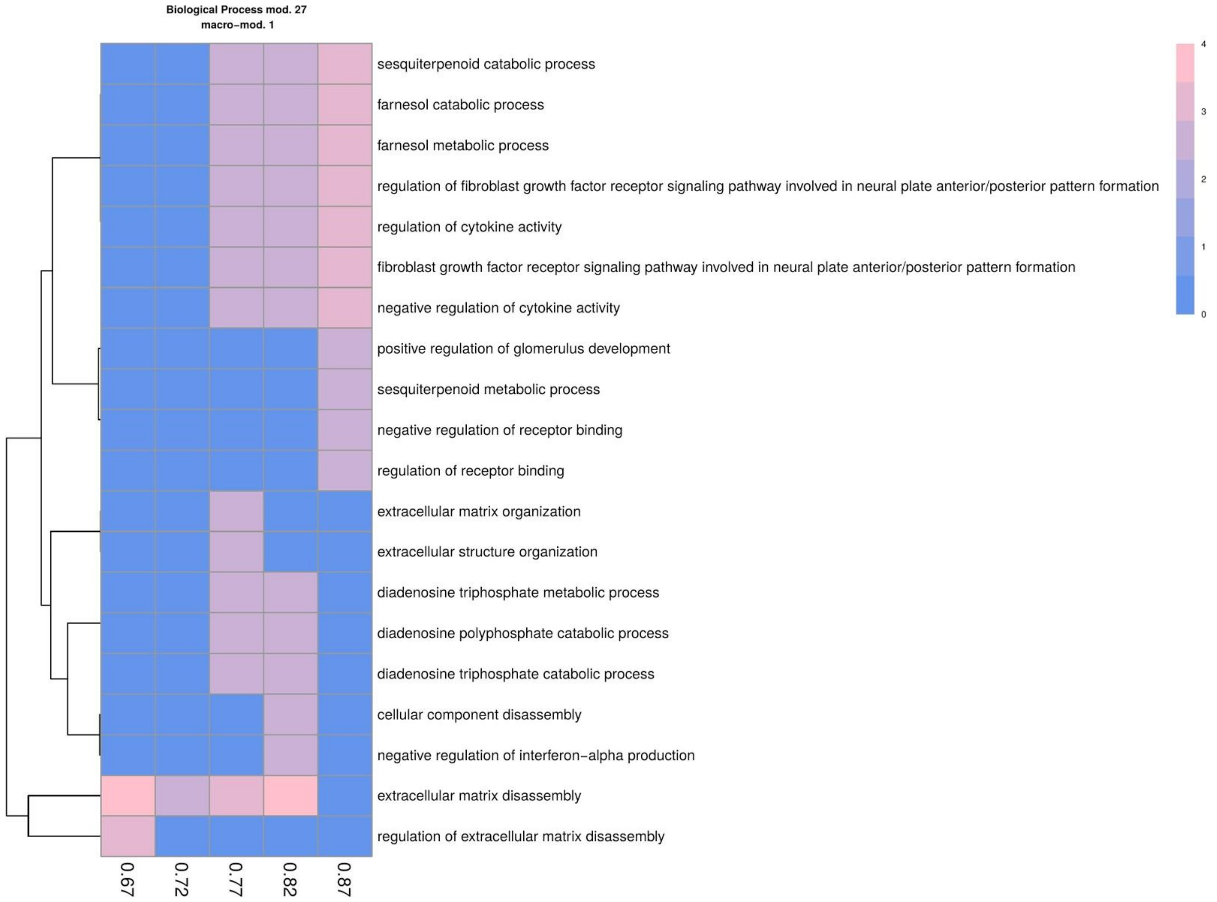

Supplementary Figure 40: BP “fibroblast” macro-module 1 (representative module 27). This is a specimen of the 3 macro-modules shared among all the GO categories. The color-code indicates the significance of the GO category (rows) as a function of the gene score in the module (columns). The categories are shown when the *p*-value is lower than 0.01 in at least one score step.

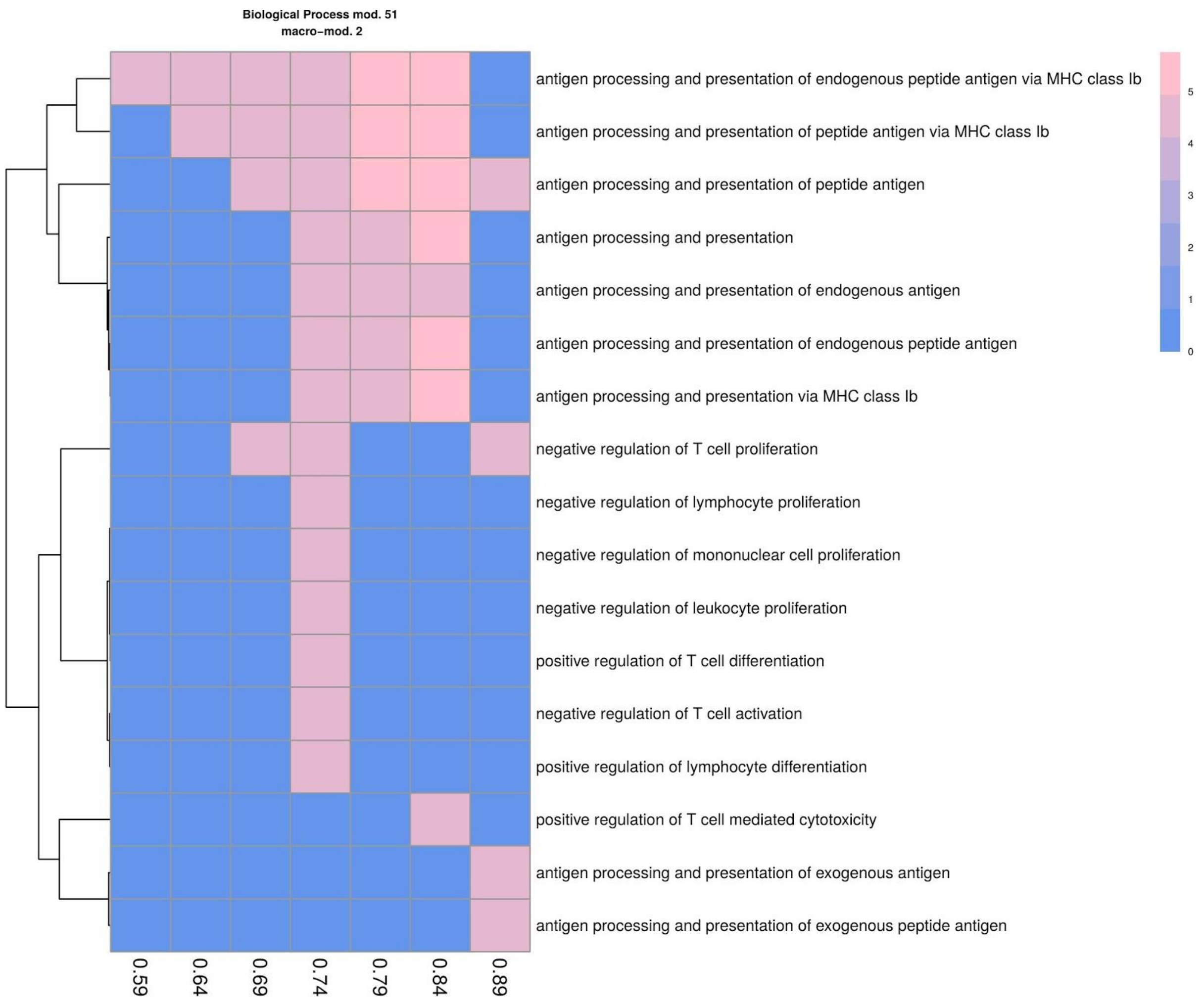

Supplementary Figure 41: BP “antigen processing and presentation” macro-module 2 (representative module 51).

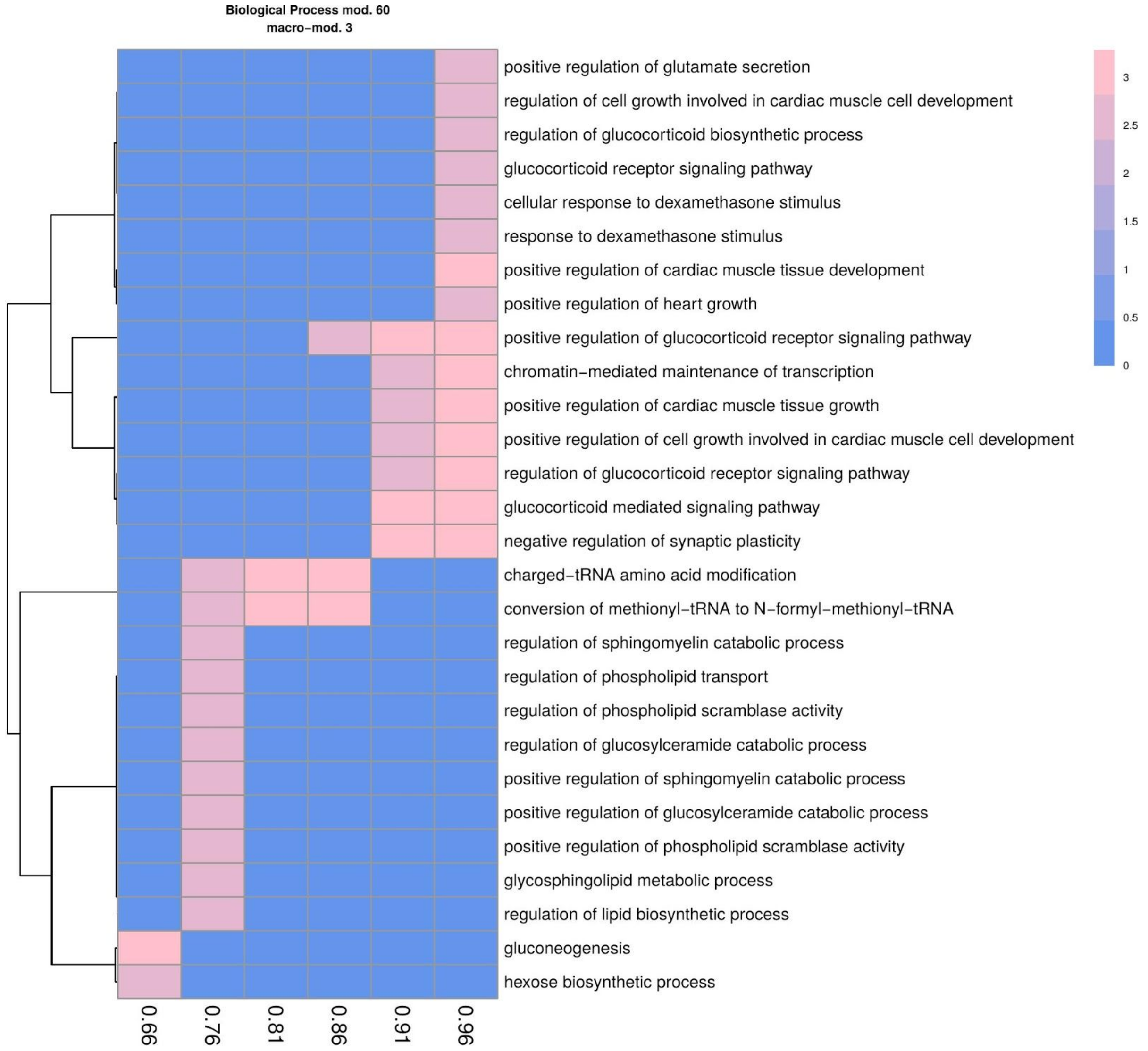

Supplementary FIgure 42 : BP “cardiac” macro-module 3 (representative module 60).

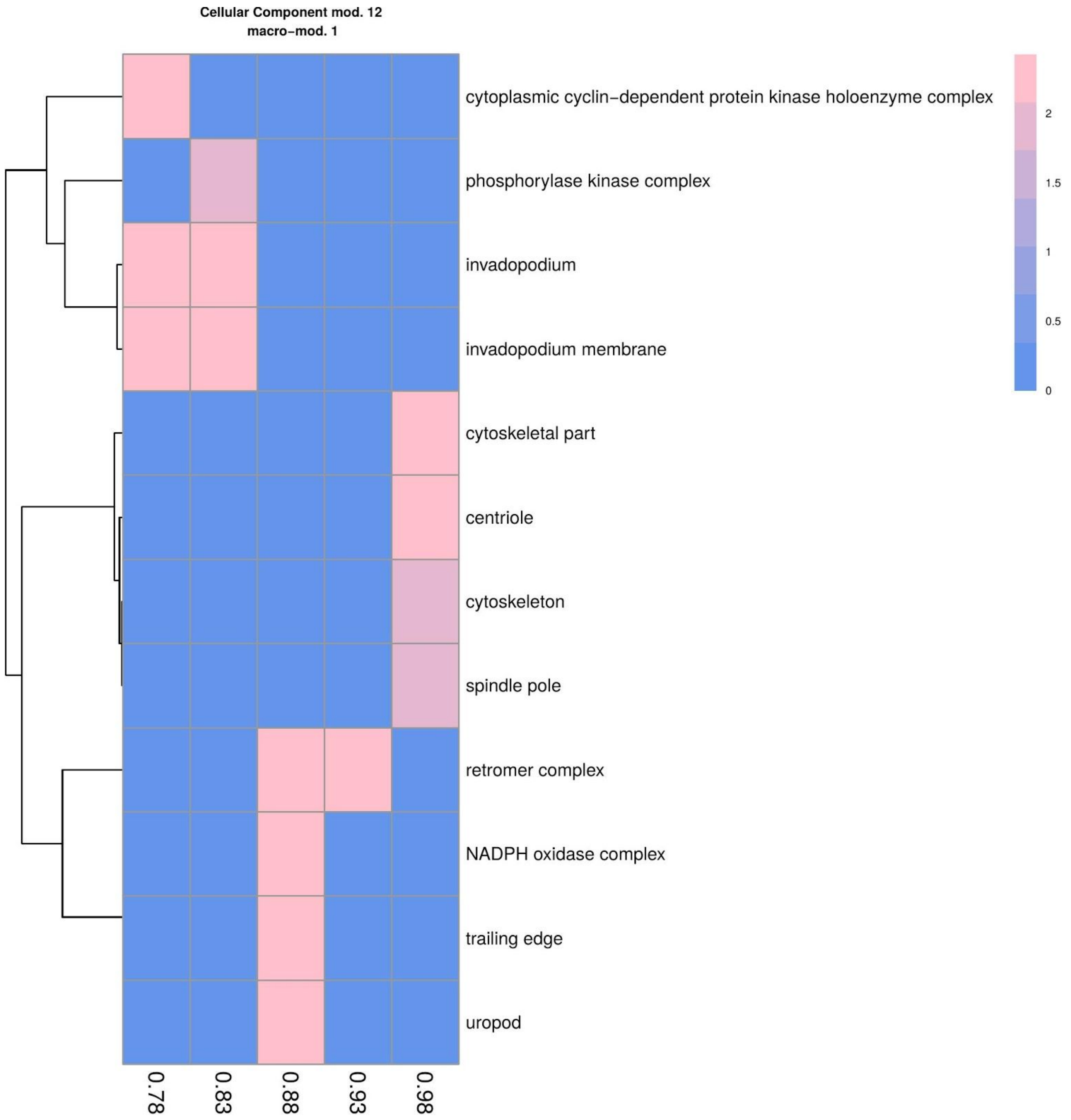

Supplementary Figure 43: CC “cytoskeleton” macro-module 1 (representative module 12).

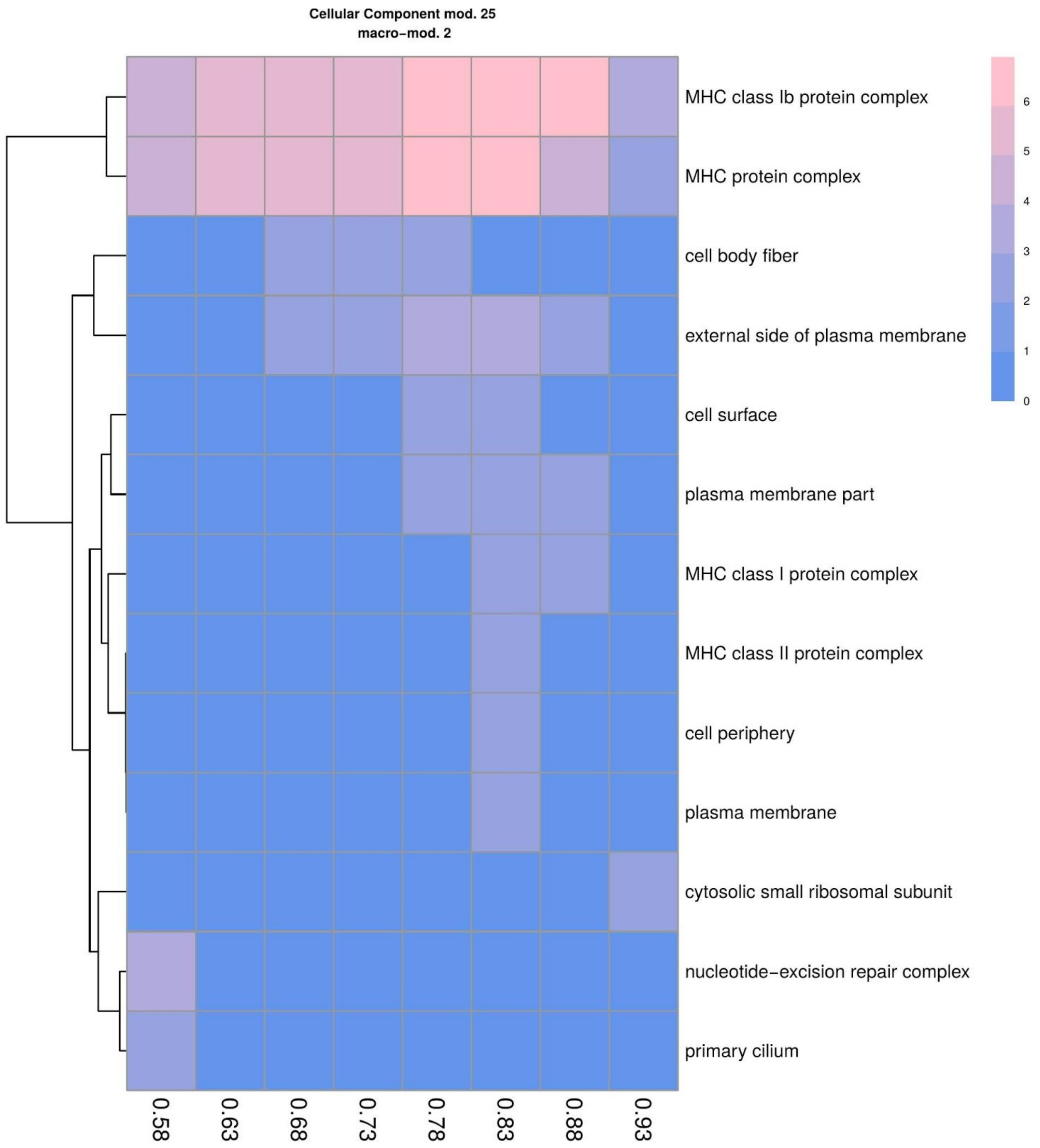

Supplementary Figure 44: CC "MHC-complex" macro-module 2 (representative module 75).

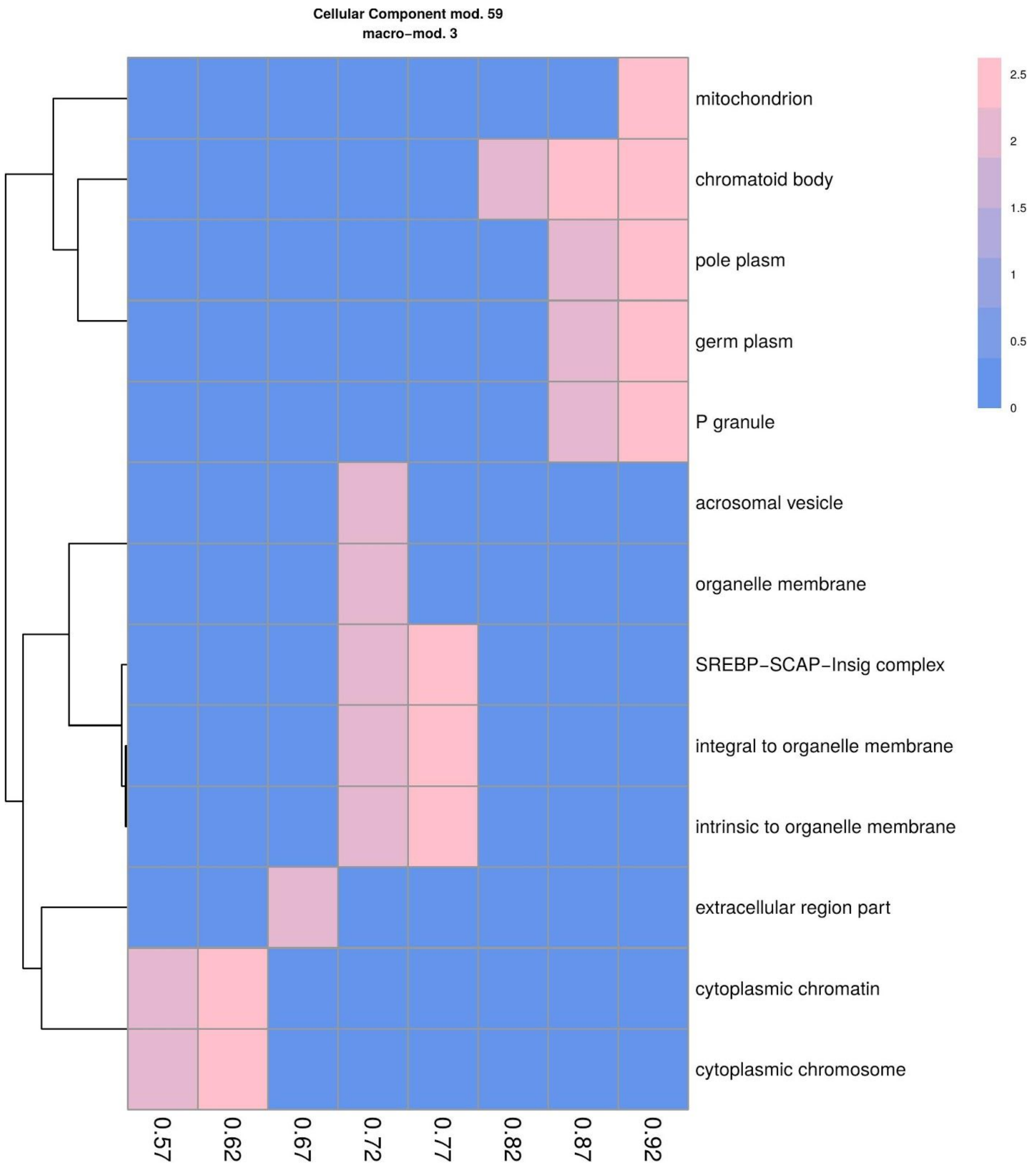

Supplementary Figure 45: CC “organelle” macro-module 3 (representative module 59).

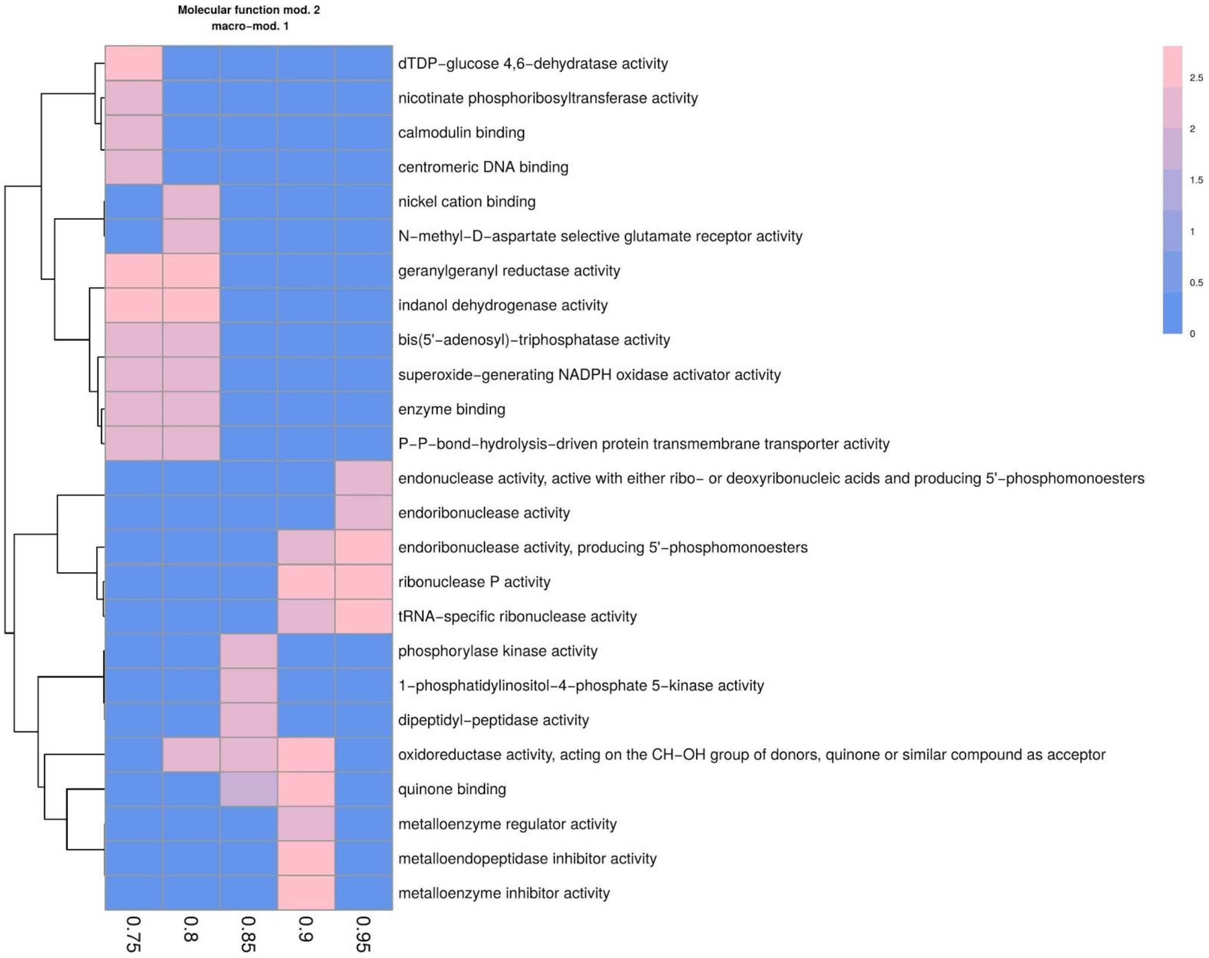

Supplementary Figure 46: MF “ribonuclease” macro-module 1 (representative module 2).

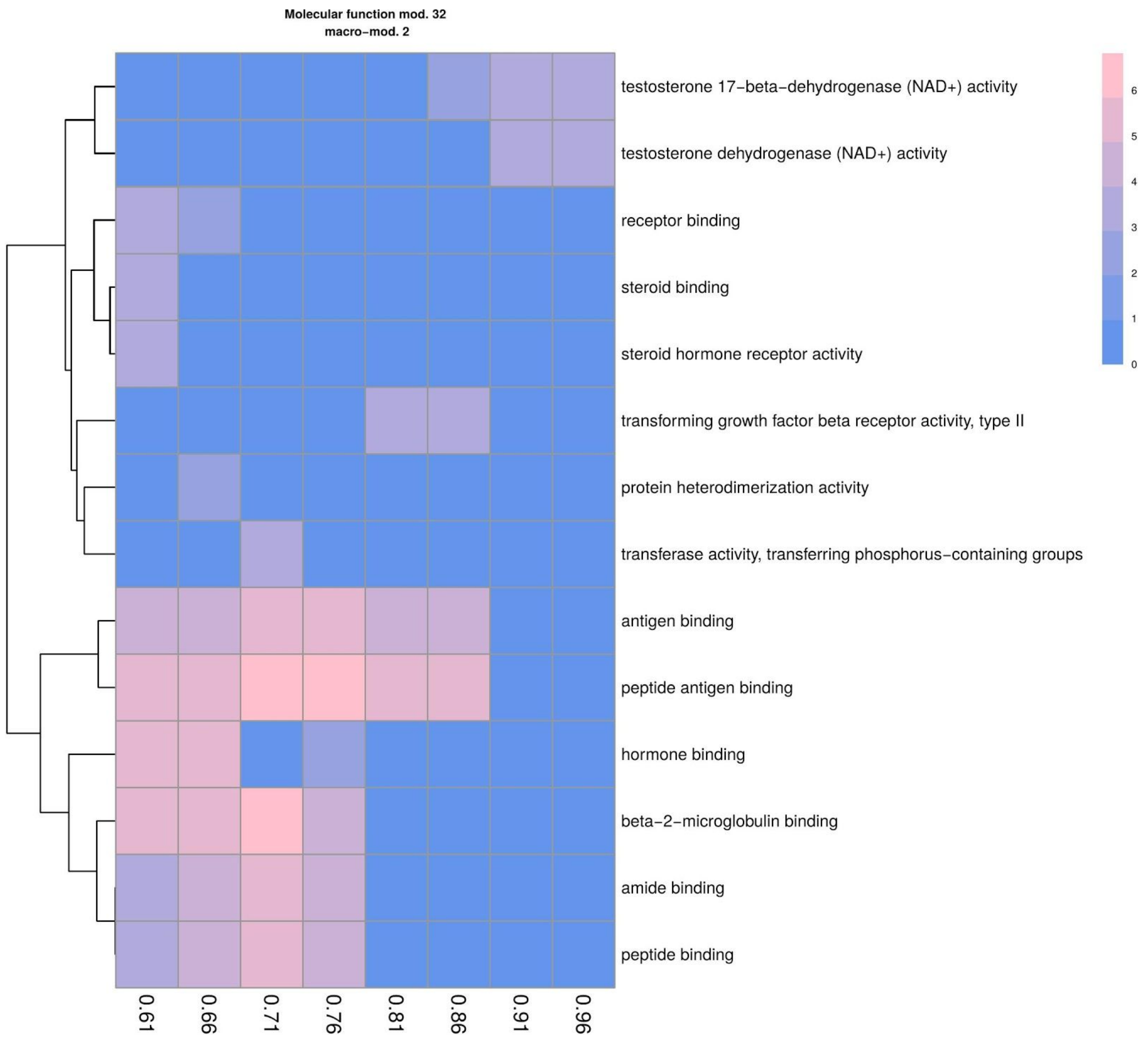

Supplementary Figure 47: MF “hormone” macro-module 2 (representative module 32).

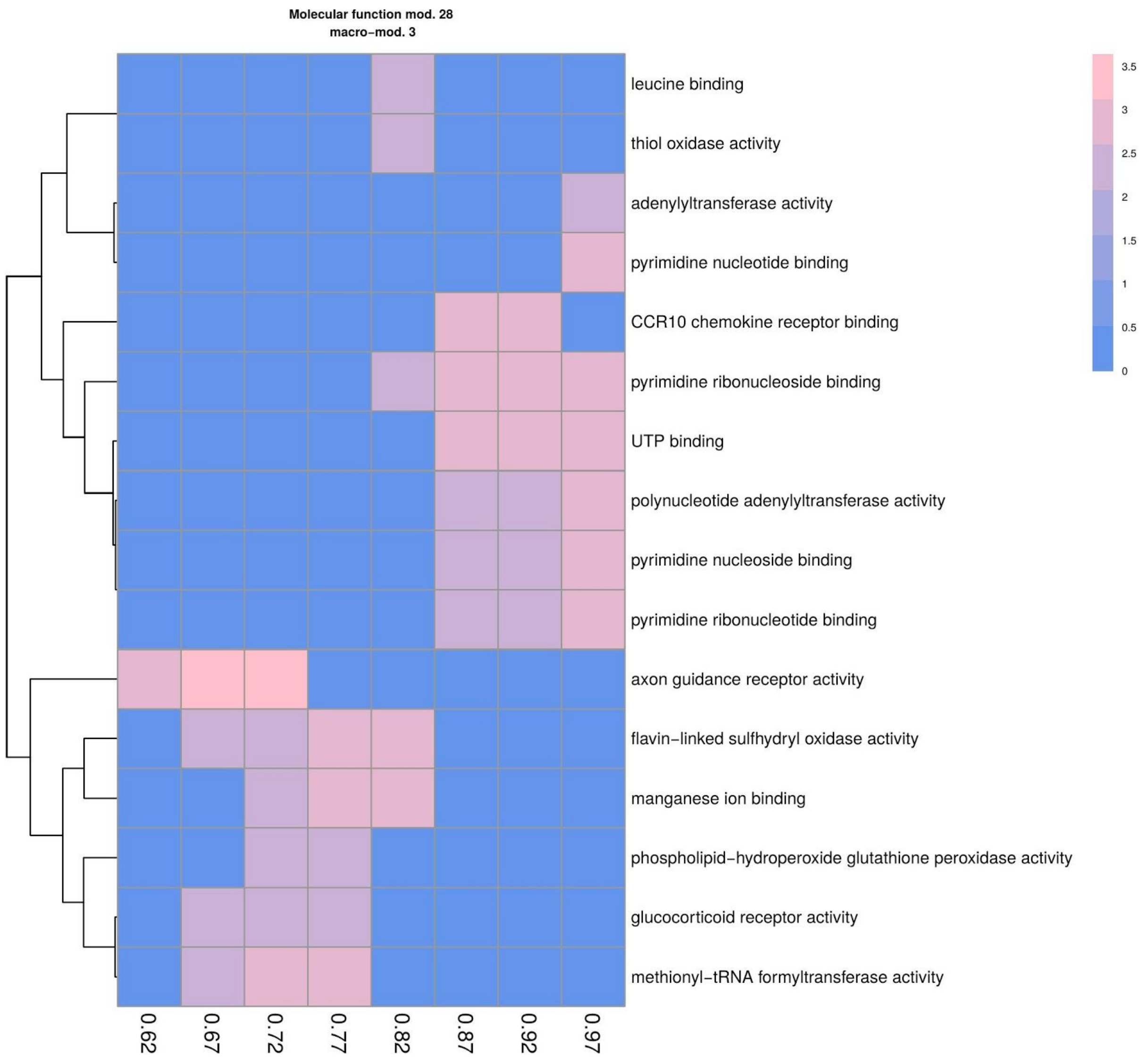

Supplementary Figure 48: MF “pyrimidine” macro-module 3 (representative module 28).

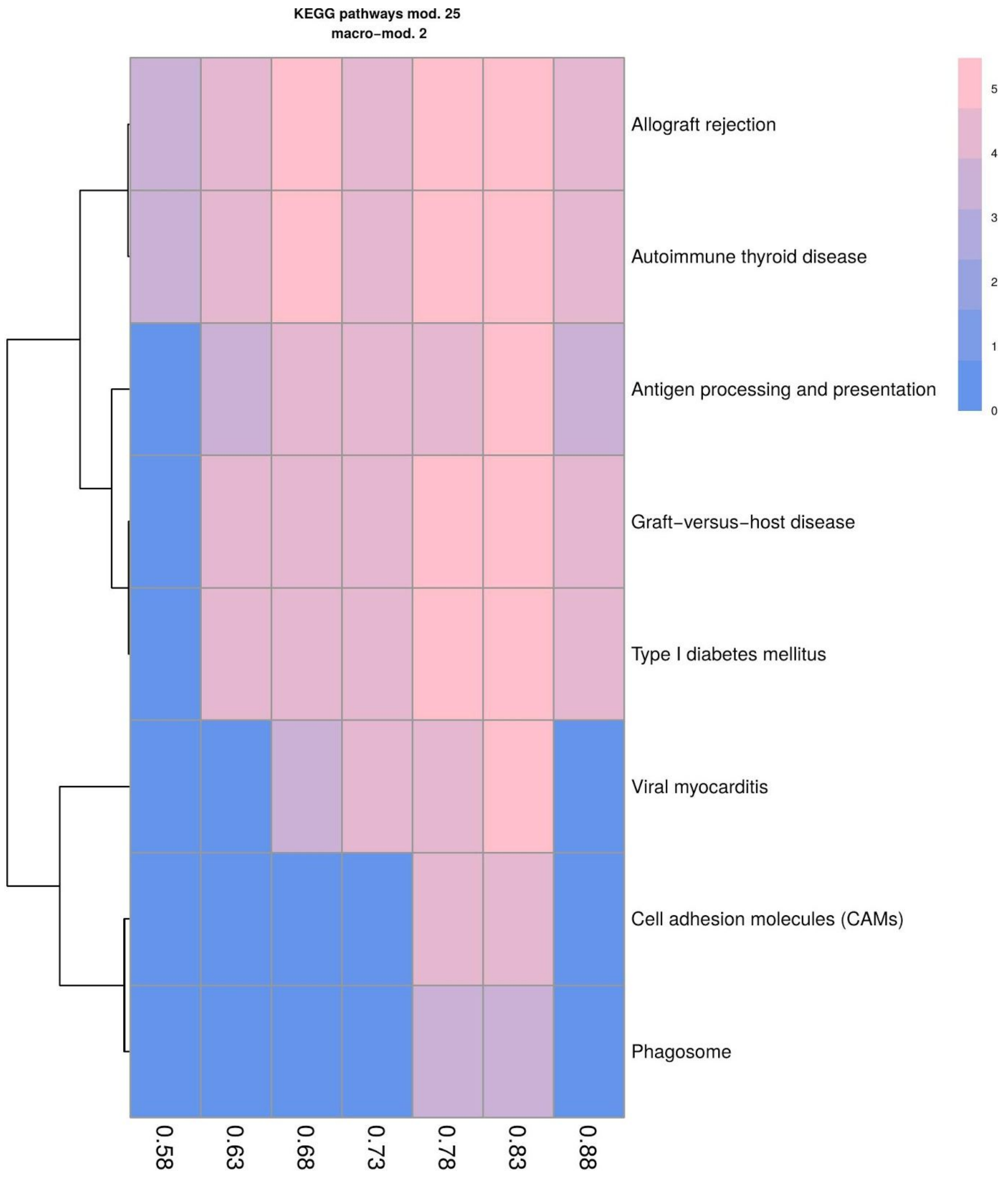

Supplementary Figure 49: KEGG “Graft” macro-module 2 (representative module 25).

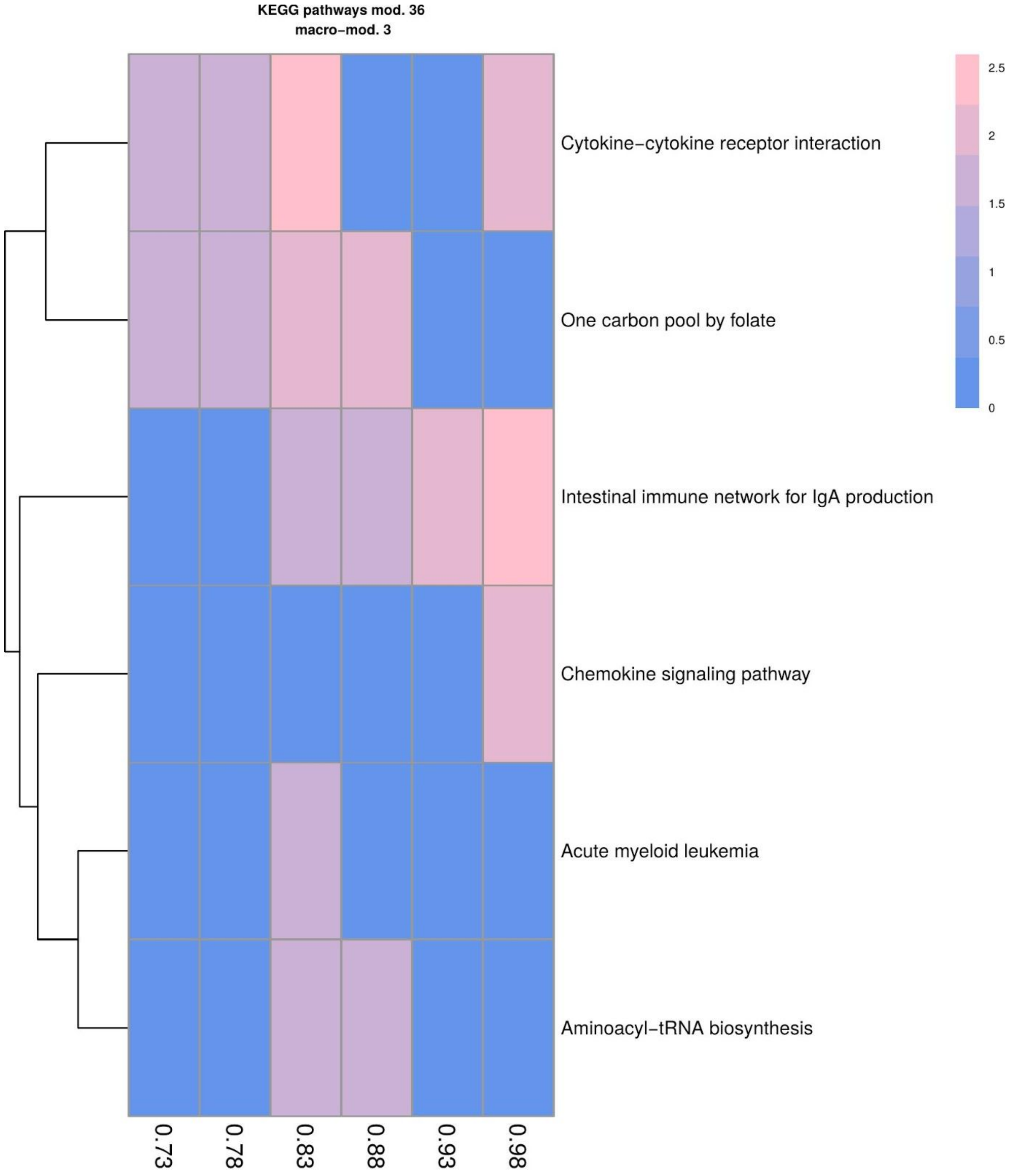

Supplementary Figure 50: KEGG “kinase” macro-module 3 (representative module 36).

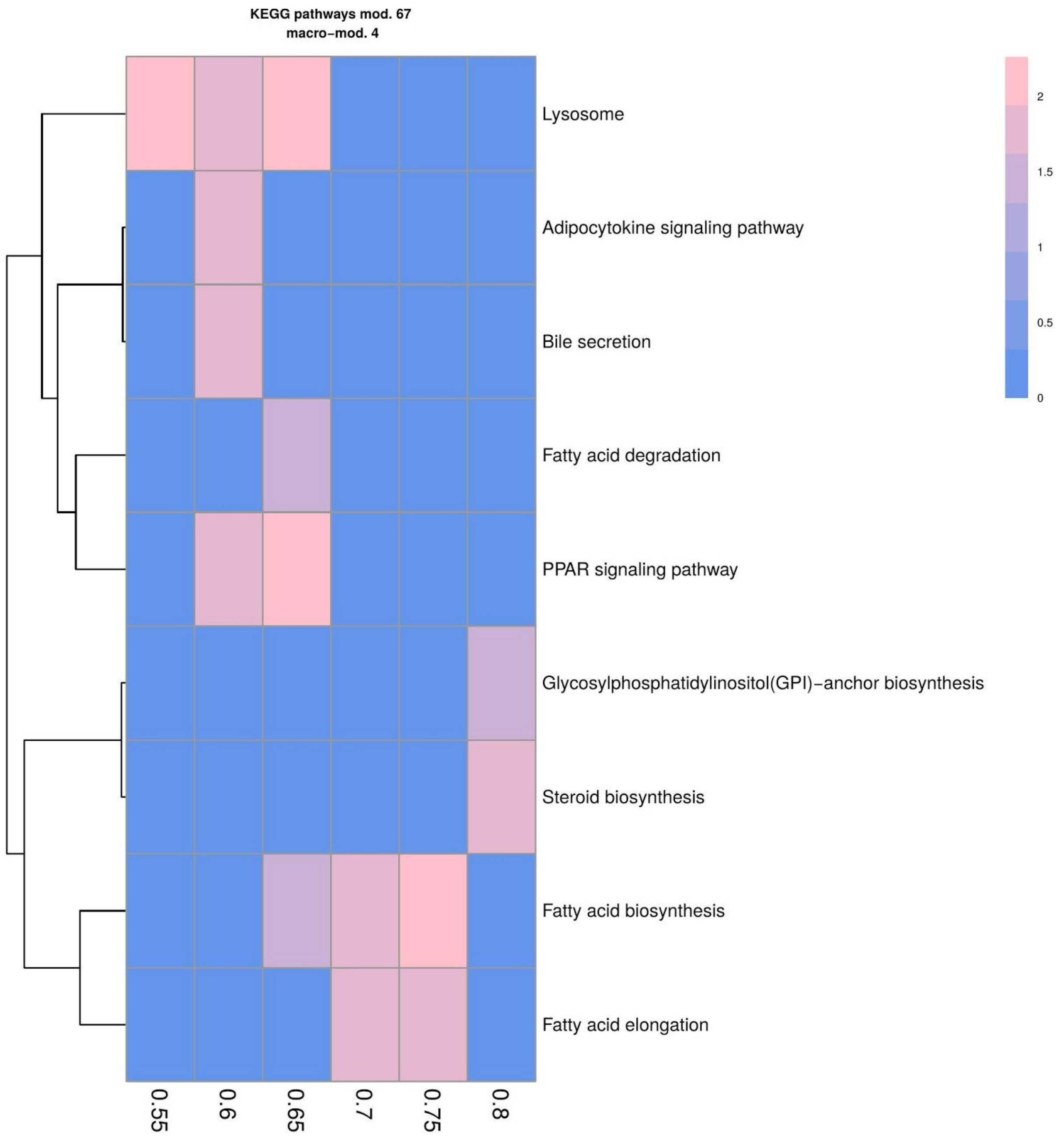

Supplementary Figure 51: KEGG “fatty acids” macro-module 4 (representative module 67).
