# Supplementary material for: RNAseq analysis of heart tissue from mice treated with atenolol and isoproterenol reveals a reciprocal transcriptional response
Source: BMC Genomics. 2016 Sep 7;17(1):717. doi: 10.1186/s12864-016-3059-6 (PMC5015234; doi:10.1186/s12864-016-3059-6)
Supplement: Additional file 2: — Supplementary tables. (PDF 3020 kb) [file 12864_2016_3059_MOESM2_ESM.pdf]

Supplementary Tables

	**Functional annotation chart of CE genes (DAVID)**		
	Category	*Term*	*p-value*
CE_{ISO}^{ATE}	GOTERM_MF_FAT	GO:0005516 (calmodulin binding)	7.3×10^{-02}
CE^{ISO}_{ATE}	GOTERM_CC_FAT	GO:0044449 (contractile fiber part)	3.4×10^{-02}
	GOTERM_CC_FAT	GO:0043292 (contractile fiber)	3.7×10^{-02}
	KEGG_PATHWAY	mmu04020 (calcium signaling pathway)	6.5×10^{-02}
CE_{ISO}^{ATE} + CE^{ISO}_{ATE}	GOTERM_BP_FAT	GO:0006936 (muscle contraction)	5.6×10^{-02}
	GOTERM_BP_FAT	GO:0003012 (muscle system process)	6.2×10^{-02}
	GOTERM_CC_FAT	GO:0044449 (contractile fiber part)	8.0×10^{-02}
	GOTERM_CC_FAT	GO:0043292 (contractile fiber)	8.7×10^{-02}
	GOTERM_CC_FAT	GO:0043228 (non-membrane-bounded organelle)	9.9×10^{-02}
	GOTERM_CC_FAT	GO:0043232 (intracellular non-membrane-bounded organelle)	9.9×10^{-02}

Supplementary Table 1: GO/KEGG analysis of the CE genes (*p*-value of GO categories <0.1 obtained with DAVID) shows their relevance to ‘muscular/contractile fibers’. The gene background is constituted by the 16397 genes surviving low-counts thresholding. The groups of counter-expressed (CE) genes are defined with a *FDR* threshold of 0.0001.

comp.	strain	surv.	FDR<0.05 all	FDR<0.05 +	FDR<0.05 -	FDR<0.01 all	FDR<0.01 +	FDR<0.01 -	FDR<0.001 all	FDR<0.001 +	FDR<0.001 -	FDR<0.0001 all	FDR<0.0001 +	FDR<0.0001 -
ISO-CTR	**AJ**	14306	225	144	81	225	144	81	225	144	81	152	104	48
	**BALBcByJ**	14571	109	77	32	109	77	32	109	77	32	62	50	12
	**BALBcJ**	14446	57	44	13	57	44	13	57	44	13	43	33	10
	**C3HHeJ**	14544	413	222	191	413	222	191	413	222	191	290	158	132
	**C57BL6J**	14386	243	128	115	243	128	115	243	128	115	162	88	74
	**C57BLKSJ**	14295	268	192	76	268	192	76	268	192	76	175	125	50
	**C58J**	14355	100	62	38	100	62	38	100	62	38	74	44	30
	**CBAJ**	14286	266	110	156	266	110	156	266	110	156	173	69	104
	**DBA2J**	14647	86	56	30	86	56	30	86	56	30	55	36	19
	**FVBNJ**	14512	216	120	96	216	120	96	216	120	96	139	75	64
	**ILnJ**	14488	179	119	60	179	119	60	179	119	60	106	77	29
	**LPJ**	14436	78	44	34	78	44	34	78	44	34	45	28	17
	**NODShiLtJ**	14591	844	474	370	844	474	370	844	474	370	610	347	263
	**NZBBLNJ**	14375	295	178	117	295	178	117	295	178	117	188	117	71
	**PLJ**	14459	291	175	116	291	175	116	291	175	116	194	117	77
	**SJLJ**	14472	300	175	125	300	175	125	300	175	125	180	107	73
	**SMJ**	14378	371	204	167	371	204	167	371	204	167	249	148	101
	**SWRJ**	14505	359	168	191	359	168	191	359	168	191	223	99	124
ATE-CTR	**AJ**	14268	25	17	8	25	17	8	25	17	8	18	12	6
	**BALBcByJ**	14508	0	0	0	0	0	0	0	0	0	0	0	0
	**BALBcJ**	14417	0	0	0	0	0	0	0	0	0	0	0	0
	**C3HHeJ**	14484	9	5	4	9	5	4	9	5	4	6	4	2
	**C57BL6J**	14327	35	18	17	35	18	17	35	18	17	17	9	8
	**C57BLKSJ**	14308	5	0	5	5	0	5	5	0	5	2	0	2
	**C58J**	14380	4	2	2	4	2	2	4	2	2	1	1	0
	**CBAJ**	14230	9	2	7	9	2	7	9	2	7	7	2	5
	**DBA2J**	14590	3	1	2	3	1	2	3	1	2	2	1	1
	**FVBNJ**	14453	4	3	1	4	3	1	4	3	1	2	2	0
	**ILnJ**	14453	0	0	0	0	0	0	0	0	0	0	0	0
	**LPJ**	14319	0	0	0	0	0	0	0	0	0	0	0	0
	**NODShiLtJ**	14492	21	6	15	21	6	15	21	6	15	11	2	9
	**NZBBLNJ**	14280	2	1	1	2	1	1	2	1	1	2	1	1
	**PLJ**	14450	13	4	9	13	4	9	13	4	9	8	2	6
	**SJLJ**	14455	6	1	5	6	1	5	6	1	5	4	0	4
	**SMJ**	14315	1	1	0	1	1	0	1	1	0	1	1	0
	**SWRJ**	14436	1	0	1	1	0	1	1	0	1	1	0	1

Supplementary Table 2: DE analysis across the 18 strains, related to the ISO-CTR and the ATE-CTR comparisons (*FDR*<0.05, 0.01, 0.001, and 0.0001). For each strain, around 14000 genes survived low-count filtering (“surv.”). The general lower transcriptional response to ATE is shown by the few resulting DE genes across all strains with respect to ISO. Strain NODShiLt/J is by far the most influenced by ISO, while strain C57BL6/J is the most responsive to ATE (see Suppl. Figures 18 and 19 for a graphical overview of DE genes at *FDR*<0.01).

GO: Biological Process	av. P-value
immune response	1.5×10^{-12}
defense response	1.8×10^{-05}
cell communication	1.3×10^{-04}
cellular component movement	1.6×10^{-04}
monocarboxylic acid metabolic process	2.3×10^{-03}
renal absorption	6.4×10^{-03}
antigen proc. And pres. Of peptide antigen	1.5×10^{-02}
extracellular matrix organization	2.1×10^{-02}
regulation of localization	3.0×10^{-02}
regulation of signaling	3.0×10^{-02}
extracellular matrix disassembly	3.2×10^{-02}
positive regulation of leukocyte differentiation	3.3×10^{-02}
lysosomal lumen acidification	4.3×10^{-02}

GO: Cellular Component	av. P-value
proteinaceous extracellular matrix	9.0×10^{-04}
cytosolic part	2.5×10^{-03}
MHC protein complex	7.0×10^{-03}
cytosolic ribosome	1.2×10^{-02}
organelle part	1.3×10^{-02}
extracellular region	1.4×10^{-02}
ribosomal subunit	1.8×10^{-02}
external side of plasma membrane	2.2×10^{-02}
organelle membrane	2.6×10^{-02}
intracellular organelle part	3.3×10^{-02}
extracellular region part	4.3×10^{-02}
cytoplasmic part	4.9×10^{-02}

GO: Molecular Function	av. P-value
tetrapyrrole binding	3.8×10^{-05}
beta-2-microglobulin binding	1.1×10^{-04}
water channel activity	5.0×10^{-03}
protease binding	8.8×10^{-03}
chemokine activity	9.3×10^{-03}
oxidoreductase activity	1.4×10^{-02}
structural constituent of ribosome	1.8×10^{-02}
immunoglobulin binding	2.0×10^{-02}
peptide antigen binding	2.0×10^{-02}
sulfur compound binding	2.4×10^{-02}
peptide binding	4.0×10^{-02}
GABA-A receptor activity	4.3×10^{-02}
enzyme binding	4.6×10^{-02}

KEGG categories	av. P-value
PPAR signaling pathway	5.5×10^{-04}
Allograft rejection	1.0×10^{-03}
Leishmaniasis	1.6×10^{-03}
Chemokine signaling pathway	2.1×10^{-03}
Antigen processing and presentation	3.7×10^{-03}
Leukocyte transendothelial migration	4.6×10^{-03}
Protein digestion and absorption	7.7×10^{-03}
Terpenoid backbone biosynthesis	1.3×10^{-02}
Osteoclast differentiation	1.5×10^{-02}
Hematopoietic cell lineage	1.9×10^{-02}
Ribosome	2.9×10^{-02}
Protein export	4.3×10^{-02}
Bile secretion	4.5×10^{-02}

Supplementary Table 3: Most significant GO and KEGG terms related to the transcriptional modules.

condition	phenotypes	
in conscious mice	**HR TC**	(pulse) heart rate (tail-cuff; beats/min)
	**BP**	(systolic) blood pressure (tail-cuff; mmHg)
	**BWS**	body weight at start (g)
	**BWE**	body weight at end (g)
	**BWG**	body weight gain (g)
under anaesthesia	**HR ECG**	heart rate (beats/min)
	**Pamp**	amplitude of p wave (mV)
	**Parea**	area of p wave (mV*ms)
	**Pdur**	duration of p wave (ms)
	**PR**	PR interval (ms)
	**RR**	RR interval (ms)
	**Qamp**	amplitude of Q wave (mV)
	**QRS**	QRS interval (ms)
	**QRSarea**	area of QRS complex (mV*ms)
	**QT**	QT interval (ms)
	**QT_carea**	QT_carea (mV*ms)
	**Ramp**	amplitude of R wave (mV)
	**Samp**	amplitude of S wave (mV)
	**ST**	ST interval (ms)
after euthanasia	**HW**	heart weight at end (mg)
	**HWI**	HW/BWE (mg/g)
	**AWI**	weight of atria over BWE (mg/g)
	**VW/AW**	weight of ventricles over weight of atria

Supplementary Table 4: Phenotypic traits selected in the present study.

anticorrelated phenotypes-modules			genes	first three leading genes
phenotypes	*modules*	*correlation*	*in module*	*(highest module score)*
**HR ECG**	module 38	−0.66	73	Map3k9, Tmed9, Cenpk
**QRS**	module 36	−0.66	56	Ccl25, Spock2, Fam221b
**PR**	module 94	−0.63	4697	Mfap5, Timm44, Dffb
**HR TC**	module 98	−0.61	8010	Dzip1l, Sipa1l2, Cygb
**ST**	module 20	−0.60	13	Nceh1, Nlrp5, Mrs2
**RR**	module 56	−0.59	211	Timm44, Dscr3, Mfap5
**Samp**	module 73	−0.58	184	AW551984, Nipal3, Dhdds
**QT**	module 64	−0.56	173	Klhl7, Chl1, Gabra1
**Pdur**	module 56	−0.53	211	Timm44, Dscr3, Mfap5
**AWI**	module 41	−0.50	55	Ppt1, Lmnb2, Aqp5
**BP**	module 41	−0.48	55	Ppt1, Lmnb2, Aqp5
**BWG**	module 12	−0.47	39	Bcas1, Cep128, Dscr3
**BWS**	module 26	−0.47	98	Hnrnpul2, Armcx4, Myh7b
**VW/AW**	module 76	−0.46	206	Timm44, Mfap5, Mrps7
**BWE**	module 26	−0.44	98	Hnrnpul2, Armcx4, Myh7b
**HWI**	module 41	−0.44	55	Ppt1, Lmnb2, Aqp5
**QTcarea**	module 28	−0.44	89	Mtpap, Klhl3, LOC100862586
**Qamp**	module 9	−0.42	49	Bcas1, Cep128, Phka1
**Ramp**	module 88	−0.42	1101	Gm14308, Gm8909, Gabra1
**Pamp**	module 60	−0.39	172	Ccl25, Mtpap, Nr3c1
**QRSarea**	module 10	−0.39	154	Zc3h12b, Star, Crlf3
**Parea**	module 46	−0.35	90	Rbm15, Erc2, Tmprss6

Supplementary Table 5: Summary of the most anti-correlated modules by phenotypes pairs. For each module, the number of genes as well as the names of the three top-scoring genes are shown.

anticorrelated phenotypes-modules: GO categories			
phenotypes	*modules*	**BP**	**CC**
**HR ECG**	module 38		
**QRS**	module 36		
**PR**	module 94	*cellular component movement (1.6x10^{-04})*	*extracellular region (3.7x10^{-03})*
**HR TC**	module 98		
**ST**	module 20		
**RR**	module 56	*extracellular matrix disassembly (3.7x10^{-02})*	*extracellular region (3.0x10^{-03})*
**Samp**	module 73		
**QT**	module 64		
**Pdur**	module 56	*extracellular matrix disassembly (3.7x10^{-02})*	*extracellular region (3.0x10^{-03})*
**AWI**	module 41		*organelle membrane (2.6x10^{-02})*
**BP**	module 41		*organelle membrane (2.6x10^{-02})*
**BWG**	module 12		
**BWS**	module 26		
**VW/AW**	module 76	*extracellular matrix disassembly (3.4x10^{-02})*	*proteinaceous extracellular matrix (3.7x10^{-06})*
**BWE**	module 26		
**HWI**	module 41		*organelle membrane (2.6x10^{-02})*
**QTcarea**	module 28		
**Qamp**	module 9		
**Ramp**	module 88		
**Pamp**	module 60		
**QRSarea**	module 10	*immune response (1.5x10^{-12})*	*MHC protein complex (5.4x10^{-04})*
**Parea**	module 46		

anticorrelated phenotypes-modules: GO categories			
phenotypes	*modules*	**MF**	**KEGG**
**HR ECG**	module 38		
**QRS**	module 36		
**PR**	module 94	*protease binding (1.7x10^{-02})*	*Leishmaniasis (1.6x10^{-03})*
**HR TC**	module 98		
**ST**	module 20		*Bile secretion (4.3x10^{-02})*
**RR**	module 56	*sulfur compound binding (1.7x10^{-02})*	
**Samp**	module 73		*Terpenoid backbone biosynthesis (7.0x10^{-03})*
**QT**	module 64		
**Pdur**	module 56	*sulfur compound binding (1.7x10^{-02})*	
**AWI**	module 41		*Bile secretion (4.8x10^{-02})*
**BP**	module 41		*Bile secretion (4.8x10^{-02})*
**BWG**	module 12		
**BWS**	module 26		
**VW/AW**	module 76	*sulfur compound binding (1.3x10^{-02})*	*Protein digestion and absorption (7.7x10^{-03})*
**BWE**	module 26		
**HWI**	module 41		*Bile secretion (4.8x10^{-02})*
**QTcarea**	module 28		
**Qamp**	module 9		
**Ramp**	module 88		
**Pamp**	module 60		
**QRSarea**	module 10	*beta-2-microglobulin binding (3.9x10^{-04})*	*Antigen processing and presentation (7.3x10^{-10})*
**Parea**	module 46		

Supplementary Table 6: Summary of the most significant GO terms related to the modules anti-correlated to phenotypes, as retrieved by GOSeq analysis. The GO term *p*-value threshold for listing the categories is 0.1. In this classical analysis, all genes within a module are considered, independently on their score. In general, only very broad GO categories (such as “immune response” and “extracellular region”) are selected.

correlated phenotypes-modules			genes	first three leading genes
phenotypes	*modules*	*correlation*	*in module*	*(highest module score)*
**HR TC**	module 56	78%	211	Timm44, Dscr3, Mfap5
**AWI**	module 40	70%	90	Timm44, Dscr3, Mrps7
**HWI**	module 40	67%	90	Timm44, Dscr3, Mrps7
**QT**	module 98	65%	8010	Dzip1l, Sipa1l2, Cygb
**RR**	module 41	62%	55	Ppt1, Lmnb2, Aqp5
**HR ECG**	module 56	59%	211	Timm44, Dscr3, Mfap5
**ST**	module 98	56%	8010	Dzip1l, Sipa1l2, Cygb
**PR**	module 19	54%	29	Dscr3, Timm44, Mrps7
**Qamp**	module 30	54%	76	AW551984, Fgd3, Tex35
**QRSarea**	module 92	53%	2423	Mfap5, Cd33, Timm44
**QTcarea**	module 72	53%	148	Klra1, LOC100862433, Klra8
**Pamp**	module 40	52%	90	Timm44, Dscr3, Mrps7
**Pdur**	module 38	52%	73	Map3k9, Tmed9, Cenpk
**BWS**	module 49	49%	42	Gm10193, LOC100048005, LOC101056294
**Samp**	module 78	49%	418	Scn3b, Klhl3, LOC100862586
**VW/AW**	module 62	48%	178	AW551984, Nipal3, Cfap70
**QRS**	module 19	47%	29	Dscr3, Timm44, Mrps7
**BWE**	module 49	45%	42	Gm10193, LOC100048005, LOC101056294
**BWG**	module 50	45%	78	Map3k9, Lmnb2, Ppt1
**Parea**	module 72	44%	148	Klra1, LOC100862433, Klra8
**BP**	module 81	42%	468	H2-M3, Rps29, Hist1h1d
**Ramp**	module 65	37%	188	Ccdc148, Ttc26, Ppt1

Supplementary Table 7: Summary of most the positively correlated modules by phenotypes pairs. For each module, the number of genes as well as the names of the three top-ranking genes are shown.

correlated phenotypes-modules: GO categories			
phenotypes	*modules*	**BP**	**CC**
**HR TC**	module 56	*extracellular matrix disassembly (3.7x10-02)*	*extracellular region (3.0x10-03)*
**AWI**	module 40		
**HWI**	module 40		
**QT**	module 98		
**RR**	module 41		*organelle membrane (2.6x10-02)*
**HR ECG**	module 56	*extracellular matrix disassembly (3.7x10-02)*	*extracellular region (3.0x10-03)*
**ST**	module 98		
**PR**	module 19		*extracellular region (4.8x10-02)*
**Qamp**	module 30		
**QRSarea**	module 92	*regulation of localization (3.0x10-02)*	*proteinaceous extracellular matrix (2.2x10-03)*
**QTcarea**	module 72		
**Pamp**	module 40		
**Pdur**	module 38		
**BWS**	module 49		
**Samp**	module 78		
**VW/AW**	module 62		
**QRS**	module 19		*extracellular region (4.8x10-02)*
**BWE**	module 49		
**BWG**	module 50	*renal absorption (6.4x10-03)*	
**Parea**	module 72		
**BP**	module 81	*defense response (3.8x10-07)*	
**Ramp**	module 65		

correlated phenotypes-modules: GO categories			
phenotypes	*modules*	**MF**	**KEGG**
**HR TC**	module 56	*sulfur compound binding (1.7x10-02)*	
**AWI**	module 40		
**HWI**	module 40		
**QT**	module 98		
**RR**	module 41		*Bile secretion (4.8x10-02)*
**HR ECG**	module 56	*sulfur compound binding (1.7x10-02)*	
**ST**	module 98		
**PR**	module 19		
**Qamp**	module 30		
**QRSarea**	module 92	*chemokine activity (1.5x10-02)*	
**QTcarea**	module 72		
**Pamp**	module 40		
**Pdur**	module 38		
**BWS**	module 49		
**Samp**	module 78		*Ribosome (4.7x10-02)*
**VW/AW**	module 62		*Terpenoid backbone biosynthesis (1.9x10-02)*
**QRS**	module 19		
**BWE**	module 49		
**BWG**	module 50	*water channel activity (5.0x10-03)*	
**Parea**	module 72		
**BP**	module 81		*Allograft rejection (3.9x10-03)*
**Ramp**	module 65		

Supplementary Table 8: Summary of the most significant GO terms related to the modules positively correlated to phenotypes. The GO term *p*-value threshold for listing the categories is 0.1. As in the previous analysis (Suppl. Table 6), all genes within a module are considered, independently on their score. Only very broad GO categories are selected.

category	*similarity*	*modules in the macro-module*	*macro-module*	*repr module*
	0.495	5 17 37 46 52		
	0.643	**4 25 32 51 75**	2 (antigene)	**51**
**BP**	0.556	**2 3 6 9 12 27**	1 (fibroblast)	**27**
	0.471	2 3 9 27		
	0.441	**28 53 54 59 60**	3 (cardiac)	**60**
	0.450	2 3 6 9 12 14 27		
	0.616	14 16 19 40 44		
	0.548	**36 43 53 59 60**	3 (organelle)	**59**
**CC**	0.481	**2 3 6 9 12 27**	1 (cytoskeleton)	**12**
	0.412	41 45 58 61 65		
	0.465	**4 25 32 51 75**	2 (MHC-complex)	**75**
	0.618	**2 3 6 9 12**	1 (ribonuclease)	**2**
	0.516	28 35 36 43 54		
	0.466	**28 35 36 53 54 59 60**	3 (pyrimidine)	**28**
	0.433	56 57 77 86 91		
	0.610	**4 25 32 51 75**	2 (hormone)	**32**
**MF**	0.591	62 73 78 80 83		
	0.420	28 35 36 43 54 59		
	0.502	40 44 56 76		
	0.445	29 38 50 61 67		
	0.416	29 38 39 50 61		
	0.456	55 77 86		
	0.615	**4 25 32 51 75**	2 (Graft)	**25**
	0.485	35 36 53 59 60		
	0.411	29 39 61 67		
	0.430	**28 36 53 54 59 60**	3 (chemokine)	**36**
	0.526	56 76 86 91 92 94		
**KEGG**	0.418	29 39 41 45 50 61		
	0.436	29 39 41 45 61 67		
	0.457	10 71 74 79 81 82 87		
	0.440	4 22 25 32 51 71 75 79 81 82		
	0.403	71 79 81 82 86 91 92 94		
	0.409	4 10 22 25 32 51 71 74 75 79 81 82 87		
	0.422	4 10 22 25 32 51 74 75 87		

Supplementary Table 9: Macro-modules obtained with the refined GO analysis of the transcriptional modules. The structure of at least 2 macro-modules is shared among all the GO categories (as shown in red and green fonts). See Suppl. Figures 36-39 for details.
