# Supplementary material for: RNAseq analysis of heart tissue from mice treated with atenolol and isoproterenol reveals a reciprocal transcriptional response
Source: BMC Genomics. 2016 Sep 7;17(1):717. doi: 10.1186/s12864-016-3059-6 (PMC5015234; doi:10.1186/s12864-016-3059-6)
Supplement: Additional file 3: — Supplementary information. (PDF 416 kb) [file 12864_2016_3059_MOESM3_ESM.pdf]

Supplementary Information

An overall-threshold method to detect specific GO terms

Common definition of differential expression of genes

A key point in the framework of gene expression analysis is to define the *differential expression* of a given gene *g* among two sets of biologically different conditions – usually a "control" condition C and a "treated" condition T. The starting point, thus, consists on two groups of values $c(g)_1$, $c(g)_2$... $c(g)_m$ and $t(g)_1$, $t(g)_2$... $t(g)_n$ indicating the multiple measures of the expression of *g* in the condition C (with *m* "biological replicates") and in the condition T (with *n* "biological replicates"), respectively. A statistical test among these two groups is then performed to assess the likelihood *p* of *g* being significantly *differentially expressed* (DE) in the two conditions. For practical reasons, the definition of DE genes is based on a fixed threshold: if the *p*-value is less than a chosen limit (often, $p<0.01$), then *g* is considered DE, otherwise not. A more refined version of this test consists in adjusting the *p*-values for multiple-testing, classically using the Benjamini-Hochberg correction [1]. Clearly, a different choice in the *p*-value thresholds (e.g. $p<0.01$ rather than $p<0.05$) may reflect in considering *g* a DE gene or not.

From DE genes to gene ontology analysis

Fixed an arbitrary threshold (either based on the *p*-values or on the adjusted *p*-values), these methods allow to define a *list* of genes which are likely to be DE. Once this DE list is obtained, it is possible to associate it to functional groups by *gene ontology* (GO) analysis. In practice, GO categories (or *terms*) consist on lists of annotated genes, already known to be involved in a certain function, either at the *biological process* level (GO BP categories, entailing ~8000 different lists of annotated genes for a given genome), at the *cellular component* level (GO CC, ~1000 categories), at the *molecular function* level (GO

MF, ~2800 categories) [2] or at the *pathways* level (from the Kyoto Encyclopedia of Genes and Genomes or KEGG terms, ~800) [3]. There are many tools to determine the probability that a selected list of DE genes is related to one or the other GO or KEGG categories, one of the most popular being DAVID [4]. However, most of these tools are based on a statistical test that takes into account the number of genes in the DE list, the number of genes in the annotated category and the number of background genes. Thus, the likelihood that the selected DE list is associated to a specific *G* category (let us call it the $GODE_G$ *p*-value) is strongly dependent on *which* genes are contained in the DE list itself (especially in cases where GO categories are defined by a small number of genes). As a consequence, the *p*-value (or adjusted *p*-value) thresholds used to define the DE list may result in very different GO and KEGG terms associated with the difference in the gene expression values among the C and the T conditions.

A method to circumvent the arbitrary definition of the DE list

Our aim is to avoid that the GO analysis is too dependent on the definition of the DE list. One way to do so is, first, to investigate the $GODE_G$ *p*-value of a given category *G* as a function of the *p*-value threshold, then to order the GO categories according to their *global* significance along the DE variable. Indeed, if a GO category is biologically involved in the C versus T comparison, the fact that one or few genes are included or excluded from the DE list should not *completely* affect the decision of considering that category relevant or not. At the same time, it could be biologically interesting to explore the range of DE significance at which a GO category is relevant. This idea can be implemented by sampling the $GODE_G$ *p*-values at a regular interval of DE genes threshold (e.g. an interval of 1 unit of $-\log_{10}p$, starting from a very loose definition of DE genes, e.g. $-\log_{10}p > 1$, up to a very stringent one, e.g. $-\log_{10}p > 10$). At each step, a larger set of genes will be accounted for the definition of the DE(p) list, resulting in a characteristic $GODE_G(p)$ function for each category. The global

significance *S* of a certain category *G* (S_G) can be defined as the area of the following function

$$S_G = \sum log_{10}(p) \times log_{10}[GODE_G(p)]$$

which can be used to collect together and to order the GO categories in a single heatmap (Figure A below). This multi-thresholding approach, supported by the further ordering of GO terms significance according to their *S*, allows the investigator to explore the *whole* functional landscape of the C by T gene expression comparison patterns.

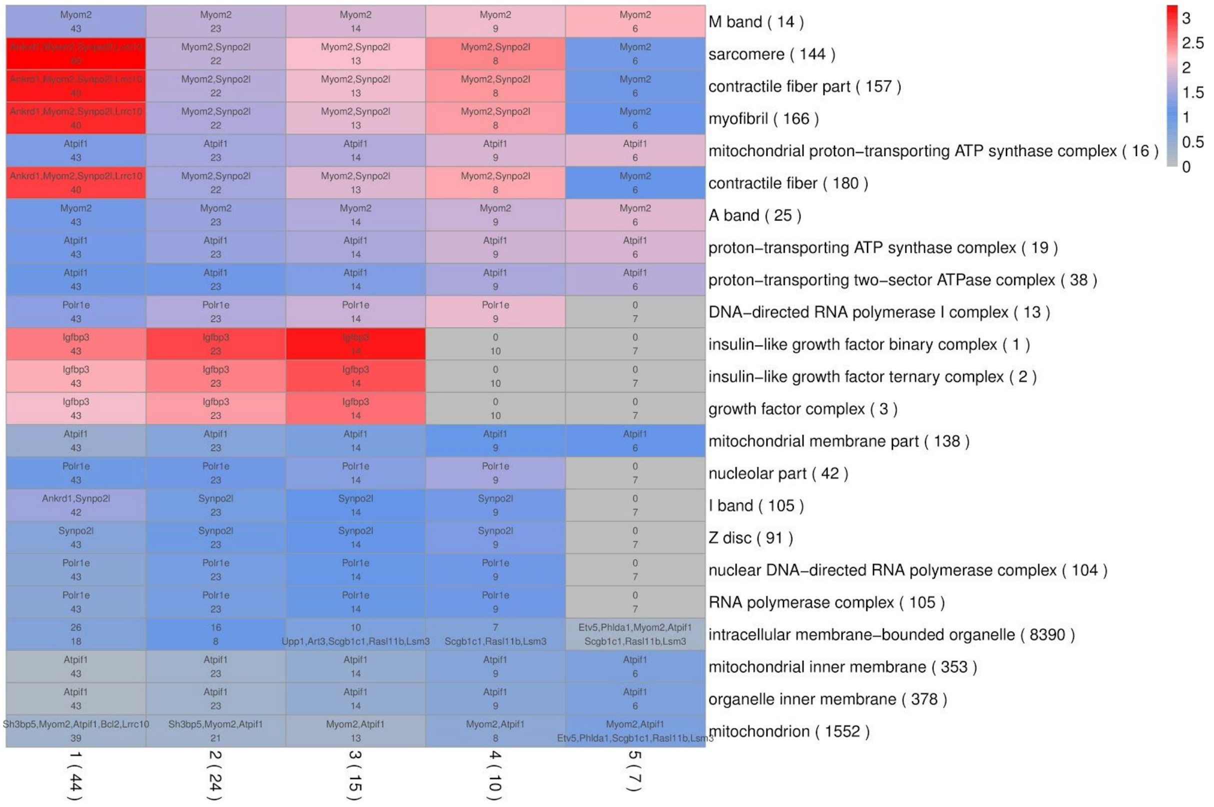

Figure A: An example of $GODE_G(p)$ analysis, here for the GO CC terms and the CE_{ISO}^{ATE} group. On the *x*-axis, the increasing values of the $-log_{10}p$ threshold (defining the DE list) is reported and the number of DE genes in each of the DE lists is shown in brackets. On the *y*-axis, KEGG terms *G*, whose global significance S_G (area) along the DE genes threshold range is >0, are reported; the number of annotated genes in the respective categories are

shown in brackets. The color code refers to the $-\log_{10}(GODE_G(p))$ significance at the respective thresholds. In this example, the DE genes threshold step is of 1 units of DE significance, while its maximum level is 5 ($p<10^{-5}$), since beyond this value there were neither significant categories, nor CE genes. Rows are ranked according to the order of the S_G (from top to bottom).

Guidelines to the navigation of the modules HTML page

The gene content and GO properties of the 98 modules of CE genes are presented as an html supplement. To extract the modules page (`modules.tar.gz`) with Windows, just double-click on the file. With MAC or Linux systems, open a terminal, go on the folder where the file has been downloaded and type:

```
> gunzip modules.tar.gz
```

and then

```
> tar -xvf modules.tar
```

This will create a directory called `modules.html`. Once this folder is extracted, move inside it and open the html file `index.html` with a web browser. With Firefox, go on File/Open File… and select the `index.html` file from the menu. From a terminal, just type:

```
> firefox index.html
```

This will open the html page with the list of the 98 transcription modules. Herein, the number of genes (**#G**), conditions or samples (**#C**) and the leading GO/KEGG terms are listed for each module. Upon clicking on the module number (leftmost column), the contents of the selected module can be explored further by accessing the corresponding gene expression matrix (genes in columns, samples in rows) and complete GOSeq analysis results. Note that on the right side of each section, the **Help/Show/Top** menu can be used to expand (**Show**) or compact (**Hide**) the section. The **Conditions** section contains the list of the samples belonging to the selected module (the number appearing in the sample names is a unique identifier used to distinguish the samples from one another, ranging from 1 to

160). At the bottom of the module page, a plot with the normalized module score for each condition is reported.

References

[1] Benjamini, Hochberg (1995), *Controlling the false discovery rate: a practical and powerful approach to multiple testing*. Journal of the Royal Statistical Society, Series B 57 (1):125-133.

[2] http://geneontology.org

[3] http://www.genome.jp/kegg/

[4] Huang, Sherman, Lempicki (2009), *Systematic and integrative analysis of large gene lists using DAVID Bioinformatics Resources*. Nat. Protoc. 4(1):44-57.
