# Supplementary material for: RNAseq analysis of heart tissue from mice treated with atenolol and isoproterenol reveals a reciprocal transcriptional response
Source: BMC Genomics. 2016 Sep 7;17(1):717. doi: 10.1186/s12864-016-3059-6 (PMC5015234; doi:10.1186/s12864-016-3059-6)
Supplement: Additional file 4: — Supplementary data. Compressed HTML files of 98 expression modules annotated for genes, strains and GO or KEGG terms (see Additional file 3 for navigation details). (GZ 11006 kb) [file 12864_2016_3059_MOESM4_ESM.gz › modules.html/module-91.html]

Module #91, TG: 1, TC: 0.25, 2278 probes, 2278 Entrez genes, 144 conditions

# Previous module | Next module Module #91, TG: 1, TC: 0.25, 2278 probes, 2278 Entrez genes, 144 conditions

- Module tree/table

- Expression data

- The BP GO tree
- The CC GO tree
- The MF GO tree

- GO BP enrichment
- GO CC enrichment
- GO MF enrichment
- KEGG enrichment
- miRNA enrichment

- Genes
- Conditions

## Help | Hide | Top Help | Show | Top Expression data

### HELP

The image plot shows the color-coded level of gene expression, for the
genes and conditions in a given transcription module. The genes are on
the horizontal, the conditions on the vertical axis.

The genes are ordered according to their ISA gene scores, similarly
the conditions are ordered according to their condition scores. The
score of a gene means the «degree of inclusion» in
the module: a high score gene is essential in the module.

Condition scores can also be negative, that means that the genes of
the module are all down-regulated in the condition. Here the absolute
value of the score gives the «degree of inclusion».

The plots above and beside the expression matrix show the gene scores
and condition scores, respectively.

Note that the plot is interactive, you can see the name of the gene
and condition under the mouse cursor.

The expression matrix was normalized to have mean zero and standard
deviation one for every gene separately across all conditions
(i.e. not just for the conditions in the module).

— Click on the *Help* button again to close this help window.

Gene:   
Condition:

Under-expression is coded with green,
over-expression with red color.

## Help | Hide | Top Help | Show | Top The GO tree — Biological processes

### HELP

This is one of three sections showing Gene Ontology enrichment of the
current module: in this case for **biological processes**.

The graph shows the hierarchy of the GO categories, their enrichment
for the current module is color coded, and the blue number beside the
category is the minus log ten p-value of the enrichment. (Calculated
using the standard hypergeometric test.) The color of the arrows code
«is a» (cyan) and «part of» relationships.

The tree was built the following way. First all GO terms with more
significant enrichment p-value than 0.05 were collected. Then all
paths from these terms to the root node of the GO tree were included
too. If a GO term is included more than once in the tree, then the
green numbers show 1) the id of the node, this makes it easier to find
other appereances of the term, and 2) the number of appearences.

Note that the same GO category might show up on the graph many
times. This is because the GO was «straightened» for this
graph, i.e. if there are more paths from a GO term to the root node of
the tree, all of them are included. The green numbers

Move the mouse cursor over the terms to get their definition. Clicking
on them takes you to the corresponding Gene Ontology web page.

If you cannot see a graph here at all, that means that there were no
significantly enriched GO categories, at the 0.05 level.

— Click on the *Help* button again to close this help window.

:   **signal transduction**

    The cellular process in which a signal is conveyed to trigger a change in the activity or state of a cell. Signal transduction begins with reception of a signal (e.g. a ligand binding to a receptor or receptor activation by a stimulus such as light), or for signal transduction in the absence of ligand, signal-withdrawal or the activity of a constitutively active receptor. Signal transduction ends with regulation of a downstream cellular process, e.g. regulation of transcription or regulation of a metabolic process. Signal transduction covers signaling from receptors located on the surface of the cell and signaling via molecules located within the cell. For signaling between cells, signal transduction is restricted to events at and within the receiving cell.
:   **biological\_process**

    Any process specifically pertinent to the functioning of integrated living units: cells, tissues, organs, and organisms. A process is a collection of molecular events with a defined beginning and end.
:   **cellular process**

    Any process that is carried out at the cellular level, but not necessarily restricted to a single cell. For example, cell communication occurs among more than one cell, but occurs at the cellular level.
:   **signaling**

    The entirety of a process in which information is transmitted within a biological system. This process begins with an active signal and ends when a cellular response has been triggered.
:   **single-organism process**

    A biological process that involves only one organism.
:   **single organism signaling**

    A signaling process occurring within a single organism.
:   **single-organism cellular process**

    Any process that is carried out at the cellular level, occurring within a single organism.
:   **regulation of biological process**

    Any process that modulates the frequency, rate or extent of a biological process. Biological processes are regulated by many means; examples include the control of gene expression, protein modification or interaction with a protein or substrate molecule.
:   **regulation of cellular process**

    Any process that modulates the frequency, rate or extent of a cellular process, any of those that are carried out at the cellular level, but are not necessarily restricted to a single cell. For example, cell communication occurs among more than one cell, but occurs at the cellular level.
:   **response to stimulus**

    Any process that results in a change in state or activity of a cell or an organism (in terms of movement, secretion, enzyme production, gene expression, etc.) as a result of a stimulus. The process begins with detection of the stimulus and ends with a change in state or activity or the cell or organism.
:   **cellular response to stimulus**

    Any process that results in a change in state or activity of a cell (in terms of movement, secretion, enzyme production, gene expression, etc.) as a result of a stimulus. The process begins with detection of the stimulus by a cell and ends with a change in state or activity or the cell.
:   **biological regulation**

    Any process that modulates a measurable attribute of any biological process, quality or function.
:   **all**

    NA
:   **NA**

    NA
:   **single-organism cellular process**

    Any process that is carried out at the cellular level, occurring within a single organism.
:   **regulation of biological process**

    Any process that modulates the frequency, rate or extent of a biological process. Biological processes are regulated by many means; examples include the control of gene expression, protein modification or interaction with a protein or substrate molecule.
:   **response to stimulus**

    Any process that results in a change in state or activity of a cell or an organism (in terms of movement, secretion, enzyme production, gene expression, etc.) as a result of a stimulus. The process begins with detection of the stimulus and ends with a change in state or activity or the cell or organism.
:   **regulation of cellular process**

    Any process that modulates the frequency, rate or extent of a cellular process, any of those that are carried out at the cellular level, but are not necessarily restricted to a single cell. For example, cell communication occurs among more than one cell, but occurs at the cellular level.
:   **biological regulation**

    Any process that modulates a measurable attribute of any biological process, quality or function.
:   **biological\_process**

    Any process specifically pertinent to the functioning of integrated living units: cells, tissues, organs, and organisms. A process is a collection of molecular events with a defined beginning and end.
:   **biological\_process**

    Any process specifically pertinent to the functioning of integrated living units: cells, tissues, organs, and organisms. A process is a collection of molecular events with a defined beginning and end.
:   **biological\_process**

    Any process specifically pertinent to the functioning of integrated living units: cells, tissues, organs, and organisms. A process is a collection of molecular events with a defined beginning and end.

## Help | Hide | Top Help | Show | Top The GO tree — Cellular Components

### HELP

This is one of three sections showing Gene Ontology enrichment of the
current module: in this case for **cellular components**.

The graph shows the hierarchy of the GO categories, their enrichment
for the current module is color coded, and the blue number beside the
category is the minus log ten p-value of the enrichment. (Calculated
using the standard hypergeometric test.) The color of the arrows code
«is a» (cyan) and «part of» relationships.

The tree was built the following way. First all GO terms with more
significant enrichment p-value than 0.05 were collected. Then all
paths from these terms to the root node of the GO tree were included
too. If a GO term is included more than once in the tree, then the
green numbers show 1) the id of the node, this makes it easier to find
other appereances of the term, and 2) the number of appearences.

Note that the same GO category might show up on the graph many
times. This is because the GO was «straightened» for this
graph, i.e. if there are more paths from a GO term to the root node of
the tree, all of them are included. The green numbers

Move the mouse cursor over the terms to get their definition. Clicking
on them takes you to the corresponding Gene Ontology web page.

If you cannot see a graph here at all, that means that there were no
significantly enriched GO categories, at the 0.05 level.

— Click on the *Help* button again to close this help window.

:   **extracellular region**

    The space external to the outermost structure of a cell. For cells without external protective or external encapsulating structures this refers to space outside of the plasma membrane. This term covers the host cell environment outside an intracellular parasite.
:   **proteinaceous extracellular matrix**

    A layer consisting mainly of proteins (especially collagen) and glycosaminoglycans (mostly as proteoglycans) that forms a sheet underlying or overlying cells such as endothelial and epithelial cells. The proteins are secreted by cells in the vicinity. An example of this component is found in Mus musculus.
:   **extracellular space**

    That part of a multicellular organism outside the cells proper, usually taken to be outside the plasma membranes, and occupied by fluid.
:   **cell**

    The basic structural and functional unit of all organisms. Includes the plasma membrane and any external encapsulating structures such as the cell wall and cell envelope.
:   **neuron projection**

    A prolongation or process extending from a nerve cell, e.g. an axon or dendrite.
:   **extracellular matrix**

    A structure lying external to one or more cells, which provides structural support for cells or tissues; may be completely external to the cell (as in animals and bacteria) or be part of the cell (as in plants).
:   **cell projection**

    A prolongation or process extending from a cell, e.g. a flagellum or axon.
:   **extracellular region part**

    Any constituent part of the extracellular region, the space external to the outermost structure of a cell. For cells without external protective or external encapsulating structures this refers to space outside of the plasma membrane. This term covers constituent parts of the host cell environment outside an intracellular parasite.
:   **cell part**

    Any constituent part of a cell, the basic structural and functional unit of all organisms.
:   **neuron part**

    Any constituent part of a neuron, the basic cellular unit of nervous tissue. A typical neuron consists of a cell body (often called the soma), an axon, and dendrites. Their purpose is to receive, conduct, and transmit impulses in the nervous system.
:   **all**

    NA
:   **NA**

    NA
:   **cell part**

    Any constituent part of a cell, the basic structural and functional unit of all organisms.
:   **neuron part**

    Any constituent part of a neuron, the basic cellular unit of nervous tissue. A typical neuron consists of a cell body (often called the soma), an axon, and dendrites. Their purpose is to receive, conduct, and transmit impulses in the nervous system.
:   **extracellular space**

    That part of a multicellular organism outside the cells proper, usually taken to be outside the plasma membranes, and occupied by fluid.
:   **extracellular matrix**

    A structure lying external to one or more cells, which provides structural support for cells or tissues; may be completely external to the cell (as in animals and bacteria) or be part of the cell (as in plants).

## Help | Hide | Top Help | Show | Top The GO tree — Molecular Function

### HELP

This is one of three sections showing Gene Ontology enrichment of the
current module: in this case for **molecular function**.

The graph shows the hierarchy of the GO categories, their enrichment
for the current module is color coded, and the blue number beside the
category is the minus log ten p-value of the enrichment. (Calculated
using the standard hypergeometric test.) The color of the arrows code
«is a» (cyan) and «part of» relationships.

The tree was built the following way. First all GO terms with more
significant enrichment p-value than 0.05 were collected. Then all
paths from these terms to the root node of the GO tree were included
too. If a GO term is included more than once in the tree, then the
green numbers show 1) the id of the node, this makes it easier to find
other appereances of the term, and 2) the number of appearences.

Note that the same GO category might show up on the graph many
times. This is because the GO was «straightened» for this
graph, i.e. if there are more paths from a GO term to the root node of
the tree, all of them are included. The green numbers

Move the mouse cursor over the terms to get their definition. Clicking
on them takes you to the corresponding Gene Ontology web page.

If you cannot see a graph here at all, that means that there were no
significantly enriched GO categories, at the 0.05 level.

— Click on the *Help* button again to close this help window.

:   **protease binding**

    Interacting selectively and non-covalently with any protease or peptidase.
:   **molecular\_function**

    Elemental activities, such as catalysis or binding, describing the actions of a gene product at the molecular level. A given gene product may exhibit one or more molecular functions.
:   **binding**

    The selective, non-covalent, often stoichiometric, interaction of a molecule with one or more specific sites on another molecule.
:   **enzyme binding**

    Interacting selectively and non-covalently with any enzyme.
:   **all**

    NA
:   **NA**

    NA

## Help | Hide | Top Help | Show | Top GO BP test for over-representation

### HELP

List of all enriched GO categories (biological processes), at the 0.05
p-value level.

The columns:

- **ExpCount** is the expected count of genes in the
  module annotated with the given GO term, just by chance.
- **Count**
  is the number of genes in the module annotated with the given GO
  term.
- **Size** is the total number of genes (in our universe)
  annotated with the GO term.

Clicking on **Count** shows the genes that drive the
enrichment. You can also click on the individual numbers in
the **Count** column, to show the driving genes for that individual
GO category.

Clicking on the GO identifiers takes you to the Gene Ontology web
pages.

— Click on the *Help* button again to close this help window.

| Id | Pvalue | ExpCount | Count | Size | Term |
| --- | --- | --- | --- | --- | --- |
| GO:0007154 | 1.327e-04 | 523.2 | 627 9430023L20Rik, 9630033F20Rik, Aatk, Adam11, Adam19, Adam22, Adam5, Adamts3, Adcy4, Adcy6, Adcy8, Adora2a, Adra1d, Agrp, Aimp1, Akap2, Akt1s1, Aloxe3, Amer1, Ankrd26, Ap3d1, Apc2, Aph1a, Aplnr, Apoc3, Aqp1, Aqp2, Arap3, Arhgap11a, Arhgap15, Arhgap18, Arhgap20, Arhgap21, Arhgap24, Arhgap31, Arhgap42, Arhgap6, Arhgef1, Arhgef10, Arhgef37, Arid1a, Arid5b, Arl11, Arl13a, Arl5c, Arl6, Arntl, Asb10, Asb13, Asb5, Asic1, Asxl1, Atg9a, Avpr1a, Avpr2, Axin1, Baiap2, Bcap31, Bcl2, Bcl2l2, Bcl9l, Bex1, Bloc1s2, Bmf, Bmp10, Bmp4, Bmpr1b, Bnip2, Brca1, C1qtnf1, C1qtnf2, C5ar2, Cacna1b, Cacna1i, Cacnb2, Canx, Caprin2, Capzb, Car8, Card6, Casp1, Casp2, Casp7, Cav3, Cck, Ccl11, Ccl17, Ccl19, Ccl2, Ccl5, Ccnd3, Ccny, Ccr1l1, Cd22, Cd28, Cd36, Cd38, Cd3d, Cd4, Cd5, Cd79a, Cdc42bpa, Cdc42bpb, Cdc42bpg, Cdh1, Cdh2, Cdh8, Cdk14, Cdk5r1, Cds2, Cflar, Chi3l1, Chn2, Cish, Cited1, Clca2, Clec2d, Cln3, Cmya5, Cntnap1, Col18a1, Col1a1, Cplx1, Cr2, Creb1, Crk, Csf1r, Csf2rb, Cspg5, Ctf1, Ctnnd2, Ctsc, Ctsh, Ctsl, Cx3cl1, Cxadr, Cxcl13, Cxcl5, Cxcr4, Cxxc4, Cysltr1, Dclk3, Ddr1, Depdc1a, Depdc7, Derl2, Dgke, Dgki, Dhx58, Dixdc1, Dkk2, Dkk3, Dlgap1, Dlgap3, Doc2b, Dock10, Dock5, Dock6, Dok4, Dsp, Dtx4, Duox2, Dusp3, Dut, Dvl3, Edn3, Efnb2, Efnb3, Egr1, Egr2, Ei24, Eif2a, Eif2b4, Emp2, Entpd1, Epas1, Ern1, Exoc3l, F2, F7, Fa2h, Fam110c, Fam13b, Fbxo8, Fbxo9, Fcgr1, Fcgr2b, Fgd5, Fgd6, Fgf10, Fgf16, Fgf20, Fgf21, Fgf6, Fgf7, Fgfbp1, Fgfr2, Fgfr4, Fhit, Fkbp8, Fosl1, Foxo6, Fpr2, Frem1, Frem2, Frzb, Fst, Fxn, Fyn, Fzd3, G2e3, Gab1, Gabbr2, Gabra1, Gabrd, Gabrr2, Garnl3, Gas6, Gata6, Gdf6, Gdi2, Gdnf, Gem, Ggct, Gjb5, Gjc2, Gli1, Gli2, Glis2, Gna12, Gnai1, Gnai2, Gnas, Gng7, Gpld1, Gpr12, Gpr150, Gpr152, Gpr156, Gpr157, Gpr171, Gpr183, Gpr4, Gpr55, Gpr61, Gpr65, Gpr84, Gps2, Gpx1, Grid2ip, Grik5, Grin2a, Grin3a, Grk4, Grk5, Grn, Guca1b, Gucy2g, Hdac4, Hdac6, Hexb, Hey2, Hfe2, Hint1, Hipk3, Hmga2, Hmgn3, Hnf1a, Hrh2, Hspa5, Hspd1, Htr2a, Hyal2, Ica1, Igf1, Igfbp4, Igfbp5, Igsf10, Il1a, Il1b, Il1r1, Il1rap, Il1rl2, Il20rb, Il2rb, Il7, Inha, Inhba, Inpp5f, Insig2, Insrr, Intu, Invs, Iqgap2, Irak1, Irak3, Irf1, Irf4, Irs2, Itch, Itga1, Itga3, Itga8, Itgad, Itgae, Itgal, Itgam, Itgav, Itgax, Itgb1, Itgb1bp1, Itgb2, Itgb4, Itk, Itm2b, Itpr3, Itsn1, Itsn2, Ivns1abp, Jag2, Jmjd6, Jun, Kcnc4, Kcnn2, Kcnn4, Kctd8, Kdm6a, Kif1b, Kif3a, Kif7, Klhl12, Kras, Krt18, Lat2, Lck, Ldb1, Lefty1, Lgals1, Lmcd1, Lpar1, Lpar2, Lrba, Lrp2, Lrp5, Ltb4r1, Ltbp3, Ltk, Lyn, Mad2l2, Magi3, Mal, Malt1, Maoa, Maob, Map3k12, Mapk1, Mapkapk2, Mark2, Med24, Men1, Mfng, Mkks, Mllt3, Mllt4, Mlxipl, Mpdz, Mras, Mrc1, Mst1r, Mt3, Myc, Myh6, Myo1e, Myocd, Myrip, Nab1, Ncdn, Ncf1, Ncoa3, Nek8, Neurl1b, Nf1, Nfat5, Nfkbib, Nlgn2, Nlrx1, Nnat, Nog, Noxo1, Npr3, Nr5a2, Nrg1, Nudt1, Nup155, Nup62, Olfr1384, Olfr543, Olfr691, Olfr78, Omp, Onecut2, Opn1sw, Opn3, Osm, P2rx4, P2rx7, Pafah1b2, Pak1, Pak3, Palm, Pcdhb16, Pdcd11, Pde1c, Pde3b, Pde4a, Pde6h, Pde7a, Pde8a, Pde8b, Pdgfb, Pdk2, Pdlim5, Pdpn, Pecam1, Peli1, Pf4, Pfdn5, Pgap2, Phrf1, Pias2, Pik3c3, Pik3r3, Pla2g6, Pla2r1, Plagl2, Plau, Plcb3, Plch1, Pld1, Plekhg3, Plekhg6, Plk3, Plxnb3, Pnp, Postn, Pou1f1, Ppara, Pparg, Ppargc1b, Ppfia3, Ppp1r14c, Ppp2r1a, Ppp2r5a, Ppp2r5e, Prex1, Prkag2, Prkdc, Prokr2, Psd4, Psme3, Ptch1, Ptger1, Ptger3, Ptgir, Ptgs2, Pth1r, Ptk2b, Ptpra, Ptpre, Ptprj, Ptprk, Rab12, Rab27a, Rab37, Rab3c, Rabl5, Rad9a, Ralb, Ralbp1, Rapgef4, Rapsn, Rasa2, Rasal1, Rasgef1c, Rasl10b, Rasl2-9, Rassf9, Rbm15, Rbm38, Rcan1, Reck, Rem1, Retn, Rgn, Rgs16, Rgs19, Rgs3, Rgs9bp, Rhod, Rims2, Rin1, Ripk2, Rit1, Rnf31, Rock1, Rorc, Rp1, Rps6ka6, Rptor, Rras, Rrm2b, Runx2, S1pr5, Scai, Scarb1, Scn10a, Scn4b, Scn5a, Scrib, Sel1l, Serinc3, Serinc5, Serpine1, Sfrp1, Sfrp2, Shank3, Shc1, Shc3, Sigirr, Siva1, Ski, Slc11a1, Slc1a2, Slc1a3, Slc35c2, Smad5, Smad6, Smo, Smpd3, Snx32, Snx33, Socs1, Socs6, Sod2, Sort1, Sox8, Sparc, Spata13, Spred2, Sstr2, Sstr4, Stat2, Stk38, Stmn3, Strn3, Stub1, Stxbp1, Stxbp3a, Sulf1, Syk, Syvn1, Tbx1, Tbx3, Tenm2, Tert, Tgfb1, Tgfb2, Tgfb3, Tgfbr1, Tgfbr3, Tgfbrap1, Thbs2, Themis, Thoc1, Tle2, Tlr1, Tlr3, Tlr7, Tmem204, Tnfaip3, Tnfrsf1a, Tnfsf15, Tnik, Tollip, Tom1l1, Tox3, Tpgs1, Traf2, Trem2, Trim38, Trio, Trp53, Trp53inp2, Trpv4, Tspan12, Tspyl5, Tti1, Ttn, Tub, Tulp4, Tyro3, Ucn, Ulk2, Usp46, Vapb, Vmn1r45, Vmn2r57, Vwc2, Wdr45, Wfikkn2, Wif1, Wisp1, Wnt1, Wnt11, Wnt16, Wnt4, Wnt6, Wnt9a, Wnt9b, Wwtr1, Xiap, Yod1, Ywhaq, Zbtb33, Zc3hc1, Zfp13, Zranb1 | 3637 | cell communication |
| GO:0023052 | 2.047e-04 | 509.5 | 611 Aatk, Adam11, Adam19, Adam22, Adam5, Adamts3, Adcy4, Adcy6, Adcy8, Adora2a, Adra1d, Agrp, Aimp1, Akap2, Akt1s1, Aloxe3, Amer1, Ankrd26, Ap3d1, Apc2, Aph1a, Aplnr, Apoc3, Aqp1, Arap3, Arhgap11a, Arhgap15, Arhgap18, Arhgap20, Arhgap21, Arhgap24, Arhgap31, Arhgap42, Arhgap6, Arhgef1, Arhgef10, Arhgef37, Arid1a, Arid5b, Arl11, Arl13a, Arl5c, Arl6, Arntl, Asb10, Asb13, Asb5, Asic1, Asxl1, Avpr1a, Avpr2, Axin1, Baiap2, Bcap31, Bcl2, Bcl2l2, Bcl9l, Bex1, Bloc1s2, Bmf, Bmp10, Bmp4, Bmpr1b, Bnip2, Brca1, C1qtnf1, C1qtnf2, C5ar2, Cacna1b, Cacna1i, Cacnb2, Canx, Caprin2, Capzb, Car8, Card6, Casp1, Casp2, Casp7, Cav3, Cck, Ccl11, Ccl17, Ccl19, Ccl2, Ccl5, Ccnd3, Ccny, Ccr1l1, Cd22, Cd28, Cd36, Cd38, Cd3d, Cd4, Cd5, Cd79a, Cdc42bpa, Cdc42bpb, Cdc42bpg, Cdh1, Cdh2, Cdh8, Cdk14, Cdk5r1, Cds2, Cflar, Chi3l1, Chn2, Cish, Cited1, Clca2, Clec2d, Cln3, Cmya5, Cntnap1, Col18a1, Col1a1, Cplx1, Cr2, Creb1, Crk, Csf1r, Csf2rb, Cspg5, Ctf1, Ctnnd2, Ctsc, Ctsh, Cx3cl1, Cxadr, Cxcl13, Cxcl5, Cxcr4, Cxxc4, Cysltr1, Dclk3, Ddr1, Depdc1a, Depdc7, Derl2, Dgke, Dgki, Dhx58, Dixdc1, Dkk2, Dkk3, Dlgap1, Dlgap3, Doc2b, Dock10, Dock5, Dock6, Dok4, Dsp, Dtx4, Duox2, Dusp3, Dut, Dvl3, Edn3, Efnb2, Efnb3, Egr1, Egr2, Ei24, Eif2a, Eif2b4, Emp2, Entpd1, Epas1, Ern1, Exoc3l, F2, F7, Fa2h, Fam110c, Fam13b, Fbxo8, Fbxo9, Fcgr1, Fcgr2b, Fgd5, Fgd6, Fgf10, Fgf16, Fgf20, Fgf21, Fgf6, Fgf7, Fgfbp1, Fgfr2, Fgfr4, Fhit, Fkbp8, Foxo6, Fpr2, Frzb, Fst, Fxn, Fyn, Fzd3, G2e3, Gab1, Gabbr2, Gabra1, Gabrd, Gabrr2, Garnl3, Gas6, Gata6, Gdf6, Gdi2, Gdnf, Gem, Ggct, Gjc2, Gli1, Gli2, Glis2, Gna12, Gnai1, Gnai2, Gnas, Gng7, Gpld1, Gpr12, Gpr150, Gpr152, Gpr156, Gpr157, Gpr171, Gpr183, Gpr4, Gpr55, Gpr61, Gpr65, Gpr84, Gps2, Gpx1, Grid2ip, Grik5, Grin2a, Grin3a, Grk4, Grk5, Grn, Guca1b, Gucy2g, Hdac4, Hdac6, Hexb, Hey2, Hfe2, Hint1, Hipk3, Hmga2, Hmgn3, Hnf1a, Hrh2, Hspa5, Hspd1, Htr2a, Hyal2, Ica1, Igf1, Igfbp4, Igfbp5, Igsf10, Il1a, Il1b, Il1r1, Il1rap, Il1rl2, Il20rb, Il2rb, Il7, Inha, Inhba, Inpp5f, Insig2, Insrr, Intu, Invs, Iqgap2, Irak1, Irak3, Irf1, Irf4, Irs2, Itch, Itga1, Itga3, Itga8, Itgad, Itgae, Itgal, Itgam, Itgav, Itgax, Itgb1, Itgb1bp1, Itgb2, Itgb4, Itk, Itm2b, Itpr3, Itsn1, Itsn2, Ivns1abp, Jag2, Jmjd6, Jun, Kcnc4, Kcne1, Kcne2, Kcnn2, Kcnn4, Kctd8, Kdm6a, Kif1b, Kif3a, Kif7, Klhl12, Kras, Krt18, Lat2, Lck, Ldb1, Lefty1, Lgals1, Lmcd1, Lpar1, Lpar2, Lrba, Lrp2, Lrp5, Ltb4r1, Ltbp3, Ltk, Lyn, Mad2l2, Magi3, Mal, Malt1, Maoa, Maob, Map3k12, Mapk1, Mapkapk2, Mark2, Med24, Men1, Mfng, Mkks, Mllt3, Mllt4, Mlxipl, Mpdz, Mras, Mrc1, Mst1r, Mt3, Myc, Myh6, Myo1e, Myocd, Myrip, Nab1, Ncdn, Ncoa3, Nek8, Neurl1b, Nf1, Nfat5, Nfkbib, Nlgn2, Nlrx1, Nnat, Nog, Npr3, Nr5a2, Nrg1, Nsf, Nup155, Nup62, Olfr1384, Olfr543, Olfr691, Olfr78, Omp, Onecut2, Opn1sw, Opn3, Osm, P2rx4, P2rx7, Pak1, Pak3, Palm, Pcdhb16, Pdcd11, Pde1c, Pde3b, Pde4a, Pde6h, Pde7a, Pde8a, Pde8b, Pdgfb, Pdk2, Pdlim5, Pdpn, Pecam1, Peli1, Pf4, Pfdn5, Pgap2, Phrf1, Pias2, Pik3c3, Pik3r3, Pla2g6, Pla2r1, Plagl2, Plau, Plcb3, Plch1, Plekhg3, Plekhg6, Plk3, Plxnb3, Pnp, Postn, Pou1f1, Ppara, Pparg, Ppargc1b, Ppfia3, Ppp1r14c, Ppp2r1a, Ppp2r5a, Ppp2r5e, Prex1, Prkag2, Prkdc, Prokr2, Psd4, Psme3, Ptch1, Ptger1, Ptger3, Ptgir, Ptgs2, Pth1r, Ptk2b, Ptpra, Ptpre, Ptprj, Ptprk, Rab12, Rab27a, Rab37, Rab3c, Rabl5, Rad9a, Ralb, Ralbp1, Rapgef4, Rapsn, Rasa2, Rasal1, Rasgef1c, Rasl10b, Rasl2-9, Rassf9, Rbm15, Rbm38, Rcan1, Reck, Rem1, Retn, Rgn, Rgs16, Rgs19, Rgs3, Rgs9bp, Rhod, Rims2, Rin1, Ripk2, Rit1, Rnf31, Rock1, Rorc, Rp1, Rps6ka6, Rptor, Rras, Rrm2b, Runx2, S1pr5, Scai, Scarb1, Scn10a, Scn4b, Scn5a, Scrib, Sel1l, Serinc3, Serinc5, Serpine1, Sfrp1, Sfrp2, Shank3, Shc1, Shc3, Sigirr, Siva1, Ski, Slc11a1, Slc1a3, Slc35c2, Smad5, Smad6, Smo, Smpd3, Socs1, Socs6, Sod2, Sort1, Sox8, Sparc, Spata13, Spred2, Sstr2, Sstr4, Stat2, Stk38, Stmn3, Strn3, Stub1, Stxbp1, Stxbp3a, Sulf1, Syk, Syvn1, Tbx1, Tbx3, Tenm2, Tert, Tgfb1, Tgfb2, Tgfb3, Tgfbr1, Tgfbr3, Tgfbrap1, Thbs2, Themis, Thoc1, Tle2, Tlr1, Tlr3, Tlr7, Tmem204, Tnfaip3, Tnfrsf1a, Tnfsf15, Tnik, Tollip, Tom1l1, Tox3, Tpgs1, Traf2, Trem2, Trim38, Trio, Trp53, Trpv4, Tspan12, Tspyl5, Tti1, Ttn, Tub, Tulp4, Tyro3, Ucn, Ulk2, Usp46, Vapb, Vmn1r45, Vmn2r57, Vwc2, Wfikkn2, Wif1, Wisp1, Wnt1, Wnt11, Wnt16, Wnt4, Wnt6, Wnt9a, Wnt9b, Wwtr1, Xiap, Yod1, Ywhaq, Zbtb33, Zc3hc1, Zfp13, Zranb1 | 3542 | signaling |
| GO:0044700 | 2.047e-04 | 509.5 | 611 Aatk, Adam11, Adam19, Adam22, Adam5, Adamts3, Adcy4, Adcy6, Adcy8, Adora2a, Adra1d, Agrp, Aimp1, Akap2, Akt1s1, Aloxe3, Amer1, Ankrd26, Ap3d1, Apc2, Aph1a, Aplnr, Apoc3, Aqp1, Arap3, Arhgap11a, Arhgap15, Arhgap18, Arhgap20, Arhgap21, Arhgap24, Arhgap31, Arhgap42, Arhgap6, Arhgef1, Arhgef10, Arhgef37, Arid1a, Arid5b, Arl11, Arl13a, Arl5c, Arl6, Arntl, Asb10, Asb13, Asb5, Asic1, Asxl1, Avpr1a, Avpr2, Axin1, Baiap2, Bcap31, Bcl2, Bcl2l2, Bcl9l, Bex1, Bloc1s2, Bmf, Bmp10, Bmp4, Bmpr1b, Bnip2, Brca1, C1qtnf1, C1qtnf2, C5ar2, Cacna1b, Cacna1i, Cacnb2, Canx, Caprin2, Capzb, Car8, Card6, Casp1, Casp2, Casp7, Cav3, Cck, Ccl11, Ccl17, Ccl19, Ccl2, Ccl5, Ccnd3, Ccny, Ccr1l1, Cd22, Cd28, Cd36, Cd38, Cd3d, Cd4, Cd5, Cd79a, Cdc42bpa, Cdc42bpb, Cdc42bpg, Cdh1, Cdh2, Cdh8, Cdk14, Cdk5r1, Cds2, Cflar, Chi3l1, Chn2, Cish, Cited1, Clca2, Clec2d, Cln3, Cmya5, Cntnap1, Col18a1, Col1a1, Cplx1, Cr2, Creb1, Crk, Csf1r, Csf2rb, Cspg5, Ctf1, Ctnnd2, Ctsc, Ctsh, Cx3cl1, Cxadr, Cxcl13, Cxcl5, Cxcr4, Cxxc4, Cysltr1, Dclk3, Ddr1, Depdc1a, Depdc7, Derl2, Dgke, Dgki, Dhx58, Dixdc1, Dkk2, Dkk3, Dlgap1, Dlgap3, Doc2b, Dock10, Dock5, Dock6, Dok4, Dsp, Dtx4, Duox2, Dusp3, Dut, Dvl3, Edn3, Efnb2, Efnb3, Egr1, Egr2, Ei24, Eif2a, Eif2b4, Emp2, Entpd1, Epas1, Ern1, Exoc3l, F2, F7, Fa2h, Fam110c, Fam13b, Fbxo8, Fbxo9, Fcgr1, Fcgr2b, Fgd5, Fgd6, Fgf10, Fgf16, Fgf20, Fgf21, Fgf6, Fgf7, Fgfbp1, Fgfr2, Fgfr4, Fhit, Fkbp8, Foxo6, Fpr2, Frzb, Fst, Fxn, Fyn, Fzd3, G2e3, Gab1, Gabbr2, Gabra1, Gabrd, Gabrr2, Garnl3, Gas6, Gata6, Gdf6, Gdi2, Gdnf, Gem, Ggct, Gjc2, Gli1, Gli2, Glis2, Gna12, Gnai1, Gnai2, Gnas, Gng7, Gpld1, Gpr12, Gpr150, Gpr152, Gpr156, Gpr157, Gpr171, Gpr183, Gpr4, Gpr55, Gpr61, Gpr65, Gpr84, Gps2, Gpx1, Grid2ip, Grik5, Grin2a, Grin3a, Grk4, Grk5, Grn, Guca1b, Gucy2g, Hdac4, Hdac6, Hexb, Hey2, Hfe2, Hint1, Hipk3, Hmga2, Hmgn3, Hnf1a, Hrh2, Hspa5, Hspd1, Htr2a, Hyal2, Ica1, Igf1, Igfbp4, Igfbp5, Igsf10, Il1a, Il1b, Il1r1, Il1rap, Il1rl2, Il20rb, Il2rb, Il7, Inha, Inhba, Inpp5f, Insig2, Insrr, Intu, Invs, Iqgap2, Irak1, Irak3, Irf1, Irf4, Irs2, Itch, Itga1, Itga3, Itga8, Itgad, Itgae, Itgal, Itgam, Itgav, Itgax, Itgb1, Itgb1bp1, Itgb2, Itgb4, Itk, Itm2b, Itpr3, Itsn1, Itsn2, Ivns1abp, Jag2, Jmjd6, Jun, Kcnc4, Kcne1, Kcne2, Kcnn2, Kcnn4, Kctd8, Kdm6a, Kif1b, Kif3a, Kif7, Klhl12, Kras, Krt18, Lat2, Lck, Ldb1, Lefty1, Lgals1, Lmcd1, Lpar1, Lpar2, Lrba, Lrp2, Lrp5, Ltb4r1, Ltbp3, Ltk, Lyn, Mad2l2, Magi3, Mal, Malt1, Maoa, Maob, Map3k12, Mapk1, Mapkapk2, Mark2, Med24, Men1, Mfng, Mkks, Mllt3, Mllt4, Mlxipl, Mpdz, Mras, Mrc1, Mst1r, Mt3, Myc, Myh6, Myo1e, Myocd, Myrip, Nab1, Ncdn, Ncoa3, Nek8, Neurl1b, Nf1, Nfat5, Nfkbib, Nlgn2, Nlrx1, Nnat, Nog, Npr3, Nr5a2, Nrg1, Nsf, Nup155, Nup62, Olfr1384, Olfr543, Olfr691, Olfr78, Omp, Onecut2, Opn1sw, Opn3, Osm, P2rx4, P2rx7, Pak1, Pak3, Palm, Pcdhb16, Pdcd11, Pde1c, Pde3b, Pde4a, Pde6h, Pde7a, Pde8a, Pde8b, Pdgfb, Pdk2, Pdlim5, Pdpn, Pecam1, Peli1, Pf4, Pfdn5, Pgap2, Phrf1, Pias2, Pik3c3, Pik3r3, Pla2g6, Pla2r1, Plagl2, Plau, Plcb3, Plch1, Plekhg3, Plekhg6, Plk3, Plxnb3, Pnp, Postn, Pou1f1, Ppara, Pparg, Ppargc1b, Ppfia3, Ppp1r14c, Ppp2r1a, Ppp2r5a, Ppp2r5e, Prex1, Prkag2, Prkdc, Prokr2, Psd4, Psme3, Ptch1, Ptger1, Ptger3, Ptgir, Ptgs2, Pth1r, Ptk2b, Ptpra, Ptpre, Ptprj, Ptprk, Rab12, Rab27a, Rab37, Rab3c, Rabl5, Rad9a, Ralb, Ralbp1, Rapgef4, Rapsn, Rasa2, Rasal1, Rasgef1c, Rasl10b, Rasl2-9, Rassf9, Rbm15, Rbm38, Rcan1, Reck, Rem1, Retn, Rgn, Rgs16, Rgs19, Rgs3, Rgs9bp, Rhod, Rims2, Rin1, Ripk2, Rit1, Rnf31, Rock1, Rorc, Rp1, Rps6ka6, Rptor, Rras, Rrm2b, Runx2, S1pr5, Scai, Scarb1, Scn10a, Scn4b, Scn5a, Scrib, Sel1l, Serinc3, Serinc5, Serpine1, Sfrp1, Sfrp2, Shank3, Shc1, Shc3, Sigirr, Siva1, Ski, Slc11a1, Slc1a3, Slc35c2, Smad5, Smad6, Smo, Smpd3, Socs1, Socs6, Sod2, Sort1, Sox8, Sparc, Spata13, Spred2, Sstr2, Sstr4, Stat2, Stk38, Stmn3, Strn3, Stub1, Stxbp1, Stxbp3a, Sulf1, Syk, Syvn1, Tbx1, Tbx3, Tenm2, Tert, Tgfb1, Tgfb2, Tgfb3, Tgfbr1, Tgfbr3, Tgfbrap1, Thbs2, Themis, Thoc1, Tle2, Tlr1, Tlr3, Tlr7, Tmem204, Tnfaip3, Tnfrsf1a, Tnfsf15, Tnik, Tollip, Tom1l1, Tox3, Tpgs1, Traf2, Trem2, Trim38, Trio, Trp53, Trpv4, Tspan12, Tspyl5, Tti1, Ttn, Tub, Tulp4, Tyro3, Ucn, Ulk2, Usp46, Vapb, Vmn1r45, Vmn2r57, Vwc2, Wfikkn2, Wif1, Wisp1, Wnt1, Wnt11, Wnt16, Wnt4, Wnt6, Wnt9a, Wnt9b, Wwtr1, Xiap, Yod1, Ywhaq, Zbtb33, Zc3hc1, Zfp13, Zranb1 | 3542 | single organism signaling |
| GO:0044699 | 6.189e-04 | 1238 | 1350 1810011O10Rik, 1810043G02Rik, 2210018M11Rik, 2610018G03Rik, 2700094K13Rik, 4930506M07Rik, 9430023L20Rik, 9630033F20Rik, Aamdc, Aatk, Abca2, Abcb7, Acsl6, Acta2, Actn3, Actr8, Adam11, Adam12, Adam15, Adam19, Adam22, Adam5, Adamts3, Adcy4, Adcy6, Adcy8, Adora2a, Adra1d, AF251705, Aff2, Agrp, Aifm3, Aimp1, Akap1, Akap2, Akt1s1, Alad, Alkbh2, Alkbh4, Alkbh5, Alox5, Aloxe3, Amer1, Anapc15, Angptl6, Ankle2, Ankrd26, Ano1, Anpep, Anxa11, Anxa2, Anxa3, Anxa7, Ap1g1, Ap3d1, Ap4e1, Ap5z1, Apbb2, Apc2, Apex1, Aph1a, Aplnr, Apoc3, Apoc4, Aqp1, Aqp2, Arap3, Arhgap11a, Arhgap15, Arhgap18, Arhgap20, Arhgap21, Arhgap24, Arhgap31, Arhgap42, Arhgap6, Arhgef1, Arhgef10, Arhgef37, Arhgef5, Arid1a, Arid5b, Arl11, Arl13a, Arl5c, Arl6, Arl6ip1, Armcx3, Arntl, Asb10, Asb13, Asb5, Asic1, Asl, Aspa, Asxl1, Atf2, Atf3, Atg9a, Atox1, Atp1b2, Atp2b4, Atp6v0a1, Atp6v0a4, Atp6v0c, Atp6v0e2, Atp8a1, Atp8b2, Atp9a, Atxn2, Avil, Avpr1a, Avpr2, Axin1, Bag5, Bahd1, Baiap2, Basp1, Bbs5, Bcap31, Bcl2, Bcl2l2, Bcl9l, Bex1, Bfsp2, Bglap3, Bhlhe40, Birc2, Bloc1s2, Blzf1, Bmf, Bmp10, Bmp4, Bmpr1b, Bnip2, Bod1, Bop1, Brca1, Brd1, Brwd3, C1qtnf1, C1qtnf2, C1s, C4bp, C5ar2, C77370, Cachd1, Cacna1b, Cacna1i, Cacna2d1, Cacnb1, Cacnb2, Cacng6, Cadm1, Cadm4, Calm1, Calm2, Calm3, Canx, Cap1, Caprin2, Capzb, Car8, Card6, Casc1, Casp1, Casp2, Casp4, Casp7, Catsper3, Cav3, Cbx4, Ccar1, Ccdc23, Ccdc39, Ccdc64, Cck, Ccl11, Ccl17, Ccl19, Ccl2, Ccl5, Ccl6, Ccnc, Ccnd3, Ccne2, Ccnt1, Ccny, Ccp110, Ccr1l1, Cct8, Cd22, Cd28, Cd2ap, Cd300a, Cd33, Cd36, Cd38, Cd3d, Cd4, Cd5, Cd55, Cd5l, Cd63, Cd79a, Cd83, Cd99l2, Cda, Cdc25c, Cdc42bpa, Cdc42bpb, Cdc42bpg, Cdc42ep2, Cdc5l, Cdca3, Cdh1, Cdh10, Cdh2, Cdh23, Cdh8, Cdhr4, Cdk14, Cdk5r1, Cdk7, Cdkn2c, Cdo1, Cds2, Cenpc1, Cenpt, Cep76, Ces1d, Cetn3, Cfh, Cflar, Chi3l1, Chl1, Chn2, Cish, Cited1, Clca2, Clcc1, Clcn4-2, Clec11a, Clec2d, Clec3b, Clec4e, Cln3, Clpb, Clstn2, Clstn3, Clvs1, Cma1, Cmya5, Cnnm1, Cnnm4, Cnot10, Cntnap1, Cntrob, Col10a1, Col11a2, Col13a1, Col14a1, Col18a1, Col19a1, Col1a1, Col28a1, Col4a4, Col4a5, Col5a1, Col5a2, Col6a2, Col7a1, Coro1a, Coro1b, Coro1c, Cp, Cplx1, Cr2, Crbn, Creb1, Creb3l2, Creb5, Crebl2, Crip3, Cript, Crk, Crkl, Crot, Cry1, Csf1r, Csf2rb, Csf3r, Cspg5, Csrp1, Cst10, Cstad, Ctf1, Ctla2a, Ctnna3, Ctnnd2, Ctsc, Ctsh, Ctsk, Ctsl, Cubn, Cul1, Cul4b, Cx3cl1, Cxadr, Cxcl13, Cxcl2, Cxcl5, Cxcr4, Cxxc4, Cyb5d2, Cyp1a2, Cyp7a1, Cysltr1, D130043K22Rik, D230025D16Rik, Daam1, Dad1, Dap3, Dclk3, Dcstamp, Dctn6, Ddb2, Ddr1, Ddx11, Ddx39, Dek, Depdc1a, Depdc7, Derl2, Dffa, Dffb, Dgke, Dgki, Dhx58, Dixdc1, Dkk2, Dkk3, Dlgap1, Dlgap3, Dlx4, Dmap1, Dmrt2, Dmrta1, Dmtf1, Dnah12, Dnajc2, Dnajc5, Dnm2, Doc2b, Dock10, Dock5, Dock6, Dok4, Dpf1, Dpp4, Dscr3, Dsp, Dstn, Dtx4, Duox2, Dusp3, Dut, Dvl3, Dync1i2, Dync2h1, Dyrk3, Ebf1, Ebp, Edil3, Edn3, Eed, Efcab4a, Efemp2, Efnb2, Efnb3, Egln2, Egr1, Egr2, Ehd1, Ehd2, Ehd4, Ei24, Eif2a, Eif2b3, Eif2b4, Eif5a, Eif5a2, Ell, Elmo2, Eme2, Emp2, Emp3, En2, Enah, Endog, Eno1, Entpd1, Epas1, Epb4.1l1, Ephx2, Epm2a, Ercc3, Ercc8, Ermp1, Ern1, Etv4, Etv6, Eva1a, Exoc3, Exoc3l, Exph5, Ext1, Eya3, Ezr, F2, F5, F7, Fa2h, Fam110c, Fam13b, Fam53a, Fam58b, Fap, Fblim1, Fbln1, Fbln2, Fbxo8, Fbxo9, Fcgr1, Fcgr2b, Fen1, Fgd5, Fgd6, Fgf10, Fgf16, Fgf20, Fgf21, Fgf6, Fgf7, Fgfbp1, Fgfr1op, Fgfr2, Fgfr4, Fgl2, Fhit, Fkbp8, Fmo2, Fmr1, Folr2, Fosl1, Foxj3, Foxo6, Foxr1, Fpr2, Frem1, Frem2, Frmd4b, Frzb, Fscn1, Fst, Fxn, Fxyd7, Fyn, Fzd3, G2e3, Gab1, Gab3, Gabbr2, Gabra1, Gabrd, Gabrr2, Garnl3, Gas6, Gata6, Gatad2a, Gbp4, Gcc2, Gcnt1, Gdf6, Gdi2, Gdnf, Gem, Gga3, Ggct, Ggn, Gjb5, Gjc2, Gli1, Gli2, Glis2, Gm11744, Gna12, Gnai1, Gnai2, Gnas, Gng7, Golga2, Gpld1, Gpr116, Gpr12, Gpr150, Gpr152, Gpr156, Gpr157, Gpr171, Gpr183, Gpr4, Gpr55, Gpr61, Gpr65, Gpr84, Gps2, Gpx1, Gramd4, Grid2ip, Grik4, Grik5, Grin2a, Grin3a, Grk4, Grk5, Grn, Guca1b, Gucy2g, Guk1, Gypa, Gzmm, H2-Ab1, H2afx, H2afy2, Has1, Haus6, Hdac10, Hdac4, Hdac6, Henmt1, Hexb, Hey2, Hfe2, Hint1, Hipk3, Hmga2, Hmgn3, Hnf1a, Hook3, Hoxd9, Hrh2, Hs6st1, Hsd17b4, Hsh2d, Hsp90b1, Hspa1b, Hspa5, Hspa8, Hspd1, Htatip2, Htr2a, Hyal2, Hyal3, Hydin, Ica1, Ift81, Igf1, Igfbp4, Igfbp5, Igfbp7, Igfbpl1, Igsf10, Igsf11, Igsf9, Il16, Il1a, Il1b, Il1f9, Il1r1, Il1rap, Il1rl2, Il20rb, Il25, Il2rb, Il5ra, Il7, Ing1, Inha, Inhba, Ino80c, Inpp5f, Insig2, Insrr, Ints7, Intu, Invs, Iqgap2, Irak1, Irak3, Irf1, Irf4, Irf9, Irs2, Irx4, Irx5, Isg15, Itch, Itga1, Itga3, Itga8, Itgad, Itgae, Itgal, Itgam, Itgav, Itgax, Itgb1, Itgb1bp1, Itgb2, Itgb4, Itk, Itm2b, Itpr3, Itsn1, Itsn2, Ivns1abp, Jag2, Jdp2, Jmjd6, Jun, Kcnab1, Kcnab2, Kcnb1, Kcnc1, Kcnc4, Kcne1, Kcne2, Kcng2, Kcng4, Kcnip1, Kcnj16, Kcnn2, Kcnn3, Kcnn4, Kcnu1, Kctd14, Kctd8, Kdm4d, Kdm6a, Kif17, Kif18a, Kif1a, Kif1b, Kif22, Kif23, Kif26b, Kif27, Kif3a, Kif3b, Kif7, Kirrel, Klhl12, Kng1, Kpna1, Kpnb1, Kras, Krt10, Krt14, Krt18, Krt19, Lat2, Lck, Ldb1, Lefty1, Lgals1, Lgals8, Limk2, Lmcd1, Lor, Loxl3, Lpar1, Lpar2, Lrba, Lrp2, Lrp5, Lrrc52, Lsm11, Ltb4r1, Ltbp3, Ltk, Lyn, Lyst, Lyz2, Lztfl1, Mad2l2, Maf, Magi3, Magoh, Mal, Malt1, Man1a2, Maoa, Maob, Map1a, Map2, Map3k12, Map4, Mapk1, Mapkapk2, Marf1, Mark2, Marveld1, Mbtps1, Mdm1, Med24, Men1, Mepce, Mfap5, Mfn1, Mfng, Mfsd9, Mgarp, Mgst1, Misp, Mkks, Mlh3, Mllt3, Mllt4, Mlxipl, Mme, Mmgt1, Mmp13, Mmp16, Mmp3, Mpdz, Mpg, Mpz, Mras, Mrc1, Msh3, Msi2, Msl3, Msn, Msra, Mst1r, Mt3, Mtfr2, Mus81, Mvd, Myb, Myc, Mycbp2, Mycbpap, Myh6, Mylk3, Myo1e, Myo1f, Myo5c, Myocd, Myrip, Nab1, Naip1, Ncam2, Ncdn, Nceh1, Ncf1, Ncf2, Ncl, Ncoa3, Ncor2, Ndufaf7, Ndufs1, Nefh, Nek1, Nek8, Nek9, Neu1, Neurl1b, Nf1, Nfat5, Nfic, Nfkbib, Nid2, Nlgn2, Nlrp1a, Nlrp5, Nlrx1, Nme6, Nmt1, Nnat, Nob1, Nog, Noxo1, Npr3, Nqo1, Nr5a2, Nrg1, Nsd1, Nsf, Ntn3, Ntng1, Ntng2, Nubpl, Nudc, Nudt1, Nup107, Nup155, Nup54, Nup62, Nusap1, Nxf2, Nxt1, Nyx, Ogn, Olfr1384, Olfr543, Olfr691, Olfr78, Omp, Onecut2, Opn1sw, Opn3, Optn, Orai1, Os9, Oser1, Osgin1, Osm, Otog, Oxr1, P2rx4, P2rx7, Pacrg, Pacsin3, Pafah1b2, Pak1, Pak3, Palm, Pcdh19, Pcdh20, Pcdhb13, Pcdhb15, Pcdhb16, Pcdhb17, Pcdhb18, Pcdhb19, Pcdhb2, Pcdhb8, Pcnt, Pcsk6, Pcyt1b, Pdcd11, Pdcd2, Pde1c, Pde3b, Pde4a, Pde6h, Pde7a, Pde8a, Pde8b, Pdgfb, Pdk2, Pdlim3, Pdlim5, Pdpn, Pdzrn3, Pecam1, Peli1, Pex7, Pf4, Pfdn5, Pfn4, Pgap2, Phf10, Phf2, Phf8, Phrf1, Pi4k2a, Pias2, Pik3c3, Pik3r3, Pip5k1a, Pitpna, Piwil2, Pla2g16, Pla2g4a, Pla2g6, Pla2r1, Plagl1, Plagl2, Plau, Plaur, Plcb3, Plch1, Pld1, Plekha7, Plekha8, Plekhg3, Plekhg6, Plk3, Plk4, Plxnb3, Pnp, Pnpla8, Podxl2, Pold3, Polg2, Poli, Polr3f, Polrmt, Pon1, Pon3, Postn, Pou1f1, Pou4f1, Ppara, Pparg, Ppargc1b, Ppfia3, Ppl, Ppm1e, Ppm1g, Ppp1cc, Ppp1r12a, Ppp1r13l, Ppp1r14c, Ppp2r1a, Ppp2r5a, Ppp2r5e, Prcc, Prex1, Prf1, Prkag2, Prkdc, Prkx, Prnd, Prokr2, Prom1, Prps1, Psd4, Psme3, Pstpip1, Ptch1, Ptgds, Ptger1, Ptger3, Ptgir, Ptgs1, Ptgs2, Pth1r, Ptk2b, Ptp4a1, Ptpla, Ptpra, Ptpre, Ptprg, Ptprj, Ptprk, Ptprs, Pycr1, Pyhin1, Qars, Rab12, Rab27a, Rab37, Rab3c, Rabl5, Rad23b, Rad9a, Raet1a, Rai1, Ralb, Ralbp1, Ranbp1, Rapgef4, Rapsn, Rasa2, Rasal1, Rasgef1a, Rasgef1c, Rasl10b, Rasl2-9, Rassf9, Rbbp5, Rbm14, Rbm15, Rbm19, Rbm38, Rcan1, Reck, Reep1, Rem1, Retn, Rffl, Rgn, Rgs16, Rgs19, Rgs3, Rgs9bp, Rhod, Rims2, Rin1, Ripk2, Rit1, Rnf31, Robo4, Rock1, Rom1, Ropn1l, Rorc, Rp1, Rprd1b, Rprm, Rps28, Rps6ka6, Rpsa, Rptor, Rras, Rrm2b, Rsph4a, Rtn4, Rtn4r, Rtn4rl2, Rtp4, Rttn, Runx2, Ruvbl1, Ryr1, S100a1, S100a10, S1pr5, Samm50, Sap30bp, Sbf1, Scai, Scarb1, Scd1, Scn10a, Scn3a, Scn4b, Scn5a, Scrib, Sct, Sdad1, Sdf4, Sdk1, Sec22b, Sec23b, Sel1l, Sell, Sema3b, Senp1, Sep15, Sept9, Serinc3, Serinc5, Serpinb6a, Serpinb9, Serpinc1, Serpine1, Sesn1, Sfrp1, Sfrp2, Sfxn1, Sfxn2, Shank3, Shc1, Shc3, Shroom4, Siah1b, Sidt2, Sigirr, Siva1, Ski, Slc10a1, Slc10a3, Slc10a4, Slc11a1, Slc12a8, Slc15a2, Slc16a1, Slc16a13, Slc16a2, Slc17a9, Slc1a1, Slc1a2, Slc1a3, Slc1a5, Slc22a15, Slc22a8, Slc24a4, Slc25a25, Slc25a36, Slc25a41, Slc27a4, Slc2a4, Slc30a4, Slc35a4, Slc35c2, Slc35d2, Slc38a10, Slc38a3, Slc38a4, Slc39a13, Slc39a2, Slc41a1, Slc43a1, Slc6a2, Slc7a4, Slc7a6, Slc8a2, Slc9a8, Slco1a1, Slco1c1, Slco4a1, Slfn3, Slitrk6, Slk, Slx1b, Smad5, Smad6, Smc1b, Smcr7, Smo, Smpd3, Sned1, Snrpe, Snx32, Snx33, Socs1, Socs6, Sod2, Sort1, Sox15, Sox8, Sp2, Sp4, Sp6, Spaca3, Sparc, Spata13, Spata22, Spata25, Spc24, Spi1, Spice1, Spr, Spred2, Spta1, Srd5a1, Srp9, Srpr, Srpx2, Ssh1, Sstr2, Sstr4, Stab2, Stag1, Stag2, Stam, Stard10, Stat2, Stau1, Stk10, Stk38, Stmn3, Stpg1, Stra13, Strip2, Strn3, Stub1, Stxbp1, Stxbp3a, Sulf1, Supv3l1, Suv39h2, Svep1, Sycn, Sycp2, Syk, Syne1, Syt3, Syvn1, Tacc1, Taf1, Taf3, Taf6, Tagln, Tarbp2, Tbata, Tbx1, Tbx3, Tecta, Tekt2, Tenm2, Tert, Tet3, Tfam, Tfeb, Tfpi, Tgfb1, Tgfb2, Tgfb3, Tgfbi, Tgfbr1, Tgfbr3, Tgfbrap1, Tgtp1, Thap1, Thbd, Thbs2, Thbs3, Themis, Thoc1, Thsd7a, Timm44, Timp1, Tk2, Tle2, Tlr1, Tlr3, Tlr7, Tmc3, Tmed10, Tmem123, Tmem165, Tmem204, Tmem219, Tmem66, Tmod3, Tmsb4x, Tmx3, Tnfaip3, Tnfrsf18, Tnfrsf1a, Tnfsf15, Tnik, Tnnt2, Tnrc6c, Tollip, Tom1l1, Tom1l2, Top1, Tox3, Tpcn1, Tpgs1, Tpm1, Tppp3, Tprn, Traf2, Trem2, Trib2, Trim38, Trim39, Trio, Trip13, Tro, Troap, Trp53, Trp53inp2, Trpc4, Trpm3, Trpm7, Trpv4, Tsc22d1, Tspan12, Tspan8, Tspo, Tspyl5, Ttc30b, Tti1, Ttn, Tub, Tubb2a, Tubgcp5, Tulp4, Twf1, Txn2, Txndc12, Txndc9, Txnl1, Tyr, Tyro3, Ubl4, Ucn, Ulk2, Unc119, Uqcr11, Usp21, Usp3, Usp37, Usp4, Usp46, Usp6nl, Uvssa, Vapb, Vcp, Vill, Vipas39, Vmn1r45, Vmn2r57, Vps11, Vps18, Vps33a, Vps33b, Vps36, Vwc2, Wash, Wdr36, Wdr45, Wdr47, Wdr77, Wfikkn2, Wif1, Wisp1, Wnt1, Wnt11, Wnt16, Wnt4, Wnt6, Wnt9a, Wnt9b, Wrap53, Wwtr1, Xiap, Xirp2, Xpnpep3, Xpo6, Yars2, Ykt6, Yod1, Ywhaq, Yy1, Zbtb33, Zc3hc1, Zc4h2, Zdhhc16, Zdhhc22, Zfp13, Zfp281, Zfp318, Zfp335, Zfp346, Zfp384, Zfp709, Zfr, Zfx, Zhx3, Zranb1 | 8607 | single-organism process |
| GO:0050896 | 6.476e-04 | 717.5 | 825 2210018M11Rik, 9430023L20Rik, 9630033F20Rik, Aatk, Actr8, Adam11, Adam19, Adam22, Adam5, Adamts3, Adcy4, Adcy6, Adcy8, Adora2a, Adra1d, Adsl, Aff2, Agrp, Aimp1, Akap1, Akap2, Akt1s1, Alad, Alkbh2, Alkbh5, Alox5, Aloxe3, Amer1, Ankrd26, Anxa11, Anxa2, Anxa3, Anxa7, Aoah, Ap5z1, Apbb2, Apc2, Apex1, Aph1a, Aplnr, Apoc3, Aqp1, Aqp2, Arap3, Arhgap11a, Arhgap15, Arhgap18, Arhgap20, Arhgap21, Arhgap24, Arhgap31, Arhgap42, Arhgap6, Arhgef1, Arhgef10, Arhgef37, Arhgef5, Arid1a, Arid5b, Arl11, Arl13a, Arl5c, Arl6, Arntl, Asb10, Asb13, Asb5, Asic1, Asl, Asxl1, Atf2, Atf6b, Atg9a, Atox1, Avpr1a, Avpr2, Axin1, Baiap2, Bcap31, Bcl2, Bcl2l2, Bcl9l, Bex1, Bfsp2, Bhlhe40, Birc2, Bloc1s2, Bmf, Bmp10, Bmp4, Bmpr1b, Bnip2, Brca1, C1qtnf1, C1qtnf2, C1s, C4bp, C5ar2, C77370, Cacna1b, Cadm1, Caprin2, Capzb, Car8, Card6, Casp1, Casp2, Casp7, Cav3, Cck, Ccl11, Ccl17, Ccl19, Ccl2, Ccl22, Ccl5, Ccl6, Ccl8, Ccl9, Ccnd3, Ccny, Ccr1l1, Cd22, Cd28, Cd300a, Cd36, Cd38, Cd3d, Cd4, Cd5, Cd55, Cd79a, Cd83, Cdc42bpa, Cdc42bpb, Cdc42bpg, Cdh1, Cdh2, Cdh23, Cdh8, Cdk14, Cdk5r1, Cdk7, Cdo1, Cds2, Ces1d, Cfh, Cflar, Chi3l1, Chl1, Chn2, Ciita, Cish, Cited1, Cited4, Clca2, Clec12a, Clec2d, Clec4e, Cln3, Clpb, Cmya5, Cnnm4, Col14a1, Col18a1, Col1a1, Col5a1, Col5a2, Coro1a, Cotl1, Cp, Cr2, Creb1, Creb3l2, Crebzf, Crk, Crot, Cry1, Csf1r, Csf2rb, Csf3r, Ctf1, Ctla2a, Ctnnd2, Ctsc, Ctsh, Cubn, Cul4b, Cx3cl1, Cxadr, Cxcl13, Cxcl2, Cxcl5, Cxcr4, Cxxc4, Cybrd1, Cyp1a2, Cyp7a1, Cysltr1, D2hgdh, Dclk3, Dcstamp, Ddb2, Ddo, Ddr1, Dek, Depdc1a, Depdc7, Derl2, Dgke, Dgki, Dhfr, Dhx58, Dixdc1, Dkk2, Dkk3, Dmap1, Dmrta1, Dnaja2, Dock10, Dock5, Dock6, Dok4, Dpp4, Dtx4, Duox2, Dusp3, Dut, Dvl3, Edn3, Efemp2, Efnb2, Efnb3, Egln2, Egr1, Egr2, Ehd4, Ei24, Eif2a, Eif2b3, Eif2b4, Elmo2, Eme2, Emp2, Enah, Endog, Eno1, Entpd1, Epas1, Ephx2, Epm2a, Ercc3, Ercc8, Ern1, Etv4, Ext1, Eya3, F2, F5, F7, Fam110c, Fam13b, Fbxo8, Fbxo9, Fcgr1, Fcgr2b, Fen1, Fgd5, Fgd6, Fgf10, Fgf16, Fgf20, Fgf21, Fgf6, Fgf7, Fgfbp1, Fgfr2, Fgfr4, Fhit, Fkbp8, Fmo2, Fosl1, Foxo6, Fpr2, Frzb, Fst, Fxn, Fyn, Fzd3, G2e3, Gab1, Gabbr2, Gabra1, Gabrr2, Garnl3, Gart, Gas6, Gata6, Gbp4, Gdf6, Gdi2, Gdnf, Gem, Ggct, Ggt5, Gjc2, Gli1, Gli2, Glis2, Gm11744, Gna12, Gnai1, Gnai2, Gnas, Gng7, Gpld1, Gpr12, Gpr150, Gpr152, Gpr156, Gpr157, Gpr171, Gpr183, Gpr4, Gpr55, Gpr61, Gpr65, Gpr84, Gps2, Gpx1, Grid2ip, Grik5, Grin2a, Grin3a, Grk4, Grk5, Grn, Guca1b, Gucy2g, Gypa, Gys2, H2-Ab1, H2afx, Has1, Hdac4, Hdac6, Hexb, Hey2, Hfe2, Hint1, Hipk3, Hmga2, Hmgn3, Hmox2, Hnf1a, Hoxd9, Hrh2, Hsp90b1, Hspa1b, Hspa5, Hspa8, Hspb6, Hspd1, Htr2a, Hyal2, Hyal3, Ifi44l, Igf1, Igfbp4, Igfbp5, Igsf10, Il16, Il17re, Il1a, Il1b, Il1f9, Il1r1, Il1rap, Il1rl2, Il20rb, Il25, Il2rb, Il5ra, Il7, Inhba, Ino80c, Inpp5f, Insig2, Insrr, Ints7, Intu, Invs, Iqgap2, Irak1, Irak3, Irf1, Irf4, Irs2, Irx5, Isg15, Itch, Itga1, Itga3, Itga8, Itgad, Itgae, Itgal, Itgam, Itgav, Itgax, Itgb1, Itgb1bp1, Itgb2, Itgb4, Itk, Itm2b, Itpr3, Itsn1, Itsn2, Ivns1abp, Jag2, Jmjd6, Jun, Kcnab1, Kcne1, Kcne2, Kcnn4, Kctd8, Kdm6a, Kif22, Kif3a, Kif7, Klhl12, Kng1, Kras, Krt10, Krt18, Kynu, Lat2, Lck, Ldb1, Lefty1, Lgals1, Lgals8, Lmcd1, Lpar1, Lpar2, Lrba, Lrp2, Lrp5, Ltb4r1, Ltbp3, Ltk, Lyn, Lyst, Lyz2, Mad2l2, Magi3, Mal, Malt1, Map2, Map3k12, Mapk1, Mapkapk2, Marf1, Mark2, Med24, Men1, Mest, Mfng, Mgarp, Mgst1, Mkks, Mlh3, Mllt3, Mllt4, Mlxipl, Mme, Mmp3, Mpg, Mras, Mrc1, Msh3, Msi1, Mst1r, Mt3, Mus81, Myb, Myc, Mycbp2, Myh6, Mylk3, Myo1e, Myo1f, Myocd, Naip1, Naprt1, Narfl, Nceh1, Ncf1, Ncf2, Ncoa3, Ndufs2, Nedd8, Nek1, Nek8, Neurl1b, Nf1, Nfat5, Nfkbib, Nlgn2, Nlrp1a, Nlrx1, Nnat, Nog, Npr3, Nqo1, Nr5a2, Nrg1, Nudt1, Nup62, Nyx, Olfr1384, Olfr543, Olfr691, Olfr78, Omp, Onecut2, Opn1sw, Opn3, Os9, Oser1, Osm, Otog, Oxr1, P2rx4, P2rx7, Pafah1b2, Pak1, Pak3, Palm, Parp16, Pdcd11, Pde1c, Pde3b, Pde4a, Pde6h, Pde7a, Pde8a, Pde8b, Pdgfb, Pdk2, Pdpn, Pecam1, Peli1, Pf4, Pfdn5, Pgap2, Phrf1, Pi4k2a, Pias2, Pik3c3, Pik3r3, Pip5k1a, Pla2g4a, Pla2g6, Pla2r1, Plagl2, Plau, Plcb3, Plch1, Pld1, Plekhg3, Plekhg6, Plk3, Plxnb3, Pnp, Pold3, Polg2, Poli, Polr3f, Pon1, Pon3, Postn, Pou1f1, Pou4f1, Ppara, Pparg, Ppargc1b, Ppm1e, Ppp1r14c, Ppp2r1a, Ppp2r5a, Ppp2r5e, Prex1, Prf1, Prkag2, Prkdc, Prokr2, Psd4, Psme3, Ptch1, Ptgds, Ptger1, Ptger3, Ptgir, Ptgs1, Ptgs2, Pth1r, Ptk2b, Ptpra, Ptpre, Ptprj, Ptprk, Pycr1, Pyhin1, Rab12, Rab27a, Rab37, Rab3c, Rabl5, Rad23b, Rad9a, Raet1a, Ralb, Ralbp1, Rapgef4, Rasa2, Rasal1, Rasgef1c, Rasl10b, Rasl2-9, Rassf9, Rbbp5, Rbm15, Rbm38, Rcan1, Reck, Rem1, Retn, Rgn, Rgs16, Rgs19, Rgs3, Rgs9bp, Rhod, Rims2, Rin1, Ripk2, Rit1, Rnf31, Rock1, Rorc, Rp1, Rps6ka6, Rptor, Rras, Rrm2b, Rtn4, Rtn4rl2, Rtp4, Runx2, Ruvbl1, Ryr1, S100a10, S1pr5, Saa2, Sbspon, Scai, Scarb1, Scd1, Scn5a, Scrib, Sct, Sdf4, Sel1l, Sell, Serinc3, Serpina1e, Serpina3f, Serpina3k, Serpina3n, Serpinb9, Serpinc1, Serpine1, Sfrp1, Sfrp2, Shank3, Shc1, Shc3, Sigirr, Siva1, Ski, Slc10a3, Slc11a1, Slc15a2, Slc16a1, Slc1a2, Slc1a3, Slc22a8, Slc25a25, Slc25a36, Slc2a4, Slc30a4, Slc35c2, Slc38a3, Slc6a2, Slitrk6, Slx1b, Smad5, Smad6, Smc1b, Smo, Smpd3, Socs1, Socs6, Sod2, Sort1, Sox15, Sox8, Sparc, Spata13, Spata22, Spr, Spred2, Srd5a1, Srxn1, Ssh1, Sstr2, Sstr4, Stab2, Stat2, Stk38, Stmn3, Stra13, Strn3, Stub1, Stxbp1, Stxbp3a, Sulf1, Syk, Syt3, Syvn1, Tarbp2, Tbx1, Tbx3, Tenm2, Tert, Tfeb, Tfpi, Tgfb1, Tgfb2, Tgfb3, Tgfbr1, Tgfbr3, Tgfbrap1, Tgtp1, Thbd, Themis, Thoc1, Tle2, Tlr1, Tlr3, Tlr7, Tmem204, Tnfaip3, Tnfrsf1a, Tnfsf15, Tnik, Tnnt2, Tollip, Tom1l1, Tox3, Tpgs1, Traf2, Trem2, Trim38, Trio, Trip13, Trp53, Trp53inp2, Trpm7, Trpv4, Tspan12, Tspan8, Tspo, Tspyl5, Tti1, Ttn, Tub, Tulp4, Tyro3, Ucn, Ulk2, Unc119, Usp3, Usp46, Uvssa, Vapb, Vcp, Vmn1r45, Vmn2r57, Vwc2, Wdr45, Wfikkn2, Wif1, Wisp1, Wnt1, Wnt11, Wnt16, Wnt4, Wnt6, Wnt9a, Wnt9b, Wwtr1, Xiap, Yod1, Ywhaq, Yy1, Zbtb33, Zc3hc1, Zfp13, Zfp709, Zfx, Zranb1 | 4988 | response to stimulus |
| GO:0051716 | 2.918e-03 | 573.4 | 669 2210018M11Rik, 9430023L20Rik, 9630033F20Rik, Aatk, Actr8, Adam11, Adam19, Adam22, Adam5, Adamts3, Adcy4, Adcy6, Adcy8, Adora2a, Adra1d, Agrp, Akap2, Akt1s1, Alad, Alkbh2, Aloxe3, Amer1, Ankrd26, Anxa2, Ap5z1, Apc2, Apex1, Aph1a, Aplnr, Apoc3, Aqp1, Aqp2, Arap3, Arhgap11a, Arhgap15, Arhgap18, Arhgap20, Arhgap21, Arhgap24, Arhgap31, Arhgap42, Arhgap6, Arhgef1, Arhgef10, Arhgef37, Arhgef5, Arid1a, Arid5b, Arl11, Arl13a, Arl5c, Arl6, Arntl, Asb10, Asb13, Asb5, Asxl1, Atf2, Atg9a, Avpr1a, Avpr2, Axin1, Baiap2, Bcap31, Bcl2, Bcl2l2, Bcl9l, Bex1, Bloc1s2, Bmf, Bmp10, Bmp4, Bmpr1b, Bnip2, Brca1, C1qtnf1, C1qtnf2, C5ar2, C77370, Caprin2, Capzb, Car8, Card6, Casp1, Casp2, Casp7, Cav3, Cck, Ccl11, Ccl17, Ccl19, Ccl2, Ccl5, Ccl6, Ccnd3, Ccny, Ccr1l1, Cd22, Cd28, Cd36, Cd38, Cd3d, Cd4, Cd5, Cd79a, Cdc42bpa, Cdc42bpb, Cdc42bpg, Cdh1, Cdh2, Cdk14, Cdk5r1, Cdk7, Cds2, Cflar, Chi3l1, Chn2, Cish, Cited1, Clca2, Clec2d, Cln3, Clpb, Cmya5, Col18a1, Col1a1, Col5a2, Coro1a, Cr2, Creb1, Crk, Cry1, Csf1r, Csf2rb, Csf3r, Ctf1, Ctsc, Ctsh, Cul4b, Cx3cl1, Cxadr, Cxcl13, Cxcl2, Cxcl5, Cxcr4, Cxxc4, Cyp1a2, Cyp7a1, Cysltr1, Dclk3, Dcstamp, Ddb2, Ddr1, Dek, Depdc1a, Depdc7, Derl2, Dgke, Dgki, Dhx58, Dixdc1, Dkk2, Dkk3, Dmap1, Dock10, Dock5, Dock6, Dok4, Dtx4, Duox2, Dusp3, Dut, Dvl3, Edn3, Efnb2, Efnb3, Egr1, Egr2, Ehd4, Ei24, Eif2a, Eif2b3, Eif2b4, Elmo2, Eme2, Emp2, Eno1, Entpd1, Epas1, Ercc3, Ercc8, Ern1, Eya3, F2, F7, Fam110c, Fam13b, Fbxo8, Fbxo9, Fcgr1, Fcgr2b, Fen1, Fgd5, Fgd6, Fgf10, Fgf16, Fgf20, Fgf21, Fgf6, Fgf7, Fgfbp1, Fgfr2, Fgfr4, Fhit, Fkbp8, Fmo2, Fosl1, Foxo6, Fpr2, Frzb, Fst, Fxn, Fyn, Fzd3, G2e3, Gab1, Gabbr2, Gabra1, Gabrr2, Garnl3, Gas6, Gata6, Gbp4, Gdf6, Gdi2, Gdnf, Gem, Ggct, Gli1, Gli2, Glis2, Gna12, Gnai1, Gnai2, Gnas, Gng7, Gpld1, Gpr12, Gpr150, Gpr152, Gpr156, Gpr157, Gpr171, Gpr183, Gpr4, Gpr55, Gpr61, Gpr65, Gpr84, Gps2, Gpx1, Grid2ip, Grik5, Grin2a, Grin3a, Grk4, Grk5, Guca1b, Gucy2g, H2-Ab1, H2afx, Has1, Hdac4, Hdac6, Hey2, Hfe2, Hint1, Hipk3, Hmga2, Hmgn3, Hnf1a, Hrh2, Hsp90b1, Hspa1b, Hspa5, Hspd1, Htr2a, Hyal2, Hyal3, Igf1, Igfbp4, Igfbp5, Igsf10, Il16, Il1a, Il1b, Il1r1, Il1rap, Il1rl2, Il20rb, Il2rb, Il5ra, Il7, Inhba, Ino80c, Inpp5f, Insig2, Insrr, Ints7, Intu, Invs, Iqgap2, Irak1, Irak3, Irf1, Irf4, Irs2, Itch, Itga1, Itga3, Itga8, Itgad, Itgae, Itgal, Itgam, Itgav, Itgax, Itgb1, Itgb1bp1, Itgb2, Itgb4, Itk, Itm2b, Itpr3, Itsn1, Itsn2, Ivns1abp, Jag2, Jmjd6, Jun, Kcne1, Kcne2, Kcnn4, Kctd8, Kdm6a, Kif22, Kif3a, Kif7, Klhl12, Kras, Krt10, Krt18, Lat2, Lck, Ldb1, Lefty1, Lgals1, Lmcd1, Lpar1, Lpar2, Lrba, Lrp5, Ltb4r1, Ltbp3, Ltk, Lyn, Lyst, Mad2l2, Magi3, Mal, Malt1, Map2, Map3k12, Mapk1, Mapkapk2, Marf1, Mark2, Med24, Men1, Mfng, Mgarp, Mgst1, Mkks, Mlh3, Mllt3, Mllt4, Mlxipl, Mme, Mmp3, Mpg, Mras, Mrc1, Msh3, Mst1r, Mt3, Mus81, Myc, Myh6, Mylk3, Myo1e, Myocd, Naip1, Nceh1, Ncf2, Ncoa3, Nek1, Nek8, Neurl1b, Nf1, Nfat5, Nfkbib, Nlgn2, Nlrx1, Nog, Npr3, Nr5a2, Nrg1, Nudt1, Nup62, Olfr1384, Olfr543, Olfr691, Olfr78, Omp, Onecut2, Opn1sw, Opn3, Os9, Oser1, Osm, Oxr1, P2rx4, P2rx7, Pafah1b2, Pak1, Pak3, Palm, Pdcd11, Pde1c, Pde3b, Pde4a, Pde6h, Pde7a, Pde8a, Pde8b, Pdgfb, Pdk2, Pdpn, Pecam1, Peli1, Pf4, Pfdn5, Pgap2, Phrf1, Pias2, Pik3c3, Pik3r3, Pip5k1a, Pla2g4a, Pla2g6, Pla2r1, Plagl2, Plau, Plcb3, Plch1, Plekhg3, Plekhg6, Plk3, Plxnb3, Pnp, Pold3, Polg2, Poli, Pon3, Postn, Pou1f1, Ppara, Pparg, Ppargc1b, Ppm1e, Ppp1r14c, Ppp2r1a, Ppp2r5a, Ppp2r5e, Prex1, Prkag2, Prkdc, Prokr2, Psd4, Psme3, Ptch1, Ptger1, Ptger3, Ptgir, Ptgs2, Pth1r, Ptk2b, Ptpra, Ptpre, Ptprj, Ptprk, Pycr1, Pyhin1, Rab12, Rab27a, Rab37, Rab3c, Rabl5, Rad23b, Rad9a, Raet1a, Ralb, Ralbp1, Rapgef4, Rasa2, Rasal1, Rasgef1c, Rasl10b, Rasl2-9, Rassf9, Rbbp5, Rbm15, Rbm38, Rcan1, Reck, Rem1, Rgn, Rgs16, Rgs19, Rgs3, Rgs9bp, Rhod, Rims2, Rin1, Ripk2, Rit1, Rnf31, Rock1, Rorc, Rp1, Rps6ka6, Rptor, Rras, Rrm2b, Rtn4, Rtn4rl2, Runx2, Ruvbl1, Ryr1, S100a10, S1pr5, Scai, Scarb1, Scn5a, Sel1l, Sell, Serinc3, Serpine1, Sfrp1, Sfrp2, Shank3, Shc1, Shc3, Sigirr, Siva1, Ski, Slc11a1, Slc16a1, Slc1a2, Slc2a4, Slc35c2, Slx1b, Smad5, Smad6, Smc1b, Smo, Smpd3, Socs1, Socs6, Sod2, Sort1, Sox8, Sparc, Spata13, Spata22, Spred2, Ssh1, Sstr2, Sstr4, Stat2, Stk38, Stmn3, Stra13, Strn3, Stub1, Sulf1, Syk, Syvn1, Tarbp2, Tbx1, Tbx3, Tenm2, Tert, Tgfb1, Tgfb2, Tgfb3, Tgfbr1, Tgfbr3, Tgfbrap1, Tgtp1, Themis, Thoc1, Tle2, Tlr1, Tlr3, Tlr7, Tmem204, Tnfaip3, Tnfrsf1a, Tnfsf15, Tnik, Tollip, Tom1l1, Tox3, Traf2, Trem2, Trim38, Trio, Trip13, Trp53, Trp53inp2, Trpv4, Tspan12, Tspyl5, Tti1, Ttn, Tub, Tulp4, Tyro3, Ucn, Ulk2, Usp3, Uvssa, Vapb, Vcp, Vmn1r45, Vmn2r57, Vwc2, Wdr45, Wfikkn2, Wif1, Wisp1, Wnt1, Wnt11, Wnt16, Wnt4, Wnt6, Wnt9a, Wnt9b, Wwtr1, Xiap, Yod1, Ywhaq, Yy1, Zbtb33, Zc3hc1, Zfp13, Zfp709, Zranb1 | 3986 | cellular response to stimulus |
| GO:0044763 | 2.971e-03 | 1129 | 1236 1810011O10Rik, 1810043G02Rik, 2210018M11Rik, 2610018G03Rik, 2700094K13Rik, 4930506M07Rik, 9430023L20Rik, 9630033F20Rik, Aamdc, Aatk, Abca2, Abcb7, Acsl6, Actn3, Actr8, Adam11, Adam12, Adam15, Adam19, Adam22, Adam5, Adamts3, Adcy4, Adcy6, Adcy8, Adora2a, Adra1d, AF251705, Agrp, Aifm3, Aimp1, Akap1, Akap2, Akt1s1, Alad, Alkbh2, Alkbh4, Alkbh5, Aloxe3, Amer1, Anapc15, Angptl6, Ankle2, Ankrd26, Ano1, Anpep, Anxa11, Anxa2, Anxa3, Anxa7, Ap1g1, Ap3d1, Ap4e1, Ap5z1, Apbb2, Apc2, Apex1, Aph1a, Aplnr, Apoc3, Aqp1, Aqp2, Arap3, Arhgap11a, Arhgap15, Arhgap18, Arhgap20, Arhgap21, Arhgap24, Arhgap31, Arhgap42, Arhgap6, Arhgef1, Arhgef10, Arhgef37, Arhgef5, Arid1a, Arid5b, Arl11, Arl13a, Arl5c, Arl6, Arl6ip1, Armcx3, Arntl, Asb10, Asb13, Asb5, Asic1, Aspa, Asxl1, Atf2, Atf3, Atg9a, Atox1, Atp1b2, Atp2b4, Atp6v0a1, Atp6v0a4, Atp6v0c, Atp6v0e2, Atxn2, Avil, Avpr1a, Avpr2, Axin1, Bag5, Bahd1, Baiap2, Basp1, Bbs5, Bcap31, Bcl2, Bcl2l2, Bcl9l, Bex1, Bfsp2, Birc2, Bloc1s2, Blzf1, Bmf, Bmp10, Bmp4, Bmpr1b, Bnip2, Bod1, Bop1, Brca1, Brd1, Brwd3, C1qtnf1, C1qtnf2, C5ar2, C77370, Cacna1b, Cacna1i, Cacna2d1, Cacnb1, Cacnb2, Cacng6, Cadm1, Cadm4, Calm1, Calm2, Calm3, Canx, Cap1, Caprin2, Capzb, Car8, Card6, Casc1, Casp1, Casp2, Casp4, Casp7, Catsper3, Cav3, Cbx4, Ccar1, Ccdc23, Ccdc39, Ccdc64, Cck, Ccl11, Ccl17, Ccl19, Ccl2, Ccl5, Ccl6, Ccnc, Ccnd3, Ccne2, Ccnt1, Ccny, Ccp110, Ccr1l1, Cct8, Cd22, Cd28, Cd2ap, Cd300a, Cd33, Cd36, Cd38, Cd3d, Cd4, Cd5, Cd5l, Cd63, Cd79a, Cd83, Cd99l2, Cda, Cdc25c, Cdc42bpa, Cdc42bpb, Cdc42bpg, Cdc42ep2, Cdc5l, Cdca3, Cdh1, Cdh10, Cdh2, Cdh23, Cdh8, Cdhr4, Cdk14, Cdk5r1, Cdk7, Cdkn2c, Cds2, Cenpc1, Cenpt, Cep76, Cetn3, Cflar, Chi3l1, Chl1, Chn2, Cish, Cited1, Clca2, Clcn4-2, Clec2d, Clec4e, Cln3, Clpb, Clstn2, Clstn3, Clvs1, Cmya5, Cnot10, Cntnap1, Cntrob, Col11a2, Col13a1, Col14a1, Col18a1, Col19a1, Col1a1, Col28a1, Col4a4, Col4a5, Col5a1, Col5a2, Col6a2, Col7a1, Coro1a, Coro1b, Coro1c, Cp, Cplx1, Cr2, Crbn, Creb1, Creb3l2, Creb5, Crebl2, Crip3, Cript, Crk, Cry1, Csf1r, Csf2rb, Csf3r, Cspg5, Csrp1, Cst10, Cstad, Ctf1, Ctla2a, Ctnna3, Ctnnd2, Ctsc, Ctsh, Ctsl, Cul1, Cul4b, Cx3cl1, Cxadr, Cxcl13, Cxcl2, Cxcl5, Cxcr4, Cxxc4, Cyp1a2, Cyp7a1, Cysltr1, D130043K22Rik, D230025D16Rik, Daam1, Dad1, Dap3, Dclk3, Dcstamp, Dctn6, Ddb2, Ddr1, Ddx11, Ddx39, Dek, Depdc1a, Depdc7, Derl2, Dffa, Dffb, Dgke, Dgki, Dhx58, Dixdc1, Dkk2, Dkk3, Dlgap1, Dlgap3, Dmap1, Dmtf1, Dnah12, Dnajc2, Dnajc5, Doc2b, Dock10, Dock5, Dock6, Dok4, Dpp4, Dscr3, Dsp, Dstn, Dtx4, Duox2, Dusp3, Dut, Dvl3, Dync1i2, Dync2h1, Dyrk3, Edil3, Edn3, Eed, Efcab4a, Efnb2, Efnb3, Egln2, Egr1, Egr2, Ehd1, Ehd2, Ehd4, Ei24, Eif2a, Eif2b3, Eif2b4, Eif5a, Eif5a2, Elmo2, Eme2, Emp2, Emp3, En2, Enah, Endog, Eno1, Entpd1, Epas1, Epb4.1l1, Ercc3, Ercc8, Ern1, Etv4, Etv6, Eva1a, Exoc3, Exoc3l, Exph5, Ext1, Eya3, Ezr, F2, F5, F7, Fa2h, Fam110c, Fam13b, Fam53a, Fam58b, Fap, Fblim1, Fbln1, Fbln2, Fbxo8, Fbxo9, Fcgr1, Fcgr2b, Fen1, Fgd5, Fgd6, Fgf10, Fgf16, Fgf20, Fgf21, Fgf6, Fgf7, Fgfbp1, Fgfr1op, Fgfr2, Fgfr4, Fgl2, Fhit, Fkbp8, Fmo2, Folr2, Fosl1, Foxo6, Fpr2, Frem1, Frem2, Frmd4b, Frzb, Fscn1, Fst, Fxn, Fyn, Fzd3, G2e3, Gab1, Gab3, Gabbr2, Gabra1, Gabrd, Gabrr2, Garnl3, Gas6, Gata6, Gatad2a, Gbp4, Gcc2, Gdf6, Gdi2, Gdnf, Gem, Gga3, Ggct, Ggn, Gjb5, Gjc2, Gli1, Gli2, Glis2, Gna12, Gnai1, Gnai2, Gnas, Gng7, Golga2, Gpld1, Gpr116, Gpr12, Gpr150, Gpr152, Gpr156, Gpr157, Gpr171, Gpr183, Gpr4, Gpr55, Gpr61, Gpr65, Gpr84, Gps2, Gpx1, Gramd4, Grid2ip, Grik5, Grin2a, Grin3a, Grk4, Grk5, Grn, Guca1b, Gucy2g, Gypa, Gzmm, H2-Ab1, H2afx, H2afy2, Has1, Haus6, Hdac10, Hdac4, Hdac6, Henmt1, Hexb, Hey2, Hfe2, Hint1, Hipk3, Hmga2, Hmgn3, Hnf1a, Hook3, Hoxd9, Hrh2, Hs6st1, Hsd17b4, Hsh2d, Hsp90b1, Hspa1b, Hspa5, Hspa8, Hspd1, Htatip2, Htr2a, Hyal2, Hyal3, Hydin, Ica1, Ift81, Igf1, Igfbp4, Igfbp5, Igfbp7, Igfbpl1, Igsf10, Igsf11, Igsf9, Il16, Il1a, Il1b, Il1r1, Il1rap, Il1rl2, Il20rb, Il25, Il2rb, Il5ra, Il7, Ing1, Inha, Inhba, Ino80c, Inpp5f, Insig2, Insrr, Ints7, Intu, Invs, Iqgap2, Irak1, Irak3, Irf1, Irf4, Irs2, Irx4, Irx5, Isg15, Itch, Itga1, Itga3, Itga8, Itgad, Itgae, Itgal, Itgam, Itgav, Itgax, Itgb1, Itgb1bp1, Itgb2, Itgb4, Itk, Itm2b, Itpr3, Itsn1, Itsn2, Ivns1abp, Jag2, Jdp2, Jmjd6, Jun, Kcnab1, Kcnab2, Kcnb1, Kcnc1, Kcnc4, Kcne1, Kcne2, Kcng2, Kcng4, Kcnip1, Kcnj16, Kcnn2, Kcnn4, Kcnu1, Kctd8, Kdm4d, Kdm6a, Kif17, Kif18a, Kif1a, Kif1b, Kif22, Kif23, Kif26b, Kif27, Kif3a, Kif3b, Kif7, Kirrel, Klhl12, Kpna1, Kpnb1, Kras, Krt10, Krt14, Krt18, Krt19, Lat2, Lck, Ldb1, Lefty1, Lgals1, Lgals8, Lmcd1, Lor, Loxl3, Lpar1, Lpar2, Lrba, Lrp2, Lrp5, Lsm11, Ltb4r1, Ltbp3, Ltk, Lyn, Lyst, Lyz2, Lztfl1, Mad2l2, Maf, Magi3, Mal, Malt1, Maoa, Maob, Map1a, Map2, Map3k12, Map4, Mapk1, Mapkapk2, Marf1, Mark2, Marveld1, Mbtps1, Med24, Men1, Mepce, Mfap5, Mfn1, Mfng, Mfsd9, Mgarp, Mgst1, Misp, Mkks, Mlh3, Mllt3, Mllt4, Mlxipl, Mme, Mmgt1, Mmp3, Mpdz, Mpg, Mpz, Mras, Mrc1, Msh3, Msi2, Msl3, Msn, Mst1r, Mt3, Mtfr2, Mus81, Myb, Myc, Mycbp2, Mycbpap, Myh6, Mylk3, Myo1e, Myo1f, Myo5c, Myocd, Myrip, Nab1, Naip1, Ncam2, Ncdn, Nceh1, Ncf1, Ncf2, Ncoa3, Ncor2, Ndufaf7, Ndufs1, Nefh, Nek1, Nek8, Nek9, Neu1, Neurl1b, Nf1, Nfat5, Nfkbib, Nid2, Nlgn2, Nlrp1a, Nlrx1, Nme6, Nnat, Nog, Noxo1, Npr3, Nqo1, Nr5a2, Nrg1, Nsd1, Nsf, Ntn3, Ntng1, Ntng2, Nubpl, Nudc, Nudt1, Nup107, Nup155, Nup54, Nup62, Nusap1, Nxf2, Nxt1, Olfr1384, Olfr543, Olfr691, Olfr78, Omp, Onecut2, Opn1sw, Opn3, Optn, Orai1, Os9, Oser1, Osgin1, Osm, Otog, Oxr1, P2rx4, P2rx7, Pacrg, Pafah1b2, Pak1, Pak3, Palm, Pcdh19, Pcdh20, Pcdhb13, Pcdhb15, Pcdhb16, Pcdhb17, Pcdhb18, Pcdhb19, Pcdhb2, Pcdhb8, Pcnt, Pcsk6, Pdcd11, Pdcd2, Pde1c, Pde3b, Pde4a, Pde6h, Pde7a, Pde8a, Pde8b, Pdgfb, Pdk2, Pdlim3, Pdlim5, Pdpn, Pdzrn3, Pecam1, Peli1, Pex7, Pf4, Pfdn5, Pfn4, Pgap2, Phf2, Phf8, Phrf1, Pi4k2a, Pias2, Pik3c3, Pik3r3, Pip5k1a, Piwil2, Pla2g16, Pla2g4a, Pla2g6, Pla2r1, Plagl1, Plagl2, Plau, Plaur, Plcb3, Plch1, Pld1, Plekha7, Plekha8, Plekhg3, Plekhg6, Plk3, Plk4, Plxnb3, Pnp, Pnpla8, Podxl2, Pold3, Polg2, Poli, Polrmt, Pon3, Postn, Pou1f1, Pou4f1, Ppara, Pparg, Ppargc1b, Ppfia3, Ppl, Ppm1e, Ppm1g, Ppp1cc, Ppp1r12a, Ppp1r13l, Ppp1r14c, Ppp2r1a, Ppp2r5a, Ppp2r5e, Prcc, Prex1, Prf1, Prkag2, Prkdc, Prkx, Prnd, Prokr2, Prom1, Psd4, Psme3, Pstpip1, Ptch1, Ptger1, Ptger3, Ptgir, Ptgs1, Ptgs2, Pth1r, Ptk2b, Ptp4a1, Ptpra, Ptpre, Ptprg, Ptprj, Ptprk, Ptprs, Pycr1, Pyhin1, Qars, Rab12, Rab27a, Rab37, Rab3c, Rabl5, Rad23b, Rad9a, Raet1a, Ralb, Ralbp1, Ranbp1, Rapgef4, Rapsn, Rasa2, Rasal1, Rasgef1a, Rasgef1c, Rasl10b, Rasl2-9, Rassf9, Rbbp5, Rbm14, Rbm15, Rbm38, Rcan1, Reck, Reep1, Rem1, Retn, Rffl, Rgn, Rgs16, Rgs19, Rgs3, Rgs9bp, Rhod, Rims2, Rin1, Ripk2, Rit1, Rnf31, Robo4, Rock1, Rom1, Ropn1l, Rorc, Rp1, Rprd1b, Rprm, Rps28, Rps6ka6, Rpsa, Rptor, Rras, Rrm2b, Rsph4a, Rtn4, Rtn4r, Rtn4rl2, Rtp4, Runx2, Ruvbl1, Ryr1, S100a1, S100a10, S1pr5, Samm50, Sap30bp, Sbf1, Scai, Scarb1, Scd1, Scn10a, Scn3a, Scn4b, Scn5a, Scrib, Sct, Sdad1, Sdf4, Sdk1, Sec22b, Sec23b, Sel1l, Sell, Sema3b, Senp1, Sep15, Sept9, Serinc3, Serinc5, Serpinb9, Serpine1, Sesn1, Sfrp1, Sfrp2, Sfxn1, Sfxn2, Shank3, Shc1, Shc3, Shroom4, Sidt2, Sigirr, Siva1, Ski, Slc11a1, Slc12a8, Slc16a1, Slc16a13, Slc16a2, Slc17a9, Slc1a1, Slc1a2, Slc1a3, Slc1a5, Slc22a15, Slc22a8, Slc24a4, Slc25a25, Slc25a36, Slc25a41, Slc2a4, Slc30a4, Slc35c2, Slc38a3, Slc38a4, Slc39a13, Slc39a2, Slc43a1, Slc6a2, Slc7a4, Slc7a6, Slc8a2, Slc9a8, Slco1a1, Slco4a1, Slitrk6, Slk, Slx1b, Smad5, Smad6, Smc1b, Smcr7, Smo, Smpd3, Sned1, Snx32, Snx33, Socs1, Socs6, Sod2, Sort1, Sox15, Sox8, Spaca3, Sparc, Spata13, Spata22, Spata25, Spc24, Spi1, Spice1, Spr, Spred2, Spta1, Srd5a1, Srp9, Srpr, Srpx2, Ssh1, Sstr2, Sstr4, Stab2, Stag1, Stag2, Stam, Stat2, Stau1, Stk10, Stk38, Stmn3, Stpg1, Stra13, Strip2, Strn3, Stub1, Stxbp1, Stxbp3a, Sulf1, Supv3l1, Suv39h2, Svep1, Sycn, Sycp2, Syk, Syne1, Syt3, Syvn1, Tacc1, Taf1, Taf3, Taf6, Tarbp2, Tbata, Tbx1, Tbx3, Tecta, Tekt2, Tenm2, Tert, Tet3, Tfam, Tfeb, Tgfb1, Tgfb2, Tgfb3, Tgfbi, Tgfbr1, Tgfbr3, Tgfbrap1, Tgtp1, Thap1, Thbs2, Thbs3, Themis, Thoc1, Thsd7a, Timm44, Timp1, Tk2, Tle2, Tlr1, Tlr3, Tlr7, Tmed10, Tmem123, Tmem165, Tmem204, Tmem219, Tmod3, Tmsb4x, Tmx3, Tnfaip3, Tnfrsf18, Tnfrsf1a, Tnfsf15, Tnik, Tnnt2, Tnrc6c, Tollip, Tom1l1, Tom1l2, Top1, Tox3, Tpcn1, Tpgs1, Tpm1, Tppp3, Traf2, Trem2, Trib2, Trim38, Trim39, Trio, Trip13, Tro, Troap, Trp53, Trp53inp2, Trpc4, Trpm3, Trpm7, Trpv4, Tsc22d1, Tspan12, Tspo, Tspyl5, Ttc30b, Tti1, Ttn, Tub, Tubb2a, Tubgcp5, Tulp4, Twf1, Txn2, Txndc12, Txndc9, Txnl1, Tyro3, Ubl4, Ucn, Ulk2, Uqcr11, Usp21, Usp3, Usp37, Usp4, Usp46, Usp6nl, Uvssa, Vapb, Vcp, Vill, Vipas39, Vmn1r45, Vmn2r57, Vps11, Vps18, Vps33a, Vps33b, Vps36, Vwc2, Wash, Wdr36, Wdr45, Wdr77, Wfikkn2, Wif1, Wisp1, Wnt1, Wnt11, Wnt16, Wnt4, Wnt6, Wnt9a, Wnt9b, Wrap53, Wwtr1, Xiap, Xirp2, Xpo6, Yars2, Ykt6, Yod1, Ywhaq, Yy1, Zbtb33, Zc3hc1, Zdhhc16, Zdhhc22, Zfp13, Zfp281, Zfp318, Zfp335, Zfp346, Zfp384, Zfp709, Zfx, Zhx3, Zranb1 | 7850 | single-organism cellular process |
| GO:0007165 | 5.960e-03 | 463.3 | 550 Aatk, Adam11, Adam19, Adam22, Adam5, Adamts3, Adcy4, Adcy6, Adcy8, Adora2a, Adra1d, Agrp, Akap2, Akt1s1, Aloxe3, Amer1, Ankrd26, Apc2, Aph1a, Aplnr, Apoc3, Arap3, Arhgap11a, Arhgap15, Arhgap18, Arhgap20, Arhgap21, Arhgap24, Arhgap31, Arhgap42, Arhgap6, Arhgef1, Arhgef10, Arhgef37, Arid1a, Arid5b, Arl11, Arl13a, Arl5c, Arl6, Arntl, Asb10, Asb13, Asb5, Asxl1, Avpr1a, Avpr2, Axin1, Baiap2, Bcap31, Bcl2, Bcl2l2, Bcl9l, Bex1, Bloc1s2, Bmf, Bmp10, Bmp4, Bmpr1b, Bnip2, Brca1, C1qtnf1, C1qtnf2, C5ar2, Caprin2, Capzb, Car8, Card6, Casp1, Casp2, Casp7, Cav3, Cck, Ccl11, Ccl17, Ccl19, Ccl2, Ccl5, Ccnd3, Ccny, Ccr1l1, Cd22, Cd28, Cd36, Cd38, Cd3d, Cd4, Cd5, Cd79a, Cdc42bpa, Cdc42bpb, Cdc42bpg, Cdh1, Cdh2, Cdk14, Cdk5r1, Cds2, Cflar, Chi3l1, Chn2, Cish, Cited1, Clca2, Clec2d, Cln3, Cmya5, Col18a1, Col1a1, Cr2, Creb1, Crk, Csf1r, Csf2rb, Ctf1, Ctsc, Ctsh, Cx3cl1, Cxcl13, Cxcl5, Cxcr4, Cxxc4, Cysltr1, Dclk3, Ddr1, Depdc1a, Depdc7, Derl2, Dgke, Dgki, Dhx58, Dixdc1, Dkk2, Dkk3, Dock10, Dock5, Dock6, Dok4, Dtx4, Duox2, Dusp3, Dut, Dvl3, Edn3, Efnb2, Efnb3, Egr1, Ei24, Eif2a, Emp2, Entpd1, Epas1, Ern1, F2, F7, Fam110c, Fam13b, Fbxo8, Fbxo9, Fcgr1, Fcgr2b, Fgd5, Fgd6, Fgf10, Fgf16, Fgf20, Fgf21, Fgf6, Fgf7, Fgfbp1, Fgfr2, Fgfr4, Fhit, Fkbp8, Foxo6, Fpr2, Frzb, Fst, Fxn, Fyn, Fzd3, G2e3, Gab1, Gabbr2, Gabra1, Gabrr2, Garnl3, Gas6, Gata6, Gdf6, Gdi2, Gdnf, Gem, Ggct, Gli1, Gli2, Glis2, Gna12, Gnai1, Gnai2, Gnas, Gng7, Gpld1, Gpr12, Gpr150, Gpr152, Gpr156, Gpr157, Gpr171, Gpr183, Gpr4, Gpr55, Gpr61, Gpr65, Gpr84, Gps2, Gpx1, Grid2ip, Grik5, Grin2a, Grin3a, Grk4, Grk5, Guca1b, Gucy2g, Hdac4, Hdac6, Hey2, Hfe2, Hint1, Hipk3, Hmga2, Hnf1a, Hrh2, Hspa5, Hspd1, Htr2a, Hyal2, Igf1, Igfbp4, Igfbp5, Igsf10, Il1a, Il1b, Il1r1, Il1rap, Il1rl2, Il20rb, Il2rb, Il7, Inhba, Inpp5f, Insig2, Insrr, Intu, Invs, Iqgap2, Irak1, Irak3, Irf1, Irf4, Irs2, Itch, Itga1, Itga3, Itga8, Itgad, Itgae, Itgal, Itgam, Itgav, Itgax, Itgb1, Itgb1bp1, Itgb2, Itgb4, Itk, Itm2b, Itpr3, Itsn1, Itsn2, Ivns1abp, Jag2, Jmjd6, Jun, Kcnn4, Kctd8, Kdm6a, Kif3a, Kif7, Klhl12, Kras, Krt18, Lat2, Lck, Ldb1, Lefty1, Lgals1, Lmcd1, Lpar1, Lpar2, Lrba, Lrp5, Ltb4r1, Ltbp3, Ltk, Lyn, Mad2l2, Magi3, Mal, Malt1, Map3k12, Mapk1, Mapkapk2, Mark2, Med24, Men1, Mfng, Mkks, Mllt3, Mllt4, Mlxipl, Mras, Mrc1, Mst1r, Mt3, Myc, Myh6, Myo1e, Myocd, Ncoa3, Nek8, Neurl1b, Nf1, Nfat5, Nfkbib, Nlgn2, Nlrx1, Nog, Npr3, Nr5a2, Nrg1, Nup62, Olfr1384, Olfr543, Olfr691, Olfr78, Omp, Onecut2, Opn1sw, Opn3, Osm, P2rx4, P2rx7, Pak1, Pak3, Palm, Pdcd11, Pde1c, Pde3b, Pde4a, Pde6h, Pde7a, Pde8a, Pde8b, Pdgfb, Pdk2, Pdpn, Pecam1, Peli1, Pf4, Pfdn5, Pgap2, Phrf1, Pias2, Pik3c3, Pik3r3, Pla2g6, Pla2r1, Plagl2, Plau, Plcb3, Plch1, Plekhg3, Plekhg6, Plk3, Plxnb3, Pnp, Postn, Pou1f1, Ppara, Pparg, Ppargc1b, Ppp1r14c, Ppp2r1a, Ppp2r5a, Ppp2r5e, Prex1, Prkag2, Prkdc, Prokr2, Psd4, Psme3, Ptch1, Ptger1, Ptger3, Ptgir, Ptgs2, Pth1r, Ptk2b, Ptpra, Ptpre, Ptprj, Ptprk, Rab12, Rab27a, Rab37, Rab3c, Rabl5, Rad9a, Ralb, Ralbp1, Rapgef4, Rasa2, Rasal1, Rasgef1c, Rasl10b, Rasl2-9, Rassf9, Rbm15, Rbm38, Rcan1, Reck, Rem1, Rgn, Rgs16, Rgs19, Rgs3, Rgs9bp, Rhod, Rims2, Rin1, Ripk2, Rit1, Rnf31, Rock1, Rorc, Rp1, Rps6ka6, Rptor, Rras, Rrm2b, Runx2, S1pr5, Scai, Scarb1, Sel1l, Serinc3, Serpine1, Sfrp1, Sfrp2, Shank3, Shc1, Shc3, Sigirr, Siva1, Ski, Slc11a1, Slc35c2, Smad5, Smad6, Smo, Smpd3, Socs1, Socs6, Sod2, Sort1, Sox8, Sparc, Spata13, Spred2, Sstr2, Sstr4, Stat2, Stk38, Stmn3, Strn3, Stub1, Sulf1, Syk, Syvn1, Tbx1, Tenm2, Tert, Tgfb1, Tgfb2, Tgfb3, Tgfbr1, Tgfbr3, Tgfbrap1, Themis, Thoc1, Tle2, Tlr1, Tlr3, Tlr7, Tmem204, Tnfaip3, Tnfrsf1a, Tnfsf15, Tnik, Tollip, Tom1l1, Tox3, Traf2, Trem2, Trim38, Trio, Trp53, Trpv4, Tspan12, Tspyl5, Tti1, Ttn, Tub, Tulp4, Tyro3, Ucn, Ulk2, Vapb, Vmn1r45, Vmn2r57, Vwc2, Wfikkn2, Wif1, Wisp1, Wnt1, Wnt11, Wnt16, Wnt4, Wnt6, Wnt9a, Wnt9b, Wwtr1, Xiap, Yod1, Ywhaq, Zbtb33, Zc3hc1, Zfp13, Zranb1 | 3221 | signal transduction |
| GO:0007166 | 2.242e-02 | 261.4 | 327 Adam11, Adam19, Adam22, Adam5, Adamts3, Adcy4, Adcy6, Adora2a, Adra1d, Agrp, Akap2, Akt1s1, Amer1, Ankrd26, Apc2, Aph1a, Aplnr, Apoc3, Arhgef1, Arid5b, Arl6, Avpr1a, Avpr2, Axin1, Bcl2, Bcl2l2, Bcl9l, Bex1, Bloc1s2, Bmp10, Bmp4, Bmpr1b, C5ar2, Caprin2, Casp2, Cav3, Cck, Ccl2, Ccl5, Ccny, Ccr1l1, Cd22, Cd36, Cd38, Cd3d, Cd4, Cd79a, Cdh1, Cdh2, Cdk14, Cdk5r1, Cflar, Cish, Cited1, Clca2, Cln3, Col18a1, Col1a1, Cr2, Creb1, Crk, Csf1r, Csf2rb, Ctf1, Cx3cl1, Cxcl13, Cxcr4, Cxxc4, Cysltr1, Ddr1, Dgke, Dixdc1, Dkk2, Dkk3, Dok4, Dtx4, Duox2, Dusp3, Dvl3, Edn3, Efnb2, Efnb3, Egr1, Emp2, Entpd1, F2, F7, Fcgr1, Fcgr2b, Fgf10, Fgf16, Fgf20, Fgf21, Fgf6, Fgf7, Fgfbp1, Fgfr2, Fgfr4, Fkbp8, Foxo6, Fpr2, Frzb, Fst, Fyn, Fzd3, Gab1, Gabbr2, Gabra1, Gabrr2, Gas6, Gata6, Gdf6, Gdnf, Gli1, Gli2, Glis2, Gna12, Gnai1, Gnai2, Gnas, Gng7, Gpld1, Gpr12, Gpr150, Gpr152, Gpr156, Gpr157, Gpr171, Gpr183, Gpr4, Gpr55, Gpr61, Gpr65, Gpr84, Gpx1, Grid2ip, Grik5, Grin2a, Grin3a, Grk4, Grk5, Hey2, Hfe2, Hnf1a, Hrh2, Hspa5, Htr2a, Hyal2, Igf1, Igfbp4, Igfbp5, Igsf10, Il1a, Il1b, Il1r1, Il1rap, Il1rl2, Il2rb, Il7, Inhba, Insrr, Intu, Invs, Irak1, Irak3, Irf1, Irs2, Itga1, Itga3, Itga8, Itgad, Itgae, Itgal, Itgam, Itgav, Itgax, Itgb1, Itgb1bp1, Itgb2, Itgb4, Itk, Itm2b, Itpr3, Jag2, Jmjd6, Jun, Kcnn4, Kctd8, Kdm6a, Kif3a, Kif7, Klhl12, Kras, Krt18, Lat2, Lck, Ldb1, Lefty1, Lpar1, Lpar2, Lrp5, Ltb4r1, Ltbp3, Ltk, Lyn, Mad2l2, Mal, Malt1, Mapk1, Mapkapk2, Mark2, Men1, Mfng, Mkks, Mllt3, Mst1r, Mt3, Myc, Myh6, Myo1e, Myocd, Neurl1b, Nf1, Nlgn2, Nog, Npr3, Nup62, Olfr1384, Olfr543, Olfr691, Olfr78, Onecut2, Opn1sw, Opn3, P2rx4, P2rx7, Palm, Pde6h, Pdgfb, Pdk2, Peli1, Pf4, Pfdn5, Pik3r3, Plcb3, Postn, Pou1f1, Pparg, Ppp2r1a, Prokr2, Psme3, Ptch1, Ptger1, Ptger3, Ptgir, Pth1r, Ptk2b, Ptpra, Ptpre, Ptprj, Ptprk, Rbm15, Reck, Rgs16, Rgs19, Rgs3, Ripk2, Rnf31, Runx2, S1pr5, Scarb1, Sel1l, Serpine1, Sfrp1, Sfrp2, Shank3, Shc1, Sigirr, Siva1, Ski, Slc35c2, Smad5, Smad6, Smo, Socs1, Sort1, Sstr2, Sstr4, Stub1, Sulf1, Syk, Tert, Tgfb1, Tgfb2, Tgfb3, Tgfbr1, Tgfbr3, Themis, Tle2, Tmem204, Tnfaip3, Tnfrsf1a, Tnik, Tom1l1, Traf2, Trp53, Tspan12, Tub, Ucn, Vmn1r45, Vmn2r57, Vwc2, Wfikkn2, Wif1, Wisp1, Wnt1, Wnt11, Wnt16, Wnt4, Wnt6, Wnt9a, Wnt9b, Wwtr1, Xiap, Zbtb33, Zc3hc1, Zranb1 | 1817 | cell surface receptor signaling pathway |
| GO:0003008 | 3.394e-02 | 169.5 | 223 Acta2, Actn3, Adam22, Adcy8, Adora2a, Adra1d, Aff2, Akap1, Alox5, Aloxe3, Anpep, Ap3d1, Aqp1, Aqp2, Arhgef10, Asic1, Atp6v0a4, Atxn2, Avpr1a, Avpr2, Axin1, Baiap2, Bcl2, Bfsp2, Bmp10, Bmp4, Cacna1b, Cacna1i, Cacnb2, Canx, Casp1, Cav3, Cck, Ccl2, Cd38, Cdh1, Cdh2, Cdh23, Cdh8, Chl1, Cln3, Cmya5, Cnnm4, Cntnap1, Col11a2, Col14a1, Col1a1, Cplx1, Cspg5, Ctnnd2, Cx3cl1, Cxcr4, Cysltr1, Dlgap1, Doc2b, Dsp, Edn3, Egr1, Egr2, Ei24, Eif2b4, Epas1, Ephx2, Epm2a, F5, Fa2h, Fen1, Fgf10, Fgfr2, Fxn, Fyn, Gabra1, Gabrd, Gabrr2, Gas6, Gata6, Gdnf, Gm11744, Gnai1, Gnai2, Gnas, Gpx1, Grid2ip, Grik5, Grin2a, Grin3a, Grn, Guca1b, Hdac4, Hexb, Hey2, Hnf1a, Hrh2, Htr2a, Ica1, Igf1, Il1a, Il1b, Inha, Inhba, Inpp5f, Irx5, Itga1, Itga3, Itga8, Itgb1bp1, Itpr3, Jag2, Jun, Kcnab1, Kcnc4, Kcne1, Kcne2, Kcnn2, Kcnn4, Kdm6a, Kif1b, Kirrel, Kng1, Kras, Lck, Lmcd1, Lrp5, Mal, Maoa, Maob, Map1a, Mapk1, Mkks, Mme, Mpdz, Myc, Myh6, Mylk3, Myo1e, Myocd, Nab1, Ncdn, Ncf1, Ncf2, Nf1, Nlgn2, Nob1, Npr3, Nrg1, Nyx, Olfr1384, Olfr543, Olfr691, Olfr78, Omp, Opn1sw, Otog, P2rx4, P2rx7, Pcdhb16, Pde1c, Pde4a, Pde6h, Pdgfb, Pdlim5, Pla2g6, Plcb3, Pon1, Pou4f1, Ppara, Pparg, Ppfia3, Ppp1r13l, Ptger3, Ptgs1, Ptgs2, Ptprj, Rapgef4, Rapsn, Rasl10b, Retn, Rgs9bp, Rims2, Rom1, Rp1, Rrm2b, Rtn4, Rtp4, Ryr1, Scn10a, Scn4b, Scn5a, Scrib, Sct, Serinc5, Shank3, Shc1, Shc3, Shroom4, Ski, Slc1a3, Slitrk6, Smad5, Smad6, Sod2, Sp4, Sstr2, Stxbp1, Sulf1, Tbx1, Tbx3, Tecta, Tgfb1, Tgfb2, Tgfbr3, Thbs2, Tnnt2, Tpgs1, Tpm1, Tprn, Trpm7, Ttn, Tub, Ucn, Unc119, Usp46, Xpnpep3 | 1178 | system process |

## Help | Hide | Top Help | Show | Top GO CC test for over-representation

### HELP

List of all enriched GO categories (cellular components), at the 0.05
p-value level.

The columns:

- **ExpCount** is the expected count of genes in the
  module annotated with the given GO term, just by chance.
- **Count**
  is the number of genes in the module annotated with the given GO
  term.
- **Size** is the total number of genes (in our universe)
  annotated with the GO term.

Clicking on **Count** shows the genes that drive the
enrichment. You can also click on the individual numbers in
the **Count** column, to show the driving genes for that individual
GO category.

Clicking on the GO identifiers takes you to the Gene Ontology web
pages.

— Click on the *Help* button again to close this help window.

| Id | Pvalue | ExpCount | Count | Size | Term |
| --- | --- | --- | --- | --- | --- |
| GO:0005576 | 3.222e-05 | 193 | 262 1110058L19Rik, Acpp, Adam22, Adamts12, Adamts3, Adpgk, Agrp, Aimp1, Alad, Ambp, Angptl2, Angptl6, Anxa2, Aoah, Apoc3, Apoc4, Aqp1, Art5, Bglap3, Bmp10, Bmp4, C1qtnf1, C1qtnf2, C1s, C4bp, Casp1, Ccdc126, Ccdc134, Cck, Ccl11, Ccl17, Ccl2, Ccl22, Ccl5, Ccl6, Ccl8, Ccl9, Cd5l, Cfh, Chgb, Chi3l1, Chl1, Clec11a, Clec3b, Cma1, Col10a1, Col11a2, Col13a1, Col14a1, Col18a1, Col19a1, Col1a1, Col20a1, Col28a1, Col4a4, Col4a5, Col5a1, Col5a2, Col6a2, Col7a1, Col9a3, Cp, Cpa1, Cst10, Ctf1, Ctla2a, Ctsh, Ctsk, Cx3cl1, Cxadr, Cxcl13, Cxcl2, Cxcl5, Cyb5d2, Dcn, Dhrs11, Dkk2, Dkk3, Dpp4, Edil3, Edn3, Efemp2, Egfbp2, Eno1, Entpd1, Ephx3, F2, F5, F7, Fam150b, Fap, Fbln1, Fbln2, Fcgr2b, Fgf10, Fgf16, Fgf20, Fgf21, Fgf6, Fgf7, Fgfbp1, Fgfr2, Fgl1, Fgl2, Fndc7, Frem1, Frem2, Frzb, Fst, Fstl5, Gas6, Gcnt1, Gdf6, Gdnf, Glb1l2, Gnas, Gpld1, Grn, Hfe2, Hgfac, Hhipl2, Hspa8, Hspd1, Hyal3, Igf1, Igfbp4, Igfbp5, Igfbp7, Igfbpl1, Igsf10, Il16, Il17re, Il1a, Il1b, Il1f9, Il1r1, Il1rap, Il7, Inha, Inhba, Isg15, Itgb1, Itgb4, Itih3, Itm2b, Izumo4, Kif23, Klk13, Kng1, Lad1, Lefty1, Lgals1, Lmcd1, Loxl1, Loxl3, Lrp2, Ltbp2, Ltbp3, Ly6g5b, Lyz2, Mamdc2, Mfap5, Mmp13, Mmp17, Mmp24, Mmp3, Msn, Muc19, Muc5b, Muc6, Nid2, Nog, Nptxr, Ntn3, Nyx, Ogn, Osm, Otog, Otogl, Pcnt, Pcsk2, Pcsk6, Pdgfb, Pecam1, Pf4, Pi4k2a, Pigr, Pla2g2d, Pla2r1, Plau, Plaur, Pnp, Pon1, Pon2, Pon3, Postn, Prf1, Prom1, Prrg2, Ptch1, Ptgds, Ptprg, Retn, Rnase10, Rpsa, Saa2, Sbsn, Sbspon, Sct, Sdf2, Sema3b, Serpina11, Serpina1e, Serpina3f, Serpina3k, Serpina3n, Serpina6, Serpinb6a, Serpinb9, Serpinc1, Serpine1, Sfrp1, Sfrp2, Slc1a3, Slc2a4, Slmap, Sned1, Spaca3, Sparc, Srpx2, Sulf1, Svep1, Tbc1d15, Tecta, Tfpi, Tgfb1, Tgfb2, Tgfb3, Tgfbi, Tgfbr3, Thbd, Thbs2, Thbs3, Thsd7a, Timp1, Tnfrsf18, Tnfrsf1a, Tnfsf15, Trem2, Tub, Ucn, Vwc2, Wfikkn2, Wif1, Wisp1, Wnt1, Wnt11, Wnt16, Wnt4, Wnt6, Wnt9a, Wnt9b | 1339 | extracellular region |
| GO:0044421 | 7.657e-05 | 123.8 | 179 Adamts12, Agrp, Aimp1, Alad, Ambp, Anxa2, Apoc3, Apoc4, Aqp1, Bmp10, Bmp4, C1qtnf1, C1qtnf2, Cck, Ccl11, Ccl17, Ccl2, Ccl22, Ccl5, Ccl6, Ccl8, Ccl9, Cfh, Chi3l1, Chl1, Clec3b, Col10a1, Col11a2, Col13a1, Col14a1, Col18a1, Col19a1, Col1a1, Col20a1, Col28a1, Col4a4, Col4a5, Col5a1, Col5a2, Col6a2, Col7a1, Col9a3, Cp, Cpa1, Ctf1, Ctsh, Ctsk, Cx3cl1, Cxadr, Cxcl13, Cxcl2, Cxcl5, Dcn, Dkk2, Dkk3, Dpp4, Edil3, Edn3, Efemp2, Egfbp2, Eno1, Entpd1, F2, F5, F7, Fap, Fbln1, Fbln2, Fgf10, Fgf7, Frem1, Frem2, Frzb, Gas6, Gcnt1, Gdf6, Gdnf, Gpld1, Grn, Hfe2, Hgfac, Hspa8, Hspd1, Igf1, Igfbp4, Igfbp5, Igfbp7, Il16, Il1a, Il1b, Il1f9, Il1r1, Il7, Inha, Inhba, Itgb1, Itgb4, Itm2b, Kif23, Klk13, Lad1, Lefty1, Lgals1, Lmcd1, Loxl1, Lrp2, Ltbp2, Ltbp3, Mamdc2, Mfap5, Mmp13, Mmp17, Mmp24, Mmp3, Msn, Nid2, Nog, Nptxr, Ntn3, Nyx, Ogn, Osm, Otog, Pcnt, Pcsk2, Pcsk6, Pdgfb, Pecam1, Pf4, Pi4k2a, Plau, Pon1, Pon3, Postn, Prf1, Prom1, Ptgds, Ptprg, Retn, Rpsa, Saa2, Sbspon, Serpina11, Serpina1e, Serpina3f, Serpina3k, Serpina3n, Serpina6, Serpinb6a, Serpinb9, Serpinc1, Serpine1, Sfrp1, Sfrp2, Slc1a3, Slc2a4, Slmap, Sparc, Sulf1, Tecta, Tfpi, Tgfb1, Tgfb2, Tgfb3, Tgfbi, Tgfbr3, Thbd, Thbs2, Timp1, Tnfrsf1a, Tnfsf15, Vwc2, Wnt1, Wnt11, Wnt16, Wnt4, Wnt6, Wnt9a, Wnt9b | 859 | extracellular region part |
| GO:0005615 | 2.748e-03 | 88.21 | 129 Agrp, Aimp1, Alad, Ambp, Apoc3, Apoc4, Bmp10, Bmp4, C1qtnf1, C1qtnf2, Cck, Ccl11, Ccl17, Ccl2, Ccl22, Ccl5, Ccl6, Ccl8, Ccl9, Cfh, Chi3l1, Clec3b, Col14a1, Col18a1, Col1a1, Col6a2, Cp, Cpa1, Ctf1, Ctsh, Ctsk, Cx3cl1, Cxadr, Cxcl13, Cxcl2, Cxcl5, Dcn, Dkk2, Dkk3, Edn3, Egfbp2, F2, F5, F7, Fap, Fbln1, Fgf10, Fgf7, Frzb, Gas6, Gcnt1, Gdf6, Gdnf, Gpld1, Grn, Hfe2, Hgfac, Hspd1, Igf1, Igfbp4, Igfbp5, Igfbp7, Il16, Il1a, Il1b, Il1f9, Il1r1, Il7, Inha, Inhba, Itm2b, Klk13, Lefty1, Lgals1, Lmcd1, Lrp2, Ltbp2, Mmp13, Mmp3, Nog, Nptxr, Ogn, Osm, Pcsk2, Pcsk6, Pdgfb, Pecam1, Pf4, Plau, Pon1, Pon3, Prf1, Prom1, Ptgds, Ptprg, Retn, Saa2, Serpina11, Serpina1e, Serpina3f, Serpina3k, Serpina3n, Serpina6, Serpinb6a, Serpinb9, Serpinc1, Serpine1, Sfrp1, Sfrp2, Slmap, Sparc, Sulf1, Tfpi, Tgfb1, Tgfb2, Tgfb3, Tgfbi, Tgfbr3, Thbd, Tnfrsf1a, Tnfsf15, Vwc2, Wnt1, Wnt11, Wnt16, Wnt4, Wnt6, Wnt9a, Wnt9b | 612 | extracellular space |
| GO:0031012 | 3.444e-03 | 50.59 | 82 Adamts12, Anxa2, Bmp4, C1qtnf1, C1qtnf2, Cdh8, Chl1, Clec3b, Cma1, Col10a1, Col11a2, Col13a1, Col14a1, Col18a1, Col19a1, Col1a1, Col20a1, Col28a1, Col4a4, Col4a5, Col5a1, Col5a2, Col6a2, Col7a1, Col9a3, Dcn, Efemp2, Entpd1, Fbln1, Fbln2, Fgf10, Fgfr2, Frem1, Frem2, Gpld1, Igf1, Igfbp7, Itgb1, Itgb4, Lad1, Lgals1, Lmcd1, Loxl1, Ltbp2, Ltbp3, Mamdc2, Mfap5, Mmp13, Mmp15, Mmp16, Mmp17, Mmp24, Mmp3, Nid2, Ntn3, Nyx, Ogn, Otog, Pcsk6, Postn, Rpsa, Sbspon, Serpine1, Sfrp1, Slc1a3, Sparc, Tecta, Tgfb1, Tgfb2, Tgfb3, Tgfbi, Tgfbr3, Thbs2, Timp1, Vwc2, Wnt1, Wnt11, Wnt16, Wnt4, Wnt6, Wnt9a, Wnt9b | 351 | extracellular matrix |
| GO:0097458 | 6.181e-03 | 121.8 | 167 4930506M07Rik, Aatk, Acp1, Adcy4, Adora2a, Agrp, Alox5, Anxa3, Ap3d1, Aqp1, Arid1a, Asic1, Asl, Atp2b4, Baiap2, Begain, Cacna1b, Cadm1, Calm1, Calm2, Calm3, Canx, Casp1, Casp4, Cck, Ccl2, Cdh1, Cdh23, Cdh8, Cdk5r1, Chl1, Cln3, Cntnap1, Cplx1, Cript, Ctla2a, Ctnnd2, Ctsl, Cxadr, Cxcr4, D230025D16Rik, Dixdc1, Dlgap1, Dlgap3, Dpf1, Eif5a, Eno1, Fmr1, Fosl1, Fzd3, Gabbr2, Gabrr2, Gnas, Golga2, Grid2ip, Grik4, Grik5, Grin2a, Grin3a, Grip2, Grk4, Guca1b, Hnf1a, Hspa8, Htr2a, Ica1, Igsf9, Inha, Itga1, Itga8, Itpr3, Itsn1, Kcnab2, Kcnb1, Kcnc1, Kcnc4, Kcnip1, Kcnn2, Kcnn3, Kcnn4, Khsrp, Kif1a, Kif1b, Kif3a, Kif5a, Kirrel, Kpna1, Lpar1, Map2, Map3k12, Mapk1, Mme, Mmp3, Mpdz, Myc, Mycbp2, Myo1d, Myrip, Naip1, Ncam2, Ncdn, Ncf1, Nefh, Nf1, Nqo1, Nrg1, Nsf, Omp, Opn1sw, Orai1, P2rx4, P2rx7, Pak1, Palm, Pcsk2, Pde1c, Pdlim5, Pi4k2a, Plk3, Plxdc1, Pou4f1, Ppp1cc, Prex1, Prom1, Ptch1, Ptgs1, Ptgs2, Ptk2b, Ptprk, Rab27a, Ralbp1, Rapgef4, Rims2, Rom1, Rp1, Rpsa, Rptor, Rtn4, S100a1, Scn10a, Scrib, Shank2, Shank3, Slc1a2, Slc1a3, Slc6a2, Slc8a2, Smo, Sort1, Srd5a1, Stau1, Stmn3, Strn3, Strn4, Syne1, Tenm2, Tgfb1, Tgfb2, Tgfb3, Tmem57, Tnfrsf1a, Top1, Tpm3, Trpm7, Trpv4, Txn2, Ucn | 845 | neuron part |
| GO:0043005 | 7.335e-03 | 104.2 | 146 4930506M07Rik, Aatk, Acp1, Adcy4, Adora2a, Alox5, Anxa3, Ap3d1, Aqp1, Asic1, Asl, Atp2b4, Baiap2, Begain, Cacna1b, Cadm1, Calm1, Calm2, Calm3, Canx, Casp1, Casp4, Cck, Cdh1, Cdh8, Cdk5r1, Chl1, Cln3, Cntnap1, Cplx1, Cript, Ctla2a, Ctnnd2, Ctsl, Cxadr, Cxcr4, D230025D16Rik, Dixdc1, Dlgap1, Dlgap3, Eif5a, Eno1, Fmr1, Fosl1, Fzd3, Gabbr2, Gabrr2, Gnas, Grid2ip, Grik4, Grik5, Grin2a, Grin3a, Grip2, Grk4, Hspa8, Htr2a, Ica1, Igsf9, Itga1, Itga8, Itpr3, Itsn1, Kcnab2, Kcnb1, Kcnc1, Kcnc4, Kcnip1, Kcnn2, Kcnn3, Khsrp, Kif1a, Kif1b, Kif3a, Kif5a, Kirrel, Kpna1, Lpar1, Map2, Map3k12, Mapk1, Mme, Mmp3, Mpdz, Myc, Mycbp2, Myo1d, Naip1, Ncam2, Ncdn, Ncf1, Nefh, Nf1, Nrg1, Nsf, Omp, Opn1sw, Orai1, P2rx4, P2rx7, Pak1, Palm, Pcsk2, Pdlim5, Pi4k2a, Plk3, Plxdc1, Pou4f1, Ppp1cc, Prex1, Ptch1, Ptgs2, Ptk2b, Ptprk, Rab27a, Ralbp1, Rapgef4, Rims2, Rptor, Rtn4, S100a1, Scn10a, Shank2, Shank3, Slc1a2, Slc1a3, Slc6a2, Slc8a2, Smo, Sort1, Srd5a1, Stau1, Stmn3, Strn3, Strn4, Syne1, Tenm2, Tgfb1, Tgfb2, Tmem57, Tnfrsf1a, Tpm3, Trpm7, Trpv4, Txn2, Ucn | 723 | neuron projection |
| GO:0005578 | 7.625e-03 | 42.23 | 70 Adamts12, Anxa2, Bmp4, C1qtnf1, C1qtnf2, Chl1, Clec3b, Col10a1, Col11a2, Col13a1, Col14a1, Col18a1, Col19a1, Col1a1, Col20a1, Col28a1, Col4a4, Col4a5, Col5a1, Col5a2, Col6a2, Col7a1, Col9a3, Dcn, Efemp2, Entpd1, Fbln1, Fbln2, Frem1, Frem2, Gpld1, Igf1, Itgb1, Itgb4, Lad1, Lgals1, Loxl1, Ltbp3, Mamdc2, Mfap5, Mmp13, Mmp17, Mmp24, Mmp3, Nid2, Ntn3, Nyx, Ogn, Otog, Postn, Rpsa, Sbspon, Sfrp1, Slc1a3, Sparc, Tecta, Tgfb1, Tgfb2, Tgfbi, Tgfbr3, Thbs2, Timp1, Vwc2, Wnt1, Wnt11, Wnt16, Wnt4, Wnt6, Wnt9a, Wnt9b | 293 | proteinaceous extracellular matrix |
| GO:0071944 | 1.021e-02 | 449.5 | 525 1810043G02Rik, Acp1, Acpp, Adam12, Adcy4, Adcy6, Adora2a, Adra1d, AF251705, Akr1a1, Alox5, Ambp, Amer1, Ano1, Ano3, Anpep, Anxa2, Anxa3, Anxa7, Apc2, Aph1a, Aplnr, Aqp1, Aqp2, Arap3, Arhgap24, Arhgef5, Arl6, Art3, Asic1, Atp1b2, Atp2b4, Atp6v0a1, Atp6v0a4, Atp8a1, AU023871, Avpr1a, Avpr2, Axin1, Baiap2, Basp1, Bbs5, Bcap31, Bcas3, Bfsp2, Birc2, Brca1, C5ar2, Cacna1b, Cacna2d1, Cacnb1, Cacnb2, Cadm1, Calm1, Calm2, Calm3, Cap1, Capzb, Catsper3, Cav3, Ccl19, Ccny, Ccr1l1, Cd22, Cd28, Cd2ap, Cd300a, Cd300lb, Cd320, Cd33, Cd36, Cd38, Cd3d, Cd4, Cd5, Cd52, Cd55, Cd63, Cd79a, Cd83, Cd8b1, Cd99l2, Cdc42bpb, Cdc42ep2, Cdh1, Cdh10, Cdh2, Cdh23, Cdh8, Cdk14, Cdk5r1, Cfh, Cflar, Chl1, Cish, Clca2, Cldn22, Clec12a, Clec12b, Clec2d, Cln3, Clstn2, Clstn3, Cnnm1, Cnnm4, Cntnap1, Col10a1, Col13a1, Col6a2, Coro1a, Cp, Cr2, Crk, Csf1r, Cspg5, Ctsl, Cubn, Cul4b, Cx3cl1, Cxadr, Cxcr4, Cybrd1, Cysltr1, D130043K22Rik, Daam1, Dcstamp, Ddr1, Dixdc1, Dlgap1, Dlgap3, Dnajc5, Dnm2, Doc2b, Dpp3, Dpp4, Dsp, Dstn, Dusp3, Dvl3, Dync2h1, Efnb2, Ehd1, Ehd2, Ehd4, Emp2, Eno1, Eps15l1, Eva1a, Eva1c, Exoc3, Exoc3l, Ezr, Fam110c, Fap, Fcgr1, Fcgr2b, Fgd5, Fgf10, Fgf6, Fgfbp1, Fgfr2, Fgfr4, Fhit, Folr2, Fpr2, Frem2, Frrs1l, Fyn, Fzd3, Gabbr2, Gabra1, Gabrd, Gabrr2, Gca, Gem, Ggt5, Gjb5, Gjc2, Gna12, Gnai1, Gnai2, Gnas, Gng7, Gpr116, Gpr12, Gpr150, Gpr152, Gpr156, Gpr157, Gpr171, Gpr183, Gpr4, Gpr55, Gpr61, Gpr65, Gpr84, Grid2ip, Grik4, Grik5, Grin2a, Grin3a, Grip2, Grk5, Guca1b, Gucy2g, Gypa, Gys2, H2-Ab1, Hdac6, Hfe2, Hint1, Hmox2, Hsp90b1, Hspa1b, Hspd1, Htr2a, Hyal2, Ifngr2, Igsf10, Igsf11, Igsf9, Il16, Il17re, Il1r1, Il1rap, Il2rb, Il3ra, Inha, Irs2, Itch, Itga1, Itga3, Itga8, Itgad, Itgae, Itgal, Itgam, Itgav, Itgax, Itgb1, Itgb1bp1, Itgb2, Itgb4, Itm2b, Itpr3, Itsn1, Jag2, Jmjd6, Kcnab1, Kcnb1, Kcnc1, Kcnc4, Kcne1, Kcne2, Kcng4, Kcnip1, Kcnj16, Kcnn2, Kcnn3, Kcnn4, Kcnu1, Kctd8, Kif18a, Kir3dl1, Kirrel, Klhl7, Klrc1, Kras, Krt14, Krt19, Lat2, Lck, Lor, Lpar1, Lrba, Lrp2, Lrp5, Lrrc52, Lrrn3, Ltb4r1, Ltk, Luc7l3, Lyn, Magi3, Mal, Mal2, Map3k12, Map4, Mapk1, Mark2, Marveld1, Mfsd9, Mllt4, Mme, Mmgt1, Mmp16, Mmp17, Mmp24, Mpdz, Mpz, Mras, Mrc1, Msn, Mst1r, Myo1d, Myo1f, Myo1g, Myrip, Naip1, Ncam2, Ncf1, Ncl, Neu1, Nf1, Nkain1, Nlgn2, Nlrp5, Nmt1, Noxo1, Nptxr, Nrg1, Nsf, Ntng1, Ntng2, Olfr543, Olfr691, Olfr78, Orai1, Osm, Otog, P2rx4, P2rx7, Pacsin3, Pafah1b2, Pak1, Palm, Pcdh19, Pcdh20, Pcdhb13, Pcdhb15, Pcdhb16, Pcdhb17, Pcdhb18, Pcdhb19, Pcdhb2, Pcdhb8, Pdgfb, Pdlim5, Pdpn, Pdzd3, Pecam1, Phf7, Phka1, Phldb2, Pi4k2a, Pigr, Pip5k1a, Pla2r1, Plaur, Plcb3, Plp2, Plxdc1, Plxnb3, Pon2, Ppl, Prex1, Prf1, Prkar2a, Prnd, Prokr2, Prom1, Psd4, Pstpip1, Ptch1, Ptger1, Ptger3, Ptgir, Ptgs2, Pth1r, Ptk2b, Ptp4a1, Ptp4a3, Ptpn7, Ptpra, Ptprcap, Ptpre, Ptprj, Ptprk, Ptprr, Rab27a, Rab3c, Raet1a, Ralb, Rapgef4, Rapgef6, Rapsn, Rasa2, Rasal1, Rasl10b, Rccd1, Reck, Rgs16, Rgs19, Rgs3, Rhod, Rit1, Rnf31, Robo4, Rock1, Rom1, Rpsa, Rras, Rtn4, Rtn4r, Rtn4rl2, Ryr1, S100a10, S1pr5, Sacm1l, Scarb1, Scn10a, Scn4b, Scn5a, Scrib, Sdf4, Sell, Sephs1, Serinc3, Sfrp1, Sfrp2, Sgce, Shank2, Shank3, Shc1, Shroom4, Slc10a1, Slc11a1, Slc15a2, Slc16a1, Slc16a13, Slc16a2, Slc1a1, Slc1a2, Slc1a3, Slc1a5, Slc22a8, Slc24a4, Slc27a4, Slc2a4, Slc38a3, Slc38a4, Slc39a2, Slc6a2, Slc7a6, Slc8a2, Slco1a1, Slco1c1, Slco4a1, Slitrk6, Slk, Slmap, Smo, Smpd3, Sntb1, Sntg2, Socs6, Sort1, Spata13, Spred2, Spta1, Ssh1, Sspn, Sstr2, Sstr4, Stab2, Stat2, Stk10, Stk38, Strn3, Stxbp1, Stxbp3a, Sulf1, Syk, Syt2, Tecta, Tenm2, Tgfb3, Tgfbr1, Tgfbr3, Thbd, Thsd7a, Timd2, Tlr1, Tlr7, Tmed10, Tmem123, Tmem204, Tmem219, Tnfrsf18, Tnfrsf1a, Tnfrsf25, Tnfsf15, Tpm3, Traf2, Trem2, Tro, Trpc4, Trpm7, Trpv4, Tspan12, Tub, Tubb2a, Twf1, Tyro3, Vmn1r45, Vmn2r57, Vwc2, Wbp4, Xpo6, Ykt6, Yy1, Zbtb33, Zc4h2, Zfp709, Zhx3 | 3119 | cell periphery |
| GO:0009986 | 1.215e-02 | 74.95 | 110 Aimp1, Ambp, Anpep, Asic1, Bmp10, Cav3, Ccl19, Cd22, Cd28, Cd33, Cd36, Cd38, Cd4, Cd5, Cd55, Cd63, Cd79a, Cd83, Cd8b1, Cdh1, Ciita, Clec12a, Clec2d, Cr2, Csf1r, Ctsl, Cx3cl1, Cxcr4, Dcstamp, Dpp4, Fcgr1, Fcgr2b, Fgf10, Fgfbp1, Fgfr2, Gdi2, Gpr116, Grin2a, Grn, Gucy2g, Gypa, H2-Ab1, Hfe2, Hspa5, Hspa8, Hspd1, Hyal2, Il1a, Il1r1, Il2rb, Itga1, Itga3, Itgae, Itgal, Itgam, Itgav, Itgax, Itgb1, Itgb2, Itgb4, Kcne1, Kcne2, Klrc1, Lgals1, Lpar1, Lpar2, Mrc1, Msn, Neu1, Nid2, Nlgn2, P2rx7, Pcsk6, Pdgfb, Pdpn, Pecam1, Pla2r1, Plau, Plaur, Prom1, Ptprj, Ptprk, Ptprr, Robo4, Rtn4r, Rtn4rl2, Scarb1, Scn5a, Sell, Sfrp1, Slc1a3, Slc2a4, Slc6a2, Sort1, Sulf1, Tgfb1, Tgfb2, Tgfb3, Tgfbr3, Thbd, Tlr3, Tmem123, Tmx3, Tnfrsf18, Tnfrsf1a, Trpc4, Trpv4, Wnt1, Wnt4, Wnt6 | 520 | cell surface |
| GO:0005886 | 3.992e-02 | 437 | 506 1810043G02Rik, Acp1, Acpp, Adam12, Adcy4, Adcy6, Adora2a, Adra1d, AF251705, Akr1a1, Alox5, Ambp, Amer1, Ano1, Ano3, Anpep, Anxa2, Anxa3, Anxa7, Apc2, Aph1a, Aplnr, Aqp1, Aqp2, Arap3, Arhgap24, Arhgef5, Arl6, Art3, Asic1, Atp1b2, Atp2b4, Atp6v0a1, Atp6v0a4, Atp8a1, AU023871, Avpr1a, Avpr2, Axin1, Baiap2, Basp1, Bbs5, Bcap31, Bfsp2, Birc2, Brca1, C5ar2, Cacna1b, Cacna2d1, Cacnb1, Cacnb2, Cadm1, Calm1, Calm2, Calm3, Cap1, Catsper3, Cav3, Ccl19, Ccny, Ccr1l1, Cd22, Cd28, Cd2ap, Cd300a, Cd300lb, Cd320, Cd33, Cd36, Cd38, Cd3d, Cd4, Cd5, Cd52, Cd55, Cd63, Cd79a, Cd83, Cd8b1, Cd99l2, Cdc42bpb, Cdc42ep2, Cdh1, Cdh10, Cdh2, Cdh23, Cdh8, Cdk14, Cdk5r1, Cfh, Cflar, Chl1, Cish, Clca2, Cldn22, Clec12a, Clec12b, Clec2d, Cln3, Clstn2, Clstn3, Cnnm1, Cnnm4, Cntnap1, Col13a1, Col6a2, Coro1a, Cp, Cr2, Crk, Csf1r, Cspg5, Ctsl, Cubn, Cul4b, Cx3cl1, Cxadr, Cxcr4, Cybrd1, Cysltr1, D130043K22Rik, Daam1, Dcstamp, Ddr1, Dlgap1, Dlgap3, Dnajc5, Dnm2, Doc2b, Dpp3, Dpp4, Dsp, Dusp3, Dync2h1, Efnb2, Ehd1, Ehd2, Ehd4, Emp2, Eno1, Eps15l1, Eva1a, Eva1c, Ezr, Fap, Fcgr1, Fcgr2b, Fgd5, Fgf10, Fgf6, Fgfbp1, Fgfr2, Fgfr4, Fhit, Folr2, Fpr2, Frem2, Frrs1l, Fyn, Fzd3, Gabbr2, Gabra1, Gabrd, Gabrr2, Gca, Gem, Ggt5, Gjb5, Gjc2, Gna12, Gnai1, Gnai2, Gnas, Gng7, Gpr116, Gpr12, Gpr150, Gpr152, Gpr156, Gpr157, Gpr171, Gpr183, Gpr4, Gpr55, Gpr61, Gpr65, Gpr84, Grid2ip, Grik4, Grik5, Grin2a, Grin3a, Grip2, Grk5, Guca1b, Gucy2g, Gypa, H2-Ab1, Hdac6, Hfe2, Hint1, Hmox2, Hsp90b1, Hspa1b, Hspd1, Htr2a, Hyal2, Ifngr2, Igsf10, Igsf11, Igsf9, Il16, Il17re, Il1r1, Il1rap, Il2rb, Il3ra, Inha, Irs2, Itch, Itga1, Itga3, Itga8, Itgad, Itgae, Itgal, Itgam, Itgav, Itgax, Itgb1, Itgb1bp1, Itgb2, Itgb4, Itm2b, Itpr3, Itsn1, Jag2, Jmjd6, Kcnab1, Kcnb1, Kcnc1, Kcnc4, Kcne1, Kcne2, Kcng4, Kcnip1, Kcnj16, Kcnn2, Kcnn3, Kcnn4, Kcnu1, Kctd8, Kif18a, Kir3dl1, Kirrel, Klhl7, Klrc1, Kras, Krt19, Lat2, Lck, Lor, Lpar1, Lrba, Lrp2, Lrp5, Lrrc52, Lrrn3, Ltb4r1, Ltk, Luc7l3, Lyn, Magi3, Mal, Mal2, Map3k12, Map4, Mapk1, Mark2, Marveld1, Mfsd9, Mllt4, Mme, Mmgt1, Mmp16, Mmp17, Mmp24, Mpdz, Mpz, Mras, Mrc1, Msn, Mst1r, Myo1d, Myo1g, Myrip, Naip1, Ncam2, Ncf1, Neu1, Nf1, Nkain1, Nlgn2, Nmt1, Noxo1, Nptxr, Nrg1, Nsf, Ntng1, Ntng2, Olfr543, Olfr691, Olfr78, Orai1, Osm, Otog, P2rx4, P2rx7, Pacsin3, Pafah1b2, Pak1, Palm, Pcdh19, Pcdh20, Pcdhb13, Pcdhb15, Pcdhb16, Pcdhb17, Pcdhb18, Pcdhb19, Pcdhb2, Pcdhb8, Pdgfb, Pdlim5, Pdpn, Pdzd3, Pecam1, Phf7, Phka1, Phldb2, Pi4k2a, Pigr, Pip5k1a, Pla2r1, Plaur, Plcb3, Plp2, Plxdc1, Plxnb3, Pon2, Ppl, Prex1, Prf1, Prkar2a, Prnd, Prokr2, Prom1, Psd4, Ptch1, Ptger1, Ptger3, Ptgir, Ptgs2, Pth1r, Ptk2b, Ptp4a1, Ptp4a3, Ptpn7, Ptpra, Ptprcap, Ptpre, Ptprj, Ptprk, Ptprr, Rab27a, Rab3c, Raet1a, Ralb, Rapgef4, Rapgef6, Rapsn, Rasa2, Rasal1, Rasl10b, Rccd1, Reck, Rgs16, Rgs19, Rgs3, Rhod, Rit1, Rnf31, Robo4, Rock1, Rom1, Rpsa, Rras, Rtn4, Rtn4r, Rtn4rl2, Ryr1, S100a10, S1pr5, Sacm1l, Scarb1, Scn10a, Scn4b, Scn5a, Scrib, Sdf4, Sell, Sephs1, Serinc3, Sfrp1, Sfrp2, Sgce, Shank2, Shank3, Shc1, Shroom4, Slc10a1, Slc11a1, Slc15a2, Slc16a1, Slc16a13, Slc16a2, Slc1a1, Slc1a2, Slc1a3, Slc1a5, Slc22a8, Slc24a4, Slc27a4, Slc2a4, Slc38a3, Slc38a4, Slc39a2, Slc6a2, Slc7a6, Slc8a2, Slco1a1, Slco1c1, Slco4a1, Slk, Slmap, Smo, Smpd3, Sntb1, Sntg2, Socs6, Sort1, Spata13, Spred2, Ssh1, Sspn, Sstr2, Sstr4, Stab2, Stat2, Stk10, Stk38, Strn3, Stxbp1, Stxbp3a, Sulf1, Syk, Syt2, Tecta, Tenm2, Tgfb3, Tgfbr1, Tgfbr3, Thbd, Thsd7a, Timd2, Tlr1, Tlr7, Tmed10, Tmem123, Tmem204, Tmem219, Tnfrsf18, Tnfrsf1a, Tnfrsf25, Tnfsf15, Traf2, Trem2, Tro, Trpc4, Trpm7, Trpv4, Tspan12, Tub, Tubb2a, Twf1, Tyro3, Vmn1r45, Vmn2r57, Vwc2, Wbp4, Xpo6, Ykt6, Yy1, Zbtb33, Zc4h2, Zfp709 | 3032 | plasma membrane |
| GO:0008305 | 4.087e-02 | 4.036 | 13 Itga1, Itga3, Itga8, Itgad, Itgae, Itgal, Itgam, Itgav, Itgax, Itgb1, Itgb2, Itgb4, Lyn | 28 | integrin complex |
| GO:0016023 | 4.097e-02 | 74.51 | 107 Abca2, Acpp, Angptl6, Anxa11, Anxa3, Anxa7, Ap1g1, Ap4e1, Aqp1, Aqp2, Axin1, Bmf, Cadm1, Capzb, Catsper3, Cd2ap, Chgb, Cln3, Clvs1, Coro1a, Cspg5, Ctsh, Ctsl, Cubn, Cxadr, Cxcr4, Cxxc4, D230025D16Rik, Dmxl2, Dnajc5, Dnm2, Doc2b, Dpp4, Ehd1, Exoc3l, F5, Galnt15, Grin2a, Hexb, Hip1r, Hspa8, Hspd1, Hyal2, Ica1, Il1b, Itga1, Itgb1, Itm2b, Itsn1, Kif1b, Kif3a, Klhl12, Klk13, Lck, Lpar1, Lpar2, Lrba, Lrp2, Lyz2, Mme, Msn, Mt3, Myo1e, Myo5c, Myrip, Ncf2, Optn, Pcsk2, Pf4, Pi4k2a, Pigr, Pla2g4a, Prf1, Rab27a, Rab37, Rab3c, Rassf9, Rffl, Rgs19, Sec23b, Sec31b, Slc11a1, Slc2a4, Slc30a4, Slc39a2, Snx33, Sort1, Spaca3, Sspn, Stxbp1, Stxbp3a, Sycn, Syk, Syt2, Syt3, Tgfb1, Tgfb2, Tgfb3, Tlr1, Tlr7, Tmed10, Trpm7, Tyr, Uqcc, Vps11, Vps33b, Vps53 | 517 | cytoplasmic membrane-bounded vesicle |

## Help | Hide | Top Help | Show | Top GO MF test for over-representation

### HELP

List of all enriched GO categories (molecular function), at the 0.05
p-value level.

The columns:

- **ExpCount** is the expected count of genes in the
  module annotated with the given GO term, just by chance.
- **Count**
  is the number of genes in the module annotated with the given GO
  term.
- **Size** is the total number of genes (in our universe)
  annotated with the GO term.

Clicking on **Count** shows the genes that drive the
enrichment. You can also click on the individual numbers in
the **Count** column, to show the driving genes for that individual
GO category.

Clicking on the GO identifiers takes you to the Gene Ontology web
pages.

— Click on the *Help* button again to close this help window.

| Id | Pvalue | ExpCount | Count | Size | Term |
| --- | --- | --- | --- | --- | --- |
| GO:0002020 | 6.335e-04 | 9.924 | 27 Bcl2, Cd28, Cflar, Chl1, Dpp4, Dvl3, Fap, Hspa1b, Hspd1, Il1r1, Itgb1, Lonp2, Malt1, Os9, Ryr1, Sell, Serpina1e, Serpinb6a, Serpinb9, Serpinc1, Serpine1, Timp1, Tnfaip3, Tnfrsf1a, Trp53, Ttn, Xiap | 69 | protease binding |
| GO:0005515 | 2.894e-03 | 836.7 | 935 2610018G03Rik, 4930506M07Rik, 9430023L20Rik, Aatk, Acp1, Acpp, Acsl6, Actn3, Adam11, Adam12, Adam15, Adam19, Adam22, Adcy4, Adcy6, Adcy8, Adora2a, AF251705, Aga, Agbl5, Agrp, Aimp1, Akap1, Akap2, Alad, Aldh1a7, Alkbh4, Alox5, Ambp, Amer1, Ankle2, Ankrd26, Anxa11, Anxa2, Anxa7, Ap1g1, Apbb2, Apc2, Apex1, Aph1a, Apoc3, Aqp1, Aqp2, Arap3, Arhgap31, Arhgap6, Arhgef1, Arhgef10, Arhgef5, Arid1a, Arl6, Arl6ip1, Armcx3, Arntl, Asic1, Asxl1, Atf2, Atf3, Atic, Atox1, Atp2b4, Atp6v0a1, Atp6v0a4, Atp6v0c, Atp8a1, Atp8b2, Atxn2, Avil, Avpr1a, Axin1, Bag5, Baiap2, Basp1, Bcas1, Bcl2, Bcl2l2, Bcl9l, Bex1, Bfsp2, Bhlhe40, Birc2, Bloc1s2, Blzf1, Bmf, Bmp10, Bmp4, Bmpr1b, Bop1, Brca1, Brd1, Bzrap1, C1qtnf2, C1s, C4bp, Cacna1b, Cacna2d1, Cacnb1, Cacnb2, Cadm1, Calm1, Calm2, Calm3, Canx, Cap1, Capn7, Caprin2, Capzb, Car8, Casc1, Casd1, Casp1, Casp2, Casp4, Casp7, Catsper3, Cav3, Cbx3, Cbx4, Ccbl2, Ccdc64, Cck, Ccl11, Ccl17, Ccl19, Ccl2, Ccl22, Ccl27a, Ccl5, Ccl6, Ccl8, Ccl9, Ccnc, Ccnd3, Ccne2, Ccnt1, Ccny, Ccp110, Cct8, Cd22, Cd28, Cd2ap, Cd300a, Cd320, Cd36, Cd3d, Cd4, Cd5, Cd63, Cd79a, Cd8b1, Cda, Cdc25c, Cdc42bpa, Cdc42bpb, Cdc42ep2, Cdc5l, Cdh1, Cdh2, Cdh23, Cdk14, Cdk5r1, Cdk7, Cdkn2c, Cenpc1, Cenpt, Cetn3, Cfh, Cflar, Chgb, Chl1, Ciita, Cish, Cited1, Clec11a, Clec12a, Clec2d, Clec3b, Clec4e, Cln3, Cmya5, Cntnap1, Cntrob, Col18a1, Col1a1, Col5a1, Col5a2, Coro1a, Coro1b, Coro1c, Cotl1, Cp, Cplx1, Cr2, Creb1, Crebl2, Crebzf, Cript, Crk, Crkl, Crot, Cry1, Csf1r, Csf2rb, Csrp1, Ctf1, Ctnna3, Ctsc, Ctse, Ctsh, Ctsl, Cubn, Cul1, Cul4b, Cx3cl1, Cxadr, Cxcl13, Cxcl2, Cxcl5, Cxcr4, Cxxc4, Cyp1a2, D230025D16Rik, Daam1, Dcn, Dctn6, Ddo, Ddr1, Dek, Dennd5a, Dffb, Dgki, Dip2b, Dixdc1, Dlg5, Dlgap1, Dlgap3, Dmap1, Dmrt2, Dmrta1, Dmxl2, Dnaja2, Dnajb9, Dnajc2, Dnajc5, Dnm2, Doc2b, Dock10, Dok4, Dpp4, Dsp, Dstn, Dut, Dvl3, Dync1i2, Dyrk3, Ebf1, Edn3, Eed, Efcab4a, Efnb2, Efnb3, Egr1, Egr2, Ehd1, Ehd2, Ehd4, Eif2b4, Eif5a, Ell, Elmo2, Emp2, Enah, Eno1, Epas1, Epb4.1l1, Ephx2, Epm2a, Eps15l1, Ercc3, Ercc8, Etv6, Exoc3l, Exph5, Ext1, Eya3, Ezr, F2, F7, Fam110c, Fam58b, Fap, Fblim1, Fbln1, Fbln2, Fbxl20, Fcgr1, Fcgr2b, Fgf10, Fgf16, Fgf20, Fgf21, Fgf6, Fgf7, Fgfbp1, Fgfr1op, Fgfr2, Fgfr4, Fhit, Fkbp10, Fmr1, Foxj3, Foxo6, Foxr1, Fpr2, Frem2, Frmd3, Frmd4b, Frzb, Fscn1, Fst, Fubp1, Fxn, Fxyd7, Fyn, Fzd3, Gab1, Gab3, Gabbr2, Gabpb2, Gabra1, Gabrr2, Gas6, Gata6, Gatad2a, Gbp4, Gca, Gcc2, Gdf6, Gdi2, Gdnf, Gemin6, Gga3, Ggct, Ggn, Gins4, Gli1, Gli2, Glis2, Gmeb1, Gna12, Gnai1, Gnai2, Gnas, Golga2, Gps2, Gpx1, Grid2ip, Grik4, Grik5, Grin2a, Grin3a, Grip2, Grn, Grpel1, Gstcd, Gtf2f1, Gypa, Gzmm, H2-Ab1, H2afx, H2afy2, Hdac10, Hdac4, Hdac6, Hexb, Hey2, Hfe2, Hip1r, Hipk3, Hist2h4, Hmga2, Hnf1a, Hook3, Hpgds, Hsd17b4, Hsh2d, Hsp90b1, Hspa1b, Hspa5, Hspa8, Hspb6, Hspd1, Htr2a, Hyal2, Ica1, Ica1l, Igf1, Igfbp4, Igfbp5, Igfbp7, Igfbpl1, Il16, Il1a, Il1b, Il1f9, Il1r1, Il1rap, Il20rb, Il25, Il2rb, Il7, Ing1, Inha, Inhba, Inpp5a, Insig2, Insrr, Invs, Iqgap2, Irak1, Irak3, Irf1, Irf4, Irf9, Irs2, Isg15, Itch, Itga1, Itga3, Itgad, Itgal, Itgam, Itgav, Itgb1, Itgb1bp1, Itgb2, Itk, Itm2b, Itpr3, Itsn1, Itsn2, Ivns1abp, Jag2, Jdp2, Jmjd6, Jun, Kcnab1, Kcnab2, Kcnb1, Kcne1, Kcne2, Kcnip1, Kcnn2, Kcnn3, Kcnn4, Kdm6a, Khdrbs3, Kif17, Kif18a, Kif1a, Kif1b, Kif22, Kif23, Kif26b, Kif27, Kif3a, Kif3b, Kif5a, Kif7, Kirrel, Klhl7, Kng1, Kpna1, Kpnb1, Kras, Krt10, Krt14, Krt18, Krt19, Kynu, Lat2, Lck, Ldb1, Lefty1, Lgals1, Lgals4, Limk2, Lipt2, Lmcd1, Lonp2, Lor, Loxl1, Lpar1, Lpar2, Lrba, Lrp2, Lrp5, Lrrn3, Lsm11, Ltbp2, Ltbp3, Lyn, Lztfl1, Mad2l2, Maf, Magi3, Malt1, Maob, Map1a, Map2, Map3k12, Map4, Mapk1, Mapkapk2, Mat1a, Med24, Men1, Metap2, Mfn1, Mfng, Mga, Mgarp, Mgst1, Mical2, Misp, Mlf1ip, Mlh3, Mllt3, Mllt4, Mlxipl, Mmp13, Mmp3, Mpdz, Mpz, Mras, Mrfap1, Msh3, Msl3, Msn, Mst1r, Mtmr7, Mtmr9, Mvb12a, Mvd, Myb, Myc, Mycbp2, Myh6, Myo1d, Myo1e, Myo1f, Myo1g, Myocd, Myrip, Naa50, Nab1, Narf, Ncdn, Ncf1, Ncf2, Ncl, Ncoa3, Ncor2, Ndufaf7, Nebl, Necab3, Nedd8, Nek8, Nek9, Neu1, Nf1, Nfkbib, Nhlrc1, Nid2, Nlgn2, Nlrp1a, Nlrp5, Nlrx1, Nog, Noxo1, Npr3, Nptxr, Nrg1, Nsd1, Nsf, Ntn3, Nubp2, Nudc, Nup210, Nup62, Nusap1, Nxf2, Nxph4, Nxt1, Ogn, Optn, Orai1, Os9, Osgin1, Osm, P2rx4, P2rx7, Pacsin3, Pafah1b2, Pah, Paics, Pak1, Pak3, Palm, Pcbp1, Pcnt, Pcp4, Pcsk2, Pcsk6, Pdcd11, Pde1c, Pde3b, Pde4dip, Pdgfb, Pdk2, Pdlim3, Pdlim5, Pdzd3, Pdzrn3, Pecam1, Peli1, Pex7, Pf4, Pfdn5, Pfn4, Pgap2, Phf10, Phf2, Phf8, Phka1, Phkg1, Phrf1, Pi4k2a, Pias2, Pigo, Pigr, Pik3r3, Pip5k1a, Pip5k1b, Piwil2, Pla2g6, Pla2r1, Plagl1, Plaur, Plcb3, Plekha7, Plk3, Plk4, Plp2, Plxdc1, Plxnb3, Polg2, Pon1, Pon2, Pon3, Pou1f1, Pou4f1, Ppara, Pparg, Ppargc1b, Ppic, Ppl, Ppp1cc, Ppp1r12a, Ppp1r13l, Ppp1r18, Ppp2r1a, Ppp2r5a, Ppp6r2, Prim1, Prkag2, Prkar2a, Prkcdbp, Prkdc, Prkx, Prom1, Prps1, Prrg2, Psme3, Pstpip1, Ptch1, Ptger3, Ptgs2, Pth1r, Ptk2b, Ptpn18, Ptpra, Ptpre, Ptprg, Ptprj, Ptprk, Ptprr, Pts, Pycr1, Qars, Rab27a, Rab37, Rab3c, Rab3il1, Rad23b, Rad9a, Raet1a, Ralbp1, Rapgef4, Rapgef6, Rapsn, Rasl10b, Rassf9, Rbbp5, Rbm15, Rbm39, Rbmxl1, Reep1, Rem1, Retn, Rgs19, Rims2, Ripk2, Rit1, Rnf31, Robo4, Rock1, Rorc, Rp1, Rprd1b, Rpsa, Rptor, Rtn4, Rtn4r, Rufy1, Runx2, Ryr1, S100a1, Saa2, Samm50, Scai, Scarb1, Scn4b, Scn5a, Scrib, Sct, Sdf4, Sec22b, Sell, Senp1, Sep15, Sephs1, Sepsecs, Serpina1e, Serpinb6a, Serpinb9, Serpinc1, Serpine1, Sfrp1, Sfrp2, Sh3bgrl2, Shank2, Shank3, Shc1, Shc3, Shroom4, Siva1, Ski, Slc11a1, Slc16a1, Slc1a3, Slc22a8, Slc2a4, Slc39a13, Slc6a2, Slk, Slmap, Smad5, Smad6, Smc1b, Smo, Sntb1, Sntb2, Sntg2, Socs1, Socs6, Sod2, Sort1, Sox15, Sox8, Sp2, Spaca3, Spi1, Spop, Spr, Spred2, Spta1, Srpx2, Srrm2, Ssh1, Sstr2, Stam, Stat2, Stau1, Stk10, Stk38, Stmn3, Strn3, Strn4, Stub1, Stxbp1, Stxbp3a, Sun3, Supv3l1, Sycp2, Syk, Syne1, Syt2, Syt3, Tacc1, Taf1, Taf3, Taf6, Tarbp2, Tbata, Tbx1, Tbx3, Tceal1, Tecta, Tenm2, Tert, Tfam, Tfap2e, Tfeb, Tgfb1, Tgfb2, Tgfb3, Tgfbr1, Tgfbr3, Tgfbrap1, Thbs2, Themis, Timm44, Timp1, Tlr1, Tlr3, Tlr7, Tmed10, Tmem204, Tmod3, Tmpo, Tmsb4x, Tnfaip3, Tnfrsf18, Tnfrsf1a, Tnfsf15, Tnik, Tnnt2, Tollip, Tom1l1, Top1, Tox3, Tpcn1, Tpgs1, Tpm1, Tpm3, Tppp3, Tprn, Traf2, Trem2, Trib2, Trim38, Trim39, Trim50, Trim8, Trip13, Trip4, Trp53, Trpc4, Trpm7, Trpv4, Ttn, Tub, Tubgcp5, Twf1, Txn2, Tyr, Tyro3, Ube2d1, Ucn, Ulk2, Uqcrfs1, Usp3, Usp37, Usp4, Uvssa, Vapb, Vbp1, Vcp, Vill, Vps11, Vps18, Vps33a, Vps33b, Vps36, Wash, Wbp4, Wfikkn2, Whamm, Wif1, Wisp1, Wnt1, Wnt11, Wnt16, Wnt4, Wnt6, Wnt9a, Wnt9b, Wwtr1, Xiap, Xirp2, Xpo6, Yars2, Ykt6, Ywhaq, Zbtb33, Zc3hc1, Zfp281, Zfp335, Zfp346, Zfp384, Zfp408, Zhx3, Zranb1, Zxdc | 5818 | protein binding |
| GO:0008009 | 3.981e-02 | 4.315 | 14 Ccl11, Ccl17, Ccl19, Ccl2, Ccl22, Ccl5, Ccl6, Ccl8, Ccl9, Cx3cl1, Cxcl13, Cxcl2, Cxcl5, Pf4 | 30 | chemokine activity |

## Help | Hide | Top Help | Show | Top KEGG Pathway test for over-representation

### HELP

List of all enriched KEGG pathways, at the 0.05
p-value level.

The columns:

- **ExpCount** is the expected count of genes in the
  module annotated with the given KEGG pathway, just by chance.
- **Count**
  is the number of genes in the module annotated with the given KEGG
  pathway.
- **Size** is the total number of genes (in our universe)
  annotated with the KEGG pathway.

Clicking on **Count** shows the genes that drive the
enrichment. You can also click on the individual numbers in
the **Count** column, to show the driving genes for that individual
KEGG pathway.

Clicking on the KEGG identifiers takes you to the KEGG web site.

— Click on the *Help* button again to close this help window.

| Id | Pvalue | ExpCount | Count | Size | Term |
| --- | --- | --- | --- | --- | --- |
| 04640 | 1.941e-02 | 11.66 | 25 Anpep, Cd22, Cd33, Cd36, Cd38, Cd3d, Cd4, Cd5, Cd55, Cd8b1, Cr2, Csf1r, Csf2ra, Csf3r, Fcgr1, Il1a, Il1b, Il1r1, Il3ra, Il5ra, Il7, Itga1, Itga3, Itgam, Mme | 74 | Hematopoietic cell lineage |
| 04810 | 2.430e-02 | 31.68 | 52 Actn3, Apc2, Arhgef1, Baiap2, Crk, Crkl, Enah, Ezr, F2, Fgf10, Fgf16, Fgf20, Fgf21, Fgf6, Fgf7, Fgfr2, Fgfr4, Gna12, Insrr, Iqgap2, Itga1, Itga3, Itga8, Itgad, Itgae, Itgal, Itgam, Itgav, Itgax, Itgb1, Itgb2, Itgb4, Kras, Limk2, Mapk1, Mras, Msn, Mylk3, Pak1, Pak3, Pak6, Pdgfb, Pfn4, Pik3r3, Pip5k1a, Pip5k1b, Ppp1cc, Ppp1r12a, Rock1, Rras, Ssh1, Tmsb4x | 201 | Regulation of actin cytoskeleton |
| 04380 | 4.857e-02 | 17.97 | 33 Creb1, Csf1r, Ctsk, Fcgr1, Fcgr2b, Fosl1, Fyn, Ifngr2, Il1a, Il1b, Il1r1, Irf9, Jun, Lck, Lilra6, Mapk1, Ncf1, Ncf2, Pik3r3, Pira1, Pira4, Pira6, Pparg, Socs1, Spi1, Stat2, Syk, Tgfb1, Tgfb2, Tgfbr1, Tnfrsf1a, Traf2, Trem2 | 114 | Osteoclast differentiation |


### HELP

List of all enriched miRNA families, at the 0.05
p-value level.

The columns:

- **ExpCount** is the expected count of genes in the
  module regulated by the given miRNA family, just by chance.
- **Count**
  is the number of genes in the module regulated by the given miRNA
  family.
- **Size** is the total number of genes (in our universe)
  regulated with the given miRNA family.

Clicking on **Count** shows the genes that drive the
enrichment. You can also click on the individual numbers in
the **Count** column, to show the driving genes for that individual
miRNA family.

The miRNA regulation data was taken from the

Top


### HELP

p-value level.

The columns:

- **ExpCount** is the expected number of genes in the- **Count**- **Size** is the total number of genes (in our universe)

Clicking on **Count** shows the genes that drive the
enrichment. You can also click on the individual numbers in
the **Count** column, to show the driving genes for that individual

— Click on the *Help* button again to close this help window.

## Help | Hide | Top Help | Show | Top Genes

### HELP

A list of all genes in the current module, in alphabetical order. The
size of the text corresponds to the gene scores.

Note that some gene symbols may show up more than once, if many
probes match the same Entrez gene.

Genes with no Entrez mapping are given separately, with their
Affymetrics probe ID.

— Click on the *Help* button again to close this help window.

### Genes Symbol

, score:

AatkUnknown, score: 0.57
Abca2Unknown, score: 0.5
Abcb7Unknown, score: 0.31
Asic1Unknown, score: 0.37
Apoc4Unknown, score: 0.26
Acp1Unknown, score: 0.63
Actn3Unknown, score: 0.68
Acta2Unknown, score: 0.81
AspaUnknown, score: 0.26
Adam11Unknown, score: 0.43
Adam12Unknown, score: 0.27
Adam15Unknown, score: 0.32
Adam19Unknown, score: 0.41
Adam22Unknown, score: 0.29
Adam5Unknown, score: 0.69
Adcy6Unknown, score: 0.52
Adcy8Unknown, score: 0.26
Adora2aUnknown, score: 0.35
Adra1dUnknown, score: 0.26
AdslUnknown, score: 0.33
AvilUnknown, score: 0.46
AgaUnknown, score: 0.61
AgrpUnknown, score: 0.5
Akap1Unknown, score: 0.4
Akap2Unknown, score: 0.37
Alox5Unknown, score: 0.26
AmbpUnknown, score: 0.43
Mat1aUnknown, score: 0.27
Anxa11Unknown, score: 0.49
Anxa3Unknown, score: 0.27
Anxa7Unknown, score: 0.66
Ap1g1Unknown, score: 0.3
Ap3d1Unknown, score: 0.4
Apbb2Unknown, score: 0.29
Apex1Unknown, score: 0.28
Birc2Unknown, score: 0.27
XiapUnknown, score: 0.27
Cd5lUnknown, score: 0.4
Apoc3Unknown, score: 0.57
Aqp1Unknown, score: 0.26
Aqp2Unknown, score: 0.66
RhodUnknown, score: 0.28
Arhgap6Unknown, score: 0.34
ArntlUnknown, score: 0.35
Art5Unknown, score: 0.28
Rab27aUnknown, score: 0.35
Serpinc1Unknown, score: 0.41
Atf2Unknown, score: 0.47
Atf3Unknown, score: 0.28
Atox1Unknown, score: 0.3
Atp1b2Unknown, score: 0.32
Atp6v0a1Unknown, score: 0.35
Atp8a1Unknown, score: 0.34
Atp9aUnknown, score: 0.45
Atp6v0cUnknown, score: 0.51
Avpr2Unknown, score: 0.54
Axin1Unknown, score: 0.34
Bcl2Unknown, score: 0.33
Bcl2l2Unknown, score: 0.38
Opn1swUnknown, score: 0.3
Bglap3Unknown, score: 0.33
Bmp10Unknown, score: 0.31
Bmp4Unknown, score: 0.27
Bmpr1bUnknown, score: 0.36
Bnip2Unknown, score: 0.39
Bop1Unknown, score: 0.37
Brca1Unknown, score: 0.28
Commd3Unknown, score: 0.38
TspoUnknown, score: 0.35
CiitaUnknown, score: 0.46
C4bpUnknown, score: 0.31
Cacna1bUnknown, score: 0.36
Cacna2d1Unknown, score: 0.29
Cacnb1Unknown, score: 0.29
Cacnb2Unknown, score: 0.44
Ddr1Unknown, score: 0.77
Anxa2Unknown, score: 0.26
Calm1Unknown, score: 0.26
Calm2Unknown, score: 0.26
Calm3Unknown, score: 0.4
Car8Unknown, score: 0.37
CanxUnknown, score: 0.45
Cap1Unknown, score: 0.54
Capn7Unknown, score: 0.26
CapzbUnknown, score: 0.27
Casp1Unknown, score: 0.5
Casp4Unknown, score: 0.28
Casp2Unknown, score: 0.71
Casp7Unknown, score: 0.32
Cav3Unknown, score: 0.37
Runx2Unknown, score: 0.48
Serpina6Unknown, score: 0.31
Cbx3Unknown, score: 0.28
Cbx4Unknown, score: 0.65
CckUnknown, score: 0.34
Ccnd3Unknown, score: 0.32
Ccne2Unknown, score: 0.26
Ccnt1Unknown, score: 0.3
Cct8Unknown, score: 0.44
Cd22Unknown, score: 0.28
Cd28Unknown, score: 0.32
Cd2apUnknown, score: 0.31
Cd33Unknown, score: 0.85
Cd36Unknown, score: 0.31
Cd38Unknown, score: 0.51
Entpd1Unknown, score: 0.26
Cd3dUnknown, score: 0.3
Cd4Unknown, score: 0.75
Cd5Unknown, score: 0.43
Cd63Unknown, score: 0.31
Cd79aUnknown, score: 0.58
Cd83Unknown, score: 0.49
Cd8b1Unknown, score: 0.47
Cdc25cUnknown, score: 0.35
Arhgap31Unknown, score: 0.35
Cdh1Unknown, score: 0.34
Cdh2Unknown, score: 0.3
Cdh8Unknown, score: 0.26
Cdk5r1Unknown, score: 0.27
Cdk7Unknown, score: 0.5
Cdkn2cUnknown, score: 0.83
Cdo1Unknown, score: 0.34
Ift81Unknown, score: 0.28
Cenpc1Unknown, score: 0.28
Cetn3Unknown, score: 0.36
CfhUnknown, score: 0.28
CflarUnknown, score: 0.44
ChgbUnknown, score: 0.41
Chil1Unknown, score: 0.26
Chl1Unknown, score: 0.37
CishUnknown, score: 0.56
Socs1Unknown, score: 0.28
Cited1Unknown, score: 0.48
CkmUnknown, score: 0.32
Coro1aUnknown, score: 0.33
Clcn4-2Unknown, score: 0.46
Cln3Unknown, score: 0.29
Cxcr4Unknown, score: 0.45
Ccr1l1Unknown, score: 0.37
Plk3Unknown, score: 0.48
Col10a1Unknown, score: 0.35
Col11a2Unknown, score: 0.63
Col13a1Unknown, score: 0.78
Col14a1Unknown, score: 0.31
Col18a1Unknown, score: 0.28
Col19a1Unknown, score: 0.53
Col4a4Unknown, score: 0.36
Col4a5Unknown, score: 0.41
Col5a1Unknown, score: 0.71
Col5a2Unknown, score: 0.46
Col6a2Unknown, score: 0.5
Col7a1Unknown, score: 0.74
Col9a3Unknown, score: 0.55
Col1a1Unknown, score: 0.46
CpUnknown, score: 0.31
Cplx1Unknown, score: 0.26
Cr2Unknown, score: 0.3
Crabp1Unknown, score: 0.33
Creb1Unknown, score: 0.3
Atf6bUnknown, score: 0.62
CrkUnknown, score: 0.31
CrklUnknown, score: 0.77
Cry1Unknown, score: 0.28
Crybb3Unknown, score: 0.29
Csf1rUnknown, score: 0.27
Csf2raUnknown, score: 0.65
Csf2rbUnknown, score: 0.61
Csf3rUnknown, score: 0.4
Dnajc5Unknown, score: 0.75
Csrp1Unknown, score: 0.33
Ctf1Unknown, score: 0.8
Ctla2aUnknown, score: 0.54
CtscUnknown, score: 0.29
CtseUnknown, score: 0.56
CtshUnknown, score: 0.32
CtskUnknown, score: 0.46
CtslUnknown, score: 0.44
CtswUnknown, score: 0.4
CxadrUnknown, score: 0.26
Cyp1a2Unknown, score: 0.29
Cyp7a1Unknown, score: 0.43
Dad1Unknown, score: 0.68
Cd55Unknown, score: 0.26
DbtUnknown, score: 0.75
DcnUnknown, score: 0.5
Dscr3Unknown, score: 0.93
Dhx15Unknown, score: 0.3
DffaUnknown, score: 0.41
DhfrUnknown, score: 0.27
DffbUnknown, score: 0.92
Dlx4Unknown, score: 0.42
Dync1i2Unknown, score: 0.46
Dnm2Unknown, score: 0.3
Doc2bUnknown, score: 0.28
Dpp4Unknown, score: 0.83
Dvl3Unknown, score: 0.39
Lefty1Unknown, score: 0.29
Ebf1Unknown, score: 0.32
EbpUnknown, score: 0.51
Opn3Unknown, score: 0.34
Edil3Unknown, score: 0.35
Edn3Unknown, score: 0.63
EedUnknown, score: 0.3
Efnb2Unknown, score: 0.34
Efnb3Unknown, score: 0.38
Klk1b26Unknown, score: 0.34
Egr1Unknown, score: 0.44
Egr2Unknown, score: 0.28
Ehd1Unknown, score: 0.26
Ei24Unknown, score: 0.42
Eif2b4Unknown, score: 0.27
EllUnknown, score: 0.53
Aimp1Unknown, score: 0.46
Mark2Unknown, score: 0.59
Emp2Unknown, score: 0.31
Emp3Unknown, score: 0.26
En2Unknown, score: 0.41
EnahUnknown, score: 0.33
EndogUnknown, score: 0.45
Eno1Unknown, score: 0.33
Epas1Unknown, score: 0.37
Epb4.1l1Unknown, score: 0.4
Ephx2Unknown, score: 0.3
Epm2aUnknown, score: 0.34
Eps15l1Unknown, score: 0.33
Ercc3Unknown, score: 0.78
Khdrbs3Unknown, score: 0.5
Fgd6Unknown, score: 0.35
Celf2Unknown, score: 0.34
Etv6Unknown, score: 0.3
Ext1Unknown, score: 0.26
Eya3Unknown, score: 0.38
Sfxn1Unknown, score: 0.33
F2Unknown, score: 0.27
F5Unknown, score: 0.35
F7Unknown, score: 0.61
Fscn1Unknown, score: 0.31
FapUnknown, score: 0.44
FblUnknown, score: 0.37
Fbln1Unknown, score: 0.78
Fbln2Unknown, score: 0.58
Fcgr1Unknown, score: 0.57
Fcgr2bUnknown, score: 0.53
Fen1Unknown, score: 0.35
Fgf10Unknown, score: 0.37
Fgf6Unknown, score: 0.42
Fgf7Unknown, score: 0.38
Fgfbp1Unknown, score: 0.27
Fgfr2Unknown, score: 0.34
Fgfr4Unknown, score: 0.7
Akr1b8Unknown, score: 0.95
Fgl2Unknown, score: 0.29
FhitUnknown, score: 0.83
Il4i1Unknown, score: 0.39
Ppm1gUnknown, score: 0.39
Fkbp10Unknown, score: 0.34
Fkbp8Unknown, score: 0.37
Fmr1Unknown, score: 0.41
Aff2Unknown, score: 0.86
Folr2Unknown, score: 0.44
Fosl1Unknown, score: 0.67
FpgsUnknown, score: 0.33
Fpr2Unknown, score: 0.55
FxnUnknown, score: 0.38
Frg1Unknown, score: 0.27
FstUnknown, score: 0.84
FynUnknown, score: 0.45
Fzd3Unknown, score: 0.29
Gab1Unknown, score: 0.29
Gabra1Unknown, score: 0.31
GabrdUnknown, score: 0.28
Gabrr2Unknown, score: 0.29
GalcUnknown, score: 0.47
GartUnknown, score: 0.56
Gas6Unknown, score: 0.39
Gata6Unknown, score: 0.43
Gcnt1Unknown, score: 0.76
Gcnt2Unknown, score: 0.62
Gdi2Unknown, score: 0.55
GdnfUnknown, score: 0.53
GemUnknown, score: 0.3
Gfpt1Unknown, score: 0.84
Ggps1Unknown, score: 0.33
Gjb5Unknown, score: 0.35
Gli1Unknown, score: 0.43
Gli2Unknown, score: 0.5
Gna12Unknown, score: 0.29
Gnai1Unknown, score: 0.28
Gnai2Unknown, score: 0.54
GnasUnknown, score: 0.31
Gng5Unknown, score: 0.56
Gng7Unknown, score: 0.45
Lrp2Unknown, score: 0.68
PdpnUnknown, score: 0.53
Gp49aUnknown, score: 0.63
Gpr12Unknown, score: 0.33
Gpr65Unknown, score: 0.45
Lpar1Unknown, score: 0.33
Gpld1Unknown, score: 0.43
Grk4Unknown, score: 0.82
Grk5Unknown, score: 0.43
Gpx1Unknown, score: 0.4
Cdca3Unknown, score: 0.43
Grik5Unknown, score: 0.52
Grin2aUnknown, score: 0.66
GrnUnknown, score: 0.33
Hspa5Unknown, score: 0.37
Gspt1Unknown, score: 0.33
Guk1Unknown, score: 0.27
GypaUnknown, score: 0.31
H2-Ab1Unknown, score: 0.29
15061Unknown, score: 0.28
HalUnknown, score: 0.64
HarsUnknown, score: 0.55
Has1Unknown, score: 0.58
Hdac6Unknown, score: 0.39
HexbUnknown, score: 0.31
Hey2Unknown, score: 0.51
HgdUnknown, score: 0.46
Hint1Unknown, score: 0.35
Hipk3Unknown, score: 0.45
H2afxUnknown, score: 0.41
Hmga2Unknown, score: 0.3
HmmrUnknown, score: 0.44
Hmox2Unknown, score: 0.66
Hoxd9Unknown, score: 0.46
Hrh2Unknown, score: 0.3
Hrsp12Unknown, score: 0.44
Hspa8Unknown, score: 0.45
Hsd17b4Unknown, score: 0.28
Hspd1Unknown, score: 0.27
Hspa1bUnknown, score: 0.42
Htr2aUnknown, score: 0.33
Hyal2Unknown, score: 0.32
Ica1Unknown, score: 0.57
Ifngr2Unknown, score: 0.36
Igf1Unknown, score: 0.4
Igfbp4Unknown, score: 0.55
Igfbp5Unknown, score: 0.41
Il16Unknown, score: 0.35
Il1aUnknown, score: 0.28
Il1bUnknown, score: 0.53
Il1r1Unknown, score: 0.33
Irak1Unknown, score: 0.26
Il1rapUnknown, score: 0.79
Il2rbUnknown, score: 0.33
Il3raUnknown, score: 0.46
Il5raUnknown, score: 0.43
Il7Unknown, score: 0.33
Lrig1Unknown, score: 0.63
Kpnb1Unknown, score: 0.29
InhaUnknown, score: 0.66
InhbaUnknown, score: 0.33
InvsUnknown, score: 0.28
Irf1Unknown, score: 0.38
Irf4Unknown, score: 0.29
Irf9Unknown, score: 0.37
ItchUnknown, score: 0.47
Itga3Unknown, score: 0.28
ItgaeUnknown, score: 0.34
ItgalUnknown, score: 0.63
ItgamUnknown, score: 0.27
ItgavUnknown, score: 0.58
ItgaxUnknown, score: 0.3
Itgb1Unknown, score: 0.3
Itgb1bp1Unknown, score: 0.48
Itgb2Unknown, score: 0.48
Itih3Unknown, score: 0.29
ItkUnknown, score: 0.46
Itm2bUnknown, score: 0.78
Itpr3Unknown, score: 0.26
Itsn1Unknown, score: 0.27
Jag2Unknown, score: 0.3
JunUnknown, score: 0.33
Kcnab1Unknown, score: 0.34
Kcnab2Unknown, score: 0.3
Kcnb1Unknown, score: 0.35
Kcnc1Unknown, score: 0.56
Kcne1Unknown, score: 0.39
Kcnj16Unknown, score: 0.39
Kcnu1Unknown, score: 0.38
Kcnn4Unknown, score: 0.41
KhsrpUnknown, score: 0.63
Kif17Unknown, score: 0.37
Kif1aUnknown, score: 0.3
Kif1bUnknown, score: 0.28
Kif3aUnknown, score: 0.37
Kif3bUnknown, score: 0.38
Kif5aUnknown, score: 0.71
Kif7Unknown, score: 0.4
Klrc1Unknown, score: 0.27
Kng1Unknown, score: 0.28
Kpna1Unknown, score: 0.34
SspnUnknown, score: 0.27
KrasUnknown, score: 0.33
Krt10Unknown, score: 0.26
Krt14Unknown, score: 0.64
Krt18Unknown, score: 0.51
Krt19Unknown, score: 0.26
Lad1Unknown, score: 0.51
RpsaUnknown, score: 0.61
AnpepUnknown, score: 0.37
Arhgef1Unknown, score: 0.43
LckUnknown, score: 0.27
Ldb1Unknown, score: 0.39
Lgals1Unknown, score: 0.74
Lgals4Unknown, score: 0.28
Limk2Unknown, score: 0.32
GzmmUnknown, score: 0.55
LorUnknown, score: 0.61
Loxl1Unknown, score: 0.38
Loxl3Unknown, score: 0.56
LrmpUnknown, score: 0.36
Lrp5Unknown, score: 0.37
Lrrn1Unknown, score: 0.39
Lrrn3Unknown, score: 0.56
Ltb4r1Unknown, score: 0.85
Ltbp2Unknown, score: 0.49
Ltbp3Unknown, score: 0.4
LtkUnknown, score: 0.52
AladUnknown, score: 0.33
LynUnknown, score: 0.41
LystUnknown, score: 0.4
Lyz2Unknown, score: 0.42
Smad5Unknown, score: 0.29
Smad6Unknown, score: 0.46
MafUnknown, score: 0.35
MagohUnknown, score: 0.5
MalUnknown, score: 0.77
Man1aUnknown, score: 0.32
Man1a2Unknown, score: 0.49
MaoaUnknown, score: 0.43
Mapkapk2Unknown, score: 0.59
MatkUnknown, score: 0.51
Matr3Unknown, score: 0.28
Cma1Unknown, score: 0.28
Mdm1Unknown, score: 0.32
Men1Unknown, score: 0.5
MestUnknown, score: 0.3
MfngUnknown, score: 0.48
Clec10aUnknown, score: 0.28
Pias2Unknown, score: 0.4
Mllt4Unknown, score: 0.39
MmeUnknown, score: 0.59
Mmp13Unknown, score: 0.55
Mmp15Unknown, score: 0.31
Mmp16Unknown, score: 0.58
Mmp24Unknown, score: 0.45
Mmp3Unknown, score: 0.29
Gbp4Unknown, score: 0.9
MpdzUnknown, score: 0.4
MpzUnknown, score: 0.44
MrasUnknown, score: 0.46
Mrc1Unknown, score: 0.72
Msh3Unknown, score: 0.38
Msi1Unknown, score: 0.73
Msl3Unknown, score: 0.29
MsnUnknown, score: 0.48
Grpel1Unknown, score: 0.51
mt-Nd6Unknown, score: 0.58
Polr2kUnknown, score: 0.7
Mt3Unknown, score: 0.28
Map1aUnknown, score: 0.51
Map2Unknown, score: 0.31
Map4Unknown, score: 0.33
Nudt1Unknown, score: 0.29
MybUnknown, score: 0.31
MycUnknown, score: 0.62
Myh6Unknown, score: 0.29
Myo1fUnknown, score: 0.52
Ppp1r12aUnknown, score: 0.42
Nab1Unknown, score: 0.31
Naip1Unknown, score: 0.28
Nat2Unknown, score: 0.7
Ncam2Unknown, score: 0.31
Ncf1Unknown, score: 0.3
Ncf2Unknown, score: 0.37
NclUnknown, score: 0.38
Ncoa3Unknown, score: 0.33
Ndufv1Unknown, score: 0.47
Nedd8Unknown, score: 0.32
Nek1Unknown, score: 0.27
Neu1Unknown, score: 0.44
Nf1Unknown, score: 0.28
NficUnknown, score: 0.35
NfkbibUnknown, score: 0.41
Nid2Unknown, score: 0.38
Nqo1Unknown, score: 0.44
Nmt1Unknown, score: 0.35
NnatUnknown, score: 0.36
NogUnknown, score: 0.9
Npr3Unknown, score: 0.34
Ctnnd2Unknown, score: 0.26
Slc11a1Unknown, score: 0.35
Nsd1Unknown, score: 0.28
NsfUnknown, score: 0.29
Ntn3Unknown, score: 0.35
NudcUnknown, score: 0.39
Nup62Unknown, score: 0.62
OgnUnknown, score: 0.69
OmpUnknown, score: 0.64
OsmUnknown, score: 0.55
OtcUnknown, score: 0.32
OtogUnknown, score: 0.39
P2rx4Unknown, score: 0.37
P2rx7Unknown, score: 0.29
Pafah1b2Unknown, score: 0.27
PahUnknown, score: 0.43
Pak1Unknown, score: 0.69
Pak3Unknown, score: 0.27
PalmUnknown, score: 0.6
PcntUnknown, score: 0.45
Pcp4Unknown, score: 0.31
Pcsk2Unknown, score: 0.44
Pcsk6Unknown, score: 0.27
Pdcd2Unknown, score: 0.29
Pdcd11Unknown, score: 0.51
Pde1cUnknown, score: 0.52
Pde3bUnknown, score: 0.3
Pde4aUnknown, score: 0.63
Pde7aUnknown, score: 0.36
Pde8aUnknown, score: 0.56
PdgfbUnknown, score: 0.43
Pdha1Unknown, score: 0.33
Padi3Unknown, score: 0.3
Pdk2Unknown, score: 0.29
Etv4Unknown, score: 0.3
Pecam1Unknown, score: 0.74
Pex7Unknown, score: 0.53
Prf1Unknown, score: 0.29
Cdk14Unknown, score: 0.36
Phf2Unknown, score: 0.56
Phka1Unknown, score: 0.73
Phkg1Unknown, score: 0.31
PigrUnknown, score: 0.49
Pik3r3Unknown, score: 0.32
Pip5k1bUnknown, score: 0.81
Pip5k1aUnknown, score: 0.5
18722Unknown, score: 0.5
Gm14548Unknown, score: 0.45
Pira2Unknown, score: 0.34
Gm15448Unknown, score: 0.5
Gm14548Unknown, score: 0.5
Gm14548Unknown, score: 0.5
Gm14548Unknown, score: 0.31
Pou1f1Unknown, score: 0.27
PitpnaUnknown, score: 0.26
Pla2r1Unknown, score: 0.48
Pla2g2dUnknown, score: 0.49
Pla2g4aUnknown, score: 0.35
Serpine1Unknown, score: 0.4
PapolaUnknown, score: 0.42
PlauUnknown, score: 0.29
PlaurUnknown, score: 0.28
Plcb3Unknown, score: 0.5
Pld1Unknown, score: 0.28
Plp2Unknown, score: 0.38
Pmp2Unknown, score: 0.34
PnpUnknown, score: 0.5
Pon1Unknown, score: 0.26
Pou4f1Unknown, score: 0.26
PparaUnknown, score: 0.59
PpargUnknown, score: 0.38
PpicUnknown, score: 0.32
PplUnknown, score: 0.28
Ppp1ccUnknown, score: 0.27
PrepUnknown, score: 0.31
Prim1Unknown, score: 0.26
Prkar2aUnknown, score: 0.37
PrkdcUnknown, score: 0.63
PrkxUnknown, score: 0.69
Prom1Unknown, score: 0.32
Prps1Unknown, score: 0.35
Psme3Unknown, score: 0.47
Pstpip1Unknown, score: 0.29
Ptch1Unknown, score: 0.28
PtgdsUnknown, score: 0.33
Ptger1Unknown, score: 0.33
Ptger3Unknown, score: 0.51
PtgirUnknown, score: 0.68
Ptgs1Unknown, score: 0.52
Ptgs2Unknown, score: 0.41
Pth1rUnknown, score: 0.43
Ptk2bUnknown, score: 0.49
Twf1Unknown, score: 0.26
Tmsb4xUnknown, score: 0.26
Ptp4a1Unknown, score: 0.36
Ptp4a3Unknown, score: 0.35
Ptpn18Unknown, score: 0.41
PtpraUnknown, score: 0.3
PtprcapUnknown, score: 0.28
PtpreUnknown, score: 0.41
PtprgUnknown, score: 0.27
PtprjUnknown, score: 0.38
PtprkUnknown, score: 0.45
PtprrUnknown, score: 0.35
PtprsUnknown, score: 0.33
PtsUnknown, score: 0.61
Rab12Unknown, score: 0.33
Dennd5aUnknown, score: 0.39
Rad23bUnknown, score: 0.42
Rad9aUnknown, score: 0.38
Raet1dUnknown, score: 0.38
Rai1Unknown, score: 0.61
Ranbp1Unknown, score: 0.31
RapsnUnknown, score: 0.55
Rasal1Unknown, score: 0.42
Rasl2-9Unknown, score: 0.26
Rbmxl1Unknown, score: 0.42
Rem1Unknown, score: 0.35
Bex1Unknown, score: 0.35
Rfx1Unknown, score: 0.79
Rfx2Unknown, score: 0.27
RgnUnknown, score: 0.41
Rgs16Unknown, score: 0.38
Ralbp1Unknown, score: 0.39
Rit1Unknown, score: 0.38
Rnps1Unknown, score: 0.51
Rock1Unknown, score: 0.68
Slc22a8Unknown, score: 0.3
Rom1Unknown, score: 0.44
Mst1rUnknown, score: 0.35
RorcUnknown, score: 0.7
Rp1Unknown, score: 0.29
RrasUnknown, score: 0.28
Ryr1Unknown, score: 0.66
S100a1Unknown, score: 0.49
S100a10Unknown, score: 0.61
S100a3Unknown, score: 0.48
Saa2Unknown, score: 0.72
Atxn2Unknown, score: 0.41
Scd1Unknown, score: 0.47
Clec11aUnknown, score: 0.32
Stmn3Unknown, score: 0.42
Scn10aUnknown, score: 0.44
Scn3aUnknown, score: 0.44
Scn5aUnknown, score: 0.31
Zc3h7bUnknown, score: 0.27
SctUnknown, score: 0.67
Ccl11Unknown, score: 0.38
Ccl17Unknown, score: 0.63
Ccl2Unknown, score: 0.57
Ccl22Unknown, score: 0.28
Ccl27aUnknown, score: 0.46
Ccl5Unknown, score: 0.57
Ccl6Unknown, score: 0.41
Ccl8Unknown, score: 0.66
Ccl9Unknown, score: 0.43
Cxcl2Unknown, score: 0.55
Cxcl5Unknown, score: 0.49
Cx3cl1Unknown, score: 0.37
Sdf2Unknown, score: 0.6
Sdf4Unknown, score: 0.27
Sfrp2Unknown, score: 0.37
Sec22bUnknown, score: 0.54
Sel1lUnknown, score: 0.64
SellUnknown, score: 0.52
Sema3bUnknown, score: 0.28
Spi1Unknown, score: 0.74
Sfrp1Unknown, score: 0.41
FrzbUnknown, score: 0.42
SgceUnknown, score: 0.49
Itsn2Unknown, score: 0.86
Shc1Unknown, score: 0.28
Shc3Unknown, score: 0.32
Siah1bUnknown, score: 0.65
St8sia4Unknown, score: 0.33
ClpbUnknown, score: 0.43
SkiUnknown, score: 0.78
Slc10a1Unknown, score: 0.39
Slc16a1Unknown, score: 0.49
Slc16a2Unknown, score: 0.76
Slc1a1Unknown, score: 0.63
Slc1a2Unknown, score: 0.44
Slc1a3Unknown, score: 0.28
Slc1a5Unknown, score: 0.43
Slc2a4Unknown, score: 0.28
Slc6a2Unknown, score: 0.53
Slfn3Unknown, score: 0.41
Ncor2Unknown, score: 0.27
Sstr2Unknown, score: 0.46
Sstr4Unknown, score: 0.53
SnrpeUnknown, score: 0.72
Sntb1Unknown, score: 0.39
Sntb2Unknown, score: 0.59
Sod2Unknown, score: 0.43
Sort1Unknown, score: 0.6
Sox15Unknown, score: 0.4
Sox8Unknown, score: 0.26
Sp4Unknown, score: 0.43
SparcUnknown, score: 0.48
Serpina1eUnknown, score: 0.66
Serpina3kUnknown, score: 0.74
Serpina3nUnknown, score: 0.33
Serpinb6aUnknown, score: 0.64
Serpinb9Unknown, score: 0.34
Spta1Unknown, score: 0.62
SpopUnknown, score: 0.78
SprUnknown, score: 0.7
Scarb1Unknown, score: 0.37
Stag1Unknown, score: 0.34
Stag2Unknown, score: 0.27
StamUnknown, score: 0.51
Stat2Unknown, score: 0.61
Stau1Unknown, score: 0.28
Stk10Unknown, score: 0.47
AC146980.1Unknown, score: 0.3
SlkUnknown, score: 0.34
Stra13Unknown, score: 0.47
Bhlhe40Unknown, score: 0.63
Stxbp1Unknown, score: 0.36
Stxbp3aUnknown, score: 0.33
SykUnknown, score: 0.33
Syt2Unknown, score: 0.28
Syt3Unknown, score: 0.29
Taf6Unknown, score: 0.41
TaglnUnknown, score: 0.52
Tarbp2Unknown, score: 0.31
Tbx1Unknown, score: 0.26
Tbx3Unknown, score: 0.49
Hnf1aUnknown, score: 0.49
TfebUnknown, score: 0.34
TectaUnknown, score: 0.3
TertUnknown, score: 0.31
TfamUnknown, score: 0.54
TfpiUnknown, score: 0.33
Tgfb1Unknown, score: 0.3
Tsc22d1Unknown, score: 0.53
Tgfb2Unknown, score: 0.34
Tgfb3Unknown, score: 0.34
TgfbiUnknown, score: 0.4
Tgfbr1Unknown, score: 0.32
Tgfbr3Unknown, score: 0.61
Tgtp1Unknown, score: 0.32
ThbdUnknown, score: 0.66
Thbs2Unknown, score: 0.79
Thbs3Unknown, score: 0.65
Timm44Unknown, score: 1
Timp1Unknown, score: 0.5
Tle2Unknown, score: 0.52
Tlr1Unknown, score: 0.58
TmpoUnknown, score: 0.27
Clec3bUnknown, score: 0.95
Tnfaip3Unknown, score: 0.7
Tnfrsf18Unknown, score: 0.44
Tnfrsf1aUnknown, score: 0.33
Tnnt2Unknown, score: 0.34
Top1Unknown, score: 0.41
Tmem165Unknown, score: 0.37
Tpm1Unknown, score: 0.27
Hsp90b1Unknown, score: 0.35
Traf2Unknown, score: 0.28
Trp53Unknown, score: 0.5
Trpc4Unknown, score: 0.35
Psmd3Unknown, score: 0.52
TtnUnknown, score: 0.31
TubUnknown, score: 0.37
Tuba3bUnknown, score: 0.34
Tubb2aUnknown, score: 0.5
TyrUnknown, score: 0.38
Tyro3Unknown, score: 0.28
Uba1Unknown, score: 0.32
UcnUnknown, score: 0.59
UgcgUnknown, score: 0.77
Ugt2b5Unknown, score: 0.41
Unc119Unknown, score: 0.28
Usp4Unknown, score: 0.51
Kdm6aUnknown, score: 0.31
Cdh23Unknown, score: 0.3
Vmn1r45Unknown, score: 0.35
Vbp1Unknown, score: 0.29
EzrUnknown, score: 0.28
VillUnknown, score: 0.69
Wbp4Unknown, score: 0.38
Wisp1Unknown, score: 0.3
Wnt1Unknown, score: 0.4
Wnt11Unknown, score: 0.41
Wnt9bUnknown, score: 0.3
Wnt4Unknown, score: 0.49
Wnt6Unknown, score: 0.39
Dctn6Unknown, score: 0.59
YwhaqUnknown, score: 0.28
Yy1Unknown, score: 0.37
Plagl1Unknown, score: 0.31
Zfp13Unknown, score: 0.3
ZfrUnknown, score: 0.33
ZfxUnknown, score: 0.41
Slc30a4Unknown, score: 0.68
Dnajc2Unknown, score: 0.3
Coro1bUnknown, score: 0.44
Coro1cUnknown, score: 0.31
AplnrUnknown, score: 0.43
Aloxe3Unknown, score: 0.32
Apc2Unknown, score: 0.27
Cd52Unknown, score: 0.28
Pappa2Unknown, score: 0.26
Dmtf1Unknown, score: 0.3
Ets2Unknown, score: 0.27
FarsbUnknown, score: 0.32
Ggt5Unknown, score: 0.83
InsrrUnknown, score: 0.31
Mmp17Unknown, score: 0.28
Tenm2Unknown, score: 0.31
Nlrp5Unknown, score: 0.56
Pcbp1Unknown, score: 0.55
Med24Unknown, score: 0.27
Ccl19Unknown, score: 0.35
SigirrUnknown, score: 0.61
Tekt2Unknown, score: 0.26
Wif1Unknown, score: 0.27
Zfp68Unknown, score: 0.44
Ing1Unknown, score: 0.26
Aldh1a7Unknown, score: 0.27
Angptl2Unknown, score: 0.47
Map3k12Unknown, score: 0.35
Mapk1Unknown, score: 0.56
Nr5a2Unknown, score: 0.38
Nubp2Unknown, score: 0.44
Plod2Unknown, score: 0.57
PrndUnknown, score: 0.28
PoliUnknown, score: 0.37
HunkUnknown, score: 0.3
NcdnUnknown, score: 0.41
Slc27a4Unknown, score: 0.28
B3galnt1Unknown, score: 0.36
Acot1Unknown, score: 0.3
GcatUnknown, score: 0.26
Zfp346Unknown, score: 0.71
Ppp2r5eUnknown, score: 0.41
Serinc3Unknown, score: 0.26
Cul1Unknown, score: 0.48
Micall1Unknown, score: 0.29
AoahUnknown, score: 0.33
Sec23bUnknown, score: 0.39
Fkbp9Unknown, score: 0.43
Srp9Unknown, score: 0.51
Bcap31Unknown, score: 0.34
Rps11Unknown, score: 0.32
Dnajb9Unknown, score: 0.33
Hsd17b6Unknown, score: 0.26
PdhxUnknown, score: 0.4
NagpaUnknown, score: 0.29
Ubl4Unknown, score: 0.26
Slco1a1Unknown, score: 0.36
Tpk1Unknown, score: 0.27
MgaUnknown, score: 0.37
Hip1rUnknown, score: 0.44
Igfbp7Unknown, score: 0.29
Stau2Unknown, score: 0.61
Dpf1Unknown, score: 0.28
Ulk2Unknown, score: 0.29
Cspg5Unknown, score: 0.41
Lmcd1Unknown, score: 0.29
Usp21Unknown, score: 0.32
Siva1Unknown, score: 0.31
Hacd1Unknown, score: 0.63
Mrps7Unknown, score: 0.9
Mfap5Unknown, score: 0.96
PostnUnknown, score: 0.41
Fbxo8Unknown, score: 0.32
Polg2Unknown, score: 0.46
Rgs3Unknown, score: 0.87
Dkk3Unknown, score: 0.56
Hs6st1Unknown, score: 0.66
Tmod3Unknown, score: 0.41
PrebUnknown, score: 0.41
C1s1Unknown, score: 0.35
Irx4Unknown, score: 0.42
GalnsUnknown, score: 0.34
Ppp2r1aUnknown, score: 0.34
CcncUnknown, score: 0.38
Fubp1Unknown, score: 0.71
Ccdc39Unknown, score: 0.32
R3hcc1lUnknown, score: 0.55
Reep1Unknown, score: 0.31
Klhl7Unknown, score: 0.5
Stbd1Unknown, score: 0.47
D6Ertd527eUnknown, score: 0.3
Rcn3Unknown, score: 0.29
Zfp644Unknown, score: 0.3
Angel2Unknown, score: 0.39
Tspan14Unknown, score: 0.28
Nudcd2Unknown, score: 0.62
Echdc1Unknown, score: 0.31
Cpsf4lUnknown, score: 0.33
Mettl2Unknown, score: 0.27
Ccdc59Unknown, score: 0.27
D19Bwg1357eUnknown, score: 0.53
RnasekUnknown, score: 0.26
Plrg1Unknown, score: 0.76
Pdlim3Unknown, score: 0.34
Cntnap1Unknown, score: 0.28
Pla2g6Unknown, score: 0.79
Txnl1Unknown, score: 0.37
Htatip2Unknown, score: 0.32
B4galt2Unknown, score: 0.26
ReckUnknown, score: 0.27
Cnot4Unknown, score: 0.28
Sept9Unknown, score: 0.26
Rfx5Unknown, score: 0.72
Lpar2Unknown, score: 0.26
Rps28Unknown, score: 0.27
Avpr1aUnknown, score: 0.29
Arl6ip1Unknown, score: 0.26
Cd320Unknown, score: 0.33
Arhgef5Unknown, score: 0.26
Irx5Unknown, score: 0.37
Rpp30Unknown, score: 0.89
Nme6Unknown, score: 0.39
Cacng6Unknown, score: 0.27
Mtmr7Unknown, score: 0.35
Stk19Unknown, score: 0.64
HgfacUnknown, score: 0.33
Nfat5Unknown, score: 0.29
TollipUnknown, score: 0.48
HpgdsUnknown, score: 0.4
Nup210Unknown, score: 0.46
Socs6Unknown, score: 0.45
Tbc1d8Unknown, score: 0.41
Wdr45Unknown, score: 0.37
Ccdc120Unknown, score: 0.34
Atp8b2Unknown, score: 0.6
Plagl2Unknown, score: 0.33
Rcan1Unknown, score: 0.36
Cadm1Unknown, score: 0.41
Pdzrn3Unknown, score: 0.62
Cxcl13Unknown, score: 0.45
Fmo2Unknown, score: 0.74
Stard10Unknown, score: 0.37
Uqcc1Unknown, score: 0.29
Lgals8Unknown, score: 0.28
DgkeUnknown, score: 0.31
PigpUnknown, score: 0.47
Rbm38Unknown, score: 0.28
TroUnknown, score: 0.48
Cited4Unknown, score: 0.37
Tspan5Unknown, score: 0.4
Actr8Unknown, score: 0.69
Rbm14Unknown, score: 0.44
Mrpl19Unknown, score: 0.29
Slc35g3Unknown, score: 0.3
Arl6Unknown, score: 0.52
Metap2Unknown, score: 0.42
Gps2Unknown, score: 0.61
AcppUnknown, score: 0.79
Mettl3Unknown, score: 0.52
B4galt4Unknown, score: 0.3
Pdlim5Unknown, score: 0.57
Trip4Unknown, score: 0.29
Ykt6Unknown, score: 0.44
Stub1Unknown, score: 0.47
Mtch2Unknown, score: 0.34
DstnUnknown, score: 0.35
Dnaja2Unknown, score: 0.29
Mbtps1Unknown, score: 0.46
Rgs19Unknown, score: 0.45
Nxt1Unknown, score: 0.39
VapbUnknown, score: 0.34
Asna1Unknown, score: 0.32
Ruvbl1Unknown, score: 0.52
Rapgef4Unknown, score: 0.26
Txn2Unknown, score: 0.38
Pfdn5Unknown, score: 0.35
Mgst1Unknown, score: 0.47
Clec4eUnknown, score: 0.76
Fgf21Unknown, score: 0.28
PigoUnknown, score: 0.46
Tdo2Unknown, score: 0.52
CriptUnknown, score: 0.36
Lat2Unknown, score: 0.29
Pf4Unknown, score: 0.36
C1qtnf1Unknown, score: 0.35
Zbtb33Unknown, score: 0.29
Gmeb1Unknown, score: 0.34
Dkk2Unknown, score: 0.3
Necab3Unknown, score: 0.55
Insm2Unknown, score: 0.37
Sap30bpUnknown, score: 0.4
RetnUnknown, score: 0.75
BC051019Unknown, score: 0.34
B4galt3Unknown, score: 0.45
Slc15a2Unknown, score: 0.28
Piwil2Unknown, score: 0.52
Fxyd7Unknown, score: 0.37
Rpl35aUnknown, score: 0.31
Tk2Unknown, score: 0.46
Il17reUnknown, score: 0.31
Ccdc126Unknown, score: 0.33
Krcc1Unknown, score: 0.3
Zfp318Unknown, score: 0.71
Cst10Unknown, score: 0.35
Rab37Unknown, score: 0.69
Fam184bUnknown, score: 0.41
Shank3Unknown, score: 0.41
CrbnUnknown, score: 0.45
Trpm7Unknown, score: 0.26
MlxiplUnknown, score: 0.31
Slco1c1Unknown, score: 0.3
Akr1a1Unknown, score: 0.38
Efemp2Unknown, score: 0.84
Cysltr1Unknown, score: 0.33
Fam13aUnknown, score: 0.34
Smpd3Unknown, score: 0.33
MkksUnknown, score: 0.35
Tpm3Unknown, score: 0.27
Pcbp4Unknown, score: 0.47
Pcbp3Unknown, score: 0.3
Nek7Unknown, score: 0.5
Dctn5Unknown, score: 0.71
Trpv4Unknown, score: 0.29
Syne1Unknown, score: 0.26
Clstn2Unknown, score: 0.58
Sdf2l1Unknown, score: 0.39
RalbUnknown, score: 0.58
Suv39h2Unknown, score: 0.45
Svep1Unknown, score: 0.29
Rtn4rUnknown, score: 0.71
Dap3Unknown, score: 0.4
Prrg2Unknown, score: 0.4
CubnUnknown, score: 0.7
TbataUnknown, score: 0.27
Txndc12Unknown, score: 0.28
Tspan13Unknown, score: 0.36
Tmem57Unknown, score: 0.26
Dnajc15Unknown, score: 0.39
Acyp1Unknown, score: 0.32
Zdhhc12Unknown, score: 0.64
Dmap1Unknown, score: 0.27
Pno1Unknown, score: 0.45
AamdcUnknown, score: 0.27
Blzf1Unknown, score: 0.63
Pcp4l1Unknown, score: 0.37
Mgst3Unknown, score: 0.32
Mrpl51Unknown, score: 0.28
CenpmUnknown, score: 0.35
Uqcr11Unknown, score: 0.26
Lrrc57Unknown, score: 0.27
Uba5Unknown, score: 0.42
Tmem41aUnknown, score: 0.36
Oser1Unknown, score: 0.35
Dcbld1Unknown, score: 0.26
Tbc1d15Unknown, score: 0.38
Uqcrfs1Unknown, score: 0.81
Henmt1Unknown, score: 0.54
Kcng4Unknown, score: 0.28
Emc2Unknown, score: 0.38
Lztr1Unknown, score: 0.52
PmpcaUnknown, score: 0.28
Cpne8Unknown, score: 0.28
Lonp2Unknown, score: 0.62
Rexo1Unknown, score: 0.29
Acad8Unknown, score: 0.32
Tmem206Unknown, score: 0.36
Trappc13Unknown, score: 0.26
Lap3Unknown, score: 0.33
Yae1d1Unknown, score: 0.51
PaicsUnknown, score: 0.47
Rps6ka6Unknown, score: 0.35
PgpUnknown, score: 0.57
D16Ertd472eUnknown, score: 0.52
Cuedc2Unknown, score: 0.34
Nkain1Unknown, score: 0.29
Lipt2Unknown, score: 0.28
Med18Unknown, score: 0.32
Prpf18Unknown, score: 0.33
Gemin6Unknown, score: 0.67
Peli1Unknown, score: 0.51
Rpl39Unknown, score: 0.41
Ndufa10Unknown, score: 0.3
Ift22Unknown, score: 0.31
Rab3cUnknown, score: 0.38
Gprasp1Unknown, score: 0.41
Zc3h13Unknown, score: 0.3
Zc2hc1aUnknown, score: 0.45
Mrpl46Unknown, score: 0.32
RfflUnknown, score: 0.31
SrprUnknown, score: 0.57
Mfn1Unknown, score: 0.34
Nudcd1Unknown, score: 0.38
Pnpla8Unknown, score: 0.48
Slc25a46Unknown, score: 0.28
IkbipUnknown, score: 0.7
Ccar1Unknown, score: 0.55
Zswim3Unknown, score: 0.31
GstcdUnknown, score: 0.36
PigmUnknown, score: 0.26
NarflUnknown, score: 0.4
Mrfap1Unknown, score: 0.37
Akt1s1Unknown, score: 0.5
NarfUnknown, score: 0.32
Nob1Unknown, score: 0.77
Bend5Unknown, score: 0.5
Spc24Unknown, score: 0.6
4930523C07RikUnknown, score: 0.27
Luc7l3Unknown, score: 0.28
Fam174aUnknown, score: 0.35
MgarpUnknown, score: 0.7
Rtp4Unknown, score: 0.29
Slc35a4Unknown, score: 0.43
Ddx55Unknown, score: 0.3
Echdc3Unknown, score: 0.55
RprmUnknown, score: 0.55
Mdp1Unknown, score: 0.37
1810043G02RikUnknown, score: 0.35
SarafUnknown, score: 0.53
Rbm18Unknown, score: 0.35
Tmem86aUnknown, score: 0.29
Ppa1Unknown, score: 0.52
Rpl41Unknown, score: 0.36
Zcchc10Unknown, score: 0.42
Pold3Unknown, score: 0.67
Tppp3Unknown, score: 0.26
CcnyUnknown, score: 0.59
TrabdUnknown, score: 0.41
Tmx3Unknown, score: 0.46
Fam134cUnknown, score: 0.43
Sdhaf4Unknown, score: 0.47
Rpl22l1Unknown, score: 0.27
N6amt2Unknown, score: 0.27
3010026O09RikUnknown, score: 0.61
Atg101Unknown, score: 0.3
Etaa1Unknown, score: 0.56
EbplUnknown, score: 0.29
Ndufa5Unknown, score: 0.46
Fam166aUnknown, score: 0.33
Trmt12Unknown, score: 0.59
Toe1Unknown, score: 0.61
Ddx39Unknown, score: 0.5
Vps53Unknown, score: 0.53
Fam114a1Unknown, score: 0.37
Tsr3Unknown, score: 0.31
SycnUnknown, score: 0.29
Ankrd13dUnknown, score: 0.3
Slc39a13Unknown, score: 0.34
Zfyve21Unknown, score: 0.47
Uckl1Unknown, score: 0.35
Tmed10Unknown, score: 0.34
Rtn4Unknown, score: 0.5
Dnajc8Unknown, score: 0.27
Samm50Unknown, score: 0.77
Rnf166Unknown, score: 0.55
Trp53inp2Unknown, score: 0.58
RbfaUnknown, score: 0.38
Tmem219Unknown, score: 0.35
Wash1Unknown, score: 0.34
Phtf2Unknown, score: 0.63
Gucd1Unknown, score: 0.33
Srpx2Unknown, score: 0.28
Tmem214Unknown, score: 0.55
Dock5Unknown, score: 0.3
Zfand2bUnknown, score: 0.73
Tulp4Unknown, score: 0.27
Rnf208Unknown, score: 0.52
1810011O10RikUnknown, score: 0.38
Fam58bUnknown, score: 0.53
Tmem110Unknown, score: 0.6
C1qtnf2Unknown, score: 0.28
Ccdc23Unknown, score: 0.52
CarkdUnknown, score: 0.27
Zfp397Unknown, score: 0.29
Kctd5Unknown, score: 0.29
3300002I08RikUnknown, score: 0.39
Slc16a13Unknown, score: 0.66
PacrgUnknown, score: 0.68
Slc38a4Unknown, score: 0.29
Cdhr4Unknown, score: 0.52
1700029J07RikUnknown, score: 0.61
Dnase1l1Unknown, score: 0.56
Bod1Unknown, score: 0.31
Fastkd3Unknown, score: 0.35
Hfe2Unknown, score: 0.34
Pcgf3Unknown, score: 0.28
Cd164l2Unknown, score: 0.44
Ndufaf1Unknown, score: 0.3
Trip13Unknown, score: 0.34
Usp46Unknown, score: 0.27
1810026J23RikUnknown, score: 0.43
Polr2fUnknown, score: 0.41
1700026L06RikUnknown, score: 0.39
Chn2Unknown, score: 0.52
Nol7Unknown, score: 0.3
Igsf23Unknown, score: 0.35
Yars2Unknown, score: 0.36
Mllt3Unknown, score: 0.29
Vps36Unknown, score: 0.35
Tmem143Unknown, score: 0.29
Gcc2Unknown, score: 0.28
Basp1Unknown, score: 0.27
Kcnip1Unknown, score: 0.43
Bag5Unknown, score: 0.52
Ica1lUnknown, score: 0.44
Polr3fUnknown, score: 0.3
Stk26Unknown, score: 0.4
2610034B18RikUnknown, score: 0.27
Taf15Unknown, score: 0.55
Wdr77Unknown, score: 0.31
Rprd1bUnknown, score: 0.39
Slc35d2Unknown, score: 0.39
DdoUnknown, score: 0.49
Btf3l4Unknown, score: 0.53
Zcchc8Unknown, score: 0.32
Gpr137cUnknown, score: 0.7
Angptl6Unknown, score: 0.35
Rasgef1aUnknown, score: 0.26
CgnUnknown, score: 0.49
Hacd2Unknown, score: 0.26
KynuUnknown, score: 0.34
Zdhhc1Unknown, score: 0.35
Ankib1Unknown, score: 0.51
Hmgxb4Unknown, score: 0.56
Eva1cUnknown, score: 0.36
Stox2Unknown, score: 0.27
UvssaUnknown, score: 0.6
Clec12bUnknown, score: 0.42
Otud1Unknown, score: 0.79
Dlg5Unknown, score: 0.85
Tbc1d9Unknown, score: 0.3
Riok1Unknown, score: 0.3
Arid5bUnknown, score: 0.28
Arhgap21Unknown, score: 0.3
Ppp6r2Unknown, score: 0.3
Alpk1Unknown, score: 0.48
GrapUnknown, score: 0.46
Fbxo9Unknown, score: 0.52
Arhgap42Unknown, score: 0.32
Izumo4Unknown, score: 0.35
Senp8Unknown, score: 0.28
Myo1eUnknown, score: 0.26
OptnUnknown, score: 0.29
4930506M07RikUnknown, score: 0.31
Cdc5lUnknown, score: 0.34
Armcx3Unknown, score: 0.27
Mus81Unknown, score: 0.44
Vps11Unknown, score: 0.62
Mamdc2Unknown, score: 0.26
Isyna1Unknown, score: 0.29
Ankle2Unknown, score: 0.41
Mtfr2Unknown, score: 0.26
Kif23Unknown, score: 0.38
Phf7Unknown, score: 0.26
Osgin1Unknown, score: 0.58
CenpuUnknown, score: 0.32
Mad2l2Unknown, score: 0.27
Noxo1Unknown, score: 0.88
Ppapdc1bUnknown, score: 0.61
Tmem79Unknown, score: 0.35
Tmem123Unknown, score: 0.33
Ephx3Unknown, score: 0.48
Cars2Unknown, score: 0.35
Tom1l1Unknown, score: 0.49
Ddx28Unknown, score: 0.31
Ercc8Unknown, score: 0.32
Alkbh4Unknown, score: 0.34
Cotl1Unknown, score: 0.68
Slc38a10Unknown, score: 0.32
Phf10Unknown, score: 0.55
Naa50Unknown, score: 0.38
2610008E11RikUnknown, score: 0.26
Trub1Unknown, score: 0.54
AdpgkUnknown, score: 0.29
Aifm3Unknown, score: 0.38
Fbxl20Unknown, score: 0.4
Zfp558Unknown, score: 0.27
CdaUnknown, score: 0.71
Smim24Unknown, score: 0.5
Lsm11Unknown, score: 0.73
Plxdc1Unknown, score: 0.3
Vps9d1Unknown, score: 0.61
Amer1Unknown, score: 0.46
Dusp3Unknown, score: 0.31
Zc2hc1cUnknown, score: 0.32
Slc43a1Unknown, score: 0.38
Ttc30bUnknown, score: 0.26
Atxn7l2Unknown, score: 0.33
Pgam5Unknown, score: 0.44
Zfp566Unknown, score: 0.38
Bbs5Unknown, score: 0.38
Cul4bUnknown, score: 0.27
Hspa12bUnknown, score: 0.4
2700094K13RikUnknown, score: 0.39
Fam76bUnknown, score: 0.37
PhykplUnknown, score: 0.48
Ccnt2Unknown, score: 0.39
Insig2Unknown, score: 0.27
Prr36Unknown, score: 0.63
Tgfbrap1Unknown, score: 0.57
Atat1Unknown, score: 0.26
1700040L02RikUnknown, score: 0.27
Lrrc69Unknown, score: 0.34
NptxrUnknown, score: 0.33
Col20a1Unknown, score: 0.47
Cybrd1Unknown, score: 0.35
Bloc1s2Unknown, score: 0.35
Ndufaf7Unknown, score: 0.46
Gucy2gUnknown, score: 0.43
Mvb12aUnknown, score: 0.71
1110008P14RikUnknown, score: 0.28
Man2c1Unknown, score: 0.4
Thap1Unknown, score: 0.32
Poldip3Unknown, score: 0.3
Arhgap18Unknown, score: 0.38
Irak3Unknown, score: 0.31
Ttf2Unknown, score: 0.27
4632428N05RikUnknown, score: 0.51
Tjap1Unknown, score: 0.37
NeblUnknown, score: 0.37
Dcaf6Unknown, score: 0.28
Rbm19Unknown, score: 0.49
CrotUnknown, score: 0.59
AcoxlUnknown, score: 0.38
Syvn1Unknown, score: 0.71
Tm9sf1Unknown, score: 0.31
Robo4Unknown, score: 0.29
Slc35f5Unknown, score: 0.42
Cmtr1Unknown, score: 0.31
Zdhhc16Unknown, score: 0.7
Muc5bUnknown, score: 0.39
Fblim1Unknown, score: 0.35
Xpo6Unknown, score: 0.41
Smu1Unknown, score: 0.37
HnrnprUnknown, score: 0.46
RptorUnknown, score: 0.53
Rmi1Unknown, score: 0.3
Lrriq3Unknown, score: 0.27
Clvs1Unknown, score: 0.64
CmipUnknown, score: 0.74
Arl13aUnknown, score: 0.3
4933433C11RikUnknown, score: 0.3
Fam53aUnknown, score: 0.38
Rasgef1cUnknown, score: 0.37
Kbtbd12Unknown, score: 0.38
Scrn3Unknown, score: 0.43
Ptchd3Unknown, score: 0.38
Rab3il1Unknown, score: 0.56
Ppa2Unknown, score: 0.34
Ccdc181Unknown, score: 0.28
Armc4Unknown, score: 0.41
Fam63aUnknown, score: 0.43
Rnase10Unknown, score: 0.33
Kif27Unknown, score: 0.29
4930505A04RikUnknown, score: 0.41
Tmem180Unknown, score: 0.35
Ccdc146Unknown, score: 0.46
Cep128Unknown, score: 0.84
Dpp3Unknown, score: 0.34
Fgfr1opUnknown, score: 0.29
4930563D23RikUnknown, score: 0.32
Tti1Unknown, score: 0.71
Igfbpl1Unknown, score: 0.85
Anapc15Unknown, score: 0.3
Ttc32Unknown, score: 0.43
Lyrm7Unknown, score: 0.71
1700019G17RikUnknown, score: 0.27
Spaca3Unknown, score: 0.31
Spata25Unknown, score: 0.32
Ccdc64Unknown, score: 0.28
Cldn22Unknown, score: 0.28
Fam65aUnknown, score: 0.31
Morc4Unknown, score: 0.43
Slx1bUnknown, score: 0.3
DcstampUnknown, score: 0.31
Wdr64Unknown, score: 0.66
Fam184aUnknown, score: 0.4
Srrm2Unknown, score: 0.38
Trappc8Unknown, score: 0.37
75973Unknown, score: 0.35
Ccdc125Unknown, score: 0.43
Arhgap15Unknown, score: 0.46
Depdc1aUnknown, score: 0.29
Ppp1r14cUnknown, score: 0.34
Usp31Unknown, score: 0.29
Atp6v0e2Unknown, score: 0.46
Slc38a3Unknown, score: 0.41
GptUnknown, score: 0.72
Asb5Unknown, score: 0.38
TgdsUnknown, score: 0.7
1700019B03RikUnknown, score: 0.39
1700028K03RikUnknown, score: 0.26
Ppp1r18Unknown, score: 0.58
Ccdc134Unknown, score: 0.28
Msrb2Unknown, score: 0.37
Cmya5Unknown, score: 0.33
Msi2Unknown, score: 0.37
Wdr38Unknown, score: 0.49
Srxn1Unknown, score: 0.31
Ppfia3Unknown, score: 0.46
2410137M14RikUnknown, score: 0.31
NubplUnknown, score: 0.45
Catsper3Unknown, score: 0.4
1700015E13RikUnknown, score: 0.51
Bcas1Unknown, score: 0.94
Slc9a8Unknown, score: 0.3
Ints7Unknown, score: 0.26
Zfp934Unknown, score: 0.65
NkrfUnknown, score: 0.27
C330018D20RikUnknown, score: 0.46
Zfp266Unknown, score: 0.54
Vps33aUnknown, score: 0.3
Rnf170Unknown, score: 0.29
Sbf1Unknown, score: 0.39
Mccc2Unknown, score: 0.41
Cox7b2Unknown, score: 0.44
Phf23Unknown, score: 0.33
Nxpe2Unknown, score: 0.47
Srbd1Unknown, score: 0.36
Pde6hUnknown, score: 0.27
CstadUnknown, score: 0.37
TroapUnknown, score: 0.58
Filip1lUnknown, score: 0.28
Zc3h6Unknown, score: 0.3
Galnt15Unknown, score: 0.73
Efcab11Unknown, score: 0.55
Hhipl2Unknown, score: 0.81
Stpg1Unknown, score: 0.5
Cnot10Unknown, score: 0.42
Pus7lUnknown, score: 0.46
MispUnknown, score: 0.78
Sp2Unknown, score: 0.27
9130019O22RikUnknown, score: 0.27
Srd5a1Unknown, score: 0.48
Ern1Unknown, score: 0.5
Trim39Unknown, score: 0.3
Ublcp1Unknown, score: 0.32
Bcl9lUnknown, score: 0.28
ZxdcUnknown, score: 0.27
Pacsin3Unknown, score: 0.44
Clca3a2Unknown, score: 0.3
Fgf20Unknown, score: 0.54
Dhx58Unknown, score: 0.26
LrbaUnknown, score: 0.73
Ntng1Unknown, score: 0.73
Fgf16Unknown, score: 0.29
LactbUnknown, score: 0.77
Gatsl2Unknown, score: 0.33
Gpr84Unknown, score: 0.36
CemipUnknown, score: 0.28
Jdp2Unknown, score: 0.58
Sorcs2Unknown, score: 0.43
Sp6Unknown, score: 0.46
Glis2Unknown, score: 0.41
Trem2Unknown, score: 0.49
Nxf2Unknown, score: 0.27
Sacm1lUnknown, score: 0.34
Cnnm1Unknown, score: 0.32
Pde4dipUnknown, score: 0.37
Slc25a2Unknown, score: 0.6
Gpr137bUnknown, score: 0.56
SlmapUnknown, score: 0.35
Pi4k2aUnknown, score: 0.26
Rnf123Unknown, score: 0.46
Tnfrsf25Unknown, score: 0.58
Trim8Unknown, score: 0.29
GlceUnknown, score: 0.41
Sep15Unknown, score: 0.38
Clec2dUnknown, score: 0.54
Lztfl1Unknown, score: 0.37
Wnt16Unknown, score: 0.35
Arid1aUnknown, score: 0.27
Igsf9Unknown, score: 0.31
Pcdhb2Unknown, score: 0.26
Pcdhb8Unknown, score: 0.42
Pcdhb13Unknown, score: 0.46
Pcdhb15Unknown, score: 0.46
Pcdhb16Unknown, score: 0.34
Pcdhb17Unknown, score: 0.36
Pcdhb18Unknown, score: 0.4
Pcdhb19Unknown, score: 0.4
Mrpl3Unknown, score: 0.32
Strn3Unknown, score: 0.37
Adarb2Unknown, score: 0.5
Cnnm4Unknown, score: 0.33
S1pr5Unknown, score: 0.46
Sfxn2Unknown, score: 0.29
PrccUnknown, score: 0.46
Tmem40Unknown, score: 0.6
Hmgn3Unknown, score: 0.65
TprnUnknown, score: 0.57
Wwtr1Unknown, score: 0.28
Hist2h4Unknown, score: 0.3
Strn4Unknown, score: 0.27
QarsUnknown, score: 0.35
C78339Unknown, score: 0.49
Gtf2f1Unknown, score: 0.27
Txndc9Unknown, score: 0.35
D2hgdhUnknown, score: 0.42
Slc41a1Unknown, score: 0.34
Gtf3c3Unknown, score: 0.3
AI182371Unknown, score: 0.33
Ehd4Unknown, score: 0.4
Usp6nlUnknown, score: 0.55
Mrps26Unknown, score: 0.39
Stard7Unknown, score: 0.61
Garnl3Unknown, score: 0.27
Golga2Unknown, score: 0.62
Magi3Unknown, score: 0.28
Wdr47Unknown, score: 0.39
Usp53Unknown, score: 0.51
Kcnc4Unknown, score: 0.4
Tmem56Unknown, score: 0.27
Ifi44Unknown, score: 0.42
Tyw1Unknown, score: 0.38
Fbxl14Unknown, score: 0.36
Phrf1Unknown, score: 0.58
Inpp5fUnknown, score: 0.32
Ccp110Unknown, score: 0.3
Ano1Unknown, score: 0.28
AW146154Unknown, score: 0.36
Fam192aUnknown, score: 0.42
Zdhhc7Unknown, score: 0.32
Cpne7Unknown, score: 0.32
Lars2Unknown, score: 0.46
Snx19Unknown, score: 0.71
Rpp25Unknown, score: 0.49
Acad11Unknown, score: 0.28
Nudt10Unknown, score: 0.36
Chchd10Unknown, score: 0.47
Nup107Unknown, score: 0.54
Slc25a41Unknown, score: 0.51
Nxph4Unknown, score: 0.28
Adcy4Unknown, score: 0.3
Ces1dUnknown, score: 0.39
Cdc42ep2Unknown, score: 0.59
RprmlUnknown, score: 0.33
MycbpapUnknown, score: 0.33
Vipas39Unknown, score: 0.4
Tmem179Unknown, score: 0.46
Fam110cUnknown, score: 0.38
IarsUnknown, score: 0.63
Nhlrc1Unknown, score: 0.29
Abhd4Unknown, score: 0.3
Ankrd28Unknown, score: 0.5
Mycbp2Unknown, score: 0.59
ScribUnknown, score: 0.36
Mal2Unknown, score: 0.34
Mfsd5Unknown, score: 0.29
SrlUnknown, score: 0.71
Stk38Unknown, score: 0.32
G6bUnknown, score: 0.32
Arap3Unknown, score: 0.42
Guca1bUnknown, score: 0.37
Ssr1Unknown, score: 0.35
Il1rl2Unknown, score: 0.37
Nt5c3Unknown, score: 0.68
Mrpl30Unknown, score: 0.4
Jmjd6Unknown, score: 0.33
Ddb2Unknown, score: 0.75
Bfsp2Unknown, score: 0.28
Gtpbp6Unknown, score: 0.58
Ap4e1Unknown, score: 0.73
Eif2b3Unknown, score: 0.34
Skiv2lUnknown, score: 0.5
Prkag2Unknown, score: 0.45
Baiap2Unknown, score: 0.27
Slco4a1Unknown, score: 0.28
AticUnknown, score: 0.31
Galnt7Unknown, score: 0.54
Ubxn8Unknown, score: 0.28
Dnajc9Unknown, score: 0.47
Gpt2Unknown, score: 0.31
Lyrm2Unknown, score: 0.32
Mex3bUnknown, score: 0.34
Ccdc122Unknown, score: 0.38
Atad3aUnknown, score: 0.29
Nusap1Unknown, score: 0.32
PrkcdbpUnknown, score: 0.27
Sephs1Unknown, score: 0.32
Gins4Unknown, score: 0.29
Ube2q2Unknown, score: 0.27
Rarres1Unknown, score: 0.64
SccpdhUnknown, score: 0.28
Orai1Unknown, score: 0.6
Fam163bUnknown, score: 0.32
DspUnknown, score: 0.38
Hyal3Unknown, score: 0.43
Cpa1Unknown, score: 0.44
Itga1Unknown, score: 0.41
MaobUnknown, score: 0.62
AslUnknown, score: 0.35
Zbtb25Unknown, score: 0.28
Art3Unknown, score: 0.55
Tpgs1Unknown, score: 0.31
Kif22Unknown, score: 0.3
DekUnknown, score: 0.28
DutUnknown, score: 0.48
110083Unknown, score: 0.26
GgctUnknown, score: 0.28
Akr7a5Unknown, score: 0.36
MsraUnknown, score: 0.54
Pp2d1Unknown, score: 0.37
Dync2h1Unknown, score: 0.28
Grik4Unknown, score: 0.45
Prps2Unknown, score: 0.35
Aldh7a1Unknown, score: 0.26
EtfbUnknown, score: 0.3
Slc8a2Unknown, score: 0.32
Cds2Unknown, score: 0.4
Egln2Unknown, score: 0.49
Laptm4bUnknown, score: 0.35
Dok4Unknown, score: 0.31
Zbtb21Unknown, score: 0.58
Crip3Unknown, score: 0.54
Rpl31Unknown, score: 0.35
Rasa2Unknown, score: 0.37
Spred2Unknown, score: 0.34
Ddhd1Unknown, score: 0.34
Rims2Unknown, score: 0.33
Derl2Unknown, score: 0.5
Pop5Unknown, score: 0.27
Ivns1abpUnknown, score: 0.28
Asb10Unknown, score: 0.32
Gjc2Unknown, score: 0.36
Igf2bp3Unknown, score: 0.34
Kcnn2Unknown, score: 0.46
Kcnn3Unknown, score: 0.56
Atp6v0a4Unknown, score: 0.54
AF251705Unknown, score: 0.32
Smc1bUnknown, score: 0.33
Plxnb3Unknown, score: 0.35
Elmo2Unknown, score: 0.36
Ubox5Unknown, score: 0.5
Ube4aUnknown, score: 0.27
Sesn1Unknown, score: 0.39
Il25Unknown, score: 0.27
Nek8Unknown, score: 0.29
Asb13Unknown, score: 0.3
Tlr3Unknown, score: 0.36
Olfr78Unknown, score: 0.55
KirrelUnknown, score: 0.57
Oxr1Unknown, score: 0.28
Tlr7Unknown, score: 0.36
Pdzd3Unknown, score: 0.35
Nup155Unknown, score: 0.29
Hdac10Unknown, score: 0.54
Rbm39Unknown, score: 0.27
Ppargc1bUnknown, score: 0.81
Grid2ipUnknown, score: 0.26
Ntng2Unknown, score: 0.37
Timd2Unknown, score: 0.4
Havcr2Unknown, score: 0.39
Slc12a8Unknown, score: 0.37
Cd99l2Unknown, score: 0.47
BmfUnknown, score: 0.26
MvdUnknown, score: 0.66
Rwdd4aUnknown, score: 0.32
Stab2Unknown, score: 0.46
Bcas3Unknown, score: 0.86
Slc25a36Unknown, score: 0.35
Ripk2Unknown, score: 0.36
Rapgef6Unknown, score: 0.53
Itgb4Unknown, score: 0.35
Dhrs11Unknown, score: 0.28
Cyb5d2Unknown, score: 0.58
Zbtb12Unknown, score: 0.37
Eme2Unknown, score: 0.29
Tet3Unknown, score: 0.3
Sun3Unknown, score: 0.28
Nlrp1aUnknown, score: 0.34
Fbxo40Unknown, score: 0.33
Fam120cUnknown, score: 0.45
Dtx4Unknown, score: 0.38
Igsf11Unknown, score: 0.36
Bzrap1Unknown, score: 0.29
Gramd1cUnknown, score: 0.37
4930539E08RikUnknown, score: 0.33
VcpkmtUnknown, score: 0.3
Phldb2Unknown, score: 0.26
Cyp4f17Unknown, score: 0.39
Shroom4Unknown, score: 0.46
Dip2cUnknown, score: 0.28
Alg3Unknown, score: 0.26
Creb3l2Unknown, score: 0.49
Eif5a2Unknown, score: 0.26
Hdac4Unknown, score: 0.61
Sned1Unknown, score: 0.26
Daam1Unknown, score: 0.59
Myo5cUnknown, score: 0.36
Pycr1Unknown, score: 0.39
Gipc3Unknown, score: 0.28
Taf3Unknown, score: 0.52
Hsh2dUnknown, score: 0.34
MtrrUnknown, score: 0.34
D130043K22RikUnknown, score: 0.33
Shank2Unknown, score: 0.34
Dock10Unknown, score: 0.42
Mtmr9Unknown, score: 0.29
Mettl14Unknown, score: 0.63
Coq10aUnknown, score: 0.63
Gab3Unknown, score: 0.3
ThemisUnknown, score: 0.27
SepsecsUnknown, score: 0.26
Kbtbd7Unknown, score: 0.28
Nrg1Unknown, score: 0.47
Exoc3Unknown, score: 0.42
Mfsd9Unknown, score: 0.26
Depdc7Unknown, score: 0.54
Plekhh1Unknown, score: 0.57
Inpp5aUnknown, score: 0.35
Zswim4Unknown, score: 0.37
Klhl32Unknown, score: 0.38
Spice1Unknown, score: 0.28
Sh3bgrl2Unknown, score: 0.34
Rsph4aUnknown, score: 0.38
TifabUnknown, score: 0.57
Gabpb2Unknown, score: 0.33
Tmem71Unknown, score: 0.48
Il20rbUnknown, score: 0.26
Fstl5Unknown, score: 0.81
Mylk3Unknown, score: 0.81
Rbbp5Unknown, score: 0.27
Plekhg6Unknown, score: 0.48
Cracr2bUnknown, score: 0.38
9530068E07RikUnknown, score: 0.66
Casd1Unknown, score: 0.5
Col28a1Unknown, score: 0.31
Trim38Unknown, score: 0.27
Pak6Unknown, score: 0.35
Disp2Unknown, score: 0.29
CrygnUnknown, score: 0.42
MyocdUnknown, score: 0.37
Parp16Unknown, score: 0.76
Tmprss4Unknown, score: 0.62
Tmprss13Unknown, score: 0.31
Duox2Unknown, score: 0.34
Sidt2Unknown, score: 0.3
Slc10a3Unknown, score: 0.3
Zfp879Unknown, score: 0.45
Sertad4Unknown, score: 0.26
Neurl3Unknown, score: 0.36
Slc39a2Unknown, score: 0.52
Trim50Unknown, score: 0.7
Il1f9Unknown, score: 0.3
Psd4Unknown, score: 0.49
Ctnna3Unknown, score: 0.48
Ube2d1Unknown, score: 0.48
IlvblUnknown, score: 0.78
PolrmtUnknown, score: 0.28
Tmem259Unknown, score: 0.28
Tspan8Unknown, score: 0.37
Os9Unknown, score: 0.3
LgalslUnknown, score: 0.34
Rufy1Unknown, score: 0.81
Acsl6Unknown, score: 0.31
Wnt9aUnknown, score: 0.29
Tom1l2Unknown, score: 0.45
CntrobUnknown, score: 0.5
Wrap53Unknown, score: 0.38
Nlgn2Unknown, score: 0.42
Wscd1Unknown, score: 0.47
Arl5cUnknown, score: 0.52
Fam171a2Unknown, score: 0.28
Cd300aUnknown, score: 0.61
Cd300lbUnknown, score: 0.32
UnkUnknown, score: 0.35
Tnrc6cUnknown, score: 0.4
EngaseUnknown, score: 0.26
Trib2Unknown, score: 0.59
G2e3Unknown, score: 0.79
Mlh3Unknown, score: 0.68
Nek9Unknown, score: 0.3
Cdc42bpbUnknown, score: 0.48
RfesdUnknown, score: 0.37
Serinc5Unknown, score: 0.41
Pde8bUnknown, score: 0.39
Dhx29Unknown, score: 0.29
3830406C13RikUnknown, score: 0.38
Zfp503Unknown, score: 0.49
Mtmr6Unknown, score: 0.54
Spata13Unknown, score: 0.29
Arl11Unknown, score: 0.56
Hmbox1Unknown, score: 0.76
Pcdh20Unknown, score: 0.41
GgactUnknown, score: 0.67
TrioUnknown, score: 0.42
NaprtUnknown, score: 0.39
Gramd4Unknown, score: 0.69
Brd1Unknown, score: 0.4
Senp1Unknown, score: 0.26
Cpped1Unknown, score: 0.27
Marf1Unknown, score: 0.39
Slc7a4Unknown, score: 0.33
D17Wsu92eUnknown, score: 0.63
Adgrf5Unknown, score: 0.26
Dlgap1Unknown, score: 0.27
Thoc1Unknown, score: 0.36
Ankrd29Unknown, score: 0.37
Ino80cUnknown, score: 0.27
Pik3c3Unknown, score: 0.28
Wdr36Unknown, score: 0.38
Fam13bUnknown, score: 0.46
Rbm27Unknown, score: 0.36
Onecut2Unknown, score: 0.49
Cep76Unknown, score: 0.61
Pla2g16Unknown, score: 0.39
Snx32Unknown, score: 0.27
Rin1Unknown, score: 0.33
Trpm3Unknown, score: 0.52
Dmrt2Unknown, score: 0.29
Ermp1Unknown, score: 0.33
Hectd2Unknown, score: 0.57
Fam160b1Unknown, score: 0.63
DarsUnknown, score: 0.32
Yod1Unknown, score: 0.27
Dyrk3Unknown, score: 0.41
Zfp281Unknown, score: 0.51
Nmnat2Unknown, score: 0.26
Aph1aUnknown, score: 0.4
Ndufs2Unknown, score: 0.31
Cdc42bpaUnknown, score: 0.29
Ppp2r5aUnknown, score: 0.46
SbsponUnknown, score: 0.4
Ndufs1Unknown, score: 0.31
Gpr55Unknown, score: 0.51
Prrc2bUnknown, score: 0.47
Slc25a25Unknown, score: 0.28
GcaUnknown, score: 0.3
AgpsUnknown, score: 0.27
1110051M20RikUnknown, score: 0.26
Kif18aUnknown, score: 0.53
Ano3Unknown, score: 0.31
Arhgap11aUnknown, score: 0.41
Bahd1Unknown, score: 0.36
Vps18Unknown, score: 0.27
6820408C15RikUnknown, score: 0.48
Asxl1Unknown, score: 0.28
Slc35c2Unknown, score: 0.3
Slc17a9Unknown, score: 0.26
Eif2aUnknown, score: 0.34
Gpr171Unknown, score: 0.27
D930015E06RikUnknown, score: 0.46
Isg20l2Unknown, score: 0.43
Gatad2bUnknown, score: 0.27
CiartUnknown, score: 0.36
Rbm15Unknown, score: 0.29
Gpr61Unknown, score: 0.43
Clcc1Unknown, score: 0.44
Trmt13Unknown, score: 0.3
Ccbl2Unknown, score: 0.28
Frrs1lUnknown, score: 0.39
6330416G13RikUnknown, score: 0.38
Haus6Unknown, score: 0.31
Cyb5rlUnknown, score: 0.31
Glis1Unknown, score: 0.35
Foxj3Unknown, score: 0.33
Pdik1lUnknown, score: 0.26
Agbl5Unknown, score: 0.26
Sh3tc1Unknown, score: 0.38
Sel1l3Unknown, score: 0.35
Slc10a4Unknown, score: 0.45
AasdhUnknown, score: 0.52
Sdad1Unknown, score: 0.34
Tmem150cUnknown, score: 0.3
Arhgap24Unknown, score: 0.29
FicdUnknown, score: 0.68
Ssh1Unknown, score: 0.27
Alkbh2Unknown, score: 0.3
MepceUnknown, score: 0.42
Ap5z1Unknown, score: 0.38
Jazf1Unknown, score: 0.27
Creb5Unknown, score: 0.26
Plekha8Unknown, score: 0.39
Eva1aUnknown, score: 0.38
Fgd5Unknown, score: 0.61
Frmd4bUnknown, score: 0.58
Zfp637Unknown, score: 0.29
Ankrd26Unknown, score: 0.45
Clstn3Unknown, score: 0.32
Clec12aUnknown, score: 0.27
Crebl2Unknown, score: 0.32
Gys2Unknown, score: 0.64
Caprin2Unknown, score: 0.3
Amn1Unknown, score: 0.48
Zc3hc1Unknown, score: 0.42
MgamUnknown, score: 0.27
Zfp772Unknown, score: 0.32
Phldb3Unknown, score: 0.28
Tubgcp5Unknown, score: 0.27
Mtmr10Unknown, score: 0.32
Vps33bUnknown, score: 0.39
Tmc3Unknown, score: 0.44
CrebzfUnknown, score: 0.31
Kctd14Unknown, score: 0.36
2210018M11RikUnknown, score: 0.26
Pgap2Unknown, score: 0.49
Plekha7Unknown, score: 0.3
Thumpd1Unknown, score: 0.51
Gtf3c1Unknown, score: 0.5
Prr14Unknown, score: 0.51
Arhgef10Unknown, score: 0.28
Fgl1Unknown, score: 0.26
Naf1Unknown, score: 0.49
Gatad2aUnknown, score: 0.43
D230025D16RikUnknown, score: 0.31
Ddx19bUnknown, score: 0.28
Klhl36Unknown, score: 0.48
Ccdc67Unknown, score: 0.44
Msantd2Unknown, score: 0.63
Tmem136Unknown, score: 0.37
DlatUnknown, score: 0.4
Dmxl2Unknown, score: 0.48
HykkUnknown, score: 0.37
Snx33Unknown, score: 0.78
Usp3Unknown, score: 0.51
Gk5Unknown, score: 0.34
Acaa1bUnknown, score: 0.29
Zfp709Unknown, score: 0.39
Pyhin1Unknown, score: 0.41
NyxUnknown, score: 0.57
Mmgt1Unknown, score: 0.48
Pcyt1bUnknown, score: 0.63
Tceal1Unknown, score: 0.26
Gnl3lUnknown, score: 0.46
Ric8bUnknown, score: 0.6
Rassf9Unknown, score: 0.32
Mief2Unknown, score: 0.3
PhbUnknown, score: 0.27
Slfn9Unknown, score: 0.57
Usp32Unknown, score: 0.31
Zdhhc22Unknown, score: 0.37
Slc24a4Unknown, score: 0.54
Serpina3fUnknown, score: 0.26
Serpinb6dUnknown, score: 0.5
Gpr150Unknown, score: 0.27
Ppwd1Unknown, score: 0.59
OgdhlUnknown, score: 0.35
HomezUnknown, score: 0.41
Slitrk6Unknown, score: 0.42
Card6Unknown, score: 0.58
Adamts12Unknown, score: 0.4
Tspyl5Unknown, score: 0.39
AardUnknown, score: 0.35
Fam83aUnknown, score: 0.41
Cacna1iUnknown, score: 0.29
239611Unknown, score: 0.63
Dip2bUnknown, score: 0.33
Ndufa11Unknown, score: 0.36
Gpr156Unknown, score: 0.27
Pnldc1Unknown, score: 0.3
Neurl1bUnknown, score: 0.42
Morc2bUnknown, score: 0.31
Fem1cUnknown, score: 0.53
Dmxl1Unknown, score: 0.6
F830016B08RikUnknown, score: 0.44
Malt1Unknown, score: 0.34
Kcng2Unknown, score: 0.26
Cdc42bpgUnknown, score: 0.28
Sec31bUnknown, score: 0.76
Mcmdc2Unknown, score: 0.83
Sulf1Unknown, score: 0.55
Klhl12Unknown, score: 0.56
Zbtb37Unknown, score: 0.45
Scyl3Unknown, score: 0.4
Dusp27Unknown, score: 0.81
Lrrc52Unknown, score: 0.38
Itga8Unknown, score: 0.54
St8sia6Unknown, score: 0.42
Xirp2Unknown, score: 0.62
Cers6Unknown, score: 0.51
Zfp804aUnknown, score: 0.29
241621Unknown, score: 0.35
Wdr76Unknown, score: 0.48
Ccdc144bUnknown, score: 0.27
D3Ertd254eUnknown, score: 0.3
Bbs12Unknown, score: 0.26
Frem2Unknown, score: 0.66
Igsf10Unknown, score: 0.27
Slc22a15Unknown, score: 0.28
Gdf6Unknown, score: 0.45
Gabbr2Unknown, score: 0.42
Grin3aUnknown, score: 0.48
Frmd3Unknown, score: 0.46
Dmrta1Unknown, score: 0.31
Dlgap3Unknown, score: 0.26
Rsbn1lUnknown, score: 0.45
Fbxl5Unknown, score: 0.65
Kctd8Unknown, score: 0.29
Sbno1Unknown, score: 0.51
Grip2Unknown, score: 0.53
Kbtbd8Unknown, score: 0.43
GgnUnknown, score: 0.64
Hspb6Unknown, score: 0.27
Rgs9bpUnknown, score: 0.26
Nars2Unknown, score: 0.38
Trim30bUnknown, score: 0.44
Tnfrsf26Unknown, score: 0.35
Lonrf1Unknown, score: 0.36
Tox3Unknown, score: 0.36
HydinUnknown, score: 0.31
Kdm4dUnknown, score: 0.51
Glb1l2Unknown, score: 0.4
Ankk1Unknown, score: 0.58
Arhgap20Unknown, score: 0.27
Zc3h12cUnknown, score: 0.27
Dclk3Unknown, score: 0.36
MyripUnknown, score: 0.38
Dcaf12l1Unknown, score: 0.59
Zc4h2Unknown, score: 0.36
C77370Unknown, score: 0.3
Kir3dl1Unknown, score: 0.39
Fam199xUnknown, score: 0.32
Mum1l1Unknown, score: 0.45
Amdhd2Unknown, score: 0.41
Atg9aUnknown, score: 0.29
Ccdc15Unknown, score: 0.31
RttnUnknown, score: 0.39
Kcne2Unknown, score: 0.29
Myo1gUnknown, score: 0.26
BivmUnknown, score: 0.4
Prokr2Unknown, score: 0.4
Dnajc28Unknown, score: 0.3
Obox6Unknown, score: 0.41
Ropn1lUnknown, score: 0.29
Tpcn1Unknown, score: 0.46
Olfr543Unknown, score: 0.26
Olfr1384Unknown, score: 0.3
Olfr691Unknown, score: 0.27
Ehd2Unknown, score: 0.42
Cadm4Unknown, score: 0.28
Gga3Unknown, score: 0.4
Plekhg3Unknown, score: 0.39
Cdk8Unknown, score: 0.57
Ly6g5bUnknown, score: 0.32
Zbtb24Unknown, score: 0.44
Scml4Unknown, score: 0.34
MpgUnknown, score: 0.43
Alkbh5Unknown, score: 0.31
Bahcc1Unknown, score: 0.39
Sntg2Unknown, score: 0.29
Zswim8Unknown, score: 0.3
Rnf31Unknown, score: 0.34
Xxylt1Unknown, score: 0.28
Gpr152Unknown, score: 0.27
Cpsf7Unknown, score: 0.39
Nup54Unknown, score: 0.56
Kif26bUnknown, score: 0.38
Nbeal1Unknown, score: 0.39
Rtn4rl2Unknown, score: 0.43
Plch1Unknown, score: 0.28
VcpUnknown, score: 0.43
Gpr157Unknown, score: 0.39
Pank4Unknown, score: 0.4
Zfp384Unknown, score: 0.52
Pon3Unknown, score: 0.47
Tspan12Unknown, score: 0.39
Nat14Unknown, score: 0.68
Vmn2r57Unknown, score: 0.28
Rccd1Unknown, score: 0.45
Adamtsl3Unknown, score: 0.28
Nup98Unknown, score: 0.36
GcdhUnknown, score: 0.42
Nlrx1Unknown, score: 0.38
Klhl18Unknown, score: 0.35
Taf1Unknown, score: 0.34
Mbtps2Unknown, score: 0.37
BC048403Unknown, score: 0.27
4933408B17RikUnknown, score: 0.31
C2cd4dUnknown, score: 0.28
Eif5aUnknown, score: 0.36
Slfn8Unknown, score: 0.26
Rasl10bUnknown, score: 0.28
Marveld1Unknown, score: 0.61
NynrinUnknown, score: 0.36
Prex1Unknown, score: 0.42
Vstm2lUnknown, score: 0.43
Fam131cUnknown, score: 0.46
Exoc3lUnknown, score: 0.26
Wfikkn2Unknown, score: 0.91
Kctd19Unknown, score: 0.41
Pcdh19Unknown, score: 0.38
SbsnUnknown, score: 0.32
Gpr4Unknown, score: 0.42
A230050P20RikUnknown, score: 0.79
C5ar2Unknown, score: 0.6
Dpep2Unknown, score: 0.4
Lrtm1Unknown, score: 0.62
Cxxc4Unknown, score: 0.48
Wdr59Unknown, score: 0.34
A430078G23RikUnknown, score: 0.3
6430573F11RikUnknown, score: 0.26
Zfp944Unknown, score: 0.29
Efcab5Unknown, score: 0.27
Usp37Unknown, score: 0.36
Podxl2Unknown, score: 0.28
SmoUnknown, score: 0.4
9630033F20RikUnknown, score: 0.3
Dock6Unknown, score: 0.56
Vwc2Unknown, score: 0.36
Flad1Unknown, score: 0.31
Nceh1Unknown, score: 0.79
Exph5Unknown, score: 0.66
6430550D23RikUnknown, score: 0.29
Fndc9Unknown, score: 0.29
DgkiUnknown, score: 0.32
BC049715Unknown, score: 0.32
Ptpn7Unknown, score: 0.34
Zmat4Unknown, score: 0.59
Tacc1Unknown, score: 0.26
Fndc7Unknown, score: 0.28
Hook3Unknown, score: 0.47
Mettl20Unknown, score: 0.36
Ddx11Unknown, score: 0.36
Fubp3Unknown, score: 0.42
ScaiUnknown, score: 0.38
Glt28d2Unknown, score: 0.29
Tmem251Unknown, score: 0.54
FryUnknown, score: 0.47
CenptUnknown, score: 0.41
Alg6Unknown, score: 0.57
P4ha3Unknown, score: 0.57
Ppm1eUnknown, score: 0.27
Lmod3Unknown, score: 0.34
Cachd1Unknown, score: 0.28
Sycp2Unknown, score: 0.42
Dennd5bUnknown, score: 0.57
Idi2Unknown, score: 0.35
Tmem88bUnknown, score: 0.42
Phf8Unknown, score: 0.34
Strip2Unknown, score: 0.29
Snrnp200Unknown, score: 0.39
Zbtb26Unknown, score: 0.32
D5Ertd579eUnknown, score: 0.58
Casc1Unknown, score: 0.26
Tmem117Unknown, score: 0.44
Zhx3Unknown, score: 0.44
Cdh10Unknown, score: 0.42
Mical2Unknown, score: 0.42
Wscd2Unknown, score: 0.47
Xpnpep3Unknown, score: 0.26
Gpr183Unknown, score: 0.54
Cdv3Unknown, score: 0.41
Tnfsf15Unknown, score: 0.3
Trmt61aUnknown, score: 0.37
9830107B12RikUnknown, score: 0.35
Arhgef37Unknown, score: 0.33
Ankrd44Unknown, score: 0.34
Zfp335Unknown, score: 0.3
Aknad1Unknown, score: 0.45
PigkUnknown, score: 0.37
Frem1Unknown, score: 0.3
Foxo6Unknown, score: 0.38
Adamts3Unknown, score: 0.39
Gal3st4Unknown, score: 0.53
Sdk1Unknown, score: 0.51
Pon2Unknown, score: 0.4
Thsd7aUnknown, score: 0.31
Ctxn1Unknown, score: 0.55
Zfp866Unknown, score: 0.4
Slc7a6Unknown, score: 0.26
Dixdc1Unknown, score: 0.84
Rgag4Unknown, score: 0.49
Tfap2eUnknown, score: 0.49
Ppp1r13lUnknown, score: 0.27
Ttc16Unknown, score: 0.26
Supv3l1Unknown, score: 0.34
Trim65Unknown, score: 0.6
Myo1dUnknown, score: 0.29
Fa2hUnknown, score: 0.28
Sfmbt2Unknown, score: 0.53
353328Unknown, score: 0.4
Hist1h3aUnknown, score: 0.37
Zranb1Unknown, score: 0.36
Speer4dUnknown, score: 0.43
Lrrc24Unknown, score: 0.33
IntuUnknown, score: 0.55
NefhUnknown, score: 0.31
Spata22Unknown, score: 0.29
Serpina11Unknown, score: 0.34
BegainUnknown, score: 0.26
Mrs2Unknown, score: 0.61
Lyrm4Unknown, score: 0.47
Zfp395Unknown, score: 0.32
Tmem106cUnknown, score: 0.27
Zfp948Unknown, score: 0.3
Gm21981Unknown, score: 0.52
Prob1Unknown, score: 0.35
Greb1lUnknown, score: 0.41
Lipo1Unknown, score: 0.3
Atp2b4Unknown, score: 0.3
Kif14Unknown, score: 0.55
Zfp408Unknown, score: 0.63
Rbm33Unknown, score: 0.41
Zcwpw1Unknown, score: 0.77
4930590J08RikUnknown, score: 0.27
2700089E24RikUnknown, score: 0.34
ItgadUnknown, score: 0.3
6430531B16RikUnknown, score: 0.31
Foxr1Unknown, score: 0.43
Odf3l1Unknown, score: 0.28
Snx22Unknown, score: 0.42
Zkscan7Unknown, score: 0.41
Brwd3Unknown, score: 0.37
A830080D01RikUnknown, score: 0.33
Atxn7l3bUnknown, score: 0.32
382522Unknown, score: 0.33
Pfn4Unknown, score: 0.28
Rrm2bUnknown, score: 0.28
Ankrd63Unknown, score: 0.58
Slc25a34Unknown, score: 0.29
Fam47eUnknown, score: 0.33
Irs2Unknown, score: 0.39
Scn4bUnknown, score: 0.54
Plcxd1Unknown, score: 0.56
H2afy2Unknown, score: 0.36
Tmem204Unknown, score: 0.55
Gm5431Unknown, score: 0.36
432798Unknown, score: 0.34
Eno1bUnknown, score: 0.26
I830077J02RikUnknown, score: 0.35
433874Unknown, score: 0.29
Ociad2Unknown, score: 0.47
WhammUnknown, score: 0.33
AmtUnknown, score: 0.3
Ccdc160Unknown, score: 0.35
A830005F24RikUnknown, score: 0.29
Zbtb9Unknown, score: 0.28
Armcx5Unknown, score: 0.43
Iqgap2Unknown, score: 0.35
Wdfy4Unknown, score: 0.27
Fam186bUnknown, score: 0.47
545487Unknown, score: 0.43
Gramd2Unknown, score: 0.4
Gm12185Unknown, score: 0.27
Zfp827Unknown, score: 0.39
Lekr1Unknown, score: 0.27
Klk13Unknown, score: 0.55
Lipo4Unknown, score: 0.47
OtoglUnknown, score: 0.45
Ctxn3Unknown, score: 0.28
Cd101Unknown, score: 0.27
631286Unknown, score: 0.26
Erich4Unknown, score: 0.33
634555Unknown, score: 0.28
636901Unknown, score: 0.31
640370Unknown, score: 0.4
Zfp784Unknown, score: 0.31
Gcnt7Unknown, score: 0.36
Isoc2aUnknown, score: 0.32
Gm14326Unknown, score: 0.31
TnikUnknown, score: 0.48
665181Unknown, score: 0.32
666448Unknown, score: 0.63
Gm8369Unknown, score: 0.36
667739Unknown, score: 0.43
667952Unknown, score: 0.33
671641Unknown, score: 0.27
Gm11744Unknown, score: 0.32
Isg15Unknown, score: 0.5
Mup9Unknown, score: 0.29
Mup10Unknown, score: 0.29
Hrct1Unknown, score: 0.3
100039794Unknown, score: 0.48
100041098Unknown, score: 0.26
Gm3194Unknown, score: 0.38
Pdcd5Unknown, score: 0.55
100043217Unknown, score: 0.4
Zfp831Unknown, score: 0.31
100046628Unknown, score: 0.28
100047518Unknown, score: 0.37
100047632Unknown, score: 0.48
100047658Unknown, score: 0.28
Cfap43Unknown, score: 0.79
Ddx43Unknown, score: 0.46
Fam150bUnknown, score: 0.3
Gm17455Unknown, score: 0.33
Ccdc13Unknown, score: 0.61
100503180Unknown, score: 0.34
100503254Unknown, score: 0.35
Ccdc149Unknown, score: 0.42
Cmtm1Unknown, score: 0.26
TmppeUnknown, score: 0.77
100862012Unknown, score: 0.6
RP23-56M18.5Unknown, score: 0.29
Gm21698Unknown, score: 0.59
100862558Unknown, score: 0.28
100862570Unknown, score: 0.38
100862584Unknown, score: 0.27
101055820Unknown, score: 0.38
101056386Unknown, score: 0.26
101056616Unknown, score: 0.3

## Help | Hide | Top Help | Show | Top Conditions

### HELP

Conditions in the module, given in the same order as on the expression
plot above. Red color means over-expression, green under-expression in
the given condition.

The barplot below shows the condition (sample) scores. A separate bar
is shown for each sample, its height is the corresponding score of the
sample in the module. The red and green numbers on the bars are the
sample scores expressed in percents, i.e. 100% is 1.0.

The red and green lines show the module thresholds, samples above
the red line and below the green line are included in the module.

The different experiments that were part of the study, are separated
by dashed vertical lines.

— Click on the *Help* button again to close this help window.

| Id |
| --- |
| A\_J-ATE\_117 |
| BALB\_cByJ-ATE\_157 |
| NOD\_ShiLtJ-ATE\_23 |
| LP\_J-ATE\_98 |
| C57BL\_6J-ATE\_109 |
| NOD\_ShiLtJ-ATE\_27 |
| FVB\_NJ-ATE\_60 |
| SJL\_J-ATE\_18 |
| A\_J-ATE\_116 |
| FVB\_NJ-ATE\_57 |
| C57BL\_6J-ATE\_95 |
| LP\_J-ATE\_105 |
| FVB\_NJ-CTR\_51 |
| LP\_J-ATE\_101 |
| PL\_J-ATE\_76 |
| A\_J-ATE\_122 |
| FVB\_NJ-ATE\_53 |
| NOD\_ShiLtJ-ATE\_22 |
| PL\_J-ATE\_75 |
| FVB\_NJ-CTR\_52 |
| SWR\_J-ATE\_135 |
| BALB\_cByJ-ATE\_160 |
| C57BLKS\_J-ATE\_112 |
| C58\_J-ATE\_29 |
| A\_J-CTR\_120 |
| C57BL\_6J-CTR\_39 |
| SJL\_J-ATE\_17 |
| NOD\_ShiLtJ-CTR\_21 |
| NZB\_BLNJ-ATE\_94 |
| SWR\_J-ATE\_140 |
| LP\_J-CTR\_97 |
| LP\_J-CTR\_104 |
| SWR\_J-CTR\_138 |
| C58\_J-ATE\_30 |
| C57BLKS\_J-ATE\_113 |
| C57BLKS\_J-ATE\_108 |
| LP\_J-CTR\_100 |
| C58\_J-ATE\_36 |
| PL\_J-CTR\_70 |
| A\_J-CTR\_119 |
| PL\_J-ATE\_71 |
| PL\_J-CTR\_74 |
| BALB\_cByJ-CTR\_158 |
| SJL\_J-CTR\_15 |
| C57BL\_6J-CTR\_38 |
| SJL\_J-CTR\_16 |
| BALB\_cByJ-ATE\_151 |
| C57BL\_6J-CTR\_35 |
| NZB\_BLNJ-ATE\_89 |
| NOD\_ShiLtJ-CTR\_20 |
| DBA\_2J-ATE\_6 |
| C3H\_HeJ-ATE\_61 |
| C57BLKS\_J-CTR\_110 |
| C57BL\_6J-ATE\_106 |
| C58\_J-CTR\_32 |
| FVB\_NJ-CTR\_56 |
| SJL\_J-ATE\_11 |
| SM\_J-CTR\_48 |
| SWR\_J-ATE\_139 |
| C57BLKS\_J-CTR\_107 |
| PL\_J-CTR\_72 |
| NOD\_ShiLtJ-CTR\_19 |
| SJL\_J-CTR\_10 |
| NZB\_BLNJ-CTR\_92 |
| SM\_J-ATE\_49 |
| C3H\_HeJ-ATE\_67 |
| SM\_J-ATE\_43 |
| SM\_J-CTR\_40 |
| C58\_J-CTR\_34 |
| NZB\_BLNJ-CTR\_93 |
| CBA\_J-ATE\_132 |
| NZB\_BLNJ-ATE\_88 |
| C57BLKS\_J-CTR\_111 |
| SWR\_J-CTR\_137 |
| I\_LnJ-CTR\_150 |
| CBA\_J-ATE\_127 |
| C58\_J-CTR\_28 |
| A\_J-CTR\_121 |
| SWR\_J-CTR\_134 |
| SM\_J-CTR\_41 |
| BALB\_cJ-ATE\_80 |
| CBA\_J-ATE\_131 |
| C3H\_HeJ-ATE\_66 |
| BALB\_cByJ-CTR\_143 |
| SM\_J-ATE\_42 |
| I\_LnJ-ATE\_154 |
| I\_LnJ-ATE\_146 |
| I\_LnJ-CTR\_153 |
| DBA\_2J-CTR\_9 |
| BALB\_cJ-ATE\_82 |
| BALB\_cByJ-CTR\_144 |
| C57BL\_6J-ISO\_47 |
| LP\_J-ISO\_102 |
| C57BL\_6J-ISO\_59 |
| SJL\_J-ISO\_14 |
| PL\_J-ISO\_78 |
| LP\_J-ISO\_103 |
| DBA\_2J-CTR\_1 |
| PL\_J-ISO\_77 |
| SJL\_J-ISO\_12 |
| FVB\_NJ-ISO\_54 |
| SJL\_J-ISO\_13 |
| C57BL\_6J-ISO\_50 |
| A\_J-ISO\_123 |
| FVB\_NJ-ISO\_58 |
| PL\_J-ISO\_73 |
| C58\_J-ISO\_37 |
| A\_J-ISO\_124 |
| C58\_J-ISO\_31 |
| A\_J-ISO\_118 |
| SWR\_J-ISO\_142 |
| BALB\_cByJ-ISO\_145 |
| C57BLKS\_J-ISO\_114 |
| SWR\_J-ISO\_136 |
| NZB\_BLNJ-ISO\_90 |
| FVB\_NJ-ISO\_55 |
| NZB\_BLNJ-ISO\_96 |
| SWR\_J-ISO\_141 |
| NZB\_BLNJ-ISO\_91 |
| BALB\_cByJ-ISO\_159 |
| I\_LnJ-ISO\_155 |
| I\_LnJ-ISO\_152 |
| SM\_J-ISO\_44 |
| SM\_J-ISO\_46 |
| CBA\_J-ISO\_133 |
| CBA\_J-ISO\_129 |
| CBA\_J-ISO\_128 |
| SM\_J-ISO\_45 |
| C58\_J-ISO\_33 |
| C57BLKS\_J-ISO\_115 |
| C3H\_HeJ-ISO\_68 |
| BALB\_cByJ-ISO\_156 |
| C3H\_HeJ-ISO\_69 |
| NOD\_ShiLtJ-ISO\_26 |
| I\_LnJ-ISO\_149 |
| NOD\_ShiLtJ-ISO\_24 |
| NOD\_ShiLtJ-ISO\_25 |
| DBA\_2J-ISO\_4 |
| DBA\_2J-ISO\_7 |
| BALB\_cJ-ISO\_84 |
| BALB\_cJ-ISO\_83 |
| BALB\_cJ-ISO\_81 |
| C3H\_HeJ-ISO\_62 |
| DBA\_2J-ISO\_8 |

© 2015 Computational Biology Group, Department of Medical Genetics,
University of Lausanne, Switzerland
