# Supplementary material for: RNAseq analysis of heart tissue from mice treated with atenolol and isoproterenol reveals a reciprocal transcriptional response
Source: BMC Genomics. 2016 Sep 7;17(1):717. doi: 10.1186/s12864-016-3059-6 (PMC5015234; doi:10.1186/s12864-016-3059-6)
Supplement: Additional file 4: — Supplementary data. Compressed HTML files of 98 expression modules annotated for genes, strains and GO or KEGG terms (see Additional file 3 for navigation details). (GZ 11006 kb) [file 12864_2016_3059_MOESM4_ESM.gz › modules.html/module-94.html]

Module #94, TG: 0.5, TC: 0.125, 4697 probes, 4697 Entrez genes, 145 conditions

# Previous module | Next module Module #94, TG: 0.5, TC: 0.125, 4697 probes, 4697 Entrez genes, 145 conditions

- Module tree/table

- Expression data

- The BP GO tree
- The CC GO tree
- The MF GO tree

- GO BP enrichment
- GO CC enrichment
- GO MF enrichment
- KEGG enrichment
- miRNA enrichment

- Genes
- Conditions

## Help | Hide | Top Help | Show | Top Expression data

### HELP

The image plot shows the color-coded level of gene expression, for the
genes and conditions in a given transcription module. The genes are on
the horizontal, the conditions on the vertical axis.

The genes are ordered according to their ISA gene scores, similarly
the conditions are ordered according to their condition scores. The
score of a gene means the «degree of inclusion» in
the module: a high score gene is essential in the module.

Condition scores can also be negative, that means that the genes of
the module are all down-regulated in the condition. Here the absolute
value of the score gives the «degree of inclusion».

The plots above and beside the expression matrix show the gene scores
and condition scores, respectively.

Note that the plot is interactive, you can see the name of the gene
and condition under the mouse cursor.

The expression matrix was normalized to have mean zero and standard
deviation one for every gene separately across all conditions
(i.e. not just for the conditions in the module).

— Click on the *Help* button again to close this help window.

Gene:   
Condition:

Under-expression is coded with green,
over-expression with red color.

## Help | Hide | Top Help | Show | Top The GO tree — Biological processes

### HELP

This is one of three sections showing Gene Ontology enrichment of the
current module: in this case for **biological processes**.

The graph shows the hierarchy of the GO categories, their enrichment
for the current module is color coded, and the blue number beside the
category is the minus log ten p-value of the enrichment. (Calculated
using the standard hypergeometric test.) The color of the arrows code
«is a» (cyan) and «part of» relationships.

The tree was built the following way. First all GO terms with more
significant enrichment p-value than 0.05 were collected. Then all
paths from these terms to the root node of the GO tree were included
too. If a GO term is included more than once in the tree, then the
green numbers show 1) the id of the node, this makes it easier to find
other appereances of the term, and 2) the number of appearences.

Note that the same GO category might show up on the graph many
times. This is because the GO was «straightened» for this
graph, i.e. if there are more paths from a GO term to the root node of
the tree, all of them are included. The green numbers

Move the mouse cursor over the terms to get their definition. Clicking
on them takes you to the corresponding Gene Ontology web page.

If you cannot see a graph here at all, that means that there were no
significantly enriched GO categories, at the 0.05 level.

— Click on the *Help* button again to close this help window.

:   **cell communication**

    Any process that mediates interactions between a cell and its surroundings. Encompasses interactions such as signaling or attachment between one cell and another cell, between a cell and an extracellular matrix, or between a cell and any other aspect of its environment.
:   **biological\_process**

    Any process specifically pertinent to the functioning of integrated living units: cells, tissues, organs, and organisms. A process is a collection of molecular events with a defined beginning and end.
:   **cellular process**

    Any process that is carried out at the cellular level, but not necessarily restricted to a single cell. For example, cell communication occurs among more than one cell, but occurs at the cellular level.
:   **signaling**

    The entirety of a process in which information is transmitted within a biological system. This process begins with an active signal and ends when a cellular response has been triggered.
:   **locomotion**

    Self-propelled movement of a cell or organism from one location to another.
:   **single-organism process**

    A biological process that involves only one organism.
:   **single organism signaling**

    A signaling process occurring within a single organism.
:   **single-organism cellular process**

    Any process that is carried out at the cellular level, occurring within a single organism.
:   **cell motility**

    Any process involved in the controlled self-propelled movement of a cell that results in translocation of the cell from one place to another.
:   **localization**

    Any process in which a cell, a substance, or a cellular entity, such as a protein complex or organelle, is transported to, and/or maintained in a specific location.
:   **localization of cell**

    Any process in which a cell is transported to, and/or maintained in, a specific location.
:   **all**

    NA
:   **NA**

    NA
:   **single-organism cellular process**

    Any process that is carried out at the cellular level, occurring within a single organism.
:   **cell motility**

    Any process involved in the controlled self-propelled movement of a cell that results in translocation of the cell from one place to another.
:   **localization**

    Any process in which a cell, a substance, or a cellular entity, such as a protein complex or organelle, is transported to, and/or maintained in a specific location.
:   **localization**

    Any process in which a cell, a substance, or a cellular entity, such as a protein complex or organelle, is transported to, and/or maintained in a specific location.

## Help | Hide | Top Help | Show | Top The GO tree — Cellular Components

### HELP

This is one of three sections showing Gene Ontology enrichment of the
current module: in this case for **cellular components**.

The graph shows the hierarchy of the GO categories, their enrichment
for the current module is color coded, and the blue number beside the
category is the minus log ten p-value of the enrichment. (Calculated
using the standard hypergeometric test.) The color of the arrows code
«is a» (cyan) and «part of» relationships.

The tree was built the following way. First all GO terms with more
significant enrichment p-value than 0.05 were collected. Then all
paths from these terms to the root node of the GO tree were included
too. If a GO term is included more than once in the tree, then the
green numbers show 1) the id of the node, this makes it easier to find
other appereances of the term, and 2) the number of appearences.

Note that the same GO category might show up on the graph many
times. This is because the GO was «straightened» for this
graph, i.e. if there are more paths from a GO term to the root node of
the tree, all of them are included. The green numbers

Move the mouse cursor over the terms to get their definition. Clicking
on them takes you to the corresponding Gene Ontology web page.

If you cannot see a graph here at all, that means that there were no
significantly enriched GO categories, at the 0.05 level.

— Click on the *Help* button again to close this help window.

:   **extracellular region**

    The space external to the outermost structure of a cell. For cells without external protective or external encapsulating structures this refers to space outside of the plasma membrane. This term covers the host cell environment outside an intracellular parasite.
:   **all**

    NA
:   **NA**

    NA

## Help | Hide | Top Help | Show | Top The GO tree — Molecular Function

### HELP

This is one of three sections showing Gene Ontology enrichment of the
current module: in this case for **molecular function**.

The graph shows the hierarchy of the GO categories, their enrichment
for the current module is color coded, and the blue number beside the
category is the minus log ten p-value of the enrichment. (Calculated
using the standard hypergeometric test.) The color of the arrows code
«is a» (cyan) and «part of» relationships.

The tree was built the following way. First all GO terms with more
significant enrichment p-value than 0.05 were collected. Then all
paths from these terms to the root node of the GO tree were included
too. If a GO term is included more than once in the tree, then the
green numbers show 1) the id of the node, this makes it easier to find
other appereances of the term, and 2) the number of appearences.

Note that the same GO category might show up on the graph many
times. This is because the GO was «straightened» for this
graph, i.e. if there are more paths from a GO term to the root node of
the tree, all of them are included. The green numbers

Move the mouse cursor over the terms to get their definition. Clicking
on them takes you to the corresponding Gene Ontology web page.

If you cannot see a graph here at all, that means that there were no
significantly enriched GO categories, at the 0.05 level.

— Click on the *Help* button again to close this help window.

## Help | Hide | Top Help | Show | Top GO BP test for over-representation

### HELP

List of all enriched GO categories (biological processes), at the 0.05
p-value level.

The columns:

- **ExpCount** is the expected count of genes in the
  module annotated with the given GO term, just by chance.
- **Count**
  is the number of genes in the module annotated with the given GO
  term.
- **Size** is the total number of genes (in our universe)
  annotated with the GO term.

Clicking on **Count** shows the genes that drive the
enrichment. You can also click on the individual numbers in
the **Count** column, to show the driving genes for that individual
GO category.

Clicking on the GO identifiers takes you to the Gene Ontology web
pages.

— Click on the *Help* button again to close this help window.

| Id | Pvalue | ExpCount | Count | Size | Term |
| --- | --- | --- | --- | --- | --- |
| GO:0006928 | 1.579e-04 | 304 | 384 Abcc1, Ablim1, Abr, Acan, Acvr1b, Ada, Adarb1, Agtr1a, Aimp1, Akap6, Anxa3, Ap3d1, Ap3m1, Apbb2, Apcdd1, Apex1, Apoh, Aqp1, Arap3, Arc, Arhgap21, Arhgap24, Arid5b, Arl3, Atn1, Atp1a2, Atp1a4, Avl9, Bbs1, Bcl2, Bcr, Bdkrb1, Bloc1s5, Bloc1s6, Bmp10, Bmp4, Bmp7, Bmper, Bmpr1b, Bmpr2, Boc, Brca1, Brk1, C3ar1, C5ar1, C5ar2, Camk1d, Cap1, Casc1, Casq2, Catsper3, Catsper4, Ccbe1, Ccdc23, Ccdc39, Cck, Ccl11, Ccl19, Ccl2, Ccl3, Ccl5, Ccl6, Ccl7, Ccr1l1, Ccr4, Cd2ap, Cd44, Cd63, Cdc42bpa, Cdc42bpb, Cdh2, Cdk5r1, Celf3, Chl1, Chst4, Clic4, Cln3, Cntnap1, Col18a1, Col1a1, Col3a1, Col5a1, Copg1, Coro1a, Csf1r, Csf3r, Ctgf, Ctsh, Cxadr, Cxcl10, Cxcl13, Cxcl2, Cxcl3, Cxcr4, Cyp1b1, D130043K22Rik, Dab2, Dab2ip, Ddit4, Ddr1, Dixdc1, Dll4, Dnah11, Dnah12, Dnah17, Dnah8, Dock4, Dpp4, Dst, Dstn, Dtnbp1, Dync1h1, Dync1i2, Dync1li2, Dync2h1, Dynlrb1, Ecm1, Edn3, Ednra, Efnb2, Efnb3, Egr2, Elmo2, Enah, Eng, Epha3, Epha8, Ephb3, Etv1, Etv4, Ext1, F2r, F2rl1, F3, F7, Fam110c, Fap, Fgf10, Fgf13, Fgf7, Fgfr1op, Fgfr4, Flt1, Flt4, Fmnl3, Foxc2, Foxh1, Fscn1, Fut7, Fyn, Fzd3, Gab1, Gas6, Gata4, Gdnf, Gfra1, Gli2, Gpld1, Gpx1, Gsk3b, Hace1, Has1, Hbegf, Hc, Hdac6, Hes1, Hexb, Hras1, Hrg, Hsp90aa1, Htr2b, Hyal2, Hydin, Icam1, Ift43, Igf1, Igfbp5, Ikbkap, Il16, Il1b, Il1rn, Inpp5b, Irs2, Itga1, Itga3, Itga4, Itga9, Itgam, Itgav, Itgb1, Itgb1bp1, Itgb2, Itgb4, Jam3, Kcnj2, Kctd13, Kdr, Kif17, Kif18a, Kif1a, Kif1b, Kif1c, Kif20a, Kif20b, Kif21b, Kif22, Kif26b, Kif27, Kif3a, Kif3b, Kif3c, Kif4, Kif5b, Kif7, L1cam, Lama3, Lbp, Lefty1, Lhx2, Lhx6, Lmna, Lmtk2, Loxl2, Lpar1, Lrp5, Lrrc16a, Lyn, Lyst, Map1b, Map4, Mapt, Mark2, Mcc, Mdga1, Mdk, Mgarp, Mien1, Mkks, Mmp3, Msn, Msx2, Mycbp2, Myh6, Myh7, Mylip, Myo18a, Myo1c, Myo1f, Myrip, Nck2, Nckap1, Nckap1l, Nde1, Ndel1, Ndnf, Nefl, Nf1, Nisch, Nog, Nr4a3, Nrg1, Nrtn, Nsmf, Nup62, Onecut2, Pcnt, Pde4d, Pdgfa, Pdgfb, Pdpn, Pecam1, Pex14, Pex7, Pf4, Phactr4, Phox2b, Pip5k1a, Pkp2, Plat, Plau, Pld1, Pld2, Plxna2, Plxna3, Podxl2, Pou4f1, Ppm1f, Ppp2r3a, Prkcz, Prkd1, Prkx, Prox1, Ptgs2, Ptk2b, Ptk6, Ptp4a1, Ptprj, Ptprk, Rac1, Rap2a, Rapgef4, Rasgef1a, Retn, Retnlg, Rfx3, Robo1, Robo2, Robo3, Robo4, Rock1, Ropn1l, Rps6kb1, Rras, Rsph4a, Rtn4, Runx3, Scai, Scarb1, Scg2, Scn5a, Scrib, Selk, Sell, Selp, Selplg, Sema5a, Sema6c, Serpine1, Sfrp1, Sfrp2, Smad4, Smo, Sord, Sox8, Spa17, Spag6, Spata13, Srcin1, Sri, Srpx2, Ssna1, Sstr4, Stk10, Stmn1, Strip2, Sulf1, Sun2, Syk, Syne2, Tac1, Tacstd2, Tbx1, Tbx5, Tek, Tekt2, Tenm2, Tgfb1, Tgfb2, Tgfbr1, Tgfbr3, Thbs1, Thbs4, Timp1, Tmf1, Tmod3, Tmsb4x, Tnf, Tnfrsf12a, Tnn, Tnnt2, Trib1, Trp53, Tspo, Ttc21b, Ttll1, Tyro3, Uty, Vasp, Wnt11, Wnt4, Wwc1, Xcl1, Zeb2, Zranb1 | 1031 | cellular component movement |
| GO:0048870 | 9.853e-03 | 235.9 | 297 Abcc1, Abr, Acan, Acvr1b, Ada, Adarb1, Agtr1a, Aimp1, Anxa3, Apbb2, Apcdd1, Apex1, Apoh, Aqp1, Arap3, Arc, Arhgap24, Arid5b, Atn1, Atp1a4, Avl9, Bbs1, Bcl2, Bcr, Bdkrb1, Bmp10, Bmp4, Bmper, Bmpr2, Brca1, Brk1, C3ar1, C5ar1, C5ar2, Camk1d, Cap1, Catsper3, Catsper4, Ccbe1, Ccdc23, Ccdc39, Cck, Ccl11, Ccl19, Ccl2, Ccl3, Ccl5, Ccl6, Ccl7, Ccr1l1, Ccr4, Cd2ap, Cd44, Cd63, Cdc42bpa, Cdc42bpb, Cdh2, Cdk5r1, Celf3, Chl1, Chst4, Clic4, Col18a1, Col1a1, Col3a1, Col5a1, Coro1a, Csf1r, Csf3r, Ctgf, Ctsh, Cxadr, Cxcl10, Cxcl13, Cxcl2, Cxcl3, Cxcr4, Cyp1b1, D130043K22Rik, Dab2, Dab2ip, Ddit4, Ddr1, Dixdc1, Dll4, Dnah11, Dock4, Dpp4, Dync2h1, Ecm1, Edn3, Ednra, Efnb2, Elmo2, Eng, Epha3, Epha8, Ephb3, F2r, F2rl1, F3, F7, Fam110c, Fap, Fgf10, Fgf13, Fgf7, Fgfr1op, Fgfr4, Flt1, Flt4, Fmnl3, Foxc2, Foxh1, Fscn1, Fut7, Fyn, Fzd3, Gab1, Gas6, Gdnf, Gfra1, Gpld1, Gpx1, Gsk3b, Hace1, Has1, Hbegf, Hc, Hdac6, Hes1, Hexb, Hras1, Hrg, Hsp90aa1, Htr2b, Hyal2, Icam1, Igf1, Igfbp5, Ikbkap, Il16, Il1b, Il1rn, Inpp5b, Irs2, Itga1, Itga3, Itga4, Itga9, Itgam, Itgav, Itgb1, Itgb1bp1, Itgb2, Itgb4, Jam3, Kctd13, Kdr, Lama3, Lbp, Lefty1, Lhx6, Lmna, Loxl2, Lpar1, Lrp5, Lrrc16a, Lyn, Lyst, Mapt, Mark2, Mcc, Mdga1, Mdk, Mien1, Mkks, Mmp3, Msn, Msx2, Myo18a, Myo1c, Myo1f, Nck2, Nckap1, Nckap1l, Nde1, Ndel1, Ndnf, Nf1, Nisch, Nog, Nrg1, Nrtn, Nsmf, Nup62, Onecut2, Pcnt, Pde4d, Pdgfa, Pdgfb, Pdpn, Pecam1, Pex7, Pf4, Phactr4, Phox2b, Pip5k1a, Pkp2, Plat, Plau, Pld1, Pld2, Plxna2, Podxl2, Pou4f1, Ppm1f, Ppp2r3a, Prkcz, Prkd1, Prkx, Prox1, Ptgs2, Ptk2b, Ptk6, Ptp4a1, Ptprj, Ptprk, Rac1, Rap2a, Rapgef4, Rasgef1a, Retn, Retnlg, Rfx3, Robo1, Robo3, Robo4, Rock1, Ropn1l, Rps6kb1, Rras, Rtn4, Scai, Scarb1, Scg2, Scrib, Selk, Sell, Selp, Selplg, Serpine1, Sfrp1, Sfrp2, Smo, Sord, Sox8, Spa17, Spag6, Spata13, Srcin1, Srpx2, Sstr4, Stk10, Strip2, Sulf1, Sun2, Syk, Syne2, Tac1, Tacstd2, Tbx1, Tbx5, Tek, Tekt2, Tgfb1, Tgfb2, Tgfbr1, Tgfbr3, Thbs1, Thbs4, Timp1, Tmf1, Tmsb4x, Tnf, Tnfrsf12a, Tnn, Trib1, Trp53, Tspo, Tyro3, Wnt11, Wnt4, Wwc1, Xcl1, Zeb2, Zranb1 | 800 | cell motility |
| GO:0051674 | 9.853e-03 | 235.9 | 297 Abcc1, Abr, Acan, Acvr1b, Ada, Adarb1, Agtr1a, Aimp1, Anxa3, Apbb2, Apcdd1, Apex1, Apoh, Aqp1, Arap3, Arc, Arhgap24, Arid5b, Atn1, Atp1a4, Avl9, Bbs1, Bcl2, Bcr, Bdkrb1, Bmp10, Bmp4, Bmper, Bmpr2, Brca1, Brk1, C3ar1, C5ar1, C5ar2, Camk1d, Cap1, Catsper3, Catsper4, Ccbe1, Ccdc23, Ccdc39, Cck, Ccl11, Ccl19, Ccl2, Ccl3, Ccl5, Ccl6, Ccl7, Ccr1l1, Ccr4, Cd2ap, Cd44, Cd63, Cdc42bpa, Cdc42bpb, Cdh2, Cdk5r1, Celf3, Chl1, Chst4, Clic4, Col18a1, Col1a1, Col3a1, Col5a1, Coro1a, Csf1r, Csf3r, Ctgf, Ctsh, Cxadr, Cxcl10, Cxcl13, Cxcl2, Cxcl3, Cxcr4, Cyp1b1, D130043K22Rik, Dab2, Dab2ip, Ddit4, Ddr1, Dixdc1, Dll4, Dnah11, Dock4, Dpp4, Dync2h1, Ecm1, Edn3, Ednra, Efnb2, Elmo2, Eng, Epha3, Epha8, Ephb3, F2r, F2rl1, F3, F7, Fam110c, Fap, Fgf10, Fgf13, Fgf7, Fgfr1op, Fgfr4, Flt1, Flt4, Fmnl3, Foxc2, Foxh1, Fscn1, Fut7, Fyn, Fzd3, Gab1, Gas6, Gdnf, Gfra1, Gpld1, Gpx1, Gsk3b, Hace1, Has1, Hbegf, Hc, Hdac6, Hes1, Hexb, Hras1, Hrg, Hsp90aa1, Htr2b, Hyal2, Icam1, Igf1, Igfbp5, Ikbkap, Il16, Il1b, Il1rn, Inpp5b, Irs2, Itga1, Itga3, Itga4, Itga9, Itgam, Itgav, Itgb1, Itgb1bp1, Itgb2, Itgb4, Jam3, Kctd13, Kdr, Lama3, Lbp, Lefty1, Lhx6, Lmna, Loxl2, Lpar1, Lrp5, Lrrc16a, Lyn, Lyst, Mapt, Mark2, Mcc, Mdga1, Mdk, Mien1, Mkks, Mmp3, Msn, Msx2, Myo18a, Myo1c, Myo1f, Nck2, Nckap1, Nckap1l, Nde1, Ndel1, Ndnf, Nf1, Nisch, Nog, Nrg1, Nrtn, Nsmf, Nup62, Onecut2, Pcnt, Pde4d, Pdgfa, Pdgfb, Pdpn, Pecam1, Pex7, Pf4, Phactr4, Phox2b, Pip5k1a, Pkp2, Plat, Plau, Pld1, Pld2, Plxna2, Podxl2, Pou4f1, Ppm1f, Ppp2r3a, Prkcz, Prkd1, Prkx, Prox1, Ptgs2, Ptk2b, Ptk6, Ptp4a1, Ptprj, Ptprk, Rac1, Rap2a, Rapgef4, Rasgef1a, Retn, Retnlg, Rfx3, Robo1, Robo3, Robo4, Rock1, Ropn1l, Rps6kb1, Rras, Rtn4, Scai, Scarb1, Scg2, Scrib, Selk, Sell, Selp, Selplg, Serpine1, Sfrp1, Sfrp2, Smo, Sord, Sox8, Spa17, Spag6, Spata13, Srcin1, Srpx2, Sstr4, Stk10, Strip2, Sulf1, Sun2, Syk, Syne2, Tac1, Tacstd2, Tbx1, Tbx5, Tek, Tekt2, Tgfb1, Tgfb2, Tgfbr1, Tgfbr3, Thbs1, Thbs4, Timp1, Tmf1, Tmsb4x, Tnf, Tnfrsf12a, Tnn, Trib1, Trp53, Tspo, Tyro3, Wnt11, Wnt4, Wwc1, Xcl1, Zeb2, Zranb1 | 800 | localization of cell |
| GO:0040011 | 1.349e-02 | 279 | 344 Abcc1, Ablim1, Abr, Acan, Ackr3, Acvr1b, Ada, Adarb1, Adora2a, Agtr1a, Aimp1, Anxa3, Apbb2, Apcdd1, Apex1, Apoh, Aqp1, Arap3, Arc, Arhgap24, Arid5b, Atn1, Atp1a2, Atp1a4, Avl9, Bbs1, Bcl2, Bcr, Bdkrb1, Bmp10, Bmp4, Bmp7, Bmper, Bmpr1b, Bmpr2, Boc, Brca1, Brk1, Bves, C3ar1, C5ar1, C5ar2, Camk1d, Cap1, Casr, Catsper3, Catsper4, Ccbe1, Ccdc23, Ccdc39, Cck, Ccl11, Ccl17, Ccl19, Ccl2, Ccl22, Ccl3, Ccl5, Ccl6, Ccl7, Ccl8, Ccl9, Ccr1l1, Ccr4, Cd2ap, Cd44, Cd63, Cdc42bpa, Cdc42bpb, Cdh2, Cdk5r1, Celf3, Chl1, Chst4, Clic4, Col18a1, Col1a1, Col3a1, Col5a1, Coro1a, Csf1r, Csf3r, Ctgf, Ctsh, Cxadr, Cxcl10, Cxcl13, Cxcl2, Cxcl3, Cxcl5, Cxcr4, Cxcr5, Cyp1b1, Cysltr1, D130043K22Rik, Dab2, Dab2ip, Ddit4, Ddr1, Dixdc1, Dll4, Dnah11, Dock4, Dpp4, Dync2h1, Ear11, Ecm1, Edn3, Ednra, Efnb2, Efnb3, Egr2, Elmo2, Enah, Eng, Epha3, Epha8, Ephb3, Etv1, Etv4, Ext1, F2r, F2rl1, F3, F7, Fam110c, Fap, Fgf10, Fgf13, Fgf7, Fgfr1op, Fgfr4, Flt1, Flt4, Fmnl3, Foxc2, Foxh1, Fpr2, Fscn1, Fut7, Fyn, Fzd3, Gab1, Gas6, Gdnf, Gfra1, Gli2, Gpld1, Gpx1, Grin2a, Grin2c, Gsk3b, Hace1, Has1, Hbegf, Hc, Hdac6, Hes1, Hexb, Hras1, Hrg, Hsp90aa1, Htr2b, Hyal2, Icam1, Igf1, Igfbp5, Ikbkap, Il16, Il1b, Il1rn, Inpp5b, Irs2, Itga1, Itga3, Itga4, Itga9, Itgam, Itgav, Itgb1, Itgb1bp1, Itgb2, Itgb4, Jam3, Kctd13, Kdr, L1cam, Lama3, Lbp, Lefty1, Lhx2, Lhx6, Lmna, Lmtk2, Loxl2, Lpar1, Lrp5, Lrrc16a, Lyn, Lyst, Mapt, Mark2, Mcc, Mdga1, Mdk, Mien1, Mkks, Mmp3, Msn, Msx2, Mycbp2, Myo18a, Myo1c, Myo1f, Nck2, Nckap1, Nckap1l, Nde1, Ndel1, Ndnf, Nefl, Nf1, Nisch, Nlgn2, Nog, Nr4a3, Nrg1, Nrtn, Nsmf, Nup62, Onecut2, Pcnt, Pde4d, Pdgfa, Pdgfb, Pdpn, Pecam1, Pex7, Pf4, Phactr4, Phox2b, Pik3c2g, Pip5k1a, Pip5k1c, Pkp2, Pla2g6, Plat, Plau, Pld1, Pld2, Plxna2, Plxna3, Podxl2, Pou4f1, Ppm1f, Ppp2r3a, Prkcz, Prkd1, Prkx, Prox1, Ptgs2, Ptk2b, Ptk6, Ptp4a1, Ptprj, Ptprk, Rac1, Rap2a, Rapgef4, Rasgef1a, Retn, Retnlg, Rfx3, Robo1, Robo2, Robo3, Robo4, Rock1, Ropn1l, Rps6kb1, Rras, Rtn4, Runx3, Scai, Scarb1, Scg2, Scrib, Selk, Sell, Selp, Selplg, Sema5a, Sema6c, Serpine1, Sfrp1, Sfrp2, Smad4, Smo, Snca, Sord, Sox8, Spa17, Spag6, Spata13, Srcin1, Srpx2, Sstr4, Stk10, Strip2, Sulf1, Sun2, Syk, Syne2, Tac1, Tacstd2, Tbx1, Tbx5, Tek, Tekt2, Tenm2, Tgfb1, Tgfb2, Tgfbr1, Tgfbr3, Thbs1, Thbs4, Timp1, Tmf1, Tmsb4x, Tnf, Tnfrsf12a, Tnn, Trib1, Trp53, Tspo, Ttn, Tyro3, Vasp, Wnt11, Wnt4, Wwc1, Xcl1, Zeb2, Zranb1 | 946 | locomotion |
| GO:0016477 | 2.246e-02 | 220 | 277 Abcc1, Abr, Acan, Acvr1b, Ada, Adarb1, Agtr1a, Aimp1, Anxa3, Apbb2, Apcdd1, Apex1, Apoh, Aqp1, Arap3, Arc, Arhgap24, Arid5b, Atn1, Avl9, Bbs1, Bcl2, Bcr, Bdkrb1, Bmp10, Bmp4, Bmper, Bmpr2, C3ar1, C5ar1, C5ar2, Camk1d, Cap1, Ccbe1, Ccdc23, Cck, Ccl11, Ccl19, Ccl2, Ccl3, Ccl5, Ccl6, Ccl7, Ccr1l1, Ccr4, Cd2ap, Cd44, Cd63, Cdc42bpa, Cdc42bpb, Cdh2, Cdk5r1, Chl1, Chst4, Clic4, Col18a1, Col1a1, Col3a1, Col5a1, Coro1a, Csf1r, Csf3r, Ctgf, Ctsh, Cxadr, Cxcl10, Cxcl13, Cxcl2, Cxcl3, Cxcr4, Cyp1b1, D130043K22Rik, Dab2, Dab2ip, Ddit4, Ddr1, Dixdc1, Dll4, Dock4, Dpp4, Ecm1, Edn3, Ednra, Efnb2, Elmo2, Eng, Epha3, Epha8, Ephb3, F2r, F2rl1, F3, F7, Fam110c, Fap, Fgf10, Fgf13, Fgf7, Fgfr1op, Fgfr4, Flt1, Flt4, Fmnl3, Foxc2, Foxh1, Fscn1, Fut7, Fyn, Fzd3, Gab1, Gas6, Gdnf, Gfra1, Gpld1, Gpx1, Gsk3b, Hace1, Has1, Hbegf, Hc, Hdac6, Hes1, Hexb, Hras1, Hrg, Hsp90aa1, Htr2b, Hyal2, Icam1, Igf1, Igfbp5, Ikbkap, Il16, Il1b, Il1rn, Irs2, Itga1, Itga3, Itga4, Itga9, Itgam, Itgav, Itgb1, Itgb1bp1, Itgb2, Jam3, Kctd13, Kdr, Lama3, Lbp, Lefty1, Lhx6, Lmna, Loxl2, Lpar1, Lrp5, Lrrc16a, Lyn, Lyst, Mapt, Mark2, Mcc, Mdga1, Mdk, Mien1, Mmp3, Msn, Msx2, Myo18a, Myo1c, Myo1f, Nck2, Nckap1, Nckap1l, Nde1, Ndel1, Ndnf, Nf1, Nisch, Nog, Nrg1, Nrtn, Nsmf, Nup62, Onecut2, Pcnt, Pde4d, Pdgfa, Pdgfb, Pdpn, Pecam1, Pex7, Pf4, Phactr4, Phox2b, Pip5k1a, Pkp2, Plat, Plau, Pld1, Pld2, Plxna2, Podxl2, Pou4f1, Ppm1f, Prkcz, Prkd1, Prkx, Prox1, Ptgs2, Ptk2b, Ptk6, Ptp4a1, Ptprj, Ptprk, Rac1, Rap2a, Rapgef4, Rasgef1a, Retn, Retnlg, Robo1, Robo3, Robo4, Rock1, Rps6kb1, Rras, Rtn4, Scai, Scarb1, Scg2, Scrib, Selk, Sell, Selp, Selplg, Serpine1, Sfrp1, Sfrp2, Smo, Sox8, Spata13, Srcin1, Srpx2, Sstr4, Stk10, Strip2, Sulf1, Sun2, Syk, Syne2, Tac1, Tacstd2, Tbx1, Tbx5, Tek, Tgfb1, Tgfb2, Tgfbr1, Tgfbr3, Thbs1, Thbs4, Timp1, Tmsb4x, Tnf, Tnfrsf12a, Tnn, Trib1, Trp53, Tspo, Tyro3, Wnt11, Wnt4, Wwc1, Xcl1, Zeb2, Zranb1 | 746 | cell migration |

## Help | Hide | Top Help | Show | Top GO CC test for over-representation

### HELP

List of all enriched GO categories (cellular components), at the 0.05
p-value level.

The columns:

- **ExpCount** is the expected count of genes in the
  module annotated with the given GO term, just by chance.
- **Count**
  is the number of genes in the module annotated with the given GO
  term.
- **Size** is the total number of genes (in our universe)
  annotated with the GO term.

Clicking on **Count** shows the genes that drive the
enrichment. You can also click on the individual numbers in
the **Count** column, to show the driving genes for that individual
GO category.

Clicking on the GO identifiers takes you to the Gene Ontology web
pages.

— Click on the *Help* button again to close this help window.

| Id | Pvalue | ExpCount | Count | Size | Term |
| --- | --- | --- | --- | --- | --- |
| GO:0005576 | 3.703e-03 | 395 | 468 1110058L19Rik, 2610507B11Rik, 3110057O12Rik, 4930578C19Rik, 5430419D17Rik, A2m, Abca1, Acan, Acp6, Acpp, Actg1, Ada, Adam22, Adamts12, Adamts17, Adamts19, Adamts2, Adamts3, Adamts7, Adamtsl4, Adamtsl5, Adpgk, Aggf1, Agrp, Ahsg, Aimp1, Alad, Alb, Ambp, Amh, Angptl1, Angptl2, Angptl6, Anxa2, Aoah, Apoa4, Apoc1, Apoc3, Apoc4, Apoh, Aqp1, Arsa, Art5, Bglap3, Bmp10, Bmp3, Bmp4, Bmp7, Bmper, Brinp2, C1qtnf1, C1qtnf2, C1qtnf5, C1qtnf6, C1qtnf7, C1ra, C1s, C2, C3, C4bp, Cask, Casp1, Ccbe1, Ccdc126, Ccdc134, Cck, Ccl11, Ccl17, Ccl2, Ccl22, Ccl3, Ccl5, Ccl6, Ccl7, Ccl8, Ccl9, Cd109, Cd40lg, Cd59b, Cd5l, Cdnf, Cep55, Cfdp1, Cfh, Cfi, Chad, Chgb, Chi3l1, Chl1, Clec11a, Clec3b, Cltc, Cma1, Col10a1, Col11a2, Col13a1, Col14a1, Col17a1, Col18a1, Col19a1, Col1a1, Col20a1, Col28a1, Col3a1, Col4a1, Col4a2, Col4a3, Col4a4, Col4a5, Col5a1, Col5a2, Col6a1, Col6a2, Col7a1, Col8a1, Col9a3, Copa, Cp, Cpa1, Creld2, Crlf1, Crlf2, Cst10, Ctf1, Ctgf, Ctla2a, Ctsd, Ctsh, Ctsk, Cxadr, Cxcl10, Cxcl13, Cxcl2, Cxcl3, Cxcl5, Cyb5d2, D17Wsu104e, Dcn, Dhrs11, Dkk2, Dkk3, Dpp4, Dync1h1, Ecm1, Ecm2, Edil3, Edn3, Efemp1, Efemp2, Egf, Egfbp2, Eng, Eno1, Enox1, Epha3, Ephb6, Ephx3, Epor, Erap1, F2, F3, F5, F7, Fam150b, Fam180a, Fam198a, Fam19a5, Fam5c, Fap, Fbln1, Fbln2, Fcgr2b, Fgf10, Fgf16, Fgf20, Fgf21, Fgf6, Fgf7, Fgfbp1, Fgg, Fgl1, Fgl2, Fjx1, Fkrp, Fmod, Fndc7, Folr4, Frem1, Frem2, Frzb, Fst, Fstl5, Fuca2, Gal, Gas6, Gbp2b, Gcnt1, Gdf10, Gdf3, Gdf6, Gdnf, Gfra1, Gfra4, Glb1l, Glb1l2, Glipr1, Gm128, Gm6484, Gnas, Gpld1, Gpx7, Grn, Hapln3, Hbegf, Hc, Hfe2, Hgfac, Hhipl1, Hhipl2, Hmcn1, Hrg, Hsd17b11, Hspa8, Hspd1, Hyal3, Icam1, Ifnar2, Igf1, Igfbp4, Igfbp5, Igfbp7, Igfbpl1, Igsf10, Il10, Il16, Il17re, Il18, Il1a, Il1b, Il1f9, Il1r1, Il1rap, Il1rn, Il7, Inha, Inhba, Isg15, Ism1, Itga4, Itgb1, Itgb4, Itih1, Itih3, Itm2b, Izumo4, Jam3, Kdr, Kif23, Klk13, Klkb1, Kng1, Lad1, Lama3, Lbp, Lcn2, Lect1, Lefty1, Lepre1, Leprel1, Lgals1, Lifr, Lmcd1, Lox, Loxl1, Loxl2, Loxl3, Lrp2, Lrpap1, Ltbp1, Ltbp2, Ltbp3, Ly6g5b, Ly6k, Lyz2, Mamdc2, Man2b2, Masp2, Mdk, Mertk, Mettl24, Mfap2, Mfap5, Mmel1, Mmp13, Mmp17, Mmp24, Mmp3, Mmrn1, Msn, Muc13, Muc19, Muc5b, Muc6, Myo1c, Nav2, Ndnf, Nenf, Ngf, Ngp, Nid1, Nid2, Nog, Nov, Npb, Nptxr, Nrtn, Nt5e, Ntn3, Nts, Nyx, Ogn, Olfm2, Osm, Otog, Otogl, Pcnt, Pcsk2, Pcyox1l, Pdgfa, Pdgfb, Pdgfrl, Pecam1, Pf4, Pi16, Pi4k2a, Pigr, Pla2g12a, Pla2g15, Pla2g2d, Pla2g2e, Pla2r1, Plat, Plau, Plaur, Pm20d1, Pnp, Pon1, Pon2, Pon3, Postn, Prf1, Prom1, Prom2, Proz, Prrg2, Prss12, Prss57, Pspn, Ptch1, Ptgds, Ptn, Ptprg, Ptprz1, Rbm44, Retn, Retnlg, Rnase10, Rpsa, Rspo2, S100b, Saa2, Sbsn, Sbspon, Scg2, Scg3, Sct, Sdc4, Sdf2, Selp, Sema3b, Serpina11, Serpina1a, Serpina1d, Serpina1e, Serpina3k, Serpina3n, Serpina6, Serpinb1c, Serpinb6a, Serpinb9, Serpinc1, Serpine1, Serpinf1, Serping1, Sez6, Sfrp1, Sfrp2, Sfrp5, Slc1a3, Slc2a4, Slmap, Slpi, Smc3, Smpdl3b, Snca, Spaca3, Sparc, Spon2, Spp2, Srcrb4d, Srgn, Srpx2, Stxbp2, Sulf1, Svep1, Tac1, Tacstd2, Tbc1d15, Tecta, Tek, Tfpi, Tgfa, Tgfb1, Tgfb2, Tgfb3, Tgfbi, Tgfbr3, Thbd, Thbs1, Thbs2, Thbs3, Thbs4, Thsd4, Thsd7a, Timp1, Tnf, Tnfrsf18, Tnfrsf1a, Tnfsf15, Tnfsf18, Tnn, Tpt1, Trem2, Tub, Tuft1, Tulp3, Twsg1, Txn1, Txndc16, Ucn, Ush2a, Vimp, Vwa7, Vwc2, Wfdc18, Wfdc3, Wfikkn2, Wif1, Wisp1, Wnt1, Wnt11, Wnt16, Wnt4, Wnt6, Wnt8b, Wnt9a, Wnt9b, Xcl1 | 1339 | extracellular region |
| GO:0044421 | 2.103e-02 | 253.4 | 308 A2m, Abca1, Acan, Actg1, Ada, Adamts12, Adamts19, Adamts2, Adamts7, Adamtsl4, Agrp, Ahsg, Aimp1, Alad, Alb, Ambp, Amh, Angptl1, Anxa2, Apoa4, Apoc1, Apoc3, Apoc4, Apoh, Aqp1, Arsa, Bmp10, Bmp3, Bmp4, Bmp7, Bmper, C1qtnf1, C1qtnf2, C1qtnf5, C1qtnf6, C1qtnf7, C3, Cask, Ccbe1, Cck, Ccl11, Ccl17, Ccl2, Ccl22, Ccl3, Ccl5, Ccl6, Ccl7, Ccl8, Ccl9, Cd109, Cd40lg, Cd59b, Cep55, Cfdp1, Cfh, Chad, Chi3l1, Chl1, Clec3b, Cltc, Col10a1, Col11a2, Col13a1, Col14a1, Col17a1, Col18a1, Col19a1, Col1a1, Col20a1, Col28a1, Col3a1, Col4a1, Col4a2, Col4a3, Col4a4, Col4a5, Col5a1, Col5a2, Col6a1, Col6a2, Col7a1, Col8a1, Col9a3, Copa, Cp, Cpa1, Creld2, Crlf1, Ctf1, Ctgf, Ctsd, Ctsh, Ctsk, Cxadr, Cxcl10, Cxcl13, Cxcl2, Cxcl3, Cxcl5, Dcn, Dkk2, Dkk3, Dpp4, Dync1h1, Ecm1, Ecm2, Edil3, Edn3, Efemp1, Efemp2, Egf, Egfbp2, Eng, Eno1, F2, F3, F5, F7, Fap, Fbln1, Fbln2, Fgf10, Fgf7, Fgg, Fjx1, Fkrp, Fmod, Frem1, Frem2, Frzb, Gas6, Gbp2b, Gcnt1, Gdf10, Gdf3, Gdf6, Gdnf, Gfra1, Gfra4, Gpld1, Grn, Hapln3, Hbegf, Hc, Hfe2, Hgfac, Hmcn1, Hspa8, Hspd1, Icam1, Ifnar2, Igf1, Igfbp4, Igfbp5, Igfbp7, Il10, Il16, Il18, Il1a, Il1b, Il1f9, Il1r1, Il1rn, Il7, Inha, Inhba, Itga4, Itgb1, Itgb4, Itm2b, Jam3, Kif23, Klk13, Klkb1, Lad1, Lama3, Lbp, Lcn2, Lefty1, Lepre1, Leprel1, Lgals1, Lmcd1, Lox, Loxl1, Loxl2, Lrp2, Ltbp1, Ltbp2, Ltbp3, Mamdc2, Mertk, Mfap2, Mfap5, Mmel1, Mmp13, Mmp17, Mmp24, Mmp3, Msn, Myo1c, Nav2, Nenf, Ngf, Nid1, Nid2, Nog, Npb, Nptxr, Nt5e, Ntn3, Nyx, Ogn, Osm, Otog, Pcnt, Pcsk2, Pdgfa, Pdgfb, Pecam1, Pf4, Pi4k2a, Plat, Plau, Pon1, Pon3, Postn, Prf1, Prom1, Prom2, Pspn, Ptgds, Ptn, Ptprg, Ptprz1, Rbm44, Retn, Retnlg, Rpsa, S100b, Saa2, Sbspon, Scg2, Selp, Serpina11, Serpina1a, Serpina1d, Serpina1e, Serpina3k, Serpina3n, Serpina6, Serpinb1c, Serpinb6a, Serpinb9, Serpinc1, Serpine1, Serpinf1, Serping1, Sez6, Sfrp1, Sfrp2, Slc1a3, Slc2a4, Slmap, Smc3, Smpdl3b, Snca, Sparc, Spon2, Srgn, Stxbp2, Sulf1, Tac1, Tacstd2, Tecta, Tfpi, Tgfa, Tgfb1, Tgfb2, Tgfb3, Tgfbi, Tgfbr3, Thbd, Thbs1, Thbs2, Thbs4, Thsd4, Timp1, Tnf, Tnfrsf1a, Tnfsf15, Tnfsf18, Tnn, Tpt1, Twsg1, Ush2a, Vimp, Vwc2, Wnt1, Wnt11, Wnt16, Wnt4, Wnt6, Wnt8b, Wnt9a, Wnt9b, Xcl1 | 859 | extracellular region part |

## Help | Hide | Top Help | Show | Top GO MF test for over-representation

### HELP

List of all enriched GO categories (molecular function), at the 0.05
p-value level.

The columns:

- **ExpCount** is the expected count of genes in the
  module annotated with the given GO term, just by chance.
- **Count**
  is the number of genes in the module annotated with the given GO
  term.
- **Size** is the total number of genes (in our universe)
  annotated with the GO term.

Clicking on **Count** shows the genes that drive the
enrichment. You can also click on the individual numbers in
the **Count** column, to show the driving genes for that individual
GO category.

Clicking on the GO identifiers takes you to the Gene Ontology web
pages.

— Click on the *Help* button again to close this help window.

| Id | Pvalue | ExpCount | Count | Size | Term |
| --- | --- | --- | --- | --- | --- |
| GO:0002020 | 1.707e-02 | 20.31 | 38 Adamtsl4, Bcl2, Casp8ap2, Cd28, Cflar, Chl1, Dpp4, Dvl3, Ecm1, F3, Fadd, Fap, Hspa1b, Hspd1, Il1r1, Itgb1, Lcn2, Lonp2, Malt1, Ndufs7, Os9, Ryr1, Sell, Serpina1a, Serpina1d, Serpina1e, Serpinb6a, Serpinb9, Serpinc1, Serpine1, Sri, Timp1, Tnf, Tnfaip3, Tnfrsf1a, Trp53, Ttn, Xiap | 69 | protease binding |

## Help | Hide | Top Help | Show | Top KEGG Pathway test for over-representation

### HELP

List of all enriched KEGG pathways, at the 0.05
p-value level.

The columns:

- **ExpCount** is the expected count of genes in the
  module annotated with the given KEGG pathway, just by chance.
- **Count**
  is the number of genes in the module annotated with the given KEGG
  pathway.
- **Size** is the total number of genes (in our universe)
  annotated with the KEGG pathway.

Clicking on **Count** shows the genes that drive the
enrichment. You can also click on the individual numbers in
the **Count** column, to show the driving genes for that individual
KEGG pathway.

Clicking on the KEGG identifiers takes you to the KEGG web site.

— Click on the *Help* button again to close this help window.

| Id | Pvalue | ExpCount | Count | Size | Term |
| --- | --- | --- | --- | --- | --- |
| 05140 | 1.635e-03 | 18.4 | 35 C3, Cyba, Elk1, Fcgr1, Fos, H2-Ab1, H2-Ob, Ifngr2, Il10, Il1a, Il1b, Irak1, Itga4, Itgam, Itgb1, Itgb2, Jak1, Jun, Map3k7, Mapk1, Mapk3, Myd88, Ncf1, Ncf2, Ncf4, Nfkb1, Nfkbib, Ptgs2, Rela, Tgfb1, Tgfb2, Tgfb3, Tlr4, Tnf, Traf6 | 60 | Leishmaniasis |
| 05144 | 3.575e-02 | 13.19 | 25 Cd36, Cd40lg, Gypa, Gypc, Hbb-b2, Icam1, Il10, Il18, Il1b, Itgal, Itgb2, Myd88, Pecam1, Sdc4, Selp, Tgfb1, Tgfb2, Tgfb3, Thbs1, Thbs2, Thbs3, Thbs4, Tlr4, Tlr9, Tnf | 43 | Malaria |


### HELP

List of all enriched miRNA families, at the 0.05
p-value level.

The columns:

- **ExpCount** is the expected count of genes in the
  module regulated by the given miRNA family, just by chance.
- **Count**
  is the number of genes in the module regulated by the given miRNA
  family.
- **Size** is the total number of genes (in our universe)
  regulated with the given miRNA family.

Clicking on **Count** shows the genes that drive the
enrichment. You can also click on the individual numbers in
the **Count** column, to show the driving genes for that individual
miRNA family.

The miRNA regulation data was taken from the

Top


### HELP

p-value level.

The columns:

- **ExpCount** is the expected number of genes in the- **Count**- **Size** is the total number of genes (in our universe)

Clicking on **Count** shows the genes that drive the
enrichment. You can also click on the individual numbers in
the **Count** column, to show the driving genes for that individual

— Click on the *Help* button again to close this help window.

## Help | Hide | Top Help | Show | Top Genes

### HELP

A list of all genes in the current module, in alphabetical order. The
size of the text corresponds to the gene scores.

Note that some gene symbols may show up more than once, if many
probes match the same Entrez gene.

Genes with no Entrez mapping are given separately, with their
Affymetrics probe ID.

— Click on the *Help* button again to close this help window.

### Genes Symbol

, score:

AatkUnknown, score: 0.55
Abca1Unknown, score: 0.14
Abca2Unknown, score: 0.5
Abcb7Unknown, score: 0.23
Abcg1Unknown, score: 0.18
Slc33a1Unknown, score: 0.14
Asic1Unknown, score: 0.41
Apoc4Unknown, score: 0.2
Acp1Unknown, score: 0.53
Actg1Unknown, score: 0.18
Actg2Unknown, score: 0.16
Actn3Unknown, score: 0.74
Acta2Unknown, score: 0.75
Acvr1bUnknown, score: 0.3
AspaUnknown, score: 0.28
AdaUnknown, score: 0.2
Adam11Unknown, score: 0.49
Adam12Unknown, score: 0.16
Adam15Unknown, score: 0.22
Adam19Unknown, score: 0.47
Adam22Unknown, score: 0.28
Adam4Unknown, score: 0.34
Adam5Unknown, score: 0.57
Adcy6Unknown, score: 0.54
Adcy7Unknown, score: 0.35
Adcy9Unknown, score: 0.23
Adora2aUnknown, score: 0.21
Adra1dUnknown, score: 0.27
Adssl1Unknown, score: 0.24
AdssUnknown, score: 0.16
AvilUnknown, score: 0.44
AgaUnknown, score: 0.49
AcanUnknown, score: 0.21
AgrpUnknown, score: 0.5
Agtr1aUnknown, score: 0.14
AhrUnknown, score: 0.26
AhrrUnknown, score: 0.19
AhsgUnknown, score: 0.17
Akap1Unknown, score: 0.4
Akap2Unknown, score: 0.29
AlbUnknown, score: 0.26
Aldh2Unknown, score: 0.15
Aldh3a1Unknown, score: 0.16
Alox5Unknown, score: 0.25
AmbpUnknown, score: 0.24
AmhUnknown, score: 0.2
Mat1aUnknown, score: 0.29
Anxa11Unknown, score: 0.44
Anxa3Unknown, score: 0.36
Anxa7Unknown, score: 0.55
Anxa8Unknown, score: 0.21
Ap1b1Unknown, score: 0.28
Ap1g1Unknown, score: 0.28
Ap1m1Unknown, score: 0.23
Ap2a2Unknown, score: 0.22
Ap3d1Unknown, score: 0.48
Ap4s1Unknown, score: 0.21
Apbb2Unknown, score: 0.34
Apex1Unknown, score: 0.29
Atg5Unknown, score: 0.19
Birc2Unknown, score: 0.34
XiapUnknown, score: 0.33
Birc5Unknown, score: 0.16
Cd5lUnknown, score: 0.4
Apoa4Unknown, score: 0.16
Apobec1Unknown, score: 0.16
Apoc1Unknown, score: 0.22
Apoc3Unknown, score: 0.46
ApohUnknown, score: 0.22
Aqp1Unknown, score: 0.28
Aqp2Unknown, score: 0.55
Aqp4Unknown, score: 0.15
Aqp5Unknown, score: 0.29
Aqp8Unknown, score: 0.19
AqrUnknown, score: 0.29
ArcUnknown, score: 0.19
Arf5Unknown, score: 0.17
RhobUnknown, score: 0.17
RhodUnknown, score: 0.35
Arhgap6Unknown, score: 0.51
Arl4aUnknown, score: 0.19
Arnt2Unknown, score: 0.17
ArntlUnknown, score: 0.38
Art5Unknown, score: 0.21
ArsbUnknown, score: 0.22
ArsaUnknown, score: 0.18
Asgr2Unknown, score: 0.19
Rab27aUnknown, score: 0.38
Serpinc1Unknown, score: 0.51
Atf1Unknown, score: 0.21
Atf2Unknown, score: 0.42
Atf3Unknown, score: 0.28
Atf4Unknown, score: 0.2
Atox1Unknown, score: 0.19
Atp1b2Unknown, score: 0.28
Atp2a2Unknown, score: 0.21
Atp5kUnknown, score: 0.22
Atp6v0a1Unknown, score: 0.36
Atp8a1Unknown, score: 0.2
Atp9aUnknown, score: 0.37
Atp6v0cUnknown, score: 0.32
Slc7a1Unknown, score: 0.2
Aup1Unknown, score: 0.23
Avpr2Unknown, score: 0.45
Axin1Unknown, score: 0.18
Bak1Unknown, score: 0.22
Bcl2Unknown, score: 0.25
Bcl2l2Unknown, score: 0.31
Opn1swUnknown, score: 0.25
Bdkrb1Unknown, score: 0.17
Bglap3Unknown, score: 0.34
BhmtUnknown, score: 0.3
BlkUnknown, score: 0.31
Cxcr5Unknown, score: 0.21
Bmi1Unknown, score: 0.16
Bmp10Unknown, score: 0.26
Bmp4Unknown, score: 0.33
Bmp7Unknown, score: 0.15
Bmpr1bUnknown, score: 0.34
Bmpr2Unknown, score: 0.16
Bnc1Unknown, score: 0.15
Bnip2Unknown, score: 0.37
Bop1Unknown, score: 0.48
BpgmUnknown, score: 0.29
Brca1Unknown, score: 0.37
Zfp36l2Unknown, score: 0.15
Birc6Unknown, score: 0.26
Btg1Unknown, score: 0.22
Btg2Unknown, score: 0.15
Btg3Unknown, score: 0.27
BtrcUnknown, score: 0.36
Bub1Unknown, score: 0.28
Commd3Unknown, score: 0.18
TspoUnknown, score: 0.28
Serping1Unknown, score: 0.18
C2Unknown, score: 0.21
CiitaUnknown, score: 0.52
C3Unknown, score: 0.2
C3ar1Unknown, score: 0.18
C4bpUnknown, score: 0.16
C5ar1Unknown, score: 0.18
Cacna1bUnknown, score: 0.29
Cacna2d1Unknown, score: 0.27
Cacnb1Unknown, score: 0.23
Cacnb2Unknown, score: 0.34
Cacnb3Unknown, score: 0.17
Cacng1Unknown, score: 0.19
Pdia4Unknown, score: 0.16
Ddr1Unknown, score: 0.73
Anxa2Unknown, score: 0.25
Calm1Unknown, score: 0.25
Calm2Unknown, score: 0.25
Calm3Unknown, score: 0.49
Car8Unknown, score: 0.46
CanxUnknown, score: 0.62
Cap1Unknown, score: 0.47
Capn1Unknown, score: 0.15
Capns1Unknown, score: 0.24
Capn5Unknown, score: 0.27
Capn7Unknown, score: 0.27
Capza1Unknown, score: 0.25
Capza2Unknown, score: 0.21
CapzbUnknown, score: 0.28
Nr1i3Unknown, score: 0.29
CatUnknown, score: 0.18
CaskUnknown, score: 0.34
Casp1Unknown, score: 0.61
Casp4Unknown, score: 0.5
Casp2Unknown, score: 0.72
Casp3Unknown, score: 0.14
Casp7Unknown, score: 0.45
Casq2Unknown, score: 0.22
CasrUnknown, score: 0.27
Cav3Unknown, score: 0.46
Runx2Unknown, score: 0.44
Runx1t1Unknown, score: 0.17
Runx3Unknown, score: 0.22
Serpina6Unknown, score: 0.3
Cbx3Unknown, score: 0.21
Cbx4Unknown, score: 0.62
Cbx5Unknown, score: 0.34
Rb1cc1Unknown, score: 0.15
CckUnknown, score: 0.49
Ccna2Unknown, score: 0.33
Ccnd3Unknown, score: 0.33
Ccne1Unknown, score: 0.17
Ccne2Unknown, score: 0.28
CcnfUnknown, score: 0.34
Ccng2Unknown, score: 0.18
Ccnt1Unknown, score: 0.4
Cct2Unknown, score: 0.2
Cct8Unknown, score: 0.32
Cd22Unknown, score: 0.18
Cd28Unknown, score: 0.49
Cd2apUnknown, score: 0.29
Cd33Unknown, score: 0.94
Cd36Unknown, score: 0.44
Cd37Unknown, score: 0.16
Cd38Unknown, score: 0.53
Cd3dUnknown, score: 0.32
Cd4Unknown, score: 0.76
Cd44Unknown, score: 0.23
Cd5Unknown, score: 0.48
Cd63Unknown, score: 0.3
Cd7Unknown, score: 0.2
Cd79aUnknown, score: 0.66
Cd83Unknown, score: 0.54
Cd8b1Unknown, score: 0.47
Cdc25aUnknown, score: 0.23
Cdc25bUnknown, score: 0.29
Cdc25cUnknown, score: 0.46
Cdc45Unknown, score: 0.25
Cdc7Unknown, score: 0.2
Arhgap31Unknown, score: 0.31
Cdh1Unknown, score: 0.34
Cdh15Unknown, score: 0.14
Cdh2Unknown, score: 0.27
Cdk5r1Unknown, score: 0.27
Cdk7Unknown, score: 0.53
Cdkn1cUnknown, score: 0.19
Cdkn2cUnknown, score: 0.67
Cdo1Unknown, score: 0.34
Cdr2Unknown, score: 0.28
Ift81Unknown, score: 0.29
CebpaUnknown, score: 0.16
CebpbUnknown, score: 0.22
Cenpc1Unknown, score: 0.26
Cetn3Unknown, score: 0.34
CfhUnknown, score: 0.18
CfiUnknown, score: 0.21
Cfl2Unknown, score: 0.3
CflarUnknown, score: 0.32
ChadUnknown, score: 0.38
ChkbUnknown, score: 0.17
ChgbUnknown, score: 0.35
Chil1Unknown, score: 0.2
Chl1Unknown, score: 0.49
ChmUnknown, score: 0.21
ChmlUnknown, score: 0.18
CishUnknown, score: 0.43
Socs1Unknown, score: 0.19
Cited1Unknown, score: 0.29
CkmUnknown, score: 0.3
Coro1aUnknown, score: 0.46
Clcn4-2Unknown, score: 0.42
Clns1aUnknown, score: 0.16
Cldn1Unknown, score: 0.21
Cldn2Unknown, score: 0.17
Cln3Unknown, score: 0.28
Cxcr4Unknown, score: 0.41
Ccr1l1Unknown, score: 0.41
Ccr4Unknown, score: 0.27
Ackr3Unknown, score: 0.15
Plk3Unknown, score: 0.46
Cnn1Unknown, score: 0.29
CoilUnknown, score: 0.14
Col10a1Unknown, score: 0.36
Col11a2Unknown, score: 0.65
Col13a1Unknown, score: 0.81
Col14a1Unknown, score: 0.35
Col17a1Unknown, score: 0.2
Col18a1Unknown, score: 0.24
Col19a1Unknown, score: 0.54
Col3a1Unknown, score: 0.33
Col4a1Unknown, score: 0.26
Col4a2Unknown, score: 0.27
Col4a3Unknown, score: 0.24
Col4a4Unknown, score: 0.3
Col4a5Unknown, score: 0.38
Col5a1Unknown, score: 0.72
Col5a2Unknown, score: 0.53
Col6a1Unknown, score: 0.28
Col6a2Unknown, score: 0.57
Col7a1Unknown, score: 0.81
Col8a1Unknown, score: 0.18
Col9a3Unknown, score: 0.59
Col1a1Unknown, score: 0.47
CopaUnknown, score: 0.27
CpUnknown, score: 0.26
Cpa3Unknown, score: 0.17
Cpeb1Unknown, score: 0.23
Cplx1Unknown, score: 0.33
Cr2Unknown, score: 0.23
Crabp1Unknown, score: 0.33
CrcpUnknown, score: 0.29
Creb1Unknown, score: 0.33
Atf6bUnknown, score: 0.67
Crhr2Unknown, score: 0.19
CrkUnknown, score: 0.28
CrklUnknown, score: 0.72
Crlf1Unknown, score: 0.18
Dpysl2Unknown, score: 0.2
Pcdha4Unknown, score: 0.2
Pcdha11Unknown, score: 0.2
Cr1lUnknown, score: 0.22
Cry1Unknown, score: 0.19
Crybb3Unknown, score: 0.33
Csf1rUnknown, score: 0.29
Csf2raUnknown, score: 0.65
Csf2rbUnknown, score: 0.63
Csf2rb2Unknown, score: 0.19
Csf3rUnknown, score: 0.45
Csnk2a1Unknown, score: 0.3
Dnajc5Unknown, score: 0.79
Smc3Unknown, score: 0.34
Csrp1Unknown, score: 0.28
Ctbp1Unknown, score: 0.16
Ctf1Unknown, score: 0.78
Ctla2aUnknown, score: 0.5
CtscUnknown, score: 0.21
CtsdUnknown, score: 0.28
CtseUnknown, score: 0.52
CtshUnknown, score: 0.41
CtskUnknown, score: 0.4
CtslUnknown, score: 0.49
CtswUnknown, score: 0.28
CttnUnknown, score: 0.21
CxadrUnknown, score: 0.18
CybaUnknown, score: 0.15
CyctUnknown, score: 0.15
Cyp1a2Unknown, score: 0.21
Cyp1b1Unknown, score: 0.22
Cyp21a1Unknown, score: 0.22
Cyp4b1Unknown, score: 0.22
Cyp7a1Unknown, score: 0.41
Cyp7b1Unknown, score: 0.18
Cyp8b1Unknown, score: 0.31
Dab2Unknown, score: 0.22
Dad1Unknown, score: 0.78
Dbil5Unknown, score: 0.2
DbtUnknown, score: 0.75
Eci1Unknown, score: 0.2
DckUnknown, score: 0.19
DcnUnknown, score: 0.56
Dscr3Unknown, score: 0.92
DdcUnknown, score: 0.15
Dhx15Unknown, score: 0.33
DesUnknown, score: 0.14
DffaUnknown, score: 0.45
Dhcr7Unknown, score: 0.3
DhfrUnknown, score: 0.16
DffbUnknown, score: 0.95
Dio2Unknown, score: 0.27
Mpp3Unknown, score: 0.24
Dlx1Unknown, score: 0.23
Dlx4Unknown, score: 0.45
Dmc1Unknown, score: 0.25
DmdUnknown, score: 0.15
Dnah11Unknown, score: 0.29
Dnah8Unknown, score: 0.32
Dync1h1Unknown, score: 0.25
Dync1i2Unknown, score: 0.39
Dnm2Unknown, score: 0.3
Dnmt3aUnknown, score: 0.16
Doc2bUnknown, score: 0.33
Dpm2Unknown, score: 0.14
Dpp4Unknown, score: 0.74
Atn1Unknown, score: 0.19
Dsc1Unknown, score: 0.15
Dsg1aUnknown, score: 0.15
DstUnknown, score: 0.22
Dvl3Unknown, score: 0.24
E2f1Unknown, score: 0.27
Lefty1Unknown, score: 0.4
Ebf1Unknown, score: 0.47
Ebf2Unknown, score: 0.22
EbpUnknown, score: 0.46
Ecm1Unknown, score: 0.36
Opn3Unknown, score: 0.47
Ect2Unknown, score: 0.19
EdarUnknown, score: 0.22
Edil3Unknown, score: 0.33
Edn3Unknown, score: 0.6
EdnraUnknown, score: 0.23
EedUnknown, score: 0.34
Efna2Unknown, score: 0.18
Efna3Unknown, score: 0.19
Efnb2Unknown, score: 0.31
Efnb3Unknown, score: 0.31
EgfUnknown, score: 0.16
Klk1b26Unknown, score: 0.33
Egr1Unknown, score: 0.42
Egr2Unknown, score: 0.23
Ehd1Unknown, score: 0.29
EhfUnknown, score: 0.16
Ei24Unknown, score: 0.42
Eif2b4Unknown, score: 0.43
Elk1Unknown, score: 0.25
EllUnknown, score: 0.37
Aimp1Unknown, score: 0.44
Mark2Unknown, score: 0.66
Emp1Unknown, score: 0.18
Emp2Unknown, score: 0.21
Emp3Unknown, score: 0.23
En2Unknown, score: 0.52
EnahUnknown, score: 0.31
EndogUnknown, score: 0.32
EngUnknown, score: 0.19
Eno1Unknown, score: 0.28
Epas1Unknown, score: 0.41
Epb4.1l1Unknown, score: 0.58
Epb4.1l2Unknown, score: 0.17
Epha3Unknown, score: 0.15
Epha8Unknown, score: 0.17
Ephb3Unknown, score: 0.17
Ephb6Unknown, score: 0.22
Ephx2Unknown, score: 0.29
Epm2aUnknown, score: 0.2
EporUnknown, score: 0.21
Eps15l1Unknown, score: 0.42
Ercc3Unknown, score: 0.76
ErfUnknown, score: 0.18
Gnb1lUnknown, score: 0.2
Esr1Unknown, score: 0.26
Khdrbs3Unknown, score: 0.43
Fgd6Unknown, score: 0.34
Celf2Unknown, score: 0.36
Etv1Unknown, score: 0.28
Etv6Unknown, score: 0.4
Mpzl2Unknown, score: 0.2
Evi2aUnknown, score: 0.21
Evi5Unknown, score: 0.2
Wfdc18Unknown, score: 0.17
Ext1Unknown, score: 0.19
Eya3Unknown, score: 0.33
Ezh1Unknown, score: 0.29
Ezh2Unknown, score: 0.23
Sfxn1Unknown, score: 0.32
F2Unknown, score: 0.32
F2rUnknown, score: 0.25
F2rl1Unknown, score: 0.15
F3Unknown, score: 0.19
F5Unknown, score: 0.31
F7Unknown, score: 0.62
FaddUnknown, score: 0.3
Fscn1Unknown, score: 0.15
FanccUnknown, score: 0.3
FapUnknown, score: 0.48
Foxh1Unknown, score: 0.17
FblUnknown, score: 0.29
Fbln1Unknown, score: 0.64
Fbln2Unknown, score: 0.6
Ms4a2Unknown, score: 0.21
Fcgr1Unknown, score: 0.62
Fcgr2bUnknown, score: 0.6
Fem1aUnknown, score: 0.3
Fen1Unknown, score: 0.42
Fgf10Unknown, score: 0.34
Fgf11Unknown, score: 0.18
Fgf13Unknown, score: 0.23
Fgf6Unknown, score: 0.65
Fgf7Unknown, score: 0.44
Fgfbp1Unknown, score: 0.23
Fgfr4Unknown, score: 0.63
Akr1b8Unknown, score: 0.94
Fgl2Unknown, score: 0.25
FhitUnknown, score: 0.73
Fhl1Unknown, score: 0.16
Fhl3Unknown, score: 0.24
Il4i1Unknown, score: 0.54
Ppm1gUnknown, score: 0.54
Sh3pxd2aUnknown, score: 0.17
CtgfUnknown, score: 0.16
Fjx1Unknown, score: 0.16
Fkbp1aUnknown, score: 0.27
Fkbp10Unknown, score: 0.45
Fkbp8Unknown, score: 0.29
Foxc2Unknown, score: 0.33
Foxm1Unknown, score: 0.33
Foxn2Unknown, score: 0.21
Foxf2Unknown, score: 0.18
Flt1Unknown, score: 0.34
Flt4Unknown, score: 0.19
Fmo1Unknown, score: 0.14
Fmo3Unknown, score: 0.2
Fmo5Unknown, score: 0.19
FmodUnknown, score: 0.24
Fmr1Unknown, score: 0.37
Aff2Unknown, score: 0.85
Folr2Unknown, score: 0.37
FosUnknown, score: 0.18
FosbUnknown, score: 0.18
Fosl1Unknown, score: 0.6
FpgsUnknown, score: 0.31
Fpr2Unknown, score: 0.42
FxnUnknown, score: 0.28
Frg1Unknown, score: 0.34
Brd2Unknown, score: 0.21
FstUnknown, score: 0.84
Fut7Unknown, score: 0.29
Dtx1Unknown, score: 0.22
FynUnknown, score: 0.32
Fzd3Unknown, score: 0.26
G6pcUnknown, score: 0.22
Gab1Unknown, score: 0.33
Gabra1Unknown, score: 0.53
Gabrb2Unknown, score: 0.16
GabrdUnknown, score: 0.37
Gabrg3Unknown, score: 0.28
Gabrr2Unknown, score: 0.25
GalUnknown, score: 0.2
GalcUnknown, score: 0.38
Galnt3Unknown, score: 0.26
GartUnknown, score: 0.51
Gas6Unknown, score: 0.44
Gata2Unknown, score: 0.2
Gata4Unknown, score: 0.18
Gata6Unknown, score: 0.41
GbasUnknown, score: 0.28
Gbp2bUnknown, score: 0.2
Usp15Unknown, score: 0.27
MtpnUnknown, score: 0.2
Gcnt1Unknown, score: 0.75
Gcnt2Unknown, score: 0.72
Gdap1Unknown, score: 0.15
Gdf10Unknown, score: 0.23
Gdf3Unknown, score: 0.33
Gdi2Unknown, score: 0.37
GdnfUnknown, score: 0.43
GemUnknown, score: 0.31
Gfpt1Unknown, score: 0.83
Gfra1Unknown, score: 0.18
Gfra4Unknown, score: 0.17
Ggps1Unknown, score: 0.47
Gjb3Unknown, score: 0.2
Gjb5Unknown, score: 0.43
GclmUnknown, score: 0.22
Gli1Unknown, score: 0.42
Gli2Unknown, score: 0.57
Galk1Unknown, score: 0.17
GlsUnknown, score: 0.28
Slc6a9Unknown, score: 0.21
Gna12Unknown, score: 0.24
Gnai1Unknown, score: 0.15
Gnai2Unknown, score: 0.61
GnalUnknown, score: 0.31
GnasUnknown, score: 0.3
Gnb5Unknown, score: 0.17
Gng12Unknown, score: 0.17
Bscl2Unknown, score: 0.23
Gng5Unknown, score: 0.4
Gng7Unknown, score: 0.41
Gng8Unknown, score: 0.28
Lrp2Unknown, score: 0.84
PdpnUnknown, score: 0.61
Gp49aUnknown, score: 0.62
Gpr12Unknown, score: 0.26
Gpr65Unknown, score: 0.37
Lpar1Unknown, score: 0.44
Gpld1Unknown, score: 0.54
Lancl1Unknown, score: 0.16
Grk4Unknown, score: 0.88
Grk5Unknown, score: 0.4
Gpx1Unknown, score: 0.43
Gpr162Unknown, score: 0.16
Cdca3Unknown, score: 0.38
AesUnknown, score: 0.22
Gria1Unknown, score: 0.15
Gria4Unknown, score: 0.19
Grik5Unknown, score: 0.5
Grin2aUnknown, score: 0.48
Grin2cUnknown, score: 0.21
GrnUnknown, score: 0.24
Hspa5Unknown, score: 0.44
Gspt1Unknown, score: 0.37
Nkx6-2Unknown, score: 0.23
Guk1Unknown, score: 0.36
GypaUnknown, score: 0.24
Gys1Unknown, score: 0.19
GzmbUnknown, score: 0.15
H2-Ab1Unknown, score: 0.26
H2-D1Unknown, score: 0.19
H2-ObUnknown, score: 0.24
H2-T3Unknown, score: 0.5
H2-T22Unknown, score: 0.48
H2-T23Unknown, score: 0.14
H2-T3Unknown, score: 0.5
H2-T22Unknown, score: 0.2
15061Unknown, score: 0.44
Mr1Unknown, score: 0.3
HalUnknown, score: 0.59
Hao1Unknown, score: 0.23
HarsUnknown, score: 0.5
Has1Unknown, score: 0.55
Hbb-bsUnknown, score: 0.15
HcUnknown, score: 0.24
HckUnknown, score: 0.29
Hcls1Unknown, score: 0.28
Hdac6Unknown, score: 0.45
HdcUnknown, score: 0.2
HbegfUnknown, score: 0.24
Hes1Unknown, score: 0.14
HexbUnknown, score: 0.27
Hey2Unknown, score: 0.42
HgdUnknown, score: 0.43
Hint1Unknown, score: 0.25
Hipk3Unknown, score: 0.29
H2afxUnknown, score: 0.4
Hivep2Unknown, score: 0.14
Hmga2Unknown, score: 0.43
HmmrUnknown, score: 0.45
Hmox2Unknown, score: 0.57
HnrnplUnknown, score: 0.15
Hoxa6Unknown, score: 0.14
Hoxd4Unknown, score: 0.23
Hoxd9Unknown, score: 0.38
Hp1bp3Unknown, score: 0.29
HpnUnknown, score: 0.33
HrasUnknown, score: 0.22
Hrh2Unknown, score: 0.39
Hrsp12Unknown, score: 0.37
Hspa8Unknown, score: 0.41
Hsd17b4Unknown, score: 0.48
Dnajb3Unknown, score: 0.18
Hspd1Unknown, score: 0.22
Hspa1bUnknown, score: 0.46
Hsp90ab1Unknown, score: 0.37
Hsp90aa1Unknown, score: 0.15
Htr2aUnknown, score: 0.3
Htr2bUnknown, score: 0.22
Slc6a4Unknown, score: 0.21
Elavl2Unknown, score: 0.15
Hyal2Unknown, score: 0.3
Ica1Unknown, score: 0.53
Icam1Unknown, score: 0.15
Id1Unknown, score: 0.2
Cxcl10Unknown, score: 0.35
Ifi203Unknown, score: 0.27
Ifi204Unknown, score: 0.29
Ifi47Unknown, score: 0.21
Ifit2Unknown, score: 0.17
Ifnar2Unknown, score: 0.15
Ifngr2Unknown, score: 0.32
Cd79bUnknown, score: 0.17
Igf1Unknown, score: 0.37
Igfbp4Unknown, score: 0.54
Igfbp5Unknown, score: 0.38
Il10Unknown, score: 0.19
Il16Unknown, score: 0.28
Il18Unknown, score: 0.23
Il1aUnknown, score: 0.28
Il1bUnknown, score: 0.62
Il1r1Unknown, score: 0.31
Irak1Unknown, score: 0.32
Il1rapUnknown, score: 0.78
Il1rnUnknown, score: 0.22
Il2rbUnknown, score: 0.46
Il3raUnknown, score: 0.4
Il5raUnknown, score: 0.4
Il7Unknown, score: 0.28
Lrig1Unknown, score: 0.65
Kpnb1Unknown, score: 0.48
InhaUnknown, score: 0.56
InhbaUnknown, score: 0.24
Inpp5bUnknown, score: 0.25
InvsUnknown, score: 0.17
Irf1Unknown, score: 0.48
Irf4Unknown, score: 0.26
Irf9Unknown, score: 0.37
ItchUnknown, score: 0.43
Itga3Unknown, score: 0.42
Itga4Unknown, score: 0.15
ItgaeUnknown, score: 0.33
ItgalUnknown, score: 0.65
ItgamUnknown, score: 0.34
ItgavUnknown, score: 0.6
ItgaxUnknown, score: 0.35
Itgb1Unknown, score: 0.25
Itgb1bp1Unknown, score: 0.5
Itgb2Unknown, score: 0.39
Itih1Unknown, score: 0.19
Itih3Unknown, score: 0.3
ItkUnknown, score: 0.53
Stt3aUnknown, score: 0.19
Itm2bUnknown, score: 0.77
Itpr1Unknown, score: 0.25
Itpr3Unknown, score: 0.16
Itsn1Unknown, score: 0.19
Jag2Unknown, score: 0.38
Jak1Unknown, score: 0.17
JunUnknown, score: 0.15
Kcna2Unknown, score: 0.19
Kcnab1Unknown, score: 0.32
Kcnab2Unknown, score: 0.3
Kcnb1Unknown, score: 0.31
Kcnc1Unknown, score: 0.38
Kcnd1Unknown, score: 0.22
Kcnd2Unknown, score: 0.33
Kcne1Unknown, score: 0.34
Kcnj12Unknown, score: 0.32
Kcnj16Unknown, score: 0.34
Kcnj2Unknown, score: 0.27
Kcnk4Unknown, score: 0.19
Kcnk7Unknown, score: 0.2
Kcnma1Unknown, score: 0.18
Kcnu1Unknown, score: 0.45
Kcnn4Unknown, score: 0.16
KdrUnknown, score: 0.31
KhsrpUnknown, score: 0.71
Kif17Unknown, score: 0.28
Kif1aUnknown, score: 0.28
Kif1bUnknown, score: 0.19
Kif1cUnknown, score: 0.14
Kif21bUnknown, score: 0.36
Kif3aUnknown, score: 0.36
Kif3bUnknown, score: 0.39
Kif3cUnknown, score: 0.37
Kif4Unknown, score: 0.25
Kif5aUnknown, score: 0.74
Kif5bUnknown, score: 0.23
Kif7Unknown, score: 0.4
KinUnknown, score: 0.27
Klf1Unknown, score: 0.16
Klf2Unknown, score: 0.31
Klkb1Unknown, score: 0.23
Klra10Unknown, score: 0.19
CU424478.2Unknown, score: 0.15
Klrc1Unknown, score: 0.29
Kng1Unknown, score: 0.22
Kpna1Unknown, score: 0.3
Kpna2Unknown, score: 0.2
Kpna3Unknown, score: 0.14
SspnUnknown, score: 0.34
KrasUnknown, score: 0.28
Krt10Unknown, score: 0.3
Krt14Unknown, score: 0.59
Krt18Unknown, score: 0.45
Krt19Unknown, score: 0.24
Ksr1Unknown, score: 0.31
L1camUnknown, score: 0.24
Lad1Unknown, score: 0.4
Stmn1Unknown, score: 0.27
Lag3Unknown, score: 0.25
Lama3Unknown, score: 0.23
Lamp1Unknown, score: 0.14
RpsaUnknown, score: 0.62
AnpepUnknown, score: 0.44
LatUnknown, score: 0.24
Arhgef1Unknown, score: 0.42
LbpUnknown, score: 0.21
LckUnknown, score: 0.29
Lcn2Unknown, score: 0.21
Ldb1Unknown, score: 0.51
Lect1Unknown, score: 0.2
Lgals1Unknown, score: 0.73
Lgals4Unknown, score: 0.34
Lhx2Unknown, score: 0.14
Lhx6Unknown, score: 0.18
LifrUnknown, score: 0.26
Lig1Unknown, score: 0.21
Lig3Unknown, score: 0.15
Limk2Unknown, score: 0.34
GzmmUnknown, score: 0.25
LmnaUnknown, score: 0.16
Lmo2Unknown, score: 0.27
Psmb8Unknown, score: 0.15
LorUnknown, score: 0.6
LoxUnknown, score: 0.15
Loxl1Unknown, score: 0.4
Loxl3Unknown, score: 0.6
Xcl1Unknown, score: 0.24
LrmpUnknown, score: 0.25
Lrp5Unknown, score: 0.34
Lrpap1Unknown, score: 0.15
Lrrn1Unknown, score: 0.25
Lrrn3Unknown, score: 0.56
Ltb4r1Unknown, score: 0.79
Ltbp2Unknown, score: 0.39
Ltbp3Unknown, score: 0.22
LtkUnknown, score: 0.52
AladUnknown, score: 0.47
LxnUnknown, score: 0.17
Muc13Unknown, score: 0.22
LynUnknown, score: 0.42
LystUnknown, score: 0.42
Lyz2Unknown, score: 0.52
AmacrUnknown, score: 0.26
Mad1l1Unknown, score: 0.19
Smad1Unknown, score: 0.27
Smad4Unknown, score: 0.18
Smad5Unknown, score: 0.19
Smad6Unknown, score: 0.31
MafUnknown, score: 0.32
MagohUnknown, score: 0.44
Mfap2Unknown, score: 0.29
MalUnknown, score: 0.74
Man1aUnknown, score: 0.35
Man1a2Unknown, score: 0.63
Man2b2Unknown, score: 0.4
MaoaUnknown, score: 0.33
Mapkapk2Unknown, score: 0.5
Mapkapk5Unknown, score: 0.14
Masp2Unknown, score: 0.17
MatkUnknown, score: 0.58
Matr3Unknown, score: 0.3
Mcl1Unknown, score: 0.25
Mcm4Unknown, score: 0.21
Anapc1Unknown, score: 0.21
Mcpt2Unknown, score: 0.26
Mcpt4Unknown, score: 0.17
Cma1Unknown, score: 0.24
MdfiUnknown, score: 0.19
MdkUnknown, score: 0.22
Mdm1Unknown, score: 0.33
Abcc1Unknown, score: 0.27
Meis1Unknown, score: 0.2
Rab8aUnknown, score: 0.21
Men1Unknown, score: 0.55
MertkUnknown, score: 0.41
MestUnknown, score: 0.42
MfngUnknown, score: 0.4
Mgat3Unknown, score: 0.2
Clec10aUnknown, score: 0.19
Pias2Unknown, score: 0.59
Mllt4Unknown, score: 0.37
Trpm1Unknown, score: 0.28
MmeUnknown, score: 0.55
Mmp13Unknown, score: 0.54
Mmp15Unknown, score: 0.39
Mmp16Unknown, score: 0.5
Mmp24Unknown, score: 0.63
Mmp3Unknown, score: 0.23
Foxk1Unknown, score: 0.23
Cd200Unknown, score: 0.25
Gbp4Unknown, score: 0.8
MpdzUnknown, score: 0.44
MplUnknown, score: 0.17
MpzUnknown, score: 0.47
MrasUnknown, score: 0.48
Mrc1Unknown, score: 0.69
Msh2Unknown, score: 0.29
Msh3Unknown, score: 0.44
Msi1Unknown, score: 0.63
Msl3Unknown, score: 0.46
MsnUnknown, score: 0.42
Msx1Unknown, score: 0.24
Msx2Unknown, score: 0.17
mt-Atp6Unknown, score: 0.19
mt-Co2Unknown, score: 0.15
Grpel1Unknown, score: 0.5
mt-Nd6Unknown, score: 0.56
Polr2kUnknown, score: 0.59
Mt2Unknown, score: 0.24
Mt3Unknown, score: 0.27
Map1aUnknown, score: 0.62
Map1bUnknown, score: 0.29
Map2Unknown, score: 0.35
Map4Unknown, score: 0.41
MaptUnknown, score: 0.2
Nudt1Unknown, score: 0.25
Mtl5Unknown, score: 0.21
Bloc1s5Unknown, score: 0.27
Commd1Unknown, score: 0.24
MybUnknown, score: 0.5
Mybl1Unknown, score: 0.2
Mybpc3Unknown, score: 0.17
MycUnknown, score: 0.52
Myd88Unknown, score: 0.23
Myh1Unknown, score: 0.14
Myh8Unknown, score: 0.23
Myh6Unknown, score: 0.32
Myl7Unknown, score: 0.32
Myo1cUnknown, score: 0.27
Myo1fUnknown, score: 0.63
Myom2Unknown, score: 0.21
Ppp1r12aUnknown, score: 0.51
Nab1Unknown, score: 0.34
NagaUnknown, score: 0.15
Naip1Unknown, score: 0.32
Nap1l4Unknown, score: 0.19
Nat1Unknown, score: 0.27
Nat2Unknown, score: 0.59
Ncam2Unknown, score: 0.37
Ncf1Unknown, score: 0.41
Ncf2Unknown, score: 0.26
Ncf4Unknown, score: 0.32
Nck2Unknown, score: 0.15
NclUnknown, score: 0.29
Ncoa3Unknown, score: 0.29
Ndufv1Unknown, score: 0.39
Nedd8Unknown, score: 0.31
Nedd9Unknown, score: 0.14
Nek1Unknown, score: 0.37
Neo1Unknown, score: 0.2
Neu1Unknown, score: 0.23
Nf1Unknown, score: 0.33
Nf2Unknown, score: 0.19
Nfatc2ipUnknown, score: 0.15
Nfe2l1Unknown, score: 0.15
NficUnknown, score: 0.27
Nfkb1Unknown, score: 0.14
NfkbibUnknown, score: 0.48
Nfkbil1Unknown, score: 0.26
NeflUnknown, score: 0.24
NfyaUnknown, score: 0.22
NfycUnknown, score: 0.17
NgfUnknown, score: 0.17
NgpUnknown, score: 0.32
Nid1Unknown, score: 0.28
Nid2Unknown, score: 0.53
Nkx2-5Unknown, score: 0.21
Nqo1Unknown, score: 0.33
Nmt1Unknown, score: 0.46
NnatUnknown, score: 0.35
NogUnknown, score: 0.82
Nr4a3Unknown, score: 0.19
NovUnknown, score: 0.34
ZfmlUnknown, score: 0.22
Npr3Unknown, score: 0.35
Ctnnd2Unknown, score: 0.17
Npy1rUnknown, score: 0.2
Slc11a1Unknown, score: 0.18
NrapUnknown, score: 0.27
NrtnUnknown, score: 0.22
Nrxn2Unknown, score: 0.3
Nsd1Unknown, score: 0.37
NsfUnknown, score: 0.29
Nsg2Unknown, score: 0.25
NsmafUnknown, score: 0.25
Ntn3Unknown, score: 0.31
NudcUnknown, score: 0.53
Nup62Unknown, score: 0.58
NxnUnknown, score: 0.29
OgnUnknown, score: 0.67
Fxyd5Unknown, score: 0.2
Oit3Unknown, score: 0.22
Olfr33Unknown, score: 0.22
OmpUnknown, score: 0.58
Sqstm1Unknown, score: 0.16
OsmUnknown, score: 0.63
OsmrUnknown, score: 0.15
OtcUnknown, score: 0.25
OtogUnknown, score: 0.5
P2rx4Unknown, score: 0.39
Bloc1s6Unknown, score: 0.19
Pabpc1Unknown, score: 0.15
Prdx1Unknown, score: 0.18
PahUnknown, score: 0.42
Pak1Unknown, score: 0.66
Pak3Unknown, score: 0.22
PalmUnknown, score: 0.42
Pax5Unknown, score: 0.16
Pbx2Unknown, score: 0.27
Pcbp2Unknown, score: 0.33
Pck1Unknown, score: 0.19
PcntUnknown, score: 0.55
Pcp4Unknown, score: 0.25
Pcsk2Unknown, score: 0.33
Pdcd2Unknown, score: 0.28
Pdcd11Unknown, score: 0.53
Pde1cUnknown, score: 0.52
Pde3bUnknown, score: 0.19
Pde4aUnknown, score: 0.56
Pde7aUnknown, score: 0.38
Pde8aUnknown, score: 0.65
PdgfaUnknown, score: 0.22
PdgfbUnknown, score: 0.44
Pdha1Unknown, score: 0.31
Padi3Unknown, score: 0.4
Pdk2Unknown, score: 0.34
Pea15aUnknown, score: 0.24
Etv4Unknown, score: 0.38
Pecam1Unknown, score: 0.62
Pex7Unknown, score: 0.46
Prf1Unknown, score: 0.39
Cdk14Unknown, score: 0.28
Abcb4Unknown, score: 0.16
PhexUnknown, score: 0.18
Phf2Unknown, score: 0.31
Phka1Unknown, score: 0.47
Phkg1Unknown, score: 0.35
PigrUnknown, score: 0.56
Pik3c2gUnknown, score: 0.21
Pik3r2Unknown, score: 0.26
Pik3r3Unknown, score: 0.29
Pip5k1cUnknown, score: 0.31
Pip5k1bUnknown, score: 0.63
Pip5k1aUnknown, score: 0.56
18722Unknown, score: 0.56
Gm14548Unknown, score: 0.34
Pira2Unknown, score: 0.33
Gm15448Unknown, score: 0.56
Gm14548Unknown, score: 0.56
Gm14548Unknown, score: 0.56
Gm14548Unknown, score: 0.4
Pou1f1Unknown, score: 0.32
PrkchUnknown, score: 0.15
Prkd1Unknown, score: 0.34
PrkczUnknown, score: 0.16
Pkd1Unknown, score: 0.15
Lypla1Unknown, score: 0.26
Pla2r1Unknown, score: 0.33
Pla2g2dUnknown, score: 0.56
Pla2g4aUnknown, score: 0.17
Serpine1Unknown, score: 0.39
PapolaUnknown, score: 0.46
PlatUnknown, score: 0.29
PlauUnknown, score: 0.24
PlaurUnknown, score: 0.28
Plcb3Unknown, score: 0.61
Pld1Unknown, score: 0.29
Pld2Unknown, score: 0.21
PlecUnknown, score: 0.21
Plp2Unknown, score: 0.46
Plxna2Unknown, score: 0.16
Plxna3Unknown, score: 0.19
Pmp2Unknown, score: 0.29
Phox2bUnknown, score: 0.24
PnmtUnknown, score: 0.15
PnnUnknown, score: 0.15
PnpUnknown, score: 0.5
PoleUnknown, score: 0.19
Pon1Unknown, score: 0.47
PorUnknown, score: 0.15
Pou2f2Unknown, score: 0.19
Pou4f1Unknown, score: 0.19
Pou6f1Unknown, score: 0.33
PparaUnknown, score: 0.57
PpargUnknown, score: 0.41
PpibUnknown, score: 0.29
PpicUnknown, score: 0.32
PplUnknown, score: 0.3
Ppp3caUnknown, score: 0.18
Ppp3cbUnknown, score: 0.21
PrepUnknown, score: 0.48
SrgnUnknown, score: 0.2
Prim1Unknown, score: 0.29
Prkar1aUnknown, score: 0.24
Prkar1bUnknown, score: 0.25
Prkar2aUnknown, score: 0.33
PrkdcUnknown, score: 0.66
Eif2ak2Unknown, score: 0.3
PrkxUnknown, score: 0.82
PrlrUnknown, score: 0.24
Prom1Unknown, score: 0.27
Prox1Unknown, score: 0.26
PrphUnknown, score: 0.41
Prps1Unknown, score: 0.3
Prss12Unknown, score: 0.14
Cyth2Unknown, score: 0.22
Cyth3Unknown, score: 0.18
Psme3Unknown, score: 0.45
PspnUnknown, score: 0.16
Pstpip1Unknown, score: 0.33
Pstpip2Unknown, score: 0.2
Ptch1Unknown, score: 0.23
PtgdsUnknown, score: 0.26
Ptger1Unknown, score: 0.31
Ptger2Unknown, score: 0.17
Ptger3Unknown, score: 0.43
PtgfrUnknown, score: 0.17
PtgfrnUnknown, score: 0.24
PtgirUnknown, score: 0.62
Ptgs1Unknown, score: 0.52
Ptgs2Unknown, score: 0.3
Pth1rUnknown, score: 0.42
Ptk2bUnknown, score: 0.59
Twf1Unknown, score: 0.35
PtmaUnknown, score: 0.4
Tmsb4xUnknown, score: 0.4
PtnUnknown, score: 0.2
Ptp4a1Unknown, score: 0.34
Ptp4a3Unknown, score: 0.23
Ptpn11Unknown, score: 0.31
Ptpn18Unknown, score: 0.51
PtpraUnknown, score: 0.32
PtprbUnknown, score: 0.28
PtprcapUnknown, score: 0.19
PtpreUnknown, score: 0.46
PtprfUnknown, score: 0.17
PtprgUnknown, score: 0.33
PtprjUnknown, score: 0.49
PtprkUnknown, score: 0.28
PtprnUnknown, score: 0.24
PtprrUnknown, score: 0.25
PtprsUnknown, score: 0.45
Ptprz1Unknown, score: 0.16
PtsUnknown, score: 0.46
PygmUnknown, score: 0.26
Rab12Unknown, score: 0.33
Rab17Unknown, score: 0.18
Rab3aUnknown, score: 0.36
Dennd5aUnknown, score: 0.52
Kif20aUnknown, score: 0.23
RabggtbUnknown, score: 0.28
Rac1Unknown, score: 0.2
Rad1Unknown, score: 0.15
Rad17Unknown, score: 0.2
Rad23bUnknown, score: 0.55
Rad51Unknown, score: 0.24
Rad9aUnknown, score: 0.41
Raet1dUnknown, score: 0.41
Raet1dUnknown, score: 0.18
Rai1Unknown, score: 0.56
Ranbp1Unknown, score: 0.35
RapsnUnknown, score: 0.4
RaraUnknown, score: 0.2
Rasal1Unknown, score: 0.21
Rasl2-9Unknown, score: 0.32
Rb1Unknown, score: 0.14
Rbbp4Unknown, score: 0.21
Robo3Unknown, score: 0.32
Rbl2Unknown, score: 0.17
Rbmxl1Unknown, score: 0.46
Rdh5Unknown, score: 0.17
Rdh16Unknown, score: 0.21
RelUnknown, score: 0.17
RelaUnknown, score: 0.14
RelbUnknown, score: 0.29
Rem1Unknown, score: 0.37
Bex1Unknown, score: 0.25
Rfx1Unknown, score: 0.59
Rfx2Unknown, score: 0.26
Rfx3Unknown, score: 0.16
RgnUnknown, score: 0.41
Rgs16Unknown, score: 0.23
Rgs4Unknown, score: 0.36
Ralbp1Unknown, score: 0.42
Rit1Unknown, score: 0.41
Xpr1Unknown, score: 0.22
Rnf2Unknown, score: 0.18
Rnps1Unknown, score: 0.47
Robo1Unknown, score: 0.27
Rock1Unknown, score: 0.72
Rock2Unknown, score: 0.22
Slc22a8Unknown, score: 0.23
Rom1Unknown, score: 0.33
Mst1rUnknown, score: 0.42
RorcUnknown, score: 0.53
Rp1Unknown, score: 0.37
Rps8Unknown, score: 0.15
RrasUnknown, score: 0.29
Dhrs3Unknown, score: 0.48
Ncor1Unknown, score: 0.22
Ryr1Unknown, score: 0.51
S100a1Unknown, score: 0.43
S100a10Unknown, score: 0.69
S100a3Unknown, score: 0.38
S100bUnknown, score: 0.2
Saa2Unknown, score: 0.65
Sart1Unknown, score: 0.22
Atxn2Unknown, score: 0.23
Scd1Unknown, score: 0.34
Scd2Unknown, score: 0.2
Scg2Unknown, score: 0.28
Scg3Unknown, score: 0.31
Clec11aUnknown, score: 0.16
Stmn3Unknown, score: 0.24
Scn10aUnknown, score: 0.48
Scn3aUnknown, score: 0.35
Scn5aUnknown, score: 0.38
Zc3h7bUnknown, score: 0.28
SctUnknown, score: 0.68
Ccl11Unknown, score: 0.35
Ccl17Unknown, score: 0.58
Ccl2Unknown, score: 0.58
Ccl22Unknown, score: 0.39
Ccl27aUnknown, score: 0.46
Ccl3Unknown, score: 0.21
Ccl5Unknown, score: 0.62
Ccl6Unknown, score: 0.31
Ccl7Unknown, score: 0.31
Ccl8Unknown, score: 0.68
Ccl9Unknown, score: 0.39
Cxcl2Unknown, score: 0.5
Cxcl5Unknown, score: 0.47
Sdf2Unknown, score: 0.66
Serpinf1Unknown, score: 0.2
Sdf4Unknown, score: 0.2
Sfrp2Unknown, score: 0.39
SordUnknown, score: 0.22
Sec22bUnknown, score: 0.51
Sel1lUnknown, score: 0.63
SellUnknown, score: 0.35
SelpUnknown, score: 0.17
SelplgUnknown, score: 0.29
Sema3bUnknown, score: 0.35
Sema5aUnknown, score: 0.32
Sema6cUnknown, score: 0.16
Sema7aUnknown, score: 0.27
Sez6Unknown, score: 0.24
Foxp3Unknown, score: 0.25
Spi1Unknown, score: 0.73
Sfrp1Unknown, score: 0.36
FrzbUnknown, score: 0.44
Srsf5Unknown, score: 0.31
SgceUnknown, score: 0.43
Zfp106Unknown, score: 0.31
Itsn2Unknown, score: 0.87
Ostf1Unknown, score: 0.17
Sorbs3Unknown, score: 0.17
Shc1Unknown, score: 0.25
Shc3Unknown, score: 0.32
Shmt1Unknown, score: 0.38
Siah1bUnknown, score: 0.54
St3gal3Unknown, score: 0.21
St3gal4Unknown, score: 0.2
St6galnac1Unknown, score: 0.28
St8sia1Unknown, score: 0.28
St8sia4Unknown, score: 0.32
Ptk6Unknown, score: 0.2
Six3Unknown, score: 0.26
ClpbUnknown, score: 0.41
SkiUnknown, score: 0.79
SkilUnknown, score: 0.24
Slc10a1Unknown, score: 0.39
Slc16a1Unknown, score: 0.53
Slc16a2Unknown, score: 0.74
Slc1a1Unknown, score: 0.65
Slc1a2Unknown, score: 0.52
Slc1a3Unknown, score: 0.29
Slc1a5Unknown, score: 0.35
Slc20a2Unknown, score: 0.18
Slc23a1Unknown, score: 0.16
Slc2a4Unknown, score: 0.27
Slc6a2Unknown, score: 0.44
Slc7a5Unknown, score: 0.21
Slc7a7Unknown, score: 0.15
Slc9a1Unknown, score: 0.21
Slfn2Unknown, score: 0.23
Slfn3Unknown, score: 0.44
SlpiUnknown, score: 0.26
Kdm5cUnknown, score: 0.27
Ncor2Unknown, score: 0.33
Sstr2Unknown, score: 0.35
Sstr4Unknown, score: 0.38
SncaUnknown, score: 0.22
SncgUnknown, score: 0.34
SnrpeUnknown, score: 0.55
Sntb1Unknown, score: 0.4
Sntb2Unknown, score: 0.64
Sod2Unknown, score: 0.41
Sort1Unknown, score: 0.72
Sos1Unknown, score: 0.26
Sox15Unknown, score: 0.19
Sox4Unknown, score: 0.2
Sox8Unknown, score: 0.24
Spa17Unknown, score: 0.16
Sp4Unknown, score: 0.55
SparcUnknown, score: 0.46
Serpina1aUnknown, score: 0.15
Serpina1aUnknown, score: 0.24
Serpina1eUnknown, score: 0.7
Serpinb9eUnknown, score: 0.19
Serpina3kUnknown, score: 0.66
Serpina3nUnknown, score: 0.37
Serpinb6aUnknown, score: 0.57
Serpinb9Unknown, score: 0.45
Spta1Unknown, score: 0.66
SpopUnknown, score: 0.74
SprUnknown, score: 0.67
Sephs2Unknown, score: 0.17
Sptlc2Unknown, score: 0.3
Scarb1Unknown, score: 0.35
Srpk1Unknown, score: 0.16
Trim21Unknown, score: 0.29
Trove2Unknown, score: 0.21
Ssr4Unknown, score: 0.3
StacUnknown, score: 0.28
Stag1Unknown, score: 0.49
Stag2Unknown, score: 0.4
StamUnknown, score: 0.5
Stat2Unknown, score: 0.54
Stau1Unknown, score: 0.3
Stk10Unknown, score: 0.27
Stk16Unknown, score: 0.31
AC146980.1Unknown, score: 0.37
SlkUnknown, score: 0.39
AurkbUnknown, score: 0.18
Stra13Unknown, score: 0.48
Bhlhe40Unknown, score: 0.59
Stra6Unknown, score: 0.17
Stx1aUnknown, score: 0.32
Stx4aUnknown, score: 0.22
Stxbp1Unknown, score: 0.39
Stxbp2Unknown, score: 0.32
Stxbp3aUnknown, score: 0.29
Abcc8Unknown, score: 0.35
Suv39h1Unknown, score: 0.23
Swap70Unknown, score: 0.16
Vamp7Unknown, score: 0.21
SykUnknown, score: 0.35
Sdc4Unknown, score: 0.21
Synj2Unknown, score: 0.14
Syt2Unknown, score: 0.19
Syt3Unknown, score: 0.2
Tac1Unknown, score: 0.23
Taf6Unknown, score: 0.24
TaglnUnknown, score: 0.48
Tagln2Unknown, score: 0.18
Taldo1Unknown, score: 0.3
Tarbp2Unknown, score: 0.27
TbcaUnknown, score: 0.17
Tbx1Unknown, score: 0.35
Tbx2Unknown, score: 0.19
Tbx3Unknown, score: 0.4
Tbx5Unknown, score: 0.16
Tbxa2rUnknown, score: 0.24
Hnf1aUnknown, score: 0.56
Tcf15Unknown, score: 0.2
Tcf21Unknown, score: 0.23
Tcf7Unknown, score: 0.24
Tcf7l2Unknown, score: 0.18
TfebUnknown, score: 0.28
Tcte2Unknown, score: 0.2
TdgUnknown, score: 0.18
Tead1Unknown, score: 0.35
TectaUnknown, score: 0.33
TefUnknown, score: 0.18
TekUnknown, score: 0.25
InmtUnknown, score: 0.14
Tep1Unknown, score: 0.35
TertUnknown, score: 0.29
Psmd2Unknown, score: 0.24
Cirh1aUnknown, score: 0.17
TfamUnknown, score: 0.46
TfpiUnknown, score: 0.33
TgfaUnknown, score: 0.19
Tgfb1Unknown, score: 0.35
Tsc22d1Unknown, score: 0.49
Tgfb2Unknown, score: 0.41
Tgfb3Unknown, score: 0.22
TgfbiUnknown, score: 0.47
Tgfbr1Unknown, score: 0.33
Tgfbr3Unknown, score: 0.7
Tgm3Unknown, score: 0.29
Ift88Unknown, score: 0.26
Tgtp1Unknown, score: 0.3
ThbdUnknown, score: 0.61
Thbs1Unknown, score: 0.24
Thbs2Unknown, score: 0.74
Thbs3Unknown, score: 0.7
Thbs4Unknown, score: 0.2
ThrspUnknown, score: 0.14
Tial1Unknown, score: 0.21
Timm44Unknown, score: 0.96
Timp1Unknown, score: 0.5
Tjp2Unknown, score: 0.21
Tle2Unknown, score: 0.45
Tle4Unknown, score: 0.28
Tlr1Unknown, score: 0.66
Tlr4Unknown, score: 0.24
Tlr6Unknown, score: 0.14
Tlx1Unknown, score: 0.17
TmpoUnknown, score: 0.26
Clec3bUnknown, score: 0.88
TnfUnknown, score: 0.18
Tnfaip3Unknown, score: 0.64
Tnfrsf10bUnknown, score: 0.17
Tnfrsf18Unknown, score: 0.41
Tnfrsf1aUnknown, score: 0.41
Tnfrsf1bUnknown, score: 0.24
Tnfrsf8Unknown, score: 0.15
Cd40lgUnknown, score: 0.18
Tnnt2Unknown, score: 0.35
Top1Unknown, score: 0.57
Top2aUnknown, score: 0.18
Tmem165Unknown, score: 0.2
Tpm1Unknown, score: 0.38
Tpm2Unknown, score: 0.15
TpmtUnknown, score: 0.24
Hsp90b1Unknown, score: 0.33
Traf2Unknown, score: 0.27
Traf4Unknown, score: 0.22
Traf6Unknown, score: 0.16
Trp53Unknown, score: 0.57
Trpc4Unknown, score: 0.33
Tpt1Unknown, score: 0.25
Rsph1Unknown, score: 0.19
Psmd3Unknown, score: 0.53
Ttc3Unknown, score: 0.21
Tgoln1Unknown, score: 0.28
TtkUnknown, score: 0.2
TtnUnknown, score: 0.22
TubUnknown, score: 0.35
Tuba3bUnknown, score: 0.32
Tubb2aUnknown, score: 0.51
Tuft1Unknown, score: 0.19
Tulp3Unknown, score: 0.27
Txn1Unknown, score: 0.15
TyrUnknown, score: 0.32
Tyro3Unknown, score: 0.28
TyrobpUnknown, score: 0.25
Uba1Unknown, score: 0.22
Usp12Unknown, score: 0.19
Sumo1Unknown, score: 0.14
UcnUnknown, score: 0.53
Slc35a2Unknown, score: 0.21
UgcgUnknown, score: 0.84
Ugt2b5Unknown, score: 0.46
Unc119Unknown, score: 0.24
Unc13bUnknown, score: 0.27
Usp4Unknown, score: 0.5
Nr1h3Unknown, score: 0.15
Usf2Unknown, score: 0.21
Ush2aUnknown, score: 0.25
Kdm6aUnknown, score: 0.31
UtyUnknown, score: 0.21
Cdh23Unknown, score: 0.37
Vmn1r45Unknown, score: 0.32
VarsUnknown, score: 0.23
VaspUnknown, score: 0.15
Vbp1Unknown, score: 0.31
EzrUnknown, score: 0.3
VillUnknown, score: 0.6
Vps45Unknown, score: 0.15
Wbp2Unknown, score: 0.17
Fmnl3Unknown, score: 0.21
Wbp4Unknown, score: 0.47
Wfs1Unknown, score: 0.21
Wisp1Unknown, score: 0.27
WizUnknown, score: 0.19
Wnt1Unknown, score: 0.37
Wnt11Unknown, score: 0.36
Wnt9bUnknown, score: 0.24
Wnt4Unknown, score: 0.35
Wnt6Unknown, score: 0.34
Wnt8bUnknown, score: 0.28
Dctn6Unknown, score: 0.64
XkUnknown, score: 0.28
Xlr3cUnknown, score: 0.29
YwhagUnknown, score: 0.15
YwhaqUnknown, score: 0.35
Yy1Unknown, score: 0.36
Plagl1Unknown, score: 0.31
Zbtb17Unknown, score: 0.26
Zfp13Unknown, score: 0.26
Sf1Unknown, score: 0.31
Trim26Unknown, score: 0.23
Zpr1Unknown, score: 0.26
Zfp30Unknown, score: 0.31
Zfp36Unknown, score: 0.33
Zfp39Unknown, score: 0.26
Zfp51Unknown, score: 0.17
Zfp54Unknown, score: 0.21
Zbtb7bUnknown, score: 0.19
Zfp85Unknown, score: 0.26
Zfp94Unknown, score: 0.14
Zfpm2Unknown, score: 0.19
ZfrUnknown, score: 0.4
ZfxUnknown, score: 0.52
Zik1Unknown, score: 0.14
Zim1Unknown, score: 0.19
Ikzf1Unknown, score: 0.18
Slc30a3Unknown, score: 0.23
Slc30a4Unknown, score: 0.61
Dnajc2Unknown, score: 0.31
Coro1bUnknown, score: 0.4
Coro1cUnknown, score: 0.28
AplnrUnknown, score: 0.56
Aloxe3Unknown, score: 0.28
Apc2Unknown, score: 0.35
Arih1Unknown, score: 0.2
Ash2lUnknown, score: 0.17
Bpnt1Unknown, score: 0.22
BvesUnknown, score: 0.19
Car14Unknown, score: 0.21
Cd52Unknown, score: 0.33
Cfdp1Unknown, score: 0.17
Klf6Unknown, score: 0.17
Pappa2Unknown, score: 0.3
Def8Unknown, score: 0.15
Dmtf1Unknown, score: 0.29
Ets2Unknown, score: 0.34
FarsbUnknown, score: 0.22
Fxr2Unknown, score: 0.38
Ggt5Unknown, score: 0.85
HcstUnknown, score: 0.17
Impdh2Unknown, score: 0.17
InsrrUnknown, score: 0.22
AadatUnknown, score: 0.23
Mta2Unknown, score: 0.14
Mmp17Unknown, score: 0.36
Nek3Unknown, score: 0.14
Nt5eUnknown, score: 0.16
Oas1bUnknown, score: 0.31
Tenm2Unknown, score: 0.26
Osr1Unknown, score: 0.15
Nlrp5Unknown, score: 0.64
Pcbp1Unknown, score: 0.56
Rai2Unknown, score: 0.27
Grk1Unknown, score: 0.28
Abce1Unknown, score: 0.23
Ccl19Unknown, score: 0.4
Sept3Unknown, score: 0.31
SgcbUnknown, score: 0.18
SgcgUnknown, score: 0.22
Sh3bp5Unknown, score: 0.16
SigirrUnknown, score: 0.55
Spry4Unknown, score: 0.15
Tekt2Unknown, score: 0.33
Rbck1Unknown, score: 0.15
Wif1Unknown, score: 0.27
Zfp68Unknown, score: 0.5
Zeb2Unknown, score: 0.24
Ing1Unknown, score: 0.26
Aldh1a7Unknown, score: 0.26
Angptl2Unknown, score: 0.44
Adgre5Unknown, score: 0.24
FtoUnknown, score: 0.18
Hsf4Unknown, score: 0.29
Map2k4Unknown, score: 0.14
Map2k7Unknown, score: 0.16
Map3k12Unknown, score: 0.39
Map3k7Unknown, score: 0.22
Mapk1Unknown, score: 0.45
Mapk3Unknown, score: 0.24
Nr5a2Unknown, score: 0.28
Nubp2Unknown, score: 0.46
Creb3l1Unknown, score: 0.15
PargUnknown, score: 0.16
Plod2Unknown, score: 0.39
PrndUnknown, score: 0.44
Psg16Unknown, score: 0.16
Psmb3Unknown, score: 0.19
PoliUnknown, score: 0.31
MokUnknown, score: 0.28
Zfp146Unknown, score: 0.2
Homer2Unknown, score: 0.19
HunkUnknown, score: 0.32
NcdnUnknown, score: 0.36
Ror1Unknown, score: 0.17
Slc27a4Unknown, score: 0.22
Cops3Unknown, score: 0.2
Abcd2Unknown, score: 0.3
B3galnt1Unknown, score: 0.48
Casp8ap2Unknown, score: 0.15
Chst4Unknown, score: 0.28
Cops7bUnknown, score: 0.29
Acot1Unknown, score: 0.31
Eif2s3yUnknown, score: 0.23
GcatUnknown, score: 0.35
Zfp346Unknown, score: 0.7
Aifm1Unknown, score: 0.17
Foxl2Unknown, score: 0.18
Ppp2r5eUnknown, score: 0.55
Serinc3Unknown, score: 0.25
Rpl8Unknown, score: 0.21
Cul1Unknown, score: 0.51
Pla2g2eUnknown, score: 0.14
Eif4e2Unknown, score: 0.26
Brd7Unknown, score: 0.21
Micall1Unknown, score: 0.35
Rps3Unknown, score: 0.19
AoahUnknown, score: 0.31
Sec23bUnknown, score: 0.42
Fkbp9Unknown, score: 0.43
Srp9Unknown, score: 0.56
Bcap31Unknown, score: 0.33
Syn3Unknown, score: 0.19
Rps11Unknown, score: 0.45
Atp1a4Unknown, score: 0.25
Trp53bp1Unknown, score: 0.2
Tnfrsf12aUnknown, score: 0.17
Pald1Unknown, score: 0.27
Sytl4Unknown, score: 0.21
Dnajb9Unknown, score: 0.31
Slc25a10Unknown, score: 0.25
Yme1l1Unknown, score: 0.19
Mmel1Unknown, score: 0.24
PignUnknown, score: 0.27
Hsd17b6Unknown, score: 0.22
PdhxUnknown, score: 0.16
Abca7Unknown, score: 0.16
Abca3Unknown, score: 0.18
NagpaUnknown, score: 0.15
Shroom3Unknown, score: 0.4
Ubl4Unknown, score: 0.27
Vwa7Unknown, score: 0.15
Rsrp1Unknown, score: 0.2
Prpf19Unknown, score: 0.16
Gfm1Unknown, score: 0.18
Larp7Unknown, score: 0.19
TwistnbUnknown, score: 0.18
MydgfUnknown, score: 0.16
Nsun2Unknown, score: 0.15
Cep63Unknown, score: 0.16
Slco1a1Unknown, score: 0.45
Tpk1Unknown, score: 0.35
MgaUnknown, score: 0.35
Hip1rUnknown, score: 0.47
Igfbp7Unknown, score: 0.21
Hspb7Unknown, score: 0.31
Stau2Unknown, score: 0.42
Sult4a1Unknown, score: 0.21
Ulk2Unknown, score: 0.36
Cspg5Unknown, score: 0.41
Clic4Unknown, score: 0.15
Dnajc12Unknown, score: 0.3
Cttnbp2Unknown, score: 0.22
Fkbp3Unknown, score: 0.18
Slc22a4Unknown, score: 0.25
Lmcd1Unknown, score: 0.3
Pttg1Unknown, score: 0.23
Usp21Unknown, score: 0.44
Adat1Unknown, score: 0.35
Siva1Unknown, score: 0.42
Hacd1Unknown, score: 0.71
Sall2Unknown, score: 0.15
Spag6Unknown, score: 0.24
Mrps7Unknown, score: 0.87
Mfap5Unknown, score: 1
PostnUnknown, score: 0.28
Sirt6Unknown, score: 0.17
Fbxo8Unknown, score: 0.28
Fbxl17Unknown, score: 0.18
Atp9bUnknown, score: 0.31
Polg2Unknown, score: 0.44
Rgs1Unknown, score: 0.3
Rgs3Unknown, score: 0.86
Dkk3Unknown, score: 0.47
Lsm4Unknown, score: 0.16
Hs6st1Unknown, score: 0.61
Fbxl3Unknown, score: 0.3
Klf13Unknown, score: 0.3
Tmod4Unknown, score: 0.32
Tmod3Unknown, score: 0.48
Stag3Unknown, score: 0.23
SclyUnknown, score: 0.18
Chek2Unknown, score: 0.38
Nckap1Unknown, score: 0.21
PrebUnknown, score: 0.36
C1s1Unknown, score: 0.25
C1raUnknown, score: 0.27
Irx4Unknown, score: 0.36
GalnsUnknown, score: 0.31
HnrnpdlUnknown, score: 0.18
Pdcd7Unknown, score: 0.17
Ppp2r1aUnknown, score: 0.27
CcncUnknown, score: 0.43
Tmem141Unknown, score: 0.16
Fubp1Unknown, score: 0.64
Atg13Unknown, score: 0.32
Rnf24Unknown, score: 0.15
Ccdc39Unknown, score: 0.49
Hn1lUnknown, score: 0.16
R3hcc1lUnknown, score: 0.55
Bbs1Unknown, score: 0.25
Tmem38bUnknown, score: 0.18
Tmem222Unknown, score: 0.19
Reep1Unknown, score: 0.4
Klhl7Unknown, score: 0.69
Stbd1Unknown, score: 0.36
Atxn1lUnknown, score: 0.23
D6Ertd527eUnknown, score: 0.27
Rcn3Unknown, score: 0.29
D1Ertd622eUnknown, score: 0.19
Zfp644Unknown, score: 0.35
Echdc2Unknown, score: 0.27
Ppp2r2dUnknown, score: 0.17
Mrpl48Unknown, score: 0.16
Angel2Unknown, score: 0.38
Carhsp1Unknown, score: 0.16
Ccdc69Unknown, score: 0.18
Tspan14Unknown, score: 0.29
NcaldUnknown, score: 0.3
Nudcd2Unknown, score: 0.63
Ldlrad4Unknown, score: 0.2
Echdc1Unknown, score: 0.22
Arhgef25Unknown, score: 0.28
Cpsf4lUnknown, score: 0.41
Mettl2Unknown, score: 0.19
ZwintUnknown, score: 0.26
Txndc17Unknown, score: 0.2
Zkscan6Unknown, score: 0.19
Ccdc59Unknown, score: 0.36
DnlzUnknown, score: 0.16
Mtg2Unknown, score: 0.18
Slx4Unknown, score: 0.15
D19Bwg1357eUnknown, score: 0.57
RnasekUnknown, score: 0.32
Atp2a3Unknown, score: 0.22
Plrg1Unknown, score: 0.77
Pdlim3Unknown, score: 0.38
Cntnap1Unknown, score: 0.44
Ube2kUnknown, score: 0.19
Pgrmc1Unknown, score: 0.33
Pla2g6Unknown, score: 0.72
Usp2Unknown, score: 0.18
Txnl1Unknown, score: 0.27
Htatip2Unknown, score: 0.29
Pcdh12Unknown, score: 0.17
Vti1bUnknown, score: 0.16
ReckUnknown, score: 0.3
Vamp5Unknown, score: 0.23
Cnot4Unknown, score: 0.45
Prrc2aUnknown, score: 0.18
Tlr5Unknown, score: 0.15
Sept9Unknown, score: 0.43
Zranb2Unknown, score: 0.23
Naip6Unknown, score: 0.24
Gal3st1Unknown, score: 0.18
Rfx5Unknown, score: 0.76
Lpar2Unknown, score: 0.32
PolmUnknown, score: 0.18
Rps28Unknown, score: 0.29
Avpr1aUnknown, score: 0.46
Rasa4Unknown, score: 0.22
Copg1Unknown, score: 0.16
Akap8lUnknown, score: 0.33
Arl6ip1Unknown, score: 0.32
Cd320Unknown, score: 0.4
Slc23a2Unknown, score: 0.26
Irx5Unknown, score: 0.37
Skap2Unknown, score: 0.26
Rpp30Unknown, score: 0.73
Nme6Unknown, score: 0.35
Cacng4Unknown, score: 0.26
Cacng6Unknown, score: 0.23
Smarcal1Unknown, score: 0.27
Phc2Unknown, score: 0.18
Mtmr7Unknown, score: 0.23
RfkUnknown, score: 0.34
Bet1lUnknown, score: 0.2
Stk19Unknown, score: 0.38
HgfacUnknown, score: 0.32
Unc93b1Unknown, score: 0.21
Nfat5Unknown, score: 0.44
TollipUnknown, score: 0.43
Dll4Unknown, score: 0.22
HpgdsUnknown, score: 0.56
Syt7Unknown, score: 0.16
Nup210Unknown, score: 0.57
CalcrlUnknown, score: 0.17
PcnxUnknown, score: 0.14
Socs6Unknown, score: 0.42
Tbc1d8Unknown, score: 0.26
Sfrp5Unknown, score: 0.33
Ftsj1Unknown, score: 0.15
Wdr45Unknown, score: 0.4
Ccdc22Unknown, score: 0.24
Ccdc120Unknown, score: 0.28
Atp8b2Unknown, score: 0.66
Atp8b1Unknown, score: 0.22
Prdx5Unknown, score: 0.16
Plagl2Unknown, score: 0.41
Rcan1Unknown, score: 0.27
Cadm1Unknown, score: 0.41
Hes6Unknown, score: 0.22
Fnbp4Unknown, score: 0.21
Stx8Unknown, score: 0.21
Ap3m1Unknown, score: 0.27
Agpat1Unknown, score: 0.19
Pdzrn3Unknown, score: 0.56
Cxcl13Unknown, score: 0.47
Snx12Unknown, score: 0.17
Fmo2Unknown, score: 0.7
Srcin1Unknown, score: 0.17
Stard10Unknown, score: 0.29
Rplp1Unknown, score: 0.26
Uso1Unknown, score: 0.16
Akr1e1Unknown, score: 0.19
Uqcc1Unknown, score: 0.2
Lgals8Unknown, score: 0.43
Gtpbp2Unknown, score: 0.22
Ammecr1Unknown, score: 0.25
DgkeUnknown, score: 0.29
Car5bUnknown, score: 0.18
GraspUnknown, score: 0.27
PigpUnknown, score: 0.39
Rbm38Unknown, score: 0.34
TroUnknown, score: 0.55
PlekUnknown, score: 0.28
EnsaUnknown, score: 0.36
Rev1Unknown, score: 0.18
Stx1bUnknown, score: 0.19
Cited4Unknown, score: 0.59
Tspan5Unknown, score: 0.23
Ube2j1Unknown, score: 0.21
Actr8Unknown, score: 0.7
Pex14Unknown, score: 0.32
Rbm14Unknown, score: 0.42
Tmem45aUnknown, score: 0.28
Mrpl19Unknown, score: 0.22
Slc35g3Unknown, score: 0.32
Higd1aUnknown, score: 0.33
Arl6Unknown, score: 0.49
Metap2Unknown, score: 0.46
Gps2Unknown, score: 0.67
AcppUnknown, score: 0.75
Mettl3Unknown, score: 0.49
Hsd17b12Unknown, score: 0.27
Arl3Unknown, score: 0.14
Ptges3Unknown, score: 0.14
Zmym3Unknown, score: 0.15
ClcnkbUnknown, score: 0.29
B4galt4Unknown, score: 0.37
Pdlim5Unknown, score: 0.56
Tmem115Unknown, score: 0.21
P3h1Unknown, score: 0.33
Trip4Unknown, score: 0.3
Ykt6Unknown, score: 0.55
Stub1Unknown, score: 0.67
Mtch2Unknown, score: 0.41
Clip1Unknown, score: 0.19
DstnUnknown, score: 0.43
Dnaja2Unknown, score: 0.31
Mbtps1Unknown, score: 0.48
Foxo1Unknown, score: 0.15
Socs5Unknown, score: 0.18
Rgs19Unknown, score: 0.47
Stmn4Unknown, score: 0.23
Fads2Unknown, score: 0.28
Nxt1Unknown, score: 0.38
VapbUnknown, score: 0.28
Asna1Unknown, score: 0.45
Tspan6Unknown, score: 0.38
Ruvbl1Unknown, score: 0.5
Rapgef4Unknown, score: 0.15
Zfp235Unknown, score: 0.21
Ripk3Unknown, score: 0.29
Txn2Unknown, score: 0.41
Pfdn5Unknown, score: 0.2
Mgst1Unknown, score: 0.39
Clec4eUnknown, score: 0.73
PollUnknown, score: 0.17
Sphk2Unknown, score: 0.25
Fgf21Unknown, score: 0.32
Gsk3bUnknown, score: 0.18
PnkdUnknown, score: 0.25
PigoUnknown, score: 0.48
MtorUnknown, score: 0.2
Tdo2Unknown, score: 0.55
CriptUnknown, score: 0.47
Rnf14Unknown, score: 0.2
Igdcc4Unknown, score: 0.25
Psrc1Unknown, score: 0.21
Pf4Unknown, score: 0.39
C1qtnf1Unknown, score: 0.31
Tacstd2Unknown, score: 0.19
Mllt11Unknown, score: 0.16
Zbtb33Unknown, score: 0.27
Gmeb1Unknown, score: 0.3
Dkk2Unknown, score: 0.17
Necab3Unknown, score: 0.57
Insm2Unknown, score: 0.39
Lmbr1Unknown, score: 0.14
NsmfUnknown, score: 0.25
Sap30bpUnknown, score: 0.31
Ly6iUnknown, score: 0.22
GabrqUnknown, score: 0.16
RetnUnknown, score: 0.73
BC051019Unknown, score: 0.22
B4galt3Unknown, score: 0.39
P2ry4Unknown, score: 0.18
Gabarapl1Unknown, score: 0.21
March7Unknown, score: 0.23
GmnnUnknown, score: 0.15
Slc15a2Unknown, score: 0.28
Piwil2Unknown, score: 0.4
Rnf25Unknown, score: 0.17
Dnajb7Unknown, score: 0.15
Fxyd7Unknown, score: 0.37
Rpl35aUnknown, score: 0.3
Tk2Unknown, score: 0.4
Il17reUnknown, score: 0.26
Ccdc126Unknown, score: 0.46
Krcc1Unknown, score: 0.33
Zfp318Unknown, score: 0.62
Crlf2Unknown, score: 0.23
Rgs20Unknown, score: 0.25
Rsad2Unknown, score: 0.41
Extl2Unknown, score: 0.29
NelfbUnknown, score: 0.22
Zbtb32Unknown, score: 0.2
Cst10Unknown, score: 0.25
Rab37Unknown, score: 0.68
Fam184bUnknown, score: 0.41
Stk4Unknown, score: 0.15
Shank3Unknown, score: 0.42
Gpr180Unknown, score: 0.2
Slc35b4Unknown, score: 0.21
FibpUnknown, score: 0.24
CrbnUnknown, score: 0.5
Trpm7Unknown, score: 0.29
MlxiplUnknown, score: 0.27
Slco1c1Unknown, score: 0.33
Akr1a1Unknown, score: 0.3
Efemp2Unknown, score: 0.8
Cysltr1Unknown, score: 0.35
Fam13aUnknown, score: 0.32
Smpd3Unknown, score: 0.33
Arhgap23Unknown, score: 0.17
Anapc5Unknown, score: 0.3
SqrdlUnknown, score: 0.16
Rrs1Unknown, score: 0.24
Thap11Unknown, score: 0.29
MkksUnknown, score: 0.41
Wsb2Unknown, score: 0.31
Stard3Unknown, score: 0.19
PnkpUnknown, score: 0.25
Slc22a17Unknown, score: 0.3
Tpm3Unknown, score: 0.3
Erbb2ipUnknown, score: 0.18
Pcbp4Unknown, score: 0.48
Pcbp3Unknown, score: 0.28
Nek7Unknown, score: 0.51
Dctn5Unknown, score: 0.76
Ms4a4bUnknown, score: 0.18
Trappc4Unknown, score: 0.19
Tmem8Unknown, score: 0.17
Fads3Unknown, score: 0.18
Capn12Unknown, score: 0.19
Mapk8ip2Unknown, score: 0.26
Trpv4Unknown, score: 0.39
GmfbUnknown, score: 0.27
Syne1Unknown, score: 0.36
PerpUnknown, score: 0.2
Clstn2Unknown, score: 0.52
Sdf2l1Unknown, score: 0.39
RalbUnknown, score: 0.42
St7Unknown, score: 0.15
Abhd8Unknown, score: 0.35
Gpr88Unknown, score: 0.25
Sirt3Unknown, score: 0.15
Polr1eUnknown, score: 0.18
NischUnknown, score: 0.18
Suv39h2Unknown, score: 0.48
Svep1Unknown, score: 0.29
Bhmt2Unknown, score: 0.18
Izumo1rUnknown, score: 0.29
Rtn4rUnknown, score: 0.66
Dap3Unknown, score: 0.36
Bean1Unknown, score: 0.22
Prrg2Unknown, score: 0.42
Asb2Unknown, score: 0.19
Twsg1Unknown, score: 0.22
Tmem176bUnknown, score: 0.16
CubnUnknown, score: 0.82
TbataUnknown, score: 0.3
Cndp2Unknown, score: 0.39
Tctex1d2Unknown, score: 0.25
Gng11Unknown, score: 0.18
SnupnUnknown, score: 0.23
Txndc12Unknown, score: 0.31
PpihUnknown, score: 0.3
Tspan13Unknown, score: 0.19
1110001J03RikUnknown, score: 0.28
Sf3b5Unknown, score: 0.14
Aaed1Unknown, score: 0.29
TipinUnknown, score: 0.16
Ska2Unknown, score: 0.3
Tmem57Unknown, score: 0.29
Dnajc15Unknown, score: 0.55
BccipUnknown, score: 0.15
PycrlUnknown, score: 0.16
Myo19Unknown, score: 0.16
Commd4Unknown, score: 0.14
Acyp1Unknown, score: 0.25
NenfUnknown, score: 0.25
Sec61bUnknown, score: 0.4
Zdhhc12Unknown, score: 0.56
Dmap1Unknown, score: 0.29
Eif1axUnknown, score: 0.17
Mrps16Unknown, score: 0.14
Pno1Unknown, score: 0.49
Cox16Unknown, score: 0.16
AamdcUnknown, score: 0.2
Smim20Unknown, score: 0.19
CenpwUnknown, score: 0.16
Senp7Unknown, score: 0.15
Pla2g12aUnknown, score: 0.17
Blzf1Unknown, score: 0.49
GmprUnknown, score: 0.22
NosipUnknown, score: 0.19
Ccdc82Unknown, score: 0.23
Asf1aUnknown, score: 0.19
AptxUnknown, score: 0.16
Pcp4l1Unknown, score: 0.3
Chchd7Unknown, score: 0.15
MagohbUnknown, score: 0.27
Mgst3Unknown, score: 0.28
Pam16Unknown, score: 0.17
Nmnat1Unknown, score: 0.19
Gtf2h5Unknown, score: 0.29
Rps21Unknown, score: 0.21
Mrpl51Unknown, score: 0.16
Dda1Unknown, score: 0.21
Slc30a7Unknown, score: 0.2
Ubxn6Unknown, score: 0.21
Rep15Unknown, score: 0.27
Rps19bp1Unknown, score: 0.27
Adamtsl5Unknown, score: 0.15
Aggf1Unknown, score: 0.35
NtpcrUnknown, score: 0.31
Gdpd1Unknown, score: 0.25
CenpmUnknown, score: 0.43
FastkUnknown, score: 0.31
Trim13Unknown, score: 0.19
Lrrc57Unknown, score: 0.46
Ormdl3Unknown, score: 0.19
Ntmt1Unknown, score: 0.16
Thg1lUnknown, score: 0.17
Golph3Unknown, score: 0.16
Pspc1Unknown, score: 0.14
Acp6Unknown, score: 0.32
Uba5Unknown, score: 0.35
Tmem41aUnknown, score: 0.27
Spryd7Unknown, score: 0.16
Oser1Unknown, score: 0.38
Trappc5Unknown, score: 0.46
Tceal8Unknown, score: 0.15
Dcbld1Unknown, score: 0.31
Tbc1d15Unknown, score: 0.34
Uqcrfs1Unknown, score: 0.94
NkaplUnknown, score: 0.4
4921524J17RikUnknown, score: 0.15
Henmt1Unknown, score: 0.45
Emc2Unknown, score: 0.29
Erich2Unknown, score: 0.28
Tmem239Unknown, score: 0.16
Hacd4Unknown, score: 0.15
Atg10Unknown, score: 0.34
Krt20Unknown, score: 0.27
PpcdcUnknown, score: 0.3
TazUnknown, score: 0.23
Rsph3aUnknown, score: 0.14
Fuca2Unknown, score: 0.15
Tanc1Unknown, score: 0.26
Lztr1Unknown, score: 0.62
PmpcaUnknown, score: 0.17
Serbp1Unknown, score: 0.27
Cpne8Unknown, score: 0.22
TrilUnknown, score: 0.16
1200014J11RikUnknown, score: 0.19
Swt1Unknown, score: 0.22
Riok3Unknown, score: 0.19
Lonp2Unknown, score: 0.54
Naa16Unknown, score: 0.23
Baiap2l1Unknown, score: 0.28
ProzUnknown, score: 0.15
Chordc1Unknown, score: 0.19
Asf1bUnknown, score: 0.15
Rexo1Unknown, score: 0.34
Acad8Unknown, score: 0.38
Tmem206Unknown, score: 0.35
Trit1Unknown, score: 0.26
Plin5Unknown, score: 0.22
Trappc13Unknown, score: 0.46
Zdhhc6Unknown, score: 0.38
Lap3Unknown, score: 0.28
Polr3kUnknown, score: 0.17
Yae1d1Unknown, score: 0.57
Ttc23Unknown, score: 0.3
Fam210bUnknown, score: 0.18
2010109I03RikUnknown, score: 0.27
Oxct1Unknown, score: 0.26
Tbc1d7Unknown, score: 0.15
Pus3Unknown, score: 0.29
NkapUnknown, score: 0.27
Rpp14Unknown, score: 0.22
PaicsUnknown, score: 0.51
Yaf2Unknown, score: 0.29
Dynlrb1Unknown, score: 0.15
Lsm14aUnknown, score: 0.14
Rps6ka6Unknown, score: 0.37
Cdc37l1Unknown, score: 0.17
Pi4k2bUnknown, score: 0.23
PgpUnknown, score: 0.61
Trappc6aUnknown, score: 0.2
2310039H08RikUnknown, score: 0.2
D16Ertd472eUnknown, score: 0.52
Cuedc2Unknown, score: 0.42
Ttc14Unknown, score: 0.18
Kbtbd4Unknown, score: 0.36
Herc6Unknown, score: 0.19
Fbxo5Unknown, score: 0.19
Fam103a1Unknown, score: 0.14
Nkain1Unknown, score: 0.19
Psmd9Unknown, score: 0.24
MtdhUnknown, score: 0.18
Lipt2Unknown, score: 0.42
Arl8bUnknown, score: 0.25
NraddUnknown, score: 0.25
Ctdnep1Unknown, score: 0.18
Ube2tUnknown, score: 0.17
Nde1Unknown, score: 0.28
Lsm1Unknown, score: 0.15
Med18Unknown, score: 0.34
Dph7Unknown, score: 0.19
Prpf18Unknown, score: 0.44
CinpUnknown, score: 0.24
Rpf2Unknown, score: 0.25
Gemin6Unknown, score: 0.51
Peli1Unknown, score: 0.68
Rpl39Unknown, score: 0.53
Tbc1d19Unknown, score: 0.15
Cers4Unknown, score: 0.24
Myl12aUnknown, score: 0.27
Ndufa10Unknown, score: 0.39
Ift22Unknown, score: 0.41
Rab3cUnknown, score: 0.53
Gprasp1Unknown, score: 0.45
CltcUnknown, score: 0.46
Zc3h13Unknown, score: 0.22
Gpx7Unknown, score: 0.17
Zc2hc1aUnknown, score: 0.41
Mrpl46Unknown, score: 0.38
Snrpd3Unknown, score: 0.18
RfflUnknown, score: 0.21
Paxbp1Unknown, score: 0.14
Zfp606Unknown, score: 0.18
Gtf3c6Unknown, score: 0.24
SrprUnknown, score: 0.47
NtsUnknown, score: 0.18
Mfn1Unknown, score: 0.4
3632451O06RikUnknown, score: 0.15
Rps20Unknown, score: 0.17
Nudcd1Unknown, score: 0.24
Ankrd33bUnknown, score: 0.17
Dusp28Unknown, score: 0.22
Pkp2Unknown, score: 0.18
Pnpla8Unknown, score: 0.61
Slc25a46Unknown, score: 0.35
IkbipUnknown, score: 0.67
Klhl13Unknown, score: 0.25
Frmd8Unknown, score: 0.24
Sf3a1Unknown, score: 0.14
MmdUnknown, score: 0.24
Slc47a1Unknown, score: 0.43
Eepd1Unknown, score: 0.25
Ccar1Unknown, score: 0.59
Ccdc50Unknown, score: 0.15
Ttc33Unknown, score: 0.19
Nudt7Unknown, score: 0.19
Zswim3Unknown, score: 0.22
Fam120bUnknown, score: 0.19
GstcdUnknown, score: 0.31
Slc25a30Unknown, score: 0.24
PigmUnknown, score: 0.28
NarflUnknown, score: 0.26
Mrfap1Unknown, score: 0.31
Alg13Unknown, score: 0.15
Cpeb4Unknown, score: 0.15
Akt1s1Unknown, score: 0.5
NarfUnknown, score: 0.31
4930453N24RikUnknown, score: 0.22
Nob1Unknown, score: 0.7
Bend5Unknown, score: 0.66
Spc24Unknown, score: 0.66
Lyrm5Unknown, score: 0.15
4930523C07RikUnknown, score: 0.33
Ift172Unknown, score: 0.3
Hapln3Unknown, score: 0.2
Pbdc1Unknown, score: 0.16
Luc7l3Unknown, score: 0.24
HypkUnknown, score: 0.4
Fam174aUnknown, score: 0.44
Fam114a2Unknown, score: 0.21
Itgb3bpUnknown, score: 0.16
Slc48a1Unknown, score: 0.26
Ribc2Unknown, score: 0.29
MgarpUnknown, score: 0.63
Rtp4Unknown, score: 0.31
Vwa5aUnknown, score: 0.14
Limd2Unknown, score: 0.19
TprglUnknown, score: 0.2
Rer1Unknown, score: 0.17
Brix1Unknown, score: 0.2
Mrpl57Unknown, score: 0.14
Slc35a4Unknown, score: 0.46
Ddx55Unknown, score: 0.38
Echdc3Unknown, score: 0.57
Yipf4Unknown, score: 0.22
RprmUnknown, score: 0.53
Mdp1Unknown, score: 0.32
1810043G02RikUnknown, score: 0.46
SarafUnknown, score: 0.45
Rbm18Unknown, score: 0.49
Tmem86aUnknown, score: 0.48
Ppa1Unknown, score: 0.54
Zfp169Unknown, score: 0.3
Zcchc3Unknown, score: 0.15
Tceb1Unknown, score: 0.18
Hcfc2Unknown, score: 0.16
Wdr55Unknown, score: 0.16
Rpl41Unknown, score: 0.47
NifkUnknown, score: 0.14
Sugt1Unknown, score: 0.14
Setd8Unknown, score: 0.19
Zcchc10Unknown, score: 0.65
Pold3Unknown, score: 0.61
Tppp3Unknown, score: 0.33
Atp2b1Unknown, score: 0.32
CcnyUnknown, score: 0.35
TrabdUnknown, score: 0.37
Gnpda2Unknown, score: 0.21
Pdzd9Unknown, score: 0.2
Tmx3Unknown, score: 0.38
Nacc2Unknown, score: 0.27
Fam134cUnknown, score: 0.46
Sdhaf4Unknown, score: 0.43
SnrpgUnknown, score: 0.29
Hist1h2bcUnknown, score: 0.33
Rpl22l1Unknown, score: 0.39
Cox19Unknown, score: 0.33
N6amt2Unknown, score: 0.19
Rps13Unknown, score: 0.26
Slc25a39Unknown, score: 0.23
3010026O09RikUnknown, score: 0.56
Fam173bUnknown, score: 0.32
Rchy1Unknown, score: 0.32
Atg101Unknown, score: 0.36
Ino80Unknown, score: 0.23
Etaa1Unknown, score: 0.59
Otub2Unknown, score: 0.15
Fam133bUnknown, score: 0.28
Fdx1lUnknown, score: 0.26
NdnfUnknown, score: 0.2
B230118H07RikUnknown, score: 0.15
EbplUnknown, score: 0.4
Coa4Unknown, score: 0.29
Ndufb2Unknown, score: 0.38
Ndufa5Unknown, score: 0.38
Diras2Unknown, score: 0.29
Gsto2Unknown, score: 0.22
Fam166aUnknown, score: 0.25
A930018P22RikUnknown, score: 0.25
Tmem86bUnknown, score: 0.14
Trmt12Unknown, score: 0.59
Toe1Unknown, score: 0.54
Ddx39Unknown, score: 0.45
Mcoln2Unknown, score: 0.22
Gm12666Unknown, score: 0.27
9530077C05RikUnknown, score: 0.2
Vps53Unknown, score: 0.65
Fam114a1Unknown, score: 0.53
Kdelc2Unknown, score: 0.15
Tsr3Unknown, score: 0.32
SycnUnknown, score: 0.31
Ankrd13dUnknown, score: 0.25
Slc39a13Unknown, score: 0.22
Tmem126bUnknown, score: 0.15
Ssna1Unknown, score: 0.16
Mrpl53Unknown, score: 0.14
Ppfia4Unknown, score: 0.2
Zfyve21Unknown, score: 0.37
2310036O22RikUnknown, score: 0.33
Uckl1Unknown, score: 0.34
Tmed10Unknown, score: 0.4
Rtn4Unknown, score: 0.51
Dnajc8Unknown, score: 0.16
Ppm1fUnknown, score: 0.16
Cryl1Unknown, score: 0.36
Tm2d3Unknown, score: 0.19
Samm50Unknown, score: 0.76
Fndc1Unknown, score: 0.21
Pcyt2Unknown, score: 0.31
Slc44a2Unknown, score: 0.24
Gtf2f2Unknown, score: 0.24
Rnf166Unknown, score: 0.41
1110032A03RikUnknown, score: 0.22
Trp53inp2Unknown, score: 0.61
RbfaUnknown, score: 0.35
Lrrc16aUnknown, score: 0.35
Mrps18cUnknown, score: 0.21
Tmem219Unknown, score: 0.52
Wash1Unknown, score: 0.26
Phtf2Unknown, score: 0.63
Gucd1Unknown, score: 0.3
Srpx2Unknown, score: 0.22
FlncUnknown, score: 0.17
Tmem214Unknown, score: 0.59
PdgfrlUnknown, score: 0.21
RgmbUnknown, score: 0.19
Prr32Unknown, score: 0.24
Elovl5Unknown, score: 0.18
Dock5Unknown, score: 0.32
Btbd10Unknown, score: 0.19
Zfand2bUnknown, score: 0.68
1110057K04RikUnknown, score: 0.32
Tulp4Unknown, score: 0.27
Rnf208Unknown, score: 0.66
Klhdc9Unknown, score: 0.16
Rasl11aUnknown, score: 0.2
Tatdn3Unknown, score: 0.14
Zfp580Unknown, score: 0.27
Mcts1Unknown, score: 0.2
Anapc10Unknown, score: 0.25
Pycr2Unknown, score: 0.17
Chac1Unknown, score: 0.29
1810011O10RikUnknown, score: 0.41
1810011H11RikUnknown, score: 0.24
Stoml1Unknown, score: 0.19
Fam58bUnknown, score: 0.54
Cnot8Unknown, score: 0.27
Tusc1Unknown, score: 0.16
Cd209fUnknown, score: 0.18
Tmem110Unknown, score: 0.4
C1qtnf2Unknown, score: 0.33
Ccdc23Unknown, score: 0.55
CarkdUnknown, score: 0.24
Zfp688Unknown, score: 0.16
Zfp397Unknown, score: 0.3
Kctd5Unknown, score: 0.28
CtdsplUnknown, score: 0.15
Sec62Unknown, score: 0.22
3300002I08RikUnknown, score: 0.37
Asb9Unknown, score: 0.18
Slc16a13Unknown, score: 0.51
PacrgUnknown, score: 0.56
1700008O03RikUnknown, score: 0.16
Slc38a4Unknown, score: 0.35
Hist1h4hUnknown, score: 0.15
Cdhr4Unknown, score: 0.47
Clic3Unknown, score: 0.22
1700029J07RikUnknown, score: 0.59
Tsr2Unknown, score: 0.2
1700030J22RikUnknown, score: 0.15
Hemk1Unknown, score: 0.21
Dnase1l1Unknown, score: 0.58
Fam65cUnknown, score: 0.17
Bod1Unknown, score: 0.37
Fastkd3Unknown, score: 0.25
Hfe2Unknown, score: 0.37
Pcgf3Unknown, score: 0.43
Ap5s1Unknown, score: 0.2
Dab2ipUnknown, score: 0.26
Cd164l2Unknown, score: 0.41
Camsap3Unknown, score: 0.29
Ndufaf1Unknown, score: 0.32
TfptUnknown, score: 0.25
Trip13Unknown, score: 0.37
1810026J23RikUnknown, score: 0.52
Slc39a11Unknown, score: 0.23
Polr2fUnknown, score: 0.31
SnrpfUnknown, score: 0.22
Nup43Unknown, score: 0.18
Polr2iUnknown, score: 0.34
Dnah17Unknown, score: 0.19
Trmt10bUnknown, score: 0.17
1700026L06RikUnknown, score: 0.51
Chn2Unknown, score: 0.5
Tspo2Unknown, score: 0.19
Nol7Unknown, score: 0.33
Igsf23Unknown, score: 0.44
2210404O09RikUnknown, score: 0.14
Meaf6Unknown, score: 0.24
Sash1Unknown, score: 0.36
Yars2Unknown, score: 0.4
Mllt3Unknown, score: 0.16
Ogfrl1Unknown, score: 0.16
Tmem143Unknown, score: 0.34
Tbc1d13Unknown, score: 0.2
Ube2cbpUnknown, score: 0.27
Basp1Unknown, score: 0.24
Ppp4r1Unknown, score: 0.22
Kcnip1Unknown, score: 0.4
Bag5Unknown, score: 0.57
Ica1lUnknown, score: 0.32
Cox10Unknown, score: 0.21
Spdl1Unknown, score: 0.18
Tmem70Unknown, score: 0.15
Polr3fUnknown, score: 0.36
Stk26Unknown, score: 0.39
2610034B18RikUnknown, score: 0.31
Tspan15Unknown, score: 0.18
Csnk1g3Unknown, score: 0.17
Polr3bUnknown, score: 0.19
Taf15Unknown, score: 0.55
Wdr77Unknown, score: 0.41
Rprd1bUnknown, score: 0.57
Slc35d2Unknown, score: 0.37
DdoUnknown, score: 0.63
Btf3l4Unknown, score: 0.43
Tmem242Unknown, score: 0.24
Txndc16Unknown, score: 0.15
5730455P16RikUnknown, score: 0.25
Filip1Unknown, score: 0.21
Dnajb14Unknown, score: 0.27
5730508B09RikUnknown, score: 0.34
Med26Unknown, score: 0.28
Dcp2Unknown, score: 0.18
Zcchc8Unknown, score: 0.36
Vcpip1Unknown, score: 0.42
Fam175aUnknown, score: 0.16
Dusp16Unknown, score: 0.3
Gpr137cUnknown, score: 0.68
Angptl6Unknown, score: 0.31
Rasgef1aUnknown, score: 0.35
CgnUnknown, score: 0.64
Hacd2Unknown, score: 0.36
Ggnbp1Unknown, score: 0.21
Klhl30Unknown, score: 0.24
KynuUnknown, score: 0.38
Zdhhc1Unknown, score: 0.44
Ankib1Unknown, score: 0.6
Hmgxb4Unknown, score: 0.45
SpdyaUnknown, score: 0.19
Nsun7Unknown, score: 0.21
Trim69Unknown, score: 0.3
4921539E11RikUnknown, score: 0.14
Mmrn1Unknown, score: 0.36
Eva1cUnknown, score: 0.45
Pgm2l1Unknown, score: 0.34
Spef1Unknown, score: 0.28
D7Ertd443eUnknown, score: 0.27
Hexim2Unknown, score: 0.15
Zfp597Unknown, score: 0.16
Stox2Unknown, score: 0.31
Tssk4Unknown, score: 0.14
UvssaUnknown, score: 0.67
Stx18Unknown, score: 0.23
OxsmUnknown, score: 0.35
NbasUnknown, score: 0.22
AsunUnknown, score: 0.22
Clec12bUnknown, score: 0.44
Otud1Unknown, score: 0.66
Dlg5Unknown, score: 0.7
Spata24Unknown, score: 0.22
Ccdc57Unknown, score: 0.16
Arhgap26Unknown, score: 0.19
Mfap3lUnknown, score: 0.15
Tbc1d9Unknown, score: 0.32
Rcbtb1Unknown, score: 0.16
Riok1Unknown, score: 0.51
Arid5bUnknown, score: 0.28
Chd6Unknown, score: 0.14
5430419D17RikUnknown, score: 0.22
5430427O19RikUnknown, score: 0.39
Arhgap21Unknown, score: 0.33
Mettl13Unknown, score: 0.16
BcorUnknown, score: 0.15
Ppp6r2Unknown, score: 0.38
Alpk1Unknown, score: 0.59
Bbs7Unknown, score: 0.35
GrapUnknown, score: 0.52
Fbxo9Unknown, score: 0.51
Arhgap42Unknown, score: 0.33
Izumo4Unknown, score: 0.34
Gdpd2Unknown, score: 0.18
Senp8Unknown, score: 0.46
Myo1eUnknown, score: 0.28
Zgrf1Unknown, score: 0.27
OptnUnknown, score: 0.31
4930506M07RikUnknown, score: 0.23
Mettl7bUnknown, score: 0.17
Rnf215Unknown, score: 0.18
0610010F05RikUnknown, score: 0.25
GypcUnknown, score: 0.39
Rbm43Unknown, score: 0.16
Cdc5lUnknown, score: 0.35
Armcx3Unknown, score: 0.32
Mus81Unknown, score: 0.39
Dram1Unknown, score: 0.17
Cdc40Unknown, score: 0.17
Osbpl3Unknown, score: 0.21
Vps11Unknown, score: 0.5
Lrwd1Unknown, score: 0.18
Mamdc2Unknown, score: 0.28
Pvrl4Unknown, score: 0.22
Tmprss6Unknown, score: 0.17
Ing3Unknown, score: 0.29
Isyna1Unknown, score: 0.25
Ankle2Unknown, score: 0.45
Slc25a18Unknown, score: 0.16
Mtfr2Unknown, score: 0.34
Ranbp3Unknown, score: 0.23
Tmem50aUnknown, score: 0.38
Kif23Unknown, score: 0.34
Dcaf7Unknown, score: 0.14
Phf7Unknown, score: 0.19
Osgin1Unknown, score: 0.5
1700008I05RikUnknown, score: 0.21
R3hcc1Unknown, score: 0.15
Pdia6Unknown, score: 0.21
Wfdc3Unknown, score: 0.14
Fam217aUnknown, score: 0.3
CenpuUnknown, score: 0.35
Mad2l2Unknown, score: 0.29
Noxo1Unknown, score: 0.79
Apol9bUnknown, score: 0.18
Ppapdc1bUnknown, score: 0.46
Bdh1Unknown, score: 0.16
Tmem79Unknown, score: 0.34
Tmem123Unknown, score: 0.35
Ephx3Unknown, score: 0.47
Cars2Unknown, score: 0.44
Tom1l1Unknown, score: 0.51
Zswim1Unknown, score: 0.15
Tdrd12Unknown, score: 0.23
Ddx28Unknown, score: 0.43
Ercc8Unknown, score: 0.29
1600002H07RikUnknown, score: 0.24
Fundc1Unknown, score: 0.32
Cnpy3Unknown, score: 0.23
Alkbh4Unknown, score: 0.3
Cotl1Unknown, score: 0.52
UrgcpUnknown, score: 0.24
Slc38a10Unknown, score: 0.28
Phf10Unknown, score: 0.42
2010111I01RikUnknown, score: 0.24
Gcnt3Unknown, score: 0.25
2010300C02RikUnknown, score: 0.24
Dusp11Unknown, score: 0.19
Jmjd8Unknown, score: 0.21
Naa50Unknown, score: 0.54
Tpx2Unknown, score: 0.16
2610008E11RikUnknown, score: 0.27
Trub1Unknown, score: 0.45
AdpgkUnknown, score: 0.28
StradaUnknown, score: 0.23
Zfp157Unknown, score: 0.15
Aifm3Unknown, score: 0.31
Mfsd8Unknown, score: 0.29
Dbndd1Unknown, score: 0.27
2510009E07RikUnknown, score: 0.19
Fbxl20Unknown, score: 0.38
Zfp558Unknown, score: 0.3
1700030K09RikUnknown, score: 0.26
Lrrc8eUnknown, score: 0.15
CdaUnknown, score: 0.73
Smim24Unknown, score: 0.47
Plekhf1Unknown, score: 0.17
Lsm11Unknown, score: 0.7
Nkg7Unknown, score: 0.17
Plxdc1Unknown, score: 0.2
Vps9d1Unknown, score: 0.42
Amer1Unknown, score: 0.5
Dusp3Unknown, score: 0.19
Zc2hc1cUnknown, score: 0.22
Tmem175Unknown, score: 0.24
Rbm12b1Unknown, score: 0.17
BrapUnknown, score: 0.19
Pinx1Unknown, score: 0.14
Slc43a1Unknown, score: 0.52
Kcnmb2Unknown, score: 0.33
Ttc30bUnknown, score: 0.42
Ccdc71Unknown, score: 0.19
Htatsf1Unknown, score: 0.33
Slc16a10Unknown, score: 0.25
Hsdl2Unknown, score: 0.17
2610507B11RikUnknown, score: 0.17
Taf4bUnknown, score: 0.16
Dzip1lUnknown, score: 0.26
Rps6kb1Unknown, score: 0.2
Pgam5Unknown, score: 0.38
Hsdl1Unknown, score: 0.32
Zfp566Unknown, score: 0.46
Pcbd2Unknown, score: 0.29
Bclaf1Unknown, score: 0.18
Lin9Unknown, score: 0.26
Bbs5Unknown, score: 0.42
Spats2Unknown, score: 0.26
ZufspUnknown, score: 0.28
Cul4bUnknown, score: 0.38
2700029M09RikUnknown, score: 0.21
Hspa12bUnknown, score: 0.43
TdrkhUnknown, score: 0.25
LinsUnknown, score: 0.25
Ccdc12Unknown, score: 0.16
2700094K13RikUnknown, score: 0.32
Serp2Unknown, score: 0.14
Skida1Unknown, score: 0.31
Adipor1Unknown, score: 0.23
Grrp1Unknown, score: 0.16
C1qtnf6Unknown, score: 0.21
Angptl1Unknown, score: 0.15
TonslUnknown, score: 0.23
72775Unknown, score: 0.29
Sass6Unknown, score: 0.17
Zfp839Unknown, score: 0.24
Zfp429Unknown, score: 0.17
Scn2bUnknown, score: 0.24
Mon1aUnknown, score: 0.16
Fam76bUnknown, score: 0.55
AC182748.2Unknown, score: 0.16
Prdm4Unknown, score: 0.16
Ndufv2Unknown, score: 0.29
PhykplUnknown, score: 0.46
Ccnt2Unknown, score: 0.54
Top1mtUnknown, score: 0.23
PrkrirUnknown, score: 0.18
Tmem138Unknown, score: 0.48
Fut11Unknown, score: 0.16
Prr36Unknown, score: 0.53
Ppil6Unknown, score: 0.22
PmpcbUnknown, score: 0.15
Rps6ka5Unknown, score: 0.39
3110009E18RikUnknown, score: 0.2
Prss57Unknown, score: 0.14
Fam101aUnknown, score: 0.14
Tgfbrap1Unknown, score: 0.65
Clec4a3Unknown, score: 0.17
Arhgap8Unknown, score: 0.15
Exo5Unknown, score: 0.17
XpotUnknown, score: 0.25
Fam118aUnknown, score: 0.2
BmperUnknown, score: 0.37
Zfp942Unknown, score: 0.14
Atat1Unknown, score: 0.34
Setd7Unknown, score: 0.23
1700037C18RikUnknown, score: 0.29
1700040L02RikUnknown, score: 0.24
Lrrc69Unknown, score: 0.33
NptxrUnknown, score: 0.31
Col20a1Unknown, score: 0.46
Prox2Unknown, score: 0.15
Spata18Unknown, score: 0.15
Cybrd1Unknown, score: 0.49
Ttc21bUnknown, score: 0.19
Rec114Unknown, score: 0.2
Bloc1s2Unknown, score: 0.43
Glipr1Unknown, score: 0.25
Ndufaf7Unknown, score: 0.39
Gucy2gUnknown, score: 0.33
Mvb12aUnknown, score: 0.59
Cst6Unknown, score: 0.23
1110008P14RikUnknown, score: 0.35
Haus7Unknown, score: 0.22
Man2c1Unknown, score: 0.29
Thap1Unknown, score: 0.38
Poldip3Unknown, score: 0.34
Ankrd45Unknown, score: 0.24
D3Ertd751eUnknown, score: 0.21
Zdbf2Unknown, score: 0.17
Psmb11Unknown, score: 0.17
Arhgap18Unknown, score: 0.4
Irak3Unknown, score: 0.4
74004Unknown, score: 0.16
Slc25a27Unknown, score: 0.31
Cpne4Unknown, score: 0.17
Msl1Unknown, score: 0.17
Nol9Unknown, score: 0.25
Nfam1Unknown, score: 0.28
Ttf2Unknown, score: 0.27
4632428N05RikUnknown, score: 0.66
Steap2Unknown, score: 0.19
Grip1Unknown, score: 0.2
Tjap1Unknown, score: 0.45
Slc35a5Unknown, score: 0.2
NeblUnknown, score: 0.31
Gga2Unknown, score: 0.15
Dcaf6Unknown, score: 0.41
Cep55Unknown, score: 0.2
Rbm19Unknown, score: 0.63
CrotUnknown, score: 0.48
Pi16Unknown, score: 0.18
AcoxlUnknown, score: 0.32
Syvn1Unknown, score: 0.6
DmgdhUnknown, score: 0.25
Sash3Unknown, score: 0.17
Smg8Unknown, score: 0.26
Robo4Unknown, score: 0.33
CluhUnknown, score: 0.3
Slc35f5Unknown, score: 0.39
Cmtr1Unknown, score: 0.25
Josd1Unknown, score: 0.26
Zdhhc16Unknown, score: 0.65
Muc5bUnknown, score: 0.4
Arpc5lUnknown, score: 0.36
Fblim1Unknown, score: 0.39
Xpo6Unknown, score: 0.43
Rbm26Unknown, score: 0.22
ChpfUnknown, score: 0.28
Smu1Unknown, score: 0.35
Mtmr3Unknown, score: 0.32
HnrnprUnknown, score: 0.37
Zfp84Unknown, score: 0.17
LrgukUnknown, score: 0.17
RptorUnknown, score: 0.42
Myo18bUnknown, score: 0.18
Ubap2lUnknown, score: 0.16
Rmi1Unknown, score: 0.34
Tc2nUnknown, score: 0.16
Eaf1Unknown, score: 0.16
Lrriq3Unknown, score: 0.37
Clvs1Unknown, score: 0.67
CmipUnknown, score: 0.69
Arl13aUnknown, score: 0.29
4933427G17RikUnknown, score: 0.27
4933433C11RikUnknown, score: 0.31
Samd4Unknown, score: 0.27
MamstrUnknown, score: 0.27
Fam53aUnknown, score: 0.38
Rasgef1cUnknown, score: 0.49
MlklUnknown, score: 0.17
LvrnUnknown, score: 0.15
Glb1lUnknown, score: 0.21
Kbtbd12Unknown, score: 0.22
Abca12Unknown, score: 0.16
Scrn3Unknown, score: 0.6
Ptchd3Unknown, score: 0.37
Spata17Unknown, score: 0.21
SetmarUnknown, score: 0.16
RhohUnknown, score: 0.15
Ddit4Unknown, score: 0.23
Rab3il1Unknown, score: 0.4
Mdga1Unknown, score: 0.28
Yipf2Unknown, score: 0.15
Ppa2Unknown, score: 0.43
Wipi2Unknown, score: 0.22
Glt8d2Unknown, score: 0.22
Tmem65Unknown, score: 0.35
Ccdc181Unknown, score: 0.3
Slc35f6Unknown, score: 0.16
Armc4Unknown, score: 0.46
Usp47Unknown, score: 0.3
Fam63aUnknown, score: 0.37
Rnase10Unknown, score: 0.37
Kif27Unknown, score: 0.41
Ccdc173Unknown, score: 0.15
Usp50Unknown, score: 0.17
4930505A04RikUnknown, score: 0.41
Tmem180Unknown, score: 0.33
Ccdc146Unknown, score: 0.37
4930538K18RikUnknown, score: 0.32
Rnf121Unknown, score: 0.25
Cep128Unknown, score: 0.64
Dpp3Unknown, score: 0.27
Zbtb3Unknown, score: 0.27
Fgfr1opUnknown, score: 0.33
Taf1dUnknown, score: 0.26
4930563D23RikUnknown, score: 0.32
Spp2Unknown, score: 0.16
Mrpl32Unknown, score: 0.26
Ndufs7Unknown, score: 0.14
Kmt2bUnknown, score: 0.17
Arl5aUnknown, score: 0.28
Tti1Unknown, score: 0.65
Igfbpl1Unknown, score: 0.72
Anapc15Unknown, score: 0.17
Dynlrb2Unknown, score: 0.31
Ttc32Unknown, score: 0.34
Tex29Unknown, score: 0.2
Lyrm7Unknown, score: 0.56
1700019G17RikUnknown, score: 0.21
Paqr9Unknown, score: 0.18
Dnali1Unknown, score: 0.25
FggyUnknown, score: 0.24
Pcdh1Unknown, score: 0.18
Calml4Unknown, score: 0.23
Chmp4bUnknown, score: 0.16
Med25Unknown, score: 0.25
Rps25Unknown, score: 0.19
Spaca3Unknown, score: 0.32
Mageh1Unknown, score: 0.19
Spata25Unknown, score: 0.27
Ssmem1Unknown, score: 0.18
Pik3r4Unknown, score: 0.17
Cldn22Unknown, score: 0.19
IppkUnknown, score: 0.18
Fam65aUnknown, score: 0.3
Bcl2l12Unknown, score: 0.24
Morc4Unknown, score: 0.35
9130401M01RikUnknown, score: 0.23
Slx1bUnknown, score: 0.26
DcstampUnknown, score: 0.33
Brsk2Unknown, score: 0.28
Cdyl2Unknown, score: 0.22
Wdr64Unknown, score: 0.73
Zfp821Unknown, score: 0.23
Dcp1aUnknown, score: 0.18
4930578C19RikUnknown, score: 0.17
Fam184aUnknown, score: 0.43
Srrm2Unknown, score: 0.42
Trappc8Unknown, score: 0.42
75973Unknown, score: 0.43
Dock11Unknown, score: 0.17
AgmatUnknown, score: 0.17
Slain2Unknown, score: 0.24
Ccdc125Unknown, score: 0.5
Jakmip1Unknown, score: 0.2
Rap2aUnknown, score: 0.16
Arhgap15Unknown, score: 0.61
Las1lUnknown, score: 0.24
Depdc1aUnknown, score: 0.36
Mcur1Unknown, score: 0.25
Ppp1r14cUnknown, score: 0.36
Usp31Unknown, score: 0.29
Ercc6l2Unknown, score: 0.17
Atp6v0e2Unknown, score: 0.4
Slc38a3Unknown, score: 0.49
Tsen54Unknown, score: 0.34
Cyp2d26Unknown, score: 0.19
GptUnknown, score: 0.79
Asb5Unknown, score: 0.4
Atp11bUnknown, score: 0.17
Erp44Unknown, score: 0.29
Cog2Unknown, score: 0.23
TgdsUnknown, score: 0.63
1700019B03RikUnknown, score: 0.3
Ift43Unknown, score: 0.41
1700028K03RikUnknown, score: 0.19
Daam2Unknown, score: 0.21
Ppp1r18Unknown, score: 0.53
Ccdc134Unknown, score: 0.38
Msrb2Unknown, score: 0.35
Cmya5Unknown, score: 0.36
Haus8Unknown, score: 0.28
Ly6kUnknown, score: 0.18
Atg2bUnknown, score: 0.22
Fam101bUnknown, score: 0.19
Mfsd2aUnknown, score: 0.22
Msi2Unknown, score: 0.45
Wdr38Unknown, score: 0.47
Srxn1Unknown, score: 0.26
Upp2Unknown, score: 0.18
Trim12aUnknown, score: 0.19
Creld2Unknown, score: 0.18
TrdnUnknown, score: 0.15
Cluap1Unknown, score: 0.21
Ppfia3Unknown, score: 0.46
2410137M14RikUnknown, score: 0.24
Tmem234Unknown, score: 0.14
Calcoco2Unknown, score: 0.25
Sdccag8Unknown, score: 0.28
NubplUnknown, score: 0.37
Dok5Unknown, score: 0.15
Hyls1Unknown, score: 0.26
Ago4Unknown, score: 0.26
Catsper3Unknown, score: 0.37
Dcun1d5Unknown, score: 0.21
Timmdc1Unknown, score: 0.22
Arrdc5Unknown, score: 0.26
1700015E13RikUnknown, score: 0.62
Chmp5Unknown, score: 0.21
Bcas1Unknown, score: 0.76
Ddrgk1Unknown, score: 0.29
Mpped2Unknown, score: 0.16
Slc9a8Unknown, score: 0.35
2510039O18RikUnknown, score: 0.15
Atg16l1Unknown, score: 0.31
Bcl7aUnknown, score: 0.36
Ints7Unknown, score: 0.28
Ocel1Unknown, score: 0.15
Klhl2Unknown, score: 0.22
Mtmr2Unknown, score: 0.3
Zfp934Unknown, score: 0.66
Ptgr2Unknown, score: 0.24
NkrfUnknown, score: 0.27
Wdr82Unknown, score: 0.18
Ankrd55Unknown, score: 0.17
C330018D20RikUnknown, score: 0.4
Zfp266Unknown, score: 0.38
Mtus2Unknown, score: 0.19
Anks1bUnknown, score: 0.16
Vps33aUnknown, score: 0.31
Ddx10Unknown, score: 0.33
Adgrf1Unknown, score: 0.16
Prss36Unknown, score: 0.29
Mrps5Unknown, score: 0.22
Rnf170Unknown, score: 0.18
Elp4Unknown, score: 0.2
PolqUnknown, score: 0.32
Sla2Unknown, score: 0.18
TchpUnknown, score: 0.16
Ypel2Unknown, score: 0.21
Hook1Unknown, score: 0.19
Sbf1Unknown, score: 0.47
Mccc2Unknown, score: 0.25
SowahbUnknown, score: 0.26
Msantd4Unknown, score: 0.27
Cox7b2Unknown, score: 0.5
9230112D13RikUnknown, score: 0.28
Phf23Unknown, score: 0.47
Nxpe2Unknown, score: 0.34
Nav2Unknown, score: 0.15
Ndufv3Unknown, score: 0.16
Nudt17Unknown, score: 0.14
WibgUnknown, score: 0.22
HelzUnknown, score: 0.25
Srbd1Unknown, score: 0.29
Pde6hUnknown, score: 0.15
CstadUnknown, score: 0.45
Acap2Unknown, score: 0.23
1700061G19RikUnknown, score: 0.21
Pus7Unknown, score: 0.23
TroapUnknown, score: 0.52
Filip1lUnknown, score: 0.38
Zc3h6Unknown, score: 0.37
Galnt15Unknown, score: 0.72
Efcab11Unknown, score: 0.55
Hhipl2Unknown, score: 0.61
Spata2lUnknown, score: 0.32
Celf3Unknown, score: 0.15
Usp54Unknown, score: 0.14
Ndor1Unknown, score: 0.15
Eml4Unknown, score: 0.22
Stpg1Unknown, score: 0.46
Stxbp5Unknown, score: 0.14
GmipUnknown, score: 0.18
Slc25a12Unknown, score: 0.15
Cacul1Unknown, score: 0.26
Coro7Unknown, score: 0.26
Cnot10Unknown, score: 0.21
AacsUnknown, score: 0.2
Pus7lUnknown, score: 0.48
MispUnknown, score: 0.68
Sp2Unknown, score: 0.37
Nadsyn1Unknown, score: 0.15
9130019O22RikUnknown, score: 0.36
Srd5a1Unknown, score: 0.48
Avl9Unknown, score: 0.17
Ern1Unknown, score: 0.54
Trim39Unknown, score: 0.41
Recql4Unknown, score: 0.15
BC005537Unknown, score: 0.32
Ublcp1Unknown, score: 0.35
Cdk5rap3Unknown, score: 0.15
Tusc3Unknown, score: 0.19
Bcl9lUnknown, score: 0.29
ZxdcUnknown, score: 0.21
Sptbn4Unknown, score: 0.16
Herpud2Unknown, score: 0.23
Pacsin3Unknown, score: 0.59
Rab27bUnknown, score: 0.33
Vps16Unknown, score: 0.18
Cwc22Unknown, score: 0.16
SelkUnknown, score: 0.28
Clca3a2Unknown, score: 0.35
Fgf20Unknown, score: 0.46
NfkbizUnknown, score: 0.27
GhdcUnknown, score: 0.16
Dhx58Unknown, score: 0.26
LrbaUnknown, score: 0.64
Ntng1Unknown, score: 0.61
Mesdc1Unknown, score: 0.27
Erap1Unknown, score: 0.14
Fgf16Unknown, score: 0.31
LactbUnknown, score: 0.56
Gatsl2Unknown, score: 0.24
Gpr84Unknown, score: 0.26
Acox3Unknown, score: 0.24
Dusp12Unknown, score: 0.23
Arl4dUnknown, score: 0.15
CemipUnknown, score: 0.35
Trim44Unknown, score: 0.4
Sil1Unknown, score: 0.21
Ankrd17Unknown, score: 0.25
Jdp2Unknown, score: 0.67
Sorcs2Unknown, score: 0.31
Tfcp2l1Unknown, score: 0.26
Tlr9Unknown, score: 0.26
Zfpl1Unknown, score: 0.27
Tfap4Unknown, score: 0.15
Sp6Unknown, score: 0.38
Glis2Unknown, score: 0.48
Ndel1Unknown, score: 0.19
Trem2Unknown, score: 0.6
Rbm5Unknown, score: 0.14
Pik3ap1Unknown, score: 0.23
Sacm1lUnknown, score: 0.3
Lin28aUnknown, score: 0.14
Cnnm1Unknown, score: 0.35
Pde4dipUnknown, score: 0.33
Nedd4lUnknown, score: 0.33
Slc25a2Unknown, score: 0.65
Gpr137bUnknown, score: 0.6
Dnaja3Unknown, score: 0.23
Jam3Unknown, score: 0.19
SlmapUnknown, score: 0.37
Pi4k2aUnknown, score: 0.22
Rnf123Unknown, score: 0.28
Tnfrsf25Unknown, score: 0.59
Trim8Unknown, score: 0.3
Zkscan8Unknown, score: 0.14
GlceUnknown, score: 0.6
Sep15Unknown, score: 0.48
Lmod1Unknown, score: 0.22
GlrxUnknown, score: 0.37
Clec2dUnknown, score: 0.48
Rnase2aUnknown, score: 0.34
Lztfl1Unknown, score: 0.42
Wnt16Unknown, score: 0.34
Arid1aUnknown, score: 0.31
Ube2nUnknown, score: 0.14
Rnf111Unknown, score: 0.24
Igsf9Unknown, score: 0.3
Brwd1Unknown, score: 0.21
Pcdhb2Unknown, score: 0.21
Pcdhb4Unknown, score: 0.24
Pcdhb7Unknown, score: 0.22
Pcdhb8Unknown, score: 0.47
Pcdhb10Unknown, score: 0.19
Pcdhb11Unknown, score: 0.21
Pcdhb12Unknown, score: 0.26
Pcdhb13Unknown, score: 0.58
Pcdhb15Unknown, score: 0.52
Pcdhb16Unknown, score: 0.45
Pcdhb17Unknown, score: 0.5
Pcdhb18Unknown, score: 0.48
Pcdhb19Unknown, score: 0.45
Pcdhb21Unknown, score: 0.26
Mrpl3Unknown, score: 0.29
Trim16Unknown, score: 0.17
Trim34aUnknown, score: 0.22
HrgUnknown, score: 0.19
Acsbg1Unknown, score: 0.15
Tnfrsf21Unknown, score: 0.23
Strn3Unknown, score: 0.34
Adarb2Unknown, score: 0.49
Cnnm4Unknown, score: 0.46
S1pr5Unknown, score: 0.51
Dtnbp1Unknown, score: 0.3
Slc24a3Unknown, score: 0.23
Sfxn2Unknown, score: 0.29
PrccUnknown, score: 0.46
Tmem40Unknown, score: 0.65
Loxl2Unknown, score: 0.21
Hmgn3Unknown, score: 0.63
TprnUnknown, score: 0.72
Wwtr1Unknown, score: 0.29
Slc9b2Unknown, score: 0.29
Nmd3Unknown, score: 0.3
Hist2h4Unknown, score: 0.37
Strn4Unknown, score: 0.22
QarsUnknown, score: 0.29
Sgsm2Unknown, score: 0.16
Exd2Unknown, score: 0.19
C78339Unknown, score: 0.41
Nlrp4fUnknown, score: 0.21
Gtf2f1Unknown, score: 0.15
KmoUnknown, score: 0.14
Txndc9Unknown, score: 0.32
D2hgdhUnknown, score: 0.48
Slamf9Unknown, score: 0.36
Smap1Unknown, score: 0.25
Slc41a1Unknown, score: 0.34
Sh3bp4Unknown, score: 0.23
Gtf3c3Unknown, score: 0.37
Atp1a2Unknown, score: 0.28
AI182371Unknown, score: 0.3
Ehd4Unknown, score: 0.34
Usp6nlUnknown, score: 0.61
Znfx1Unknown, score: 0.18
Mrps26Unknown, score: 0.42
Stard7Unknown, score: 0.61
Anapc2Unknown, score: 0.42
Commd7Unknown, score: 0.18
Garnl3Unknown, score: 0.38
Golga2Unknown, score: 0.67
Magi3Unknown, score: 0.32
Wdr47Unknown, score: 0.34
Usp53Unknown, score: 0.55
FggUnknown, score: 0.25
4933434E20RikUnknown, score: 0.17
Kcnc4Unknown, score: 0.35
Tmem56Unknown, score: 0.26
Prmt6Unknown, score: 0.31
Ifi44Unknown, score: 0.38
TiparpUnknown, score: 0.19
Phactr4Unknown, score: 0.17
Stx12Unknown, score: 0.25
Smpdl3bUnknown, score: 0.2
Fam46bUnknown, score: 0.15
Zfp518bUnknown, score: 0.29
Spon2Unknown, score: 0.19
AcacbUnknown, score: 0.25
Ugt2b34Unknown, score: 0.18
Chpf2Unknown, score: 0.15
Tyw1Unknown, score: 0.33
Nfxl1Unknown, score: 0.26
Itfg2Unknown, score: 0.18
Pot1aUnknown, score: 0.27
Zfp956Unknown, score: 0.31
Brk1Unknown, score: 0.15
Fbxl14Unknown, score: 0.23
Phrf1Unknown, score: 0.46
Plekha1Unknown, score: 0.37
Inpp5fUnknown, score: 0.39
WtipUnknown, score: 0.18
Zfp575Unknown, score: 0.22
Ccp110Unknown, score: 0.24
Vrk3Unknown, score: 0.15
Nlrp6Unknown, score: 0.24
Pwwp2bUnknown, score: 0.14
Spty2d1Unknown, score: 0.28
Ano1Unknown, score: 0.31
AW146154Unknown, score: 0.41
Ints4Unknown, score: 0.16
DohhUnknown, score: 0.21
Fam192aUnknown, score: 0.35
Enkd1Unknown, score: 0.24
Zdhhc7Unknown, score: 0.38
Cpne7Unknown, score: 0.45
Lars2Unknown, score: 0.56
Snx19Unknown, score: 0.52
Rpp25Unknown, score: 0.49
Acad11Unknown, score: 0.27
Phldb1Unknown, score: 0.19
TctaUnknown, score: 0.18
Slc6a8Unknown, score: 0.19
D330045A20RikUnknown, score: 0.33
Nudt10Unknown, score: 0.38
Sept10Unknown, score: 0.2
Chchd10Unknown, score: 0.41
Csnk1g2Unknown, score: 0.24
Tmem263Unknown, score: 0.2
Nup107Unknown, score: 0.68
Smg6Unknown, score: 0.2
Mien1Unknown, score: 0.3
Slc25a41Unknown, score: 0.56
Zfp692Unknown, score: 0.15
Rpn1Unknown, score: 0.21
GckUnknown, score: 0.19
Synj1Unknown, score: 0.19
SncbUnknown, score: 0.25
Nxph4Unknown, score: 0.18
Itga9Unknown, score: 0.21
Ces1dUnknown, score: 0.35
Cdc42ep2Unknown, score: 0.55
Arl1Unknown, score: 0.2
Csnk1dUnknown, score: 0.26
Rcor2Unknown, score: 0.22
0610010K14RikUnknown, score: 0.21
RprmlUnknown, score: 0.4
MycbpapUnknown, score: 0.44
Vipas39Unknown, score: 0.48
Tmem179Unknown, score: 0.48
Fam110cUnknown, score: 0.29
IarsUnknown, score: 0.71
Nhlrc1Unknown, score: 0.34
Rnf44Unknown, score: 0.29
Slc9a3Unknown, score: 0.21
Txndc5Unknown, score: 0.25
Gmpr2Unknown, score: 0.21
Abhd4Unknown, score: 0.31
Ankrd28Unknown, score: 0.51
Mycbp2Unknown, score: 0.71
ScribUnknown, score: 0.32
Kdelr3Unknown, score: 0.31
Sgsm3Unknown, score: 0.14
Mal2Unknown, score: 0.4
Nckap1lUnknown, score: 0.17
105892Unknown, score: 0.25
Fam19a5Unknown, score: 0.19
Mfsd5Unknown, score: 0.32
Txndc11Unknown, score: 0.31
Nsun3Unknown, score: 0.32
SrlUnknown, score: 0.55
Stk38Unknown, score: 0.36
Scaf8Unknown, score: 0.23
G6bUnknown, score: 0.34
Dhx57Unknown, score: 0.24
Afap1l1Unknown, score: 0.39
Arap3Unknown, score: 0.47
Gramd3Unknown, score: 0.19
Vps37cUnknown, score: 0.3
Tm9sf3Unknown, score: 0.29
Exoc6Unknown, score: 0.25
Guca1bUnknown, score: 0.16
Atf5Unknown, score: 0.26
Ssr1Unknown, score: 0.25
Ece2Unknown, score: 0.32
Gimap4Unknown, score: 0.16
Il1rl2Unknown, score: 0.31
Arl2bpUnknown, score: 0.23
Nt5c3Unknown, score: 0.66
Pi4kbUnknown, score: 0.3
Mrpl30Unknown, score: 0.37
BmycUnknown, score: 0.18
Jmjd6Unknown, score: 0.37
Pacs1Unknown, score: 0.21
Ddb2Unknown, score: 0.75
Bfsp2Unknown, score: 0.27
Gtpbp6Unknown, score: 0.4
Ap4e1Unknown, score: 0.79
Srsf9Unknown, score: 0.33
Eif2b3Unknown, score: 0.28
Grm7Unknown, score: 0.15
Skiv2lUnknown, score: 0.47
Prkag2Unknown, score: 0.34
Baiap2Unknown, score: 0.16
Slco4a1Unknown, score: 0.26
NapaUnknown, score: 0.27
AticUnknown, score: 0.22
Galnt2Unknown, score: 0.22
Galnt7Unknown, score: 0.68
Adamts7Unknown, score: 0.22
Ubxn8Unknown, score: 0.36
Fam210aUnknown, score: 0.28
108660Unknown, score: 0.16
Dnajc9Unknown, score: 0.49
Gpt2Unknown, score: 0.42
Edem2Unknown, score: 0.18
Lyrm2Unknown, score: 0.25
Mex3bUnknown, score: 0.22
Calr4Unknown, score: 0.19
Ccdc122Unknown, score: 0.37
Tmem74bUnknown, score: 0.23
Atad3aUnknown, score: 0.3
TbcdUnknown, score: 0.36
Nusap1Unknown, score: 0.4
Rcc2Unknown, score: 0.34
LhfpUnknown, score: 0.29
Fam73bUnknown, score: 0.19
E2f8Unknown, score: 0.23
Tbc1d10cUnknown, score: 0.2
Ciapin1Unknown, score: 0.24
Nabp1Unknown, score: 0.23
Sp110Unknown, score: 0.2
PrkcdbpUnknown, score: 0.34
Sephs1Unknown, score: 0.3
Gins4Unknown, score: 0.29
Chd9Unknown, score: 0.15
Ube2q2Unknown, score: 0.35
Atl3Unknown, score: 0.29
SobpUnknown, score: 0.35
Rarres1Unknown, score: 0.67
SccpdhUnknown, score: 0.29
Lrrc39Unknown, score: 0.21
AdtrpUnknown, score: 0.19
Ssc4dUnknown, score: 0.16
R3hdm4Unknown, score: 0.21
Orai1Unknown, score: 0.67
C1qtnf7Unknown, score: 0.2
Cdcp1Unknown, score: 0.35
Fam163bUnknown, score: 0.38
Fam175bUnknown, score: 0.24
SriUnknown, score: 0.22
DspUnknown, score: 0.45
Hyal3Unknown, score: 0.41
Cpa1Unknown, score: 0.63
Itga1Unknown, score: 0.44
MaobUnknown, score: 0.56
Cyb5r3Unknown, score: 0.28
BlvraUnknown, score: 0.17
Pgm3Unknown, score: 0.17
VimpUnknown, score: 0.15
Cbr3Unknown, score: 0.28
AslUnknown, score: 0.27
Mcf2Unknown, score: 0.38
Rap1aUnknown, score: 0.26
AbrUnknown, score: 0.2
Art3Unknown, score: 0.56
Tpgs1Unknown, score: 0.41
Kif22Unknown, score: 0.37
DekUnknown, score: 0.34
DutUnknown, score: 0.38
Bmp3Unknown, score: 0.15
110083Unknown, score: 0.35
Phka2Unknown, score: 0.4
Akr7a5Unknown, score: 0.32
MsraUnknown, score: 0.6
BcrUnknown, score: 0.17
Pp2d1Unknown, score: 0.39
Dync2h1Unknown, score: 0.19
Sec13Unknown, score: 0.25
Acat1Unknown, score: 0.15
Acat2Unknown, score: 0.19
DgkqUnknown, score: 0.21
Adarb1Unknown, score: 0.26
Grik4Unknown, score: 0.58
Prps2Unknown, score: 0.46
Aldh7a1Unknown, score: 0.28
EtfbUnknown, score: 0.26
Ppp2r4Unknown, score: 0.27
Slc18a1Unknown, score: 0.14
Slc8a2Unknown, score: 0.29
Cds2Unknown, score: 0.43
Egln2Unknown, score: 0.37
Egln3Unknown, score: 0.2
2010002M12RikUnknown, score: 0.22
Vmn1r42Unknown, score: 0.16
Laptm4bUnknown, score: 0.4
Foxp2Unknown, score: 0.17
Dok4Unknown, score: 0.33
Slc28a3Unknown, score: 0.19
Slc5a5Unknown, score: 0.16
Zbtb21Unknown, score: 0.44
Crip3Unknown, score: 0.54
Rpl31Unknown, score: 0.37
BrdtUnknown, score: 0.14
Hsd17b11Unknown, score: 0.19
Rasa2Unknown, score: 0.37
Spred2Unknown, score: 0.5
Ddhd1Unknown, score: 0.3
CygbUnknown, score: 0.48
Fgfrl1Unknown, score: 0.2
Lsm10Unknown, score: 0.16
Rims2Unknown, score: 0.4
Mta3Unknown, score: 0.31
Derl2Unknown, score: 0.47
Tgs1Unknown, score: 0.29
Pop5Unknown, score: 0.22
Ttyh2Unknown, score: 0.14
Ivns1abpUnknown, score: 0.36
Asb10Unknown, score: 0.39
HelbUnknown, score: 0.17
BocUnknown, score: 0.2
Gjc2Unknown, score: 0.28
Zfp358Unknown, score: 0.24
Igf2bp3Unknown, score: 0.33
Kcnn2Unknown, score: 0.43
Kcnn3Unknown, score: 0.56
Atp6v0a4Unknown, score: 0.55
AF251705Unknown, score: 0.32
Rxfp2Unknown, score: 0.2
Smc1bUnknown, score: 0.21
Plxnb3Unknown, score: 0.28
Elmo2Unknown, score: 0.4
Ubox5Unknown, score: 0.47
Ube4aUnknown, score: 0.18
Cacng5Unknown, score: 0.27
Sesn1Unknown, score: 0.49
Myh7Unknown, score: 0.37
Il25Unknown, score: 0.18
Nek8Unknown, score: 0.2
Asb13Unknown, score: 0.42
Tlr3Unknown, score: 0.34
Elovl6Unknown, score: 0.22
Slc2a10Unknown, score: 0.2
Sp7Unknown, score: 0.19
Olfr78Unknown, score: 0.55
KirrelUnknown, score: 0.5
Ubn1Unknown, score: 0.18
Tmem37Unknown, score: 0.14
Idh3bUnknown, score: 0.19
Oxr1Unknown, score: 0.28
Kcnh7Unknown, score: 0.15
Sertad3Unknown, score: 0.29
Tlr7Unknown, score: 0.4
Tlr8Unknown, score: 0.14
Mtmr4Unknown, score: 0.19
Sgk3Unknown, score: 0.14
Slc8b1Unknown, score: 0.15
Atp13a1Unknown, score: 0.42
Pdzd3Unknown, score: 0.38
Nup155Unknown, score: 0.32
Bbc3Unknown, score: 0.16
Glcci1Unknown, score: 0.19
Cd209dUnknown, score: 0.23
Hdac10Unknown, score: 0.45
Rbm39Unknown, score: 0.29
Ppargc1bUnknown, score: 0.75
Tram2Unknown, score: 0.16
Grid2ipUnknown, score: 0.21
Zfp617Unknown, score: 0.31
Ntng2Unknown, score: 0.26
Timd2Unknown, score: 0.4
Havcr2Unknown, score: 0.38
Slc12a8Unknown, score: 0.41
Cd99l2Unknown, score: 0.56
Creld1Unknown, score: 0.16
BmfUnknown, score: 0.34
Mical1Unknown, score: 0.17
HelqUnknown, score: 0.22
MvdUnknown, score: 0.54
Casc3Unknown, score: 0.2
Pcdha9Unknown, score: 0.2
Rwdd4aUnknown, score: 0.22
Stab1Unknown, score: 0.4
Stab2Unknown, score: 0.46
Med9Unknown, score: 0.22
Shkbp1Unknown, score: 0.18
Ash1lUnknown, score: 0.2
Bcas3Unknown, score: 0.74
Prom2Unknown, score: 0.3
Hps4Unknown, score: 0.14
Slc25a36Unknown, score: 0.35
Pla2g15Unknown, score: 0.22
Ripk2Unknown, score: 0.55
Lrrc75bUnknown, score: 0.32
Rapgef6Unknown, score: 0.5
Itgb4Unknown, score: 0.3
Dhrs11Unknown, score: 0.29
Lrrc75aUnknown, score: 0.23
Cyb5d2Unknown, score: 0.5
Zfp3Unknown, score: 0.19
Zbtb12Unknown, score: 0.41
Eme2Unknown, score: 0.37
Cnksr1Unknown, score: 0.26
Tet3Unknown, score: 0.3
Sun3Unknown, score: 0.24
Nlrp1aUnknown, score: 0.25
195209Unknown, score: 0.18
Zfp691Unknown, score: 0.18
Cetn4Unknown, score: 0.26
Fbxo40Unknown, score: 0.29
Zbtb7cUnknown, score: 0.27
Fchsd2Unknown, score: 0.19
Fam120cUnknown, score: 0.32
Wdr11Unknown, score: 0.33
Baiap2l2Unknown, score: 0.23
Dtx4Unknown, score: 0.35
Thsd4Unknown, score: 0.17
Skor1Unknown, score: 0.15
Igsf11Unknown, score: 0.4
Bzrap1Unknown, score: 0.22
Csrnp2Unknown, score: 0.14
BC034090Unknown, score: 0.17
Gramd1cUnknown, score: 0.32
SmagpUnknown, score: 0.18
4930539E08RikUnknown, score: 0.25
Fmr1nbUnknown, score: 0.2
Fam228bUnknown, score: 0.15
Alg11Unknown, score: 0.38
VcpkmtUnknown, score: 0.32
Ubap1lUnknown, score: 0.23
Map6d1Unknown, score: 0.18
Fam180aUnknown, score: 0.23
Cyp4f17Unknown, score: 0.34
Shroom4Unknown, score: 0.46
Dip2cUnknown, score: 0.33
Alg3Unknown, score: 0.34
Creb3l2Unknown, score: 0.52
CblbUnknown, score: 0.2
Creb3l3Unknown, score: 0.34
Eif5a2Unknown, score: 0.26
Hdac4Unknown, score: 0.69
Tmem63aUnknown, score: 0.26
Daam1Unknown, score: 0.48
Zdhhc9Unknown, score: 0.18
Myo5cUnknown, score: 0.32
Thnsl1Unknown, score: 0.24
NpbUnknown, score: 0.17
Pycr1Unknown, score: 0.48
Gipc3Unknown, score: 0.48
Snx30Unknown, score: 0.14
Zfp710Unknown, score: 0.15
Gtf2h3Unknown, score: 0.41
Taf3Unknown, score: 0.51
GpkowUnknown, score: 0.21
Hace1Unknown, score: 0.17
Hsh2dUnknown, score: 0.27
Nudcd3Unknown, score: 0.16
Frmd4aUnknown, score: 0.27
LcorlUnknown, score: 0.3
Tmc7Unknown, score: 0.22
MtrrUnknown, score: 0.17
Tmem194Unknown, score: 0.22
D130043K22RikUnknown, score: 0.39
LppUnknown, score: 0.29
Shank2Unknown, score: 0.41
Dock10Unknown, score: 0.47
Mtmr9Unknown, score: 0.25
Mettl14Unknown, score: 0.5
P3h2Unknown, score: 0.17
Tbc1d31Unknown, score: 0.16
Coq10aUnknown, score: 0.48
Prrt3Unknown, score: 0.31
Gab3Unknown, score: 0.24
ThemisUnknown, score: 0.29
SepsecsUnknown, score: 0.23
March11Unknown, score: 0.14
Kbtbd7Unknown, score: 0.21
Nrg1Unknown, score: 0.61
Ncoa7Unknown, score: 0.28
Exoc3Unknown, score: 0.42
Kcnj14Unknown, score: 0.21
Nomo1Unknown, score: 0.33
Tfdp2Unknown, score: 0.2
Plac9aUnknown, score: 0.24
Wwc1Unknown, score: 0.45
Arfgef1Unknown, score: 0.29
Trib1Unknown, score: 0.26
Mfsd9Unknown, score: 0.19
Depdc7Unknown, score: 0.59
Asap2Unknown, score: 0.19
Plekhh1Unknown, score: 0.5
Inpp5aUnknown, score: 0.31
2610015P09RikUnknown, score: 0.18
Zswim4Unknown, score: 0.2
Wdr25Unknown, score: 0.4
Mapre2Unknown, score: 0.23
Fam149aUnknown, score: 0.23
Mms22lUnknown, score: 0.29
Klhl32Unknown, score: 0.35
LcorUnknown, score: 0.15
AA986860Unknown, score: 0.34
Mtg1Unknown, score: 0.15
Spice1Unknown, score: 0.35
Trmt1Unknown, score: 0.35
Sh3bgrl2Unknown, score: 0.42
Prpsap2Unknown, score: 0.17
N4bp3Unknown, score: 0.18
Chpt1Unknown, score: 0.19
Ddx46Unknown, score: 0.18
Rsph4aUnknown, score: 0.44
Pm20d1Unknown, score: 0.16
TifabUnknown, score: 0.58
Ifitm6Unknown, score: 0.2
Aox2Unknown, score: 0.18
Gabpb2Unknown, score: 0.35
Tmem71Unknown, score: 0.47
Il20rbUnknown, score: 0.22
Rnf26Unknown, score: 0.14
Fstl5Unknown, score: 0.74
Fbxl21Unknown, score: 0.22
Prdm9Unknown, score: 0.17
Mylk3Unknown, score: 0.78
Zcchc5Unknown, score: 0.18
Nudt18Unknown, score: 0.15
Szrd1Unknown, score: 0.26
Arhgef11Unknown, score: 0.17
Plekhg6Unknown, score: 0.43
Cracr2bUnknown, score: 0.46
Dync2li1Unknown, score: 0.14
9530068E07RikUnknown, score: 0.59
Tbl3Unknown, score: 0.2
Casd1Unknown, score: 0.53
Col28a1Unknown, score: 0.23
Ccdc186Unknown, score: 0.21
Sox30Unknown, score: 0.18
4933430I17RikUnknown, score: 0.14
Arhgap29Unknown, score: 0.2
Ago3Unknown, score: 0.19
Trim38Unknown, score: 0.23
Pak6Unknown, score: 0.24
Disp2Unknown, score: 0.31
Nudt15Unknown, score: 0.38
CrygnUnknown, score: 0.24
Hhipl1Unknown, score: 0.14
MyocdUnknown, score: 0.24
Parp16Unknown, score: 0.76
Cdk5rap2Unknown, score: 0.21
Tmprss4Unknown, score: 0.49
Tmprss13Unknown, score: 0.4
SheUnknown, score: 0.22
Spg11Unknown, score: 0.23
Duox2Unknown, score: 0.18
Sidt2Unknown, score: 0.23
Slc10a3Unknown, score: 0.3
Papd5Unknown, score: 0.17
Rcor3Unknown, score: 0.29
Zfp879Unknown, score: 0.46
Sertad4Unknown, score: 0.19
Neurl3Unknown, score: 0.28
Lman2lUnknown, score: 0.31
Csnk1g1Unknown, score: 0.29
Slc39a2Unknown, score: 0.26
Trim50Unknown, score: 0.61
Slc35f1Unknown, score: 0.22
Il1f9Unknown, score: 0.41
Brinp3Unknown, score: 0.4
Zbtb8bUnknown, score: 0.17
Psd4Unknown, score: 0.53
Rassf2Unknown, score: 0.27
Ccdc92Unknown, score: 0.17
AdgbUnknown, score: 0.16
Phactr2Unknown, score: 0.16
BC021785Unknown, score: 0.18
McuUnknown, score: 0.23
Lrrc20Unknown, score: 0.19
Lrrtm3Unknown, score: 0.19
Ctnna3Unknown, score: 0.49
Ube2d1Unknown, score: 0.26
Trappc10Unknown, score: 0.16
IlvblUnknown, score: 0.81
PolrmtUnknown, score: 0.28
Tmem259Unknown, score: 0.16
Rab21Unknown, score: 0.15
Zfc3h1Unknown, score: 0.21
Tspan8Unknown, score: 0.37
Rab3ipUnknown, score: 0.32
Os9Unknown, score: 0.22
LgalslUnknown, score: 0.49
Efemp1Unknown, score: 0.22
Hbq1aUnknown, score: 0.15
Rufy1Unknown, score: 0.77
Adamts2Unknown, score: 0.16
Acsl6Unknown, score: 0.34
Wnt9aUnknown, score: 0.3
Tom1l2Unknown, score: 0.26
Usp22Unknown, score: 0.2
Arhgap44Unknown, score: 0.22
CntrobUnknown, score: 0.58
Wrap53Unknown, score: 0.34
Nlgn2Unknown, score: 0.36
Slc16a11Unknown, score: 0.14
Wscd1Unknown, score: 0.37
Trp53i13Unknown, score: 0.29
Fam222bUnknown, score: 0.18
Xylt2Unknown, score: 0.18
Arl5cUnknown, score: 0.48
Rundc1Unknown, score: 0.27
Fam171a2Unknown, score: 0.35
Abca8aUnknown, score: 0.14
Cd300aUnknown, score: 0.62
Cd300lbUnknown, score: 0.33
Hid1Unknown, score: 0.23
UnkUnknown, score: 0.19
Tnrc6cUnknown, score: 0.2
EngaseUnknown, score: 0.16
Rab40bUnknown, score: 0.23
Trib2Unknown, score: 0.7
DgkbUnknown, score: 0.31
Stxbp6Unknown, score: 0.28
G2e3Unknown, score: 0.88
Gm527Unknown, score: 0.32
Mis18bp1Unknown, score: 0.2
Acot6Unknown, score: 0.31
Mlh3Unknown, score: 0.49
Nek9Unknown, score: 0.24
Unc79Unknown, score: 0.17
Cdc42bpbUnknown, score: 0.61
Cep170bUnknown, score: 0.21
Rapgef5Unknown, score: 0.2
Slc17a2Unknown, score: 0.25
MylipUnknown, score: 0.2
Cdc14bUnknown, score: 0.19
Zfp455Unknown, score: 0.34
RfesdUnknown, score: 0.36
Ttc37Unknown, score: 0.16
Rasa1Unknown, score: 0.27
Serinc5Unknown, score: 0.41
Lhfpl2Unknown, score: 0.2
Pde8bUnknown, score: 0.31
Btf3Unknown, score: 0.15
SgtbUnknown, score: 0.28
Depdc1bUnknown, score: 0.42
Dhx29Unknown, score: 0.35
CcnoUnknown, score: 0.36
Arl15Unknown, score: 0.16
Paip1Unknown, score: 0.27
PxkUnknown, score: 0.34
3830406C13RikUnknown, score: 0.5
Zfp503Unknown, score: 0.39
Polr3aUnknown, score: 0.23
Fam208aUnknown, score: 0.19
ChdhUnknown, score: 0.14
WapalUnknown, score: 0.14
Wdhd1Unknown, score: 0.22
Mapk1ip1lUnknown, score: 0.33
Tmem55bUnknown, score: 0.14
KhnynUnknown, score: 0.24
CenpjUnknown, score: 0.19
Zmym5Unknown, score: 0.16
Phf11aUnknown, score: 0.31
Shisa2Unknown, score: 0.22
Mtmr6Unknown, score: 0.63
Spata13Unknown, score: 0.23
Arl11Unknown, score: 0.48
Fam167aUnknown, score: 0.19
Hmbox1Unknown, score: 0.75
Pcdh17Unknown, score: 0.32
Tdrd3Unknown, score: 0.23
Pcdh20Unknown, score: 0.36
GgactUnknown, score: 0.84
Ugt3a2Unknown, score: 0.25
TrioUnknown, score: 0.39
Dcaf13Unknown, score: 0.15
Eny2Unknown, score: 0.14
E430025E21RikUnknown, score: 0.16
NaprtUnknown, score: 0.29
Sun2Unknown, score: 0.17
Ldoc1lUnknown, score: 0.21
Gramd4Unknown, score: 0.48
Brd1Unknown, score: 0.3
SeloUnknown, score: 0.19
Rapgef3Unknown, score: 0.14
Senp1Unknown, score: 0.26
Krt79Unknown, score: 0.18
Cpped1Unknown, score: 0.33
Marf1Unknown, score: 0.45
SpidrUnknown, score: 0.17
Slc7a4Unknown, score: 0.2
NrrosUnknown, score: 0.22
Ubxn7Unknown, score: 0.16
Dirc2Unknown, score: 0.29
Poglut1Unknown, score: 0.33
Dzip3Unknown, score: 0.35
Lemd2Unknown, score: 0.23
D17Wsu92eUnknown, score: 0.44
Adgrf5Unknown, score: 0.35
Ubr2Unknown, score: 0.23
Plcl2Unknown, score: 0.2
Crb3Unknown, score: 0.14
Dlgap1Unknown, score: 0.35
Srsf7Unknown, score: 0.19
Ttc7Unknown, score: 0.26
SvilUnknown, score: 0.15
WacUnknown, score: 0.2
Thoc1Unknown, score: 0.39
Mib1Unknown, score: 0.29
Ankrd29Unknown, score: 0.26
Rsl24d1Unknown, score: 0.38
Ino80cUnknown, score: 0.37
AW554918Unknown, score: 0.2
Pik3c3Unknown, score: 0.19
Ammecr1lUnknown, score: 0.17
Wdr36Unknown, score: 0.44
Fam13bUnknown, score: 0.4
Reep2Unknown, score: 0.29
Rbm27Unknown, score: 0.32
Pggt1bUnknown, score: 0.16
Prdm6Unknown, score: 0.28
Cep120Unknown, score: 0.34
Slc27a6Unknown, score: 0.16
Sh3tc2Unknown, score: 0.18
Onecut2Unknown, score: 0.42
Cep76Unknown, score: 0.64
Pla2g16Unknown, score: 0.32
Snx32Unknown, score: 0.21
Rin1Unknown, score: 0.29
Lrfn4Unknown, score: 0.29
DakUnknown, score: 0.15
D030056L22RikUnknown, score: 0.24
Trpm3Unknown, score: 0.73
Cbwd1Unknown, score: 0.26
Dmrt2Unknown, score: 0.32
Ric1Unknown, score: 0.15
Ermp1Unknown, score: 0.51
Hectd2Unknown, score: 0.63
Ablim1Unknown, score: 0.15
Fam160b1Unknown, score: 0.51
Eno4Unknown, score: 0.16
Rab3gap1Unknown, score: 0.19
DarsUnknown, score: 0.21
Yod1Unknown, score: 0.31
Dyrk3Unknown, score: 0.33
Zfp281Unknown, score: 0.52
Nmnat2Unknown, score: 0.28
Aph1aUnknown, score: 0.42
SucoUnknown, score: 0.21
Rcsd1Unknown, score: 0.23
Fam78bUnknown, score: 0.19
Ndufs2Unknown, score: 0.36
Tstd1Unknown, score: 0.31
CnstUnknown, score: 0.15
Cdc42bpaUnknown, score: 0.3
Ppp2r5aUnknown, score: 0.41
SbsponUnknown, score: 0.29
Actr1bUnknown, score: 0.16
Slc39a10Unknown, score: 0.15
HibchUnknown, score: 0.19
Ndufs1Unknown, score: 0.34
Cps1Unknown, score: 0.26
Fam134aUnknown, score: 0.23
Gpr55Unknown, score: 0.49
Rpp38Unknown, score: 0.32
CdnfUnknown, score: 0.41
Camk1dUnknown, score: 0.28
Man1b1Unknown, score: 0.24
BC029214Unknown, score: 0.21
Rexo4Unknown, score: 0.18
Sh3glb2Unknown, score: 0.18
Prrc2bUnknown, score: 0.36
Slc25a25Unknown, score: 0.2
1700019L03RikUnknown, score: 0.25
Dennd1aUnknown, score: 0.3
Epc2Unknown, score: 0.2
GcaUnknown, score: 0.32
Klhl41Unknown, score: 0.23
Atp5g3Unknown, score: 0.16
AgpsUnknown, score: 0.28
Kif18aUnknown, score: 0.55
Ano3Unknown, score: 0.48
Arhgap11aUnknown, score: 0.46
Bahd1Unknown, score: 0.39
Frmd5Unknown, score: 0.32
MallUnknown, score: 0.17
4930402H24RikUnknown, score: 0.19
SmoxUnknown, score: 0.16
6820408C15RikUnknown, score: 0.45
Asxl1Unknown, score: 0.21
PiguUnknown, score: 0.26
Slc35c2Unknown, score: 0.35
Zfp217Unknown, score: 0.15
Slc17a9Unknown, score: 0.19
QrfprUnknown, score: 0.25
Eif2aUnknown, score: 0.42
Gpr171Unknown, score: 0.27
D930015E06RikUnknown, score: 0.44
GatbUnknown, score: 0.21
Isg20l2Unknown, score: 0.31
Smg5Unknown, score: 0.18
Gatad2bUnknown, score: 0.17
Gm128Unknown, score: 0.14
PruneUnknown, score: 0.27
Adamtsl4Unknown, score: 0.17
CiartUnknown, score: 0.43
Trim45Unknown, score: 0.28
Vangl1Unknown, score: 0.18
Rsbn1Unknown, score: 0.17
Rbm15Unknown, score: 0.34
Gpr61Unknown, score: 0.24
Clcc1Unknown, score: 0.54
Slc25a24Unknown, score: 0.14
Trmt13Unknown, score: 0.38
Slc35a3Unknown, score: 0.21
Lppr4Unknown, score: 0.25
Tram1l1Unknown, score: 0.4
Ccbl2Unknown, score: 0.28
Coq3Unknown, score: 0.36
Fam214bUnknown, score: 0.19
IkbkapUnknown, score: 0.15
Frrs1lUnknown, score: 0.43
AI314180Unknown, score: 0.21
6330416G13RikUnknown, score: 0.48
Megf9Unknown, score: 0.27
Haus6Unknown, score: 0.3
FocadUnknown, score: 0.38
Cyb5rlUnknown, score: 0.26
Glis1Unknown, score: 0.42
Zfyve9Unknown, score: 0.22
Ttc39aUnknown, score: 0.17
Szt2Unknown, score: 0.23
Foxj3Unknown, score: 0.45
Eva1bUnknown, score: 0.21
Aim1lUnknown, score: 0.15
Pdik1lUnknown, score: 0.39
NcmapUnknown, score: 0.19
Zbtb40Unknown, score: 0.23
Ajap1Unknown, score: 0.21
Agbl5Unknown, score: 0.43
Zfyve28Unknown, score: 0.2
Sh3tc1Unknown, score: 0.26
Cytl1Unknown, score: 0.26
AF366264Unknown, score: 0.21
Cc2d2aUnknown, score: 0.28
Sel1l3Unknown, score: 0.45
Slc10a4Unknown, score: 0.48
AasdhUnknown, score: 0.53
Polr2bUnknown, score: 0.17
Sdad1Unknown, score: 0.44
Tmem150cUnknown, score: 0.39
Arhgap24Unknown, score: 0.38
Lrrc8dUnknown, score: 0.25
ChfrUnknown, score: 0.26
FicdUnknown, score: 0.64
Tmem119Unknown, score: 0.24
Ssh1Unknown, score: 0.33
Alkbh2Unknown, score: 0.3
MepceUnknown, score: 0.35
BC037034Unknown, score: 0.17
Ap5z1Unknown, score: 0.31
Lmtk2Unknown, score: 0.17
Gimap6Unknown, score: 0.16
Fam221aUnknown, score: 0.23
Jazf1Unknown, score: 0.37
Creb5Unknown, score: 0.27
Plekha8Unknown, score: 0.26
Eva1aUnknown, score: 0.5
C87436Unknown, score: 0.3
Fgd5Unknown, score: 0.6
Tmf1Unknown, score: 0.19
Frmd4bUnknown, score: 0.71
Gxylt2Unknown, score: 0.41
Zfp637Unknown, score: 0.39
Ankrd26Unknown, score: 0.46
A2mUnknown, score: 0.25
Clstn3Unknown, score: 0.25
BC035044Unknown, score: 0.27
Clec12aUnknown, score: 0.41
Crebl2Unknown, score: 0.26
Gys2Unknown, score: 0.58
Caprin2Unknown, score: 0.38
Amn1Unknown, score: 0.46
Zc3hc1Unknown, score: 0.43
MgamUnknown, score: 0.27
Suv420h2Unknown, score: 0.14
Zfp418Unknown, score: 0.19
Zfp772Unknown, score: 0.34
Zscan18Unknown, score: 0.19
Phldb3Unknown, score: 0.31
Zfp574Unknown, score: 0.17
Zfp420Unknown, score: 0.24
Zfp382Unknown, score: 0.29
Ffar1Unknown, score: 0.17
233147Unknown, score: 0.28
AI987944Unknown, score: 0.33
Ano5Unknown, score: 0.24
SiglechUnknown, score: 0.22
Tubgcp5Unknown, score: 0.19
Mtmr10Unknown, score: 0.23
Adamts17Unknown, score: 0.26
Vps33bUnknown, score: 0.57
Tmc3Unknown, score: 0.54
CrebzfUnknown, score: 0.36
Kctd14Unknown, score: 0.6
2210018M11RikUnknown, score: 0.48
Pgap2Unknown, score: 0.59
Twf1Unknown, score: 0.18
Plekha7Unknown, score: 0.19
Thumpd1Unknown, score: 0.61
Abca16Unknown, score: 0.26
Palb2Unknown, score: 0.18
Tnrc6aUnknown, score: 0.2
Gtf3c1Unknown, score: 0.39
Kctd13Unknown, score: 0.19
Prr14Unknown, score: 0.36
Gpr26Unknown, score: 0.16
Ppfia1Unknown, score: 0.17
Adprhl1Unknown, score: 0.22
Tmco3Unknown, score: 0.32
Arhgef10Unknown, score: 0.33
Fgl1Unknown, score: 0.31
HeltUnknown, score: 0.23
Cbr4Unknown, score: 0.16
234311Unknown, score: 0.23
Naf1Unknown, score: 0.44
Psd3Unknown, score: 0.18
Gatad2aUnknown, score: 0.25
Ushbp1Unknown, score: 0.2
Cib3Unknown, score: 0.27
Dync1li2Unknown, score: 0.19
Nae1Unknown, score: 0.15
D230025D16RikUnknown, score: 0.19
Ddx19bUnknown, score: 0.28
AtminUnknown, score: 0.34
Klhl36Unknown, score: 0.38
MthfsdUnknown, score: 0.15
BC021891Unknown, score: 0.23
Ccdc67Unknown, score: 0.37
Mbd3l2Unknown, score: 0.17
Sept7Unknown, score: 0.22
Foxred1Unknown, score: 0.19
Msantd2Unknown, score: 0.65
Tmem136Unknown, score: 0.4
C1qtnf5Unknown, score: 0.29
DlatUnknown, score: 0.31
Dmxl2Unknown, score: 0.52
HykkUnknown, score: 0.34
Snx33Unknown, score: 0.77
Coro2bUnknown, score: 0.17
Usp3Unknown, score: 0.65
Gtf2a2Unknown, score: 0.16
Fam63bUnknown, score: 0.34
Slc17a5Unknown, score: 0.39
Cd109Unknown, score: 0.21
Gk5Unknown, score: 0.2
Ppp2r3aUnknown, score: 0.29
Atp2c1Unknown, score: 0.14
Dusp7Unknown, score: 0.24
Acaa1bUnknown, score: 0.41
Zfp709Unknown, score: 0.48
Pyhin1Unknown, score: 0.47
5730507C01RikUnknown, score: 0.22
NyxUnknown, score: 0.49
Usp11Unknown, score: 0.17
Gpr119Unknown, score: 0.16
Mmgt1Unknown, score: 0.38
Slc9a6Unknown, score: 0.21
BC023829Unknown, score: 0.22
Pcyt1bUnknown, score: 0.54
Klhl15Unknown, score: 0.32
Stard8Unknown, score: 0.24
Ercc6lUnknown, score: 0.33
Tceal1Unknown, score: 0.37
Gnl3lUnknown, score: 0.36
Zc3h12dUnknown, score: 0.15
Aldh8a1Unknown, score: 0.25
L3mbtl3Unknown, score: 0.25
Ric8bUnknown, score: 0.65
Gas2l3Unknown, score: 0.22
Ccdc38Unknown, score: 0.25
Tmtc3Unknown, score: 0.19
Rassf9Unknown, score: 0.29
Stac3Unknown, score: 0.17
Gpr75Unknown, score: 0.26
Btnl9Unknown, score: 0.26
Mief2Unknown, score: 0.26
Slc13a5Unknown, score: 0.24
Tusc5Unknown, score: 0.39
Sarm1Unknown, score: 0.22
PhbUnknown, score: 0.4
Slfn9Unknown, score: 0.6
Usp32Unknown, score: 0.41
Gpatch8Unknown, score: 0.14
Dock4Unknown, score: 0.17
Akap6Unknown, score: 0.33
Rps6kl1Unknown, score: 0.39
Irf2bplUnknown, score: 0.14
Zdhhc22Unknown, score: 0.43
Slc24a4Unknown, score: 0.46
Serpinb6dUnknown, score: 0.38
Gpr150Unknown, score: 0.28
Ppwd1Unknown, score: 0.44
Pde4dUnknown, score: 0.2
OgdhlUnknown, score: 0.34
HomezUnknown, score: 0.42
Il17dUnknown, score: 0.16
Enox1Unknown, score: 0.14
Slitrk6Unknown, score: 0.4
Card6Unknown, score: 0.59
Adamts12Unknown, score: 0.37
Tspyl5Unknown, score: 0.31
Rspo2Unknown, score: 0.23
AardUnknown, score: 0.3
Fam83aUnknown, score: 0.36
Phf20l1Unknown, score: 0.23
Zfp647Unknown, score: 0.22
Cacna1iUnknown, score: 0.3
Ttc38Unknown, score: 0.22
239611Unknown, score: 0.47
Dip2bUnknown, score: 0.19
Ndufa11Unknown, score: 0.36
PigzUnknown, score: 0.2
Gpr156Unknown, score: 0.3
Pnldc1Unknown, score: 0.23
LnpepUnknown, score: 0.15
Neurl1bUnknown, score: 0.38
Zfp952Unknown, score: 0.16
Morc2bUnknown, score: 0.26
Cchcr1Unknown, score: 0.15
H2-M5Unknown, score: 0.2
Zfp119bUnknown, score: 0.19
Ccdc112Unknown, score: 0.22
Fem1cUnknown, score: 0.59
Dmxl1Unknown, score: 0.59
Adamts19Unknown, score: 0.24
F830016B08RikUnknown, score: 0.42
Pcyox1lUnknown, score: 0.15
Malt1Unknown, score: 0.4
Kcng2Unknown, score: 0.37
Cdc42bpgUnknown, score: 0.2
Kcnv2Unknown, score: 0.27
Slc16a12Unknown, score: 0.34
Kif20bUnknown, score: 0.16
Sec31bUnknown, score: 0.62
Dusp5Unknown, score: 0.16
Mcmdc2Unknown, score: 0.85
Sulf1Unknown, score: 0.59
Klhl12Unknown, score: 0.64
Brinp2Unknown, score: 0.15
Zbtb37Unknown, score: 0.38
Tnfsf18Unknown, score: 0.18
Scyl3Unknown, score: 0.26
Dusp27Unknown, score: 0.69
Lrrc52Unknown, score: 0.34
Itga8Unknown, score: 0.54
St8sia6Unknown, score: 0.38
Galnt5Unknown, score: 0.25
Xirp2Unknown, score: 0.61
Cers6Unknown, score: 0.47
Zfp804aUnknown, score: 0.29
Lrrc55Unknown, score: 0.15
Tspan18Unknown, score: 0.2
Lrrc4cUnknown, score: 0.18
Ldlrad3Unknown, score: 0.4
241621Unknown, score: 0.25
Wdr76Unknown, score: 0.52
Lzts3Unknown, score: 0.18
Tspyl3Unknown, score: 0.25
Ccdc144bUnknown, score: 0.35
D3Ertd254eUnknown, score: 0.39
Bbs12Unknown, score: 0.33
Frem2Unknown, score: 0.72
Igsf10Unknown, score: 0.29
Rxfp4Unknown, score: 0.16
Zfp697Unknown, score: 0.19
Slc22a15Unknown, score: 0.3
Slc44a5Unknown, score: 0.17
Fam110bUnknown, score: 0.15
Gdf6Unknown, score: 0.51
Atp6v0d2Unknown, score: 0.31
Rgp1Unknown, score: 0.22
Tmem8bUnknown, score: 0.19
Gabbr2Unknown, score: 0.42
Grin3aUnknown, score: 0.48
Frmd3Unknown, score: 0.53
Dmrta1Unknown, score: 0.27
Kank4Unknown, score: 0.15
Rims3Unknown, score: 0.14
Dlgap3Unknown, score: 0.35
Lrrc38Unknown, score: 0.22
Gnat3Unknown, score: 0.21
Rsbn1lUnknown, score: 0.53
Fbxl5Unknown, score: 0.64
Kctd8Unknown, score: 0.41
TecrlUnknown, score: 0.14
Sbno1Unknown, score: 0.53
Zfp775Unknown, score: 0.24
Grip2Unknown, score: 0.54
Kbtbd8Unknown, score: 0.41
Iqsec3Unknown, score: 0.38
FkrpUnknown, score: 0.18
GgnUnknown, score: 0.61
Zfp14Unknown, score: 0.19
Hspb6Unknown, score: 0.23
Rgs9bpUnknown, score: 0.32
Nars2Unknown, score: 0.23
Trim30bUnknown, score: 0.47
Tnfrsf26Unknown, score: 0.3
MrgpreUnknown, score: 0.14
Dlgap2Unknown, score: 0.22
Kat6aUnknown, score: 0.29
Lonrf1Unknown, score: 0.38
Wdr17Unknown, score: 0.22
Zfp791Unknown, score: 0.24
Tox3Unknown, score: 0.33
Rpgrip1lUnknown, score: 0.26
Pskh1Unknown, score: 0.19
Phlpp2Unknown, score: 0.17
HydinUnknown, score: 0.34
Sipa1l2Unknown, score: 0.38
Kdm4dUnknown, score: 0.54
Zfp317Unknown, score: 0.16
Zfp846Unknown, score: 0.19
Olfm2Unknown, score: 0.15
Glb1l2Unknown, score: 0.42
Ankk1Unknown, score: 0.69
Arhgap20Unknown, score: 0.22
Zc3h12cUnknown, score: 0.38
ScaperUnknown, score: 0.17
Klhl31Unknown, score: 0.3
Dclk3Unknown, score: 0.26
MyripUnknown, score: 0.41
Fam198aUnknown, score: 0.34
RetnlgUnknown, score: 0.32
9930111J21Rik2Unknown, score: 0.17
Dcaf12l1Unknown, score: 0.5
Dkc1Unknown, score: 0.37
Zc4h2Unknown, score: 0.44
Gm614Unknown, score: 0.22
C77370Unknown, score: 0.43
Zfp711Unknown, score: 0.19
Kir3dl1Unknown, score: 0.41
Fam199xUnknown, score: 0.44
Mum1l1Unknown, score: 0.51
Amdhd2Unknown, score: 0.36
Atg9aUnknown, score: 0.22
Fam71f2Unknown, score: 0.19
Ccdc15Unknown, score: 0.27
Vps54Unknown, score: 0.23
RttnUnknown, score: 0.41
Myo1gUnknown, score: 0.32
BivmUnknown, score: 0.49
Prokr2Unknown, score: 0.35
Rhobtb2Unknown, score: 0.21
Oas3Unknown, score: 0.16
Oas1hUnknown, score: 0.3
Dnajc28Unknown, score: 0.2
Obox6Unknown, score: 0.51
Usp7Unknown, score: 0.28
Ropn1lUnknown, score: 0.37
Tpcn1Unknown, score: 0.46
Olfr543Unknown, score: 0.22
Olfr1034Unknown, score: 0.28
Olfr1384Unknown, score: 0.28
Olfr691Unknown, score: 0.45
Olfr520Unknown, score: 0.25
Olfr550Unknown, score: 0.17
Olfr559Unknown, score: 0.2
Ehd2Unknown, score: 0.44
Cadm4Unknown, score: 0.31
Gga3Unknown, score: 0.32
Nphp4Unknown, score: 0.28
Cdc42ep3Unknown, score: 0.3
Plekhg3Unknown, score: 0.49
Spata2Unknown, score: 0.22
Cdk8Unknown, score: 0.49
Ly6g5bUnknown, score: 0.36
AcmsdUnknown, score: 0.22
Rps15aUnknown, score: 0.33
Zbtb24Unknown, score: 0.46
Scml4Unknown, score: 0.25
Kcnc2Unknown, score: 0.23
MpgUnknown, score: 0.44
Alkbh5Unknown, score: 0.25
Rab11fip4Unknown, score: 0.14
Bahcc1Unknown, score: 0.23
Sntg2Unknown, score: 0.38
GphnUnknown, score: 0.17
Zfp759Unknown, score: 0.33
Ccnb1Unknown, score: 0.23
Slc38a9Unknown, score: 0.16
Zswim8Unknown, score: 0.29
Rnf31Unknown, score: 0.23
Agxt2Unknown, score: 0.15
Adck5Unknown, score: 0.2
Xxylt1Unknown, score: 0.32
Robo2Unknown, score: 0.18
Arhgap28Unknown, score: 0.25
Ltbp1Unknown, score: 0.24
Gpr152Unknown, score: 0.18
Cpsf7Unknown, score: 0.5
Nup54Unknown, score: 0.53
Kif26bUnknown, score: 0.36
Inpp4aUnknown, score: 0.19
Nbeal1Unknown, score: 0.27
PaskUnknown, score: 0.21
Rtn4rl2Unknown, score: 0.43
Muc15Unknown, score: 0.15
3110057O12RikUnknown, score: 0.22
Plch1Unknown, score: 0.3
Lrig2Unknown, score: 0.16
VcpUnknown, score: 0.25
Tex10Unknown, score: 0.33
Zscan20Unknown, score: 0.25
Gpr157Unknown, score: 0.26
Pank4Unknown, score: 0.35
Ppp2r2cUnknown, score: 0.22
Gm15800Unknown, score: 0.23
Zfp664Unknown, score: 0.14
Clip2Unknown, score: 0.18
Lhfpl4Unknown, score: 0.28
Zfp384Unknown, score: 0.56
Pon3Unknown, score: 0.64
Tspan12Unknown, score: 0.34
Nat14Unknown, score: 0.65
Vmn2r57Unknown, score: 0.39
Ttll13Unknown, score: 0.14
Rccd1Unknown, score: 0.43
Adamtsl3Unknown, score: 0.22
Nup98Unknown, score: 0.17
GcdhUnknown, score: 0.35
Irf2bp2Unknown, score: 0.3
Nlrx1Unknown, score: 0.29
Rab39Unknown, score: 0.26
Klhl18Unknown, score: 0.24
Zfp651Unknown, score: 0.17
Spin4Unknown, score: 0.19
Taf1Unknown, score: 0.36
Mbtps2Unknown, score: 0.46
Map3k15Unknown, score: 0.17
BC048403Unknown, score: 0.23
Tmem132eUnknown, score: 0.19
Ankdd1bUnknown, score: 0.24
4933408B17RikUnknown, score: 0.31
Adcy10Unknown, score: 0.18
Rpusd2Unknown, score: 0.2
C2cd4dUnknown, score: 0.26
Tstd2Unknown, score: 0.25
B3gnt6Unknown, score: 0.3
Acsm5Unknown, score: 0.24
Tango6Unknown, score: 0.15
Eif5aUnknown, score: 0.35
Gemin4Unknown, score: 0.26
Ccdc42Unknown, score: 0.18
Slfn8Unknown, score: 0.22
Rasl10bUnknown, score: 0.25
Marveld1Unknown, score: 0.69
277089Unknown, score: 0.16
NynrinUnknown, score: 0.35
Prex1Unknown, score: 0.39
Vstm2lUnknown, score: 0.3
Fam131cUnknown, score: 0.34
Cyp4a12aUnknown, score: 0.16
Depdc5Unknown, score: 0.16
Exoc3lUnknown, score: 0.3
Armcx6Unknown, score: 0.2
Wfikkn2Unknown, score: 0.79
Lrrc10bUnknown, score: 0.32
Kctd19Unknown, score: 0.4
Tlr13Unknown, score: 0.3
Pcdh19Unknown, score: 0.31
Lix1lUnknown, score: 0.15
SbsnUnknown, score: 0.27
Slc24a5Unknown, score: 0.15
Gimap5Unknown, score: 0.33
Gimap9Unknown, score: 0.3
Hist1h3cUnknown, score: 0.3
Hist1h3dUnknown, score: 0.3
Hist1h3bUnknown, score: 0.3
Hist1h3eUnknown, score: 0.21
Hist1h3hUnknown, score: 0.21
Hist1h3iUnknown, score: 0.3
Hist3h2aUnknown, score: 0.17
Hist1h2acUnknown, score: 0.17
Hist1h2adUnknown, score: 0.17
Hist1h2aeUnknown, score: 0.17
Hist1h2agUnknown, score: 0.17
Hist1h2ahUnknown, score: 0.17
Hist1h2anUnknown, score: 0.17
Hist1h2apUnknown, score: 0.17
Hist2h2beUnknown, score: 0.17
Gpr4Unknown, score: 0.4
SctrUnknown, score: 0.34
Fchsd1Unknown, score: 0.2
A230050P20RikUnknown, score: 0.67
Ubald2Unknown, score: 0.31
C5ar2Unknown, score: 0.62
Dpep2Unknown, score: 0.52
Pld5Unknown, score: 0.25
Lrtm1Unknown, score: 0.55
6030419C18RikUnknown, score: 0.3
Cxxc4Unknown, score: 0.32
Wdr59Unknown, score: 0.31
A430078G23RikUnknown, score: 0.32
Dusp4Unknown, score: 0.17
Zfp750Unknown, score: 0.15
Zfp182Unknown, score: 0.21
Syne2Unknown, score: 0.17
6430573F11RikUnknown, score: 0.37
Lig4Unknown, score: 0.2
Zfp653Unknown, score: 0.25
Zfp944Unknown, score: 0.29
Efcab5Unknown, score: 0.29
Nt5dc1Unknown, score: 0.17
Usp37Unknown, score: 0.45
Podxl2Unknown, score: 0.35
Eml5Unknown, score: 0.22
Zfyve27Unknown, score: 0.26
SmoUnknown, score: 0.48
Rfx7Unknown, score: 0.24
9630033F20RikUnknown, score: 0.33
Cobll1Unknown, score: 0.21
Dock6Unknown, score: 0.65
Ism1Unknown, score: 0.14
Vwc2Unknown, score: 0.31
A530016L24RikUnknown, score: 0.2
Flad1Unknown, score: 0.19
Ttll1Unknown, score: 0.25
Kif6Unknown, score: 0.22
Ccdc79Unknown, score: 0.23
Nceh1Unknown, score: 0.81
Exph5Unknown, score: 0.49
6430550D23RikUnknown, score: 0.23
ReltUnknown, score: 0.21
Slc38a11Unknown, score: 0.3
Prr18Unknown, score: 0.2
Fndc9Unknown, score: 0.34
DgkiUnknown, score: 0.21
Adrbk2Unknown, score: 0.16
BC049715Unknown, score: 0.25
Ptpn7Unknown, score: 0.26
Zmat4Unknown, score: 0.52
Tacc1Unknown, score: 0.27
Fndc7Unknown, score: 0.31
Lrrc58Unknown, score: 0.33
Hook3Unknown, score: 0.59
Mettl20Unknown, score: 0.35
Tmem91Unknown, score: 0.15
Ddx11Unknown, score: 0.34
Ccdc171Unknown, score: 0.34
Ccdc66Unknown, score: 0.33
Ttll5Unknown, score: 0.24
Fubp3Unknown, score: 0.46
ScaiUnknown, score: 0.3
Glt28d2Unknown, score: 0.27
Tmem251Unknown, score: 0.56
FryUnknown, score: 0.47
CenptUnknown, score: 0.45
Trank1Unknown, score: 0.22
Alg6Unknown, score: 0.65
P4ha3Unknown, score: 0.63
Ppm1eUnknown, score: 0.38
Ipcef1Unknown, score: 0.31
Lmod3Unknown, score: 0.36
Lmbrd2Unknown, score: 0.27
Cachd1Unknown, score: 0.21
Sycp2Unknown, score: 0.55
Dennd5bUnknown, score: 0.62
Idi2Unknown, score: 0.26
Tmem88bUnknown, score: 0.33
Phf8Unknown, score: 0.24
Strip2Unknown, score: 0.22
Snrnp200Unknown, score: 0.45
Zbtb26Unknown, score: 0.37
D5Ertd579eUnknown, score: 0.62
Casc1Unknown, score: 0.2
DctdUnknown, score: 0.17
Ccdc158Unknown, score: 0.26
Tmem117Unknown, score: 0.56
Mdga2Unknown, score: 0.27
Tmem154Unknown, score: 0.19
Zhx3Unknown, score: 0.26
Cdh10Unknown, score: 0.48
Mical2Unknown, score: 0.48
Wscd2Unknown, score: 0.52
Ccbe1Unknown, score: 0.17
Tnpo3Unknown, score: 0.25
Arl4cUnknown, score: 0.28
Xpnpep3Unknown, score: 0.31
Serac1Unknown, score: 0.24
Gpr183Unknown, score: 0.58
Cdv3Unknown, score: 0.45
Tnfsf15Unknown, score: 0.31
Mettl24Unknown, score: 0.15
Tmem26Unknown, score: 0.14
Ppfia2Unknown, score: 0.27
PiglUnknown, score: 0.16
Dtd2Unknown, score: 0.34
Fam179bUnknown, score: 0.39
Trmt61aUnknown, score: 0.24
Apol11bUnknown, score: 0.22
Vwa5b2Unknown, score: 0.15
9830107B12RikUnknown, score: 0.31
MccUnknown, score: 0.19
Arhgef37Unknown, score: 0.33
Zfp532Unknown, score: 0.22
Zfp236Unknown, score: 0.15
Ankrd44Unknown, score: 0.4
Rbm44Unknown, score: 0.17
Dennd1bUnknown, score: 0.15
TnnUnknown, score: 0.15
Ptrh1Unknown, score: 0.31
Zfp335Unknown, score: 0.27
Aknad1Unknown, score: 0.52
PigkUnknown, score: 0.34
Tmem67Unknown, score: 0.2
Fam166bUnknown, score: 0.14
Frem1Unknown, score: 0.27
Foxo6Unknown, score: 0.42
Catsper4Unknown, score: 0.28
Slc5a6Unknown, score: 0.23
Adamts3Unknown, score: 0.36
Cxcl3Unknown, score: 0.26
Kctd10Unknown, score: 0.31
Gal3st4Unknown, score: 0.52
Sdk1Unknown, score: 0.5
Pon2Unknown, score: 0.37
Thsd7aUnknown, score: 0.29
Gcfc2Unknown, score: 0.18
Tmcc1Unknown, score: 0.2
Btbd16Unknown, score: 0.23
Ctxn1Unknown, score: 0.49
Zfp866Unknown, score: 0.4
DhpsUnknown, score: 0.2
Slc7a6Unknown, score: 0.33
OpcmlUnknown, score: 0.18
Dixdc1Unknown, score: 0.74
Snapc5Unknown, score: 0.2
331374Unknown, score: 0.17
Rgag4Unknown, score: 0.52
XkrxUnknown, score: 0.21
Tfap2eUnknown, score: 0.48
Ppp1r13lUnknown, score: 0.28
Cd59bUnknown, score: 0.24
Ttc16Unknown, score: 0.34
Zfp780bUnknown, score: 0.22
Supv3l1Unknown, score: 0.38
Trim65Unknown, score: 0.58
Myo1dUnknown, score: 0.21
Tmem220Unknown, score: 0.29
Fa2hUnknown, score: 0.3
Agap1Unknown, score: 0.2
Zfp931Unknown, score: 0.24
Prune2Unknown, score: 0.2
Pcdha2Unknown, score: 0.2
Pcdhac1Unknown, score: 0.2
Ltv1Unknown, score: 0.2
Sfmbt2Unknown, score: 0.35
Rtl1Unknown, score: 0.28
353328Unknown, score: 0.4
Myo18aUnknown, score: 0.21
Hist1h3aUnknown, score: 0.27
Zranb1Unknown, score: 0.3
Speer4dUnknown, score: 0.42
Pram1Unknown, score: 0.22
Lrrc24Unknown, score: 0.32
IntuUnknown, score: 0.37
NefhUnknown, score: 0.43
Slc47a2Unknown, score: 0.19
Spata22Unknown, score: 0.16
Serpina11Unknown, score: 0.34
Mrs2Unknown, score: 0.76
Serpinb1cUnknown, score: 0.15
Lyrm4Unknown, score: 0.4
Stmnd1Unknown, score: 0.18
Zfp395Unknown, score: 0.34
Lrch1Unknown, score: 0.28
Alg10bUnknown, score: 0.29
Tmem106cUnknown, score: 0.22
ErmardUnknown, score: 0.3
Zfp948Unknown, score: 0.38
Dnph1Unknown, score: 0.14
Gm21981Unknown, score: 0.44
Prob1Unknown, score: 0.36
Greb1lUnknown, score: 0.61
Naaladl1Unknown, score: 0.25
Fam189a2Unknown, score: 0.28
Lipo1Unknown, score: 0.4
Atp2b4Unknown, score: 0.34
Kif14Unknown, score: 0.45
Rc3h1Unknown, score: 0.22
BC055324Unknown, score: 0.28
2810408M09RikUnknown, score: 0.17
Zfp408Unknown, score: 0.55
Gm14085Unknown, score: 0.15
Trim55Unknown, score: 0.21
S100a7aUnknown, score: 0.24
Ccdc24Unknown, score: 0.42
Tbc1d2Unknown, score: 0.21
Rbm33Unknown, score: 0.44
Zcwpw1Unknown, score: 0.67
Wdr95Unknown, score: 0.2
N4bp2l2Unknown, score: 0.24
4930590J08RikUnknown, score: 0.22
Cracr2aUnknown, score: 0.22
2700089E24RikUnknown, score: 0.34
ItgadUnknown, score: 0.23
6430531B16RikUnknown, score: 0.3
Zfp882Unknown, score: 0.17
Foxr1Unknown, score: 0.27
Odf3l1Unknown, score: 0.19
Snx22Unknown, score: 0.57
Cep162Unknown, score: 0.24
Susd5Unknown, score: 0.21
Zkscan7Unknown, score: 0.26
Fdxacb1Unknown, score: 0.15
8030474K03RikUnknown, score: 0.2
Brwd3Unknown, score: 0.39
Tmem29Unknown, score: 0.33
A830080D01RikUnknown, score: 0.32
Atxn7l3bUnknown, score: 0.29
382522Unknown, score: 0.39
Pfn4Unknown, score: 0.32
Tmed8Unknown, score: 0.16
Rrm2bUnknown, score: 0.31
Ankrd63Unknown, score: 0.52
Tlr12Unknown, score: 0.16
Slc25a34Unknown, score: 0.29
Fam47eUnknown, score: 0.28
Trim56Unknown, score: 0.16
Cyp2t4Unknown, score: 0.18
Irs2Unknown, score: 0.5
Frmd7Unknown, score: 0.18
Rnf39Unknown, score: 0.21
Slitrk3Unknown, score: 0.2
Mettl7a2Unknown, score: 0.25
Flrt1Unknown, score: 0.21
Map4k5Unknown, score: 0.41
Scn4bUnknown, score: 0.52
Btbd6Unknown, score: 0.34
Plcxd1Unknown, score: 0.46
H2afy2Unknown, score: 0.47
Bex4Unknown, score: 0.2
Ndufa4l2Unknown, score: 0.26
Ecm2Unknown, score: 0.23
Tmem204Unknown, score: 0.63
GnptabUnknown, score: 0.21
Adcy1Unknown, score: 0.29
Gm5431Unknown, score: 0.41
432798Unknown, score: 0.36
Rpl30Unknown, score: 0.27
Ly6g6fUnknown, score: 0.19
Eno1bUnknown, score: 0.41
I830077J02RikUnknown, score: 0.36
Ankrd13cUnknown, score: 0.18
Minos1Unknown, score: 0.26
Gm13251Unknown, score: 0.28
433874Unknown, score: 0.33
Ociad2Unknown, score: 0.43
Eid2bUnknown, score: 0.33
WhammUnknown, score: 0.35
Trim72Unknown, score: 0.18
AmtUnknown, score: 0.42
434624Unknown, score: 0.22
Rhox8Unknown, score: 0.18
Ccdc160Unknown, score: 0.38
434782Unknown, score: 0.17
Adgrf2Unknown, score: 0.17
Rufy4Unknown, score: 0.26
A830005F24RikUnknown, score: 0.27
Zbtb9Unknown, score: 0.27
Armcx5Unknown, score: 0.34
Apcdd1Unknown, score: 0.23
544944Unknown, score: 0.35
Iqgap2Unknown, score: 0.42
Wdfy4Unknown, score: 0.31
Fam186bUnknown, score: 0.36
Cyp2c67Unknown, score: 0.22
Hmcn1Unknown, score: 0.18
BC094916Unknown, score: 0.39
545487Unknown, score: 0.39
Ptpn3Unknown, score: 0.17
Gm13290Unknown, score: 0.17
Gm13289Unknown, score: 0.17
Gm13277Unknown, score: 0.17
Gm13276Unknown, score: 0.17
Gm13279Unknown, score: 0.24
Mterf1aUnknown, score: 0.2
Gramd2Unknown, score: 0.39
Zc3h12bUnknown, score: 0.17
Cxx1bUnknown, score: 0.31
Serinc4Unknown, score: 0.17
Ndufs5Unknown, score: 0.2
Gm6086Unknown, score: 0.26
619973Unknown, score: 0.17
Gm12185Unknown, score: 0.35
Kctd21Unknown, score: 0.14
Ccdc107Unknown, score: 0.21
Zfp827Unknown, score: 0.37
623286Unknown, score: 0.32
Gm6484Unknown, score: 0.16
Lekr1Unknown, score: 0.37
Rpl30Unknown, score: 0.23
Zfp951Unknown, score: 0.32
Gm14322Unknown, score: 0.32
Klk13Unknown, score: 0.52
Gm11992Unknown, score: 0.24
628061Unknown, score: 0.18
Lipo4Unknown, score: 0.43
Gm6904Unknown, score: 0.3
Phf11cUnknown, score: 0.14
OtoglUnknown, score: 0.48
Serpina3iUnknown, score: 0.26
Ctxn3Unknown, score: 0.34
Cd101Unknown, score: 0.32
631286Unknown, score: 0.26
Erich4Unknown, score: 0.34
RerglUnknown, score: 0.18
634555Unknown, score: 0.31
635087Unknown, score: 0.18
635470Unknown, score: 0.15
635999Unknown, score: 0.3
636306Unknown, score: 0.3
636901Unknown, score: 0.21
640370Unknown, score: 0.38
Zfp784Unknown, score: 0.33
Gcnt7Unknown, score: 0.23
CtcflUnknown, score: 0.15
Gm7356Unknown, score: 0.15
Rpl30Unknown, score: 0.15
Isoc2aUnknown, score: 0.47
Gm14326Unknown, score: 0.31
TnikUnknown, score: 0.34
Sec14l5Unknown, score: 0.15
665181Unknown, score: 0.47
Plb1Unknown, score: 0.16
CsprsUnknown, score: 0.17
665611Unknown, score: 0.26
666448Unknown, score: 0.66
Gm8369Unknown, score: 0.48
667035Unknown, score: 0.25
667739Unknown, score: 0.43
Piezo2Unknown, score: 0.31
Myh15Unknown, score: 0.48
667952Unknown, score: 0.41
Gm8909Unknown, score: 0.33
Mettl7a3Unknown, score: 0.17
671641Unknown, score: 0.33
674846Unknown, score: 0.18
675851Unknown, score: 0.29
677654Unknown, score: 0.17
Tmsb15b2Unknown, score: 0.17
Gm11744Unknown, score: 0.32
Cep85lUnknown, score: 0.19
Isg15Unknown, score: 0.56
Gm14548Unknown, score: 0.19
Mup9Unknown, score: 0.3
Mup10Unknown, score: 0.3
Hrct1Unknown, score: 0.42
100039794Unknown, score: 0.4
Ildr2Unknown, score: 0.26
Gm12942Unknown, score: 0.27
100040052Unknown, score: 0.16
100040500Unknown, score: 0.2
Dynlt1fUnknown, score: 0.2
G630090E17RikUnknown, score: 0.15
100041098Unknown, score: 0.35
Gm15448Unknown, score: 0.42
Gm3194Unknown, score: 0.37
Supt4aUnknown, score: 0.24
Zkscan16Unknown, score: 0.24
Gm3558Unknown, score: 0.16
Nrg2Unknown, score: 0.24
Pdcd5Unknown, score: 0.65
Eif3j2Unknown, score: 0.35
100043217Unknown, score: 0.3
Zbed4Unknown, score: 0.17
Gm14308Unknown, score: 0.33
Gng5Unknown, score: 0.14
Zfp831Unknown, score: 0.25
Gm4724Unknown, score: 0.29
100044729Unknown, score: 0.2
100045901Unknown, score: 0.27
100046628Unknown, score: 0.16
100047468Unknown, score: 0.19
100047518Unknown, score: 0.3
100047632Unknown, score: 0.61
100047658Unknown, score: 0.35
100048117Unknown, score: 0.14
100048119Unknown, score: 0.16
100048410Unknown, score: 0.38
Cfap43Unknown, score: 0.88
Ddx43Unknown, score: 0.52
Mup19Unknown, score: 0.23
Fam150bUnknown, score: 0.36
Gm17455Unknown, score: 0.41
Gm14431Unknown, score: 0.2
Il4i1Unknown, score: 0.22
1700021K19RikUnknown, score: 0.14
Ccdc13Unknown, score: 0.69
Pdzd7Unknown, score: 0.25
100503180Unknown, score: 0.29
100503235Unknown, score: 0.31
100503254Unknown, score: 0.34
100503428Unknown, score: 0.16
CsprsUnknown, score: 0.25
Ccdc149Unknown, score: 0.39
100503895Unknown, score: 0.16
CsprsUnknown, score: 0.23
Cmtm1Unknown, score: 0.35
100504174Unknown, score: 0.23
Ccdc170Unknown, score: 0.35
TmppeUnknown, score: 0.67
Schip1Unknown, score: 0.3
100862012Unknown, score: 0.65
Hmgb1Unknown, score: 0.21
RP23-56M18.5Unknown, score: 0.3
Entpd4Unknown, score: 0.21
Gm21698Unknown, score: 0.44
Gm5616Unknown, score: 0.35
100862473Unknown, score: 0.23
100862531Unknown, score: 0.25
100862558Unknown, score: 0.35
100862570Unknown, score: 0.35
100862578Unknown, score: 0.29
100862584Unknown, score: 0.38
Sytl3Unknown, score: 0.15
101055820Unknown, score: 0.37
101056061Unknown, score: 0.24
101056348Unknown, score: 0.19
101056386Unknown, score: 0.27
101056482Unknown, score: 0.32
101056577Unknown, score: 0.22
101056584Unknown, score: 0.33
101056616Unknown, score: 0.34
101056690Unknown, score: 0.15

## Help | Hide | Top Help | Show | Top Conditions

### HELP

Conditions in the module, given in the same order as on the expression
plot above. Red color means over-expression, green under-expression in
the given condition.

The barplot below shows the condition (sample) scores. A separate bar
is shown for each sample, its height is the corresponding score of the
sample in the module. The red and green numbers on the bars are the
sample scores expressed in percents, i.e. 100% is 1.0.

The red and green lines show the module thresholds, samples above
the red line and below the green line are included in the module.

The different experiments that were part of the study, are separated
by dashed vertical lines.

— Click on the *Help* button again to close this help window.

| Id |
| --- |
| NOD\_ShiLtJ-ATE\_23 |
| SWR\_J-ATE\_135 |
| SJL\_J-ATE\_18 |
| A\_J-ATE\_117 |
| FVB\_NJ-ATE\_60 |
| LP\_J-ATE\_98 |
| LP\_J-ATE\_105 |
| SWR\_J-CTR\_138 |
| NOD\_ShiLtJ-ATE\_27 |
| A\_J-ATE\_116 |
| FVB\_NJ-ATE\_57 |
| A\_J-ATE\_122 |
| SWR\_J-ATE\_140 |
| NZB\_BLNJ-ATE\_94 |
| NOD\_ShiLtJ-ATE\_22 |
| PL\_J-ATE\_76 |
| SJL\_J-CTR\_15 |
| A\_J-CTR\_120 |
| SJL\_J-ATE\_17 |
| C57BL\_6J-ATE\_109 |
| LP\_J-CTR\_97 |
| FVB\_NJ-CTR\_51 |
| NZB\_BLNJ-ATE\_89 |
| NOD\_ShiLtJ-CTR\_21 |
| PL\_J-ATE\_75 |
| NZB\_BLNJ-CTR\_92 |
| C58\_J-ATE\_29 |
| FVB\_NJ-ATE\_53 |
| FVB\_NJ-CTR\_52 |
| A\_J-CTR\_119 |
| LP\_J-ATE\_101 |
| PL\_J-CTR\_74 |
| PL\_J-ATE\_71 |
| BALB\_cByJ-ATE\_157 |
| NOD\_ShiLtJ-CTR\_19 |
| LP\_J-CTR\_104 |
| PL\_J-CTR\_70 |
| C58\_J-ATE\_30 |
| SJL\_J-CTR\_16 |
| SWR\_J-ATE\_139 |
| C57BLKS\_J-ATE\_112 |
| BALB\_cByJ-ATE\_160 |
| C57BL\_6J-CTR\_39 |
| FVB\_NJ-CTR\_56 |
| SWR\_J-CTR\_137 |
| PL\_J-CTR\_72 |
| C57BL\_6J-CTR\_38 |
| SJL\_J-ATE\_11 |
| NZB\_BLNJ-ATE\_88 |
| C57BL\_6J-CTR\_35 |
| SWR\_J-CTR\_134 |
| LP\_J-CTR\_100 |
| NOD\_ShiLtJ-CTR\_20 |
| SJL\_J-CTR\_10 |
| C57BLKS\_J-CTR\_107 |
| C58\_J-ATE\_36 |
| C57BLKS\_J-ATE\_108 |
| C58\_J-CTR\_32 |
| C57BLKS\_J-ATE\_113 |
| NZB\_BLNJ-CTR\_93 |
| BALB\_cByJ-ATE\_151 |
| C57BLKS\_J-CTR\_110 |
| C57BL\_6J-ATE\_95 |
| BALB\_cByJ-CTR\_158 |
| A\_J-CTR\_121 |
| C58\_J-CTR\_34 |
| C58\_J-CTR\_28 |
| C3H\_HeJ-ATE\_61 |
| NZB\_BLNJ-CTR\_87 |
| C3H\_HeJ-ATE\_67 |
| C57BLKS\_J-CTR\_111 |
| I\_LnJ-CTR\_150 |
| BALB\_cJ-ATE\_80 |
| SM\_J-CTR\_48 |
| DBA\_2J-ATE\_6 |
| SM\_J-ATE\_49 |
| C3H\_HeJ-ATE\_66 |
| SM\_J-CTR\_40 |
| C3H\_HeJ-CTR\_65 |
| SM\_J-ATE\_43 |
| BALB\_cByJ-CTR\_143 |
| CBA\_J-CTR\_126 |
| CBA\_J-CTR\_130 |
| C57BL\_6J-ISO\_47 |
| SM\_J-ATE\_42 |
| LP\_J-ISO\_102 |
| DBA\_2J-ATE\_5 |
| SJL\_J-ISO\_14 |
| CBA\_J-CTR\_125 |
| PL\_J-ISO\_78 |
| SJL\_J-ISO\_13 |
| LP\_J-ISO\_103 |
| SWR\_J-ISO\_136 |
| PL\_J-ISO\_73 |
| SJL\_J-ISO\_12 |
| PL\_J-ISO\_77 |
| DBA\_2J-CTR\_2 |
| C57BL\_6J-ISO\_59 |
| NZB\_BLNJ-ISO\_90 |
| A\_J-ISO\_118 |
| A\_J-ISO\_123 |
| FVB\_NJ-ISO\_58 |
| NZB\_BLNJ-ISO\_91 |
| NZB\_BLNJ-ISO\_96 |
| FVB\_NJ-ISO\_55 |
| FVB\_NJ-ISO\_54 |
| SWR\_J-ISO\_142 |
| C57BL\_6J-ISO\_50 |
| A\_J-ISO\_124 |
| C58\_J-ISO\_31 |
| BALB\_cJ-ATE\_86 |
| DBA\_2J-CTR\_9 |
| C57BLKS\_J-ISO\_114 |
| C58\_J-ISO\_37 |
| BALB\_cJ-CTR\_85 |
| SWR\_J-ISO\_141 |
| BALB\_cByJ-ISO\_145 |
| DBA\_2J-CTR\_1 |
| BALB\_cJ-ATE\_82 |
| I\_LnJ-ISO\_155 |
| NOD\_ShiLtJ-ISO\_26 |
| BALB\_cByJ-CTR\_144 |
| C57BLKS\_J-ISO\_115 |
| C58\_J-ISO\_33 |
| BALB\_cByJ-ISO\_159 |
| NOD\_ShiLtJ-ISO\_24 |
| I\_LnJ-ISO\_152 |
| SM\_J-ISO\_44 |
| BALB\_cByJ-ISO\_156 |
| CBA\_J-ISO\_129 |
| C3H\_HeJ-ISO\_68 |
| NOD\_ShiLtJ-ISO\_25 |
| C3H\_HeJ-ISO\_69 |
| CBA\_J-ISO\_133 |
| SM\_J-ISO\_46 |
| SM\_J-ISO\_45 |
| I\_LnJ-ISO\_149 |
| CBA\_J-ISO\_128 |
| DBA\_2J-ISO\_4 |
| BALB\_cJ-ISO\_81 |
| DBA\_2J-ISO\_7 |
| C3H\_HeJ-ISO\_62 |
| BALB\_cJ-ISO\_84 |
| BALB\_cJ-ISO\_83 |
| DBA\_2J-ISO\_8 |

© 2015 Computational Biology Group, Department of Medical Genetics,
University of Lausanne, Switzerland
