# Supplementary material for: RNAseq analysis of heart tissue from mice treated with atenolol and isoproterenol reveals a reciprocal transcriptional response
Source: BMC Genomics. 2016 Sep 7;17(1):717. doi: 10.1186/s12864-016-3059-6 (PMC5015234; doi:10.1186/s12864-016-3059-6)
Supplement: Additional file 4: — Supplementary data. Compressed HTML files of 98 expression modules annotated for genes, strains and GO or KEGG terms (see Additional file 3 for navigation details). (GZ 11006 kb) [file 12864_2016_3059_MOESM4_ESM.gz › modules.html/module-95.html]

Module #95, TG: 0, TC: 0, 7837 probes, 7837 Entrez genes, 160 conditions

# Previous module | Next module Module #95, TG: 0, TC: 0, 7837 probes, 7837 Entrez genes, 160 conditions

- Module tree/table

- Expression data

- The BP GO tree
- The CC GO tree
- The MF GO tree

- GO BP enrichment
- GO CC enrichment
- GO MF enrichment
- KEGG enrichment
- miRNA enrichment

- Genes
- Conditions

## Help | Hide | Top Help | Show | Top Expression data

### HELP

The image plot shows the color-coded level of gene expression, for the
genes and conditions in a given transcription module. The genes are on
the horizontal, the conditions on the vertical axis.

The genes are ordered according to their ISA gene scores, similarly
the conditions are ordered according to their condition scores. The
score of a gene means the «degree of inclusion» in
the module: a high score gene is essential in the module.

Condition scores can also be negative, that means that the genes of
the module are all down-regulated in the condition. Here the absolute
value of the score gives the «degree of inclusion».

The plots above and beside the expression matrix show the gene scores
and condition scores, respectively.

Note that the plot is interactive, you can see the name of the gene
and condition under the mouse cursor.

The expression matrix was normalized to have mean zero and standard
deviation one for every gene separately across all conditions
(i.e. not just for the conditions in the module).

— Click on the *Help* button again to close this help window.

Gene:   
Condition:

Under-expression is coded with green,
over-expression with red color.

## Help | Hide | Top Help | Show | Top The GO tree — Biological processes

### HELP

This is one of three sections showing Gene Ontology enrichment of the
current module: in this case for **biological processes**.

The graph shows the hierarchy of the GO categories, their enrichment
for the current module is color coded, and the blue number beside the
category is the minus log ten p-value of the enrichment. (Calculated
using the standard hypergeometric test.) The color of the arrows code
«is a» (cyan) and «part of» relationships.

The tree was built the following way. First all GO terms with more
significant enrichment p-value than 0.05 were collected. Then all
paths from these terms to the root node of the GO tree were included
too. If a GO term is included more than once in the tree, then the
green numbers show 1) the id of the node, this makes it easier to find
other appereances of the term, and 2) the number of appearences.

Note that the same GO category might show up on the graph many
times. This is because the GO was «straightened» for this
graph, i.e. if there are more paths from a GO term to the root node of
the tree, all of them are included. The green numbers

Move the mouse cursor over the terms to get their definition. Clicking
on them takes you to the corresponding Gene Ontology web page.

If you cannot see a graph here at all, that means that there were no
significantly enriched GO categories, at the 0.05 level.

— Click on the *Help* button again to close this help window.

## Help | Hide | Top Help | Show | Top The GO tree — Cellular Components

### HELP

This is one of three sections showing Gene Ontology enrichment of the
current module: in this case for **cellular components**.

The graph shows the hierarchy of the GO categories, their enrichment
for the current module is color coded, and the blue number beside the
category is the minus log ten p-value of the enrichment. (Calculated
using the standard hypergeometric test.) The color of the arrows code
«is a» (cyan) and «part of» relationships.

The tree was built the following way. First all GO terms with more
significant enrichment p-value than 0.05 were collected. Then all
paths from these terms to the root node of the GO tree were included
too. If a GO term is included more than once in the tree, then the
green numbers show 1) the id of the node, this makes it easier to find
other appereances of the term, and 2) the number of appearences.

Note that the same GO category might show up on the graph many
times. This is because the GO was «straightened» for this
graph, i.e. if there are more paths from a GO term to the root node of
the tree, all of them are included. The green numbers

Move the mouse cursor over the terms to get their definition. Clicking
on them takes you to the corresponding Gene Ontology web page.

If you cannot see a graph here at all, that means that there were no
significantly enriched GO categories, at the 0.05 level.

— Click on the *Help* button again to close this help window.

:   **cellular\_component**

    The part of a cell or its extracellular environment in which a gene product is located. A gene product may be located in one or more parts of a cell and its location may be as specific as a particular macromolecular complex, that is, a stable, persistent association of macromolecules that function together.
:   **cytosol**

    The part of the cytoplasm that does not contain organelles but which does contain other particulate matter, such as protein complexes.
:   **cell**

    The basic structural and functional unit of all organisms. Includes the plasma membrane and any external encapsulating structures such as the cell wall and cell envelope.
:   **cytoplasm**

    All of the contents of a cell excluding the plasma membrane and nucleus, but including other subcellular structures.
:   **intracellular part**

    Any constituent part of the living contents of a cell; the matter contained within (but not including) the plasma membrane, usually taken to exclude large vacuoles and masses of secretory or ingested material. In eukaryotes it includes the nucleus and cytoplasm.
:   **cytoplasmic part**

    Any constituent part of the cytoplasm, all of the contents of a cell excluding the plasma membrane and nucleus, but including other subcellular structures.
:   **cytosolic part**

    Any constituent part of cytosol, that part of the cytoplasm that does not contain membranous or particulate subcellular components.
:   **cell part**

    Any constituent part of a cell, the basic structural and functional unit of all organisms.
:   **all**

    NA
:   **NA**

    NA
:   **all**

    NA
:   **cytoplasmic part**

    Any constituent part of the cytoplasm, all of the contents of a cell excluding the plasma membrane and nucleus, but including other subcellular structures.
:   **cytosolic part**

    Any constituent part of cytosol, that part of the cytoplasm that does not contain membranous or particulate subcellular components.
:   **cell part**

    Any constituent part of a cell, the basic structural and functional unit of all organisms.

## Help | Hide | Top Help | Show | Top The GO tree — Molecular Function

### HELP

This is one of three sections showing Gene Ontology enrichment of the
current module: in this case for **molecular function**.

The graph shows the hierarchy of the GO categories, their enrichment
for the current module is color coded, and the blue number beside the
category is the minus log ten p-value of the enrichment. (Calculated
using the standard hypergeometric test.) The color of the arrows code
«is a» (cyan) and «part of» relationships.

The tree was built the following way. First all GO terms with more
significant enrichment p-value than 0.05 were collected. Then all
paths from these terms to the root node of the GO tree were included
too. If a GO term is included more than once in the tree, then the
green numbers show 1) the id of the node, this makes it easier to find
other appereances of the term, and 2) the number of appearences.

Note that the same GO category might show up on the graph many
times. This is because the GO was «straightened» for this
graph, i.e. if there are more paths from a GO term to the root node of
the tree, all of them are included. The green numbers

Move the mouse cursor over the terms to get their definition. Clicking
on them takes you to the corresponding Gene Ontology web page.

If you cannot see a graph here at all, that means that there were no
significantly enriched GO categories, at the 0.05 level.

— Click on the *Help* button again to close this help window.

## Help | Hide | Top Help | Show | Top GO BP test for over-representation

### HELP

List of all enriched GO categories (biological processes), at the 0.05
p-value level.

The columns:

- **ExpCount** is the expected count of genes in the
  module annotated with the given GO term, just by chance.
- **Count**
  is the number of genes in the module annotated with the given GO
  term.
- **Size** is the total number of genes (in our universe)
  annotated with the GO term.

Clicking on **Count** shows the genes that drive the
enrichment. You can also click on the individual numbers in
the **Count** column, to show the driving genes for that individual
GO category.

Clicking on the GO identifiers takes you to the Gene Ontology web
pages.

— Click on the *Help* button again to close this help window.

No enriched terms

## Help | Hide | Top Help | Show | Top GO CC test for over-representation

### HELP

List of all enriched GO categories (cellular components), at the 0.05
p-value level.

The columns:

- **ExpCount** is the expected count of genes in the
  module annotated with the given GO term, just by chance.
- **Count**
  is the number of genes in the module annotated with the given GO
  term.
- **Size** is the total number of genes (in our universe)
  annotated with the GO term.

Clicking on **Count** shows the genes that drive the
enrichment. You can also click on the individual numbers in
the **Count** column, to show the driving genes for that individual
GO category.

Clicking on the GO identifiers takes you to the Gene Ontology web
pages.

— Click on the *Help* button again to close this help window.

| Id | Pvalue | ExpCount | Count | Size | Term |
| --- | --- | --- | --- | --- | --- |
| GO:0044445 | 2.521e-03 | 83.8 | 114 Aip, Apaf1, Apod, Bag6, Bloc1s1, Bloc1s3, Bloc1s4, Casp9, Cct3, Cct4, Cct5, Cct6b, Cct7, Chuk, Ciao1, Dapp1, Ddx3x, Eif2ak4, Eno2, Erc1, Fam96b, Fau, Get4, Gipc1, Gucy1a3, Gucy1b3, H2-Ke2, Hba-a1, Hba-a2, Hbb-b1, Hbb-bs, Ide, Ikbkb, Ikbkg, Kxd1, Mapk14, Nufip1, Pdrg1, Pfdn2, Pfdn5, Pfkl, Pfkm, Pik3c2b, Pik3ca, Pik3cb, Pik3cd, Pik3cg, Pik3r1, Pik3r5, Pik3r6, Prdx3, Psmc4, Psmc5, Psmc6, Pycard, Rpl10, Rpl10a, Rpl11, Rpl12, Rpl13a, Rpl14, Rpl15, Rpl17, Rpl18, Rpl18a, Rpl19, Rpl21, Rpl22, Rpl23a, Rpl26, Rpl27, Rpl28, Rpl29, Rpl3, Rpl30, Rpl32, Rpl35, Rpl36a, Rpl37a, Rpl38, Rpl3l, Rpl4, Rpl6, Rpl7, Rpl9, Rplp0, Rplp2, Rps10, Rps12, Rps14, Rps15, Rps16, Rps17, Rps18, Rps19, Rps2, Rps23, Rps24, Rps26, Rps27a, Rps29, Rps3a1, Rps4x, Rps6, Rps7, Snap47, Sra1, Tcp1, Tsc1, Tsc2, Uba52, Uri1, Uxt, Zcchc17 | 172 | cytosolic part |
| GO:0044391 | 1.067e-02 | 60.9 | 85 Ddx3x, Fau, Gnb2l1, Hba-a1, Hba-a2, Ict1, Mrpl11, Mrpl13, Mrpl15, Mrpl16, Mrpl17, Mrpl2, Mrpl20, Mrpl22, Mrpl27, Mrpl34, Mrpl36, Mrpl39, Mrpl41, Mrpl42, Mrps10, Mrps11, Mrps12, Mrps14, Mrps15, Mrps18a, Mrps2, Mrps21, Mrps22, Mrps23, Mrps25, Mrps31, Npm1, Rpl10, Rpl10a, Rpl11, Rpl12, Rpl13a, Rpl14, Rpl15, Rpl17, Rpl18, Rpl18a, Rpl19, Rpl21, Rpl22, Rpl23a, Rpl26, Rpl27, Rpl28, Rpl29, Rpl3, Rpl30, Rpl32, Rpl35, Rpl36a, Rpl37a, Rpl38, Rpl3l, Rpl4, Rpl6, Rpl7, Rpl9, Rplp0, Rplp2, Rps10, Rps12, Rps14, Rps15, Rps16, Rps17, Rps18, Rps19, Rps2, Rps23, Rps24, Rps26, Rps27a, Rps29, Rps3a1, Rps4x, Rps6, Rps7, Uba52, Zcchc17 | 125 | ribosomal subunit |
| GO:0022626 | 3.200e-02 | 40.44 | 59 Apod, Ddx3x, Eif2ak4, Fau, Hba-a1, Hba-a2, Nufip1, Rpl10, Rpl10a, Rpl11, Rpl12, Rpl13a, Rpl14, Rpl15, Rpl17, Rpl18, Rpl18a, Rpl19, Rpl21, Rpl22, Rpl23a, Rpl26, Rpl27, Rpl28, Rpl29, Rpl3, Rpl30, Rpl32, Rpl35, Rpl36a, Rpl37a, Rpl38, Rpl3l, Rpl4, Rpl6, Rpl7, Rpl9, Rplp0, Rplp2, Rps10, Rps12, Rps14, Rps15, Rps16, Rps17, Rps18, Rps19, Rps2, Rps23, Rps24, Rps26, Rps27a, Rps29, Rps3a1, Rps4x, Rps6, Rps7, Uba52, Zcchc17 | 83 | cytosolic ribosome |

## Help | Hide | Top Help | Show | Top GO MF test for over-representation

### HELP

List of all enriched GO categories (molecular function), at the 0.05
p-value level.

The columns:

- **ExpCount** is the expected count of genes in the
  module annotated with the given GO term, just by chance.
- **Count**
  is the number of genes in the module annotated with the given GO
  term.
- **Size** is the total number of genes (in our universe)
  annotated with the GO term.

Clicking on **Count** shows the genes that drive the
enrichment. You can also click on the individual numbers in
the **Count** column, to show the driving genes for that individual
GO category.

Clicking on the GO identifiers takes you to the Gene Ontology web
pages.

— Click on the *Help* button again to close this help window.

No enriched terms

## Help | Hide | Top Help | Show | Top KEGG Pathway test for over-representation

### HELP

List of all enriched KEGG pathways, at the 0.05
p-value level.

The columns:

- **ExpCount** is the expected count of genes in the
  module annotated with the given KEGG pathway, just by chance.
- **Count**
  is the number of genes in the module annotated with the given KEGG
  pathway.
- **Size** is the total number of genes (in our universe)
  annotated with the KEGG pathway.

Clicking on **Count** shows the genes that drive the
enrichment. You can also click on the individual numbers in
the **Count** column, to show the driving genes for that individual
KEGG pathway.

Clicking on the KEGG identifiers takes you to the KEGG web site.

— Click on the *Help* button again to close this help window.

| Id | Pvalue | ExpCount | Count | Size | Term |
| --- | --- | --- | --- | --- | --- |
| 03010 | 1.866e-02 | 45.69 | 64 Fau, Gm10420, Gm15501, Gm6139, Gm8841, LOC100505031, LOC677113, Mrpl13, Rpl10, Rpl10a, Rpl11, Rpl12, Rpl13a, Rpl14, Rpl15, Rpl17, Rpl18, Rpl18a, Rpl19, Rpl21, Rpl22, Rpl23a, Rpl26, Rpl27, Rpl28, Rpl29, Rpl3, Rpl30, Rpl32, Rpl34, Rpl35, Rpl36, Rpl36a, Rpl37a, Rpl38, Rpl39l, Rpl3l, Rpl4, Rpl5, Rpl6, Rpl7, Rpl9, Rplp0, Rplp2, Rps10, Rps12, Rps14, Rps15, Rps16, Rps17, Rps18, Rps19, Rps2, Rps23, Rps24, Rps26, Rps27a, Rps27l, Rps29, Rps3a1, Rps4x, Rps6, Rps7, Uba52 | 93 | Ribosome |


### HELP

List of all enriched miRNA families, at the 0.05
p-value level.

The columns:

- **ExpCount** is the expected count of genes in the
  module regulated by the given miRNA family, just by chance.
- **Count**
  is the number of genes in the module regulated by the given miRNA
  family.
- **Size** is the total number of genes (in our universe)
  regulated with the given miRNA family.

Clicking on **Count** shows the genes that drive the
enrichment. You can also click on the individual numbers in
the **Count** column, to show the driving genes for that individual
miRNA family.

The miRNA regulation data was taken from the

Top


### HELP

p-value level.

The columns:

- **ExpCount** is the expected number of genes in the- **Count**- **Size** is the total number of genes (in our universe)

Clicking on **Count** shows the genes that drive the
enrichment. You can also click on the individual numbers in
the **Count** column, to show the driving genes for that individual

— Click on the *Help* button again to close this help window.

## Help | Hide | Top Help | Show | Top Genes

### HELP

A list of all genes in the current module, in alphabetical order. The
size of the text corresponds to the gene scores.

Note that some gene symbols may show up more than once, if many
probes match the same Entrez gene.

Genes with no Entrez mapping are given separately, with their
Affymetrics probe ID.

— Click on the *Help* button again to close this help window.

### Genes Symbol

, score:

AanatUnknown, score: 0.18
Abca4Unknown, score: 0.3
Abi1Unknown, score: 0.09
Abl1Unknown, score: 0.1
Abl2Unknown, score: 0.11
AcadlUnknown, score: 0.25
AcadmUnknown, score: 0.33
AcadvlUnknown, score: 0.57
AcadsUnknown, score: 0.25
AceUnknown, score: 0.31
Macf1Unknown, score: 0.27
Aco1Unknown, score: 0.14
Aco2Unknown, score: 0.28
Acox1Unknown, score: 0.29
Acp2Unknown, score: 0.15
Acp5Unknown, score: 0.32
AcrUnknown, score: 0.3
ActbUnknown, score: 0.49
Actc1Unknown, score: 0.3
Actn2Unknown, score: 0.19
Acvr1Unknown, score: 0.2
Acvr2aUnknown, score: 0.55
Acvr2bUnknown, score: 0.09
Adam10Unknown, score: 0.03
Adam8Unknown, score: 0.04
Adamts1Unknown, score: 0.23
Adcy8Unknown, score: 0.05
Adcyap1r1Unknown, score: 0.23
Add1Unknown, score: 0.03
Plin2Unknown, score: 0.03
Adh7Unknown, score: 0.04
Adh5Unknown, score: 0.18
AdkUnknown, score: 0.25
AdmUnknown, score: 0.01
Gpr182Unknown, score: 0.24
CfdUnknown, score: 0.18
AdnpUnknown, score: 0.36
Adora1Unknown, score: 0.24
Adora2bUnknown, score: 0.22
Adora3Unknown, score: 0.14
AdprhUnknown, score: 0.02
Parp2Unknown, score: 0.61
Adra1bUnknown, score: 0.29
Adra1aUnknown, score: 0.42
Adra2aUnknown, score: 0.38
Adra2bUnknown, score: 0.38
Adra2cUnknown, score: 0.48
Adrb1Unknown, score: 0.41
Adrb2Unknown, score: 0.03
Adrb3Unknown, score: 0.23
AdslUnknown, score: 0.11
Aebp2Unknown, score: 0.24
AgerUnknown, score: 0.15
Angpt1Unknown, score: 0.01
Angpt2Unknown, score: 0.12
Angpt4Unknown, score: 0.08
AgrnUnknown, score: 0.23
AgtUnknown, score: 0.56
Agtr2Unknown, score: 0.12
AgxtUnknown, score: 0.17
Aim1Unknown, score: 0.37
AipUnknown, score: 0.22
AireUnknown, score: 0.29
Ak1Unknown, score: 0.28
Ak2Unknown, score: 0.14
Ak4Unknown, score: 0.22
AlplUnknown, score: 0.02
Akt1Unknown, score: 0.22
Akt2Unknown, score: 0.07
Alas1Unknown, score: 0.15
AlcamUnknown, score: 0.07
Aldh1a1Unknown, score: 0.95
Aldh3a2Unknown, score: 0.16
AldoaUnknown, score: 0.06
AldocUnknown, score: 0.44
Akr1b3Unknown, score: 0.48
Alox12Unknown, score: 0.03
Alox8Unknown, score: 0.25
Alox5apUnknown, score: 0.59
Amd1Unknown, score: 0.22
Ank1Unknown, score: 0.1
Ank3Unknown, score: 0
Anp32aUnknown, score: 0.24
Slc25a4Unknown, score: 0.18
Slc25a5Unknown, score: 0.04
Aoc3Unknown, score: 0.29
Prdx3Unknown, score: 0.03
Prdx6Unknown, score: 0.02
Aox1Unknown, score: 0.52
Ap1g2Unknown, score: 0.09
Ap1s1Unknown, score: 0.08
Fabp4Unknown, score: 0.14
Ap2a1Unknown, score: 0.23
Ap3b1Unknown, score: 0.25
Ap3s1Unknown, score: 0.05
Ap3s2Unknown, score: 0.22
Apaf1Unknown, score: 0.07
Apba2Unknown, score: 0.09
Apbb1Unknown, score: 0.14
SpegUnknown, score: 0.37
Api5Unknown, score: 0.16
ApodUnknown, score: 0.26
AppUnknown, score: 0.17
AprtUnknown, score: 0.7
Aqp6Unknown, score: 0.23
Aqp7Unknown, score: 0.3
ArUnknown, score: 0.07
ArafUnknown, score: 0.42
Rplp0Unknown, score: 0.14
AregUnknown, score: 0.23
Arf1Unknown, score: 0.26
Arf4Unknown, score: 0.58
Arg1Unknown, score: 0.07
Arg2Unknown, score: 0.02
RhoaUnknown, score: 0.09
RhocUnknown, score: 0.18
Rnd2Unknown, score: 0.32
ArntUnknown, score: 0.02
Arpc1bUnknown, score: 0.15
Art1Unknown, score: 0.23
ArtnUnknown, score: 0.58
ArxUnknown, score: 0.03
Asgr1Unknown, score: 0.15
Zfhx3Unknown, score: 0.15
AtmUnknown, score: 0.17
Atp1a1Unknown, score: 0.17
Atp1b1Unknown, score: 0.04
Fxyd2Unknown, score: 0.2
Atp4aUnknown, score: 0.1
Atp4bUnknown, score: 0.28
Atp5a1Unknown, score: 0.32
Atp5bUnknown, score: 0.06
Atp5c1Unknown, score: 0.15
Atp5f1Unknown, score: 0.41
Atp5g1Unknown, score: 0.02
Atp5jUnknown, score: 0.56
Atp6v1aUnknown, score: 0.27
Atp6v1b2Unknown, score: 0.38
Atp6v0d1Unknown, score: 0.15
Atp6v1e1Unknown, score: 0.15
Atp7aUnknown, score: 0.04
Atp7bUnknown, score: 0.12
Atp10aUnknown, score: 0.05
Atpif1Unknown, score: 0.05
Slc7a2Unknown, score: 0.01
Slc7a3Unknown, score: 0.13
HnrnpdUnknown, score: 0.35
Pcdh15Unknown, score: 0.27
Azgp1Unknown, score: 0.14
B2mUnknown, score: 0.19
Bach1Unknown, score: 0.15
Bach2Unknown, score: 0.2
Bag1Unknown, score: 0.04
Bard1Unknown, score: 0.05
Barx2Unknown, score: 0.22
Bcl6bUnknown, score: 0.05
BcanUnknown, score: 0.08
Bcat1Unknown, score: 0.51
Bcat2Unknown, score: 0.29
BcheUnknown, score: 0.72
BckdhaUnknown, score: 0.37
BckdhbUnknown, score: 0.63
BckdkUnknown, score: 0.01
Bcl2a1bUnknown, score: 0.07
Bcl2l1Unknown, score: 0.18
Bcl6Unknown, score: 0.05
Bcl7bUnknown, score: 0.29
Bdkrb2Unknown, score: 0.15
Bfsp1Unknown, score: 0.35
Glb1Unknown, score: 0
BidUnknown, score: 0.3
HrkUnknown, score: 0.44
BikUnknown, score: 0.23
Bcl2l11Unknown, score: 0.06
Fabp7Unknown, score: 0.16
Prdm1Unknown, score: 0.23
BlmUnknown, score: 0.17
Bmp2Unknown, score: 0.18
Bmp5Unknown, score: 0.11
Bmp6Unknown, score: 0.24
Bmpr1aUnknown, score: 0.03
Bnip3Unknown, score: 0.1
Bnip3lUnknown, score: 0.11
Smyd1Unknown, score: 0.75
Bst1Unknown, score: 0.02
Brca2Unknown, score: 0
Zfp36l1Unknown, score: 0.08
Chic1Unknown, score: 0.29
BsgUnknown, score: 0.01
BtcUnknown, score: 0.05
Klf5Unknown, score: 0.07
BtkUnknown, score: 0.12
Bub1bUnknown, score: 0.15
Commd3Unknown, score: 0.2
C1qaUnknown, score: 0.03
C1qbUnknown, score: 0.34
C1qbpUnknown, score: 0.05
C4bpUnknown, score: 0.02
C9Unknown, score: 0.16
Hyou1Unknown, score: 0.09
Cacna1dUnknown, score: 0.28
Cacna1eUnknown, score: 0.48
Cacna1gUnknown, score: 0.03
Cacna2d3Unknown, score: 0.01
Pdia4Unknown, score: 0.01
AspmUnknown, score: 0.16
CaluUnknown, score: 0.1
Camk2aUnknown, score: 0.08
Camk2bUnknown, score: 0.03
Camk4Unknown, score: 0.04
CamlUnknown, score: 0.12
CapgUnknown, score: 0.09
Capn2Unknown, score: 0.14
Capn3Unknown, score: 0.16
Car11Unknown, score: 0.07
Car2Unknown, score: 0.12
Car4Unknown, score: 0.19
Car5aUnknown, score: 0.19
Car7Unknown, score: 0
Casp12Unknown, score: 0.39
Casp6Unknown, score: 0.07
Casp8Unknown, score: 0.76
Casp9Unknown, score: 0.08
CastUnknown, score: 0
Ctnna1Unknown, score: 0.26
Cav1Unknown, score: 0.01
Cav2Unknown, score: 0
Runx1Unknown, score: 0.38
Cbfa2t2Unknown, score: 0.11
Cbfa2t3Unknown, score: 0.04
CblUnknown, score: 0.26
Cbr1Unknown, score: 0.54
Cbr2Unknown, score: 0.33
Cbx1Unknown, score: 0.08
Ccnd1Unknown, score: 0.21
Ccnd2Unknown, score: 0.09
Ccng1Unknown, score: 0.11
Ccr6Unknown, score: 0.41
Cct3Unknown, score: 0.01
Cct4Unknown, score: 0.12
Cct5Unknown, score: 0.24
Cct6bUnknown, score: 0.21
Cct7Unknown, score: 0
Cd14Unknown, score: 0
Ctla4Unknown, score: 0.06
Cd19Unknown, score: 0.36
Cd1d1Unknown, score: 0.12
Ms4a1Unknown, score: 0.08
Cd24aUnknown, score: 0.45
Scarb2Unknown, score: 0.22
Entpd1Unknown, score: 0.03
Entpd2Unknown, score: 0.23
Entpd6Unknown, score: 0.14
Entpd5Unknown, score: 0.35
Cd3gUnknown, score: 0.4
Cd247Unknown, score: 0.12
Cd48Unknown, score: 0.28
Cd59aUnknown, score: 0.25
Cd6Unknown, score: 0.05
Cd68Unknown, score: 0.11
Cd72Unknown, score: 0.16
Cd80Unknown, score: 0.16
Cd81Unknown, score: 0.24
Cd86Unknown, score: 0.26
Cd8aUnknown, score: 0.28
Cdk11bUnknown, score: 0.22
Cdc42Unknown, score: 0.16
Cdh11Unknown, score: 0.19
Cdh13Unknown, score: 0.08
Cdh5Unknown, score: 0.2
Cdh6Unknown, score: 0.33
Cdk2Unknown, score: 0.41
Cdk4Unknown, score: 0.14
Cdk5Unknown, score: 0.43
Cdk6Unknown, score: 0.34
Cdkn1bUnknown, score: 0.29
Cdkn2aUnknown, score: 0.07
Cdkn2bUnknown, score: 0.03
MiaUnknown, score: 0.11
CdylUnknown, score: 0.22
CebpzUnknown, score: 0.45
CebpgUnknown, score: 0.35
Celsr1Unknown, score: 0.45
CenpaUnknown, score: 0.27
Ces1gUnknown, score: 0.06
12631Unknown, score: 0.26
Ch25hUnknown, score: 0.22
Chek1Unknown, score: 0.18
Chil3Unknown, score: 0.11
Ovgp1Unknown, score: 0.26
ChmlUnknown, score: 0.1
ChukUnknown, score: 0.06
CideaUnknown, score: 0.06
CidebUnknown, score: 0.15
Elovl3Unknown, score: 0.22
InadlUnknown, score: 0.4
CirbpUnknown, score: 0.24
Socs3Unknown, score: 0.32
CitUnknown, score: 0.07
Cited1Unknown, score: 0.04
CkbUnknown, score: 0.21
Ckmt1Unknown, score: 0.09
Clca3a1Unknown, score: 0.53
Clcn1Unknown, score: 0.47
Clcn2Unknown, score: 0.23
Clcn5Unknown, score: 0.37
Clk2Unknown, score: 0.06
Tpp1Unknown, score: 0.04
CltaUnknown, score: 0.08
Cxcr2Unknown, score: 0.04
Cxcr3Unknown, score: 0.1
Ccr1Unknown, score: 0.07
Ccr5Unknown, score: 0.09
Ccr7Unknown, score: 0.31
Ccr10Unknown, score: 0.26
Cnih1Unknown, score: 0.32
Cnih2Unknown, score: 0.01
Cnn2Unknown, score: 0.11
Cnr1Unknown, score: 0.06
Cnr2Unknown, score: 0.05
CntfUnknown, score: 0.35
Hps3Unknown, score: 0.03
CoblUnknown, score: 0.02
CochUnknown, score: 0.21
Col12a1Unknown, score: 0.42
Col15a1Unknown, score: 0.37
Col6a3Unknown, score: 0.15
Col9a2Unknown, score: 0.09
CompUnknown, score: 0.46
ComtUnknown, score: 0.04
Cops2Unknown, score: 0.25
CortUnknown, score: 0.21
Cox17Unknown, score: 0.34
Cox4i1Unknown, score: 0.59
Cox5aUnknown, score: 0.66
Cox5bUnknown, score: 0.21
Cox6a1Unknown, score: 0.38
Cox6a2Unknown, score: 0.33
Cox6cUnknown, score: 0.09
Cox7a1Unknown, score: 0.37
Cox7a2Unknown, score: 0.3
Cox7cUnknown, score: 0.52
Cox8aUnknown, score: 0.57
Cox8bUnknown, score: 0.02
CpdUnknown, score: 0.37
CpeUnknown, score: 0.07
Cys1Unknown, score: 0.14
Cplx2Unknown, score: 0.23
Cpne6Unknown, score: 0.43
CpoxUnknown, score: 0.14
Cpt1aUnknown, score: 0.61
Cpt1bUnknown, score: 0.32
Crabp2Unknown, score: 0.02
CraddUnknown, score: 0.38
CratUnknown, score: 0.24
CrebbpUnknown, score: 0.02
CremUnknown, score: 0.06
CrhbpUnknown, score: 0.57
Crip1Unknown, score: 0.13
Pcdha11Unknown, score: 0.08
Cry1Unknown, score: 0.06
Cry2Unknown, score: 0.3
CryabUnknown, score: 0.1
Cryba4Unknown, score: 0.1
Crybb1Unknown, score: 0.18
CrymUnknown, score: 0.21
CryzUnknown, score: 0.39
CsUnknown, score: 0.22
Csf1Unknown, score: 0.06
CskUnknown, score: 0.13
Csnk2a2Unknown, score: 0.19
Csrp2Unknown, score: 0.32
Csrp3Unknown, score: 0.05
Cst3Unknown, score: 0.09
Cst7Unknown, score: 0.18
CstbUnknown, score: 0.19
CtcfUnknown, score: 0.23
Ctla2bUnknown, score: 0.02
Pcyt1aUnknown, score: 0.14
CtssUnknown, score: 0.18
Cux1Unknown, score: 0.26
Cux2Unknown, score: 0.09
Cx3cr1Unknown, score: 0.56
CxadrUnknown, score: 0.02
Cyb561Unknown, score: 0.37
CybbUnknown, score: 0
CycsUnknown, score: 0.29
CyctUnknown, score: 0.07
Cyp11a1Unknown, score: 0.03
Cyp2a5Unknown, score: 0.19
Cyp3a11Unknown, score: 0.13
Cyp27b1Unknown, score: 0.08
Cyp4a14Unknown, score: 0.08
Dach1Unknown, score: 0.19
Dag1Unknown, score: 0.03
DgkaUnknown, score: 0.12
DaoUnknown, score: 0.3
Dapk2Unknown, score: 0.19
DaxxUnknown, score: 0.19
DazlUnknown, score: 0.51
DbhUnknown, score: 0.14
DbnlUnknown, score: 0.2
DbpUnknown, score: 0.21
Dclk1Unknown, score: 0.76
Pcbd1Unknown, score: 0.24
DctUnknown, score: 0.38
Dctn1Unknown, score: 0.32
Ddb1Unknown, score: 0.19
Asap1Unknown, score: 0.47
Gadd45aUnknown, score: 0
Ddit3Unknown, score: 0.13
DdnUnknown, score: 0.19
DdostUnknown, score: 0.59
Ddx3xUnknown, score: 0.15
Ddx5Unknown, score: 0.09
Ddx6Unknown, score: 0.02
Slc29a2Unknown, score: 0.01
Twist2Unknown, score: 0.05
Ackr1Unknown, score: 0.35
Dgat1Unknown, score: 0.14
Dgcr6Unknown, score: 0.48
Dgcr2Unknown, score: 0.12
Slc25a1Unknown, score: 0.13
DhfrUnknown, score: 0.03
Diap1Unknown, score: 0.68
DldUnknown, score: 0.14
Dlg1Unknown, score: 0.03
Dlk1Unknown, score: 0.42
Dll1Unknown, score: 0.16
Dlx5Unknown, score: 0.39
DmpkUnknown, score: 0.19
Dmp1Unknown, score: 0.17
Dnajc1Unknown, score: 0.6
Dnase1Unknown, score: 0.28
Dnase1l3Unknown, score: 0.03
Dnase2aUnknown, score: 0.06
Dnm1Unknown, score: 0.18
Trdmt1Unknown, score: 0.09
Dnmt3bUnknown, score: 0.13
DnpepUnknown, score: 0.42
Cdk2ap1Unknown, score: 0.27
Dok1Unknown, score: 0.11
Dok2Unknown, score: 0.28
Reep5Unknown, score: 0.2
Dpagt1Unknown, score: 0.23
Dpep1Unknown, score: 0
Dpm1Unknown, score: 0.56
Dr1Unknown, score: 0.1
Drd1Unknown, score: 0.04
Drg1Unknown, score: 0.02
Drp2Unknown, score: 0.01
Dsc1Unknown, score: 0.02
Dsc2Unknown, score: 0.06
DscamUnknown, score: 0.06
Dsg3Unknown, score: 0.01
Slc26a2Unknown, score: 0.2
Adam28Unknown, score: 0.03
DtnaUnknown, score: 0.02
Usp17laUnknown, score: 0.21
Dusp2Unknown, score: 0.12
Dvl2Unknown, score: 0.19
Dyrk1aUnknown, score: 0.25
E2f5Unknown, score: 0.41
Mapre1Unknown, score: 0.13
Ebf3Unknown, score: 0.2
Sparcl1Unknown, score: 0.43
EdaUnknown, score: 0.1
S1pr1Unknown, score: 0.14
S1pr3Unknown, score: 0.51
Edn1Unknown, score: 0.21
EdnrbUnknown, score: 0.11
Phc1Unknown, score: 0.2
Eef1a1Unknown, score: 0.31
Eef1a2Unknown, score: 0
Eef2Unknown, score: 0.28
Eef2kUnknown, score: 0.52
Efna4Unknown, score: 0.24
Efna5Unknown, score: 0.05
EfsUnknown, score: 0.28
Klk1b22Unknown, score: 0.31
EgfrUnknown, score: 0.65
Rhbdf1Unknown, score: 0.06
Egr3Unknown, score: 0.1
Eif2s1Unknown, score: 0.02
Eif2ak3Unknown, score: 0.15
Ddx19aUnknown, score: 0.19
Eif4a2Unknown, score: 0.06
Eif4eUnknown, score: 0.29
Elf5Unknown, score: 0.17
Elk4Unknown, score: 0.28
ElnUnknown, score: 0.02
EmdUnknown, score: 0.01
Emp2Unknown, score: 0.1
Emx2Unknown, score: 0
Enc1Unknown, score: 0.59
Eno2Unknown, score: 0.13
EomesUnknown, score: 0.1
Epb4.1l4aUnknown, score: 0.01
Epb4.2Unknown, score: 0.03
DmtnUnknown, score: 0.16
StomUnknown, score: 0.13
Epha1Unknown, score: 0.13
Epha2Unknown, score: 0.1
Epha5Unknown, score: 0.04
Ephb2Unknown, score: 0.7
Ephb4Unknown, score: 0.31
Ephx1Unknown, score: 0.31
Stx2Unknown, score: 0.31
Epn1Unknown, score: 0.36
Epn2Unknown, score: 0.48
Eps15Unknown, score: 0.1
Eps8Unknown, score: 0.82
Nr2f6Unknown, score: 0.08
Nr2f1Unknown, score: 0.26
Erbb2Unknown, score: 0.15
Erbb3Unknown, score: 0.08
Erbb4Unknown, score: 0.17
Ercc1Unknown, score: 0.21
ErgUnknown, score: 0.19
EsdUnknown, score: 0.44
Ces1eUnknown, score: 0.16
Ces3bUnknown, score: 0.13
Amz2Unknown, score: 0.49
Smarcad1Unknown, score: 0.21
Gm14288Unknown, score: 0.01
DroshaUnknown, score: 0.78
Chchd2Unknown, score: 0.14
EvlUnknown, score: 0.14
EvplUnknown, score: 0.41
Ewsr1Unknown, score: 0.06
Eya1Unknown, score: 0.37
Eya2Unknown, score: 0.06
Eya4Unknown, score: 0.02
F2rl2Unknown, score: 0.29
F2rl3Unknown, score: 0.09
F8Unknown, score: 0.4
F9Unknown, score: 0.02
FaahUnknown, score: 0.21
Fabp1Unknown, score: 0.15
Acsl1Unknown, score: 0.27
Ptk2Unknown, score: 0.04
Faf1Unknown, score: 0.36
Fscn1Unknown, score: 0.04
FancaUnknown, score: 0.31
FasUnknown, score: 0.03
FaslUnknown, score: 0.33
Srsf10Unknown, score: 0.01
Fat1Unknown, score: 0.4
FauUnknown, score: 0.12
Fbn1Unknown, score: 0.32
Fbn2Unknown, score: 0.17
Fcer1aUnknown, score: 0.12
Fcer1gUnknown, score: 0.12
Fcgr3Unknown, score: 0.02
Fdft1Unknown, score: 0.65
Fdx1Unknown, score: 0.76
FdxrUnknown, score: 0.39
FechUnknown, score: 0.41
Fem1bUnknown, score: 0.11
FerUnknown, score: 0.06
FesUnknown, score: 0.1
FgaUnknown, score: 0.04
Fgf1Unknown, score: 0.22
Fgf12Unknown, score: 0.13
Fgf18Unknown, score: 0.07
Fgfr1Unknown, score: 0.04
Fgfr2Unknown, score: 0.14
Fgfr3Unknown, score: 0.12
FgrUnknown, score: 0.06
Fhl2Unknown, score: 0.2
Fhl4Unknown, score: 0.26
Smc2Unknown, score: 0.12
Fkbp1bUnknown, score: 0
Fkbp2Unknown, score: 0.51
Fkbp5Unknown, score: 0.35
Fkbp7Unknown, score: 0.24
Foxs1Unknown, score: 0.06
Fli1Unknown, score: 0.02
FliiUnknown, score: 0.38
Flot1Unknown, score: 0.17
Flt3Unknown, score: 0.11
Flt3lUnknown, score: 0.03
Fmn1Unknown, score: 0.2
Srgap2Unknown, score: 0.17
FntaUnknown, score: 0.13
Fosl2Unknown, score: 0.48
Fpr1Unknown, score: 0.38
FrkUnknown, score: 0.25
FtcdUnknown, score: 0.52
Fth1Unknown, score: 0.03
Ftl1Unknown, score: 0.01
AktipUnknown, score: 0.07
Fut1Unknown, score: 0.27
Fut4Unknown, score: 0.22
Fv1Unknown, score: 0.64
Timm10bUnknown, score: 0.16
Fxr1Unknown, score: 0.27
Fzd1Unknown, score: 0.07
Fzd4Unknown, score: 0.2
Fzd7Unknown, score: 0.32
Fzd8Unknown, score: 0.52
Fzd9Unknown, score: 0.26
G6pdxUnknown, score: 0.35
Slc37a4Unknown, score: 0.2
Gabpb1Unknown, score: 0.14
Gabrb3Unknown, score: 0
GabreUnknown, score: 0.09
Gabrr1Unknown, score: 0.33
Slc6a12Unknown, score: 0.08
Slc6a13Unknown, score: 0.14
Gad2Unknown, score: 0.04
B4galnt1Unknown, score: 0.19
Galnt1Unknown, score: 0.12
Galr2Unknown, score: 0.78
GcatUnknown, score: 0.16
GaltUnknown, score: 0.56
GapdhUnknown, score: 0.14
GapdhsUnknown, score: 0.2
Gas1Unknown, score: 0.01
Gas2Unknown, score: 0.32
Gas7Unknown, score: 0.12
Gata1Unknown, score: 0.09
Gata5Unknown, score: 0.15
GbaUnknown, score: 0.07
Gbp2Unknown, score: 0.54
Rabac1Unknown, score: 0.32
GcsamUnknown, score: 0.17
GcgrUnknown, score: 0.05
Gcm1Unknown, score: 0.76
Bloc1s1Unknown, score: 0.11
Kat2aUnknown, score: 0.21
Nr6a1Unknown, score: 0.59
GdaUnknown, score: 0.06
Mrps33Unknown, score: 0.14
Gpd1Unknown, score: 0.23
Gdf9Unknown, score: 0.14
Gpd2Unknown, score: 0.09
Gfpt2Unknown, score: 0.06
Gfra2Unknown, score: 0.41
Gfra3Unknown, score: 0.34
Ggta1Unknown, score: 0.21
B4galt1Unknown, score: 0.12
Ggt1Unknown, score: 0.03
GhrUnknown, score: 0.07
Tsc22d3Unknown, score: 0.32
Gja1Unknown, score: 0.52
Gja3Unknown, score: 0.24
Gja4Unknown, score: 0.01
Gja5Unknown, score: 0.16
Gli3Unknown, score: 0.52
GlulUnknown, score: 0.39
Glrp1Unknown, score: 0.13
Glud1Unknown, score: 0.16
Gnl1Unknown, score: 0.26
Gna11Unknown, score: 0.27
Gna13Unknown, score: 0.08
Gna14Unknown, score: 0.1
Gnai3Unknown, score: 0.26
Gnao1Unknown, score: 0.12
GnaqUnknown, score: 0.4
Gnat1Unknown, score: 0.07
Gnat2Unknown, score: 0.05
Gnb1Unknown, score: 0.61
Gnb2Unknown, score: 0.64
Gnb2l1Unknown, score: 0.58
Gnb4Unknown, score: 0.04
Gng2Unknown, score: 0.23
Gng3Unknown, score: 0.51
Gng4Unknown, score: 0.4
Gngt2Unknown, score: 0.74
GnmtUnknown, score: 0.21
GnpatUnknown, score: 0.1
Gnrh1Unknown, score: 0.3
GnrhrUnknown, score: 0.1
Got1Unknown, score: 0.16
Got2Unknown, score: 0.09
Gp1baUnknown, score: 0.08
Lilrb4Unknown, score: 0.2
Gp5Unknown, score: 0.72
Gpaa1Unknown, score: 0.2
Gpc1Unknown, score: 0.04
Gpc4Unknown, score: 0.22
Cmklr1Unknown, score: 0
Gpr3Unknown, score: 0.28
Gpi1Unknown, score: 0.07
Gpr19Unknown, score: 0.85
Gpr37Unknown, score: 0.05
Adgrg1Unknown, score: 0
Nmur1Unknown, score: 0.31
GsrUnknown, score: 0.21
Grb10Unknown, score: 0.2
Grb7Unknown, score: 0.14
Rhpn1Unknown, score: 0.01
P3h3Unknown, score: 0.86
Grcc10Unknown, score: 0.25
Emg1Unknown, score: 0.15
Spsb2Unknown, score: 0.1
Grik2Unknown, score: 0.1
Grik3Unknown, score: 0.48
Nr3c1Unknown, score: 0.32
Grm1Unknown, score: 0.22
Gspt2Unknown, score: 0.62
GssUnknown, score: 0.3
Gsta1Unknown, score: 0.15
Gsta3Unknown, score: 0.17
Gsta4Unknown, score: 0.55
Gstm1Unknown, score: 0.12
Gstm2Unknown, score: 0.12
Gstm4Unknown, score: 0
Gstm5Unknown, score: 0.15
Gstp2Unknown, score: 0.45
Gstp1Unknown, score: 0.51
Gstt1Unknown, score: 0.57
Gstt2Unknown, score: 0.46
Gsto1Unknown, score: 0.05
Gtf2h1Unknown, score: 0.23
Gtf2h4Unknown, score: 0.2
Gtf2iUnknown, score: 0.29
Cfap20Unknown, score: 0.06
Trip12Unknown, score: 0.45
Gucy2eUnknown, score: 0.62
Magi1Unknown, score: 0.42
GykUnknown, score: 0.15
GzmkUnknown, score: 0.16
H13Unknown, score: 0.01
Hist1h1dUnknown, score: 0.07
H2-AaUnknown, score: 0.09
CfbUnknown, score: 0.04
H2-BlUnknown, score: 0.29
H2-Eb1Unknown, score: 0.15
Pfdn6Unknown, score: 0.04
Slc39a7Unknown, score: 0.31
H2-Ke6Unknown, score: 0.29
H2-D1Unknown, score: 0.08
H2-M2Unknown, score: 0.08
H2-M3Unknown, score: 0.17
H2-OaUnknown, score: 0.16
H2-Q1Unknown, score: 0.13
H2-Q10Unknown, score: 0
H2-Q4Unknown, score: 0.14
H2-Q7Unknown, score: 0.16
H2-Q6Unknown, score: 0.61
H2-T10Unknown, score: 0.11
H2-T24Unknown, score: 0.11
Hist2h3c1Unknown, score: 0.43
H3f3aUnknown, score: 0.43
H3f3aUnknown, score: 0.2
HadhUnknown, score: 0.53
Hap1Unknown, score: 0.01
Has2Unknown, score: 0.17
Has3Unknown, score: 0.13
Hba-a1Unknown, score: 0.32
Hbb-bsUnknown, score: 0.32
Serpind1Unknown, score: 0.27
Hcn1Unknown, score: 0.67
Hcn2Unknown, score: 0.17
Hdac2Unknown, score: 0.03
Hdac3Unknown, score: 0.2
Hdac5Unknown, score: 0.06
HdgfUnknown, score: 0.21
HttUnknown, score: 0.31
Hebp1Unknown, score: 0.44
HephUnknown, score: 0.06
Herc2Unknown, score: 0.3
Hes5Unknown, score: 0.01
Hesx1Unknown, score: 0.18
HexaUnknown, score: 0.34
Hey1Unknown, score: 0.1
Foxq1Unknown, score: 0.35
Foxd3Unknown, score: 0.01
Foxj1Unknown, score: 0.01
Mst1Unknown, score: 0.13
HhexUnknown, score: 0.06
Hiat1Unknown, score: 0.31
Hic1Unknown, score: 0.05
Hif1aUnknown, score: 0.45
Hipk1Unknown, score: 0.16
HiraUnknown, score: 0.42
Hk1Unknown, score: 0.2
Tfb2mUnknown, score: 0.16
HlxUnknown, score: 0.43
Hmgb1Unknown, score: 0.3
Hmgn1Unknown, score: 0.54
Hmgn2Unknown, score: 0.1
Hmgb3Unknown, score: 0.47
HmgcrUnknown, score: 0.23
Hmgcs2Unknown, score: 0.55
Hmga1Unknown, score: 0.14
Hmox1Unknown, score: 0.18
Nr4a1Unknown, score: 0.13
HnrnpcUnknown, score: 0.18
Hnrnpa1Unknown, score: 0.46
HnrnpabUnknown, score: 0.15
HnrnpkUnknown, score: 0.34
Hoxa3Unknown, score: 0.08
Hoxa5Unknown, score: 0.12
Hoxa9Unknown, score: 0.05
Hoxb3Unknown, score: 0.09
Hoxb4Unknown, score: 0.09
Hoxb5Unknown, score: 0.01
Hoxb7Unknown, score: 0.21
Hoxd3Unknown, score: 0.21
Hoxd8Unknown, score: 0.11
HpUnknown, score: 0.01
HpseUnknown, score: 0.19
HpdUnknown, score: 0.19
HpxUnknown, score: 0.05
HrUnknown, score: 0.34
Agfg1Unknown, score: 0.07
HrcUnknown, score: 0.16
Hrh1Unknown, score: 0.51
Eif2ak1Unknown, score: 0.31
Prmt2Unknown, score: 0.13
Hspa1lUnknown, score: 0.26
Hsd11b2Unknown, score: 0.22
Hsd17b2Unknown, score: 0.06
Hsd17b7Unknown, score: 0.14
Hsd3b5Unknown, score: 0.42
Hsph1Unknown, score: 0.12
Hspb1Unknown, score: 0.3
Hspa2Unknown, score: 0.11
Hspa9Unknown, score: 0.41
Sdc2Unknown, score: 0.04
Hspg2Unknown, score: 0.02
Ndst1Unknown, score: 0.47
Trmt2aUnknown, score: 0.18
Htr3aUnknown, score: 0.09
Htr7Unknown, score: 0.23
Elavl1Unknown, score: 0.33
Elavl4Unknown, score: 0.3
Hus1Unknown, score: 0.14
Hyal1Unknown, score: 0.26
Icam2Unknown, score: 0.12
Irf8Unknown, score: 0.27
Id2Unknown, score: 0.46
Id3Unknown, score: 0.04
Id4Unknown, score: 0.1
IdeUnknown, score: 0.01
Idh1Unknown, score: 0.18
Ido1Unknown, score: 0.09
IdsUnknown, score: 0.39
Ier2Unknown, score: 0.27
Ier3Unknown, score: 0.31
Ifit3Unknown, score: 0.21
Ifnar1Unknown, score: 0.11
Ifngr1Unknown, score: 0.54
Ifrd1Unknown, score: 0.34
Ifrd2Unknown, score: 0.09
Igf1rUnknown, score: 0.17
Igf2rUnknown, score: 0.15
IgfalsUnknown, score: 0.35
Igfbp2Unknown, score: 0.35
Igfbp3Unknown, score: 0.21
Igfbp6Unknown, score: 0.54
Il18bpUnknown, score: 0.11
IgtpUnknown, score: 0.06
IhhUnknown, score: 0.02
Cd74Unknown, score: 0.21
IkbkbUnknown, score: 0.18
IkbkgUnknown, score: 0.02
Il10raUnknown, score: 0.34
Il10rbUnknown, score: 0.02
Il11Unknown, score: 0.58
Il11ra1Unknown, score: 0.21
Gm13305Unknown, score: 0.08
Il12aUnknown, score: 0.08
Il12rb1Unknown, score: 0.18
Il12rb2Unknown, score: 0.07
Il13ra1Unknown, score: 0.08
Il15Unknown, score: 0.13
Il15raUnknown, score: 0.01
Il18rapUnknown, score: 0.01
Il1r2Unknown, score: 0.44
Il2raUnknown, score: 0.09
Il2rgUnknown, score: 0.27
Il4raUnknown, score: 0.12
Il5Unknown, score: 0.04
Il6stUnknown, score: 0.01
Il7rUnknown, score: 0.07
Il9rUnknown, score: 0.29
Gimap1Unknown, score: 0.37
IncenpUnknown, score: 0.35
Inpp5dUnknown, score: 0.12
Inppl1Unknown, score: 0.4
IppUnknown, score: 0.01
Irg1Unknown, score: 0.31
Irs1Unknown, score: 0.02
Irs3Unknown, score: 0.27
Irx1Unknown, score: 0.37
Irx2Unknown, score: 0.52
Irx3Unknown, score: 0.05
Itga2Unknown, score: 0.34
Itga2bUnknown, score: 0.36
Itga6Unknown, score: 0.37
Itgb3Unknown, score: 0.17
Eif6Unknown, score: 0.46
Itgb5Unknown, score: 0.24
Itgb6Unknown, score: 0.3
Itgb7Unknown, score: 0.03
Itih4Unknown, score: 0.1
Cuzd1Unknown, score: 0.34
Itpr2Unknown, score: 0.2
Jag1Unknown, score: 0.75
Jak2Unknown, score: 0.39
Jak3Unknown, score: 0.01
F11rUnknown, score: 0.18
AtcayUnknown, score: 0.3
Jarid2Unknown, score: 0.22
JrkUnknown, score: 0.09
Ush1gUnknown, score: 0.28
JunUnknown, score: 0.09
JunbUnknown, score: 0.12
JundUnknown, score: 0.31
JupUnknown, score: 0.23
Kcna1Unknown, score: 0.12
Kcna3Unknown, score: 0.33
Kcna5Unknown, score: 0.08
Kcna7Unknown, score: 0.03
Kcnab3Unknown, score: 0.36
Kcnc3Unknown, score: 0.06
Kcnh1Unknown, score: 0.18
Kcnh2Unknown, score: 0.09
Kcnh3Unknown, score: 0.19
Kcnj10Unknown, score: 0.62
Kcnj3Unknown, score: 0.11
Kcnj4Unknown, score: 0.23
Kcnj8Unknown, score: 0.17
Kcnk2Unknown, score: 0.23
Kcnk3Unknown, score: 0.07
Kcnmb1Unknown, score: 0.11
Kcnn4Unknown, score: 0.08
Kcnq1Unknown, score: 0.06
Kcns1Unknown, score: 0.15
MdficUnknown, score: 0.73
Kif13aUnknown, score: 0
Kif16bUnknown, score: 0.26
Kif21aUnknown, score: 0.07
Kif5cUnknown, score: 0.15
Kif9Unknown, score: 0.07
Kifap3Unknown, score: 0.18
Kifc5bUnknown, score: 0.45
Kifc2Unknown, score: 0.01
KitUnknown, score: 0.27
Fabp5Unknown, score: 0.08
Klc1Unknown, score: 0.12
Klc2Unknown, score: 0.24
Klf12Unknown, score: 0.42
Klf4Unknown, score: 0.36
Klf9Unknown, score: 0.31
Serpina3cUnknown, score: 0.35
Klra1Unknown, score: 0.49
Klra2Unknown, score: 0.29
CU424478.2Unknown, score: 0.28
Klra4Unknown, score: 0.04
Klra5Unknown, score: 0.46
Klra8Unknown, score: 0.5
Kpna4Unknown, score: 0.01
MafbUnknown, score: 0.38
Krt8Unknown, score: 0.09
Ktn1Unknown, score: 0.4
Aff3Unknown, score: 0.13
Lama2Unknown, score: 0.29
Lama4Unknown, score: 0.63
Lamb1Unknown, score: 0.29
Lamb2Unknown, score: 0.4
Lamb3Unknown, score: 0.01
Lamc2Unknown, score: 0.01
Lamp2Unknown, score: 0.57
Lasp1Unknown, score: 0.24
Arhgef2Unknown, score: 0.23
Lbx2Unknown, score: 0.08
LcatUnknown, score: 0.18
Lcp2Unknown, score: 0.24
Ldb2Unknown, score: 0.18
LdhaUnknown, score: 0.32
LdhbUnknown, score: 0.17
Cog1Unknown, score: 0.25
LdlrUnknown, score: 0.29
Lect2Unknown, score: 0.27
Lef1Unknown, score: 0.22
LepUnknown, score: 0.01
LeprUnknown, score: 0.09
LfngUnknown, score: 0.34
Lgals3Unknown, score: 0.17
Lgals9Unknown, score: 0.28
LifUnknown, score: 0.05
Lig3Unknown, score: 0.04
Limk1Unknown, score: 0.19
LipaUnknown, score: 0.4
LipgUnknown, score: 0.27
Llgl1Unknown, score: 0.52
Rps2Unknown, score: 0.54
GzmmUnknown, score: 0.18
Lmnb1Unknown, score: 0.2
Lmnb2Unknown, score: 0.21
Lmo4Unknown, score: 0.04
MyclUnknown, score: 0.15
PhyhUnknown, score: 0.09
Sh2b3Unknown, score: 0.01
Lnx1Unknown, score: 0.22
LplUnknown, score: 0.04
Lrp6Unknown, score: 0.08
Lrp8Unknown, score: 0.23
Lrrfip1Unknown, score: 0.08
Lsp1Unknown, score: 0.21
LssUnknown, score: 0.3
Lst1Unknown, score: 0.02
LtaUnknown, score: 0.53
Lta4hUnknown, score: 0.32
LtbUnknown, score: 0.33
Ltbp3Unknown, score: 0.05
LtbrUnknown, score: 0.73
Ltc4sUnknown, score: 0.08
LtfUnknown, score: 0.2
LumUnknown, score: 0.29
Klrb1aUnknown, score: 0.22
Klrb1cUnknown, score: 0.22
Ly6c1Unknown, score: 0.27
Ly6eUnknown, score: 0.2
Ly75Unknown, score: 0.05
Cd180Unknown, score: 0.01
Il1rl1Unknown, score: 0.5
Ly9Unknown, score: 0.05
LyarUnknown, score: 0.28
Lyz1Unknown, score: 0.37
Tm4sf1Unknown, score: 0
M6prUnknown, score: 0.08
Mab21l1Unknown, score: 0.04
MarcksUnknown, score: 0.16
Mxd3Unknown, score: 0.16
Madcam1Unknown, score: 0.06
MaffUnknown, score: 0.58
MafgUnknown, score: 0.1
MagUnknown, score: 0.36
Ccndbp1Unknown, score: 0.27
Man2a1Unknown, score: 0.08
Man2b1Unknown, score: 0.19
Mapkapk5Unknown, score: 0.04
MarcoUnknown, score: 0.5
Mark3Unknown, score: 0.43
Mas1Unknown, score: 0.01
Ascl1Unknown, score: 0.05
Masp1Unknown, score: 0.23
Matn2Unknown, score: 0
MazUnknown, score: 0
Mbd2Unknown, score: 0.16
Mbd3Unknown, score: 0.11
Mbd4Unknown, score: 0.19
MbpUnknown, score: 0.04
Mc2rUnknown, score: 0.07
Mcm2Unknown, score: 0.05
Mcm5Unknown, score: 0.04
Mcm6Unknown, score: 0.25
Tpsb2Unknown, score: 0.2
Mgrn1Unknown, score: 0.05
Mdm4Unknown, score: 0.43
Slc3a2Unknown, score: 0.21
Mef2aUnknown, score: 0.11
Mef2cUnknown, score: 0.08
Mef2dUnknown, score: 0.21
MelkUnknown, score: 0.16
Fyco1Unknown, score: 0.06
Meox2Unknown, score: 0.16
MetUnknown, score: 0.3
Mettl1Unknown, score: 0.28
Foxc1Unknown, score: 0.28
Foxd2Unknown, score: 0.17
Mfge8Unknown, score: 0.28
Sypl2Unknown, score: 0.54
Mgat1Unknown, score: 0.03
KitlUnknown, score: 0.35
MgpUnknown, score: 0.43
MgmtUnknown, score: 0.21
Mid1Unknown, score: 0.22
MifUnknown, score: 0.11
Cxcl9Unknown, score: 0.3
Minpp1Unknown, score: 0.11
Bhlha15Unknown, score: 0.07
Mknk1Unknown, score: 0.32
Mknk2Unknown, score: 0.3
Mlf1Unknown, score: 0.19
Mlh1Unknown, score: 0.03
Mllt10Unknown, score: 0.25
Aff1Unknown, score: 0.02
Mmp12Unknown, score: 0.16
Mmp11Unknown, score: 0.13
Mmp14Unknown, score: 0.3
Mmp2Unknown, score: 0.16
Mmp8Unknown, score: 0.17
Mmp9Unknown, score: 0.01
Mnat1Unknown, score: 0.22
Ndst2Unknown, score: 0.19
Mns1Unknown, score: 0.26
MntUnknown, score: 0.05
Me1Unknown, score: 0.07
Grap2Unknown, score: 0.04
Mdh2Unknown, score: 0.26
Mdh1Unknown, score: 0.16
Psmd7Unknown, score: 0.09
Mpeg1Unknown, score: 0.29
Mpp1Unknown, score: 0.09
Mre11aUnknown, score: 0.01
Meis2Unknown, score: 0.11
Meis3Unknown, score: 0.01
Mrvi1Unknown, score: 0.36
Cited2Unknown, score: 0.31
Msh6Unknown, score: 0.13
MstnUnknown, score: 0.06
mt-CytbUnknown, score: 0.5
Mt1Unknown, score: 0.31
Map7Unknown, score: 0.36
Mtcp1Unknown, score: 0.02
Mtf1Unknown, score: 0.07
Mtf2Unknown, score: 0.31
Mthfd2Unknown, score: 0.65
MthfrUnknown, score: 0.2
Mtm1Unknown, score: 0.32
Laptm4aUnknown, score: 0.23
MttpUnknown, score: 0.13
Fam89bUnknown, score: 0.15
Muc1Unknown, score: 0.06
Mup3Unknown, score: 0.48
Usp34Unknown, score: 0.11
MutUnknown, score: 0.41
MvkUnknown, score: 0.14
Mxi1Unknown, score: 0.11
Mybl2Unknown, score: 0.16
Ppp1r15aUnknown, score: 0.45
Gadd45bUnknown, score: 0.12
Myef2Unknown, score: 0.08
Myh9Unknown, score: 0.05
Myl4Unknown, score: 0.61
Myl3Unknown, score: 0.16
Myl2Unknown, score: 0.11
MylpfUnknown, score: 0.21
Myo10Unknown, score: 0.07
Myo6Unknown, score: 0.17
Myo7aUnknown, score: 0.25
Myo7bUnknown, score: 0.19
Myo9bUnknown, score: 0.35
MyocUnknown, score: 0.22
Myod1Unknown, score: 0.23
Myom1Unknown, score: 0.56
Nab2Unknown, score: 0.14
NacaUnknown, score: 0.01
Nap1l2Unknown, score: 0.02
Ncam1Unknown, score: 0.03
Nck1Unknown, score: 0.4
Ncoa1Unknown, score: 0.04
Ncoa2Unknown, score: 0.08
Ndrg1Unknown, score: 0.95
Ndufa2Unknown, score: 0.48
Ndufa4Unknown, score: 0.48
Ndufs4Unknown, score: 0.63
NebUnknown, score: 0.12
Sept2Unknown, score: 0.13
Nek2Unknown, score: 0.15
NesUnknown, score: 0.08
Neu1Unknown, score: 0.01
Neurl1aUnknown, score: 0.13
Nfatc1Unknown, score: 0.53
Nfatc2Unknown, score: 0.09
Nfatc3Unknown, score: 0.17
Nfe2Unknown, score: 0.17
NfibUnknown, score: 0.12
Nfil3Unknown, score: 0.17
NfixUnknown, score: 0.02
Nfkb2Unknown, score: 0.06
NfkbiaUnknown, score: 0.55
NfkbieUnknown, score: 0.42
NefmUnknown, score: 0.23
Nfs1Unknown, score: 0.36
NfybUnknown, score: 0.07
NinUnknown, score: 0.18
Ninj1Unknown, score: 0.05
Nipsnap1Unknown, score: 0.11
Nkx2-3Unknown, score: 0.32
NlkUnknown, score: 0.27
NmbrUnknown, score: 0.07
Nme1Unknown, score: 0.36
Nme2Unknown, score: 0.41
Nqo2Unknown, score: 0.11
Cd244Unknown, score: 0.3
Nmt2Unknown, score: 0.06
MycnUnknown, score: 0.2
NnmtUnknown, score: 0.01
Rrp1Unknown, score: 0.2
NodalUnknown, score: 0.03
Mrpl49Unknown, score: 0.14
Nos1Unknown, score: 0.22
Nos2Unknown, score: 0.05
Nos3Unknown, score: 0.19
Notch1Unknown, score: 0.04
Ints6Unknown, score: 0.05
Notch4Unknown, score: 0.03
Nup50Unknown, score: 0.16
Npas1Unknown, score: 0.26
Npc1Unknown, score: 0.13
Npm1Unknown, score: 0.79
NppbUnknown, score: 0.25
Nptx1Unknown, score: 0.12
Nr1i2Unknown, score: 0.14
Slc11a1Unknown, score: 0.06
Slc11a2Unknown, score: 0.27
NrasUnknown, score: 0.2
Nrf1Unknown, score: 0.02
Nrp2Unknown, score: 0.71
Nrxn1Unknown, score: 0.22
NsdhlUnknown, score: 0.29
Nsg1Unknown, score: 0.23
MuskUnknown, score: 0.08
Ntan1Unknown, score: 0.35
Ntf3Unknown, score: 0.31
Nthl1Unknown, score: 0.14
Ntn1Unknown, score: 0.19
Ntrk1Unknown, score: 0.09
Ntrk2Unknown, score: 0.27
Ntrk3Unknown, score: 0.05
Ddr2Unknown, score: 0.68
Ntsr2Unknown, score: 0.11
Dusp8Unknown, score: 0.02
Nucb1Unknown, score: 0.08
NumbUnknown, score: 0.17
NumblUnknown, score: 0.24
Nr4a2Unknown, score: 0.28
Nxph1Unknown, score: 0.34
OatUnknown, score: 0.58
Oaz1Unknown, score: 0.01
OclnUnknown, score: 0.32
Odf2Unknown, score: 0.13
OgdhUnknown, score: 0.27
Olfr18Unknown, score: 0.04
Olfr56Unknown, score: 0.01
OmgUnknown, score: 0.11
Tnfrsf11bUnknown, score: 0.36
Oprl1Unknown, score: 0.12
Orc2Unknown, score: 0.42
Orm1Unknown, score: 0.17
Orm2Unknown, score: 0.25
Orm3Unknown, score: 0.2
Slc25a15Unknown, score: 0.12
Cldn11Unknown, score: 0.02
Ovol1Unknown, score: 0.01
OxtrUnknown, score: 0.08
Oca2Unknown, score: 0.19
Mybbp1aUnknown, score: 0.11
P2rx1Unknown, score: 0.12
P2rx7Unknown, score: 0.04
P2rx6Unknown, score: 0.22
P2ry1Unknown, score: 0.26
P4ha1Unknown, score: 0.35
P4ha2Unknown, score: 0.1
Pafah1b2Unknown, score: 0.13
PamUnknown, score: 0.23
PappaUnknown, score: 0.08
Pax3Unknown, score: 0.06
Pax6Unknown, score: 0.01
Pbx1Unknown, score: 0.11
Kat2bUnknown, score: 0.02
Pcdh10Unknown, score: 0.32
Pcdh8Unknown, score: 0.19
Pcm1Unknown, score: 0.23
Pcmt1Unknown, score: 0.07
PcnaUnknown, score: 0.32
Pcp2Unknown, score: 0.29
Pcsk1Unknown, score: 0.01
FurinUnknown, score: 0.44
Pcsk5Unknown, score: 0.03
Pcsk6Unknown, score: 0.27
Pcsk7Unknown, score: 0.12
Cdk16Unknown, score: 0.63
Cdk18Unknown, score: 0.05
PctpUnknown, score: 0.46
PcxUnknown, score: 0.14
Pdcd1Unknown, score: 0.31
Pdcd4Unknown, score: 0.19
Pdcd6ipUnknown, score: 0.24
Pde4bUnknown, score: 0.19
Pde6gUnknown, score: 0.16
PdgfrbUnknown, score: 0.15
Padi4Unknown, score: 0.18
Enpp1Unknown, score: 0.35
Peg3Unknown, score: 0.26
PemtUnknown, score: 0.35
PenkUnknown, score: 0.34
PepdUnknown, score: 0.11
Per1Unknown, score: 0.07
Per2Unknown, score: 0.26
Per3Unknown, score: 0.11
Pex11aUnknown, score: 0.01
Pex11bUnknown, score: 0.27
Pex16Unknown, score: 0.69
CfpUnknown, score: 0.18
Pfdn2Unknown, score: 0.16
Pfkfb2Unknown, score: 0.12
PfklUnknown, score: 0.36
PfkmUnknown, score: 0.18
Pfn1Unknown, score: 0.18
Pgam1Unknown, score: 0.07
PgfUnknown, score: 0.07
Pgk1Unknown, score: 0.36
Abcb1bUnknown, score: 0.01
Abcb1aUnknown, score: 0.38
PhbUnknown, score: 0.22
Slc25a3Unknown, score: 0.06
Phtf1Unknown, score: 0.7
Pick1Unknown, score: 0.29
PigaUnknown, score: 0.11
Pik3caUnknown, score: 0.2
Pik3cdUnknown, score: 0.04
Pik3r1Unknown, score: 0.53
PikfyveUnknown, score: 0.46
Pim1Unknown, score: 0.29
Pim2Unknown, score: 0.11
Pip4k2aUnknown, score: 0.13
PirbUnknown, score: 0.05
PitpnaUnknown, score: 0.17
Pitx1Unknown, score: 0.41
Pitx2Unknown, score: 0.56
Pitx3Unknown, score: 0.34
PkmUnknown, score: 0.41
PrkcbUnknown, score: 0.09
PrkcgUnknown, score: 0.05
PrkciUnknown, score: 0.01
PrkcqUnknown, score: 0.29
PrkczUnknown, score: 0
Pkd2Unknown, score: 0.09
PkdrejUnknown, score: 0.41
PkibUnknown, score: 0
Pla2g2cUnknown, score: 0.44
Pla2g4aUnknown, score: 0
Plcb1Unknown, score: 0.21
Plcb2Unknown, score: 0.21
Plcb4Unknown, score: 0.61
Plcd1Unknown, score: 0.04
Plcd4Unknown, score: 0.43
Pld3Unknown, score: 0.44
Plod1Unknown, score: 0.02
Plp1Unknown, score: 0.14
Plscr2Unknown, score: 0.13
PltpUnknown, score: 0.06
Plxna2Unknown, score: 0.03
PmlUnknown, score: 0
Pmp22Unknown, score: 0.01
Pms2Unknown, score: 0.11
Pnliprp1Unknown, score: 0.27
Sept5Unknown, score: 0.04
PolbUnknown, score: 0.32
Pold2Unknown, score: 0.21
Pole2Unknown, score: 0.51
PolgUnknown, score: 0.11
Pou2af1Unknown, score: 0.43
Pou4f1Unknown, score: 0.02
EndouUnknown, score: 0.2
Ppap2aUnknown, score: 0.13
Med1Unknown, score: 0.34
Ppargc1aUnknown, score: 0.62
Scand1Unknown, score: 0.1
Ppef2Unknown, score: 0.28
SyplUnknown, score: 0.04
Lgals3bpUnknown, score: 0.3
Ppm1aUnknown, score: 0.11
Ppm1bUnknown, score: 0.31
PpoxUnknown, score: 0.2
Ppp1cbUnknown, score: 0.1
Ppp1ccUnknown, score: 0.2
Ppp3r1Unknown, score: 0.31
Ppp5cUnknown, score: 0.31
Ppt1Unknown, score: 0.1
Mob4Unknown, score: 0.02
Prim2Unknown, score: 0
Prkab1Unknown, score: 0.31
Prkag1Unknown, score: 0.15
Mapk11Unknown, score: 0.05
ProcUnknown, score: 0.02
ProcrUnknown, score: 0.62
ProdhUnknown, score: 0.2
Prpf4bUnknown, score: 0.2
PrxUnknown, score: 0.17
NpeppsUnknown, score: 0.19
PsapUnknown, score: 0.05
Psen1Unknown, score: 0.31
Psen2Unknown, score: 0.08
Psma3Unknown, score: 0.33
Psmb1Unknown, score: 0.2
Psmb10Unknown, score: 0.6
Psmb4Unknown, score: 0.63
Psmb5Unknown, score: 0.61
Psmb6Unknown, score: 0.15
Psmb7Unknown, score: 0.21
Psmc2Unknown, score: 0.58
Psmc3Unknown, score: 0.24
Psmc3ipUnknown, score: 0.15
Psmc5Unknown, score: 0.4
Psmd4Unknown, score: 0.11
Psme1Unknown, score: 0.13
PipoxUnknown, score: 0.23
PspnUnknown, score: 0.08
PtafrUnknown, score: 0.01
Ptbp1Unknown, score: 0.33
Ptch2Unknown, score: 0.26
PterUnknown, score: 0.29
Ptger4Unknown, score: 0.32
PtgisUnknown, score: 0.14
PthlhUnknown, score: 0.11
Ptp4a2Unknown, score: 0.02
Ptpn13Unknown, score: 0.06
Ptpn14Unknown, score: 0.05
Dusp1Unknown, score: 0.31
PtprcUnknown, score: 0.3
PtprdUnknown, score: 0.15
PtprmUnknown, score: 0.51
Ptprn2Unknown, score: 0.05
PtrfUnknown, score: 0.11
Ptx3Unknown, score: 0.03
PuraUnknown, score: 0.06
PvalbUnknown, score: 0.54
Pvrl2Unknown, score: 0.12
Pex19Unknown, score: 0.1
Abcd3Unknown, score: 0.13
Abcd4Unknown, score: 0.49
Pxmp2Unknown, score: 0.17
Pex2Unknown, score: 0.14
PxnUnknown, score: 0.12
Pex5Unknown, score: 0.04
QkUnknown, score: 0.05
Rab18Unknown, score: 0.14
Rab19Unknown, score: 0.77
Rab20Unknown, score: 0.09
Rab23Unknown, score: 0.67
Rab3dUnknown, score: 0.01
Rab4aUnknown, score: 0.41
Rab4bUnknown, score: 0.02
Rab5bUnknown, score: 0.07
Rab7Unknown, score: 0.36
Rac2Unknown, score: 0.08
Rad21Unknown, score: 0.02
Rad23aUnknown, score: 0.33
Rad51bUnknown, score: 0.02
Rad51dUnknown, score: 0.24
Rad54lUnknown, score: 0.24
Rab34Unknown, score: 0.3
Aldh1a2Unknown, score: 0.33
RalyUnknown, score: 0.34
Rangap1Unknown, score: 0.32
Rasgrp2Unknown, score: 0.58
Rasa3Unknown, score: 0.09
Rasal1Unknown, score: 0.02
Rasd1Unknown, score: 0.03
Rasgrf1Unknown, score: 0.05
Rasgrp1Unknown, score: 0.02
Rbbp6Unknown, score: 0.06
Rbl1Unknown, score: 0.07
Rbm6Unknown, score: 0.12
RbmxUnknown, score: 0.14
Rbp1Unknown, score: 0.04
RbpmsUnknown, score: 0.19
RbpjUnknown, score: 0.12
RbpjlUnknown, score: 0.29
Rcn1Unknown, score: 0.29
Pitpnm2Unknown, score: 0.12
RelaUnknown, score: 0.02
RenbpUnknown, score: 0.03
Upf1Unknown, score: 0.03
Dpf2Unknown, score: 0.13
Rfc2Unknown, score: 0.4
RfngUnknown, score: 0.07
Trim27Unknown, score: 0.13
RfxankUnknown, score: 0.49
Slc50a1Unknown, score: 0.08
RalgdsUnknown, score: 0.07
Rgl1Unknown, score: 0.26
Rgs2Unknown, score: 0.27
Rgs5Unknown, score: 0.02
RhagUnknown, score: 0.04
RhebUnknown, score: 0.04
Rnase1Unknown, score: 0.25
Ring1Unknown, score: 0.03
Ripk1Unknown, score: 0.42
Uri1Unknown, score: 0.21
Rnf4Unknown, score: 0.09
Rnf7Unknown, score: 0.18
RoraUnknown, score: 0.43
Rp2hUnknown, score: 0.56
RpgrUnknown, score: 0.2
RpiaUnknown, score: 0.61
Rpl10aUnknown, score: 0.32
Rpl18Unknown, score: 0.26
Rpl19Unknown, score: 0.14
Rpl21Unknown, score: 0.32
Rpl22Unknown, score: 0.65
Rpl26Unknown, score: 0.4
Rpl27Unknown, score: 0.53
Rpl28Unknown, score: 0.23
Rpl29Unknown, score: 0.3
Rpl30Unknown, score: 0.39
Rpl32Unknown, score: 0.34
Rpl37aUnknown, score: 0.67
Rpl36aUnknown, score: 0.34
Rpl6Unknown, score: 0.37
Rpl7Unknown, score: 0.28
Rpl9Unknown, score: 0.45
Rpn2Unknown, score: 0.49
Polr1cUnknown, score: 0.64
Polr1dUnknown, score: 0.36
Polr1aUnknown, score: 0.15
Polr2cUnknown, score: 0.24
Sub1Unknown, score: 0.77
Rps12Unknown, score: 0.44
Rps14Unknown, score: 0.38
Rps15Unknown, score: 0.24
Rps16Unknown, score: 0.76
Rps17Unknown, score: 0.06
Rps18Unknown, score: 0.68
Rps19Unknown, score: 0.06
Rps24Unknown, score: 0.6
Rps29Unknown, score: 0.26
Rps3a1Unknown, score: 0.23
Rps4xUnknown, score: 0.72
Rps6Unknown, score: 0.39
Rps6ka1Unknown, score: 0.18
Rps6ka2Unknown, score: 0.29
Rps7Unknown, score: 0.09
Rrm1Unknown, score: 0.08
Rrm2Unknown, score: 0.09
Rs1Unknown, score: 0.06
Rsu1Unknown, score: 0.22
RtknUnknown, score: 0.39
Rtn2Unknown, score: 0.05
Rtn3Unknown, score: 0.73
Hps6Unknown, score: 0.2
Ruvbl2Unknown, score: 0.4
RxraUnknown, score: 0.7
RxrbUnknown, score: 0.2
Nr1h4Unknown, score: 0.01
RykUnknown, score: 0.03
Ryr2Unknown, score: 0.11
Ryr3Unknown, score: 0.11
S100a11Unknown, score: 0.22
S100a13Unknown, score: 0.08
S100a4Unknown, score: 0.17
S100a6Unknown, score: 0.09
S100a8Unknown, score: 0.05
Saa1Unknown, score: 0.04
Saa4Unknown, score: 0.25
Acsm3Unknown, score: 0.33
Sap18Unknown, score: 0.24
Sf3a2Unknown, score: 0.31
Sar1aUnknown, score: 0.03
SarsUnknown, score: 0.05
Satb1Unknown, score: 0.16
Clec11aUnknown, score: 0.06
Stmn2Unknown, score: 0.42
ScinUnknown, score: 0.08
Scn1bUnknown, score: 0.08
Scn7aUnknown, score: 0.04
Scn8aUnknown, score: 0.19
Scnn1bUnknown, score: 0.05
Scp2Unknown, score: 0.08
Msr1Unknown, score: 0.58
ScxUnknown, score: 0.23
Ccl25Unknown, score: 0.16
Cx3cl1Unknown, score: 0.14
Cxcl12Unknown, score: 0.48
Frrs1Unknown, score: 0.19
SdprUnknown, score: 0.02
Sec23aUnknown, score: 0.25
SeleUnknown, score: 0.42
Glg1Unknown, score: 0.3
Selenbp1Unknown, score: 0.01
Sema3aUnknown, score: 0.05
Sema3eUnknown, score: 0.09
Sema3fUnknown, score: 0.19
Sema4aUnknown, score: 0.33
Sema4bUnknown, score: 0.36
Sema4cUnknown, score: 0.66
Sema4dUnknown, score: 0.09
Sema4fUnknown, score: 0.09
Sema6aUnknown, score: 0.38
Sema6bUnknown, score: 0.84
Sept8Unknown, score: 0.05
Sepp1Unknown, score: 0.18
Serf1Unknown, score: 0.02
Sfrp4Unknown, score: 0.48
Srsf3Unknown, score: 0.06
SftpbUnknown, score: 0.39
Sgpl1Unknown, score: 0.28
Sh2b1Unknown, score: 0.08
Sh3bp1Unknown, score: 0.19
Sh3gl2Unknown, score: 0.06
20408Unknown, score: 0.13
Sorbs1Unknown, score: 0.07
Shcbp1Unknown, score: 0.02
ShdUnknown, score: 0.25
Shox2Unknown, score: 0.05
Cyfip1Unknown, score: 0.05
PmelUnknown, score: 0.02
Siah1aUnknown, score: 0.38
St6gal1Unknown, score: 0.24
St3gal1Unknown, score: 0.26
St3gal2Unknown, score: 0.22
St6galnac2Unknown, score: 0.12
St6galnac3Unknown, score: 0.14
St6galnac4Unknown, score: 0.14
St3gal5Unknown, score: 0.39
StilUnknown, score: 0.09
Tra2bUnknown, score: 0.17
Sin3aUnknown, score: 0.36
Sin3bUnknown, score: 0.13
Sipa1Unknown, score: 0.18
Six1Unknown, score: 0.15
Six2Unknown, score: 0.04
Six4Unknown, score: 0.17
Vps4bUnknown, score: 0.42
SlaUnknown, score: 0.02
Slc12a2Unknown, score: 0.15
Slc12a3Unknown, score: 0.2
Slc12a7Unknown, score: 0.08
Slc16a7Unknown, score: 0.54
Slc20a1Unknown, score: 0.47
Slc22a1Unknown, score: 0.08
Slc22a3Unknown, score: 0.43
Slc22a5Unknown, score: 0.1
Slc22a12Unknown, score: 0.15
Slc25a17Unknown, score: 0.16
Slc2a2Unknown, score: 0.18
Slc2a3Unknown, score: 0.34
Slc31a1Unknown, score: 0.08
Slc3a1Unknown, score: 0.09
Slc4a1apUnknown, score: 0.11
Slc4a2Unknown, score: 0.42
Slc4a3Unknown, score: 0.55
Slc8a1Unknown, score: 0.26
Slfn4Unknown, score: 0.01
Slit2Unknown, score: 0
Slit3Unknown, score: 0.11
Smarcc1Unknown, score: 0.4
Ighmbp2Unknown, score: 0.04
Smn1Unknown, score: 0.09
Smpd1Unknown, score: 0.5
Smpd2Unknown, score: 0.17
Sstr3Unknown, score: 0
Snai1Unknown, score: 0.36
Snap91Unknown, score: 0.14
Plk2Unknown, score: 0.12
SnnUnknown, score: 0.03
SnrkUnknown, score: 0.22
Eftud2Unknown, score: 0.23
SnrpcUnknown, score: 0.21
Snrnp70Unknown, score: 0.53
Snrpd1Unknown, score: 0.14
SnrpnUnknown, score: 0.17
Soat1Unknown, score: 0.52
Sod1Unknown, score: 0.44
Sod3Unknown, score: 0.09
SonUnknown, score: 0.01
Sorl1Unknown, score: 0.16
Sos2Unknown, score: 0.38
Sox10Unknown, score: 0.15
Sox12Unknown, score: 0.14
Sox15Unknown, score: 0.09
Sox17Unknown, score: 0.39
Sox18Unknown, score: 0.05
Sox2Unknown, score: 0.19
Sox5Unknown, score: 0.09
Sox6Unknown, score: 0.04
Sox7Unknown, score: 0.05
Sp3Unknown, score: 0.09
Serpinb9bUnknown, score: 0.2
Serpinb6bUnknown, score: 0
Serpina3mUnknown, score: 0.02
Serpinb8Unknown, score: 0.28
SpicUnknown, score: 0.02
Spin1Unknown, score: 0.23
Spink4Unknown, score: 0.45
Spint2Unknown, score: 0.24
SpnUnknown, score: 0.14
Sptan1Unknown, score: 0.25
Sptbn2Unknown, score: 0.26
StrbpUnknown, score: 0.15
Spock1Unknown, score: 0.16
Spp1Unknown, score: 0.21
Sprr1aUnknown, score: 0.34
SqleUnknown, score: 0.28
TmieUnknown, score: 0.17
SrcUnknown, score: 0.4
Srebf1Unknown, score: 0.33
SrfUnknown, score: 0.2
SrmUnknown, score: 0.31
SrmsUnknown, score: 0.32
Srpk2Unknown, score: 0.02
SsbUnknown, score: 0.23
Nhp2l1Unknown, score: 0.39
Stat3Unknown, score: 0.11
Stat4Unknown, score: 0.57
Stat5aUnknown, score: 0.19
Stc1Unknown, score: 0.13
Stc2Unknown, score: 0.42
AurkcUnknown, score: 0.25
AurkaUnknown, score: 0.33
Sult1a1Unknown, score: 0
Stx3Unknown, score: 0.09
Sucla2Unknown, score: 0.13
Suclg2Unknown, score: 0.19
Eif1Unknown, score: 0.05
Supt5Unknown, score: 0.22
Supt6Unknown, score: 0.44
Abcc9Unknown, score: 0.37
Surf2Unknown, score: 0.12
Surf4Unknown, score: 0.28
Med22Unknown, score: 0.22
Surf6Unknown, score: 0.08
Syn2Unknown, score: 0.18
Sdc3Unknown, score: 0.41
Syngr1Unknown, score: 0.54
Syngr3Unknown, score: 0.14
SypUnknown, score: 0.09
Taf1aUnknown, score: 0.06
Taf1bUnknown, score: 0.45
Tal1Unknown, score: 0.02
Tal2Unknown, score: 0.15
TankUnknown, score: 0.04
Tap1Unknown, score: 0.24
TapbpUnknown, score: 0.51
Slc6a6Unknown, score: 0.19
Tbrg1Unknown, score: 0.15
Tbrg4Unknown, score: 0.31
Tbx15Unknown, score: 0.44
Tbx6Unknown, score: 0.38
Tbxas1Unknown, score: 0.47
Tcea1Unknown, score: 0.38
Skp1aUnknown, score: 0.19
Tcf12Unknown, score: 0.38
Tcf20Unknown, score: 0.15
Tcf4Unknown, score: 0.2
Tcf7l1Unknown, score: 0.01
Tfap2cUnknown, score: 0.09
Tfcp2Unknown, score: 0.29
Tcf3Unknown, score: 0.5
TfecUnknown, score: 0.19
UbtfUnknown, score: 0.13
Tcn2Unknown, score: 0.1
Tcp1Unknown, score: 0.09
Dynlt1bUnknown, score: 0.55
Phf1Unknown, score: 0.56
Tdgf1Unknown, score: 0.28
Prdx2Unknown, score: 0.09
Tead2Unknown, score: 0.38
Tead3Unknown, score: 0.13
AlyrefUnknown, score: 0.36
TecUnknown, score: 0.08
TectbUnknown, score: 0.24
Tekt1Unknown, score: 0.44
Terf2Unknown, score: 0.09
TesUnknown, score: 0.07
Morf4l1Unknown, score: 0.29
Tex2Unknown, score: 0.03
Tex261Unknown, score: 0.14
Tex264Unknown, score: 0.24
Zfand3Unknown, score: 0.06
Ppp2r5dUnknown, score: 0.13
TfgUnknown, score: 0.1
Tfpi2Unknown, score: 0.46
Tgfb1i1Unknown, score: 0.29
Tgfb3Unknown, score: 0.04
Tgif1Unknown, score: 0.17
Tgm1Unknown, score: 0.37
Tgm2Unknown, score: 0.11
ThpoUnknown, score: 0.23
ThraUnknown, score: 0.2
ThrbUnknown, score: 0.41
Thy1Unknown, score: 0.07
Tiam1Unknown, score: 0.12
Trim28Unknown, score: 0.03
TimelessUnknown, score: 0.18
Timm17aUnknown, score: 0.18
Timm17bUnknown, score: 0.16
Timp3Unknown, score: 0
Tk1Unknown, score: 0.13
TktUnknown, score: 0.1
Tle1Unknown, score: 0.43
Tle3Unknown, score: 0.03
Tll1Unknown, score: 0.6
Tln1Unknown, score: 0.18
Tspan7Unknown, score: 0.15
TncUnknown, score: 0.16
Tnnc2Unknown, score: 0.1
Tnfaip2Unknown, score: 0.02
Tnfrsf11aUnknown, score: 0.29
Tnfrsf17Unknown, score: 0.15
Cd40Unknown, score: 0.26
DeddUnknown, score: 0
Pglyrp1Unknown, score: 0.13
Tnfsf8Unknown, score: 0.1
Tnfsf9Unknown, score: 0.02
TnksUnknown, score: 0.01
Tnni1Unknown, score: 0.24
Tnni2Unknown, score: 0.1
Tnni3Unknown, score: 0.09
Tnnt1Unknown, score: 0.13
Tnnt3Unknown, score: 0.1
Tns1Unknown, score: 0.53
Tmem165Unknown, score: 0.06
TpbgUnknown, score: 0.17
Tpi1Unknown, score: 0.03
Tpp2Unknown, score: 0.03
Tpst1Unknown, score: 0.01
Tpst2Unknown, score: 0.01
Nr2c1Unknown, score: 0.04
Nr2c2Unknown, score: 0
Traf1Unknown, score: 0.27
Traf3Unknown, score: 0.12
Tnfsf10Unknown, score: 0.02
TraipUnknown, score: 0.23
Plscr1Unknown, score: 0.01
TrfUnknown, score: 0.12
TfrcUnknown, score: 0.08
Trip6Unknown, score: 0.16
Trp63Unknown, score: 0.18
Trpc1Unknown, score: 0.56
Trpc2Unknown, score: 0.07
Trpc3Unknown, score: 0.06
Trpc6Unknown, score: 0.35
Ctr9Unknown, score: 0.51
Tsc2Unknown, score: 0.05
TshbUnknown, score: 0.06
TsnUnknown, score: 0.1
Tspyl1Unknown, score: 0.1
Tssk1Unknown, score: 0.18
Tssk2Unknown, score: 0.07
TsksUnknown, score: 0.39
TstUnknown, score: 0.76
Rpl13aUnknown, score: 0.74
Tsta3Unknown, score: 0.42
Ttf1Unknown, score: 0.02
Tgoln1Unknown, score: 0.02
Tuba1aUnknown, score: 0.3
Tuba1bUnknown, score: 0.31
Tuba3aUnknown, score: 0.35
Tuba4aUnknown, score: 0.01
Tuba1cUnknown, score: 0.31
Tubb3Unknown, score: 0.05
Tubb4aUnknown, score: 0.18
Twist1Unknown, score: 0.21
Tnfrsf4Unknown, score: 0.07
TxkUnknown, score: 0.2
TymsUnknown, score: 0.06
Zrsr2Unknown, score: 0.08
U2af2Unknown, score: 0.16
Uba52Unknown, score: 0.35
UbbUnknown, score: 0.03
UbcUnknown, score: 0.04
Ube2mUnknown, score: 0.39
Ube2e3Unknown, score: 0
Uba3Unknown, score: 0.13
Ube2aUnknown, score: 0.14
Ube2bUnknown, score: 0.04
Ube2g2Unknown, score: 0.11
Ube3aUnknown, score: 0.11
Ubr1Unknown, score: 0.13
Uchl1Unknown, score: 0.2
Usp10Unknown, score: 0.24
Usp5Unknown, score: 0.33
Ucp2Unknown, score: 0.17
Ucp3Unknown, score: 0.19
Ufd1lUnknown, score: 0.46
Dpysl3Unknown, score: 0.12
Ulk1Unknown, score: 0.4
UmpsUnknown, score: 0.46
Unc5cUnknown, score: 0.07
UngUnknown, score: 0.18
Upk1bUnknown, score: 0.09
Upk2Unknown, score: 0.33
Upp1Unknown, score: 0.31
UqcrqUnknown, score: 0.37
Uqcrc1Unknown, score: 0.8
UrodUnknown, score: 0.01
UrosUnknown, score: 0.23
Usf1Unknown, score: 0.22
UxtUnknown, score: 0.74
Vamp1Unknown, score: 0.05
Vamp2Unknown, score: 0
Vamp3Unknown, score: 0.18
Vamp8Unknown, score: 0.38
Vav1Unknown, score: 0.38
Vav2Unknown, score: 0.08
Vcam1Unknown, score: 0.17
VclUnknown, score: 0.09
Vdac1Unknown, score: 0.09
Vdac2Unknown, score: 0.24
Vdac3Unknown, score: 0.37
VegfaUnknown, score: 0.36
VegfbUnknown, score: 0.04
VegfcUnknown, score: 0.31
Vezf1Unknown, score: 0.48
Vil1Unknown, score: 0.22
Vipr1Unknown, score: 0.07
VldlrUnknown, score: 0.31
Vnn1Unknown, score: 0.2
Vrk1Unknown, score: 0.3
Trpv2Unknown, score: 0.1
VtnUnknown, score: 0.24
WapUnknown, score: 0.06
WarsUnknown, score: 0
WasUnknown, score: 0.32
Wbp1Unknown, score: 0.12
Wbp5Unknown, score: 0.17
Wdr1Unknown, score: 0.01
Wee1Unknown, score: 0.36
Zmat3Unknown, score: 0.09
Wisp2Unknown, score: 0.03
Wnt10bUnknown, score: 0.4
Wnt2Unknown, score: 0.27
Wnt2bUnknown, score: 0.11
Wnt5aUnknown, score: 0.52
Wnt5bUnknown, score: 0.42
Wt1Unknown, score: 0.23
XdhUnknown, score: 0.05
XlrUnknown, score: 0.23
Xlr3aUnknown, score: 0.04
XpaUnknown, score: 0.31
Ercc5Unknown, score: 0.15
Xrcc5Unknown, score: 0.16
Slc6a20bUnknown, score: 0.35
Yes1Unknown, score: 0.03
YwhaeUnknown, score: 0.08
YwhazUnknown, score: 0.11
Zap70Unknown, score: 0.38
Zfp1Unknown, score: 0.33
Zfp101Unknown, score: 0.06
Zfp11Unknown, score: 0.06
Mkrn3Unknown, score: 0.15
Zbtb14Unknown, score: 0.17
Zfand5Unknown, score: 0.11
Zfp239Unknown, score: 0.12
Zfp26Unknown, score: 0.17
Zfp27Unknown, score: 0.01
Zfp28Unknown, score: 0.24
Zscan2Unknown, score: 0.25
Zfp35Unknown, score: 0.15
Zfp37Unknown, score: 0.36
Zfp41Unknown, score: 0.08
Zfp52Unknown, score: 0.02
Zfp57Unknown, score: 0.01
Zfp62Unknown, score: 0.11
Zfp64Unknown, score: 0.44
Zfp93Unknown, score: 0.11
Zkscan5Unknown, score: 0.01
Zscan12Unknown, score: 0.57
Zfpm1Unknown, score: 0.03
Zhx1Unknown, score: 0.07
Ikzf3Unknown, score: 0.04
Slc30a1Unknown, score: 0.17
ZyxUnknown, score: 0.02
Adamts5Unknown, score: 0.01
Akt3Unknown, score: 0.14
AmfrUnknown, score: 0.2
Bace1Unknown, score: 0.26
C1ql1Unknown, score: 0.06
Capn10Unknown, score: 0.4
Xcr1Unknown, score: 0.06
Cdc6Unknown, score: 0.66
Def6Unknown, score: 0.4
Dlg2Unknown, score: 0.13
Ets1Unknown, score: 0.15
Fbln5Unknown, score: 0.1
Fiz1Unknown, score: 0.19
FybUnknown, score: 0.14
G3bp2Unknown, score: 0.38
Gadd45gUnknown, score: 0.05
Gmcl1Unknown, score: 0.07
Gdf15Unknown, score: 0.59
Hax1Unknown, score: 0.11
Hs2st1Unknown, score: 0.31
RhofUnknown, score: 0.29
Impdh1Unknown, score: 0.31
Sh2b2Unknown, score: 0.14
KelUnknown, score: 0.38
Lamc3Unknown, score: 0.09
Ly6hUnknown, score: 0.34
Map2k5Unknown, score: 0.16
MgllUnknown, score: 0.03
Mid2Unknown, score: 0.19
Neu2Unknown, score: 0.5
Nr0b2Unknown, score: 0.36
Oas1gUnknown, score: 0.08
Tenm3Unknown, score: 0.07
Tenm4Unknown, score: 0.26
Pacsin1Unknown, score: 0.14
Pacsin2Unknown, score: 0.11
Papss2Unknown, score: 0.35
Pebp1Unknown, score: 0.21
Pde10aUnknown, score: 0.25
Slc26a4Unknown, score: 0.42
Eci2Unknown, score: 0.63
Pin1Unknown, score: 0.18
Med24Unknown, score: 0.2
Cib1Unknown, score: 0.16
PrkraUnknown, score: 0.15
Klk7Unknown, score: 0.33
Dazap2Unknown, score: 0.04
Psmc4Unknown, score: 0.12
Psmd13Unknown, score: 0.28
Tiam2Unknown, score: 0.11
IkUnknown, score: 0.12
Rgs7Unknown, score: 0.04
RngttUnknown, score: 0.64
Mrps12Unknown, score: 0.38
Scamp2Unknown, score: 0.07
Scamp3Unknown, score: 0.27
SgcdUnknown, score: 0.32
Sh3bp2Unknown, score: 0.04
Sh3yl1Unknown, score: 0.57
Spry1Unknown, score: 0.32
Spry2Unknown, score: 0.21
Sra1Unknown, score: 0.01
SufuUnknown, score: 0.62
Taf7Unknown, score: 0.19
Taf10Unknown, score: 0.06
Tlk2Unknown, score: 0.07
Tlr2Unknown, score: 0.01
Tnfsf13bUnknown, score: 0.49
Tpra1Unknown, score: 0.09
UbdUnknown, score: 0.12
Ubl3Unknown, score: 0.02
Best1Unknown, score: 0.28
NelfaUnknown, score: 0.1
Xrn2Unknown, score: 0.06
AxlUnknown, score: 0.16
Ceacam1Unknown, score: 0.18
Ceacam2Unknown, score: 0.06
Cetn2Unknown, score: 0.48
Ciao1Unknown, score: 0.36
Clcn6Unknown, score: 0.1
Rfwd2Unknown, score: 0.12
Dapp1Unknown, score: 0.35
Decr2Unknown, score: 0.48
EsrraUnknown, score: 0.39
EsrrgUnknown, score: 0.59
Fgd2Unknown, score: 0.21
Gnpda1Unknown, score: 0.38
Grk6Unknown, score: 0.25
Ifi202bUnknown, score: 0.01
Mapkbp1Unknown, score: 0.62
Lypla2Unknown, score: 0.2
Map2k1Unknown, score: 0.4
Map2k2Unknown, score: 0.26
Map3k1Unknown, score: 0.2
Map3k2Unknown, score: 0.24
Map3k3Unknown, score: 0.16
Map3k4Unknown, score: 0.01
Map4k1Unknown, score: 0.18
Mapk10Unknown, score: 0
Mapk13Unknown, score: 0.28
Mapk14Unknown, score: 0
Mapk8Unknown, score: 0.02
NbeaUnknown, score: 0.09
Nubp1Unknown, score: 0.23
Psg17Unknown, score: 0.23
Psma4Unknown, score: 0.22
Psma6Unknown, score: 0.33
Psma7Unknown, score: 0.36
Psmb2Unknown, score: 0.53
Rbbp9Unknown, score: 0.1
Sema4gUnknown, score: 0.8
Slc27a2Unknown, score: 0.01
Slc27a5Unknown, score: 0.41
Vnn3Unknown, score: 0.28
Zfp260Unknown, score: 0.14
Homer1Unknown, score: 0.14
Homer3Unknown, score: 0.2
Mmp23Unknown, score: 0.69
Ror2Unknown, score: 0.05
Slc27a3Unknown, score: 0.34
Rcn2Unknown, score: 0.07
Cops5Unknown, score: 0.61
Dpysl4Unknown, score: 0.46
B3galt1Unknown, score: 0.34
CenphUnknown, score: 0.05
Clec4a2Unknown, score: 0.01
Cops4Unknown, score: 0.49
Cops6Unknown, score: 0.16
Cops7aUnknown, score: 0.16
Med14Unknown, score: 0.36
Ddx3yUnknown, score: 0.43
Deb1Unknown, score: 0.04
Exo1Unknown, score: 0.78
Gprin1Unknown, score: 0.1
H2afyUnknown, score: 0.09
Map4k4Unknown, score: 0.3
MecrUnknown, score: 0.15
MpripUnknown, score: 0.02
St6galnac5Unknown, score: 0.21
Polr3eUnknown, score: 0.32
Vat1Unknown, score: 0.01
Zw10Unknown, score: 0.38
IslrUnknown, score: 0
Pla2g2fUnknown, score: 0.02
Klrk1Unknown, score: 0.33
PolkUnknown, score: 0.13
Tspan32Unknown, score: 0.1
ErmapUnknown, score: 0.01
SgshUnknown, score: 0.1
G3bp1Unknown, score: 0.3
OmdUnknown, score: 0.13
Etv3Unknown, score: 0.41
AsnsUnknown, score: 0.04
Irf5Unknown, score: 0.04
Ncoa4Unknown, score: 0.3
Sh3d19Unknown, score: 0.22
Tcirg1Unknown, score: 0.09
CadpsUnknown, score: 0.39
Zfp275Unknown, score: 0.06
Xlr4bUnknown, score: 0.03
Trappc3Unknown, score: 0.01
Eif2ak4Unknown, score: 0.47
NrkUnknown, score: 0.24
Azi2Unknown, score: 0.31
Pla2g7Unknown, score: 0.03
Plek2Unknown, score: 0.51
Dok3Unknown, score: 0.02
Zfp354bUnknown, score: 0.01
Nufip1Unknown, score: 0.17
Plekhb1Unknown, score: 0
NbnUnknown, score: 0.43
Insl6Unknown, score: 0.1
Add3Unknown, score: 0.04
Txnl4aUnknown, score: 0.37
Rpl3Unknown, score: 0.12
Tbl2Unknown, score: 0.53
DguokUnknown, score: 0.35
Rps26Unknown, score: 0.08
Sh2d2aUnknown, score: 0.03
Csnk1eUnknown, score: 0.09
Prmt5Unknown, score: 0.06
Tjp3Unknown, score: 0.71
Akr1c13Unknown, score: 0.1
Npas3Unknown, score: 0.34
Sh2d3cUnknown, score: 0.5
Ptdss2Unknown, score: 0.09
Dusp13Unknown, score: 0.18
Mrpl39Unknown, score: 0.37
Mrpl15Unknown, score: 0.46
Mrpl17Unknown, score: 0.51
Mrpl2Unknown, score: 0.17
PdhxUnknown, score: 0.19
Abca8bUnknown, score: 0.14
Abcg3Unknown, score: 0.31
Abcf3Unknown, score: 0.05
Abcf2Unknown, score: 0.08
Abcg5Unknown, score: 0.38
Abcb11Unknown, score: 0.05
SergefUnknown, score: 0.04
Abcc5Unknown, score: 0
Mkln1Unknown, score: 0.35
Abcc6Unknown, score: 0.35
Klra1Unknown, score: 0.38
Atp5lUnknown, score: 0.47
NagpaUnknown, score: 0.02
NelfeUnknown, score: 0.18
1700088E04RikUnknown, score: 0.05
Snf8Unknown, score: 0.09
Lsm2Unknown, score: 0.19
Commd8Unknown, score: 0.12
Zdhhc8Unknown, score: 0.37
Tada1Unknown, score: 0.63
Tango2Unknown, score: 0.26
Dgcr14Unknown, score: 0.07
Spg21Unknown, score: 0.37
Rrp9Unknown, score: 0.28
CherpUnknown, score: 0.12
Eif3bUnknown, score: 0.15
Rsrp1Unknown, score: 0.03
Efhd2Unknown, score: 0.29
Imp4Unknown, score: 0.35
Fam3cUnknown, score: 0.34
Fam21Unknown, score: 0.04
Polr2mUnknown, score: 0.35
Mrpl50Unknown, score: 0.08
D6Wsu163eUnknown, score: 0.14
Ept1Unknown, score: 0.63
Desi1Unknown, score: 0.6
Med10Unknown, score: 0.21
Fam104aUnknown, score: 0.18
Vps25Unknown, score: 0.54
RtcbUnknown, score: 0.13
Trim36Unknown, score: 0.52
D10Wsu102eUnknown, score: 0.25
Tinf2Unknown, score: 0.15
Serp1Unknown, score: 0.35
AponUnknown, score: 0.22
Dcaf11Unknown, score: 0.38
Slco1b2Unknown, score: 0.42
D10Jhu81eUnknown, score: 0.19
Limd1Unknown, score: 0.07
Rabgap1lUnknown, score: 0.03
Bag3Unknown, score: 0.1
Ndrg3Unknown, score: 0.02
Bcar3Unknown, score: 0.02
Tnfrsf19Unknown, score: 0.28
SmtnUnknown, score: 0.05
Mapk12Unknown, score: 0.22
Pde7bUnknown, score: 0.15
Hdgfrp3Unknown, score: 0.22
Zfp292Unknown, score: 0.06
Scd3Unknown, score: 0.27
Fbxw2Unknown, score: 0.24
Rnf17Unknown, score: 0.66
Timm13Unknown, score: 0.32
Timm9Unknown, score: 0.2
Timm8bUnknown, score: 0.05
Timm8a1Unknown, score: 0.56
Timm10Unknown, score: 0.5
Slc39a1Unknown, score: 0.43
Adamts8Unknown, score: 0.42
Fbxw4Unknown, score: 0.2
Fbxw5Unknown, score: 0.82
Fbxl6Unknown, score: 0.26
Kdm2bUnknown, score: 0.65
Fbxl12Unknown, score: 0.25
Angptl3Unknown, score: 0.1
Glrx3Unknown, score: 0.83
Vps26aUnknown, score: 0.11
Tor1aUnknown, score: 0.05
Zfp330Unknown, score: 0.32
Tor2aUnknown, score: 0.32
Tor3aUnknown, score: 0.47
Slc46a2Unknown, score: 0.21
Fgd3Unknown, score: 0.03
Usp25Unknown, score: 0.27
Zfp354cUnknown, score: 0.05
Rnf19aUnknown, score: 0.3
Abt1Unknown, score: 0.02
Bin1Unknown, score: 0.04
Lcmt1Unknown, score: 0.13
Cbx8Unknown, score: 0.41
Cngb3Unknown, score: 0.1
Schip1Unknown, score: 0.54
Pik3cgUnknown, score: 0.25
AassUnknown, score: 0.28
VapaUnknown, score: 0.05
Nox4Unknown, score: 0.4
Thop1Unknown, score: 0.07
Txnrd1Unknown, score: 0.03
E2f6Unknown, score: 0.14
Hspa14Unknown, score: 0.34
TtpaUnknown, score: 0.16
Ercc4Unknown, score: 0.21
Ero1lUnknown, score: 0.07
Tmprss2Unknown, score: 0.03
SacsUnknown, score: 0.13
IcoslUnknown, score: 0.22
Sap30lUnknown, score: 0.25
Fbxw7Unknown, score: 0.09
Fbxo18Unknown, score: 0.05
Fbxo16Unknown, score: 0.2
Fbxo17Unknown, score: 0.04
Tfr2Unknown, score: 0.26
Crim1Unknown, score: 0.32
Pnpla6Unknown, score: 0.01
Dlc1Unknown, score: 0.11
Atp11aUnknown, score: 0.27
Mapk6Unknown, score: 0.17
Nt5cUnknown, score: 0.32
Rgs6Unknown, score: 0.18
Rgs11Unknown, score: 0.32
Lsm4Unknown, score: 0.03
Ppap2cUnknown, score: 0.19
Hs6st2Unknown, score: 0.16
Fbxl8Unknown, score: 0
Acsl4Unknown, score: 0.18
Orc3Unknown, score: 0.31
GneUnknown, score: 0.19
Slc25a13Unknown, score: 0.23
Capn15Unknown, score: 0.23
SpastUnknown, score: 0.25
Park2Unknown, score: 0.36
Tmod2Unknown, score: 0.16
Neu3Unknown, score: 0.24
Exosc9Unknown, score: 0.07
Exosc10Unknown, score: 0.13
Grb14Unknown, score: 0.18
Klrg1Unknown, score: 0.03
Mink1Unknown, score: 0.14
Slc7a8Unknown, score: 0.05
St6galnac6Unknown, score: 0.05
Cpsf2Unknown, score: 0.5
H2afzUnknown, score: 0.3
Tnk2Unknown, score: 0.17
Rgs14Unknown, score: 0.31
Ddah2Unknown, score: 0.1
SrpxUnknown, score: 0.02
Srrm1Unknown, score: 0.17
CtpsUnknown, score: 0.18
Ech1Unknown, score: 0.09
Rundc3aUnknown, score: 0.14
BokUnknown, score: 0.56
Ramp1Unknown, score: 0.06
HnrnpuUnknown, score: 0.01
Clec4fUnknown, score: 0.44
KnstrnUnknown, score: 0.09
Kctd18Unknown, score: 0.89
Ppp1r10Unknown, score: 0.57
Rab11fip5Unknown, score: 0.14
Mfhas1Unknown, score: 0.04
PvrUnknown, score: 0.23
Agpat5Unknown, score: 0.4
Ccdc97Unknown, score: 0.05
Kcnk6Unknown, score: 0.14
Camk1Unknown, score: 0.54
Odf2lUnknown, score: 0.06
RragdUnknown, score: 0.13
Anapc4Unknown, score: 0.36
Commd2Unknown, score: 0.24
Cdca8Unknown, score: 0.12
Wwc2Unknown, score: 0
Sept11Unknown, score: 0.22
Rhpn2Unknown, score: 0.62
Tet1Unknown, score: 0.29
Slc46a1Unknown, score: 0.29
Ctdsp2Unknown, score: 0.42
Coa3Unknown, score: 0.16
CenpoUnknown, score: 0.28
Ddx56Unknown, score: 0.12
Zfp622Unknown, score: 0.26
Nhp2Unknown, score: 0.3
Mettl17Unknown, score: 0.34
Acaa2Unknown, score: 0.33
SgtaUnknown, score: 0.24
Parp8Unknown, score: 0.7
Cdc23Unknown, score: 0.21
Dhrs1Unknown, score: 0.11
Brms1lUnknown, score: 0.56
Cbx7Unknown, score: 0.24
Cdkn2aipnlUnknown, score: 0.14
Esyt2Unknown, score: 0.52
Cisd1Unknown, score: 0.05
Ifi27Unknown, score: 0.01
E2f7Unknown, score: 0.01
Ncaph2Unknown, score: 0
Cd300lgUnknown, score: 0.34
Krr1Unknown, score: 0.13
Zfp410Unknown, score: 0.25
Slc52a2Unknown, score: 0.29
Ccdc43Unknown, score: 0.26
Tspyl2Unknown, score: 0.84
LdhdUnknown, score: 0.17
Rufy3Unknown, score: 0.15
Pnrc2Unknown, score: 0.05
Tmx4Unknown, score: 0.27
Dbndd2Unknown, score: 0.11
Cnot11Unknown, score: 0.18
Sgsm1Unknown, score: 0.17
Lair1Unknown, score: 0.56
Gramd1aUnknown, score: 0.23
Rgs7bpUnknown, score: 0.12
Rbfox3Unknown, score: 0.11
Ahi1Unknown, score: 0.16
Zmiz2Unknown, score: 0.02
Dlg3Unknown, score: 0.03
MybphUnknown, score: 0.03
Nub1Unknown, score: 0.08
BatfUnknown, score: 0.37
Nxf1Unknown, score: 0.27
BanpUnknown, score: 0.03
Vamp4Unknown, score: 0.23
Stx7Unknown, score: 0.07
Mtmr1Unknown, score: 0.53
Gosr1Unknown, score: 0.28
Chst3Unknown, score: 0.11
Mtx2Unknown, score: 0.15
Psmd10Unknown, score: 0.44
Exoc7Unknown, score: 0.16
Stk39Unknown, score: 0.24
Hif3aUnknown, score: 0.11
B4galt2Unknown, score: 0.09
CorinUnknown, score: 0.1
Syt5Unknown, score: 0.15
Ybx2Unknown, score: 0.38
Dctn3Unknown, score: 0.25
Cd164Unknown, score: 0.02
Timm23Unknown, score: 0.11
TslpUnknown, score: 0.06
Nap1l1Unknown, score: 0.23
SnrpaUnknown, score: 0.15
Map3k6Unknown, score: 0.58
ClasrpUnknown, score: 0
NonoUnknown, score: 0.24
Vti1aUnknown, score: 0.21
Fut8Unknown, score: 0.29
BlcapUnknown, score: 0.73
Gria3Unknown, score: 0
Cldn7Unknown, score: 0.29
B3gnt2Unknown, score: 0.15
PorcnUnknown, score: 0.1
Oaz3Unknown, score: 0.32
Ddx39bUnknown, score: 0.18
Tuba8Unknown, score: 0.23
Cntn6Unknown, score: 0.27
Caprin1Unknown, score: 0.25
Slc5a3Unknown, score: 0.41
Celsr2Unknown, score: 0.05
Nphp1Unknown, score: 0.09
Cdkl2Unknown, score: 0.44
Nudt5Unknown, score: 0.2
ClppUnknown, score: 0.29
Slc7a10Unknown, score: 0.24
Rcan2Unknown, score: 0.17
Slc40a1Unknown, score: 0.1
Gpatch11Unknown, score: 0.22
Ddx20Unknown, score: 0.12
Diap2Unknown, score: 0.01
Deaf1Unknown, score: 0.64
Irf7Unknown, score: 0.18
Cks1bUnknown, score: 0.17
Arhgef7Unknown, score: 0.72
Pmm2Unknown, score: 0.01
Actr1aUnknown, score: 0.44
Irf3Unknown, score: 0.13
Pdlim1Unknown, score: 0.1
LsrUnknown, score: 0.4
AcrbpUnknown, score: 0.01
Atxn10Unknown, score: 0.01
Irf6Unknown, score: 0.1
Cyhr1Unknown, score: 0.64
Dnal4Unknown, score: 0.1
Egfl6Unknown, score: 0.29
Copg2Unknown, score: 0.02
Kat6bUnknown, score: 0.11
Cpsf4Unknown, score: 0.01
Gucy1b3Unknown, score: 0.09
Pabpn1Unknown, score: 0.29
Snx3Unknown, score: 0.35
Ccrl2Unknown, score: 0.69
Zfp316Unknown, score: 0.42
Sept1Unknown, score: 0
Golga4Unknown, score: 0.04
Cd160Unknown, score: 0.21
Pcdh7Unknown, score: 0.4
Rpl36Unknown, score: 0.22
B3galt4Unknown, score: 0.3
Arhgef5Unknown, score: 0.09
Elovl2Unknown, score: 0.02
Atf7ipUnknown, score: 0.39
Elp5Unknown, score: 0.53
Rassf5Unknown, score: 0.01
Zfp326Unknown, score: 0.24
Gp9Unknown, score: 0.08
Chst2Unknown, score: 0.01
CpqUnknown, score: 0.21
Mcm3apUnknown, score: 0.48
Hils1Unknown, score: 0.15
Sit1Unknown, score: 0.01
Irgm2Unknown, score: 0.01
Ppt2Unknown, score: 0.46
Slc4a4Unknown, score: 0.36
Ndufa1Unknown, score: 0.27
Ramp2Unknown, score: 0.06
Fmn2Unknown, score: 0.04
Barhl1Unknown, score: 0.04
Dnmt3lUnknown, score: 0.13
Cpsf3Unknown, score: 0.43
MefvUnknown, score: 0.3
Mkrn1Unknown, score: 0.2
Nap1l3Unknown, score: 0.44
Foxo4Unknown, score: 0
Abhd2Unknown, score: 0.43
Pde3aUnknown, score: 0.21
St3gal6Unknown, score: 0.03
Prpf40bUnknown, score: 0.07
Paf1Unknown, score: 0.36
Pqbp1Unknown, score: 0.52
PdgfcUnknown, score: 0.46
Gripap1Unknown, score: 0.01
Ppp1r3fUnknown, score: 0.16
Usp27xUnknown, score: 0.05
Cacna1fUnknown, score: 0.06
Sh3glb1Unknown, score: 0.04
Zfp108Unknown, score: 0.29
CrtamUnknown, score: 0.24
Eif3iUnknown, score: 0.15
Hs3st3b1Unknown, score: 0.21
Dfna5Unknown, score: 0.18
Tfip11Unknown, score: 0.01
Syt8Unknown, score: 0.72
Rp9Unknown, score: 0.1
ApomUnknown, score: 0.34
Dclre1aUnknown, score: 0.19
SfnUnknown, score: 0.35
Eef1b2Unknown, score: 0.27
Bri3Unknown, score: 0.47
Ift20Unknown, score: 0.19
Impa1Unknown, score: 0.05
Cpxm2Unknown, score: 0.19
Nop58Unknown, score: 0.1
Trim3Unknown, score: 0.1
Msh4Unknown, score: 0.05
Alyref2Unknown, score: 0.1
Pgam2Unknown, score: 0.24
Hebp2Unknown, score: 0.65
Slc2a8Unknown, score: 0.32
Tmem131Unknown, score: 0.41
PpieUnknown, score: 0.41
Nprl2Unknown, score: 0.2
Ccnl2Unknown, score: 0.83
Samhd1Unknown, score: 0.28
Cyp39a1Unknown, score: 0.23
Tcerg1Unknown, score: 0.24
Lgals12Unknown, score: 0.37
Pdss1Unknown, score: 0.45
Ubqln1Unknown, score: 0.02
Dnah10Unknown, score: 0.12
Ramp3Unknown, score: 0.05
Ftsj3Unknown, score: 0.2
Mad2l1Unknown, score: 0.11
NagkUnknown, score: 0.04
Bace2Unknown, score: 0.09
Olfm1Unknown, score: 0.58
Prodh2Unknown, score: 0.53
HeylUnknown, score: 0.19
Ddx21Unknown, score: 0.07
Uchl5Unknown, score: 0.08
Gde1Unknown, score: 0.06
RhogUnknown, score: 0.47
Htra1Unknown, score: 0.23
Mpp5Unknown, score: 0.65
Patz1Unknown, score: 0.2
Extl1Unknown, score: 0.12
Zfp386Unknown, score: 0.18
Ccl24Unknown, score: 0.16
Thsd1Unknown, score: 0.11
Hdac7Unknown, score: 0.27
Ak3Unknown, score: 0.35
Cpxm1Unknown, score: 0.11
Stk3Unknown, score: 0.28
Gkap1Unknown, score: 0.56
Fam69bUnknown, score: 0.24
Mrpl37Unknown, score: 0.52
Rassf1Unknown, score: 0.37
StyxUnknown, score: 0.43
Ptpn9Unknown, score: 0.28
Fam60aUnknown, score: 0.04
MycbpUnknown, score: 0.04
Nupr1Unknown, score: 0.12
Zfp113Unknown, score: 0.33
GgcxUnknown, score: 0.35
Anapc7Unknown, score: 0.02
AatfUnknown, score: 0.56
Stam2Unknown, score: 0.65
Abcb9Unknown, score: 0.34
Arl2Unknown, score: 0.28
Pdcd5Unknown, score: 0.03
B4galt5Unknown, score: 0.2
TxnipUnknown, score: 0.11
Eif3cUnknown, score: 0.1
Net1Unknown, score: 0.03
GltpUnknown, score: 0.75
IvdUnknown, score: 0.07
Copz2Unknown, score: 0.03
Acot9Unknown, score: 0.26
Pus1Unknown, score: 0.27
Tmeff2Unknown, score: 0.53
ScocUnknown, score: 0.4
Cyb561d2Unknown, score: 0.08
Tagln3Unknown, score: 0.29
Fzr1Unknown, score: 0.14
Arpc3Unknown, score: 0.2
Arid3bUnknown, score: 0.11
Cyp3a25Unknown, score: 0.17
Stx5aUnknown, score: 0.48
Shoc2Unknown, score: 0.31
Morf4l2Unknown, score: 0.08
Chp1Unknown, score: 0.14
Akap8Unknown, score: 0.26
Ncoa6Unknown, score: 0.06
Trpc4apUnknown, score: 0.28
Nudt3Unknown, score: 0.23
Cbln3Unknown, score: 0.15
AdarUnknown, score: 0.18
Diap3Unknown, score: 0.04
Ppp4cUnknown, score: 0.1
Hbs1lUnknown, score: 0.27
Tubd1Unknown, score: 0.35
Tspan3Unknown, score: 0.45
Adrm1Unknown, score: 0.36
RradUnknown, score: 0.51
Snx1Unknown, score: 0.16
Serinc1Unknown, score: 0.78
Copz1Unknown, score: 0.45
Cyp2d22Unknown, score: 0.02
Ybx3Unknown, score: 0.74
Suclg1Unknown, score: 0.09
Orc6Unknown, score: 0.17
Dynll1Unknown, score: 0.06
Actl6aUnknown, score: 0.07
Sae1Unknown, score: 0.07
Pkp3Unknown, score: 0.21
Kcnip3Unknown, score: 0.37
Mtch1Unknown, score: 0.21
CtsfUnknown, score: 0.11
Pias1Unknown, score: 0.03
Tbk1Unknown, score: 0.37
Slc2a5Unknown, score: 0.28
GabarapUnknown, score: 0.16
IkbkeUnknown, score: 0
Zbtb20Unknown, score: 0.3
Gosr2Unknown, score: 0.01
Ankrd49Unknown, score: 0.62
Srpk3Unknown, score: 0.15
Rapgef4Unknown, score: 0.18
Rnf138Unknown, score: 0.23
Rbms2Unknown, score: 0.27
Slc22a21Unknown, score: 0.24
Mpp6Unknown, score: 0.43
Sept6Unknown, score: 0.03
Sec11aUnknown, score: 0.03
Rgs17Unknown, score: 0.17
Hspb3Unknown, score: 0.16
Pex3Unknown, score: 0.31
IckUnknown, score: 0.24
Kcnd3Unknown, score: 0.02
Sec1Unknown, score: 0
Ube2d2aUnknown, score: 0.53
Pfdn5Unknown, score: 0.15
Rps6ka4Unknown, score: 0.18
Clec4nUnknown, score: 0.07
Adam21Unknown, score: 0.15
Dnase2bUnknown, score: 0
Trim17Unknown, score: 0.23
Clec7aUnknown, score: 0.49
MlycdUnknown, score: 0.18
Lamtor3Unknown, score: 0
CrtapUnknown, score: 0.51
PhaxUnknown, score: 0.23
Cdc42ep4Unknown, score: 0.28
Hist1h1bUnknown, score: 0.3
Zfp111Unknown, score: 0.3
Clcf1Unknown, score: 0.48
Dnajb12Unknown, score: 0.01
Rabgef1Unknown, score: 0.3
Mlst8Unknown, score: 0.15
Sh3bgrlUnknown, score: 0.11
Tulp2Unknown, score: 0.12
Krt71Unknown, score: 0.14
Alg2Unknown, score: 0.61
Mocs1Unknown, score: 0.17
Lat2Unknown, score: 0.24
Nfu1Unknown, score: 0.35
DhodhUnknown, score: 0.35
Mbnl1Unknown, score: 0.17
Ralgapa1Unknown, score: 0.05
Hacl1Unknown, score: 0.06
Scamp5Unknown, score: 0.29
Dnajb2Unknown, score: 0.44
Ccl28Unknown, score: 0.24
Lgi1Unknown, score: 0.09
Trpm5Unknown, score: 0.31
Aldh1a3Unknown, score: 0.05
Slc37a2Unknown, score: 0.17
Cldn9Unknown, score: 0.23
Psg23Unknown, score: 0.05
Zfp109Unknown, score: 0.08
Rnf32Unknown, score: 0.29
Rbms1Unknown, score: 0.39
Gtf2ird1Unknown, score: 0.21
Slc12a5Unknown, score: 0.46
Tbx20Unknown, score: 0.29
Xpo4Unknown, score: 0.14
Tob2Unknown, score: 0.27
Ltb4r2Unknown, score: 0.02
Brd4Unknown, score: 0.53
RetnlaUnknown, score: 0.31
Fzd2Unknown, score: 0.1
Cxcl14Unknown, score: 0.33
Apba3Unknown, score: 0.71
Slc16a8Unknown, score: 0.18
Vsig2Unknown, score: 0.45
BcamUnknown, score: 0.44
Slc25a20Unknown, score: 0.26
IcmtUnknown, score: 0.37
Mrps31Unknown, score: 0.55
NelfcdUnknown, score: 0.27
Wdr46Unknown, score: 0.22
C1dUnknown, score: 0.11
Smpdl3aUnknown, score: 0.19
Terf2ipUnknown, score: 0.59
Jph1Unknown, score: 0.11
Jph3Unknown, score: 0.06
As3mtUnknown, score: 0.39
Cramp1lUnknown, score: 0
Srd5a3Unknown, score: 0.11
Smarce1Unknown, score: 0.26
MogsUnknown, score: 0.06
Psors1c2Unknown, score: 0.44
Atp5j2Unknown, score: 0.27
Sult5a1Unknown, score: 0.13
Dnajc4Unknown, score: 0.05
Zc3h8Unknown, score: 0.18
Kcne3Unknown, score: 0.2
Fbxo3Unknown, score: 0.01
Isg20Unknown, score: 0.24
Stk32cUnknown, score: 0.14
Sec61a2Unknown, score: 0.47
Zfp112Unknown, score: 0.21
JmyUnknown, score: 0.1
Noc3lUnknown, score: 0.25
Fhl5Unknown, score: 0.09
Pglyrp2Unknown, score: 0.09
Ntn4Unknown, score: 0.08
Tbx21Unknown, score: 0.48
Ttyh1Unknown, score: 0.1
Fmnl1Unknown, score: 0.36
Cd200r1Unknown, score: 0.04
RbakUnknown, score: 0.11
Tnip1Unknown, score: 0.01
Bin3Unknown, score: 0.4
CdonUnknown, score: 0.48
Kcne4Unknown, score: 0.09
Spata5Unknown, score: 0.09
TescUnknown, score: 0.21
Eral1Unknown, score: 0.23
Adck2Unknown, score: 0.27
Hacd3Unknown, score: 0.12
Angptl4Unknown, score: 0.06
Cdc42se1Unknown, score: 0.02
Pidd1Unknown, score: 0.23
Tbc1d1Unknown, score: 0.08
Sorcs1Unknown, score: 0.24
Hic2Unknown, score: 0.04
Rqcd1Unknown, score: 0.02
Rad18Unknown, score: 0.13
Vstm2bUnknown, score: 0
Sall1Unknown, score: 0.48
Ppp1r1aUnknown, score: 0.28
Zbp1Unknown, score: 0.23
Pdcd1lg2Unknown, score: 0.03
Slc43a3Unknown, score: 0.37
Bcl11bUnknown, score: 0
Srrm3Unknown, score: 0.15
Trem1Unknown, score: 0.29
Mmp19Unknown, score: 0.12
Cacna1hUnknown, score: 0.18
Efcc1Unknown, score: 0.09
Rnf8Unknown, score: 0.08
Dnaja4Unknown, score: 0.22
Pvrl1Unknown, score: 0.15
Nkain4Unknown, score: 0.57
DexiUnknown, score: 0.23
Hs1bp3Unknown, score: 0.21
Stx6Unknown, score: 0.22
1700123O20RikUnknown, score: 0.02
Eid1Unknown, score: 0.29
Pmaip1Unknown, score: 0.32
Kcnmb4Unknown, score: 0.04
1700027J19RikUnknown, score: 0.15
Rnase4Unknown, score: 0.07
Pex5lUnknown, score: 0.22
HibadhUnknown, score: 0.37
Sumf1Unknown, score: 0
MyotUnknown, score: 0.04
Rps6kb2Unknown, score: 0.04
F12Unknown, score: 0.03
Pole3Unknown, score: 0.27
Wrap73Unknown, score: 0.03
MaeaUnknown, score: 0.42
Pias4Unknown, score: 0.28
Trappc2lUnknown, score: 0.02
Myoz2Unknown, score: 0.17
Ngly1Unknown, score: 0.22
Sh3rf1Unknown, score: 0.27
Hnrnph1Unknown, score: 0.32
Nup160Unknown, score: 0.32
Rab2aUnknown, score: 0.4
Edf1Unknown, score: 0.13
Huwe1Unknown, score: 0.03
NamptUnknown, score: 0.47
Psmd14Unknown, score: 0.07
Ppp2r3cUnknown, score: 0.02
Dact1Unknown, score: 0.15
Pxmp4Unknown, score: 0
Rhot1Unknown, score: 0.41
Stk25Unknown, score: 0.18
CopeUnknown, score: 0.13
Rnf130Unknown, score: 0.33
Arpp19Unknown, score: 0.36
C1galt1c1Unknown, score: 0.45
Nsa2Unknown, score: 0.03
Mettl9Unknown, score: 0.78
Hgh1Unknown, score: 0.25
Zfp191Unknown, score: 0
Bhlhe22Unknown, score: 0.14
FetubUnknown, score: 0.24
MidnUnknown, score: 0.13
Jph2Unknown, score: 0.03
Nek6Unknown, score: 0.01
EmcnUnknown, score: 0.13
Wbp11Unknown, score: 0.28
Chst7Unknown, score: 0.19
FignUnknown, score: 0.24
Cldn15Unknown, score: 0.02
Rbm8aUnknown, score: 0.24
Sap30Unknown, score: 0.48
CenpkUnknown, score: 0.44
Doc2gUnknown, score: 0.01
Iigp1Unknown, score: 0.12
Mrpl38Unknown, score: 0.59
Il21rUnknown, score: 0.71
Qtrt1Unknown, score: 0.58
Acss2Unknown, score: 0.1
FancgUnknown, score: 0.56
Actn4Unknown, score: 0
Gucy1a3Unknown, score: 0.2
Trp53inp1Unknown, score: 0.01
Foxj2Unknown, score: 0.37
Kcnq4Unknown, score: 0.41
Fn3kUnknown, score: 0.2
Taf8Unknown, score: 0.16
Bco1Unknown, score: 0.21
Zfp296Unknown, score: 0.26
Fam129aUnknown, score: 0.28
Dusp10Unknown, score: 0.23
Rbp7Unknown, score: 0.16
Cables1Unknown, score: 0.07
Ube4bUnknown, score: 0.14
Slc29a1Unknown, score: 0.01
Sav1Unknown, score: 0.35
NrgnUnknown, score: 0.07
Yeats4Unknown, score: 0.49
Sv2aUnknown, score: 0.26
Smoc2Unknown, score: 0.1
Smoc1Unknown, score: 0.23
Gpr35Unknown, score: 0.12
ParvgUnknown, score: 0.41
CtszUnknown, score: 0.25
Mllt1Unknown, score: 0.14
Sv2bUnknown, score: 0.27
Rgs18Unknown, score: 0.07
NgbUnknown, score: 0.1
PtgesUnknown, score: 0.09
Stk32bUnknown, score: 0.19
Itm2cUnknown, score: 0.12
Tmub1Unknown, score: 0.46
Gprc5bUnknown, score: 0.15
Gng13Unknown, score: 0.34
Fndc4Unknown, score: 0.09
Dhx38Unknown, score: 0.08
Ms4a4cUnknown, score: 0
Ms4a8aUnknown, score: 0.49
Sirt2Unknown, score: 0.16
Cyp4f14Unknown, score: 0.11
Sp5Unknown, score: 0.49
FcamrUnknown, score: 0.34
Inpp5eUnknown, score: 0
Gpr85Unknown, score: 0.04
Zfp280bUnknown, score: 0.29
Ireb2Unknown, score: 0.37
Fgf23Unknown, score: 0.31
Mrps22Unknown, score: 0.19
Mrps23Unknown, score: 0.02
Mrps10Unknown, score: 0.1
Mrps25Unknown, score: 0.36
Mrps14Unknown, score: 0.71
Keg1Unknown, score: 0.28
DpysUnknown, score: 0.04
Lpin2Unknown, score: 0.46
Tsc1Unknown, score: 0.17
Ap3m2Unknown, score: 0.04
Pes1Unknown, score: 0.16
Cldn12Unknown, score: 0.07
Irak1bp1Unknown, score: 0.05
Nif3l1Unknown, score: 0.07
Arl6ip6Unknown, score: 0.02
Arl6ip4Unknown, score: 0.06
Arl6ip5Unknown, score: 0.17
Lrp10Unknown, score: 0.28
Pmepa1Unknown, score: 0.55
Vps35Unknown, score: 0.09
Slc15a3Unknown, score: 0.04
Xpo7Unknown, score: 0.17
Asb1Unknown, score: 0.18
Dpysl5Unknown, score: 0.48
Asb4Unknown, score: 0.27
Asb3Unknown, score: 0.15
Clstn1Unknown, score: 0.07
Utp3Unknown, score: 0.08
Slc9a3r2Unknown, score: 0.49
ZakUnknown, score: 0.1
Lima1Unknown, score: 0.01
Tmem254aUnknown, score: 0.04
Sostdc1Unknown, score: 0.4
Atp5dUnknown, score: 0.31
Dtd1Unknown, score: 0.29
Ndufb5Unknown, score: 0.42
Mrpl54Unknown, score: 0.84
Emc6Unknown, score: 0.18
RogdiUnknown, score: 0.2
0610009B22RikUnknown, score: 0.2
Ppil2Unknown, score: 0.21
Sf3b6Unknown, score: 0.36
Zfp524Unknown, score: 0.07
Tmem176aUnknown, score: 0.49
Krtcap2Unknown, score: 0.73
Cystm1Unknown, score: 0.3
Gtpbp8Unknown, score: 0.16
Cwc15Unknown, score: 0.17
Chchd3Unknown, score: 0.11
Aurkaip1Unknown, score: 0.95
Tsen34Unknown, score: 0.48
Tmem42Unknown, score: 0.01
Rmnd1Unknown, score: 0.29
Eif3fUnknown, score: 0.19
FopnlUnknown, score: 0.21
Emc3Unknown, score: 0.27
Rmnd5bUnknown, score: 0.24
Ypel3Unknown, score: 0.35
Ndufa3Unknown, score: 0.23
GhitmUnknown, score: 0.16
Lsm7Unknown, score: 0.42
Lamtor4Unknown, score: 0.07
Ube2d3Unknown, score: 0.13
SmpxUnknown, score: 0.33
Ndufa9Unknown, score: 0.22
Tmed3Unknown, score: 0.02
Apoa5Unknown, score: 0.27
Dnajc30Unknown, score: 0.11
Cml1Unknown, score: 0.67
SarnpUnknown, score: 0.11
Tomm6Unknown, score: 0.09
Fkbp11Unknown, score: 0.54
Chchd1Unknown, score: 0.33
Josd2Unknown, score: 0.9
Elof1Unknown, score: 0.21
1110008L16RikUnknown, score: 0.43
Znrd1Unknown, score: 0.3
Wbscr22Unknown, score: 0.19
Ifitm3Unknown, score: 0.32
Eef1e1Unknown, score: 0.33
Necap2Unknown, score: 0.22
Prr13Unknown, score: 0.11
Uqcr10Unknown, score: 0.35
Tmem14cUnknown, score: 0
Ufc1Unknown, score: 0.34
Anapc11Unknown, score: 0.6
Pop4Unknown, score: 0.48
Bola2Unknown, score: 0.38
Tma7Unknown, score: 0.07
GrinaUnknown, score: 0.47
Tomm7Unknown, score: 0.43
Chchd5Unknown, score: 0.79
PglsUnknown, score: 0.21
Med11Unknown, score: 0.36
Nudt14Unknown, score: 0.07
Mustn1Unknown, score: 0.32
Nat9Unknown, score: 0.19
Ubl5Unknown, score: 0.51
Ogfod3Unknown, score: 0.58
P3h4Unknown, score: 0.13
Acer3Unknown, score: 0.3
Ier3ip1Unknown, score: 0.08
Lage3Unknown, score: 0.22
Pithd1Unknown, score: 0.3
Cks2Unknown, score: 0.4
1110059G10RikUnknown, score: 0.12
Cd302Unknown, score: 0.06
1110059E24RikUnknown, score: 0.53
InipUnknown, score: 0.59
Rpl3lUnknown, score: 0.03
Med7Unknown, score: 0.39
RgccUnknown, score: 0.21
Ndufb9Unknown, score: 0.14
Serpinb1aUnknown, score: 0.61
LlphUnknown, score: 0.1
Trappc2Unknown, score: 0.1
Rpl7l1Unknown, score: 0.82
Thoc7Unknown, score: 0.19
Msmo1Unknown, score: 0.2
Atp6v1g2Unknown, score: 0.17
Kcne1lUnknown, score: 0.45
Tmem9Unknown, score: 0.52
Hspbp1Unknown, score: 0.73
Alg5Unknown, score: 0.11
Arfgap3Unknown, score: 0.21
Dimt1Unknown, score: 0.27
Hsbp1l1Unknown, score: 0.22
Ssr2Unknown, score: 0.37
Nicn1Unknown, score: 0.23
Mrps17Unknown, score: 0
Tm4sf20Unknown, score: 0.39
Ing5Unknown, score: 0.31
EappUnknown, score: 0.22
PigylUnknown, score: 0.11
Fam134bUnknown, score: 0.32
Tmem126aUnknown, score: 0.16
Lyrm9Unknown, score: 0.03
1810009A15RikUnknown, score: 0.45
Klf15Unknown, score: 0.32
Tma16Unknown, score: 0.21
Sec11cUnknown, score: 0.3
Atp6v1g1Unknown, score: 0.25
Smim8Unknown, score: 0.35
Mrps21Unknown, score: 0.12
Haus2Unknown, score: 0.85
Rmdn1Unknown, score: 0.22
Fam53cUnknown, score: 0.06
Isoc1Unknown, score: 0.26
MplkipUnknown, score: 0.4
Dpy30Unknown, score: 0.34
Smurf2Unknown, score: 0.09
Wdr61Unknown, score: 0.49
Tmem208Unknown, score: 0.05
1700001K19RikUnknown, score: 0.09
Dnajc5bUnknown, score: 0
Susd3Unknown, score: 0.23
1700020L24RikUnknown, score: 0.39
Aqp11Unknown, score: 0.14
CenppUnknown, score: 0.1
Fam229bUnknown, score: 0.01
Cdrt4Unknown, score: 0.09
Tmem177Unknown, score: 0.1
Atp5slUnknown, score: 0.02
Snw1Unknown, score: 0.37
Knop1Unknown, score: 0.15
OstcUnknown, score: 0.11
Cox20Unknown, score: 0.23
Zfand1Unknown, score: 0.2
Ccdc90bUnknown, score: 0.26
2310022A10RikUnknown, score: 0.15
RtcaUnknown, score: 0.42
Chmp4cUnknown, score: 0.22
Lsm5Unknown, score: 0.38
2310011J03RikUnknown, score: 0.22
Dhrs7Unknown, score: 0.48
Ndufc1Unknown, score: 0.5
Cox14Unknown, score: 0.22
Ppp1r7Unknown, score: 0.59
Nudt8Unknown, score: 0.54
CutcUnknown, score: 0.2
AhnakUnknown, score: 0.09
Sar1bUnknown, score: 0.64
Commd5Unknown, score: 0.05
TsfmUnknown, score: 0.28
Alkbh7Unknown, score: 0.57
Nudt2Unknown, score: 0.23
Rtfdc1Unknown, score: 0.48
Mcts2Unknown, score: 0.49
Mrps15Unknown, score: 0.18
Mterf3Unknown, score: 0.42
Arrdc4Unknown, score: 0.09
Ndufa12Unknown, score: 0.85
Ndufa7Unknown, score: 0.22
Mrpl11Unknown, score: 0.47
Polr2eUnknown, score: 0.26
2410004B18RikUnknown, score: 0.46
Dctpp1Unknown, score: 0.36
CoprsUnknown, score: 0.15
Cyb5bUnknown, score: 0.56
Oxld1Unknown, score: 0
Slc7a6osUnknown, score: 0.19
Uggt2Unknown, score: 0.58
Hamp2Unknown, score: 0
2010012O05RikUnknown, score: 0.09
Cdc26Unknown, score: 0.08
Spc25Unknown, score: 0.18
Tnfaip8l1Unknown, score: 0.21
Cyc1Unknown, score: 0.38
Exosc7Unknown, score: 0.65
Mrpl20Unknown, score: 0.57
PyurfUnknown, score: 0.55
Sys1Unknown, score: 0.26
Taf12Unknown, score: 0.35
Ska1Unknown, score: 0.14
Anp32eUnknown, score: 0.31
Rps23Unknown, score: 0.34
Usmg5Unknown, score: 0.34
Rpl15Unknown, score: 0.59
Exoc2Unknown, score: 0.67
Fam136aUnknown, score: 0.44
Rpl35Unknown, score: 0.47
Mrpl51Unknown, score: 0.03
Prelid1Unknown, score: 0.24
Ndufb3Unknown, score: 0.36
PpdpfUnknown, score: 0.42
Cmss1Unknown, score: 0.33
Zmynd11Unknown, score: 0.49
Psmg3Unknown, score: 0.19
Lamtor1Unknown, score: 0.17
ChtopUnknown, score: 0.54
Tab1Unknown, score: 0.11
Asrgl1Unknown, score: 0
Rwdd1Unknown, score: 0.53
2810004N23RikUnknown, score: 0.55
Timm50Unknown, score: 0.01
Tceanc2Unknown, score: 0.18
Cmc2Unknown, score: 0.17
Nipsnap3bUnknown, score: 0.08
PompUnknown, score: 0.28
Immp1lUnknown, score: 0.25
Sppl2aUnknown, score: 0.03
Metap1dUnknown, score: 0.26
Dzip1Unknown, score: 0.31
Esf1Unknown, score: 0.11
Exosc1Unknown, score: 0.35
Crls1Unknown, score: 0.08
Cmpk1Unknown, score: 0.09
Ube2v1Unknown, score: 0.22
Mad2l1bpUnknown, score: 0.19
Stoml2Unknown, score: 0.06
DiabloUnknown, score: 0.07
Uqcr11Unknown, score: 0.03
Aste1Unknown, score: 0.43
3110001I22RikUnknown, score: 0.53
Rdm1Unknown, score: 0.14
Tmigd1Unknown, score: 0.29
Ms4a4dUnknown, score: 0
Cryzl1Unknown, score: 0.04
Ribc1Unknown, score: 0.2
Gpatch4Unknown, score: 0.31
Snx9Unknown, score: 0.39
Snrnp27Unknown, score: 0.08
Ubr7Unknown, score: 0.14
Spcs2Unknown, score: 0.01
PnisrUnknown, score: 0.18
Cdip1Unknown, score: 0.78
Hiatl1Unknown, score: 0.27
Dph6Unknown, score: 0.3
Mcm8Unknown, score: 0.18
Tsen15Unknown, score: 0.04
Sike1Unknown, score: 0.28
Ctnnbl1Unknown, score: 0.11
RpeUnknown, score: 0.31
Tpgs2Unknown, score: 0.16
NepnUnknown, score: 0.29
Brf2Unknown, score: 0.44
Eef1dUnknown, score: 0.46
Ccdc51Unknown, score: 0.48
Srp72Unknown, score: 0.63
Hspbap1Unknown, score: 0.03
CcnhUnknown, score: 0.34
Tmed7Unknown, score: 0.74
Rae1Unknown, score: 0.17
Pgm1Unknown, score: 0.09
Klhl28Unknown, score: 0.1
Gapvd1Unknown, score: 0.53
Spryd4Unknown, score: 0.3
Rbm4bUnknown, score: 0.18
Dnase1l2Unknown, score: 0.36
Ndufaf3Unknown, score: 0.02
Actr2Unknown, score: 0.07
Ccdc96Unknown, score: 0.3
Lrrk2Unknown, score: 0.15
Hrasls5Unknown, score: 0.06
Ankrd61Unknown, score: 0.1
Kcng4Unknown, score: 0.11
Cfap97Unknown, score: 0.2
Adat2Unknown, score: 0.02
4933411K16RikUnknown, score: 0.05
Gid4Unknown, score: 0.05
GskipUnknown, score: 0.07
Alg14Unknown, score: 0.09
Grtp1Unknown, score: 0.1
Prkrip1Unknown, score: 0
Rbm22Unknown, score: 0.16
Duoxa2Unknown, score: 0.07
Thap2Unknown, score: 0.19
Tmem170Unknown, score: 0.52
Smim7Unknown, score: 0.03
Bcs1lUnknown, score: 0.12
Fbxo25Unknown, score: 0.61
PycardUnknown, score: 0.28
Nacc1Unknown, score: 0.46
Acot13Unknown, score: 0.23
Nxf1Unknown, score: 0.27
EtfdhUnknown, score: 0.28
Ormdl2Unknown, score: 0.01
Mrpl33Unknown, score: 0.33
Ppp1r2Unknown, score: 0.44
Pnpla2Unknown, score: 0.06
Trim35Unknown, score: 0.03
Tcf25Unknown, score: 0.32
Plbd1Unknown, score: 0.2
Dnajc10Unknown, score: 0.14
Clec14aUnknown, score: 0.09
Nhlrc2Unknown, score: 0.07
Hmg20aUnknown, score: 0.02
Mfsd1Unknown, score: 0.1
Zfp869Unknown, score: 0.12
Rsrc1Unknown, score: 0.1
Appbp2Unknown, score: 0.07
AcadsbUnknown, score: 0
Rnf128Unknown, score: 0.23
Lman2Unknown, score: 0.26
Wwp2Unknown, score: 0.24
Fip1l1Unknown, score: 0.08
MtapUnknown, score: 0.56
PccbUnknown, score: 0.48
Plin3Unknown, score: 0.06
Tmem107Unknown, score: 0.54
Nudt16l1Unknown, score: 0.2
Bzw2Unknown, score: 0.27
Kdelr2Unknown, score: 0.29
TonslUnknown, score: 0.41
Myeov2Unknown, score: 0.42
Prpf38bUnknown, score: 0.05
Pbrm1Unknown, score: 0.28
Trmt6Unknown, score: 0.41
Tmem261Unknown, score: 0.15
Sh3d21Unknown, score: 0.33
AagabUnknown, score: 0.12
Shisa5Unknown, score: 0.06
Ddx18Unknown, score: 0.44
Pqlc1Unknown, score: 0.33
SdhaUnknown, score: 0.04
Cdca7Unknown, score: 0.26
Fam188aUnknown, score: 0.47
Golt1bUnknown, score: 0.3
Ctu2Unknown, score: 0.03
Edem3Unknown, score: 0.45
Ssbp2Unknown, score: 0.06
Cdk5rap1Unknown, score: 0.11
Slc25a23Unknown, score: 0.32
Nuf2Unknown, score: 0.11
Luc7lUnknown, score: 0.41
Zfp830Unknown, score: 0.16
Kctd20Unknown, score: 0.26
Smarcd3Unknown, score: 0.14
Cep19Unknown, score: 0.04
Zcchc18Unknown, score: 0.02
Psmd5Unknown, score: 0.25
Uqcrc2Unknown, score: 0.05
Mettl6Unknown, score: 0.47
Oma1Unknown, score: 0.25
Ccdc91Unknown, score: 0.21
Tbc1d2bUnknown, score: 0.01
Actr6Unknown, score: 0.16
Tmem88Unknown, score: 0.64
Use1Unknown, score: 0.52
Rpl11Unknown, score: 0.29
Thap4Unknown, score: 0.18
Dnajb4Unknown, score: 0.33
Mrpl45Unknown, score: 0.09
Pmf1Unknown, score: 0.13
Rbm25Unknown, score: 0.11
Ddx17Unknown, score: 0.07
Syap1Unknown, score: 0
Chd1Unknown, score: 0.29
Ndc80Unknown, score: 0.27
Ola1Unknown, score: 0.11
Slc25a53Unknown, score: 0.43
2610002M06RikUnknown, score: 0.09
Polr3dUnknown, score: 0.6
Mon2Unknown, score: 0.02
Magt1Unknown, score: 0.05
Tex40Unknown, score: 0.72
1700019D03RikUnknown, score: 0.12
Ctnnbip1Unknown, score: 0.64
Cand2Unknown, score: 0.13
Psmc6Unknown, score: 0.46
GatmUnknown, score: 0.31
Trak1Unknown, score: 0.15
MmachcUnknown, score: 0.9
Rps10Unknown, score: 0.02
Mettl21aUnknown, score: 0.33
Ptgr1Unknown, score: 0.06
Timm21Unknown, score: 0.14
Zbtb8osUnknown, score: 0.22
Zfp787Unknown, score: 0.07
NaaaUnknown, score: 0.31
Rpl14Unknown, score: 0.04
BfarUnknown, score: 0.16
Ubap1Unknown, score: 0.28
Tspan31Unknown, score: 0.73
Ube2g1Unknown, score: 0.37
Ndufa6Unknown, score: 0.1
Acbd4Unknown, score: 0.12
Nop56Unknown, score: 0.21
Lrrc40Unknown, score: 0.29
Tomm34Unknown, score: 0.13
Rnf141Unknown, score: 0.44
Smarca2Unknown, score: 0.05
2610301B20RikUnknown, score: 0.16
Sft2d3Unknown, score: 0.16
Ccdc47Unknown, score: 0.52
Lpar6Unknown, score: 0.31
Dram2Unknown, score: 0.06
Zmat5Unknown, score: 0.03
Ccdc25Unknown, score: 0.03
Yipf5Unknown, score: 0.04
Pdzk1ip1Unknown, score: 0.52
Ndufa13Unknown, score: 0.57
Rplp2Unknown, score: 0.36
Zmynd19Unknown, score: 0.13
Spats2lUnknown, score: 0.18
Ccdc77Unknown, score: 0.06
Glod4Unknown, score: 0.02
Utp11lUnknown, score: 0.05
Gatad1Unknown, score: 0.27
Armc10Unknown, score: 0.43
Cmtm6Unknown, score: 0.42
Mboat2Unknown, score: 0.25
L3hypdhUnknown, score: 0.51
Plekho1Unknown, score: 0.03
Rrp15Unknown, score: 0.27
Rnpc3Unknown, score: 0.15
Tmem19Unknown, score: 0.26
Zfp329Unknown, score: 0.05
Tbc1d20Unknown, score: 0.12
Smc6Unknown, score: 0.12
2810474O19RikUnknown, score: 0.41
Marc2Unknown, score: 0.4
Zfp422Unknown, score: 0.14
Zswim6Unknown, score: 0.64
Ndufb8Unknown, score: 0.11
Fam69aUnknown, score: 0.55
Uqcc2Unknown, score: 0.21
Mrpl42Unknown, score: 0.36
Cmtm5Unknown, score: 0.03
Pagr1aUnknown, score: 0.48
Med31Unknown, score: 0.51
Ccdc53Unknown, score: 0.35
Cwc27Unknown, score: 0.05
Srek1ip1Unknown, score: 0.21
Ccdc137Unknown, score: 0.25
Socs4Unknown, score: 0.36
Pbld2Unknown, score: 0.05
NanpUnknown, score: 0.05
1700022I11RikUnknown, score: 0.39
Cstf1Unknown, score: 0.27
Herc4Unknown, score: 0.01
1700093K21RikUnknown, score: 0.19
Jam2Unknown, score: 0.05
Bbs2Unknown, score: 0.32
Brd3Unknown, score: 0.19
Bag4Unknown, score: 0.35
Unc50Unknown, score: 0.3
1110008F13RikUnknown, score: 0.66
Fam132aUnknown, score: 0.33
Rnmtl1Unknown, score: 0
Fundc2Unknown, score: 0.37
4833420G17RikUnknown, score: 0.1
Cxxc5Unknown, score: 0.35
Pdlim7Unknown, score: 0.03
Armcx2Unknown, score: 0.52
Far1Unknown, score: 0.16
Eps8l1Unknown, score: 0.55
Adck3Unknown, score: 0.36
Ccdc127Unknown, score: 0.35
Ssr3Unknown, score: 0.56
MtpapUnknown, score: 0.04
Isoc2bUnknown, score: 0.38
RetsatUnknown, score: 0.2
Map1lc3bUnknown, score: 0.19
IlkapUnknown, score: 0.85
C1qtnf4Unknown, score: 0.29
Plxdc2Unknown, score: 0.11
Ergic2Unknown, score: 0.07
NvlUnknown, score: 0.22
Decr1Unknown, score: 0.11
Poc5Unknown, score: 0.26
Entpd4Unknown, score: 0.19
Gpalpp1Unknown, score: 0.12
Gpatch1Unknown, score: 0.17
Mtfr1Unknown, score: 0.25
Ero1lbUnknown, score: 0.23
Dhx40Unknown, score: 0.16
Calcoco1Unknown, score: 0.12
Ufl1Unknown, score: 0.27
Rnf151Unknown, score: 0.49
Saysd1Unknown, score: 0.48
Tvp23bUnknown, score: 0.07
Tmed9Unknown, score: 0.5
Agpat2Unknown, score: 0.48
Atg12Unknown, score: 0.24
UqcrbUnknown, score: 0.33
Slc39a8Unknown, score: 0.02
Larp6Unknown, score: 0.22
Wdr48Unknown, score: 0.04
Tmem35Unknown, score: 0.19
Tbc1d23Unknown, score: 0.59
Ubxn11Unknown, score: 0.08
Rnf41Unknown, score: 0.15
Tctn3Unknown, score: 0.1
Tespa1Unknown, score: 0.14
Necap1Unknown, score: 0.09
Dusp6Unknown, score: 0.24
Get4Unknown, score: 0.48
FibinUnknown, score: 0.15
AasdhpptUnknown, score: 0.43
Lrp2bpUnknown, score: 0.21
Mxra7Unknown, score: 0.18
Anp32bUnknown, score: 0.53
Ctdp1Unknown, score: 0.04
4930548H24RikUnknown, score: 0.16
Rabl3Unknown, score: 0.29
Dctn4Unknown, score: 0.19
l7Rn6Unknown, score: 0.35
Rpl38Unknown, score: 0.33
Tceb2Unknown, score: 0.05
Trmt112Unknown, score: 0.53
CutaUnknown, score: 0.41
Rpp21Unknown, score: 0.22
SdhbUnknown, score: 0.55
Aldh3b1Unknown, score: 0.02
Ift74Unknown, score: 0.78
Ost4Unknown, score: 0.47
Rnf149Unknown, score: 0.41
1810037I17RikUnknown, score: 0.06
Taf6lUnknown, score: 0.01
Pcnxl4Unknown, score: 0.39
Polr2gUnknown, score: 0.06
Slc25a37Unknown, score: 0.11
Dnajc19Unknown, score: 0.21
Pop1Unknown, score: 0.03
Nudt13Unknown, score: 0.17
Stx17Unknown, score: 0.59
Dph2Unknown, score: 0.04
Mansc1Unknown, score: 0.23
Fbxo32Unknown, score: 0.02
Samsn1Unknown, score: 0.29
EqtnUnknown, score: 0.11
Ddx47Unknown, score: 0.18
PlgrktUnknown, score: 0.24
Slc38a2Unknown, score: 0.16
Prpsap1Unknown, score: 0.37
N6amt1Unknown, score: 0.05
Chd8Unknown, score: 0.25
Ilf2Unknown, score: 0.18
Sfr1Unknown, score: 0.75
Dalrd3Unknown, score: 0.01
Rab39bUnknown, score: 0.08
Snrnp48Unknown, score: 0.38
Dgat2Unknown, score: 0.09
Snx2Unknown, score: 0.2
Rmdn3Unknown, score: 0.19
Sec14l2Unknown, score: 0.49
Atp1b4Unknown, score: 0.29
Nmral1Unknown, score: 0.04
Snap47Unknown, score: 0.32
Idh3aUnknown, score: 0.48
Wdr83Unknown, score: 0.02
Dnajb11Unknown, score: 0.55
Gpsm1Unknown, score: 0.67
Atg3Unknown, score: 0.37
Nop9Unknown, score: 0.17
SncaipUnknown, score: 0.01
1700021F05RikUnknown, score: 0.23
Asprv1Unknown, score: 0.45
Ppp6cUnknown, score: 0.14
2310033P09RikUnknown, score: 0.45
Slc25a11Unknown, score: 0.09
Rgs10Unknown, score: 0.56
Wfdc1Unknown, score: 0.15
Paip2Unknown, score: 0.17
Enoph1Unknown, score: 0.14
MrrfUnknown, score: 0.5
Nsmce4aUnknown, score: 0.53
Mri1Unknown, score: 0.35
Coq10bUnknown, score: 0.11
Tmem33Unknown, score: 0.64
DcxrUnknown, score: 0.28
Uxs1Unknown, score: 0.17
Camsap2Unknown, score: 0.36
Ufm1Unknown, score: 0.03
Rpl4Unknown, score: 0.6
Coa6Unknown, score: 0.05
Fam45aUnknown, score: 0.19
Ccdc80Unknown, score: 0.02
RnmtUnknown, score: 0.17
Pef1Unknown, score: 0.29
Cmc1Unknown, score: 0.61
Mtfp1Unknown, score: 0.49
Sumf2Unknown, score: 0.3
Gipc1Unknown, score: 0.36
Ppm1mUnknown, score: 0.18
1600012H06RikUnknown, score: 0.3
Ppap2bUnknown, score: 0.14
Ube2fUnknown, score: 0.15
Fam32aUnknown, score: 0.06
Myl12bUnknown, score: 0.22
Prorsd1Unknown, score: 0.14
Rps27lUnknown, score: 0.43
Atp5g2Unknown, score: 0.2
Mesdc2Unknown, score: 0.25
Spata6Unknown, score: 0.26
NifkUnknown, score: 0
U2surpUnknown, score: 0.03
Mphosph10Unknown, score: 0.11
67978Unknown, score: 0.17
Nudt12Unknown, score: 0.35
Mrps11Unknown, score: 0.26
Srsf6Unknown, score: 0.23
Ddx59Unknown, score: 0.48
1110004E09RikUnknown, score: 0.42
BambiUnknown, score: 0.39
ZwilchUnknown, score: 0.21
MurcUnknown, score: 0.37
Col4a3bpUnknown, score: 0.3
Apopt1Unknown, score: 0.57
2810417H13RikUnknown, score: 0.2
Tmem178Unknown, score: 0.33
Rnf146Unknown, score: 0.29
Emc4Unknown, score: 0.06
Fam122aUnknown, score: 0.53
Rbm42Unknown, score: 0.23
Zfp706Unknown, score: 0.14
Chid1Unknown, score: 0.13
NmbUnknown, score: 0.57
Zfp593Unknown, score: 0.52
Mid1ip1Unknown, score: 0.56
Chac2Unknown, score: 0.1
2700060E02RikUnknown, score: 0.11
2700062C07RikUnknown, score: 0.46
MpndUnknown, score: 0.34
Akirin1Unknown, score: 0.07
Nutf2Unknown, score: 0.43
Serpina12Unknown, score: 0.13
Chd1lUnknown, score: 0.3
Tm9sf2Unknown, score: 0.13
Lurap1Unknown, score: 0.31
Gltscr2Unknown, score: 0.73
Pdcd2lUnknown, score: 0.3
Dusp19Unknown, score: 0.14
DcakdUnknown, score: 0.05
Arpc4Unknown, score: 0.11
Ociad1Unknown, score: 0.26
Dynll2Unknown, score: 0.26
Fam92aUnknown, score: 0.1
Nt5c3bUnknown, score: 0.27
Cntd1Unknown, score: 0.13
Sdccag3Unknown, score: 0.12
9430016H08RikUnknown, score: 0.08
ApoolUnknown, score: 0.44
Cep70Unknown, score: 0.51
GcshUnknown, score: 0.1
Upf3bUnknown, score: 0.1
Eif3hUnknown, score: 0.35
Rpl39lUnknown, score: 0.2
Cgnl1Unknown, score: 0.35
Bcas2Unknown, score: 0.01
DenrUnknown, score: 0.14
Fam135aUnknown, score: 0.01
SympkUnknown, score: 0.37
Leprotl1Unknown, score: 0.13
Hsbp1Unknown, score: 0.32
Ndufc2Unknown, score: 0.26
Ccdc34Unknown, score: 0.14
Urm1Unknown, score: 0.45
Rnaseh2cUnknown, score: 0.32
Tmbim4Unknown, score: 0.4
Fam98bUnknown, score: 0.31
Efcab2Unknown, score: 0.28
AI846148Unknown, score: 0.02
MturnUnknown, score: 0.1
Rpa3Unknown, score: 0.66
Fam195aUnknown, score: 0.17
Fam96aUnknown, score: 0.19
Babam1Unknown, score: 0.16
Ift80Unknown, score: 0
Agpat4Unknown, score: 0.06
PdhbUnknown, score: 0.01
Rbm28Unknown, score: 0.21
Pomgnt1Unknown, score: 0.24
Rpa1Unknown, score: 0.5
2310057M21RikUnknown, score: 0.08
Stt3bUnknown, score: 0.22
Mfsd10Unknown, score: 0.25
Ncapd2Unknown, score: 0
Zmym1Unknown, score: 0.57
Lypd2Unknown, score: 0.61
Gstm7Unknown, score: 0.01
ApooUnknown, score: 0.23
Aph1cUnknown, score: 0.3
Nudt22Unknown, score: 0.33
Rab13Unknown, score: 0
Sdhaf1Unknown, score: 0.31
Crip2Unknown, score: 0.12
Ndufb10Unknown, score: 0.52
Sirt5Unknown, score: 0.73
0610011F06RikUnknown, score: 0.4
Ndufs3Unknown, score: 0.73
Mul1Unknown, score: 0.61
0610030E20RikUnknown, score: 0.09
Rab14Unknown, score: 0.77
Tmem129Unknown, score: 0.11
Pbld2Unknown, score: 0.1
Ndufa8Unknown, score: 0.16
Ciz1Unknown, score: 0.27
Tlcd1Unknown, score: 0.18
Mogat1Unknown, score: 0.14
Ccdc163Unknown, score: 0.26
G6pc3Unknown, score: 0.34
Nrn1Unknown, score: 0.38
Ankrd13aUnknown, score: 0.1
Lmbrd1Unknown, score: 0.03
Steap3Unknown, score: 0.45
Fbxl15Unknown, score: 0.09
Rpl34Unknown, score: 0.15
Dusp23Unknown, score: 0.27
RragaUnknown, score: 0.17
Gpihbp1Unknown, score: 0.43
Ppp1r14aUnknown, score: 0.28
Dhrs7cUnknown, score: 0.07
Mob1bUnknown, score: 0.26
Rmnd5aUnknown, score: 0.35
Phf5aUnknown, score: 0.41
Tmem140Unknown, score: 0.38
Ndufaf4Unknown, score: 0.29
Arel1Unknown, score: 0.09
Tspan11Unknown, score: 0.44
Nsmce2Unknown, score: 0.13
Vps51Unknown, score: 0.4
Ptx4Unknown, score: 0.31
Ints1Unknown, score: 0.16
Micu2Unknown, score: 0.47
Myadml2Unknown, score: 0.03
Eml1Unknown, score: 0.63
Fam189bUnknown, score: 0.43
Fam96bUnknown, score: 0.09
Wipf2Unknown, score: 0
Mphosph6Unknown, score: 0.09
Mrpl13Unknown, score: 0.3
Tmem109Unknown, score: 0.17
Sgol2aUnknown, score: 0.14
TefmUnknown, score: 0
Col6a4Unknown, score: 0.14
CebpzosUnknown, score: 0.62
Ankra2Unknown, score: 0.65
Pdrg1Unknown, score: 0.5
Nufip2Unknown, score: 0.06
Mrps18aUnknown, score: 0.15
CalyUnknown, score: 0.12
Cgref1Unknown, score: 0.38
Ict1Unknown, score: 0.07
Lamtor5Unknown, score: 0.02
Cthrc1Unknown, score: 0.12
MocosUnknown, score: 0.01
Syf2Unknown, score: 0.08
PmvkUnknown, score: 0.04
Ppm1fUnknown, score: 0.01
SerhlUnknown, score: 0.51
Ube2cUnknown, score: 0.31
Gdpd3Unknown, score: 0.38
1110012L19RikUnknown, score: 0.04
Cfap57Unknown, score: 0.16
Elac2Unknown, score: 0.31
Fbxw9Unknown, score: 0.24
Myct1Unknown, score: 0.8
Fahd1Unknown, score: 0.44
Tmem216Unknown, score: 0.52
Abhd14aUnknown, score: 0.26
Tab2Unknown, score: 0.26
Fam198bUnknown, score: 0.03
Scgb3a1Unknown, score: 0.31
Trpm4Unknown, score: 0.38
Fam172aUnknown, score: 0.14
Smtnl1Unknown, score: 0.69
Fitm1Unknown, score: 0.63
Hddc3Unknown, score: 0.29
Rabl2Unknown, score: 0.25
Cilp2Unknown, score: 0.07
Ifitm1Unknown, score: 0.56
Arl8aUnknown, score: 0.1
1110032F04RikUnknown, score: 0.08
Trim37Unknown, score: 0.65
Dus1lUnknown, score: 0.3
Smek1Unknown, score: 0.42
Tyw5Unknown, score: 0.1
Angel1Unknown, score: 0.27
Acss1Unknown, score: 0.09
AnlnUnknown, score: 0.21
Zfp740Unknown, score: 0.18
Rreb1Unknown, score: 0.25
MybphlUnknown, score: 0.01
Cgrrf1Unknown, score: 0.47
Abhd11Unknown, score: 0.37
Synpo2lUnknown, score: 0.22
Cdhr3Unknown, score: 0.38
Ms4a6dUnknown, score: 0.2
Taf11Unknown, score: 0.16
Tmem53Unknown, score: 0.59
Ubr3Unknown, score: 0.33
NexnUnknown, score: 0.08
Ppil1Unknown, score: 0.05
Ddi2Unknown, score: 0.23
Pdcl3Unknown, score: 0.52
Mrpl52Unknown, score: 0
Foxk2Unknown, score: 0.26
Lrrn4clUnknown, score: 0.11
Asb11Unknown, score: 0.07
1190002N15RikUnknown, score: 0.46
Arv1Unknown, score: 0.17
Rnf122Unknown, score: 0.26
Tmcc2Unknown, score: 0.58
Xrcc6bp1Unknown, score: 0.22
Maf1Unknown, score: 0.01
Prpf6Unknown, score: 0.23
Gkn3Unknown, score: 0.02
Ubac2Unknown, score: 0.07
Cd177Unknown, score: 0.12
Abhd13Unknown, score: 0.4
Zfp467Unknown, score: 0.47
Pygo2Unknown, score: 0.36
Cdkal1Unknown, score: 0.66
Hint2Unknown, score: 0.28
1190005I06RikUnknown, score: 0.7
1110065P20RikUnknown, score: 0.36
Dnaic1Unknown, score: 0.18
Rpap1Unknown, score: 0.1
Ubap2Unknown, score: 0.26
Ptcd2Unknown, score: 0.2
Mospd3Unknown, score: 0.35
Smim11Unknown, score: 0.53
Aspscr1Unknown, score: 0.61
Chmp2bUnknown, score: 0.26
Tmco1Unknown, score: 0.17
Chst8Unknown, score: 0.02
Fam216aUnknown, score: 0.53
1500012F01RikUnknown, score: 0.08
Fam57bUnknown, score: 0.12
Srrm4Unknown, score: 0.33
Paqr6Unknown, score: 0.47
Phkg2Unknown, score: 0.04
Ctc1Unknown, score: 0.32
NgdnUnknown, score: 0.34
Cdan1Unknown, score: 0.62
Eif1bUnknown, score: 0.02
Med27Unknown, score: 0.54
Wdr53Unknown, score: 0.37
Snrpa1Unknown, score: 0.13
Prpf31Unknown, score: 0.22
Ssu72Unknown, score: 0.35
Cab39lUnknown, score: 0.36
Thap7Unknown, score: 0.31
Anapc13Unknown, score: 0.08
Prrt2Unknown, score: 0.24
Spcs1Unknown, score: 0.38
Zfp707Unknown, score: 0.27
Mitd1Unknown, score: 0.07
Zdhhc3Unknown, score: 0.17
Tmem258Unknown, score: 0.11
Isca1Unknown, score: 0.09
Slc30a5Unknown, score: 0.49
1810013L24RikUnknown, score: 0.19
FuomUnknown, score: 0.13
Kdf1Unknown, score: 0.37
Psmd11Unknown, score: 0.38
Zcchc9Unknown, score: 0.14
Oxa1lUnknown, score: 0.28
Vps26bUnknown, score: 0.71
Trim15Unknown, score: 0.38
YdjcUnknown, score: 0
March5Unknown, score: 0.33
Alkbh3Unknown, score: 0.15
Ubr4Unknown, score: 0.11
1810022K09RikUnknown, score: 0.46
Vstm5Unknown, score: 0.24
2200002J24RikUnknown, score: 0.07
Snx4Unknown, score: 0.28
LzicUnknown, score: 0.06
1810030O07RikUnknown, score: 0.41
Comtd1Unknown, score: 0.38
Rhebl1Unknown, score: 0.2
ManbalUnknown, score: 0.03
Sec31aUnknown, score: 0.13
Mrpl44Unknown, score: 0.13
Cd209bUnknown, score: 0.59
Faim3Unknown, score: 0.5
Cnppd1Unknown, score: 0
Snx5Unknown, score: 0.08
Dyrk2Unknown, score: 0.05
Dtwd1Unknown, score: 0.51
DymUnknown, score: 0.02
Pdia2Unknown, score: 0.11
Srsf11Unknown, score: 0.46
Sat2Unknown, score: 0.03
Plekha4Unknown, score: 0.05
Ddah1Unknown, score: 0.38
Zfp746Unknown, score: 0.28
Qrich1Unknown, score: 0.18
Gtpbp4Unknown, score: 0.55
Polr2dUnknown, score: 0.23
Elf2Unknown, score: 0.43
Ing2Unknown, score: 0.14
Rfc3Unknown, score: 0.26
Rhobtb1Unknown, score: 0.11
TesclUnknown, score: 0.28
Pxt1Unknown, score: 0.3
1700007K13RikUnknown, score: 0.5
Necab1Unknown, score: 0.25
Smco2Unknown, score: 0.32
Mocs3Unknown, score: 0.41
C8gUnknown, score: 0.04
Plac8l1Unknown, score: 0.01
Dnajc17Unknown, score: 0.32
Dennd6bUnknown, score: 0.16
1700023F06RikUnknown, score: 0.2
Tmem127Unknown, score: 0.81
2300009A05RikUnknown, score: 0.28
Nup35Unknown, score: 0.09
Ndufaf5Unknown, score: 0.52
Zfp932Unknown, score: 0
Rwdd2aUnknown, score: 0.11
EsamUnknown, score: 0.15
Avpi1Unknown, score: 0.18
Ten1Unknown, score: 0.47
Trnp1Unknown, score: 0.4
Nkpd1Unknown, score: 0.8
2310009B15RikUnknown, score: 0.25
Bst2Unknown, score: 0.22
2310022B05RikUnknown, score: 0.34
Klhdc2Unknown, score: 0.56
Cdk13Unknown, score: 0.03
Vkorc1l1Unknown, score: 0.67
Mfsd3Unknown, score: 0.26
HilpdaUnknown, score: 0.7
CmblUnknown, score: 0.29
Smco1Unknown, score: 0.13
RhouUnknown, score: 0.03
Plekhm2Unknown, score: 0.08
Tnfsf13Unknown, score: 0.43
Gpx8Unknown, score: 0.4
Afg3l2Unknown, score: 0.19
MtfmtUnknown, score: 0.54
Sec24dUnknown, score: 0.28
Kansl2Unknown, score: 0.04
Pitrm1Unknown, score: 0.25
Arhgef12Unknown, score: 0.25
ClyblUnknown, score: 0.25
Dapk1Unknown, score: 0.3
Exosc8Unknown, score: 0.08
Fam83gUnknown, score: 0
Wdr20Unknown, score: 0.05
MlipUnknown, score: 0.38
PirUnknown, score: 0.05
Tmbim1Unknown, score: 0.4
2310061I04RikUnknown, score: 0.37
Ddx51Unknown, score: 0.63
Psmg4Unknown, score: 0.12
Ccdc115Unknown, score: 0.77
Tmem52Unknown, score: 0.08
Txndc15Unknown, score: 0.21
PxdnUnknown, score: 0.22
Emc10Unknown, score: 0.46
Aarsd1Unknown, score: 0.34
Hddc2Unknown, score: 0.05
IqcgUnknown, score: 0.15
Pin4Unknown, score: 0.33
IpmkUnknown, score: 0.26
Nkiras1Unknown, score: 0.1
RpainUnknown, score: 0.41
Rnaseh2aUnknown, score: 0.05
Smyd3Unknown, score: 0.1
Usp46Unknown, score: 0.18
Nup37Unknown, score: 0.13
TtlUnknown, score: 0.03
Dph5Unknown, score: 0.27
Tm2d2Unknown, score: 0.21
Casz1Unknown, score: 0.15
Pold4Unknown, score: 0.53
Zswim7Unknown, score: 0.16
Aldh16a1Unknown, score: 0.02
Zfp511Unknown, score: 0.26
1600015I10RikUnknown, score: 0.09
Tnfaip8l2Unknown, score: 0.39
Bdh2Unknown, score: 0.22
Ms4a6bUnknown, score: 0.06
1500009L16RikUnknown, score: 0.07
TprkbUnknown, score: 0.21
Med30Unknown, score: 0.02
Med6Unknown, score: 0.45
Cox11Unknown, score: 0.75
Tmem147Unknown, score: 0.05
Trim32Unknown, score: 0.18
Clec4b1Unknown, score: 0.12
Krtcap3Unknown, score: 0.34
Mzb1Unknown, score: 0.08
Fyttd1Unknown, score: 0.13
Glod5Unknown, score: 0.38
Pla2g12bUnknown, score: 0.01
Pcgf1Unknown, score: 0.29
Wnk4Unknown, score: 0.18
Tcf23Unknown, score: 0.29
Eif1adUnknown, score: 0.33
Polr3glUnknown, score: 0.3
Ppp1r35Unknown, score: 0.82
Ndufa11Unknown, score: 0.51
Thap3Unknown, score: 0.24
AunipUnknown, score: 0.39
Zfp219Unknown, score: 0.16
Coa7Unknown, score: 0.18
2010107G23RikUnknown, score: 0.06
Mfsd11Unknown, score: 0.07
Mrto4Unknown, score: 0.1
Rasip1Unknown, score: 0.08
Slc25a32Unknown, score: 0.13
Rab3bUnknown, score: 0.24
Nabp2Unknown, score: 0.38
Vrk2Unknown, score: 0.18
AgkUnknown, score: 0.2
Apitd1Unknown, score: 0.04
Exoc1Unknown, score: 0.09
2810021J22RikUnknown, score: 0.66
Fars2Unknown, score: 0.35
Ptcd3Unknown, score: 0.45
Cdc16Unknown, score: 0.71
Mettl18Unknown, score: 0.02
Tmem30aUnknown, score: 0.03
Ace2Unknown, score: 0.29
Cep85Unknown, score: 0.45
Ino80bUnknown, score: 0.04
Mcm10Unknown, score: 0.37
Acot7Unknown, score: 0.1
Dopey2Unknown, score: 0.16
Cmtm8Unknown, score: 0.14
Trnt1Unknown, score: 0.02
Prpf4Unknown, score: 0.02
Ccdc89Unknown, score: 0.21
Degs2Unknown, score: 0.01
Lysmd2Unknown, score: 0.47
MetrnUnknown, score: 0.33
Smc4Unknown, score: 0.46
Cyp4f16Unknown, score: 0.12
Znhit1Unknown, score: 0.1
Ifi35Unknown, score: 0.48
SrrdUnknown, score: 0.39
Dpf3Unknown, score: 0.34
Lrch3Unknown, score: 0.19
Mettl7a1Unknown, score: 0.29
Vps36Unknown, score: 0.18
Abhd17cUnknown, score: 0.31
Fam162aUnknown, score: 0.15
Cd209gUnknown, score: 0.08
Taco1Unknown, score: 0.12
Med23Unknown, score: 0.25
NarsUnknown, score: 0.08
Ppil3Unknown, score: 0.14
Zfp619Unknown, score: 0.04
Gorasp2Unknown, score: 0.27
Poc1aUnknown, score: 0.02
Rnf168Unknown, score: 0.12
Gtf3c5Unknown, score: 0.34
Psmd1Unknown, score: 0.2
Dazap1Unknown, score: 0.43
2010107E04RikUnknown, score: 0.14
Chp2Unknown, score: 0.36
Rpf1Unknown, score: 0.36
Afap1Unknown, score: 0.22
Rnf126Unknown, score: 0.47
Gcc2Unknown, score: 0.22
FuzUnknown, score: 0.11
Plscr3Unknown, score: 0.37
CactinUnknown, score: 0.45
Rabep2Unknown, score: 0.03
Hdac8Unknown, score: 0.48
Ndufab1Unknown, score: 0.09
Cd3eapUnknown, score: 0.42
IydUnknown, score: 0.51
Secisbp2lUnknown, score: 0.07
Gprc5cUnknown, score: 0.26
Steap1Unknown, score: 0.62
Lman1Unknown, score: 0.02
Derl3Unknown, score: 0.06
Mospd1Unknown, score: 0.1
Tecpr1Unknown, score: 0.47
Kctd2Unknown, score: 0.21
Ttc9cUnknown, score: 0.14
Asb12Unknown, score: 0.56
KptnUnknown, score: 0
Asnsd1Unknown, score: 0.26
Ints2Unknown, score: 0.16
Tekt5Unknown, score: 0.13
Rufy2Unknown, score: 0.02
DraxinUnknown, score: 0.27
Cd248Unknown, score: 0.15
CenplUnknown, score: 0.54
2610318N02RikUnknown, score: 0.25
Crtc3Unknown, score: 0.13
Ckap2lUnknown, score: 0.14
Atad2Unknown, score: 0.18
Atp6ap2Unknown, score: 0.09
Arhgap17Unknown, score: 0.27
BbxUnknown, score: 0.05
Rnf167Unknown, score: 0.12
StambpUnknown, score: 0.05
Zdhhc2Unknown, score: 0.11
Tmtc4Unknown, score: 0.24
Lrrc56Unknown, score: 0.4
Cpne3Unknown, score: 0.14
Ipo5Unknown, score: 0.15
Tbccd1Unknown, score: 0.18
CpmUnknown, score: 0.33
Zc3h11aUnknown, score: 0.05
Pak4Unknown, score: 0.04
Ssfa2Unknown, score: 0.01
EcdUnknown, score: 0.43
Fbxo33Unknown, score: 0.45
Tmem230Unknown, score: 0.13
Ube2v2Unknown, score: 0.5
Oip5Unknown, score: 0.03
Tmem144Unknown, score: 0.05
Sik3Unknown, score: 0.25
Gulp1Unknown, score: 0
Utp20Unknown, score: 0.19
3830403N18RikUnknown, score: 0.14
Nup205Unknown, score: 0.32
MedagUnknown, score: 0.09
Hmha1Unknown, score: 0.38
Nos1apUnknown, score: 0.18
Tspan2Unknown, score: 0.08
Gpr173Unknown, score: 0.52
Dennd1cUnknown, score: 0.48
Ubr5Unknown, score: 0.59
Hars2Unknown, score: 0.1
Pwwp2aUnknown, score: 0.36
Pgrmc2Unknown, score: 0.45
Krt25Unknown, score: 0.02
Trak2Unknown, score: 0.15
P2ry12Unknown, score: 0.35
Cnbd2Unknown, score: 0.09
Armc3Unknown, score: 0.14
Ccdc81Unknown, score: 0.41
Ints10Unknown, score: 0.22
Ttll7Unknown, score: 0.07
70893Unknown, score: 0.07
Fam71dUnknown, score: 0.28
Lpcat2bUnknown, score: 0.01
Spata1Unknown, score: 0.26
4931429I11RikUnknown, score: 0.23
Phf6Unknown, score: 0.22
Naa40Unknown, score: 0.02
Prss41Unknown, score: 0.05
Spats1Unknown, score: 0.37
Pcgf6Unknown, score: 0.22
4933405L10RikUnknown, score: 0.19
Cdkl1Unknown, score: 0.24
Sh2d6Unknown, score: 0
Zfp689Unknown, score: 0.07
CabyrUnknown, score: 0.11
Rfx4Unknown, score: 0
Scara5Unknown, score: 0.38
Golga7bUnknown, score: 0.06
Mier1Unknown, score: 0.09
4933413G19RikUnknown, score: 0.12
Eri2Unknown, score: 0.21
NipblUnknown, score: 0.11
Cage1Unknown, score: 0.11
Sdhaf3Unknown, score: 0.11
Osbpl7Unknown, score: 0.1
Lrrfip2Unknown, score: 0.02
Slc29a3Unknown, score: 0.29
Treml1Unknown, score: 0.09
Col24a1Unknown, score: 0.19
Aifm2Unknown, score: 0.02
Pdss2Unknown, score: 0.08
Foxn3Unknown, score: 0.14
Fmnl2Unknown, score: 0.1
Flrt3Unknown, score: 0.22
WrbUnknown, score: 0.32
Tmem80Unknown, score: 0.19
Ankrd40Unknown, score: 0.06
Ptk7Unknown, score: 0.63
Usp19Unknown, score: 0.42
9030624J02RikUnknown, score: 0.19
Cyp2u1Unknown, score: 0.1
Pds5aUnknown, score: 0.21
Ggt6Unknown, score: 0.35
Fam217bUnknown, score: 0.28
AfmidUnknown, score: 0.34
Mcm9Unknown, score: 0.29
Ifih1Unknown, score: 0.05
Zfp251Unknown, score: 0.15
Snx20Unknown, score: 0.1
TraddUnknown, score: 0.26
9130011E15RikUnknown, score: 0.16
Tmem248Unknown, score: 0.35
Acy3Unknown, score: 0.4
BroxUnknown, score: 0.28
Atp5hUnknown, score: 0.46
Galnt14Unknown, score: 0.39
Esm1Unknown, score: 0.18
Colec11Unknown, score: 0.04
Arhgef3Unknown, score: 0.51
Slc46a3Unknown, score: 0.14
Ubiad1Unknown, score: 0.09
Lrrcc1Unknown, score: 0.48
Dhx35Unknown, score: 0.37
Telo2Unknown, score: 0.11
Fam13cUnknown, score: 0.14
CicUnknown, score: 0.75
Dhx34Unknown, score: 0.37
Aox3Unknown, score: 0.07
Smug1Unknown, score: 0.38
Stk11ipUnknown, score: 0.34
Rgs12Unknown, score: 0.15
Susd2Unknown, score: 0.22
Ulk3Unknown, score: 0.28
Cul2Unknown, score: 0.45
R3hdm2Unknown, score: 0.03
DhdhUnknown, score: 0.49
EtnpplUnknown, score: 0.03
Amdhd1Unknown, score: 0.18
C2cd2lUnknown, score: 0.15
Klhdc3Unknown, score: 0.38
Raver1Unknown, score: 0.46
Tysnd1Unknown, score: 0.16
VwceUnknown, score: 0.2
Ap2b1Unknown, score: 0.43
Ugt2b1Unknown, score: 0.01
1300017J02RikUnknown, score: 0.37
Klhl5Unknown, score: 0.03
March8Unknown, score: 0.4
PdgfdUnknown, score: 0.21
Anxa9Unknown, score: 0.01
Pitpnc1Unknown, score: 0.28
Plekhf2Unknown, score: 0.13
Nup93Unknown, score: 0.12
Tars2Unknown, score: 0.03
Rnf180Unknown, score: 0.14
PdiltUnknown, score: 0
CslUnknown, score: 0.2
Shcbp1lUnknown, score: 0.42
1700003E16RikUnknown, score: 0.03
Tekt4Unknown, score: 0.03
Nupl1Unknown, score: 0.15
Syce2Unknown, score: 0.32
Fam83dUnknown, score: 0.38
ApmapUnknown, score: 0.79
Coq2Unknown, score: 0.13
2310002L09RikUnknown, score: 0.45
Ppm1jUnknown, score: 0.25
Tmem106bUnknown, score: 0.57
Cand1Unknown, score: 0.15
Paqr7Unknown, score: 0.24
Serpina9Unknown, score: 0.02
Cldn23Unknown, score: 0.23
Jsrp1Unknown, score: 0.03
Antxr2Unknown, score: 0.25
Zcchc24Unknown, score: 0.05
Rpap3Unknown, score: 0.09
EpgnUnknown, score: 0.2
2310047M10RikUnknown, score: 0.15
Tube1Unknown, score: 0.08
Apol6Unknown, score: 0.57
Endod1Unknown, score: 0.35
2310067B10RikUnknown, score: 0.12
Cers5Unknown, score: 0.28
Gpc2Unknown, score: 0.31
2410016O06RikUnknown, score: 0.15
Ist1Unknown, score: 0.22
Rnf135Unknown, score: 0.28
Cpsf3lUnknown, score: 0.32
Myh14Unknown, score: 0.33
Nkiras2Unknown, score: 0.23
Wdr73Unknown, score: 0.06
Zbed5Unknown, score: 0.24
DnmbpUnknown, score: 0.01
Rbpms2Unknown, score: 0.03
Snx10Unknown, score: 0.56
Tmco6Unknown, score: 0.15
Sars2Unknown, score: 0.4
Acad10Unknown, score: 0.34
Esco2Unknown, score: 0.45
Rpusd4Unknown, score: 0.51
Ddx54Unknown, score: 0.24
Cnn3Unknown, score: 0.06
Erv3Unknown, score: 0.18
Smg9Unknown, score: 0.03
Lmntd2Unknown, score: 0.16
Fndc3bUnknown, score: 0.2
Zfyve19Unknown, score: 0.34
Cyb5r1Unknown, score: 0.15
Zfp654Unknown, score: 0.14
Slc35f2Unknown, score: 0.27
Cyb561d1Unknown, score: 0.27
TrmuUnknown, score: 0.03
Slc39a4Unknown, score: 0.01
Cdhr5Unknown, score: 0.53
Sulf2Unknown, score: 0.11
2010001E11RikUnknown, score: 0.19
Ddx42Unknown, score: 0.08
Tnfrsf13cUnknown, score: 0.09
Kdelc1Unknown, score: 0.11
Tmub2Unknown, score: 0.24
Cyp4f18Unknown, score: 0.01
Rap2cUnknown, score: 0.21
Anks4bUnknown, score: 0.73
Sapcd2Unknown, score: 0.29
Mzt2Unknown, score: 0.25
Tmem68Unknown, score: 0.1
AplfUnknown, score: 0.14
Ddhd2Unknown, score: 0.65
Zbed3Unknown, score: 0.03
Dennd2dUnknown, score: 0.18
Pygo1Unknown, score: 0.27
Chst14Unknown, score: 0.35
Slc37a3Unknown, score: 0.05
Wdfy3Unknown, score: 0.24
TdrpUnknown, score: 0.33
Rfc5Unknown, score: 0.04
CenpnUnknown, score: 0.13
Pgm2Unknown, score: 0.31
Dhx36Unknown, score: 0.15
Thumpd2Unknown, score: 0.01
Trim29Unknown, score: 0.26
Chchd4Unknown, score: 0.19
Shq1Unknown, score: 0.01
Fbxl2Unknown, score: 0.2
Snx6Unknown, score: 0.02
Klhl35Unknown, score: 0
Scaf11Unknown, score: 0.13
Skiv2l2Unknown, score: 0.47
Otud6bUnknown, score: 0.61
Eml2Unknown, score: 0.14
1700001P01RikUnknown, score: 0.04
Tbc1d5Unknown, score: 0.36
1600014C10RikUnknown, score: 0.28
Tram1Unknown, score: 0.22
2200002D01RikUnknown, score: 0.26
Ccpg1Unknown, score: 0.13
Sh2d4aUnknown, score: 0.3
Oraov1Unknown, score: 0.65
Nkd2Unknown, score: 0.09
Rusc1Unknown, score: 0.01
1810041L15RikUnknown, score: 0.26
Zfp777Unknown, score: 0.38
2510002D24RikUnknown, score: 0.19
Brf1Unknown, score: 0.29
Tmem158Unknown, score: 0.11
FrylUnknown, score: 0.19
Ccdc74aUnknown, score: 0.05
2510003E04RikUnknown, score: 0.24
Xpo5Unknown, score: 0.45
Asb6Unknown, score: 0.1
Klhl40Unknown, score: 0.11
Wdr89Unknown, score: 0.55
Elp6Unknown, score: 0.14
Ptar1Unknown, score: 0.06
Ttc4Unknown, score: 0.29
Cdpf1Unknown, score: 0.18
2210016L21RikUnknown, score: 0.06
Ces2gUnknown, score: 0.11
PscaUnknown, score: 0.26
Ripk4Unknown, score: 0.09
Cdkn3Unknown, score: 0.29
Faim2Unknown, score: 0.05
Wdr44Unknown, score: 0.08
Sgol1Unknown, score: 0.06
LrpprcUnknown, score: 0
Katnbl1Unknown, score: 0.07
Dnajc25Unknown, score: 0.01
Prr5lUnknown, score: 0.2
PrcpUnknown, score: 0
Rrp1bUnknown, score: 0.78
Plcd3Unknown, score: 0.17
Tmem87bUnknown, score: 0.16
Tspyl4Unknown, score: 0.31
Acbd6Unknown, score: 0.02
Rnf219Unknown, score: 0.69
Cwf19l1Unknown, score: 0.07
Tmem173Unknown, score: 0.05
Tmem55aUnknown, score: 0.53
Atxn7l2Unknown, score: 0.25
Reep4Unknown, score: 0.12
Naalad2Unknown, score: 0.56
Lypd1Unknown, score: 0.08
Pan3Unknown, score: 0.2
Zfp655Unknown, score: 0.12
Pih1d2Unknown, score: 0.27
Pdzd11Unknown, score: 0.07
Mex3aUnknown, score: 0.02
2810006K23RikUnknown, score: 0.24
Ints8Unknown, score: 0.49
2700097O09RikUnknown, score: 0.39
Dis3Unknown, score: 0
Zfp444Unknown, score: 0.03
Dnajc6Unknown, score: 0.14
HnrnpllUnknown, score: 0.14
Zcchc12Unknown, score: 0.21
Zfp618Unknown, score: 0.3
Zfp74Unknown, score: 0.46
TbccUnknown, score: 0.11
B3gat3Unknown, score: 0.15
Cdc42se2Unknown, score: 0.22
Zkscan3Unknown, score: 0.1
Tmem161bUnknown, score: 0.36
Ttc39cUnknown, score: 0.08
Hdhd3Unknown, score: 0.06
Fam117bUnknown, score: 0.3
Arhgef10lUnknown, score: 0.11
Tmem135Unknown, score: 0.12
Rint1Unknown, score: 0.32
Neil1Unknown, score: 0.34
Rspo3Unknown, score: 0.02
Ttc19Unknown, score: 0.19
Pard3bUnknown, score: 0.01
Ubash3bUnknown, score: 0.45
Mblac2Unknown, score: 0.01
Cxx1cUnknown, score: 0.04
Ccdc176Unknown, score: 0.47
Zdhhc4Unknown, score: 0.35
Ccdc94Unknown, score: 0.06
Setd5Unknown, score: 0.13
Asphd2Unknown, score: 0.19
Macrod2Unknown, score: 0.18
March1Unknown, score: 0.06
HepacamUnknown, score: 0.03
Ppp2r2bUnknown, score: 0.87
Swi5Unknown, score: 0.1
Hspb11Unknown, score: 0.07
Lrrc47Unknown, score: 0.39
TpppUnknown, score: 0.14
Slc17a7Unknown, score: 0.22
TympUnknown, score: 0.12
Ccser2Unknown, score: 0.11
Fbxo47Unknown, score: 0.07
Appl1Unknown, score: 0.17
Insig2Unknown, score: 0.02
Gpr22Unknown, score: 0.04
Kremen2Unknown, score: 0.05
Emc7Unknown, score: 0.49
Glrx5Unknown, score: 0.28
Camk2n2Unknown, score: 0.41
3110007F17RikUnknown, score: 0.29
Ppp1r16aUnknown, score: 0.85
Tmem192Unknown, score: 0.02
Sgip1Unknown, score: 0.45
Slc25a42Unknown, score: 0.43
Slc22a23Unknown, score: 0.13
AbraclUnknown, score: 0.14
Tmed5Unknown, score: 0.57
Slc25a16Unknown, score: 0.01
Prrc1Unknown, score: 0.28
Larp1Unknown, score: 0.13
Otud3Unknown, score: 0.51
Pcdh18Unknown, score: 0.09
Nfatc4Unknown, score: 0.51
3110043O21RikUnknown, score: 0.15
3110082I17RikUnknown, score: 0.8
Prl8a1Unknown, score: 0.29
Gpbp1Unknown, score: 0.43
Rhobtb3Unknown, score: 0.02
Calr3Unknown, score: 0.03
Clhc1Unknown, score: 0.25
Slc25a31Unknown, score: 0.02
Itpripl1Unknown, score: 0.17
Arhgef6Unknown, score: 0.28
1700056E22RikUnknown, score: 0.19
Dcbld2Unknown, score: 0.22
Hbp1Unknown, score: 0.05
Msl3l2Unknown, score: 0.05
TeppUnknown, score: 0
Nme8Unknown, score: 0.12
CcsapUnknown, score: 0
Tex35Unknown, score: 0.41
Hspa12aUnknown, score: 0.15
Zfp763Unknown, score: 0.42
1700067K01RikUnknown, score: 0.08
Izumo1Unknown, score: 0
Mipol1Unknown, score: 0.19
Vgll3Unknown, score: 0.11
CamkmtUnknown, score: 0.05
1700001O22RikUnknown, score: 0.24
Capn9Unknown, score: 0.12
Ms4a6cUnknown, score: 0.39
Spns1Unknown, score: 0.03
Cabp4Unknown, score: 0.23
2410004P03RikUnknown, score: 0.09
Sult6b1Unknown, score: 0.18
Psma8Unknown, score: 0
Trmt11Unknown, score: 0.29
Atg16l2Unknown, score: 0.32
2410089E03RikUnknown, score: 0.11
Ppp2r1bUnknown, score: 0.11
Tubb2bUnknown, score: 0.1
DmknUnknown, score: 0.28
Rbm20Unknown, score: 0.17
1110017D15RikUnknown, score: 0.06
Sh3bgrl3Unknown, score: 0.25
MceeUnknown, score: 0.08
PsdUnknown, score: 0.06
Fcf1Unknown, score: 0.44
Cby1Unknown, score: 0.64
Gadl1Unknown, score: 0.12
Kif2cUnknown, score: 0.25
Fam83eUnknown, score: 0.17
Mfsd12Unknown, score: 0
Ppp1r21Unknown, score: 0.17
Tmem198bUnknown, score: 0.45
Eif3kUnknown, score: 0.63
Atp6v1dUnknown, score: 0.07
Ifitm5Unknown, score: 0.39
Ankrd42Unknown, score: 0.27
4930415F15RikUnknown, score: 0.2
Fam161aUnknown, score: 0.15
Tmem202Unknown, score: 0.15
Ift57Unknown, score: 0.21
Lyrm1Unknown, score: 0.05
Atl1Unknown, score: 0.41
Herc3Unknown, score: 0.01
Psd2Unknown, score: 0.05
Dnm1lUnknown, score: 0.45
Btbd11Unknown, score: 0.59
ArsgUnknown, score: 0.17
Rftn2Unknown, score: 0.07
Traf3ip1Unknown, score: 0.14
Glyr1Unknown, score: 0.2
Rd3Unknown, score: 0.04
Sdr42e1Unknown, score: 0.23
DdiasUnknown, score: 0.35
Pex26Unknown, score: 0.13
Plce1Unknown, score: 0.03
Lmntd1Unknown, score: 0.11
Syce1Unknown, score: 0.58
Slc7a13Unknown, score: 0.07
Them7Unknown, score: 0.01
Paqr5Unknown, score: 0.28
NplUnknown, score: 0.37
Hvcn1Unknown, score: 0.47
0610037L13RikUnknown, score: 0.2
Abcb6Unknown, score: 0.29
ParnUnknown, score: 0.17
Actr3Unknown, score: 0.13
Zfp263Unknown, score: 0.12
Tmem43Unknown, score: 0.02
Armc8Unknown, score: 0.47
Cyp2s1Unknown, score: 0.27
Sec14l1Unknown, score: 0.31
Nuak2Unknown, score: 0.42
Tm9sf1Unknown, score: 0.16
Opa1Unknown, score: 0.02
F13a1Unknown, score: 0.29
EhhadhUnknown, score: 0.21
Zfp946Unknown, score: 0.26
Stra6lUnknown, score: 0.23
Uba7Unknown, score: 0.18
Acot12Unknown, score: 0.38
Acbd5Unknown, score: 0.39
Nfx1Unknown, score: 0.38
Tmem38aUnknown, score: 0.27
Nudt9Unknown, score: 0.03
Gtsf1Unknown, score: 0.06
Gpcpd1Unknown, score: 0.22
Perm1Unknown, score: 0.13
Ccdc3Unknown, score: 0.13
Katnb1Unknown, score: 0.16
Phactr3Unknown, score: 0.54
Exoc3l4Unknown, score: 0.05
P2ry13Unknown, score: 0.17
Rnd3Unknown, score: 0.32
Gtf2e1Unknown, score: 0.19
Cep97Unknown, score: 0.89
Eif4enif1Unknown, score: 0.29
Acsl3Unknown, score: 0.12
Sipa1l3Unknown, score: 0.29
IqceUnknown, score: 0.21
Slx4ipUnknown, score: 0.29
Atg7Unknown, score: 0.02
CtbsUnknown, score: 0.22
GaleUnknown, score: 0.02
Lrrc2Unknown, score: 0.35
Klrg2Unknown, score: 0.46
CyldUnknown, score: 0.12
Tspan17Unknown, score: 0.11
AvenUnknown, score: 0.95
Chic2Unknown, score: 0.12
Spatc1Unknown, score: 0.09
Prss46Unknown, score: 0.25
1700092M07RikUnknown, score: 0.43
Isca2Unknown, score: 0.5
HopxUnknown, score: 0.23
Mettl23Unknown, score: 0.09
Wdr33Unknown, score: 0.17
Dnajc14Unknown, score: 0.23
Ranbp10Unknown, score: 0.11
Xrcc3Unknown, score: 0.1
Palm3Unknown, score: 0.03
Ahcyl2Unknown, score: 0.17
Lrrtm1Unknown, score: 0.33
Tldc1Unknown, score: 0.23
Ddx23Unknown, score: 0.14
Smchd1Unknown, score: 0.51
4931428F04RikUnknown, score: 0.12
Lonrf3Unknown, score: 0.02
Clec16aUnknown, score: 0.11
Gcc1Unknown, score: 0.2
Myo18bUnknown, score: 0.01
Dpp8Unknown, score: 0.08
Specc1lUnknown, score: 0.29
Ttc25Unknown, score: 0.49
Ppapdc2Unknown, score: 0.01
Gle1Unknown, score: 0.15
Polr3cUnknown, score: 0.38
Tmc5Unknown, score: 0.24
4933402D24RikUnknown, score: 0.09
4930452B06RikUnknown, score: 0.1
Sgms2Unknown, score: 0.19
P4htmUnknown, score: 0.28
Slc9b1Unknown, score: 0.2
Pank2Unknown, score: 0.28
Pgs1Unknown, score: 0.05
Cfap53Unknown, score: 0.24
Nsun6Unknown, score: 0.14
74463Unknown, score: 0.03
Cep72Unknown, score: 0.12
Snx29Unknown, score: 0.66
Snx11Unknown, score: 0.08
Lrrc71Unknown, score: 0.1
Osbpl10Unknown, score: 0.34
Lrrc15Unknown, score: 0.26
Tnks2Unknown, score: 0.35
Gorasp1Unknown, score: 0.27
SostUnknown, score: 0.21
Lrrc17Unknown, score: 0.05
Cyp2j9Unknown, score: 0.06
Ppp4r4Unknown, score: 0.04
8430419L09RikUnknown, score: 0.03
Gsdmc4Unknown, score: 0.22
Mau2Unknown, score: 0.29
Pck2Unknown, score: 0.28
Nipal3Unknown, score: 0.25
Gvin1Unknown, score: 0.19
Elovl7Unknown, score: 0.23
Pyroxd2Unknown, score: 0.39
Sppl3Unknown, score: 0.11
Kbtbd12Unknown, score: 0.07
Cd200r3Unknown, score: 0.37
Scpep1Unknown, score: 0.18
Tmem81Unknown, score: 0.07
ShpkUnknown, score: 0.16
Fam46cUnknown, score: 0.2
PomkUnknown, score: 0.09
Zfp943Unknown, score: 0.03
Pcf11Unknown, score: 0.03
C2cd5Unknown, score: 0.22
Slamf8Unknown, score: 0.38
Dhcr24Unknown, score: 0
Mxra8Unknown, score: 0.01
Naa60Unknown, score: 0.24
Klc4Unknown, score: 0.15
Yipf2Unknown, score: 0
Pik3cbUnknown, score: 0.44
HhatlUnknown, score: 0.14
Atp13a2Unknown, score: 0.42
Lmbr1lUnknown, score: 0.2
Rrp7aUnknown, score: 0.09
Naa15Unknown, score: 0.21
ManfUnknown, score: 0.06
Kbtbd11Unknown, score: 0.24
Rab11fip2Unknown, score: 0.3
Sf3a3Unknown, score: 0.34
Zbtb49Unknown, score: 0.39
Ube2dnl2Unknown, score: 0.37
Nxnl2Unknown, score: 0.01
75136Unknown, score: 0.09
Rprd2Unknown, score: 0.44
Rasd2Unknown, score: 0.08
Tex38Unknown, score: 0.32
MeiobUnknown, score: 0.21
Sv2cUnknown, score: 0.32
Dusp18Unknown, score: 0.04
Rnf19bUnknown, score: 0.41
Tomm20lUnknown, score: 0.02
Ppp1r1cUnknown, score: 0.37
Bcdin3dUnknown, score: 0.13
Slc35f4Unknown, score: 0.21
Slamf7Unknown, score: 0.19
Arhgap36Unknown, score: 0.72
Slitrk5Unknown, score: 0.27
Nop14Unknown, score: 0.13
Zfp820Unknown, score: 0.12
1700011I03RikUnknown, score: 0.11
Ascc2Unknown, score: 0.07
1700008F21RikUnknown, score: 0.87
Phpt1Unknown, score: 0.03
Cfap126Unknown, score: 0.15
OplahUnknown, score: 0.06
1700003F12RikUnknown, score: 0.03
Gpx6Unknown, score: 0.55
EppinUnknown, score: 0.04
Nme5Unknown, score: 0.23
Fam71e1Unknown, score: 0.02
FpgtUnknown, score: 0.61
Akap13Unknown, score: 0.76
Zc3h14Unknown, score: 0
Ep400Unknown, score: 0.08
Rsph9Unknown, score: 0.18
Ccdc101Unknown, score: 0.2
75570Unknown, score: 0.21
Spata9Unknown, score: 0.18
Acyp2Unknown, score: 0.22
2310007L24RikUnknown, score: 0.26
Zbtb4Unknown, score: 0.31
Yipf7Unknown, score: 0
Dusp9Unknown, score: 0.6
Kdm5bUnknown, score: 0.17
GnsUnknown, score: 0.28
Smim15Unknown, score: 0
Fastkd2Unknown, score: 0.16
Kxd1Unknown, score: 0.21
Tex30Unknown, score: 0.25
Metap1Unknown, score: 0.11
Snapc1Unknown, score: 0.03
1700029I15RikUnknown, score: 0.12
Rai14Unknown, score: 0.15
Wdr54Unknown, score: 0.28
Lin37Unknown, score: 0.29
Ccdc64Unknown, score: 0.05
Rasl10aUnknown, score: 0
Nudt16Unknown, score: 0.05
Higd1bUnknown, score: 0.49
Vsig10lUnknown, score: 0.31
Rilpl1Unknown, score: 0.25
C2cd4bUnknown, score: 0.08
Fam35aUnknown, score: 0.05
Eif4bUnknown, score: 0.32
Rbm12Unknown, score: 0.26
Fam227aUnknown, score: 0.14
IdnkUnknown, score: 0.01
IqcdUnknown, score: 0.15
MffUnknown, score: 0.21
Pank1Unknown, score: 0.57
Mpp7Unknown, score: 0.24
Egfem1Unknown, score: 0
Sesn3Unknown, score: 0.2
Slc10a6Unknown, score: 0.05
Ipo4Unknown, score: 0.3
Dcaf17Unknown, score: 0.04
Rab11fip1Unknown, score: 0
Pnpla5Unknown, score: 0.02
Lca5Unknown, score: 0.08
Ckap5Unknown, score: 0.35
Smurf1Unknown, score: 0.08
NlnUnknown, score: 0.04
Tasp1Unknown, score: 0.12
Hormad2Unknown, score: 0.11
IspdUnknown, score: 0.12
Tex26Unknown, score: 0
Arl5bUnknown, score: 0.17
Tcam1Unknown, score: 0.15
Exoc6bUnknown, score: 0.01
4930579G24RikUnknown, score: 0.01
Rab30Unknown, score: 0.1
Zc3h18Unknown, score: 0.16
Gon4lUnknown, score: 0.24
Ncapg2Unknown, score: 0.28
GancUnknown, score: 0.23
Mgea5Unknown, score: 0.03
Rnf183Unknown, score: 0.1
Gbp8Unknown, score: 0.06
Dock8Unknown, score: 0.15
FaxcUnknown, score: 0.11
Ccdc138Unknown, score: 0.52
Fam131bUnknown, score: 0.05
Rsg1Unknown, score: 0.13
Snrnp35Unknown, score: 0.17
Abca6Unknown, score: 0.37
Adhfe1Unknown, score: 0.16
Abhd12Unknown, score: 0.14
Med13lUnknown, score: 0.36
Stard3nlUnknown, score: 0.09
Jakmip2Unknown, score: 0.04
Arxes1Unknown, score: 0.06
Agbl3Unknown, score: 0.04
76224Unknown, score: 0.24
Dnttip1Unknown, score: 0.18
Ttc8Unknown, score: 0.09
0610040J01RikUnknown, score: 0.53
Gstk1Unknown, score: 0.28
Fads1Unknown, score: 0.09
Tax1bp3Unknown, score: 0.57
Mfap4Unknown, score: 0.67
PcnpUnknown, score: 0.06
Slc18b1Unknown, score: 0.34
Rab1bUnknown, score: 0.07
Trmt5Unknown, score: 0.02
Mtif3Unknown, score: 0.43
Trp53rkUnknown, score: 0.12
Zfp773Unknown, score: 0.51
1700012A03RikUnknown, score: 0.03
Znrd1asUnknown, score: 0.01
Gid8Unknown, score: 0.32
LhppUnknown, score: 0.24
Prss23Unknown, score: 0.63
Fbxo31Unknown, score: 0.47
Casc5Unknown, score: 0.11
Pcolce2Unknown, score: 0.38
Smndc1Unknown, score: 0.09
3110002H16RikUnknown, score: 0.25
Ppp1r3gUnknown, score: 0.18
Ppp1r11Unknown, score: 0.27
Clasp2Unknown, score: 0.27
Ip6k2Unknown, score: 0.4
Commd9Unknown, score: 0.05
Plet1Unknown, score: 0.32
Trappc9Unknown, score: 0.16
Lsm8Unknown, score: 0.47
Cln6Unknown, score: 0.46
Fam204aUnknown, score: 0.26
Snx7Unknown, score: 0.06
Faf2Unknown, score: 0.61
Mib2Unknown, score: 0.03
Ipo11Unknown, score: 0.11
Dnajc18Unknown, score: 0.11
Lrrc27Unknown, score: 0
1700112E06RikUnknown, score: 0.08
1700113H08RikUnknown, score: 0.07
Clip3Unknown, score: 0.6
Arfrp1Unknown, score: 0.16
Arpc2Unknown, score: 0.06
Kansl1Unknown, score: 0.03
Efr3aUnknown, score: 0.19
Snx27Unknown, score: 0.12
Mospd2Unknown, score: 0.17
AlpiUnknown, score: 0.16
Slc10a7Unknown, score: 0.2
Mtif2Unknown, score: 0.13
Klhdc10Unknown, score: 0.32
Mzt1Unknown, score: 0.08
2410131K14RikUnknown, score: 0.07
Snip1Unknown, score: 0.1
Tbc1d9bUnknown, score: 0.04
Kdm4cUnknown, score: 0.49
Rpl18aUnknown, score: 0.24
Bri3bpUnknown, score: 0.42
Fam49aUnknown, score: 0.13
Mtfr1lUnknown, score: 0.45
DtlUnknown, score: 1
SpoplUnknown, score: 0.02
Morn1Unknown, score: 0.02
Rhbdd1Unknown, score: 0.03
Ccdc116Unknown, score: 0.05
Fam81aUnknown, score: 0.14
Memo1Unknown, score: 0.01
Rnft1Unknown, score: 0.2
Mettl15Unknown, score: 0.13
Golga1Unknown, score: 0
Ssbp4Unknown, score: 0.16
Jade2Unknown, score: 0.68
Lrg1Unknown, score: 0.02
Mnd1Unknown, score: 0.15
Flywch2Unknown, score: 0.23
TsaccUnknown, score: 0.23
Arfip2Unknown, score: 0.46
Ifi27l2aUnknown, score: 0.13
Rbm17Unknown, score: 0.36
Ndufaf6Unknown, score: 0.1
Nt5c2Unknown, score: 0.35
2610028H24RikUnknown, score: 0.24
2700049A03RikUnknown, score: 0.09
2810007J24RikUnknown, score: 0.3
UrahUnknown, score: 0.04
Arxes2Unknown, score: 0.31
Scfd1Unknown, score: 0.27
Hdhd2Unknown, score: 0.07
TicrrUnknown, score: 0.01
Col25a1Unknown, score: 0.36
Tstd3Unknown, score: 0.16
1700109H08RikUnknown, score: 0.03
Arfgap2Unknown, score: 0.42
Arid2Unknown, score: 0.56
Cep83Unknown, score: 0.35
Sun1Unknown, score: 0.34
Tmco4Unknown, score: 0.01
Ccdc183Unknown, score: 0.1
Ankrd11Unknown, score: 0.63
Tanc2Unknown, score: 0.52
Il33Unknown, score: 0.16
CrebrfUnknown, score: 0.67
Hnrnpa0Unknown, score: 0.03
9430038I01RikUnknown, score: 0.46
Yif1bUnknown, score: 0.45
Sec24aUnknown, score: 0.11
Rab35Unknown, score: 0.31
Esrp2Unknown, score: 0
Heg1Unknown, score: 0.06
JrklUnknown, score: 0.03
Shisa4Unknown, score: 0.11
Limch1Unknown, score: 0.16
Spns3Unknown, score: 0.26
Myh10Unknown, score: 0.39
Mboat7Unknown, score: 0.06
NotumUnknown, score: 0.03
Nup210lUnknown, score: 0.13
Rbm12b1Unknown, score: 0.27
H2afvUnknown, score: 0.03
Ccdc151Unknown, score: 0.14
Apex2Unknown, score: 0.2
Smpd4Unknown, score: 0.3
Snapc3Unknown, score: 0.02
C330007P06RikUnknown, score: 0
Trat1Unknown, score: 0.16
Ehmt1Unknown, score: 0.26
MmabUnknown, score: 0.37
9230104L09RikUnknown, score: 0.14
BoraUnknown, score: 0.1
Csrnp3Unknown, score: 0.06
Adamtsl2Unknown, score: 0.01
Esco1Unknown, score: 0.05
Lrrc42Unknown, score: 0.82
Krba1Unknown, score: 0.15
6030458C11RikUnknown, score: 0.28
LbhUnknown, score: 0.28
Yipf6Unknown, score: 0.1
Fam53bUnknown, score: 0.02
Rpgrip1Unknown, score: 0.02
Rdh12Unknown, score: 0.21
Tmem50bUnknown, score: 0.08
Nuak1Unknown, score: 0.09
Ascc3Unknown, score: 0.19
CutalUnknown, score: 0.11
Prr15Unknown, score: 0.09
Ccdc150Unknown, score: 0.41
Mccc2Unknown, score: 0.02
Lcn8Unknown, score: 0
Lpar4Unknown, score: 0.17
NinlUnknown, score: 0.06
Trappc6bUnknown, score: 0.2
Dnajc21Unknown, score: 0.26
Armcx1Unknown, score: 0.23
Zfp712Unknown, score: 0.01
Ralgps2Unknown, score: 0.12
Lrrc9Unknown, score: 0.35
Zfp687Unknown, score: 0.21
Creb3l4Unknown, score: 0.3
RbsnUnknown, score: 0.58
Rps27aUnknown, score: 0.12
Hist3h2baUnknown, score: 0.68
Naa38Unknown, score: 0.17
78306Unknown, score: 0.29
Gpr108Unknown, score: 0.3
Cul9Unknown, score: 0.27
Ccdc88bUnknown, score: 0.19
Ankrd23Unknown, score: 0.17
Fam219bUnknown, score: 0.28
Cdk19Unknown, score: 0.3
Ttyh3Unknown, score: 0.3
2210407C18RikUnknown, score: 0.22
Icam4Unknown, score: 0.11
Snrnp25Unknown, score: 0.07
Sapcd1Unknown, score: 0.3
Ddx52Unknown, score: 0.1
Ntf5Unknown, score: 0.81
Fam131aUnknown, score: 0.43
1700084C01RikUnknown, score: 0.16
Micu3Unknown, score: 0.06
B230219D22RikUnknown, score: 0.34
Mrpl9Unknown, score: 0.08
Nrip3Unknown, score: 0.17
Zfp449Unknown, score: 0.09
Bola3Unknown, score: 0.05
Eif3j1Unknown, score: 0.09
Ncapd3Unknown, score: 0.78
Plekhj1Unknown, score: 0.58
RictorUnknown, score: 0.01
Cfap61Unknown, score: 0.12
Enthd2Unknown, score: 0.14
Zc3hav1Unknown, score: 0.16
Brpf1Unknown, score: 0
Clip4Unknown, score: 0.31
Armc9Unknown, score: 0.07
Zcchc4Unknown, score: 0.53
Ak7Unknown, score: 0.05
Ttc30a1Unknown, score: 0.3
4930562C15RikUnknown, score: 0.11
P2ry10Unknown, score: 0.57
Tsc22d4Unknown, score: 0.15
Zfp623Unknown, score: 0.25
Wsb1Unknown, score: 0.19
Scyl1Unknown, score: 0
Crispld2Unknown, score: 0.45
Cnot10Unknown, score: 0.07
1500015O10RikUnknown, score: 0.59
Igsf3Unknown, score: 0.2
Asb15Unknown, score: 0.32
Fndc8Unknown, score: 0.06
DlstUnknown, score: 0.04
Gas2l1Unknown, score: 0.08
PigtUnknown, score: 0.3
Agbl4Unknown, score: 0.17
Popdc3Unknown, score: 0.31
Spsb3Unknown, score: 0.6
Mrps34Unknown, score: 0.57
Nme3Unknown, score: 0.33
Osbpl5Unknown, score: 0.05
Tnfrsf23Unknown, score: 0.26
Hdac9Unknown, score: 0.09
Zfp319Unknown, score: 0.34
LratUnknown, score: 0.01
Krit1Unknown, score: 0.03
Bhlhe41Unknown, score: 0.02
Wbscr27Unknown, score: 0.37
Sh3bp5lUnknown, score: 0.13
Cttnbp2nlUnknown, score: 0.67
Abtb1Unknown, score: 0.28
Smim12Unknown, score: 0.16
Parp9Unknown, score: 0.37
Apobec3Unknown, score: 0.25
Lysmd3Unknown, score: 0.19
Gpr146Unknown, score: 0.08
Rilpl2Unknown, score: 0.44
Pofut2Unknown, score: 0.41
Kcnip4Unknown, score: 0.52
WwoxUnknown, score: 0.03
Igsf6Unknown, score: 0.07
MynnUnknown, score: 0.47
Car15Unknown, score: 0.58
BC004004Unknown, score: 0.39
Lrfn1Unknown, score: 0.04
Rnf34Unknown, score: 0.07
Fam20cUnknown, score: 0.18
Klrb1bUnknown, score: 0.22
Ifitm2Unknown, score: 0.05
Slc16a3Unknown, score: 0.31
Kank3Unknown, score: 0.34
Hcar2Unknown, score: 0.22
Senp3Unknown, score: 0.31
Hspb8Unknown, score: 0.26
Trim2Unknown, score: 0.02
Cxcr6Unknown, score: 0.05
Zfp202Unknown, score: 0.05
Dtx3Unknown, score: 0.08
PolhUnknown, score: 0.53
Kcnip2Unknown, score: 0.14
Pum2Unknown, score: 0.13
Uck2Unknown, score: 0.41
Syt13Unknown, score: 0.1
MrgprhUnknown, score: 0.52
Ckap2Unknown, score: 0.27
Vmn1r58Unknown, score: 0.21
Rnf114Unknown, score: 0.1
Dnajb1Unknown, score: 0.38
Sgpp1Unknown, score: 0.22
Kat5Unknown, score: 0.49
Zbtb22Unknown, score: 0.24
C1qtnf3Unknown, score: 0.43
TnxbUnknown, score: 0.26
Ift122Unknown, score: 0.14
Sf3b1Unknown, score: 0.22
Cyp4x1Unknown, score: 0.05
Tmem108Unknown, score: 0.27
SigleceUnknown, score: 0.28
Akap12Unknown, score: 0.05
Ndst3Unknown, score: 0.23
Gimap3Unknown, score: 0.34
Cstf2tUnknown, score: 0.28
CtnsUnknown, score: 0.03
Plekha2Unknown, score: 0.14
Mov10l1Unknown, score: 0.08
Tex13Unknown, score: 0.03
Tex14Unknown, score: 0.12
Gtf2a1Unknown, score: 0.11
Elovl4Unknown, score: 0.34
Sytl2Unknown, score: 0.05
Sytl3Unknown, score: 0.25
Dbr1Unknown, score: 0.2
Slc12a9Unknown, score: 0.36
Wasf1Unknown, score: 0.15
Dpp7Unknown, score: 0.34
Tas1r3Unknown, score: 0.1
Tnk1Unknown, score: 0.04
CenpqUnknown, score: 0.11
Trps1Unknown, score: 0.28
PhipUnknown, score: 0.36
Nrg4Unknown, score: 0.06
Btbd1Unknown, score: 0.15
Enpp5Unknown, score: 0.27
McamUnknown, score: 0.14
Kremen1Unknown, score: 0.44
PlvapUnknown, score: 0.38
Sucnr1Unknown, score: 0.42
Cd96Unknown, score: 0.4
Fam126aUnknown, score: 0.06
Hes7Unknown, score: 0.05
Pla1aUnknown, score: 0.25
KarsUnknown, score: 0.54
Emc9Unknown, score: 0.04
Sec16bUnknown, score: 0.07
Cml2Unknown, score: 0
Lmod2Unknown, score: 0.34
Entpd7Unknown, score: 0.17
Klf7Unknown, score: 0.08
GpnmbUnknown, score: 0.19
Pcdhga9Unknown, score: 0.25
Acox2Unknown, score: 0.24
Aff4Unknown, score: 0.13
Pard6gUnknown, score: 0.26
Gprc5dUnknown, score: 0.29
Echs1Unknown, score: 0.44
Sirt1Unknown, score: 0.26
Peli2Unknown, score: 0.08
AmnUnknown, score: 0.23
Dach2Unknown, score: 0.35
Vangl2Unknown, score: 0.12
Uchl4Unknown, score: 0.25
PnckUnknown, score: 0.13
Pcdhb5Unknown, score: 0.23
Pcdhb22Unknown, score: 0.18
Cers1Unknown, score: 0.04
Nkd1Unknown, score: 0.46
Klra8Unknown, score: 0.35
Klra1Unknown, score: 0.28
ClmnUnknown, score: 0.77
Bcl2l13Unknown, score: 0.13
Mrpl16Unknown, score: 0.45
Mrpl27Unknown, score: 0.55
Mrpl34Unknown, score: 0.08
Mrpl36Unknown, score: 0.17
Trim9Unknown, score: 0.37
Trim11Unknown, score: 0.08
Dock2Unknown, score: 0.29
Mcoln1Unknown, score: 0.03
NansUnknown, score: 0.32
Pdxdc1Unknown, score: 0.45
Ophn1Unknown, score: 0.29
Spock2Unknown, score: 0.21
Col4a6Unknown, score: 0.37
Lrp1bUnknown, score: 0.3
Cnnm2Unknown, score: 0.07
Dgcr8Unknown, score: 0.5
Pi15Unknown, score: 0.22
Cpsf1Unknown, score: 0.15
Fkbp6Unknown, score: 0.03
Arid4bUnknown, score: 0.07
Hecw1Unknown, score: 0.41
Wbscr16Unknown, score: 0.12
Maged1Unknown, score: 0.14
Sfxn4Unknown, score: 0.24
Sfxn5Unknown, score: 0.1
Cadm3Unknown, score: 0.07
Prg4Unknown, score: 0.07
Susd4Unknown, score: 0.12
Tmem62Unknown, score: 0.4
Ptges2Unknown, score: 0.31
Hist2h3c2Unknown, score: 0.04
C77080Unknown, score: 0.2
Hmgb2Unknown, score: 0.26
HadhaUnknown, score: 0.12
Naa11Unknown, score: 0.22
B3gnt9Unknown, score: 0.52
Cog8Unknown, score: 0.02
4833439L19RikUnknown, score: 0.35
Hist1h3gUnknown, score: 0.54
Nol12Unknown, score: 0.12
Gtf2f1Unknown, score: 0.04
Tmem132aUnknown, score: 0.31
Dcaf8Unknown, score: 0.07
Eif3mUnknown, score: 0.14
Stk17bUnknown, score: 0.13
Efhd1Unknown, score: 0.16
GorabUnknown, score: 0.49
Chst10Unknown, score: 0.32
Cnih4Unknown, score: 0.17
MaelUnknown, score: 0.33
Trmt1lUnknown, score: 0.21
Rdh10Unknown, score: 0
Rab3gap2Unknown, score: 0.39
HnrnpfUnknown, score: 0.51
Cdc123Unknown, score: 0.43
Eps8l2Unknown, score: 0.02
Myl9Unknown, score: 0.34
Fam102aUnknown, score: 0.03
Lpcat4Unknown, score: 0.48
Pomt1Unknown, score: 0.33
Osbpl6Unknown, score: 0.58
Cep152Unknown, score: 0.44
CercamUnknown, score: 0.04
Ssx2ipUnknown, score: 0.08
Tm9sf4Unknown, score: 0.16
Hrh3Unknown, score: 0.05
Arfgef2Unknown, score: 0.01
Cul4aUnknown, score: 0.29
Sall4Unknown, score: 0.51
DpydUnknown, score: 0.13
Adgrl2Unknown, score: 0.12
Eps8l3Unknown, score: 0.03
Sec24bUnknown, score: 0.13
Cept1Unknown, score: 0.17
Arfip1Unknown, score: 0.27
Kdm1aUnknown, score: 0.12
Mdn1Unknown, score: 0.01
Camta1Unknown, score: 0.23
Kti12Unknown, score: 0
Rcc1Unknown, score: 0.38
Pcsk9Unknown, score: 0.08
AI481877Unknown, score: 0.57
Zmym6Unknown, score: 0.3
AknaUnknown, score: 0.3
Tmem64Unknown, score: 0.48
Adprhl2Unknown, score: 0.31
Gpn2Unknown, score: 0.08
Osbpl9Unknown, score: 0.16
AU040320Unknown, score: 0.16
Slc44a1Unknown, score: 0.09
Lao1Unknown, score: 0
Rell1Unknown, score: 0.3
Lrrc8cUnknown, score: 0.27
Noc4lUnknown, score: 0.4
Nsun5Unknown, score: 0.1
N4bp2l1Unknown, score: 0.04
PsphUnknown, score: 0.09
Gbp6Unknown, score: 0.12
Mapre3Unknown, score: 0.15
Dcun1d4Unknown, score: 0.12
Ube3cUnknown, score: 0.29
Tbc1d14Unknown, score: 0.2
HscbUnknown, score: 0.31
Emilin1Unknown, score: 0.12
Rab28Unknown, score: 0
Akap9Unknown, score: 0.13
Zfp513Unknown, score: 0.08
Ttll3Unknown, score: 0.33
Tmem168Unknown, score: 0.34
Rpusd3Unknown, score: 0.13
Parp11Unknown, score: 0.14
Tra2aUnknown, score: 0.45
Wdr91Unknown, score: 0.01
EogtUnknown, score: 0.21
Adamts9Unknown, score: 0.2
Ceacam15Unknown, score: 0.07
Slco2b1Unknown, score: 0.29
Plekhg2Unknown, score: 0.29
Hsd3b7Unknown, score: 0.38
WtipUnknown, score: 0.01
AI467606Unknown, score: 0.28
E430018J23RikUnknown, score: 0.45
Psip1Unknown, score: 0.2
Rrp8Unknown, score: 0.31
Unc45aUnknown, score: 0.03
Sf3b3Unknown, score: 0.29
D8Ertd738eUnknown, score: 0.17
Ces2aUnknown, score: 0.23
Smim19Unknown, score: 0.15
Exoc8Unknown, score: 0.17
Gadd45gip1Unknown, score: 0.1
PhkbUnknown, score: 0.11
Mtus1Unknown, score: 0.7
Snx25Unknown, score: 0.4
Taf5lUnknown, score: 0.17
Prmt10Unknown, score: 0.07
Snapc2Unknown, score: 0.07
Dcun1d2Unknown, score: 0.07
Ankrd10Unknown, score: 0.28
Cog4Unknown, score: 0.47
Clk3Unknown, score: 0.34
HinfpUnknown, score: 0.34
Dennd4aUnknown, score: 0.01
XylbUnknown, score: 0.36
Imp3Unknown, score: 0.06
Pls1Unknown, score: 0.03
Cmtm7Unknown, score: 0.08
Ano10Unknown, score: 0.36
Mapkapk3Unknown, score: 0.2
OafUnknown, score: 0.13
Cd276Unknown, score: 0.09
Lrrc49Unknown, score: 0.12
Bbs4Unknown, score: 0.56
CenpiUnknown, score: 0.21
Slc6a15Unknown, score: 0.2
Pwp1Unknown, score: 0.22
Rdh9Unknown, score: 0.2
Upb1Unknown, score: 0.01
ApofUnknown, score: 0.22
Fig4Unknown, score: 0.38
Traf3ip2Unknown, score: 0.4
BC030307Unknown, score: 0.37
Cep57l1Unknown, score: 0.26
Zc3h10Unknown, score: 0.21
Zfr2Unknown, score: 0.42
Nt5dc3Unknown, score: 0.08
Fam26eUnknown, score: 0.24
Mbtd1Unknown, score: 0.34
E130012A19RikUnknown, score: 0.17
Sec14l4Unknown, score: 0.09
Tmed4Unknown, score: 0.13
Slc35e4Unknown, score: 0.11
PnpoUnknown, score: 0.37
Tbc1d10aUnknown, score: 0.11
Tubg1Unknown, score: 0.37
Pex12Unknown, score: 0.3
Tmem98Unknown, score: 0.02
Tubg2Unknown, score: 0.03
Wdr92Unknown, score: 0.18
Maml1Unknown, score: 0.37
Cuedc1Unknown, score: 0.12
Inca1Unknown, score: 0.33
Nt5mUnknown, score: 0.05
Hoxb2Unknown, score: 0.17
Dnm3Unknown, score: 0.34
Plin1Unknown, score: 0.01
Rtn1Unknown, score: 0.06
Qsox1Unknown, score: 0.17
Cdh22Unknown, score: 0.09
Nxph3Unknown, score: 0.02
Wdr7Unknown, score: 0.25
Cyp27a1Unknown, score: 0.11
Adcy4Unknown, score: 0.13
AclyUnknown, score: 0.55
Ndufb11Unknown, score: 0.62
Etv5Unknown, score: 0.38
GldcUnknown, score: 0.3
BlmhUnknown, score: 0.23
Cabin1Unknown, score: 0.25
Zfp119aUnknown, score: 0.2
Isl2Unknown, score: 0.21
Meig1Unknown, score: 0.5
E2f4Unknown, score: 0.34
Pcnxl3Unknown, score: 0.11
Bap1Unknown, score: 0.23
Rexo2Unknown, score: 0.03
Cdc42ep1Unknown, score: 0.3
RarsUnknown, score: 0.26
Smek2Unknown, score: 0.06
Tsr1Unknown, score: 0.3
Slc16a6Unknown, score: 0.14
Pik3r6Unknown, score: 0.1
Ddx1Unknown, score: 0.16
SptssaUnknown, score: 0.05
4930427A07RikUnknown, score: 0.09
Pld4Unknown, score: 0.07
JkampUnknown, score: 0.11
Aldh6a1Unknown, score: 0.11
FancmUnknown, score: 0.15
AspgUnknown, score: 0.18
Cbll1Unknown, score: 0.15
Tecpr2Unknown, score: 0.2
Spata7Unknown, score: 0.11
Tdp1Unknown, score: 0.24
Rab15Unknown, score: 0.32
Slc25a47Unknown, score: 0.09
Adi1Unknown, score: 0.07
Dnal1Unknown, score: 0.36
Fam84aUnknown, score: 0.05
Rdh14Unknown, score: 0.3
Arrdc3Unknown, score: 0.31
Fam208bUnknown, score: 0.13
Brd9Unknown, score: 0.22
Cdk20Unknown, score: 0.05
Epdr1Unknown, score: 0.23
Golm1Unknown, score: 0.17
AW209491Unknown, score: 0.14
Dusp22Unknown, score: 0.26
Ankrd32Unknown, score: 0.04
Dock9Unknown, score: 0.08
Fam170bUnknown, score: 0.25
Chmp7Unknown, score: 0.05
Mbnl2Unknown, score: 0.09
Zfp957Unknown, score: 0.11
PhyhipUnknown, score: 0.13
ThtpaUnknown, score: 0.33
Rcbtb2Unknown, score: 0.46
Slc38a1Unknown, score: 0.17
Fam83hUnknown, score: 0.02
Prkaa1Unknown, score: 0.18
Ccdc65Unknown, score: 0.06
MtbpUnknown, score: 0.2
Dennd3Unknown, score: 0.48
Card10Unknown, score: 0.1
Csdc2Unknown, score: 0.22
Espl1Unknown, score: 0.65
ToporsUnknown, score: 0.31
Gga1Unknown, score: 0.23
Fbxo4Unknown, score: 0.13
AW549877Unknown, score: 0.28
Slc45a4Unknown, score: 0.22
Osbpl11Unknown, score: 0.15
Rfc4Unknown, score: 0.03
Ypel1Unknown, score: 0.04
Slc51aUnknown, score: 0.1
Gpsm3Unknown, score: 0.27
TecrUnknown, score: 0.33
PpcsUnknown, score: 0.19
Rab31Unknown, score: 0.08
Itfg3Unknown, score: 0.13
Wdr90Unknown, score: 0.02
Trip10Unknown, score: 0.18
Ift140Unknown, score: 0.54
VmacUnknown, score: 0.01
Cyp4f15Unknown, score: 0.3
AI413582Unknown, score: 0.12
Rpusd1Unknown, score: 0.15
Ticam1Unknown, score: 0.37
Ttbk1Unknown, score: 0.22
Stap2Unknown, score: 0.02
Tcf19Unknown, score: 0.13
Oard1Unknown, score: 0.2
Unc119bUnknown, score: 0.27
Abhd3Unknown, score: 0.01
Smim3Unknown, score: 0.15
Kctd1Unknown, score: 0.5
Slc39a3Unknown, score: 0.01
Slc39a6Unknown, score: 0.03
Fbxo38Unknown, score: 0.31
LarsUnknown, score: 0.17
Psmg2Unknown, score: 0.16
Lrrtm2Unknown, score: 0.43
GlyatUnknown, score: 0.32
Btaf1Unknown, score: 0.52
Uqcc3Unknown, score: 0.49
Macrod1Unknown, score: 0.62
AC109138.1Unknown, score: 0.26
AI837181Unknown, score: 0.39
Kazald1Unknown, score: 0.1
Otub1Unknown, score: 0.09
Psat1Unknown, score: 0.35
LpxnUnknown, score: 0.62
Trpt1Unknown, score: 0.15
Slc25a45Unknown, score: 0.37
Hat1Unknown, score: 0.07
Guca1bUnknown, score: 0.16
EprsUnknown, score: 0.07
Lgr4Unknown, score: 0.13
Magee1Unknown, score: 0.1
Wwp1Unknown, score: 0.15
MylkUnknown, score: 0.1
Rdh1Unknown, score: 0.48
Nod1Unknown, score: 0.14
Uap1Unknown, score: 0.04
Coro2aUnknown, score: 0.51
Snrpd2Unknown, score: 0.23
Sf3b4Unknown, score: 0.47
Slc12a6Unknown, score: 0.38
Mrpl41Unknown, score: 0.4
Rapgef1Unknown, score: 0.32
Prrxl1Unknown, score: 0.08
HaaoUnknown, score: 0.28
Tm6sf1Unknown, score: 0.14
Whsc1Unknown, score: 0.03
Thoc5Unknown, score: 0.18
Adgrb1Unknown, score: 0.04
Usp9yUnknown, score: 0.2
CthUnknown, score: 0.29
MthfsUnknown, score: 0.06
Chd4Unknown, score: 0.56
Celsr3Unknown, score: 0.34
Pom121Unknown, score: 0.06
Cdk9Unknown, score: 0.63
BreUnknown, score: 0.25
Cdc20Unknown, score: 0.61
Celf4Unknown, score: 0.21
Lin7aUnknown, score: 0.24
Slc14a1Unknown, score: 0.04
Cstf2Unknown, score: 0.01
Grm3Unknown, score: 0.03
Grm7Unknown, score: 0.06
Ltbp4Unknown, score: 0.33
Rnf144aUnknown, score: 0.11
Prkab2Unknown, score: 0.03
Med21Unknown, score: 0.01
Baiap2Unknown, score: 0.09
B3gnt5Unknown, score: 0.06
Eif4ebp3Unknown, score: 0.03
Slco3a1Unknown, score: 0.39
NapgUnknown, score: 0.01
Ak6Unknown, score: 0.19
Sema3dUnknown, score: 0.44
OgtUnknown, score: 0.33
Fam50aUnknown, score: 0.14
Mat2bUnknown, score: 0.15
Foxp1Unknown, score: 0.37
Rnpepl1Unknown, score: 0.33
Ccdc86Unknown, score: 0.14
Cops8Unknown, score: 0.18
Ccdc88aUnknown, score: 0.25
Chn1Unknown, score: 0.02
Fam207aUnknown, score: 0.06
Card11Unknown, score: 0.13
Oxsr1Unknown, score: 0.55
Galnt16Unknown, score: 0.12
Jmjd1cUnknown, score: 0.38
IbtkUnknown, score: 0.29
Rdh13Unknown, score: 0.24
Ankhd1Unknown, score: 0.83
Aif1lUnknown, score: 0.63
Fam72aUnknown, score: 0.37
B4gat1Unknown, score: 0.13
AidaUnknown, score: 0.3
Rnf169Unknown, score: 0.01
Trmt10aUnknown, score: 0.1
Ppp1r15bUnknown, score: 0
Apol7cUnknown, score: 0.05
Irak2Unknown, score: 0.14
TprUnknown, score: 0.55
Fam212bUnknown, score: 0.07
Pfdn4Unknown, score: 0.37
Dnaaf2Unknown, score: 0.92
Exosc4Unknown, score: 0.26
Ints5Unknown, score: 0.27
Rars2Unknown, score: 0.27
Uhrf2Unknown, score: 0.05
MmadhcUnknown, score: 0.15
Plekha5Unknown, score: 0.44
Trip11Unknown, score: 0.03
Fam64aUnknown, score: 0.41
Ms4a7Unknown, score: 0.05
Fam118bUnknown, score: 0.41
Mbd5Unknown, score: 0.21
Kif24Unknown, score: 0.03
RlfUnknown, score: 0.28
Me3Unknown, score: 0.07
Prr5Unknown, score: 0.03
Mybpc1Unknown, score: 0.59
Actr5Unknown, score: 0.22
Prex2Unknown, score: 0.43
Rnf20Unknown, score: 0.1
Pkn2Unknown, score: 0.37
Ankrd39Unknown, score: 0.34
Cald1Unknown, score: 0.24
Upk1aUnknown, score: 0.12
NpyUnknown, score: 0.61
Acy1Unknown, score: 0.03
TxlnaUnknown, score: 0.16
CtrlUnknown, score: 0.2
Cyb5aUnknown, score: 0.09
Ampd2Unknown, score: 0.1
Ank2Unknown, score: 0.24
Arrb1Unknown, score: 0
Actn1Unknown, score: 0.21
Glo1Unknown, score: 0.21
C7Unknown, score: 0.23
BrafUnknown, score: 0.5
Mzf1Unknown, score: 0.05
Zbtb25Unknown, score: 0.23
Art4Unknown, score: 0.2
GusbUnknown, score: 0.46
Syt17Unknown, score: 0.62
Dnah1Unknown, score: 0.11
Nop2Unknown, score: 0.18
Ehmt2Unknown, score: 0.28
Gpr18Unknown, score: 0.12
Slc35b1Unknown, score: 0.04
ManbaUnknown, score: 0.43
GgctUnknown, score: 0.1
FdpsUnknown, score: 0.17
DgkgUnknown, score: 0.82
PgdUnknown, score: 0.22
Tmbim6Unknown, score: 0.01
TriobpUnknown, score: 0.13
Hba-a2Unknown, score: 0.09
Krt7Unknown, score: 0.93
Rap1gapUnknown, score: 0.24
Adrbk1Unknown, score: 0.11
C8bUnknown, score: 0.2
Pde4cUnknown, score: 0.74
QdprUnknown, score: 0.4
PighUnknown, score: 0.1
Ly6aUnknown, score: 0.43
Hivep1Unknown, score: 0.18
Amhr2Unknown, score: 0.16
H2-Q6Unknown, score: 0.14
110558Unknown, score: 0.02
Prdm2Unknown, score: 0.15
Arhgef28Unknown, score: 0.09
Atxn3Unknown, score: 0.14
Rps6ka3Unknown, score: 0.3
Cse1lUnknown, score: 0.5
Adam33Unknown, score: 0.21
Nr3c2Unknown, score: 0.01
Tshz1Unknown, score: 0.02
Srsf1Unknown, score: 0.59
Pwp2Unknown, score: 0.13
PccaUnknown, score: 0.47
Lims1Unknown, score: 0.4
EtfaUnknown, score: 0.57
Scn2a1Unknown, score: 0.01
Scn4aUnknown, score: 0.23
Slc8a3Unknown, score: 0
Hspa13Unknown, score: 0.11
HlcsUnknown, score: 0.37
Rpl10Unknown, score: 0.11
TarsUnknown, score: 0.31
Erc1Unknown, score: 0.34
PecrUnknown, score: 0.55
Hmga1-rs1Unknown, score: 0.38
DxoUnknown, score: 0.04
Egln1Unknown, score: 0.45
C030039L03RikUnknown, score: 0.03
Vmn1r53Unknown, score: 0.74
Kiss1rUnknown, score: 0.05
NpntUnknown, score: 0.01
PalmdUnknown, score: 0.43
Lyve1Unknown, score: 0.05
Clic1Unknown, score: 0.05
Ehbp1l1Unknown, score: 0.01
Zmynd10Unknown, score: 0.08
Tle6Unknown, score: 0.06
Elac1Unknown, score: 0.51
Oas1cUnknown, score: 0.19
Slc13a3Unknown, score: 0.14
Ly6g6dUnknown, score: 0.32
4930444G20RikUnknown, score: 0.08
Gtf2ird2Unknown, score: 0.09
Spred1Unknown, score: 0.2
PawrUnknown, score: 0.11
ProscUnknown, score: 0.29
Dcun1d1Unknown, score: 0.16
Afg3l1Unknown, score: 0.04
Vps4aUnknown, score: 0.29
Mta1Unknown, score: 0.4
Stim2Unknown, score: 0
Alpk3Unknown, score: 0.28
Dph1Unknown, score: 0.27
Slc19a2Unknown, score: 0.27
Fam57aUnknown, score: 0.07
Acsm1Unknown, score: 0.07
TirapUnknown, score: 0.28
Steap4Unknown, score: 0.29
1110038F14RikUnknown, score: 0.39
Bloc1s4Unknown, score: 0.25
Stk33Unknown, score: 0.18
Asb7Unknown, score: 0.36
Slc2a9Unknown, score: 0.67
Klf16Unknown, score: 0.11
Mrps2Unknown, score: 0.05
Mmp28Unknown, score: 0.28
Cspg4Unknown, score: 0.71
Muc4Unknown, score: 0.3
Man2a2Unknown, score: 0.07
HnmtUnknown, score: 0.09
Pofut1Unknown, score: 0.02
Ppp1r3aUnknown, score: 0.04
Ube2j2Unknown, score: 0.24
Acap3Unknown, score: 0.06
Eri3Unknown, score: 0.31
Smc1bUnknown, score: 0.09
Igsf8Unknown, score: 0.48
Plxnb2Unknown, score: 0.33
Ankrd6Unknown, score: 0.11
Elmo1Unknown, score: 0.19
Emid1Unknown, score: 0.2
Col26a1Unknown, score: 0.32
Caskin2Unknown, score: 0.13
Sec63Unknown, score: 0.69
Bmp2kUnknown, score: 0.07
Ttbk2Unknown, score: 0.24
Wdr5Unknown, score: 0.49
Lnx2Unknown, score: 0.05
Dclre1bUnknown, score: 0.21
Zcchc14Unknown, score: 0.25
Asb14Unknown, score: 0
Stard4Unknown, score: 0.03
Stard5Unknown, score: 0.17
Stard6Unknown, score: 0.57
Recql5Unknown, score: 0.53
Snx18Unknown, score: 0.17
Hpcal4Unknown, score: 0.37
Peg10Unknown, score: 0.05
Cdhr1Unknown, score: 0.05
Usp48Unknown, score: 0.27
Cyp4f13Unknown, score: 0.19
Nxf7Unknown, score: 0.03
Rtn4ip1Unknown, score: 0.05
Arr3Unknown, score: 0.1
ParvbUnknown, score: 0.18
Znrf1Unknown, score: 0.01
Xpnpep2Unknown, score: 0.43
Smco4Unknown, score: 0.45
Xpnpep1Unknown, score: 0.02
Bco2Unknown, score: 0.03
Zfp704Unknown, score: 0
Adgrl4Unknown, score: 0.42
Rac3Unknown, score: 0.12
Ripply3Unknown, score: 0.27
Pfkfb3Unknown, score: 0.67
Cd209cUnknown, score: 0.12
Cd209aUnknown, score: 0.24
Crb1Unknown, score: 0.32
Hook2Unknown, score: 0.41
Sumo2Unknown, score: 0.13
Zfp369Unknown, score: 0.03
Myoz3Unknown, score: 0.04
Prima1Unknown, score: 0.56
Il17rcUnknown, score: 0
Fut10Unknown, score: 0.01
Mbnl3Unknown, score: 0.35
Syt12Unknown, score: 0.13
Arhgap4Unknown, score: 0.17
Asic3Unknown, score: 0.07
Acot2Unknown, score: 0.23
Galnt10Unknown, score: 0.06
Acot3Unknown, score: 0.19
Il17rdUnknown, score: 0.39
Gpr37l1Unknown, score: 0.19
ApobrUnknown, score: 0.39
MlphUnknown, score: 0.28
Dicer1Unknown, score: 0.13
SugctUnknown, score: 0.56
Socs7Unknown, score: 0.01
Prpf8Unknown, score: 0.03
Pcdha12Unknown, score: 0.5
Ufsp2Unknown, score: 0.01
Eif4a3Unknown, score: 0.79
Fam195bUnknown, score: 0.32
Rwdd4aUnknown, score: 0.05
FlnaUnknown, score: 0.03
NadkUnknown, score: 0.16
Pkhd1l1Unknown, score: 0.25
Edem1Unknown, score: 0.07
Btnl10Unknown, score: 0.04
Luc7l2Unknown, score: 0.01
Rspo1Unknown, score: 0.06
Tmem47Unknown, score: 0.22
Hexim1Unknown, score: 0.07
Phf21aUnknown, score: 0.12
TmlheUnknown, score: 0.51
Nrbp1Unknown, score: 0.1
Zfp286Unknown, score: 0.35
Wdr81Unknown, score: 0.42
Ttc36Unknown, score: 0.39
Ell2Unknown, score: 0.16
Rfpl4Unknown, score: 0.11
ArhgdiaUnknown, score: 0.02
Abcg4Unknown, score: 0.24
Rassf3Unknown, score: 0.3
NacadUnknown, score: 0.37
PirtUnknown, score: 0.14
BC049762Unknown, score: 0.44
Fam65bUnknown, score: 0.19
Rnf185Unknown, score: 0.21
Hspa1aUnknown, score: 0.59
Abhd16aUnknown, score: 0.87
Kdm4bUnknown, score: 0.33
Mtmr11Unknown, score: 0.17
Vps37dUnknown, score: 0.04
Reps2Unknown, score: 0.03
Slc25a43Unknown, score: 0.38
Pld6Unknown, score: 0.05
Jmjd4Unknown, score: 0.23
Zzef1Unknown, score: 0.35
Tmem199Unknown, score: 0.31
Dcdc2aUnknown, score: 0.38
Gm13152Unknown, score: 0.05
Rbms3Unknown, score: 0.47
Ggt7Unknown, score: 0.04
Larp4Unknown, score: 0.11
Kctd12bUnknown, score: 0.36
Camkk2Unknown, score: 0.21
Tbc1d16Unknown, score: 0.27
Wdr37Unknown, score: 0.12
Gtpbp10Unknown, score: 0.15
Pde2aUnknown, score: 0.44
C2cd2Unknown, score: 0.13
Galnt6Unknown, score: 0.08
Urb1Unknown, score: 0.16
Pif1Unknown, score: 0.34
Dhx37Unknown, score: 0.24
Yeats2Unknown, score: 0.11
BtlaUnknown, score: 0.02
Phldb2Unknown, score: 0.03
ExogUnknown, score: 0.5
Alg1Unknown, score: 0.11
Tmem132cUnknown, score: 0.1
Mob3aUnknown, score: 0.09
Tor1aip1Unknown, score: 0.55
Dot1lUnknown, score: 0.27
Zfp871Unknown, score: 0.04
Rpp40Unknown, score: 0.46
Sgms1Unknown, score: 0.68
1810043H04RikUnknown, score: 0.25
Cep78Unknown, score: 0.1
Nek11Unknown, score: 0.06
Etl4Unknown, score: 0.13
Kntc1Unknown, score: 0.17
Tspan10Unknown, score: 0.42
Slc25a38Unknown, score: 0.02
Akr1d1Unknown, score: 0.04
Dis3l2Unknown, score: 0.35
Prrg3Unknown, score: 0.12
Sde2Unknown, score: 0.49
Sned1Unknown, score: 0.12
Dock3Unknown, score: 0.03
Slc26a7Unknown, score: 0.08
Unc13cUnknown, score: 0.14
Cpeb3Unknown, score: 0.14
Adamts18Unknown, score: 0
Zfp280cUnknown, score: 0.04
Hmgcll1Unknown, score: 0.05
Rbmx2Unknown, score: 0.22
Sirt7Unknown, score: 0.04
Ulk4Unknown, score: 0.31
Vps8Unknown, score: 0.1
Zc3hav1lUnknown, score: 0.38
Tns2Unknown, score: 0.3
Samd9lUnknown, score: 0.02
Ido2Unknown, score: 0.03
Osgin2Unknown, score: 0.04
Enox2Unknown, score: 0.51
GanUnknown, score: 0.08
Igsf1Unknown, score: 0.23
Wfdc6aUnknown, score: 0.2
Eif2b1Unknown, score: 0.25
Itih5Unknown, score: 0.09
Tbc1d12Unknown, score: 0.09
Tyw3Unknown, score: 0.48
Sectm1aUnknown, score: 0.13
Erich3Unknown, score: 0.1
Bend7Unknown, score: 0.54
Kif15Unknown, score: 0.05
Dennd2aUnknown, score: 0.32
Pgbd5Unknown, score: 0.02
B3gntl1Unknown, score: 0.35
MtrrUnknown, score: 0.02
Adcy2Unknown, score: 0.29
Zfp658Unknown, score: 0.12
Zfp719Unknown, score: 0.33
Papd7Unknown, score: 0.05
Zfp180Unknown, score: 0.1
IrgqUnknown, score: 0.36
Slc30a6Unknown, score: 0.12
Zkscan2Unknown, score: 0.18
Zfp526Unknown, score: 0
Lrch2Unknown, score: 0.28
Nckap5Unknown, score: 0.02
Zfp677Unknown, score: 0.16
Tdrd6Unknown, score: 0.43
Pamr1Unknown, score: 0.14
McmbpUnknown, score: 0.02
MkxUnknown, score: 0.19
Brcc3Unknown, score: 0.13
Tbc1d4Unknown, score: 0.06
Zfp947Unknown, score: 0.05
Gltscr1lUnknown, score: 0.24
D15Ertd621eUnknown, score: 0.07
Trim41Unknown, score: 0.05
Alkbh1Unknown, score: 0.24
D130040H23RikUnknown, score: 0.33
Churc1Unknown, score: 0.14
Vmn2r15Unknown, score: 0.1
Lrrc25Unknown, score: 0.03
Mtrf1Unknown, score: 0.06
Cln5Unknown, score: 0.06
Pank3Unknown, score: 0.09
6720489N17RikUnknown, score: 0.01
SuoxUnknown, score: 0.38
Mtss1Unknown, score: 0.17
Tsga10Unknown, score: 0.13
AdoUnknown, score: 0.09
Ccdc114Unknown, score: 0.23
MrgprfUnknown, score: 0.07
Ptchd1Unknown, score: 0.1
Cspp1Unknown, score: 0.01
Mgst2Unknown, score: 0.05
Pcdh9Unknown, score: 0.15
Vstm2aUnknown, score: 0.45
Dennd6aUnknown, score: 0.19
Dsg1cUnknown, score: 0.55
Ccdc73Unknown, score: 0.39
Pde12Unknown, score: 0.2
Asxl3Unknown, score: 0.24
Zfyve26Unknown, score: 0.24
Tmem18Unknown, score: 0.12
Hk3Unknown, score: 0
Syne3Unknown, score: 0.46
Nhlrc3Unknown, score: 0.32
Dcaf15Unknown, score: 0.41
Proser1Unknown, score: 0.03
GsapUnknown, score: 0.25
Ubxn10Unknown, score: 0.06
Ccdc110Unknown, score: 0.5
HjurpUnknown, score: 0.11
9330159F19RikUnknown, score: 0.24
Fam193bUnknown, score: 0.32
PaoxUnknown, score: 0.29
SprnUnknown, score: 0.35
Gm266Unknown, score: 0.22
BC027231Unknown, score: 0.57
Iffo2Unknown, score: 0.5
Aldh4a1Unknown, score: 0.25
Catsper2Unknown, score: 0.45
Mars2Unknown, score: 0.13
Satb2Unknown, score: 0.26
DseUnknown, score: 0.12
Fam46aUnknown, score: 0.14
Athl1Unknown, score: 0.18
Slc45a3Unknown, score: 0.2
Scfd2Unknown, score: 0.36
Wbscr17Unknown, score: 0.07
Tnpo2Unknown, score: 0.02
Zfp583Unknown, score: 0.32
Abhd10Unknown, score: 0.11
Pdlim2Unknown, score: 0.12
Fam126bUnknown, score: 0.06
Wdr19Unknown, score: 0.31
Ankrd35Unknown, score: 0.14
TapbplUnknown, score: 0.26
Dnd1Unknown, score: 0.1
Txndc2Unknown, score: 0.04
Scyl2Unknown, score: 0.35
Rassf4Unknown, score: 0.13
Armc2Unknown, score: 0.24
Klhdc8aUnknown, score: 0.06
Gpr174Unknown, score: 0.26
DstykUnknown, score: 0.13
Rbbp5Unknown, score: 0.14
Lgi3Unknown, score: 0.24
Fbxo42Unknown, score: 0.03
Plekhh2Unknown, score: 0.38
Map9Unknown, score: 0.24
Zfp598Unknown, score: 0.1
PreplUnknown, score: 0.58
Atg9bUnknown, score: 0.19
Fam83fUnknown, score: 0.06
Fbxw10Unknown, score: 0.02
Tnrc6bUnknown, score: 0.63
Tmem82Unknown, score: 0.22
Megf11Unknown, score: 0.14
Kmt2aUnknown, score: 0.08
A430105I19RikUnknown, score: 0.02
Etnk2Unknown, score: 0.13
SynaUnknown, score: 0.22
Gm4787Unknown, score: 0.4
Lrrc1Unknown, score: 0.45
Gm4788Unknown, score: 0.08
CilpUnknown, score: 0.03
Cdc73Unknown, score: 0.12
Cep164Unknown, score: 0.56
Prmt7Unknown, score: 0.1
Aldh5a1Unknown, score: 0.16
PstkUnknown, score: 0.01
Duox2Unknown, score: 0.01
Cped1Unknown, score: 0.22
Slc25a29Unknown, score: 0.23
L3mbtl2Unknown, score: 0.3
ChadlUnknown, score: 0.06
IqubUnknown, score: 0.12
Mb21d1Unknown, score: 0.07
Edrf1Unknown, score: 0.01
Mmp21Unknown, score: 0.02
Arid5aUnknown, score: 0.1
Chtf18Unknown, score: 0.55
Fam173aUnknown, score: 0.16
Fbxl16Unknown, score: 0.44
Mob3bUnknown, score: 0.36
Rhbdl1Unknown, score: 0.15
Sema6dUnknown, score: 0.04
Chtf8Unknown, score: 0.24
Wfikkn1Unknown, score: 0.11
Bud13Unknown, score: 0.03
ManealUnknown, score: 0.27
Hip1Unknown, score: 0.34
DiexfUnknown, score: 0.06
Kri1Unknown, score: 0.04
Trmt2bUnknown, score: 0.27
Tmem120aUnknown, score: 0.09
Traf3ip3Unknown, score: 0.17
Wipf1Unknown, score: 0.45
Camk1gUnknown, score: 0.17
Senp6Unknown, score: 0.1
NcaphUnknown, score: 0.18
Slc35e3Unknown, score: 0.31
Entpd3Unknown, score: 0.01
Gpat2Unknown, score: 0.46
Prr14lUnknown, score: 0.03
A3galt2Unknown, score: 0.02
RnpepUnknown, score: 0.02
Zmat1Unknown, score: 0.36
Arrdc1Unknown, score: 0.01
Fam73aUnknown, score: 0.33
Mfsd6lUnknown, score: 0.28
Ginm1Unknown, score: 0.02
Adgrg6Unknown, score: 0.25
Ccdc28aUnknown, score: 0.74
Fam26fUnknown, score: 0.52
Lace1Unknown, score: 0.07
Micu1Unknown, score: 0.14
Stox1Unknown, score: 0.17
Ube2d1Unknown, score: 0
YbeyUnknown, score: 0.1
PdxkUnknown, score: 0.47
Shc2Unknown, score: 0.39
Cdc34Unknown, score: 0.43
Lppr3Unknown, score: 0.73
Med16Unknown, score: 0.45
Wdr18Unknown, score: 0.22
Tmem259Unknown, score: 0.09
Sbno2Unknown, score: 0.73
Plk5Unknown, score: 0.36
Abhd17aUnknown, score: 0.28
AU041133Unknown, score: 0.17
Aldh1l2Unknown, score: 0.16
Appl2Unknown, score: 0.01
Ckap4Unknown, score: 0.34
Cep290Unknown, score: 0.13
Mettl25Unknown, score: 0.03
Tmem5Unknown, score: 0.19
March9Unknown, score: 0.19
Agap2Unknown, score: 0
Os9Unknown, score: 0.04
Slc26a10Unknown, score: 0.29
MarsUnknown, score: 0.18
Gls2Unknown, score: 0.31
Myl6bUnknown, score: 0.4
Ccm2Unknown, score: 0.06
AftphUnknown, score: 0.01
Ccdc85aUnknown, score: 0.45
Cfap36Unknown, score: 0.03
4931440F15RikUnknown, score: 0.17
Fnip1Unknown, score: 0.12
Mfap3Unknown, score: 0.25
Mrpl22Unknown, score: 0.46
Iba57Unknown, score: 0.32
Nlrp3Unknown, score: 0.55
FlcnUnknown, score: 0.11
Dhrs7bUnknown, score: 0.07
Tmem11Unknown, score: 0.24
Mmgt2Unknown, score: 0.07
Usp43Unknown, score: 0.37
Chd3Unknown, score: 0.07
Kdm6bUnknown, score: 0.57
Kctd11Unknown, score: 0.05
Acap1Unknown, score: 0.38
Neurl4Unknown, score: 0.45
Arrb2Unknown, score: 0.09
Gltpd2Unknown, score: 0.17
Camta2Unknown, score: 0.14
Dhx33Unknown, score: 0.39
Spns2Unknown, score: 0.2
Git1Unknown, score: 0.29
Taok1Unknown, score: 0.01
Proca1Unknown, score: 0.2
Utp6Unknown, score: 0.07
Adap2Unknown, score: 0.16
Nle1Unknown, score: 0.29
SynrgUnknown, score: 0.02
Tada2aUnknown, score: 0.18
Ptrh2Unknown, score: 0.17
Trim25Unknown, score: 0.18
Gm525Unknown, score: 0.09
HlfUnknown, score: 0.1
Utp18Unknown, score: 0.37
Spata20Unknown, score: 0.43
Kat7Unknown, score: 0.08
Prr15lUnknown, score: 0.75
Scrn2Unknown, score: 0
Gpr179Unknown, score: 0.2
Cisd3Unknown, score: 0
Stac2Unknown, score: 0.01
Tns4Unknown, score: 0.2
Klhl11Unknown, score: 0.62
Plekhh3Unknown, score: 0.04
BC030867Unknown, score: 0.12
Atxn7l3Unknown, score: 0.24
Abca5Unknown, score: 0.02
Cd300ldUnknown, score: 0.19
Cd300eUnknown, score: 0.12
Slc16a5Unknown, score: 0.8
Llgl2Unknown, score: 0.32
UnkUnknown, score: 0.12
Fbf1Unknown, score: 0.22
Rnf157Unknown, score: 0.03
Qrich2Unknown, score: 0.31
Ube2oUnknown, score: 0.25
Tnrc6cUnknown, score: 0.18
Tmc6Unknown, score: 0.36
Tmc8Unknown, score: 0.51
Nploc4Unknown, score: 0.04
Lrrc45Unknown, score: 0.44
Uts2rUnknown, score: 0.17
BC017643Unknown, score: 0.14
Ubxn2aUnknown, score: 0.24
Nol10Unknown, score: 0.43
Trappc12Unknown, score: 0.02
Ankmy2Unknown, score: 0.08
MbipUnknown, score: 0.01
Mgat2Unknown, score: 0.53
L2hgdhUnknown, score: 0.32
Sipa1l1Unknown, score: 0.17
Zfyve1Unknown, score: 0.08
Fam161bUnknown, score: 0.36
Mfsd7cUnknown, score: 0.07
CipcUnknown, score: 0.44
Tmem63cUnknown, score: 0.24
Ahsa1Unknown, score: 0.01
Ism2Unknown, score: 0.05
Lysmd1Unknown, score: 0.02
Kcnk13Unknown, score: 0.35
Nrde2Unknown, score: 0.31
9030617O03RikUnknown, score: 0.14
Rin3Unknown, score: 0.2
Eif5Unknown, score: 0.18
Rd3lUnknown, score: 0.16
BC022687Unknown, score: 0.16
Wdr60Unknown, score: 0.04
Mboat1Unknown, score: 0.24
GmdsUnknown, score: 0.12
Phactr1Unknown, score: 0.05
Nup153Unknown, score: 0
Kdm1bUnknown, score: 0.54
Rnf144bUnknown, score: 0.25
Fam120aUnknown, score: 0.23
B4galt7Unknown, score: 0
Zfp595Unknown, score: 0.01
Ice1Unknown, score: 0.09
Clptm1lUnknown, score: 0.23
Zfyve16Unknown, score: 0.18
Fcho2Unknown, score: 0.22
Mier3Unknown, score: 0.1
RarbUnknown, score: 0.01
Ube2e2Unknown, score: 0.18
Sec24cUnknown, score: 0.29
Sema3gUnknown, score: 0.13
Dlgap5Unknown, score: 0.34
Tmem260Unknown, score: 0.35
Ttc5Unknown, score: 0.37
Haus4Unknown, score: 0.25
Phf11dUnknown, score: 0.27
Scara3Unknown, score: 0.35
Akap11Unknown, score: 0.23
Vwa8Unknown, score: 0.01
Stk24Unknown, score: 0.24
DapUnknown, score: 0.13
AbraUnknown, score: 0.15
Fam49bUnknown, score: 0.62
Them6Unknown, score: 0.12
Zc3h3Unknown, score: 0.28
Nrbp2Unknown, score: 0.18
Mroh1Unknown, score: 0.54
Lrrc14Unknown, score: 0.27
Arhgap39Unknown, score: 0.19
Apol9aUnknown, score: 0.11
Ankrd54Unknown, score: 0.17
Tmem184bUnknown, score: 0.38
Tomm22Unknown, score: 0.44
Mkl1Unknown, score: 0.64
Ttll12Unknown, score: 0.28
5031439G07RikUnknown, score: 0.08
CerkUnknown, score: 0.56
Zbed4Unknown, score: 0.42
Alg12Unknown, score: 0.54
Pim3Unknown, score: 0.34
Adamts20Unknown, score: 0.09
Spryd3Unknown, score: 0.01
AaasUnknown, score: 0.24
Rmi2Unknown, score: 0.03
Fgd4Unknown, score: 0.55
Tmem191cUnknown, score: 0.26
Pi4kaUnknown, score: 0.38
Slc7a4Unknown, score: 0.07
Scarf2Unknown, score: 0.02
Cyp2ab1Unknown, score: 0.16
Atp13a3Unknown, score: 0.06
Lsg1Unknown, score: 0.17
Pak2Unknown, score: 0.14
Golgb1Unknown, score: 0.07
Cldnd1Unknown, score: 0.01
224273Unknown, score: 0.12
Scaf4Unknown, score: 0.47
Tfb1mUnknown, score: 0.06
Zfp758Unknown, score: 0.13
Flywch1Unknown, score: 0.25
Tbc1d24Unknown, score: 0.1
Traf7Unknown, score: 0.32
Rab40cUnknown, score: 0.47
Bnip1Unknown, score: 0.4
Uhrf1bp1Unknown, score: 0.3
Anks1Unknown, score: 0.12
Slc26a8Unknown, score: 0.02
Btbd9Unknown, score: 0.12
Slc37a1Unknown, score: 0.12
Adamts10Unknown, score: 0.47
March2Unknown, score: 0.07
Vps52Unknown, score: 0.29
Bag6Unknown, score: 0.01
Enpp4Unknown, score: 0.24
Clic5Unknown, score: 0.24
Aars2Unknown, score: 0.02
Tmem63bUnknown, score: 0.31
Lrrc73Unknown, score: 0.53
Rrp36Unknown, score: 0.22
Pex6Unknown, score: 0.07
224829Unknown, score: 0.01
Usp49Unknown, score: 0.15
Treml4Unknown, score: 0.53
Dpp9Unknown, score: 0.04
SafbUnknown, score: 0.51
2410015M20RikUnknown, score: 0.25
Dus3lUnknown, score: 0.1
Pja2Unknown, score: 0.31
Fez2Unknown, score: 0.18
Map4k3Unknown, score: 0.32
Fbxo11Unknown, score: 0.06
Rbbp8Unknown, score: 0.21
Zfp521Unknown, score: 0
Rprd1aUnknown, score: 0.11
Lims2Unknown, score: 0.08
Etf1Unknown, score: 0.64
Sra1Unknown, score: 0.08
Ticam2Unknown, score: 0.22
Pde6aUnknown, score: 0.14
Alpk2Unknown, score: 0.26
Mppe1Unknown, score: 0.08
Slmo1Unknown, score: 0.08
St8sia5Unknown, score: 0.19
Haus1Unknown, score: 0.09
Zadh2Unknown, score: 0.2
Cd226Unknown, score: 0.07
Ppp2r5bUnknown, score: 0.22
Npas4Unknown, score: 0.44
Kdm2aUnknown, score: 0.05
BC021614Unknown, score: 0.3
Suv420h1Unknown, score: 0.31
Ubxn1Unknown, score: 0.23
MyrfUnknown, score: 0.32
Cyb561a3Unknown, score: 0.28
Patl1Unknown, score: 0.25
Nmrk1Unknown, score: 0.3
Abhd17bUnknown, score: 0.3
Smc5Unknown, score: 0.4
Tmem252Unknown, score: 0.48
Cyp2c70Unknown, score: 0.19
Morn4Unknown, score: 0.26
Cox15Unknown, score: 0.05
Erlin1Unknown, score: 0.06
Fam178aUnknown, score: 0.15
Peo1Unknown, score: 0.21
DpcdUnknown, score: 0.1
Pprc1Unknown, score: 0.05
Wbp1lUnknown, score: 0.03
InaUnknown, score: 0.03
Taf5Unknown, score: 0.41
Habp2Unknown, score: 0.19
Epb4.1l5Unknown, score: 0.15
Zranb3Unknown, score: 0.08
R3hdm1Unknown, score: 0.1
LctUnknown, score: 0.03
5430435G22RikUnknown, score: 0.02
Rab29Unknown, score: 0.14
BC003331Unknown, score: 0.11
Smg7Unknown, score: 0.03
Rasal2Unknown, score: 0.33
BC026585Unknown, score: 0.2
Klhl20Unknown, score: 0.09
Prrc2cUnknown, score: 0.06
Fmo4Unknown, score: 0.21
TiprlUnknown, score: 0.25
Atf6Unknown, score: 0.25
Arhgap30Unknown, score: 0.3
Ahctf1Unknown, score: 0.03
Wdr26Unknown, score: 0.15
C130074G19RikUnknown, score: 0.08
Slc30a10Unknown, score: 0.18
Lyplal1Unknown, score: 0.25
Kctd3Unknown, score: 0.28
Smyd2Unknown, score: 0.15
Vash2Unknown, score: 0.05
Lpgat1Unknown, score: 0.27
HhatUnknown, score: 0
Kcnq5Unknown, score: 0.12
Arhgef4Unknown, score: 0.07
Plekhb2Unknown, score: 0.2
Kansl3Unknown, score: 0.09
Tmem194bUnknown, score: 0.09
Pms1Unknown, score: 0.02
StradbUnknown, score: 0.1
Ino80dUnknown, score: 0.18
Gpbar1Unknown, score: 0.31
AampUnknown, score: 0.04
Ctdsp1Unknown, score: 0.19
Gigyf2Unknown, score: 0.03
Fam132bUnknown, score: 0.14
Ppip5k2Unknown, score: 0.04
Dclre1cUnknown, score: 0.06
Proser2Unknown, score: 0.09
C1ql3Unknown, score: 0.07
Tor4aUnknown, score: 0.14
Tubb4bUnknown, score: 0.65
Tmem203Unknown, score: 0.15
Uap1l1Unknown, score: 0.33
Obp2aUnknown, score: 0.34
Camsap1Unknown, score: 0.51
Qsox2Unknown, score: 0.51
Snapc4Unknown, score: 0.09
Sec16aUnknown, score: 0.11
Slc2a6Unknown, score: 0.05
Gbgt1Unknown, score: 0.04
Ddx31Unknown, score: 0.51
Trub2Unknown, score: 0.45
Coq4Unknown, score: 0.33
Zer1Unknown, score: 0.33
D2Wsu81eUnknown, score: 0.19
Phyhd1Unknown, score: 0.48
DolkUnknown, score: 0.45
Nup188Unknown, score: 0.24
Exosc2Unknown, score: 0.24
QrfpUnknown, score: 0.19
Nup214Unknown, score: 0.26
Ppapdc3Unknown, score: 0.46
Fam129bUnknown, score: 0.43
Lrsam1Unknown, score: 0.18
Mapkap1Unknown, score: 0.19
RabepkUnknown, score: 0.26
Rabgap1Unknown, score: 0.2
Ccdc148Unknown, score: 0.03
Pkp4Unknown, score: 0.24
Pdk1Unknown, score: 0.47
Zdhhc5Unknown, score: 0.09
Tnks1bp1Unknown, score: 0.25
MaddUnknown, score: 0.06
1110051M20RikUnknown, score: 0.18
Arhgap1Unknown, score: 0.07
Ambra1Unknown, score: 0.04
Prrg4Unknown, score: 0.15
Zfp770Unknown, score: 0.25
RhovUnknown, score: 0.54
Vps18Unknown, score: 0.11
F830045P16RikUnknown, score: 0.35
Ebf4Unknown, score: 0.16
MavsUnknown, score: 0.22
Btbd3Unknown, score: 0.18
Csrp2bpUnknown, score: 0.55
KizUnknown, score: 0.27
Sdcbp2Unknown, score: 0.13
Psmf1Unknown, score: 0.45
Trib3Unknown, score: 0.11
Mylk2Unknown, score: 0.18
Bpifb1Unknown, score: 0.2
Phf20Unknown, score: 0.2
Tgif2Unknown, score: 0.21
D630003M21RikUnknown, score: 0.04
Gdap1l1Unknown, score: 0.16
Fitm2Unknown, score: 0.46
Pcif1Unknown, score: 0.25
Ncoa5Unknown, score: 0.24
Zfp334Unknown, score: 0.2
Tshz2Unknown, score: 0.14
Stx16Unknown, score: 0.12
Npepl1Unknown, score: 0.31
Ppp1r3dUnknown, score: 0.06
Taf4aUnknown, score: 0.13
Osbpl2Unknown, score: 0.11
Ythdf1Unknown, score: 0.14
Arfgap1Unknown, score: 0.06
Helz2Unknown, score: 0.32
Gmeb2Unknown, score: 0.17
ZgpatUnknown, score: 0.38
Ythdf3Unknown, score: 0.32
Acad9Unknown, score: 0.07
Nudt6Unknown, score: 0.15
Spg20Unknown, score: 0.02
Clrn1Unknown, score: 0.1
CtsoUnknown, score: 0.27
Fhdc1Unknown, score: 0.28
Fam160a1Unknown, score: 0.06
Slc25a44Unknown, score: 0.12
Syt11Unknown, score: 0.23
Pbxip1Unknown, score: 0.38
Ints3Unknown, score: 0.17
PogzUnknown, score: 0.01
Golph3lUnknown, score: 0.39
BC028528Unknown, score: 0.05
Pias3Unknown, score: 0.33
Csde1Unknown, score: 0.03
St7lUnknown, score: 0.12
Chil5Unknown, score: 0.08
CymUnknown, score: 0.09
Slc6a17Unknown, score: 0.18
Strip1Unknown, score: 0.41
Ahcyl1Unknown, score: 0.01
Amigo1Unknown, score: 0.05
5330417C22RikUnknown, score: 0.22
Gbp7Unknown, score: 0.17
Znhit6Unknown, score: 0.05
Ak5Unknown, score: 0.14
Ddx58Unknown, score: 0.74
Ndufb6Unknown, score: 0.51
Nol6Unknown, score: 0.2
N28178Unknown, score: 0.11
Arhgef39Unknown, score: 0.04
Car9Unknown, score: 0.52
Npr2Unknown, score: 0.2
Zbtb5Unknown, score: 0.2
Slc25a51Unknown, score: 0.14
ShbUnknown, score: 0.07
Galnt12Unknown, score: 0.03
Zfp189Unknown, score: 0.49
AldobUnknown, score: 0.08
E130308A19RikUnknown, score: 0.4
Acer2Unknown, score: 0.58
Usp1Unknown, score: 0.04
Ttc22Unknown, score: 0.45
Pars2Unknown, score: 0.26
Yipf1Unknown, score: 0.13
Nrd1Unknown, score: 0.06
Efcab14Unknown, score: 0.24
Atpaf1Unknown, score: 0.33
Tmem69Unknown, score: 0.12
Tesk2Unknown, score: 0.67
Ipo13Unknown, score: 0.05
Kdm4aUnknown, score: 0.08
Tmem125Unknown, score: 0.03
AU022252Unknown, score: 0.08
Zmpste24Unknown, score: 0.35
Nt5c1aUnknown, score: 0.31
Pabpc4Unknown, score: 0.19
Rhbdl2Unknown, score: 0.26
Gnl2Unknown, score: 0.2
Oscp1Unknown, score: 0.39
5730409E04RikUnknown, score: 0.14
Zfp362Unknown, score: 0.14
Fam167bUnknown, score: 0.26
IqccUnknown, score: 0.17
Tmem39bUnknown, score: 0.08
Adgrb2Unknown, score: 0.34
Hcrtr1Unknown, score: 0.11
Serinc2Unknown, score: 0.41
Themis2Unknown, score: 0.02
Fam76aUnknown, score: 0.28
Ahdc1Unknown, score: 0.4
Wdtc1Unknown, score: 0.53
PigvUnknown, score: 0.22
Man1c1Unknown, score: 0.16
Grhl3Unknown, score: 0.15
Il22ra1Unknown, score: 0.58
Asap3Unknown, score: 0.13
Eif4g3Unknown, score: 0.01
Sh2d5Unknown, score: 0.18
Emc1Unknown, score: 0.01
CroccUnknown, score: 0.07
Vps13dUnknown, score: 0.09
NppaUnknown, score: 0.06
TardbpUnknown, score: 0.38
Gm572Unknown, score: 0.42
Phf13Unknown, score: 0.03
Cep104Unknown, score: 0.19
Megf6Unknown, score: 0.14
Arhgef16Unknown, score: 0.28
Tnfrsf14Unknown, score: 0.18
9430015G10RikUnknown, score: 0.04
Plekhn1Unknown, score: 0.61
Klhl17Unknown, score: 0
Galnt11Unknown, score: 0.08
Kmt2cUnknown, score: 0.31
Insig1Unknown, score: 0.64
HadhbUnknown, score: 0.08
Haus3Unknown, score: 0.16
Fam193aUnknown, score: 0.04
Tnip2Unknown, score: 0.49
Tada2bUnknown, score: 0.13
Tapt1Unknown, score: 0.54
Guf1Unknown, score: 0.07
Lrrc66Unknown, score: 0.29
Ugt2b36Unknown, score: 0.04
Grsf1Unknown, score: 0.1
Parm1Unknown, score: 0.04
Fras1Unknown, score: 0.26
Paqr3Unknown, score: 0.31
Lin54Unknown, score: 0.02
Plac8Unknown, score: 0.26
Rpap2Unknown, score: 0.13
GakUnknown, score: 0.26
Slc26a1Unknown, score: 0.14
Oasl1Unknown, score: 0.34
Vsig10Unknown, score: 0.42
Fbxo21Unknown, score: 0.36
Fbxw8Unknown, score: 0.4
Trafd1Unknown, score: 0.01
Naa25Unknown, score: 0.25
Fam109aUnknown, score: 0.14
Rad9bUnknown, score: 0.02
Rimbp2Unknown, score: 0.11
SfswapUnknown, score: 0.29
Lrch4Unknown, score: 0.14
Agfg2Unknown, score: 0.14
PilraUnknown, score: 0.39
Adap1Unknown, score: 0
Tmem184aUnknown, score: 0.19
Snx8Unknown, score: 0.58
Brat1Unknown, score: 0.07
Amz1Unknown, score: 0.23
RadilUnknown, score: 0.13
Tnrc18Unknown, score: 0.14
Fbxl18Unknown, score: 0.28
Zfp12Unknown, score: 0.16
E130309D02RikUnknown, score: 0
DaglbUnknown, score: 0.03
Ccz1Unknown, score: 0.19
Bud31Unknown, score: 0.04
Ccser1Unknown, score: 0.08
Thnsl2Unknown, score: 0.01
Mat2aUnknown, score: 0.15
Mob1aUnknown, score: 0.58
Paip2bUnknown, score: 0.28
Smyd5Unknown, score: 0.2
Arhgap25Unknown, score: 0.38
HmcesUnknown, score: 0.31
Txnrd3Unknown, score: 0.04
Iqsec1Unknown, score: 0.28
Ccdc174Unknown, score: 0.05
Ppp4r2Unknown, score: 0.08
Slc6a1Unknown, score: 0.23
Vgll4Unknown, score: 0.25
Wnk1Unknown, score: 0.42
C1rlUnknown, score: 0.11
Klrb1fUnknown, score: 0.27
Clec2eUnknown, score: 0.03
Clec9aUnknown, score: 0.09
Gprc5aUnknown, score: 0.23
H2afjUnknown, score: 0.26
RergUnknown, score: 0.2
Pyroxd1Unknown, score: 0.03
Stk38lUnknown, score: 0.25
Mrps35Unknown, score: 0.11
Ccdc136Unknown, score: 0.07
Tspan33Unknown, score: 0.33
Tcaf2Unknown, score: 0.27
Zfp212Unknown, score: 0.11
OscarUnknown, score: 0.28
Cnot3Unknown, score: 0.3
Ppp1r12cUnknown, score: 0.24
Shisa7Unknown, score: 0.25
Zfp628Unknown, score: 0.07
Ccdc106Unknown, score: 0.06
Zfp954Unknown, score: 0
Zscan22Unknown, score: 0.29
Arhgap35Unknown, score: 0.37
Ap2s1Unknown, score: 0.43
Ccdc61Unknown, score: 0.41
MypopUnknown, score: 0.09
Klc3Unknown, score: 0.05
Mark4Unknown, score: 0.34
Bloc1s3Unknown, score: 0.47
Zfp114Unknown, score: 0.2
Zfp428Unknown, score: 0.14
Atp1a3Unknown, score: 0.49
Zfp574Unknown, score: 0.03
B3gnt8Unknown, score: 0.17
B9d2Unknown, score: 0.03
ItpkcUnknown, score: 0.28
BlvrbUnknown, score: 0.29
Hipk4Unknown, score: 0.21
Pak4Unknown, score: 0.22
Fbxo27Unknown, score: 0.01
Rasgrp4Unknown, score: 0.14
Zfp790Unknown, score: 0.09
Wdr62Unknown, score: 0.23
Alkbh6Unknown, score: 0.21
Syne4Unknown, score: 0.3
Lrfn3Unknown, score: 0.07
Arhgap33Unknown, score: 0.22
U2af1l4Unknown, score: 0.22
Ffar2Unknown, score: 0.14
4931406P16RikUnknown, score: 0.08
SiglecfUnknown, score: 0.37
Mybpc2Unknown, score: 0.31
Tbc1d17Unknown, score: 0.27
Scaf1Unknown, score: 0.28
Luzp2Unknown, score: 0.09
Nipa1Unknown, score: 0.01
Prc1Unknown, score: 0.2
Zfp592Unknown, score: 0.08
PicalmUnknown, score: 0.15
Mogat2Unknown, score: 0.18
P2ry6Unknown, score: 0.4
Dchs1Unknown, score: 0.4
Ipo7Unknown, score: 0.46
Smg1Unknown, score: 0.05
Dcun1d3Unknown, score: 0.13
BC030336Unknown, score: 0.3
Vwa3aUnknown, score: 0.39
Cog7Unknown, score: 0.01
Slc5a11Unknown, score: 0.13
D430042O09RikUnknown, score: 0.51
TufmUnknown, score: 0.06
Atxn2lUnknown, score: 0.01
Hirip3Unknown, score: 0.01
Sez6l2Unknown, score: 0.14
Asphd1Unknown, score: 0.16
Zfp553Unknown, score: 0.01
Zfp764Unknown, score: 0.2
Rnf40Unknown, score: 0.02
Fbxl19Unknown, score: 0.19
Zfp646Unknown, score: 0.04
FusUnknown, score: 0.53
Armc5Unknown, score: 0.13
Ppfia1Unknown, score: 0.1
Arglu1Unknown, score: 0.21
Pcid2Unknown, score: 0.1
Whsc1l1Unknown, score: 0.06
Sorbs2Unknown, score: 0.33
Gpm6aUnknown, score: 0
Zfp930Unknown, score: 0.03
Zfp868Unknown, score: 0.56
Gatad2aUnknown, score: 0.02
Tmem161aUnknown, score: 0.58
Sugp2Unknown, score: 0.32
Ddx49Unknown, score: 0.06
Klhl26Unknown, score: 0.03
Mpv17l2Unknown, score: 0.43
Ccdc124Unknown, score: 0.08
Ankle1Unknown, score: 0.09
Zfp961Unknown, score: 0.16
Inpp4bUnknown, score: 0.11
Heatr3Unknown, score: 0.06
Ces1fUnknown, score: 0.01
Ccdc102aUnknown, score: 0.14
Ndrg4Unknown, score: 0.09
Cnot1Unknown, score: 0.38
Ces2bUnknown, score: 0.45
Ces2eUnknown, score: 0.4
Elmo3Unknown, score: 0.34
Lrrc29Unknown, score: 0.6
Fhod1Unknown, score: 0.4
RltprUnknown, score: 0.58
Edc4Unknown, score: 0.31
Nrn1lUnknown, score: 0.26
Zfp612Unknown, score: 0.17
Cmtr2Unknown, score: 0.18
Vac14Unknown, score: 0.46
FukUnknown, score: 0.25
AarsUnknown, score: 0.6
Rfwd3Unknown, score: 0.17
Tmem231Unknown, score: 0.16
Plcg2Unknown, score: 0.26
6430548M08RikUnknown, score: 0.41
Klhdc4Unknown, score: 0.19
Piezo1Unknown, score: 0.33
Spg7Unknown, score: 0.49
Chmp1aUnknown, score: 0.1
Ttc13Unknown, score: 0.22
Gucy1a2Unknown, score: 0.1
Mmp27Unknown, score: 0.21
Med17Unknown, score: 0.17
Slc36a4Unknown, score: 0.06
Zfp426Unknown, score: 0.37
PpanUnknown, score: 0.11
Atg4dUnknown, score: 0.22
Kank2Unknown, score: 0.58
Tmem205Unknown, score: 0.18
Zfp809Unknown, score: 0.25
Zfp599Unknown, score: 0.15
Igsf9bUnknown, score: 0.05
Zbtb44Unknown, score: 0.3
NfrkbUnknown, score: 0.2
Tmem45bUnknown, score: 0.25
Scn3bUnknown, score: 0.29
Gramd1bUnknown, score: 0.02
Sc5dUnknown, score: 0.16
Rnf214Unknown, score: 0.08
Zbtb16Unknown, score: 0.21
Usp28Unknown, score: 0.12
Rab8bUnknown, score: 0.18
Zfp280dUnknown, score: 0.01
PrtgUnknown, score: 0.37
Leo1Unknown, score: 0.15
Gk5Unknown, score: 0
Atp2c1Unknown, score: 0.05
GlyctkUnknown, score: 0.48
Parp3Unknown, score: 0.23
6430571L13RikUnknown, score: 0.23
ApehUnknown, score: 0.25
AtripUnknown, score: 0.38
Plxnb1Unknown, score: 0.51
Setd2Unknown, score: 0.59
Nbeal2Unknown, score: 0.22
Rtp3Unknown, score: 0.22
Zfp825Unknown, score: 0.28
Alms1Unknown, score: 0.3
Phf11aUnknown, score: 0.03
Ago1Unknown, score: 0.18
Gbp9Unknown, score: 0.08
Spry3Unknown, score: 0.14
Sytl5Unknown, score: 0.14
Slc9a7Unknown, score: 0.37
Rbm10Unknown, score: 0.09
Arhgef9Unknown, score: 0.17
Ofd1Unknown, score: 0.46
Lrp11Unknown, score: 0.11
Sh3rf3Unknown, score: 0.4
Lrrc3Unknown, score: 0.05
C2cd4cUnknown, score: 0.49
Mex3dUnknown, score: 0.29
Zfp938Unknown, score: 0.31
TrhdeUnknown, score: 0.08
MyrflUnknown, score: 0.39
Lrrc10Unknown, score: 0.05
Ankrd52Unknown, score: 0.26
Pla2g3Unknown, score: 0.17
Npc1l1Unknown, score: 0.16
Eml6Unknown, score: 0.17
Zfp454Unknown, score: 0.19
Col23a1Unknown, score: 0.41
Zfp867Unknown, score: 0.26
Smcr8Unknown, score: 0.41
Rtn4rl1Unknown, score: 0.16
Ssh2Unknown, score: 0.14
Atad5Unknown, score: 0.38
Brip1Unknown, score: 0.18
Rsad1Unknown, score: 0.47
Phospho1Unknown, score: 0.08
Aoc2Unknown, score: 0.08
Cdr2lUnknown, score: 0
Fscn2Unknown, score: 0.28
HexdcUnknown, score: 0.1
Fn3krpUnknown, score: 0.03
ApobUnknown, score: 0.16
Kcns3Unknown, score: 0.22
Arid4aUnknown, score: 0.2
Tmem30bUnknown, score: 0.01
Akap5Unknown, score: 0.04
Elmsan1Unknown, score: 0.28
Vash1Unknown, score: 0.26
Samd15Unknown, score: 0.14
Gpr68Unknown, score: 0.17
Btbd7Unknown, score: 0.05
MtrUnknown, score: 0.23
Zfp72Unknown, score: 0.09
Zfp366Unknown, score: 0.17
GaptUnknown, score: 0.04
Cdc20bUnknown, score: 0.18
Erc2Unknown, score: 0.09
Ccnb1ip1Unknown, score: 0
Zfhx2Unknown, score: 0.3
Setdb2Unknown, score: 0.07
C1qtnf9Unknown, score: 0.01
Gucy1b2Unknown, score: 0.11
Pnma2Unknown, score: 0.06
Gm27179Unknown, score: 0.2
Fam160b2Unknown, score: 0.07
Abcc4Unknown, score: 0.02
Ago2Unknown, score: 0.37
Gpr20Unknown, score: 0.16
Apol8Unknown, score: 0.34
Foxred2Unknown, score: 0.06
Mief1Unknown, score: 0.14
A4galtUnknown, score: 0.32
Pdzrn4Unknown, score: 0.02
Pced1bUnknown, score: 0.18
Ccdc184Unknown, score: 0.07
Zfp641Unknown, score: 0.03
AU021092Unknown, score: 0.18
Mettl22Unknown, score: 0.06
Mkl2Unknown, score: 0.16
LiphUnknown, score: 0.82
OstnUnknown, score: 0.26
Mb21d2Unknown, score: 0.36
LmlnUnknown, score: 0.02
Ccdc14Unknown, score: 0.02
Arid1bUnknown, score: 0.21
Zfp945Unknown, score: 0.19
Mmp25Unknown, score: 0.4
Syngap1Unknown, score: 0.16
Zfp811Unknown, score: 0.14
Fsd1Unknown, score: 0.35
Rasgrp3Unknown, score: 0.04
9430020K01RikUnknown, score: 0.07
Adnp2Unknown, score: 0.37
Zfp407Unknown, score: 0.19
Cdc42bpgUnknown, score: 0.03
Peli3Unknown, score: 0.11
Slc35g1Unknown, score: 0.06
CcnjUnknown, score: 0.04
Slco5a1Unknown, score: 0.4
Pik3c2bUnknown, score: 0.2
Lax1Unknown, score: 0.06
Kcnt2Unknown, score: 0.36
Teddm2Unknown, score: 0.06
Teddm1aUnknown, score: 0.05
Tor1aip2Unknown, score: 0.01
Mettl11bUnknown, score: 0.05
Gpr161Unknown, score: 0.04
Adamts4Unknown, score: 0.12
Vsig8Unknown, score: 0.06
CarfUnknown, score: 0.22
Prkag3Unknown, score: 0.08
Ccdc108Unknown, score: 0.09
Ankmy1Unknown, score: 0.25
Gpr158Unknown, score: 0.02
Pnpla7Unknown, score: 0.04
Ppp1r26Unknown, score: 0.06
Lrrc8aUnknown, score: 0.2
Fam78aUnknown, score: 0.16
Ralgps1Unknown, score: 0.04
Zbtb6Unknown, score: 0.12
Dhrs9Unknown, score: 0.46
Pde11aUnknown, score: 0.23
Zfp385bUnknown, score: 0.01
Fam171bUnknown, score: 0.06
D430041D05RikUnknown, score: 0.18
Slc5a12Unknown, score: 0.1
Atp8b4Unknown, score: 0.03
Pak7Unknown, score: 0.09
Ralgapa2Unknown, score: 0.28
Rims4Unknown, score: 0.01
Kcng1Unknown, score: 0.39
Lsm14bUnknown, score: 0.17
Phc3Unknown, score: 0.28
Pabpc4lUnknown, score: 0.43
Ppm1lUnknown, score: 0.14
Pde5aUnknown, score: 0.13
Impad1Unknown, score: 0
ManeaUnknown, score: 0.37
Fam221bUnknown, score: 0.2
Dcaf10Unknown, score: 0.23
Zfp462Unknown, score: 0.31
D630039A03RikUnknown, score: 0.01
Bnc2Unknown, score: 0.05
Klhl9Unknown, score: 0.08
Atg4cUnknown, score: 0.04
Wdr78Unknown, score: 0.01
1700024P16RikUnknown, score: 0.38
Slc1a7Unknown, score: 0.16
PodnUnknown, score: 0.01
Dmrta2Unknown, score: 0.86
Tctex1d4Unknown, score: 0.14
Rab42Unknown, score: 0.03
Wasf2Unknown, score: 0.25
Gpatch3Unknown, score: 0.28
Ifnlr1Unknown, score: 0.55
Myom3Unknown, score: 0
Klhdc7aUnknown, score: 0.23
Zfp933Unknown, score: 0.06
Ptchd2Unknown, score: 0.81
Klhl21Unknown, score: 0.08
Ttc34Unknown, score: 0.13
Rundc3bUnknown, score: 0.07
Lrrd1Unknown, score: 0.07
Cct8l1Unknown, score: 0.62
CpzUnknown, score: 0.16
Hsd17b13Unknown, score: 0.36
Mfsd7aUnknown, score: 0.05
2900026A02RikUnknown, score: 0.03
Hcar1Unknown, score: 0.06
Nyap1Unknown, score: 0
243302Unknown, score: 0.19
A430033K04RikUnknown, score: 0.04
Elfn1Unknown, score: 0.2
Tmem130Unknown, score: 0.11
Stard13Unknown, score: 0.45
SspoUnknown, score: 0.12
Lrrc61Unknown, score: 0.54
Gimap8Unknown, score: 0.08
Ppm1kUnknown, score: 0.4
Ccdc142Unknown, score: 0.09
Ccdc37Unknown, score: 0.47
Prickle2Unknown, score: 0
Ssu2Unknown, score: 0.22
Clec1aUnknown, score: 0.15
Ppp1r9aUnknown, score: 0.11
Plxna4Unknown, score: 0.01
2010107G12RikUnknown, score: 0.14
Slc13a4Unknown, score: 0.05
Parp12Unknown, score: 0.01
E330009J07RikUnknown, score: 0.16
Leng9Unknown, score: 0.12
Gp6Unknown, score: 0.11
Zfp128Unknown, score: 0.21
Zfp324Unknown, score: 0.23
Gltscr1Unknown, score: 0.11
Ccdc9Unknown, score: 0.04
Mill2Unknown, score: 0.39
Fbxo46Unknown, score: 0.03
Zfp568Unknown, score: 0.3
NfkbidUnknown, score: 0.29
Tshz3Unknown, score: 0.09
Zfp536Unknown, score: 0.25
SiglecgUnknown, score: 0.26
Ntn5Unknown, score: 0.3
Mctp2Unknown, score: 0.16
TskuUnknown, score: 0.03
Olfml1Unknown, score: 0.27
Nlrp10Unknown, score: 0.1
Cyp2r1Unknown, score: 0.57
Zfp771Unknown, score: 0.35
Ctf2Unknown, score: 0.27
Zfp668Unknown, score: 0.03
Cd163l1Unknown, score: 0.09
Myo16Unknown, score: 0
Ppp1r3bUnknown, score: 0.55
D8Ertd82eUnknown, score: 0.05
Podnl1Unknown, score: 0.24
Nanos3Unknown, score: 0.08
Ccdc113Unknown, score: 0.39
Mtss1lUnknown, score: 0.31
SprtnUnknown, score: 0.06
Disc1Unknown, score: 0.11
Cwf19l2Unknown, score: 0.02
Hephl1Unknown, score: 0.2
Dpy19l1Unknown, score: 0.2
AW551984Unknown, score: 0.05
LaynUnknown, score: 0.08
NpatUnknown, score: 0
Tnfaip8l3Unknown, score: 0.1
Slc35g2Unknown, score: 0.01
Zfp937Unknown, score: 0.03
Apol10aUnknown, score: 0.04
Tmem255aUnknown, score: 0.14
Slitrk4Unknown, score: 0.13
HdxUnknown, score: 0.01
Gprasp2Unknown, score: 0.16
Tbc1d8bUnknown, score: 0.33
Frmpd3Unknown, score: 0.08
Iqsec2Unknown, score: 0.01
Klf8Unknown, score: 0.08
Cnksr2Unknown, score: 0.09
Rbbp7Unknown, score: 0.42
Fat2Unknown, score: 0.27
Trappc1Unknown, score: 0.01
Polr2hUnknown, score: 0.33
Ssh3Unknown, score: 0.03
Pcmtd2Unknown, score: 0.15
Map7d1Unknown, score: 0.09
Wasf3Unknown, score: 0.08
Rbm47Unknown, score: 0.73
Slc36a2Unknown, score: 0.11
Onecut3Unknown, score: 0.08
Atxn7Unknown, score: 0.57
Rhbdl3Unknown, score: 0.03
Kcne2Unknown, score: 0.29
VasnUnknown, score: 0.08
FktnUnknown, score: 0.02
OtoaUnknown, score: 0.15
Zfp277Unknown, score: 0.4
Mllt6Unknown, score: 0.74
MpstUnknown, score: 0
Ovca2Unknown, score: 0.54
CsadUnknown, score: 0.04
Cd207Unknown, score: 0.25
Hps5Unknown, score: 0.55
Slc25a28Unknown, score: 0.04
Defb19Unknown, score: 0.2
Apoa1bpUnknown, score: 0.01
Emilin2Unknown, score: 0.09
Rgs13Unknown, score: 0.54
Oas1aUnknown, score: 0.09
Cd300lfUnknown, score: 0.14
AdigUnknown, score: 0
Atpaf2Unknown, score: 0.04
Trpv3Unknown, score: 0.38
Ackr4Unknown, score: 0.33
Ap1s3Unknown, score: 0.12
Grhl2Unknown, score: 0.1
Nod2Unknown, score: 0.27
Acsf3Unknown, score: 0.43
SdslUnknown, score: 0.02
Olfr544Unknown, score: 0.08
Olfr212Unknown, score: 0.02
Olfr1396Unknown, score: 0.03
Olfr1420Unknown, score: 0.14
Olfr877Unknown, score: 0.11
Olfr1388Unknown, score: 0.18
Olfr1393Unknown, score: 0.12
Olfr873Unknown, score: 0.04
Olfr39Unknown, score: 0.14
Olfr457Unknown, score: 0.12
MfrpUnknown, score: 0.17
Prrt1Unknown, score: 0.07
FevUnknown, score: 0.04
OtosUnknown, score: 0.12
Nav3Unknown, score: 0.2
Creg2Unknown, score: 0.21
Pkn3Unknown, score: 0.19
Ttc26Unknown, score: 0.1
Irak4Unknown, score: 0.14
Cpne1Unknown, score: 0.21
LgsnUnknown, score: 0.7
Snx17Unknown, score: 0.21
Rnf217Unknown, score: 0.03
Ahsa2Unknown, score: 0.28
Sh3pxd2bUnknown, score: 0.16
Zkscan17Unknown, score: 0.13
Phf12Unknown, score: 0.36
Rpl23aUnknown, score: 0.12
Ube2zUnknown, score: 0.28
Rapgefl1Unknown, score: 0.36
Lsm12Unknown, score: 0.04
Mgat5bUnknown, score: 0.2
Slc26a11Unknown, score: 0.38
Tmem229bUnknown, score: 0.25
Fam107aUnknown, score: 0.32
Arhgef40Unknown, score: 0.17
Tox4Unknown, score: 0.37
Lrrc16bUnknown, score: 0.34
GuloUnknown, score: 0.08
Mtmr12Unknown, score: 0.34
Klhl38Unknown, score: 0.47
Rbfox1Unknown, score: 0.22
AbatUnknown, score: 0.41
Fbxo45Unknown, score: 0.04
LsampUnknown, score: 0.24
Nrip1Unknown, score: 0.14
Pkmyt1Unknown, score: 0.06
Caskin1Unknown, score: 0.22
Grm4Unknown, score: 0.12
Brpf3Unknown, score: 0.08
Nlrc4Unknown, score: 0.21
Ss18Unknown, score: 0.17
Sap130Unknown, score: 0.66
Zfp608Unknown, score: 0.1
4930503L19RikUnknown, score: 0.03
CtifUnknown, score: 0.18
DaglaUnknown, score: 0.01
OptcUnknown, score: 0.11
Colgalt2Unknown, score: 0.13
Stk36Unknown, score: 0.07
Fam171a1Unknown, score: 0.11
Rpl12Unknown, score: 0.16
Vps39Unknown, score: 0.05
Ell3Unknown, score: 0.24
Slc28a2Unknown, score: 0.07
AhcyUnknown, score: 0.18
Tox2Unknown, score: 0.18
Ss18l1Unknown, score: 0.22
Rtel1Unknown, score: 0.38
Znf512bUnknown, score: 0.16
Jade1Unknown, score: 0.14
Nkain3Unknown, score: 0.4
Fbxl4Unknown, score: 0.24
ClspnUnknown, score: 0.04
Epb4.1Unknown, score: 0.17
Luzp1Unknown, score: 0.43
Chd5Unknown, score: 0.34
Rbm48Unknown, score: 0.36
Wdr86Unknown, score: 0.29
Zfp512Unknown, score: 0.48
Nat8lUnknown, score: 0.36
Rnft2Unknown, score: 0.03
Orai2Unknown, score: 0.2
Cntn4Unknown, score: 0.25
Clec4a1Unknown, score: 0.12
Ssc5dUnknown, score: 0.21
Zfp446Unknown, score: 0.41
Megf8Unknown, score: 0.28
269881Unknown, score: 0.09
Chsy1Unknown, score: 0.67
Idh2Unknown, score: 0.21
Nup98Unknown, score: 0.03
Gsg1lUnknown, score: 0.27
Zfp747Unknown, score: 0.27
Orai3Unknown, score: 0.23
Letm2Unknown, score: 0.61
Slc35e1Unknown, score: 0.69
Lrrc36Unknown, score: 0.25
Vat1lUnknown, score: 0.46
Pcnxl2Unknown, score: 0.04
Fat3Unknown, score: 0.75
Amica1Unknown, score: 0.14
AU019823Unknown, score: 0.05
Myo9aUnknown, score: 0.34
ClpxUnknown, score: 0.22
Ephb1Unknown, score: 0.2
Rab6bUnknown, score: 0.34
Mthfd1lUnknown, score: 0.23
Prr11Unknown, score: 0.75
5031414D18RikUnknown, score: 0.27
Phf21bUnknown, score: 0.2
Zbtb11Unknown, score: 0.31
Tmem169Unknown, score: 0.24
Agbl2Unknown, score: 0.46
Pla2g4fUnknown, score: 0.28
Shc4Unknown, score: 0.45
TbckUnknown, score: 0.07
Lppr1Unknown, score: 0.24
PolnUnknown, score: 0.06
Arntl2Unknown, score: 0.03
Zfp398Unknown, score: 0.41
Irf2bp1Unknown, score: 0.77
Lrrc4bUnknown, score: 0.2
SpibUnknown, score: 0.37
Tarsl2Unknown, score: 0.17
Tmem255bUnknown, score: 0.66
Gins2Unknown, score: 0.1
TbcelUnknown, score: 0.12
Esyt3Unknown, score: 0.3
Smtnl2Unknown, score: 0.27
PigsUnknown, score: 0.07
D11Wsu47eUnknown, score: 0.51
Timd4Unknown, score: 0.27
Armc7Unknown, score: 0.29
277089Unknown, score: 0
Kdm3bUnknown, score: 0.01
Klhl23Unknown, score: 0.04
Trp53i11Unknown, score: 0.06
Gpr107Unknown, score: 0.14
Slc39a12Unknown, score: 0.44
BC049635Unknown, score: 0.2
Slc9a5Unknown, score: 0.47
Spaca5Unknown, score: 0.05
Tmtc2Unknown, score: 0.04
Zfp385cUnknown, score: 0.15
Stkld1Unknown, score: 0.83
279067Unknown, score: 0.04
Nup62clUnknown, score: 0.5
Rhbdd3Unknown, score: 0.16
Emilin3Unknown, score: 0.07
Adam1bUnknown, score: 0.29
Adam1aUnknown, score: 0.1
FlnbUnknown, score: 0.12
Kif19aUnknown, score: 0.03
C1s2Unknown, score: 0.2
Sec22aUnknown, score: 0.06
Hist2h3bUnknown, score: 0.04
Hist1h4cUnknown, score: 0.04
Hist1h4dUnknown, score: 0.04
Hist1h4fUnknown, score: 0.04
Hist1h4iUnknown, score: 0.04
Hist1h4jUnknown, score: 0.04
Hist1h4kUnknown, score: 0.04
Hist1h2abUnknown, score: 0.07
Hist1h2afUnknown, score: 0.2
Hist1h2bcUnknown, score: 0.01
Hist1h2bgUnknown, score: 0.03
Hist1h2bhUnknown, score: 0.01
Hist1h2bjUnknown, score: 0.01
Hist1h2blUnknown, score: 0.01
Hist1h2bnUnknown, score: 0
Hist2h2bbUnknown, score: 0.11
Hist1h2aiUnknown, score: 0.27
Rpl17Unknown, score: 0.25
Pgbd1Unknown, score: 0.06
Trim12cUnknown, score: 0.22
Pcmtd1Unknown, score: 0.35
A230046K03RikUnknown, score: 0.02
Snhg11Unknown, score: 0.14
PianpUnknown, score: 0.57
Adgrl3Unknown, score: 0.52
Hs3st5Unknown, score: 0.42
Fndc3aUnknown, score: 0.45
Ppm1hUnknown, score: 0.27
Itga11Unknown, score: 0.11
9530053A07RikUnknown, score: 0.23
NrcamUnknown, score: 0.33
Syt15Unknown, score: 0.38
Pced1aUnknown, score: 0.01
PdprUnknown, score: 0.44
Idi1Unknown, score: 0.19
Nwd1Unknown, score: 0.22
Celf5Unknown, score: 0.16
Hif1anUnknown, score: 0.07
Fam168aUnknown, score: 0.44
Fsd1lUnknown, score: 0.03
Slc25a40Unknown, score: 0.68
AgmoUnknown, score: 0.13
Fbxo48Unknown, score: 0.19
Cacna2d4Unknown, score: 0.52
Mpzl3Unknown, score: 0.48
Zfp865Unknown, score: 0.14
Rc3h2Unknown, score: 0.18
Bbs9Unknown, score: 0.08
Zcchc7Unknown, score: 0.05
E030030I06RikUnknown, score: 0.14
Apba1Unknown, score: 0.04
Sbf2Unknown, score: 0.06
Tns3Unknown, score: 0.35
Taf2Unknown, score: 0.34
Flad1Unknown, score: 0.02
Ercc6Unknown, score: 0.17
Cc2d1bUnknown, score: 0.29
319974Unknown, score: 0.01
Jph4Unknown, score: 0.08
A630001G21RikUnknown, score: 0.28
Tmem198Unknown, score: 0.3
Fstl4Unknown, score: 0.26
Rnf222Unknown, score: 0.13
Zbtb39Unknown, score: 0.17
Ano4Unknown, score: 0.07
Fbxl13Unknown, score: 0.12
Rps6kc1Unknown, score: 0.35
B430306N03RikUnknown, score: 0.23
Zdhhc17Unknown, score: 0.01
Pik3r5Unknown, score: 0.32
Senp5Unknown, score: 0.14
Catsperg1Unknown, score: 0
Dlec1Unknown, score: 0.1
Rasgef1bUnknown, score: 0.02
Rnf152Unknown, score: 0.04
Hist4h4Unknown, score: 0.04
Lypd6Unknown, score: 0.01
Lrig3Unknown, score: 0
ItpkbUnknown, score: 0.26
Klri2Unknown, score: 0.55
GchfrUnknown, score: 0.18
RinlUnknown, score: 0.05
Heatr5bUnknown, score: 0.07
Tmem215Unknown, score: 0.06
Vps13cUnknown, score: 0.28
Slc35e2Unknown, score: 0.3
Atp8b5Unknown, score: 0.32
SvoplUnknown, score: 0.19
Dopey1Unknown, score: 0.29
OcrlUnknown, score: 0.25
Cyb5r2Unknown, score: 0.28
Cass4Unknown, score: 0.02
Samd12Unknown, score: 0.05
Bend6Unknown, score: 0.03
Atp2b3Unknown, score: 0.11
Mysm1Unknown, score: 0.11
Trappc11Unknown, score: 0.19
Pptc7Unknown, score: 0.24
Slc26a9Unknown, score: 0.21
Ipo8Unknown, score: 0.12
Vstm4Unknown, score: 0.24
Chd7Unknown, score: 0.07
Pkn1Unknown, score: 0.1
Zhx3Unknown, score: 0
Ifitm10Unknown, score: 0.02
Gfm2Unknown, score: 0.17
Dcaf5Unknown, score: 0.04
Negr1Unknown, score: 0.44
Amigo3Unknown, score: 0.17
Spata33Unknown, score: 0.16
Mtap7d3Unknown, score: 0.39
Atp11cUnknown, score: 0.01
PisdUnknown, score: 0.02
Lrrn4Unknown, score: 0.03
Lrif1Unknown, score: 0.29
VprbpUnknown, score: 0.03
6330408A02RikUnknown, score: 0.2
Tpm4Unknown, score: 0.04
Hist1h4aUnknown, score: 0.04
Wisp3Unknown, score: 0.45
Ubtd2Unknown, score: 0.21
Cyb5d1Unknown, score: 0.19
Dnah2Unknown, score: 0.16
ScimpUnknown, score: 0.28
Med13Unknown, score: 0.15
Hsf5Unknown, score: 0.06
Fads6Unknown, score: 0.04
Prpf39Unknown, score: 0.02
Slc39a9Unknown, score: 0.35
Gfod1Unknown, score: 0.16
Mast4Unknown, score: 0.07
Zmiz1Unknown, score: 0.31
Parp4Unknown, score: 0.14
KcnrgUnknown, score: 0.15
Ep300Unknown, score: 0.24
Rab26Unknown, score: 0.01
Hs3st6Unknown, score: 0.27
MslnlUnknown, score: 0.25
Ubash3aUnknown, score: 0.5
Zfp414Unknown, score: 0.04
A530064D06RikUnknown, score: 0.02
Zfp516Unknown, score: 0.18
Pkd2l1Unknown, score: 0.22
Cpa6Unknown, score: 0.07
Abi2Unknown, score: 0.17
Ppp1r12bUnknown, score: 0.1
1700101E01RikUnknown, score: 0.1
NostrinUnknown, score: 0.23
Myo3bUnknown, score: 0.09
Pla2g4eUnknown, score: 0.38
Ctdspl2Unknown, score: 0.32
Nol4lUnknown, score: 0.04
Gm826Unknown, score: 0.05
Fat4Unknown, score: 0.22
E130311K13RikUnknown, score: 0.08
Fnip2Unknown, score: 0.1
Fam102bUnknown, score: 0.17
AI464131Unknown, score: 0.04
Usp24Unknown, score: 0.26
Acot11Unknown, score: 0.5
Col8a2Unknown, score: 0.4
Fam185aUnknown, score: 0.66
Hfm1Unknown, score: 0.09
Ccdc63Unknown, score: 0.35
Tmem120bUnknown, score: 0.07
Vps37bUnknown, score: 0.11
Mblac1Unknown, score: 0.14
D630045J12RikUnknown, score: 0.15
Zfp786Unknown, score: 0.42
Wipf3Unknown, score: 0.26
Fam188bUnknown, score: 0
B4galnt3Unknown, score: 0.06
Cecr2Unknown, score: 0.48
Zfp78Unknown, score: 0.14
Zc3h4Unknown, score: 0.13
Ceacam16Unknown, score: 0.37
Gm5113Unknown, score: 0.45
Fan1Unknown, score: 0.09
Saxo2Unknown, score: 0.08
Trim66Unknown, score: 0.31
Dock1Unknown, score: 0.24
B4galnt4Unknown, score: 0.08
Htra4Unknown, score: 0.3
Hapln4Unknown, score: 0.2
Rnf150Unknown, score: 0.51
Adgrl1Unknown, score: 0.5
4933402J07RikUnknown, score: 0.37
Pate2Unknown, score: 0.19
AI593442Unknown, score: 0.05
Hcn4Unknown, score: 0.11
Slc9a9Unknown, score: 0.6
GmppbUnknown, score: 0.1
UprtUnknown, score: 0.4
Tceal5Unknown, score: 0.36
Bend3Unknown, score: 0.1
Krt78Unknown, score: 0.24
Zdhhc23Unknown, score: 0.04
Nanos1Unknown, score: 0.12
Card9Unknown, score: 0.17
Zmynd12Unknown, score: 0.03
KcpUnknown, score: 0.55
Cox6b2Unknown, score: 0.26
Proser3Unknown, score: 0.15
Gpd1lUnknown, score: 0.1
Mamld1Unknown, score: 0.15
Mia2Unknown, score: 0.03
Cog3Unknown, score: 0.26
CntlnUnknown, score: 0.37
Fkbp15Unknown, score: 0.38
UstUnknown, score: 0.01
NalcnUnknown, score: 0.25
EndovUnknown, score: 0
Map3k9Unknown, score: 0.4
Caps2Unknown, score: 0.04
Plekhm1Unknown, score: 0.14
Gjd3Unknown, score: 0.43
TxlngUnknown, score: 0.08
GarsUnknown, score: 0.05
Adam32Unknown, score: 0.28
Pcdhac2Unknown, score: 0.06
Tmc4Unknown, score: 0.05
Hcfc1r1Unknown, score: 0.39
Col27a1Unknown, score: 0.13
Nlrp12Unknown, score: 0.13
TxlnbUnknown, score: 0.31
MafaUnknown, score: 0.06
Morn2Unknown, score: 0.02
Bpifb3Unknown, score: 0.56
Serf2Unknown, score: 0.53
Fastkd5Unknown, score: 0.29
Tagap1Unknown, score: 0.27
HecaUnknown, score: 0.2
Lemd3Unknown, score: 0.02
Cnrip1Unknown, score: 0.12
CcnjlUnknown, score: 0.35
Shisa6Unknown, score: 0.37
Spata22Unknown, score: 0.05
Rap1gap2Unknown, score: 0.11
Tlcd2Unknown, score: 0.2
Scarf1Unknown, score: 0.18
Rph3alUnknown, score: 0.13
Milr1Unknown, score: 0.44
Atxn7l1Unknown, score: 0.52
Lsmem1Unknown, score: 0.27
BegainUnknown, score: 0
Rsl1Unknown, score: 0.29
Tmem171Unknown, score: 0.24
Olfm4Unknown, score: 0.08
Kmt2dUnknown, score: 0.03
Ccdc58Unknown, score: 0.01
Zfp229Unknown, score: 0.01
NpwUnknown, score: 0.22
Ccdc78Unknown, score: 0.16
Tbc1d22bUnknown, score: 0.27
H2-Eb2Unknown, score: 0.28
Rmdn2Unknown, score: 0
Cdkl4Unknown, score: 0.08
Arl14eplUnknown, score: 0.13
Ccdc68Unknown, score: 0.38
Tmem151aUnknown, score: 0.25
Ap5b1Unknown, score: 0.23
4430402I18RikUnknown, score: 0.04
Tmem237Unknown, score: 0.08
Gm973Unknown, score: 0.04
HjurpUnknown, score: 0.41
MndaUnknown, score: 0.13
6330403A02RikUnknown, score: 0.04
Nsl1Unknown, score: 0.02
Fam178bUnknown, score: 0.1
Lonrf2Unknown, score: 0.16
Tmem182Unknown, score: 0.17
Spag6lUnknown, score: 0.43
Mamdc4Unknown, score: 0.17
Gm996Unknown, score: 0.68
Bpifb4Unknown, score: 0.1
Pabpc1lUnknown, score: 0.34
Cdh26Unknown, score: 0.02
Nr1h5Unknown, score: 0.23
Gm5150Unknown, score: 0.11
Rxfp1Unknown, score: 0.11
Ube2uUnknown, score: 0.24
Mroh7Unknown, score: 0.03
Zfp69Unknown, score: 0.03
Xkr8Unknown, score: 0.16
Tmem240Unknown, score: 0.26
5031410I06RikUnknown, score: 0
Adgrf3Unknown, score: 0.08
AtraidUnknown, score: 0.62
Cep135Unknown, score: 0.14
Fbrsl1Unknown, score: 0.01
Nxpe5Unknown, score: 0
Tsen2Unknown, score: 0.04
Clec4b2Unknown, score: 0
Lpar5Unknown, score: 0.06
Apold1Unknown, score: 0.58
Sbk3Unknown, score: 0.69
Sbk2Unknown, score: 0.11
2310014L17RikUnknown, score: 0.64
Alg8Unknown, score: 0.16
Taok2Unknown, score: 0.29
Zbtb2Unknown, score: 0.24
Cep44Unknown, score: 0.5
Ano8Unknown, score: 0.49
Cnep1r1Unknown, score: 0.43
Gse1Unknown, score: 0.02
Ces1bUnknown, score: 0.07
Adgrg5Unknown, score: 0.4
Ces3aUnknown, score: 0.66
Crtc1Unknown, score: 0.09
Prdm10Unknown, score: 0.19
Ccdc84Unknown, score: 0.03
Ccdc33Unknown, score: 0.02
Gm1123Unknown, score: 0.23
382099Unknown, score: 0.18
Poc1bUnknown, score: 0.05
Best3Unknown, score: 0.12
Cd300lhUnknown, score: 0.14
Zbtb42Unknown, score: 0.06
Mtx3Unknown, score: 0.58
Zfp488Unknown, score: 0.01
Ypel5Unknown, score: 0.06
Aim2Unknown, score: 0.27
Glipr2Unknown, score: 0.11
Fndc5Unknown, score: 0.3
Ephx4Unknown, score: 0.09
Nova2Unknown, score: 0.12
Zfp667Unknown, score: 0.16
Kng2Unknown, score: 0.43
Lca5lUnknown, score: 0.06
Zfp174Unknown, score: 0.05
CdsnUnknown, score: 0.2
Thoc6Unknown, score: 0.09
Nsfl1cUnknown, score: 0.32
Tmtc1Unknown, score: 0.07
Znrf2Unknown, score: 0.36
Zhx2Unknown, score: 0.01
Ugt1a7cUnknown, score: 0.45
Ugt1a1Unknown, score: 0.49
Flrt2Unknown, score: 0.18
BC052040Unknown, score: 0.11
Ccdc87Unknown, score: 0.1
Fam84bUnknown, score: 0.16
Tcaf3Unknown, score: 0.35
Msantd1Unknown, score: 0.09
Cyp2c50Unknown, score: 0.3
Olfr1383Unknown, score: 0.04
Iqgap3Unknown, score: 0.03
Tmem189Unknown, score: 0.18
Taf9bUnknown, score: 0.17
BC051142Unknown, score: 0.19
Zfp941Unknown, score: 0.07
BC031181Unknown, score: 0.16
Baz2bUnknown, score: 0.34
Zfp873Unknown, score: 0.04
Zfp738Unknown, score: 0.45
BC024978Unknown, score: 0.12
414077Unknown, score: 0.25
Gja6Unknown, score: 0.26
Zyg11bUnknown, score: 0.65
Akap7Unknown, score: 0.14
Hnrnph3Unknown, score: 0.13
Cpsf6Unknown, score: 0.06
Myo1aUnknown, score: 0.47
Specc1Unknown, score: 0.24
Gm11541Unknown, score: 0.12
Dnaic2Unknown, score: 0.14
Trim80Unknown, score: 0.33
Mfsd2bUnknown, score: 0.12
Akr1c19Unknown, score: 0.14
Zscan26Unknown, score: 0.23
Prr7Unknown, score: 0.28
Rslcan18Unknown, score: 0.12
Lrrc14bUnknown, score: 0.1
432800Unknown, score: 0.04
Gprin2Unknown, score: 0.11
OtulinUnknown, score: 0.19
432950Unknown, score: 0.3
433064Unknown, score: 0.07
Acsl5Unknown, score: 0.37
Rbm10Unknown, score: 0.15
Mettl21cUnknown, score: 0.14
433319Unknown, score: 0.2
Teddm1bUnknown, score: 0.22
Syndig1Unknown, score: 0.58
Akirin2Unknown, score: 0.06
Gm12429Unknown, score: 0.12
Ncbp1Unknown, score: 0.37
433748Unknown, score: 0.13
Hdac1Unknown, score: 0.24
Gm13154Unknown, score: 0.78
Chchd2Unknown, score: 0.65
Rnf207Unknown, score: 0.52
Nom1Unknown, score: 0.28
Mn1Unknown, score: 0.16
Fam222aUnknown, score: 0.41
Dnaaf5Unknown, score: 0.01
Tmem178bUnknown, score: 0.01
434050Unknown, score: 0.05
Ccdc8Unknown, score: 0.31
Slc28a1Unknown, score: 0.06
Lrrc32Unknown, score: 0.11
2610020H08RikUnknown, score: 0.52
Zfp560Unknown, score: 0.67
Gm5617Unknown, score: 0.3
434428Unknown, score: 0.37
Rpl10Unknown, score: 0.23
Sp140Unknown, score: 0.03
Gm10220Unknown, score: 0.07
434693Unknown, score: 0.11
Ldoc1Unknown, score: 0.1
Gm5640Unknown, score: 0.77
434843Unknown, score: 0.22
Dupd1Unknown, score: 0.16
FcrlbUnknown, score: 0.28
Tnni3kUnknown, score: 0.8
Lrp3Unknown, score: 0.29
Dnaaf3Unknown, score: 0.16
Fam92bUnknown, score: 0.35
Gpr62Unknown, score: 0.16
Rab44Unknown, score: 0.46
Xrra1Unknown, score: 0.15
Fbxl7Unknown, score: 0.53
Zfp213Unknown, score: 0.37
Xkr4Unknown, score: 0.22
AcdUnknown, score: 0.07
Ear2Unknown, score: 0.34
NpcdUnknown, score: 0.12
Trcg1Unknown, score: 0.12
Tbc1d32Unknown, score: 0.38
Myh13Unknown, score: 0.21
Arhgap27Unknown, score: 0.06
Bdp1Unknown, score: 0.43
H3f3aUnknown, score: 0.06
Cma2Unknown, score: 0.23
Wdr70Unknown, score: 0.18
TdgUnknown, score: 0.15
KalrnUnknown, score: 0.45
Baiap3Unknown, score: 0.06
ArsiUnknown, score: 0.67
Cfhr2Unknown, score: 0.13
Cep170Unknown, score: 0.14
545423Unknown, score: 0.28
Ccdc141Unknown, score: 0.39
545459Unknown, score: 0.07
Zfp345Unknown, score: 0.21
Arhgap40Unknown, score: 0.13
Tubb1Unknown, score: 0.1
Erich6Unknown, score: 0.51
545531Unknown, score: 0.11
545600Unknown, score: 0.24
Fam159aUnknown, score: 0.02
Pilrb2Unknown, score: 0.18
Zfp607Unknown, score: 0.01
Cers3Unknown, score: 0.03
CrxosUnknown, score: 0.13
C330021F23RikUnknown, score: 0.1
546058Unknown, score: 0.28
Prrg1Unknown, score: 0.21
Klhl33Unknown, score: 0.59
Cyp26c1Unknown, score: 0.1
Gm13178Unknown, score: 0.16
Parp14Unknown, score: 0.52
Gm6034Unknown, score: 0.29
Btnl2Unknown, score: 0.6
Gpr17Unknown, score: 0.12
Zmynd15Unknown, score: 0.16
Tceal3Unknown, score: 0.47
Gsk3aUnknown, score: 0.01
Tmem253Unknown, score: 0.08
Tnfsfm13Unknown, score: 0.09
Defb42Unknown, score: 0.22
Zcchc17Unknown, score: 0.03
Klf14Unknown, score: 0.3
619937Unknown, score: 0.12
619941Unknown, score: 0.2
C130026I21RikUnknown, score: 0.52
620248Unknown, score: 0.09
Tmem28Unknown, score: 0.1
Ttc30a2Unknown, score: 0.36
AI429214Unknown, score: 0.4
621542Unknown, score: 0.36
AC087559.2Unknown, score: 0
Aldh3b2Unknown, score: 0.13
Cldn20Unknown, score: 0.39
Rpl32Unknown, score: 0.12
621705Unknown, score: 0.13
Psme2Unknown, score: 0.07
Tmem170bUnknown, score: 0
622335Unknown, score: 0.28
Arhgef26Unknown, score: 0.29
1700123I01RikUnknown, score: 0.2
Ccdc17Unknown, score: 0.18
Pydc4Unknown, score: 0.09
623174Unknown, score: 0.3
Tmem200bUnknown, score: 0.18
Rad54bUnknown, score: 0.08
Lipt1Unknown, score: 0.14
Gm14137Unknown, score: 0.16
Speer4eUnknown, score: 0.05
Gpx4Unknown, score: 0.3
Tmem236Unknown, score: 0.43
H3f3aUnknown, score: 0.04
Gm6583Unknown, score: 0.02
Fam43bUnknown, score: 0.77
626048Unknown, score: 0.22
Wdr93Unknown, score: 0.01
Gbp10Unknown, score: 0.15
Gm6710Unknown, score: 0.16
Zfp800Unknown, score: 0.14
Morf4l1Unknown, score: 0.43
627371Unknown, score: 0.54
627375Unknown, score: 0.04
Gm6792Unknown, score: 0.26
627901Unknown, score: 0.07
Gm4631Unknown, score: 0.43
628161Unknown, score: 0.16
628648Unknown, score: 0.09
628794Unknown, score: 0.2
Fam124aUnknown, score: 0.07
Mroh8Unknown, score: 0
Lin52Unknown, score: 0.11
Zfp808Unknown, score: 0
630855Unknown, score: 0.25
631033Unknown, score: 0.18
631040Unknown, score: 0.29
Fer1l6Unknown, score: 0.19
632329Unknown, score: 0.09
Vmn2r18Unknown, score: 0.26
Rbm46Unknown, score: 0.06
633406Unknown, score: 0.11
633417Unknown, score: 0.48
Gm21967Unknown, score: 0.14
Susd1Unknown, score: 0.32
Usp51Unknown, score: 0.1
Zfp964Unknown, score: 0.57
Nlrp1bUnknown, score: 0.4
637553Unknown, score: 0.18
637733Unknown, score: 0.15
637796Unknown, score: 0.51
638399Unknown, score: 0.09
639541Unknown, score: 0.52
639606Unknown, score: 0.03
639905Unknown, score: 0.09
639931Unknown, score: 0.06
640549Unknown, score: 0.41
640972Unknown, score: 0.01
Nrbf2Unknown, score: 0.54
Tomm40lUnknown, score: 0.45
Tmem243Unknown, score: 0.14
Alkbh1Unknown, score: 0.28
654450Unknown, score: 0.32
Defb26Unknown, score: 0.07
Defb25Unknown, score: 0.05
Tctn1Unknown, score: 0.15
Sdr39u1Unknown, score: 0.27
Angptl7Unknown, score: 0.17
Gcnt7Unknown, score: 0.03
Ankrd37Unknown, score: 0.07
Nova1Unknown, score: 0.71
Tmem238Unknown, score: 0.3
Gm14393Unknown, score: 0.76
Col6a5Unknown, score: 0.04
Srp54bUnknown, score: 0.16
Clec2lUnknown, score: 0.4
Gm14326Unknown, score: 0.19
Hist1h2aoUnknown, score: 0.77
665463Unknown, score: 0.12
665503Unknown, score: 0.77
665522Unknown, score: 0.51
Mthfd2lUnknown, score: 0.77
665579Unknown, score: 0.77
Bod1lUnknown, score: 0.33
Vps13bUnknown, score: 0.12
Tomm5Unknown, score: 0.36
Atg4aUnknown, score: 0.36
Gm13139Unknown, score: 0.36
666548Unknown, score: 0.23
Rpl29Unknown, score: 0.1
666648Unknown, score: 0.01
Samd1Unknown, score: 0.03
Trim43cUnknown, score: 0.36
Trim43bUnknown, score: 0.36
Pnp2Unknown, score: 0.58
9930111J21Rik1Unknown, score: 0.43
H3f3aUnknown, score: 0.14
667384Unknown, score: 0.31
667414Unknown, score: 0.07
Zfp600Unknown, score: 0.31
667803Unknown, score: 0.25
Trim5Unknown, score: 0.52
667846Unknown, score: 0.52
Gm8898Unknown, score: 0.16
Gm14305Unknown, score: 0.07
Gm14434Unknown, score: 0.16
668114Unknown, score: 0.42
Ccdc85cUnknown, score: 0.04
ZxdbUnknown, score: 0.04
Pex10Unknown, score: 0.02
Gm13288Unknown, score: 0.12
Efr3bUnknown, score: 0.12
Dgat2l6Unknown, score: 0.29
Kif26aUnknown, score: 0.07
668455Unknown, score: 0.11
668548Unknown, score: 0.18
2410002F23RikUnknown, score: 0.07
668830Unknown, score: 0.23
668894Unknown, score: 0.07
668936Unknown, score: 0.62
Myh7bUnknown, score: 0.15
670211Unknown, score: 0.77
670832Unknown, score: 0.54
671242Unknown, score: 0.24
Parp10Unknown, score: 0.77
Zfp605Unknown, score: 0.32
676710Unknown, score: 0.06
677113Unknown, score: 0.1
Cyp4f37Unknown, score: 0.03
Gm9733Unknown, score: 0.08
Sap25Unknown, score: 0.04
TomtUnknown, score: 0.06
Pydc3Unknown, score: 0.19
Wfdc17Unknown, score: 0.33
Mfap1bUnknown, score: 0.11
Dnajc3Unknown, score: 0.14
Fam129cUnknown, score: 0.61
Rsph3bUnknown, score: 0.24
Fam174bUnknown, score: 0.22
Gm14548Unknown, score: 0.02
Gm1979Unknown, score: 0.77
100038969Unknown, score: 0.21
Gm10471Unknown, score: 0.27
Ccl19Unknown, score: 0.07
Mup14Unknown, score: 0.26
Gm14295Unknown, score: 0.26
Ccdc152Unknown, score: 0.1
Tmem254cUnknown, score: 0.1
100039478Unknown, score: 0.04
100039484Unknown, score: 0.04
100039532Unknown, score: 0.07
100039674Unknown, score: 0.28
MthfslUnknown, score: 0.53
100039731Unknown, score: 0.35
Tgtp2Unknown, score: 0.33
100039826Unknown, score: 0.02
100040018Unknown, score: 0.12
100040260Unknown, score: 0.09
100040298Unknown, score: 0.46
Dynlt1cUnknown, score: 0.06
Gm15319Unknown, score: 0.03
FancfUnknown, score: 0.11
100040682Unknown, score: 0.04
Gm10408Unknown, score: 0.27
Gm3194Unknown, score: 0.15
100041194Unknown, score: 0.04
Hist1h4mUnknown, score: 0.13
Ndufb4Unknown, score: 0.05
Gm13247Unknown, score: 0.36
ErmardUnknown, score: 0.36
AC087559.2Unknown, score: 0.03
Gm13157Unknown, score: 0.01
Gm10094Unknown, score: 0.23
GapdhUnknown, score: 0.62
Gm3646Unknown, score: 0.31
100042069Unknown, score: 0.01
100042074Unknown, score: 0.01
100042100Unknown, score: 0.26
Gm3696Unknown, score: 0.53
100042235Unknown, score: 0.3
Gsta1Unknown, score: 0.29
Gm10639Unknown, score: 0.21
Ube2l3Unknown, score: 0.28
Nhsl2Unknown, score: 0.13
Ndufb4Unknown, score: 0.21
Gm13305Unknown, score: 0.25
100042625Unknown, score: 0.02
100042773Unknown, score: 0.09
Prdm11Unknown, score: 0.07
Gm4070Unknown, score: 0.27
100043002Unknown, score: 0.25
100043027Unknown, score: 0
Rex2Unknown, score: 0.13
100043059Unknown, score: 0.08
Gm11710Unknown, score: 0.08
Gm11711Unknown, score: 0.13
9130023H24RikUnknown, score: 0.14
100043257Unknown, score: 0.06
TigitUnknown, score: 0.1
100043324Unknown, score: 0.14
Rpl21Unknown, score: 0.07
Gm14305Unknown, score: 0.1
Gm4477Unknown, score: 0.4
SrcapUnknown, score: 0.34
100043670Unknown, score: 0.51
Rbx1Unknown, score: 0.43
100043695Unknown, score: 0.4
Josd1Unknown, score: 0.34
100043805Unknown, score: 0.27
100043872Unknown, score: 0.09
100043882Unknown, score: 0.07
100044322Unknown, score: 0.16
100044374Unknown, score: 0.3
100044391Unknown, score: 0.1
100044398Unknown, score: 0.56
Tgfbr3lUnknown, score: 0.07
Rpl23Unknown, score: 0.22
100044742Unknown, score: 0.12
100045326Unknown, score: 0.26
100045367Unknown, score: 0.78
100045688Unknown, score: 0.08
100045924Unknown, score: 0.23
100045968Unknown, score: 0.34
100046034Unknown, score: 0.27
100046048Unknown, score: 0.66
100046119Unknown, score: 0.22
100046151Unknown, score: 0.38
100046223Unknown, score: 0.18
100046289Unknown, score: 0.35
100046628Unknown, score: 0.03
100046650Unknown, score: 0.42
100046684Unknown, score: 0.11
100046899Unknown, score: 0.32
100047252Unknown, score: 0.21
100047429Unknown, score: 0.09
100047577Unknown, score: 0.12
100048268Unknown, score: 0.4
100048447Unknown, score: 0.52
100048483Unknown, score: 0.33
100048499Unknown, score: 0.02
100048557Unknown, score: 0.3
100048613Unknown, score: 0.22
Dnah7cUnknown, score: 0.42
100137011Unknown, score: 0.18
Gm14306Unknown, score: 0.25
Ttll2Unknown, score: 0.47
AK010878Unknown, score: 0.07
Dynlt1aUnknown, score: 0.26
100502680Unknown, score: 0.58
Kifc1Unknown, score: 0.11
100502777Unknown, score: 0.51
Rpl37Unknown, score: 0.1
Epg5Unknown, score: 0.04
Gm684Unknown, score: 0.41
Armcx4Unknown, score: 0.15
100503055Unknown, score: 0.53
Klhl3Unknown, score: 0.23
Btbd8Unknown, score: 0.32
100503217Unknown, score: 0
Gm14440Unknown, score: 0.03
Zfp534Unknown, score: 0.32
Hbb-bsUnknown, score: 0.29
DosUnknown, score: 0.07
Rpl5Unknown, score: 0.11
Dnajc19Unknown, score: 0.54
100503799Unknown, score: 0.1
Gm5901Unknown, score: 0.29
Gm8923Unknown, score: 0.08
100504089Unknown, score: 0.1
Prss51Unknown, score: 0.1
100504173Unknown, score: 0.14
Ifi203Unknown, score: 0.32
Prr22Unknown, score: 0.34
Gm3194Unknown, score: 0.27
100504500Unknown, score: 0.07
3425401B19RikUnknown, score: 0.2
Cisd3Unknown, score: 0.15
Mettl21bUnknown, score: 0.14
Atg14Unknown, score: 0.26
100504821Unknown, score: 0.32
100504863Unknown, score: 0.39
100504872Unknown, score: 0.34
100504912Unknown, score: 0.22
100504922Unknown, score: 0.19
100504934Unknown, score: 0.17
100504959Unknown, score: 0.47
100504983Unknown, score: 0.32
100504988Unknown, score: 0.24
100505015Unknown, score: 0.57
100505031Unknown, score: 0.45
100505237Unknown, score: 0.14
100505283Unknown, score: 0.1
Gm11127Unknown, score: 0.5
Gm20604Unknown, score: 0.03
Mup18Unknown, score: 0.34
100861947Unknown, score: 0.21
Tmed2Unknown, score: 0.23
100862206Unknown, score: 0.77
100862223Unknown, score: 0.36
RP23-56M18.6Unknown, score: 0.26
100862401Unknown, score: 0.24
100862433Unknown, score: 0.5
100862437Unknown, score: 0.03
Ftl1Unknown, score: 0.07
Rpl23Unknown, score: 0.31
Polr2kUnknown, score: 0.11
Gm13154Unknown, score: 0.23
100862515Unknown, score: 0.62
100862563Unknown, score: 0.12
100862586Unknown, score: 0.23
100862595Unknown, score: 0.29
101055633Unknown, score: 0.08
101055647Unknown, score: 0.1
101055652Unknown, score: 0.16
Lipo2Unknown, score: 0.1
101055716Unknown, score: 0.05
101055738Unknown, score: 0.05
101055761Unknown, score: 0.48
101055764Unknown, score: 0.11
101055802Unknown, score: 0.13
101055828Unknown, score: 0.19
101055829Unknown, score: 0.5
101055854Unknown, score: 0.09
101055925Unknown, score: 0.16
101055956Unknown, score: 0.13
101055995Unknown, score: 0.37
101055997Unknown, score: 0.4
101056010Unknown, score: 0.09
101056016Unknown, score: 0.19
101056094Unknown, score: 0.08
101056131Unknown, score: 0.27
101056140Unknown, score: 0.2
101056341Unknown, score: 0.22
101056352Unknown, score: 0.06
101056362Unknown, score: 0.06
101056365Unknown, score: 0.3
101056370Unknown, score: 0.1
101056381Unknown, score: 0.03
101056392Unknown, score: 0.15
101056496Unknown, score: 0.19
101056500Unknown, score: 0.11
101056514Unknown, score: 0.02
101056542Unknown, score: 0.44
101056544Unknown, score: 0.17
101056559Unknown, score: 0.23
101056574Unknown, score: 0.05
101056596Unknown, score: 0.1
101056614Unknown, score: 0.06
101056649Unknown, score: 0.24
101056658Unknown, score: 0.43
101056659Unknown, score: 0.06
101056688Unknown, score: 0.87
101056691Unknown, score: 0.32

## Help | Hide | Top Help | Show | Top Conditions

### HELP

Conditions in the module, given in the same order as on the expression
plot above. Red color means over-expression, green under-expression in
the given condition.

The barplot below shows the condition (sample) scores. A separate bar
is shown for each sample, its height is the corresponding score of the
sample in the module. The red and green numbers on the bars are the
sample scores expressed in percents, i.e. 100% is 1.0.

The red and green lines show the module thresholds, samples above
the red line and below the green line are included in the module.

The different experiments that were part of the study, are separated
by dashed vertical lines.

— Click on the *Help* button again to close this help window.

| Id |
| --- |
| BALB\_cJ-ISO\_84 |
| DBA\_2J-ISO\_8 |
| BALB\_cJ-ISO\_83 |
| BALB\_cByJ-CTR\_144 |
| CBA\_J-ISO\_133 |
| DBA\_2J-ISO\_7 |
| BALB\_cJ-ATE\_82 |
| CBA\_J-ISO\_128 |
| CBA\_J-ISO\_129 |
| BALB\_cJ-CTR\_85 |
| BALB\_cJ-ATE\_86 |
| SM\_J-ISO\_45 |
| SM\_J-ISO\_46 |
| CBA\_J-CTR\_130 |
| SM\_J-ISO\_44 |
| DBA\_2J-CTR\_9 |
| C3H\_HeJ-ISO\_62 |
| SM\_J-ATE\_42 |
| I\_LnJ-ISO\_155 |
| BALB\_cJ-ISO\_81 |
| DBA\_2J-CTR\_1 |
| C3H\_HeJ-ISO\_69 |
| I\_LnJ-ISO\_149 |
| I\_LnJ-ISO\_152 |
| DBA\_2J-ISO\_4 |
| C3H\_HeJ-ISO\_68 |
| CBA\_J-CTR\_125 |
| BALB\_cByJ-ISO\_156 |
| CBA\_J-ATE\_127 |
| CBA\_J-ATE\_132 |
| BALB\_cByJ-ISO\_159 |
| C57BL\_6J-ATE\_95 |
| BALB\_cByJ-ISO\_145 |
| C58\_J-ISO\_37 |
| CBA\_J-CTR\_126 |
| C57BL\_6J-ATE\_106 |
| CBA\_J-ATE\_131 |
| SM\_J-CTR\_41 |
| C57BLKS\_J-ISO\_115 |
| SM\_J-ATE\_43 |
| NOD\_ShiLtJ-ISO\_25 |
| C58\_J-ISO\_33 |
| DBA\_2J-CTR\_2 |
| I\_LnJ-ATE\_154 |
| DBA\_2J-ATE\_5 |
| SM\_J-CTR\_40 |
| C58\_J-ISO\_31 |
| SM\_J-ATE\_49 |
| I\_LnJ-ATE\_148 |
| SWR\_J-ISO\_142 |
| NOD\_ShiLtJ-ISO\_24 |
| I\_LnJ-ATE\_146 |
| DBA\_2J-ATE\_6 |
| I\_LnJ-CTR\_153 |
| FVB\_NJ-ISO\_58 |
| C57BL\_6J-ISO\_50 |
| SWR\_J-ISO\_141 |
| A\_J-ISO\_124 |
| C57BLKS\_J-ISO\_114 |
| FVB\_NJ-ISO\_54 |
| SM\_J-CTR\_48 |
| BALB\_cByJ-CTR\_143 |
| NOD\_ShiLtJ-ISO\_26 |
| I\_LnJ-CTR\_147 |
| LP\_J-ISO\_103 |
| C57BLKS\_J-CTR\_110 |
| C3H\_HeJ-CTR\_63 |
| A\_J-ISO\_123 |
| PL\_J-ISO\_77 |
| C57BL\_6J-ISO\_59 |
| BALB\_cJ-CTR\_79 |
| SJL\_J-ISO\_12 |
| FVB\_NJ-ISO\_55 |
| C57BLKS\_J-CTR\_111 |
| I\_LnJ-CTR\_150 |
| A\_J-ISO\_118 |
| LP\_J-ISO\_99 |
| C58\_J-CTR\_34 |
| NZB\_BLNJ-ISO\_91 |
| SJL\_J-ISO\_13 |
| SWR\_J-ISO\_136 |
| NZB\_BLNJ-ISO\_96 |
| PL\_J-ISO\_78 |
| C57BL\_6J-ISO\_47 |
| BALB\_cJ-ATE\_80 |
| PL\_J-ISO\_73 |
| C3H\_HeJ-CTR\_64 |
| C57BLKS\_J-ATE\_113 |
| NZB\_BLNJ-ISO\_90 |
| LP\_J-ISO\_102 |
| C3H\_HeJ-ATE\_67 |
| SJL\_J-ISO\_14 |
| BALB\_cByJ-ATE\_157 |
| C3H\_HeJ-ATE\_66 |
| C57BLKS\_J-ATE\_108 |
| C3H\_HeJ-CTR\_65 |
| DBA\_2J-ATE\_3 |
| C3H\_HeJ-ATE\_61 |
| C58\_J-CTR\_32 |
| LP\_J-CTR\_100 |
| C57BLKS\_J-ATE\_112 |
| LP\_J-ATE\_101 |
| BALB\_cByJ-ATE\_151 |
| LP\_J-CTR\_104 |
| C58\_J-ATE\_36 |
| BALB\_cByJ-CTR\_158 |
| NZB\_BLNJ-CTR\_87 |
| C57BLKS\_J-CTR\_107 |
| LP\_J-CTR\_97 |
| A\_J-CTR\_121 |
| NZB\_BLNJ-ATE\_88 |
| PL\_J-CTR\_70 |
| C57BL\_6J-CTR\_39 |
| BALB\_cByJ-ATE\_160 |
| FVB\_NJ-CTR\_51 |
| FVB\_NJ-ATE\_53 |
| C58\_J-CTR\_28 |
| FVB\_NJ-CTR\_52 |
| C57BL\_6J-CTR\_38 |
| C57BL\_6J-CTR\_35 |
| LP\_J-ATE\_98 |
| NZB\_BLNJ-CTR\_93 |
| A\_J-CTR\_119 |
| NOD\_ShiLtJ-CTR\_20 |
| SWR\_J-ATE\_139 |
| LP\_J-ATE\_105 |
| C57BL\_6J-ATE\_109 |
| NZB\_BLNJ-CTR\_92 |
| FVB\_NJ-CTR\_56 |
| A\_J-ATE\_116 |
| A\_J-ATE\_122 |
| A\_J-CTR\_120 |
| SJL\_J-ATE\_11 |
| A\_J-ATE\_117 |
| SJL\_J-CTR\_10 |
| C58\_J-ATE\_29 |
| PL\_J-CTR\_72 |
| C58\_J-ATE\_30 |
| SWR\_J-CTR\_134 |
| FVB\_NJ-ATE\_57 |
| PL\_J-ATE\_75 |
| SJL\_J-CTR\_16 |
| PL\_J-CTR\_74 |
| NOD\_ShiLtJ-ATE\_22 |
| SWR\_J-CTR\_137 |
| SJL\_J-ATE\_17 |
| PL\_J-ATE\_76 |
| NOD\_ShiLtJ-CTR\_21 |
| NZB\_BLNJ-ATE\_89 |
| NZB\_BLNJ-ATE\_94 |
| NOD\_ShiLtJ-CTR\_19 |
| FVB\_NJ-ATE\_60 |
| SWR\_J-ATE\_140 |
| SJL\_J-CTR\_15 |
| PL\_J-ATE\_71 |
| NOD\_ShiLtJ-ATE\_27 |
| SWR\_J-ATE\_135 |
| NOD\_ShiLtJ-ATE\_23 |
| SWR\_J-CTR\_138 |
| SJL\_J-ATE\_18 |

© 2015 Computational Biology Group, Department of Medical Genetics,
University of Lausanne, Switzerland
