# Supplementary material for: RNAseq analysis of heart tissue from mice treated with atenolol and isoproterenol reveals a reciprocal transcriptional response
Source: BMC Genomics. 2016 Sep 7;17(1):717. doi: 10.1186/s12864-016-3059-6 (PMC5015234; doi:10.1186/s12864-016-3059-6)
Supplement: Additional file 4: — Supplementary data. Compressed HTML files of 98 expression modules annotated for genes, strains and GO or KEGG terms (see Additional file 3 for navigation details). (GZ 11006 kb) [file 12864_2016_3059_MOESM4_ESM.gz › modules.html/module-96.html]

Module #96, TG: 0, TC: 0, 7946 probes, 7946 Entrez genes, 160 conditions

# Previous module | Next module Module #96, TG: 0, TC: 0, 7946 probes, 7946 Entrez genes, 160 conditions

- Module tree/table

- Expression data

- The BP GO tree
- The CC GO tree
- The MF GO tree

- GO BP enrichment
- GO CC enrichment
- GO MF enrichment
- KEGG enrichment
- miRNA enrichment

- Genes
- Conditions

## Help | Hide | Top Help | Show | Top Expression data

### HELP

The image plot shows the color-coded level of gene expression, for the
genes and conditions in a given transcription module. The genes are on
the horizontal, the conditions on the vertical axis.

The genes are ordered according to their ISA gene scores, similarly
the conditions are ordered according to their condition scores. The
score of a gene means the «degree of inclusion» in
the module: a high score gene is essential in the module.

Condition scores can also be negative, that means that the genes of
the module are all down-regulated in the condition. Here the absolute
value of the score gives the «degree of inclusion».

The plots above and beside the expression matrix show the gene scores
and condition scores, respectively.

Note that the plot is interactive, you can see the name of the gene
and condition under the mouse cursor.

The expression matrix was normalized to have mean zero and standard
deviation one for every gene separately across all conditions
(i.e. not just for the conditions in the module).

— Click on the *Help* button again to close this help window.

Gene:   
Condition:

Under-expression is coded with green,
over-expression with red color.

## Help | Hide | Top Help | Show | Top The GO tree — Biological processes

### HELP

This is one of three sections showing Gene Ontology enrichment of the
current module: in this case for **biological processes**.

The graph shows the hierarchy of the GO categories, their enrichment
for the current module is color coded, and the blue number beside the
category is the minus log ten p-value of the enrichment. (Calculated
using the standard hypergeometric test.) The color of the arrows code
«is a» (cyan) and «part of» relationships.

The tree was built the following way. First all GO terms with more
significant enrichment p-value than 0.05 were collected. Then all
paths from these terms to the root node of the GO tree were included
too. If a GO term is included more than once in the tree, then the
green numbers show 1) the id of the node, this makes it easier to find
other appereances of the term, and 2) the number of appearences.

Note that the same GO category might show up on the graph many
times. This is because the GO was «straightened» for this
graph, i.e. if there are more paths from a GO term to the root node of
the tree, all of them are included. The green numbers

Move the mouse cursor over the terms to get their definition. Clicking
on them takes you to the corresponding Gene Ontology web page.

If you cannot see a graph here at all, that means that there were no
significantly enriched GO categories, at the 0.05 level.

— Click on the *Help* button again to close this help window.

## Help | Hide | Top Help | Show | Top The GO tree — Cellular Components

### HELP

This is one of three sections showing Gene Ontology enrichment of the
current module: in this case for **cellular components**.

The graph shows the hierarchy of the GO categories, their enrichment
for the current module is color coded, and the blue number beside the
category is the minus log ten p-value of the enrichment. (Calculated
using the standard hypergeometric test.) The color of the arrows code
«is a» (cyan) and «part of» relationships.

The tree was built the following way. First all GO terms with more
significant enrichment p-value than 0.05 were collected. Then all
paths from these terms to the root node of the GO tree were included
too. If a GO term is included more than once in the tree, then the
green numbers show 1) the id of the node, this makes it easier to find
other appereances of the term, and 2) the number of appearences.

Note that the same GO category might show up on the graph many
times. This is because the GO was «straightened» for this
graph, i.e. if there are more paths from a GO term to the root node of
the tree, all of them are included. The green numbers

Move the mouse cursor over the terms to get their definition. Clicking
on them takes you to the corresponding Gene Ontology web page.

If you cannot see a graph here at all, that means that there were no
significantly enriched GO categories, at the 0.05 level.

— Click on the *Help* button again to close this help window.

## Help | Hide | Top Help | Show | Top The GO tree — Molecular Function

### HELP

This is one of three sections showing Gene Ontology enrichment of the
current module: in this case for **molecular function**.

The graph shows the hierarchy of the GO categories, their enrichment
for the current module is color coded, and the blue number beside the
category is the minus log ten p-value of the enrichment. (Calculated
using the standard hypergeometric test.) The color of the arrows code
«is a» (cyan) and «part of» relationships.

The tree was built the following way. First all GO terms with more
significant enrichment p-value than 0.05 were collected. Then all
paths from these terms to the root node of the GO tree were included
too. If a GO term is included more than once in the tree, then the
green numbers show 1) the id of the node, this makes it easier to find
other appereances of the term, and 2) the number of appearences.

Note that the same GO category might show up on the graph many
times. This is because the GO was «straightened» for this
graph, i.e. if there are more paths from a GO term to the root node of
the tree, all of them are included. The green numbers

Move the mouse cursor over the terms to get their definition. Clicking
on them takes you to the corresponding Gene Ontology web page.

If you cannot see a graph here at all, that means that there were no
significantly enriched GO categories, at the 0.05 level.

— Click on the *Help* button again to close this help window.

## Help | Hide | Top Help | Show | Top GO BP test for over-representation

### HELP

List of all enriched GO categories (biological processes), at the 0.05
p-value level.

The columns:

- **ExpCount** is the expected count of genes in the
  module annotated with the given GO term, just by chance.
- **Count**
  is the number of genes in the module annotated with the given GO
  term.
- **Size** is the total number of genes (in our universe)
  annotated with the GO term.

Clicking on **Count** shows the genes that drive the
enrichment. You can also click on the individual numbers in
the **Count** column, to show the driving genes for that individual
GO category.

Clicking on the GO identifiers takes you to the Gene Ontology web
pages.

— Click on the *Help* button again to close this help window.

No enriched terms

## Help | Hide | Top Help | Show | Top GO CC test for over-representation

### HELP

List of all enriched GO categories (cellular components), at the 0.05
p-value level.

The columns:

- **ExpCount** is the expected count of genes in the
  module annotated with the given GO term, just by chance.
- **Count**
  is the number of genes in the module annotated with the given GO
  term.
- **Size** is the total number of genes (in our universe)
  annotated with the GO term.

Clicking on **Count** shows the genes that drive the
enrichment. You can also click on the individual numbers in
the **Count** column, to show the driving genes for that individual
GO category.

Clicking on the GO identifiers takes you to the Gene Ontology web
pages.

— Click on the *Help* button again to close this help window.

No enriched terms

## Help | Hide | Top Help | Show | Top GO MF test for over-representation

### HELP

List of all enriched GO categories (molecular function), at the 0.05
p-value level.

The columns:

- **ExpCount** is the expected count of genes in the
  module annotated with the given GO term, just by chance.
- **Count**
  is the number of genes in the module annotated with the given GO
  term.
- **Size** is the total number of genes (in our universe)
  annotated with the GO term.

Clicking on **Count** shows the genes that drive the
enrichment. You can also click on the individual numbers in
the **Count** column, to show the driving genes for that individual
GO category.

Clicking on the GO identifiers takes you to the Gene Ontology web
pages.

— Click on the *Help* button again to close this help window.

No enriched terms

## Help | Hide | Top Help | Show | Top KEGG Pathway test for over-representation

### HELP

List of all enriched KEGG pathways, at the 0.05
p-value level.

The columns:

- **ExpCount** is the expected count of genes in the
  module annotated with the given KEGG pathway, just by chance.
- **Count**
  is the number of genes in the module annotated with the given KEGG
  pathway.
- **Size** is the total number of genes (in our universe)
  annotated with the KEGG pathway.

Clicking on **Count** shows the genes that drive the
enrichment. You can also click on the individual numbers in
the **Count** column, to show the driving genes for that individual
KEGG pathway.

Clicking on the KEGG identifiers takes you to the KEGG web site.

— Click on the *Help* button again to close this help window.

No enriched terms


### HELP

List of all enriched miRNA families, at the 0.05
p-value level.

The columns:

- **ExpCount** is the expected count of genes in the
  module regulated by the given miRNA family, just by chance.
- **Count**
  is the number of genes in the module regulated by the given miRNA
  family.
- **Size** is the total number of genes (in our universe)
  regulated with the given miRNA family.

Clicking on **Count** shows the genes that drive the
enrichment. You can also click on the individual numbers in
the **Count** column, to show the driving genes for that individual
miRNA family.

The miRNA regulation data was taken from the

Top


### HELP

p-value level.

The columns:

- **ExpCount** is the expected number of genes in the- **Count**- **Size** is the total number of genes (in our universe)

Clicking on **Count** shows the genes that drive the
enrichment. You can also click on the individual numbers in
the **Count** column, to show the driving genes for that individual

— Click on the *Help* button again to close this help window.

## Help | Hide | Top Help | Show | Top Genes

### HELP

A list of all genes in the current module, in alphabetical order. The
size of the text corresponds to the gene scores.

Note that some gene symbols may show up more than once, if many
probes match the same Entrez gene.

Genes with no Entrez mapping are given separately, with their
Affymetrics probe ID.

— Click on the *Help* button again to close this help window.

### Genes Symbol

, score:

AatkUnknown, score: 0.22
Abca4Unknown, score: 0.53
Abca2Unknown, score: 0.04
Abcb7Unknown, score: 0.36
Abi1Unknown, score: 0.3
Abl1Unknown, score: 0.51
AcadmUnknown, score: 0.09
AcadvlUnknown, score: 0.38
AcadsUnknown, score: 0.05
Slc33a1Unknown, score: 0.02
Asic1Unknown, score: 0.09
AceUnknown, score: 0.49
Apoc4Unknown, score: 0.28
Macf1Unknown, score: 0.14
Aco1Unknown, score: 0.14
Acox1Unknown, score: 0.17
Acp1Unknown, score: 0.46
Acp2Unknown, score: 0.43
AcrUnknown, score: 0.2
ActbUnknown, score: 0.45
Actc1Unknown, score: 0.43
Actg1Unknown, score: 0.12
Actg2Unknown, score: 0.06
Actl7bUnknown, score: 0.23
Acta2Unknown, score: 0.28
Acvr2aUnknown, score: 0.23
Acvr2bUnknown, score: 0.13
AspaUnknown, score: 0.18
Adam12Unknown, score: 0.43
Adam15Unknown, score: 0.37
Adam22Unknown, score: 0.23
Adam5Unknown, score: 0.5
Adam8Unknown, score: 0.06
Adamts1Unknown, score: 0.2
Adcy6Unknown, score: 0.05
Adcy8Unknown, score: 0.45
Adcyap1r1Unknown, score: 0.25
Add1Unknown, score: 0.13
Gpr182Unknown, score: 0.23
CfdUnknown, score: 0.46
AdnpUnknown, score: 0.25
Adora2aUnknown, score: 0.46
Adora3Unknown, score: 0.06
Parp2Unknown, score: 0.64
Adra1aUnknown, score: 0.35
Adra2bUnknown, score: 0.29
Adra2cUnknown, score: 0.3
Adrb1Unknown, score: 0.19
Adrb2Unknown, score: 0.12
AdslUnknown, score: 0.62
AdssUnknown, score: 0.11
AvilUnknown, score: 0.24
Aebp2Unknown, score: 0.26
AgaUnknown, score: 0.45
Angpt1Unknown, score: 0.07
Angpt2Unknown, score: 0.29
Angpt4Unknown, score: 0.19
AgrnUnknown, score: 0.32
AgrpUnknown, score: 0.08
GlaUnknown, score: 0.27
Agtr2Unknown, score: 0.08
AgxtUnknown, score: 0.09
Aim1Unknown, score: 0.53
AipUnknown, score: 0.22
AireUnknown, score: 0.17
Ak2Unknown, score: 0.42
Akap1Unknown, score: 0.2
Akap2Unknown, score: 0.39
AlplUnknown, score: 0.08
Akt1Unknown, score: 0.14
Alas1Unknown, score: 0.15
AlcamUnknown, score: 0.29
Aldh1a1Unknown, score: 0.32
Aldh3a1Unknown, score: 0.14
Aldh3a2Unknown, score: 0.14
AldoaUnknown, score: 0.22
AldocUnknown, score: 0.27
Akr1b3Unknown, score: 0.1
Alox5Unknown, score: 0.1
Alox5apUnknown, score: 0.25
GferUnknown, score: 0.05
AmbpUnknown, score: 0.55
Amd1Unknown, score: 0.07
AngUnknown, score: 0.18
Ank1Unknown, score: 0.08
Ank3Unknown, score: 0.11
Anp32aUnknown, score: 0.11
Slc25a4Unknown, score: 0.31
Slc25a5Unknown, score: 0.17
Anxa11Unknown, score: 0.17
Anxa7Unknown, score: 0.35
Anxa8Unknown, score: 0.3
Aoc3Unknown, score: 0.14
Aox1Unknown, score: 0.2
Ap1g1Unknown, score: 0.26
Ap1g2Unknown, score: 0.1
Ap1m1Unknown, score: 0.1
Fabp4Unknown, score: 0.3
Ap2a1Unknown, score: 0.14
Ap2a2Unknown, score: 0.26
Ap3b1Unknown, score: 0.16
Ap3b2Unknown, score: 0.11
Ap3s1Unknown, score: 0.12
Ap3s2Unknown, score: 0.18
Apba2Unknown, score: 0.13
ApcUnknown, score: 0.14
SpegUnknown, score: 0.27
Birc5Unknown, score: 0.04
Cd5lUnknown, score: 0.12
Apoc3Unknown, score: 0.41
Nr2f2Unknown, score: 0.03
AppUnknown, score: 0.31
Aqp2Unknown, score: 0.44
ArUnknown, score: 0.06
ArafUnknown, score: 0.21
ArcUnknown, score: 0.15
AregUnknown, score: 0.34
Arf1Unknown, score: 0.25
Arf2Unknown, score: 0.2
Arf4Unknown, score: 0.23
Arf6Unknown, score: 0.1
Arg1Unknown, score: 0.25
RhocUnknown, score: 0.16
ArhgdibUnknown, score: 0.19
Rnd2Unknown, score: 0.39
ArntUnknown, score: 0.19
Arnt2Unknown, score: 0.11
ArntlUnknown, score: 0.15
Art1Unknown, score: 0.47
Art5Unknown, score: 0.28
ArsbUnknown, score: 0.18
Rab27aUnknown, score: 0.09
Ass1Unknown, score: 0.18
Ate1Unknown, score: 0.06
Atf2Unknown, score: 0.2
Atf3Unknown, score: 0.09
AtmUnknown, score: 0.17
Atox1Unknown, score: 0.22
Atp1a1Unknown, score: 0.15
Atp1b2Unknown, score: 0.14
Atp2b2Unknown, score: 0.28
Atp4aUnknown, score: 0.08
Atp4bUnknown, score: 0.13
Atp5a1Unknown, score: 0.01
Atp5f1Unknown, score: 0.04
Atp5jUnknown, score: 0.14
Atp5kUnknown, score: 0.03
Atp6v1aUnknown, score: 0.12
Atp6v1b2Unknown, score: 0.06
Atp6v1e1Unknown, score: 0.17
Atp6v0a1Unknown, score: 0.01
Atp7aUnknown, score: 0.05
Atp8a1Unknown, score: 0.43
Atp9aUnknown, score: 0.27
Atpif1Unknown, score: 0.21
Atp6v0cUnknown, score: 0.64
Slc7a1Unknown, score: 0.2
Slc7a2Unknown, score: 0.17
Slc7a3Unknown, score: 0.33
AuhUnknown, score: 0.12
Pcdh15Unknown, score: 0.09
Avpr2Unknown, score: 0.49
Axin1Unknown, score: 0.51
Azgp1Unknown, score: 0.2
Bach2Unknown, score: 0.23
Bag1Unknown, score: 0.09
Bard1Unknown, score: 0.09
Barx2Unknown, score: 0.45
Phb2Unknown, score: 0.23
Bcat1Unknown, score: 0.29
Bcat2Unknown, score: 0.27
BcheUnknown, score: 0.19
BckdhaUnknown, score: 0.09
BckdhbUnknown, score: 0.3
BckdkUnknown, score: 0.12
Bcl2Unknown, score: 0.27
Bcl2a1bUnknown, score: 0.14
Bcl2l2Unknown, score: 0.4
Bcl3Unknown, score: 0.19
Opn1swUnknown, score: 0.06
Bdkrb1Unknown, score: 0.14
Bet1Unknown, score: 0.02
Bfsp1Unknown, score: 0.15
Bglap3Unknown, score: 0.15
HrkUnknown, score: 0.29
BikUnknown, score: 0.39
Bmi1Unknown, score: 0.18
Bmp10Unknown, score: 0.18
Bmp5Unknown, score: 0.47
Bmp6Unknown, score: 0.09
Bmpr1bUnknown, score: 0.2
BmxUnknown, score: 0.13
Bnip2Unknown, score: 0.21
Smyd1Unknown, score: 0.46
Bst1Unknown, score: 0.05
Brca2Unknown, score: 0.01
Zfp36l1Unknown, score: 0.25
Chic1Unknown, score: 0.03
Klf5Unknown, score: 0.11
BtkUnknown, score: 0.19
Bub1bUnknown, score: 0.25
Commd3Unknown, score: 0.61
TspoUnknown, score: 0.38
Serping1Unknown, score: 0.02
C1qbpUnknown, score: 0.1
C1qcUnknown, score: 0.04
C3ar1Unknown, score: 0.37
C4bpUnknown, score: 0.52
C5ar1Unknown, score: 0.05
Hyou1Unknown, score: 0.09
Cacna1bUnknown, score: 0.31
Cacna1dUnknown, score: 0.06
Cacna1eUnknown, score: 0.63
Cacna2d1Unknown, score: 0.1
Cacnb1Unknown, score: 0.36
Cacnb2Unknown, score: 0.48
Pdia4Unknown, score: 0.44
Ddr1Unknown, score: 0.37
Anxa2Unknown, score: 0.19
Calm1Unknown, score: 0.19
Calm2Unknown, score: 0.19
CalrUnknown, score: 0.08
CaluUnknown, score: 0.04
Cap1Unknown, score: 0.35
CapgUnknown, score: 0.45
Capn2Unknown, score: 0.28
Capn3Unknown, score: 0.22
Capns1Unknown, score: 0.12
Capn7Unknown, score: 0.15
CapzbUnknown, score: 0.16
Car11Unknown, score: 0.08
Car4Unknown, score: 0.24
Casp12Unknown, score: 0.21
Casp2Unknown, score: 0.15
Casp3Unknown, score: 0.04
Casp8Unknown, score: 0.42
Casq1Unknown, score: 0.04
Casq2Unknown, score: 0.13
CastUnknown, score: 0.18
Cav2Unknown, score: 0.21
Runx2Unknown, score: 0.21
Runx1t1Unknown, score: 0.27
Cbfa2t2Unknown, score: 0.09
Cbfa2t3Unknown, score: 0.27
Serpina6Unknown, score: 0.04
CblUnknown, score: 0.03
Cbln1Unknown, score: 0.12
Cbr1Unknown, score: 0.29
Cbr2Unknown, score: 0.05
Cbx1Unknown, score: 0.42
Cbx3Unknown, score: 0.41
Cbx4Unknown, score: 0.13
Ccnd2Unknown, score: 0.19
Ccnd3Unknown, score: 0.06
Ccne1Unknown, score: 0.02
Ccne2Unknown, score: 0.05
CcniUnknown, score: 0.03
Ccr6Unknown, score: 0.36
Cct4Unknown, score: 0.05
Cct5Unknown, score: 0.11
Cct8Unknown, score: 0.34
Cd14Unknown, score: 0.29
Cd151Unknown, score: 0.21
Cd1d1Unknown, score: 0.15
Cd22Unknown, score: 0.28
Cd24aUnknown, score: 0.22
Cd2apUnknown, score: 0.14
Cd33Unknown, score: 0.05
Scarb2Unknown, score: 0.16
Cd38Unknown, score: 0.09
Entpd1Unknown, score: 0.32
Entpd5Unknown, score: 0.2
Cd3dUnknown, score: 0.18
Cd247Unknown, score: 0.21
Cd4Unknown, score: 0.12
Cd48Unknown, score: 0.12
Cd53Unknown, score: 0.08
Cd59aUnknown, score: 0.33
Cd6Unknown, score: 0.09
Cd63Unknown, score: 0.22
Cd68Unknown, score: 0.19
Cd69Unknown, score: 0.07
Cd79aUnknown, score: 0.09
Cd80Unknown, score: 0.07
Cd86Unknown, score: 0.02
Cd8aUnknown, score: 0.16
Cd8b1Unknown, score: 0.18
Cdc25aUnknown, score: 0.13
Arhgap31Unknown, score: 0.19
Cdh1Unknown, score: 0.08
Cdh11Unknown, score: 0.16
Cdh13Unknown, score: 0.03
Cdh15Unknown, score: 0.41
Cdh16Unknown, score: 0.21
Cdh17Unknown, score: 0.13
Cdh2Unknown, score: 0.12
Cdh5Unknown, score: 0.27
Cdh6Unknown, score: 0.2
Cdh8Unknown, score: 0.33
Cdk2Unknown, score: 0.01
Cdk4Unknown, score: 0.41
Cdk7Unknown, score: 0.2
Cdkn2aUnknown, score: 0.31
Cdkn2bUnknown, score: 0.13
Cdkn2cUnknown, score: 0.57
Cdo1Unknown, score: 0.09
Ift81Unknown, score: 0.07
CdylUnknown, score: 0.22
CebpzUnknown, score: 0.51
CebpgUnknown, score: 0.21
Celsr1Unknown, score: 0.22
CenpaUnknown, score: 0.3
CfhUnknown, score: 0.2
CfiUnknown, score: 0.02
CflarUnknown, score: 0.37
Ch25hUnknown, score: 0.06
ChgbUnknown, score: 0.42
Chil1Unknown, score: 0.23
ChmUnknown, score: 0.06
ChmlUnknown, score: 0.32
InadlUnknown, score: 0.3
CirbpUnknown, score: 0.18
CishUnknown, score: 0.55
Socs3Unknown, score: 0.12
Socs1Unknown, score: 0.4
CitUnknown, score: 0.02
Cited1Unknown, score: 0.63
CkbUnknown, score: 0.1
CkmUnknown, score: 0.08
Ckmt1Unknown, score: 0.31
Clcn1Unknown, score: 0.14
Clcn4-2Unknown, score: 0.08
Clcn5Unknown, score: 0.02
Clns1aUnknown, score: 0.15
Clk4Unknown, score: 0.41
Cln3Unknown, score: 0.09
CltaUnknown, score: 0.4
CmasUnknown, score: 0.21
Cxcr2Unknown, score: 0.15
Cxcr4Unknown, score: 0.21
Ccr1Unknown, score: 0.13
Ccr5Unknown, score: 0.29
Ccr7Unknown, score: 0.43
Ccr10Unknown, score: 0.17
CnbpUnknown, score: 0.03
Cnih1Unknown, score: 0.28
Cnih2Unknown, score: 0.04
Plk3Unknown, score: 0.16
CnpUnknown, score: 0.16
Cnr2Unknown, score: 0.1
CoilUnknown, score: 0.1
Col11a2Unknown, score: 0.1
Col13a1Unknown, score: 0.05
Col15a1Unknown, score: 0.47
Col17a1Unknown, score: 0.2
Col18a1Unknown, score: 0.15
Col19a1Unknown, score: 0.11
Col4a4Unknown, score: 0.24
Col4a5Unknown, score: 0.04
Col5a1Unknown, score: 0.05
Col6a2Unknown, score: 0.04
Col6a3Unknown, score: 0.23
Col9a2Unknown, score: 0.03
Col9a3Unknown, score: 0.05
Col1a1Unknown, score: 0.03
Cops2Unknown, score: 0.13
CortUnknown, score: 0.03
Cox17Unknown, score: 0.2
Cox4i1Unknown, score: 0.46
Cox5aUnknown, score: 0.32
Cox6a1Unknown, score: 0.04
Cox6a2Unknown, score: 0.06
Cox6cUnknown, score: 0.05
Cox7a1Unknown, score: 0.02
Cox7a2Unknown, score: 0.09
Cox7cUnknown, score: 0.11
Cox8aUnknown, score: 0.08
Cpa3Unknown, score: 0.08
Cys1Unknown, score: 0.13
Cplx2Unknown, score: 0.02
Cpne6Unknown, score: 0.51
Cr2Unknown, score: 0.34
Crabp1Unknown, score: 0.07
Creb3Unknown, score: 0.17
CrebbpUnknown, score: 0.25
CremUnknown, score: 0.11
CrhUnknown, score: 0.3
CrhbpUnknown, score: 0.38
Crhr2Unknown, score: 0.02
Crip1Unknown, score: 0.28
Bcar1Unknown, score: 0.09
CrkUnknown, score: 0.27
CrklUnknown, score: 0.37
Dpysl2Unknown, score: 0.12
Pcdha4Unknown, score: 0.12
Pcdha11Unknown, score: 0.12
Pcdha11Unknown, score: 0.04
Cr1lUnknown, score: 0.02
Cry1Unknown, score: 0.4
CryabUnknown, score: 0.06
Cryba4Unknown, score: 0.45
Crybb1Unknown, score: 0.03
CrymUnknown, score: 0.33
CryzUnknown, score: 0.16
Csf2raUnknown, score: 0.27
Csf2rbUnknown, score: 0.21
Csf2rb2Unknown, score: 0.14
Csf3rUnknown, score: 0.03
Csnk2a2Unknown, score: 0.07
Csnk2bUnknown, score: 0.23
Dnajc5Unknown, score: 0.05
Csrp1Unknown, score: 0.34
Csrp3Unknown, score: 0.05
Cst3Unknown, score: 0.17
Cst7Unknown, score: 0.25
Ctbp1Unknown, score: 0.04
CtcfUnknown, score: 0.04
Ctf1Unknown, score: 0.28
Ctla2aUnknown, score: 0.27
Ctla2bUnknown, score: 0.07
Pcyt1aUnknown, score: 0.24
CtscUnknown, score: 0.43
CtseUnknown, score: 0.28
CtskUnknown, score: 0.12
CtslUnknown, score: 0.02
CtssUnknown, score: 0.16
CtswUnknown, score: 0.38
CttnUnknown, score: 0.08
Celf1Unknown, score: 0.19
Cux1Unknown, score: 0.11
CxadrUnknown, score: 0.37
Cyb561Unknown, score: 0.11
CycsUnknown, score: 0.31
CyctUnknown, score: 0.37
Cyp1a2Unknown, score: 0.18
Cyp1b1Unknown, score: 0.13
Cyp2a5Unknown, score: 0.39
Cyp3a11Unknown, score: 0.48
Cyp3a13Unknown, score: 0.06
Cyp27b1Unknown, score: 0.26
Cyp4a14Unknown, score: 0.04
Cyp7a1Unknown, score: 0.12
Cyp7b1Unknown, score: 0.32
Dach1Unknown, score: 0.12
Cd55Unknown, score: 0.5
DgkaUnknown, score: 0.02
DaoUnknown, score: 0.24
Dapk2Unknown, score: 0.4
DaxxUnknown, score: 0.1
DazlUnknown, score: 0.21
Dbil5Unknown, score: 0.03
DbnlUnknown, score: 0.17
DbtUnknown, score: 0.12
Pcbd1Unknown, score: 0.04
Dscr3Unknown, score: 0.23
DctUnknown, score: 0.05
Dctn1Unknown, score: 0.11
Gadd45aUnknown, score: 0.11
Ddit3Unknown, score: 0.13
DdnUnknown, score: 0.04
DdostUnknown, score: 0.15
Dhx15Unknown, score: 0.1
Ddx4Unknown, score: 0.04
Slc29a2Unknown, score: 0.29
Twist2Unknown, score: 0.06
DesUnknown, score: 0.1
Ackr1Unknown, score: 0.17
Dgat1Unknown, score: 0.36
Dgcr6Unknown, score: 0.42
Dgcr2Unknown, score: 0.26
Slc25a1Unknown, score: 0.25
DhfrUnknown, score: 0.41
DhhUnknown, score: 0.04
Diap1Unknown, score: 0.27
DffbUnknown, score: 0.17
DldUnknown, score: 0.06
Dlg1Unknown, score: 0.28
Mpp3Unknown, score: 0.15
Dlk1Unknown, score: 0.02
Dlx4Unknown, score: 0.16
Dlx5Unknown, score: 0.1
DmpkUnknown, score: 0.22
DmdUnknown, score: 0.22
Dnajc1Unknown, score: 0.08
Dnase1Unknown, score: 0.07
Dnase1l3Unknown, score: 0.2
Dnase2aUnknown, score: 0.08
Dync1h1Unknown, score: 0.04
Dync1i2Unknown, score: 0.35
Dnm2Unknown, score: 0.16
Dnmt3aUnknown, score: 0.07
Dnmt3bUnknown, score: 0.06
DnpepUnknown, score: 0.34
Cdk2ap1Unknown, score: 0.03
Dok1Unknown, score: 0.12
Dok2Unknown, score: 0.44
Reep5Unknown, score: 0.31
Dpagt1Unknown, score: 0.05
Dpm1Unknown, score: 0.49
Dpp4Unknown, score: 0.56
Dr1Unknown, score: 0.06
Drg1Unknown, score: 0.09
Arid3aUnknown, score: 0.02
Drp2Unknown, score: 0.5
Atn1Unknown, score: 0.02
Dsc1Unknown, score: 0.27
Dsg1aUnknown, score: 0.1
Slc26a2Unknown, score: 0.23
DtnaUnknown, score: 0.23
Usp17laUnknown, score: 0.18
Dusp2Unknown, score: 0.27
Dvl2Unknown, score: 0.57
Dvl3Unknown, score: 0.43
Dyrk1aUnknown, score: 0.44
Dyrk1bUnknown, score: 0.15
E2f5Unknown, score: 0.24
Mapre1Unknown, score: 0.15
Ebf3Unknown, score: 0.34
EbpUnknown, score: 0.43
Sparcl1Unknown, score: 0.2
Ect2Unknown, score: 0.09
EdaUnknown, score: 0.08
S1pr3Unknown, score: 0.31
S1pr4Unknown, score: 0.11
Edil3Unknown, score: 0.12
Edn3Unknown, score: 0.29
Phc1Unknown, score: 0.07
Eef1a1Unknown, score: 0.08
Eef1a2Unknown, score: 0.22
Eef2Unknown, score: 0.1
Efna2Unknown, score: 0.18
Efna3Unknown, score: 0.15
Efna4Unknown, score: 0.19
Efna5Unknown, score: 0.31
Efnb2Unknown, score: 0.35
Efnb3Unknown, score: 0.53
Klk1b22Unknown, score: 0.26
EgfrUnknown, score: 0.56
Egr1Unknown, score: 0.28
Egr2Unknown, score: 0.31
Egr3Unknown, score: 0.09
Ddx19aUnknown, score: 0.05
Eif4ebp1Unknown, score: 0.06
Elk4Unknown, score: 0.28
EllUnknown, score: 0.48
Aimp1Unknown, score: 0.06
EmbUnknown, score: 0.04
EmdUnknown, score: 0.2
Emp1Unknown, score: 0.14
Emp2Unknown, score: 0.5
Emp3Unknown, score: 0.06
Adgre1Unknown, score: 0.08
Emx2Unknown, score: 0.39
EnahUnknown, score: 0.13
Enc1Unknown, score: 0.55
EndogUnknown, score: 0.72
EngUnknown, score: 0.06
Eno1Unknown, score: 0.11
Eno2Unknown, score: 0.24
Epas1Unknown, score: 0.12
Epb4.2Unknown, score: 0.36
Epc1Unknown, score: 0.16
Epha1Unknown, score: 0.05
Epha2Unknown, score: 0.16
Epha3Unknown, score: 0.38
Ephb2Unknown, score: 0.19
Ephb3Unknown, score: 0.13
Ephb4Unknown, score: 0.37
Ephb6Unknown, score: 0.06
Ephx2Unknown, score: 0.1
Stx2Unknown, score: 0.08
Epm2aUnknown, score: 0.45
Epn1Unknown, score: 0.4
Eps15Unknown, score: 0.07
Eps8Unknown, score: 0.84
Nr2f1Unknown, score: 0.35
Erbb3Unknown, score: 0.07
Erbb4Unknown, score: 0.18
Ercc3Unknown, score: 0.22
EregUnknown, score: 0.28
Amz2Unknown, score: 0.38
Khdrbs3Unknown, score: 0.34
DroshaUnknown, score: 0.49
Bcl11aUnknown, score: 0.02
EvplUnknown, score: 0.12
Ext1Unknown, score: 0.3
Eya1Unknown, score: 0.34
Eya2Unknown, score: 0.15
Eya3Unknown, score: 0.14
Sfxn1Unknown, score: 0.07
F2rl1Unknown, score: 0.15
F2rl3Unknown, score: 0.1
F5Unknown, score: 0.04
F7Unknown, score: 0.12
F8Unknown, score: 0.42
Faf1Unknown, score: 0.04
Fscn1Unknown, score: 0.54
FancaUnknown, score: 0.05
FapUnknown, score: 0.09
FasUnknown, score: 0.05
FaslUnknown, score: 0.57
Srsf10Unknown, score: 0.08
Fat1Unknown, score: 0.24
FblUnknown, score: 0.33
Fbln1Unknown, score: 0.59
Fbln2Unknown, score: 0.16
Fbp1Unknown, score: 0.1
Fcer1aUnknown, score: 0.13
Ms4a2Unknown, score: 0.02
FcgrtUnknown, score: 0.32
FcnaUnknown, score: 0.26
Fdft1Unknown, score: 0.54
Fdx1Unknown, score: 0.58
FechUnknown, score: 0.42
Fem1bUnknown, score: 0.2
FerUnknown, score: 0.18
FesUnknown, score: 0.03
FgaUnknown, score: 0.24
Fgf1Unknown, score: 0.03
Fgf10Unknown, score: 0.11
Fgf18Unknown, score: 0.08
Fgfbp1Unknown, score: 0.25
Fgfr2Unknown, score: 0.62
Fgfr3Unknown, score: 0.18
Fgfr4Unknown, score: 0.33
Akr1b8Unknown, score: 0.3
Fgl2Unknown, score: 0.22
FhitUnknown, score: 0.47
Fhl1Unknown, score: 0.18
Smc2Unknown, score: 0.23
Sh3pxd2aUnknown, score: 0.21
CtgfUnknown, score: 0.25
Fkbp4Unknown, score: 0.09
Fkbp7Unknown, score: 0.33
Fkbp8Unknown, score: 0.32
Foxn2Unknown, score: 0.08
Foxs1Unknown, score: 0.44
Fli1Unknown, score: 0.11
FliiUnknown, score: 0.31
Flt3Unknown, score: 0.12
Flt4Unknown, score: 0.01
Fmn1Unknown, score: 0.2
Fmo1Unknown, score: 0.05
Fmr1Unknown, score: 0.16
Aff2Unknown, score: 0.28
Folr2Unknown, score: 0.18
FosUnknown, score: 0.19
FosbUnknown, score: 0.16
Fosl1Unknown, score: 0.34
Fosl2Unknown, score: 0.29
FpgsUnknown, score: 0.18
Fpr2Unknown, score: 0.47
Fpr1Unknown, score: 0.33
FxnUnknown, score: 0.37
FrkUnknown, score: 0.09
CidecUnknown, score: 0.09
FstUnknown, score: 0.19
Fstl1Unknown, score: 0.04
FtcdUnknown, score: 0.51
Fut4Unknown, score: 0.15
Fv1Unknown, score: 0.24
Timm10bUnknown, score: 0.27
Fxr1Unknown, score: 0.11
FynUnknown, score: 0.39
Fzd1Unknown, score: 0.13
Fzd3Unknown, score: 0.18
Fzd7Unknown, score: 0.1
Xrcc6Unknown, score: 0.01
G6pcUnknown, score: 0.21
G6pdxUnknown, score: 0.12
Slc37a4Unknown, score: 0.42
GaaUnknown, score: 0.24
GabreUnknown, score: 0.42
Gabrr1Unknown, score: 0.38
Gabrr2Unknown, score: 0.02
Slc6a13Unknown, score: 0.07
GalUnknown, score: 0.19
GalcUnknown, score: 0.51
B4galnt1Unknown, score: 0.26
Galnt1Unknown, score: 0.09
Galnt4Unknown, score: 0.18
Galr2Unknown, score: 0.24
GaltUnknown, score: 0.23
GapdhUnknown, score: 0.07
GapdhsUnknown, score: 0.05
GartUnknown, score: 0.27
Gas2Unknown, score: 0.17
Gas7Unknown, score: 0.26
Gata1Unknown, score: 0.1
Gata2Unknown, score: 0.23
Gata4Unknown, score: 0.04
Gata5Unknown, score: 0.52
Gata6Unknown, score: 0.29
Gbp2Unknown, score: 0.23
Rabac1Unknown, score: 0.22
GcsamUnknown, score: 0.04
GcgrUnknown, score: 0.34
Gcm1Unknown, score: 0.3
Bloc1s1Unknown, score: 0.05
Kat2aUnknown, score: 0.12
Nr6a1Unknown, score: 0.33
Gcnt1Unknown, score: 0.14
Mrps33Unknown, score: 0.02
Gdf11Unknown, score: 0.12
Gdf9Unknown, score: 0.07
Gdi2Unknown, score: 0.52
GdnfUnknown, score: 0.36
Gfpt1Unknown, score: 0.21
Gfpt2Unknown, score: 0.02
Gfra2Unknown, score: 0.13
Gfra3Unknown, score: 0.14
Ggta1Unknown, score: 0.15
B4galt1Unknown, score: 0.04
GhrUnknown, score: 0.33
Gpr83Unknown, score: 0.01
Gja1Unknown, score: 0.51
Gja3Unknown, score: 0.17
Gjb3Unknown, score: 0.19
GclmUnknown, score: 0.07
Gli1Unknown, score: 0.24
Gli3Unknown, score: 0.4
Glrp1Unknown, score: 0.15
Glud1Unknown, score: 0.38
Gnl1Unknown, score: 0.05
Gna11Unknown, score: 0.26
Gna12Unknown, score: 0.12
Gna13Unknown, score: 0.06
Gnai1Unknown, score: 0.31
Gnai3Unknown, score: 0.1
GnaqUnknown, score: 0.13
GnasUnknown, score: 0.26
Gnat2Unknown, score: 0.16
Gnb1Unknown, score: 0.52
Gnb2Unknown, score: 0.28
Gnb4Unknown, score: 0.28
Gng2Unknown, score: 0.21
Gng3Unknown, score: 0.49
Gng4Unknown, score: 0.31
Gng5Unknown, score: 0.37
Gng7Unknown, score: 0.16
Gngt2Unknown, score: 0.22
GnpatUnknown, score: 0.14
Gnrh1Unknown, score: 0.1
GnrhrUnknown, score: 0.24
Got1Unknown, score: 0.11
Gp1baUnknown, score: 0.13
Gp49aUnknown, score: 0.12
Lilrb4Unknown, score: 0.26
Gp5Unknown, score: 0.95
Gpc1Unknown, score: 0.05
Gpr12Unknown, score: 0.29
Gpr65Unknown, score: 0.14
Lpar1Unknown, score: 0.02
Gpr3Unknown, score: 0.11
Gpi1Unknown, score: 0.1
PigqUnknown, score: 0.04
Gpm6bUnknown, score: 0.06
Gpr19Unknown, score: 0.45
Gpr37Unknown, score: 0.16
Ptgdr2Unknown, score: 0.05
Gpr50Unknown, score: 0.19
Grk4Unknown, score: 0.11
Grk5Unknown, score: 0.18
Gpx1Unknown, score: 0.18
Grb7Unknown, score: 0.03
Gpr162Unknown, score: 0.21
P3h3Unknown, score: 0.21
Grcc10Unknown, score: 0.16
Lpcat3Unknown, score: 0.06
Cdca3Unknown, score: 0.22
Spsb2Unknown, score: 0.13
Grik3Unknown, score: 0.38
Grik5Unknown, score: 0.15
Grin2aUnknown, score: 0.47
Grin2cUnknown, score: 0.05
Nr3c1Unknown, score: 0.32
Grm1Unknown, score: 0.12
GrnUnknown, score: 0.42
Gspt2Unknown, score: 0.3
GssUnknown, score: 0.18
Gsta4Unknown, score: 0.57
Gstm1Unknown, score: 0.21
Gstm2Unknown, score: 0.1
Gstp2Unknown, score: 0.25
Gstt1Unknown, score: 0.19
Gstt2Unknown, score: 0.05
Gtf2h1Unknown, score: 0.27
Gtf2h4Unknown, score: 0.12
Gtf2iUnknown, score: 0.05
Trip12Unknown, score: 0.51
Thumpd3Unknown, score: 0.03
Guca1aUnknown, score: 0.02
Gucy2dUnknown, score: 0.04
GykUnknown, score: 0.01
GypaUnknown, score: 0.41
GzmkUnknown, score: 0.27
H13Unknown, score: 0.24
H2-Ab1Unknown, score: 0.16
H2-BlUnknown, score: 0.25
Pfdn6Unknown, score: 0.26
Slc39a7Unknown, score: 0.04
H2-Ke6Unknown, score: 0.25
H2-D1Unknown, score: 0.1
H2-M2Unknown, score: 0.11
H2-Q1Unknown, score: 0.09
H2-Q4Unknown, score: 0.03
H2-Q6Unknown, score: 0.52
H2-T10Unknown, score: 0.08
H2-T24Unknown, score: 0.08
Hist2h3c1Unknown, score: 0.11
H3f3aUnknown, score: 0.11
Hsd17b10Unknown, score: 0.08
HalUnknown, score: 0.41
Hap1Unknown, score: 0.04
HarsUnknown, score: 0.3
Has1Unknown, score: 0.17
Hba-a1Unknown, score: 0.23
Hbb-bsUnknown, score: 0.23
Serpind1Unknown, score: 0.25
Hcfc1Unknown, score: 0.04
Hcls1Unknown, score: 0.11
Hcn2Unknown, score: 0.1
Hdac3Unknown, score: 0.2
Hdac5Unknown, score: 0.19
Hdac6Unknown, score: 0.09
Hdgfrp2Unknown, score: 0.22
HephUnknown, score: 0.3
Hesx1Unknown, score: 0.16
HexbUnknown, score: 0.26
Hey2Unknown, score: 0.38
HfeUnknown, score: 0.04
Foxq1Unknown, score: 0.24
Foxj1Unknown, score: 0.3
HgdUnknown, score: 0.08
Mst1Unknown, score: 0.02
HhexUnknown, score: 0.05
Hiat1Unknown, score: 0.3
Hif1aUnknown, score: 0.55
Hint1Unknown, score: 0.37
Hipk1Unknown, score: 0.41
Hipk2Unknown, score: 0.24
Hipk3Unknown, score: 0.53
HiraUnknown, score: 0.41
H2afxUnknown, score: 0.18
Hivep2Unknown, score: 0.27
Hk1Unknown, score: 0.12
Tfb2mUnknown, score: 0.18
HlxUnknown, score: 0.33
Hmgb1Unknown, score: 0.09
Hmgn2Unknown, score: 0.45
Hmg20bUnknown, score: 0.02
Hmgb3Unknown, score: 0.09
Hmgcs2Unknown, score: 0.49
Hmga1Unknown, score: 0.04
HmmrUnknown, score: 0.12
Hmox2Unknown, score: 0.4
Nr4a1Unknown, score: 0.07
Hnrnpa1Unknown, score: 0.31
HnrnpabUnknown, score: 0.18
HnrnpkUnknown, score: 0.15
HnrnplUnknown, score: 0.13
Hoxa2Unknown, score: 0.06
Hoxa3Unknown, score: 0.11
Hoxa5Unknown, score: 0.03
Hoxa9Unknown, score: 0.12
Hoxb4Unknown, score: 0.08
Hoxb5Unknown, score: 0.08
Hoxb7Unknown, score: 0.32
Hoxd3Unknown, score: 0.32
Hoxd8Unknown, score: 0.15
Hoxd9Unknown, score: 0.32
HpseUnknown, score: 0.28
HpxUnknown, score: 0.16
HrUnknown, score: 0.44
HrcUnknown, score: 0.06
Hrh1Unknown, score: 0.04
Eif2ak1Unknown, score: 0.33
Prmt2Unknown, score: 0.02
Prmt1Unknown, score: 0.06
Hrsp12Unknown, score: 0.41
Hspa8Unknown, score: 0.11
Hsd11b1Unknown, score: 0.08
Hsd3b5Unknown, score: 0.28
Hspd1Unknown, score: 0.22
Hspa1bUnknown, score: 0.07
Hspa9Unknown, score: 0.06
Sdc2Unknown, score: 0.13
Hspg2Unknown, score: 0.32
Ndst1Unknown, score: 0.29
Htr2aUnknown, score: 0.21
Htr2bUnknown, score: 0.1
Htr7Unknown, score: 0.19
Elavl1Unknown, score: 0.28
Elavl2Unknown, score: 0.2
Elavl3Unknown, score: 0.03
Elavl4Unknown, score: 0.18
Hus1Unknown, score: 0.36
Hyal1Unknown, score: 0.44
Ica1Unknown, score: 0.41
Icam1Unknown, score: 0.18
Icam2Unknown, score: 0.25
Irf8Unknown, score: 0.47
Id1Unknown, score: 0.08
Id2Unknown, score: 0.34
Id3Unknown, score: 0.07
Idh1Unknown, score: 0.02
IdsUnknown, score: 0.1
IduaUnknown, score: 0.15
Ier2Unknown, score: 0.69
Ifnar1Unknown, score: 0.08
Ifngr2Unknown, score: 0.18
Igf1Unknown, score: 0.17
Igf1rUnknown, score: 0.11
Igf2Unknown, score: 0.08
Igf2rUnknown, score: 0.05
Igfbp3Unknown, score: 0.17
Igfbp4Unknown, score: 0.19
Igfbp5Unknown, score: 0.19
IgtpUnknown, score: 0.06
Cd74Unknown, score: 0.22
Il11Unknown, score: 0.2
Il11ra1Unknown, score: 0.26
Gm13305Unknown, score: 0.03
Il12aUnknown, score: 0.1
Il13ra1Unknown, score: 0.17
Il16Unknown, score: 0.37
Il18rapUnknown, score: 0.08
Il1aUnknown, score: 0.05
Il1r1Unknown, score: 0.14
Il1r2Unknown, score: 0.12
Il1rapUnknown, score: 0.25
Il2rgUnknown, score: 0.52
Il3raUnknown, score: 0.38
Il4raUnknown, score: 0.04
Il5Unknown, score: 0.08
Il5raUnknown, score: 0.15
Il6raUnknown, score: 0.04
Il7Unknown, score: 0.22
Il7rUnknown, score: 0.1
Il9rUnknown, score: 0.26
IlkUnknown, score: 0.02
Gimap1Unknown, score: 0.46
Lrig1Unknown, score: 0.02
IncenpUnknown, score: 0.44
InhaUnknown, score: 0.41
InhbaUnknown, score: 0.21
Inpp1Unknown, score: 0.09
Inpp5bUnknown, score: 0.03
Inpp5dUnknown, score: 0.2
Inppl1Unknown, score: 0.4
InvsUnknown, score: 0.26
Irf4Unknown, score: 0.11
Irg1Unknown, score: 0.14
Irs1Unknown, score: 0.07
Irs3Unknown, score: 0.67
Irx1Unknown, score: 0.89
ItchUnknown, score: 0.21
Itga2Unknown, score: 0.51
Itga2bUnknown, score: 0.36
Itga4Unknown, score: 0.22
Itga6Unknown, score: 0.16
Itga7Unknown, score: 0.15
ItgaeUnknown, score: 0.4
ItgalUnknown, score: 0.15
ItgavUnknown, score: 0.08
Itgb1Unknown, score: 0.1
Itgb1bp1Unknown, score: 0.19
Itgb2Unknown, score: 0.3
Itgb3Unknown, score: 0.28
Eif6Unknown, score: 0.17
Itgb6Unknown, score: 0.23
Itgb7Unknown, score: 0.02
Itih3Unknown, score: 0.11
Itih4Unknown, score: 0.1
Itm2bUnknown, score: 0.1
Cuzd1Unknown, score: 0.21
Itpr2Unknown, score: 0.31
Itpr3Unknown, score: 0.27
Itsn1Unknown, score: 0.3
Jag1Unknown, score: 0.53
Jak2Unknown, score: 0.51
Jak3Unknown, score: 0.12
Jarid2Unknown, score: 0.05
Ush1gUnknown, score: 0.27
AjubaUnknown, score: 0.09
JunUnknown, score: 0.58
JunbUnknown, score: 0.26
JundUnknown, score: 0.06
Kcna2Unknown, score: 0.26
Kcna7Unknown, score: 0.14
Kcnab1Unknown, score: 0.3
Kcnab2Unknown, score: 0.05
Kcnab3Unknown, score: 0.61
Kcnb1Unknown, score: 0.19
Kcnc1Unknown, score: 0.68
Kcne1Unknown, score: 0.26
Kcnh1Unknown, score: 0.53
Kcnh2Unknown, score: 0.3
Kcnj10Unknown, score: 0.25
Kcnj16Unknown, score: 0.35
Kcnj3Unknown, score: 0.35
Kcnj4Unknown, score: 0.18
Kcnk2Unknown, score: 0.44
Kcnk3Unknown, score: 0.05
Kcnk5Unknown, score: 0.02
Kcnmb1Unknown, score: 0.34
Kcnn4Unknown, score: 0.76
Kcnq1Unknown, score: 0.06
Kcns1Unknown, score: 0.27
MdficUnknown, score: 0.35
KhkUnknown, score: 0.1
Kif11Unknown, score: 0.35
Kif13aUnknown, score: 0.22
Kif16bUnknown, score: 0.08
Kif17Unknown, score: 0.37
Kif1aUnknown, score: 0.13
Kif1bUnknown, score: 0.42
Kif21aUnknown, score: 0.21
Kif3aUnknown, score: 0.15
Kif3bUnknown, score: 0.09
Kif5aUnknown, score: 0.08
Kif5cUnknown, score: 0.07
Kif7Unknown, score: 0.11
Kifap3Unknown, score: 0.19
Kifc5bUnknown, score: 0.02
Kifc2Unknown, score: 0.29
KitUnknown, score: 0.4
Fabp5Unknown, score: 0.15
Klc1Unknown, score: 0.04
Klc2Unknown, score: 0.13
Klf12Unknown, score: 0.59
Klf3Unknown, score: 0.11
Klf9Unknown, score: 0.26
Serpina3cUnknown, score: 0.12
Klra1Unknown, score: 0.47
Klra2Unknown, score: 0.14
Klra5Unknown, score: 0.43
Klra8Unknown, score: 0.36
Klrc1Unknown, score: 0.15
Kpna1Unknown, score: 0.26
Kpna4Unknown, score: 0.28
KrasUnknown, score: 0.37
Hivep3Unknown, score: 0.13
MafbUnknown, score: 0.38
Krt14Unknown, score: 0.22
Krt18Unknown, score: 0.46
Krt19Unknown, score: 0.04
Krt8Unknown, score: 0.36
L1camUnknown, score: 0.3
Lad1Unknown, score: 0.38
Aff3Unknown, score: 0.07
Stmn1Unknown, score: 0.06
Lama1Unknown, score: 0.02
Lama2Unknown, score: 0.38
Lama4Unknown, score: 0.56
Lamb1Unknown, score: 0.23
Lamb2Unknown, score: 0.5
Lamb3Unknown, score: 0.3
Lamc2Unknown, score: 0.28
Lamp2Unknown, score: 0.39
RpsaUnknown, score: 0.14
Lasp1Unknown, score: 0.28
Lats1Unknown, score: 0.22
Arhgef2Unknown, score: 0.3
Arhgef1Unknown, score: 0.25
LcatUnknown, score: 0.26
LckUnknown, score: 0.18
Lcp2Unknown, score: 0.06
Ldb2Unknown, score: 0.33
LdhbUnknown, score: 0.27
Cog1Unknown, score: 0.45
Lef1Unknown, score: 0.07
LeprUnknown, score: 0.37
Lgals1Unknown, score: 0.22
Lgals4Unknown, score: 0.09
Lgals9Unknown, score: 0.39
Eif2dUnknown, score: 0.06
Lhx6Unknown, score: 0.06
LifUnknown, score: 0.08
Lig1Unknown, score: 0.04
Lig3Unknown, score: 0.36
Limk1Unknown, score: 0.02
Limk2Unknown, score: 0.09
LipaUnknown, score: 0.2
LipgUnknown, score: 0.42
Llgl1Unknown, score: 0.23
Rps2Unknown, score: 0.2
GzmmUnknown, score: 1
Lmnb1Unknown, score: 0.19
Psmb8Unknown, score: 0.32
Lnx1Unknown, score: 0.32
LorUnknown, score: 0.24
LoxUnknown, score: 0.21
Xcl1Unknown, score: 0.05
Zbtb7aUnknown, score: 0.04
LrmpUnknown, score: 0.3
Lrp1Unknown, score: 0.1
Lrp5Unknown, score: 0.03
Lrp6Unknown, score: 0.24
Lrpap1Unknown, score: 0.07
Lrrn1Unknown, score: 0.4
Lrrn3Unknown, score: 0.16
LssUnknown, score: 0.22
LtaUnknown, score: 0.36
Lta4hUnknown, score: 0.17
LtbUnknown, score: 0.3
Ltb4r1Unknown, score: 0.31
Ltbp2Unknown, score: 0.43
Ltbp3Unknown, score: 0.75
LtbrUnknown, score: 0.38
Ltc4sUnknown, score: 0.32
LtfUnknown, score: 0.13
LtkUnknown, score: 0.04
Klrb1aUnknown, score: 0.03
Klrb1cUnknown, score: 0.13
Cd93Unknown, score: 0.16
Ly6dUnknown, score: 0.08
Ly6eUnknown, score: 0.16
Il1rl1Unknown, score: 0.62
LyarUnknown, score: 0.27
LynUnknown, score: 0.17
Lyz1Unknown, score: 0.38
Mab21l1Unknown, score: 0.08
MarcksUnknown, score: 0.23
Mad1l1Unknown, score: 0.04
Mxd4Unknown, score: 0.08
Smad2Unknown, score: 0.24
Smad3Unknown, score: 0.06
Smad5Unknown, score: 0.48
Smad6Unknown, score: 0.52
MafUnknown, score: 0.13
MaffUnknown, score: 0.36
MafgUnknown, score: 0.24
MagUnknown, score: 0.05
MagohUnknown, score: 0.43
Ccndbp1Unknown, score: 0.2
MalUnknown, score: 0.29
Man2a1Unknown, score: 0.06
Man2b1Unknown, score: 0.35
MaoaUnknown, score: 0.38
Mapkapk2Unknown, score: 0.29
Mapkapk5Unknown, score: 0.36
MarcoUnknown, score: 0.37
Nprl3Unknown, score: 0.18
Mark3Unknown, score: 0.12
Ascl1Unknown, score: 0.34
Masp1Unknown, score: 0.11
Fxyd3Unknown, score: 0.06
Matr3Unknown, score: 0.04
MaxUnknown, score: 0.01
Mbd2Unknown, score: 0.25
Mbd3Unknown, score: 0.06
MbpUnknown, score: 0.03
Mc2rUnknown, score: 0.41
Mcf2lUnknown, score: 0.09
Mcm5Unknown, score: 0.17
Cd46Unknown, score: 0.29
Cma1Unknown, score: 0.04
Tpsb2Unknown, score: 0.41
Mdm2Unknown, score: 0.1
Mdm4Unknown, score: 0.05
Rdh11Unknown, score: 0.17
Slc3a2Unknown, score: 0.05
Mef2aUnknown, score: 0.2
Mef2cUnknown, score: 0.13
Fyco1Unknown, score: 0.21
Men1Unknown, score: 0.04
Meox2Unknown, score: 0.18
Mettl1Unknown, score: 0.17
Foxc1Unknown, score: 0.22
Foxd2Unknown, score: 0.3
Mfge8Unknown, score: 0.21
MfngUnknown, score: 0.3
Sypl2Unknown, score: 0.44
Mgat1Unknown, score: 0.12
Clec10aUnknown, score: 0.32
MgmtUnknown, score: 0.27
Mid1Unknown, score: 0.44
Cxcl9Unknown, score: 0.13
Minpp1Unknown, score: 0.14
Mknk1Unknown, score: 0.33
Mknk2Unknown, score: 0.12
Mlh1Unknown, score: 0.03
Mllt10Unknown, score: 0.02
Mllt4Unknown, score: 0.12
MmeUnknown, score: 0.14
Mmp12Unknown, score: 0.41
Mmp13Unknown, score: 0.14
Mmp14Unknown, score: 0.35
Mmp16Unknown, score: 0.25
Mmp3Unknown, score: 0.21
Mmp8Unknown, score: 0.28
Mnat1Unknown, score: 0.17
Mns1Unknown, score: 0.38
MntUnknown, score: 0.21
Me1Unknown, score: 0.22
Mdh2Unknown, score: 0.09
Mdh1Unknown, score: 0.02
Gbp4Unknown, score: 0.56
Mpeg1Unknown, score: 0.17
MpzUnknown, score: 0.05
Mrc1Unknown, score: 0.3
Mrc2Unknown, score: 0.05
Meis2Unknown, score: 0.22
Mrvi1Unknown, score: 0.1
MscUnknown, score: 0.09
Msi1Unknown, score: 0.5
MsnUnknown, score: 0.18
MstnUnknown, score: 0.41
Msx1Unknown, score: 0.03
mt-CytbUnknown, score: 0.16
Grpel1Unknown, score: 0.09
mt-Nd6Unknown, score: 0.29
Mt1Unknown, score: 0.36
Polr2kUnknown, score: 0.4
Mt3Unknown, score: 0.14
Map2Unknown, score: 0.16
Mtcp1Unknown, score: 0.43
Mtf2Unknown, score: 0.3
Nudt1Unknown, score: 0.27
Mthfd2Unknown, score: 0.17
Mtm1Unknown, score: 0.1
Laptm4aUnknown, score: 0.33
MttpUnknown, score: 0.28
Fam89bUnknown, score: 0.09
Mtx1Unknown, score: 0.15
Bloc1s5Unknown, score: 0.01
Muc1Unknown, score: 0.14
MutUnknown, score: 0.43
MvkUnknown, score: 0.05
Mx2Unknown, score: 0.08
Mxi1Unknown, score: 0.06
Mybl1Unknown, score: 0.23
Mybl2Unknown, score: 0.11
MycUnknown, score: 0.5
Gadd45bUnknown, score: 0.23
Myh1Unknown, score: 0.18
Myh2Unknown, score: 0.08
Myl3Unknown, score: 0.1
Myl1Unknown, score: 0.15
Myo10Unknown, score: 0.13
Myo1bUnknown, score: 0.18
Myo7aUnknown, score: 0.1
Myo9bUnknown, score: 0.47
Nab1Unknown, score: 0.03
Nab2Unknown, score: 0.11
NacaUnknown, score: 0.04
NagaUnknown, score: 0.1
Nat2Unknown, score: 0.43
Ncf2Unknown, score: 0.24
NclUnknown, score: 0.47
Ncoa1Unknown, score: 0.15
Ncoa3Unknown, score: 0.16
NdnUnknown, score: 0.2
Ndrg1Unknown, score: 0.51
Ndufa2Unknown, score: 0.16
Ndufa4Unknown, score: 0.1
Ndufs4Unknown, score: 0.21
Ndufv1Unknown, score: 0.25
NebUnknown, score: 0.05
Nedd8Unknown, score: 0.16
Nek2Unknown, score: 0.12
NesUnknown, score: 0.08
Neu1Unknown, score: 0.58
Nf2Unknown, score: 0.17
Nfatc2Unknown, score: 0.18
Nfatc2ipUnknown, score: 0.05
Nfatc3Unknown, score: 0.12
Nfe2Unknown, score: 0.14
NficUnknown, score: 0.26
NfixUnknown, score: 0.21
Nfkb1Unknown, score: 0.14
Nfkb2Unknown, score: 0.08
NfkbiaUnknown, score: 0.3
NfkbibUnknown, score: 0.03
NfkbieUnknown, score: 0.5
Nfkbil1Unknown, score: 0.23
NeflUnknown, score: 0.05
NefmUnknown, score: 0.17
Nfs1Unknown, score: 0.13
NfybUnknown, score: 0.19
NgfUnknown, score: 0.09
NgfrUnknown, score: 0.22
NinUnknown, score: 0.56
NktrUnknown, score: 0.06
Nkx2-3Unknown, score: 0.78
NlkUnknown, score: 0.05
Mrpl40Unknown, score: 0.06
NmbrUnknown, score: 0.01
Nme1Unknown, score: 0.19
Nme2Unknown, score: 0.17
Nqo1Unknown, score: 0.29
Nqo2Unknown, score: 0.11
Nmt2Unknown, score: 0.03
Rrp1Unknown, score: 0.27
NogUnknown, score: 0.43
Nos2Unknown, score: 0.12
Nos3Unknown, score: 0.38
Notch1Unknown, score: 0.35
Ints6Unknown, score: 0.4
Notch4Unknown, score: 0.49
Nup50Unknown, score: 0.25
Npas2Unknown, score: 0.25
Npc1Unknown, score: 0.4
Npm1Unknown, score: 0.23
NppbUnknown, score: 0.15
Npr3Unknown, score: 0.02
Ctnnd2Unknown, score: 0.39
Nptx1Unknown, score: 0.09
Nr1i2Unknown, score: 0.09
Slc11a1Unknown, score: 0.61
Slc11a2Unknown, score: 0.25
NrasUnknown, score: 0.18
Nrp2Unknown, score: 0.6
Nrxn1Unknown, score: 0.08
Nrxn3Unknown, score: 0.07
NsdhlUnknown, score: 0.21
NsfUnknown, score: 0.08
Nsg1Unknown, score: 0.26
Ntf3Unknown, score: 0.12
Nthl1Unknown, score: 0.42
Ntn1Unknown, score: 0.45
Ntn3Unknown, score: 0.1
Ntrk1Unknown, score: 0.1
Ntrk2Unknown, score: 0.13
Ntrk3Unknown, score: 0.16
Ntsr2Unknown, score: 0.09
Dusp8Unknown, score: 0.02
Nucb1Unknown, score: 0.1
NumbUnknown, score: 0.39
NumblUnknown, score: 0.31
Nup62Unknown, score: 0.17
OatUnknown, score: 0.61
Oaz1Unknown, score: 0.04
Odf2Unknown, score: 0.12
OgdhUnknown, score: 0.38
OgnUnknown, score: 0.24
Fxyd5Unknown, score: 0.12
Oit3Unknown, score: 0.13
Olfr56Unknown, score: 0.04
OmgUnknown, score: 0.05
OmpUnknown, score: 0.27
Tnfrsf11bUnknown, score: 0.19
Oprl1Unknown, score: 0.11
Orc2Unknown, score: 0.25
Orm1Unknown, score: 0.19
Orm2Unknown, score: 0.34
Slc25a15Unknown, score: 0.05
Sqstm1Unknown, score: 0.16
OtcUnknown, score: 0.33
Cldn11Unknown, score: 0.03
Oca2Unknown, score: 0.03
Mybbp1aUnknown, score: 0.25
P2rx1Unknown, score: 0.34
P2rx4Unknown, score: 0.03
P2rx7Unknown, score: 0.43
P2ry1Unknown, score: 0.24
Pabpc1Unknown, score: 0.03
Pafah1b2Unknown, score: 0.41
PahUnknown, score: 0.04
Pak1Unknown, score: 0.19
Pak3Unknown, score: 0.42
PalmUnknown, score: 0.58
Pax6Unknown, score: 0.07
Pbx1Unknown, score: 0.03
Kat2bUnknown, score: 0.14
Pcdh10Unknown, score: 0.33
Pcdh8Unknown, score: 0.02
Pcm1Unknown, score: 0.17
PcnaUnknown, score: 0.58
Pcp4Unknown, score: 0.37
Pcsk1Unknown, score: 0.13
Pcsk2Unknown, score: 0.61
FurinUnknown, score: 0.1
Pcsk4Unknown, score: 0.14
Pcsk5Unknown, score: 0.09
Pcsk6Unknown, score: 0.76
Pcsk7Unknown, score: 0.07
Cdk18Unknown, score: 0.08
PctpUnknown, score: 0.44
PcxUnknown, score: 0.24
Pdcd1Unknown, score: 0.02
Pdcd2Unknown, score: 0.05
Pdcd4Unknown, score: 0.05
Pdcd6Unknown, score: 0.12
Pdcd6ipUnknown, score: 0.11
Pdcd11Unknown, score: 0.08
Pde1aUnknown, score: 0.01
Pde1cUnknown, score: 0.08
Pde3bUnknown, score: 0.52
Pde4aUnknown, score: 0.39
Pde6gUnknown, score: 0.35
PdgfaUnknown, score: 0.12
PdgfbUnknown, score: 0.09
Pdha1Unknown, score: 0.34
Padi4Unknown, score: 0.16
Enpp1Unknown, score: 0.07
Enpp2Unknown, score: 0.15
Pecam1Unknown, score: 0.51
Peg3Unknown, score: 0.24
PenkUnknown, score: 0.13
PepdUnknown, score: 0.35
Per1Unknown, score: 0.08
Pex16Unknown, score: 0.31
Pex7Unknown, score: 0.41
Pfkfb2Unknown, score: 0.55
PfklUnknown, score: 0.22
Cdk14Unknown, score: 0.45
Pgam1Unknown, score: 0.11
PgfUnknown, score: 0.09
Pgk1Unknown, score: 0.02
Abcb1bUnknown, score: 0.08
Abcb4Unknown, score: 0.11
Abcb1aUnknown, score: 0.18
PhbUnknown, score: 0.18
PhexUnknown, score: 0.11
Phf2Unknown, score: 0.61
Phka1Unknown, score: 0.8
Phtf1Unknown, score: 0.34
Pik3caUnknown, score: 0.27
Pik3cdUnknown, score: 0.2
Pik3r1Unknown, score: 0.36
Pik3r3Unknown, score: 0.2
PikfyveUnknown, score: 0.36
Pim2Unknown, score: 0.06
Pip4k2aUnknown, score: 0.15
Pip5k1bUnknown, score: 0.71
Pip5k1aUnknown, score: 0.2
18722Unknown, score: 0.2
Gm14548Unknown, score: 0.55
Pira2Unknown, score: 0.32
Gm15448Unknown, score: 0.2
Gm14548Unknown, score: 0.2
Gm14548Unknown, score: 0.2
PitpnaUnknown, score: 0.7
Pitx1Unknown, score: 0.45
Pitx2Unknown, score: 0.34
Pitx3Unknown, score: 0.15
Pja1Unknown, score: 0.06
PrkcbUnknown, score: 0.09
PrkcgUnknown, score: 0.1
PrkcqUnknown, score: 0.37
PrkczUnknown, score: 0.37
Pkd2Unknown, score: 0.53
PkibUnknown, score: 0.07
Pla2r1Unknown, score: 0.47
Pla2g4aUnknown, score: 0.34
Serpine1Unknown, score: 0.05
PlauUnknown, score: 0.35
PlaurUnknown, score: 0.12
Plcb1Unknown, score: 0.23
Plcb2Unknown, score: 0.39
Plcb4Unknown, score: 0.2
Plcd4Unknown, score: 0.03
Pld1Unknown, score: 0.14
Pld2Unknown, score: 0.33
Pld3Unknown, score: 0.54
Serpinf2Unknown, score: 0.04
PlnUnknown, score: 0.02
PltpUnknown, score: 0.11
Plxna2Unknown, score: 0.23
Plxna3Unknown, score: 0.08
PmlUnknown, score: 0.04
Pmp2Unknown, score: 0.24
Pmp22Unknown, score: 0.21
PnnUnknown, score: 0.23
PnpUnknown, score: 0.09
Sept5Unknown, score: 0.21
PolbUnknown, score: 0.21
Pole2Unknown, score: 0.15
PolgUnknown, score: 0.24
PomcUnknown, score: 0.07
Pou2af1Unknown, score: 0.1
Pou2f2Unknown, score: 0.02
Pou2f3Unknown, score: 0.28
Pou4f1Unknown, score: 0.32
EndouUnknown, score: 0.25
Ppap2aUnknown, score: 0.39
PparaUnknown, score: 0.27
Med1Unknown, score: 0.6
Ppargc1aUnknown, score: 0.42
Scand1Unknown, score: 0.04
Ppfibp2Unknown, score: 0.09
PpicUnknown, score: 0.28
Lgals3bpUnknown, score: 0.08
Ppm1aUnknown, score: 0.11
Ppm1bUnknown, score: 0.17
PpoxUnknown, score: 0.08
Ppp1caUnknown, score: 0.07
Ppp1cbUnknown, score: 0.05
Ppp1ccUnknown, score: 0.74
Ppp3r1Unknown, score: 0.16
Ppp5cUnknown, score: 0.16
Prkab1Unknown, score: 0.1
Prkag1Unknown, score: 0.21
Prkar1aUnknown, score: 0.09
Prkar1bUnknown, score: 0.11
Prkar2aUnknown, score: 0.05
PrkdcUnknown, score: 0.1
Mapk11Unknown, score: 0.3
PrlrUnknown, score: 0.1
ProcUnknown, score: 0.07
ProcrUnknown, score: 0.09
ProdhUnknown, score: 0.19
Prom1Unknown, score: 0.05
Pros1Unknown, score: 0.04
Prox1Unknown, score: 0.07
Prps1Unknown, score: 0.3
Prss12Unknown, score: 0.12
Prtn3Unknown, score: 0.11
PrxUnknown, score: 0.16
NpeppsUnknown, score: 0.04
PsapUnknown, score: 0.03
Cyth1Unknown, score: 0.2
Psen1Unknown, score: 0.14
Psmb10Unknown, score: 0.27
Psmb4Unknown, score: 0.43
Psmb5Unknown, score: 0.42
Psmc3Unknown, score: 0.33
Psmc3ipUnknown, score: 0.09
Psmc5Unknown, score: 0.14
Psmd4Unknown, score: 0.22
Psme1Unknown, score: 0.09
Psme2Unknown, score: 0.06
Psme3Unknown, score: 0.2
PipoxUnknown, score: 0.11
PspnUnknown, score: 0.43
Rhox6Unknown, score: 0.11
PtafrUnknown, score: 0.02
Ptbp1Unknown, score: 0.33
Ptch1Unknown, score: 0.23
PtgdsUnknown, score: 0.39
Ptger1Unknown, score: 0.22
Ptger2Unknown, score: 0.04
Ptger3Unknown, score: 0.35
Ptger4Unknown, score: 0.28
PtgfrUnknown, score: 0.17
PtgirUnknown, score: 0.3
PtgisUnknown, score: 0.07
Ptgs1Unknown, score: 0.05
Ptgs2Unknown, score: 0.34
PthlhUnknown, score: 0.32
Pth1rUnknown, score: 0.1
Ptp4a2Unknown, score: 0.38
Ptp4a3Unknown, score: 0.32
Ptpn1Unknown, score: 0.23
Ptpn12Unknown, score: 0.08
Ptpn13Unknown, score: 0.07
Ptpn14Unknown, score: 0.3
Dusp1Unknown, score: 0.06
Ptpn22Unknown, score: 0.03
PtpraUnknown, score: 0.02
PtprcUnknown, score: 0.16
PtprcapUnknown, score: 0.22
PtprkUnknown, score: 0.56
Ptprn2Unknown, score: 0.27
PtproUnknown, score: 0.12
PtprrUnknown, score: 0.38
Ptprz1Unknown, score: 0.12
PtsUnknown, score: 0.43
PvalbUnknown, score: 0.3
Abcd3Unknown, score: 0.06
Pxmp2Unknown, score: 0.12
Pex2Unknown, score: 0.09
PxnUnknown, score: 0.11
Pex5Unknown, score: 0.03
Rab10Unknown, score: 0.01
Rab23Unknown, score: 0.09
Rab33bUnknown, score: 0.23
Rab3dUnknown, score: 0.26
Rab4aUnknown, score: 0.31
Rab4bUnknown, score: 0.03
Rab5bUnknown, score: 0.13
Rac1Unknown, score: 0.02
Rac2Unknown, score: 0.03
Rad51ap1Unknown, score: 0.25
Rad51dUnknown, score: 0.14
Rad54lUnknown, score: 0.26
Rab34Unknown, score: 0.35
Rai1Unknown, score: 0.25
Aldh1a2Unknown, score: 0.22
RalyUnknown, score: 0.22
Ranbp2Unknown, score: 0.12
Rasgrp2Unknown, score: 0.38
RapsnUnknown, score: 0.68
RaraUnknown, score: 0.02
Rasa3Unknown, score: 0.17
Rasal1Unknown, score: 0.62
Rasd1Unknown, score: 0.02
Rasgrf1Unknown, score: 0.11
RbmxUnknown, score: 0.37
Rbp1Unknown, score: 0.21
RbpjUnknown, score: 0.06
RbpjlUnknown, score: 0.34
Pitpnm2Unknown, score: 0.17
RelUnknown, score: 0.33
RelaUnknown, score: 0.26
RelnUnknown, score: 0.08
Rem1Unknown, score: 0.01
RenbpUnknown, score: 0.04
Dpf2Unknown, score: 0.24
RestUnknown, score: 0.02
Bex1Unknown, score: 0.38
Rfc2Unknown, score: 0.45
Trim27Unknown, score: 0.44
Rfx1Unknown, score: 0.84
Rfx2Unknown, score: 0.08
Rfx3Unknown, score: 0.06
Slc50a1Unknown, score: 0.19
RalgdsUnknown, score: 0.03
Rgl1Unknown, score: 0.18
Rgl2Unknown, score: 0.02
RgnUnknown, score: 0.16
Rgs16Unknown, score: 0.47
Rgs2Unknown, score: 0.13
Rgs5Unknown, score: 0.26
Rgs9Unknown, score: 0.14
Rnase1Unknown, score: 0.06
Ralbp1Unknown, score: 0.08
Ripk1Unknown, score: 0.11
Uri1Unknown, score: 0.06
Trim10Unknown, score: 0.11
Rnps1Unknown, score: 0.12
Rock1Unknown, score: 0.05
Slc22a8Unknown, score: 0.21
Rom1Unknown, score: 0.34
RorcUnknown, score: 0.59
RpgrUnknown, score: 0.11
RpiaUnknown, score: 0.12
Rpl22Unknown, score: 0.28
Rpl26Unknown, score: 0.32
Rpl27Unknown, score: 0.4
Rpl28Unknown, score: 0.07
Rpl29Unknown, score: 0.08
Rpl32Unknown, score: 0.1
Rpl37aUnknown, score: 0.36
Rpl36aUnknown, score: 0.03
Rpl7Unknown, score: 0.1
Rpl9Unknown, score: 0.26
Rpn2Unknown, score: 0.03
Polr1cUnknown, score: 0.27
Polr1dUnknown, score: 0.17
Polr1aUnknown, score: 0.05
Polr2cUnknown, score: 0.08
Sub1Unknown, score: 0.64
Rps12Unknown, score: 0.06
Rps15Unknown, score: 0.1
Rps18Unknown, score: 0.13
Rps24Unknown, score: 0.27
Rps29Unknown, score: 0.13
Rps4xUnknown, score: 0.25
Rps6Unknown, score: 0.39
Rps6ka1Unknown, score: 0.15
RrasUnknown, score: 0.05
Rrm1Unknown, score: 0.17
Rrm2Unknown, score: 0.05
Rsu1Unknown, score: 0.41
RtknUnknown, score: 0.07
Rtn3Unknown, score: 0.49
Ruvbl2Unknown, score: 0.8
RxraUnknown, score: 0.73
RxrbUnknown, score: 0.04
Nr1h4Unknown, score: 0.13
Ryr1Unknown, score: 0.5
S100a1Unknown, score: 0.09
S100a10Unknown, score: 0.05
S100a11Unknown, score: 0.19
S100a13Unknown, score: 0.04
S100a3Unknown, score: 0.33
S100a4Unknown, score: 0.3
S100a6Unknown, score: 0.27
S100a8Unknown, score: 0.28
S100bUnknown, score: 0.26
Saa1Unknown, score: 0.08
Saa2Unknown, score: 0.36
Saa4Unknown, score: 0.13
Acsm3Unknown, score: 0.13
Sap18Unknown, score: 0.19
SarsUnknown, score: 0.01
Satb1Unknown, score: 0.48
Atxn1Unknown, score: 0.2
Atxn2Unknown, score: 0.54
Scd1Unknown, score: 0.54
Clec11aUnknown, score: 0.64
ScinUnknown, score: 0.1
Stmn3Unknown, score: 0.71
Scn3aUnknown, score: 0.5
Scn7aUnknown, score: 0.12
Scn8aUnknown, score: 0.02
Scnn1aUnknown, score: 0.18
Zc3h7bUnknown, score: 0.12
SctUnknown, score: 0.16
Msr1Unknown, score: 0.04
Ccl11Unknown, score: 0.24
Ccl12Unknown, score: 0.13
Ccl17Unknown, score: 0.43
Ccl2Unknown, score: 0.17
Ccl25Unknown, score: 0.2
Ccl27aUnknown, score: 0.1
Ccl3Unknown, score: 0.15
Ccl5Unknown, score: 0.02
Ccl6Unknown, score: 0.38
Ccl7Unknown, score: 0.09
Ccl8Unknown, score: 0.11
Ccl9Unknown, score: 0.21
Cxcl2Unknown, score: 0.28
Cxcl5Unknown, score: 0.23
Cx3cl1Unknown, score: 0.8
Cxcl12Unknown, score: 0.31
Sdf2Unknown, score: 0.07
Sdf4Unknown, score: 0.13
Sfrp2Unknown, score: 0.14
Frrs1Unknown, score: 0.09
Sec22bUnknown, score: 0.08
Sel1lUnknown, score: 0.14
Glg1Unknown, score: 0.4
Selenbp1Unknown, score: 0.21
SellUnknown, score: 0.45
SelpUnknown, score: 0.16
Sema3aUnknown, score: 0.22
Sema3eUnknown, score: 0.52
Sema3fUnknown, score: 0.12
Sema4aUnknown, score: 0.2
Sema4bUnknown, score: 0.25
Sema6aUnknown, score: 0.28
Sema6bUnknown, score: 0.29
Sema6cUnknown, score: 0.08
Sepp1Unknown, score: 0.39
Sez6Unknown, score: 0.09
Foxp3Unknown, score: 0.13
Spi1Unknown, score: 0.03
Sfrp1Unknown, score: 0.06
FrzbUnknown, score: 0.07
Sfrp4Unknown, score: 0.06
Srsf2Unknown, score: 0.05
SftpbUnknown, score: 0.17
SgceUnknown, score: 0.24
Sgpl1Unknown, score: 0.21
Sh2b1Unknown, score: 0.2
Sh2d1aUnknown, score: 0.2
Sh3bp1Unknown, score: 0.21
Itsn2Unknown, score: 0.21
Sh3gl2Unknown, score: 0.14
Ostf1Unknown, score: 0.37
Sorbs3Unknown, score: 0.11
Sorbs1Unknown, score: 0.08
Shc1Unknown, score: 0.15
Shc3Unknown, score: 0.02
Shcbp1Unknown, score: 0.19
ShdUnknown, score: 0.32
Shox2Unknown, score: 0.18
Cyfip1Unknown, score: 0.03
PmelUnknown, score: 0.15
Siah1aUnknown, score: 0.07
Siah1bUnknown, score: 0.57
St6gal1Unknown, score: 0.29
St3gal3Unknown, score: 0.12
St3gal1Unknown, score: 0.49
St3gal2Unknown, score: 0.11
St6galnac2Unknown, score: 0.12
St6galnac3Unknown, score: 0.18
St8sia4Unknown, score: 0.13
St3gal5Unknown, score: 0.27
Ptk6Unknown, score: 0.02
Sin3aUnknown, score: 0.42
Sin3bUnknown, score: 0.05
Sipa1Unknown, score: 0.3
Six1Unknown, score: 0.43
Six2Unknown, score: 0.04
Six4Unknown, score: 0.12
Vps4bUnknown, score: 0.05
ClpbUnknown, score: 0.3
SkiUnknown, score: 0.06
Slc12a2Unknown, score: 0.28
Slc12a4Unknown, score: 0.29
Slc12a7Unknown, score: 0.13
Slc16a1Unknown, score: 0.19
Slc16a2Unknown, score: 0.13
Slc16a7Unknown, score: 0.48
Slc1a1Unknown, score: 0.11
Slc1a3Unknown, score: 0.04
Slc1a5Unknown, score: 0.28
Slc20a1Unknown, score: 0.02
Slc22a5Unknown, score: 0.18
Slc23a1Unknown, score: 0.02
Slc25a17Unknown, score: 0.62
Slc2a2Unknown, score: 0.13
Slc2a3Unknown, score: 0.4
Slc2a4Unknown, score: 0.05
Slc31a1Unknown, score: 0.1
Slc4a1apUnknown, score: 0.45
Slc4a2Unknown, score: 0.43
Slc4a3Unknown, score: 0.27
Slc5a1Unknown, score: 0.02
Slc6a2Unknown, score: 0.3
Slc7a5Unknown, score: 0.27
Slc7a7Unknown, score: 0.21
Slc8a1Unknown, score: 0.59
Slfn4Unknown, score: 0.14
Slit2Unknown, score: 0.09
Slit3Unknown, score: 0.35
Smarca4Unknown, score: 0.03
Smarcc1Unknown, score: 0.51
Ighmbp2Unknown, score: 0.16
Smn1Unknown, score: 0.32
Smpd2Unknown, score: 0.48
Sstr2Unknown, score: 0.47
Sstr3Unknown, score: 0.06
Sstr4Unknown, score: 0.55
Sumo3Unknown, score: 0.03
Siglec1Unknown, score: 0.35
Snai1Unknown, score: 0.1
Snap91Unknown, score: 0.11
SncaUnknown, score: 0.34
SnrkUnknown, score: 0.11
Eftud2Unknown, score: 0.25
SnrpcUnknown, score: 0.08
Snrnp70Unknown, score: 0.6
SnrpeUnknown, score: 0.56
SnrpnUnknown, score: 0.3
Sntb1Unknown, score: 0.02
Soat1Unknown, score: 0.04
Sod1Unknown, score: 0.25
Sod2Unknown, score: 0.23
SonUnknown, score: 0.13
Sox12Unknown, score: 0.4
Sox15Unknown, score: 0.69
Sox17Unknown, score: 0.38
Sox18Unknown, score: 0.02
Sox5Unknown, score: 0.08
Sox6Unknown, score: 0.14
Sox7Unknown, score: 0.01
Sox8Unknown, score: 0.04
SparcUnknown, score: 0.13
Serpina1eUnknown, score: 0.11
Serpina3kUnknown, score: 0.43
Serpina3mUnknown, score: 0.16
Serpinb6aUnknown, score: 0.33
Spin1Unknown, score: 0.37
Spink4Unknown, score: 0.42
Spta1Unknown, score: 0.05
Sptan1Unknown, score: 0.43
SptbUnknown, score: 0.01
Sptbn1Unknown, score: 0.03
Sptbn2Unknown, score: 0.05
Spock1Unknown, score: 0.18
SpopUnknown, score: 0.28
Spp1Unknown, score: 0.43
SprUnknown, score: 0.41
Sprr1aUnknown, score: 0.33
Sephs2Unknown, score: 0.09
Sptlc2Unknown, score: 0.06
SqleUnknown, score: 0.03
Scarb1Unknown, score: 0.19
SrcUnknown, score: 0.66
Srebf1Unknown, score: 0.37
Srebf2Unknown, score: 0.34
SrfUnknown, score: 0.16
SrmUnknown, score: 0.22
SrmsUnknown, score: 0.14
Srpk2Unknown, score: 0.16
SsbUnknown, score: 0.12
Nhp2l1Unknown, score: 0.21
Ssrp1Unknown, score: 0.09
StamUnknown, score: 0.22
Stat2Unknown, score: 0.5
Stat3Unknown, score: 0.11
Stat4Unknown, score: 0.16
Stat5aUnknown, score: 0.42
Stat5bUnknown, score: 0.1
Stau1Unknown, score: 0.03
Stc2Unknown, score: 0.22
Stk10Unknown, score: 0.73
AurkcUnknown, score: 0.76
SlkUnknown, score: 0.15
Stra13Unknown, score: 0.28
Bhlhe40Unknown, score: 0.38
StrapUnknown, score: 0.05
Stx4aUnknown, score: 0.13
Stxbp1Unknown, score: 0.07
Stxbp3aUnknown, score: 0.07
Suclg2Unknown, score: 0.21
Eif1Unknown, score: 0.05
Supt4aUnknown, score: 0.23
Supt5Unknown, score: 0.23
Supt6Unknown, score: 0.14
Surf2Unknown, score: 0.04
Sycp3Unknown, score: 0.23
SykUnknown, score: 0.11
Syn2Unknown, score: 0.5
Sdc1Unknown, score: 0.05
Sdc4Unknown, score: 0.1
Syngr1Unknown, score: 0.3
Syngr2Unknown, score: 0.13
Synj2Unknown, score: 0.19
SypUnknown, score: 0.49
Syt2Unknown, score: 0.38
Syt3Unknown, score: 0.38
Tacc3Unknown, score: 0.07
Taf1bUnknown, score: 0.41
Taf1cUnknown, score: 0.14
Taf6Unknown, score: 0.4
TaglnUnknown, score: 0.28
Tagln2Unknown, score: 0.04
Tal1Unknown, score: 0.09
Tal2Unknown, score: 0.08
Tap1Unknown, score: 0.21
Tap2Unknown, score: 0.13
TapbpUnknown, score: 0.35
Tarbp2Unknown, score: 0.26
Slc6a6Unknown, score: 0.39
TbcaUnknown, score: 0.09
TbpUnknown, score: 0.07
Tbrg1Unknown, score: 0.33
Tbx15Unknown, score: 0.45
Tbx2Unknown, score: 0.13
Tbx3Unknown, score: 0.49
Tbx5Unknown, score: 0.33
Tbx6Unknown, score: 0.34
Tbxas1Unknown, score: 0.64
Tcf12Unknown, score: 0.27
Zfp354aUnknown, score: 0.25
Tcf20Unknown, score: 0.46
Tcf4Unknown, score: 0.32
Tfcp2Unknown, score: 0.13
Tcf3Unknown, score: 0.61
TfebUnknown, score: 0.26
TfecUnknown, score: 0.33
UbtfUnknown, score: 0.26
Tcp10bUnknown, score: 0.1
Tcp11Unknown, score: 0.04
Dynlt1bUnknown, score: 0.41
Tdgf1Unknown, score: 0.08
Prdx2Unknown, score: 0.25
Tead2Unknown, score: 0.16
Tead3Unknown, score: 0.4
AlyrefUnknown, score: 0.03
TecUnknown, score: 0.11
TekUnknown, score: 0.24
Tekt1Unknown, score: 0.05
InmtUnknown, score: 0.2
Terf2Unknown, score: 0.08
TertUnknown, score: 0.32
TesUnknown, score: 0.33
Morf4l1Unknown, score: 0.2
Tex2Unknown, score: 0.14
Tex261Unknown, score: 0.05
Tex264Unknown, score: 0.29
Ppp2r5dUnknown, score: 0.04
TfamUnknown, score: 0.39
TfgUnknown, score: 0.06
Tfpi2Unknown, score: 0.45
TgfaUnknown, score: 0.26
Tgfb1Unknown, score: 0.04
Tsc22d1Unknown, score: 0.07
Tgfb3Unknown, score: 0.4
Tgfbr1Unknown, score: 0.13
Tgif1Unknown, score: 0.21
Tgm1Unknown, score: 0.13
Tgm2Unknown, score: 0.17
Tgtp1Unknown, score: 0.21
ThbdUnknown, score: 0.27
Thbs1Unknown, score: 0.11
Thbs2Unknown, score: 0.33
Thbs4Unknown, score: 0.17
ThpoUnknown, score: 0.23
ThrbUnknown, score: 0.18
ThrspUnknown, score: 0.24
Tiam1Unknown, score: 0.04
Trim24Unknown, score: 0.03
Timm17aUnknown, score: 0.28
Timm44Unknown, score: 0.37
Timp1Unknown, score: 0.04
Tk1Unknown, score: 0.38
TktUnknown, score: 0.1
Tle1Unknown, score: 0.38
Tle2Unknown, score: 0.31
Tll1Unknown, score: 0.53
Tln1Unknown, score: 0.1
Tlr6Unknown, score: 0.34
DtymkUnknown, score: 0.09
TmpoUnknown, score: 0.08
Clec3bUnknown, score: 0.37
TncUnknown, score: 0.05
Tnfaip3Unknown, score: 0.33
Tnfrsf11aUnknown, score: 0.09
Tnfrsf17Unknown, score: 0.23
Tnfrsf18Unknown, score: 0.48
Tnfrsf1bUnknown, score: 0.18
Cd40Unknown, score: 0.24
Tnfrsf9Unknown, score: 0.03
DeddUnknown, score: 0.13
Cd40lgUnknown, score: 0.07
Tnfsf8Unknown, score: 0.31
Tnni1Unknown, score: 0.19
Tnni2Unknown, score: 0.11
Tnnt2Unknown, score: 0.06
Tnnt3Unknown, score: 0.09
TnrUnknown, score: 0.12
Tns1Unknown, score: 0.14
Top3aUnknown, score: 0.26
Ppp1r13bUnknown, score: 0.12
Tmem165Unknown, score: 0.49
TpbgUnknown, score: 0.14
Tpd52l1Unknown, score: 0.11
Tpm2Unknown, score: 0.03
Tpst1Unknown, score: 0.48
Nr2c1Unknown, score: 0.35
Hsp90b1Unknown, score: 0.17
Traf1Unknown, score: 0.2
Traf2Unknown, score: 0.22
Traf3Unknown, score: 0.19
TraipUnknown, score: 0.1
TrfUnknown, score: 0.14
TfrcUnknown, score: 0.32
Trip6Unknown, score: 0.29
Trpc1Unknown, score: 0.18
Trpc4Unknown, score: 0.06
Ctr9Unknown, score: 0.62
Rsph1Unknown, score: 0.13
Tspyl1Unknown, score: 0.03
Tssk1Unknown, score: 0.12
TsksUnknown, score: 0.63
TstUnknown, score: 0.45
Rpl13aUnknown, score: 0.18
Tsta3Unknown, score: 0.29
Ttf1Unknown, score: 0.05
Tgoln1Unknown, score: 0.05
TtkUnknown, score: 0.09
TtnUnknown, score: 0.17
TtrUnknown, score: 0.06
TubUnknown, score: 0.19
Tuba1aUnknown, score: 0.24
Tuba3aUnknown, score: 0.05
Tuba4aUnknown, score: 0.11
Tuba3bUnknown, score: 0.25
Tubb2aUnknown, score: 0.2
Tubb3Unknown, score: 0.22
Tubb4aUnknown, score: 0.33
Twist1Unknown, score: 0.02
Tnfrsf4Unknown, score: 0.04
TyrUnknown, score: 0.2
U2af2Unknown, score: 0.3
Uba52Unknown, score: 0.25
UbbUnknown, score: 0.1
UbcUnknown, score: 0.1
Ube2mUnknown, score: 0.35
Uba3Unknown, score: 0.06
Uba1Unknown, score: 0.25
Ube2aUnknown, score: 0.07
Ube2bUnknown, score: 0.19
Ube2g2Unknown, score: 0.15
Ube3aUnknown, score: 0.04
Ubr1Unknown, score: 0.46
Uchl1Unknown, score: 0.13
Usp5Unknown, score: 0.24
UcnUnknown, score: 0.5
Ufd1lUnknown, score: 0.33
Dpysl3Unknown, score: 0.18
Ulk1Unknown, score: 0.49
Uck1Unknown, score: 0.07
UmpsUnknown, score: 0.25
Unc119Unknown, score: 0.23
Unc5cUnknown, score: 0.16
Usp4Unknown, score: 0.33
Nr1h3Unknown, score: 0.3
Upp1Unknown, score: 0.2
UqcrqUnknown, score: 0.19
Uqcrc1Unknown, score: 0.68
UrosUnknown, score: 0.29
Usf1Unknown, score: 0.23
Ush2aUnknown, score: 0.03
UtyUnknown, score: 0.22
Slc45a2Unknown, score: 0.03
Vmn1r45Unknown, score: 0.26
Vamp1Unknown, score: 0.02
Vamp3Unknown, score: 0.12
Vamp8Unknown, score: 0.28
VclUnknown, score: 0.13
VegfaUnknown, score: 0.2
VegfcUnknown, score: 0.07
Lin7cUnknown, score: 0.09
Vezf1Unknown, score: 0.19
EzrUnknown, score: 0.03
VillUnknown, score: 0.2
Vipr1Unknown, score: 0.08
VldlrUnknown, score: 0.16
Vps45Unknown, score: 0.05
Vrk1Unknown, score: 0.29
Trpv2Unknown, score: 0.05
VtnUnknown, score: 0.29
WapUnknown, score: 0.05
WasUnknown, score: 0.02
Wbp1Unknown, score: 0.1
Wbp5Unknown, score: 0.04
Baz1bUnknown, score: 0.08
Wdr1Unknown, score: 0.13
Wee1Unknown, score: 0.73
Wfs1Unknown, score: 0.06
Zmat3Unknown, score: 0.48
Wisp1Unknown, score: 0.03
Wisp2Unknown, score: 0.05
WizUnknown, score: 0.13
Wnt1Unknown, score: 0.17
Wnt10bUnknown, score: 0.62
Wnt11Unknown, score: 0.29
Wnt9bUnknown, score: 0.18
Wnt2Unknown, score: 0.15
Wnt4Unknown, score: 0.42
Wnt5aUnknown, score: 0.25
Wnt6Unknown, score: 0.16
Dctn6Unknown, score: 0.17
XpcUnknown, score: 0.04
Ercc5Unknown, score: 0.1
Slc6a20bUnknown, score: 0.31
SiaeUnknown, score: 0.29
Map3k19Unknown, score: 0.17
YwhaeUnknown, score: 0.24
YwhazUnknown, score: 0.03
Yy1Unknown, score: 0.22
Plagl1Unknown, score: 0.13
ZanUnknown, score: 0.1
Zfp1Unknown, score: 0.25
Zfp11Unknown, score: 0.11
Mkrn3Unknown, score: 0.2
Zfp13Unknown, score: 0.35
Zbtb14Unknown, score: 0.03
Zfand5Unknown, score: 0.31
Zscan2Unknown, score: 0.02
Zfp35Unknown, score: 0.09
Zfp40Unknown, score: 0.12
Zfp41Unknown, score: 0.13
Zfp62Unknown, score: 0.3
Zfp64Unknown, score: 0.06
Zfp90Unknown, score: 0.03
Zfp93Unknown, score: 0.16
Zfp94Unknown, score: 0.23
Zscan12Unknown, score: 0.44
Zfpm1Unknown, score: 0.2
Zhx1Unknown, score: 0.03
Ikzf4Unknown, score: 0.1
Slc30a1Unknown, score: 0.05
Slc30a4Unknown, score: 0.34
Dnajc2Unknown, score: 0.17
ZyxUnknown, score: 0.11
Coro1bUnknown, score: 0.26
Coro1cUnknown, score: 0.25
Akt3Unknown, score: 0.16
Aloxe3Unknown, score: 0.21
AmfrUnknown, score: 0.38
Arih1Unknown, score: 0.19
Ash2lUnknown, score: 0.09
Banf1Unknown, score: 0.04
BvesUnknown, score: 0.05
C1ql1Unknown, score: 0.38
Xcr1Unknown, score: 0.22
Klf6Unknown, score: 0.13
Pappa2Unknown, score: 0.17
Def6Unknown, score: 0.12
Def8Unknown, score: 0.06
Dmtf1Unknown, score: 0.02
Dlg2Unknown, score: 0.42
FarsbUnknown, score: 0.42
Fbln5Unknown, score: 0.11
Fiz1Unknown, score: 0.21
FybUnknown, score: 0.08
G3bp2Unknown, score: 0.02
Gdf15Unknown, score: 0.36
Ggt5Unknown, score: 0.2
Hax1Unknown, score: 0.17
RhofUnknown, score: 0.38
Impdh1Unknown, score: 0.21
InsrrUnknown, score: 0.49
Sh2b2Unknown, score: 0.16
Katna1Unknown, score: 0.03
Lamc3Unknown, score: 0.09
Map2k5Unknown, score: 0.29
Mta2Unknown, score: 0.17
Esyt1Unknown, score: 0.25
Mid2Unknown, score: 0.14
Nek3Unknown, score: 0.05
Neu2Unknown, score: 0.1
Tenm2Unknown, score: 0.16
Tenm4Unknown, score: 0.22
Pacsin1Unknown, score: 0.06
Pacsin2Unknown, score: 0.14
Papss2Unknown, score: 0.29
Pebp1Unknown, score: 0.21
Pcbp1Unknown, score: 0.11
Pde10aUnknown, score: 0.3
Eci2Unknown, score: 0.37
Pin1Unknown, score: 0.27
Med24Unknown, score: 0.5
Cib1Unknown, score: 0.05
PrkraUnknown, score: 0.06
Dazap2Unknown, score: 0.23
Twf2Unknown, score: 0.27
Ptpn21Unknown, score: 0.16
RngttUnknown, score: 0.35
Mrps12Unknown, score: 0.38
Scamp2Unknown, score: 0.07
Scamp3Unknown, score: 0.14
Ccl19Unknown, score: 0.01
SgcbUnknown, score: 0.02
SgcdUnknown, score: 0.4
SgcgUnknown, score: 0.18
Sh3yl1Unknown, score: 0.46
SigirrUnknown, score: 0.39
Sra1Unknown, score: 0.19
SufuUnknown, score: 0.21
Taf7Unknown, score: 0.03
Taf10Unknown, score: 0.29
Tlr2Unknown, score: 0.15
Tnfsf13bUnknown, score: 0.29
Tpra1Unknown, score: 0.17
UbdUnknown, score: 0.19
Usp18Unknown, score: 0.23
Best1Unknown, score: 0.02
Wif1Unknown, score: 0.05
Aldh1a7Unknown, score: 0.22
Angptl2Unknown, score: 0.25
AxlUnknown, score: 0.36
Ceacam1Unknown, score: 0.18
Ceacam2Unknown, score: 0.04
Cetn2Unknown, score: 0.29
Ciao1Unknown, score: 0.34
Clcn6Unknown, score: 0.22
Rfwd2Unknown, score: 0.24
Dapp1Unknown, score: 0.2
Decr2Unknown, score: 0.55
EsrraUnknown, score: 0.08
EsrrbUnknown, score: 0.12
EsrrgUnknown, score: 0.2
Fgd2Unknown, score: 0.06
FtoUnknown, score: 0.15
Gnpda1Unknown, score: 0.24
Grk6Unknown, score: 0.22
Hsf4Unknown, score: 0.05
Ifi202bUnknown, score: 0.16
Mapkbp1Unknown, score: 0.58
Lypla2Unknown, score: 0.41
Map2k1Unknown, score: 0.36
Map2k2Unknown, score: 0.17
Map2k4Unknown, score: 0.27
Map2k6Unknown, score: 0.06
Map3k1Unknown, score: 0.13
Map3k2Unknown, score: 0.52
Map3k3Unknown, score: 0.34
Map3k7Unknown, score: 0.05
Map4k1Unknown, score: 0.05
Mapk1Unknown, score: 0.48
Mapk10Unknown, score: 0.2
Mapk14Unknown, score: 0.12
Nr5a2Unknown, score: 0.44
Nubp1Unknown, score: 0.18
Nubp2Unknown, score: 0.19
Orc5Unknown, score: 0.07
PargUnknown, score: 0.02
Plod2Unknown, score: 0.8
Psma7Unknown, score: 0.06
Psmb2Unknown, score: 0.26
PoliUnknown, score: 0.33
Sema4gUnknown, score: 0.29
Slc27a5Unknown, score: 0.11
Txnrd2Unknown, score: 0.11
Zfp260Unknown, score: 0.37
Cul3Unknown, score: 0.14
Homer2Unknown, score: 0.32
HunkUnknown, score: 0.03
Mmp23Unknown, score: 0.46
NcdnUnknown, score: 0.28
Ror1Unknown, score: 0.13
Ror2Unknown, score: 0.31
Slc27a3Unknown, score: 0.4
Slc27a4Unknown, score: 0.25
Slc7a11Unknown, score: 0.04
Cops5Unknown, score: 0.26
B3galt2Unknown, score: 0.08
CenphUnknown, score: 0.13
Cops4Unknown, score: 0.17
Cops7aUnknown, score: 0.43
Acot1Unknown, score: 0.02
Ddx3yUnknown, score: 0.26
Deb1Unknown, score: 0.12
Exo1Unknown, score: 0.24
Gprin1Unknown, score: 0.26
H2afyUnknown, score: 0.28
Zfp346Unknown, score: 0.24
Map4k4Unknown, score: 0.18
MecrUnknown, score: 0.22
Aifm1Unknown, score: 0.02
Foxl2Unknown, score: 0.22
Racgap1Unknown, score: 0.05
St6galnac5Unknown, score: 0.14
Polr3eUnknown, score: 0.18
Serinc3Unknown, score: 0.21
Vat1Unknown, score: 0.18
Zw10Unknown, score: 0.15
IslrUnknown, score: 0.12
Pla2g2eUnknown, score: 0.1
Klrk1Unknown, score: 0.27
PolkUnknown, score: 0.34
ErmapUnknown, score: 0.25
G3bp1Unknown, score: 0.09
Rps3Unknown, score: 0.1
AoahUnknown, score: 0.1
AsnsUnknown, score: 0.06
Fkbp9Unknown, score: 0.17
Irf5Unknown, score: 0.09
Sh3d19Unknown, score: 0.39
Bcap31Unknown, score: 0.04
CadpsUnknown, score: 0.57
Zfp275Unknown, score: 0.03
Xlr4bUnknown, score: 0.09
Trappc3Unknown, score: 0.05
Eif2ak4Unknown, score: 0.4
Syn3Unknown, score: 0.24
Azi2Unknown, score: 0.05
Trp53bp1Unknown, score: 0.03
Tceb3Unknown, score: 0.19
Ddx24Unknown, score: 0.31
Pla2g7Unknown, score: 0.38
Plek2Unknown, score: 0.63
Dok3Unknown, score: 0.12
NbnUnknown, score: 0.02
Sytl4Unknown, score: 0.11
Dnajb9Unknown, score: 0.24
Rpl3Unknown, score: 0.13
Tbl2Unknown, score: 0.14
DguokUnknown, score: 0.1
Rps26Unknown, score: 0.05
Prmt5Unknown, score: 0.08
Tjp3Unknown, score: 0.27
Npas3Unknown, score: 0.12
Sh2d3cUnknown, score: 0.34
Ptdss2Unknown, score: 0.05
Dusp13Unknown, score: 0.02
Mrpl39Unknown, score: 0.18
Mrpl15Unknown, score: 0.16
Mrpl17Unknown, score: 0.27
Mrpl2Unknown, score: 0.22
PdhxUnknown, score: 0.88
Abca8bUnknown, score: 0.22
Abcg3Unknown, score: 0.12
Abcf3Unknown, score: 0.1
Abcg5Unknown, score: 0.33
Abcb11Unknown, score: 0.33
Abcc6Unknown, score: 0.12
Klra1Unknown, score: 0.2
Atp5lUnknown, score: 0.44
NagpaUnknown, score: 0.5
Ubl4Unknown, score: 0.23
1700088E04RikUnknown, score: 0.21
Snf8Unknown, score: 0.09
Lsm2Unknown, score: 0.48
Commd8Unknown, score: 0.27
Zdhhc8Unknown, score: 0.11
Dgcr14Unknown, score: 0.08
Rrp9Unknown, score: 0.39
CherpUnknown, score: 0.02
Rsrp1Unknown, score: 0.29
Efhd2Unknown, score: 0.04
Imp4Unknown, score: 0.03
Fam3cUnknown, score: 0.13
Fam21Unknown, score: 0.03
Polr2mUnknown, score: 0.09
Ing4Unknown, score: 0.06
D6Wsu163eUnknown, score: 0.23
Ept1Unknown, score: 0.6
Desi1Unknown, score: 0.16
Atp5oUnknown, score: 0.27
Vps25Unknown, score: 0.23
RtcbUnknown, score: 0.23
Trim36Unknown, score: 0.51
D10Wsu102eUnknown, score: 0.18
AponUnknown, score: 0.42
Dhrs4Unknown, score: 0.14
Slco1b2Unknown, score: 0.35
D10Jhu81eUnknown, score: 0.04
MgaUnknown, score: 0.03
Rabgap1lUnknown, score: 0.07
Ndrg2Unknown, score: 0.03
Ndrg3Unknown, score: 0.22
Bcar3Unknown, score: 0.15
Igfbp7Unknown, score: 0.41
Stau2Unknown, score: 0.62
Tnfrsf19Unknown, score: 0.29
SmtnUnknown, score: 0.02
Mapk12Unknown, score: 0.07
Dpf1Unknown, score: 0.32
Rnf11Unknown, score: 0.22
Cspg5Unknown, score: 0.15
Hdgfrp3Unknown, score: 0.55
Scd3Unknown, score: 0.09
Fbxw2Unknown, score: 0.29
Rnf17Unknown, score: 0.61
Timm13Unknown, score: 0.15
Timm8a1Unknown, score: 0.02
Timm10Unknown, score: 0.15
Cttnbp2Unknown, score: 0.03
Slc39a1Unknown, score: 0.52
Fkbp3Unknown, score: 0.11
Slc22a4Unknown, score: 0.04
Adamts8Unknown, score: 0.55
Fbxw4Unknown, score: 0.2
Fbxw5Unknown, score: 0.41
Fbxl6Unknown, score: 0.42
Kdm2bUnknown, score: 0.31
Fbxl12Unknown, score: 0.12
Zfp330Unknown, score: 0.41
Tor2aUnknown, score: 0.38
Lmcd1Unknown, score: 0.07
Usp25Unknown, score: 0.13
Rnf19aUnknown, score: 0.32
Lcmt1Unknown, score: 0.51
Cngb3Unknown, score: 0.12
Schip1Unknown, score: 0.37
Pik3cgUnknown, score: 0.03
AassUnknown, score: 0.1
VapaUnknown, score: 0.05
Nox4Unknown, score: 0.23
Thop1Unknown, score: 0.36
Txnrd1Unknown, score: 0.25
Hspa14Unknown, score: 0.19
TtpaUnknown, score: 0.1
Ercc4Unknown, score: 0.28
Lats2Unknown, score: 0.02
Sall2Unknown, score: 0.03
Ero1lUnknown, score: 0.2
Mrps7Unknown, score: 0.32
Mfap5Unknown, score: 0.06
PostnUnknown, score: 0.3
SacsUnknown, score: 0.38
Sirt6Unknown, score: 0.09
Fbxo8Unknown, score: 0.08
Fbxo18Unknown, score: 0.08
Fbxo16Unknown, score: 0.34
Fbxo17Unknown, score: 0.19
Tfr2Unknown, score: 0.46
Crim1Unknown, score: 0.18
Pnpla6Unknown, score: 0.13
Dlc1Unknown, score: 0.05
Atp8a2Unknown, score: 0.15
Atp11aUnknown, score: 0.47
Mapk6Unknown, score: 0.15
Nt5cUnknown, score: 0.09
Polg2Unknown, score: 0.06
Rgs6Unknown, score: 0.18
Rgs3Unknown, score: 0.18
Dkk3Unknown, score: 0.34
Rgs11Unknown, score: 0.06
Lsm4Unknown, score: 0.15
Ppap2cUnknown, score: 0.11
Hs6st1Unknown, score: 0.3
Hs6st2Unknown, score: 0.4
Orc3Unknown, score: 0.28
Copb2Unknown, score: 0.09
Slc25a13Unknown, score: 0.28
Capn15Unknown, score: 0.42
SpastUnknown, score: 0.06
Keap1Unknown, score: 0.2
Tmod4Unknown, score: 0.11
Tmod2Unknown, score: 0.08
Neu3Unknown, score: 0.15
Hmgn5Unknown, score: 0.07
PrebUnknown, score: 0.39
C1s1Unknown, score: 0.34
Exosc9Unknown, score: 0.09
Grb14Unknown, score: 0.17
Irx4Unknown, score: 0.3
GalnsUnknown, score: 0.25
Klrg1Unknown, score: 0.07
Il27raUnknown, score: 0.23
Uchl3Unknown, score: 0.13
Slc7a8Unknown, score: 0.18
St6galnac6Unknown, score: 0.13
Cpsf2Unknown, score: 0.55
H2afzUnknown, score: 0.76
Tnk2Unknown, score: 0.43
Rgs14Unknown, score: 0.24
Ppp2r1aUnknown, score: 0.23
Ddah2Unknown, score: 0.4
Ech1Unknown, score: 0.16
Rundc3aUnknown, score: 0.27
Clec4fUnknown, score: 0.39
Tmem141Unknown, score: 0.14
Fubp1Unknown, score: 0.42
KnstrnUnknown, score: 0.15
Kctd18Unknown, score: 0.53
Bbs1Unknown, score: 0.02
PbkUnknown, score: 0.06
Ppp1r10Unknown, score: 0.14
Rab11fip5Unknown, score: 0.09
PvrUnknown, score: 0.13
Agpat5Unknown, score: 0.23
Ccdc97Unknown, score: 0.2
Camk1Unknown, score: 0.12
Anapc4Unknown, score: 0.28
Stbd1Unknown, score: 0.52
Vps37aUnknown, score: 0.07
Wwc2Unknown, score: 0.1
D6Ertd527eUnknown, score: 0.11
Rcn3Unknown, score: 0.09
Sept11Unknown, score: 0.41
Rhpn2Unknown, score: 0.27
Mrpl48Unknown, score: 0.06
Tet1Unknown, score: 0.15
Slc46a1Unknown, score: 0.27
Ctdsp2Unknown, score: 0.26
Angel2Unknown, score: 0.26
Nhp2Unknown, score: 0.17
Acaa2Unknown, score: 0.31
Parp8Unknown, score: 0.13
Trmt10cUnknown, score: 0.06
Dhrs1Unknown, score: 0.11
Brms1lUnknown, score: 0.18
Cbx7Unknown, score: 0.08
Esyt2Unknown, score: 0.17
Nudcd2Unknown, score: 0.19
Ldlrad4Unknown, score: 0.1
Echdc1Unknown, score: 0.35
Arhgef25Unknown, score: 0.04
E2f7Unknown, score: 0.08
Cd300lgUnknown, score: 0.16
Mettl2Unknown, score: 0.17
Zfp410Unknown, score: 0.37
Pnrc2Unknown, score: 0.11
Tmx4Unknown, score: 0.17
Dbndd2Unknown, score: 0.13
Cnot11Unknown, score: 0.21
Lair1Unknown, score: 0.02
Gramd1aUnknown, score: 0.14
Ahi1Unknown, score: 0.33
Nub1Unknown, score: 0.26
Atp2a3Unknown, score: 0.09
BatfUnknown, score: 0.02
Plrg1Unknown, score: 0.27
Pdlim3Unknown, score: 0.08
Nxf1Unknown, score: 0.03
Ube2kUnknown, score: 0.13
BanpUnknown, score: 0.03
Stx7Unknown, score: 0.06
Mtmr1Unknown, score: 0.44
Eif3gUnknown, score: 0.26
Pla2g6Unknown, score: 0.53
Chst3Unknown, score: 0.17
Mtx2Unknown, score: 0.08
Psmd10Unknown, score: 0.58
Txnl1Unknown, score: 0.27
Exoc7Unknown, score: 0.33
Htatip2Unknown, score: 0.16
Stk39Unknown, score: 0.03
Hif3aUnknown, score: 0.38
B4galt2Unknown, score: 0.58
CorinUnknown, score: 0.36
Syt5Unknown, score: 0.42
Sec61a1Unknown, score: 0.38
Ybx2Unknown, score: 0.3
Cd164Unknown, score: 0.06
Hpcal1Unknown, score: 0.03
Nap1l1Unknown, score: 0.19
SnrpaUnknown, score: 0.26
Map3k6Unknown, score: 0.39
NonoUnknown, score: 0.13
Fut8Unknown, score: 0.07
BlcapUnknown, score: 0.27
Gria3Unknown, score: 0.16
Cldn7Unknown, score: 0.2
B3gnt2Unknown, score: 0.08
PorcnUnknown, score: 0.3
Prrc2aUnknown, score: 0.2
Tlr5Unknown, score: 0.09
Oaz3Unknown, score: 0.16
Rwdd2bUnknown, score: 0.13
Map3k14Unknown, score: 0.06
Zranb2Unknown, score: 0.02
Cntn6Unknown, score: 0.24
Caprin1Unknown, score: 0.2
Slc5a3Unknown, score: 0.73
Celsr2Unknown, score: 0.22
ClppUnknown, score: 0.22
Slc7a10Unknown, score: 0.1
Rcan2Unknown, score: 0.35
Rfx5Unknown, score: 0.11
Diap2Unknown, score: 0.13
UevldUnknown, score: 0.03
Irf7Unknown, score: 0.04
Cks1bUnknown, score: 0.28
Arhgef7Unknown, score: 0.57
Rps28Unknown, score: 0.12
Pdlim1Unknown, score: 0.4
LsrUnknown, score: 0.18
Rdh7Unknown, score: 0.14
Dnal4Unknown, score: 0.13
Copg1Unknown, score: 0.17
IcosUnknown, score: 0.04
Kat6bUnknown, score: 0.11
Pabpn1Unknown, score: 0.19
Snx3Unknown, score: 0.16
Ccrl2Unknown, score: 0.37
Golga4Unknown, score: 0.01
Cd160Unknown, score: 0.16
Pcdh7Unknown, score: 0.19
Rpl36Unknown, score: 0.28
B3galt4Unknown, score: 0.2
Arhgef5Unknown, score: 0.42
Gnpnat1Unknown, score: 0.05
Atf7ipUnknown, score: 0.18
Elp5Unknown, score: 0.11
Irx5Unknown, score: 0.18
Rpp30Unknown, score: 0.65
Zfp326Unknown, score: 0.12
Gp9Unknown, score: 0.15
Nme6Unknown, score: 0.03
Cacng6Unknown, score: 0.32
CpqUnknown, score: 0.42
Mtmr7Unknown, score: 0.4
Mcm3apUnknown, score: 0.04
Hils1Unknown, score: 0.1
Gabbr1Unknown, score: 0.03
Irgm2Unknown, score: 0.29
Ppt2Unknown, score: 0.11
YwhabUnknown, score: 0.14
Stk19Unknown, score: 0.73
Slc4a4Unknown, score: 0.22
Ndufa1Unknown, score: 0.21
Ramp2Unknown, score: 0.14
Fmn2Unknown, score: 0.06
Barhl1Unknown, score: 0.4
HgfacUnknown, score: 0.19
Dnmt3lUnknown, score: 0.33
Unc93b1Unknown, score: 0.06
Cpsf3Unknown, score: 0.21
TollipUnknown, score: 0.16
MefvUnknown, score: 0.28
Apbb1ipUnknown, score: 0.1
Syt6Unknown, score: 0.32
Nap1l3Unknown, score: 0.31
CalcrlUnknown, score: 0.16
Foxo4Unknown, score: 0.27
Socs6Unknown, score: 0.38
Abhd2Unknown, score: 0.28
Tbc1d8Unknown, score: 0.43
Pde3aUnknown, score: 0.46
St3gal6Unknown, score: 0.04
Prpf40bUnknown, score: 0.15
Paf1Unknown, score: 0.28
Pqbp1Unknown, score: 0.3
PdgfcUnknown, score: 0.15
Wdr45Unknown, score: 0.11
Praf2Unknown, score: 0.31
Gripap1Unknown, score: 0.34
Ppp1r3fUnknown, score: 0.19
Ccdc120Unknown, score: 0.07
Usp27xUnknown, score: 0.09
Cacna1fUnknown, score: 0.19
Atp8b1Unknown, score: 0.03
CrtamUnknown, score: 0.14
Hs3st3b1Unknown, score: 0.02
Rcan1Unknown, score: 0.34
Dfna5Unknown, score: 0.2
Cadm1Unknown, score: 0.1
Syt8Unknown, score: 0.48
ApomUnknown, score: 0.11
Ap3m1Unknown, score: 0.12
Dclre1aUnknown, score: 0.07
Eef1b2Unknown, score: 0.09
Bri3Unknown, score: 0.26
Ebag9Unknown, score: 0.12
Ift20Unknown, score: 0.29
Agpat1Unknown, score: 0.09
Impa1Unknown, score: 0.27
PigbUnknown, score: 0.17
Pdzrn3Unknown, score: 0.36
Nop58Unknown, score: 0.03
Fmo2Unknown, score: 0.32
Panx1Unknown, score: 0.12
Smad9Unknown, score: 0.05
Hebp2Unknown, score: 0.27
Slc2a8Unknown, score: 0.11
Stard10Unknown, score: 0.27
PpieUnknown, score: 0.31
Ccnl2Unknown, score: 0.62
Uqcc1Unknown, score: 0.29
Cyp39a1Unknown, score: 0.2
Tcerg1Unknown, score: 0.11
Lgals12Unknown, score: 0.18
DgkeUnknown, score: 0.07
SetUnknown, score: 0.02
Psmg1Unknown, score: 0.16
Ftsj3Unknown, score: 0.08
PigpUnknown, score: 0.3
Olfm1Unknown, score: 0.35
Prodh2Unknown, score: 0.48
Uchl5Unknown, score: 0.11
Rev1Unknown, score: 0.18
RhogUnknown, score: 0.31
Htra1Unknown, score: 0.21
Acin1Unknown, score: 0.14
Mpp5Unknown, score: 0.21
Patz1Unknown, score: 0.33
Ccl24Unknown, score: 0.16
Tspan5Unknown, score: 0.53
Ube2j1Unknown, score: 0.12
Thsd1Unknown, score: 0.24
Actr8Unknown, score: 0.12
Cpxm1Unknown, score: 0.02
Stk3Unknown, score: 0.2
Rbm14Unknown, score: 0.08
Gkap1Unknown, score: 0.31
Fam69bUnknown, score: 0.11
Mrpl37Unknown, score: 0.47
Mrpl19Unknown, score: 0.24
Rassf1Unknown, score: 0.39
StyxUnknown, score: 0.31
Ptpn9Unknown, score: 0.15
Arl6Unknown, score: 0.04
Gps2Unknown, score: 0.04
Zfp113Unknown, score: 0.28
GgcxUnknown, score: 0.48
Anapc7Unknown, score: 0.14
AcppUnknown, score: 0.4
AatfUnknown, score: 0.28
Timm22Unknown, score: 0.11
Stam2Unknown, score: 0.46
Abcb9Unknown, score: 0.15
Mettl3Unknown, score: 0.31
Dnajc7Unknown, score: 0.06
IvdUnknown, score: 0.03
Acot9Unknown, score: 0.45
Pus1Unknown, score: 0.22
Tmeff2Unknown, score: 0.12
ScocUnknown, score: 0.33
Tagln3Unknown, score: 0.34
Fzr1Unknown, score: 0.06
Tmem59Unknown, score: 0.17
Pdlim5Unknown, score: 0.14
Arid3bUnknown, score: 0.01
Cyp3a25Unknown, score: 0.04
Stx5aUnknown, score: 0.21
Shoc2Unknown, score: 0.45
Morf4l2Unknown, score: 0.2
Akap8Unknown, score: 0.18
Trip4Unknown, score: 0.03
Dusp14Unknown, score: 0.19
Ncoa6Unknown, score: 0.09
Trpc4apUnknown, score: 0.28
Nudt3Unknown, score: 0.06
Cbln3Unknown, score: 0.17
Noa1Unknown, score: 0.12
AdarUnknown, score: 0.32
Diap3Unknown, score: 0.15
Ppp4cUnknown, score: 0.28
Hbs1lUnknown, score: 0.27
Tubd1Unknown, score: 0.03
Tspan3Unknown, score: 0.54
Adrm1Unknown, score: 0.46
RradUnknown, score: 0.36
Rbx1Unknown, score: 0.06
Snx1Unknown, score: 0.36
Serinc1Unknown, score: 0.47
Dnaja2Unknown, score: 0.11
Copz1Unknown, score: 0.36
Orc6Unknown, score: 0.12
Mbtps1Unknown, score: 0.11
Dynll1Unknown, score: 0.09
Actl6aUnknown, score: 0.06
Pkp3Unknown, score: 0.05
Kcnip3Unknown, score: 0.52
CtsfUnknown, score: 0.14
Socs5Unknown, score: 0.19
Rgs19Unknown, score: 0.15
Stmn4Unknown, score: 0.13
Slc2a5Unknown, score: 0.21
GabarapUnknown, score: 0.35
Nxt1Unknown, score: 0.14
IkbkeUnknown, score: 0.08
Zbtb20Unknown, score: 0.12
VapbUnknown, score: 0.2
Gosr2Unknown, score: 0.21
Ankrd49Unknown, score: 0.18
Srpk3Unknown, score: 0.03
Ruvbl1Unknown, score: 0.23
Rapgef4Unknown, score: 0.62
Rnf138Unknown, score: 0.2
Rbms2Unknown, score: 0.41
Mpp6Unknown, score: 0.02
Sept6Unknown, score: 0.22
Ylpm1Unknown, score: 0.05
Pex3Unknown, score: 0.18
IckUnknown, score: 0.09
Ube2d2aUnknown, score: 0.12
Pfdn5Unknown, score: 0.51
Mgst1Unknown, score: 0.36
Clec4eUnknown, score: 0.37
Adam21Unknown, score: 0.16
Trim17Unknown, score: 0.16
Lamtor3Unknown, score: 0.24
CrtapUnknown, score: 0.02
PnkdUnknown, score: 0.02
PhaxUnknown, score: 0.27
Cdc42ep4Unknown, score: 0.23
Hist1h1bUnknown, score: 0.47
Zfp111Unknown, score: 0.56
Clcf1Unknown, score: 0.45
Rabgef1Unknown, score: 0.37
Mlst8Unknown, score: 0.28
Tdo2Unknown, score: 0.1
Sh3bgrlUnknown, score: 0.06
Alg2Unknown, score: 0.31
Psrc1Unknown, score: 0.05
Lat2Unknown, score: 0.71
Pf4Unknown, score: 0.04
C1qtnf1Unknown, score: 0.16
Sez6lUnknown, score: 0.27
Nfu1Unknown, score: 0.21
Aldh9a1Unknown, score: 0.03
Tacstd2Unknown, score: 0.07
Mbnl1Unknown, score: 0.22
Scube2Unknown, score: 0.02
Hacl1Unknown, score: 0.09
Zbtb33Unknown, score: 0.07
Gmeb1Unknown, score: 0.16
Dkk2Unknown, score: 0.48
Dnajb2Unknown, score: 0.25
Trpm5Unknown, score: 0.17
Tssc4Unknown, score: 0.03
Aldh1a3Unknown, score: 0.17
Insm2Unknown, score: 0.08
Slc37a2Unknown, score: 0.18
Cldn9Unknown, score: 0.36
Psg23Unknown, score: 0.11
Rnf32Unknown, score: 0.4
Rbms1Unknown, score: 0.15
Sh3bp1Unknown, score: 0.14
Gtf2ird1Unknown, score: 0.15
Slc12a5Unknown, score: 0.5
Sap30bpUnknown, score: 0.31
Tbx20Unknown, score: 0.41
Xpo4Unknown, score: 0.17
Tob2Unknown, score: 0.25
Ltb4r2Unknown, score: 0.16
RetnlaUnknown, score: 0.23
RetnUnknown, score: 0.2
Fzd2Unknown, score: 0.19
Cxcl14Unknown, score: 0.07
Apba3Unknown, score: 0.1
Slc16a8Unknown, score: 0.16
Vsig2Unknown, score: 0.25
Slc25a20Unknown, score: 0.29
IcmtUnknown, score: 0.31
Psmd8Unknown, score: 0.03
Mrps31Unknown, score: 0.37
Wdr46Unknown, score: 0.13
C1dUnknown, score: 0.14
Srsf4Unknown, score: 0.16
Smpdl3aUnknown, score: 0.09
Terf2ipUnknown, score: 0.31
Jph1Unknown, score: 0.05
Jph3Unknown, score: 0.09
As3mtUnknown, score: 0.24
BC051019Unknown, score: 0.39
B4galt3Unknown, score: 0.19
Smarce1Unknown, score: 0.43
MogsUnknown, score: 0.02
Atp5j2Unknown, score: 0.3
Sult5a1Unknown, score: 0.14
Xrcc2Unknown, score: 0.11
March7Unknown, score: 0.02
Kcne3Unknown, score: 0.01
Slc15a2Unknown, score: 0.04
Stk32cUnknown, score: 0.04
Piwil2Unknown, score: 0.47
Noc3lUnknown, score: 0.05
Dnajb7Unknown, score: 0.15
Fhl5Unknown, score: 0.11
Pglyrp2Unknown, score: 0.42
Tbx21Unknown, score: 0.29
Ttyh1Unknown, score: 0.17
RbakUnknown, score: 0.18
Tnip1Unknown, score: 0.08
Rpl35aUnknown, score: 0.2
Tk2Unknown, score: 0.29
Adck2Unknown, score: 0.2
Angptl4Unknown, score: 0.1
Il17reUnknown, score: 0.16
Zfp318Unknown, score: 0.42
Cdc42se1Unknown, score: 0.19
Pidd1Unknown, score: 0.54
Tbc1d1Unknown, score: 0.05
Hic2Unknown, score: 0.1
Prokr1Unknown, score: 0.08
Rad18Unknown, score: 0.13
Sh3kbp1Unknown, score: 0.11
Ppp1r1aUnknown, score: 0.11
Pdcd1lg2Unknown, score: 0.04
Slc43a3Unknown, score: 0.1
Bcl11bUnknown, score: 0.18
Srrm3Unknown, score: 0.02
Cst10Unknown, score: 0.39
Trem1Unknown, score: 0.38
Rab37Unknown, score: 0.2
Mmp19Unknown, score: 0.17
Cacna1hUnknown, score: 0.11
Fam184bUnknown, score: 0.18
Rnf8Unknown, score: 0.12
Dnaja4Unknown, score: 0.2
Shank3Unknown, score: 0.15
Pvrl1Unknown, score: 0.04
Nkain4Unknown, score: 0.42
DexiUnknown, score: 0.24
Hs1bp3Unknown, score: 0.06
Nap1l5Unknown, score: 0.06
Stx6Unknown, score: 0.12
Slc35b4Unknown, score: 0.21
Eid1Unknown, score: 0.05
Pmaip1Unknown, score: 0.2
Kcnmb4Unknown, score: 0.35
MlxiplUnknown, score: 0.22
Rnase4Unknown, score: 0.28
Akr1a1Unknown, score: 0.32
Efemp2Unknown, score: 0.24
HibadhUnknown, score: 0.3
Fam13aUnknown, score: 0.04
Sumf1Unknown, score: 0.41
F12Unknown, score: 0.17
Smpd3Unknown, score: 0.15
Pole3Unknown, score: 0.29
Wrap73Unknown, score: 0.17
MaeaUnknown, score: 0.62
Pias4Unknown, score: 0.03
Myoz2Unknown, score: 0.06
Ngly1Unknown, score: 0.21
Anapc5Unknown, score: 0.03
Sh3rf1Unknown, score: 0.02
SqrdlUnknown, score: 0.09
Nup160Unknown, score: 0.24
Edf1Unknown, score: 0.25
NamptUnknown, score: 0.2
MkksUnknown, score: 0.18
Ppp2r3cUnknown, score: 0.14
Slc4a8Unknown, score: 0.25
Pxmp4Unknown, score: 0.04
Rhot1Unknown, score: 0.02
Stk25Unknown, score: 0.16
Rnf130Unknown, score: 0.57
Arpp19Unknown, score: 0.54
C1galt1c1Unknown, score: 0.38
Mettl9Unknown, score: 0.23
Hgh1Unknown, score: 0.06
Mrps30Unknown, score: 0.32
EvcUnknown, score: 0.04
Bhlhe22Unknown, score: 0.08
Erbb2ipUnknown, score: 0.32
FetubUnknown, score: 0.33
MidnUnknown, score: 0.13
Jph2Unknown, score: 0.22
Pcbp4Unknown, score: 0.14
Pcbp3Unknown, score: 0.03
Nek7Unknown, score: 0.1
Nek6Unknown, score: 0.18
Dctn5Unknown, score: 0.12
EmcnUnknown, score: 0.25
Chst7Unknown, score: 0.26
FignUnknown, score: 0.19
Sap30Unknown, score: 0.08
Mrpl38Unknown, score: 0.49
Tmem8Unknown, score: 0.14
Il21rUnknown, score: 0.54
Cd274Unknown, score: 0.03
FancgUnknown, score: 0.43
Capn12Unknown, score: 0.12
Kcnq4Unknown, score: 0.07
Fn3kUnknown, score: 0.13
Taf8Unknown, score: 0.1
Zfp296Unknown, score: 0.61
Fam129aUnknown, score: 0.09
Dusp10Unknown, score: 0.04
Rbp7Unknown, score: 0.2
Ube4bUnknown, score: 0.17
Sav1Unknown, score: 0.15
Yeats4Unknown, score: 0.25
Sv2aUnknown, score: 0.14
Clstn2Unknown, score: 0.34
Gpr35Unknown, score: 0.41
ParvgUnknown, score: 0.07
Sdf2l1Unknown, score: 0.09
RalbUnknown, score: 0.7
Sv2bUnknown, score: 0.13
Herpud1Unknown, score: 0.04
Rgs18Unknown, score: 0.09
NgbUnknown, score: 0.24
PtgesUnknown, score: 0.26
Itm2cUnknown, score: 0.43
Tmub1Unknown, score: 0.7
Gng13Unknown, score: 0.39
Fndc4Unknown, score: 0.25
Ms4a8aUnknown, score: 0.29
Sirt2Unknown, score: 0.19
Polr1eUnknown, score: 0.12
FcamrUnknown, score: 0.29
Gpr85Unknown, score: 0.4
Dip2aUnknown, score: 0.09
Zfp280bUnknown, score: 0.42
Ireb2Unknown, score: 0.46
Fgf23Unknown, score: 0.17
Mrps25Unknown, score: 0.09
Mrps14Unknown, score: 0.43
Keg1Unknown, score: 0.31
DpysUnknown, score: 0.1
Svep1Unknown, score: 0.08
Lpin2Unknown, score: 0.1
ScelUnknown, score: 0.17
Tsc1Unknown, score: 0.08
Rtn4rUnknown, score: 0.35
Irak1bp1Unknown, score: 0.09
Nif3l1Unknown, score: 0.14
Arl6ip6Unknown, score: 0.25
Arl6ip5Unknown, score: 0.47
Lrp10Unknown, score: 0.36
Dap3Unknown, score: 0.19
Pmepa1Unknown, score: 0.41
Vps35Unknown, score: 0.33
Bean1Unknown, score: 0.33
Prrg2Unknown, score: 0.13
Xpo7Unknown, score: 0.5
Asb1Unknown, score: 0.24
Asb4Unknown, score: 0.29
Asb2Unknown, score: 0.04
Asb3Unknown, score: 0.27
Clstn1Unknown, score: 0.26
Slc9a3r2Unknown, score: 0.22
Tmem176bUnknown, score: 0.2
ZakUnknown, score: 0.27
TbataUnknown, score: 0.21
Ranbp17Unknown, score: 0.18
Sostdc1Unknown, score: 0.5
Ndufb5Unknown, score: 0.14
Mrpl54Unknown, score: 0.32
Emc6Unknown, score: 0.21
RogdiUnknown, score: 0.04
0610009B22RikUnknown, score: 0.19
SdhcUnknown, score: 0.08
Sf3b6Unknown, score: 0.38
Tmem176aUnknown, score: 0.35
Krtcap2Unknown, score: 0.72
Cystm1Unknown, score: 0.24
Cwc15Unknown, score: 0.15
Aurkaip1Unknown, score: 0.53
Tsen34Unknown, score: 0.07
Tmem42Unknown, score: 0.28
Eif3fUnknown, score: 0.16
Emc3Unknown, score: 0.4
Rmnd5bUnknown, score: 0.31
Ypel3Unknown, score: 0.14
Ndufa3Unknown, score: 0.02
Lsm7Unknown, score: 0.42
SmpxUnknown, score: 0.11
Tspan13Unknown, score: 0.34
Cml1Unknown, score: 0.19
SarnpUnknown, score: 0.16
Tomm6Unknown, score: 0.19
Fkbp11Unknown, score: 0.13
Chchd1Unknown, score: 0.12
Josd2Unknown, score: 0.81
Sf3b5Unknown, score: 0.06
Elof1Unknown, score: 0.1
1110008L16RikUnknown, score: 0.23
Znrd1Unknown, score: 0.14
Ska2Unknown, score: 0.04
Ifitm3Unknown, score: 0.05
Eef1e1Unknown, score: 0.2
Tmem57Unknown, score: 0.05
Necap2Unknown, score: 0.04
Prr13Unknown, score: 0.18
Fbxo36Unknown, score: 0.14
Tmem14cUnknown, score: 0.11
Ufc1Unknown, score: 0.34
Anapc11Unknown, score: 0.45
Pop4Unknown, score: 0.19
Bola2Unknown, score: 0.15
Tma7Unknown, score: 0.39
GrinaUnknown, score: 0.47
Tomm7Unknown, score: 0.06
Chchd5Unknown, score: 0.49
PglsUnknown, score: 0.2
Nudt14Unknown, score: 0.43
Mustn1Unknown, score: 0.16
Nat9Unknown, score: 0.05
Ubl5Unknown, score: 0.25
Ogfod3Unknown, score: 0.07
Acer3Unknown, score: 0.1
Pithd1Unknown, score: 0.09
PycrlUnknown, score: 0.1
Cks2Unknown, score: 0.38
Acyp1Unknown, score: 0.12
1110059E24RikUnknown, score: 0.22
InipUnknown, score: 0.65
Rpl3lUnknown, score: 0.03
Med7Unknown, score: 0.23
RgccUnknown, score: 0.13
Ndufb9Unknown, score: 0.41
Zdhhc12Unknown, score: 0.42
Serpinb1aUnknown, score: 0.21
Trappc2Unknown, score: 0.13
Rpl7l1Unknown, score: 0.25
Thoc7Unknown, score: 0.03
Msmo1Unknown, score: 0.17
Eif1axUnknown, score: 0.08
Atp6v1g2Unknown, score: 0.04
Kcne1lUnknown, score: 0.46
Tmem9Unknown, score: 0.44
NemfUnknown, score: 0.3
Hspbp1Unknown, score: 0.36
Hsbp1l1Unknown, score: 0.03
Nicn1Unknown, score: 0.27
Mrps17Unknown, score: 0.02
EappUnknown, score: 0.22
Fam134bUnknown, score: 0.07
Tmem126aUnknown, score: 0.17
Cox16Unknown, score: 0.2
AamdcUnknown, score: 0.24
1810009A15RikUnknown, score: 0.38
Sec11cUnknown, score: 0.21
Smim8Unknown, score: 0.04
Mrps21Unknown, score: 0.27
Haus2Unknown, score: 0.64
Rmdn1Unknown, score: 0.4
MplkipUnknown, score: 0.25
Dpy30Unknown, score: 0.32
Wdr61Unknown, score: 0.37
Tmem208Unknown, score: 0.02
1700001K19RikUnknown, score: 0.21
Dnajc5bUnknown, score: 0.17
Susd3Unknown, score: 0.52
1700020L24RikUnknown, score: 0.18
Aqp11Unknown, score: 0.11
Fam229bUnknown, score: 0.02
PsenenUnknown, score: 0.27
Tmem177Unknown, score: 0.15
Atp5slUnknown, score: 0.02
Blzf1Unknown, score: 0.54
OstcUnknown, score: 0.22
Zfand1Unknown, score: 0.05
Ccdc90bUnknown, score: 0.11
2310022A10RikUnknown, score: 0.31
Lsm5Unknown, score: 0.62
2310011J03RikUnknown, score: 0.08
Dhrs7Unknown, score: 0.06
Ndufc1Unknown, score: 0.35
Cox14Unknown, score: 0.32
Ppp1r7Unknown, score: 0.18
CutcUnknown, score: 0.02
Slmo2Unknown, score: 0.07
NosipUnknown, score: 0.16
Sar1bUnknown, score: 0.18
Commd5Unknown, score: 0.16
TsfmUnknown, score: 0.16
Nudt2Unknown, score: 0.14
Rtfdc1Unknown, score: 0.27
Mcts2Unknown, score: 0.51
Mrps15Unknown, score: 0.29
Mterf3Unknown, score: 0.02
Ndufa12Unknown, score: 0.31
Mrpl11Unknown, score: 0.12
Polr2eUnknown, score: 0.15
Dctpp1Unknown, score: 0.17
CoprsUnknown, score: 0.09
Pcp4l1Unknown, score: 0.32
Cyb5bUnknown, score: 0.45
Uggt2Unknown, score: 0.26
Tnfaip8l1Unknown, score: 0.13
Cyc1Unknown, score: 0.13
Exosc7Unknown, score: 0.02
Mgst3Unknown, score: 0.29
Mrpl20Unknown, score: 0.29
Pam16Unknown, score: 0.17
PyurfUnknown, score: 0.06
Sys1Unknown, score: 0.34
Rps23Unknown, score: 0.04
Usmg5Unknown, score: 0.12
Rpl15Unknown, score: 0.29
Exoc2Unknown, score: 0.36
Fam136aUnknown, score: 0.26
Rpl35Unknown, score: 0.53
Zmat2Unknown, score: 0.04
Mrpl51Unknown, score: 0.5
Prelid1Unknown, score: 0.14
Ndufb3Unknown, score: 0.11
PpdpfUnknown, score: 0.18
Cmss1Unknown, score: 0.52
Zmynd11Unknown, score: 0.58
Psmg3Unknown, score: 0.2
ChtopUnknown, score: 0.26
Tab1Unknown, score: 0.16
Rwdd1Unknown, score: 0.41
2810004N23RikUnknown, score: 0.21
Tceanc2Unknown, score: 0.18
Cmc2Unknown, score: 0.03
PompUnknown, score: 0.17
Rps19bp1Unknown, score: 0.02
Adamtsl5Unknown, score: 0.15
Metap1dUnknown, score: 0.17
Dzip1Unknown, score: 0.22
Exosc1Unknown, score: 0.04
Crls1Unknown, score: 0.02
Cmpk1Unknown, score: 0.27
Ube2v1Unknown, score: 0.3
FarsaUnknown, score: 0.05
Stoml2Unknown, score: 0.17
DiabloUnknown, score: 0.17
Uqcr11Unknown, score: 0.43
Aste1Unknown, score: 0.24
3110001I22RikUnknown, score: 0.11
Cryzl1Unknown, score: 0.37
Abi3Unknown, score: 0.02
Ribc1Unknown, score: 0.36
Gpatch4Unknown, score: 0.33
Atg4bUnknown, score: 0.01
Snx9Unknown, score: 0.5
Snrnp27Unknown, score: 0.03
PnisrUnknown, score: 0.16
Cdip1Unknown, score: 0.34
Thg1lUnknown, score: 0.05
Golph3Unknown, score: 0.08
Hiatl1Unknown, score: 0.03
Mcm8Unknown, score: 0.28
Tsen15Unknown, score: 0.04
Ctnnbl1Unknown, score: 0.08
RpeUnknown, score: 0.14
Tpgs2Unknown, score: 0.43
NepnUnknown, score: 0.51
Brf2Unknown, score: 0.09
Eef1dUnknown, score: 0.29
Ccdc51Unknown, score: 0.5
Srp72Unknown, score: 0.25
Uba5Unknown, score: 0.31
Tmem41aUnknown, score: 0.38
CcnhUnknown, score: 0.17
Tmed7Unknown, score: 0.29
Rae1Unknown, score: 0.26
Oser1Unknown, score: 0.1
Tceal8Unknown, score: 0.09
Tbc1d15Unknown, score: 0.07
Klhl28Unknown, score: 0.04
Gapvd1Unknown, score: 0.34
Rbm4bUnknown, score: 0.17
Dnase1l2Unknown, score: 0.36
Henmt1Unknown, score: 0.39
Lrrk2Unknown, score: 0.13
Hrasls5Unknown, score: 0.09
Ankrd61Unknown, score: 0.2
Kcng4Unknown, score: 0.55
Emc2Unknown, score: 0.56
Cfap97Unknown, score: 0.26
Adat2Unknown, score: 0.16
PacrglUnknown, score: 0.03
GskipUnknown, score: 0.11
Alg14Unknown, score: 0.15
Prkrip1Unknown, score: 0.14
Rbm22Unknown, score: 0.11
Tmem170Unknown, score: 0.14
Smim7Unknown, score: 0.15
Bcs1lUnknown, score: 0.2
Fbxo25Unknown, score: 0.33
Ttc1Unknown, score: 0.35
Nacc1Unknown, score: 0.76
Nxf1Unknown, score: 0.08
EtfdhUnknown, score: 0.24
Fuca2Unknown, score: 0.03
Ppp1r2Unknown, score: 0.05
Pnpla2Unknown, score: 0.14
Dnajc10Unknown, score: 0.03
PmpcaUnknown, score: 0.29
Hmg20aUnknown, score: 0.08
Cpne8Unknown, score: 0.28
Rsrc1Unknown, score: 0.05
Lonp2Unknown, score: 0.24
Rnf128Unknown, score: 0.12
Lman2Unknown, score: 0.24
Wwp2Unknown, score: 0.37
Fip1l1Unknown, score: 0.12
Plin3Unknown, score: 0.35
Bzw2Unknown, score: 0.22
Kdelr2Unknown, score: 0.15
TonslUnknown, score: 0.21
Myeov2Unknown, score: 0.03
Pbrm1Unknown, score: 0.37
Trmt6Unknown, score: 0.05
Tmem261Unknown, score: 0.13
Asf1bUnknown, score: 0.11
1700010I14RikUnknown, score: 0.27
Cir1Unknown, score: 0.16
Sh3d21Unknown, score: 0.27
AagabUnknown, score: 0.49
Ddx18Unknown, score: 0.17
Trim59Unknown, score: 0.03
Tmem206Unknown, score: 0.14
Tmx2Unknown, score: 0.02
Fam188aUnknown, score: 0.08
Golt1bUnknown, score: 0.18
Cdk5rap1Unknown, score: 0.18
Slc25a23Unknown, score: 0.17
Luc7lUnknown, score: 0.25
Zfp830Unknown, score: 0.19
Lap3Unknown, score: 0.25
Kctd20Unknown, score: 0.17
Smarcd3Unknown, score: 0.19
Cep19Unknown, score: 0.05
Psmd5Unknown, score: 0.02
Polr3kUnknown, score: 0.15
Ccdc91Unknown, score: 0.15
Actr6Unknown, score: 0.29
Tmem88Unknown, score: 0.4
Use1Unknown, score: 0.32
Rpl11Unknown, score: 0.15
Thap4Unknown, score: 0.09
Mrpl45Unknown, score: 0.01
Rbm25Unknown, score: 0.03
Ddx17Unknown, score: 0.06
Oxct1Unknown, score: 0.03
Chd1Unknown, score: 0.04
Rpp14Unknown, score: 0.06
Ola1Unknown, score: 0.03
Slc25a53Unknown, score: 0.26
Polr3dUnknown, score: 0.19
Romo1Unknown, score: 0.08
Lsm14aUnknown, score: 0.05
Rps6ka6Unknown, score: 0.01
Pi4k2bUnknown, score: 0.14
Tex40Unknown, score: 0.75
PgpUnknown, score: 0.07
Ctnnbip1Unknown, score: 0.23
Psmc6Unknown, score: 0.41
GatmUnknown, score: 0.38
MmachcUnknown, score: 0.12
Mettl21aUnknown, score: 0.28
2310039H08RikUnknown, score: 0.13
D16Ertd472eUnknown, score: 0.05
Zbtb8osUnknown, score: 0.09
Zfp787Unknown, score: 0.3
NaaaUnknown, score: 0.12
Dynlt3Unknown, score: 0.03
Ubap1Unknown, score: 0.21
Tspan31Unknown, score: 0.48
Ube2g1Unknown, score: 0.33
Nop56Unknown, score: 0.23
Lrrc40Unknown, score: 0.14
Nkain1Unknown, score: 0.15
Rnf141Unknown, score: 0.21
Lpar6Unknown, score: 0.21
Dram2Unknown, score: 0.16
Ccdc25Unknown, score: 0.05
Pdzk1ip1Unknown, score: 0.05
Ndufa13Unknown, score: 0.29
Rplp2Unknown, score: 0.23
Zmynd19Unknown, score: 0.26
Glod4Unknown, score: 0.15
Armc10Unknown, score: 0.13
Mrpl55Unknown, score: 0.11
Cmtm6Unknown, score: 0.1
L3hypdhUnknown, score: 0.22
Rrp15Unknown, score: 0.51
Rnpc3Unknown, score: 0.02
Tmem19Unknown, score: 0.03
Zfp329Unknown, score: 0.28
Tbc1d20Unknown, score: 0.08
Smc6Unknown, score: 0.02
Gemin6Unknown, score: 0.47
2810474O19RikUnknown, score: 0.28
Marc2Unknown, score: 0.07
Tbc1d19Unknown, score: 0.02
Zfp422Unknown, score: 0.1
Zswim6Unknown, score: 0.35
Ndufb8Unknown, score: 0.05
Fam69aUnknown, score: 0.32
Pagr1aUnknown, score: 0.31
Med31Unknown, score: 0.43
Ccdc53Unknown, score: 0.28
Cwc27Unknown, score: 0.11
Srek1ip1Unknown, score: 0.13
Ccdc137Unknown, score: 0.49
Gprasp1Unknown, score: 0.01
Zc3h13Unknown, score: 0.28
Zc2hc1aUnknown, score: 0.22
1700022I11RikUnknown, score: 0.72
Snrpd3Unknown, score: 0.26
Cstf1Unknown, score: 0.07
RfflUnknown, score: 0.13
1700093K21RikUnknown, score: 0.11
Paxbp1Unknown, score: 0.03
Bbs2Unknown, score: 0.48
Med4Unknown, score: 0.03
Bag4Unknown, score: 0.27
Unc50Unknown, score: 0.21
1110008F13RikUnknown, score: 0.51
Fam132aUnknown, score: 0.13
Fundc2Unknown, score: 0.23
4833420G17RikUnknown, score: 0.05
SrprUnknown, score: 0.48
Pdlim7Unknown, score: 0.38
Soga3Unknown, score: 0.05
Armcx2Unknown, score: 0.31
Ppil4Unknown, score: 0.46
Far1Unknown, score: 0.35
Adck3Unknown, score: 0.2
Nudcd1Unknown, score: 0.56
4921536K21RikUnknown, score: 0.05
Ssr3Unknown, score: 0.16
MtpapUnknown, score: 0.16
RetsatUnknown, score: 0.07
IlkapUnknown, score: 0.57
C1qtnf4Unknown, score: 0.24
IkbipUnknown, score: 0.16
Ergic2Unknown, score: 0.29
Frmd8Unknown, score: 0.08
Decr1Unknown, score: 0.31
Poc5Unknown, score: 0.18
Entpd4Unknown, score: 0.12
Gpatch1Unknown, score: 0.2
Ero1lbUnknown, score: 0.13
Rnf151Unknown, score: 0.44
Saysd1Unknown, score: 0.16
Tvp23bUnknown, score: 0.03
Agpat2Unknown, score: 0.62
Atg12Unknown, score: 0.09
Zswim3Unknown, score: 0.12
GstcdUnknown, score: 0.19
PigmUnknown, score: 0.05
Wdr48Unknown, score: 0.1
NarflUnknown, score: 0.44
Tmem35Unknown, score: 0.22
Mrfap1Unknown, score: 0.32
Tbc1d23Unknown, score: 0.33
Slc25a26Unknown, score: 0.14
Ubxn11Unknown, score: 0.2
Necap1Unknown, score: 0.06
Dusp6Unknown, score: 0.3
Get4Unknown, score: 0.71
Akt1s1Unknown, score: 0.05
Zfp788Unknown, score: 0.03
AasdhpptUnknown, score: 0.18
Nob1Unknown, score: 0.47
Mxra7Unknown, score: 0.05
Anp32bUnknown, score: 0.06
Rabl3Unknown, score: 0.11
Dctn4Unknown, score: 0.03
l7Rn6Unknown, score: 0.14
Rpl38Unknown, score: 0.25
Tceb2Unknown, score: 0.13
Trmt112Unknown, score: 0.44
CutaUnknown, score: 0.21
Luc7l3Unknown, score: 0.24
Ift74Unknown, score: 0.42
Ost4Unknown, score: 0.17
Rnf149Unknown, score: 0.07
1810037I17RikUnknown, score: 0.18
Stx17Unknown, score: 0.42
Dph2Unknown, score: 0.24
Mansc1Unknown, score: 0.06
Fbxo32Unknown, score: 0.41
PpidUnknown, score: 0.38
Samsn1Unknown, score: 0.2
MgarpUnknown, score: 0.35
Slc38a2Unknown, score: 0.19
Prpsap1Unknown, score: 0.41
Caap1Unknown, score: 0.11
Chd8Unknown, score: 0.16
Ilf2Unknown, score: 0.09
Sfr1Unknown, score: 0.17
Rab39bUnknown, score: 0.05
RnlsUnknown, score: 0.02
Dgat2Unknown, score: 0.31
Snx2Unknown, score: 0.14
Rmdn3Unknown, score: 0.15
Sec14l2Unknown, score: 0.15
Nmral1Unknown, score: 0.07
Snap47Unknown, score: 0.07
Idh3aUnknown, score: 0.22
Dnajb11Unknown, score: 0.34
Gpsm1Unknown, score: 0.27
Atg3Unknown, score: 0.21
Nop9Unknown, score: 0.19
1700021F05RikUnknown, score: 0.21
Asprv1Unknown, score: 0.22
Ppp6cUnknown, score: 0.13
2310033P09RikUnknown, score: 0.08
Rgs10Unknown, score: 0.29
Wfdc1Unknown, score: 0.26
Paip2Unknown, score: 0.5
Nsmce4aUnknown, score: 0.19
RprmUnknown, score: 0.14
Tmem33Unknown, score: 0.07
Mdp1Unknown, score: 0.23
Uxs1Unknown, score: 0.34
SarafUnknown, score: 0.07
Rpl4Unknown, score: 0.24
Coa6Unknown, score: 0.3
Fam45aUnknown, score: 0.1
Ppa1Unknown, score: 0.12
RnmtUnknown, score: 0.25
Cmc1Unknown, score: 0.24
Mtfp1Unknown, score: 0.47
Sumf2Unknown, score: 0.25
Gipc1Unknown, score: 0.38
Ppm1mUnknown, score: 0.18
1600012H06RikUnknown, score: 0.31
Ube2fUnknown, score: 0.1
Fam32aUnknown, score: 0.02
Tceb1Unknown, score: 0.15
Rps27lUnknown, score: 0.26
Atp5g2Unknown, score: 0.02
Spata6Unknown, score: 0.28
NifkUnknown, score: 0.26
U2surpUnknown, score: 0.15
Pold3Unknown, score: 0.43
CcnyUnknown, score: 0.87
TrabdUnknown, score: 0.26
Atad1Unknown, score: 0.17
Tmx3Unknown, score: 0.35
Nudt12Unknown, score: 0.37
Ddx59Unknown, score: 0.15
Fam134cUnknown, score: 0.02
1110004E09RikUnknown, score: 0.32
Sdhaf4Unknown, score: 0.32
BambiUnknown, score: 0.13
MurcUnknown, score: 0.08
Col4a3bpUnknown, score: 0.35
Apopt1Unknown, score: 0.17
BphlUnknown, score: 0.05
Tmem178Unknown, score: 0.1
Rnf146Unknown, score: 0.04
Emc4Unknown, score: 0.06
Fam122aUnknown, score: 0.53
Zfp706Unknown, score: 0.02
NmbUnknown, score: 0.56
Zfp593Unknown, score: 0.31
N6amt2Unknown, score: 0.14
2700060E02RikUnknown, score: 0.05
2700062C07RikUnknown, score: 0.17
MpndUnknown, score: 0.12
AenUnknown, score: 0.11
Nutf2Unknown, score: 0.31
Chd1lUnknown, score: 0.12
3010026O09RikUnknown, score: 0.39
Fam173bUnknown, score: 0.05
Lurap1Unknown, score: 0.21
Gltscr2Unknown, score: 0.38
Pdcd2lUnknown, score: 0.14
Pak1ip1Unknown, score: 0.06
DcakdUnknown, score: 0.05
Ociad1Unknown, score: 0.18
Fam92aUnknown, score: 0.09
Nt5c3bUnknown, score: 0.19
Cntd1Unknown, score: 0.23
9430016H08RikUnknown, score: 0.07
ApoolUnknown, score: 0.1
Cep70Unknown, score: 0.16
Eif3hUnknown, score: 0.35
Tigd2Unknown, score: 0.13
Gar1Unknown, score: 0.22
Otub2Unknown, score: 0.07
Gtf2e2Unknown, score: 0.1
B230118H07RikUnknown, score: 0.07
Cgnl1Unknown, score: 0.33
Fam135aUnknown, score: 0.25
SympkUnknown, score: 0.25
Hsbp1Unknown, score: 0.03
Ndufc2Unknown, score: 0.25
Ndufa5Unknown, score: 0.13
Urm1Unknown, score: 0.31
Fam98bUnknown, score: 0.44
Fam166aUnknown, score: 0.19
Efcab2Unknown, score: 0.25
Rpa3Unknown, score: 0.48
Fam96aUnknown, score: 0.2
Babam1Unknown, score: 0.38
Trmt12Unknown, score: 0.24
Pomgnt1Unknown, score: 0.05
Rpa1Unknown, score: 0.28
Toe1Unknown, score: 0.23
2310057M21RikUnknown, score: 0.23
Ddx39Unknown, score: 0.36
4930430F08RikUnknown, score: 0.18
9530077C05RikUnknown, score: 0.04
Stt3bUnknown, score: 0.41
Mfsd10Unknown, score: 0.19
Aar2Unknown, score: 0.04
Aph1cUnknown, score: 0.09
Nudt22Unknown, score: 0.2
Tsr3Unknown, score: 0.04
Sdhaf1Unknown, score: 0.18
Sirt5Unknown, score: 0.13
0610011F06RikUnknown, score: 0.23
Ndufs3Unknown, score: 0.39
Mul1Unknown, score: 0.22
0610030E20RikUnknown, score: 0.09
Rab14Unknown, score: 0.35
Pbld2Unknown, score: 0.05
Ndufa8Unknown, score: 0.13
Tlcd1Unknown, score: 0.13
Ccdc163Unknown, score: 0.13
Nrn1Unknown, score: 0.11
Ankrd13aUnknown, score: 0.1
Lmbrd1Unknown, score: 0.22
Ankrd13dUnknown, score: 0.41
Slc39a13Unknown, score: 0.3
Steap3Unknown, score: 0.58
Dusp23Unknown, score: 0.02
RragaUnknown, score: 0.64
Gpihbp1Unknown, score: 0.29
Dhrs7cUnknown, score: 0.38
Mob1bUnknown, score: 0.4
Ssna1Unknown, score: 0.23
Rmnd5aUnknown, score: 0.28
Phf5aUnknown, score: 0.95
1110007C09RikUnknown, score: 0.23
Tmem140Unknown, score: 0.36
Arel1Unknown, score: 0.11
Tspan11Unknown, score: 0.24
Nsmce2Unknown, score: 0.23
Ptx4Unknown, score: 0.25
Micu2Unknown, score: 0.27
Myadml2Unknown, score: 0.08
Eml1Unknown, score: 0.12
Zfyve21Unknown, score: 0.56
Fam189bUnknown, score: 0.69
Fam96bUnknown, score: 0.18
Wipf2Unknown, score: 0.04
Mrpl13Unknown, score: 0.36
Tmem109Unknown, score: 0.28
CebpzosUnknown, score: 0.24
Uckl1Unknown, score: 0.09
Pdrg1Unknown, score: 0.23
Nufip2Unknown, score: 0.15
Mrps18aUnknown, score: 0.1
Cgref1Unknown, score: 0.08
Ict1Unknown, score: 0.18
Lamtor5Unknown, score: 0.09
Dnajc8Unknown, score: 0.3
PmvkUnknown, score: 0.08
Ppm1fUnknown, score: 0.15
SerhlUnknown, score: 0.37
Mrpl28Unknown, score: 0.11
Ube2cUnknown, score: 0.04
Letmd1Unknown, score: 0.11
Gdpd3Unknown, score: 0.13
1110012L19RikUnknown, score: 0.05
Elac2Unknown, score: 0.31
Myct1Unknown, score: 0.34
Fahd1Unknown, score: 0.21
Tmem216Unknown, score: 0.19
Abhd14aUnknown, score: 0.16
Tab2Unknown, score: 0.17
Samm50Unknown, score: 0.24
Scgb3a1Unknown, score: 0.07
Trpm4Unknown, score: 0.1
Fam172aUnknown, score: 0.12
Smtnl1Unknown, score: 0.64
Fitm1Unknown, score: 0.17
Hnrnpul2Unknown, score: 0.23
Hddc3Unknown, score: 0.05
Ppp1r27Unknown, score: 0.21
Rabl2Unknown, score: 0.11
Cilp2Unknown, score: 0.27
Rnf166Unknown, score: 0.63
1110032F04RikUnknown, score: 0.06
Trim37Unknown, score: 0.76
Dus1lUnknown, score: 0.12
RbfaUnknown, score: 0.21
Smek1Unknown, score: 0.23
Tyw5Unknown, score: 0.07
Acss1Unknown, score: 0.27
AnlnUnknown, score: 0.15
Zfp740Unknown, score: 0.2
MybphlUnknown, score: 0.21
Cgrrf1Unknown, score: 0.23
Synpo2lUnknown, score: 0.13
Cdhr3Unknown, score: 0.11
Wash1Unknown, score: 0.29
Phtf2Unknown, score: 0.1
Taf11Unknown, score: 0.37
Tmem53Unknown, score: 0.31
Gucd1Unknown, score: 0.26
Srpx2Unknown, score: 0.34
FlncUnknown, score: 0.09
Ubr3Unknown, score: 0.3
Tmem214Unknown, score: 0.08
RgmbUnknown, score: 0.07
Prr32Unknown, score: 0.09
Elovl5Unknown, score: 0.08
Btbd10Unknown, score: 0.18
Ppil1Unknown, score: 0.16
Ddi2Unknown, score: 0.42
Zfand2bUnknown, score: 0.12
Pdcl3Unknown, score: 0.39
Mrpl52Unknown, score: 0.03
Foxk2Unknown, score: 0.04
Tulp4Unknown, score: 0.04
Pih1d1Unknown, score: 0.2
1190002N15RikUnknown, score: 0.1
Arv1Unknown, score: 0.05
Rnf122Unknown, score: 0.24
Klhdc9Unknown, score: 0.33
Tmcc2Unknown, score: 0.37
Xrcc6bp1Unknown, score: 0.21
Gkn3Unknown, score: 0.2
Ubac2Unknown, score: 0.42
Cd177Unknown, score: 0.19
Rasl11aUnknown, score: 0.17
Disp1Unknown, score: 0.12
Abhd13Unknown, score: 0.55
Zfp467Unknown, score: 0.29
Pygo2Unknown, score: 0.08
Cdkal1Unknown, score: 0.16
Hint2Unknown, score: 0.39
1190005I06RikUnknown, score: 0.57
Dnaic1Unknown, score: 0.23
Ubap2Unknown, score: 0.24
Ptcd2Unknown, score: 0.3
Smim11Unknown, score: 0.43
Chmp2bUnknown, score: 0.2
Tmco1Unknown, score: 0.3
Fam216aUnknown, score: 0.09
Srrm4Unknown, score: 0.07
Paqr6Unknown, score: 0.06
Phkg2Unknown, score: 0.21
NgdnUnknown, score: 0.1
Cdan1Unknown, score: 0.52
Eif1bUnknown, score: 0.22
Tatdn3Unknown, score: 0.19
Med27Unknown, score: 0.29
Wdr53Unknown, score: 0.06
Ssu72Unknown, score: 0.31
Cab39lUnknown, score: 0.6
Thap7Unknown, score: 0.11
Anapc13Unknown, score: 0.33
Spcs1Unknown, score: 0.12
Zfp707Unknown, score: 0.27
Slc30a5Unknown, score: 0.3
Pycr2Unknown, score: 0.04
1810013L24RikUnknown, score: 0.23
FuomUnknown, score: 0.46
1810011O10RikUnknown, score: 0.13
1810011H11RikUnknown, score: 0.03
Psmd11Unknown, score: 0.23
Zc3h15Unknown, score: 0.07
Zcchc9Unknown, score: 0.03
Oxa1lUnknown, score: 0.11
Vps26bUnknown, score: 0.69
Trim15Unknown, score: 0.29
Stoml1Unknown, score: 0.22
Fam58bUnknown, score: 0.08
Alkbh3Unknown, score: 0.3
Ubr4Unknown, score: 0.33
1810022K09RikUnknown, score: 0.02
Tusc1Unknown, score: 0.27
Vstm5Unknown, score: 0.27
Cd209fUnknown, score: 0.06
GsdmdUnknown, score: 0.14
Snx4Unknown, score: 0.06
1810030O07RikUnknown, score: 0.43
Comtd1Unknown, score: 0.05
Rhebl1Unknown, score: 0.29
ManbalUnknown, score: 0.16
Sec31aUnknown, score: 0.16
Cd209bUnknown, score: 0.4
Faim3Unknown, score: 0.22
Snx5Unknown, score: 0.22
Tmem110Unknown, score: 0.66
Dyrk2Unknown, score: 0.12
Dtwd1Unknown, score: 0.48
Kmt2eUnknown, score: 0.17
Mcemp1Unknown, score: 0.16
Pdia2Unknown, score: 0.23
Dhx16Unknown, score: 0.01
Tmem121Unknown, score: 0.16
Ccdc23Unknown, score: 0.18
Ddah1Unknown, score: 0.06
CarkdUnknown, score: 0.08
Zfp746Unknown, score: 0.12
Qrich1Unknown, score: 0.16
Gtpbp4Unknown, score: 0.19
Polr2dUnknown, score: 0.47
Hspb2Unknown, score: 0.04
Zfp397Unknown, score: 0.08
Elf2Unknown, score: 0.21
3300002I08RikUnknown, score: 0.17
Rhobtb1Unknown, score: 0.25
Asb9Unknown, score: 0.1
Pxt1Unknown, score: 0.17
Slc16a13Unknown, score: 0.51
PacrgUnknown, score: 0.44
1700007K13RikUnknown, score: 0.36
Slc38a4Unknown, score: 0.05
Glrx2Unknown, score: 0.03
Smco2Unknown, score: 0.16
Mocs3Unknown, score: 0.2
C8gUnknown, score: 0.17
Hist1h4hUnknown, score: 0.19
Cdhr4Unknown, score: 0.21
1700025G04RikUnknown, score: 0.02
Dennd6bUnknown, score: 0.02
1700023F06RikUnknown, score: 0.11
Tmem45a2Unknown, score: 0.06
Tmem127Unknown, score: 0.41
1700029J07RikUnknown, score: 0.17
EsamUnknown, score: 0.07
1700030J22RikUnknown, score: 0.37
Avpi1Unknown, score: 0.45
Ten1Unknown, score: 0.39
Hemk1Unknown, score: 0.19
Dnase1l1Unknown, score: 0.01
Antxr1Unknown, score: 0.16
Trnp1Unknown, score: 0.34
Nkpd1Unknown, score: 0.56
2310009B15RikUnknown, score: 0.18
Bst2Unknown, score: 0.15
2310022B05RikUnknown, score: 0.2
Cdk13Unknown, score: 0.09
Vkorc1l1Unknown, score: 0.65
CmblUnknown, score: 0.42
Smco1Unknown, score: 0.07
Fastkd3Unknown, score: 0.27
RhouUnknown, score: 0.16
Tnfsf13Unknown, score: 0.19
Hfe2Unknown, score: 0.07
Gpx8Unknown, score: 0.35
Afg3l2Unknown, score: 0.43
MtfmtUnknown, score: 0.6
Sec24dUnknown, score: 0.37
Pitrm1Unknown, score: 0.39
Arhgef12Unknown, score: 0.1
Fam83gUnknown, score: 0.32
MlipUnknown, score: 0.15
Cd164l2Unknown, score: 0.11
Tmbim1Unknown, score: 0.33
2310061I04RikUnknown, score: 0.16
Ddx51Unknown, score: 0.2
Ccdc115Unknown, score: 0.24
Txndc15Unknown, score: 0.47
PxdnUnknown, score: 0.28
Aarsd1Unknown, score: 0.03
Tatdn1Unknown, score: 0.11
Ndufaf1Unknown, score: 0.03
IqcgUnknown, score: 0.43
RpainUnknown, score: 0.4
Usp46Unknown, score: 0.58
Dph5Unknown, score: 0.33
Tm2d2Unknown, score: 0.27
Casz1Unknown, score: 0.25
Pold4Unknown, score: 0.02
Zswim7Unknown, score: 0.51
Zfp511Unknown, score: 0.11
1600015I10RikUnknown, score: 0.3
1600002K03RikUnknown, score: 0.11
Bdh2Unknown, score: 0.19
Med6Unknown, score: 0.41
Cox11Unknown, score: 0.61
Tmem147Unknown, score: 0.17
Slc39a11Unknown, score: 0.26
Clec4b1Unknown, score: 0.16
Krtcap3Unknown, score: 0.07
Fyttd1Unknown, score: 0.03
Polr2fUnknown, score: 0.4
Wnk4Unknown, score: 0.16
Eif1adUnknown, score: 0.22
Polr3glUnknown, score: 0.17
Ppp1r35Unknown, score: 0.57
Ndufa11Unknown, score: 0.24
Thap3Unknown, score: 0.17
AunipUnknown, score: 0.44
2010107G23RikUnknown, score: 0.13
Mrto4Unknown, score: 0.03
Slc25a32Unknown, score: 0.25
Nabp2Unknown, score: 0.24
AgkUnknown, score: 0.11
Scrn1Unknown, score: 0.09
2810021J22RikUnknown, score: 0.3
Cdc16Unknown, score: 0.43
Mettl18Unknown, score: 0.07
Tmem30aUnknown, score: 0.21
Chn2Unknown, score: 0.23
Ace2Unknown, score: 0.28
Cep85Unknown, score: 0.18
Mcm10Unknown, score: 0.07
Acot7Unknown, score: 0.16
Tspo2Unknown, score: 0.13
Dopey2Unknown, score: 0.44
Cmtm8Unknown, score: 0.11
Ccdc89Unknown, score: 0.36
Nol7Unknown, score: 0.13
2210404O09RikUnknown, score: 0.21
Lysmd2Unknown, score: 0.32
MetrnUnknown, score: 0.4
Cyp4f16Unknown, score: 0.17
Znhit1Unknown, score: 0.07
Ifi35Unknown, score: 0.22
Odf3bUnknown, score: 0.07
SrrdUnknown, score: 0.02
Mllt3Unknown, score: 0.56
Vps36Unknown, score: 0.82
Abhd17cUnknown, score: 0.08
Fam162aUnknown, score: 0.09
Cd209gUnknown, score: 0.25
Taco1Unknown, score: 0.06
Med23Unknown, score: 0.13
Zfp619Unknown, score: 0.19
Rnf168Unknown, score: 0.25
Gtf3c5Unknown, score: 0.17
Psmd1Unknown, score: 0.39
Dazap1Unknown, score: 0.16
Chp2Unknown, score: 0.28
Afap1Unknown, score: 0.3
Rnf126Unknown, score: 0.37
Gcc2Unknown, score: 0.76
FuzUnknown, score: 0.22
Plscr3Unknown, score: 0.64
CactinUnknown, score: 0.14
Hdac8Unknown, score: 0.21
Reep6Unknown, score: 0.06
IydUnknown, score: 0.15
Copb1Unknown, score: 0.11
Kcnip1Unknown, score: 0.02
Steap1Unknown, score: 0.48
Lman1Unknown, score: 0.08
Actl10Unknown, score: 0.09
Fbln7Unknown, score: 0.05
Gpatch2lUnknown, score: 0.08
Ica1lUnknown, score: 0.37
Kctd2Unknown, score: 0.2
Cox10Unknown, score: 0.02
Asb12Unknown, score: 0.13
Tmem70Unknown, score: 0.07
Csnk1g3Unknown, score: 0.17
Tekt5Unknown, score: 0.4
Polr3bUnknown, score: 0.05
Rufy2Unknown, score: 0.2
DraxinUnknown, score: 0.65
Taf15Unknown, score: 0.29
Unc13dUnknown, score: 0.08
CenplUnknown, score: 0.33
2610318N02RikUnknown, score: 0.23
Ckap2lUnknown, score: 0.37
Atad2Unknown, score: 0.04
MipepUnknown, score: 0.23
Slc35d2Unknown, score: 0.02
Atp6ap2Unknown, score: 0.14
Rnf167Unknown, score: 0.18
Btf3l4Unknown, score: 0.42
Zdhhc2Unknown, score: 0.12
Tln2Unknown, score: 0.08
Tmtc4Unknown, score: 0.37
Lrrc56Unknown, score: 0.04
Cpne3Unknown, score: 0.13
Ipo5Unknown, score: 0.1
Tbccd1Unknown, score: 0.23
CpmUnknown, score: 0.45
Zc3h11aUnknown, score: 0.37
Filip1Unknown, score: 0.19
EcdUnknown, score: 0.26
Fbxo33Unknown, score: 0.02
Tmem230Unknown, score: 0.24
Ube2v2Unknown, score: 0.58
Fam189a1Unknown, score: 0.05
Tmem144Unknown, score: 0.05
Sik3Unknown, score: 0.05
Fam175aUnknown, score: 0.01
3830403N18RikUnknown, score: 0.23
Nup205Unknown, score: 0.03
Gpr137cUnknown, score: 0.16
MedagUnknown, score: 0.05
Hmha1Unknown, score: 0.31
Angptl6Unknown, score: 0.08
Tspan2Unknown, score: 0.04
Gpr173Unknown, score: 0.18
Dennd1cUnknown, score: 0.17
Ubr5Unknown, score: 0.15
Hars2Unknown, score: 0.13
Arrdc2Unknown, score: 0.07
Hmgxb4Unknown, score: 0.49
Trak2Unknown, score: 0.39
P2ry12Unknown, score: 0.27
Cnbd2Unknown, score: 0.15
Armc3Unknown, score: 0.01
Ints10Unknown, score: 0.01
SpdyaUnknown, score: 0.17
70893Unknown, score: 0.03
Fam71dUnknown, score: 0.25
Spata1Unknown, score: 0.26
4931406C07RikUnknown, score: 0.12
4931429I11RikUnknown, score: 0.4
Naa40Unknown, score: 0.07
D7Ertd443eUnknown, score: 0.01
Spats1Unknown, score: 0.23
Pcgf6Unknown, score: 0.3
4933405L10RikUnknown, score: 0.39
Cdkl1Unknown, score: 0.12
Gpr39Unknown, score: 0.07
Stx18Unknown, score: 0.11
Sh2d6Unknown, score: 0.07
Zfp689Unknown, score: 0.03
Rfx4Unknown, score: 0.25
Scara5Unknown, score: 0.07
Golga7bUnknown, score: 0.19
Mier1Unknown, score: 0.11
NipblUnknown, score: 0.06
Fbxo24Unknown, score: 0.02
Clec12bUnknown, score: 0.09
Otud1Unknown, score: 0.65
Nudt4Unknown, score: 0.07
Cage1Unknown, score: 0.11
Gpr15Unknown, score: 0.02
Dlg5Unknown, score: 0.52
Sdhaf3Unknown, score: 0.1
Spata24Unknown, score: 0.21
Noxred1Unknown, score: 0.02
Ccdc57Unknown, score: 0.02
Slc29a3Unknown, score: 0.25
Mfap3lUnknown, score: 0.25
Tbc1d9Unknown, score: 0.1
Rassf8Unknown, score: 0.01
Tchhl1Unknown, score: 0.01
Rcbtb1Unknown, score: 0.2
Aifm2Unknown, score: 0.1
Pdss2Unknown, score: 0.06
Arid5bUnknown, score: 0.07
Prr16Unknown, score: 0.09
Foxn3Unknown, score: 0.14
AmtnUnknown, score: 0.02
Arhgap21Unknown, score: 0.02
Flrt3Unknown, score: 0.18
WrbUnknown, score: 0.02
Ankrd40Unknown, score: 0.18
BcorUnknown, score: 0.31
Ptk7Unknown, score: 0.64
Usp19Unknown, score: 0.32
9030624J02RikUnknown, score: 0.32
Pds5aUnknown, score: 0.06
Ggt6Unknown, score: 0.46
Fam217bUnknown, score: 0.58
AfmidUnknown, score: 0.19
Izumo4Unknown, score: 0.15
Mcm9Unknown, score: 0.46
9130008F23RikUnknown, score: 0.04
Zfp251Unknown, score: 0.33
Myo1eUnknown, score: 0.02
Snx20Unknown, score: 0.19
TraddUnknown, score: 0.06
4930506M07RikUnknown, score: 0.2
Mettl7bUnknown, score: 0.09
Acy3Unknown, score: 0.13
Atp5hUnknown, score: 0.04
Rbm43Unknown, score: 0.14
Galnt14Unknown, score: 0.2
Esm1Unknown, score: 0.08
Colec11Unknown, score: 0.47
Arhgef3Unknown, score: 0.06
Slc46a3Unknown, score: 0.3
Lrrcc1Unknown, score: 0.05
Mus81Unknown, score: 0.03
Cdc40Unknown, score: 0.08
Dhx35Unknown, score: 0.36
Telo2Unknown, score: 0.03
Fam13cUnknown, score: 0.25
CicUnknown, score: 0.43
Dhx34Unknown, score: 0.08
Aox3Unknown, score: 0.24
Smug1Unknown, score: 0.31
Stk11ipUnknown, score: 0.61
Rgs12Unknown, score: 0.05
Vps11Unknown, score: 0.49
Mamdc2Unknown, score: 0.11
Pvrl4Unknown, score: 0.15
Ulk3Unknown, score: 0.37
Cul2Unknown, score: 0.05
Rgl3Unknown, score: 0.14
DhdhUnknown, score: 0.12
Amdhd1Unknown, score: 0.49
C2cd2lUnknown, score: 0.07
Klhdc3Unknown, score: 0.33
Raver1Unknown, score: 0.49
Tysnd1Unknown, score: 0.11
Bbs10Unknown, score: 0.03
Ap2b1Unknown, score: 0.24
1300017J02RikUnknown, score: 0.04
Klhl5Unknown, score: 0.06
March8Unknown, score: 0.49
Isyna1Unknown, score: 0.11
PdgfdUnknown, score: 0.12
Pitpnc1Unknown, score: 0.32
Plekhf2Unknown, score: 0.13
Tars2Unknown, score: 0.31
Rnf180Unknown, score: 0.09
Kif23Unknown, score: 0.34
Wdr34Unknown, score: 0.13
CslUnknown, score: 0.16
Shcbp1lUnknown, score: 0.2
Phf7Unknown, score: 0.39
Osgin1Unknown, score: 0.28
Tekt4Unknown, score: 0.13
1700008I05RikUnknown, score: 0.06
Pdia6Unknown, score: 0.07
Wfdc3Unknown, score: 0.21
Efhc1Unknown, score: 0.02
Fam83dUnknown, score: 0.29
ApmapUnknown, score: 0.4
Coq2Unknown, score: 0.53
2310002L09RikUnknown, score: 0.07
Ppm1jUnknown, score: 0.41
Noxo1Unknown, score: 0.47
Apol9bUnknown, score: 0.11
Tmem106bUnknown, score: 0.17
Fam219aUnknown, score: 0.27
Cand1Unknown, score: 0.24
Cldn23Unknown, score: 0.29
Ppapdc1bUnknown, score: 0.48
Bdh1Unknown, score: 0.05
Jsrp1Unknown, score: 0.05
Tmem79Unknown, score: 0.06
Dus4lUnknown, score: 0.15
EpgnUnknown, score: 0.27
Tube1Unknown, score: 0.06
Ephx3Unknown, score: 0.19
Car13Unknown, score: 0.18
Apol6Unknown, score: 0.3
Tom1l1Unknown, score: 0.03
Endod1Unknown, score: 0.38
2310067B10RikUnknown, score: 0.1
Cers5Unknown, score: 0.17
Gpc2Unknown, score: 0.34
2410016O06RikUnknown, score: 0.26
Rnf135Unknown, score: 0.13
Cpsf3lUnknown, score: 0.37
Myh14Unknown, score: 0.27
Nkiras2Unknown, score: 0.16
Zbed5Unknown, score: 0.68
Zswim1Unknown, score: 0.07
DnmbpUnknown, score: 0.31
Snx10Unknown, score: 0.54
Tmco6Unknown, score: 0.6
Sars2Unknown, score: 0.21
Acad10Unknown, score: 0.34
Rpusd4Unknown, score: 0.02
Ddx54Unknown, score: 0.23
Ercc8Unknown, score: 0.15
Erv3Unknown, score: 0.18
Fbxo22Unknown, score: 0.08
Lmntd2Unknown, score: 0.28
Fndc3bUnknown, score: 0.24
Zfyve19Unknown, score: 0.36
1600002H07RikUnknown, score: 0.06
Zfp654Unknown, score: 0.07
Slc35f2Unknown, score: 0.06
Cyb561d1Unknown, score: 0.31
TrmuUnknown, score: 0.17
Cdhr5Unknown, score: 0.39
Alkbh4Unknown, score: 0.34
Cotl1Unknown, score: 0.71
2010001E11RikUnknown, score: 0.13
Tnfrsf13cUnknown, score: 0.04
Kdelc1Unknown, score: 0.36
Tmub2Unknown, score: 0.27
Cyp4f18Unknown, score: 0.37
Slc38a10Unknown, score: 0.25
Phf10Unknown, score: 0.5
Rap2cUnknown, score: 0.01
Anks4bUnknown, score: 0.4
Mzt2Unknown, score: 0.07
Mettl10Unknown, score: 0.26
Tmem68Unknown, score: 0.02
AplfUnknown, score: 0.36
Ddhd2Unknown, score: 0.02
Zbed3Unknown, score: 0.16
Trub1Unknown, score: 0.28
Pygo1Unknown, score: 0.34
Wdsub1Unknown, score: 0.05
Cep89Unknown, score: 0.02
AdpgkUnknown, score: 0.1
Slc37a3Unknown, score: 0.09
Wdfy3Unknown, score: 0.07
TdrpUnknown, score: 0.17
Thumpd2Unknown, score: 0.13
Aifm3Unknown, score: 0.27
Trim29Unknown, score: 0.22
Fbxl2Unknown, score: 0.04
Snx6Unknown, score: 0.15
Klhl35Unknown, score: 0.21
Scaf11Unknown, score: 0.01
Fbxl20Unknown, score: 0.21
Skiv2l2Unknown, score: 0.05
Otud6bUnknown, score: 0.54
Eml2Unknown, score: 0.07
1700001P01RikUnknown, score: 0.02
Zfp558Unknown, score: 0.08
1600014C10RikUnknown, score: 0.03
Lrrc8eUnknown, score: 0.1
CdaUnknown, score: 0.2
Smim24Unknown, score: 0.3
Fam186aUnknown, score: 0.23
Oraov1Unknown, score: 0.45
Plekhf1Unknown, score: 0.12
Lsm11Unknown, score: 0.16
Nkd2Unknown, score: 0.36
Rusc1Unknown, score: 0.07
B3gnt3Unknown, score: 0.19
1810041L15RikUnknown, score: 0.24
Zfp777Unknown, score: 0.1
2510002D24RikUnknown, score: 0.11
Nkg7Unknown, score: 0.16
FrylUnknown, score: 0.23
Cyth4Unknown, score: 0.01
2510003E04RikUnknown, score: 0.17
Xpo5Unknown, score: 0.42
Plxdc1Unknown, score: 0.4
Vps9d1Unknown, score: 0.65
Klhl40Unknown, score: 0.29
Wdr89Unknown, score: 0.05
Elp6Unknown, score: 0.14
Amer1Unknown, score: 0.07
Dusp3Unknown, score: 0.38
Zc2hc1cUnknown, score: 0.28
Ptar1Unknown, score: 0.12
Ttc4Unknown, score: 0.26
Ces2gUnknown, score: 0.2
PscaUnknown, score: 0.08
Cdkn3Unknown, score: 0.36
Tmem175Unknown, score: 0.02
Rbm12b1Unknown, score: 0.04
LrpprcUnknown, score: 0.08
Spink5Unknown, score: 0.11
Prr5lUnknown, score: 0.21
Plcd3Unknown, score: 0.05
Slc16a10Unknown, score: 0.12
Hsdl2Unknown, score: 0.24
Tspyl4Unknown, score: 0.2
Rnf219Unknown, score: 0.63
Cwf19l1Unknown, score: 0.29
Taf4bUnknown, score: 0.24
Tmem55aUnknown, score: 0.38
Atxn7l2Unknown, score: 0.77
Pgam5Unknown, score: 0.47
Mvb12bUnknown, score: 0.08
Reep4Unknown, score: 0.16
Naalad2Unknown, score: 0.24
Pan3Unknown, score: 0.19
Ppme1Unknown, score: 0.08
Pdia5Unknown, score: 0.05
Pih1d2Unknown, score: 0.23
Pdzd11Unknown, score: 0.27
Hspa12bUnknown, score: 0.03
Ints8Unknown, score: 0.38
2700094K13RikUnknown, score: 0.22
2700097O09RikUnknown, score: 0.22
Dis3Unknown, score: 0.25
Dnajc6Unknown, score: 0.6
Grrp1Unknown, score: 0.21
HnrnpllUnknown, score: 0.09
Zcchc12Unknown, score: 0.2
Lime1Unknown, score: 0.03
Zfp618Unknown, score: 0.4
C1qtnf6Unknown, score: 0.12
Zfp248Unknown, score: 0.07
Zfp74Unknown, score: 0.22
TbccUnknown, score: 0.42
Tmem161bUnknown, score: 0.06
Ttc39cUnknown, score: 0.16
Hdhd3Unknown, score: 0.13
Fam117bUnknown, score: 0.24
Tmem135Unknown, score: 0.08
Rint1Unknown, score: 0.07
Neil1Unknown, score: 0.29
Sass6Unknown, score: 0.08
Ttc19Unknown, score: 0.13
Pard3bUnknown, score: 0.3
Mon1aUnknown, score: 0.07
Ubash3bUnknown, score: 0.39
Prdm4Unknown, score: 0.44
Ccdc176Unknown, score: 0.32
Zdhhc4Unknown, score: 0.22
Ccdc94Unknown, score: 0.15
Setd5Unknown, score: 0.37
March1Unknown, score: 0.36
HepacamUnknown, score: 0.23
Ppp2r2bUnknown, score: 0.41
Swi5Unknown, score: 0.21
Hspb11Unknown, score: 0.17
Lrrc47Unknown, score: 0.09
PhykplUnknown, score: 0.23
Zfp493Unknown, score: 0.02
Top1mtUnknown, score: 0.15
Slc17a7Unknown, score: 0.23
PrkrirUnknown, score: 0.13
Insig2Unknown, score: 0.51
Gpr22Unknown, score: 0.13
Emc7Unknown, score: 0.08
Glrx5Unknown, score: 0.32
Camk2n2Unknown, score: 0.32
3110007F17RikUnknown, score: 0.35
Ppp1r16aUnknown, score: 0.33
Fut11Unknown, score: 0.15
Prr36Unknown, score: 0.41
PmpcbUnknown, score: 0.21
Slc25a42Unknown, score: 0.08
Slc22a23Unknown, score: 0.02
3110009E18RikUnknown, score: 0.1
AbraclUnknown, score: 0.17
Slc25a16Unknown, score: 0.19
Larp1Unknown, score: 0.3
Otud3Unknown, score: 0.53
Tm7sf2Unknown, score: 0.1
Pcdh18Unknown, score: 0.09
WaslUnknown, score: 0.21
Nfatc4Unknown, score: 0.39
3110043O21RikUnknown, score: 0.08
3110082I17RikUnknown, score: 0.76
Sppl2bUnknown, score: 0.07
Zfp942Unknown, score: 0.15
Atat1Unknown, score: 0.02
Prl8a1Unknown, score: 0.41
Ccdc18Unknown, score: 0.15
1700040L02RikUnknown, score: 0.18
Ccdc132Unknown, score: 0.19
Rhobtb3Unknown, score: 0.07
Lrrc69Unknown, score: 0.26
Clhc1Unknown, score: 0.38
Itpripl1Unknown, score: 0.24
NptxrUnknown, score: 0.24
Col20a1Unknown, score: 0.11
Phospho2Unknown, score: 0.15
Hbp1Unknown, score: 0.1
Msl3l2Unknown, score: 0.1
CcsapUnknown, score: 0.08
1700049G17RikUnknown, score: 0.02
Wdr13Unknown, score: 0.06
Zfp763Unknown, score: 0.14
1700067K01RikUnknown, score: 0.15
Rnf38Unknown, score: 0.35
Spata18Unknown, score: 0.07
CamkmtUnknown, score: 0.26
Capn9Unknown, score: 0.06
Ms4a6cUnknown, score: 0.38
Thoc3Unknown, score: 0.02
Sult6b1Unknown, score: 0.37
Trmt11Unknown, score: 0.05
Atg16l2Unknown, score: 0.08
Ndufaf7Unknown, score: 0.2
Ppp2r1bUnknown, score: 0.22
Gucy2gUnknown, score: 0.42
Tubb2bUnknown, score: 0.25
Mvb12aUnknown, score: 0.56
DmknUnknown, score: 0.04
Cst6Unknown, score: 0.04
1110017D15RikUnknown, score: 0.28
MceeUnknown, score: 0.34
Fcf1Unknown, score: 0.43
Haus7Unknown, score: 0.15
Cby1Unknown, score: 0.51
Man2c1Unknown, score: 0.35
1110034G24RikUnknown, score: 0.12
Gadl1Unknown, score: 0.53
WhrnUnknown, score: 0.01
Fam83eUnknown, score: 0.48
Ppp1r21Unknown, score: 0.22
Eif3kUnknown, score: 0.43
Atp6v1dUnknown, score: 0.16
Ifitm5Unknown, score: 0.54
Slc35b2Unknown, score: 0.05
Ankrd42Unknown, score: 0.4
D3Ertd751eUnknown, score: 0.13
Fam161aUnknown, score: 0.27
Tmem202Unknown, score: 0.04
Arhgap18Unknown, score: 0.2
Lyrm1Unknown, score: 0.26
Dnm1lUnknown, score: 0.05
Btbd11Unknown, score: 0.34
Rftn2Unknown, score: 0.11
Cpne4Unknown, score: 0.09
Glyr1Unknown, score: 0.14
Rd3Unknown, score: 0.18
Rin2Unknown, score: 0.05
Sdr42e1Unknown, score: 0.33
Nol9Unknown, score: 0.13
DdiasUnknown, score: 0.06
Pex26Unknown, score: 0.29
Ttf2Unknown, score: 0.23
Grip1Unknown, score: 0.15
Lmntd1Unknown, score: 0.09
Cep350Unknown, score: 0.08
Paqr5Unknown, score: 0.46
NplUnknown, score: 0.29
Hvcn1Unknown, score: 0.22
0610037L13RikUnknown, score: 0.07
NeblUnknown, score: 0.37
Abcb6Unknown, score: 0.38
Gga2Unknown, score: 0.05
ParnUnknown, score: 0.32
CrotUnknown, score: 0.29
Pi16Unknown, score: 0.03
Zfp263Unknown, score: 0.24
AcoxlUnknown, score: 0.21
Tmem43Unknown, score: 0.31
Foxp4Unknown, score: 0.05
Armc8Unknown, score: 0.43
Syvn1Unknown, score: 0.61
DmgdhUnknown, score: 0.13
Sash3Unknown, score: 0.07
Rnf6Unknown, score: 0.04
Cyp2s1Unknown, score: 0.58
Sec14l1Unknown, score: 0.05
Nuak2Unknown, score: 0.66
Tm9sf1Unknown, score: 0.8
Opa1Unknown, score: 0.1
Robo4Unknown, score: 0.02
EhhadhUnknown, score: 0.33
Zfp946Unknown, score: 0.13
Slc35f5Unknown, score: 0.07
Stra6lUnknown, score: 0.05
Uba7Unknown, score: 0.27
UnklUnknown, score: 0.33
Acot12Unknown, score: 0.24
Cmtr1Unknown, score: 0.15
Acbd5Unknown, score: 0.05
Fbxl22Unknown, score: 0.02
Nudt9Unknown, score: 0.19
Zdhhc16Unknown, score: 0.27
Gtsf1Unknown, score: 0.21
Muc5bUnknown, score: 0.02
Gpcpd1Unknown, score: 0.39
Perm1Unknown, score: 0.21
Gbe1Unknown, score: 0.37
Ccdc3Unknown, score: 0.53
Katnb1Unknown, score: 0.28
Phactr3Unknown, score: 0.24
Rnd3Unknown, score: 0.09
Gtf2e1Unknown, score: 0.56
Cep97Unknown, score: 0.22
Fblim1Unknown, score: 0.04
Eif4enif1Unknown, score: 0.06
Xpo6Unknown, score: 0.04
Acsl3Unknown, score: 0.22
Sipa1l3Unknown, score: 0.23
Rbm26Unknown, score: 0.03
Mterf2Unknown, score: 0.02
IqceUnknown, score: 0.63
Slx4ipUnknown, score: 0.24
CtbsUnknown, score: 0.46
Lrrc2Unknown, score: 0.6
Klrg2Unknown, score: 0.21
CyldUnknown, score: 0.29
AvenUnknown, score: 0.61
Chic2Unknown, score: 0.19
Spatc1Unknown, score: 0.04
Prss46Unknown, score: 0.02
1700092M07RikUnknown, score: 0.14
Osbp2Unknown, score: 0.07
Isca2Unknown, score: 0.27
HopxUnknown, score: 0.08
Wdr33Unknown, score: 0.24
HnrnprUnknown, score: 0.35
Dnajc14Unknown, score: 0.31
Ranbp10Unknown, score: 0.35
Palm3Unknown, score: 0.08
Ahcyl2Unknown, score: 0.17
Lrrtm1Unknown, score: 0.5
Tldc1Unknown, score: 0.15
Smchd1Unknown, score: 0.39
Lonrf3Unknown, score: 0.25
RptorUnknown, score: 0.52
Clec16aUnknown, score: 0.14
Gcc1Unknown, score: 0.1
Myo18bUnknown, score: 0.24
Hsf2bpUnknown, score: 0.05
Rmi1Unknown, score: 0.03
4932438H23RikUnknown, score: 0.05
Dpp8Unknown, score: 0.08
Map10Unknown, score: 0.04
Ppapdc2Unknown, score: 0.2
Gle1Unknown, score: 0.08
Polr3cUnknown, score: 0.27
Tmc5Unknown, score: 0.11
4933402D24RikUnknown, score: 0.08
Eaf1Unknown, score: 0.19
Clvs1Unknown, score: 0.13
CmipUnknown, score: 0.23
Sgms2Unknown, score: 0.43
P4htmUnknown, score: 0.15
Slc9b1Unknown, score: 0.12
Arl13aUnknown, score: 0.06
Pank2Unknown, score: 0.33
74463Unknown, score: 0.38
Cep72Unknown, score: 0.2
4933427D14RikUnknown, score: 0.12
Snx29Unknown, score: 0.37
Snx11Unknown, score: 0.03
Batf2Unknown, score: 0.1
Lrrc71Unknown, score: 0.1
Lrrc15Unknown, score: 0.2
Tnks2Unknown, score: 0.15
SostUnknown, score: 0.29
Cyp2j9Unknown, score: 0.03
Morc2aUnknown, score: 0.05
8430419L09RikUnknown, score: 0.18
Gzf1Unknown, score: 0.07
Gsdmc4Unknown, score: 0.04
Mau2Unknown, score: 0.24
Pck2Unknown, score: 0.02
Nipal3Unknown, score: 0.14
Gvin1Unknown, score: 0.22
Elovl7Unknown, score: 0.05
MlklUnknown, score: 0.11
Zkscan1Unknown, score: 0.08
Pyroxd2Unknown, score: 0.35
Sppl3Unknown, score: 0.38
Kbtbd12Unknown, score: 0.51
Abca12Unknown, score: 0.18
Cd200r3Unknown, score: 0.25
Scpep1Unknown, score: 0.03
ShpkUnknown, score: 0.15
Fam46cUnknown, score: 0.29
S100pbpUnknown, score: 0.12
Ptchd3Unknown, score: 0.17
Wdr35Unknown, score: 0.09
Tdrd9Unknown, score: 0.1
SetmarUnknown, score: 0.33
C2cd5Unknown, score: 0.24
Slamf8Unknown, score: 0.05
Dhcr24Unknown, score: 0.17
Rab3il1Unknown, score: 0.59
Mxra8Unknown, score: 0.19
Naa60Unknown, score: 0.62
Klc4Unknown, score: 0.21
Yipf2Unknown, score: 0.25
Pik3cbUnknown, score: 0.66
HhatlUnknown, score: 0.46
Atp13a2Unknown, score: 0.09
Rrp7aUnknown, score: 0.04
Usp38Unknown, score: 0.04
Ccdc181Unknown, score: 0.04
Kbtbd11Unknown, score: 0.47
Slc35f6Unknown, score: 0.06
Rab11fip2Unknown, score: 0.15
Fam63aUnknown, score: 0.32
Sf3a3Unknown, score: 0.39
Zbtb49Unknown, score: 0.1
4930505A04RikUnknown, score: 0.07
Uhrf1bp1lUnknown, score: 0.01
Ube2dnl2Unknown, score: 0.29
Mmd2Unknown, score: 0.08
Rprd2Unknown, score: 0.07
Rasd2Unknown, score: 0.14
Tmem180Unknown, score: 0.12
Ccdc146Unknown, score: 0.25
Tex38Unknown, score: 0.17
MeiobUnknown, score: 0.1
Sv2cUnknown, score: 0.23
Prr3Unknown, score: 0.03
Cep128Unknown, score: 0.76
Dusp18Unknown, score: 0.17
Dpp3Unknown, score: 0.51
Rnf19bUnknown, score: 0.3
Tomm20lUnknown, score: 0.05
Pelp1Unknown, score: 0.11
Slc35f4Unknown, score: 0.22
Prkd3Unknown, score: 0.02
Fgfr1opUnknown, score: 0.04
Arhgap36Unknown, score: 0.61
Slitrk5Unknown, score: 0.27
Secisbp2Unknown, score: 0.08
Tti1Unknown, score: 0.42
Igfbpl1Unknown, score: 0.51
Anapc15Unknown, score: 0.45
1700011I03RikUnknown, score: 0.17
Ascc2Unknown, score: 0.08
1700008F21RikUnknown, score: 0.58
Phpt1Unknown, score: 0.14
1700001C19RikUnknown, score: 0.14
Cfap126Unknown, score: 0.21
OplahUnknown, score: 0.05
1700003F12RikUnknown, score: 0.17
Ttc32Unknown, score: 0.29
EppinUnknown, score: 0.13
Lyrm7Unknown, score: 0.41
FpgtUnknown, score: 0.32
1700019G17RikUnknown, score: 0.12
Paqr9Unknown, score: 0.09
Zc3h14Unknown, score: 0.22
Ep400Unknown, score: 0.04
Dnali1Unknown, score: 0.05
Ccdc101Unknown, score: 0.03
Spata9Unknown, score: 0.27
2310007L24RikUnknown, score: 0.37
FggyUnknown, score: 0.06
Zbtb4Unknown, score: 0.26
Yipf7Unknown, score: 0.09
Dusp9Unknown, score: 0.35
Ndufaf2Unknown, score: 0.12
Pcdh1Unknown, score: 0.26
Calml4Unknown, score: 0.02
Kdm5bUnknown, score: 0.4
Chmp4bUnknown, score: 0.03
GnsUnknown, score: 0.45
Fastkd2Unknown, score: 0.3
Kxd1Unknown, score: 0.1
Metap1Unknown, score: 0.02
Snapc1Unknown, score: 0.13
Spata25Unknown, score: 0.34
Rai14Unknown, score: 0.19
Ssmem1Unknown, score: 0.08
Wdr54Unknown, score: 0.35
Lin37Unknown, score: 0.17
Ccdc64Unknown, score: 0.6
Rasl10aUnknown, score: 0.04
Pik3r4Unknown, score: 0.14
Cldn22Unknown, score: 0.17
Nudt16Unknown, score: 0.29
Fam65aUnknown, score: 0.22
Vsig10lUnknown, score: 0.34
Rilpl1Unknown, score: 0.12
Fam35aUnknown, score: 0.08
Rbm12Unknown, score: 0.09
Cul5Unknown, score: 0.2
Bcl2l12Unknown, score: 0.06
Morc4Unknown, score: 0.39
Sesn3Unknown, score: 0.23
Dcaf17Unknown, score: 0.16
Slx1bUnknown, score: 0.15
DcstampUnknown, score: 0.02
Rab11fip1Unknown, score: 0.14
Ckap5Unknown, score: 0.43
Smurf1Unknown, score: 0.08
IspdUnknown, score: 0.04
Tex26Unknown, score: 0.26
Arl5bUnknown, score: 0.15
Tcam1Unknown, score: 0.17
Dcp1aUnknown, score: 0.12
AgmatUnknown, score: 0.01
Slain2Unknown, score: 0.07
Zc3h18Unknown, score: 0.6
Gon4lUnknown, score: 0.37
Ncapg2Unknown, score: 0.1
Mgea5Unknown, score: 0.16
Jakmip1Unknown, score: 0.09
Rapgef2Unknown, score: 0.09
Ppp1r14cUnknown, score: 0.13
Rsg1Unknown, score: 0.16
Snrnp35Unknown, score: 0.35
Abhd12Unknown, score: 0.14
Stard3nlUnknown, score: 0.08
Agbl3Unknown, score: 0.03
Atp6v0e2Unknown, score: 0.32
0610040J01RikUnknown, score: 0.14
Gstk1Unknown, score: 0.1
Fads1Unknown, score: 0.02
Cyp2d26Unknown, score: 0.21
Tax1bp3Unknown, score: 0.56
Slc18b1Unknown, score: 0.42
Rab1bUnknown, score: 0.09
Cog2Unknown, score: 0.08
TgdsUnknown, score: 0.36
Trmt5Unknown, score: 0.15
Mtif3Unknown, score: 0.39
Zfp773Unknown, score: 0.25
1700012A03RikUnknown, score: 0.06
1700019B03RikUnknown, score: 0.45
1700028K03RikUnknown, score: 0.25
LhppUnknown, score: 0.27
Ppp1r18Unknown, score: 0.18
Prss23Unknown, score: 0.5
Fbxo31Unknown, score: 0.26
Msrb2Unknown, score: 0.12
Smndc1Unknown, score: 0.1
3110002H16RikUnknown, score: 0.55
Glt8d1Unknown, score: 0.06
Ppp1r11Unknown, score: 0.47
Ip6k2Unknown, score: 0.22
Commd9Unknown, score: 0.08
Plet1Unknown, score: 0.26
Trappc9Unknown, score: 0.04
Lsm8Unknown, score: 0.28
Cln6Unknown, score: 0.41
Il34Unknown, score: 0.03
Fam204aUnknown, score: 0.14
Tmem101Unknown, score: 0.08
Ccdc6Unknown, score: 0.16
Fam101bUnknown, score: 0.09
Ift46Unknown, score: 0.1
Faf2Unknown, score: 0.63
Ipo11Unknown, score: 0.06
Dnajc18Unknown, score: 0.05
Wdr38Unknown, score: 0.14
Srxn1Unknown, score: 0.11
Upp2Unknown, score: 0.09
Trim12aUnknown, score: 0.17
Clip3Unknown, score: 0.17
Arfrp1Unknown, score: 0.24
Arpc2Unknown, score: 0.35
Kansl1Unknown, score: 0.07
Ckmt2Unknown, score: 0.03
Efr3aUnknown, score: 0.1
Mospd2Unknown, score: 0.14
AlpiUnknown, score: 0.16
Mtif2Unknown, score: 0.28
Ppfia3Unknown, score: 0.12
Mzt1Unknown, score: 0.14
Snip1Unknown, score: 0.21
2410137M14RikUnknown, score: 0.29
Rpl18aUnknown, score: 0.07
Bri3bpUnknown, score: 0.3
Armc6Unknown, score: 0.05
NubplUnknown, score: 0.29
DtlUnknown, score: 0.42
Ago4Unknown, score: 0.24
Catsper3Unknown, score: 0.07
SpoplUnknown, score: 0.2
Rhbdd1Unknown, score: 0.05
Ccdc116Unknown, score: 0.03
Fam81aUnknown, score: 0.45
Rnft1Unknown, score: 0.16
Mettl15Unknown, score: 0.09
Golga1Unknown, score: 0.32
Jade2Unknown, score: 0.29
Timmdc1Unknown, score: 0.03
Flywch2Unknown, score: 0.11
TsaccUnknown, score: 0.07
Arfip2Unknown, score: 0.67
Ifi27l2aUnknown, score: 0.25
Rbm17Unknown, score: 0.03
Ndufaf6Unknown, score: 0.16
Bcas1Unknown, score: 0.74
2610028H24RikUnknown, score: 0.25
2700049A03RikUnknown, score: 0.2
TicrrUnknown, score: 0.37
Col25a1Unknown, score: 0.22
Tstd3Unknown, score: 0.26
1700109H08RikUnknown, score: 0.06
MrapUnknown, score: 0.05
Arfgap2Unknown, score: 0.04
ArskUnknown, score: 0.11
Arid2Unknown, score: 0.13
Cep83Unknown, score: 0.13
Sun1Unknown, score: 0.23
Tmco4Unknown, score: 0.29
Ints7Unknown, score: 0.04
Ankrd11Unknown, score: 0.5
Ocel1Unknown, score: 0.07
Tanc2Unknown, score: 0.36
Gpbp1l1Unknown, score: 0.18
Klhl2Unknown, score: 0.02
Zfp934Unknown, score: 0.03
CrebrfUnknown, score: 0.49
Ptgr2Unknown, score: 0.05
9430038I01RikUnknown, score: 0.33
Yif1bUnknown, score: 0.33
NkrfUnknown, score: 0.21
Raph1Unknown, score: 0.06
Sec24aUnknown, score: 0.19
Rab35Unknown, score: 0.41
C330018D20RikUnknown, score: 0.38
Heg1Unknown, score: 0.14
Zfp266Unknown, score: 0.52
Mtus2Unknown, score: 0.13
Limch1Unknown, score: 0.02
Vps33aUnknown, score: 0.02
Spns3Unknown, score: 0.33
Myh10Unknown, score: 0.19
Mboat7Unknown, score: 0.38
Rbm12b1Unknown, score: 0.27
H2afvUnknown, score: 0.08
Apex2Unknown, score: 0.03
Smpd4Unknown, score: 0.16
SphkapUnknown, score: 0.05
C330007P06RikUnknown, score: 0.13
Trat1Unknown, score: 0.15
Ehmt1Unknown, score: 0.08
MmabUnknown, score: 0.27
9230104L09RikUnknown, score: 0.14
Rnf170Unknown, score: 0.29
Csrnp3Unknown, score: 0.1
Esco1Unknown, score: 0.13
Lrrc42Unknown, score: 0.18
LbhUnknown, score: 0.48
Yipf6Unknown, score: 0.59
Hook1Unknown, score: 0.08
Nuak1Unknown, score: 0.35
Sbf1Unknown, score: 0.03
Ascc3Unknown, score: 0.19
Mccc2Unknown, score: 0.59
Lcn8Unknown, score: 0.04
Lpar4Unknown, score: 0.08
Nxpe2Unknown, score: 0.42
Ralgps2Unknown, score: 0.07
Lrrc9Unknown, score: 0.25
Zfp687Unknown, score: 0.47
RbsnUnknown, score: 0.15
Hist3h2baUnknown, score: 0.38
Naa38Unknown, score: 0.1
78306Unknown, score: 0.32
Gpr108Unknown, score: 0.18
Cul9Unknown, score: 0.25
Ccdc88bUnknown, score: 0.13
Cdk19Unknown, score: 0.37
Ttyh3Unknown, score: 0.12
2210407C18RikUnknown, score: 0.6
Icam4Unknown, score: 0.37
Nudt17Unknown, score: 0.19
Sapcd1Unknown, score: 0.02
Ntf5Unknown, score: 0.17
Fam131aUnknown, score: 0.16
WibgUnknown, score: 0.1
1700084C01RikUnknown, score: 0.11
Mrpl9Unknown, score: 0.18
Asb8Unknown, score: 0.08
Htra3Unknown, score: 0.17
Srbd1Unknown, score: 0.28
Nrip3Unknown, score: 0.15
Pde6hUnknown, score: 0.37
Zfp449Unknown, score: 0.37
Ncapd3Unknown, score: 0.4
Plekhj1Unknown, score: 0.5
Nol3Unknown, score: 0.13
TroapUnknown, score: 0.24
Galnt15Unknown, score: 0.15
Efcab11Unknown, score: 0.25
Hhipl2Unknown, score: 0.73
Cfap61Unknown, score: 0.21
Enthd2Unknown, score: 0.44
Zc3hav1Unknown, score: 0.03
Brpf1Unknown, score: 0.06
Celf3Unknown, score: 0.12
Usp54Unknown, score: 0.14
Zcchc4Unknown, score: 0.51
Eml4Unknown, score: 0.1
Ak7Unknown, score: 0.07
Stpg1Unknown, score: 0.17
Stxbp5Unknown, score: 0.17
4930562C15RikUnknown, score: 0.04
Tsc22d4Unknown, score: 0.44
Zfp623Unknown, score: 0.3
Wsb1Unknown, score: 0.21
Trmt44Unknown, score: 0.05
Crispld2Unknown, score: 0.16
Cnot10Unknown, score: 0.68
Pus7lUnknown, score: 0.04
1500015O10RikUnknown, score: 0.17
MispUnknown, score: 0.51
Igsf3Unknown, score: 0.19
Asb15Unknown, score: 0.32
Ltn1Unknown, score: 0.05
DlstUnknown, score: 0.06
Srd5a1Unknown, score: 0.36
Gas2l1Unknown, score: 0.4
Polr3hUnknown, score: 0.04
Avl9Unknown, score: 0.16
Fbxo34Unknown, score: 0.16
Popdc3Unknown, score: 0.44
Spsb3Unknown, score: 0.62
Mrps34Unknown, score: 0.36
Nme3Unknown, score: 0.28
Osbpl5Unknown, score: 0.17
Tnfrsf23Unknown, score: 0.18
Hdac9Unknown, score: 0.16
Recql4Unknown, score: 0.12
Wbscr27Unknown, score: 0.17
Cdk5rap3Unknown, score: 0.19
Cttnbp2nlUnknown, score: 0.44
Abtb1Unknown, score: 0.19
Parp9Unknown, score: 0.25
Apobec3Unknown, score: 0.37
Lysmd3Unknown, score: 0.35
Gpr146Unknown, score: 0.23
Rilpl2Unknown, score: 0.08
ZxdcUnknown, score: 0.33
Pofut2Unknown, score: 0.21
Tusc2Unknown, score: 0.11
Herpud2Unknown, score: 0.04
Igsf6Unknown, score: 0.26
MynnUnknown, score: 0.16
Car15Unknown, score: 0.08
BC004004Unknown, score: 0.13
Rnf34Unknown, score: 0.25
Fam20cUnknown, score: 0.55
Klrb1bUnknown, score: 0.2
RhojUnknown, score: 0.05
Fgf20Unknown, score: 0.24
GhdcUnknown, score: 0.03
Dhx58Unknown, score: 0.18
LrbaUnknown, score: 0.57
Slc16a3Unknown, score: 0.14
Kank3Unknown, score: 0.13
Ntng1Unknown, score: 0.48
Hcar2Unknown, score: 0.08
Hspb8Unknown, score: 0.61
Trim2Unknown, score: 0.11
FcrlsUnknown, score: 0.27
Zfhx4Unknown, score: 0.08
Erap1Unknown, score: 0.03
Fgf16Unknown, score: 0.28
Dtx3Unknown, score: 0.12
Kcnip2Unknown, score: 0.08
LactbUnknown, score: 0.71
Gatsl2Unknown, score: 0.38
Gpr84Unknown, score: 0.42
Acox3Unknown, score: 0.05
Pum2Unknown, score: 0.34
Uck2Unknown, score: 0.07
Dusp12Unknown, score: 0.08
Syt13Unknown, score: 0.37
MrgprhUnknown, score: 0.07
Arl4dUnknown, score: 0.09
Ckap2Unknown, score: 0.26
NckipsdUnknown, score: 0.04
Trim23Unknown, score: 0.04
Rnf114Unknown, score: 0.07
Sgpp1Unknown, score: 0.09
Kat5Unknown, score: 0.35
Ankrd17Unknown, score: 0.08
C1qtnf3Unknown, score: 0.15
Sorcs2Unknown, score: 0.53
TnxbUnknown, score: 0.25
Ift122Unknown, score: 0.16
Tlr9Unknown, score: 0.08
Sf3b1Unknown, score: 0.36
Tmem108Unknown, score: 0.09
Rrbp1Unknown, score: 0.11
Sp6Unknown, score: 0.48
Ndst3Unknown, score: 0.19
Gimap3Unknown, score: 0.25
Cstf2tUnknown, score: 0.13
CtnsUnknown, score: 0.05
Il23aUnknown, score: 0.06
Ndel1Unknown, score: 0.03
Nxf2Unknown, score: 0.34
Mov10l1Unknown, score: 0.22
Sacm1lUnknown, score: 0.36
Fstl3Unknown, score: 0.11
Tex13Unknown, score: 0.06
Elovl4Unknown, score: 0.84
Sytl3Unknown, score: 0.17
Cnnm1Unknown, score: 0.03
Bicc1Unknown, score: 0.18
Pde4dipUnknown, score: 0.25
Crispld1Unknown, score: 0.27
Dbr1Unknown, score: 0.39
Slc12a9Unknown, score: 0.2
OtofUnknown, score: 0.31
Wasf1Unknown, score: 0.27
Dpp7Unknown, score: 0.11
Tas1r3Unknown, score: 0.1
CenpqUnknown, score: 0.18
Cep41Unknown, score: 0.12
PhipUnknown, score: 0.11
Enpp5Unknown, score: 0.13
SlmapUnknown, score: 0.08
McamUnknown, score: 0.08
Kremen1Unknown, score: 0.3
PlvapUnknown, score: 0.44
Pi4k2aUnknown, score: 0.1
Sucnr1Unknown, score: 0.3
HampUnknown, score: 0.05
Cd96Unknown, score: 0.33
Rnf123Unknown, score: 0.42
Hes7Unknown, score: 0.11
Cox4i2Unknown, score: 0.14
Tnfrsf25Unknown, score: 0.24
KarsUnknown, score: 0.38
Emc9Unknown, score: 0.36
Sec16bUnknown, score: 0.04
Lmod2Unknown, score: 0.32
Zkscan8Unknown, score: 0.06
Entpd7Unknown, score: 0.28
Klf7Unknown, score: 0.23
Clec2dUnknown, score: 0.3
GpnmbUnknown, score: 0.02
Ice2Unknown, score: 0.14
Pcdhgb6Unknown, score: 0.14
Pcdhgc3Unknown, score: 0.14
Pcdhga8Unknown, score: 0.14
Pcdhgc5Unknown, score: 0.14
Pcdhga3Unknown, score: 0.14
Pcdhga8Unknown, score: 0.14
Pcdhga9Unknown, score: 0.2
Cpn1Unknown, score: 0.14
Pcdhga11Unknown, score: 0.14
Mpv17lUnknown, score: 0.04
Wnt16Unknown, score: 0.12
Aff4Unknown, score: 0.08
Pard3Unknown, score: 0.28
Gprc5dUnknown, score: 0.13
Sirt1Unknown, score: 0.27
Ube2nUnknown, score: 0.07
Nipa2Unknown, score: 0.03
Peli2Unknown, score: 0.19
AmnUnknown, score: 0.1
Uchl4Unknown, score: 0.05
Brwd1Unknown, score: 0.06
Pcdhb2Unknown, score: 0.07
Pcdhb5Unknown, score: 0.24
Pcdhb6Unknown, score: 0.1
Pcdhb7Unknown, score: 0.07
Pcdhb9Unknown, score: 0.1
Pcdhb10Unknown, score: 0.13
Pcdhb22Unknown, score: 0.47
Glp2rUnknown, score: 0.24
Cers1Unknown, score: 0.33
Nkd1Unknown, score: 0.43
Klra8Unknown, score: 0.12
ClmnUnknown, score: 0.28
Tm2d1Unknown, score: 0.03
Mrpl3Unknown, score: 0.09
Mrpl16Unknown, score: 0.24
Mrpl27Unknown, score: 0.24
Mrpl34Unknown, score: 0.06
Mrpl36Unknown, score: 0.25
Trim6Unknown, score: 0.09
Trim9Unknown, score: 0.08
Trim34aUnknown, score: 0.02
Csmd1Unknown, score: 0.26
Dock2Unknown, score: 0.22
Mcoln1Unknown, score: 0.03
NansUnknown, score: 0.08
Strn3Unknown, score: 0.3
Ophn1Unknown, score: 0.03
Adarb2Unknown, score: 0.07
Ddx50Unknown, score: 0.17
Spock2Unknown, score: 0.35
Cnnm2Unknown, score: 0.21
GopcUnknown, score: 0.09
Dgcr8Unknown, score: 0.58
Pi15Unknown, score: 0.11
Cpsf1Unknown, score: 0.08
Ubqln4Unknown, score: 0.09
Fkbp6Unknown, score: 0.02
Arid4bUnknown, score: 0.09
Hecw1Unknown, score: 0.19
Wbscr16Unknown, score: 0.5
Sfxn2Unknown, score: 0.15
Sfxn4Unknown, score: 0.08
Sfxn5Unknown, score: 0.39
PrccUnknown, score: 0.29
Tmem40Unknown, score: 0.04
Hmgn3Unknown, score: 0.22
Prg4Unknown, score: 0.04
Tmem62Unknown, score: 0.47
Ptges2Unknown, score: 0.29
Hist2h3c2Unknown, score: 0.17
C77080Unknown, score: 0.45
Strn4Unknown, score: 0.23
B3gnt9Unknown, score: 0.5
QarsUnknown, score: 0.45
C78339Unknown, score: 0.19
Nlrp4fUnknown, score: 0.01
Hist1h3gUnknown, score: 0.19
Gtf2f1Unknown, score: 0.57
Tmem132aUnknown, score: 0.2
Eif3mUnknown, score: 0.22
Txndc9Unknown, score: 0.16
Stk17bUnknown, score: 0.05
GorabUnknown, score: 0.35
LbrUnknown, score: 0.1
Chst10Unknown, score: 0.17
Atp1a2Unknown, score: 0.12
Trmt1lUnknown, score: 0.17
Rdh10Unknown, score: 0.13
Rab3gap2Unknown, score: 0.41
FcrlaUnknown, score: 0.1
HnrnpfUnknown, score: 0.11
AI182371Unknown, score: 0.19
Ehd4Unknown, score: 0.36
Myl9Unknown, score: 0.11
Clp1Unknown, score: 0.04
Qser1Unknown, score: 0.12
Lpcat4Unknown, score: 0.44
Pomt1Unknown, score: 0.22
Osbpl6Unknown, score: 0.34
Mrps26Unknown, score: 0.09
Cep152Unknown, score: 0.18
Stard7Unknown, score: 0.17
CercamUnknown, score: 0.2
Tm9sf4Unknown, score: 0.1
Dnajc24Unknown, score: 0.13
Cul4aUnknown, score: 0.16
Sall4Unknown, score: 0.1
Abtb2Unknown, score: 0.03
Golga2Unknown, score: 0.06
Wdr47Unknown, score: 0.14
Usp53Unknown, score: 0.12
DpydUnknown, score: 0.02
Adgrl2Unknown, score: 0.04
4933434E20RikUnknown, score: 0.18
Eps8l3Unknown, score: 0.28
Sec24bUnknown, score: 0.14
Kcnc4Unknown, score: 0.07
Tmem56Unknown, score: 0.05
Ifi44Unknown, score: 0.13
TiparpUnknown, score: 0.03
Kdm1aUnknown, score: 0.03
Camta1Unknown, score: 0.37
Kti12Unknown, score: 0.04
Rcc1Unknown, score: 0.33
Zbtb48Unknown, score: 0.06
Pcsk9Unknown, score: 0.12
Tdrd7Unknown, score: 0.05
Pafah2Unknown, score: 0.24
Zmym6Unknown, score: 0.46
AknaUnknown, score: 0.13
Tmem64Unknown, score: 0.21
Adprhl2Unknown, score: 0.41
Gpn2Unknown, score: 0.3
Osbpl9Unknown, score: 0.02
AU040320Unknown, score: 0.25
Smpdl3bUnknown, score: 0.02
Mob3cUnknown, score: 0.12
Rell1Unknown, score: 0.33
Lrrc8cUnknown, score: 0.46
Noc4lUnknown, score: 0.36
N4bp2l1Unknown, score: 0.14
PsphUnknown, score: 0.31
TrrapUnknown, score: 0.17
Gbp6Unknown, score: 0.09
Dcun1d4Unknown, score: 0.02
Usp30Unknown, score: 0.11
Ube3cUnknown, score: 0.33
HscbUnknown, score: 0.37
Chpf2Unknown, score: 0.09
Tyw1Unknown, score: 0.25
Akap9Unknown, score: 0.37
Zfp513Unknown, score: 0.46
Ttll3Unknown, score: 0.59
Tmem168Unknown, score: 0.31
Tra2aUnknown, score: 0.02
EogtUnknown, score: 0.3
Fbxl14Unknown, score: 0.51
Ceacam15Unknown, score: 0.2
Dhx32Unknown, score: 0.21
Phrf1Unknown, score: 0.27
Ric8Unknown, score: 0.14
Inpp5fUnknown, score: 0.11
Plekhg2Unknown, score: 0.43
Hsd3b7Unknown, score: 0.12
Mob2Unknown, score: 0.12
Prkd2Unknown, score: 0.22
WtipUnknown, score: 0.15
Ccp110Unknown, score: 0.28
Eftud1Unknown, score: 0.24
AI467606Unknown, score: 0.2
E430018J23RikUnknown, score: 0.04
Nlrp6Unknown, score: 0.15
Trim68Unknown, score: 0.01
Ano1Unknown, score: 0.14
Ints4Unknown, score: 0.07
Rrp8Unknown, score: 0.45
Unc45aUnknown, score: 0.04
Sf3b3Unknown, score: 0.34
D8Ertd738eUnknown, score: 0.05
Usb1Unknown, score: 0.09
Smim19Unknown, score: 0.24
PhkbUnknown, score: 0.18
Arhgef18Unknown, score: 0.1
Mtus1Unknown, score: 0.54
Fam192aUnknown, score: 0.31
Snx25Unknown, score: 0.76
Taf5lUnknown, score: 0.29
Prmt10Unknown, score: 0.41
Zdhhc7Unknown, score: 0.1
Snapc2Unknown, score: 0.2
Cyp4v3Unknown, score: 0.02
Dcun1d2Unknown, score: 0.19
Clk3Unknown, score: 0.19
HinfpUnknown, score: 0.19
Dennd4aUnknown, score: 0.1
XylbUnknown, score: 0.11
Pls1Unknown, score: 0.07
Ano10Unknown, score: 0.35
Alg9Unknown, score: 0.02
Plekho2Unknown, score: 0.03
Snx19Unknown, score: 0.81
Rpp25Unknown, score: 0.14
Mapkapk3Unknown, score: 0.24
Acad11Unknown, score: 0.19
OafUnknown, score: 0.42
Cd276Unknown, score: 0.12
Bbs4Unknown, score: 0.06
TctaUnknown, score: 0.24
Slc6a8Unknown, score: 0.05
CenpiUnknown, score: 0.06
Slc6a15Unknown, score: 0.12
Pwp1Unknown, score: 0.2
Gstt3Unknown, score: 0.03
ApofUnknown, score: 0.13
Chchd10Unknown, score: 0.31
Fig4Unknown, score: 0.15
BC030307Unknown, score: 0.24
Cep57l1Unknown, score: 0.32
Zfr2Unknown, score: 0.33
Fam26eUnknown, score: 0.39
Mbtd1Unknown, score: 0.6
Xpo1Unknown, score: 0.25
Smg6Unknown, score: 0.19
Tmed4Unknown, score: 0.37
PnpoUnknown, score: 0.36
Tbc1d10aUnknown, score: 0.14
Tubg2Unknown, score: 0.12
Slc25a41Unknown, score: 0.08
Wdr92Unknown, score: 0.31
Maml1Unknown, score: 0.34
Zfp692Unknown, score: 0.05
Inca1Unknown, score: 0.1
Nt5mUnknown, score: 0.05
Dnm3Unknown, score: 0.13
Qsox1Unknown, score: 0.41
Synj1Unknown, score: 0.18
SncbUnknown, score: 0.17
Nxph3Unknown, score: 0.3
Nxph4Unknown, score: 0.36
Adcy4Unknown, score: 0.58
Adcy3Unknown, score: 0.08
AclyUnknown, score: 0.43
Etv5Unknown, score: 0.17
Ces1dUnknown, score: 0.19
GldcUnknown, score: 0.06
RhoqUnknown, score: 0.2
Cdc42ep2Unknown, score: 0.35
Arl1Unknown, score: 0.32
Zfp119aUnknown, score: 0.06
Rcor2Unknown, score: 0.03
E2f4Unknown, score: 0.48
Pcnxl3Unknown, score: 0.41
Bap1Unknown, score: 0.21
Rexo2Unknown, score: 0.14
RarsUnknown, score: 0.01
Smek2Unknown, score: 0.22
Ddx1Unknown, score: 0.11
SptssaUnknown, score: 0.23
4930427A07RikUnknown, score: 0.15
Aldh6a1Unknown, score: 0.21
FancmUnknown, score: 0.26
AspgUnknown, score: 0.24
Cbll1Unknown, score: 0.03
Tmem179Unknown, score: 0.1
Adi1Unknown, score: 0.09
Fam110cUnknown, score: 0.34
Dnal1Unknown, score: 0.17
Fam84aUnknown, score: 0.11
Rdh14Unknown, score: 0.37
IarsUnknown, score: 0.06
Arrdc3Unknown, score: 0.07
Fam208bUnknown, score: 0.19
Slc9a3Unknown, score: 0.01
Brd9Unknown, score: 0.25
Golm1Unknown, score: 0.04
AW209491Unknown, score: 0.15
Dusp22Unknown, score: 0.05
Akr1c14Unknown, score: 0.05
Gmpr2Unknown, score: 0.1
Fam170bUnknown, score: 0.11
Chmp7Unknown, score: 0.23
Mbnl2Unknown, score: 0.16
Dph3Unknown, score: 0.2
PhyhipUnknown, score: 0.13
ThtpaUnknown, score: 0.07
Ano6Unknown, score: 0.14
Slc38a1Unknown, score: 0.41
Fam83hUnknown, score: 0.38
ScribUnknown, score: 0.28
Amigo2Unknown, score: 0.04
Ccdc65Unknown, score: 0.23
MtbpUnknown, score: 0.24
Dennd3Unknown, score: 0.53
Mal2Unknown, score: 0.02
Nckap1lUnknown, score: 0.32
105892Unknown, score: 0.07
Espl1Unknown, score: 0.3
ToporsUnknown, score: 0.38
SharpinUnknown, score: 0.1
Prickle1Unknown, score: 0.15
AW549877Unknown, score: 0.34
Slc45a4Unknown, score: 0.4
Cggbp1Unknown, score: 0.05
Eaf2Unknown, score: 0.08
SrlUnknown, score: 0.55
Gpsm3Unknown, score: 0.11
PkdccUnknown, score: 0.16
PpcsUnknown, score: 0.09
Dlk2Unknown, score: 0.13
Rab31Unknown, score: 0.4
Itfg3Unknown, score: 0.15
NrmUnknown, score: 0.17
Ankrd12Unknown, score: 0.11
Trip10Unknown, score: 0.09
Ift140Unknown, score: 0.19
Cyp4f15Unknown, score: 0.14
AI413582Unknown, score: 0.34
Rpusd1Unknown, score: 0.08
Ticam1Unknown, score: 0.19
Ttbk1Unknown, score: 0.15
Oard1Unknown, score: 0.53
Tnfaip8Unknown, score: 0.06
Smim3Unknown, score: 0.39
Hmgxb3Unknown, score: 0.12
Kctd1Unknown, score: 0.37
Arap3Unknown, score: 0.07
Wdr74Unknown, score: 0.09
GlyatUnknown, score: 0.59
Btaf1Unknown, score: 0.06
Uqcc3Unknown, score: 0.29
Macrod1Unknown, score: 0.12
AC109138.1Unknown, score: 0.25
AI837181Unknown, score: 0.39
Otub1Unknown, score: 0.09
Psat1Unknown, score: 0.32
Trpt1Unknown, score: 0.31
Fam111aUnknown, score: 0.19
Slc25a45Unknown, score: 0.39
Hat1Unknown, score: 0.06
Unc5aUnknown, score: 0.02
Unc5bUnknown, score: 0.07
Guca1bUnknown, score: 0.74
EprsUnknown, score: 0.09
Ssr1Unknown, score: 0.28
Gimap4Unknown, score: 0.12
Il1rl2Unknown, score: 0.26
Magee1Unknown, score: 0.26
Nt5c3Unknown, score: 0.23
Dio3Unknown, score: 0.08
Osr2Unknown, score: 0.12
Rdh1Unknown, score: 0.21
Uap1Unknown, score: 0.12
Coro2aUnknown, score: 0.36
Snrpd2Unknown, score: 0.48
Sf3b4Unknown, score: 0.4
Rnh1Unknown, score: 0.05
Slc12a6Unknown, score: 0.29
Mrpl30Unknown, score: 0.31
Tm6sf1Unknown, score: 0.31
Thoc5Unknown, score: 0.18
Adgrb1Unknown, score: 0.13
CthUnknown, score: 0.47
MthfsUnknown, score: 0.21
Celsr3Unknown, score: 0.72
Pom121Unknown, score: 0.18
Cdk9Unknown, score: 0.56
Frs3Unknown, score: 0.04
BreUnknown, score: 0.08
Ddb2Unknown, score: 0.09
Bfsp2Unknown, score: 0.11
Cdc20Unknown, score: 0.59
Gtpbp6Unknown, score: 0.64
Celf4Unknown, score: 0.37
Lin7aUnknown, score: 0.06
Eif2b3Unknown, score: 0.23
Grm3Unknown, score: 0.14
Grm7Unknown, score: 0.38
Ltbp4Unknown, score: 0.26
Skiv2lUnknown, score: 0.27
Med21Unknown, score: 0.05
Prkag2Unknown, score: 0.43
Baiap2Unknown, score: 0.41
B3gnt5Unknown, score: 0.08
Eif4ebp3Unknown, score: 0.14
Slco4a1Unknown, score: 0.27
Slco3a1Unknown, score: 0.2
NapgUnknown, score: 0.09
AticUnknown, score: 0.37
Sema3dUnknown, score: 0.28
Adamts7Unknown, score: 0.02
Fam50aUnknown, score: 0.07
Mat2bUnknown, score: 0.18
Fam210aUnknown, score: 0.17
Foxp1Unknown, score: 0.13
Rnpepl1Unknown, score: 0.34
108660Unknown, score: 0.02
Cops8Unknown, score: 0.16
Ccdc88aUnknown, score: 0.43
Edem2Unknown, score: 0.13
Pttg1ipUnknown, score: 0.17
Fam207aUnknown, score: 0.09
Card11Unknown, score: 0.02
Lyrm2Unknown, score: 0.36
Pnrc1Unknown, score: 0.32
Mex3bUnknown, score: 0.33
Jmjd1cUnknown, score: 0.53
Tmem74bUnknown, score: 0.11
IbtkUnknown, score: 0.5
Ankhd1Unknown, score: 0.29
Atad3aUnknown, score: 0.07
Aif1lUnknown, score: 0.19
Fam72aUnknown, score: 0.4
B4gat1Unknown, score: 0.33
AidaUnknown, score: 0.32
Trmt10aUnknown, score: 0.15
Zzz3Unknown, score: 0.03
Apol7cUnknown, score: 0.13
Irak2Unknown, score: 0.02
TprUnknown, score: 0.33
Tbc1d10cUnknown, score: 0.28
Nabp1Unknown, score: 0.05
PrkcdbpUnknown, score: 0.03
Fam212bUnknown, score: 0.15
Pfdn4Unknown, score: 0.22
Dnaaf2Unknown, score: 0.54
Exosc4Unknown, score: 0.14
Ints5Unknown, score: 0.25
Sephs1Unknown, score: 0.21
Rars2Unknown, score: 0.31
MmadhcUnknown, score: 0.25
Plekha5Unknown, score: 0.2
Gins4Unknown, score: 0.07
Fam64aUnknown, score: 0.12
Rarres1Unknown, score: 0.09
Fam118bUnknown, score: 0.32
SccpdhUnknown, score: 0.02
Mbd5Unknown, score: 0.09
Lrrc39Unknown, score: 0.11
RlfUnknown, score: 0.26
Me3Unknown, score: 0.2
Mybpc1Unknown, score: 0.34
Prex2Unknown, score: 0.38
Pkn2Unknown, score: 0.29
Ankrd39Unknown, score: 0.46
SriUnknown, score: 0.04
Cald1Unknown, score: 0.21
Upk1aUnknown, score: 0.07
NpyUnknown, score: 0.16
Acy1Unknown, score: 0.03
CtrlUnknown, score: 0.03
Cyb5aUnknown, score: 0.28
Ank2Unknown, score: 0.12
Hyal3Unknown, score: 0.19
Arrb1Unknown, score: 0.17
Actn1Unknown, score: 0.18
MaobUnknown, score: 0.41
Glo1Unknown, score: 0.07
VimpUnknown, score: 0.09
C7Unknown, score: 0.33
BrafUnknown, score: 0.25
AslUnknown, score: 0.27
Zfp91Unknown, score: 0.17
Zbtb25Unknown, score: 0.81
Art3Unknown, score: 0.1
GusbUnknown, score: 0.53
DutUnknown, score: 0.39
PygbUnknown, score: 0.05
Dnah1Unknown, score: 0.34
PyglUnknown, score: 0.02
Nop2Unknown, score: 0.21
FgbUnknown, score: 0.02
Ehmt2Unknown, score: 0.18
GgctUnknown, score: 0.49
FdpsUnknown, score: 0.12
DgkgUnknown, score: 0.1
Akr7a5Unknown, score: 0.27
PgdUnknown, score: 0.08
Tmbim6Unknown, score: 0.22
Hba-a2Unknown, score: 0.09
MsraUnknown, score: 0.16
BcrUnknown, score: 0.25
Krt7Unknown, score: 0.46
Cox6b1Unknown, score: 0.04
Pp2d1Unknown, score: 0.04
Dync2h1Unknown, score: 0.46
Rap1gapUnknown, score: 0.31
Adrbk1Unknown, score: 0.33
Sec13Unknown, score: 0.02
C8bUnknown, score: 0.13
Pde4cUnknown, score: 0.39
QdprUnknown, score: 0.21
Acat1Unknown, score: 0.29
Ly6aUnknown, score: 0.27
Acat2Unknown, score: 0.02
Hivep1Unknown, score: 0.1
DgkqUnknown, score: 0.24
Adarb1Unknown, score: 0.1
H2-Q6Unknown, score: 0.03
Arhgef28Unknown, score: 0.26
FntbUnknown, score: 0.04
Atxn3Unknown, score: 0.26
Adam33Unknown, score: 0.24
Nr3c2Unknown, score: 0.06
Srsf1Unknown, score: 0.17
EtfbUnknown, score: 0.05
Lims1Unknown, score: 0.18
EtfaUnknown, score: 0.34
Ppp2r4Unknown, score: 0.03
Scn2a1Unknown, score: 0.02
Scn4aUnknown, score: 0.31
Slc8a2Unknown, score: 0.16
Hspa13Unknown, score: 0.28
HlcsUnknown, score: 0.14
Rpl10Unknown, score: 0.18
Nudt19Unknown, score: 0.03
TarsUnknown, score: 0.31
Erc1Unknown, score: 0.08
PecrUnknown, score: 0.49
DxoUnknown, score: 0.43
Egln1Unknown, score: 0.41
Egln2Unknown, score: 0.45
Egln3Unknown, score: 0.04
C030039L03RikUnknown, score: 0.18
2610305D13RikUnknown, score: 0.09
Vmn1r42Unknown, score: 0.22
Vmn1r53Unknown, score: 0.49
Foxp2Unknown, score: 0.02
Kiss1rUnknown, score: 0.18
Dok4Unknown, score: 0.07
PalmdUnknown, score: 0.18
Slc28a3Unknown, score: 0.02
Lyve1Unknown, score: 0.12
Zbtb21Unknown, score: 0.41
Crip3Unknown, score: 0.17
Clic1Unknown, score: 0.18
Ehbp1l1Unknown, score: 0.12
Zmynd10Unknown, score: 0.05
Prdm15Unknown, score: 0.03
Elac1Unknown, score: 0.49
Rpl31Unknown, score: 0.05
BrdtUnknown, score: 0.13
Oas1cUnknown, score: 0.22
Slc13a3Unknown, score: 0.16
Hsd17b11Unknown, score: 0.05
4930444G20RikUnknown, score: 0.16
Gtf2ird2Unknown, score: 0.16
Rasa2Unknown, score: 0.09
Ddhd1Unknown, score: 0.27
Dcun1d1Unknown, score: 0.11
Afg3l1Unknown, score: 0.25
Fgfrl1Unknown, score: 0.12
Vps4aUnknown, score: 0.14
PrelpUnknown, score: 0.08
Baz2aUnknown, score: 0.05
Mta1Unknown, score: 0.16
Derl2Unknown, score: 0.33
Tgs1Unknown, score: 0.01
Pop5Unknown, score: 0.25
Acsm1Unknown, score: 0.36
TirapUnknown, score: 0.04
Pip4k2cUnknown, score: 0.17
Steap4Unknown, score: 0.01
1110038F14RikUnknown, score: 0.28
Stk33Unknown, score: 0.06
Asb7Unknown, score: 0.2
Slc2a9Unknown, score: 0.34
HelbUnknown, score: 0.06
Srgap1Unknown, score: 0.07
BocUnknown, score: 0.08
Gjc3Unknown, score: 0.1
Mrps2Unknown, score: 0.37
Mmp28Unknown, score: 0.02
Gjc2Unknown, score: 0.34
Cspg4Unknown, score: 0.59
Muc4Unknown, score: 0.39
Man2a2Unknown, score: 0.11
HnmtUnknown, score: 0.35
Kcnn2Unknown, score: 0.13
Kcnn3Unknown, score: 0.15
Atp6v0a4Unknown, score: 0.15
AF251705Unknown, score: 0.02
Ube2j2Unknown, score: 0.22
Acap3Unknown, score: 0.05
Eri3Unknown, score: 0.16
Smc1bUnknown, score: 0.51
Igsf8Unknown, score: 0.09
Plxnb2Unknown, score: 0.65
Plxnb3Unknown, score: 0.31
Ankrd6Unknown, score: 0.15
Elmo1Unknown, score: 0.1
Ubox5Unknown, score: 0.14
Ube4aUnknown, score: 0.32
Emid1Unknown, score: 0.02
Col26a1Unknown, score: 0.26
Caskin2Unknown, score: 0.06
P2ry14Unknown, score: 0.01
Il25Unknown, score: 0.04
Ttbk2Unknown, score: 0.04
Wdr5Unknown, score: 0.04
Nek8Unknown, score: 0.35
Dclre1bUnknown, score: 0.51
Elovl6Unknown, score: 0.04
Stard6Unknown, score: 0.36
Snx18Unknown, score: 0.32
Hpcal4Unknown, score: 0.5
Olfr78Unknown, score: 0.13
KirrelUnknown, score: 0.5
Peg10Unknown, score: 0.1
Cdhr1Unknown, score: 0.12
Tmem37Unknown, score: 0.16
Usp48Unknown, score: 0.08
Cyp4f13Unknown, score: 0.11
Oxr1Unknown, score: 0.12
Rtn4ip1Unknown, score: 0.2
Mfn2Unknown, score: 0.1
Klra17Unknown, score: 0.05
Arr3Unknown, score: 0.12
ParvbUnknown, score: 0.12
Tlr8Unknown, score: 0.19
Bco2Unknown, score: 0.32
Sgk3Unknown, score: 0.22
Adgrl4Unknown, score: 0.19
Rac3Unknown, score: 0.4
Pdzd3Unknown, score: 0.05
Pfkfb3Unknown, score: 0.59
Bbc3Unknown, score: 0.17
Cd209cUnknown, score: 0.02
Cd209aUnknown, score: 0.33
Hdac10Unknown, score: 0.21
Crb1Unknown, score: 0.44
GlmnUnknown, score: 0.05
Ppargc1bUnknown, score: 0.41
Tram2Unknown, score: 0.13
Hook2Unknown, score: 0.43
Sumo2Unknown, score: 0.05
Grid2ipUnknown, score: 0.16
Prima1Unknown, score: 0.3
Il17rcUnknown, score: 0.13
Fut10Unknown, score: 0.07
Mbnl3Unknown, score: 0.32
Ntng2Unknown, score: 0.46
Syt12Unknown, score: 0.2
Arhgap4Unknown, score: 0.18
Acot2Unknown, score: 0.44
Timd2Unknown, score: 0.1
Havcr2Unknown, score: 0.05
Slc12a8Unknown, score: 0.01
Gpr37l1Unknown, score: 0.2
Creld1Unknown, score: 0.26
MlphUnknown, score: 0.17
Mical1Unknown, score: 0.01
Dicer1Unknown, score: 0.05
MvdUnknown, score: 0.61
Socs7Unknown, score: 0.12
Casc3Unknown, score: 0.12
Pcdha9Unknown, score: 0.12
Ufsp2Unknown, score: 0.21
Eif4a3Unknown, score: 0.43
Fam195bUnknown, score: 0.2
Rwdd4aUnknown, score: 0.53
FlnaUnknown, score: 0.08
NadkUnknown, score: 0.17
Stab2Unknown, score: 0.11
Pkhd1l1Unknown, score: 0.14
Med9Unknown, score: 0.19
Btnl10Unknown, score: 0.27
Luc7l2Unknown, score: 0.12
Bcas3Unknown, score: 0.5
Rspo1Unknown, score: 0.06
Tmem47Unknown, score: 0.22
Hexim1Unknown, score: 0.26
TmlheUnknown, score: 0.05
Nrbp1Unknown, score: 0.1
Zfp286Unknown, score: 0.22
Wdr81Unknown, score: 0.26
Ttc36Unknown, score: 0.2
Ell2Unknown, score: 0.02
ArhgdiaUnknown, score: 0.1
Abcg4Unknown, score: 0.14
Rapgef6Unknown, score: 0.43
Itgb4Unknown, score: 0.3
NacadUnknown, score: 0.54
Lrrc75aUnknown, score: 0.03
Cyb5d2Unknown, score: 0.5
PirtUnknown, score: 0.07
Slu7Unknown, score: 0.08
Fam65bUnknown, score: 0.07
Zfp184Unknown, score: 0.17
Rnf185Unknown, score: 0.26
Hspa1aUnknown, score: 0.37
Abhd16aUnknown, score: 0.52
Kdm4bUnknown, score: 0.09
Mcfd2Unknown, score: 0.04
RimklaUnknown, score: 0.2
Vps37dUnknown, score: 0.03
Tet3Unknown, score: 0.05
Reps2Unknown, score: 0.02
Slc25a43Unknown, score: 0.22
Pld6Unknown, score: 0.03
Jmjd4Unknown, score: 0.22
Sun3Unknown, score: 0.21
Zzef1Unknown, score: 0.16
Tmem199Unknown, score: 0.11
Nlrp1aUnknown, score: 0.32
Dcdc2aUnknown, score: 0.23
Utp14bUnknown, score: 0.07
Grhl1Unknown, score: 0.05
Rbms3Unknown, score: 0.38
Ggt7Unknown, score: 0.06
Fbxo40Unknown, score: 0.33
Sec23ipUnknown, score: 0.13
Fam120cUnknown, score: 0.54
Kctd12bUnknown, score: 0.13
Dtx4Unknown, score: 0.26
Camkk2Unknown, score: 0.38
Tbc1d16Unknown, score: 0.1
Thsd4Unknown, score: 0.15
Ccdc40Unknown, score: 0.03
Wdr37Unknown, score: 0.04
Skor1Unknown, score: 0.18
Pde2aUnknown, score: 0.61
Ubald1Unknown, score: 0.1
Rnf43Unknown, score: 0.02
Bzrap1Unknown, score: 0.41
Csrnp2Unknown, score: 0.5
BC034090Unknown, score: 0.03
Gramd1cUnknown, score: 0.27
4930539E08RikUnknown, score: 0.39
Galnt6Unknown, score: 0.07
Fmr1nbUnknown, score: 0.17
Fam228bUnknown, score: 0.38
Urb1Unknown, score: 0.4
VcpkmtUnknown, score: 0.04
Pif1Unknown, score: 0.26
Chmp6Unknown, score: 0.23
Aph1bUnknown, score: 0.05
Map6d1Unknown, score: 0.09
Phldb2Unknown, score: 0.32
ExogUnknown, score: 0.26
Alg1Unknown, score: 0.11
Tmem132cUnknown, score: 0.46
Mob3aUnknown, score: 0.33
Tor1aip1Unknown, score: 0.19
Dot1lUnknown, score: 0.28
Cyp4f17Unknown, score: 0.19
Rpp40Unknown, score: 0.55
Asb18Unknown, score: 0.03
Shroom4Unknown, score: 0.14
Klhl29Unknown, score: 0.15
Sgms1Unknown, score: 0.41
1810043H04RikUnknown, score: 0.03
Cep78Unknown, score: 0.22
Nek11Unknown, score: 0.08
Etl4Unknown, score: 0.4
Creb3l2Unknown, score: 0.13
CblbUnknown, score: 0.12
Eif5a2Unknown, score: 0.1
Dis3l2Unknown, score: 0.41
Sde2Unknown, score: 0.06
Sned1Unknown, score: 0.54
Daam1Unknown, score: 0.43
Cpeb3Unknown, score: 0.08
Myo5cUnknown, score: 0.19
Hmgcll1Unknown, score: 0.04
Rbmx2Unknown, score: 0.02
Sirt7Unknown, score: 0.18
Vps8Unknown, score: 0.27
Zc3hav1lUnknown, score: 0.16
Tns2Unknown, score: 0.25
Ido2Unknown, score: 0.35
Enox2Unknown, score: 0.37
Zfp710Unknown, score: 0.15
Igsf1Unknown, score: 0.32
Wfdc6aUnknown, score: 0.24
Taf3Unknown, score: 0.05
Itih5Unknown, score: 0.07
GpkowUnknown, score: 0.06
Tfe3Unknown, score: 0.05
Tbc1d12Unknown, score: 0.05
Hsh2dUnknown, score: 0.2
Tmem164Unknown, score: 0.04
Tyw3Unknown, score: 0.1
Nudcd3Unknown, score: 0.12
Sectm1aUnknown, score: 0.11
Frmd4aUnknown, score: 0.07
Bend7Unknown, score: 0.09
Tmc7Unknown, score: 0.13
Dennd2aUnknown, score: 0.07
Slc38a5Unknown, score: 0.23
B3gntl1Unknown, score: 0.17
MtrrUnknown, score: 0.52
Slc35f3Unknown, score: 0.24
Tmem194Unknown, score: 0.09
Adcy2Unknown, score: 0.07
Zfp719Unknown, score: 0.14
Papd7Unknown, score: 0.06
Zfp180Unknown, score: 0.21
Irgc1Unknown, score: 0.1
IrgqUnknown, score: 0.2
Slc30a6Unknown, score: 0.22
Zkscan2Unknown, score: 0.31
Zfp526Unknown, score: 0.2
Nckap5Unknown, score: 0.17
Mtmr9Unknown, score: 0.19
Tdrd6Unknown, score: 0.09
Mettl14Unknown, score: 0.35
Tbc1d31Unknown, score: 0.25
Coq10aUnknown, score: 0.55
Pamr1Unknown, score: 0.2
Gab3Unknown, score: 0.26
McmbpUnknown, score: 0.07
Kbtbd2Unknown, score: 0.03
SepsecsUnknown, score: 0.36
Vmn2r15Unknown, score: 0.35
Lrrc25Unknown, score: 0.2
6720489N17RikUnknown, score: 0.35
Tsga10Unknown, score: 0.21
Tmem87aUnknown, score: 0.04
Ccdc114Unknown, score: 0.09
Ap1arUnknown, score: 0.32
MrgprfUnknown, score: 0.04
Tfdp2Unknown, score: 0.05
Ptchd1Unknown, score: 0.19
Pcdh9Unknown, score: 0.07
Mfsd9Unknown, score: 0.08
Depdc7Unknown, score: 0.1
Dennd6aUnknown, score: 0.01
Dsg1cUnknown, score: 0.35
Ccdc73Unknown, score: 0.28
Plekhh1Unknown, score: 0.27
Asxl3Unknown, score: 0.15
Zfyve26Unknown, score: 0.09
Tmem18Unknown, score: 0.32
Hk3Unknown, score: 0.21
Syne3Unknown, score: 0.19
Tmem60Unknown, score: 0.03
Inpp5aUnknown, score: 0.16
Nhlrc3Unknown, score: 0.21
Dcaf15Unknown, score: 0.33
Proser1Unknown, score: 0.32
2610015P09RikUnknown, score: 0.04
GsapUnknown, score: 0.29
Zswim4Unknown, score: 0.44
Ubxn10Unknown, score: 0.1
Klhl32Unknown, score: 0.19
LcorUnknown, score: 0.04
Ccdc110Unknown, score: 0.14
HjurpUnknown, score: 0.31
9330159F19RikUnknown, score: 0.18
Fam193bUnknown, score: 0.19
PaoxUnknown, score: 0.45
SprnUnknown, score: 0.19
BC027231Unknown, score: 0.41
Iffo2Unknown, score: 0.12
Catsper2Unknown, score: 0.34
N4bp3Unknown, score: 0.12
Satb2Unknown, score: 0.5
Ddx46Unknown, score: 0.06
Pm20d1Unknown, score: 0.02
TifabUnknown, score: 0.18
Fam46aUnknown, score: 0.19
Athl1Unknown, score: 0.21
Wbscr17Unknown, score: 0.37
Zfp583Unknown, score: 0.19
Abhd10Unknown, score: 0.13
Pdlim2Unknown, score: 0.09
Aox2Unknown, score: 0.38
Fam126bUnknown, score: 0.12
Tmem71Unknown, score: 0.16
Ankrd35Unknown, score: 0.17
Il20rbUnknown, score: 0.12
TapbplUnknown, score: 0.13
Dnd1Unknown, score: 0.23
Fstl5Unknown, score: 0.42
Txndc2Unknown, score: 0.22
Scyl2Unknown, score: 0.19
Armc2Unknown, score: 0.11
Mylk3Unknown, score: 0.3
Gpr174Unknown, score: 0.25
Rbbp5Unknown, score: 0.64
Nudt18Unknown, score: 0.15
Szrd1Unknown, score: 0.11
Fbxo42Unknown, score: 0.31
Plekhg6Unknown, score: 0.35
Bag2Unknown, score: 0.08
Plekhh2Unknown, score: 0.2
Map9Unknown, score: 0.19
9530068E07RikUnknown, score: 0.34
Duoxa1Unknown, score: 0.34
PreplUnknown, score: 0.59
Col28a1Unknown, score: 0.36
Atg9bUnknown, score: 0.08
Fam83fUnknown, score: 0.02
Tmem82Unknown, score: 0.32
Megf11Unknown, score: 0.05
Dnajc16Unknown, score: 0.02
Sox30Unknown, score: 0.04
Nipal4Unknown, score: 0.12
Trim38Unknown, score: 0.25
Kmt2aUnknown, score: 0.29
Pak6Unknown, score: 0.41
A430105I19RikUnknown, score: 0.1
SynaUnknown, score: 0.15
CrygnUnknown, score: 0.51
Hhipl1Unknown, score: 0.03
Lrrc1Unknown, score: 0.35
Tmem51Unknown, score: 0.18
MyocdUnknown, score: 0.34
Gm4788Unknown, score: 0.09
Parp16Unknown, score: 0.15
CilpUnknown, score: 0.15
Fnbp1lUnknown, score: 0.16
Tmprss4Unknown, score: 0.57
SheUnknown, score: 0.02
Cep164Unknown, score: 0.3
Prmt7Unknown, score: 0.18
Spg11Unknown, score: 0.02
Duox2Unknown, score: 0.56
Sidt2Unknown, score: 0.3
Slc10a3Unknown, score: 0.08
Papd5Unknown, score: 0.3
Cped1Unknown, score: 0.58
Slc25a29Unknown, score: 0.14
L3mbtl2Unknown, score: 0.55
Edrf1Unknown, score: 0.15
Sertad4Unknown, score: 0.19
Syde2Unknown, score: 0.03
Neurl3Unknown, score: 0.44
Chtf18Unknown, score: 0.35
Slc39a2Unknown, score: 0.79
Fbxl16Unknown, score: 0.17
Mob3bUnknown, score: 0.19
Rhbdl1Unknown, score: 0.5
Rhot2Unknown, score: 0.07
Sema6dUnknown, score: 0.13
Chtf8Unknown, score: 0.12
VeztUnknown, score: 0.17
Fam20bUnknown, score: 0.25
Bud13Unknown, score: 0.25
Trim50Unknown, score: 0.31
Hip1Unknown, score: 0.11
Trmt2bUnknown, score: 0.2
Traf3ip3Unknown, score: 0.15
Camk1gUnknown, score: 0.32
Senp6Unknown, score: 0.14
NcaphUnknown, score: 0.45
Slc35e3Unknown, score: 0.56
Gpat2Unknown, score: 0.4
Sec22cUnknown, score: 0.03
Prr14lUnknown, score: 0.14
A3galt2Unknown, score: 0.16
Pomgnt2Unknown, score: 0.13
Fam117aUnknown, score: 0.16
RnpepUnknown, score: 0.04
Zbtb8bUnknown, score: 0.08
Psd4Unknown, score: 0.11
Zmat1Unknown, score: 0.34
Arrdc1Unknown, score: 0.31
Fam73aUnknown, score: 0.05
Ccdc28aUnknown, score: 0.44
Nhsl1Unknown, score: 0.1
McuUnknown, score: 0.05
Micu1Unknown, score: 0.25
Stox1Unknown, score: 0.11
Lrrtm3Unknown, score: 0.05
Ctnna3Unknown, score: 0.04
Ube2d1Unknown, score: 0.66
YbeyUnknown, score: 0.3
Trappc10Unknown, score: 0.03
PdxkUnknown, score: 0.28
IlvblUnknown, score: 0.12
Shc2Unknown, score: 0.14
Cdc34Unknown, score: 0.28
PolrmtUnknown, score: 0.17
Lppr3Unknown, score: 0.38
Wdr18Unknown, score: 0.37
Tmem259Unknown, score: 0.54
Sbno2Unknown, score: 0.4
Plk5Unknown, score: 0.53
Aldh1l2Unknown, score: 0.06
Ckap4Unknown, score: 0.37
Mettl25Unknown, score: 0.07
Tspan8Unknown, score: 0.1
Tmem5Unknown, score: 0.23
March9Unknown, score: 0.25
Os9Unknown, score: 0.47
Slc26a10Unknown, score: 0.42
Arhgap9Unknown, score: 0.22
Gls2Unknown, score: 0.66
Pik3ip1Unknown, score: 0.09
Ccdc157Unknown, score: 0.03
Ccm2Unknown, score: 0.17
AftphUnknown, score: 0.02
Ccdc85aUnknown, score: 0.06
Cfap36Unknown, score: 0.15
Rufy1Unknown, score: 0.24
Fnip1Unknown, score: 0.12
Mfap3Unknown, score: 0.06
Mrpl22Unknown, score: 0.03
Iba57Unknown, score: 0.13
Wnt9aUnknown, score: 0.06
Nlrp3Unknown, score: 0.63
FlcnUnknown, score: 0.43
Tom1l2Unknown, score: 0.53
Dhrs7bUnknown, score: 0.22
Mmgt2Unknown, score: 0.26
Arhgap44Unknown, score: 0.26
Usp43Unknown, score: 0.15
Chd3Unknown, score: 0.17
Kdm6bUnknown, score: 0.47
Wrap53Unknown, score: 0.31
Nlgn2Unknown, score: 0.33
Acap1Unknown, score: 0.11
Slc16a11Unknown, score: 0.07
Spag7Unknown, score: 0.05
Dhx33Unknown, score: 0.25
Wscd1Unknown, score: 0.53
Coro6Unknown, score: 0.27
Git1Unknown, score: 0.36
Taok1Unknown, score: 0.23
Adap2Unknown, score: 0.17
Nle1Unknown, score: 0.11
SynrgUnknown, score: 0.06
Tada2aUnknown, score: 0.45
Trim25Unknown, score: 0.12
Utp18Unknown, score: 0.3
Spata20Unknown, score: 0.28
Ppp1r9bUnknown, score: 0.09
Prr15lUnknown, score: 0.06
Gpr179Unknown, score: 0.04
Arl5cUnknown, score: 0.37
Stac2Unknown, score: 0.16
Tns4Unknown, score: 0.13
Klhl11Unknown, score: 0.56
Plekhh3Unknown, score: 0.05
Tmem106aUnknown, score: 0.04
NagsUnknown, score: 0.03
BC030867Unknown, score: 0.05
Asb16Unknown, score: 0.08
Atxn7l3Unknown, score: 0.45
Cd300aUnknown, score: 0.19
Cd300lbUnknown, score: 0.04
Cd300ldUnknown, score: 0.29
Cd300eUnknown, score: 0.02
Hid1Unknown, score: 0.13
Slc16a5Unknown, score: 0.55
Llgl2Unknown, score: 0.28
UnkUnknown, score: 0.63
Trim47Unknown, score: 0.24
Fbf1Unknown, score: 0.04
Qrich2Unknown, score: 0.08
Ube2oUnknown, score: 0.56
Tnrc6cUnknown, score: 0.67
Tmc6Unknown, score: 0.41
EngaseUnknown, score: 0.23
Nploc4Unknown, score: 0.3
Lrrc45Unknown, score: 0.32
Uts2rUnknown, score: 0.25
BC017643Unknown, score: 0.4
Ubxn2aUnknown, score: 0.11
Nol10Unknown, score: 0.25
Ankmy2Unknown, score: 0.07
L2hgdhUnknown, score: 0.17
Plekhd1Unknown, score: 0.04
Sipa1l1Unknown, score: 0.2
Fam161bUnknown, score: 0.22
Lin52Unknown, score: 0.06
Mlh3Unknown, score: 0.62
Nek9Unknown, score: 0.1
Mfsd7cUnknown, score: 0.1
Lysmd1Unknown, score: 0.12
Kcnk13Unknown, score: 0.14
Nrde2Unknown, score: 0.08
9030617O03RikUnknown, score: 0.06
Rin3Unknown, score: 0.26
Unc79Unknown, score: 0.16
Rcor1Unknown, score: 0.06
Eif5Unknown, score: 0.1
Rd3lUnknown, score: 0.56
Cep170bUnknown, score: 0.06
Tmem196Unknown, score: 0.25
Phactr1Unknown, score: 0.36
Kdm1bUnknown, score: 0.06
Rnf144bUnknown, score: 0.12
Fam120aUnknown, score: 0.19
Ice1Unknown, score: 0.03
RfesdUnknown, score: 0.02
Serinc5Unknown, score: 0.07
Wdr41Unknown, score: 0.09
Pde8bUnknown, score: 0.36
Mrps27Unknown, score: 0.01
Dhx29Unknown, score: 0.13
Slc4a7Unknown, score: 0.31
Lrrc3bUnknown, score: 0.35
RarbUnknown, score: 0.13
Ube2e2Unknown, score: 0.12
Zfp503Unknown, score: 0.21
ChdhUnknown, score: 0.33
Sema3gUnknown, score: 0.11
Tmem260Unknown, score: 0.24
Ttc5Unknown, score: 0.24
Tmem55bUnknown, score: 0.23
Haus4Unknown, score: 0.45
Ska3Unknown, score: 0.05
Phf11dUnknown, score: 0.26
Spata13Unknown, score: 0.22
Arl11Unknown, score: 0.33
Xkr6Unknown, score: 0.04
Hmbox1Unknown, score: 0.22
Scara3Unknown, score: 0.19
Pcdh20Unknown, score: 0.15
Stk24Unknown, score: 0.31
Fam105aUnknown, score: 0.06
TrioUnknown, score: 0.16
Dcaf13Unknown, score: 0.05
Eny2Unknown, score: 0.15
Fam49bUnknown, score: 0.05
Them6Unknown, score: 0.04
Zc3h3Unknown, score: 0.32
NaprtUnknown, score: 0.37
Nrbp2Unknown, score: 0.33
Mroh1Unknown, score: 0.51
Lrrc14Unknown, score: 0.11
Apol9aUnknown, score: 0.19
Ankrd54Unknown, score: 0.05
Eif3lUnknown, score: 0.2
Tomm22Unknown, score: 0.22
Sun2Unknown, score: 0.28
Mkl1Unknown, score: 0.3
McatUnknown, score: 0.04
Ttll12Unknown, score: 0.41
Ldoc1lUnknown, score: 0.14
5031439G07RikUnknown, score: 0.3
Gramd4Unknown, score: 0.77
CerkUnknown, score: 0.61
Brd1Unknown, score: 0.35
Zbed4Unknown, score: 0.22
Alg12Unknown, score: 0.36
Pim3Unknown, score: 0.72
Adamts20Unknown, score: 0.18
Rapgef3Unknown, score: 0.15
Senp1Unknown, score: 0.08
Rnd1Unknown, score: 0.15
Spryd3Unknown, score: 0.1
Rmi2Unknown, score: 0.17
Cpped1Unknown, score: 0.03
Fgd4Unknown, score: 0.17
Tmem191cUnknown, score: 0.1
Pi4kaUnknown, score: 0.56
Slc7a4Unknown, score: 0.47
Klhl22Unknown, score: 0.17
Cyp2ab1Unknown, score: 0.25
Atp13a3Unknown, score: 0.08
Ubxn7Unknown, score: 0.18
224273Unknown, score: 0.02
Scaf4Unknown, score: 0.02
Zfp758Unknown, score: 0.21
Flywch1Unknown, score: 0.6
Tbc1d24Unknown, score: 0.4
Traf7Unknown, score: 0.21
Bnip1Unknown, score: 0.36
Lemd2Unknown, score: 0.2
D17Wsu92eUnknown, score: 0.77
Uhrf1bp1Unknown, score: 0.41
Anks1Unknown, score: 0.5
Slc26a8Unknown, score: 0.25
Btbd9Unknown, score: 0.21
Zfp81Unknown, score: 0.04
Adamts10Unknown, score: 0.52
Vps52Unknown, score: 0.18
Abcf1Unknown, score: 0.09
Clic5Unknown, score: 0.35
Aars2Unknown, score: 0.36
Tmem63bUnknown, score: 0.31
Lrrc73Unknown, score: 0.61
Ubr2Unknown, score: 0.13
224829Unknown, score: 0.06
Zfp959Unknown, score: 0.06
SafbUnknown, score: 0.08
2410015M20RikUnknown, score: 0.19
Dus3lUnknown, score: 0.06
Pja2Unknown, score: 0.19
BC027072Unknown, score: 0.08
Lclat1Unknown, score: 0.05
Map4k3Unknown, score: 0.23
Ankrd29Unknown, score: 0.32
Rprd1aUnknown, score: 0.3
Pik3c3Unknown, score: 0.35
Fam13bUnknown, score: 0.27
Etf1Unknown, score: 0.43
Sra1Unknown, score: 0.05
Rbm27Unknown, score: 0.12
Ticam2Unknown, score: 0.13
Pde6aUnknown, score: 0.15
Onecut2Unknown, score: 0.43
Alpk2Unknown, score: 0.31
Mppe1Unknown, score: 0.03
Slmo1Unknown, score: 0.22
Cep76Unknown, score: 0.08
Mapk4Unknown, score: 0.03
St8sia5Unknown, score: 0.21
Zadh2Unknown, score: 0.06
Pla2g16Unknown, score: 0.33
Ppp2r5bUnknown, score: 0.14
Snx32Unknown, score: 0.37
Rin1Unknown, score: 0.21
Npas4Unknown, score: 0.64
Lrfn4Unknown, score: 0.01
Kdm2aUnknown, score: 0.09
BC021614Unknown, score: 0.15
Suv420h1Unknown, score: 0.33
Ubxn1Unknown, score: 0.3
MyrfUnknown, score: 0.29
DakUnknown, score: 0.06
Nmrk1Unknown, score: 0.02
D030056L22RikUnknown, score: 0.04
Abhd17bUnknown, score: 0.02
Smc5Unknown, score: 0.57
Tmem252Unknown, score: 0.18
Cbwd1Unknown, score: 0.12
Dmrt2Unknown, score: 0.06
Cyp2c70Unknown, score: 0.29
Morn4Unknown, score: 0.09
Fam178aUnknown, score: 0.15
Peo1Unknown, score: 0.1
DpcdUnknown, score: 0.31
Pprc1Unknown, score: 0.51
Habp2Unknown, score: 0.09
Plekhs1Unknown, score: 0.03
Fam160b1Unknown, score: 0.43
Eno4Unknown, score: 0.02
Epb4.1l5Unknown, score: 0.03
Zranb3Unknown, score: 0.13
R3hdm1Unknown, score: 0.12
DarsUnknown, score: 0.3
Dyrk3Unknown, score: 0.26
5430435G22RikUnknown, score: 0.21
Zfp281Unknown, score: 0.04
BC003331Unknown, score: 0.14
Smg7Unknown, score: 0.25
Nmnat2Unknown, score: 0.09
Rasal2Unknown, score: 0.14
BC026585Unknown, score: 0.03
Dars2Unknown, score: 0.19
Klhl20Unknown, score: 0.28
Prrc2cUnknown, score: 0.13
Fam78bUnknown, score: 0.23
Atf6Unknown, score: 0.03
Arhgap30Unknown, score: 0.17
Ifi205Unknown, score: 0.24
C130074G19RikUnknown, score: 0.26
Slc30a10Unknown, score: 0.39
Lyplal1Unknown, score: 0.26
Smyd2Unknown, score: 0.17
Vash2Unknown, score: 0.02
Ppp2r5aUnknown, score: 0.09
Lpgat1Unknown, score: 0.24
SbsponUnknown, score: 0.54
Arhgef4Unknown, score: 0.21
Plekhb2Unknown, score: 0.1
Tmem194bUnknown, score: 0.08
Pms1Unknown, score: 0.01
Plcl1Unknown, score: 0.05
Cxcr1Unknown, score: 0.06
Gpbar1Unknown, score: 0.22
AampUnknown, score: 0.17
Ctdsp1Unknown, score: 0.3
DnerUnknown, score: 0.02
Gpr55Unknown, score: 0.23
Gigyf2Unknown, score: 0.11
Farp2Unknown, score: 0.1
Ppip5k2Unknown, score: 0.05
Dclre1cUnknown, score: 0.2
Camk1dUnknown, score: 0.08
C1ql3Unknown, score: 0.25
Tor4aUnknown, score: 0.31
Tubb4bUnknown, score: 0.6
Tmem203Unknown, score: 0.16
Uap1l1Unknown, score: 0.03
BC029214Unknown, score: 0.05
Kcnt1Unknown, score: 0.1
Camsap1Unknown, score: 0.24
Qsox2Unknown, score: 0.25
Snapc4Unknown, score: 0.27
Rexo4Unknown, score: 0.3
Slc2a6Unknown, score: 0.18
Gbgt1Unknown, score: 0.04
Ddx31Unknown, score: 0.16
Trub2Unknown, score: 0.4
Coq4Unknown, score: 0.12
Zer1Unknown, score: 0.12
Phyhd1Unknown, score: 0.42
DolkUnknown, score: 0.58
Nup188Unknown, score: 0.07
Exosc2Unknown, score: 0.19
QrfpUnknown, score: 0.37
Nup214Unknown, score: 0.46
Ppapdc3Unknown, score: 0.25
Prrc2bUnknown, score: 0.3
Slc25a25Unknown, score: 0.36
1700019L03RikUnknown, score: 0.18
Fam129bUnknown, score: 0.26
Lrsam1Unknown, score: 0.14
Mapkap1Unknown, score: 0.04
RabepkUnknown, score: 0.4
Rabgap1Unknown, score: 0.15
Epc2Unknown, score: 0.02
GcaUnknown, score: 0.01
Pdk1Unknown, score: 0.27
AgpsUnknown, score: 0.1
MaddUnknown, score: 0.09
1110051M20RikUnknown, score: 0.68
Lrp4Unknown, score: 0.11
Arhgap1Unknown, score: 0.18
Ambra1Unknown, score: 0.02
Gyltl1bUnknown, score: 0.1
Kif18aUnknown, score: 0.14
Zfp770Unknown, score: 0.2
RhovUnknown, score: 0.78
Vps18Unknown, score: 0.5
ItpkaUnknown, score: 0.03
F830045P16RikUnknown, score: 0.21
Ebf4Unknown, score: 0.3
4930402H24RikUnknown, score: 0.28
MavsUnknown, score: 0.17
Btbd3Unknown, score: 0.13
KizUnknown, score: 0.29
Psmf1Unknown, score: 0.46
Trib3Unknown, score: 0.07
6820408C15RikUnknown, score: 0.26
Mylk2Unknown, score: 0.12
Ccm2lUnknown, score: 0.07
Asxl1Unknown, score: 0.25
Bpifb1Unknown, score: 0.23
Zfp341Unknown, score: 0.3
D630003M21RikUnknown, score: 0.08
Gdap1l1Unknown, score: 0.29
Fitm2Unknown, score: 0.38
Pcif1Unknown, score: 0.09
Ncoa5Unknown, score: 0.35
Zfp334Unknown, score: 0.18
Tshz2Unknown, score: 0.08
Stx16Unknown, score: 0.28
Taf4aUnknown, score: 0.07
Osbpl2Unknown, score: 0.36
Slc17a9Unknown, score: 0.23
Ythdf1Unknown, score: 0.19
Arfgap1Unknown, score: 0.21
Helz2Unknown, score: 0.23
Gmeb2Unknown, score: 0.09
ZgpatUnknown, score: 0.42
Hnrnpa3Unknown, score: 0.08
D930015E06RikUnknown, score: 0.24
GatbUnknown, score: 0.05
Fam160a1Unknown, score: 0.08
Isg20l2Unknown, score: 0.54
Smg5Unknown, score: 0.26
Slc25a44Unknown, score: 0.16
Msto1Unknown, score: 0.32
Pbxip1Unknown, score: 0.23
Dennd4bUnknown, score: 0.1
Gatad2bUnknown, score: 0.2
Ints3Unknown, score: 0.29
PogzUnknown, score: 0.18
Gm128Unknown, score: 0.18
Golph3lUnknown, score: 0.48
Adamtsl4Unknown, score: 0.12
Pias3Unknown, score: 0.11
Trim45Unknown, score: 0.08
Csde1Unknown, score: 0.1
Ampd1Unknown, score: 0.13
St7lUnknown, score: 0.09
Chil5Unknown, score: 0.1
Slc16a4Unknown, score: 0.31
Slc6a17Unknown, score: 0.49
Gpr61Unknown, score: 0.76
Znhit6Unknown, score: 0.04
Ddx58Unknown, score: 0.37
Ndufb6Unknown, score: 0.17
N28178Unknown, score: 0.21
Arhgef39Unknown, score: 0.15
Gba2Unknown, score: 0.04
Zbtb5Unknown, score: 0.2
Slc25a51Unknown, score: 0.18
Fam206aUnknown, score: 0.01
Ptbp3Unknown, score: 0.02
E130308A19RikUnknown, score: 0.04
Haus6Unknown, score: 0.13
Acer2Unknown, score: 0.25
Usp1Unknown, score: 0.19
C8aUnknown, score: 0.07
Ttc22Unknown, score: 0.55
Pars2Unknown, score: 0.11
Cyb5rlUnknown, score: 0.36
Yipf1Unknown, score: 0.14
Zfyve9Unknown, score: 0.05
Atpaf1Unknown, score: 0.61
Tmem69Unknown, score: 0.1
Kdm4aUnknown, score: 0.26
Zmpste24Unknown, score: 0.67
Pabpc4Unknown, score: 0.03
Rhbdl2Unknown, score: 0.05
Gnl2Unknown, score: 0.52
Oscp1Unknown, score: 0.48
Thrap3Unknown, score: 0.05
5730409E04RikUnknown, score: 0.24
Zfp362Unknown, score: 0.13
IqccUnknown, score: 0.44
Tmem39bUnknown, score: 0.44
Adgrb2Unknown, score: 0.32
Hcrtr1Unknown, score: 0.1
Serinc2Unknown, score: 0.55
Themis2Unknown, score: 0.04
Fam76aUnknown, score: 0.28
Ahdc1Unknown, score: 0.42
Wdtc1Unknown, score: 0.32
PigvUnknown, score: 0.11
Man1c1Unknown, score: 0.24
NcmapUnknown, score: 0.1
Grhl3Unknown, score: 0.06
Il22ra1Unknown, score: 0.06
Asap3Unknown, score: 0.28
Zbtb40Unknown, score: 0.21
Eif4g3Unknown, score: 0.07
Sh2d5Unknown, score: 0.27
CroccUnknown, score: 0.11
Fbxo2Unknown, score: 0.06
TardbpUnknown, score: 0.27
Gm572Unknown, score: 0.36
Tmem201Unknown, score: 0.03
Dnajc11Unknown, score: 0.16
Ajap1Unknown, score: 0.15
Cep104Unknown, score: 0.47
Megf6Unknown, score: 0.06
Arhgef16Unknown, score: 0.16
Tnfrsf14Unknown, score: 0.18
9430015G10RikUnknown, score: 0.08
Plekhn1Unknown, score: 0.38
Galnt11Unknown, score: 0.03
HadhbUnknown, score: 0.33
Haus3Unknown, score: 0.35
Fam193aUnknown, score: 0.05
Tnip2Unknown, score: 0.3
Dok7Unknown, score: 0.04
Sh3tc1Unknown, score: 0.53
Ablim2Unknown, score: 0.05
Tada2bUnknown, score: 0.45
Guf1Unknown, score: 0.25
Slc10a4Unknown, score: 0.06
Lrrc66Unknown, score: 0.11
AasdhUnknown, score: 0.12
Ugt2b36Unknown, score: 0.03
Lin54Unknown, score: 0.15
Rpap2Unknown, score: 0.21
GakUnknown, score: 0.09
Slc26a1Unknown, score: 0.07
Galnt9Unknown, score: 0.09
FicdUnknown, score: 0.26
Ssh1Unknown, score: 0.04
Alkbh2Unknown, score: 0.03
Oasl1Unknown, score: 0.27
Gcn1l1Unknown, score: 0.02
Fbxo21Unknown, score: 0.25
Fbxw8Unknown, score: 0.08
SdsUnknown, score: 0.11
Naa25Unknown, score: 0.06
Rad9bUnknown, score: 0.09
SfswapUnknown, score: 0.3
Lrch4Unknown, score: 0.35
Agfg2Unknown, score: 0.04
MepceUnknown, score: 0.29
PilraUnknown, score: 0.36
Adap1Unknown, score: 0.06
Tmem184aUnknown, score: 0.21
Snx8Unknown, score: 0.24
Brat1Unknown, score: 0.24
Amz1Unknown, score: 0.12
Ap5z1Unknown, score: 0.27
RadilUnknown, score: 0.36
Tnrc18Unknown, score: 0.23
Zfp12Unknown, score: 0.48
E130309D02RikUnknown, score: 0.03
Ccz1Unknown, score: 0.09
Bud31Unknown, score: 0.02
Creb5Unknown, score: 0.08
Plekha8Unknown, score: 0.36
Elmod3Unknown, score: 0.23
Paip2bUnknown, score: 0.35
Smyd5Unknown, score: 0.12
Arhgap25Unknown, score: 0.56
Txnrd3Unknown, score: 0.18
Iqsec1Unknown, score: 0.16
Ccdc174Unknown, score: 0.1
Fgd5Unknown, score: 0.11
Ppp4r2Unknown, score: 0.04
Slc6a1Unknown, score: 0.26
Vgll4Unknown, score: 0.2
Ankrd26Unknown, score: 0.17
Wnk1Unknown, score: 0.13
Clstn3Unknown, score: 0.27
Klrb1fUnknown, score: 0.13
Clec9aUnknown, score: 0.09
Crebl2Unknown, score: 0.18
Gprc5aUnknown, score: 0.06
H2afjUnknown, score: 0.34
RergUnknown, score: 0.07
Gys2Unknown, score: 0.23
Stk38lUnknown, score: 0.08
Mrps35Unknown, score: 0.14
Amn1Unknown, score: 0.2
Tspan33Unknown, score: 0.21
Zc3hc1Unknown, score: 0.06
MgamUnknown, score: 0.12
Tcaf2Unknown, score: 0.05
OscarUnknown, score: 0.01
Cnot3Unknown, score: 0.33
Lilra5Unknown, score: 0.25
Ppp1r12cUnknown, score: 0.35
Shisa7Unknown, score: 0.4
Zfp628Unknown, score: 0.18
Ccdc106Unknown, score: 0.13
Zfp772Unknown, score: 0.04
Zscan22Unknown, score: 0.4
Pla2g4cUnknown, score: 0.18
Arhgap35Unknown, score: 0.34
Ap2s1Unknown, score: 0.16
Ccdc61Unknown, score: 0.78
MypopUnknown, score: 0.08
Klc3Unknown, score: 0.15
Mark4Unknown, score: 0.47
Ppp1r37Unknown, score: 0.05
Zfp114Unknown, score: 0.46
Zfp428Unknown, score: 0.14
Phldb3Unknown, score: 0.11
Atp1a3Unknown, score: 0.51
Zfp574Unknown, score: 0.36
B3gnt8Unknown, score: 0.43
B9d2Unknown, score: 0.29
ItpkcUnknown, score: 0.22
BlvrbUnknown, score: 0.1
Hipk4Unknown, score: 0.41
Pak4Unknown, score: 0.04
Fbxo27Unknown, score: 0.03
Zfp790Unknown, score: 0.16
Zfp420Unknown, score: 0.04
Wdr62Unknown, score: 0.45
Alkbh6Unknown, score: 0.37
Syne4Unknown, score: 0.55
Lrfn3Unknown, score: 0.23
Arhgap33Unknown, score: 0.05
U2af1l4Unknown, score: 0.14
Ffar2Unknown, score: 0.14
Ffar1Unknown, score: 0.14
4931406P16RikUnknown, score: 0.49
SiglecfUnknown, score: 0.26
Mybpc2Unknown, score: 0.14
Tbc1d17Unknown, score: 0.37
Scaf1Unknown, score: 0.27
Tubgcp5Unknown, score: 0.2
Nipa1Unknown, score: 0.21
Mtmr10Unknown, score: 0.32
Lrrk1Unknown, score: 0.05
Adamts17Unknown, score: 0.04
Prc1Unknown, score: 0.38
Zfp592Unknown, score: 0.21
CrebzfUnknown, score: 0.08
Rsf1Unknown, score: 0.12
Mogat2Unknown, score: 0.43
Gdpd5Unknown, score: 0.17
Twf1Unknown, score: 0.23
Dchs1Unknown, score: 0.1
Ipo7Unknown, score: 0.2
Plekha7Unknown, score: 0.39
Vwa3aUnknown, score: 0.07
Slc5a11Unknown, score: 0.08
Gtf3c1Unknown, score: 0.48
D430042O09RikUnknown, score: 0.18
TufmUnknown, score: 0.15
Hirip3Unknown, score: 0.25
Zfp553Unknown, score: 0.09
Zfp768Unknown, score: 0.07
Prr14Unknown, score: 0.56
Rnf40Unknown, score: 0.15
Fbxl19Unknown, score: 0.32
Setd1aUnknown, score: 0.17
Zfp646Unknown, score: 0.06
FusUnknown, score: 0.52
Armc5Unknown, score: 0.17
Ppfia1Unknown, score: 0.38
Whsc1l1Unknown, score: 0.12
Sorbs2Unknown, score: 0.33
Naf1Unknown, score: 0.28
Zfp868Unknown, score: 0.07
234365Unknown, score: 0.36
Gatad2aUnknown, score: 0.73
Tmem161aUnknown, score: 0.15
Sugp2Unknown, score: 0.24
Ddx49Unknown, score: 0.15
Klhl26Unknown, score: 0.04
Mpv17l2Unknown, score: 0.17
Ces1fUnknown, score: 0.05
Ccdc102aUnknown, score: 0.27
Ces2bUnknown, score: 0.07
Ces2eUnknown, score: 0.4
D230025D16RikUnknown, score: 0.42
Elmo3Unknown, score: 0.15
Lrrc29Unknown, score: 0.17
Fhod1Unknown, score: 0.14
RltprUnknown, score: 0.51
Edc4Unknown, score: 0.16
Nrn1lUnknown, score: 0.17
Zfp612Unknown, score: 0.18
Vac14Unknown, score: 0.33
FukUnknown, score: 0.17
Ddx19bUnknown, score: 0.11
AarsUnknown, score: 0.05
Rfwd3Unknown, score: 0.01
Tmem231Unknown, score: 0.3
Plcg2Unknown, score: 0.12
Klhl36Unknown, score: 0.5
6430548M08RikUnknown, score: 0.7
Klhdc4Unknown, score: 0.02
Spg7Unknown, score: 0.38
Chmp1aUnknown, score: 0.34
Cdk10Unknown, score: 0.07
Spire2Unknown, score: 0.08
Ttc13Unknown, score: 0.09
Gucy1a2Unknown, score: 0.25
Mmp27Unknown, score: 0.18
Med17Unknown, score: 0.03
Ccdc67Unknown, score: 0.38
Slc36a4Unknown, score: 0.2
Zfp426Unknown, score: 0.04
PpanUnknown, score: 0.05
Atg4dUnknown, score: 0.23
Tmem205Unknown, score: 0.14
Sept7Unknown, score: 0.28
Zbtb44Unknown, score: 0.09
NfrkbUnknown, score: 0.02
Foxred1Unknown, score: 0.14
Msantd2Unknown, score: 0.2
Gramd1bUnknown, score: 0.24
Rnf214Unknown, score: 0.17
Usp28Unknown, score: 0.06
DlatUnknown, score: 0.43
Sik2Unknown, score: 0.05
HykkUnknown, score: 0.26
Snx33Unknown, score: 0.2
Lman1lUnknown, score: 0.02
LctlUnknown, score: 0.08
Gtf2a2Unknown, score: 0.04
PrtgUnknown, score: 0.31
Leo1Unknown, score: 0.2
Gk5Unknown, score: 0.54
Atp2c1Unknown, score: 0.26
GlyctkUnknown, score: 0.02
6430571L13RikUnknown, score: 0.11
ApehUnknown, score: 0.17
AtripUnknown, score: 0.09
Plxnb1Unknown, score: 0.44
Setd2Unknown, score: 0.56
Nbeal2Unknown, score: 0.35
Zfp825Unknown, score: 0.44
Phf11aUnknown, score: 0.28
Ago1Unknown, score: 0.3
Gbp9Unknown, score: 0.11
Spry3Unknown, score: 0.06
NyxUnknown, score: 0.41
Slc9a7Unknown, score: 0.2
Rbm10Unknown, score: 0.04
Usp11Unknown, score: 0.04
Mmgt1Unknown, score: 0.35
BC023829Unknown, score: 0.04
Pcyt1bUnknown, score: 0.18
Arhgef9Unknown, score: 0.12
Nxt2Unknown, score: 0.05
Gnl3lUnknown, score: 0.34
Tbpl1Unknown, score: 0.1
L3mbtl3Unknown, score: 0.02
Sh3rf3Unknown, score: 0.16
Adamts14Unknown, score: 0.3
Lrrc3Unknown, score: 0.13
C2cd4cUnknown, score: 0.62
Mex3dUnknown, score: 0.62
Tmtc3Unknown, score: 0.04
Rassf9Unknown, score: 0.12
TrhdeUnknown, score: 0.2
Stac3Unknown, score: 0.21
Ankrd52Unknown, score: 0.05
Pla2g3Unknown, score: 0.16
Npc1l1Unknown, score: 0.02
Eml6Unknown, score: 0.04
Zfp867Unknown, score: 0.42
Mief2Unknown, score: 0.21
Smcr8Unknown, score: 0.32
Ssh2Unknown, score: 0.13
Atad5Unknown, score: 0.18
Brip1Unknown, score: 0.08
Rsad1Unknown, score: 0.61
Phospho1Unknown, score: 0.29
Aoc2Unknown, score: 0.14
Gpatch8Unknown, score: 0.19
Sdk2Unknown, score: 0.26
Cdr2lUnknown, score: 0.27
Fscn2Unknown, score: 0.26
ApobUnknown, score: 0.18
Gdf7Unknown, score: 0.1
Kcns3Unknown, score: 0.19
Dock4Unknown, score: 0.07
Arid4aUnknown, score: 0.14
Elmsan1Unknown, score: 0.18
Vash1Unknown, score: 0.27
Irf2bplUnknown, score: 0.15
Samd15Unknown, score: 0.3
Gpr68Unknown, score: 0.03
Slc24a4Unknown, score: 0.37
Btbd7Unknown, score: 0.17
Serpina3fUnknown, score: 0.47
Serpinb6dUnknown, score: 0.27
Gpr150Unknown, score: 0.04
Tnpo1Unknown, score: 0.08
Ppwd1Unknown, score: 0.52
OgdhlUnknown, score: 0.07
Ccnb1ip1Unknown, score: 0.16
HomezUnknown, score: 0.22
Zfhx2Unknown, score: 0.07
C1qtnf9Unknown, score: 0.14
Gucy1b2Unknown, score: 0.08
Gm27179Unknown, score: 0.29
Fam160b2Unknown, score: 0.27
Slitrk6Unknown, score: 0.01
Abcc4Unknown, score: 0.21
Card6Unknown, score: 0.12
Adamts12Unknown, score: 0.11
Tspyl5Unknown, score: 0.33
Lrp12Unknown, score: 0.17
AardUnknown, score: 0.27
Fam83aUnknown, score: 0.11
Phf20l1Unknown, score: 0.14
Ago2Unknown, score: 0.09
Gpr20Unknown, score: 0.24
Foxred2Unknown, score: 0.16
Mief1Unknown, score: 0.08
Cacna1iUnknown, score: 0.08
A4galtUnknown, score: 0.28
239611Unknown, score: 0.68
Pdzrn4Unknown, score: 0.14
Dip2bUnknown, score: 0.68
AU021092Unknown, score: 0.22
Mettl22Unknown, score: 0.19
Klhl6Unknown, score: 0.04
LiphUnknown, score: 0.57
Ndufa11Unknown, score: 0.11
Mb21d2Unknown, score: 0.26
Ccdc14Unknown, score: 0.14
Cadm2Unknown, score: 0.09
Arid1bUnknown, score: 0.19
Pnldc1Unknown, score: 0.17
Dact2Unknown, score: 0.1
Zfp945Unknown, score: 0.18
Mmp25Unknown, score: 0.14
Neurl1bUnknown, score: 0.34
Syngap1Unknown, score: 0.25
Cpne5Unknown, score: 0.1
Morc2bUnknown, score: 0.21
Cchcr1Unknown, score: 0.11
Mdc1Unknown, score: 0.11
Zfp119bUnknown, score: 0.11
Rasgrp3Unknown, score: 0.22
ThadaUnknown, score: 0.04
Zfp438Unknown, score: 0.01
Dmxl1Unknown, score: 0.17
F830016B08RikUnknown, score: 0.18
Mex3cUnknown, score: 0.08
Setbp1Unknown, score: 0.32
Adnp2Unknown, score: 0.71
Zfp407Unknown, score: 0.13
Cdc42bpgUnknown, score: 0.43
Peli3Unknown, score: 0.02
CcnjUnknown, score: 0.12
Sec31bUnknown, score: 0.55
Dusp5Unknown, score: 0.08
Mcmdc2Unknown, score: 0.16
Sulf1Unknown, score: 0.07
Slco5a1Unknown, score: 0.05
Pik3c2bUnknown, score: 0.18
Kcnt2Unknown, score: 0.17
Teddm2Unknown, score: 0.04
Teddm1aUnknown, score: 0.19
Tor1aip2Unknown, score: 0.12
Zbtb37Unknown, score: 0.28
Scyl3Unknown, score: 0.45
Dusp27Unknown, score: 0.57
Lrrc52Unknown, score: 0.29
Adamts4Unknown, score: 0.12
CarfUnknown, score: 0.08
Gpr1Unknown, score: 0.15
Prkag3Unknown, score: 0.11
Ankmy1Unknown, score: 0.04
Itga8Unknown, score: 0.2
St8sia6Unknown, score: 0.23
Gpr158Unknown, score: 0.08
Pnpla7Unknown, score: 0.17
Ppp1r26Unknown, score: 0.22
Lrrc8aUnknown, score: 0.24
Zbtb6Unknown, score: 0.2
Xirp2Unknown, score: 0.15
Cers6Unknown, score: 0.2
Dhrs9Unknown, score: 0.19
Pde11aUnknown, score: 0.13
Fam171bUnknown, score: 0.19
D430041D05RikUnknown, score: 0.04
241621Unknown, score: 0.39
Wdr76Unknown, score: 0.01
Atp8b4Unknown, score: 0.35
Ralgapa2Unknown, score: 0.28
Tspyl3Unknown, score: 0.06
L3mbtl1Unknown, score: 0.07
Rims4Unknown, score: 0.02
Lsm14bUnknown, score: 0.32
Abhd16bUnknown, score: 0.1
Igsf10Unknown, score: 0.02
Impad1Unknown, score: 0.26
Fam110bUnknown, score: 0.23
ManeaUnknown, score: 0.08
Pm20d2Unknown, score: 0.02
Fam221bUnknown, score: 0.24
Dcaf10Unknown, score: 0.17
Gabbr2Unknown, score: 0.16
Grin3aUnknown, score: 0.11
Palm2Unknown, score: 0.29
Bnc2Unknown, score: 0.03
Klhl9Unknown, score: 0.23
Dmrta1Unknown, score: 0.26
Slc1a7Unknown, score: 0.01
PodnUnknown, score: 0.19
Dmrta2Unknown, score: 0.33
HpdlUnknown, score: 0.16
Tctex1d4Unknown, score: 0.05
Cldn19Unknown, score: 0.04
Rims3Unknown, score: 0.02
Gpatch3Unknown, score: 0.15
Myom3Unknown, score: 0.13
Klhdc7aUnknown, score: 0.04
Lrrc38Unknown, score: 0.12
Zfp933Unknown, score: 0.07
Ptchd2Unknown, score: 0.73
Klhl21Unknown, score: 0.28
Ttc34Unknown, score: 0.04
Rundc3bUnknown, score: 0.23
NapepldUnknown, score: 0.04
Actr3bUnknown, score: 0.08
Fbxl5Unknown, score: 0.25
Hsd17b13Unknown, score: 0.13
Mfsd7aUnknown, score: 0.04
Sbno1Unknown, score: 0.29
Adgrd1Unknown, score: 0.14
A430033K04RikUnknown, score: 0.14
Elfn1Unknown, score: 0.55
Tmem130Unknown, score: 0.32
Stard13Unknown, score: 0.23
SspoUnknown, score: 0.15
Lrrc61Unknown, score: 0.48
Gimap8Unknown, score: 0.31
Ppm1kUnknown, score: 0.19
Ccdc142Unknown, score: 0.08
Grip2Unknown, score: 0.05
Kbtbd8Unknown, score: 0.17
Ssu2Unknown, score: 0.01
Clec1aUnknown, score: 0.03
Ppp1r9aUnknown, score: 0.3
E330009J07RikUnknown, score: 0.18
Gp6Unknown, score: 0.14
Zfp128Unknown, score: 0.35
Zfp324Unknown, score: 0.27
Gltscr1Unknown, score: 0.14
Ccdc9Unknown, score: 0.19
FkrpUnknown, score: 0.17
Mill2Unknown, score: 0.18
Fbxo46Unknown, score: 0.29
GgnUnknown, score: 0.23
Zfp568Unknown, score: 0.26
NfkbidUnknown, score: 0.11
Hspb6Unknown, score: 0.29
Lgi4Unknown, score: 0.05
Tshz3Unknown, score: 0.08
Zfp536Unknown, score: 0.04
Shank1Unknown, score: 0.04
Zfp473Unknown, score: 0.12
Ntn5Unknown, score: 0.09
Zdhhc13Unknown, score: 0.02
Mctp2Unknown, score: 0.39
Chd2Unknown, score: 0.08
Nars2Unknown, score: 0.41
TskuUnknown, score: 0.07
Trim30bUnknown, score: 0.03
Nlrp10Unknown, score: 0.1
Cyp2r1Unknown, score: 0.51
Zfp771Unknown, score: 0.2
Ctf2Unknown, score: 0.31
Zfp668Unknown, score: 0.11
Cd163l1Unknown, score: 0.11
Tnfrsf26Unknown, score: 0.21
MrgpreUnknown, score: 0.16
Myo16Unknown, score: 0.14
Podnl1Unknown, score: 0.2
Nanos3Unknown, score: 0.09
Zfp791Unknown, score: 0.1
Tox3Unknown, score: 0.09
Ccdc113Unknown, score: 0.45
Pskh1Unknown, score: 0.09
Pkd1l3Unknown, score: 0.04
HydinUnknown, score: 0.11
Mtss1lUnknown, score: 0.52
Cwf19l2Unknown, score: 0.34
Cntn5Unknown, score: 0.04
Kdm4dUnknown, score: 0.07
Hephl1Unknown, score: 0.07
Zfp846Unknown, score: 0.16
Dpy19l1Unknown, score: 0.26
Glb1l2Unknown, score: 0.08
Arhgap20Unknown, score: 0.19
NpatUnknown, score: 0.06
Tnfaip8l3Unknown, score: 0.31
AtrUnknown, score: 0.11
Zbtb38Unknown, score: 0.27
Dclk3Unknown, score: 0.25
Zfp937Unknown, score: 0.27
9930111J21Rik2Unknown, score: 0.11
Apol10aUnknown, score: 0.06
Tmem255aUnknown, score: 0.15
Dcaf12l1Unknown, score: 0.29
Slitrk2Unknown, score: 0.01
Nlgn3Unknown, score: 0.01
Frmpd3Unknown, score: 0.14
RragbUnknown, score: 0.11
Klf8Unknown, score: 0.29
Cnksr2Unknown, score: 0.07
Rbbp7Unknown, score: 0.12
Fat2Unknown, score: 0.11
Polr2hUnknown, score: 0.4
Amdhd2Unknown, score: 0.3
Ssh3Unknown, score: 0.33
Atg9aUnknown, score: 0.22
Pcmtd2Unknown, score: 0.24
Map7d1Unknown, score: 0.25
Wasf3Unknown, score: 0.04
Fam71f2Unknown, score: 0.1
Ccdc15Unknown, score: 0.04
Vps54Unknown, score: 0.13
Rbm47Unknown, score: 0.03
Slc36a2Unknown, score: 0.19
Onecut3Unknown, score: 0.06
RttnUnknown, score: 0.12
Atxn7Unknown, score: 0.57
Rhbdl3Unknown, score: 0.01
Kcne2Unknown, score: 0.88
VasnUnknown, score: 0.2
FktnUnknown, score: 0.09
Zfp277Unknown, score: 0.24
Mllt6Unknown, score: 0.53
MpstUnknown, score: 0.08
Prokr2Unknown, score: 0.09
Hps5Unknown, score: 0.25
Apoa1bpUnknown, score: 0.24
Emilin2Unknown, score: 0.23
Rhobtb2Unknown, score: 0.1
Oas3Unknown, score: 0.11
Oas2Unknown, score: 0.04
Oas1hUnknown, score: 0.02
Dnajc28Unknown, score: 0.44
Trpv3Unknown, score: 0.17
Ackr4Unknown, score: 0.28
ToxUnknown, score: 0.06
Odf4Unknown, score: 0.02
Ap1s3Unknown, score: 0.11
Grhl2Unknown, score: 0.14
252974Unknown, score: 0.04
Nod2Unknown, score: 0.23
Acsf3Unknown, score: 0.26
SdslUnknown, score: 0.02
Olfr543Unknown, score: 0.12
Olfr1396Unknown, score: 0.12
Olfr1420Unknown, score: 0.27
Olfr877Unknown, score: 0.02
Olfr1388Unknown, score: 0.17
Olfr1393Unknown, score: 0.05
Olfr1384Unknown, score: 0.09
Olfr99Unknown, score: 0.07
Olfr920Unknown, score: 0.17
Olfr1336Unknown, score: 0.05
Olfr559Unknown, score: 0.09
MfrpUnknown, score: 0.39
Ehd2Unknown, score: 0.04
Srgap3Unknown, score: 0.03
Prrt1Unknown, score: 0.14
FevUnknown, score: 0.08
OtosUnknown, score: 0.18
Gga3Unknown, score: 0.49
Hist1h3fUnknown, score: 0.03
Creg2Unknown, score: 0.61
Pkn3Unknown, score: 0.4
Cdk8Unknown, score: 0.31
Irak4Unknown, score: 0.09
Cyb5r4Unknown, score: 0.08
Cpne1Unknown, score: 0.12
LgsnUnknown, score: 0.25
Samd3Unknown, score: 0.11
Rnf217Unknown, score: 0.24
Scml4Unknown, score: 0.32
Ahsa2Unknown, score: 0.07
MpgUnknown, score: 0.2
Sh3pxd2bUnknown, score: 0.17
Zkscan17Unknown, score: 0.33
Alkbh5Unknown, score: 0.37
Rpl23aUnknown, score: 0.22
Ube2zUnknown, score: 0.31
Krt222Unknown, score: 0.15
Mgat5bUnknown, score: 0.14
Slc26a11Unknown, score: 0.53
Bahcc1Unknown, score: 0.41
Greb1Unknown, score: 0.09
Slc38a9Unknown, score: 0.36
Fam107aUnknown, score: 0.13
Zswim8Unknown, score: 0.18
Arhgef40Unknown, score: 0.17
Lrrc16bUnknown, score: 0.15
Rnf31Unknown, score: 0.45
Agxt2Unknown, score: 0.21
Mtmr12Unknown, score: 0.32
Klhl38Unknown, score: 0.43
Fbxo45Unknown, score: 0.13
Nrip1Unknown, score: 0.15
Pkmyt1Unknown, score: 0.19
Caskin1Unknown, score: 0.57
Wdr24Unknown, score: 0.02
Grm4Unknown, score: 0.27
Scube3Unknown, score: 0.06
Brpf3Unknown, score: 0.05
Nlrc4Unknown, score: 0.18
Ss18Unknown, score: 0.04
Sap130Unknown, score: 0.22
Zfp608Unknown, score: 0.08
4930503L19RikUnknown, score: 0.15
CtifUnknown, score: 0.09
Gpr152Unknown, score: 0.29
Nup54Unknown, score: 0.25
NfascUnknown, score: 0.12
OptcUnknown, score: 0.05
Kif26bUnknown, score: 0.14
Nbeal1Unknown, score: 0.53
Stk36Unknown, score: 0.02
PaskUnknown, score: 0.06
Fam171a1Unknown, score: 0.02
Rpl12Unknown, score: 0.27
Rtn4rl2Unknown, score: 0.17
Slc28a2Unknown, score: 0.19
Tox2Unknown, score: 0.25
Ss18l1Unknown, score: 0.15
Rtel1Unknown, score: 0.25
Jade1Unknown, score: 0.02
Nkain3Unknown, score: 0.09
Fbxl4Unknown, score: 0.1
VcpUnknown, score: 0.71
ClspnUnknown, score: 0.24
Zscan20Unknown, score: 0.08
Epb4.1Unknown, score: 0.16
Sytl1Unknown, score: 0.1
Luzp1Unknown, score: 0.09
Gpr157Unknown, score: 0.57
Pank4Unknown, score: 0.26
Rbm48Unknown, score: 0.19
Wdr86Unknown, score: 0.01
Zfp512Unknown, score: 0.3
Nat8lUnknown, score: 0.23
Golga3Unknown, score: 0.1
Rnft2Unknown, score: 0.07
Zfp664Unknown, score: 0.23
Clip2Unknown, score: 0.22
Orai2Unknown, score: 0.39
Aak1Unknown, score: 0.24
Cntn4Unknown, score: 0.38
Clec4a1Unknown, score: 0.02
Tspan12Unknown, score: 0.35
Nat14Unknown, score: 0.29
Zfp446Unknown, score: 0.44
Megf8Unknown, score: 0.84
269881Unknown, score: 0.04
Chsy1Unknown, score: 0.01
Idh2Unknown, score: 0.3
Ttll13Unknown, score: 0.08
Rccd1Unknown, score: 0.07
Adamtsl3Unknown, score: 0.26
Nup98Unknown, score: 0.69
Gsg1lUnknown, score: 0.12
Zfp747Unknown, score: 0.34
Letm2Unknown, score: 0.6
Map1sUnknown, score: 0.24
Slc35e1Unknown, score: 0.09
GcdhUnknown, score: 0.2
Lrrc36Unknown, score: 0.55
Vat1lUnknown, score: 0.32
Pcnxl2Unknown, score: 0.35
Fat3Unknown, score: 0.69
Nlrx1Unknown, score: 0.16
ClpxUnknown, score: 0.07
Ephb1Unknown, score: 0.08
Rab6bUnknown, score: 0.1
Pfkfb4Unknown, score: 0.3
Klhl18Unknown, score: 0.4
Zfp651Unknown, score: 0.15
Taf1Unknown, score: 0.04
Map3k15Unknown, score: 0.09
Mthfd1lUnknown, score: 0.28
BC048403Unknown, score: 0.11
Prr11Unknown, score: 0.02
4933408B17RikUnknown, score: 0.03
Agbl2Unknown, score: 0.27
Rpusd2Unknown, score: 0.21
Pla2g4fUnknown, score: 0.03
Shc4Unknown, score: 0.15
C2cd4dUnknown, score: 0.07
ArsjUnknown, score: 0.23
Lppr1Unknown, score: 0.4
PolnUnknown, score: 0.18
Arntl2Unknown, score: 0.15
Zfp398Unknown, score: 0.29
Irf2bp1Unknown, score: 0.3
Lrrc4bUnknown, score: 0.08
B3gnt6Unknown, score: 0.03
Acsm5Unknown, score: 0.23
Tmem255bUnknown, score: 0.02
Gins2Unknown, score: 0.27
Esyt3Unknown, score: 0.29
Eif5aUnknown, score: 0.22
Smtnl2Unknown, score: 0.2
Timd4Unknown, score: 0.36
Armc7Unknown, score: 0.08
Slfn8Unknown, score: 0.3
Rasl10bUnknown, score: 0.22
Marveld1Unknown, score: 0.05
277089Unknown, score: 0.25
Tm4sf19Unknown, score: 0.04
Kdm3bUnknown, score: 0.14
Tcfl5Unknown, score: 0.14
Prex1Unknown, score: 0.14
Trp53i11Unknown, score: 0.15
Vstm2lUnknown, score: 0.4
Gpr107Unknown, score: 0.22
Slc39a12Unknown, score: 0.58
Fam131cUnknown, score: 0.46
BC049635Unknown, score: 0.17
Depdc5Unknown, score: 0.01
Slc9a5Unknown, score: 0.23
Armcx6Unknown, score: 0.16
Vsig4Unknown, score: 0.06
Zfp385cUnknown, score: 0.03
Wfikkn2Unknown, score: 0.53
Stkld1Unknown, score: 0.62
279067Unknown, score: 0.02
Kctd19Unknown, score: 0.19
Pcdh19Unknown, score: 0.26
Nup62clUnknown, score: 0.46
Adam1aUnknown, score: 0.17
SbsnUnknown, score: 0.25
FlnbUnknown, score: 0.05
C1s2Unknown, score: 0.1
Hist1h3eUnknown, score: 0.01
Hist1h3hUnknown, score: 0.01
Hist2h3bUnknown, score: 0.17
Hist1h4cUnknown, score: 0.17
Hist1h4dUnknown, score: 0.17
Hist1h4fUnknown, score: 0.17
Hist1h4iUnknown, score: 0.17
Hist1h4jUnknown, score: 0.17
Hist1h4kUnknown, score: 0.17
Hist3h2aUnknown, score: 0.07
Hist1h2acUnknown, score: 0.07
Hist1h2adUnknown, score: 0.07
Hist1h2aeUnknown, score: 0.07
Hist1h2agUnknown, score: 0.07
Hist1h2ahUnknown, score: 0.07
Hist1h2anUnknown, score: 0.07
Hist1h2apUnknown, score: 0.07
Hist1h2abUnknown, score: 0.08
Hist1h2afUnknown, score: 0.18
Hist1h2bbUnknown, score: 0.05
Hist1h2bcUnknown, score: 0.1
Hist1h2bfUnknown, score: 0.05
Hist1h2bgUnknown, score: 0.08
Hist1h2bhUnknown, score: 0.1
Hist1h2bjUnknown, score: 0.1
Hist1h2blUnknown, score: 0.1
Hist1h2bnUnknown, score: 0.08
Hist2h2beUnknown, score: 0.07
Hist1h2aiUnknown, score: 0.1
Rpl17Unknown, score: 0.32
Gpr4Unknown, score: 0.07
Pgbd1Unknown, score: 0.16
Trim12cUnknown, score: 0.32
Pcmtd1Unknown, score: 0.23
A230050P20RikUnknown, score: 0.56
PianpUnknown, score: 0.31
Adgrl3Unknown, score: 0.22
Hs3st5Unknown, score: 0.07
C5ar2Unknown, score: 0.1
Ppm1hUnknown, score: 0.37
Lrtm1Unknown, score: 0.28
Cxxc4Unknown, score: 0.42
Wdr59Unknown, score: 0.15
9530053A07RikUnknown, score: 0.12
NrcamUnknown, score: 0.14
Syt15Unknown, score: 0.41
Pced1aUnknown, score: 0.14
PdprUnknown, score: 0.35
Dusp4Unknown, score: 0.25
Nwd1Unknown, score: 0.12
Lig4Unknown, score: 0.17
Celf5Unknown, score: 0.27
Hif1anUnknown, score: 0.02
Zfp653Unknown, score: 0.02
Zfp944Unknown, score: 0.16
Slc25a40Unknown, score: 0.23
AgmoUnknown, score: 0.05
Cep295Unknown, score: 0.01
Frmd6Unknown, score: 0.15
Ablim3Unknown, score: 0.02
Cacna2d4Unknown, score: 0.42
Zfyve27Unknown, score: 0.03
Mpzl3Unknown, score: 0.55
Zfp865Unknown, score: 0.02
Igf2bp2Unknown, score: 0.15
Slc22a30Unknown, score: 0.13
Smyd4Unknown, score: 0.23
Cobll1Unknown, score: 0.15
Zcchc7Unknown, score: 0.13
E030030I06RikUnknown, score: 0.12
OacylUnknown, score: 0.16
Vwc2Unknown, score: 0.39
Sbf2Unknown, score: 0.24
A530016L24RikUnknown, score: 0.05
Taf2Unknown, score: 0.28
Flad1Unknown, score: 0.52
Ercc6Unknown, score: 0.15
Jph4Unknown, score: 0.17
A630001G21RikUnknown, score: 0.39
Tmem198Unknown, score: 0.07
Nceh1Unknown, score: 0.19
Fstl4Unknown, score: 0.1
Rnf222Unknown, score: 0.11
Exph5Unknown, score: 0.74
Olfml2bUnknown, score: 0.06
6430550D23RikUnknown, score: 0.36
Rps6kc1Unknown, score: 0.02
DgkiUnknown, score: 0.36
BC049715Unknown, score: 0.17
Ptpn7Unknown, score: 0.33
B430306N03RikUnknown, score: 0.09
Zdhhc17Unknown, score: 0.1
Zmat4Unknown, score: 0.4
Tacc1Unknown, score: 0.05
Mettl20Unknown, score: 0.24
Tmem91Unknown, score: 0.01
Ddx11Unknown, score: 0.09
Catsperg1Unknown, score: 0.27
Ttll5Unknown, score: 0.04
March3Unknown, score: 0.01
Dlec1Unknown, score: 0.44
ScaiUnknown, score: 0.27
Iqcb1Unknown, score: 0.19
Rnf152Unknown, score: 0.17
Hist4h4Unknown, score: 0.09
Lypd6Unknown, score: 0.1
Ric3Unknown, score: 0.26
FryUnknown, score: 0.29
CenptUnknown, score: 0.07
ItpkbUnknown, score: 0.13
Klri2Unknown, score: 0.13
GchfrUnknown, score: 0.2
Lmbrd2Unknown, score: 0.03
Cachd1Unknown, score: 0.15
Vps13cUnknown, score: 0.55
Slc35e2Unknown, score: 0.25
Dennd5bUnknown, score: 0.07
Atp8b5Unknown, score: 0.32
Idi2Unknown, score: 0.23
Tmem88bUnknown, score: 0.24
Phf8Unknown, score: 0.41
Strip2Unknown, score: 0.27
Dopey1Unknown, score: 0.19
Cyb5r2Unknown, score: 0.35
Pgap3Unknown, score: 0.06
D5Ertd579eUnknown, score: 0.05
Casc1Unknown, score: 0.18
Cass4Unknown, score: 0.13
Iffo1Unknown, score: 0.01
Samd12Unknown, score: 0.07
Bend6Unknown, score: 0.23
Atp2b3Unknown, score: 0.38
Abi3bpUnknown, score: 0.02
Mysm1Unknown, score: 0.03
Trappc11Unknown, score: 0.37
Lingo4Unknown, score: 0.25
Mdga2Unknown, score: 0.05
Chd7Unknown, score: 0.44
Zhx3Unknown, score: 0.69
Ifitm10Unknown, score: 0.05
Samd5Unknown, score: 0.25
Negr1Unknown, score: 0.51
Spata33Unknown, score: 0.04
Cdh10Unknown, score: 0.02
PisdUnknown, score: 0.02
Lrif1Unknown, score: 0.1
Tpm4Unknown, score: 0.17
Hist1h4aUnknown, score: 0.17
Upf2Unknown, score: 0.27
Tnfsf15Unknown, score: 0.17
Ppip5k1Unknown, score: 0.07
Wisp3Unknown, score: 0.36
Mettl24Unknown, score: 0.15
Ppfia2Unknown, score: 0.03
Frs2Unknown, score: 0.14
Cyb5d1Unknown, score: 0.07
Dnah2Unknown, score: 0.07
ScimpUnknown, score: 0.57
Med13Unknown, score: 0.05
Hsf5Unknown, score: 0.28
Prpf39Unknown, score: 0.02
Slc39a9Unknown, score: 0.32
Trmt61aUnknown, score: 0.49
Mast4Unknown, score: 0.41
Zmiz1Unknown, score: 0.16
Parp4Unknown, score: 0.21
KcnrgUnknown, score: 0.08
Apol11bUnknown, score: 0.07
Ep300Unknown, score: 0.08
Tubgcp6Unknown, score: 0.19
Rab26Unknown, score: 0.16
Hs3st6Unknown, score: 0.24
MslnlUnknown, score: 0.1
Ubash3aUnknown, score: 0.06
Zfp414Unknown, score: 0.15
9830107B12RikUnknown, score: 0.19
A530064D06RikUnknown, score: 0.01
Treml2Unknown, score: 0.05
Arhgef37Unknown, score: 0.14
Zfp236Unknown, score: 0.3
Atg2aUnknown, score: 0.04
Cpa6Unknown, score: 0.23
Abi2Unknown, score: 0.36
Rbm44Unknown, score: 0.27
Dennd1bUnknown, score: 0.02
TnnUnknown, score: 0.38
Syt14Unknown, score: 0.13
1700101E01RikUnknown, score: 0.36
NostrinUnknown, score: 0.2
Myo3bUnknown, score: 0.04
Pla2g4eUnknown, score: 0.37
Lcmt2Unknown, score: 0.03
Ctdspl2Unknown, score: 0.21
Gm826Unknown, score: 0.15
Zfp335Unknown, score: 0.2
Fat4Unknown, score: 0.01
Fnip2Unknown, score: 0.23
Fam102bUnknown, score: 0.19
PigkUnknown, score: 0.13
Tmem67Unknown, score: 0.08
AI464131Unknown, score: 0.13
Frem1Unknown, score: 0.17
Usp24Unknown, score: 0.27
Acot11Unknown, score: 0.51
Col8a2Unknown, score: 0.07
Fam185aUnknown, score: 0.39
Adamts3Unknown, score: 0.13
Hfm1Unknown, score: 0.1
Ccdc63Unknown, score: 0.22
Tmem120bUnknown, score: 0.37
Vps37bUnknown, score: 0.06
Mblac1Unknown, score: 0.17
Gal3st4Unknown, score: 0.22
Sdk1Unknown, score: 0.03
Pon2Unknown, score: 0.18
Thsd7aUnknown, score: 0.16
Wipf3Unknown, score: 0.24
Tmcc1Unknown, score: 0.19
Zfp78Unknown, score: 0.26
Zc3h4Unknown, score: 0.34
Ceacam16Unknown, score: 0.24
Saxo2Unknown, score: 0.16
Trim66Unknown, score: 0.3
B4galnt4Unknown, score: 0.02
Ctxn1Unknown, score: 0.33
Zfp866Unknown, score: 0.12
Rnf150Unknown, score: 0.5
Adgrl1Unknown, score: 0.18
DhpsUnknown, score: 0.1
4933402J07RikUnknown, score: 0.28
Arhgap32Unknown, score: 0.02
Pate2Unknown, score: 0.41
Dixdc1Unknown, score: 0.34
AI593442Unknown, score: 0.23
Slc9a9Unknown, score: 0.35
GmppbUnknown, score: 0.02
Tgm4Unknown, score: 0.04
Rgag4Unknown, score: 0.11
UprtUnknown, score: 0.13
Krt78Unknown, score: 0.09
Zdhhc23Unknown, score: 0.31
Tigd3Unknown, score: 0.02
Nanos1Unknown, score: 0.21
Zmynd12Unknown, score: 0.3
Tfap2eUnknown, score: 0.29
Proser3Unknown, score: 0.31
Gpd1lUnknown, score: 0.04
Mamld1Unknown, score: 0.23
Mia2Unknown, score: 0.28
Cog3Unknown, score: 0.03
Gpr21Unknown, score: 0.08
CntlnUnknown, score: 0.09
Fkbp15Unknown, score: 0.2
Trim65Unknown, score: 0.25
Myo1dUnknown, score: 0.15
NalcnUnknown, score: 0.2
Kdm7aUnknown, score: 0.02
Caps2Unknown, score: 0.2
Gjd3Unknown, score: 0.33
TxlngUnknown, score: 0.13
Adam32Unknown, score: 0.11
Prune2Unknown, score: 0.12
Pcdha2Unknown, score: 0.12
Pcdhac1Unknown, score: 0.12
Sfmbt2Unknown, score: 0.67
353328Unknown, score: 0.2
Tmc4Unknown, score: 0.07
Myo18aUnknown, score: 0.01
Hist1h3aUnknown, score: 0.56
Zranb1Unknown, score: 0.14
Speer4dUnknown, score: 0.02
Col27a1Unknown, score: 0.11
TxlnbUnknown, score: 0.42
MafaUnknown, score: 0.02
Morn2Unknown, score: 0.02
Bpifb3Unknown, score: 0.13
Serf2Unknown, score: 0.23
Fastkd5Unknown, score: 0.07
IntuUnknown, score: 0.6
HecaUnknown, score: 0.23
Lemd3Unknown, score: 0.05
Cnrip1Unknown, score: 0.14
CcnjlUnknown, score: 0.21
Shisa6Unknown, score: 0.29
Spata22Unknown, score: 0.54
Tlcd2Unknown, score: 0.04
Scarf1Unknown, score: 0.31
Milr1Unknown, score: 0.28
Lsmem1Unknown, score: 0.05
Serpina11Unknown, score: 0.13
BegainUnknown, score: 0.34
Serpinb1cUnknown, score: 0.05
Lyrm4Unknown, score: 0.38
Tmem171Unknown, score: 0.06
Zfp395Unknown, score: 0.1
Lmo7Unknown, score: 0.07
Tmem106cUnknown, score: 0.34
Nckap5lUnknown, score: 0.06
ZfatUnknown, score: 0.13
Kmt2dUnknown, score: 0.14
Zfp229Unknown, score: 0.11
NpwUnknown, score: 0.09
Ccdc78Unknown, score: 0.19
Tbc1d22bUnknown, score: 0.18
H2-Eb2Unknown, score: 0.35
Gm21981Unknown, score: 0.35
Arl14eplUnknown, score: 0.28
Ccdc68Unknown, score: 0.45
Tmem151aUnknown, score: 0.46
Ap5b1Unknown, score: 0.07
Tmem237Unknown, score: 0.13
HjurpUnknown, score: 0.31
E030010N08RikUnknown, score: 0.22
Kif14Unknown, score: 0.24
Nsl1Unknown, score: 0.02
Tmem182Unknown, score: 0.1
Spag6lUnknown, score: 0.4
Mamdc4Unknown, score: 0.17
Gm996Unknown, score: 0.36
Pabpc1lUnknown, score: 0.14
Zfp408Unknown, score: 0.35
Gpr176Unknown, score: 0.04
Nr1h5Unknown, score: 0.19
Rxfp1Unknown, score: 0.09
Mroh7Unknown, score: 0.07
Gm1661Unknown, score: 0.05
Xkr8Unknown, score: 0.18
Tmem240Unknown, score: 0.14
5031410I06RikUnknown, score: 0.16
Rbm33Unknown, score: 0.02
AtraidUnknown, score: 0.11
Gm1673Unknown, score: 0.07
Cep135Unknown, score: 0.17
Fbrsl1Unknown, score: 0.18
Zcwpw1Unknown, score: 0.41
Nxpe5Unknown, score: 0.18
N4bp2l2Unknown, score: 0.08
4930590J08RikUnknown, score: 0.21
Tsen2Unknown, score: 0.1
Clec4b2Unknown, score: 0.17
2700089E24RikUnknown, score: 0.08
Sbk3Unknown, score: 0.45
Sbk2Unknown, score: 0.11
2310014L17RikUnknown, score: 0.38
GiprUnknown, score: 0.07
Alg8Unknown, score: 0.03
Taok2Unknown, score: 0.22
ItgadUnknown, score: 0.27
6430531B16RikUnknown, score: 0.16
Cep44Unknown, score: 0.43
Cnep1r1Unknown, score: 0.69
Gse1Unknown, score: 0.04
Pabpn1lUnknown, score: 0.04
Ces1bUnknown, score: 0.25
Ces3aUnknown, score: 0.54
Crtc1Unknown, score: 0.25
Prdm10Unknown, score: 0.18
Foxr1Unknown, score: 0.53
Odf3l1Unknown, score: 0.38
Gm1123Unknown, score: 0.19
382099Unknown, score: 0.28
Zkscan7Unknown, score: 0.41
Brwd3Unknown, score: 0.14
A830080D01RikUnknown, score: 0.29
Atxn7l3bUnknown, score: 0.1
Tmed8Unknown, score: 0.18
Kctd16Unknown, score: 0.1
Aim2Unknown, score: 0.44
Ankrd63Unknown, score: 0.22
Slc25a34Unknown, score: 0.02
Fam47eUnknown, score: 0.26
Nova2Unknown, score: 0.29
Zfp667Unknown, score: 0.34
Kng2Unknown, score: 0.22
Rnf39Unknown, score: 0.27
CdsnUnknown, score: 0.12
Slitrk3Unknown, score: 0.08
Tmtc1Unknown, score: 0.13
Znrf2Unknown, score: 0.39
Zhx2Unknown, score: 0.07
Ugt1a7cUnknown, score: 0.6
Ugt1a1Unknown, score: 0.21
Scn4bUnknown, score: 0.15
Flrt2Unknown, score: 0.36
BC052040Unknown, score: 0.01
Fam84bUnknown, score: 0.2
Tcaf3Unknown, score: 0.32
Plcxd1Unknown, score: 0.53
Cyp2c50Unknown, score: 0.11
Olfr1383Unknown, score: 0.09
Bex4Unknown, score: 0.25
Tmem189Unknown, score: 0.02
Taf9bUnknown, score: 0.07
BC051142Unknown, score: 0.29
Zfp941Unknown, score: 0.17
BC031181Unknown, score: 0.43
Baz2bUnknown, score: 0.12
Zfp738Unknown, score: 0.04
BC024978Unknown, score: 0.21
Zyg11bUnknown, score: 0.26
Hnrnph3Unknown, score: 0.14
Tmprss9Unknown, score: 0.03
4930404N11RikUnknown, score: 0.06
Gm11541Unknown, score: 0.02
Trim80Unknown, score: 0.17
Zscan26Unknown, score: 0.47
Prr7Unknown, score: 0.1
Rslcan18Unknown, score: 0.02
Gprin2Unknown, score: 0.18
OtulinUnknown, score: 0.21
432950Unknown, score: 0.08
433064Unknown, score: 0.08
Ly6g6fUnknown, score: 0.06
Acsl5Unknown, score: 0.2
433319Unknown, score: 0.04
Creg1Unknown, score: 0.02
Gm13547Unknown, score: 0.13
Maml3Unknown, score: 0.2
Spag8Unknown, score: 0.04
Hdac1Unknown, score: 0.16
Gm13212Unknown, score: 0.13
Gm13154Unknown, score: 0.49
Rnf207Unknown, score: 0.51
Nom1Unknown, score: 0.24
Ociad2Unknown, score: 0.21
Lrrc8bUnknown, score: 0.12
Mn1Unknown, score: 0.44
Fam222aUnknown, score: 0.68
Tmem178bUnknown, score: 0.21
434050Unknown, score: 0.2
Pnmal2Unknown, score: 0.08
Gm5595Unknown, score: 0.06
Slc28a1Unknown, score: 0.07
WhammUnknown, score: 0.19
Lrrc32Unknown, score: 0.24
Trim34bUnknown, score: 0.1
2610020H08RikUnknown, score: 0.11
Trim72Unknown, score: 0.06
Zfp560Unknown, score: 0.16
Gm5617Unknown, score: 0.24
434428Unknown, score: 0.14
Rpl10Unknown, score: 0.21
434624Unknown, score: 0.14
Ccdc160Unknown, score: 0.15
434782Unknown, score: 0.01
Gm5640Unknown, score: 0.64
Dupd1Unknown, score: 0.22
Rufy4Unknown, score: 0.05
FcrlbUnknown, score: 0.27
Tnni3kUnknown, score: 0.43
Lrp3Unknown, score: 0.16
Fam92bUnknown, score: 0.2
Gpr62Unknown, score: 0.1
Arhgef15Unknown, score: 0.07
A830005F24RikUnknown, score: 0.17
Rab44Unknown, score: 0.28
Znhit3Unknown, score: 0.28
Fbxl7Unknown, score: 0.45
Zfp213Unknown, score: 0.31
Zbtb9Unknown, score: 0.12
Armcx5Unknown, score: 0.34
Xkr4Unknown, score: 0.3
AcdUnknown, score: 0.1
Ear2Unknown, score: 0.13
Trcg1Unknown, score: 0.19
1190007I07RikUnknown, score: 0.16
Myh13Unknown, score: 0.13
Arhgap27Unknown, score: 0.02
Bdp1Unknown, score: 0.11
H3f3aUnknown, score: 0.26
Cma2Unknown, score: 0.21
TdgUnknown, score: 0.2
Fam186bUnknown, score: 0.49
KalrnUnknown, score: 0.28
ArsiUnknown, score: 0.47
Ccdc141Unknown, score: 0.29
Zfp345Unknown, score: 0.16
Arhgap40Unknown, score: 0.18
545487Unknown, score: 0.18
Erich6Unknown, score: 0.36
Mterf1aUnknown, score: 0.1
Pilrb2Unknown, score: 0.42
546052Unknown, score: 0.26
546058Unknown, score: 0.39
Gramd2Unknown, score: 0.17
Prrg1Unknown, score: 0.23
Klhl33Unknown, score: 0.29
Cyp26c1Unknown, score: 0.02
Gm13178Unknown, score: 0.22
Parp14Unknown, score: 0.42
Gm6034Unknown, score: 0.22
Btnl2Unknown, score: 0.45
Gpr17Unknown, score: 0.03
Serinc4Unknown, score: 0.04
Zmynd15Unknown, score: 0.1
Tceal3Unknown, score: 0.32
Ndufs5Unknown, score: 0.2
Zfp551Unknown, score: 0.02
Zcchc17Unknown, score: 0.06
Klf14Unknown, score: 0.08
619937Unknown, score: 0.21
619941Unknown, score: 0.36
619973Unknown, score: 0.03
C130026I21RikUnknown, score: 0.23
620499Unknown, score: 0.02
621542Unknown, score: 0.23
Rpl32Unknown, score: 0.02
621705Unknown, score: 0.09
Nutf2Unknown, score: 0.06
622335Unknown, score: 0.05
Arhgef26Unknown, score: 0.08
Zfp827Unknown, score: 0.08
623174Unknown, score: 0.72
Tmem200bUnknown, score: 0.02
Rad54bUnknown, score: 0.13
Gm14137Unknown, score: 0.34
Gm6484Unknown, score: 0.03
Gpx4Unknown, score: 0.08
Rpl30Unknown, score: 0.02
Tmem236Unknown, score: 0.11
Gm6583Unknown, score: 0.13
Fam43bUnknown, score: 0.64
626048Unknown, score: 0.09
Zfp951Unknown, score: 0.1
Gbp10Unknown, score: 0.15
Gm6710Unknown, score: 0.11
Klk13Unknown, score: 0.2
Etohi1Unknown, score: 0.02
Morf4l1Unknown, score: 0.11
Gm6792Unknown, score: 0.17
Gm4631Unknown, score: 0.31
628161Unknown, score: 0.05
Lipo4Unknown, score: 0.29
628648Unknown, score: 0.1
628794Unknown, score: 0.12
OtoglUnknown, score: 0.12
Dact3Unknown, score: 0.2
Lin52Unknown, score: 0.15
Zfp808Unknown, score: 0.05
631033Unknown, score: 0.02
631040Unknown, score: 0.17
631286Unknown, score: 0.18
Fer1l6Unknown, score: 0.21
Vmn2r18Unknown, score: 0.08
633406Unknown, score: 0.08
633417Unknown, score: 0.41
Susd1Unknown, score: 0.03
635087Unknown, score: 0.14
636306Unknown, score: 0.04
Zfp964Unknown, score: 0.39
636901Unknown, score: 0.3
Vmn2r3Unknown, score: 0.07
Nlrp1bUnknown, score: 0.19
637796Unknown, score: 0.49
638399Unknown, score: 0.26
639541Unknown, score: 0.23
639931Unknown, score: 0.13
640370Unknown, score: 0.2
640549Unknown, score: 0.01
Nrbf2Unknown, score: 0.14
Tomm40lUnknown, score: 0.46
Tmem243Unknown, score: 0.12
654450Unknown, score: 0.13
Defb26Unknown, score: 0.11
Defb25Unknown, score: 0.2
Tctn1Unknown, score: 0.35
Sdr39u1Unknown, score: 0.02
Angptl7Unknown, score: 0.05
Gcnt7Unknown, score: 0.51
Nova1Unknown, score: 0.47
Tmem238Unknown, score: 0.08
Gm14393Unknown, score: 0.23
Gm14326Unknown, score: 0.02
TnikUnknown, score: 0.52
Srp54bUnknown, score: 0.1
Gm14326Unknown, score: 0.15
CsprsUnknown, score: 0.07
Hist1h2aoUnknown, score: 0.64
665463Unknown, score: 0.04
665503Unknown, score: 0.64
665522Unknown, score: 0.49
Mthfd2lUnknown, score: 0.64
665579Unknown, score: 0.64
Bod1lUnknown, score: 0.24
666548Unknown, score: 0.07
666738Unknown, score: 0.09
Rpl23aUnknown, score: 0.1
Bend4Unknown, score: 0.17
Pnp2Unknown, score: 0.15
9930111J21Rik1Unknown, score: 0.11
Gm14446Unknown, score: 0.02
667414Unknown, score: 0.16
Zfp600Unknown, score: 0.27
667739Unknown, score: 0.06
667803Unknown, score: 0.14
Trim5Unknown, score: 0.23
667846Unknown, score: 0.23
667847Unknown, score: 0.15
Gm8898Unknown, score: 0.04
Gm14434Unknown, score: 0.03
668114Unknown, score: 0.73
Ccdc85cUnknown, score: 0.16
ZxdbUnknown, score: 0.06
Efr3bUnknown, score: 0.08
Dgat2l6Unknown, score: 0.29
668455Unknown, score: 0.05
668830Unknown, score: 0.27
Myh7bUnknown, score: 0.32
670211Unknown, score: 0.64
671242Unknown, score: 0.15
Parp10Unknown, score: 0.64
674846Unknown, score: 0.37
675521Unknown, score: 0.04
Zfp605Unknown, score: 0.03
677113Unknown, score: 0.06
677654Unknown, score: 0.15
Gm9733Unknown, score: 0.06
TomtUnknown, score: 0.04
Pydc3Unknown, score: 0.02
Mfap1bUnknown, score: 0.01
Dnajc3Unknown, score: 0.03
Fam129cUnknown, score: 0.74
Rsph3bUnknown, score: 0.36
Fam174bUnknown, score: 0.4
Gm11744Unknown, score: 0.15
Isg15Unknown, score: 0.2
Gm14548Unknown, score: 0.27
Mup9Unknown, score: 0.14
Gm1979Unknown, score: 0.64
100038969Unknown, score: 0.26
Mup10Unknown, score: 0.14
Gm10471Unknown, score: 0.14
Mup14Unknown, score: 0.17
Gm14295Unknown, score: 0.14
Ccdc152Unknown, score: 0.19
Mup16Unknown, score: 0.18
Tmem254cUnknown, score: 0.19
100039252Unknown, score: 0.18
100039478Unknown, score: 0.17
100039484Unknown, score: 0.17
100039674Unknown, score: 0.1
MthfslUnknown, score: 0.4
100039731Unknown, score: 0.35
100039794Unknown, score: 0.12
100040018Unknown, score: 0.21
CsprsUnknown, score: 0.06
100040298Unknown, score: 0.32
Dynlt1cUnknown, score: 0.19
Gm10408Unknown, score: 0.1
100041194Unknown, score: 0.17
Ndufb4Unknown, score: 0.05
ErmardUnknown, score: 0.23
Zkscan16Unknown, score: 0.12
Gm13157Unknown, score: 0.31
GapdhUnknown, score: 0.31
Gm3646Unknown, score: 0.21
100042074Unknown, score: 0.06
100042100Unknown, score: 0.24
Gm3696Unknown, score: 0.32
100042235Unknown, score: 0.18
Gsta1Unknown, score: 0.11
Ndufb4Unknown, score: 0.26
100042625Unknown, score: 0.09
Prdm11Unknown, score: 0.23
Gm4070Unknown, score: 0.1
100043027Unknown, score: 0.12
100043059Unknown, score: 0.3
Gm11710Unknown, score: 0.3
100043217Unknown, score: 0.35
Gm14305Unknown, score: 0.14
Gm4477Unknown, score: 0.31
100043508Unknown, score: 0.21
SrcapUnknown, score: 0.12
100043670Unknown, score: 0.36
Rbx1Unknown, score: 0.31
100043695Unknown, score: 0.19
Zfp831Unknown, score: 0.15
Josd1Unknown, score: 0.12
100043805Unknown, score: 0.1
Gm4724Unknown, score: 0.05
100044322Unknown, score: 0.15
100044374Unknown, score: 0.09
100045191Unknown, score: 0.03
100045367Unknown, score: 0.49
100045688Unknown, score: 0.03
100045924Unknown, score: 0.12
100045968Unknown, score: 0.22
100046034Unknown, score: 0.25
100046048Unknown, score: 0.32
100046119Unknown, score: 0.18
100046289Unknown, score: 0.1
100046628Unknown, score: 0.5
100046650Unknown, score: 0.35
100046684Unknown, score: 0.22
100047468Unknown, score: 0.21
100047518Unknown, score: 0.32
100048119Unknown, score: 0.06
100048268Unknown, score: 0.31
100048447Unknown, score: 0.11
100048557Unknown, score: 0.09
100048613Unknown, score: 0.27
Dnah7cUnknown, score: 0.41
Sco2Unknown, score: 0.04
Gm14306Unknown, score: 0.02
Ttll2Unknown, score: 0.32
Fam150bUnknown, score: 0.08
100502680Unknown, score: 0.06
Kifc1Unknown, score: 0.3
100502777Unknown, score: 0.43
Rpl37Unknown, score: 0.07
Epg5Unknown, score: 0.14
Ccdc13Unknown, score: 0.05
Gm684Unknown, score: 0.09
Pdzd7Unknown, score: 0.06
Armcx4Unknown, score: 0.32
100503055Unknown, score: 0.52
Klhl3Unknown, score: 0.07
100503125Unknown, score: 0.07
100503180Unknown, score: 0.18
100503235Unknown, score: 0.09
Zfp534Unknown, score: 0.23
Hbb-bsUnknown, score: 0.3
100503799Unknown, score: 0.29
Ccdc149Unknown, score: 0.05
Gm8923Unknown, score: 0.2
Prss51Unknown, score: 0.24
MicalclUnknown, score: 0.02
Ifi203Unknown, score: 0.37
Prr22Unknown, score: 0.49
Gm3194Unknown, score: 0.19
3425401B19RikUnknown, score: 0.04
Cisd3Unknown, score: 0.19
Atg14Unknown, score: 0.49
TmppeUnknown, score: 0.41
100504872Unknown, score: 0.04
100504912Unknown, score: 0.23
100504922Unknown, score: 0.02
100504934Unknown, score: 0.27
100504959Unknown, score: 0.32
100504968Unknown, score: 0.06
100504983Unknown, score: 0.14
100504988Unknown, score: 0.1
100505015Unknown, score: 0.39
100505031Unknown, score: 0.23
100505160Unknown, score: 0.05
100505283Unknown, score: 0.12
Gm11127Unknown, score: 0.56
Mup18Unknown, score: 0.27
100861947Unknown, score: 0.26
100862012Unknown, score: 0.04
Tmed2Unknown, score: 0.19
100862206Unknown, score: 0.64
100862223Unknown, score: 0.23
RP23-56M18.5Unknown, score: 0.14
RP23-56M18.6Unknown, score: 0.18
Gm21698Unknown, score: 0.52
100862401Unknown, score: 0.1
100862433Unknown, score: 0.36
Rpl23Unknown, score: 0.36
Polr2kUnknown, score: 0.08
100862459Unknown, score: 0.13
Gm13154Unknown, score: 0.12
100862515Unknown, score: 0.48
100862570Unknown, score: 0.24
100862586Unknown, score: 0.07
100862595Unknown, score: 0.22
100862604Unknown, score: 0.17
101055633Unknown, score: 0.17
101055647Unknown, score: 0.09
101055652Unknown, score: 0.03
Lipo2Unknown, score: 0.31
101055761Unknown, score: 0.41
101055764Unknown, score: 0.02
101055828Unknown, score: 0.09
101055829Unknown, score: 0.35
101055997Unknown, score: 0.2
101055998Unknown, score: 0.06
101056010Unknown, score: 0.11
101056089Unknown, score: 0.27
101056094Unknown, score: 0.08
101056131Unknown, score: 0.1
101056140Unknown, score: 0.23
101056240Unknown, score: 0.13
101056348Unknown, score: 0.06
101056352Unknown, score: 0.13
101056370Unknown, score: 0.39
101056500Unknown, score: 0.01
101056514Unknown, score: 0.11
101056542Unknown, score: 0.06
101056559Unknown, score: 0.17
101056596Unknown, score: 0.31
101056614Unknown, score: 0.02
101056649Unknown, score: 0.14
101056658Unknown, score: 0.11
101056659Unknown, score: 0.05
101056688Unknown, score: 0.41
101056691Unknown, score: 0.23

## Help | Hide | Top Help | Show | Top Conditions

### HELP

Conditions in the module, given in the same order as on the expression
plot above. Red color means over-expression, green under-expression in
the given condition.

The barplot below shows the condition (sample) scores. A separate bar
is shown for each sample, its height is the corresponding score of the
sample in the module. The red and green numbers on the bars are the
sample scores expressed in percents, i.e. 100% is 1.0.

The red and green lines show the module thresholds, samples above
the red line and below the green line are included in the module.

The different experiments that were part of the study, are separated
by dashed vertical lines.

— Click on the *Help* button again to close this help window.

| Id |
| --- |
| BALB\_cByJ-CTR\_144 |
| BALB\_cJ-ATE\_86 |
| C57BL\_6J-ATE\_95 |
| BALB\_cJ-CTR\_85 |
| BALB\_cJ-ATE\_82 |
| C57BL\_6J-ATE\_106 |
| BALB\_cJ-ISO\_84 |
| BALB\_cJ-ISO\_83 |
| BALB\_cByJ-ATE\_157 |
| CBA\_J-ATE\_132 |
| CBA\_J-CTR\_130 |
| DBA\_2J-ATE\_5 |
| DBA\_2J-ATE\_6 |
| CBA\_J-ATE\_127 |
| DBA\_2J-CTR\_9 |
| BALB\_cByJ-CTR\_143 |
| CBA\_J-ATE\_131 |
| DBA\_2J-CTR\_1 |
| CBA\_J-CTR\_125 |
| DBA\_2J-CTR\_2 |
| I\_LnJ-ATE\_154 |
| SM\_J-ATE\_43 |
| C57BLKS\_J-ATE\_108 |
| C57BLKS\_J-ATE\_113 |
| CBA\_J-CTR\_126 |
| C57BLKS\_J-CTR\_110 |
| BALB\_cByJ-CTR\_158 |
| SM\_J-ATE\_42 |
| BALB\_cByJ-ATE\_151 |
| SM\_J-CTR\_40 |
| I\_LnJ-CTR\_153 |
| SM\_J-ATE\_49 |
| SM\_J-CTR\_48 |
| SM\_J-CTR\_41 |
| C58\_J-ATE\_36 |
| C3H\_HeJ-ATE\_61 |
| C3H\_HeJ-ATE\_67 |
| I\_LnJ-ATE\_146 |
| C58\_J-CTR\_32 |
| C57BLKS\_J-ATE\_112 |
| BALB\_cJ-CTR\_79 |
| BALB\_cJ-ATE\_80 |
| DBA\_2J-ISO\_8 |
| C58\_J-CTR\_34 |
| FVB\_NJ-ATE\_53 |
| C57BL\_6J-ATE\_109 |
| C57BL\_6J-CTR\_39 |
| DBA\_2J-ATE\_3 |
| C57BLKS\_J-CTR\_111 |
| I\_LnJ-CTR\_150 |
| BALB\_cByJ-ATE\_160 |
| I\_LnJ-ATE\_148 |
| CBA\_J-ISO\_133 |
| C3H\_HeJ-ATE\_66 |
| C3H\_HeJ-CTR\_63 |
| A\_J-ATE\_117 |
| C58\_J-ATE\_30 |
| FVB\_NJ-CTR\_51 |
| LP\_J-ATE\_101 |
| C58\_J-ATE\_29 |
| A\_J-ATE\_116 |
| C3H\_HeJ-CTR\_64 |
| I\_LnJ-CTR\_147 |
| FVB\_NJ-CTR\_52 |
| A\_J-ATE\_122 |
| C58\_J-CTR\_28 |
| NOD\_ShiLtJ-ATE\_22 |
| CBA\_J-ISO\_129 |
| LP\_J-CTR\_104 |
| CBA\_J-ISO\_128 |
| LP\_J-CTR\_100 |
| DBA\_2J-ISO\_7 |
| C3H\_HeJ-CTR\_65 |
| C57BLKS\_J-CTR\_107 |
| NOD\_ShiLtJ-ATE\_23 |
| C57BL\_6J-CTR\_38 |
| A\_J-CTR\_120 |
| PL\_J-CTR\_70 |
| NOD\_ShiLtJ-ATE\_27 |
| NOD\_ShiLtJ-CTR\_20 |
| PL\_J-ATE\_75 |
| A\_J-CTR\_121 |
| LP\_J-ATE\_98 |
| FVB\_NJ-ATE\_60 |
| A\_J-CTR\_119 |
| NOD\_ShiLtJ-CTR\_21 |
| C57BL\_6J-CTR\_35 |
| BALB\_cByJ-ISO\_159 |
| LP\_J-CTR\_97 |
| PL\_J-ATE\_76 |
| FVB\_NJ-ATE\_57 |
| BALB\_cByJ-ISO\_145 |
| LP\_J-ATE\_105 |
| C58\_J-ISO\_37 |
| I\_LnJ-ISO\_155 |
| SM\_J-ISO\_46 |
| SJL\_J-CTR\_16 |
| C58\_J-ISO\_33 |
| SJL\_J-ATE\_18 |
| SJL\_J-CTR\_10 |
| PL\_J-ATE\_71 |
| C3H\_HeJ-ISO\_69 |
| SJL\_J-ATE\_17 |
| SJL\_J-ATE\_11 |
| I\_LnJ-ISO\_152 |
| SWR\_J-ATE\_139 |
| C58\_J-ISO\_31 |
| SM\_J-ISO\_45 |
| BALB\_cByJ-ISO\_156 |
| C57BL\_6J-ISO\_50 |
| C57BL\_6J-ISO\_59 |
| PL\_J-CTR\_72 |
| A\_J-ISO\_124 |
| C3H\_HeJ-ISO\_68 |
| C57BL\_6J-ISO\_47 |
| SM\_J-ISO\_44 |
| NZB\_BLNJ-ATE\_88 |
| BALB\_cJ-ISO\_81 |
| SWR\_J-ATE\_140 |
| NZB\_BLNJ-CTR\_93 |
| PL\_J-CTR\_74 |
| I\_LnJ-ISO\_149 |
| C57BLKS\_J-ISO\_114 |
| SJL\_J-CTR\_15 |
| NOD\_ShiLtJ-CTR\_19 |
| NZB\_BLNJ-ATE\_94 |
| NZB\_BLNJ-CTR\_87 |
| FVB\_NJ-CTR\_56 |
| DBA\_2J-ISO\_4 |
| C57BLKS\_J-ISO\_115 |
| NZB\_BLNJ-ATE\_89 |
| A\_J-ISO\_123 |
| SWR\_J-CTR\_138 |
| SWR\_J-ATE\_135 |
| FVB\_NJ-ISO\_54 |
| C3H\_HeJ-ISO\_62 |
| NZB\_BLNJ-CTR\_92 |
| LP\_J-ISO\_99 |
| SJL\_J-ISO\_12 |
| SWR\_J-CTR\_137 |
| SWR\_J-CTR\_134 |
| PL\_J-ISO\_77 |
| LP\_J-ISO\_103 |
| NOD\_ShiLtJ-ISO\_25 |
| A\_J-ISO\_118 |
| PL\_J-ISO\_78 |
| SWR\_J-ISO\_142 |
| SJL\_J-ISO\_13 |
| SWR\_J-ISO\_141 |
| SJL\_J-ISO\_14 |
| FVB\_NJ-ISO\_58 |
| PL\_J-ISO\_73 |
| LP\_J-ISO\_102 |
| NOD\_ShiLtJ-ISO\_24 |
| FVB\_NJ-ISO\_55 |
| NOD\_ShiLtJ-ISO\_26 |
| NZB\_BLNJ-ISO\_96 |
| NZB\_BLNJ-ISO\_91 |
| NZB\_BLNJ-ISO\_90 |
| SWR\_J-ISO\_136 |

© 2015 Computational Biology Group, Department of Medical Genetics,
University of Lausanne, Switzerland
