# Supplementary material for: RNAseq analysis of heart tissue from mice treated with atenolol and isoproterenol reveals a reciprocal transcriptional response
Source: BMC Genomics. 2016 Sep 7;17(1):717. doi: 10.1186/s12864-016-3059-6 (PMC5015234; doi:10.1186/s12864-016-3059-6)
Supplement: Additional file 4: — Supplementary data. Compressed HTML files of 98 expression modules annotated for genes, strains and GO or KEGG terms (see Additional file 3 for navigation details). (GZ 11006 kb) [file 12864_2016_3059_MOESM4_ESM.gz › modules.html/module-97.html]

Module #97, TG: 0, TC: 0, 8063 probes, 8063 Entrez genes, 160 conditions

# Previous module | Next module Module #97, TG: 0, TC: 0, 8063 probes, 8063 Entrez genes, 160 conditions

- Module tree/table

- Expression data

- The BP GO tree
- The CC GO tree
- The MF GO tree

- GO BP enrichment
- GO CC enrichment
- GO MF enrichment
- KEGG enrichment
- miRNA enrichment

- Genes
- Conditions

## Help | Hide | Top Help | Show | Top Expression data

### HELP

The image plot shows the color-coded level of gene expression, for the
genes and conditions in a given transcription module. The genes are on
the horizontal, the conditions on the vertical axis.

The genes are ordered according to their ISA gene scores, similarly
the conditions are ordered according to their condition scores. The
score of a gene means the «degree of inclusion» in
the module: a high score gene is essential in the module.

Condition scores can also be negative, that means that the genes of
the module are all down-regulated in the condition. Here the absolute
value of the score gives the «degree of inclusion».

The plots above and beside the expression matrix show the gene scores
and condition scores, respectively.

Note that the plot is interactive, you can see the name of the gene
and condition under the mouse cursor.

The expression matrix was normalized to have mean zero and standard
deviation one for every gene separately across all conditions
(i.e. not just for the conditions in the module).

— Click on the *Help* button again to close this help window.

Gene:   
Condition:

Under-expression is coded with green,
over-expression with red color.

## Help | Hide | Top Help | Show | Top The GO tree — Biological processes

### HELP

This is one of three sections showing Gene Ontology enrichment of the
current module: in this case for **biological processes**.

The graph shows the hierarchy of the GO categories, their enrichment
for the current module is color coded, and the blue number beside the
category is the minus log ten p-value of the enrichment. (Calculated
using the standard hypergeometric test.) The color of the arrows code
«is a» (cyan) and «part of» relationships.

The tree was built the following way. First all GO terms with more
significant enrichment p-value than 0.05 were collected. Then all
paths from these terms to the root node of the GO tree were included
too. If a GO term is included more than once in the tree, then the
green numbers show 1) the id of the node, this makes it easier to find
other appereances of the term, and 2) the number of appearences.

Note that the same GO category might show up on the graph many
times. This is because the GO was «straightened» for this
graph, i.e. if there are more paths from a GO term to the root node of
the tree, all of them are included. The green numbers

Move the mouse cursor over the terms to get their definition. Clicking
on them takes you to the corresponding Gene Ontology web page.

If you cannot see a graph here at all, that means that there were no
significantly enriched GO categories, at the 0.05 level.

— Click on the *Help* button again to close this help window.

## Help | Hide | Top Help | Show | Top The GO tree — Cellular Components

### HELP

This is one of three sections showing Gene Ontology enrichment of the
current module: in this case for **cellular components**.

The graph shows the hierarchy of the GO categories, their enrichment
for the current module is color coded, and the blue number beside the
category is the minus log ten p-value of the enrichment. (Calculated
using the standard hypergeometric test.) The color of the arrows code
«is a» (cyan) and «part of» relationships.

The tree was built the following way. First all GO terms with more
significant enrichment p-value than 0.05 were collected. Then all
paths from these terms to the root node of the GO tree were included
too. If a GO term is included more than once in the tree, then the
green numbers show 1) the id of the node, this makes it easier to find
other appereances of the term, and 2) the number of appearences.

Note that the same GO category might show up on the graph many
times. This is because the GO was «straightened» for this
graph, i.e. if there are more paths from a GO term to the root node of
the tree, all of them are included. The green numbers

Move the mouse cursor over the terms to get their definition. Clicking
on them takes you to the corresponding Gene Ontology web page.

If you cannot see a graph here at all, that means that there were no
significantly enriched GO categories, at the 0.05 level.

— Click on the *Help* button again to close this help window.

## Help | Hide | Top Help | Show | Top The GO tree — Molecular Function

### HELP

This is one of three sections showing Gene Ontology enrichment of the
current module: in this case for **molecular function**.

The graph shows the hierarchy of the GO categories, their enrichment
for the current module is color coded, and the blue number beside the
category is the minus log ten p-value of the enrichment. (Calculated
using the standard hypergeometric test.) The color of the arrows code
«is a» (cyan) and «part of» relationships.

The tree was built the following way. First all GO terms with more
significant enrichment p-value than 0.05 were collected. Then all
paths from these terms to the root node of the GO tree were included
too. If a GO term is included more than once in the tree, then the
green numbers show 1) the id of the node, this makes it easier to find
other appereances of the term, and 2) the number of appearences.

Note that the same GO category might show up on the graph many
times. This is because the GO was «straightened» for this
graph, i.e. if there are more paths from a GO term to the root node of
the tree, all of them are included. The green numbers

Move the mouse cursor over the terms to get their definition. Clicking
on them takes you to the corresponding Gene Ontology web page.

If you cannot see a graph here at all, that means that there were no
significantly enriched GO categories, at the 0.05 level.

— Click on the *Help* button again to close this help window.

## Help | Hide | Top Help | Show | Top GO BP test for over-representation

### HELP

List of all enriched GO categories (biological processes), at the 0.05
p-value level.

The columns:

- **ExpCount** is the expected count of genes in the
  module annotated with the given GO term, just by chance.
- **Count**
  is the number of genes in the module annotated with the given GO
  term.
- **Size** is the total number of genes (in our universe)
  annotated with the GO term.

Clicking on **Count** shows the genes that drive the
enrichment. You can also click on the individual numbers in
the **Count** column, to show the driving genes for that individual
GO category.

Clicking on the GO identifiers takes you to the Gene Ontology web
pages.

— Click on the *Help* button again to close this help window.

No enriched terms

## Help | Hide | Top Help | Show | Top GO CC test for over-representation

### HELP

List of all enriched GO categories (cellular components), at the 0.05
p-value level.

The columns:

- **ExpCount** is the expected count of genes in the
  module annotated with the given GO term, just by chance.
- **Count**
  is the number of genes in the module annotated with the given GO
  term.
- **Size** is the total number of genes (in our universe)
  annotated with the GO term.

Clicking on **Count** shows the genes that drive the
enrichment. You can also click on the individual numbers in
the **Count** column, to show the driving genes for that individual
GO category.

Clicking on the GO identifiers takes you to the Gene Ontology web
pages.

— Click on the *Help* button again to close this help window.

| Id | Pvalue | ExpCount | Count | Size | Term |
| --- | --- | --- | --- | --- | --- |
| GO:0044391 | 1.824e-02 | 62.62 | 86 Ddx3x, Fau, Gnb2l1, Hba-a2, Mrpl1, Mrpl10, Mrpl11, Mrpl12, Mrpl15, Mrpl16, Mrpl17, Mrpl20, Mrpl22, Mrpl23, Mrpl27, Mrpl32, Mrpl39, Mrpl41, Mrpl42, Mrpl43, Mrps10, Mrps11, Mrps14, Mrps16, Mrps18a, Mrps18c, Mrps22, Mrps23, Mrps24, Mrps25, Mrps31, Mrps36, Mterfd2, Npm1, Nsun4, Rpl10a, Rpl11, Rpl13, Rpl13a, Rpl14, Rpl15, Rpl18, Rpl18a, Rpl19, Rpl21, Rpl22, Rpl23, Rpl27a, Rpl28, Rpl29, Rpl3, Rpl30, Rpl32, Rpl36a, Rpl36al, Rpl37, Rpl37a, Rpl38, Rpl4, Rpl6, Rpl7, Rpl7a, Rpl9, Rplp0, Rplp1, Rps10, Rps12, Rps13, Rps14, Rps15, Rps16, Rps17, Rps18, Rps19, Rps2, Rps23, Rps24, Rps25, Rps27a, Rps3a1, Rps4x, Rps5, Rps7, Rps8, Uba52, Zcchc17 | 125 | ribosomal subunit |

## Help | Hide | Top Help | Show | Top GO MF test for over-representation

### HELP

List of all enriched GO categories (molecular function), at the 0.05
p-value level.

The columns:

- **ExpCount** is the expected count of genes in the
  module annotated with the given GO term, just by chance.
- **Count**
  is the number of genes in the module annotated with the given GO
  term.
- **Size** is the total number of genes (in our universe)
  annotated with the GO term.

Clicking on **Count** shows the genes that drive the
enrichment. You can also click on the individual numbers in
the **Count** column, to show the driving genes for that individual
GO category.

Clicking on the GO identifiers takes you to the Gene Ontology web
pages.

— Click on the *Help* button again to close this help window.

| Id | Pvalue | ExpCount | Count | Size | Term |
| --- | --- | --- | --- | --- | --- |
| GO:0003735 | 1.828e-02 | 59.17 | 83 Fau, Mrp63, Mrpl1, Mrpl10, Mrpl11, Mrpl12, Mrpl14, Mrpl15, Mrpl16, Mrpl17, Mrpl20, Mrpl21, Mrpl22, Mrpl23, Mrpl24, Mrpl27, Mrpl32, Mrpl33, Mrpl35, Mrpl39, Mrpl4, Mrpl41, Mrpl43, Mrpl47, Mrpl49, Mrps10, Mrps11, Mrps14, Mrps16, Mrps17, Mrps18a, Mrps18c, Mrps22, Mrps23, Mrps24, Mrps25, Mrps31, Mrps6, Mrps9, Rpl10a, Rpl11, Rpl13, Rpl13a, Rpl14, Rpl15, Rpl18, Rpl18a, Rpl19, Rpl21, Rpl22, Rpl23, Rpl27a, Rpl28, Rpl3, Rpl30, Rpl32, Rpl37a, Rpl38, Rpl4, Rpl5, Rpl6, Rpl7, Rpl9, Rplp0, Rplp1, Rps10, Rps14, Rps15, Rps16, Rps17, Rps18, Rps19, Rps2, Rps23, Rps24, Rps25, Rps27a, Rps27l, Rps3a1, Rps4x, Rps5, Rps7, Rps8 | 118 | structural constituent of ribosome |

## Help | Hide | Top Help | Show | Top KEGG Pathway test for over-representation

### HELP

List of all enriched KEGG pathways, at the 0.05
p-value level.

The columns:

- **ExpCount** is the expected count of genes in the
  module annotated with the given KEGG pathway, just by chance.
- **Count**
  is the number of genes in the module annotated with the given KEGG
  pathway.
- **Size** is the total number of genes (in our universe)
  annotated with the KEGG pathway.

Clicking on **Count** shows the genes that drive the
enrichment. You can also click on the individual numbers in
the **Count** column, to show the driving genes for that individual
KEGG pathway.

Clicking on the KEGG identifiers takes you to the KEGG web site.

— Click on the *Help* button again to close this help window.

No enriched terms


### HELP

List of all enriched miRNA families, at the 0.05
p-value level.

The columns:

- **ExpCount** is the expected count of genes in the
  module regulated by the given miRNA family, just by chance.
- **Count**
  is the number of genes in the module regulated by the given miRNA
  family.
- **Size** is the total number of genes (in our universe)
  regulated with the given miRNA family.

Clicking on **Count** shows the genes that drive the
enrichment. You can also click on the individual numbers in
the **Count** column, to show the driving genes for that individual
miRNA family.

The miRNA regulation data was taken from the

Top


### HELP

p-value level.

The columns:

- **ExpCount** is the expected number of genes in the- **Count**- **Size** is the total number of genes (in our universe)

Clicking on **Count** shows the genes that drive the
enrichment. You can also click on the individual numbers in
the **Count** column, to show the driving genes for that individual

— Click on the *Help* button again to close this help window.

## Help | Hide | Top Help | Show | Top Genes

### HELP

A list of all genes in the current module, in alphabetical order. The
size of the text corresponds to the gene scores.

Note that some gene symbols may show up more than once, if many
probes match the same Entrez gene.

Genes with no Entrez mapping are given separately, with their
Affymetrics probe ID.

— Click on the *Help* button again to close this help window.

### Genes Symbol

, score:

PzpUnknown, score: 0.04
AanatUnknown, score: 0.24
Abca1Unknown, score: 0.08
Abcg1Unknown, score: 0.13
Abl2Unknown, score: 0.44
AcadlUnknown, score: 0.29
AcadmUnknown, score: 0.12
AcadvlUnknown, score: 0.05
AcheUnknown, score: 0.2
Macf1Unknown, score: -0.01
Aco1Unknown, score: -0.01
Aco2Unknown, score: 0.29
Acp5Unknown, score: 0.19
AdipoqUnknown, score: 0.59
Actl7aUnknown, score: 0.03
Actn2Unknown, score: 0.09
Acvr1Unknown, score: 0.24
Acvr1bUnknown, score: 0.06
Acvr2aUnknown, score: 0.15
Acvr2bUnknown, score: 0.01
Acvrl1Unknown, score: 0.17
Adam10Unknown, score: 0.18
Adam17Unknown, score: 0.19
Adam4Unknown, score: -0.01
Adam8Unknown, score: 0
Adam9Unknown, score: 0.07
Adamts1Unknown, score: -0.01
Add2Unknown, score: 0.25
Plin2Unknown, score: 0.13
Adh7Unknown, score: 0.3
Adh5Unknown, score: 0.45
AdkUnknown, score: 0.23
AdmUnknown, score: 0.35
AdnpUnknown, score: 0.02
Adora1Unknown, score: 0.22
Adora2bUnknown, score: 0.46
Adora3Unknown, score: 0.08
AdprhUnknown, score: 0.05
Parp1Unknown, score: 0.15
Adra1bUnknown, score: 0.46
Adra2aUnknown, score: 0.25
Adra2cUnknown, score: 0.07
Adrb1Unknown, score: 0.03
Adrb3Unknown, score: 0.17
Adssl1Unknown, score: 0
Aebp1Unknown, score: -0.01
AgerUnknown, score: 0.22
AgtUnknown, score: 0.4
Agtr1aUnknown, score: 0.1
Agtr2Unknown, score: 0.06
AgtrapUnknown, score: 0.09
AhrUnknown, score: 0.1
AhrrUnknown, score: 0.2
Aif1Unknown, score: 0.05
AireUnknown, score: 0
Ak1Unknown, score: 0.24
Ak4Unknown, score: 0.38
AlplUnknown, score: 0
Akt1Unknown, score: 0.03
Akt2Unknown, score: 0
Alas2Unknown, score: 0.15
Abcd1Unknown, score: 0.49
Aldh1a1Unknown, score: 0.31
AldocUnknown, score: 0
Akr1b3Unknown, score: 0.24
Alox12Unknown, score: -0.01
Alox8Unknown, score: 0.38
Alox5apUnknown, score: 0.14
Alx4Unknown, score: 0.17
Amd1Unknown, score: 0.06
Ampd3Unknown, score: 0.25
Amy1Unknown, score: 0.02
AnkUnknown, score: 0.08
Ankfy1Unknown, score: 0.07
Anp32aUnknown, score: 0.03
Anxa4Unknown, score: 0.14
Anxa5Unknown, score: 0.14
Anxa6Unknown, score: 0.2
Aoc3Unknown, score: 0.04
Prdx3Unknown, score: 0.04
Prdx6Unknown, score: 0.27
Aox1Unknown, score: 0.11
Ap1g2Unknown, score: 0.07
Ap1s1Unknown, score: 0.22
Ap2a1Unknown, score: 0.04
Ap2m1Unknown, score: 0.22
Ap3b1Unknown, score: 0.03
Ap4m1Unknown, score: 0.49
Apaf1Unknown, score: 0.26
Apba2Unknown, score: -0.01
Apbb1Unknown, score: 0.13
SpegUnknown, score: 0.05
Birc3Unknown, score: 0.19
Api5Unknown, score: 0.24
Aplp1Unknown, score: 0.27
Aplp2Unknown, score: 0.32
Apoa1Unknown, score: 0.29
Apoa2Unknown, score: 0.14
Apoa4Unknown, score: 0.03
Apobec2Unknown, score: 0.13
Apoc1Unknown, score: 0.05
Apoc2Unknown, score: 0.16
ApodUnknown, score: 0.26
ApoeUnknown, score: 0.14
ApohUnknown, score: 0.06
AprtUnknown, score: 0.58
Aqp4Unknown, score: 0.27
Aqp5Unknown, score: 0.34
Aqp6Unknown, score: 0.53
Aqp7Unknown, score: 0.51
AqrUnknown, score: 0.02
ArUnknown, score: 0.01
ArafUnknown, score: 0.08
Rplp0Unknown, score: 0.14
Arf3Unknown, score: 0
Arf4Unknown, score: 0.11
Arf5Unknown, score: 0.02
Arg2Unknown, score: 0.24
RhoaUnknown, score: 0.12
RhobUnknown, score: -0.01
RhocUnknown, score: 0
Arhgap5Unknown, score: 0
Arpc1bUnknown, score: 0.16
ArtnUnknown, score: 0.54
AC084822.3Unknown, score: 0.14
ArxUnknown, score: 0.39
Asah1Unknown, score: 0.18
Asgr1Unknown, score: 0.12
Astn1Unknown, score: 0.22
Zfhx3Unknown, score: 0
Atf1Unknown, score: 0.04
Atp1b1Unknown, score: 0.26
Atp1b3Unknown, score: 0.14
Fxyd2Unknown, score: 0.33
Atp2a1Unknown, score: 0.01
Atp2a2Unknown, score: 0.05
Atp4aUnknown, score: 0.01
Atp4bUnknown, score: 0.05
Atp5a1Unknown, score: 0.26
Atp5bUnknown, score: 0.14
Atp5c1Unknown, score: 0.21
Atp5f1Unknown, score: 0.28
Atp5g1Unknown, score: 0.02
Atp5jUnknown, score: 0.2
Atp6v1aUnknown, score: 0.06
Atp6v1b2Unknown, score: 0.22
Atp6v0d1Unknown, score: 0.1
Atp6v0eUnknown, score: 0.13
Atp7bUnknown, score: 0.26
Atp10aUnknown, score: 0.5
AtrnUnknown, score: 0.25
HnrnpdUnknown, score: 0.23
Pcdh15Unknown, score: 0.09
AvpUnknown, score: 0.16
Axin2Unknown, score: 0.12
Cep131Unknown, score: 0.08
B2mUnknown, score: 0.21
Bach1Unknown, score: 0.21
BadUnknown, score: 0.1
BaxUnknown, score: 0.15
Bcl6bUnknown, score: 0.02
BcanUnknown, score: 0.37
Bcap29Unknown, score: 0.1
Bcat1Unknown, score: 0.02
BcheUnknown, score: 0.29
BckdhaUnknown, score: 0.13
BckdhbUnknown, score: 0.2
Bcl10Unknown, score: 0.46
Bcl2l1Unknown, score: 0.13
Bcl6Unknown, score: 0.2
Bcl7bUnknown, score: 0.39
Bdkrb2Unknown, score: 0.69
BdnfUnknown, score: 0.12
Bet1Unknown, score: -0.01
Ngfrap1Unknown, score: -0.01
Bfsp1Unknown, score: 0.06
Glb1Unknown, score: 0.03
BgnUnknown, score: 0.19
BhmtUnknown, score: 0.19
BidUnknown, score: 0.29
HrkUnknown, score: 0.05
Bcl2l11Unknown, score: 0.11
Fabp7Unknown, score: 0.22
Prdm1Unknown, score: 0.5
BlmUnknown, score: 0.13
Bmp2Unknown, score: 0.17
Bmp6Unknown, score: 0.06
Bmp7Unknown, score: 0.03
Bmpr1aUnknown, score: 0.06
Bmpr2Unknown, score: 0.27
Bnip3Unknown, score: 0.15
Bnip3lUnknown, score: 0.13
Smyd1Unknown, score: 0.15
Birc6Unknown, score: 0.14
Chic1Unknown, score: 0.14
BsgUnknown, score: 0.04
BsnUnknown, score: 0.1
BtcUnknown, score: 0.11
Btg2Unknown, score: 0.26
Bub3Unknown, score: 0.07
C1qaUnknown, score: -0.01
C1qbUnknown, score: 0.32
C4bUnknown, score: 0.14
C6Unknown, score: 0.16
C9Unknown, score: 0.16
Cab39Unknown, score: 0.08
Cacna1aUnknown, score: 0.03
Cacna1cUnknown, score: 0.12
Cacna1dUnknown, score: 0.08
Cacna1gUnknown, score: 0.72
Cacna1sUnknown, score: 0.28
Cacna2d3Unknown, score: 0.26
Cacnb4Unknown, score: 0.06
CacybpUnknown, score: 0.05
AspmUnknown, score: 0.46
CaluUnknown, score: 0.1
Camk2aUnknown, score: 0.1
Camk2bUnknown, score: 0.26
Camk4Unknown, score: 0.12
CamlUnknown, score: 0.15
Capn5Unknown, score: 0.09
Capn6Unknown, score: 0.08
Capza1Unknown, score: 0.12
Capza2Unknown, score: 0.01
Car2Unknown, score: 0.08
Car3Unknown, score: 0.36
Car5aUnknown, score: 0.26
Car7Unknown, score: 0.33
CaskUnknown, score: 0.06
Casp6Unknown, score: 0.06
Casp8Unknown, score: 0.04
Casp9Unknown, score: 0.23
Ctnna1Unknown, score: 0.1
Ctnnb1Unknown, score: 0.02
Ctnnd1Unknown, score: 0.03
Cav1Unknown, score: 0.15
Runx1Unknown, score: 0.39
Cbfa2t2Unknown, score: 0.02
Runx3Unknown, score: 0.06
CbfbUnknown, score: 0.12
CblUnknown, score: 0.15
Serpinh1Unknown, score: 0.22
Cbr1Unknown, score: -0.01
Cbr2Unknown, score: 0.29
CbsUnknown, score: 0.13
Cbx2Unknown, score: 0.04
Ccnb2Unknown, score: 0.01
Ccnd1Unknown, score: 0.12
Ccng1Unknown, score: 0.26
Ccr6Unknown, score: -0.01
CcsUnknown, score: 0.04
Cct3Unknown, score: 0.19
Cct6bUnknown, score: 0.22
Cct7Unknown, score: 0.05
Ctla4Unknown, score: 0.17
Cd19Unknown, score: 0.33
Ms4a1Unknown, score: 0.07
Cd24aUnknown, score: 0
Cd34Unknown, score: 0.23
Cd36Unknown, score: 0.07
Cd37Unknown, score: 0.11
Entpd2Unknown, score: 0.51
Entpd6Unknown, score: 0.14
Cd3gUnknown, score: 0.35
Cd48Unknown, score: 0.04
Cd6Unknown, score: -0.01
Cd7Unknown, score: 0.12
Cd72Unknown, score: 0.09
Cd80Unknown, score: 0.04
Cd81Unknown, score: 0.15
Cd82Unknown, score: 0.28
Cd84Unknown, score: 0.18
Cd86Unknown, score: 0.07
Cd8aUnknown, score: 0.01
Cd9Unknown, score: 0.33
Cdk1Unknown, score: 0.31
Cdk11bUnknown, score: 0.32
Cdc37Unknown, score: 0.21
Cdc42Unknown, score: 0.29
Cdh13Unknown, score: 0.01
Cdh3Unknown, score: 0.11
Cdh4Unknown, score: 0.02
Cdh6Unknown, score: 0.01
Cdk2Unknown, score: 0.24
Cdk5Unknown, score: 0.49
Cdk6Unknown, score: 0.23
Cdkn1aUnknown, score: 0.32
Cdkn1bUnknown, score: 0.49
Cdr2Unknown, score: 0.12
MiaUnknown, score: 0.23
CebpaUnknown, score: 0.39
CebpgUnknown, score: 0.08
Celsr1Unknown, score: 0.08
CenpbUnknown, score: 0.01
Ces1gUnknown, score: 0.17
12631Unknown, score: 0.12
Ch25hUnknown, score: 0.07
ChadUnknown, score: 0
Chd1Unknown, score: 0.01
Chek1Unknown, score: 0.61
Chil3Unknown, score: 0.23
Ovgp1Unknown, score: 0.24
ChkaUnknown, score: 0.26
Chl1Unknown, score: 0.08
ChukUnknown, score: 0.32
CideaUnknown, score: 0.23
CidebUnknown, score: 0.2
Elovl3Unknown, score: 0.38
CirbpUnknown, score: 0
Socs3Unknown, score: 0.05
CitUnknown, score: 0.08
CkbUnknown, score: 0.08
Clca3a1Unknown, score: 0.45
Clcn1Unknown, score: 0.15
Clcn2Unknown, score: 0.19
Clcn3Unknown, score: 0.02
Clcn5Unknown, score: 0.08
ClcnkaUnknown, score: 0.04
Cldn3Unknown, score: 0.1
Cldn5Unknown, score: 0.14
Clk1Unknown, score: 0.12
Tpp1Unknown, score: 0.1
ClockUnknown, score: 0.24
CluUnknown, score: 0.04
CmahUnknown, score: 0.3
Cxcr3Unknown, score: 0.44
Ccr9Unknown, score: 0.19
Ccr2Unknown, score: 0.2
Ccr4Unknown, score: -0.01
Ackr3Unknown, score: 0.02
Abcc2Unknown, score: 0.25
Cnga3Unknown, score: 0.07
Cnih2Unknown, score: 0.02
Cnn2Unknown, score: 0.09
Cnr1Unknown, score: 0.03
CntfUnknown, score: 0.42
CntfrUnknown, score: 0.18
Cntn1Unknown, score: 0.22
Hps3Unknown, score: 0.08
CoblUnknown, score: 0
CochUnknown, score: 0.06
Col11a1Unknown, score: 0
Col12a1Unknown, score: 0.37
Col4a2Unknown, score: 0.08
Col4a3Unknown, score: 0.07
Col1a2Unknown, score: 0.12
CompUnknown, score: 0.41
ComtUnknown, score: 0.13
Coq7Unknown, score: 0.07
CortUnknown, score: 0.04
Cox17Unknown, score: -0.01
Cox5aUnknown, score: 0.12
Cox5bUnknown, score: 0.15
Cox6a1Unknown, score: 0.31
Cox6a2Unknown, score: 0.16
Cox6cUnknown, score: 0.03
Cox7a1Unknown, score: 0.18
Cox7a2Unknown, score: 0.11
Cox7cUnknown, score: 0.25
Cox8aUnknown, score: 0.19
Cox8bUnknown, score: 0.04
CpdUnknown, score: 0.28
CpeUnknown, score: 0.54
Cys1Unknown, score: 0.03
Cplx2Unknown, score: 0.16
CpoxUnknown, score: 0.56
Cpt1aUnknown, score: 0.56
Cpt1bUnknown, score: 0.38
Cpt2Unknown, score: 0.09
Crabp2Unknown, score: 0.09
CraddUnknown, score: 0.3
CratUnknown, score: 0.24
CrhbpUnknown, score: 0.04
Pcdha11Unknown, score: 0.12
CrpUnknown, score: 0.21
Cry2Unknown, score: 0.14
Crybb1Unknown, score: 0.06
CryzUnknown, score: 0.16
CsUnknown, score: 0.13
CskUnknown, score: 0.26
Csnk2a2Unknown, score: 0.14
VcanUnknown, score: 0.17
Csrp2Unknown, score: 0.24
Csrp3Unknown, score: 0.02
CstbUnknown, score: 0.17
CtcfUnknown, score: 0.09
CtsbUnknown, score: 0.11
Cux1Unknown, score: 0.03
Cux2Unknown, score: 0.1
Cx3cr1Unknown, score: 0.43
Cyb561Unknown, score: 0.13
CybaUnknown, score: 0.18
CybbUnknown, score: 0.18
Cyp11a1Unknown, score: 0.29
Cyp1a1Unknown, score: 0.14
Cyp26a1Unknown, score: 0.23
Cyp2a12Unknown, score: 0.1
Cyp2b10Unknown, score: 0.21
Cyp2c29Unknown, score: 0.16
Cyp2c37Unknown, score: 0.18
Cyp2d9Unknown, score: 0.11
Cyp2e1Unknown, score: 0.13
Cyp2f2Unknown, score: 0.21
Cyp2j5Unknown, score: 0.44
Cyp2j6Unknown, score: 0.23
Cyp46a1Unknown, score: 0.31
Cyp4a10Unknown, score: 0.08
Cyp4a14Unknown, score: 0.02
Cyp4b1Unknown, score: -0.01
Cyp51Unknown, score: 0.05
Dab2Unknown, score: 0.18
Dach1Unknown, score: 0.09
Dag1Unknown, score: 0.12
DgkaUnknown, score: 0.07
Dapk3Unknown, score: 0.05
DazlUnknown, score: 0.11
DbhUnknown, score: 0.25
DbiUnknown, score: 0.08
DbpUnknown, score: 0.36
Dclk1Unknown, score: 0.72
DckUnknown, score: 0.04
Pcbd1Unknown, score: 0.13
DctUnknown, score: 0.21
Dctn1Unknown, score: 0.1
Ddb1Unknown, score: 0.17
Asap1Unknown, score: 0.38
DdnUnknown, score: 0.05
DdostUnknown, score: 0.22
DdtUnknown, score: 0.09
Ddx3xUnknown, score: 0.06
Ddx5Unknown, score: 0.13
Ddx6Unknown, score: 0
Dhx9Unknown, score: 0
Degs1Unknown, score: 0.15
Ackr1Unknown, score: 0.13
Diap1Unknown, score: 0.15
DldUnknown, score: 0.01
Dlg4Unknown, score: 0.09
Dlk1Unknown, score: 0.23
Dll1Unknown, score: 0.2
Dlx1Unknown, score: 0.01
Dlx5Unknown, score: 0.17
DmwdUnknown, score: 0.12
Dmc1Unknown, score: 0.04
Dmp1Unknown, score: 0.31
Dnah11Unknown, score: 0.18
Dnah8Unknown, score: 0.03
Dnajc1Unknown, score: 0.31
Dnase1Unknown, score: 0.06
Dnase2aUnknown, score: 0
Dync1i1Unknown, score: 0.06
Dnm1Unknown, score: 0.2
Dnmt1Unknown, score: 0.09
Trdmt1Unknown, score: 0.07
Dnmt3bUnknown, score: 0.06
Cdk2ap1Unknown, score: 0.15
Dpagt1Unknown, score: 0.12
Dpep1Unknown, score: 0.36
Dpm2Unknown, score: 0.07
Dr1Unknown, score: 0.01
Drd1Unknown, score: 0.22
Drd2Unknown, score: 0.14
Drg1Unknown, score: 0
Arid3aUnknown, score: 0.01
Dsc2Unknown, score: 0.03
DscamUnknown, score: 0.22
Dsg2Unknown, score: 0.18
EpycUnknown, score: 0.05
Adam28Unknown, score: 0.31
DtnbUnknown, score: 0.04
Dvl1Unknown, score: 0.12
E2f3Unknown, score: 0.22
E2f5Unknown, score: 0.09
E4f1Unknown, score: 0.16
Ear2Unknown, score: 0.13
Ebf2Unknown, score: 0.05
Ecm1Unknown, score: 0.03
Sparcl1Unknown, score: 0.04
S1pr1Unknown, score: 0.06
S1pr3Unknown, score: 0.06
Edn1Unknown, score: 0.51
EdnraUnknown, score: 0.19
EdnrbUnknown, score: 0.26
Eef1a1Unknown, score: 0.18
Eef2Unknown, score: 0.14
Eef2kUnknown, score: 0.59
Efna1Unknown, score: 0.11
Efnb1Unknown, score: 0.21
EfsUnknown, score: 0.27
EgfUnknown, score: 0.02
Rhbdf1Unknown, score: 0.15
EhfUnknown, score: 0.03
Eif2s1Unknown, score: 0.09
Eif2ak3Unknown, score: 0.18
Eif3aUnknown, score: 0.01
Ddx19aUnknown, score: 0.1
Eif4a1Unknown, score: 0.06
Eif4a2Unknown, score: 0.7
Eif4eUnknown, score: 0.34
Eif4ebp1Unknown, score: -0.01
Eif4ebp2Unknown, score: 0
Elf1Unknown, score: 0.07
Elf5Unknown, score: 0.23
Elk3Unknown, score: 0.14
Elk4Unknown, score: 0.07
ElnUnknown, score: 0.21
EmbUnknown, score: 0.02
EnpepUnknown, score: 0.05
EomesUnknown, score: 0.15
Epb4.1l2Unknown, score: 0.09
Epb4.1l3Unknown, score: 0.21
Epb4.1l4aUnknown, score: -0.01
DmtnUnknown, score: 0.7
StomUnknown, score: 0.48
Epha1Unknown, score: 0.07
Epha4Unknown, score: 0.28
Epha5Unknown, score: 0.16
Epha6Unknown, score: 0.2
Epha7Unknown, score: 0.12
Ephb2Unknown, score: 0.29
Ephx1Unknown, score: 0.63
Stx2Unknown, score: 0.09
Epn2Unknown, score: 0.33
Eps15Unknown, score: 0.04
Nr2f6Unknown, score: 0.09
Erbb2Unknown, score: 0.14
Erbb4Unknown, score: 0.06
Ercc1Unknown, score: 0.14
Ercc2Unknown, score: 0.12
ErfUnknown, score: 0.01
ErgUnknown, score: 0.13
ErhUnknown, score: 0.18
Ces1cUnknown, score: 0.3
EsdUnknown, score: 0.5
Ces1eUnknown, score: 0.38
Ces3bUnknown, score: 0.22
Smarcad1Unknown, score: 0.18
Gm14288Unknown, score: 0.1
Chchd2Unknown, score: 0.31
Mpzl2Unknown, score: 0.14
MecomUnknown, score: 0
Evi2aUnknown, score: 0.07
Evi5Unknown, score: 0.24
EvlUnknown, score: 0.17
EvplUnknown, score: 0.15
Ewsr1Unknown, score: 0.03
Ext2Unknown, score: 0.25
Eya4Unknown, score: 0.08
Ezh1Unknown, score: 0.06
F10Unknown, score: 0.15
F13bUnknown, score: 0.15
F2rl2Unknown, score: 0.21
F2rl3Unknown, score: 0.01
F8aUnknown, score: 0.02
F9Unknown, score: 0.03
FaahUnknown, score: 0.21
Fabp3Unknown, score: 0.23
Fabp1Unknown, score: 0.37
Acsl1Unknown, score: 0.44
Ptk2Unknown, score: 0.26
Faf1Unknown, score: 0.25
FahUnknown, score: 0.12
FancaUnknown, score: 0.18
FanccUnknown, score: 0.02
Fat1Unknown, score: 0.02
FauUnknown, score: 0.05
Fbn1Unknown, score: 0.36
Fbn2Unknown, score: 0.64
Fbp2Unknown, score: 0.25
FbrsUnknown, score: 0.01
Fcer1gUnknown, score: 0.13
Fcgr3Unknown, score: 0.12
FdxrUnknown, score: 0.69
FesUnknown, score: 0.04
Lgr5Unknown, score: 0.23
Fgd1Unknown, score: 0.55
Fgf1Unknown, score: 0.02
Fgf11Unknown, score: 0.13
Fgf12Unknown, score: 0.3
Fgf14Unknown, score: 0.15
Fgf17Unknown, score: 0.1
Fgf2Unknown, score: 0.18
Fgf5Unknown, score: 0.39
Fgf6Unknown, score: 0.09
Fgf9Unknown, score: 0.08
Fgfr1Unknown, score: 0.46
FgrUnknown, score: 0.11
Fh1Unknown, score: 0.26
Fhl2Unknown, score: 0.26
Fhl3Unknown, score: 0.13
Fhl4Unknown, score: 0.24
FigfUnknown, score: 0.14
Fkbp1bUnknown, score: 0.28
Fkbp2Unknown, score: 0.35
Fkbp5Unknown, score: 0.27
Lpin1Unknown, score: 0.13
FliiUnknown, score: 0
Flot1Unknown, score: 0.31
Flt1Unknown, score: 0.02
Flt3Unknown, score: 0
Flt3lUnknown, score: 0.11
Fmn1Unknown, score: 0
Fmo5Unknown, score: 0.02
FmodUnknown, score: 0.03
Fn1Unknown, score: 0.03
Fnbp1Unknown, score: 0.38
Srgap2Unknown, score: 0.17
FntaUnknown, score: 0.35
Fosl2Unknown, score: 0.14
Ncs1Unknown, score: 0.26
FrkUnknown, score: -0.01
Fth1Unknown, score: 0.1
Ftl1Unknown, score: 0.07
AktipUnknown, score: 0.07
Fut1Unknown, score: 0.17
Fut2Unknown, score: 0.01
Fut4Unknown, score: 0.02
Fut7Unknown, score: 0.01
Fv1Unknown, score: 0.16
Fxr1Unknown, score: 0.09
Fzd4Unknown, score: 0.21
Fzd5Unknown, score: 0.11
Fzd6Unknown, score: 0.06
Fzd7Unknown, score: 0.13
Fzd8Unknown, score: 0.42
Fzd9Unknown, score: 0.28
G0s2Unknown, score: 0.34
GanabUnknown, score: 0.14
G6pdxUnknown, score: 0.06
GabpaUnknown, score: 0.02
Gabpb1Unknown, score: 0.31
Gabra1Unknown, score: 0.15
Gabra3Unknown, score: 0.24
Gabrg3Unknown, score: 0.03
Slc6a12Unknown, score: 0.11
Slc6a13Unknown, score: 0
Gad2Unknown, score: 0.16
B4galnt2Unknown, score: 0.17
Galnt1Unknown, score: -0.01
Galr2Unknown, score: 0.27
GcatUnknown, score: 0.08
GaltUnknown, score: 0.09
GapdhUnknown, score: 0.04
GapdhsUnknown, score: 0.12
Gas1Unknown, score: 0.14
Gas2Unknown, score: 0.05
Gata1Unknown, score: 0
Gata3Unknown, score: 0.05
GbaUnknown, score: 0.05
GbasUnknown, score: -0.01
Gbp2Unknown, score: 0.13
Rabac1Unknown, score: 0
Gbx2Unknown, score: 0.23
GcUnknown, score: 0.11
MtpnUnknown, score: 0.03
GcsamUnknown, score: 0.08
Gch1Unknown, score: 0.18
Gcm1Unknown, score: 0.12
Bloc1s1Unknown, score: 0.06
Nr6a1Unknown, score: 0.04
GdaUnknown, score: 0.14
Gdap1Unknown, score: 0.05
Gdap2Unknown, score: 0.16
Mrps33Unknown, score: 0.13
Gpd1Unknown, score: 0.35
Gdf9Unknown, score: 0.05
Gdi1Unknown, score: 0.19
ArhgdigUnknown, score: 0.16
Gpd2Unknown, score: 0.28
GfapUnknown, score: 0
Gfpt2Unknown, score: 0.05
Gfra1Unknown, score: 0.08
Gfra2Unknown, score: 0.17
Gfra3Unknown, score: 0.14
Gfra4Unknown, score: -0.01
GghUnknown, score: 0.15
Ggta1Unknown, score: 0.06
B4galt1Unknown, score: 0.1
Tsc22d3Unknown, score: 0.2
Gja4Unknown, score: 0.08
Gja5Unknown, score: 0.2
Gjc1Unknown, score: 0.21
Gjb1Unknown, score: 0.05
Gjb2Unknown, score: 0
Gjb5Unknown, score: 0
Ostm1Unknown, score: 0.06
GclcUnknown, score: 0.14
GclmUnknown, score: -0.01
Gli3Unknown, score: 0.04
Galk1Unknown, score: 0.27
GlulUnknown, score: 0.42
HaghUnknown, score: 0.35
GlrbUnknown, score: 0.17
Slc6a9Unknown, score: 0.03
Gm2aUnknown, score: 0.41
Gnl1Unknown, score: 0.11
Gna13Unknown, score: -0.01
Gna14Unknown, score: 0.09
Gna15Unknown, score: 0.05
Gnai3Unknown, score: -0.01
Gnao1Unknown, score: 0.21
GnaqUnknown, score: 0.07
Gnat1Unknown, score: 0.03
GnazUnknown, score: 0.06
Gnb2Unknown, score: 0.12
Gnb2l1Unknown, score: 0.83
Gnb3Unknown, score: 0.11
Gng10Unknown, score: -0.01
Gng12Unknown, score: 0.17
Gngt2Unknown, score: 0.33
GnmtUnknown, score: 0.19
GnpatUnknown, score: -0.01
Gnrh1Unknown, score: 0.06
Got1Unknown, score: 0.01
Got2Unknown, score: 0.4
Gp1bbUnknown, score: 0.07
Gpaa1Unknown, score: 0.28
GpamUnknown, score: 0
Gpc1Unknown, score: 0.01
Gpc3Unknown, score: 0.03
Gpc4Unknown, score: 0.25
Cmklr1Unknown, score: 0.2
Gpr3Unknown, score: 0.09
PigqUnknown, score: -0.01
Gpr19Unknown, score: 0.12
Gpr27Unknown, score: 0.1
Adgrg1Unknown, score: 0.1
Nmur1Unknown, score: 0.22
Gpx3Unknown, score: 0.01
GsrUnknown, score: 0.21
Grb10Unknown, score: 0.19
Grb2Unknown, score: 0.03
Grb7Unknown, score: 0.1
Rhpn1Unknown, score: 0.08
P3h3Unknown, score: 0.31
Emg1Unknown, score: 0.35
Spsb2Unknown, score: 0.01
Gria1Unknown, score: 0.09
Gria4Unknown, score: 0
Grid1Unknown, score: 0.1
Grik2Unknown, score: 0.2
Grin2dUnknown, score: 0.15
Grm1Unknown, score: 0.02
Cxcl1Unknown, score: 0.1
Pdia3Unknown, score: 0.13
Gsg2Unknown, score: 0.09
Gspt2Unknown, score: 0.1
GssUnknown, score: 0.01
Gsta1Unknown, score: 0.14
Gsta2Unknown, score: 0.09
Gsta3Unknown, score: 0.43
Gstm2Unknown, score: 0
Gstm3Unknown, score: 0.23
Gstm4Unknown, score: -0.01
Gstm5Unknown, score: 0.48
Gstm6Unknown, score: 0.27
Gstp2Unknown, score: 0.05
Gstp1Unknown, score: 0.45
Gstt1Unknown, score: 0.16
Gstt2Unknown, score: 0.29
Gsto1Unknown, score: 0.29
Gstz1Unknown, score: 0.19
Gtf2h4Unknown, score: 0.08
Gtf2iUnknown, score: 0.11
Cfap20Unknown, score: 0.16
Gtpbp1Unknown, score: 0.29
Thumpd3Unknown, score: 0.01
Nkx6-2Unknown, score: 0.02
Guca1aUnknown, score: -0.01
Gucy2eUnknown, score: 0.39
Magi1Unknown, score: 0.3
GykUnknown, score: 0.05
Gys1Unknown, score: 0.21
GzmaUnknown, score: 0.01
Hist1h1dUnknown, score: 0.22
CfbUnknown, score: 0.04
H2-D1Unknown, score: 0.19
H2-Eb1Unknown, score: 0.07
H2-K1Unknown, score: 0.26
Slc39a7Unknown, score: 0.22
H2-DMaUnknown, score: 0.07
H2-DMb1Unknown, score: 0.05
H2-OaUnknown, score: 0.17
H2-ObUnknown, score: -0.01
H2-Q1Unknown, score: 0.06
H2-Q10Unknown, score: 0.07
H2-Q2Unknown, score: 0.17
H2-Q7Unknown, score: -0.01
H2-T3Unknown, score: 0.1
H2-T22Unknown, score: 0.1
H2-T3Unknown, score: 0.1
H2-T22Unknown, score: 0.04
15061Unknown, score: 0.01
Mr1Unknown, score: 0.02
Hist2h3c1Unknown, score: 0.14
H3f3aUnknown, score: 0.14
H3f3aUnknown, score: 0.51
HadhUnknown, score: 0.48
Hand2Unknown, score: 0.16
Has2Unknown, score: 0.37
Has3Unknown, score: 0.11
Hbb-bsUnknown, score: 0.06
HccsUnknown, score: 0.1
Hcn1Unknown, score: 0.51
Hcn2Unknown, score: -0.01
Hcn3Unknown, score: 0.08
Ptpn6Unknown, score: 0.14
Hdac2Unknown, score: 0.12
HdcUnknown, score: 0.05
HdgfUnknown, score: 0.22
HttUnknown, score: 0.36
Hebp1Unknown, score: 0.47
Herc2Unknown, score: 0.38
Hes5Unknown, score: 0.28
Hesx1Unknown, score: -0.01
HexaUnknown, score: 0.16
Hey1Unknown, score: 0.1
Foxq1Unknown, score: 0.04
Foxd3Unknown, score: 0.1
Foxf1Unknown, score: 0.07
HgfUnknown, score: 0.12
Mst1Unknown, score: 0.14
HgsUnknown, score: 0.13
Hic1Unknown, score: 0.44
Hk1Unknown, score: 0
Hk2Unknown, score: 0.12
Hmgb1Unknown, score: 0.02
Hmgn1Unknown, score: 0.27
Hmgb3Unknown, score: 0.15
HmgclUnknown, score: 0
HmgcrUnknown, score: 0.68
Hmga1Unknown, score: 0.07
Hmox1Unknown, score: 0.17
Hn1Unknown, score: 0.17
Hnf4aUnknown, score: 0.13
Onecut1Unknown, score: 0.6
HnrnpcUnknown, score: 0.19
HnrnpkUnknown, score: 0.08
Hoxa1Unknown, score: 0.14
Hoxa4Unknown, score: 0.05
Hoxa5Unknown, score: 0.04
Hoxa6Unknown, score: 0.2
Hoxa7Unknown, score: 0.13
Hoxb6Unknown, score: 0.02
HpUnknown, score: 0.01
HpcaUnknown, score: 0.17
HpdUnknown, score: 0.63
HpgdUnknown, score: 0.02
LipcUnknown, score: 0.19
HpnUnknown, score: 0.21
HprtUnknown, score: 0.06
Agfg1Unknown, score: 0.26
Hrh1Unknown, score: 0.28
Prmt2Unknown, score: 0.08
Hs3st1Unknown, score: 0.12
Hs3st3a1Unknown, score: 0.06
Hspa1lUnknown, score: 0.22
Hsd11b2Unknown, score: 0.2
Hsd17b1Unknown, score: 0.15
Hsd17b2Unknown, score: 0.27
Hsd17b4Unknown, score: 0.22
Hsd17b7Unknown, score: 0.26
Hsd3b2Unknown, score: 0.42
Hsd3b3Unknown, score: 0.22
Hsd3b5Unknown, score: 0.06
Hsf2Unknown, score: 0.07
Hsph1Unknown, score: 0.19
Hspb1Unknown, score: 0.37
Hspa2Unknown, score: 0.18
Hspa4Unknown, score: 0.09
Hspa9Unknown, score: 0.11
Ndst1Unknown, score: 0.05
Trmt2aUnknown, score: 0.19
Htr3aUnknown, score: 0.05
Elavl3Unknown, score: -0.01
Id4Unknown, score: 0.11
IdeUnknown, score: 0.1
Idh1Unknown, score: 0.09
Ido1Unknown, score: 0.21
IdsUnknown, score: 0.16
Ier3Unknown, score: 0.23
Ier5Unknown, score: 0
Cxcl10Unknown, score: 0.01
Ifit1Unknown, score: 0.24
Ifit3Unknown, score: 0.19
Ifngr1Unknown, score: 0.56
Ifrd1Unknown, score: 0.25
Ifrd2Unknown, score: 0.09
Igf1rUnknown, score: 0.05
Igf2rUnknown, score: 0.07
IgfalsUnknown, score: 0.66
Cyr61Unknown, score: 0.03
Igfbp2Unknown, score: 0.69
Igfbp6Unknown, score: 0.31
Il18bpUnknown, score: 0.21
IgtpUnknown, score: 0.07
IhhUnknown, score: 0.09
IkbkbUnknown, score: 0.13
Il10raUnknown, score: 0.34
Il10rbUnknown, score: 0.12
Il11Unknown, score: 0.17
Gm13305Unknown, score: -0.01
Il12rb1Unknown, score: 0.25
Il12rb2Unknown, score: 0.3
Il15Unknown, score: 0.19
Il15raUnknown, score: 0.29
Il1r2Unknown, score: 0.2
Il1rnUnknown, score: -0.01
Il18r1Unknown, score: 0.28
Il2raUnknown, score: 0.03
Il4raUnknown, score: 0.08
Il6stUnknown, score: 0
Ilf3Unknown, score: 0.15
ImpactUnknown, score: 0.08
InhbbUnknown, score: 0.05
Insl3Unknown, score: 0.24
InsrUnknown, score: 0.28
IppUnknown, score: 0.02
Irf2Unknown, score: 0.14
Irg1Unknown, score: 0.08
Irx2Unknown, score: 0.68
Irx3Unknown, score: 0.04
Itga2bUnknown, score: 0
Itga5Unknown, score: 0.47
Itga6Unknown, score: 0.09
Eif6Unknown, score: 0.2
Itgb5Unknown, score: 0.34
Cd47Unknown, score: 0.16
Itih1Unknown, score: -0.01
Itih2Unknown, score: 0.15
Stt3aUnknown, score: 0.01
Itm2aUnknown, score: 0.05
ItpaUnknown, score: 0.02
Jak1Unknown, score: -0.01
F11rUnknown, score: 0.51
AtcayUnknown, score: 0.26
Jarid2Unknown, score: 0.11
JrkUnknown, score: 0.27
JundUnknown, score: 0.17
JupUnknown, score: 0.51
Kcna1Unknown, score: 0.17
Kcna3Unknown, score: 0.61
Kcna4Unknown, score: -0.01
Kcna5Unknown, score: 0.44
Kcna6Unknown, score: 0.09
Kcnd2Unknown, score: 0.1
Kcnh3Unknown, score: 0.07
Kcnj10Unknown, score: 0.06
Kcnj12Unknown, score: 0.03
Kcnj15Unknown, score: 0.02
Kcnj5Unknown, score: 0.08
Kcnj6Unknown, score: 0.08
Kcnj8Unknown, score: 0.36
Kcnk1Unknown, score: 0.24
Kcnk3Unknown, score: -0.01
Kcnq1Unknown, score: 0
NapsaUnknown, score: -0.01
KdrUnknown, score: 0.02
MdficUnknown, score: 0.11
Kif16bUnknown, score: 0.13
Kif1cUnknown, score: 0.02
Kif2aUnknown, score: 0.1
Kif21bUnknown, score: 0.19
Kif4Unknown, score: 0.08
Kif5cUnknown, score: 0.11
Kif9Unknown, score: 0.23
Kifc5bUnknown, score: 0.39
Kifc3Unknown, score: 0.16
Uhmk1Unknown, score: 0.38
Klc1Unknown, score: 0.07
Klc2Unknown, score: 0.01
Klf4Unknown, score: 0.23
Klk1b26Unknown, score: 0.04
Serpina3cUnknown, score: 0.05
Klra2Unknown, score: -0.01
CU424478.2Unknown, score: 0.14
Klra4Unknown, score: 0.04
CU424478.2Unknown, score: 0.01
Klrc2Unknown, score: 0.12
Klrd1Unknown, score: 0.23
Kpna3Unknown, score: 0.08
Kpna6Unknown, score: 0.2
Ksr1Unknown, score: 0
Ktn1Unknown, score: 0.38
KyUnknown, score: 0.28
Aff3Unknown, score: 0.02
Lag3Unknown, score: 0
Dsg4Unknown, score: 0
Lama5Unknown, score: 0.13
Lamb1Unknown, score: 0.02
Lamp1Unknown, score: 0.13
LbpUnknown, score: 0.2
Lbx2Unknown, score: 0.16
Lcp2Unknown, score: 0.14
LdhaUnknown, score: 0.32
LdlrUnknown, score: 0.36
Lect1Unknown, score: -0.01
Lect2Unknown, score: 0.16
Lef1Unknown, score: 0.05
LepUnknown, score: 0.03
LfngUnknown, score: 0.24
Lgals3Unknown, score: 0.5
Eif2dUnknown, score: 0
Lhx2Unknown, score: 0.1
Limk1Unknown, score: 0.17
LipaUnknown, score: 0.18
LipeUnknown, score: 0.02
Llgl1Unknown, score: 0.14
Rps2Unknown, score: 0.13
LmnaUnknown, score: 0.1
Lmnb2Unknown, score: 0.8
Lmo4Unknown, score: 0.08
Lmx1bUnknown, score: 0.03
MyclUnknown, score: 0.14
PhyhUnknown, score: 0.25
Sh2b3Unknown, score: 0.16
Anxa1Unknown, score: 0.22
LplUnknown, score: 0.11
Lrp8Unknown, score: 0.29
Lrrc23Unknown, score: 0.45
Lrrfip1Unknown, score: 0.25
Lrrn2Unknown, score: 0.16
Lsp1Unknown, score: 0.17
Lst1Unknown, score: 0.08
Lta4hUnknown, score: 0
LtbrUnknown, score: 0.12
LumUnknown, score: 0.26
Klrb1aUnknown, score: 0.07
Ly6c1Unknown, score: 0.1
Ly75Unknown, score: 0.01
Cd180Unknown, score: 0.34
Tmed1Unknown, score: 0.16
Ly9Unknown, score: 0.2
Ncr1Unknown, score: 0
Ly96Unknown, score: 0.53
Lyl1Unknown, score: 0
Tm4sf1Unknown, score: 0.04
M6prUnknown, score: 0.14
Mab21l1Unknown, score: -0.01
AmacrUnknown, score: 0.07
Mxd1Unknown, score: 0.04
Mxd3Unknown, score: 0.11
Madcam1Unknown, score: 0.23
Smad7Unknown, score: 0.37
MaffUnknown, score: 0.04
MafkUnknown, score: 0.02
MagUnknown, score: 0.12
Mfap2Unknown, score: 0.01
Man2b2Unknown, score: 0.03
MarcoUnknown, score: 0.01
Mark3Unknown, score: 0.09
Mas1Unknown, score: 0.09
Masp1Unknown, score: 0.15
Masp2Unknown, score: 0.01
Matn2Unknown, score: 0.02
Matn4Unknown, score: 0.19
MazUnknown, score: 0.2
MbUnknown, score: 0.12
Mbd1Unknown, score: 0.05
Mbd4Unknown, score: 0.2
Mcl1Unknown, score: 0.02
Mcm3Unknown, score: 0.05
Mcm2Unknown, score: 0.03
Mcm6Unknown, score: 0.1
Anapc1Unknown, score: 0.02
Mgrn1Unknown, score: 0.11
MdfiUnknown, score: -0.01
Mdm4Unknown, score: 0.18
Slc3a2Unknown, score: 0.15
Mea1Unknown, score: 0.01
Mecp2Unknown, score: -0.01
Mef2bUnknown, score: 0.16
Mef2cUnknown, score: 0.03
Mef2dUnknown, score: 0.47
Meis1Unknown, score: 0.02
MelkUnknown, score: 0.19
Meox1Unknown, score: 0.08
MertkUnknown, score: 0.03
MestUnknown, score: 0
MetUnknown, score: 0.35
Mettl1Unknown, score: 0.02
Mfge8Unknown, score: 0
KitlUnknown, score: 0.3
MgpUnknown, score: 0.46
MifUnknown, score: 0.13
Bhlha15Unknown, score: 0.38
MitfUnknown, score: 0.07
Mki67Unknown, score: 0.02
Mknk1Unknown, score: 0.02
Mlf1Unknown, score: 0.15
Mlh1Unknown, score: 0.05
Mllt10Unknown, score: 0.14
Aff1Unknown, score: 0.35
Marcksl1Unknown, score: 0.12
Trpm1Unknown, score: 0.04
Mmp11Unknown, score: 0.15
Mmp2Unknown, score: 0.21
Mmp9Unknown, score: 0.25
Mnat1Unknown, score: 0.02
Ndst2Unknown, score: 0.22
Foxk1Unknown, score: 0.24
Mocs2Unknown, score: 0.33
Grap2Unknown, score: 0.02
Mdh2Unknown, score: 0.06
Mdh1Unknown, score: 0.08
Mov10Unknown, score: 0.05
Psmd7Unknown, score: 0.24
Clec4dUnknown, score: 0.01
MplUnknown, score: 0.01
Mpp1Unknown, score: 0.15
Gm29609Unknown, score: 0.21
Mre11aUnknown, score: 0.19
Meis3Unknown, score: 0.17
Mrvi1Unknown, score: 0.06
Cited2Unknown, score: 0.3
Msh5Unknown, score: 0.06
Msh6Unknown, score: 0.1
Sik1Unknown, score: -0.01
Msx2Unknown, score: 0.2
mt-Atp6Unknown, score: 0.15
mt-Atp8Unknown, score: 0.21
mt-Co1Unknown, score: 0.15
mt-Co2Unknown, score: 0.15
mt-Co3Unknown, score: 0.13
mt-CytbUnknown, score: 0.14
Grpel2Unknown, score: 0.26
mt-Nd2Unknown, score: 0.27
mt-Nd3Unknown, score: 0.25
mt-Nd4Unknown, score: 0.36
mt-Nd5Unknown, score: 0.39
Map1bUnknown, score: -0.01
Map6Unknown, score: 0.31
Map7Unknown, score: 0.52
MaptUnknown, score: 0.03
Mtf1Unknown, score: 0.07
Mthfd2Unknown, score: 0.27
MthfrUnknown, score: 0.51
Mtm1Unknown, score: 0.09
Mast2Unknown, score: 0.63
Fam89bUnknown, score: 0.07
17831Unknown, score: 0.37
Mug1Unknown, score: 0.2
Mup1Unknown, score: 0.27
Mup3Unknown, score: 0.36
Usp34Unknown, score: 0.22
Mxi1Unknown, score: 0.03
Mybl2Unknown, score: -0.01
Mybpc3Unknown, score: 0
Ppp1r15aUnknown, score: 0.32
Myef2Unknown, score: 0.25
Myh11Unknown, score: 0.04
Myh3Unknown, score: 0.12
Myh9Unknown, score: 0.15
Myl4Unknown, score: 0.51
Myl3Unknown, score: 0
Myl7Unknown, score: 0.02
Myl6Unknown, score: 0.16
Myl2Unknown, score: 0.11
MylpfUnknown, score: 0.13
Myo5aUnknown, score: 0.29
Myo5bUnknown, score: 0.11
Myo6Unknown, score: 0.08
Myo7aUnknown, score: 0.04
Myo7bUnknown, score: 0.39
MyocUnknown, score: 0.18
Myod1Unknown, score: 0.49
Myom1Unknown, score: 0.85
Myom2Unknown, score: 0.02
Nab2Unknown, score: 0.08
NacaUnknown, score: 0.04
Naip6Unknown, score: 0.09
Nap1l2Unknown, score: 0.05
Nap1l4Unknown, score: 0.11
NapbUnknown, score: 0.03
Nat1Unknown, score: -0.01
Nbl1Unknown, score: 0.07
Nbr1Unknown, score: 0.03
Ncam1Unknown, score: 0.04
Nck1Unknown, score: 0.24
Nck2Unknown, score: 0.12
Ncoa2Unknown, score: 0.09
Ndrg1Unknown, score: 0.11
Ndufa2Unknown, score: 0.19
Ndufa4Unknown, score: 0.11
Ndufs4Unknown, score: 0.17
Nedd1Unknown, score: 0.14
Nedd4Unknown, score: 0.04
Sept2Unknown, score: 0.06
Nedd9Unknown, score: 0
NesUnknown, score: 0.03
Neurl1aUnknown, score: 0.15
Nfatc1Unknown, score: 0.4
Nfe2l2Unknown, score: 0.14
Nfe2l3Unknown, score: 0.11
NfiaUnknown, score: 0.08
NfibUnknown, score: 0.12
Nfil3Unknown, score: 0.13
NfkbiaUnknown, score: 0.17
Nfs1Unknown, score: 0.11
NfycUnknown, score: 0.02
Ninj1Unknown, score: 0.17
Nipsnap1Unknown, score: 0.16
NlkUnknown, score: 0.14
NmbrUnknown, score: 0.09
Nme1Unknown, score: 0.09
Nme2Unknown, score: 0.11
Cd244Unknown, score: 0.35
MycnUnknown, score: 0.26
NnmtUnknown, score: 0.08
Emc8Unknown, score: 0.04
NodalUnknown, score: 0.08
Mrpl49Unknown, score: 0.2
Nr4a3Unknown, score: 0
Nos1Unknown, score: 0.4
Notch2Unknown, score: 0.11
Notch3Unknown, score: 0.21
Npas1Unknown, score: 0.32
Npdc1Unknown, score: 0.22
Npm1Unknown, score: 0.27
Npm3Unknown, score: 0.52
NppbUnknown, score: 0.07
Npr1Unknown, score: 0.15
Nptx1Unknown, score: 0.01
Slc11a2Unknown, score: 0.04
NrapUnknown, score: 0.01
Nrf1Unknown, score: 0.2
Nrp1Unknown, score: 0.14
NrtnUnknown, score: -0.01
Nrxn1Unknown, score: 0.13
MuskUnknown, score: 0.14
NsmafUnknown, score: 0.12
Ntan1Unknown, score: 0.46
Ntf3Unknown, score: 0.04
Ntrk1Unknown, score: 0
Ntrk2Unknown, score: 0
Ddr2Unknown, score: 0.53
Dusp8Unknown, score: 0.06
Nucb1Unknown, score: 0
Nr4a2Unknown, score: 0.24
NxnUnknown, score: 0.19
Nxph1Unknown, score: 0.41
Oaz1Unknown, score: 0.08
OclnUnknown, score: 0.66
Odc1Unknown, score: 0.06
Odf2Unknown, score: 0.09
Ogg1Unknown, score: 0.38
Olfr18Unknown, score: 0.05
OmgUnknown, score: 0.06
Tnfrsf11bUnknown, score: 0.06
Oprd1Unknown, score: 0.1
Oprl1Unknown, score: -0.01
Orc2Unknown, score: 0.03
Slc22a18Unknown, score: 0.12
Orm3Unknown, score: 0.17
Slc25a15Unknown, score: 0.11
Hspa4lUnknown, score: 0.24
Cldn11Unknown, score: 0.03
Ovol1Unknown, score: 0.14
OxtrUnknown, score: 0.02
Oca2Unknown, score: 0.03
P2rx6Unknown, score: 0.16
P4ha1Unknown, score: 0.21
P4ha2Unknown, score: 0.32
P4hbUnknown, score: 0.19
Bloc1s6Unknown, score: 0.15
Pafah1b1Unknown, score: 0.07
Prdx1Unknown, score: 0.12
PamUnknown, score: 0.3
PappaUnknown, score: 0.15
Pax3Unknown, score: 0.17
Pax5Unknown, score: 0.02
Pax6Unknown, score: 0.01
Pbx1Unknown, score: 0.08
Pbx3Unknown, score: 0.45
Igbp1Unknown, score: -0.01
Pcdh8Unknown, score: 0.13
Pck1Unknown, score: 0.08
Pcm1Unknown, score: 0
Pcmt1Unknown, score: 0.04
Pcp2Unknown, score: 0.59
FurinUnknown, score: 0.3
Pcsk7Unknown, score: 0.05
Cdk16Unknown, score: 0.58
Cdk18Unknown, score: 0.08
Pdcd1Unknown, score: 0.14
Pdcd4Unknown, score: 0.02
Pdcd6ipUnknown, score: 0.04
Pde1aUnknown, score: 0.03
Pde1bUnknown, score: 0.07
Pde4bUnknown, score: 0.24
Pde6dUnknown, score: 0.27
Pde9aUnknown, score: 0.01
Pde6bUnknown, score: 0.2
PdgfrbUnknown, score: 0.16
Padi2Unknown, score: -0.01
Padi4Unknown, score: 0.06
Enpp1Unknown, score: 0.13
Pdpk1Unknown, score: 0.05
Pea15aUnknown, score: 0.02
Peg3Unknown, score: 0.01
PemtUnknown, score: 0.29
PenkUnknown, score: 0.04
Per2Unknown, score: 0.12
Per3Unknown, score: 0.48
Pex11aUnknown, score: 0.04
Pex11bUnknown, score: 0.23
Pex16Unknown, score: 0.14
CfpUnknown, score: 0.44
Pfdn2Unknown, score: 0.22
Pfkfb1Unknown, score: 0.16
PfklUnknown, score: 0.04
PfkmUnknown, score: 0.41
Pfn1Unknown, score: 0.24
Pfn2Unknown, score: 0.15
Pgk1Unknown, score: 0.16
Abcb1bUnknown, score: 0
Abcb1aUnknown, score: 0
Slc25a3Unknown, score: 0.19
Phtf1Unknown, score: 0.12
Pick1Unknown, score: 0.24
PigaUnknown, score: 0.11
PigfUnknown, score: 0.25
Pik3c2aUnknown, score: 0.13
Pim1Unknown, score: 0.21
Pim2Unknown, score: 0.04
PirbUnknown, score: 0.02
Pitpnm1Unknown, score: 0.01
Pitx2Unknown, score: 0.08
Pitx3Unknown, score: 0.11
PkmUnknown, score: 0.3
PrkacaUnknown, score: 0.11
PrkacbUnknown, score: 0.09
PrkcdUnknown, score: 0.04
PrkceUnknown, score: 0.09
PrkchUnknown, score: 0.06
PrkciUnknown, score: 0.15
Prkd1Unknown, score: 0.11
PkdrejUnknown, score: 0.5
PkiaUnknown, score: 0.54
PkigUnknown, score: 0.18
Pknox1Unknown, score: 0.09
Pla2g2cUnknown, score: 0.31
Pla2g5Unknown, score: 0.1
PlaaUnknown, score: 0.32
PlatUnknown, score: 0.02
Plcb4Unknown, score: 0.22
Plcd1Unknown, score: 0.11
Plcd4Unknown, score: 0.26
Plcg1Unknown, score: 0.03
PlecUnknown, score: 0.15
Pa2g4Unknown, score: 0.22
PlgUnknown, score: 0.17
Plod1Unknown, score: 0.61
Plp1Unknown, score: 0.23
Lcp1Unknown, score: 0.3
Plscr2Unknown, score: 0.15
Plxna1Unknown, score: 0.11
Pms2Unknown, score: 0.29
Prrx1Unknown, score: 0.12
Ppp1r14bUnknown, score: 0.21
Pnliprp1Unknown, score: 0.21
PnmtUnknown, score: 0.22
Pola1Unknown, score: 0.09
Pola2Unknown, score: 0.08
PolbUnknown, score: 0
Pold1Unknown, score: 0.07
Pold2Unknown, score: 0.52
Pole2Unknown, score: 0.19
Pon1Unknown, score: 0.05
Cnot7Unknown, score: 0.12
PorUnknown, score: 0.01
Pou2af1Unknown, score: 0.08
Pou2f1Unknown, score: 0.16
Pou6f1Unknown, score: 0.11
PpardUnknown, score: 0.22
Ppargc1aUnknown, score: 0.01
Scand1Unknown, score: 0
Ppef2Unknown, score: 0.65
CtsaUnknown, score: 0.12
SyplUnknown, score: 0.05
Lgals3bpUnknown, score: 0.11
Ppm1bUnknown, score: 0.01
PpoxUnknown, score: 0.08
Ppp1r1bUnknown, score: -0.01
Ppp2caUnknown, score: 0.1
Ppp2cbUnknown, score: 0.25
Ppp3cbUnknown, score: 0.03
Ppp3r1Unknown, score: 0.12
Ppp5cUnknown, score: 0.1
Inpp5kUnknown, score: 0.22
Ppt1Unknown, score: 0.73
Npy4rUnknown, score: 0.1
Nup88Unknown, score: 0.05
Mob4Unknown, score: 0.13
Prim2Unknown, score: 0.02
Prkab1Unknown, score: 0.13
Prkar2bUnknown, score: 0.06
PrkcshUnknown, score: 0.01
Prkg2Unknown, score: 0.17
Eif2ak2Unknown, score: 0.12
PrnpUnknown, score: 0.21
ProcrUnknown, score: 0.26
Pros1Unknown, score: 0
PrphUnknown, score: 0.04
Prpf4bUnknown, score: 0.21
St14Unknown, score: 0.01
NpeppsUnknown, score: 0.12
PsapUnknown, score: 0.04
Cyth3Unknown, score: 0.03
Psen1Unknown, score: 0.08
Psen2Unknown, score: 0.19
Psma2Unknown, score: 0.01
Psma3Unknown, score: 0.18
Psmb1Unknown, score: 0.25
Psmb10Unknown, score: 0.08
Psmb6Unknown, score: 0.08
Psmb7Unknown, score: 0.32
Psmc1Unknown, score: 0.2
Psmc2Unknown, score: 0.59
Psmc3ipUnknown, score: 0.03
Psmc5Unknown, score: 0.16
PipoxUnknown, score: 0.03
PtafrUnknown, score: 0.03
Ptch2Unknown, score: 0.48
Ptdss1Unknown, score: 0.13
PtenUnknown, score: 0.28
PterUnknown, score: 0.25
PtgisUnknown, score: 0.04
Tmsb10Unknown, score: 0.12
Tmsb4xUnknown, score: 0.07
PtnUnknown, score: 0.33
Ptpn13Unknown, score: 0.04
Dusp1Unknown, score: 0.07
Ptpn2Unknown, score: 0.32
Ptpn4Unknown, score: 0.01
SirpaUnknown, score: 0.17
PtprcUnknown, score: 0.03
PtprdUnknown, score: 0.47
PtprfUnknown, score: 0.01
PtpruUnknown, score: 0.01
PtprmUnknown, score: 0.53
PtprsUnknown, score: 0
PtprtUnknown, score: 0.35
PtrfUnknown, score: 0.41
Ptx3Unknown, score: 0.12
Igdcc3Unknown, score: 0.16
PuraUnknown, score: 0.17
PvalbUnknown, score: 0.01
Pvrl2Unknown, score: 0.38
Pex19Unknown, score: 0.3
Abcd3Unknown, score: 0.02
Abcd4Unknown, score: 0.3
Pex2Unknown, score: 0.04
Pex5Unknown, score: 0.05
QkUnknown, score: 0.15
Rab1Unknown, score: 0.12
Rab10Unknown, score: 0.07
Rab11bUnknown, score: 0.54
Rab17Unknown, score: -0.01
Rab18Unknown, score: 0.06
Rab19Unknown, score: 0.55
Rab20Unknown, score: 0.11
Rab22aUnknown, score: 0.03
Rab23Unknown, score: 0.34
Rab24Unknown, score: 0.09
Rab33aUnknown, score: 0.18
Rab3aUnknown, score: 0.04
Rab4bUnknown, score: 0.05
Rab5cUnknown, score: 0.05
Rab6aUnknown, score: 0.1
Rab7Unknown, score: 0.24
RabggtbUnknown, score: 0.04
Rad1Unknown, score: 0.06
Rad21Unknown, score: 0.15
Rad23aUnknown, score: 0.24
Rad50Unknown, score: 0.16
Rad51Unknown, score: 0.06
Rad51bUnknown, score: 0.18
Rad51dUnknown, score: 0.07
Rad52Unknown, score: 0
RanUnknown, score: 0.19
Rangap1Unknown, score: 0.43
Rasgrp2Unknown, score: 0.04
RargUnknown, score: 0.32
Rasd1Unknown, score: 0.03
Rasgrp1Unknown, score: 0.1
Rb1Unknown, score: -0.01
Rbbp4Unknown, score: 0.02
Rbbp6Unknown, score: 0.17
Robo3Unknown, score: 0.1
Rbl1Unknown, score: 0.26
Rbm6Unknown, score: 0.19
Rbp4Unknown, score: 0.03
RbpmsUnknown, score: 0.02
RbpjUnknown, score: 0.04
Rce1Unknown, score: 0.18
Rcn1Unknown, score: 0.2
Rdh5Unknown, score: 0.1
RdxUnknown, score: 0.65
RecqlUnknown, score: 0.03
RenbpUnknown, score: 0.01
Upf1Unknown, score: 0.17
Rev3lUnknown, score: 0.61
RfngUnknown, score: 0.16
RfxankUnknown, score: 0.49
RalgdsUnknown, score: 0
Rgs2Unknown, score: 0.02
RhebUnknown, score: 0.04
RhdUnknown, score: 0.28
Rnase1Unknown, score: 0.16
Ring1Unknown, score: 0.04
Ripk1Unknown, score: 0.17
Xpr1Unknown, score: 0.04
Uri1Unknown, score: 0.03
Rnaseh1Unknown, score: 0.06
RlimUnknown, score: 0.17
Rnf2Unknown, score: 0.06
Rnf4Unknown, score: 0.17
Rnf7Unknown, score: 0.02
Rock2Unknown, score: 0.04
RoraUnknown, score: 0.37
Rp2hUnknown, score: 0.5
Rpa2Unknown, score: 0.09
RpiaUnknown, score: 0.37
Rpl10aUnknown, score: 0.27
Rpl18Unknown, score: 0.39
Rpl19Unknown, score: 0.1
Rpl21Unknown, score: 0.23
Rpl22Unknown, score: 0.19
Mrpl23Unknown, score: 0.1
Rpl28Unknown, score: 0.14
Rpl29Unknown, score: 0.11
Rpl30Unknown, score: 0.28
Rpl32Unknown, score: 0.09
Rpl37aUnknown, score: 0
Rpl36aUnknown, score: 0.19
Rpl6Unknown, score: 0.24
Rpl7Unknown, score: 0.04
Rpl9Unknown, score: 0.06
Rpn2Unknown, score: 0.22
Polr1cUnknown, score: 0.12
Polr1bUnknown, score: 0.09
Polr1dUnknown, score: -0.01
Polr1aUnknown, score: 0.12
Polr2aUnknown, score: 0.02
Polr2cUnknown, score: 0.11
Polr2jUnknown, score: 0.42
Rps12Unknown, score: 0.18
Rps14Unknown, score: 0.31
Rps15Unknown, score: 0.03
Rps16Unknown, score: 0.58
Rps17Unknown, score: 0.26
Rps18Unknown, score: 0.23
Rps19Unknown, score: 0.12
Rps24Unknown, score: 0.04
Rps3a1Unknown, score: 0.12
Rps4xUnknown, score: 0.21
Rps5Unknown, score: 0.31
Rps6ka2Unknown, score: 0.32
Rps7Unknown, score: 0.19
Rps8Unknown, score: 0.02
Rrm2Unknown, score: -0.01
Rs1Unknown, score: 0.16
Dhrs3Unknown, score: 0.21
RtknUnknown, score: 0.19
Rtn2Unknown, score: 0.06
Hps6Unknown, score: 0.22
RxrbUnknown, score: 0.06
RxrgUnknown, score: 0.05
Uimc1Unknown, score: 0.3
RykUnknown, score: 0.05
Ryr2Unknown, score: 0.61
Ryr3Unknown, score: 0.43
S100a13Unknown, score: 0.04
S100a9Unknown, score: 0.14
Saa1Unknown, score: 0.02
Saa3Unknown, score: 0.26
Saa4Unknown, score: -0.01
Acsm3Unknown, score: 0.01
Khdrbs1Unknown, score: 0.21
ApcsUnknown, score: 0.13
Sap18Unknown, score: 0.06
Sf3a2Unknown, score: 0.26
Sar1aUnknown, score: 0.15
SarsUnknown, score: 0.01
Scg3Unknown, score: 0.04
Stmn2Unknown, score: 0.61
Scn1bUnknown, score: 0.04
Scn8aUnknown, score: 0.15
Scnn1bUnknown, score: 0.19
Scp2Unknown, score: 0.2
Msr1Unknown, score: 0.37
ScxUnknown, score: 0.13
Ccl4Unknown, score: 0.23
Cxcl12Unknown, score: 0.03
Frrs1Unknown, score: 0.06
SdprUnknown, score: 0.01
Sec23aUnknown, score: 0.19
Sec61gUnknown, score: 0.18
Exoc4Unknown, score: -0.01
SeleUnknown, score: 0.27
SelplgUnknown, score: 0.06
Sema3cUnknown, score: 0.15
Sema4aUnknown, score: 0.06
Sema4cUnknown, score: 0.53
Sema4dUnknown, score: 0.11
Sema4fUnknown, score: 0.19
Sema5aUnknown, score: 0.04
Sema5bUnknown, score: 0.31
Sema6aUnknown, score: 0.01
Sema6bUnknown, score: 0.27
Sept8Unknown, score: 0.4
Sepw1Unknown, score: 0.2
Serf1Unknown, score: 0.36
Sfrp4Unknown, score: 0.24
Srsf3Unknown, score: 0.1
Srsf5Unknown, score: 0
SftpbUnknown, score: 0.17
SgcaUnknown, score: 0.18
Sgk1Unknown, score: 0.1
Scg5Unknown, score: 0.06
Sgpl1Unknown, score: 0.04
Sh3gl1Unknown, score: 0.14
20408Unknown, score: 0.13
Shfm1Unknown, score: 0.31
Cyfip1Unknown, score: 0.03
Siah1aUnknown, score: 0.11
Siah2Unknown, score: 0.25
St3gal2Unknown, score: 0.02
St6galnac1Unknown, score: 0.19
St6galnac4Unknown, score: 0.1
St8sia1Unknown, score: 0.07
St8sia2Unknown, score: 0.13
StilUnknown, score: 0.1
Tra2bUnknown, score: 0.08
Cox7a2lUnknown, score: 0
Sin3bUnknown, score: 0
Six4Unknown, score: 0.04
Vps4bUnknown, score: 0.27
SlaUnknown, score: 0
SlbpUnknown, score: 0.03
Slc12a3Unknown, score: 0.17
Slc19a1Unknown, score: 0.07
Slc20a1Unknown, score: 0.35
Slc22a1Unknown, score: 0.03
Slc22a3Unknown, score: 0.36
Slc22a12Unknown, score: 0.14
Slc25a14Unknown, score: 0.15
Slc2a1Unknown, score: 0.02
Slc31a2Unknown, score: 0.08
Slc3a1Unknown, score: 0
Slc4a1Unknown, score: 0.06
Slc4a3Unknown, score: 0.07
Slit1Unknown, score: 0.18
Snai2Unknown, score: 0.25
HltfUnknown, score: 0.11
Smarcb1Unknown, score: 0.19
Kdm5cUnknown, score: 0.08
Kdm5dUnknown, score: 0.07
Smpd1Unknown, score: 0.41
SmsUnknown, score: 0.06
Sstr1Unknown, score: 0
Snai1Unknown, score: 0.11
SnapinUnknown, score: 0.22
SncgUnknown, score: 0.24
Snap23Unknown, score: 0.26
Plk2Unknown, score: 0.29
SnnUnknown, score: 0.07
SnrkUnknown, score: 0.05
SnrpcUnknown, score: 0.05
SnrpbUnknown, score: 0.22
Snrpb2Unknown, score: 0.06
Snrpd1Unknown, score: 0.03
Snta1Unknown, score: 0.37
Soat1Unknown, score: 0.3
Sod1Unknown, score: 0.05
Sod3Unknown, score: 0.08
Sorl1Unknown, score: 0.18
Sos2Unknown, score: 0.42
Sox10Unknown, score: 0.26
Sox13Unknown, score: 0.07
Sox18Unknown, score: 0.03
Sox2Unknown, score: 0.29
Sox4Unknown, score: 0.12
Sox7Unknown, score: 0.04
Sox9Unknown, score: 0.02
Sp1Unknown, score: 0.15
Sp100Unknown, score: 0.17
Spa17Unknown, score: 0.11
Sp3Unknown, score: 0.22
Sphk1Unknown, score: 0.1
Serpina1aUnknown, score: 0
Serpina1bUnknown, score: 0.24
Serpina1cUnknown, score: 0.1
Serpina1aUnknown, score: 0.23
Serpinb9bUnknown, score: 0.67
Serpinb6bUnknown, score: 0.02
Serpini1Unknown, score: 0.24
Serpine2Unknown, score: 0.17
Serpinb8Unknown, score: 0.17
SpicUnknown, score: 0.18
Spint2Unknown, score: 0
SpnUnknown, score: 0.41
Sptbn2Unknown, score: 0.08
StrbpUnknown, score: 0.24
SqleUnknown, score: 0.18
TmieUnknown, score: 0.26
SrmsUnknown, score: 0.06
Srpk1Unknown, score: 0.1
SrprbUnknown, score: 0.13
Trove2Unknown, score: 0.03
Nhp2l1Unknown, score: 0.1
StarUnknown, score: 0
Stat4Unknown, score: 0.19
Stat6Unknown, score: 0.08
Stc1Unknown, score: 0.26
Stc2Unknown, score: 0.06
Stim1Unknown, score: 0.07
Stk11Unknown, score: 0.01
AurkaUnknown, score: 0.56
Sult1a1Unknown, score: 0.22
Stra6Unknown, score: 0.11
Stx3Unknown, score: 0.04
Stxbp4Unknown, score: 0.35
Sucla2Unknown, score: 0.08
Supt6Unknown, score: 0.2
Abcc8Unknown, score: 0.04
Abcc9Unknown, score: 0.25
Surf1Unknown, score: 0.26
Surf2Unknown, score: 0.09
Surf4Unknown, score: 0.18
Med22Unknown, score: 0.17
Surf6Unknown, score: 0.2
Swap70Unknown, score: 0.01
Sycp1Unknown, score: 0.15
Syn1Unknown, score: 0.19
Sdc3Unknown, score: 0.38
Syngr1Unknown, score: 0.04
Syngr3Unknown, score: 0.31
Tac1Unknown, score: 0.09
Tacr3Unknown, score: 0.34
Taf1aUnknown, score: 0.2
Tal1Unknown, score: 0
Cntn2Unknown, score: 0.1
Tbl1xUnknown, score: 0.18
Tbrg4Unknown, score: 0.27
Tbx1Unknown, score: 0.02
Tcea1Unknown, score: 0.25
Tcea2Unknown, score: 0.28
Skp1aUnknown, score: 0.21
Tcf12Unknown, score: 0
Tcf15Unknown, score: 0.03
Tcf21Unknown, score: 0.04
Tcf7l1Unknown, score: 0.26
Tfap2aUnknown, score: 0.12
Tfap2bUnknown, score: 0.03
Tfap2cUnknown, score: 0.09
Tfcp2Unknown, score: 0.07
Vps72Unknown, score: 0.02
MlxUnknown, score: 0.07
Tcn2Unknown, score: 0.16
Tcof1Unknown, score: 0.01
Tcp1Unknown, score: 0.11
Tcte2Unknown, score: 0.22
Dynlt1bUnknown, score: 0.07
Phf1Unknown, score: 0.74
Phlda1Unknown, score: 0.03
TdgUnknown, score: 0.1
Tdgf1Unknown, score: 0.14
Tead2Unknown, score: 0.12
Tead4Unknown, score: 0.05
AlyrefUnknown, score: 0.16
TectbUnknown, score: 0.22
Tekt1Unknown, score: 0.24
Terf1Unknown, score: 0.39
Tesk1Unknown, score: 0.16
Morf4l1Unknown, score: 0.01
Tex261Unknown, score: 0.21
Tex264Unknown, score: 0
Zfand3Unknown, score: 0.21
Ppp2r5dUnknown, score: 0.06
Tex9Unknown, score: 0.26
Tgfb1i1Unknown, score: 0.37
Tgfbr2Unknown, score: 0.05
Tgm1Unknown, score: 0.18
TgUnknown, score: 0.18
Ift88Unknown, score: 0.11
ThraUnknown, score: 0.15
ThrbUnknown, score: 0.01
Thy1Unknown, score: 0
Tia1Unknown, score: 0.22
Tiam1Unknown, score: 0.04
Tie1Unknown, score: 0.22
Klf10Unknown, score: 0.43
TimelessUnknown, score: 0.04
Timm17bUnknown, score: 0.04
Timp2Unknown, score: 0.05
Timp3Unknown, score: 0.21
Atp6v0a2Unknown, score: 0.02
Tjp1Unknown, score: 0.09
TktUnknown, score: 0
Tle3Unknown, score: 0.27
Tlx1Unknown, score: 0.03
Tspan7Unknown, score: 0.12
Tmod1Unknown, score: 0.02
TncUnknown, score: 0.1
Tnnc1Unknown, score: 0.1
Tnnc2Unknown, score: 0.07
Tnfaip1Unknown, score: 0
Tnfaip2Unknown, score: 0.05
Tnfaip6Unknown, score: -0.01
Tnfrsf10bUnknown, score: 0.03
Tnfrsf11aUnknown, score: 0.07
Cd27Unknown, score: 0.01
Tnfsf11Unknown, score: 0.09
Pglyrp1Unknown, score: 0.06
Tnfsf9Unknown, score: 0.1
TnksUnknown, score: 0.19
Tnni2Unknown, score: 0.01
Tnni3Unknown, score: 0.55
Tnnt1Unknown, score: 0.19
Tns1Unknown, score: 0.22
Tom1Unknown, score: 0.01
Top2bUnknown, score: 0.08
Top3bUnknown, score: 0.04
Tpd52Unknown, score: 0.3
Tpi1Unknown, score: 0.14
TpmtUnknown, score: 0.02
Tpp2Unknown, score: 0.53
Tpst2Unknown, score: 0.28
Nr2c2Unknown, score: 0.45
Traf6Unknown, score: 0.01
Tnfsf10Unknown, score: 0.23
TraipUnknown, score: 0.09
Plscr1Unknown, score: 0.15
Trex1Unknown, score: 0.04
Tob1Unknown, score: 0.09
Trp63Unknown, score: 0.29
Trpc1Unknown, score: 0.1
Trpc2Unknown, score: 0.04
Trpc3Unknown, score: 0.02
Trpc6Unknown, score: 0.21
Tpt1Unknown, score: 0
Tsc2Unknown, score: 0.1
Tsg101Unknown, score: 0.15
TshbUnknown, score: 0.02
TshrUnknown, score: 0.01
TsnUnknown, score: 0.1
Tspyl1Unknown, score: 0.01
Tssk1Unknown, score: 0.01
Tssk2Unknown, score: 0.1
Rpl13aUnknown, score: 0.25
Tsta3Unknown, score: 0.06
Tuba1bUnknown, score: 0.23
Tuba3aUnknown, score: 0.2
Tuba4aUnknown, score: 0
Tuba1cUnknown, score: 0.23
Tubb5Unknown, score: 0.11
Twist1Unknown, score: 0.11
TxkUnknown, score: 0.12
Txn1Unknown, score: -0.01
Cmpk2Unknown, score: 0.11
TymsUnknown, score: 0.21
TyrobpUnknown, score: 0.05
Zrsr2Unknown, score: 0.09
Uba52Unknown, score: 0.08
Ube2e3Unknown, score: 0.13
Ube2e1Unknown, score: 0.15
Ube2l3Unknown, score: 0.12
Ube2iUnknown, score: 0.16
Uba3Unknown, score: 0.07
Ube2aUnknown, score: 0.01
Ube2hUnknown, score: 0.27
Usp12Unknown, score: 0.11
Ubp1Unknown, score: 0.01
Uchl1Unknown, score: 0.05
Usp10Unknown, score: 0.26
Ucp2Unknown, score: 0.6
Ucp3Unknown, score: 0.39
Slc35a2Unknown, score: 0.35
UgdhUnknown, score: 0.2
Dpysl3Unknown, score: 0
UmpsUnknown, score: 0.06
UngUnknown, score: 0.23
Nr1h2Unknown, score: 0.19
UoxUnknown, score: 0.07
Upk1bUnknown, score: 0.09
Upk2Unknown, score: 0.72
Upp1Unknown, score: 0
UqcrqUnknown, score: 0.07
UrodUnknown, score: 0.14
Scgb1a1Unknown, score: 0.03
UtrnUnknown, score: -0.01
UxtUnknown, score: 0.63
Vamp1Unknown, score: 0.07
Vamp2Unknown, score: 0.04
Vamp3Unknown, score: 0.01
Vamp8Unknown, score: 0.01
Vav1Unknown, score: 0.38
Vav2Unknown, score: 0.08
Vcam1Unknown, score: 0.16
Vdac1Unknown, score: 0.01
Vdac2Unknown, score: 0.13
Vdac3Unknown, score: 0.37
VdrUnknown, score: 0.18
VegfaUnknown, score: 0.15
VegfbUnknown, score: 0.25
VegfcUnknown, score: 0.04
Lin7bUnknown, score: 0.05
Vezf1Unknown, score: 0.05
Vil1Unknown, score: 0.38
VipUnknown, score: 0.36
Vipr1Unknown, score: 0.07
Vipr2Unknown, score: 0.27
VldlrUnknown, score: 0.05
Vnn1Unknown, score: 0.15
Vpreb3Unknown, score: 0.07
Trpv2Unknown, score: 0.03
VwfUnknown, score: 0.12
WasUnknown, score: 0.26
Wbp1Unknown, score: 0.1
Fmnl3Unknown, score: 0.14
Wbp5Unknown, score: 0.07
Eif4hUnknown, score: 0.19
Wisp2Unknown, score: 0.09
Wnt2Unknown, score: 0.09
Wnt2bUnknown, score: 0.07
Wnt5aUnknown, score: 0.04
Wnt5bUnknown, score: 0.32
Wnt7aUnknown, score: 0.08
WrnUnknown, score: 0.12
Wt1Unknown, score: 0.17
Xbp1Unknown, score: 0
XdhUnknown, score: 0.05
Xirp1Unknown, score: 0.16
XkUnknown, score: 0.17
XlrUnknown, score: 0.17
Xlr3aUnknown, score: 0.07
AtrxUnknown, score: 0.28
XpaUnknown, score: 0.48
Ercc5Unknown, score: 0.08
Xrcc1Unknown, score: 0.14
Xrcc5Unknown, score: 0.12
Yap1Unknown, score: 0.2
Ybx1Unknown, score: 0.17
Yes1Unknown, score: 0.17
Slc23a3Unknown, score: 0.28
YwhahUnknown, score: 0.12
YwhazUnknown, score: 0.03
Zap70Unknown, score: 0.38
Zbtb17Unknown, score: 0.1
Zfp101Unknown, score: 0.03
Zfp105Unknown, score: 0.03
Zfp148Unknown, score: -0.01
Zbtb14Unknown, score: 0.08
Zfp185Unknown, score: 0.32
Zfp2Unknown, score: 0.05
Zfp207Unknown, score: 0.09
Zfp239Unknown, score: 0.16
Zfp26Unknown, score: 0.18
Zfp27Unknown, score: 0.33
Zfp28Unknown, score: 0.32
Zscan2Unknown, score: 0.16
Zfp35Unknown, score: 0.12
Zfp36Unknown, score: 0.05
Zfp37Unknown, score: 0.26
Zscan21Unknown, score: 0.09
Zfp46Unknown, score: 0.12
Zfp52Unknown, score: 0.03
Zfp57Unknown, score: 0.31
Zfp59Unknown, score: 0.33
Zfp60Unknown, score: 0.05
Zfp61Unknown, score: 0.09
Zfp64Unknown, score: 0.26
Zbtb7bUnknown, score: 0.22
Zfp85Unknown, score: 0
Zfp9Unknown, score: 0.05
Zkscan5Unknown, score: 0.09
Zscan12Unknown, score: 0.05
Ikzf1Unknown, score: 0
Ikzf2Unknown, score: 0.12
Ikzf3Unknown, score: 0.23
Slc30a1Unknown, score: 0.1
Zp2Unknown, score: 0.25
Adam23Unknown, score: 0.15
Adamts5Unknown, score: 0.03
Akt3Unknown, score: -0.01
Arih2Unknown, score: 0.42
Bace1Unknown, score: 0.37
Capn10Unknown, score: 0.29
Cdc6Unknown, score: 0.46
Cdh20Unknown, score: 0.21
Clec5aUnknown, score: 0.07
Def6Unknown, score: 0.17
Dido1Unknown, score: 0.1
Ets1Unknown, score: 0.06
FaimUnknown, score: 0.21
Fbln5Unknown, score: 0.02
FybUnknown, score: 0.02
G3bp2Unknown, score: 0.26
Gadd45gUnknown, score: 0.22
Gmcl1Unknown, score: 0.39
Gdf15Unknown, score: 0.01
Gpc6Unknown, score: 0.01
Gpr34Unknown, score: 0.17
Grem2Unknown, score: 0.19
Hs2st1Unknown, score: 0.32
Impdh1Unknown, score: -0.01
Impdh2Unknown, score: 0.07
JtbUnknown, score: 0.13
KelUnknown, score: 0.43
Ly6hUnknown, score: 0.44
Lynx1Unknown, score: 0.18
Mab21l2Unknown, score: 0.05
Mapk7Unknown, score: 0.05
MgllUnknown, score: 0.47
Mid2Unknown, score: 0.04
Mmp17Unknown, score: 0.02
Dnajb6Unknown, score: 0.11
Nek4Unknown, score: 0.23
Neu2Unknown, score: 0.23
Nr0b2Unknown, score: 0.41
Oas1gUnknown, score: 0.01
Oasl2Unknown, score: 0.16
Tenm1Unknown, score: 0
Tenm3Unknown, score: 0.03
Tenm4Unknown, score: 0.02
Osr1Unknown, score: 0.01
Pacsin1Unknown, score: 0.08
Papss1Unknown, score: 0.32
Papss2Unknown, score: 0.06
Slc26a4Unknown, score: 0.67
Cib1Unknown, score: 0
PrkraUnknown, score: 0
Klk7Unknown, score: 0.32
Psmc4Unknown, score: 0.14
Psmd13Unknown, score: 0.47
Tiam2Unknown, score: 0.24
Rai2Unknown, score: 0.07
IkUnknown, score: 0.47
Rgs7Unknown, score: 0.13
Grk1Unknown, score: 0.18
RnaselUnknown, score: 0.17
Abce1Unknown, score: 0.17
Rnf13Unknown, score: 0.08
RngttUnknown, score: 0.11
Scamp2Unknown, score: 0.02
Scamp3Unknown, score: 0.05
Sh3bp2Unknown, score: 0.39
Slco2a1Unknown, score: 0.07
Slc35a1Unknown, score: 0.1
Smc1aUnknown, score: 0.22
Spry1Unknown, score: 0.26
Spry2Unknown, score: 0.44
Spry4Unknown, score: 0.18
SufuUnknown, score: 0.24
Mpdu1Unknown, score: 0.13
Synj2bpUnknown, score: 0.15
Taf7Unknown, score: 0.15
Natd1Unknown, score: 0.02
Tlk2Unknown, score: 0.29
Tnfsf13bUnknown, score: 0.06
Ubl3Unknown, score: 0.12
Best1Unknown, score: 0.17
NelfaUnknown, score: 0.41
Xrn1Unknown, score: 0.41
Xrn2Unknown, score: 0.13
Ldb3Unknown, score: 0.16
Zfp53Unknown, score: 0.17
Abcg2Unknown, score: 0.3
Adgre5Unknown, score: 0
Ciao1Unknown, score: 0
Clcn7Unknown, score: 0.17
Dapp1Unknown, score: 0
EsrraUnknown, score: 0.28
EsrrgUnknown, score: 0.05
Fgd2Unknown, score: 0.09
Grk6Unknown, score: 0.07
Map2k2Unknown, score: 0.04
Map2k3Unknown, score: 0.06
Map3k1Unknown, score: 0.01
Map3k11Unknown, score: 0.02
Map3k4Unknown, score: 0.19
Map3k5Unknown, score: 0.01
Map3k8Unknown, score: 0.13
Map4k1Unknown, score: 0.09
Mapk13Unknown, score: 0.49
Mapk8Unknown, score: 0.16
NbeaUnknown, score: 0.08
Nr5a1Unknown, score: 0.18
Nubp1Unknown, score: 0.06
Orc4Unknown, score: 0.28
Git2Unknown, score: 0.32
Plod3Unknown, score: 0.19
Psg16Unknown, score: 0.01
Psg17Unknown, score: 0.09
Psma1Unknown, score: 0.27
Psma4Unknown, score: 0.17
Psma5Unknown, score: 0.13
Psma6Unknown, score: 0.34
Psma7Unknown, score: 0.18
Psmb2Unknown, score: 0.11
MokUnknown, score: 0.09
Rbbp9Unknown, score: 0.4
Rpl27aUnknown, score: 0.16
Sema4gUnknown, score: 0.3
Slc27a1Unknown, score: 0.19
Slc27a2Unknown, score: 0.19
Slc27a5Unknown, score: 0.19
Vnn3Unknown, score: 0.12
Zfp146Unknown, score: 0.06
Itgb1bp2Unknown, score: 0.01
Homer1Unknown, score: 0.18
Homer3Unknown, score: 0.29
Mmp23Unknown, score: 0.08
Rcn2Unknown, score: 0.05
Cops5Unknown, score: 0.16
Dpysl4Unknown, score: 0.35
Abcd2Unknown, score: 0.12
PcloUnknown, score: 0.21
B3galt1Unknown, score: 0.45
Clec4a2Unknown, score: 0.06
Cln8Unknown, score: 0.18
Cops4Unknown, score: 0.18
Cops6Unknown, score: 0.31
Med14Unknown, score: 0.76
Ddx3yUnknown, score: 0
DysfUnknown, score: 0.21
Eif2s3xUnknown, score: 0.22
Exo1Unknown, score: 0.27
Map4k4Unknown, score: 0.02
Ppp2r5cUnknown, score: 0.03
MpripUnknown, score: 0.1
St6galnac5Unknown, score: 0.05
Polr3eUnknown, score: 0.12
EcsitUnknown, score: 0.19
Slc9a3r1Unknown, score: 0.06
Spag1Unknown, score: 0.03
Zw10Unknown, score: 0.07
Pla2g2fUnknown, score: 0.02
Spo11Unknown, score: 0
Tspan32Unknown, score: 0.11
SgshUnknown, score: 0.28
G3bp1Unknown, score: 0.17
Nit1Unknown, score: 0.35
OmdUnknown, score: 0.16
Etv3Unknown, score: 0.26
Ncoa4Unknown, score: 0.09
Tcirg1Unknown, score: 0.05
B9d1Unknown, score: 0.33
Trappc3Unknown, score: -0.01
Rpl7aUnknown, score: 0.23
PodxlUnknown, score: 0.26
NrkUnknown, score: 0.29
Dbf4Unknown, score: 0.04
Azi2Unknown, score: 0.1
Slamf1Unknown, score: 0.12
Chaf1aUnknown, score: 0.15
CarsUnknown, score: 0.35
Pdk4Unknown, score: 0.17
Zfp354bUnknown, score: 0.35
Nufip1Unknown, score: 0.33
Plekhb1Unknown, score: 0.07
Golga5Unknown, score: 0.2
Phlda3Unknown, score: 0.25
HraslsUnknown, score: 0.2
NbnUnknown, score: 0.17
Insl6Unknown, score: 0.4
GygUnknown, score: 0.05
Add3Unknown, score: 0.04
Msrb1Unknown, score: 0.24
SrrUnknown, score: 0.2
Txnl4aUnknown, score: 0.24
Rpl3Unknown, score: 0
Tbl2Unknown, score: 0.17
DguokUnknown, score: 0.18
Sh2d2aUnknown, score: 0.16
Csnk1eUnknown, score: 0.62
Tjp3Unknown, score: 0.25
Slc25a10Unknown, score: 0.12
Yme1l1Unknown, score: 0.05
Akr1c13Unknown, score: 0.04
Npas3Unknown, score: 0.16
Sh2d3cUnknown, score: 0.04
Ptdss2Unknown, score: 0.06
Dusp13Unknown, score: 0.12
Mrpl39Unknown, score: 0.07
Mrpl15Unknown, score: 0.05
Mrpl17Unknown, score: 0.05
Ip6k1Unknown, score: 0.1
Abca7Unknown, score: 0.19
Abcg3Unknown, score: 0.1
Abcf2Unknown, score: 0.2
Abcg5Unknown, score: 0
Peg12Unknown, score: 0.26
SergefUnknown, score: 0.17
Abcc5Unknown, score: 0.04
Mkln1Unknown, score: 0.26
NagluUnknown, score: 0.17
Abcc6Unknown, score: 0.05
Shroom3Unknown, score: 0.15
AmotUnknown, score: 0.07
NrepUnknown, score: 0.13
NelfeUnknown, score: 0.22
Snf8Unknown, score: 0.01
Vwa7Unknown, score: 0.23
Zdhhc8Unknown, score: 0.12
Tada1Unknown, score: 0.59
Tango2Unknown, score: 0.18
Spg21Unknown, score: 0.27
CherpUnknown, score: 0.1
Eif3bUnknown, score: 0.12
Efhd2Unknown, score: 0.19
Imp4Unknown, score: 0.24
Fam3cUnknown, score: 0.11
Prpf19Unknown, score: 0.12
MiipUnknown, score: 0.14
Polr2mUnknown, score: 0.11
Ubfd1Unknown, score: 0.11
Mrpl50Unknown, score: 0.41
Gfm1Unknown, score: 0.11
Usp39Unknown, score: 0.38
Yipf3Unknown, score: 0.32
Desi1Unknown, score: 0.16
Med10Unknown, score: 0.2
Fam104aUnknown, score: 0.23
Vps25Unknown, score: 0.15
MydgfUnknown, score: 0.06
Tinf2Unknown, score: 0.42
Nsun2Unknown, score: 0.02
Nop16Unknown, score: 0.29
Serp1Unknown, score: 0.37
Agpat3Unknown, score: 0
Tomm70aUnknown, score: 0.26
Reep3Unknown, score: 0.11
Dcaf11Unknown, score: 0.51
Trpm2Unknown, score: 0.22
Slco1a1Unknown, score: -0.01
Slco1a4Unknown, score: 0.04
Slco1b2Unknown, score: -0.01
D10Jhu81eUnknown, score: 0.1
Limd1Unknown, score: 0.24
Rabgap1lUnknown, score: 0.02
Bag3Unknown, score: 0.09
Zfp385aUnknown, score: 0.28
SmtnUnknown, score: 0.07
Mapk12Unknown, score: 0.1
Pmm1Unknown, score: 0.09
Pde7bUnknown, score: 0.07
Gtse1Unknown, score: 0.14
Scmh1Unknown, score: 0.11
Iqgap1Unknown, score: 0.05
Clic4Unknown, score: 0.01
Opn4Unknown, score: 0.16
Zfp292Unknown, score: 0.17
Scd3Unknown, score: 0.06
Pcsk1nUnknown, score: 0.07
Timm9Unknown, score: 0.12
Timm8bUnknown, score: 0.1
Timm8a1Unknown, score: 0.33
Timm10Unknown, score: 0.15
Pdlim4Unknown, score: 0.33
Fbxw5Unknown, score: 0.16
Kdm2bUnknown, score: 0.18
Fbxl12Unknown, score: 0.09
Mlf2Unknown, score: 0.38
Gnl3Unknown, score: 0.18
AplnUnknown, score: 0.17
Angptl3Unknown, score: 0.08
Slamf6Unknown, score: 0.65
Glrx3Unknown, score: 0.79
Snai3Unknown, score: 0.02
Zbtb18Unknown, score: 0.24
Vps26aUnknown, score: 0.06
Tor1aUnknown, score: 0.06
Tor1bUnknown, score: 0.02
Tor3aUnknown, score: 0.28
Slc46a2Unknown, score: 0.52
Fgd3Unknown, score: 0.21
Usp25Unknown, score: 0.1
Zfp354cUnknown, score: 0.16
Abt1Unknown, score: 0.2
Adat1Unknown, score: 0.08
Bin1Unknown, score: 0.21
Cbx8Unknown, score: 0.75
Cngb3Unknown, score: 0
Pik3cgUnknown, score: 0.17
AassUnknown, score: 0.11
VapaUnknown, score: -0.01
Nox4Unknown, score: 0.12
E2f6Unknown, score: 0.08
Ebi3Unknown, score: 0.37
TtpaUnknown, score: 0.03
Spag6Unknown, score: 0.01
Tmprss2Unknown, score: 0.11
Hist1h1cUnknown, score: 0.39
IcoslUnknown, score: 0.14
Sap30lUnknown, score: 0.13
Fbxw7Unknown, score: 0.07
Fbxl17Unknown, score: 0.12
Fbxo6Unknown, score: 0
Crim1Unknown, score: 0.05
Dlc1Unknown, score: 0.05
Nt5cUnknown, score: 0.11
Rgs6Unknown, score: 0.03
Rgs11Unknown, score: 0.24
Ppap2cUnknown, score: 0.06
Fbxl8Unknown, score: 0.18
Acsl4Unknown, score: 0.45
Magi2Unknown, score: 0.27
Sh3bgrUnknown, score: 0.13
GneUnknown, score: 0.26
Rnf10Unknown, score: 0.18
SpastUnknown, score: 0.13
Park2Unknown, score: 0.51
Tmod2Unknown, score: -0.01
SclyUnknown, score: 0.09
Chek2Unknown, score: 0.05
Nckap1Unknown, score: 0.02
Il17rbUnknown, score: 0.17
C1raUnknown, score: 0.13
Exosc9Unknown, score: -0.01
Exosc10Unknown, score: 0.25
Grb14Unknown, score: 0.01
MyadmUnknown, score: 0
HnrnpdlUnknown, score: 0.03
NaspUnknown, score: 0.06
Tnfsf14Unknown, score: 0.07
Mink1Unknown, score: 0.4
Uba2Unknown, score: 0.15
Rgs14Unknown, score: 0
SrpxUnknown, score: 0.32
Srrm1Unknown, score: 0.02
CtpsUnknown, score: 0.32
BokUnknown, score: 0.84
Ramp1Unknown, score: 0.07
HnrnpuUnknown, score: 0.42
Mcrs1Unknown, score: 0.05
Rif1Unknown, score: 0.09
Tubgcp4Unknown, score: 0.08
Atg13Unknown, score: 0.03
Rnf24Unknown, score: 0.06
Kctd18Unknown, score: 0.08
Cdk2ap2Unknown, score: 0.06
Nus1Unknown, score: 0.22
Ppp6r3Unknown, score: 0.05
Ppp1r10Unknown, score: 0.32
Rab11fip5Unknown, score: 0.04
Coq5Unknown, score: 0.02
Mfhas1Unknown, score: 0.07
PvrUnknown, score: -0.01
HgsnatUnknown, score: 0.39
Agpat5Unknown, score: 0.08
Kcnk6Unknown, score: 0.34
Camk1Unknown, score: 0.17
Tmem222Unknown, score: 0.21
Odf2lUnknown, score: 0.11
RragdUnknown, score: 0.13
Rbm34Unknown, score: 0.03
Commd2Unknown, score: 0.19
Cdca8Unknown, score: 0.09
Adgra1Unknown, score: -0.01
D1Ertd622eUnknown, score: 0.05
Rhpn2Unknown, score: 0.29
Ppp2r2dUnknown, score: 0.08
Tax1bp1Unknown, score: 0.18
Tet1Unknown, score: 0.12
Ctdsp2Unknown, score: 0.02
Coa3Unknown, score: 0.01
CenpoUnknown, score: 0.22
Ddx56Unknown, score: 0.35
Zfp622Unknown, score: 0.19
Nhp2Unknown, score: 0.12
Mettl17Unknown, score: 0.37
Acaa2Unknown, score: 0.14
SgtaUnknown, score: 0.32
Parp8Unknown, score: 0.23
Cdc23Unknown, score: 0.14
Brms1lUnknown, score: 0.26
Adgre4Unknown, score: 0.07
Suz12Unknown, score: 0.14
Cdkn2aipnlUnknown, score: 0.13
Esyt2Unknown, score: 0.12
Cisd1Unknown, score: 0.23
Wipi1Unknown, score: 0.07
Ncaph2Unknown, score: 0.13
Cd300lgUnknown, score: 0.07
Setd3Unknown, score: 0.3
ZwintUnknown, score: 0.3
Txndc17Unknown, score: 0.19
Krr1Unknown, score: 0.04
Slc52a2Unknown, score: 0.25
Zkscan6Unknown, score: 0.25
Ccdc43Unknown, score: 0.5
Anapc16Unknown, score: 0.1
Tspyl2Unknown, score: 0.81
LdhdUnknown, score: 0.11
Rufy3Unknown, score: 0.21
Lurap1lUnknown, score: 0.53
Tmx4Unknown, score: 0.01
Dbndd2Unknown, score: 0.01
Sgsm1Unknown, score: 0.29
Lair1Unknown, score: 0.32
Gramd1aUnknown, score: 0.02
CdiptUnknown, score: 0.02
Slx4Unknown, score: 0.31
Rgs7bpUnknown, score: 0.1
Sco1Unknown, score: 0
Rbfox3Unknown, score: 0.25
Zmiz2Unknown, score: 0.33
Dlg3Unknown, score: 0
MybphUnknown, score: 0.23
BatfUnknown, score: 0.08
Nxf1Unknown, score: 0.05
BanpUnknown, score: 0.02
Pgrmc1Unknown, score: 0.03
Vamp4Unknown, score: 0.07
Tomm40Unknown, score: 0.13
Gosr1Unknown, score: 0.22
Mtx2Unknown, score: 0.02
Usp2Unknown, score: 0.01
SdcbpUnknown, score: 0.19
Hnrnpa2b1Unknown, score: 0.12
Prdx4Unknown, score: 0.15
Ppp1r3cUnknown, score: 0.39
ByslUnknown, score: 0.07
Stk39Unknown, score: 0.25
Dctn3Unknown, score: 0.17
Timm23Unknown, score: 0.3
TslpUnknown, score: 0.21
ZpbpUnknown, score: 0.13
Nap1l1Unknown, score: 0
Map3k6Unknown, score: 0.05
ClasrpUnknown, score: 0.04
NonoUnknown, score: 0.02
Vti1aUnknown, score: 0.17
Fut8Unknown, score: 0.09
BlcapUnknown, score: 0.12
Vamp5Unknown, score: 0.14
Cnot4Unknown, score: 0.09
Cldn7Unknown, score: 0.06
B3gnt2Unknown, score: 0.14
Oaz3Unknown, score: 0.06
Ddx39bUnknown, score: 0.34
Tuba8Unknown, score: 0.37
Sept9Unknown, score: 0.01
Col5a3Unknown, score: 0.39
Rab11aUnknown, score: 0.21
Pkd2l2Unknown, score: 0.01
Ear6Unknown, score: 0.16
Nphp1Unknown, score: 0.24
Cdkl2Unknown, score: 0.38
Sart3Unknown, score: 0.21
Nudt5Unknown, score: 0.2
Slc7a10Unknown, score: 0.07
Rcan3Unknown, score: 0.2
Slc40a1Unknown, score: 0.19
Gpatch11Unknown, score: 0.17
NgefUnknown, score: 0.11
Ddx20Unknown, score: 0.21
Deaf1Unknown, score: 0.37
Irf7Unknown, score: -0.01
Pmm2Unknown, score: 0.09
Actr1aUnknown, score: 0.29
Irf3Unknown, score: 0.1
LsrUnknown, score: 0.14
AcrbpUnknown, score: 0.29
Atxn10Unknown, score: 0.12
Irf6Unknown, score: 0.47
Spag5Unknown, score: 0.27
Cyhr1Unknown, score: 0.38
Egfl6Unknown, score: 0.15
Copg2Unknown, score: 0.11
RragcUnknown, score: 0.07
Cpsf4Unknown, score: 0.22
Rabep1Unknown, score: 0.17
Gucy1b3Unknown, score: 0.25
Pabpn1Unknown, score: 0.04
Rnf5Unknown, score: 0.03
Snx3Unknown, score: 0.03
Ccrl2Unknown, score: 0.1
Zfp316Unknown, score: 0.24
Sept1Unknown, score: 0.09
Pcdh7Unknown, score: 0.06
Elovl1Unknown, score: 0.18
Elovl2Unknown, score: -0.01
Slc23a2Unknown, score: 0.05
Gnpnat1Unknown, score: 0
Atf7ipUnknown, score: 0.07
Elp5Unknown, score: 0.33
Rassf5Unknown, score: 0.2
Epb4.1l4bUnknown, score: 0.25
Ctnnal1Unknown, score: 0.11
Zfp326Unknown, score: 0.07
Chst2Unknown, score: 0.21
Cacng4Unknown, score: 0.07
Smarcal1Unknown, score: 0.06
Mcm3apUnknown, score: 0.13
Sit1Unknown, score: 0.12
NcapgUnknown, score: 0.12
Ppt2Unknown, score: 0.17
Ndufa1Unknown, score: 0.02
Asah2Unknown, score: -0.01
Cpsf3Unknown, score: 0.09
Mkrn1Unknown, score: 0.26
Dll4Unknown, score: 0.02
Syt7Unknown, score: 0.17
PcnxUnknown, score: 0
Abhd2Unknown, score: 0.11
St3gal6Unknown, score: 0.01
NpffUnknown, score: -0.01
Extl3Unknown, score: 0.07
Paf1Unknown, score: 0.07
Prickle3Unknown, score: 0.14
Nphs1Unknown, score: 0
Ftsj1Unknown, score: 0.18
Pqbp1Unknown, score: 0.09
MagixUnknown, score: 0.35
PdgfcUnknown, score: 0.2
Ccdc22Unknown, score: 0
Otud5Unknown, score: 0.11
Sfmbt1Unknown, score: 0.01
Adgrg3Unknown, score: 0.25
Sh3glb1Unknown, score: 0.19
Zfp108Unknown, score: 0.22
CrtamUnknown, score: 0.05
Eif3iUnknown, score: 0.06
Hs3st3b1Unknown, score: 0.14
Plxnc1Unknown, score: 0.21
Tyk2Unknown, score: 0.31
Tfip11Unknown, score: 0.16
Hes6Unknown, score: 0.06
Gbp3Unknown, score: 0.15
Rp9Unknown, score: 0.13
Fnbp4Unknown, score: 0
Ctps2Unknown, score: 0.04
ApomUnknown, score: 0.13
Sertad1Unknown, score: 0.22
Eif3dUnknown, score: 0.29
Dclre1aUnknown, score: 0.07
SfnUnknown, score: 0.2
Eef1b2Unknown, score: 0.12
Bri3Unknown, score: 0
Mpc1Unknown, score: 0.16
Slc1a4Unknown, score: 0.01
Paxip1Unknown, score: 0.14
Cpxm2Unknown, score: 0.1
Snx12Unknown, score: 0.1
Nop58Unknown, score: 0.13
Trim3Unknown, score: 0.15
Msh4Unknown, score: 0.4
Alyref2Unknown, score: 0.26
Pgam2Unknown, score: 0.16
Srcin1Unknown, score: 0.05
Hebp2Unknown, score: 0.19
Slc2a8Unknown, score: 0.15
Tmem131Unknown, score: 0.33
Nprl2Unknown, score: 0.17
Rplp1Unknown, score: 0.01
Uso1Unknown, score: 0.2
Akr1e1Unknown, score: 0.14
RalaUnknown, score: 0.28
Samhd1Unknown, score: 0.32
MslnUnknown, score: 0.27
Tcerg1Unknown, score: 0.01
Pdss1Unknown, score: 0.46
Astn2Unknown, score: 0.01
Ubqln1Unknown, score: 0.32
Dnah10Unknown, score: 0.18
Ramp3Unknown, score: 0.35
Ftsj3Unknown, score: 0.1
Mad2l1Unknown, score: 0.16
NagkUnknown, score: 0.54
Bace2Unknown, score: 0.07
Olfm1Unknown, score: 0.04
RabggtaUnknown, score: -0.01
Fxyd1Unknown, score: 0.03
Rbm38Unknown, score: -0.01
PlekUnknown, score: 0.04
Prpf40aUnknown, score: 0.25
Ptbp2Unknown, score: 0.22
Tdp2Unknown, score: 0.09
HeylUnknown, score: 0.17
Abcb10Unknown, score: 0.04
Ddx21Unknown, score: 0.05
EnsaUnknown, score: 0.11
Becn1Unknown, score: 0.12
Gde1Unknown, score: 0.02
Scamp4Unknown, score: 0.06
Stx1bUnknown, score: 0
Mpp5Unknown, score: 0.24
Extl1Unknown, score: 0.3
Zfp386Unknown, score: 0.06
Cited4Unknown, score: 0.19
EspnUnknown, score: 0.39
Hdac7Unknown, score: 0.27
Ak3Unknown, score: 0.38
Hnrnph2Unknown, score: 0
Cpxm1Unknown, score: 0.09
Tmem45aUnknown, score: 0.01
Gkap1Unknown, score: 0.14
Fam69bUnknown, score: 0.06
Mrpl12Unknown, score: 0.03
Naa10Unknown, score: 0.03
Ptpn9Unknown, score: -0.01
Higd1aUnknown, score: 0.02
Atl2Unknown, score: 0.12
FkbplUnknown, score: 0.17
PitpnbUnknown, score: 0
Fam60aUnknown, score: 0.14
MycbpUnknown, score: 0.19
Nupr1Unknown, score: 0.34
Dbn1Unknown, score: 0.14
AatfUnknown, score: 0.03
Dnajb5Unknown, score: 0.01
Stam2Unknown, score: 0.01
Abcb9Unknown, score: 0.15
Arl2Unknown, score: 0.22
Pdcd5Unknown, score: 0.09
Amotl2Unknown, score: 0
Tmed2Unknown, score: 0.14
B4galt5Unknown, score: 0.61
TxnipUnknown, score: 0.16
Eif3cUnknown, score: 0.14
Net1Unknown, score: 0.42
Ptges3Unknown, score: 0.01
RybpUnknown, score: 0.21
GltpUnknown, score: 0.51
Copz2Unknown, score: 0.16
Tmeff2Unknown, score: 0.24
Zmym3Unknown, score: 0.12
ClcnkbUnknown, score: 0.11
ScocUnknown, score: -0.01
Cyb561d2Unknown, score: 0.06
ApipUnknown, score: 0.15
Fzr1Unknown, score: 0.02
1110004F10RikUnknown, score: 0.34
Cpb2Unknown, score: 0.03
Arpc3Unknown, score: 0.36
Arid3bUnknown, score: 0.11
SpenUnknown, score: 0.26
Rab9Unknown, score: 0.15
Letm1Unknown, score: 0.04
B4galt6Unknown, score: 0.25
Cyp3a25Unknown, score: 0.04
Stx5aUnknown, score: 0.1
Sssca1Unknown, score: 0.06
Chp1Unknown, score: 0.07
Akap8Unknown, score: -0.01
Ncoa6Unknown, score: -0.01
Nudt3Unknown, score: 0.01
PfkpUnknown, score: 0.05
Pdcd10Unknown, score: 0.16
Tubd1Unknown, score: 0.17
DptUnknown, score: 0.38
Clip1Unknown, score: 0.09
Vps29Unknown, score: 0.17
Nat6Unknown, score: 0.1
Arpc1aUnknown, score: 0.36
Actr10Unknown, score: 0.08
Cyp2d22Unknown, score: 0.21
Ybx3Unknown, score: 0.47
Suclg1Unknown, score: 0.14
Actl6aUnknown, score: 0.04
Clptm1Unknown, score: 0.17
Foxo1Unknown, score: 0
Sae1Unknown, score: -0.01
Pkp3Unknown, score: 0.07
Mtch1Unknown, score: 0.17
Snd1Unknown, score: 0.16
Fads2Unknown, score: 0
Tbk1Unknown, score: 0.38
Foxo3Unknown, score: 0.08
Slc2a5Unknown, score: 0.06
Zbtb20Unknown, score: 0.01
Tspan6Unknown, score: 0.02
Elf4Unknown, score: 0.04
Ankrd49Unknown, score: 0.24
Srpk3Unknown, score: 0.12
Cib2Unknown, score: 0.2
Pard6aUnknown, score: 0.33
Slc22a21Unknown, score: 0.28
Nme4Unknown, score: 0.05
PapolbUnknown, score: 0.54
Mpp6Unknown, score: 0.31
Mast1Unknown, score: 0.28
Sec11aUnknown, score: 0.06
Cnpy2Unknown, score: -0.01
Ripk3Unknown, score: 0.32
Rgs17Unknown, score: 0.43
Hspb3Unknown, score: 0.33
Pex3Unknown, score: 0.06
Klk11Unknown, score: 0.05
Habp4Unknown, score: 0.04
IckUnknown, score: 0.12
Kcnd3Unknown, score: 0.12
Vmn2r1Unknown, score: 0.04
Sec1Unknown, score: 0.11
Ube2d2aUnknown, score: 0.26
Raet1dUnknown, score: 0.03
Rps6ka4Unknown, score: 0.15
Clec4nUnknown, score: 0.17
Dnase2bUnknown, score: 0.11
Trim17Unknown, score: 0.06
Ankrd2Unknown, score: 0.33
Clec7aUnknown, score: 0.64
MlycdUnknown, score: 0.21
CrtapUnknown, score: 0.29
Akap10Unknown, score: 0.28
Ranbp9Unknown, score: 0.14
Ccnl1Unknown, score: 0.02
Clcf1Unknown, score: 0.07
Dnajb12Unknown, score: 0.09
Brinp1Unknown, score: 0.04
MtorUnknown, score: 0.14
LitafUnknown, score: 0.1
Sh3bgrlUnknown, score: 0.05
Tulp2Unknown, score: 0.06
Krt71Unknown, score: 0.09
Alg2Unknown, score: 0.12
Mocs1Unknown, score: 0.18
Rec8Unknown, score: 0.2
DhodhUnknown, score: 0.62
Clec1bUnknown, score: 0.11
Ralgapa1Unknown, score: 0
Tmem9bUnknown, score: 0.27
Supt20Unknown, score: 0.08
Ube2l6Unknown, score: 0.02
Stap1Unknown, score: 0.09
Hacl1Unknown, score: 0.03
Scamp5Unknown, score: 0.19
Cacna2d2Unknown, score: 0.01
Dnajb2Unknown, score: 0.02
Ccl28Unknown, score: 0.61
Lgi1Unknown, score: 0.16
Trpm5Unknown, score: 0.06
Zfp109Unknown, score: 0.1
Lmbr1Unknown, score: 0.03
NsmfUnknown, score: 0.08
Rbms1Unknown, score: 0.06
Dolpp1Unknown, score: 0.18
Zfp276Unknown, score: 0.07
GabrqUnknown, score: 0.07
Vav3Unknown, score: 0.21
Brd4Unknown, score: 0.49
Cxcl14Unknown, score: 0.18
Apba3Unknown, score: 0.37
Slc16a8Unknown, score: 0
LenepUnknown, score: 0.12
Vsig2Unknown, score: -0.01
BcamUnknown, score: 0.43
Mrps31Unknown, score: 0
NelfcdUnknown, score: 0.29
Smpdl3aUnknown, score: -0.01
Park7Unknown, score: 0.08
Terf2ipUnknown, score: 0.16
Gigyf1Unknown, score: 0.31
Jph1Unknown, score: 0.02
ParvaUnknown, score: 0.34
PpbpUnknown, score: 0.13
Cramp1lUnknown, score: 0
Srd5a3Unknown, score: 0.22
Akip1Unknown, score: 0.07
MogsUnknown, score: 0.04
Psors1c2Unknown, score: 0.23
Sult5a1Unknown, score: 0.01
Dnajc4Unknown, score: 0.33
Zc3h8Unknown, score: 0.08
Plin4Unknown, score: 0.15
Gabarapl1Unknown, score: 0
Golga7Unknown, score: 0.22
Tmem183aUnknown, score: 0.01
Ehd3Unknown, score: 0.14
Kcne3Unknown, score: 0.07
Isg20Unknown, score: 0.22
Sec61a2Unknown, score: 0.38
Zfp112Unknown, score: 0.34
JmyUnknown, score: 0.07
Wdr12Unknown, score: 0.07
Rnf25Unknown, score: 0.21
Tacc2Unknown, score: 0.14
Noc3lUnknown, score: 0.2
Fhl5Unknown, score: 0.01
Ntn4Unknown, score: 0.13
Tbx21Unknown, score: 0.06
Wdr4Unknown, score: 0.26
Fmnl1Unknown, score: 0.39
Cd200r1Unknown, score: 0.03
Bin3Unknown, score: 0.3
RangrfUnknown, score: 0.1
CdonUnknown, score: 0.35
Kcne4Unknown, score: 0.21
Spata5Unknown, score: 0.29
TescUnknown, score: 0.21
Eral1Unknown, score: 0.48
Hacd3Unknown, score: 0.69
GsdmaUnknown, score: 0.02
Crlf2Unknown, score: -0.01
Tnfrsf13bUnknown, score: 0.03
Sertad2Unknown, score: 0.05
RhbgUnknown, score: 0.1
Sorcs1Unknown, score: 0.37
Rqcd1Unknown, score: 0.19
Rsad2Unknown, score: 0.09
Sall1Unknown, score: 0.51
Ppp1r1aUnknown, score: 0.02
Zbp1Unknown, score: 0.12
Pdcd1lg2Unknown, score: -0.01
Srrm3Unknown, score: 0.05
Efcc1Unknown, score: 0.07
Dnaja4Unknown, score: 0.03
Pvrl1Unknown, score: 0.07
Nkain4Unknown, score: -0.01
DexiUnknown, score: 0.02
Hs1bp3Unknown, score: 0.07
Nudt11Unknown, score: 0.16
Stx6Unknown, score: -0.01
1700123O20RikUnknown, score: 0.12
FibpUnknown, score: 0.08
Chst11Unknown, score: 0.02
BC100451Unknown, score: 0.09
0610007P14RikUnknown, score: 0.28
Eid1Unknown, score: 0.11
Trim54Unknown, score: 0.13
Elp2Unknown, score: 0.08
Pmaip1Unknown, score: 0.04
1700027J19RikUnknown, score: 0.23
Pex5lUnknown, score: 0.27
Repin1Unknown, score: 0.46
MyotUnknown, score: 0.11
Rps6kb2Unknown, score: 0.03
GhrlUnknown, score: 0.16
Pvrl3Unknown, score: 0.17
Pias4Unknown, score: 0.16
Trappc2lUnknown, score: 0.47
Myoz2Unknown, score: 0
Sh3rf1Unknown, score: 0.15
Moxd1Unknown, score: 0.1
Hnrnph1Unknown, score: 0.23
Rab2aUnknown, score: 0.37
Med12Unknown, score: 0.02
Usp14Unknown, score: 0.07
Huwe1Unknown, score: 0.17
NamptUnknown, score: 0.12
Rcl1Unknown, score: 0.03
Psmd14Unknown, score: 0.01
Chst12Unknown, score: 0
Carm1Unknown, score: -0.01
Dact1Unknown, score: 0.4
Rhot1Unknown, score: 0.24
CopeUnknown, score: 0.19
Wsb2Unknown, score: 0.01
Stard3Unknown, score: 0.14
PnkpUnknown, score: 0.04
C1galt1c1Unknown, score: -0.01
Slc22a17Unknown, score: 0.1
Nsa2Unknown, score: 0.31
Mettl9Unknown, score: 0.28
Hgh1Unknown, score: 0.1
Zfp191Unknown, score: -0.01
Bhlhe22Unknown, score: 0.04
Fxyd6Unknown, score: 0.16
NcstnUnknown, score: 0.2
Wbp11Unknown, score: 0.16
Nrip2Unknown, score: 0.26
Ms4a4bUnknown, score: 0.04
Cldn15Unknown, score: 0.02
DonsonUnknown, score: 0.2
Rbm8aUnknown, score: 0.5
Sap30Unknown, score: 0.15
Trappc4Unknown, score: -0.01
CenpkUnknown, score: 0.87
Doc2gUnknown, score: 0.06
Iigp1Unknown, score: 0.11
Mrpl38Unknown, score: -0.01
Il21rUnknown, score: -0.01
Qtrt1Unknown, score: 0.39
Acss2Unknown, score: 0.21
Fignl1Unknown, score: 0.12
WtapUnknown, score: 0.18
Actn4Unknown, score: 0.04
Gucy1a3Unknown, score: 0.24
Trp53inp1Unknown, score: 0.09
Foxj2Unknown, score: 0.7
Kcnq4Unknown, score: 0.2
Fn3kUnknown, score: 0.04
Taf8Unknown, score: 0.01
Bco1Unknown, score: 0.11
Trpv4Unknown, score: -0.01
Fam129aUnknown, score: 0.08
Dusp10Unknown, score: 0.12
Cables1Unknown, score: 0.18
Slc29a1Unknown, score: 0.08
Sav1Unknown, score: 0.1
NrgnUnknown, score: 0.12
Yeats4Unknown, score: 0.08
Sv2aUnknown, score: 0.01
PerpUnknown, score: 0.14
Smoc2Unknown, score: 0.4
Smoc1Unknown, score: 0.3
Popdc2Unknown, score: 0.12
ParvgUnknown, score: 0.11
CtszUnknown, score: 0.15
Mllt1Unknown, score: 0.42
Sv2bUnknown, score: 0.1
St7Unknown, score: 0.09
Stk32bUnknown, score: 0.22
Gprc5bUnknown, score: 0.17
Dhx38Unknown, score: 0.01
Ms4a4cUnknown, score: 0.16
Ms4a8aUnknown, score: 0.13
Sirt3Unknown, score: 0.14
Cyp4f14Unknown, score: 0.26
Sp5Unknown, score: 0.34
FcamrUnknown, score: 0
Inpp5eUnknown, score: 0.06
Tspan4Unknown, score: 0.01
NischUnknown, score: 0.02
Mrps22Unknown, score: 0.16
Mrps23Unknown, score: 0.12
Mrps10Unknown, score: 0.27
Mrps25Unknown, score: 0.03
Mrps14Unknown, score: 0.03
Mrps24Unknown, score: 0.23
NmiUnknown, score: 0.12
Htra2Unknown, score: 0.09
Scube1Unknown, score: 0.2
Lpin2Unknown, score: 0.16
Lpin3Unknown, score: 0.18
Tsc1Unknown, score: 0.02
Ap3m2Unknown, score: 0.15
Pes1Unknown, score: 0.27
Cldn12Unknown, score: 0.4
Rpl23Unknown, score: 0.25
Zfand6Unknown, score: 0.05
Arl6ip4Unknown, score: 0.06
Ndfip1Unknown, score: 0.15
Slc15a3Unknown, score: 0.14
Dpysl5Unknown, score: 0.64
Twsg1Unknown, score: 0.33
Utp3Unknown, score: 0.15
Slc9a3r2Unknown, score: 0.14
Lima1Unknown, score: 0.1
Ifi30Unknown, score: 0.22
AsphUnknown, score: 0.09
Tmem254aUnknown, score: 0.13
Atp5dUnknown, score: 0.23
Dtd1Unknown, score: 0.2
Ndufb5Unknown, score: 0.15
Mrpl54Unknown, score: 0.2
Emc6Unknown, score: 0.04
RogdiUnknown, score: 0
Ppil2Unknown, score: 0.07
Cndp2Unknown, score: 0.12
Zfp524Unknown, score: 0.1
Tmem176aUnknown, score: -0.01
Tctex1d2Unknown, score: 0.03
Gng11Unknown, score: 0.12
Gtpbp8Unknown, score: 0.29
SnupnUnknown, score: 0.1
Cwc15Unknown, score: 0.02
Ethe1Unknown, score: 0.25
Sdhaf2Unknown, score: 0.33
Tmem167Unknown, score: 0.18
Chchd3Unknown, score: 0.4
Aurkaip1Unknown, score: 0.09
Tsen34Unknown, score: 0.26
Abhd6Unknown, score: 0.14
Setd6Unknown, score: 0.2
Rmnd1Unknown, score: 0.27
FopnlUnknown, score: 0.3
Ypel3Unknown, score: 0.13
Ndufa3Unknown, score: 0.08
GhitmUnknown, score: 0.28
Lamtor4Unknown, score: 0.19
Chchd6Unknown, score: 0.11
Cxcl16Unknown, score: 0.16
Ube2d3Unknown, score: 0.09
SmpxUnknown, score: 0.13
Ndufa9Unknown, score: 0.32
Tmed3Unknown, score: 0.23
Marc1Unknown, score: 0.17
Apoa5Unknown, score: 0.35
Dnajc30Unknown, score: 0.28
Cml1Unknown, score: 0.22
1110001J03RikUnknown, score: 0.13
Fkbp11Unknown, score: 0.11
Chchd1Unknown, score: 0.11
Elof1Unknown, score: 0.09
Mrps36Unknown, score: 0.2
TipinUnknown, score: 0.11
1110008L16RikUnknown, score: 0.08
Znrd1Unknown, score: 0.08
Wbscr22Unknown, score: 0.3
Ifitm3Unknown, score: 0.15
Cox7bUnknown, score: 0.11
Eef1e1Unknown, score: 0.03
Atp6v1fUnknown, score: 0.05
Necap2Unknown, score: 0.1
Uqcr10Unknown, score: 0.38
Cxx1aUnknown, score: 0.05
Pop4Unknown, score: 0.17
Bola2Unknown, score: 0.13
Mrpl4Unknown, score: 0.04
Nip7Unknown, score: 0.15
BccipUnknown, score: 0.33
Tomm7Unknown, score: 0.26
Chchd5Unknown, score: 0.01
PglsUnknown, score: 0.02
Med11Unknown, score: 0.49
Mustn1Unknown, score: 0.08
Nat9Unknown, score: 0.06
Ubl5Unknown, score: 0.13
Ogfod3Unknown, score: 0.34
P3h4Unknown, score: 0.28
Nop10Unknown, score: 0.1
1110037F02RikUnknown, score: 0.16
Acer3Unknown, score: 0.06
Ier3ip1Unknown, score: 0.14
Lage3Unknown, score: 0.28
Pithd1Unknown, score: 0.12
Commd4Unknown, score: 0.06
Commd6Unknown, score: 0.13
Vta1Unknown, score: 0.1
1110059G10RikUnknown, score: 0.11
Cd302Unknown, score: 0.1
1110059E24RikUnknown, score: 0.16
Sec61bUnknown, score: 0.14
Med7Unknown, score: 0.01
RgccUnknown, score: 0.03
Serpinb1aUnknown, score: 0.03
Mrpl35Unknown, score: 0.18
LlphUnknown, score: 0.24
Rpl7l1Unknown, score: 0.23
Mrps28Unknown, score: 0.41
Thoc7Unknown, score: 0.15
Atp6v1g2Unknown, score: 0.11
Mrps16Unknown, score: 0.09
Hspbp1Unknown, score: 0.11
OsgepUnknown, score: 0.14
Alg5Unknown, score: 0.3
Arfgap3Unknown, score: 0.47
Aig1Unknown, score: 0.12
Dimt1Unknown, score: 0.34
Hsbp1l1Unknown, score: 0.15
Ssr2Unknown, score: 0.13
Mrps17Unknown, score: -0.01
Camk2n1Unknown, score: 0.09
Tm4sf20Unknown, score: 0.38
Ing5Unknown, score: 0.13
Ccdc28bUnknown, score: 0.01
EappUnknown, score: 0.02
PigylUnknown, score: 0.44
Fam134bUnknown, score: 0.06
Lyrm9Unknown, score: 0.21
Klf15Unknown, score: 0.33
Tmem218Unknown, score: 0.09
Tma16Unknown, score: 0.43
Sec11cUnknown, score: -0.01
Atp6v1g1Unknown, score: 0.36
Smim8Unknown, score: 0.2
Fam3aUnknown, score: 0.14
Inafm1Unknown, score: 0.2
Fam53cUnknown, score: 0.21
Isoc1Unknown, score: 0.16
Tmem128Unknown, score: 0.07
Smurf2Unknown, score: 0.14
Tpd52l2Unknown, score: 0.35
1700020L24RikUnknown, score: 0.06
Atp6v1c1Unknown, score: 0.56
CenppUnknown, score: 0.19
Cdrt4Unknown, score: 0.13
Atp5slUnknown, score: 0.06
Pla2g12aUnknown, score: 0.12
Riiad1Unknown, score: 0.33
Snw1Unknown, score: 0.36
Knop1Unknown, score: 0.07
AdprmUnknown, score: 0.19
Cox20Unknown, score: 0.3
Zfand1Unknown, score: 0.07
Exosc3Unknown, score: 0.26
Ccdc90bUnknown, score: 0.08
Ergic3Unknown, score: 0.1
RtcaUnknown, score: 0.5
Dus2Unknown, score: 0.43
Chmp4cUnknown, score: 0.2
2310011J03RikUnknown, score: 0.1
Dhrs7Unknown, score: 0.15
IscuUnknown, score: 0.17
Srp19Unknown, score: 0.11
Ppp1r7Unknown, score: 0.3
Nudt8Unknown, score: 0.35
CutcUnknown, score: 0.12
AhnakUnknown, score: 0.1
Ccdc82Unknown, score: 0.1
Sar1bUnknown, score: 0.23
TsfmUnknown, score: 0.02
Alkbh7Unknown, score: 0.3
SlnUnknown, score: 0.03
Asf1aUnknown, score: 0.05
Rtfdc1Unknown, score: 0.01
Sac3d1Unknown, score: 0.11
AptxUnknown, score: 0.25
Rsl1d1Unknown, score: 0.13
Mterf3Unknown, score: 0.22
Arrdc4Unknown, score: 0.21
Psmd6Unknown, score: 0.13
Ndufa12Unknown, score: 0.21
Ndufa7Unknown, score: 0.18
Mrpl11Unknown, score: 0.21
Polr2eUnknown, score: 0.02
2410004B18RikUnknown, score: 0.39
Dctpp1Unknown, score: 0.06
Oxld1Unknown, score: 0.13
Slc7a6osUnknown, score: 0.15
Uggt2Unknown, score: 0.09
Fis1Unknown, score: 0.03
Hamp2Unknown, score: 0.27
2010012O05RikUnknown, score: 0.28
Cdc26Unknown, score: 0.28
Spc25Unknown, score: 0.26
Tnfaip8l1Unknown, score: 0.01
Cyc1Unknown, score: 0.08
Exosc7Unknown, score: 0.39
Mrpl20Unknown, score: 0.1
Cnpy4Unknown, score: 0.27
PyurfUnknown, score: 0.25
2810428I15RikUnknown, score: 0.12
Taf12Unknown, score: 0.47
Ska1Unknown, score: 0.12
Fam213bUnknown, score: 0.2
Anp32eUnknown, score: 0.3
Rps23Unknown, score: 0.15
Usmg5Unknown, score: 0.01
Rpl15Unknown, score: 0.07
Exoc2Unknown, score: 0
Rpl36alUnknown, score: 0.15
Polr2lUnknown, score: 0.41
Ndufb3Unknown, score: 0.24
Lamtor1Unknown, score: 0.62
Rnf181Unknown, score: 0.04
ChtopUnknown, score: 0.17
Asrgl1Unknown, score: 0.13
Cul7Unknown, score: 0.18
Pgpep1Unknown, score: 0.48
2810004N23RikUnknown, score: 0.09
Timm50Unknown, score: 0.15
Tceanc2Unknown, score: -0.01
Cmc2Unknown, score: 0.07
Rep15Unknown, score: 0.18
Nipsnap3bUnknown, score: 0.4
PompUnknown, score: 0.02
Immp1lUnknown, score: 0.19
Aggf1Unknown, score: -0.01
Sppl2aUnknown, score: 0.23
Drap1Unknown, score: -0.01
NtpcrUnknown, score: 0.37
Rwdd3Unknown, score: 0.11
Dzip1Unknown, score: 0.04
UqcrhUnknown, score: -0.01
Mis18aUnknown, score: 0.37
Esf1Unknown, score: 0.24
Exosc1Unknown, score: 0.17
Snrnp40Unknown, score: 0.29
Crls1Unknown, score: 0.08
Mad2l1bpUnknown, score: 0.1
Aste1Unknown, score: -0.01
Trim13Unknown, score: 0.18
3110001I22RikUnknown, score: 0.19
Rdm1Unknown, score: 0.04
Tmigd1Unknown, score: 0.17
Gemin2Unknown, score: 0.31
Lrrc57Unknown, score: -0.01
Ms4a4dUnknown, score: 0.13
Abi3Unknown, score: -0.01
Ormdl3Unknown, score: 0.23
Ntmt1Unknown, score: 0
Snrnp27Unknown, score: 0.03
Ubr7Unknown, score: 0.39
Spcs2Unknown, score: 0.15
Cdip1Unknown, score: 0.14
Ogfod2Unknown, score: 0.37
Hiatl1Unknown, score: 0.09
Dph6Unknown, score: 0.32
Sike1Unknown, score: 0.41
Lix1Unknown, score: 0.1
Pspc1Unknown, score: 0.04
RpeUnknown, score: 0.02
Ndnl2Unknown, score: 0.09
Brf2Unknown, score: 0.18
Acp6Unknown, score: 0.27
SltmUnknown, score: 0.13
Srp72Unknown, score: 0.07
Msantd3Unknown, score: 0.19
Hspbap1Unknown, score: 0.58
CcnhUnknown, score: 0
Spryd7Unknown, score: 0.1
Tmed7Unknown, score: 0.18
Pgm1Unknown, score: 0.05
Trappc5Unknown, score: 0.1
Klhl28Unknown, score: 0
Tmem186Unknown, score: 0.08
AspnUnknown, score: 0.16
Chmp3Unknown, score: 0.03
Spryd4Unknown, score: 0.15
Ndufaf3Unknown, score: 0.29
NkaplUnknown, score: 0.15
SbdsUnknown, score: 0.07
Actr2Unknown, score: 0.3
4921524J17RikUnknown, score: -0.01
Ccdc96Unknown, score: 0.2
Tab3Unknown, score: 0.11
Map1lc3aUnknown, score: 0.44
Rnf220Unknown, score: 0.13
Erich2Unknown, score: 0.1
Erlec1Unknown, score: 0.11
4933411K16RikUnknown, score: 0.05
Tmem239Unknown, score: -0.01
Gid4Unknown, score: 0.02
Grtp1Unknown, score: 0.11
Atg10Unknown, score: 0.04
Ube2wUnknown, score: 0.03
9030624G23RikUnknown, score: 0.04
Rbm22Unknown, score: 0.01
Duoxa2Unknown, score: 0.14
PpcdcUnknown, score: 0.13
Thap2Unknown, score: 0.12
Tmem170Unknown, score: 0.17
Fbxo25Unknown, score: 0.01
PycardUnknown, score: 0.19
Rsph3aUnknown, score: 0.16
Acot13Unknown, score: 0.3
Nxf1Unknown, score: 0.11
0610009O20RikUnknown, score: 0.11
Wdr45bUnknown, score: 0.21
Ormdl2Unknown, score: 0.01
Mrpl33Unknown, score: 0.17
Hint3Unknown, score: 0.15
Ppp1r2Unknown, score: 0.35
Trim35Unknown, score: 0.13
Tcf25Unknown, score: 0.19
Plbd1Unknown, score: 0.5
Slc16a9Unknown, score: 0.01
Dnajc10Unknown, score: 0.04
Clec14aUnknown, score: 0.15
Nhlrc2Unknown, score: 0.42
Mfsd1Unknown, score: 0.44
Zfp869Unknown, score: 0.25
1200014J11RikUnknown, score: 0.03
Crnkl1Unknown, score: 0.08
Rsrc1Unknown, score: 0
Pcyox1Unknown, score: 0.07
Appbp2Unknown, score: 0.13
AcadsbUnknown, score: 0.05
Pxdc1Unknown, score: 0.02
Naa16Unknown, score: 0.26
Baiap2l1Unknown, score: 0.17
Fip1l1Unknown, score: -0.01
ProzUnknown, score: 0.25
MtapUnknown, score: 0.45
PccbUnknown, score: 0.26
Tmem107Unknown, score: 0.45
Nudt16l1Unknown, score: 0.11
Kdelr2Unknown, score: 0.14
TonslUnknown, score: 0.06
Myeov2Unknown, score: 0.24
Chordc1Unknown, score: 0.08
Prpf38bUnknown, score: 0.12
Rras2Unknown, score: 0.16
SdhdUnknown, score: 0.26
Trmt6Unknown, score: 0.18
Dsn1Unknown, score: 0.23
Shisa5Unknown, score: 0.17
Ddx18Unknown, score: 0.15
Pqlc1Unknown, score: 0.3
SdhaUnknown, score: 0.08
2310030G06RikUnknown, score: 0.04
Cdca7Unknown, score: 0.22
Dusp26Unknown, score: 0.38
Fam188aUnknown, score: 0.24
Golt1bUnknown, score: 0
Ctu2Unknown, score: 0.27
Edem3Unknown, score: 0.43
Ssbp2Unknown, score: 0.12
Slc25a23Unknown, score: 0.13
Nuf2Unknown, score: 0.32
Pole4Unknown, score: 0.08
Zdhhc6Unknown, score: 0.01
Rassf7Unknown, score: 0.28
Kctd20Unknown, score: 0.02
Zcchc18Unknown, score: 0.27
Psmd12Unknown, score: 0.06
Psmd5Unknown, score: 0.07
Cisd2Unknown, score: 0.35
Rbm7Unknown, score: 0.01
Mettl6Unknown, score: 0.26
Oma1Unknown, score: 0.27
Tbc1d2bUnknown, score: 0.1
Tmem88Unknown, score: 0.05
Use1Unknown, score: -0.01
Rpl11Unknown, score: 0.07
Thap4Unknown, score: 0.15
Mkrn2Unknown, score: 0.14
2610002M06RikUnknown, score: 0.2
FanclUnknown, score: 0.06
Upf3aUnknown, score: 0.29
Dnajb4Unknown, score: 0.26
Mrpl45Unknown, score: 0.06
Pmf1Unknown, score: 0.15
2010109I03RikUnknown, score: 0.32
Rbm25Unknown, score: 0.03
Ift27Unknown, score: 0.12
Syap1Unknown, score: 0.19
Higd2aUnknown, score: 0.38
Chd1Unknown, score: 0.3
Vma21Unknown, score: 0.08
Pus3Unknown, score: -0.01
Ndc80Unknown, score: 0.08
Yaf2Unknown, score: 0.12
Ola1Unknown, score: -0.01
Slc25a53Unknown, score: 0.05
Tmem246Unknown, score: 0.14
2610002M06RikUnknown, score: 0.44
Polr3dUnknown, score: 0.22
Dynlrb1Unknown, score: -0.01
Mon2Unknown, score: 0.2
Magt1Unknown, score: 0.16
1700019D03RikUnknown, score: 0.24
Ctnnbip1Unknown, score: 0.21
Cand2Unknown, score: 0.14
Psmc6Unknown, score: 0
Trak1Unknown, score: 0.37
MmachcUnknown, score: 0.4
Rps10Unknown, score: 0.02
Mettl21aUnknown, score: 0.02
Ptgr1Unknown, score: 0.26
Timm21Unknown, score: 0.02
Zbtb8osUnknown, score: 0.13
NaaaUnknown, score: 0.09
Rpl14Unknown, score: 0.12
BfarUnknown, score: 0.25
Ccdc159Unknown, score: 0.11
MastlUnknown, score: 0.24
NrarpUnknown, score: 0.02
Atp5eUnknown, score: 0.38
Ndufa6Unknown, score: 0.35
Acbd4Unknown, score: 0.25
Mis12Unknown, score: 0.02
Ikzf5Unknown, score: 0.01
Lrrc40Unknown, score: 0.03
Tomm34Unknown, score: 0
Fam103a1Unknown, score: 0.55
Rnf141Unknown, score: 0.04
Rnaseh2bUnknown, score: 0.2
MtdhUnknown, score: 0.15
Smarca2Unknown, score: 0.1
2610301B20RikUnknown, score: 0.3
Sft2d3Unknown, score: 0.26
Eef1gUnknown, score: 0.11
Sclt1Unknown, score: 0.11
Ccdc47Unknown, score: 0.3
Lipt2Unknown, score: 0.04
NraddUnknown, score: 0.22
Cdt1Unknown, score: 0.11
Zmat5Unknown, score: 0.22
Ccdc25Unknown, score: 0
Yipf5Unknown, score: 0.27
Pdzk1ip1Unknown, score: 0.35
Ndufa13Unknown, score: 0.05
Ube2tUnknown, score: 0.06
Zcrb1Unknown, score: 0.14
Spats2lUnknown, score: 0.15
Pfdn1Unknown, score: 0.22
Ccdc77Unknown, score: 0.26
Nde1Unknown, score: 0.29
Eif2s2Unknown, score: 0.36
Utp11lUnknown, score: 0.04
Lsm1Unknown, score: -0.01
Gatad1Unknown, score: 0.48
Armc10Unknown, score: 0.14
Cmtm6Unknown, score: 0.13
Mboat2Unknown, score: 0.25
L3hypdhUnknown, score: 0.11
Plekho1Unknown, score: 0.19
Srfbp1Unknown, score: 0.1
Med29Unknown, score: 0.4
Rnpc3Unknown, score: 0.05
Tmem19Unknown, score: 0.08
Dph7Unknown, score: 0.27
Zkscan14Unknown, score: 0.19
CinpUnknown, score: 0.35
Rpf2Unknown, score: 0.06
2810474O19RikUnknown, score: 0.02
Marc2Unknown, score: 0.07
Cap2Unknown, score: 0.35
Zfp422Unknown, score: -0.01
Cers4Unknown, score: 0.02
Zswim6Unknown, score: 0.01
Uqcc2Unknown, score: 0.39
Agtpbp1Unknown, score: 0.08
Mrpl42Unknown, score: 0.3
Cmtm5Unknown, score: 0.09
Eri1Unknown, score: 0.27
Pagr1aUnknown, score: 0.01
Rpl37Unknown, score: 0.2
Slc25a19Unknown, score: 0.28
Parp6Unknown, score: 0.19
Srek1ip1Unknown, score: 0.02
3110040N11RikUnknown, score: 0.17
PigcUnknown, score: 0.21
Socs4Unknown, score: 0.45
Dock7Unknown, score: 0.07
Gpx7Unknown, score: -0.01
Pbld2Unknown, score: 0.04
NanpUnknown, score: 0.1
1700037H04RikUnknown, score: 0.27
Stk35Unknown, score: 0.06
Cstf1Unknown, score: 0.15
Herc4Unknown, score: 0.06
1700093K21RikUnknown, score: -0.01
QpctlUnknown, score: 0.11
Gtf3c6Unknown, score: 0.14
Jam2Unknown, score: 0.09
QprtUnknown, score: 0.32
Dedd2Unknown, score: 0.15
Brd3Unknown, score: 0.4
Fam132aUnknown, score: 0.06
4833420G17RikUnknown, score: 0.05
Cxxc5Unknown, score: 0.23
Erp29Unknown, score: -0.01
Armcx2Unknown, score: 0.03
Ears2Unknown, score: 0.14
DhddsUnknown, score: 0.11
Eps8l1Unknown, score: 0.34
Adck3Unknown, score: 0.06
Hoga1Unknown, score: 0.17
Ccdc127Unknown, score: 0.4
Ankrd33bUnknown, score: 0.14
Ssr3Unknown, score: 0.24
Xab2Unknown, score: 0.11
Isoc2bUnknown, score: 0.43
RetsatUnknown, score: 0.2
Map1lc3bUnknown, score: 0.26
Plxdc2Unknown, score: 0.31
Pkp2Unknown, score: 0
Klhl13Unknown, score: 0.08
Ergic1Unknown, score: 0.19
NvlUnknown, score: 0.79
Entpd4Unknown, score: 0
Sf3a1Unknown, score: 0
Gpalpp1Unknown, score: 0.34
Abhd5Unknown, score: 0.41
Mtfr1Unknown, score: 0.66
Snap29Unknown, score: 0.06
Ero1lbUnknown, score: -0.01
Abhd15Unknown, score: 0.1
Cwc25Unknown, score: 0.05
Eepd1Unknown, score: 0.21
Polr3gUnknown, score: 0.19
Dhx40Unknown, score: 0.48
Calcoco1Unknown, score: 0.17
Ap4b1Unknown, score: 0.13
Ufl1Unknown, score: 0.12
Mettl16Unknown, score: 0.28
Tmem167bUnknown, score: 0.02
Ccdc50Unknown, score: 0.03
Saysd1Unknown, score: 0.04
Tmed9Unknown, score: 0.9
2610002J02RikUnknown, score: 0.08
Ttc33Unknown, score: 0.03
Trim62Unknown, score: 0.2
Atg12Unknown, score: 0.03
Nudt7Unknown, score: 0.01
Fgfr1op2Unknown, score: 0.13
UqcrbUnknown, score: 0.17
Mfap1aUnknown, score: 0.12
Ppfibp1Unknown, score: 0.15
Ttll4Unknown, score: 0.11
Cog6Unknown, score: 0.25
Slc39a8Unknown, score: 0.17
Larp6Unknown, score: 0.12
Loxl4Unknown, score: 0.02
Alg13Unknown, score: 0.06
Cpeb4Unknown, score: 0.01
Lrrc18Unknown, score: 0.19
Rnf41Unknown, score: 0.16
Tctn3Unknown, score: 0.05
Tespa1Unknown, score: 0.21
Necap1Unknown, score: 0
FibinUnknown, score: 0.23
4930453N24RikUnknown, score: 0.07
Rspry1Unknown, score: 0.11
Ube2r2Unknown, score: 0.45
AasdhpptUnknown, score: 0.09
Lrp2bpUnknown, score: 0.49
Mxra7Unknown, score: 0.05
Tm7sf3Unknown, score: 0.29
Anp32bUnknown, score: 0.22
Samd8Unknown, score: 0.19
Lyrm5Unknown, score: 0.16
Ctdp1Unknown, score: 0.19
4930548H24RikUnknown, score: 0.11
Rabl3Unknown, score: 0.09
Rnf125Unknown, score: 0.3
Dctn4Unknown, score: 0.14
Alkbh8Unknown, score: 0.14
l7Rn6Unknown, score: 0.06
Rpl38Unknown, score: 0.01
Tceb2Unknown, score: -0.01
CutaUnknown, score: 0.08
Rpp21Unknown, score: 0.14
SdhbUnknown, score: 0.42
Mrpl18Unknown, score: 0.07
Pbdc1Unknown, score: 0.16
Aldh3b1Unknown, score: 0.1
HypkUnknown, score: 0.04
Ift74Unknown, score: 0.08
Ost4Unknown, score: 0.12
Rnf149Unknown, score: 0.17
Taf6lUnknown, score: 0.23
Mrpl24Unknown, score: 0.11
Pcnxl4Unknown, score: 0.4
Polr2gUnknown, score: 0.13
Nsmce1Unknown, score: 0.04
Slc25a37Unknown, score: 0.21
Dnajc19Unknown, score: 0.19
Pop1Unknown, score: 0.15
Nudt13Unknown, score: 0.14
Fam114a2Unknown, score: 0.09
Mansc1Unknown, score: 0.08
Iah1Unknown, score: 0.04
Itgb3bpUnknown, score: 0.13
Ccdc130Unknown, score: 0.11
Ttc39dUnknown, score: 0.38
Samsn1Unknown, score: -0.01
EqtnUnknown, score: 0.19
Ddx47Unknown, score: 0.38
PlgrktUnknown, score: 0.21
Jagn1Unknown, score: 0.52
N6amt1Unknown, score: 0.05
Gpatch2Unknown, score: 0.3
Chd8Unknown, score: -0.01
Kat8Unknown, score: 0.1
Loh12cr1Unknown, score: 0.05
Vwa5aUnknown, score: 0
Zfp639Unknown, score: 0.19
Ilf2Unknown, score: 0.02
Plxnd1Unknown, score: 0.03
Zmym4Unknown, score: 0.16
Sfr1Unknown, score: 0.29
Dalrd3Unknown, score: 0.01
RnlsUnknown, score: 0.01
Snrnp48Unknown, score: 0.54
PllpUnknown, score: 0.08
Limd2Unknown, score: 0.06
Snx2Unknown, score: 0.05
TprglUnknown, score: 0.1
Poldip2Unknown, score: 0.02
Ubxn4Unknown, score: -0.01
Sec14l2Unknown, score: 0.14
Derl1Unknown, score: 0.17
Atp1b4Unknown, score: 0.57
Snap47Unknown, score: 0.05
Rer1Unknown, score: 0.39
Idh3aUnknown, score: 0.05
Wdr83Unknown, score: 0.23
Dnajb11Unknown, score: 0.01
Gpsm1Unknown, score: 0.21
Mrpl57Unknown, score: 0.33
Atg3Unknown, score: 0.04
Rab32Unknown, score: 0.03
Rnf115Unknown, score: 0.03
Tmem39aUnknown, score: 0.43
SncaipUnknown, score: 0
Cdca5Unknown, score: 0
Asprv1Unknown, score: 0.09
S100a16Unknown, score: 0.41
Akr1b10Unknown, score: 0.16
2310033P09RikUnknown, score: 0.22
Slc25a11Unknown, score: 0.26
Yipf4Unknown, score: 0.1
Rgs10Unknown, score: 0.01
Lrrc28Unknown, score: 0.31
Enoph1Unknown, score: 0.31
MrrfUnknown, score: 0.33
Nsmce4aUnknown, score: 0.13
Mri1Unknown, score: 0.37
Naa20Unknown, score: 0.04
Tmem33Unknown, score: 0.4
DcxrUnknown, score: 0.22
Camsap2Unknown, score: 0.33
Tmem100Unknown, score: -0.01
Ufm1Unknown, score: 0.26
Rpl4Unknown, score: 0.06
Tmem86aUnknown, score: 0.04
Ccdc80Unknown, score: 0.09
Pef1Unknown, score: 0.32
Cmc1Unknown, score: 0.14
Coq9Unknown, score: 0.13
Ppap2bUnknown, score: 0.27
Zcchc3Unknown, score: 0.08
Ube2fUnknown, score: -0.01
Hcfc2Unknown, score: 0.1
Wdr55Unknown, score: 0.07
Myl12bUnknown, score: 0.1
Prorsd1Unknown, score: 0.18
Rps27lUnknown, score: 0.05
Atp5g2Unknown, score: 0.13
Mesdc2Unknown, score: 0.18
Fbxo28Unknown, score: 0.25
Tubb6Unknown, score: 0.05
Sugt1Unknown, score: 0.14
Setd8Unknown, score: 0.09
Puf60Unknown, score: 0.02
Npc2Unknown, score: 0.08
Atp2b1Unknown, score: 0.03
Mphosph10Unknown, score: 0.1
67978Unknown, score: 0.24
Pdzd9Unknown, score: 0.08
Mrps11Unknown, score: 0.08
Srsf6Unknown, score: 0.37
Ddx59Unknown, score: 0.15
BambiUnknown, score: 0.06
SnrpgUnknown, score: 0.02
ZwilchUnknown, score: 0.29
Trap1Unknown, score: 0.41
MurcUnknown, score: 0.09
Ftsj2Unknown, score: 0.02
Apopt1Unknown, score: 0.19
2810417H13RikUnknown, score: 0.1
Rnf146Unknown, score: 0.2
Emc4Unknown, score: 0.08
Cox19Unknown, score: 0.13
Rbm42Unknown, score: 0.13
Zfp706Unknown, score: 0.04
Chid1Unknown, score: 0.21
Zfp593Unknown, score: 0.07
Mid1ip1Unknown, score: 0.38
Chac2Unknown, score: 0.3
2700060E02RikUnknown, score: 0.05
2700062C07RikUnknown, score: 0.11
MpndUnknown, score: 0.09
Akirin1Unknown, score: 0.3
Nutf2Unknown, score: 0.04
Rps13Unknown, score: 0.03
Ubxn2bUnknown, score: 0.21
Serpina12Unknown, score: 0.32
Atp5sUnknown, score: 0.09
Tm9sf2Unknown, score: 0.22
Pdzd2Unknown, score: 0.01
Lurap1Unknown, score: 0.07
Gltscr2Unknown, score: 0.06
Pdcd2lUnknown, score: 0.11
Gpn3Unknown, score: 0.08
Dusp19Unknown, score: 0.11
Arpc4Unknown, score: 0.13
Yif1aUnknown, score: 0.3
Ncbp2Unknown, score: 0.04
Smarcc2Unknown, score: 0.15
Dynll2Unknown, score: 0.25
Rchy1Unknown, score: 0.13
Fam92aUnknown, score: 0
Sdccag3Unknown, score: 0.07
Mum1Unknown, score: 0.21
ApoolUnknown, score: 0.2
Cmtm3Unknown, score: 0.36
Cep70Unknown, score: 0.1
Fahd2aUnknown, score: 0.43
GcshUnknown, score: 0.44
Upf3bUnknown, score: 0.28
Kdelr1Unknown, score: 0.17
Ino80Unknown, score: 0.09
Arl13bUnknown, score: 0.12
Stx19Unknown, score: 0.38
Fdx1lUnknown, score: -0.01
Spire1Unknown, score: 0.23
NdnfUnknown, score: 0.19
Rpl39lUnknown, score: 0.14
Fam212aUnknown, score: 0.27
HyiUnknown, score: 0.14
Bcas2Unknown, score: 0.5
DenrUnknown, score: 0.24
Leprotl1Unknown, score: 0.14
Ndufb4Unknown, score: 0.21
Hsbp1Unknown, score: 0.12
Ndufb2Unknown, score: 0.1
Ccdc34Unknown, score: 0.21
Rnaseh2cUnknown, score: 0.16
Tmbim4Unknown, score: 0.25
Gsto2Unknown, score: 0.24
Nudt21Unknown, score: 0.18
Efcab2Unknown, score: 0.05
AI846148Unknown, score: 0.03
MturnUnknown, score: 0.09
Fam195aUnknown, score: 0.07
A930018P22RikUnknown, score: -0.01
Ift80Unknown, score: 0.17
Agpat4Unknown, score: 0.29
PdhbUnknown, score: 0.07
Slc25a22Unknown, score: 0.01
Rbm28Unknown, score: 0.16
Pomgnt1Unknown, score: 0.08
Rpa1Unknown, score: 0.05
Mcoln2Unknown, score: 0.13
Mto1Unknown, score: 0.12
Mfsd10Unknown, score: -0.01
Ncapd2Unknown, score: 0.15
Kdelc2Unknown, score: 0.01
Zmym1Unknown, score: 0.47
Lypd2Unknown, score: 0.43
Gstm7Unknown, score: 0.06
ApooUnknown, score: 0.24
Aph1cUnknown, score: 0.16
Nudt22Unknown, score: 0.09
Rab13Unknown, score: 0.27
Sdhaf1Unknown, score: 0.07
Crip2Unknown, score: 0.34
Ccdc88cUnknown, score: 0.05
Ndufb10Unknown, score: 0.44
Sirt5Unknown, score: 0.35
Ndufs3Unknown, score: 0.09
Mul1Unknown, score: 0.24
AspdhUnknown, score: 0.35
Rab14Unknown, score: 0.24
Tmem129Unknown, score: 0.25
Pbld2Unknown, score: 0.04
Ndufa8Unknown, score: 0.05
Ciz1Unknown, score: 0.17
Mogat1Unknown, score: 0.23
Ccdc163Unknown, score: 0.15
G6pc3Unknown, score: 0.16
Nrn1Unknown, score: 0.15
Ankrd13aUnknown, score: -0.01
Fbxl15Unknown, score: 0.16
Rpl34Unknown, score: 0.24
Dusp23Unknown, score: 0.13
Tbc1d10bUnknown, score: 0.25
Ppp1r14aUnknown, score: 0.29
Mrpl14Unknown, score: 0.37
Adipor2Unknown, score: 0.18
Tmem126bUnknown, score: -0.01
Mpzl1Unknown, score: 0.09
Tmem140Unknown, score: 0.01
Zfp579Unknown, score: 0.09
Ndufaf4Unknown, score: 0.37
Arel1Unknown, score: 0.01
Tspan11Unknown, score: 0.02
Vps51Unknown, score: 0.44
Ptx4Unknown, score: -0.01
Ints1Unknown, score: 0
Dcdc2cUnknown, score: 0.14
Tomm5Unknown, score: 0.49
Eml1Unknown, score: 0.23
Evc2Unknown, score: 0.16
Gpr155Unknown, score: 0.34
Mphosph6Unknown, score: 0.19
EcscrUnknown, score: 0.31
Sgol2aUnknown, score: 0.18
TefmUnknown, score: 0.05
Smim14Unknown, score: 0.08
Col6a4Unknown, score: 0.49
CebpzosUnknown, score: 0.14
Ankra2Unknown, score: 0.46
Pdrg1Unknown, score: 0.03
Dpm3Unknown, score: 0.03
Mrps18aUnknown, score: 0.01
CalyUnknown, score: 0.45
Cgref1Unknown, score: 0.2
Cthrc1Unknown, score: 0.31
MocosUnknown, score: 0.33
Syf2Unknown, score: 0.11
Ccdc167Unknown, score: 0.18
Ube2cUnknown, score: 0.14
Gdpd3Unknown, score: 0.09
Mtcl1Unknown, score: 0.04
Cfap57Unknown, score: 0.17
Fbxw9Unknown, score: 0.34
Myct1Unknown, score: 0.19
Tm2d3Unknown, score: 0.05
Fahd1Unknown, score: 0.12
Tmem216Unknown, score: 0.21
Nadk2Unknown, score: -0.01
Tab2Unknown, score: 0.03
Fndc1Unknown, score: 0.1
Scgb3a1Unknown, score: 0.17
Trpm4Unknown, score: 0.19
Fam172aUnknown, score: -0.01
Fitm1Unknown, score: 0.21
Slc44a2Unknown, score: 0.24
Kansl1lUnknown, score: 0.11
Hddc3Unknown, score: 0.06
Gtf2f2Unknown, score: 0.14
Rabl2Unknown, score: 0.05
Ifitm1Unknown, score: 0.39
1110032A03RikUnknown, score: 0.1
Arl8aUnknown, score: 0.16
1110032F04RikUnknown, score: 0.02
Dus1lUnknown, score: 0.1
Lrrc16aUnknown, score: -0.01
Mrps18cUnknown, score: 0.01
Tyw5Unknown, score: 0.04
Angel1Unknown, score: 0.43
AnlnUnknown, score: 0.01
Rreb1Unknown, score: 0.5
Cgrrf1Unknown, score: 0.14
Abhd11Unknown, score: 0.43
Cdhr3Unknown, score: 0.17
Ms4a6dUnknown, score: 0.11
Tmem53Unknown, score: 0.16
MypnUnknown, score: 0.32
NexnUnknown, score: 0.1
SyncUnknown, score: 0.03
1110057K04RikUnknown, score: 0.1
Foxk2Unknown, score: 0.07
Ankrd46Unknown, score: 0.14
Lrrn4clUnknown, score: 0.1
Asb11Unknown, score: 0.13
Dtwd2Unknown, score: 0.18
1190002N15RikUnknown, score: 0.15
Arv1Unknown, score: 0.18
Tmcc2Unknown, score: -0.01
Maf1Unknown, score: 0.21
Prpf6Unknown, score: 0.17
Zfp467Unknown, score: 0
Pygo2Unknown, score: 0.17
Vars2Unknown, score: 0.24
Cdkal1Unknown, score: 0.2
1110065P20RikUnknown, score: 0.84
Rpap1Unknown, score: 0.26
Mospd3Unknown, score: 0.26
Aspscr1Unknown, score: 0.76
Rasl11bUnknown, score: 0.31
Chst8Unknown, score: 0.35
Fam216aUnknown, score: 0.17
1500012F01RikUnknown, score: 0.09
Fam57bUnknown, score: 0.11
Chmp2aUnknown, score: 0.5
Srrm4Unknown, score: 0.15
Paqr6Unknown, score: 0.29
Ctc1Unknown, score: 0.29
NgdnUnknown, score: 0.11
Dcaf12Unknown, score: -0.01
Tamm41Unknown, score: 0.09
Med27Unknown, score: 0.1
HaghlUnknown, score: 0
Nol11Unknown, score: 0.19
Wdr53Unknown, score: 0.17
Snrpa1Unknown, score: 0.25
Prpf31Unknown, score: 0.17
Ssu72Unknown, score: -0.01
Zfp580Unknown, score: 0.1
Mcts1Unknown, score: 0.06
Thap7Unknown, score: 0.13
Prrt2Unknown, score: 0.13
Spcs1Unknown, score: 0.2
Snx15Unknown, score: 0.16
Mitd1Unknown, score: 0.19
Smdt1Unknown, score: 0.05
Lyzl4Unknown, score: 0.01
Zdhhc3Unknown, score: 0.21
Tmem258Unknown, score: 0.1
Isca1Unknown, score: 0.19
Tmem97Unknown, score: 0.04
Ebna1bp2Unknown, score: 0.08
Kdf1Unknown, score: 0.18
Triap1Unknown, score: 0.1
Psmd11Unknown, score: 0.04
GmppaUnknown, score: 0.18
Zcchc9Unknown, score: 0.13
Oxa1lUnknown, score: 0.15
Ascc1Unknown, score: 0.04
Trim15Unknown, score: 0.01
YdjcUnknown, score: 0.07
March5Unknown, score: 0.48
Cnot8Unknown, score: 0.05
1810022K09RikUnknown, score: 0.28
Cdk12Unknown, score: 0.02
2200002J24RikUnknown, score: 0.15
Kbtbd3Unknown, score: 0.16
Snx4Unknown, score: 0.05
LzicUnknown, score: 0.08
Comtd1Unknown, score: 0.15
Mrpl44Unknown, score: 0.08
Bola1Unknown, score: 0.08
Faim3Unknown, score: 0.14
Erp27Unknown, score: 0.05
DymUnknown, score: 0.07
PtmsUnknown, score: 0.09
Srsf11Unknown, score: 0.35
Sat2Unknown, score: 0.32
Plekha4Unknown, score: 0.07
Ddah1Unknown, score: 0.19
Snx24Unknown, score: 0.08
SeltUnknown, score: 0.03
Zfp746Unknown, score: 0.04
Zfp688Unknown, score: 0.3
Gtpbp4Unknown, score: 0.05
Elf2Unknown, score: 0.05
Ing2Unknown, score: 0.09
Rfc3Unknown, score: 0.39
Gins1Unknown, score: 0.08
CtdsplUnknown, score: 0.04
Lrrc46Unknown, score: 0.11
TesclUnknown, score: 0.28
DcpsUnknown, score: 0.04
Pxt1Unknown, score: 0.05
1700008O03RikUnknown, score: 0.19
Necab1Unknown, score: 0.22
Lrrc51Unknown, score: 0.06
Wdfy1Unknown, score: 0.12
Smco2Unknown, score: 0.06
Mocs3Unknown, score: 0.05
Plac8l1Unknown, score: 0.37
Dnajc17Unknown, score: 0.55
Dennd6bUnknown, score: 0.13
1700023F06RikUnknown, score: 0.03
Clic3Unknown, score: 0.13
Commd10Unknown, score: 0.02
Tmem45a2Unknown, score: 0.01
Tmco2Unknown, score: 0.13
Tmem127Unknown, score: 0.15
2300009A05RikUnknown, score: 0.57
Ttc9Unknown, score: 0.01
Nup35Unknown, score: 0.07
Ndufaf5Unknown, score: 0.5
Zfp932Unknown, score: 0.01
Rwdd2aUnknown, score: 0.12
EsamUnknown, score: 0.04
Mrps9Unknown, score: 0.05
Klk10Unknown, score: 0.53
Wdr5bUnknown, score: 0.23
Mapk1ip1Unknown, score: 0.16
2310022B05RikUnknown, score: 0.04
Fam65cUnknown, score: 0.09
Klhdc2Unknown, score: 0.53
Mfsd3Unknown, score: 0.47
HilpdaUnknown, score: 0.36
Smco1Unknown, score: 0.03
Plekhm2Unknown, score: 0.01
Tnfsf13Unknown, score: 0.04
Pcgf3Unknown, score: -0.01
Gpx8Unknown, score: 0.01
LnpUnknown, score: 0.16
Kansl2Unknown, score: 0.28
Arhgef12Unknown, score: 0.07
ClyblUnknown, score: 0.4
Dapk1Unknown, score: 0.12
Exosc8Unknown, score: 0.09
Wdr20Unknown, score: 0.29
MlipUnknown, score: 0.11
Dctn2Unknown, score: 0.15
PirUnknown, score: 0.03
Tmbim1Unknown, score: 0
2310061I04RikUnknown, score: 0.14
Ddx51Unknown, score: 0.28
Psmg4Unknown, score: 0.28
Ccdc115Unknown, score: 0.18
Tmem52Unknown, score: 0.05
Mif4gdUnknown, score: 0.02
Emc10Unknown, score: 0.39
Aarsd1Unknown, score: 0.12
Hddc2Unknown, score: 0.04
Camsap3Unknown, score: 0.03
Slc52a3Unknown, score: 0.22
Col22a1Unknown, score: 0.13
Pin4Unknown, score: 0.53
TfptUnknown, score: 0.06
IpmkUnknown, score: 0.29
CadUnknown, score: 0.3
Nkiras1Unknown, score: 0.14
Rnaseh2aUnknown, score: 0.1
Smyd3Unknown, score: 0.09
Gemin7Unknown, score: 0.04
Nup37Unknown, score: 0.15
TtlUnknown, score: 0.25
Pold4Unknown, score: 0.27
Aldh16a1Unknown, score: 0.02
Zfp511Unknown, score: 0.04
Fbxo7Unknown, score: 0.55
Leng1Unknown, score: 0.13
Tnfaip8l2Unknown, score: 0.38
Ms4a6bUnknown, score: 0.43
Smap2Unknown, score: 0.39
1500009L16RikUnknown, score: 0.22
TprkbUnknown, score: 0.37
Med30Unknown, score: 0.08
Trim32Unknown, score: 0.27
Krtcap3Unknown, score: 0.12
Mzb1Unknown, score: 0
Mterf4Unknown, score: 0.06
Fyttd1Unknown, score: 0.01
Glod5Unknown, score: 0.45
Rab43Unknown, score: 0.03
Pla2g12bUnknown, score: 0.11
Pcgf1Unknown, score: 0.31
Wnk4Unknown, score: 0.03
Tcf23Unknown, score: 0.2
Eif1adUnknown, score: 0.01
Ttc39bUnknown, score: 0.07
A1cfUnknown, score: 0.23
Ppp1r35Unknown, score: 0.06
Ndufa11Unknown, score: 0.09
Thap3Unknown, score: -0.01
SnrpfUnknown, score: 0.09
Vwa9Unknown, score: 0.03
Zfp219Unknown, score: 0.12
Coa7Unknown, score: 0.62
Mfsd11Unknown, score: 0.18
Mrto4Unknown, score: 0.01
Rasip1Unknown, score: 0.06
Rab3bUnknown, score: 0.29
Nup43Unknown, score: 0.05
Nabp2Unknown, score: 0.06
Polr2iUnknown, score: 0.15
Vrk2Unknown, score: 0.26
AgkUnknown, score: 0.07
Apitd1Unknown, score: 0.16
Zfp715Unknown, score: 0.46
Exoc1Unknown, score: 0.07
2810021J22RikUnknown, score: 0.12
Fars2Unknown, score: 0.38
Ptcd3Unknown, score: 0.18
Cdc16Unknown, score: 0.05
Rpp25lUnknown, score: 0.28
Galk2Unknown, score: 0.25
Cep85Unknown, score: 0.13
Ino80bUnknown, score: 0.18
Mcm10Unknown, score: 0.22
Tut1Unknown, score: 0.08
Trnt1Unknown, score: 0.15
Prpf4Unknown, score: 0.2
Degs2Unknown, score: 0.08
Lysmd2Unknown, score: -0.01
Meaf6Unknown, score: -0.01
Ube2q1Unknown, score: 0.08
Sash1Unknown, score: 0.16
Smc4Unknown, score: 0.51
Ifi35Unknown, score: 0.14
SrrdUnknown, score: 0.15
Dpf3Unknown, score: 0.3
2210011C24RikUnknown, score: 0.22
Lrch3Unknown, score: 0.36
Mettl7a1Unknown, score: 0.29
2210016F16RikUnknown, score: 0.31
Ogfrl1Unknown, score: 0.17
Abhd17cUnknown, score: 0.08
Fam162aUnknown, score: 0.01
Taco1Unknown, score: 0.04
Med23Unknown, score: 0.11
Kif18bUnknown, score: 0.01
NarsUnknown, score: -0.01
Ppil3Unknown, score: 0.11
Gorasp2Unknown, score: 0.39
Cd2bp2Unknown, score: 0.18
Poc1aUnknown, score: 0.52
Bhlhb9Unknown, score: 0.02
Gtf3c5Unknown, score: 0.08
Ufsp1Unknown, score: 0.16
Dazap1Unknown, score: 0.1
2010107E04RikUnknown, score: 0.29
Ccbl1Unknown, score: 0.22
Rpf1Unknown, score: 0.22
Mkrn2osUnknown, score: 0.33
Tbc1d13Unknown, score: 0.08
CactinUnknown, score: 0.22
Rabep2Unknown, score: 0.09
Hdac8Unknown, score: 0.11
Ndufab1Unknown, score: 0.16
Arl16Unknown, score: 0.01
PigwUnknown, score: 0.34
Cd3eapUnknown, score: 0.69
IydUnknown, score: 0.13
Secisbp2lUnknown, score: 0.11
Gprc5cUnknown, score: 0.07
St13Unknown, score: 0.15
Derl3Unknown, score: 0.36
Mospd1Unknown, score: 0.36
Tecpr1Unknown, score: 0.41
Ttc9cUnknown, score: 0.29
Asb12Unknown, score: 0.23
Asnsd1Unknown, score: 0.14
Calml3Unknown, score: 0.02
Ints2Unknown, score: 0.31
Tspan15Unknown, score: 0.09
Mier2Unknown, score: 0.11
Polr3bUnknown, score: -0.01
TbceUnknown, score: 0.15
Inf2Unknown, score: 0.17
Cd248Unknown, score: 0.22
Dhrs13Unknown, score: 0.12
CenplUnknown, score: 0
Crtc3Unknown, score: 0.17
Atad2Unknown, score: 0.07
Pnma1Unknown, score: 0.04
Arhgap17Unknown, score: 0.58
BbxUnknown, score: 0.07
Eef2kmtUnknown, score: 0.05
StambpUnknown, score: 0.06
Lrfn2Unknown, score: 0.23
Tmem242Unknown, score: -0.01
Zdhhc2Unknown, score: -0.01
Lrrc56Unknown, score: 0.17
Slc25a33Unknown, score: 0.2
Wars2Unknown, score: 0.17
Txndc16Unknown, score: 0.08
Fam213aUnknown, score: 0.1
Fra10ac1Unknown, score: 0.07
Cpne3Unknown, score: 0.05
Gfod2Unknown, score: 0.29
Pak4Unknown, score: 0.05
Ssfa2Unknown, score: 0.13
EcdUnknown, score: 0.03
MutyhUnknown, score: 0.04
Fbxo33Unknown, score: 0.19
Ankrd24Unknown, score: 0.38
Sugp1Unknown, score: 0.17
Oip5Unknown, score: 0.04
Naa30Unknown, score: 0.11
Tmem144Unknown, score: 0
Sik3Unknown, score: 0.11
Vcpip1Unknown, score: 0.05
Gulp1Unknown, score: 0.31
Utp20Unknown, score: 0.07
Dusp16Unknown, score: 0.11
Nup205Unknown, score: 0.17
Hmha1Unknown, score: 0
Nos1apUnknown, score: 0.15
6330409D20RikUnknown, score: 0.09
Tspan2Unknown, score: 0
KdsrUnknown, score: 0.21
Dclk2Unknown, score: 0.04
Prpf3Unknown, score: 0.18
Gpr173Unknown, score: 0.19
Ggnbp1Unknown, score: 0.14
Prdm5Unknown, score: 0.24
Dennd1cUnknown, score: 0.11
Ubr5Unknown, score: 0.13
Hars2Unknown, score: 0.05
Cep192Unknown, score: 0.48
Pwwp2aUnknown, score: 0.27
Pgrmc2Unknown, score: 0.35
Clec2gUnknown, score: 0.28
Krt25Unknown, score: 0.12
4921507P07RikUnknown, score: 0.08
Spag9Unknown, score: 0.21
Armc3Unknown, score: 0.08
Ccdc81Unknown, score: 0.12
Ints10Unknown, score: 0.07
Ttll7Unknown, score: 0.13
Lpcat2bUnknown, score: 0.08
Trim69Unknown, score: 0.06
Nol8Unknown, score: 0.09
4921539E11RikUnknown, score: 0.11
Mmrn1Unknown, score: 0.17
Pgm2l1Unknown, score: 0.12
Spef1Unknown, score: 0.04
Phf6Unknown, score: 0.31
Prss41Unknown, score: 0.49
Prss55Unknown, score: 0.05
Hexim2Unknown, score: 0.02
Zfp597Unknown, score: 0.06
Arhgap19Unknown, score: 0.12
Cdkl1Unknown, score: 0.15
Atoh8Unknown, score: 0.05
Tssk4Unknown, score: 0.08
Sh2d6Unknown, score: 0.02
CabyrUnknown, score: 0.09
Scara5Unknown, score: 0.17
4933413G19RikUnknown, score: 0.09
Eri2Unknown, score: 0.27
NipblUnknown, score: 0.02
AsunUnknown, score: 0.23
EnkurUnknown, score: 0.09
Osbpl7Unknown, score: 0.41
Naif1Unknown, score: 0.06
Lrrfip2Unknown, score: 0.24
Noxred1Unknown, score: 0.05
Arhgap26Unknown, score: 0.02
Rassf8Unknown, score: 0.09
Tchhl1Unknown, score: 0.02
Treml1Unknown, score: 0.24
RbksUnknown, score: 0.28
Riok1Unknown, score: 0.08
Wdr31Unknown, score: 0.12
Col24a1Unknown, score: 0.18
Pdss2Unknown, score: 0.05
Pex1Unknown, score: 0.06
Chd6Unknown, score: 0
Kctd6Unknown, score: 0.03
Fmnl2Unknown, score: 0.17
WrbUnknown, score: 0.17
Tmem80Unknown, score: 0.16
Usp19Unknown, score: 0.02
Bbs7Unknown, score: 0.11
SfpqUnknown, score: 0.03
Cyp2u1Unknown, score: 0.33
Pds5aUnknown, score: 0.07
KaznUnknown, score: 0.29
AfmidUnknown, score: 0.08
ClmpUnknown, score: 0.15
9130008F23RikUnknown, score: -0.01
Gdpd2Unknown, score: 0
Ifih1Unknown, score: 0.12
PogkUnknown, score: 0.03
TraddUnknown, score: 0.15
9130011E15RikUnknown, score: 0.12
Zfp949Unknown, score: 0.17
Zgrf1Unknown, score: 0.2
Rarres2Unknown, score: 0.24
Fuca1Unknown, score: -0.01
Tmem248Unknown, score: 0.35
Acy3Unknown, score: 0.12
0610010F05RikUnknown, score: 0.09
BroxUnknown, score: 0.19
Atp5hUnknown, score: 0.3
Galnt14Unknown, score: -0.01
Tmem25Unknown, score: 0.3
Pnpt1Unknown, score: 0.26
Arhgef3Unknown, score: 0.25
Ubiad1Unknown, score: 0.23
Syde1Unknown, score: 0.19
Lrrcc1Unknown, score: 0.25
Telo2Unknown, score: 0.07
CicUnknown, score: 0.14
Dhx34Unknown, score: 0.13
Rgs12Unknown, score: 0.08
Susd2Unknown, score: 0.19
Cul2Unknown, score: 0.36
R3hdm2Unknown, score: 0.11
Map3k13Unknown, score: 0.16
Gm29609Unknown, score: 0.12
Tmprss6Unknown, score: 0.17
DhdhUnknown, score: 0.15
Cpn2Unknown, score: 0.07
EtnpplUnknown, score: 0.1
C2cd2lUnknown, score: 0.08
Tysnd1Unknown, score: 0.02
VwceUnknown, score: 0.38
Ap2b1Unknown, score: 0.08
Plbd2Unknown, score: 0.26
Ugt2b1Unknown, score: 0.01
Shroom1Unknown, score: 0.08
1300017J02RikUnknown, score: 0.29
Tha1Unknown, score: 0.11
Slc16a14Unknown, score: 0.03
PdgfdUnknown, score: 0
Trnau1apUnknown, score: 0.09
Anxa9Unknown, score: 0.28
Ints12Unknown, score: 0.08
Plekhf2Unknown, score: 0.02
Slc25a18Unknown, score: 0.03
Nup93Unknown, score: 0.08
Ddi1Unknown, score: 0.15
PdiltUnknown, score: 0
Lancl2Unknown, score: 0.09
Shcbp1lUnknown, score: 0.1
1700003E16RikUnknown, score: 0.19
Syce2Unknown, score: 0.35
Dpep3Unknown, score: 0.11
Cfap52Unknown, score: 0.16
Gpr160Unknown, score: 0.01
Fbxo30Unknown, score: 0.01
Cfap45Unknown, score: 0.12
ApmapUnknown, score: 0.06
2310003H01RikUnknown, score: 0.15
2310002L09RikUnknown, score: 0.23
Epn3Unknown, score: 0.14
Tmem106bUnknown, score: 0.27
Paqr7Unknown, score: 0.22
Serpina9Unknown, score: 0.1
Haus5Unknown, score: 0.05
Antxr2Unknown, score: 0.22
Zcchc24Unknown, score: 0.39
Rpap3Unknown, score: 0.15
2310047M10RikUnknown, score: 0.13
Itfg1Unknown, score: 0.06
Apol6Unknown, score: 0.05
Cers5Unknown, score: -0.01
Suds3Unknown, score: 0.05
Ist1Unknown, score: 0.2
Rnf135Unknown, score: 0.02
Myh14Unknown, score: -0.01
Gatsl3Unknown, score: 0.06
Cdca4Unknown, score: 0.19
Nkiras2Unknown, score: 0.01
Wdr73Unknown, score: 0.08
Rbpms2Unknown, score: 0.05
Prmt3Unknown, score: 0.2
Ppp2r2aUnknown, score: 0.22
Tdrd12Unknown, score: 0.11
Sars2Unknown, score: 0.08
Esco2Unknown, score: 0.35
Rpusd4Unknown, score: 0.36
Cnn3Unknown, score: 0.04
Smg9Unknown, score: 0.13
Slc25a35Unknown, score: 0.04
Cyb5r1Unknown, score: 0.06
Fundc1Unknown, score: 0.03
Zfp654Unknown, score: 0
Slc35f2Unknown, score: 0.13
Slc39a4Unknown, score: 0.18
Cnpy3Unknown, score: 0.05
Tsc22d2Unknown, score: 0.2
Mccc1Unknown, score: 0.06
Sulf2Unknown, score: 0.13
2010001E11RikUnknown, score: 0
UrgcpUnknown, score: 0.2
Ddx42Unknown, score: 0.11
Tnfrsf13cUnknown, score: 0
1810055G02RikUnknown, score: 0.15
2010111I01RikUnknown, score: 0.08
Rap2cUnknown, score: 0.12
Cnot2Unknown, score: 0.13
Anks4bUnknown, score: 0.05
OgfrUnknown, score: 0
Gcnt3Unknown, score: 0.02
Sapcd2Unknown, score: 0.21
Mzt2Unknown, score: 0.1
PigxUnknown, score: 0.25
Osgepl1Unknown, score: 0.08
Ush1cUnknown, score: 0.18
Ugt2a3Unknown, score: 0.05
Tmem68Unknown, score: 0.04
Dscc1Unknown, score: 0.01
Ddhd2Unknown, score: 0.37
Adck1Unknown, score: 0.09
Dennd2dUnknown, score: 0.68
Ccdc71lUnknown, score: 0.05
Seh1lUnknown, score: 0.39
Pex13Unknown, score: 0.27
Chst14Unknown, score: 0.23
Wdfy3Unknown, score: 0.1
Zbtb46Unknown, score: 0.28
TdrpUnknown, score: 0.07
StradaUnknown, score: 0.1
Rfc5Unknown, score: 0.06
CenpnUnknown, score: 0.15
Pgm2Unknown, score: 0.39
Tmem163Unknown, score: 0.3
Dhx36Unknown, score: 0.2
Chchd4Unknown, score: 0.13
Shq1Unknown, score: 0.15
Fbxl2Unknown, score: 0.1
Zfp661Unknown, score: 0
Nsun4Unknown, score: 0.17
Scaf11Unknown, score: 0.15
Supt7lUnknown, score: -0.01
Skiv2l2Unknown, score: 0.28
Mms19Unknown, score: 0.08
Eml2Unknown, score: 0.07
Tbc1d5Unknown, score: 0.25
1600014C10RikUnknown, score: 0.14
Tram1Unknown, score: 0.19
2200002D01RikUnknown, score: 0.14
Ccpg1Unknown, score: 0.34
Sh2d4aUnknown, score: 0.51
Zfp777Unknown, score: 0.16
Brf1Unknown, score: 0.23
Tmem158Unknown, score: 0.14
Asb6Unknown, score: 0.09
PalldUnknown, score: 0.32
Wdr89Unknown, score: 0.22
Elp6Unknown, score: 0.01
Cdpf1Unknown, score: 0.19
2210016L21RikUnknown, score: 0.09
2210408I21RikUnknown, score: 0.12
PscaUnknown, score: 0.14
Ripk4Unknown, score: 0.02
Faim2Unknown, score: 0.31
BrapUnknown, score: 0.08
Pinx1Unknown, score: 0.11
Wdr44Unknown, score: 0.06
Sgol1Unknown, score: 0.19
Ttc30bUnknown, score: 0
Katnbl1Unknown, score: 0.15
Dnajc25Unknown, score: -0.01
Ccdc71Unknown, score: 0.07
PrcpUnknown, score: 0.16
Rrp1bUnknown, score: 0.62
Plcd3Unknown, score: 0.03
Ssbp3Unknown, score: 0.12
Tmem87bUnknown, score: 0.46
Tspyl4Unknown, score: 0.1
Acbd6Unknown, score: -0.01
Ier5lUnknown, score: 0.02
Dzip1lUnknown, score: 0.46
Tmem173Unknown, score: 0.31
Fgfbp3Unknown, score: 0.05
Wdr43Unknown, score: 0.1
Tmem55aUnknown, score: 0.01
Aldh1b1Unknown, score: 0.03
Hsdl1Unknown, score: -0.01
Utp14aUnknown, score: 0.22
Naalad2Unknown, score: 0.11
Pcbd2Unknown, score: 0.1
Bclaf1Unknown, score: 0.06
Cul4bUnknown, score: 0.05
Lypd1Unknown, score: 0.22
Pan3Unknown, score: 0.03
Usp13Unknown, score: 0.19
Zfp655Unknown, score: 0.6
Anks3Unknown, score: 0.04
Mex3aUnknown, score: 0.48
Tmem209Unknown, score: 0.05
2810006K23RikUnknown, score: 0.34
2700097O09RikUnknown, score: 0.06
Serp2Unknown, score: -0.01
Zfp444Unknown, score: 0.01
Adipor1Unknown, score: -0.01
HnrnpllUnknown, score: -0.01
Angptl1Unknown, score: 0.09
Fam98aUnknown, score: 0.12
Zfp74Unknown, score: 0.04
B3gat3Unknown, score: 0.1
Cdc42se2Unknown, score: 0.24
Tmx1Unknown, score: 0.29
Zkscan3Unknown, score: 0.16
Tmem161bUnknown, score: 0.15
Hdhd3Unknown, score: -0.01
Arhgef10lUnknown, score: 0.02
Rint1Unknown, score: 0.07
Neil1Unknown, score: 0
72775Unknown, score: 0
Dnajc22Unknown, score: 0.24
Rspo3Unknown, score: 0.38
Ndc1Unknown, score: 0.04
Zfp839Unknown, score: 0.14
Dhx30Unknown, score: 0.09
Crtac1Unknown, score: 0.03
Kctd17Unknown, score: 0.41
Mblac2Unknown, score: 0.27
Cxx1cUnknown, score: 0.53
Ccdc176Unknown, score: 0.12
Zdhhc4Unknown, score: 0.05
Xlr4bUnknown, score: 0.17
Asphd2Unknown, score: 0.07
Macrod2Unknown, score: 0.3
Ppp2r2bUnknown, score: 0.21
Ddx41Unknown, score: 0.23
Lrrc47Unknown, score: 0.24
TpppUnknown, score: 0.31
Zfp493Unknown, score: 0.02
Slc17a7Unknown, score: 0.08
TympUnknown, score: 0.2
Ccser2Unknown, score: 0.12
Fbxo47Unknown, score: 0.25
Tmem138Unknown, score: 0.01
Appl1Unknown, score: 0.03
Kremen2Unknown, score: 0.02
Emc7Unknown, score: 0.18
Ppp1r16aUnknown, score: 0.29
Tmem192Unknown, score: 0.04
Ppil6Unknown, score: 0.02
Rps6ka5Unknown, score: 0.13
Sgip1Unknown, score: 0.35
Slc25a42Unknown, score: 0.13
Slc22a23Unknown, score: 0.14
Prss57Unknown, score: 0.19
Fam101aUnknown, score: 0.06
Golim4Unknown, score: 0.08
Tmed5Unknown, score: 0.49
Prrc1Unknown, score: 0.29
Clec4a3Unknown, score: 0
Exo5Unknown, score: 0.14
Pcdh18Unknown, score: 0.03
Pear1Unknown, score: 0.07
3110043O21RikUnknown, score: 0.06
3110052M02RikUnknown, score: 0.08
BmperUnknown, score: 0.07
MrgbpUnknown, score: 0.09
Gpbp1Unknown, score: 0.47
Ddit4lUnknown, score: 0.26
Ccdc103Unknown, score: 0.03
Calr3Unknown, score: 0.06
Ube2d2bUnknown, score: 0.07
Pradc1Unknown, score: 0.21
Ccdc30Unknown, score: 0.03
Slc25a31Unknown, score: 0.12
Arhgef6Unknown, score: 0.29
1700056E22RikUnknown, score: 0.21
Dcbld2Unknown, score: 0.33
TeppUnknown, score: 0.05
Nme8Unknown, score: 0.15
1700052N19RikUnknown, score: 0.11
CcsapUnknown, score: 0.01
Prox2Unknown, score: 0.1
Tex35Unknown, score: 0.53
Hspa12aUnknown, score: 0.07
1700066B19RikUnknown, score: 0.28
Zfp763Unknown, score: 0.1
Izumo1Unknown, score: 0.2
1700055N04RikUnknown, score: 0.09
1700066M21RikUnknown, score: 0.02
Mipol1Unknown, score: 0.18
Vgll3Unknown, score: 0.09
1700001O22RikUnknown, score: 0.35
Capn9Unknown, score: 0.04
Spns1Unknown, score: 0.15
Cabp4Unknown, score: 0.35
2410004P03RikUnknown, score: 0.38
Rec114Unknown, score: 0.13
Wdr75Unknown, score: 0.05
Psma8Unknown, score: 0.04
Trmt11Unknown, score: 0.1
Atg16l2Unknown, score: 0.06
2410089E03RikUnknown, score: 0.08
DmknUnknown, score: 0.15
Rbm20Unknown, score: 0.34
Sh3bgrl3Unknown, score: 0.19
PsdUnknown, score: 0.29
1110008P14RikUnknown, score: 0.03
Cby1Unknown, score: 0.03
WhrnUnknown, score: 0
Kif2cUnknown, score: 0.4
Mfsd12Unknown, score: 0.11
Tmem198bUnknown, score: 0.56
Dcaf4Unknown, score: 0.29
Eif3kUnknown, score: 0.08
Fam98cUnknown, score: 0.08
Slc35b2Unknown, score: -0.01
Fam110aUnknown, score: 0.34
4930415F15RikUnknown, score: 0.16
4930415O20RikUnknown, score: 0.06
Tmem202Unknown, score: 0
Psmb11Unknown, score: 0.3
Ift57Unknown, score: 0.27
Fam151bUnknown, score: 0.06
Otud4Unknown, score: 0.03
Atl1Unknown, score: 0.51
Psd2Unknown, score: 0.07
74004Unknown, score: 0.01
Dnm1lUnknown, score: 0.17
Btbd11Unknown, score: 0.12
ArsgUnknown, score: 0.55
Slc25a27Unknown, score: -0.01
Rap2bUnknown, score: 0.3
Fcho1Unknown, score: 0.04
Phf19Unknown, score: 0.15
Als2Unknown, score: 0.1
Traf3ip1Unknown, score: 0.24
Msl1Unknown, score: 0.18
Rin2Unknown, score: 0.02
DdiasUnknown, score: 0.12
Plce1Unknown, score: 0.15
Serpina3aUnknown, score: 0.1
Syce1Unknown, score: 0.39
Nmnat3Unknown, score: 0.09
Slc7a13Unknown, score: 0.18
Them7Unknown, score: 0.09
Hvcn1Unknown, score: 0.05
Pop7Unknown, score: 0.15
0610037L13RikUnknown, score: 0.08
Arpp21Unknown, score: 0.12
Usp16Unknown, score: 0.24
Actr3Unknown, score: 0.13
Krt80Unknown, score: 0.15
Sec14l1Unknown, score: 0.15
Lonp1Unknown, score: 0.03
F13a1Unknown, score: 0.54
CluhUnknown, score: 0.09
Stra6lUnknown, score: 0
Errfi1Unknown, score: 0.02
Acot12Unknown, score: 0.04
Acbd5Unknown, score: 0.21
Nfx1Unknown, score: 0.24
Fbxl22Unknown, score: 0.01
Tmem38aUnknown, score: 0.32
Stk40Unknown, score: 0.12
Phactr3Unknown, score: 0.13
Exoc3l4Unknown, score: 0.1
P2ry13Unknown, score: 0.15
Arpc5lUnknown, score: 0.01
Rnd3Unknown, score: 0.08
Elp3Unknown, score: 0.29
Ttc27Unknown, score: 0.2
Dtx2Unknown, score: 0.22
VitUnknown, score: 0.12
2810403A07RikUnknown, score: 0.22
Cep97Unknown, score: 0.3
Eif4enif1Unknown, score: 0.14
Sipa1l3Unknown, score: 0.07
1700017B05RikUnknown, score: 0.11
Paqr8Unknown, score: 0.15
Tubgcp2Unknown, score: 0.62
Atg7Unknown, score: 0.02
GaleUnknown, score: 0.22
Ankrd9Unknown, score: 0.06
Armc1Unknown, score: 0.02
Klrg2Unknown, score: 0.04
Gpn1Unknown, score: 0.08
Tspan17Unknown, score: 0.15
AvenUnknown, score: 0.07
Spatc1Unknown, score: 0
Kcmf1Unknown, score: 0.04
Prss46Unknown, score: 0.11
1700092M07RikUnknown, score: 0.21
Rnf145Unknown, score: 0.35
Isca2Unknown, score: 0.03
HopxUnknown, score: 0.03
Mettl23Unknown, score: 0.13
Cxxc1Unknown, score: 0.06
CltbUnknown, score: 0.24
Xrcc3Unknown, score: 0.41
Crtc2Unknown, score: 0.27
Tldc1Unknown, score: 0.03
Fam160a2Unknown, score: 0.06
Ddx23Unknown, score: 0.18
Zfp84Unknown, score: 0
LrgukUnknown, score: -0.01
4931428F04RikUnknown, score: 0.14
4931414P19RikUnknown, score: 0.18
Gcc1Unknown, score: 0.09
Ap5m1Unknown, score: 0.24
Dpp8Unknown, score: -0.01
Specc1lUnknown, score: 0.26
Ttc25Unknown, score: 0.41
Ttll11Unknown, score: 0.11
Gle1Unknown, score: -0.01
Tc2nUnknown, score: 0
Tmc5Unknown, score: 0.03
4930452B06RikUnknown, score: 0.25
P4htmUnknown, score: 0.02
Pgs1Unknown, score: 0.05
Cfap53Unknown, score: 0.46
Nsun6Unknown, score: 0.16
Zswim5Unknown, score: 0
4933427G17RikUnknown, score: 0.17
Pus10Unknown, score: 0.06
Snx29Unknown, score: 0.01
Snx11Unknown, score: 0.06
Osbpl10Unknown, score: 0.16
Lrrc15Unknown, score: -0.01
Tnks2Unknown, score: 0.18
Gorasp1Unknown, score: 0.64
Lrrc17Unknown, score: 0.36
Neto2Unknown, score: 0.14
Cyp2j9Unknown, score: 0.05
Ppp4r4Unknown, score: 0.07
Mgme1Unknown, score: -0.01
Gsdmc4Unknown, score: 0.12
Mau2Unknown, score: -0.01
Pck2Unknown, score: 0.15
Elovl7Unknown, score: 0.16
Glb1lUnknown, score: 0
Cds1Unknown, score: 0.01
Mrpl47Unknown, score: 0.03
Cd200r3Unknown, score: 0.04
OcstampUnknown, score: 0.23
Scpep1Unknown, score: 0.05
Tmem81Unknown, score: 0.18
Spsb1Unknown, score: 0.11
PomkUnknown, score: 0.05
Lrrc48Unknown, score: 0.07
Zfp943Unknown, score: -0.01
74684Unknown, score: 0.12
Tbc1d30Unknown, score: 0.19
Spata17Unknown, score: 0.03
Snx16Unknown, score: 0.03
Trim14Unknown, score: 0.27
Pcf11Unknown, score: 0.13
Slamf8Unknown, score: 0.13
5830415F09RikUnknown, score: 0.03
Atp13a2Unknown, score: 0.18
Lmbr1lUnknown, score: 0.31
Sepn1Unknown, score: 0.18
Rrp7aUnknown, score: 0.01
Glt8d2Unknown, score: 0.04
Naa15Unknown, score: 0.35
Mss51Unknown, score: 0.1
Rab11fip2Unknown, score: 0.11
Zbtb49Unknown, score: 0.18
Lysmd4Unknown, score: 0.11
Nxnl2Unknown, score: 0.25
75136Unknown, score: 0.22
Rprd2Unknown, score: 0.26
MeiobUnknown, score: 0.04
Spaca6Unknown, score: 0.19
Sv2cUnknown, score: -0.01
Prr3Unknown, score: 0
Rnf121Unknown, score: 0.02
Ppp1r1cUnknown, score: 0.19
Bcdin3dUnknown, score: 0.08
Zbtb3Unknown, score: -0.01
4930550C14RikUnknown, score: 0.29
ParpbpUnknown, score: 0.14
Etnk1Unknown, score: 0.25
Ccdc83Unknown, score: 0.44
Mphosph8Unknown, score: 0.05
Slamf7Unknown, score: 0.17
Sirt4Unknown, score: 0.1
Spp2Unknown, score: 0.08
Mrpl32Unknown, score: 0.15
Ndufs7Unknown, score: 0.26
Kmt2bUnknown, score: 0.04
Arhgap12Unknown, score: 0.27
Nop14Unknown, score: 0.08
Mettl5Unknown, score: 0
Zfp820Unknown, score: 0.25
Fam183bUnknown, score: 0.13
CklfUnknown, score: 0.04
Dynlrb2Unknown, score: 0.06
OplahUnknown, score: 0.02
Fabp12Unknown, score: 0.06
Gpx6Unknown, score: 0.3
Tex29Unknown, score: 0.13
Nme5Unknown, score: 0.3
Fam71e1Unknown, score: 0.06
FpgtUnknown, score: 0.08
Akap13Unknown, score: 0.86
Ep400Unknown, score: 0
Rsph9Unknown, score: 0.32
Ccdc101Unknown, score: 0.11
75570Unknown, score: 0.22
Acyp2Unknown, score: 0.34
Dusp9Unknown, score: 0.05
Malsu1Unknown, score: 0.1
Tm4sf5Unknown, score: 0.1
Med25Unknown, score: 0.13
Smim15Unknown, score: 0.3
Rps25Unknown, score: 0.09
Kxd1Unknown, score: 0.03
Tex30Unknown, score: 0.23
Metap1Unknown, score: 0.03
Mageh1Unknown, score: 0.1
1700029I15RikUnknown, score: 0.01
Lin37Unknown, score: 0.06
Rasl10aUnknown, score: 0
Higd1bUnknown, score: 0.57
Anks6Unknown, score: 0.24
Nr2c2apUnknown, score: 0.14
Rilpl1Unknown, score: 0.12
C2cd4bUnknown, score: 0.26
Fam35aUnknown, score: 0.02
Eif4bUnknown, score: 0.12
Rbm12Unknown, score: 0.04
Amotl1Unknown, score: 0.16
Phf14Unknown, score: 0.09
Fam227aUnknown, score: 0.16
IdnkUnknown, score: 0.02
IqcdUnknown, score: 0.03
MffUnknown, score: 0.53
Pank1Unknown, score: 0.38
Mpp7Unknown, score: 0.13
Egfem1Unknown, score: 0.08
SvipUnknown, score: 0.56
Sesn3Unknown, score: 0
Slc10a6Unknown, score: 0.27
Ipo4Unknown, score: 0.37
Dcaf17Unknown, score: -0.01
Pnpla5Unknown, score: 0.05
Them4Unknown, score: -0.01
Lca5Unknown, score: 0.17
Klhl24Unknown, score: 0.03
Cdyl2Unknown, score: 0.22
4930447C04RikUnknown, score: 0.29
NlnUnknown, score: 0.08
Tasp1Unknown, score: 0.18
Senp2Unknown, score: 0.09
Hormad2Unknown, score: 0.14
Rnf139Unknown, score: 0.29
IspdUnknown, score: 0.03
Clec4gUnknown, score: 0.04
Arl5bUnknown, score: 0
AdalUnknown, score: 0.03
4930578C19RikUnknown, score: 0.15
Vmp1Unknown, score: 0.03
Exoc6bUnknown, score: 0.2
4930579G24RikUnknown, score: 0.08
Zdhhc20Unknown, score: 0.07
Rab30Unknown, score: 0.19
Zmym2Unknown, score: 0.05
Cant1Unknown, score: 0.18
Ncapg2Unknown, score: 0.2
GancUnknown, score: 0.13
Rnf183Unknown, score: 0.32
Pcgf5Unknown, score: 0.03
Gbp8Unknown, score: 0.18
TtpalUnknown, score: 0.27
Dock8Unknown, score: 0.03
Gpsm2Unknown, score: 0.16
FaxcUnknown, score: 0.14
Ccdc138Unknown, score: 0.35
Fam131bUnknown, score: 0.13
Rsg1Unknown, score: 0.02
Abca6Unknown, score: 0.5
Adhfe1Unknown, score: 0.19
Abhd12Unknown, score: 0
Med13lUnknown, score: 0.15
Jakmip2Unknown, score: 0.2
Arxes1Unknown, score: 0.4
76224Unknown, score: 0.27
Dnttip1Unknown, score: 0.21
GrhprUnknown, score: 0.04
Ercc6l2Unknown, score: 0.06
Ttc8Unknown, score: 0.39
0610040J01RikUnknown, score: 0.24
Gstk1Unknown, score: 0.12
Tsen54Unknown, score: 0.19
Ndfip2Unknown, score: 0.17
Mfap4Unknown, score: 0.73
Atp11bUnknown, score: 0.17
PcnpUnknown, score: 0.25
OsbpUnknown, score: 0.07
Tbx18Unknown, score: 0.14
Zfp773Unknown, score: 0.07
Slc24a2Unknown, score: 0.12
Cep112Unknown, score: 0.12
Abcc3Unknown, score: 0.19
Ift43Unknown, score: 0.23
Znrd1asUnknown, score: 0.2
Gid8Unknown, score: 0.57
Rftn1Unknown, score: 0.08
Prss23Unknown, score: 0.03
Fbxo31Unknown, score: 0.04
Casc5Unknown, score: 0.24
Pcolce2Unknown, score: 0.8
Haus8Unknown, score: 0.07
Smndc1Unknown, score: 0.01
Lmf1Unknown, score: 0.25
Ly6kUnknown, score: 0.17
Ppp1r3gUnknown, score: 0.33
Abhd14bUnknown, score: 0.19
Clasp2Unknown, score: 0.52
Ip6k2Unknown, score: 0.02
Trappc9Unknown, score: 0.04
Lsm8Unknown, score: 0.07
Fam204aUnknown, score: 0.05
Atg2bUnknown, score: 0.06
Snx7Unknown, score: 0.1
Mib2Unknown, score: 0.48
Dnajc18Unknown, score: 0.1
Hectd3Unknown, score: 0.28
Lrrc27Unknown, score: 0.13
ImmtUnknown, score: 0
Stambpl1Unknown, score: 0.26
1700112E06RikUnknown, score: 0.29
1700113H08RikUnknown, score: 0.13
Cfap70Unknown, score: 0.06
Clip3Unknown, score: 0.23
Snx27Unknown, score: 0.12
Dapl1Unknown, score: 0.23
TrdnUnknown, score: 0.22
Wdyhv1Unknown, score: -0.01
Slc10a7Unknown, score: 0.28
Mettl4Unknown, score: 0.18
Klhdc10Unknown, score: 0.2
2410131K14RikUnknown, score: 0.03
Tbc1d9bUnknown, score: 0.3
Usp42Unknown, score: 0.15
Kdm4cUnknown, score: 0.26
Rpl18aUnknown, score: 0.19
Calcoco2Unknown, score: 0.02
Fam49aUnknown, score: 0.11
Mtfr1lUnknown, score: 0.36
Dok5Unknown, score: 0.14
DtlUnknown, score: 0.22
Gper1Unknown, score: 0.13
Morn1Unknown, score: 0.01
Ccdc116Unknown, score: 0.03
Rab36Unknown, score: 0.21
Cyfip2Unknown, score: 0.4
Memo1Unknown, score: 0.15
Cers2Unknown, score: 0.02
Mettl15Unknown, score: 0.03
Bicd2Unknown, score: 0.01
Ssbp4Unknown, score: 0.2
Jade2Unknown, score: 0.26
Lrg1Unknown, score: 0.44
Mnd1Unknown, score: 0.14
Flywch2Unknown, score: 0.03
Arrdc5Unknown, score: 0.02
TsaccUnknown, score: 0.12
HnrnpmUnknown, score: 0.12
Rbm17Unknown, score: 0.07
Ndufaf6Unknown, score: -0.01
Nt5c2Unknown, score: 0.35
St5Unknown, score: 0
Chmp5Unknown, score: -0.01
Chst1Unknown, score: 0.19
2810007J24RikUnknown, score: 0.72
UrahUnknown, score: 0.2
Arxes2Unknown, score: 0.66
Scfd1Unknown, score: 0.21
Hdhd2Unknown, score: 0.35
Mpped2Unknown, score: 0.04
Col25a1Unknown, score: 0.03
2510039O18RikUnknown, score: 0.16
Kdm8Unknown, score: 0.07
1700109H08RikUnknown, score: 0.01
Arfgap2Unknown, score: 0.19
Arid2Unknown, score: 0.34
Cep83Unknown, score: 0.13
Sun1Unknown, score: 0.08
Ston1Unknown, score: 0.11
Ccdc183Unknown, score: 0.08
Tmem181aUnknown, score: 0.3
Il33Unknown, score: 0.19
Hnrnpa0Unknown, score: 0.17
Tmem200aUnknown, score: 0.21
Yif1bUnknown, score: 0.05
Zfp142Unknown, score: 0.03
Wdr82Unknown, score: 0.2
Esrp2Unknown, score: 0.2
Tmem116Unknown, score: 0.12
Kidins220Unknown, score: 0.08
Anks1bUnknown, score: 0.01
JrklUnknown, score: 0.25
Shisa4Unknown, score: 0.05
AglUnknown, score: 0.48
Limch1Unknown, score: 0.1
Myh10Unknown, score: 0.07
NotumUnknown, score: 0.3
Chst15Unknown, score: 0.34
Usp45Unknown, score: -0.01
Nup210lUnknown, score: 0.11
Adgrf1Unknown, score: 0.3
H2afvUnknown, score: 0
Ccdc151Unknown, score: 0.19
Prss36Unknown, score: 0.11
Prelid2Unknown, score: 0.18
Apex2Unknown, score: 0.14
Prdm8Unknown, score: 0.28
Snapc3Unknown, score: 0.15
Trat1Unknown, score: -0.01
Ehmt1Unknown, score: 0.18
MmabUnknown, score: 0
Adamtsl1Unknown, score: 0.34
BoraUnknown, score: 0.06
Epm2aip1Unknown, score: 0.39
Adamtsl2Unknown, score: 0.03
Sla2Unknown, score: 0.01
Lrrc42Unknown, score: 0.45
Krba1Unknown, score: 0.15
MlanaUnknown, score: 0.25
Msl2Unknown, score: 0.11
Thyn1Unknown, score: 0.1
Ypel2Unknown, score: 0.13
6030458C11RikUnknown, score: 0.5
Ube2sUnknown, score: 0.36
Fam53bUnknown, score: 0.09
Rpgrip1Unknown, score: 0.08
Cyp20a1Unknown, score: 0.23
Rdh12Unknown, score: 0.46
Tmem50bUnknown, score: 0.2
CutalUnknown, score: 0.13
Prr15Unknown, score: 0.42
Ccdc150Unknown, score: 0.3
Cpt1cUnknown, score: 0.05
Msantd4Unknown, score: 0.06
Lpar4Unknown, score: 0.04
NinlUnknown, score: 0.05
Trappc6bUnknown, score: 0.12
9230112D13RikUnknown, score: 0.08
Dnajc21Unknown, score: 0.29
Acbd7Unknown, score: -0.01
Phf23Unknown, score: 0.05
Armcx1Unknown, score: 0.17
Adgrf4Unknown, score: 0.33
Zfp712Unknown, score: 0.11
Lrrc9Unknown, score: 0.04
Klhdc8bUnknown, score: 0.14
Map7d2Unknown, score: 0.08
Creb3l4Unknown, score: 0.36
RbsnUnknown, score: 0.17
Rps27aUnknown, score: 0.15
Hist3h2baUnknown, score: 0.1
Naa38Unknown, score: 0.05
Gpr108Unknown, score: 0.11
Ankrd23Unknown, score: 0.11
Fam219bUnknown, score: 0.21
Ttyh3Unknown, score: -0.01
Snrnp25Unknown, score: 0.07
Sapcd1Unknown, score: 0.2
MvpUnknown, score: 0.18
Ddx52Unknown, score: 0.24
Ntf5Unknown, score: 0.3
Fam131aUnknown, score: 0.13
3110062M04RikUnknown, score: 0.19
Pgpep1lUnknown, score: 0
HelzUnknown, score: 0.06
Skap1Unknown, score: 0.3
Micu3Unknown, score: 0.28
B230219D22RikUnknown, score: 0.52
Adgra2Unknown, score: 0.17
Utp23Unknown, score: 0.17
UvragUnknown, score: 0.11
1700061G19RikUnknown, score: 0.15
Lsm6Unknown, score: 0.1
Bola3Unknown, score: 0.24
Eif3j1Unknown, score: 0.4
Brd8Unknown, score: 0.13
Ncapd3Unknown, score: 0.12
Plekhj1Unknown, score: 0.04
Csgalnact2Unknown, score: 0.31
RictorUnknown, score: 0.21
Mctp1Unknown, score: 0.04
Zc3hav1Unknown, score: 0.07
Clip4Unknown, score: 0.29
Armc9Unknown, score: 0.34
Ak7Unknown, score: 0.02
Ttc30a1Unknown, score: 0.24
4930562C15RikUnknown, score: 0.04
P2ry10Unknown, score: 0.34
Gins3Unknown, score: 0.12
Sfi1Unknown, score: 0.25
Trmt44Unknown, score: 0.01
Scyl1Unknown, score: 0.43
Crispld2Unknown, score: 0.09
AacsUnknown, score: 0.06
1500015O10RikUnknown, score: 0.18
Igsf3Unknown, score: 0.05
Fndc8Unknown, score: 0.15
Chsy3Unknown, score: 0.12
PigtUnknown, score: 0.22
Agbl4Unknown, score: 0.17
Saal1Unknown, score: 0.11
Mrps34Unknown, score: 0
Tnfrsf22Unknown, score: 0.13
Zfp319Unknown, score: 0.1
LratUnknown, score: 0.08
Krit1Unknown, score: 0.27
Bhlhe41Unknown, score: 0.01
LiasUnknown, score: 0.15
CptpUnknown, score: 0.03
Wbscr27Unknown, score: 0.07
Sh3bp5lUnknown, score: 0.14
Cttnbp2nlUnknown, score: 0.06
Abtb1Unknown, score: 0.03
Smim12Unknown, score: 0.06
Rilpl2Unknown, score: 0.26
Pofut2Unknown, score: 0.11
Kcnip4Unknown, score: 0.45
Med8Unknown, score: 0.14
WwoxUnknown, score: 0.01
Pbx4Unknown, score: 0.09
MynnUnknown, score: 0.13
Car15Unknown, score: 0.31
Vps16Unknown, score: 0.08
Cwc22Unknown, score: 0.05
BC004004Unknown, score: 0.16
Lrfn1Unknown, score: 0.01
N4bp1Unknown, score: 0.02
CblcUnknown, score: 0.04
Hist1h1aUnknown, score: 0.04
NfkbizUnknown, score: 0.24
Ifitm2Unknown, score: 0.02
Slc16a3Unknown, score: 0.06
Kank3Unknown, score: 0.15
Hcar2Unknown, score: 0.07
Senp3Unknown, score: 0.56
Cxcr6Unknown, score: 0.21
Zfp202Unknown, score: 0.11
PolhUnknown, score: 0.47
Kcnip2Unknown, score: -0.01
Uck2Unknown, score: 0.12
MrgprhUnknown, score: 0.28
Rad54l2Unknown, score: 0.14
Tbl1xr1Unknown, score: 0.17
Gpr63Unknown, score: 0.07
Vmn1r65Unknown, score: 0.13
Vmn1r58Unknown, score: 0.27
Dnajb1Unknown, score: 0.42
Sgpp1Unknown, score: 0.04
Kat5Unknown, score: 0.07
Zbtb22Unknown, score: 0.16
Egfl8Unknown, score: 0.03
C1qtnf3Unknown, score: 0.1
Tfcp2l1Unknown, score: 0.07
Cacng7Unknown, score: 0.22
Cyp4x1Unknown, score: 0.16
Tmem108Unknown, score: 0.13
Zfpl1Unknown, score: 0.1
SigleceUnknown, score: 0.23
Tfap4Unknown, score: 0
Akap12Unknown, score: 0.05
Lamtor2Unknown, score: 0.25
Cstf2tUnknown, score: -0.01
Plekha2Unknown, score: 0.19
NgrnUnknown, score: 0.22
Rbm5Unknown, score: 0.03
Lin28aUnknown, score: 0.05
Tex14Unknown, score: 0.42
Gtf2a1Unknown, score: 0.15
Wdr6Unknown, score: 0
Sytl2Unknown, score: 0.26
Sytl3Unknown, score: 0
SrrtUnknown, score: 0.13
Akr1c6Unknown, score: 0.17
Slc12a9Unknown, score: 0.02
Dpp7Unknown, score: 0.12
Tas1r3Unknown, score: 0.02
Smarcd2Unknown, score: 0.15
Smarcd1Unknown, score: 0.03
Tnk1Unknown, score: 0.45
Nedd4lUnknown, score: -0.01
Tmem2Unknown, score: 0.05
Trps1Unknown, score: 0.17
Dnaja3Unknown, score: 0.11
PhipUnknown, score: 0.07
Nrg4Unknown, score: 0.1
Btbd1Unknown, score: 0.16
Jam3Unknown, score: 0.19
Enpp5Unknown, score: 0.02
Tssk6Unknown, score: 0.21
McamUnknown, score: 0.1
Kremen1Unknown, score: 0.08
Kcnn1Unknown, score: 0.07
Usp8Unknown, score: 0.31
Sucnr1Unknown, score: 0.04
Ptov1Unknown, score: 0.13
Setdb1Unknown, score: 0.03
Fam126aUnknown, score: 0.13
Pla1aUnknown, score: 0.21
Sec16bUnknown, score: 0.04
Tac4Unknown, score: 0.08
Cd163Unknown, score: 0.17
Clec2iUnknown, score: 0.21
Csnk1a1Unknown, score: 0.02
GlrxUnknown, score: 0.23
GpnmbUnknown, score: 0.11
Ear6Unknown, score: 0.12
Pcdhga12Unknown, score: 0
Rnase2aUnknown, score: 0.07
Acox2Unknown, score: 0.43
Pard6gUnknown, score: 0.25
Gabarapl2Unknown, score: 0.01
Gprc5dUnknown, score: 0.03
Echs1Unknown, score: 0.6
Immp2lUnknown, score: 0.01
Smarca5Unknown, score: -0.01
Nipa2Unknown, score: -0.01
Rnf111Unknown, score: 0.16
Dach2Unknown, score: 0.25
Dqx1Unknown, score: 0.13
Vangl2Unknown, score: -0.01
Uchl4Unknown, score: 0.05
PnckUnknown, score: 0.1
Pcdhb3Unknown, score: 0.01
Pcdhb11Unknown, score: 0.06
Pcdhb12Unknown, score: 0.01
Pcdhb21Unknown, score: 0.06
Klra8Unknown, score: 0.05
Klra1Unknown, score: 0.14
Klra4Unknown, score: 0.17
ClmnUnknown, score: 0.15
Bcl2l13Unknown, score: 0.37
P2rx5Unknown, score: 0.29
Mrpl1Unknown, score: 0.02
Mrpl16Unknown, score: 0.08
Mrpl27Unknown, score: 0.15
Mrpl43Unknown, score: -0.01
Trim7Unknown, score: 0.13
Trim9Unknown, score: 0.13
Trim11Unknown, score: 0.18
Trim16Unknown, score: 0.01
Trim33Unknown, score: 0.06
Med15Unknown, score: 0.23
Dock2Unknown, score: 0.01
Mcoln1Unknown, score: 0.01
NansUnknown, score: 0.1
Pdxdc1Unknown, score: 0.32
Zfp423Unknown, score: 0.16
Ophn1Unknown, score: 0.23
C1galt1Unknown, score: 0.14
Pag1Unknown, score: 0.08
Col4a6Unknown, score: 0.29
Lrp1bUnknown, score: 0.31
Cnnm3Unknown, score: 0.09
Pi15Unknown, score: 0.04
Cpsf1Unknown, score: 0.05
Tinagl1Unknown, score: 0
Fkbp6Unknown, score: 0.01
Arid4bUnknown, score: 0.01
Hecw1Unknown, score: 0.03
Maged1Unknown, score: 0.45
Sfxn3Unknown, score: 0.25
Sfxn4Unknown, score: 0.08
Cadm3Unknown, score: 0.06
Loxl2Unknown, score: 0.02
Prg4Unknown, score: 0.1
Susd4Unknown, score: 0.38
Slc9b2Unknown, score: 0.09
Nmd3Unknown, score: 0.02
A430005L14RikUnknown, score: 0.4
Hmgb2Unknown, score: 0.5
HadhaUnknown, score: 0.11
Naa11Unknown, score: 0.17
Mtmr14Unknown, score: 0.07
Cog8Unknown, score: 0.13
4833439L19RikUnknown, score: 0.26
B3galnt2Unknown, score: 0.12
Hist1h3gUnknown, score: 0.12
Nol12Unknown, score: 0.52
Tmem132aUnknown, score: 0.02
Dcaf8Unknown, score: 0.25
Lrrc59Unknown, score: 0.03
KmoUnknown, score: 0.36
Stk17bUnknown, score: 0.05
Slamf9Unknown, score: 0.06
Chst10Unknown, score: 0.07
Sh3bp4Unknown, score: 0
Zfp451Unknown, score: 0.25
AI597479Unknown, score: 0.24
Nucks1Unknown, score: 0.11
Cnih4Unknown, score: 0.15
Phlpp1Unknown, score: 0.09
Pid1Unknown, score: 0.13
MaelUnknown, score: 0.24
Mfsd6Unknown, score: 0.21
RabifUnknown, score: 0.03
Obsl1Unknown, score: 0.08
HnrnpfUnknown, score: 0.16
Ubac1Unknown, score: 0.15
Cdc123Unknown, score: 0.32
Eps8l2Unknown, score: 0.21
Myl9Unknown, score: 0.18
Fam102aUnknown, score: 0.2
Nat10Unknown, score: 0.18
Osbpl6Unknown, score: 0.04
Cep152Unknown, score: 0
Anapc2Unknown, score: 0.17
Ssx2ipUnknown, score: 0.06
Tm9sf4Unknown, score: 0.03
Hrh3Unknown, score: -0.01
Zscan29Unknown, score: 0.01
Arfgef2Unknown, score: 0.03
Cul4aUnknown, score: 0.09
Sall4Unknown, score: 0.23
Dnttip2Unknown, score: 0.06
Olfml3Unknown, score: 0.18
DpydUnknown, score: 0.05
Adgrl2Unknown, score: 0
TchhUnknown, score: 0.08
Ankrd50Unknown, score: 0.1
Cept1Unknown, score: 0.22
Taf13Unknown, score: 0.16
Arfip1Unknown, score: 0.29
Prmt6Unknown, score: 0.04
Kdm1aUnknown, score: 0.1
Ldlrap1Unknown, score: 0.03
Mdn1Unknown, score: 0.25
Gpr153Unknown, score: 0.02
AI481877Unknown, score: 0.25
Phactr4Unknown, score: 0.13
AknaUnknown, score: 0.08
Tmem64Unknown, score: 0.07
Osbpl9Unknown, score: 0.13
Ppp1r8Unknown, score: 0.16
Bsdc1Unknown, score: 0.16
Slc44a1Unknown, score: 0.06
Lao1Unknown, score: 0.02
Zfand2aUnknown, score: 0.19
Nsun5Unknown, score: 0.27
Upk3bUnknown, score: 0.08
Gbp6Unknown, score: 0.02
Pds5bUnknown, score: 0.12
Papd4Unknown, score: 0.11
Ugt2b34Unknown, score: 0
Mapre3Unknown, score: 0.12
Dcun1d4Unknown, score: 0.11
Ube3cUnknown, score: 0.02
Tbc1d14Unknown, score: 0.25
Emilin1Unknown, score: 0.1
Rab28Unknown, score: 0.05
Snx21Unknown, score: 0.09
Rpusd3Unknown, score: 0.2
B630005N14RikUnknown, score: 0.16
Parp11Unknown, score: 0.21
Hepacam2Unknown, score: 0.16
Tada3Unknown, score: 0.05
Tra2aUnknown, score: 0.3
Wdr91Unknown, score: 0.17
Prrt4Unknown, score: 0.04
Adamts9Unknown, score: 0.3
Plekha1Unknown, score: 0.07
Slco2b1Unknown, score: 0.22
Hsd3b7Unknown, score: 0.19
Klk9Unknown, score: 0.01
Vrk3Unknown, score: 0.08
AI467606Unknown, score: 0.01
E430018J23RikUnknown, score: 0.21
Grwd1Unknown, score: 0.09
Spty2d1Unknown, score: 0.18
Trim68Unknown, score: 0
Numa1Unknown, score: 0.17
Psip1Unknown, score: 0.19
Spred3Unknown, score: 0.16
C230052I12RikUnknown, score: 0.14
Unc45aUnknown, score: 0.02
Igflr1Unknown, score: 0.04
D8Ertd738eUnknown, score: 0.14
Champ1Unknown, score: 0.24
Ces2aUnknown, score: 0.47
Exoc8Unknown, score: 0.15
Gadd45gip1Unknown, score: 0.08
Plekhg4Unknown, score: 0.22
DohhUnknown, score: 0.17
Agpat6Unknown, score: 0.21
Cpne7Unknown, score: -0.01
Ankrd10Unknown, score: 0.22
Cog4Unknown, score: 0.5
MyzapUnknown, score: 0.11
Clk3Unknown, score: 0.04
HinfpUnknown, score: 0
XylbUnknown, score: 0.12
Imp3Unknown, score: 0.1
Cmtm7Unknown, score: 0.06
Cd276Unknown, score: 0.02
Lrrc49Unknown, score: 0.47
Bbs4Unknown, score: 0.25
CenpiUnknown, score: 0.07
Slc6a15Unknown, score: 0.04
Pan2Unknown, score: 0.11
Rdh9Unknown, score: 0.4
Upb1Unknown, score: 0.11
ApofUnknown, score: 0.09
Fig4Unknown, score: 0.12
Traf3ip2Unknown, score: 0.68
BC030307Unknown, score: 0.01
Zc3h10Unknown, score: 0.22
Zfr2Unknown, score: 0.01
NclnUnknown, score: 0.15
Nt5dc3Unknown, score: 0.11
Mgat4bUnknown, score: 0.12
E130012A19RikUnknown, score: 0.04
Psme4Unknown, score: 0.09
Fbxw11Unknown, score: 0.02
Sec14l4Unknown, score: 0.05
Slc35e4Unknown, score: 0.01
Tubg1Unknown, score: 0.66
Pex12Unknown, score: 0.21
Mien1Unknown, score: 0.09
Tmem98Unknown, score: 0.26
Tmem17Unknown, score: 0.22
Cuedc1Unknown, score: 0.43
Inca1Unknown, score: 0.05
Hoxb2Unknown, score: 0.11
Dnm3Unknown, score: 0.05
Plin1Unknown, score: 0.3
GckUnknown, score: 0.03
Rtn1Unknown, score: 0.12
Cdh22Unknown, score: 0.04
SynpoUnknown, score: 0.14
Wdr7Unknown, score: 0.37
Cyp27a1Unknown, score: 0.5
Ndufb11Unknown, score: 0.85
Etv5Unknown, score: 0.1
GldcUnknown, score: 0.15
Sbk1Unknown, score: 0.41
BlmhUnknown, score: 0.25
Cabin1Unknown, score: 0.32
Tex15Unknown, score: 0.18
Csnk1dUnknown, score: 0
Gas8Unknown, score: 0.16
Zfp120Unknown, score: 0.21
Zfp119aUnknown, score: 0.08
Isl2Unknown, score: 0.62
Meig1Unknown, score: 0.37
Rhox9Unknown, score: 0.18
DgkzUnknown, score: 0.13
Cdc42ep1Unknown, score: 0.24
0610010K14RikUnknown, score: 0.01
RarsUnknown, score: 0
Ccdc117Unknown, score: 0.09
Cnot6Unknown, score: 0.18
Tsr1Unknown, score: 0.48
Slc16a6Unknown, score: 0.42
Pik3r6Unknown, score: 0.39
Ttc7bUnknown, score: 0.06
Pld4Unknown, score: 0.23
JkampUnknown, score: 0.13
Cbll1Unknown, score: 0
Tecpr2Unknown, score: 0.18
Spata7Unknown, score: 0.27
Tdp1Unknown, score: 0.49
Rab15Unknown, score: 0.17
Slc25a47Unknown, score: 0.39
Dnal1Unknown, score: 0.08
PeloUnknown, score: 0.04
Arrdc3Unknown, score: 0.11
Rnf44Unknown, score: 0.17
Txndc5Unknown, score: 0.09
Cdk20Unknown, score: 0.49
Epdr1Unknown, score: 0.34
Golm1Unknown, score: 0.01
Dusp22Unknown, score: 0.02
Utp15Unknown, score: 0.27
Ankrd32Unknown, score: 0.12
Fam149bUnknown, score: 0.08
Kctd9Unknown, score: 0.06
Dock9Unknown, score: 0.15
Mmrn2Unknown, score: 0.02
Fam170bUnknown, score: 0.1
Zfp957Unknown, score: 0.2
PhyhipUnknown, score: 0.04
ThtpaUnknown, score: 0.05
Rcbtb2Unknown, score: 0.42
PpifUnknown, score: 0.02
Prkaa1Unknown, score: 0.13
Sgsm3Unknown, score: 0.1
Card10Unknown, score: 0.2
Lmf2Unknown, score: 0.1
Csdc2Unknown, score: 0.35
Espl1Unknown, score: 0.2
ToporsUnknown, score: -0.01
Gga1Unknown, score: 0.28
Fbxo4Unknown, score: 0.25
Zc3h7aUnknown, score: 0.12
Qtrtd1Unknown, score: 0.15
Rrn3Unknown, score: 0.09
Osbpl11Unknown, score: -0.01
Nsun3Unknown, score: 0.13
Rfc4Unknown, score: 0.04
Ildr1Unknown, score: 0.06
Ypel1Unknown, score: 0.18
Slc51aUnknown, score: 0.14
Sft2d1Unknown, score: 0.2
Gpsm3Unknown, score: 0.07
TecrUnknown, score: 0.35
PpcsUnknown, score: 0.02
Scaf8Unknown, score: 0.19
Wdr90Unknown, score: 0.34
Trip10Unknown, score: 0.02
Ift140Unknown, score: 0.19
VmacUnknown, score: 0.29
Cyp4f15Unknown, score: 0.11
Rpusd1Unknown, score: 0.01
CatsperdUnknown, score: 0.16
Ticam1Unknown, score: 0
Ttbk1Unknown, score: 0.06
Stap2Unknown, score: 0.06
Tcf19Unknown, score: 0.22
Unc119bUnknown, score: 0.42
Abhd3Unknown, score: 0.11
Afap1l1Unknown, score: 0.15
Kctd1Unknown, score: -0.01
Slc39a3Unknown, score: 0.07
Slc39a6Unknown, score: 0.33
Gramd3Unknown, score: 0.05
Me2Unknown, score: 0.14
Fbxo38Unknown, score: 0.17
LarsUnknown, score: 0.42
Psmg2Unknown, score: 0.07
Lrrtm2Unknown, score: 0.24
Rrp12Unknown, score: 0.08
Cyp2c50Unknown, score: 0.11
Btaf1Unknown, score: 0.27
Uqcc3Unknown, score: 0.05
Macrod1Unknown, score: 0.28
AC109138.1Unknown, score: 0.06
Kazald1Unknown, score: 0.13
Otub1Unknown, score: 0
YarsUnknown, score: 0.24
Psat1Unknown, score: 0.02
LpxnUnknown, score: 0.38
Gbf1Unknown, score: 0
Kank1Unknown, score: 0
Pdzd8Unknown, score: 0.08
Brms1Unknown, score: 0.2
AcacaUnknown, score: 0.28
Atf5Unknown, score: 0.15
Lgr4Unknown, score: 0.05
Ece2Unknown, score: 0.04
Arl2bpUnknown, score: 0.14
Wwp1Unknown, score: 0.1
Col16a1Unknown, score: 0.04
MylkUnknown, score: 0.14
Rdh1Unknown, score: 0.09
Nod1Unknown, score: 0.1
Slc12a6Unknown, score: -0.01
Mrpl10Unknown, score: 0.2
Mrpl41Unknown, score: 0.32
Rapgef1Unknown, score: 0.35
Aldh1l1Unknown, score: 0.07
Prrxl1Unknown, score: 0.23
Lgals2Unknown, score: 0.35
Ankrd1Unknown, score: 0.15
HaaoUnknown, score: 0.19
Tm6sf2Unknown, score: 0.23
BmycUnknown, score: 0.13
Whsc1Unknown, score: 0.2
Usp9yUnknown, score: 0.37
Mgat5Unknown, score: 0.02
Chd4Unknown, score: 0.25
BreUnknown, score: 0.11
CenpfUnknown, score: 0.06
Ap1s2Unknown, score: 0.2
Lin7aUnknown, score: 0.13
Shmt2Unknown, score: 0.29
Slc14a1Unknown, score: 0.36
Camk2dUnknown, score: 0.11
Cstf2Unknown, score: 0.14
Prkaa2Unknown, score: 0.26
Pip4k2bUnknown, score: 0.04
Rnf216Unknown, score: 0.4
Rnf144aUnknown, score: 0.76
Fermt3Unknown, score: 0.01
B3gnt5Unknown, score: 0.06
Slco3a1Unknown, score: 0.01
U2af1Unknown, score: 0.2
NapaUnknown, score: 0.06
Xrcc4Unknown, score: 0.36
Ak6Unknown, score: 0.35
Galnt2Unknown, score: 0.17
Sema3dUnknown, score: 0.03
Adamts6Unknown, score: 0.12
OgtUnknown, score: 0.16
Mthfd1Unknown, score: 0.16
Fam50aUnknown, score: 0
Foxp1Unknown, score: 0.13
Epsti1Unknown, score: 0.16
Zdhhc15Unknown, score: 0.18
Ccdc86Unknown, score: 0.08
Obfc1Unknown, score: 0.16
Chn1Unknown, score: 0.18
Card11Unknown, score: 0.12
Sft2d2Unknown, score: 0.53
Oxsr1Unknown, score: 0.51
Galnt16Unknown, score: 0.16
Rdh13Unknown, score: 0.12
Mtrf1lUnknown, score: 0.11
Ankhd1Unknown, score: 0.23
Aif1lUnknown, score: 0.2
2700081O15RikUnknown, score: 0.16
Cdca2Unknown, score: 0.15
Rnf169Unknown, score: 0.41
Ppp1r15bUnknown, score: 0.05
Irak2Unknown, score: 0.14
E2f8Unknown, score: 0
Ciapin1Unknown, score: 0.21
Pfdn4Unknown, score: 0.04
Dnaaf2Unknown, score: 0.05
Exosc4Unknown, score: 0.04
Fbxw17Unknown, score: 0.37
Uhrf2Unknown, score: 0.43
Supt3Unknown, score: 0.17
Plekha5Unknown, score: 0.06
MmaaUnknown, score: 0.37
Trip11Unknown, score: 0.2
SobpUnknown, score: -0.01
Fam64aUnknown, score: 0.12
Ms4a7Unknown, score: 0.02
Mbd5Unknown, score: -0.01
Kif24Unknown, score: 0.32
Tspan9Unknown, score: 0.05
AdtrpUnknown, score: 0.12
Prr5Unknown, score: 0.32
Mybpc1Unknown, score: 0.07
Actr5Unknown, score: 0.17
R3hdm4Unknown, score: 0.02
Prex2Unknown, score: -0.01
C1qtnf7Unknown, score: -0.01
Rnf20Unknown, score: 0.35
Fam175bUnknown, score: 0.25
Cald1Unknown, score: 0
Upk1aUnknown, score: 0.01
NpyUnknown, score: 0.24
TxlnaUnknown, score: 0.13
CtrlUnknown, score: 0.13
Ampd2Unknown, score: 0.1
Ank2Unknown, score: 0.02
Actn1Unknown, score: 0.02
BlvraUnknown, score: 0.22
Pgm3Unknown, score: 0.28
Glo1Unknown, score: 0.03
BrafUnknown, score: 0.03
Mzf1Unknown, score: 0.14
Cela1Unknown, score: 0.16
Mcf2Unknown, score: 0
AbrUnknown, score: 0.14
Art4Unknown, score: 0.63
Syt17Unknown, score: 0.42
Bmp3Unknown, score: 0.17
Phka2Unknown, score: 0.21
MpiUnknown, score: 0.25
Gpr18Unknown, score: 0.27
Slc35b1Unknown, score: 0.3
ManbaUnknown, score: 0.66
FdpsUnknown, score: -0.01
DgkgUnknown, score: 0.49
PgdUnknown, score: 0.07
TriobpUnknown, score: 0.11
Hba-a2Unknown, score: 0.03
Krt7Unknown, score: 0.18
Cox6b1Unknown, score: 0.03
Tas1r1Unknown, score: 0.07
Shroom2Unknown, score: 0.2
C8bUnknown, score: 0.03
Pde4cUnknown, score: 0.05
QdprUnknown, score: 0.01
PighUnknown, score: 0.15
Ly6aUnknown, score: -0.01
Amhr2Unknown, score: -0.01
110558Unknown, score: 0.1
Prdm2Unknown, score: 0.58
HdlbpUnknown, score: -0.01
Rps6ka3Unknown, score: 0.46
Cse1lUnknown, score: 0.39
Srsf1Unknown, score: 0.22
Pwp2Unknown, score: 0.25
PccaUnknown, score: 0.38
Lims1Unknown, score: 0.04
EtfaUnknown, score: 0.03
Scn2a1Unknown, score: 0.02
Slc18a1Unknown, score: 0.02
Slc8a3Unknown, score: 0.05
Slc9a4Unknown, score: 0.7
HlcsUnknown, score: 0.05
M1apUnknown, score: 0.29
TarsUnknown, score: 0.03
Mbd6Unknown, score: 0.01
Erc1Unknown, score: 0.12
Hmga1-rs1Unknown, score: 0.32
1700102P08RikUnknown, score: 0.19
2010002M12RikUnknown, score: 0.12
Acaa1aUnknown, score: 0.16
NpntUnknown, score: 0.21
PalmdUnknown, score: 0.07
Slc5a5Unknown, score: 0.01
Tle6Unknown, score: 0.1
Ly6g6dUnknown, score: 0.45
Impa2Unknown, score: 0.4
SelmUnknown, score: 0.22
Rad51cUnknown, score: 0.37
Spred1Unknown, score: 0.4
Supt16Unknown, score: 0.09
PawrUnknown, score: 0.11
ProscUnknown, score: 0.5
CygbUnknown, score: 0.2
Dcun1d1Unknown, score: 0.02
Pcdha1Unknown, score: 0.03
Tsga13Unknown, score: 0.07
Vps4aUnknown, score: 0.11
Lsm10Unknown, score: 0.02
Mta1Unknown, score: 0.14
Mta3Unknown, score: 0.02
Stim2Unknown, score: 0.13
Alpk3Unknown, score: 0.3
Dph1Unknown, score: 0.5
Slc19a2Unknown, score: 0.2
Pnpla3Unknown, score: 0.19
Fam57aUnknown, score: 0.05
Ube3bUnknown, score: 0.17
TirapUnknown, score: 0.14
Steap4Unknown, score: 0.18
Bloc1s4Unknown, score: 0.52
Stk33Unknown, score: 0.1
Asb7Unknown, score: 0.09
Slc2a9Unknown, score: 0.02
B3galt6Unknown, score: 0
Klf16Unknown, score: 0.46
Synpo2Unknown, score: 0.09
Mmp28Unknown, score: 0.19
Mrps6Unknown, score: 0.46
Man2a2Unknown, score: 0.08
Pofut1Unknown, score: 0.01
Ppp1r3aUnknown, score: -0.01
Rxfp2Unknown, score: 0.03
Acap3Unknown, score: 0.02
Eri3Unknown, score: 0.07
Igsf8Unknown, score: 0.24
Elmo1Unknown, score: 0.06
Emid1Unknown, score: 0.06
Col26a1Unknown, score: 0.02
Caskin2Unknown, score: 0.01
Cacng5Unknown, score: 0.02
Sec63Unknown, score: 0.52
Bmp2kUnknown, score: 0.26
Myh7Unknown, score: 0.11
Colec12Unknown, score: 0
P2ry14Unknown, score: 0.02
Ttbk2Unknown, score: 0.1
Wdr5Unknown, score: 0.3
Lnx2Unknown, score: 0.09
Zcchc14Unknown, score: 0.29
Asb14Unknown, score: 0.23
Stard4Unknown, score: 0.24
Stard5Unknown, score: 0.03
Stard6Unknown, score: 0.03
Recql5Unknown, score: 0.53
Grin3bUnknown, score: 0.02
Ubn1Unknown, score: 0.04
Usp48Unknown, score: 0.11
Cyp4f13Unknown, score: 0.06
PaplnUnknown, score: -0.01
Nxf7Unknown, score: 0.02
Klra17Unknown, score: -0.01
ParvbUnknown, score: 0.02
Znrf1Unknown, score: 0.27
Pilrb1Unknown, score: 0.03
Xpnpep2Unknown, score: 0.31
Smco4Unknown, score: 0.47
Xpnpep1Unknown, score: 0.1
Zfp704Unknown, score: 0.01
Slc8b1Unknown, score: 0.08
Adgrl4Unknown, score: 0.06
Atp13a1Unknown, score: 0.08
Acbd3Unknown, score: 0.12
Ripply3Unknown, score: 0.16
Cd209cUnknown, score: 0.05
Cd209dUnknown, score: 0
Acot8Unknown, score: 0.12
Rtkn2Unknown, score: 0.04
Usp33Unknown, score: 0.32
GlmnUnknown, score: 0.01
Inpp5jUnknown, score: 0.07
Sumo2Unknown, score: 0.03
Zfp369Unknown, score: 0.27
Myoz3Unknown, score: 0.1
Prima1Unknown, score: 0.04
Acer1Unknown, score: 0.14
Asic3Unknown, score: 0.37
Galnt10Unknown, score: 0.31
Acot3Unknown, score: 0.48
Acot4Unknown, score: 0.17
Il17rdUnknown, score: 0.31
ApobrUnknown, score: 0.38
Nme7Unknown, score: 0.27
Dicer1Unknown, score: 0.07
BspryUnknown, score: 0.15
SugctUnknown, score: 0.44
Prpf8Unknown, score: 0.23
Pcdha12Unknown, score: 0.43
SardhUnknown, score: 0.13
Eif4a3Unknown, score: 0.17
Fam195bUnknown, score: 0.04
Edem1Unknown, score: 0.06
Lrrc4Unknown, score: 0.03
Rspo1Unknown, score: 0.01
Hps1Unknown, score: 0.02
Phf21aUnknown, score: 0.22
TmlheUnknown, score: 0.36
Cabp7Unknown, score: 0.18
Zfp286Unknown, score: -0.01
Wdr81Unknown, score: 0.07
Ell2Unknown, score: 0.1
Rfpl4Unknown, score: 0.13
Abcg4Unknown, score: 0.01
Rassf3Unknown, score: 0.56
Lrrc75bUnknown, score: 0.16
PirtUnknown, score: 0.02
Trpv1Unknown, score: 0.09
Zfp3Unknown, score: 0.18
BC049762Unknown, score: 0.73
Fam65bUnknown, score: 0.08
Hspa1aUnknown, score: 0.06
Abhd16aUnknown, score: 0.12
Kdm4bUnknown, score: 0.07
Mcfd2Unknown, score: 0
Mtmr11Unknown, score: 0.43
Cnksr1Unknown, score: 0.15
Vps37dUnknown, score: 0.01
Mical3Unknown, score: 0.09
Reps2Unknown, score: 0.01
Pld6Unknown, score: 0.05
Zzef1Unknown, score: 0.01
Tmem199Unknown, score: 0.04
Dcdc2aUnknown, score: 0.09
Zfp691Unknown, score: 0.13
Gm13152Unknown, score: 0.16
Skint3Unknown, score: 0.27
NhsUnknown, score: 0.3
BptfUnknown, score: 0.02
Ggt7Unknown, score: 0.05
Arhgef17Unknown, score: 0.07
Larp4Unknown, score: 0.21
Zbtb7cUnknown, score: 0.02
Elfn2Unknown, score: 0.15
Wdr11Unknown, score: 0.04
Kctd12bUnknown, score: 0.13
Baiap2l2Unknown, score: 0.02
Tbc1d16Unknown, score: 0.12
Wdr37Unknown, score: 0.01
Cfap69Unknown, score: 0.09
Gtpbp10Unknown, score: 0.14
C2cd2Unknown, score: 0.12
Gm608Unknown, score: 0.06
Klhl25Unknown, score: 0.17
Alg11Unknown, score: 0.17
Pknox2Unknown, score: 0.04
Pif1Unknown, score: 0.02
MlxipUnknown, score: 0.01
9830147E19RikUnknown, score: 0.3
Aph1bUnknown, score: 0.03
Dhx37Unknown, score: 0.21
Yeats2Unknown, score: 0.11
BtlaUnknown, score: 0.12
Fam180aUnknown, score: 0.29
ExogUnknown, score: 0.1
Btbd2Unknown, score: 0.1
Tor1aip1Unknown, score: 0.18
Zfp871Unknown, score: 0.77
Sgms1Unknown, score: 0.04
1810043H04RikUnknown, score: 0.11
Mterf1bUnknown, score: 0.29
Rsrc2Unknown, score: 0.19
Kntc1Unknown, score: 0.24
Tspan10Unknown, score: 0.41
Slc25a38Unknown, score: 0.26
Eif4g1Unknown, score: 0.12
Fam20aUnknown, score: 0.22
Akr1d1Unknown, score: 0.25
Creb3l3Unknown, score: 0.08
Prrg3Unknown, score: 0.2
Sde2Unknown, score: 0.18
FanciUnknown, score: 0.17
Dock3Unknown, score: 0.09
Slc26a7Unknown, score: 0.11
Unc13cUnknown, score: 0.26
Ccdc62Unknown, score: 0
Cpeb3Unknown, score: 0.04
Adamts18Unknown, score: 0.21
Thnsl1Unknown, score: 0.02
Zfp280cUnknown, score: 0.12
Hmgcll1Unknown, score: 0.01
NpbUnknown, score: 0.06
Rbmx2Unknown, score: 0.12
Ulk4Unknown, score: 0.4
Zc3hav1lUnknown, score: 0.15
Samd9lUnknown, score: 0.36
Clic6Unknown, score: 0.21
Dtx3lUnknown, score: 0.23
Osgin2Unknown, score: 0.25
GanUnknown, score: 0.3
Gps1Unknown, score: 0.06
Gen1Unknown, score: 0.16
Eif2b1Unknown, score: 0.14
Gtf2h3Unknown, score: 0.03
Trim30dUnknown, score: 0.11
Trp53bp2Unknown, score: 0.2
Hace1Unknown, score: -0.01
Rgag1Unknown, score: 0.01
Enpp3Unknown, score: 0.07
Tyw3Unknown, score: 0.2
Sectm1aUnknown, score: 0
Erich3Unknown, score: 0.12
Bend7Unknown, score: 0.26
Dhtkd1Unknown, score: 0.01
Kif15Unknown, score: 0.01
Dennd2aUnknown, score: 0.21
Tbc1d25Unknown, score: 0.2
Pgbd5Unknown, score: 0.04
B3gntl1Unknown, score: 0.06
MetrnlUnknown, score: 0.03
Adcy2Unknown, score: 0.17
Zfp658Unknown, score: 0.22
Zfp719Unknown, score: -0.01
IrgqUnknown, score: -0.01
Lrch2Unknown, score: 0.14
Zfp677Unknown, score: 0.11
Tdrd6Unknown, score: 0.06
MkxUnknown, score: 0.1
Ppp1r36Unknown, score: 0.01
Brcc3Unknown, score: 0.29
Tbc1d4Unknown, score: 0.23
Lacc1Unknown, score: 0.07
Zfp947Unknown, score: 0.11
Ints9Unknown, score: 0.11
Kbtbd2Unknown, score: -0.01
Gltscr1lUnknown, score: 0.24
D15Ertd621eUnknown, score: 0.05
Trim41Unknown, score: 0.02
Alkbh1Unknown, score: 0.23
Lzts1Unknown, score: 0.07
D130040H23RikUnknown, score: 0.44
March11Unknown, score: -0.01
Churc1Unknown, score: 0.36
Lrtm2Unknown, score: 0.2
Cpne9Unknown, score: 0.11
Mtrf1Unknown, score: 0.06
Cln5Unknown, score: 0.7
Pank3Unknown, score: 0.07
SuoxUnknown, score: 0.41
Mtss1Unknown, score: 0.08
EfhbUnknown, score: 0.33
AdoUnknown, score: 0.16
Ccdc114Unknown, score: 0.13
Nomo1Unknown, score: 0.13
TifaUnknown, score: -0.01
MrgprfUnknown, score: 0.05
Fancd2Unknown, score: 0.32
Wwc1Unknown, score: 0.04
Cspp1Unknown, score: 0.13
Mgst2Unknown, score: 0.24
Pcdh9Unknown, score: 0.01
Vstm2aUnknown, score: 0.44
Trib1Unknown, score: -0.01
Asap2Unknown, score: 0.1
Dennd6aUnknown, score: 0.1
Pde12Unknown, score: 0.12
Asxl3Unknown, score: 0.02
Zfyve26Unknown, score: 0.01
Syne3Unknown, score: 0.06
Nhlrc3Unknown, score: 0.04
Dcaf15Unknown, score: 0.06
Cc2d1aUnknown, score: 0.05
8030462N17RikUnknown, score: 0.12
GsapUnknown, score: -0.01
Wdr25Unknown, score: 0.01
Zfp729aUnknown, score: 0.21
Arap2Unknown, score: 0.15
Mapre2Unknown, score: 0.07
Mms22lUnknown, score: 0.12
Ccdc110Unknown, score: 0.2
Frat2Unknown, score: 0.19
Lactb2Unknown, score: 0.07
9330159F19RikUnknown, score: 0.1
Fam193bUnknown, score: 0.01
SprnUnknown, score: 0.07
Gm266Unknown, score: 0.19
RhoUnknown, score: 0.23
Pqlc2Unknown, score: 0.41
Zfp273Unknown, score: 0.2
Iffo2Unknown, score: 0.25
Aldh4a1Unknown, score: 0.39
Mars2Unknown, score: 0.2
Gm17296Unknown, score: 0.05
Ccdc64bUnknown, score: 0.02
Arl14epUnknown, score: 0.35
Chpt1Unknown, score: 0.05
DseUnknown, score: 0.32
Kctd7Unknown, score: 0.13
Slc45a3Unknown, score: 0.2
Scfd2Unknown, score: 0.5
Tnpo2Unknown, score: 0.05
Mfsd4Unknown, score: 0.13
Pdlim2Unknown, score: 0.06
Evi5lUnknown, score: 0.32
Wdr19Unknown, score: 0.52
Cdkl3Unknown, score: 0.03
Phf3Unknown, score: 0.12
213119Unknown, score: 0.04
TapbplUnknown, score: 0.09
Scyl2Unknown, score: 0.04
Pddc1Unknown, score: 0.07
Rassf4Unknown, score: 0.21
8430408G22RikUnknown, score: 0.27
Armc2Unknown, score: 0.04
Klhdc8aUnknown, score: 0.07
DstykUnknown, score: 0.14
Lgi3Unknown, score: 0.23
Dis3lUnknown, score: 0.11
Plekhh2Unknown, score: 0.05
Dync2li1Unknown, score: 0.1
Map9Unknown, score: 0
Slc44a3Unknown, score: 0.16
Arhgef19Unknown, score: -0.01
Zfp598Unknown, score: 0.16
Tbl3Unknown, score: 0
Plekhg1Unknown, score: 0.18
Arcn1Unknown, score: 0.3
Bms1Unknown, score: 0.22
Atg9bUnknown, score: 0.04
Fam83fUnknown, score: 0.04
Fbxw10Unknown, score: 0.05
Tnrc6bUnknown, score: 0.74
Agap3Unknown, score: 0.33
Ccdc186Unknown, score: 0.12
Larp1bUnknown, score: 0.41
Megf11Unknown, score: 0.06
Slc18a2Unknown, score: 0.23
Slc24a1Unknown, score: 0.06
Tet2Unknown, score: 0.12
Ago3Unknown, score: 0.09
Etnk2Unknown, score: 0.15
Gm4787Unknown, score: 0.44
Fam168bUnknown, score: 0.47
Cdc73Unknown, score: 0.08
GnptgUnknown, score: 0.24
Cep164Unknown, score: 0.14
Aldh5a1Unknown, score: 0.02
PstkUnknown, score: 0.1
4930486L24RikUnknown, score: 0.34
Slc25a29Unknown, score: 0.03
ChadlUnknown, score: 0.14
IqubUnknown, score: 0.25
Rcor3Unknown, score: 0.03
Mb21d1Unknown, score: 0.2
Mmp21Unknown, score: 0.26
Arid5aUnknown, score: 0.25
Csnk1g1Unknown, score: -0.01
Kdm5aUnknown, score: 0.08
Chtf18Unknown, score: 0.01
Fam173aUnknown, score: 0.18
Fbxl16Unknown, score: 0.15
Cecr5Unknown, score: 0.17
Mob3bUnknown, score: 0.11
Chtf8Unknown, score: 0.08
Wfikkn1Unknown, score: 0.28
Vgll2Unknown, score: 0.13
ManealUnknown, score: 0.51
Slc43a2Unknown, score: 0.09
Hip1Unknown, score: 0.18
DiexfUnknown, score: 0.25
Kri1Unknown, score: 0.19
Trmt2bUnknown, score: -0.01
Wipf1Unknown, score: 0.48
Slc36a1Unknown, score: -0.01
Brinp3Unknown, score: 0.02
Csrnp1Unknown, score: 0.08
Rab11fip3Unknown, score: 0.07
Entpd3Unknown, score: 0.03
Rap1bUnknown, score: 0.09
Pomgnt2Unknown, score: 0.02
Nav1Unknown, score: 0.34
Fam73aUnknown, score: 0.15
Mfsd6lUnknown, score: 0.58
Cnksr3Unknown, score: 0.14
Ginm1Unknown, score: 0.04
Phactr2Unknown, score: 0.2
Adgrg6Unknown, score: 0.44
Ccdc28aUnknown, score: 0.06
Fam26fUnknown, score: 0.35
BC021785Unknown, score: 0.01
Lace1Unknown, score: 0.37
Zfp365Unknown, score: 0.45
PdxkUnknown, score: 0.14
Shc2Unknown, score: 0.12
Cdc34Unknown, score: 0.02
Lppr3Unknown, score: 0.17
Med16Unknown, score: 0.33
Sbno2Unknown, score: 0.12
Abhd17aUnknown, score: 0.23
AU041133Unknown, score: 0.32
Aldh1l2Unknown, score: 0.09
Appl2Unknown, score: 0.63
Tcp11l2Unknown, score: 0.12
Slc17a8Unknown, score: 0.07
Socs2Unknown, score: 0.07
Eea1Unknown, score: 0.18
Cep290Unknown, score: 0.41
Rab21Unknown, score: 0.11
Zfc3h1Unknown, score: 0.17
Rab3ipUnknown, score: 0.02
Tmem5Unknown, score: 0.03
Agap2Unknown, score: 0.18
MarsUnknown, score: 0.52
Myl6bUnknown, score: 0.36
LgalslUnknown, score: 0.02
Ugp2Unknown, score: 0.02
WdpcpUnknown, score: 0.11
Ehbp1Unknown, score: 0.15
PapolgUnknown, score: 0.21
Ccdc85aUnknown, score: 0.19
Efemp1Unknown, score: 0.27
4931440F15RikUnknown, score: 0.07
Hbq1aUnknown, score: 0.16
Clint1Unknown, score: 0.06
Adamts2Unknown, score: 0
Mfap3Unknown, score: 0.14
Gemin5Unknown, score: 0.02
Mrpl22Unknown, score: 0.35
Iba57Unknown, score: 0.1
Tmem11Unknown, score: 0.36
Usp22Unknown, score: 0.04
Usp43Unknown, score: 0.14
Kctd11Unknown, score: 0.08
Acap1Unknown, score: 0.16
Neurl4Unknown, score: 0.27
Mgl2Unknown, score: 0.12
Arrb2Unknown, score: 0.15
Gltpd2Unknown, score: 0.25
Camta2Unknown, score: 0.06
Dhx33Unknown, score: 0.01
Spns2Unknown, score: 0.3
Fam222bUnknown, score: 0.15
Proca1Unknown, score: 0.37
Utp6Unknown, score: 0.13
Adap2Unknown, score: 0.04
Nle1Unknown, score: 0.03
Mrm1Unknown, score: 0.03
Ptrh2Unknown, score: 0.31
Gm525Unknown, score: 0.35
HlfUnknown, score: 0.19
Spata20Unknown, score: 0.01
Xylt2Unknown, score: 0.02
Kat7Unknown, score: 0.07
Prr15lUnknown, score: 0.4
Scrn2Unknown, score: 0.09
Gpr179Unknown, score: 0
Nr1d1Unknown, score: 0.05
Plekhh3Unknown, score: 0.09
Tmem106aUnknown, score: 0
Dhx8Unknown, score: 0.05
BC030867Unknown, score: 0.1
Cdc27Unknown, score: 0.15
Abca8aUnknown, score: -0.01
Abca9Unknown, score: 0
Abca5Unknown, score: 0.17
Cd300eUnknown, score: 0.16
Slc16a5Unknown, score: 0.03
Llgl2Unknown, score: -0.01
Fbf1Unknown, score: 0.18
Rnf157Unknown, score: 0.11
Qrich2Unknown, score: 0.18
Rhbdf2Unknown, score: 0.07
Tmc8Unknown, score: 0.44
Nol10Unknown, score: 0
Trappc12Unknown, score: 0.46
Snx13Unknown, score: 0.26
Stxbp6Unknown, score: 0.04
Baz1aUnknown, score: 0.12
MbipUnknown, score: 0.02
Slc25a21Unknown, score: 0.36
Ctage5Unknown, score: 0.1
Gm527Unknown, score: 0.02
Mgat2Unknown, score: 0.32
Plekhd1Unknown, score: -0.01
Susd6Unknown, score: 0.05
Zfyve1Unknown, score: 0.36
Fam161bUnknown, score: 0.07
Coq6Unknown, score: 0.22
Eif2b2Unknown, score: 0.06
CipcUnknown, score: 0.5
Tmem63cUnknown, score: 0.18
Pomt2Unknown, score: 0.03
Ahsa1Unknown, score: 0.07
Ism2Unknown, score: 0.24
Kcnk13Unknown, score: 0.11
Nrde2Unknown, score: 0.21
9030617O03RikUnknown, score: 0.07
Itpk1Unknown, score: 0.08
Ifi27l2bUnknown, score: 0.19
Eif5Unknown, score: 0.09
BC022687Unknown, score: 0.31
Pacs2Unknown, score: 0.09
Wdr60Unknown, score: 0.02
Cdca7lUnknown, score: 0.21
Larp4bUnknown, score: 0
Heatr1Unknown, score: 0.25
Vps41Unknown, score: 0.39
AmphUnknown, score: 0.23
Zfp322aUnknown, score: 0.16
Mboat1Unknown, score: 0.3
GmdsUnknown, score: 0.06
Nup153Unknown, score: 0.19
Kdm1bUnknown, score: 0.28
Rnf144bUnknown, score: 0.04
B4galt7Unknown, score: 0.21
Cdc14bUnknown, score: 0.17
Zfp595Unknown, score: 0.18
Ice1Unknown, score: 0.04
Clptm1lUnknown, score: 0.19
Zfyve16Unknown, score: 0.32
Lhfpl2Unknown, score: 0.19
Gcnt4Unknown, score: 0.28
Btf3Unknown, score: 0.08
Fcho2Unknown, score: 0.35
Mrps27Unknown, score: 0.09
Marveld2Unknown, score: 0.18
Srek1Unknown, score: 0.09
Mier3Unknown, score: 0.16
CcnoUnknown, score: 0.07
Paip1Unknown, score: 0.02
PxkUnknown, score: 0.09
Ube2e2Unknown, score: 0.03
Sec24cUnknown, score: 0.16
Polr3aUnknown, score: 0.02
Fam208aUnknown, score: 0.04
Oxnad1Unknown, score: 0.12
WapalUnknown, score: 0.02
Dlgap5Unknown, score: 0.23
KhnynUnknown, score: 0.09
CenpjUnknown, score: 0.3
Scara3Unknown, score: 0.06
Ccar2Unknown, score: 0.16
Akap11Unknown, score: 0.66
Vwa8Unknown, score: 0.2
Pcdh17Unknown, score: 0.05
Farp1Unknown, score: 0.15
Itgbl1Unknown, score: 0.18
DapUnknown, score: 0.08
March6Unknown, score: 0.11
Nipal2Unknown, score: 0.15
AbraUnknown, score: 0.18
E430025E21RikUnknown, score: 0.1
Fam49bUnknown, score: 0.32
Them6Unknown, score: 0.02
Lrrc14Unknown, score: 0.09
Lrrc24Unknown, score: 0.1
Arhgap39Unknown, score: 0.35
Ankrd54Unknown, score: 0.07
Tmem184bUnknown, score: 0.25
Tomm22Unknown, score: 0.11
Mkl1Unknown, score: 0.07
McatUnknown, score: 0.02
Zbed4Unknown, score: 0.06
Alg12Unknown, score: 0.04
Gxylt1Unknown, score: 0.23
Pphln1Unknown, score: 0.18
Soat2Unknown, score: 0.11
AaasUnknown, score: 0.25
SpidrUnknown, score: 0.04
Fgd4Unknown, score: 0.23
Tmem191cUnknown, score: 0.07
Scarf2Unknown, score: 0.06
Eif2b5Unknown, score: 0.03
Atp13a4Unknown, score: 0.02
Tmem44Unknown, score: 0.07
Lsg1Unknown, score: 0.05
Fam43aUnknown, score: 0.06
Pak2Unknown, score: 0.01
NrrosUnknown, score: -0.01
Adcy5Unknown, score: 0.03
Dirc2Unknown, score: 0.11
Golgb1Unknown, score: 0.11
Impg2Unknown, score: 0.06
Cldnd1Unknown, score: 0.09
224273Unknown, score: -0.01
Rbm11Unknown, score: 0.15
Cyyr1Unknown, score: 0.03
Map3k7clUnknown, score: 0.03
Scaf4Unknown, score: 0.24
Setd4Unknown, score: 0.13
Zdhhc14Unknown, score: 0.34
Tfb1mUnknown, score: 0.06
Acat3Unknown, score: 0.04
Traf7Unknown, score: 0
Rab40cUnknown, score: 0.27
Bnip1Unknown, score: -0.01
Btbd9Unknown, score: 0
Slc37a1Unknown, score: 0.32
Zfp472Unknown, score: 0.26
March2Unknown, score: 0.01
Vps52Unknown, score: 0.07
Bag6Unknown, score: 0.12
Adgrf5Unknown, score: -0.01
Enpp4Unknown, score: 0.44
Abcc10Unknown, score: 0.06
Rrp36Unknown, score: 0.21
Pex6Unknown, score: 0.17
224829Unknown, score: 0.05
AI661453Unknown, score: 0.19
Usp49Unknown, score: 0.12
Treml4Unknown, score: 0.5
Plcl2Unknown, score: 0.2
Dpp9Unknown, score: 0.13
SafbUnknown, score: 0.22
2410015M20RikUnknown, score: 0.04
Dus3lUnknown, score: 0
Crb3Unknown, score: 0.01
Pja2Unknown, score: 0.03
Fez2Unknown, score: 0.39
Srsf7Unknown, score: 0.09
Map4k3Unknown, score: 0.11
Ttc7Unknown, score: 0.05
Fbxo11Unknown, score: 0.31
SvilUnknown, score: -0.01
WacUnknown, score: 0.04
Rbbp8Unknown, score: 0.28
Zfp521Unknown, score: 0.23
Rsl24d1Unknown, score: 0.12
Fhod3Unknown, score: 0.21
AW554918Unknown, score: 0.13
Lims2Unknown, score: 0.51
Etf1Unknown, score: 0.01
Sra1Unknown, score: 0
Rell2Unknown, score: 0.12
Ticam2Unknown, score: 0
Cep120Unknown, score: 0.05
Slc27a6Unknown, score: 0.02
Gm4841Unknown, score: 0.21
Sh3tc2Unknown, score: 0.12
Mapk4Unknown, score: 0
Rnf165Unknown, score: 0.18
Haus1Unknown, score: 0.16
Cd226Unknown, score: 0.38
Ppp2r5bUnknown, score: 0.09
BC021614Unknown, score: 0.13
Taf6lUnknown, score: 0.18
Eml3Unknown, score: 0.37
Cyb561a3Unknown, score: 0.2
Patl1Unknown, score: 0.13
Nmrk1Unknown, score: 0.11
Trpm6Unknown, score: 0.36
RorbUnknown, score: 0.14
Abhd17bUnknown, score: 0.1
Tmem252Unknown, score: 0.13
Glis3Unknown, score: 0.03
Ermp1Unknown, score: 0.01
MyofUnknown, score: 0.22
Morn4Unknown, score: 0.06
Cox15Unknown, score: 0.08
Cyp2c44Unknown, score: 0.01
Erlin1Unknown, score: 0.14
Fam178aUnknown, score: 0.05
Peo1Unknown, score: 0.06
Wbp1lUnknown, score: 0.23
InaUnknown, score: 0.07
Taf5Unknown, score: 0.61
Habp2Unknown, score: 0.01
Afap1l2Unknown, score: 0.31
Ablim1Unknown, score: 0.11
Epb4.1l5Unknown, score: 0.05
LctUnknown, score: 0.25
Rab29Unknown, score: 0.29
Ipo9Unknown, score: 0.1
Zbtb41Unknown, score: 0.47
Rasal2Unknown, score: 0.16
BC026585Unknown, score: 0.11
Fmo4Unknown, score: 0.12
TiprlUnknown, score: 0.4
Atf6Unknown, score: 0.18
AI607873Unknown, score: 0.14
Ahctf1Unknown, score: 0.08
Wdr26Unknown, score: 0.18
Mark1Unknown, score: 0.09
Kctd3Unknown, score: 0.19
HhatUnknown, score: 0.02
Kcnq5Unknown, score: 0.06
Plekhb2Unknown, score: 0.05
Kansl3Unknown, score: 0.09
Actr1bUnknown, score: 0.21
Eif5bUnknown, score: 0.33
Slc9a2Unknown, score: 0.01
Dnah7bUnknown, score: 0.07
Slc39a10Unknown, score: 0.09
HibchUnknown, score: 0.04
Ormdl1Unknown, score: 0.08
StradbUnknown, score: 0.23
Ino80dUnknown, score: 0.21
Ccnyl1Unknown, score: 0.16
Gpbar1Unknown, score: 0.04
B3gnt7Unknown, score: 0.08
DgkdUnknown, score: 0.51
Usp40Unknown, score: 0.1
EspnlUnknown, score: 0.01
Fam132bUnknown, score: 0.17
2310035C23RikUnknown, score: 0.12
Zcchc2Unknown, score: 0.39
Cdh19Unknown, score: 0.26
Rpp38Unknown, score: 0.06
Proser2Unknown, score: 0.06
Uap1l1Unknown, score: 0.3
Rabl6Unknown, score: 0.19
Obp2aUnknown, score: 0.29
Camsap1Unknown, score: 0.1
Qsox2Unknown, score: 0.11
Sec16aUnknown, score: 0.12
Ddx31Unknown, score: 0.15
Coq4Unknown, score: 0.17
Zer1Unknown, score: 0.11
D2Wsu81eUnknown, score: 0.09
Nup188Unknown, score: 0.09
Sh3glb2Unknown, score: 0.34
BC005624Unknown, score: 0
Ppapdc3Unknown, score: 0.1
Fam129bUnknown, score: 0.11
Lrsam1Unknown, score: 0.01
Mapkap1Unknown, score: 0.04
GsnUnknown, score: 0.09
Rabgap1Unknown, score: 0.04
Dennd1aUnknown, score: 0.01
Gtdc1Unknown, score: 0.13
CytipUnknown, score: 0.25
Ccdc148Unknown, score: 0.73
Pkp4Unknown, score: 0.4
Klhl41Unknown, score: 0.29
PpigUnknown, score: 0.01
Tlk1Unknown, score: 0.21
Mettl8Unknown, score: 0.21
Pdk1Unknown, score: -0.01
Atp5g3Unknown, score: 0.04
Sestd1Unknown, score: 0.04
CerklUnknown, score: 0.13
Zdhhc5Unknown, score: 0.1
P2rx3Unknown, score: 0.03
Tnks1bp1Unknown, score: 0.14
MaddUnknown, score: 0.01
Slc35c1Unknown, score: 0.14
Prrg4Unknown, score: 0.07
Zfp770Unknown, score: 0.03
MallUnknown, score: 0.25
F830045P16RikUnknown, score: 0.05
Csrp2bpUnknown, score: 0.24
Gm561Unknown, score: 0.12
Sdcbp2Unknown, score: 0.2
Trib3Unknown, score: 0.03
Mylk2Unknown, score: 0.03
Phf20Unknown, score: 0.57
Dlgap4Unknown, score: 0.05
Tgif2Unknown, score: 0.25
RalgapbUnknown, score: 0.01
Ppp1r16bUnknown, score: 0
Pcif1Unknown, score: 0.03
Zfp334Unknown, score: 0
Zmynd8Unknown, score: 0.15
Ddx27Unknown, score: 0.13
Tshz2Unknown, score: 0.02
Zfp217Unknown, score: 0.02
Npepl1Unknown, score: 0.25
Ppp1r3dUnknown, score: 0.12
Taf4aUnknown, score: 0.07
Helz2Unknown, score: 0.04
Gmeb2Unknown, score: 0.07
Samd10Unknown, score: 0.14
Zbtb10Unknown, score: -0.01
Ythdf3Unknown, score: 0.33
Acad9Unknown, score: 0.16
4932438A13RikUnknown, score: 0.32
Nudt6Unknown, score: 0.03
Hnrnpa3Unknown, score: 0
Spg20Unknown, score: 0.06
Tm4sf4Unknown, score: 0.18
Clrn1Unknown, score: 0.04
GmpsUnknown, score: 0
CtsoUnknown, score: 0.39
Fhdc1Unknown, score: 0.41
Fcrl1Unknown, score: 0.14
Rrnad1Unknown, score: 0.06
Syt11Unknown, score: 0.42
Pbxip1Unknown, score: 0.03
BC028528Unknown, score: 0.16
Otud7bUnknown, score: 0.08
Pias3Unknown, score: 0.08
Vangl1Unknown, score: 0.05
Rsbn1Unknown, score: 0.03
CymUnknown, score: 0.38
Strip1Unknown, score: 0.24
Ahcyl1Unknown, score: 0.12
Amigo1Unknown, score: 0.26
5330417C22RikUnknown, score: 0.12
Slc25a24Unknown, score: 0.06
Cdc14aUnknown, score: 0.03
Slc35a3Unknown, score: 0.13
CenpeUnknown, score: 0.21
Gbp7Unknown, score: 0.31
Gtf2bUnknown, score: 0.08
Clca2Unknown, score: 0.49
Ak5Unknown, score: 0.31
Ddx58Unknown, score: 0.08
Ndufb6Unknown, score: 0.27
Nol6Unknown, score: 0.33
Car9Unknown, score: 0.43
Npr2Unknown, score: 0.06
Slc25a51Unknown, score: 0.02
ShbUnknown, score: 0
Galnt12Unknown, score: 0.53
Tmeff1Unknown, score: 0.19
Zfp189Unknown, score: 0.48
AldobUnknown, score: 0.31
IkbkapUnknown, score: 0.08
E130308A19RikUnknown, score: 0.21
Acer2Unknown, score: 0.15
FocadUnknown, score: 0.08
LeprotUnknown, score: 0.26
Pars2Unknown, score: 0.1
Zcchc11Unknown, score: 0.08
Prpf38aUnknown, score: 0.06
Nrd1Unknown, score: 0.27
Efcab14Unknown, score: 0.14
Lrrc41Unknown, score: 0.11
Tmem69Unknown, score: 0.01
Tesk2Unknown, score: 0.46
Ipo13Unknown, score: 0.07
Szt2Unknown, score: 0.03
Tmem125Unknown, score: 0.06
AU022252Unknown, score: 0.2
Foxj3Unknown, score: 0
Nt5c1aUnknown, score: 0.36
Pabpc4Unknown, score: 0.04
Rhbdl2Unknown, score: 0.07
YrdcUnknown, score: 0.28
Zc3h12aUnknown, score: 0.11
Eva1bUnknown, score: 0
Fam167bUnknown, score: 0.34
Hcrtr1Unknown, score: 0.01
Sesn2Unknown, score: 0.1
Wdtc1Unknown, score: 0.07
PigvUnknown, score: 0.03
Aim1lUnknown, score: 0
Slc30a2Unknown, score: 0.12
Grhl3Unknown, score: 0.07
Il22ra1Unknown, score: 0.23
Emc1Unknown, score: 0.32
Igsf21Unknown, score: 0.07
CroccUnknown, score: -0.01
Vps13dUnknown, score: 0.29
NppaUnknown, score: 0.02
Phf13Unknown, score: 0.16
Megf6Unknown, score: 0.01
B930041F14RikUnknown, score: 0.11
Klhl17Unknown, score: 0.13
Nupl2Unknown, score: 0.03
Gbx1Unknown, score: 0.17
Galnt11Unknown, score: 0.01
Kmt2cUnknown, score: 0.25
Insig1Unknown, score: 0.47
Zfyve28Unknown, score: 0.16
Fam193aUnknown, score: -0.01
Tnip2Unknown, score: 0.13
Tapt1Unknown, score: 0.54
Lrrc66Unknown, score: 0
PpatUnknown, score: 0.03
Polr2bUnknown, score: 0
Uba6Unknown, score: 0.06
Ythdc1Unknown, score: 0.26
Grsf1Unknown, score: 0.2
Cox18Unknown, score: 0.48
Parm1Unknown, score: 0.29
Fras1Unknown, score: 0.37
Paqr3Unknown, score: 0.28
Plac8Unknown, score: 0.73
Agpat9Unknown, score: 0.16
Lrrc8dUnknown, score: 0.06
GakUnknown, score: 0.14
Slc26a1Unknown, score: 0.04
Gcn1l1Unknown, score: -0.01
Vsig10Unknown, score: 0.51
Fbxo21Unknown, score: 0.02
Fbxw8Unknown, score: 0.2
Trafd1Unknown, score: 0.23
Naa25Unknown, score: 0.14
Fam109aUnknown, score: 0.32
B3gnt4Unknown, score: 0.21
Rimbp2Unknown, score: 0.22
Agfg2Unknown, score: 0.1
BC037034Unknown, score: 0.31
Micall2Unknown, score: 0.09
Snx8Unknown, score: 0.15
Amz1Unknown, score: 0.15
Fbxl18Unknown, score: 0.15
DaglbUnknown, score: 0.14
Aimp2Unknown, score: 0.19
Ccz1Unknown, score: 0.05
Lmtk2Unknown, score: 0.03
Pdap1Unknown, score: 0.11
Bud31Unknown, score: 0.03
Katnal1Unknown, score: 0.07
Uspl1Unknown, score: 0.14
Fam221aUnknown, score: 0.04
Fkbp14Unknown, score: 0.04
Vopp1Unknown, score: 0.22
Ccser1Unknown, score: 0.25
Thnsl2Unknown, score: 0.11
Tmem150aUnknown, score: 0.09
Mat2aUnknown, score: 0.26
Mob1aUnknown, score: 0.78
Cyp26b1Unknown, score: 0.26
C87436Unknown, score: 0.05
HmcesUnknown, score: 0.38
Iqsec1Unknown, score: 0.06
Hdac11Unknown, score: 0.45
Tmf1Unknown, score: 0.02
Gxylt2Unknown, score: 0.12
Ppp4r2Unknown, score: 0.05
Vgll4Unknown, score: 0.02
Wnk1Unknown, score: 0.04
C1rlUnknown, score: 0.33
Clec2eUnknown, score: 0.54
Clec9aUnknown, score: 0.01
Gprc5aUnknown, score: 0.12
RergUnknown, score: -0.01
DeraUnknown, score: 0.09
Pyroxd1Unknown, score: 0.16
Stk38lUnknown, score: 0.09
Klhl42Unknown, score: 0.3
Ccdc136Unknown, score: 0.17
Tcaf2Unknown, score: 0.12
Zfp212Unknown, score: 0.41
OscarUnknown, score: 0.23
Leng8Unknown, score: 0.3
Suv420h2Unknown, score: 0.02
Zfp954Unknown, score: 0.15
Zbtb45Unknown, score: 0.12
Ap2s1Unknown, score: 0.07
Bloc1s3Unknown, score: 0.36
Hnrnpul1Unknown, score: -0.01
ItpkcUnknown, score: 0
BlvrbUnknown, score: 0.11
Pak4Unknown, score: 0.08
Rasgrp4Unknown, score: 0.1
Zfp940Unknown, score: 0.27
Arhgap33Unknown, score: 0.16
U2af1l4Unknown, score: -0.01
Kctd15Unknown, score: 0.13
Dpy19l3Unknown, score: 0.28
SiglecfUnknown, score: 0.02
Ctu1Unknown, score: 0.15
Mybpc2Unknown, score: 0.09
Scaf1Unknown, score: 0.01
Mrgprb1Unknown, score: 0.26
Ano5Unknown, score: 0.14
Luzp2Unknown, score: 0.38
PicalmUnknown, score: 0.23
Kctd14Unknown, score: 0
2210018M11RikUnknown, score: 0.07
P2ry6Unknown, score: 0.6
Dchs1Unknown, score: 0.04
Tmem41bUnknown, score: 0.15
Ipo7Unknown, score: 0.06
Galnt18Unknown, score: 0.17
Spon1Unknown, score: 0.09
Smg1Unknown, score: 0.01
Dcun1d3Unknown, score: 0.22
BC030336Unknown, score: 0.36
Vwa3aUnknown, score: 0.18
Cog7Unknown, score: 0.13
Tnrc6aUnknown, score: -0.01
Slc5a11Unknown, score: 0.03
D430042O09RikUnknown, score: 0.13
Atxn2lUnknown, score: 0.15
Ino80eUnknown, score: 0.1
Sez6l2Unknown, score: 0.2
Asphd1Unknown, score: 0.23
Zfp553Unknown, score: 0.03
Zfp764Unknown, score: 0.31
Gm166Unknown, score: 0.02
BC017158Unknown, score: 0.27
Tpcn2Unknown, score: 0.37
Zfp958Unknown, score: 0.34
Arglu1Unknown, score: 0.19
Pcid2Unknown, score: 0.42
Tmco3Unknown, score: 0.04
Erich1Unknown, score: 0.07
Tti2Unknown, score: 0.19
Mboat4Unknown, score: 0.07
234159Unknown, score: 0.17
Neil3Unknown, score: 0.53
Gpm6aUnknown, score: 0.06
Cbr4Unknown, score: 0.04
Psd3Unknown, score: 0.02
Csgalnact1Unknown, score: 0.11
Zfp930Unknown, score: -0.01
Zfp868Unknown, score: 0.34
Tmem161aUnknown, score: 0.24
Sugp2Unknown, score: 0.01
Klhl26Unknown, score: 0.14
Mpv17l2Unknown, score: 0.2
Ccdc124Unknown, score: 0.28
Ankle1Unknown, score: 0.09
Nxnl1Unknown, score: 0.01
Colgalt1Unknown, score: 0.32
Zfp961Unknown, score: 0.25
Cib3Unknown, score: 0.09
Inpp4bUnknown, score: 0.23
Heatr3Unknown, score: 0.18
Cpne2Unknown, score: -0.01
Ndrg4Unknown, score: 0.09
Cnot1Unknown, score: 0.35
Dync1li2Unknown, score: 0.28
Nae1Unknown, score: 0.14
Ces2bUnknown, score: 0.18
Elmo3Unknown, score: 0.07
Lrrc29Unknown, score: 0.24
Fhod1Unknown, score: 0.1
Edc4Unknown, score: 0.04
Nrn1lUnknown, score: 0.01
Txnl4bUnknown, score: 0.17
TatUnknown, score: 0.14
Cmtr2Unknown, score: 0.18
Vac14Unknown, score: -0.01
FukUnknown, score: 0.04
AarsUnknown, score: 0.28
Rfwd3Unknown, score: 0.12
Plcg2Unknown, score: 0.06
MthfsdUnknown, score: 0.17
Klhdc4Unknown, score: 0.14
Piezo1Unknown, score: 0.4
Spg7Unknown, score: 0
Nup133Unknown, score: 0.13
Ttc13Unknown, score: 0.13
9230110C19RikUnknown, score: 0.54
Cep126Unknown, score: 0.05
Med17Unknown, score: -0.01
Mbd3l2Unknown, score: 0.17
Zfp426Unknown, score: 0.24
PpanUnknown, score: 0.07
Kank2Unknown, score: 0.48
Tmem205Unknown, score: 0.1
Lppr2Unknown, score: 0.09
Zfp809Unknown, score: 0.15
Zfp599Unknown, score: 0.51
Zfp810Unknown, score: 0.16
Igsf9bUnknown, score: 0.27
NtmUnknown, score: 0.17
Zbtb44Unknown, score: 0.12
NfrkbUnknown, score: 0.06
Tmem45bUnknown, score: 0.31
Fez1Unknown, score: 0.17
Scn3bUnknown, score: 0.21
Sc5dUnknown, score: 0.77
Zbtb16Unknown, score: 0.21
Usp28Unknown, score: 0.09
Ttc12Unknown, score: 0.12
GldnUnknown, score: 0.14
Lingo1Unknown, score: 0.01
Herc1Unknown, score: 0.09
Rab8bUnknown, score: 0.06
Fam63bUnknown, score: 0.16
Zfp280dUnknown, score: 0.1
Fam214aUnknown, score: 0.14
Slc17a5Unknown, score: -0.01
Plscr4Unknown, score: 0.03
Pxylp1Unknown, score: 0.15
Dnajc13Unknown, score: 0.22
GlyctkUnknown, score: 0.29
Dusp7Unknown, score: 0.04
Parp3Unknown, score: 0.19
6430571L13RikUnknown, score: 0.05
ApehUnknown, score: -0.01
AtripUnknown, score: 0.14
ScapUnknown, score: 0.17
Als2clUnknown, score: 0.2
Rtp3Unknown, score: 0.27
Dync1li1Unknown, score: 0.32
Zfp445Unknown, score: 0.1
Zfp65Unknown, score: 0.12
DHRSXUnknown, score: 0.18
Alms1Unknown, score: 0.18
Spry3Unknown, score: 0.1
Sytl5Unknown, score: 0.28
Slc9a7Unknown, score: 0.05
Rbm10Unknown, score: 0.05
Ddx26bUnknown, score: 0.15
Slc9a6Unknown, score: -0.01
Pdk3Unknown, score: 0.05
Klhl15Unknown, score: 0.17
Stard8Unknown, score: 0.01
Ercc6lUnknown, score: 0.27
Klhl4Unknown, score: 0.19
Nox1Unknown, score: 0.1
Rbm41Unknown, score: 0.16
Adgrg2Unknown, score: 0.48
FancbUnknown, score: 0.06
Gemin8Unknown, score: 0.41
Ofd1Unknown, score: 0.59
Lrp11Unknown, score: -0.01
Il22ra2Unknown, score: 0.08
Sh3rf3Unknown, score: 0.02
Lingo3Unknown, score: 0.08
Zfp938Unknown, score: 0.19
Cdk17Unknown, score: 0.11
Osbpl8Unknown, score: 0.1
MyrflUnknown, score: 0.37
Ankrd52Unknown, score: 0.14
Npc1l1Unknown, score: 0.09
Eml6Unknown, score: 0.07
Gpr75Unknown, score: 0.31
Zfp454Unknown, score: 0.22
Col23a1Unknown, score: 0.26
SowahaUnknown, score: 0.21
PfasUnknown, score: 0.02
Rtn4rl1Unknown, score: 0.29
Tusc5Unknown, score: 0.14
Atad5Unknown, score: 0
Brip1Unknown, score: 0.05
HexdcUnknown, score: 0.3
Fn3krpUnknown, score: 0.2
BC068281Unknown, score: 0.19
Cog5Unknown, score: 0.07
Akap6Unknown, score: 0.16
Arid4aUnknown, score: 0
Gpr135Unknown, score: 0.11
Tmem30bUnknown, score: 0.41
Akap5Unknown, score: 0.07
Gpr68Unknown, score: 0.07
MtrUnknown, score: 0.61
Mylk4Unknown, score: 0.08
Zfp367Unknown, score: 0.1
Zfp458Unknown, score: 0.35
Zfp874aUnknown, score: 0.07
Zfp58Unknown, score: 0.17
Zfp72Unknown, score: 0.1
Zfp366Unknown, score: 0.03
GaptUnknown, score: 0.21
Cdc20bUnknown, score: 0.36
Erc2Unknown, score: 0.38
Arhgap22Unknown, score: 0.13
Tlr11Unknown, score: 0.1
Cdh24Unknown, score: 0.11
Zfhx2Unknown, score: 0.1
Setdb2Unknown, score: 0.2
Pnma2Unknown, score: 0.19
Enox1Unknown, score: 0.21
Plcxd3Unknown, score: 0.15
Rspo2Unknown, score: 0.02
Colec10Unknown, score: 0.02
Ago2Unknown, score: 0.15
Apol8Unknown, score: 0.38
Mief1Unknown, score: -0.01
A4galtUnknown, score: 0.05
Slc2a13Unknown, score: 0.1
Pced1bUnknown, score: 0.18
Ccdc184Unknown, score: 0.21
Zfp641Unknown, score: 0.21
Mkl2Unknown, score: 0.21
Rimbp3Unknown, score: 0.2
LiphUnknown, score: 0.06
OstnUnknown, score: 0.41
PigzUnknown, score: -0.01
LmlnUnknown, score: 0.21
Cd200r4Unknown, score: 0.17
LnpepUnknown, score: 0.2
Zfp760Unknown, score: 0.49
Mmp25Unknown, score: 0.05
Zfp811Unknown, score: 0.1
Zfp563Unknown, score: 0.13
H2-M5Unknown, score: 0.13
Fsd1Unknown, score: 0.47
Lrrc30Unknown, score: 0.17
9430020K01RikUnknown, score: 0.19
Ythdc2Unknown, score: 0.21
Adamts19Unknown, score: 0
Gm4951Unknown, score: 0.08
Slc6a7Unknown, score: 0.07
Zfp407Unknown, score: 0.05
Peli3Unknown, score: 0.09
Ranbp6Unknown, score: 0.13
Slc16a12Unknown, score: 0.02
Slc35g1Unknown, score: 0.04
CcnjUnknown, score: -0.01
St18Unknown, score: 0
Slco5a1Unknown, score: 0.18
Pik3c2bUnknown, score: 0.09
Plekha6Unknown, score: 0.01
Lax1Unknown, score: 0
Kcnt2Unknown, score: 0.14
Tnfsf18Unknown, score: 0.05
Mettl11bUnknown, score: 0
Gpr161Unknown, score: 0.29
Vsig8Unknown, score: 0.11
Pgap1Unknown, score: 0.13
CarfUnknown, score: 0.12
CatipUnknown, score: 0.18
Ccdc108Unknown, score: 0.14
Asic4Unknown, score: 0.06
Fam124bUnknown, score: 0.01
Ankmy1Unknown, score: 0.01
Gpr158Unknown, score: 0.08
Fam78aUnknown, score: 0.52
Ralgps1Unknown, score: 0.17
Zbtb34Unknown, score: 0.09
Crb2Unknown, score: 0.07
Olfml2aUnknown, score: 0.03
Dhrs9Unknown, score: 0.04
Rbm45Unknown, score: 0.28
Zfp385bUnknown, score: 0.23
Ypel4Unknown, score: 0.36
Harbi1Unknown, score: 0.15
Tspan18Unknown, score: 0.01
Lrrc4cUnknown, score: 0.18
Ldlrad3Unknown, score: 0.02
D430041D05RikUnknown, score: 0.08
Slc5a12Unknown, score: 0.4
Exd1Unknown, score: 0.08
Lzts3Unknown, score: 0.1
Pak7Unknown, score: 0.18
Dzank1Unknown, score: -0.01
SnphUnknown, score: -0.01
Rims4Unknown, score: -0.01
Kcng1Unknown, score: 0.24
Phc3Unknown, score: 0.43
Slc7a14Unknown, score: 0.04
Pabpc4lUnknown, score: 0.46
Ppm1lUnknown, score: 0.16
Rxfp4Unknown, score: 0.38
Zfp697Unknown, score: -0.01
Pde5aUnknown, score: 0.27
Wdr63Unknown, score: 0.07
Atp6v0d2Unknown, score: 0
ManeaUnknown, score: 0.16
Pm20d2Unknown, score: 0.01
Rgp1Unknown, score: 0.23
Dcaf10Unknown, score: 0.03
Zfp462Unknown, score: 0.18
Tmem245Unknown, score: 0.24
D630039A03RikUnknown, score: 0.21
Kank4Unknown, score: 0.21
Atg4cUnknown, score: 0.02
Raver2Unknown, score: 0.14
Wdr78Unknown, score: 0.21
1700024P16RikUnknown, score: 0.36
Slc1a7Unknown, score: 0.15
Dmrta2Unknown, score: 0.18
Tctex1d4Unknown, score: 0.01
Rab42Unknown, score: 0.03
Wasf2Unknown, score: 0.14
Gpatch3Unknown, score: 0.08
Ifnlr1Unknown, score: 0.5
E2f2Unknown, score: 0.24
Klhdc7aUnknown, score: 0.12
Pramef8Unknown, score: 0.13
Zfp933Unknown, score: 0
Ttc34Unknown, score: 0.03
Lrrd1Unknown, score: 0.28
NapepldUnknown, score: -0.01
Cct8l1Unknown, score: 0.63
GaremlUnknown, score: 0.27
CpzUnknown, score: 0.15
TecrlUnknown, score: 0.14
Hsd17b13Unknown, score: 0.1
Mfsd7aUnknown, score: 0.07
2900026A02RikUnknown, score: 0.04
Hcar1Unknown, score: 0.05
Nyap1Unknown, score: 0.3
243302Unknown, score: 0.22
Slc29a4Unknown, score: 0.35
Stard13Unknown, score: 0.08
Zfp775Unknown, score: 0.19
Ppm1kUnknown, score: 0.06
Ccdc142Unknown, score: 0.05
H1fxUnknown, score: 0.15
Uroc1Unknown, score: 0.08
Ccdc37Unknown, score: 0.65
Prickle2Unknown, score: 0.07
Ssu2Unknown, score: 0.11
Clec1aUnknown, score: 0.02
Klre1Unknown, score: 0.16
Styk1Unknown, score: 0.32
Plxna4Unknown, score: -0.01
2010107G12RikUnknown, score: 0.22
Parp12Unknown, score: 0.06
Leng9Unknown, score: 0.01
Ppp6r1Unknown, score: 0.3
Gltscr1Unknown, score: -0.01
Mill2Unknown, score: 0.08
NfkbidUnknown, score: 0.15
Zfp536Unknown, score: 0.12
SiglecgUnknown, score: 0.26
Ntn5Unknown, score: 0.02
RgmaUnknown, score: 0.17
244071Unknown, score: -0.01
Fsd2Unknown, score: 0.33
Olfml1Unknown, score: 0.82
Zfp771Unknown, score: 0
Dlgap2Unknown, score: 0.15
Mcph1Unknown, score: 0.13
Erlin2Unknown, score: 0.14
Ppp1r3bUnknown, score: 0.4
D8Ertd82eUnknown, score: -0.01
Elmod2Unknown, score: -0.01
Nanos3Unknown, score: 0
Rpgrip1lUnknown, score: 0.01
SprtnUnknown, score: 0.22
Disc1Unknown, score: 0.11
Sipa1l2Unknown, score: 0.28
Cntn5Unknown, score: -0.01
Hephl1Unknown, score: 0.07
Zfp317Unknown, score: 0.02
Olfm2Unknown, score: -0.01
AW551984Unknown, score: 0.05
Nxpe4Unknown, score: 0.1
LaynUnknown, score: 0.11
NpatUnknown, score: -0.01
AI118078Unknown, score: 0.2
Peak1Unknown, score: 0.02
Mrap2Unknown, score: 0.03
Snx14Unknown, score: 0.02
Slc35g2Unknown, score: 0.4
Col6a6Unknown, score: 0.18
RetnlgUnknown, score: 0.12
Zfp300Unknown, score: 0.28
Slitrk4Unknown, score: 0.14
Pdzd4Unknown, score: 0.35
Dkc1Unknown, score: -0.01
Awat2Unknown, score: 0.04
Gm614Unknown, score: 0.08
C77370Unknown, score: 0.04
HdxUnknown, score: 0.34
Gprasp2Unknown, score: 0.1
Tbc1d8bUnknown, score: 0.7
Iqsec2Unknown, score: 0.34
Klhl34Unknown, score: 0.04
Rbbp7Unknown, score: 0.12
TceancUnknown, score: 0.19
Fat2Unknown, score: 0.1
Trappc1Unknown, score: 0.12
Spag4Unknown, score: 0.16
Ift52Unknown, score: 0.21
Wasf3Unknown, score: 0.02
Ankrd27Unknown, score: 0.26
Rbm47Unknown, score: 0.47
Rhbdl3Unknown, score: 0
OtoaUnknown, score: 0.21
Zfp277Unknown, score: 0.01
Vwa1Unknown, score: 0.11
Fcgr4Unknown, score: 0.33
Ovca2Unknown, score: 0.31
CsadUnknown, score: 0.12
Cd207Unknown, score: 0.11
Klhl8Unknown, score: 0.12
Lgi2Unknown, score: 0.15
Neto1Unknown, score: 0.18
Hps5Unknown, score: 0.16
Slc25a28Unknown, score: 0.09
Defb19Unknown, score: 0.22
Rgs13Unknown, score: 0.42
Cd300lfUnknown, score: 0.12
Il27Unknown, score: 0.04
Atpaf2Unknown, score: 0.18
Trpv3Unknown, score: 0.07
MiosUnknown, score: 0.06
Gin1Unknown, score: 0.22
Cables2Unknown, score: 0
Acsf3Unknown, score: 0.09
Olfr544Unknown, score: 0.08
Olfr212Unknown, score: 0.14
Olfr877Unknown, score: 0.06
Olfr1392Unknown, score: 0.17
Olfr1393Unknown, score: 0.05
Olfr873Unknown, score: 0.16
Olfr1033Unknown, score: 0.02
Olfr39Unknown, score: 0.44
Olfr457Unknown, score: 0.14
Olfr691Unknown, score: 0.06
Olfr520Unknown, score: 0
Olfr558Unknown, score: 0.2
Olfr613Unknown, score: 0.13
Olfr550Unknown, score: 0.1
Klk8Unknown, score: 0.04
Srgap3Unknown, score: 0.03
FevUnknown, score: 0.02
Nav3Unknown, score: 0.51
Cdc42ep3Unknown, score: 0.02
Ttc26Unknown, score: 0.72
Acsf2Unknown, score: 0
Cpne1Unknown, score: 0.02
LgsnUnknown, score: 0.16
Snx17Unknown, score: 0.18
ShprhUnknown, score: 0.05
Kcnc2Unknown, score: 0.09
Fam19a2Unknown, score: 0.22
PpiaUnknown, score: 0.15
Ahsa2Unknown, score: 0.14
Ankrd13bUnknown, score: 0.02
Phf12Unknown, score: 0.19
Eme1Unknown, score: 0.04
Zfp652Unknown, score: 0.1
Rapgefl1Unknown, score: 0.5
Lsm12Unknown, score: 0.17
Gm1564Unknown, score: 0.28
Mgat5bUnknown, score: 0.02
Zbtb1Unknown, score: 0.28
GphnUnknown, score: 0.07
Tmem229bUnknown, score: 0.18
Sptlc1Unknown, score: 0.24
Ccnb1Unknown, score: 0.07
Fam107aUnknown, score: 0.11
Tox4Unknown, score: 0.78
Lrrc16bUnknown, score: 0.06
Wdfy2Unknown, score: 0.18
GuloUnknown, score: 0.21
9930012K11RikUnknown, score: 0.11
EgflamUnknown, score: 0.05
Mtmr12Unknown, score: -0.01
Adck5Unknown, score: 0.17
Nlrc3Unknown, score: 0.21
Rbfox1Unknown, score: 0.27
AbatUnknown, score: 0.27
Stfa2l1Unknown, score: 0.13
LsampUnknown, score: 0.16
Robo2Unknown, score: 0.2
Capn11Unknown, score: 0.01
StrnUnknown, score: 0.19
Ss18Unknown, score: 0.11
Sap130Unknown, score: 0.16
Sh3rf2Unknown, score: 0.19
Stk32aUnknown, score: 0.44
CtifUnknown, score: 0.05
DaglaUnknown, score: 0.28
Colgalt2Unknown, score: 0.4
Mgat4aUnknown, score: 0.07
SetxUnknown, score: 0.07
Muc15Unknown, score: 0
Ccdc32Unknown, score: 0.31
Vps39Unknown, score: 0.04
Ell3Unknown, score: 0.39
Slc4a11Unknown, score: 0.07
AhcyUnknown, score: 0.17
Ss18l1Unknown, score: 0.02
Rtel1Unknown, score: 0.11
Znf512bUnknown, score: 0.16
3110057O12RikUnknown, score: -0.01
Jade1Unknown, score: 0
Nkain3Unknown, score: 0.17
Fbxl4Unknown, score: 0.03
Fbxo10Unknown, score: 0.16
Epb4.1Unknown, score: -0.01
Luzp1Unknown, score: 0.14
Chd5Unknown, score: 0.19
Plch2Unknown, score: 0.2
Rbm48Unknown, score: 0.01
Wdr86Unknown, score: 0.16
Nat8lUnknown, score: -0.01
Rnft2Unknown, score: -0.01
Gm15800Unknown, score: 0.22
269701Unknown, score: 0.13
Mphosph9Unknown, score: 0
Clec4a1Unknown, score: 0.09
Ssc5dUnknown, score: 0.13
269881Unknown, score: 0.06
Chsy1Unknown, score: 0.47
Gdpgp1Unknown, score: 0.08
Gsg1lUnknown, score: 0.09
Orai3Unknown, score: 0.31
Slc35e1Unknown, score: 0.29
Lpcat2Unknown, score: 0.14
Ogfod1Unknown, score: 0.16
Mon1bUnknown, score: 0.1
Rpl13Unknown, score: 0.23
Irf2bp2Unknown, score: 0.06
Maml2Unknown, score: 0.11
Amica1Unknown, score: 0.22
AU019823Unknown, score: -0.01
Elmod1Unknown, score: 0.15
Myo9aUnknown, score: 0.27
ClpxUnknown, score: 0.01
Rab6bUnknown, score: 0.05
Prr11Unknown, score: 0.41
Klhdc1Unknown, score: 0.23
5031414D18RikUnknown, score: 0.33
Phf21bUnknown, score: 0.24
Zbtb11Unknown, score: 0.32
Ip6k3Unknown, score: 0.12
Rab5aUnknown, score: 0.15
Vps13aUnknown, score: 0.02
Adcy10Unknown, score: 0.17
Cdk15Unknown, score: 0.02
Tmem169Unknown, score: 0.6
Agbl2Unknown, score: 0.04
Pla2g4fUnknown, score: 0.14
Shc4Unknown, score: 0.15
TbckUnknown, score: 0.21
Tstd2Unknown, score: 0.18
Zfp398Unknown, score: 0.06
Irf2bp1Unknown, score: 0.28
Lrrc4bUnknown, score: 0.05
SpibUnknown, score: 0.3
Tarsl2Unknown, score: 0.13
Tmem255bUnknown, score: 0.35
Tango6Unknown, score: 0.09
TbcelUnknown, score: 0.18
Smtnl2Unknown, score: -0.01
PigsUnknown, score: 0.16
D11Wsu47eUnknown, score: 0.35
Armc7Unknown, score: 0.09
Trpa1Unknown, score: 0.03
Klhl23Unknown, score: 0.18
Gm694Unknown, score: 0.24
Cyp4a12aUnknown, score: 0.07
C2cd3Unknown, score: 0.06
Slc9a5Unknown, score: 0.04
Spaca5Unknown, score: 0.08
Spin2cUnknown, score: 0.1
Tmtc2Unknown, score: 0.08
Zfp385cUnknown, score: 0.2
Lrrc10bUnknown, score: 0.12
Adamts13Unknown, score: 0.04
279067Unknown, score: 0
Rhbdd3Unknown, score: 0.22
RilpUnknown, score: 0.46
Lix1lUnknown, score: -0.01
Emilin3Unknown, score: 0.09
B3gat2Unknown, score: 0.09
AfmUnknown, score: 0.21
Adam1bUnknown, score: 0.35
Kif19aUnknown, score: 0.14
Klk14Unknown, score: 0.01
C1s2Unknown, score: -0.01
Sec22aUnknown, score: 0.11
Slc24a5Unknown, score: 0
Gimap5Unknown, score: 0.1
Gimap9Unknown, score: 0.02
Hist1h3cUnknown, score: 0.02
Hist1h3dUnknown, score: 0.02
Hist1h3bUnknown, score: 0.02
Hist1h3iUnknown, score: 0.02
Hist1h4nUnknown, score: 0.36
Hist1h2bnUnknown, score: 0.01
Hist2h2bbUnknown, score: 0.56
Hist1h2aiUnknown, score: 0.05
SctrUnknown, score: 0.26
Fchsd1Unknown, score: 0.07
A230046K03RikUnknown, score: 0.2
Snhg11Unknown, score: 0.11
Sf3b2Unknown, score: 0.21
Ubald2Unknown, score: 0.01
Adgrl3Unknown, score: 0.18
Hs3st5Unknown, score: 0.12
Fndc3aUnknown, score: 0.4
6030419C18RikUnknown, score: 0.06
Itga11Unknown, score: 0.13
9530053A07RikUnknown, score: 0
NrcamUnknown, score: 0.08
Zfp750Unknown, score: 0.04
Zfp182Unknown, score: 0.08
B230216G23RikUnknown, score: 0.07
Idi1Unknown, score: 0.18
Nwd1Unknown, score: 0.04
Syne2Unknown, score: 0.01
Xkr5Unknown, score: 0.02
Hif1anUnknown, score: 0.09
Fam168aUnknown, score: 0.29
SybuUnknown, score: 0.01
Dcp1bUnknown, score: 0.09
GalmUnknown, score: 0.14
Fsd1lUnknown, score: 0.04
Nt5dc1Unknown, score: -0.01
Rab9bUnknown, score: 0.09
Slc25a40Unknown, score: 0.17
Fbxo48Unknown, score: 0.07
Cacna2d4Unknown, score: 0.06
Zfp865Unknown, score: 0.06
C130050O18RikUnknown, score: 0.14
Rc3h2Unknown, score: 0.11
Bbs9Unknown, score: 0.27
Tmcc3Unknown, score: 0.02
DselUnknown, score: 0.04
Ism1Unknown, score: 0.05
Apba1Unknown, score: 0.18
Tns3Unknown, score: 0.42
Taf2Unknown, score: 0
Ercc6Unknown, score: 0.01
Cc2d1bUnknown, score: 0.45
319974Unknown, score: 0.06
Kif6Unknown, score: 0.23
Tmem198Unknown, score: 0.13
Uggt1Unknown, score: 0.1
Ccdc79Unknown, score: 0.17
Fstl4Unknown, score: 0.13
Zbtb39Unknown, score: 0.12
Ano4Unknown, score: 0.15
ReltUnknown, score: -0.01
Fbxl13Unknown, score: 0.14
Rps6kc1Unknown, score: 0.16
Fam179aUnknown, score: 0.3
Cep95Unknown, score: 0.11
Pik3r5Unknown, score: 0.2
Senp5Unknown, score: 0.09
Maats1Unknown, score: 0
Ccdc171Unknown, score: 0.17
Rasgef1bUnknown, score: 0.29
Bcorl1Unknown, score: 0.12
Lrig3Unknown, score: 0
Cadps2Unknown, score: 0.12
Klri2Unknown, score: 0.27
RinlUnknown, score: 0.37
Ppm1eUnknown, score: 0.04
Heatr5bUnknown, score: -0.01
Rasal3Unknown, score: 0.15
Heatr5aUnknown, score: 0.08
A830018L16RikUnknown, score: 0.1
Ipcef1Unknown, score: 0.1
Tmem215Unknown, score: 0.47
Tmem104Unknown, score: 0.02
Ubn2Unknown, score: -0.01
Slc35e2Unknown, score: 0
Tcp11l1Unknown, score: 0.15
Fam169aUnknown, score: 0.44
Islr2Unknown, score: 0.02
SvoplUnknown, score: 0.06
Dopey1Unknown, score: 0.07
OcrlUnknown, score: 0.54
Samd12Unknown, score: 0.03
Zfp629Unknown, score: 0.44
Soga1Unknown, score: 0.17
Mysm1Unknown, score: 0.03
Pptc7Unknown, score: 0.37
Slc26a9Unknown, score: 0.33
Fastkd1Unknown, score: -0.01
Ipo8Unknown, score: 0.04
Vstm4Unknown, score: 0.31
Pkn1Unknown, score: 0.1
Ifitm10Unknown, score: 0
Gfm2Unknown, score: 0.11
Dcaf5Unknown, score: 0.17
Atad2bUnknown, score: 0.05
C530008M17RikUnknown, score: 0.44
Amigo3Unknown, score: 0.41
Spata33Unknown, score: 0.06
Mtap7d3Unknown, score: 0.47
Atp11cUnknown, score: 0.24
PisdUnknown, score: 0.03
Lrrn4Unknown, score: 0.24
Enpp6Unknown, score: 0.1
Arl4cUnknown, score: 0.09
Lrif1Unknown, score: 0.09
VprbpUnknown, score: 0.1
6330408A02RikUnknown, score: 0.04
Hist1h4bUnknown, score: 0.16
Ubtd2Unknown, score: 0.42
Cyb5d1Unknown, score: 0.03
Pitpnm3Unknown, score: 0.04
Slfn5Unknown, score: 0.03
Med13Unknown, score: 0.07
Fads6Unknown, score: 0.12
Fam179bUnknown, score: -0.01
Prpf39Unknown, score: 0.02
Gfod1Unknown, score: 0.15
Sh2d4bUnknown, score: 0.28
KcnrgUnknown, score: 0.03
Npm2Unknown, score: 0.02
Ep300Unknown, score: 0.06
Vwa5b2Unknown, score: 0.02
MslnlUnknown, score: 0.02
Ubash3aUnknown, score: 0.3
MccUnknown, score: 0.13
Zfp532Unknown, score: -0.01
Zfp516Unknown, score: 0.2
Pkd2l1Unknown, score: 0.46
Scd4Unknown, score: 0.08
Hecw2Unknown, score: 0.09
Unc80Unknown, score: 0.02
Ppp1r12bUnknown, score: 0.23
Lgr6Unknown, score: 0.22
Myo3bUnknown, score: 0.05
AccsUnknown, score: 0.09
Ctdspl2Unknown, score: 0.01
1810024B03RikUnknown, score: 0.05
Nol4lUnknown, score: 0.38
Fat4Unknown, score: 0.12
E130311K13RikUnknown, score: 0.11
Dennd2cUnknown, score: 0.16
Fam19a3Unknown, score: 0.04
Fam166bUnknown, score: 0.04
Dennd4cUnknown, score: 0.11
Col8a2Unknown, score: 0.23
Catsper4Unknown, score: 0.03
Fhad1Unknown, score: 0.17
Fam185aUnknown, score: 0.12
Slc5a6Unknown, score: 0.3
Taok3Unknown, score: 0.33
Ccdc63Unknown, score: 0.06
Vps37bUnknown, score: 0.03
D630045J12RikUnknown, score: 0.24
Zfp786Unknown, score: 0.59
Fam188bUnknown, score: 0.21
Dnah6Unknown, score: 0.11
Gcfc2Unknown, score: 0.04
B4galnt3Unknown, score: 0.37
Cecr2Unknown, score: 0.31
Far2Unknown, score: 0.07
Tmem150bUnknown, score: 0.29
Ceacam16Unknown, score: -0.01
Gm5113Unknown, score: 0.29
Fan1Unknown, score: 0.19
Prss53Unknown, score: 0.06
Dock1Unknown, score: 0.16
B4galnt4Unknown, score: 0.08
Htra4Unknown, score: 0.41
Hapln4Unknown, score: 0.08
Adgrl1Unknown, score: 0.17
Slc7a6Unknown, score: 0.02
Piwil4Unknown, score: 0.33
OpcmlUnknown, score: 0.03
Hcn4Unknown, score: 0.11
GmppbUnknown, score: 0.06
331374Unknown, score: 0.11
UprtUnknown, score: 0.16
XkrxUnknown, score: 0.16
Tceal5Unknown, score: 0.28
Bend3Unknown, score: 0.02
Mapk15Unknown, score: 0.09
Krt78Unknown, score: 0.09
Card9Unknown, score: 0.22
KcpUnknown, score: 0.68
Cox6b2Unknown, score: 0.2
Cngb1Unknown, score: 0.47
Gpd1lUnknown, score: 0.09
N4bp2Unknown, score: 0.2
Cd59bUnknown, score: 0.18
Cog3Unknown, score: 0.09
CntlnUnknown, score: 0.22
Acad12Unknown, score: 0.06
Akap17bUnknown, score: 0
Zfp780bUnknown, score: 0
Fkbp15Unknown, score: 0.09
UstUnknown, score: 0.16
Slc41a2Unknown, score: 0.12
338366Unknown, score: 0.02
Fam109bUnknown, score: 0.02
Tmem220Unknown, score: 0.03
NalcnUnknown, score: 0
EndovUnknown, score: 0.02
Map3k9Unknown, score: 1
Scgb1c1Unknown, score: 0.04
Morc3Unknown, score: 0.17
Agap1Unknown, score: 0.16
Plekhm1Unknown, score: 0.08
Slc2a12Unknown, score: 0.45
GarsUnknown, score: 0.09
Nr1d2Unknown, score: 0.09
Adam32Unknown, score: 0.07
Edc3Unknown, score: 0.12
Pcdhac2Unknown, score: 0.04
Mrpl21Unknown, score: 0.11
Ltv1Unknown, score: 0
Zfp703Unknown, score: 0.28
Gpr141Unknown, score: 0.15
Tmc4Unknown, score: 0.09
Hcfc1r1Unknown, score: 0.33
Trim46Unknown, score: 0.09
Nlrp12Unknown, score: 0.11
Nanos2Unknown, score: 0.25
MafaUnknown, score: 0.04
Pram1Unknown, score: 0.18
Morn2Unknown, score: 0.01
Bpifb3Unknown, score: 0.3
Serf2Unknown, score: 0.2
Fastkd5Unknown, score: 0.1
Tagap1Unknown, score: 0.4
CcnjlUnknown, score: 0.09
Tmem102Unknown, score: 0.14
Rap1gap2Unknown, score: 0.33
Tlcd2Unknown, score: 0
Rph3alUnknown, score: 0.25
Tssc1Unknown, score: 0.24
Atxn7l1Unknown, score: 0.72
Lsmem1Unknown, score: 0.08
SlirpUnknown, score: 0.13
Stmnd1Unknown, score: -0.01
Gm5141Unknown, score: 0.16
Rsl1Unknown, score: 0.7
Tmem171Unknown, score: 0.17
Olfm4Unknown, score: 0.13
ParlUnknown, score: 0.06
Ccdc58Unknown, score: 0.1
NpwUnknown, score: -0.01
Ccdc78Unknown, score: 0.02
Dnph1Unknown, score: 0.05
Tmem232Unknown, score: 0.3
Rmdn2Unknown, score: 0.06
Arhgef33Unknown, score: 0.01
Cdkl4Unknown, score: 0.12
GaremUnknown, score: 0.18
Greb1lUnknown, score: 0
Ap5b1Unknown, score: 0.07
Naaladl1Unknown, score: 0.01
4430402I18RikUnknown, score: 0.01
Cfap58Unknown, score: 0.23
Gm973Unknown, score: 0.27
MregUnknown, score: 0.19
MndaUnknown, score: 0.19
6330403A02RikUnknown, score: 0.21
Iars2Unknown, score: 0.04
Batf3Unknown, score: 0.11
Fam178bUnknown, score: 0.3
Lonrf2Unknown, score: 0.25
Tmem182Unknown, score: 0
Gm996Unknown, score: 0.09
Cacfd1Unknown, score: 0.17
Med19Unknown, score: 0.06
Bpifb4Unknown, score: 0.12
Pabpc1lUnknown, score: 0.05
2810408M09RikUnknown, score: 0.1
Cdh26Unknown, score: 0.07
Gm14085Unknown, score: 0
Gm5150Unknown, score: 0.11
Trim55Unknown, score: -0.01
Dpy19l4Unknown, score: 0.19
Pdp1Unknown, score: 0.22
Mup20Unknown, score: 0.18
Mup21Unknown, score: 0.15
Ube2uUnknown, score: 0.33
Ccdc24Unknown, score: 0.07
Zfp69Unknown, score: 0.09
Tmem240Unknown, score: 0.07
Adgrf3Unknown, score: 0.16
AtraidUnknown, score: 0.24
Cep135Unknown, score: 0
VgfUnknown, score: 0.34
Wdr95Unknown, score: 0.21
Drc1Unknown, score: 0.23
Ssbp1Unknown, score: 0.05
Tatdn2Unknown, score: 0.32
Lpar5Unknown, score: 0.2
Cracr2aUnknown, score: 0.12
Apold1Unknown, score: 0.33
Sbk2Unknown, score: 0.04
2310014L17RikUnknown, score: 0.08
Alg8Unknown, score: 0.08
Taok2Unknown, score: -0.01
Brsk1Unknown, score: 0.01
Zbtb2Unknown, score: 0.12
Ano8Unknown, score: 0.66
Zfp882Unknown, score: -0.01
Gse1Unknown, score: -0.01
Urb2Unknown, score: 0.14
Adgrg5Unknown, score: 0.27
Pdp2Unknown, score: 0.21
Ces3aUnknown, score: 0.04
AB124611Unknown, score: 0.07
Ccdc84Unknown, score: 0.08
Ccdc33Unknown, score: 0.33
Gm1123Unknown, score: 0
TcaimUnknown, score: 0.29
Fdxacb1Unknown, score: 0.03
Jade3Unknown, score: 0.01
8030474K03RikUnknown, score: 0.05
Tmem29Unknown, score: 0.01
Cdkl5Unknown, score: 0.14
Poc1bUnknown, score: 0.11
Best3Unknown, score: 0.15
Cd300lhUnknown, score: 0.43
Zbtb42Unknown, score: 0.12
Mtx3Unknown, score: 0.76
ColqUnknown, score: 0.24
Zfp488Unknown, score: 0.18
Neil2Unknown, score: 0.15
Tvp23aUnknown, score: 0.23
Ypel5Unknown, score: 0.13
Gpr25Unknown, score: 0
Glipr2Unknown, score: 0.02
Tlr12Unknown, score: 0.15
Fndc5Unknown, score: 0.65
Ephx4Unknown, score: 0.21
GatcUnknown, score: 0.11
Cyp2t4Unknown, score: 0.07
Frmd7Unknown, score: 0.01
Nxpe3Unknown, score: 0.25
Lca5lUnknown, score: 0.25
Zfp174Unknown, score: 0.28
CdsnUnknown, score: 0
Thoc6Unknown, score: 0.23
Nsfl1cUnknown, score: 0.24
Eid2Unknown, score: 0.09
Tas2r135Unknown, score: 0.1
Ugt1a6bUnknown, score: 0.02
Ugt1a1Unknown, score: 0.02
Map4k5Unknown, score: 0.12
Btbd6Unknown, score: 0.13
BC052040Unknown, score: 0.07
Tmsb15lUnknown, score: 0.08
Ccdc87Unknown, score: 0.35
Msantd1Unknown, score: 0.05
Opa3Unknown, score: 0.11
Cyp2c50Unknown, score: 0.11
Iqgap3Unknown, score: 0.39
Panx2Unknown, score: 0.27
Tmem189Unknown, score: 0.06
Ndufs6Unknown, score: 0
Taf9bUnknown, score: 0.07
Ndufa4l2Unknown, score: 0.31
Ecm2Unknown, score: 0.09
Baz2bUnknown, score: 0.09
PrimpolUnknown, score: 0.03
BC049352Unknown, score: 0.03
Zfp873Unknown, score: 0.14
Zfp456Unknown, score: 0.09
Zfp874bUnknown, score: 0.05
Zfp738Unknown, score: 0.21
414077Unknown, score: 0.19
Tnip3Unknown, score: 0
Gja6Unknown, score: 0.25
Cfap43Unknown, score: 0.1
Zyg11bUnknown, score: 0.09
Zfp457Unknown, score: 0.43
Akap7Unknown, score: 0.33
4930404N11RikUnknown, score: -0.01
GnptabUnknown, score: 0.05
Cpsf6Unknown, score: 0.02
Myo1aUnknown, score: 0.76
Specc1Unknown, score: 0.14
E130309D14RikUnknown, score: 0.04
Gm11541Unknown, score: 0.06
Dnaic2Unknown, score: 0.23
Mfsd2bUnknown, score: 0.05
Akr1c19Unknown, score: 0.1
432725Unknown, score: 0.09
Prr7Unknown, score: 0.04
432768Unknown, score: 0.51
Gm28557Unknown, score: 0.22
Rslcan18Unknown, score: 0.14
Lrrc14bUnknown, score: 0.4
432800Unknown, score: 0.1
Gm5464Unknown, score: 0.05
432879Unknown, score: 0.19
432950Unknown, score: 0.11
Plcxd2Unknown, score: 0.11
Vmn2r96Unknown, score: 0.15
Acsl5Unknown, score: 0.05
Rbm10Unknown, score: 0.13
Mettl21cUnknown, score: 0.1
433319Unknown, score: 0.05
Sgpp2Unknown, score: -0.01
Teddm1bUnknown, score: 0.28
AA467197Unknown, score: 0.25
Syndig1Unknown, score: 0.44
Akirin2Unknown, score: 0.12
Gm12429Unknown, score: 0.1
Ncbp1Unknown, score: 0.24
433745Unknown, score: 0.18
Trim63Unknown, score: 0.26
Minos1Unknown, score: 0.03
Gm13251Unknown, score: 0.03
Chchd2Unknown, score: 0.52
Pusl1Unknown, score: 0.2
433874Unknown, score: 0.04
PiggUnknown, score: 0.06
Dnaaf5Unknown, score: 0.04
Pnmal2Unknown, score: 0.01
Ccdc8Unknown, score: 0.29
434175Unknown, score: 0.39
IqckUnknown, score: 0.11
2610020H08RikUnknown, score: 0.22
Zfp560Unknown, score: 0.29
434428Unknown, score: 0.05
Rpl10Unknown, score: -0.01
Sp140Unknown, score: 0.1
Gm10220Unknown, score: 0.18
434693Unknown, score: 0.11
Akap14Unknown, score: 0.09
Ldoc1Unknown, score: 0.22
Xlr4aUnknown, score: 0.11
434843Unknown, score: 0.26
Atp6ap1lUnknown, score: 0.04
ShfUnknown, score: 0.13
Tnni3kUnknown, score: 0.11
Lrp3Unknown, score: 0
Dnaaf3Unknown, score: 0.08
Fam92bUnknown, score: 0.06
Gpr62Unknown, score: 0.04
Rab44Unknown, score: 0
Nup85Unknown, score: 0.16
Xrra1Unknown, score: 0.05
Cbx6Unknown, score: 0.07
Zdhhc18Unknown, score: 0.16
Ear2Unknown, score: 0.1
NpcdUnknown, score: 0.03
Cfap74Unknown, score: 0.25
Tbc1d32Unknown, score: 0.18
Hbq1bUnknown, score: 0.03
Myh13Unknown, score: 0.09
Zkscan4Unknown, score: 0.04
Bdp1Unknown, score: 0.14
Cma2Unknown, score: 0.01
Wdr70Unknown, score: 0.19
Hnrnpa1Unknown, score: 0.03
KalrnUnknown, score: 0.06
Baiap3Unknown, score: 0.03
Gal3st3Unknown, score: 0.25
Cfhr2Unknown, score: 0.22
Cep170Unknown, score: 0.1
545423Unknown, score: 0.64
Ccdc141Unknown, score: 0.02
545459Unknown, score: 0.05
Tubb1Unknown, score: 0.4
545531Unknown, score: 0.37
Ankrd34aUnknown, score: 0.15
545578Unknown, score: 0.36
545600Unknown, score: 0.52
Ptpn3Unknown, score: 0
Gm13290Unknown, score: 0
Gm13289Unknown, score: 0
Gm13277Unknown, score: 0
Gm13276Unknown, score: 0
Fam159aUnknown, score: 0.11
Mansc4Unknown, score: 0.08
Zfp607Unknown, score: 0.15
Cers3Unknown, score: -0.01
CrxosUnknown, score: 0.14
C330021F23RikUnknown, score: 0.4
Wdr72Unknown, score: 0.4
Klhl33Unknown, score: 0.07
546663Unknown, score: 0
Zc3h12bUnknown, score: 0
Gpr17Unknown, score: 0
Fam196bUnknown, score: 0.14
Zmynd15Unknown, score: 0.01
Xlr3bUnknown, score: 0.11
Tceal3Unknown, score: -0.01
Gsk3aUnknown, score: 0.14
Rfx8Unknown, score: 0.09
Tmem253Unknown, score: 0.15
Tnfsfm13Unknown, score: 0.16
Rpl34Unknown, score: 0.18
Defb42Unknown, score: 0.17
Gm6086Unknown, score: 0.36
Zcchc17Unknown, score: -0.01
Klf14Unknown, score: 0.11
C130026I21RikUnknown, score: 0.14
620248Unknown, score: 0.09
620499Unknown, score: 0.02
Tmem28Unknown, score: 0.17
Ttc30a2Unknown, score: 0.19
AI429214Unknown, score: 0.32
621542Unknown, score: 0.03
AC087559.2Unknown, score: 0.2
Aldh3b2Unknown, score: 0.16
Cldn20Unknown, score: 0.28
Psme2Unknown, score: 0.3
Tmem170bUnknown, score: 0.21
Kctd21Unknown, score: 0.19
622335Unknown, score: 0.16
Ccdc107Unknown, score: 0.19
Arhgef26Unknown, score: 0.09
1700123I01RikUnknown, score: 0.24
Ccdc17Unknown, score: 0.41
Gm6377Unknown, score: 0.02
Pydc4Unknown, score: 0.16
Tmem200bUnknown, score: 0.06
Lipt1Unknown, score: 0.18
Speer4eUnknown, score: 0.2
Cks1brtUnknown, score: 0.21
Lekr1Unknown, score: 0.03
Slc38a6Unknown, score: 0.03
Gpx4Unknown, score: 0.11
Tmem236Unknown, score: 0.14
H3f3aUnknown, score: 0
626048Unknown, score: 0.09
Wdr93Unknown, score: 0.09
Rgs22Unknown, score: 0.06
Gm14322Unknown, score: 0.23
Gm6710Unknown, score: 0.03
Etohi1Unknown, score: 0.08
Zfp800Unknown, score: 0.09
Fam196aUnknown, score: 0.16
Morf4l1Unknown, score: 0.14
627371Unknown, score: 0.27
627375Unknown, score: 0.07
Gm6792Unknown, score: 0.01
627901Unknown, score: 0.15
Gm4631Unknown, score: 0.04
628161Unknown, score: 0
Gm14420Unknown, score: 0.04
Phf11cUnknown, score: 0.01
Hs3st4Unknown, score: 0.04
Serpina3iUnknown, score: 0.01
Fam124aUnknown, score: 0.04
Mroh8Unknown, score: 0.3
629595Unknown, score: 0.22
2010315B03RikUnknown, score: 0.07
630855Unknown, score: 0.21
631033Unknown, score: 0.06
631287Unknown, score: 0.43
631966Unknown, score: 0.08
632329Unknown, score: 0.17
632465Unknown, score: 0.05
Vmn2r18Unknown, score: 0.03
Erich4Unknown, score: -0.01
RerglUnknown, score: 0.07
Rbm46Unknown, score: 0.29
Gm21967Unknown, score: 0.1
634339Unknown, score: 0.04
Susd1Unknown, score: 0.12
Usp51Unknown, score: 0.4
635999Unknown, score: 0.1
Nlrp1bUnknown, score: 0.06
637553Unknown, score: 0.21
637733Unknown, score: 0.03
639541Unknown, score: 0.14
639606Unknown, score: 0.14
Skint7Unknown, score: 0.18
639905Unknown, score: 0.17
640549Unknown, score: 0.24
640611Unknown, score: 0.19
640972Unknown, score: 0.19
Nrbf2Unknown, score: 0.16
641387Unknown, score: 0.22
Alkbh1Unknown, score: 0.14
654450Unknown, score: 0.14
Sdr39u1Unknown, score: 0.22
Angptl7Unknown, score: 0.07
Ankrd37Unknown, score: 0.28
Nova1Unknown, score: -0.01
Tmem238Unknown, score: 0.11
Rpl30Unknown, score: 0.19
Gm14393Unknown, score: 0.29
Isoc2aUnknown, score: 0
Col6a5Unknown, score: 0.19
Sec14l5Unknown, score: 0.18
Srp54bUnknown, score: -0.01
Clec2lUnknown, score: 0.37
665181Unknown, score: 0
Plb1Unknown, score: 0.01
665463Unknown, score: 0
665562Unknown, score: 0.15
Frmpd1Unknown, score: 0.07
Vps13bUnknown, score: 0.21
Zscan25Unknown, score: 0.06
Apol7eUnknown, score: 0.14
Tomm5Unknown, score: 0.19
Atg4aUnknown, score: 0.19
666501Unknown, score: 0
666513Unknown, score: 0.1
Gm13139Unknown, score: 0.19
666548Unknown, score: 0.14
Rpl29Unknown, score: 0.4
666648Unknown, score: 0.19
Samd1Unknown, score: 0.17
Trim43cUnknown, score: 0.19
Trim43bUnknown, score: 0.19
Rbm24Unknown, score: 0.04
Pnp2Unknown, score: 0.17
9930111J21Rik1Unknown, score: 0.14
H3f3aUnknown, score: 0.02
667253Unknown, score: 0
H60bUnknown, score: 0.06
I830012O16RikUnknown, score: 0.17
Gm14446Unknown, score: -0.01
667384Unknown, score: 0.3
Zfp600Unknown, score: 0.02
Hist1h2alUnknown, score: 0.31
Piezo2Unknown, score: 0.15
Myh15Unknown, score: 0.1
Trim5Unknown, score: 0.14
667846Unknown, score: 0.14
Gm8898Unknown, score: 0.04
Gm8909Unknown, score: 0.29
Gm14305Unknown, score: 0.15
Gm14434Unknown, score: 0
ZxdbUnknown, score: 0.09
Mettl7a3Unknown, score: 0
Gm13288Unknown, score: 0.29
Efr3bUnknown, score: 0
Kif26aUnknown, score: 0.18
668455Unknown, score: 0.05
668459Unknown, score: 0
Zfp507Unknown, score: 0.22
668548Unknown, score: 0.24
2410002F23RikUnknown, score: 0.3
668894Unknown, score: 0.34
668936Unknown, score: 0.54
670832Unknown, score: 0.27
SmsUnknown, score: 0
Rnf213Unknown, score: 0.43
674321Unknown, score: 0.15
Zfp605Unknown, score: 0.12
676710Unknown, score: 0.12
677113Unknown, score: 0.02
Cyp4f37Unknown, score: 0.04
Gm9733Unknown, score: 0.07
Sap25Unknown, score: 0.16
TomtUnknown, score: 0.03
Zglp1Unknown, score: 0.14
Wfdc17Unknown, score: 0.17
Mfap1bUnknown, score: 0.07
Tmsb15b2Unknown, score: 0.09
Dnajc3Unknown, score: -0.01
Sirpb1cUnknown, score: 0.22
Gm13305Unknown, score: 0.23
Gm10471Unknown, score: 0.05
Ccl19Unknown, score: 0.15
1810037I17RikUnknown, score: 0.2
Mup14Unknown, score: 0.01
Gm14295Unknown, score: 0.01
Plac9aUnknown, score: 0.22
Plac9bUnknown, score: 0.18
Tmem254bUnknown, score: 0.1
100039503Unknown, score: 0.1
100039532Unknown, score: 0.05
100039674Unknown, score: -0.01
Ildr2Unknown, score: 0.11
Tgtp2Unknown, score: 0.32
100039826Unknown, score: 0.09
100040052Unknown, score: 0.01
100040260Unknown, score: 0.19
100040416Unknown, score: 0.16
MndalUnknown, score: 0.14
100040500Unknown, score: 0.22
Dynlt1fUnknown, score: 0.22
Gm15319Unknown, score: 0.18
FancfUnknown, score: 0.24
Gm2897Unknown, score: 0.3
100040682Unknown, score: -0.01
100040697Unknown, score: -0.01
Mroh2aUnknown, score: 0.17
Cyp4a32Unknown, score: 0.07
Gm10408Unknown, score: 0.05
Rpl17Unknown, score: 0.15
100040961Unknown, score: 0.46
Tceal7Unknown, score: 0.19
Gm3194Unknown, score: 0.33
G630090E17RikUnknown, score: 0.33
Gm15448Unknown, score: 0.26
Hist1h4mUnknown, score: 0.14
Supt4aUnknown, score: 0.02
Gm13247Unknown, score: 0.19
100041478Unknown, score: 0.13
Ly6c2Unknown, score: 0.1
ErmardUnknown, score: 0.03
AC087559.2Unknown, score: 0.18
Amd2Unknown, score: 0.22
ErmardUnknown, score: 0.28
Mup7Unknown, score: 0.07
100041680Unknown, score: 0.07
Gm10408Unknown, score: 0.16
Gm3558Unknown, score: 0.01
CsprsUnknown, score: 0.13
Gm10094Unknown, score: 0.13
GapdhUnknown, score: 0.02
Gm3646Unknown, score: -0.01
100042069Unknown, score: 0.1
Nrg2Unknown, score: 0.08
BC005561Unknown, score: 0.15
100042235Unknown, score: 0.01
Gsta1Unknown, score: 0.12
Gm10639Unknown, score: 0.18
100042335Unknown, score: 0.15
Ube2l3Unknown, score: 0.22
Nhsl2Unknown, score: 0.14
Gm13305Unknown, score: 0.19
100042561Unknown, score: 0.27
100042773Unknown, score: 0.21
Gm4070Unknown, score: 0.05
100042880Unknown, score: 0.18
100043002Unknown, score: 0.19
Rex2Unknown, score: 0
Gm11711Unknown, score: 0.11
9130023H24RikUnknown, score: 0.1
100043184Unknown, score: 0.1
100043257Unknown, score: 0.06
TigitUnknown, score: 0.15
100043324Unknown, score: 0.1
Rpl21Unknown, score: 0.15
Gm14308Unknown, score: 0.29
SrcapUnknown, score: 0.01
Rbx1Unknown, score: 0.04
100043695Unknown, score: 0.06
100043718Unknown, score: 0
Josd1Unknown, score: 0.01
100043805Unknown, score: 0.05
100043872Unknown, score: 0.16
Rpl34Unknown, score: 0.06
100043882Unknown, score: 0.15
100044322Unknown, score: -0.01
100044324Unknown, score: 0.11
100044374Unknown, score: 0.02
100044391Unknown, score: 0.15
100044398Unknown, score: 0.42
Tgfbr3lUnknown, score: 0.4
Rpl23Unknown, score: 0.2
100044729Unknown, score: 0.33
100044742Unknown, score: 0.05
100044829Unknown, score: 0.12
100044900Unknown, score: 0.19
100045191Unknown, score: 0.02
100045326Unknown, score: 0.39
Ect2lUnknown, score: 0.11
100045848Unknown, score: 0.15
100045999Unknown, score: 0.1
100046048Unknown, score: 0.12
100046079Unknown, score: -0.01
100046151Unknown, score: 0.31
100046223Unknown, score: 0.06
100046289Unknown, score: 0.18
100046297Unknown, score: 0.22
100046650Unknown, score: -0.01
100046899Unknown, score: 0.36
100047082Unknown, score: 0.24
100047252Unknown, score: 0.2
100047429Unknown, score: 0.09
100047577Unknown, score: 0.08
100047658Unknown, score: 0.03
100048410Unknown, score: 0.17
100048447Unknown, score: 0.25
100048483Unknown, score: 0.17
100048499Unknown, score: 0.17
100048557Unknown, score: 0.11
100048759Unknown, score: 0.28
Mup17Unknown, score: 0.01
Adat3Unknown, score: 0.14
100137011Unknown, score: 0.22
Ubl4Unknown, score: 0.19
Gm3194Unknown, score: 0.22
Mup19Unknown, score: 0.04
Gm14306Unknown, score: 0.12
AK010878Unknown, score: 0.09
Gm14431Unknown, score: 0.22
Dynlt1aUnknown, score: 0.24
Il4i1Unknown, score: 0.04
Zfp729bUnknown, score: 0.15
100502680Unknown, score: 0.29
Rpl37Unknown, score: -0.01
100502897Unknown, score: 0.26
Gm684Unknown, score: 0.09
Klhl3Unknown, score: 0.14
Btbd8Unknown, score: 0.35
100503217Unknown, score: 0.13
Gm14440Unknown, score: 0.07
100503428Unknown, score: 0.01
Gm10354Unknown, score: 0.01
DosUnknown, score: 0.34
Rpl5Unknown, score: 0.21
Dnajc19Unknown, score: 0.27
Gm5901Unknown, score: 0.27
100503895Unknown, score: 0.01
CsprsUnknown, score: 0.04
100504089Unknown, score: 0.06
100504173Unknown, score: 0.1
100504174Unknown, score: 0.15
Ccdc170Unknown, score: 0.01
100504500Unknown, score: 0.29
3425401B19RikUnknown, score: 0
Mettl21bUnknown, score: 0.19
100504821Unknown, score: 0.23
100504863Unknown, score: 0.28
100504872Unknown, score: 0.15
100504959Unknown, score: -0.01
100504983Unknown, score: 0.06
100504988Unknown, score: 0.03
100505031Unknown, score: 0.08
100505089Unknown, score: 0.09
100505237Unknown, score: 0.1
100505283Unknown, score: 0
Schip1Unknown, score: 0.02
Gm20604Unknown, score: 0.06
Gm3411Unknown, score: 0.22
Loxl2Unknown, score: 0
Tmed2Unknown, score: 0
100862223Unknown, score: 0.03
100862401Unknown, score: 0.03
100862437Unknown, score: 0.1
Ftl1Unknown, score: 0.4
Gm5616Unknown, score: 0.01
100862473Unknown, score: -0.01
100862563Unknown, score: 0.08
100862584Unknown, score: 0.04
100862586Unknown, score: 0.14
100862594Unknown, score: 0.1
100862597Unknown, score: 0.04
100862618Unknown, score: 0.28
100862620Unknown, score: 0.05
101055647Unknown, score: -0.01
101055652Unknown, score: 0
101055707Unknown, score: 0.1
101055716Unknown, score: 0.26
101055738Unknown, score: 0.26
Sytl3Unknown, score: 0.33
101055764Unknown, score: 0.1
101055794Unknown, score: 0.41
101055802Unknown, score: 0.14
101055854Unknown, score: 0.19
101055915Unknown, score: 0.07
101055925Unknown, score: 0.26
101055956Unknown, score: 0.09
101055995Unknown, score: 0.34
101055997Unknown, score: 0.18
101056016Unknown, score: 0.13
101056061Unknown, score: 0.13
101056131Unknown, score: 0.05
101056167Unknown, score: 0.07
101056341Unknown, score: 0.14
101056362Unknown, score: 0.12
101056365Unknown, score: 0.37
101056381Unknown, score: 0.1
101056392Unknown, score: 0.1
101056447Unknown, score: 0.21
101056452Unknown, score: 0.16
101056460Unknown, score: 0.07
101056496Unknown, score: 0.07
101056500Unknown, score: 0.07
101056542Unknown, score: 0.18
101056544Unknown, score: 0.42
101056547Unknown, score: 0.02
101056558Unknown, score: 0.03
101056574Unknown, score: 0.1
101056577Unknown, score: 0.12
101056619Unknown, score: 0.07
101056627Unknown, score: 0.1
101056654Unknown, score: 0.07
101056658Unknown, score: 0.14
101056688Unknown, score: 0.21
101056690Unknown, score: 0

## Help | Hide | Top Help | Show | Top Conditions

### HELP

Conditions in the module, given in the same order as on the expression
plot above. Red color means over-expression, green under-expression in
the given condition.

The barplot below shows the condition (sample) scores. A separate bar
is shown for each sample, its height is the corresponding score of the
sample in the module. The red and green numbers on the bars are the
sample scores expressed in percents, i.e. 100% is 1.0.

The red and green lines show the module thresholds, samples above
the red line and below the green line are included in the module.

The different experiments that were part of the study, are separated
by dashed vertical lines.

— Click on the *Help* button again to close this help window.

| Id |
| --- |
| SWR\_J-ISO\_136 |
| SWR\_J-ISO\_141 |
| SWR\_J-ISO\_142 |
| DBA\_2J-ISO\_8 |
| FVB\_NJ-ISO\_58 |
| FVB\_NJ-ISO\_55 |
| NOD\_ShiLtJ-ISO\_26 |
| NOD\_ShiLtJ-ISO\_25 |
| NZB\_BLNJ-ISO\_91 |
| NOD\_ShiLtJ-ISO\_24 |
| SM\_J-ISO\_45 |
| NZB\_BLNJ-ISO\_90 |
| NZB\_BLNJ-ISO\_96 |
| DBA\_2J-ISO\_4 |
| SM\_J-ISO\_44 |
| I\_LnJ-ISO\_149 |
| SJL\_J-ISO\_13 |
| SJL\_J-ISO\_12 |
| C3H\_HeJ-ISO\_62 |
| SM\_J-ISO\_46 |
| FVB\_NJ-ISO\_54 |
| I\_LnJ-ISO\_152 |
| BALB\_cJ-ISO\_81 |
| LP\_J-ISO\_102 |
| SJL\_J-ISO\_14 |
| PL\_J-ISO\_73 |
| LP\_J-ISO\_103 |
| C3H\_HeJ-ISO\_68 |
| PL\_J-ISO\_78 |
| I\_LnJ-ISO\_155 |
| PL\_J-ISO\_77 |
| C57BLKS\_J-ISO\_115 |
| DBA\_2J-ISO\_7 |
| C3H\_HeJ-ISO\_69 |
| C57BL\_6J-ISO\_50 |
| BALB\_cByJ-ISO\_156 |
| LP\_J-ISO\_99 |
| C58\_J-ISO\_33 |
| C58\_J-ISO\_31 |
| C58\_J-ISO\_37 |
| A\_J-ISO\_118 |
| BALB\_cByJ-ISO\_145 |
| C57BL\_6J-ISO\_59 |
| BALB\_cJ-ISO\_83 |
| C57BLKS\_J-ISO\_114 |
| BALB\_cByJ-ISO\_159 |
| CBA\_J-ISO\_128 |
| A\_J-ISO\_123 |
| C57BL\_6J-ISO\_47 |
| CBA\_J-ISO\_129 |
| SWR\_J-CTR\_134 |
| BALB\_cJ-ISO\_84 |
| SWR\_J-CTR\_137 |
| FVB\_NJ-CTR\_56 |
| NZB\_BLNJ-CTR\_87 |
| CBA\_J-ISO\_133 |
| A\_J-ISO\_124 |
| SWR\_J-ATE\_139 |
| NZB\_BLNJ-CTR\_93 |
| SWR\_J-CTR\_138 |
| NZB\_BLNJ-CTR\_92 |
| SM\_J-ATE\_42 |
| NZB\_BLNJ-ATE\_88 |
| SJL\_J-ATE\_11 |
| SWR\_J-ATE\_135 |
| SJL\_J-CTR\_10 |
| I\_LnJ-ATE\_146 |
| I\_LnJ-CTR\_147 |
| SM\_J-CTR\_41 |
| SWR\_J-ATE\_140 |
| SJL\_J-CTR\_15 |
| PL\_J-CTR\_74 |
| NZB\_BLNJ-ATE\_89 |
| I\_LnJ-CTR\_153 |
| I\_LnJ-CTR\_150 |
| SM\_J-CTR\_48 |
| DBA\_2J-CTR\_1 |
| I\_LnJ-ATE\_148 |
| SJL\_J-CTR\_16 |
| DBA\_2J-CTR\_2 |
| PL\_J-CTR\_72 |
| SM\_J-ATE\_49 |
| DBA\_2J-CTR\_9 |
| SM\_J-ATE\_43 |
| SJL\_J-ATE\_17 |
| LP\_J-CTR\_100 |
| NZB\_BLNJ-ATE\_94 |
| I\_LnJ-ATE\_154 |
| LP\_J-CTR\_104 |
| C57BL\_6J-CTR\_38 |
| LP\_J-ATE\_105 |
| FVB\_NJ-ATE\_57 |
| SM\_J-CTR\_40 |
| C57BL\_6J-CTR\_35 |
| C3H\_HeJ-CTR\_64 |
| LP\_J-CTR\_97 |
| C3H\_HeJ-CTR\_63 |
| FVB\_NJ-CTR\_52 |
| FVB\_NJ-CTR\_51 |
| C58\_J-CTR\_28 |
| SJL\_J-ATE\_18 |
| PL\_J-ATE\_71 |
| BALB\_cJ-CTR\_79 |
| C3H\_HeJ-CTR\_65 |
| C57BLKS\_J-CTR\_107 |
| C57BLKS\_J-CTR\_111 |
| PL\_J-CTR\_70 |
| LP\_J-ATE\_101 |
| C58\_J-CTR\_34 |
| LP\_J-ATE\_98 |
| FVB\_NJ-ATE\_53 |
| FVB\_NJ-ATE\_60 |
| BALB\_cJ-ATE\_82 |
| PL\_J-ATE\_75 |
| PL\_J-ATE\_76 |
| NOD\_ShiLtJ-CTR\_19 |
| C3H\_HeJ-ATE\_66 |
| C58\_J-CTR\_32 |
| DBA\_2J-ATE\_3 |
| NOD\_ShiLtJ-CTR\_20 |
| BALB\_cByJ-CTR\_143 |
| BALB\_cJ-ATE\_80 |
| A\_J-CTR\_121 |
| C57BL\_6J-CTR\_39 |
| C58\_J-ATE\_36 |
| C58\_J-ATE\_30 |
| NOD\_ShiLtJ-ATE\_27 |
| C57BLKS\_J-CTR\_110 |
| DBA\_2J-ATE\_5 |
| C58\_J-ATE\_29 |
| A\_J-CTR\_119 |
| CBA\_J-CTR\_125 |
| NOD\_ShiLtJ-CTR\_21 |
| C3H\_HeJ-ATE\_61 |
| BALB\_cByJ-CTR\_144 |
| C57BLKS\_J-ATE\_108 |
| BALB\_cJ-CTR\_85 |
| C57BLKS\_J-ATE\_112 |
| NOD\_ShiLtJ-ATE\_22 |
| C3H\_HeJ-ATE\_67 |
| C57BLKS\_J-ATE\_113 |
| DBA\_2J-ATE\_6 |
| C57BL\_6J-ATE\_109 |
| A\_J-CTR\_120 |
| CBA\_J-CTR\_126 |
| BALB\_cByJ-ATE\_160 |
| CBA\_J-ATE\_131 |
| A\_J-ATE\_122 |
| BALB\_cJ-ATE\_86 |
| BALB\_cByJ-CTR\_158 |
| BALB\_cByJ-ATE\_151 |
| NOD\_ShiLtJ-ATE\_23 |
| A\_J-ATE\_116 |
| A\_J-ATE\_117 |
| C57BL\_6J-ATE\_106 |
| CBA\_J-ATE\_127 |
| CBA\_J-ATE\_132 |
| CBA\_J-CTR\_130 |
| C57BL\_6J-ATE\_95 |
| BALB\_cByJ-ATE\_157 |

© 2015 Computational Biology Group, Department of Medical Genetics,
University of Lausanne, Switzerland
