# Supplementary material for: RNAseq analysis of heart tissue from mice treated with atenolol and isoproterenol reveals a reciprocal transcriptional response
Source: BMC Genomics. 2016 Sep 7;17(1):717. doi: 10.1186/s12864-016-3059-6 (PMC5015234; doi:10.1186/s12864-016-3059-6)
Supplement: Additional file 4: — Supplementary data. Compressed HTML files of 98 expression modules annotated for genes, strains and GO or KEGG terms (see Additional file 3 for navigation details). (GZ 11006 kb) [file 12864_2016_3059_MOESM4_ESM.gz › modules.html/module-76.html]

Module #76, TG: 2.5, TC: 0.625, 206 probes, 206 Entrez genes, 96 conditions

# Previous module | Next module Module #76, TG: 2.5, TC: 0.625, 206 probes, 206 Entrez genes, 96 conditions

- Module tree/table

- Expression data

- The BP GO tree
- The CC GO tree
- The MF GO tree

- GO BP enrichment
- GO CC enrichment
- GO MF enrichment
- KEGG enrichment
- miRNA enrichment

- Genes
- Conditions

## Help | Hide | Top Help | Show | Top Expression data

### HELP

The image plot shows the color-coded level of gene expression, for the
genes and conditions in a given transcription module. The genes are on
the horizontal, the conditions on the vertical axis.

The genes are ordered according to their ISA gene scores, similarly
the conditions are ordered according to their condition scores. The
score of a gene means the «degree of inclusion» in
the module: a high score gene is essential in the module.

Condition scores can also be negative, that means that the genes of
the module are all down-regulated in the condition. Here the absolute
value of the score gives the «degree of inclusion».

The plots above and beside the expression matrix show the gene scores
and condition scores, respectively.

Note that the plot is interactive, you can see the name of the gene
and condition under the mouse cursor.

The expression matrix was normalized to have mean zero and standard
deviation one for every gene separately across all conditions
(i.e. not just for the conditions in the module).

— Click on the *Help* button again to close this help window.

Gene:   
Condition:


Under-expression is coded with green,
over-expression with red color.

## Help | Hide | Top Help | Show | Top The GO tree — Biological processes

### HELP

This is one of three sections showing Gene Ontology enrichment of the
current module: in this case for **biological processes**.

The graph shows the hierarchy of the GO categories, their enrichment
for the current module is color coded, and the blue number beside the
category is the minus log ten p-value of the enrichment. (Calculated
using the standard hypergeometric test.) The color of the arrows code
«is a» (cyan) and «part of» relationships.

The tree was built the following way. First all GO terms with more
significant enrichment p-value than 0.05 were collected. Then all
paths from these terms to the root node of the GO tree were included
too. If a GO term is included more than once in the tree, then the
green numbers show 1) the id of the node, this makes it easier to find
other appereances of the term, and 2) the number of appearences.

Note that the same GO category might show up on the graph many
times. This is because the GO was «straightened» for this
graph, i.e. if there are more paths from a GO term to the root node of
the tree, all of them are included. The green numbers

Move the mouse cursor over the terms to get their definition. Clicking
on them takes you to the corresponding Gene Ontology web page.

If you cannot see a graph here at all, that means that there were no
significantly enriched GO categories, at the 0.05 level.

— Click on the *Help* button again to close this help window.

## Help | Hide | Top Help | Show | Top The GO tree — Cellular Components

### HELP

This is one of three sections showing Gene Ontology enrichment of the
current module: in this case for **cellular components**.

The graph shows the hierarchy of the GO categories, their enrichment
for the current module is color coded, and the blue number beside the
category is the minus log ten p-value of the enrichment. (Calculated
using the standard hypergeometric test.) The color of the arrows code
«is a» (cyan) and «part of» relationships.

The tree was built the following way. First all GO terms with more
significant enrichment p-value than 0.05 were collected. Then all
paths from these terms to the root node of the GO tree were included
too. If a GO term is included more than once in the tree, then the
green numbers show 1) the id of the node, this makes it easier to find
other appereances of the term, and 2) the number of appearences.

Note that the same GO category might show up on the graph many
times. This is because the GO was «straightened» for this
graph, i.e. if there are more paths from a GO term to the root node of
the tree, all of them are included. The green numbers

Move the mouse cursor over the terms to get their definition. Clicking
on them takes you to the corresponding Gene Ontology web page.

If you cannot see a graph here at all, that means that there were no
significantly enriched GO categories, at the 0.05 level.

— Click on the *Help* button again to close this help window.

:   **extracellular region**

    The space external to the outermost structure of a cell. For cells without external protective or external encapsulating structures this refers to space outside of the plasma membrane. This term covers the host cell environment outside an intracellular parasite.
:   **collagen**

    Any of the various assemblies in which collagen chains form a left-handed triple helix; may assemble into higher order structures.
:   **proteinaceous extracellular matrix**

    A layer consisting mainly of proteins (especially collagen) and glycosaminoglycans (mostly as proteoglycans) that forms a sheet underlying or overlying cells such as endothelial and epithelial cells. The proteins are secreted by cells in the vicinity. An example of this component is found in Mus musculus.
:   **extracellular matrix**

    A structure lying external to one or more cells, which provides structural support for cells or tissues; may be completely external to the cell (as in animals and bacteria) or be part of the cell (as in plants).
:   **extracellular matrix part**

    Any constituent part of the extracellular matrix, the structure lying external to one or more cells, which provides structural support for cells or tissues; may be completely external to the cell (as in animals) or be part of the cell (as often seen in plants).
:   **extracellular region part**

    Any constituent part of the extracellular region, the space external to the outermost structure of a cell. For cells without external protective or external encapsulating structures this refers to space outside of the plasma membrane. This term covers constituent parts of the host cell environment outside an intracellular parasite.
:   **all**

    NA
:   **NA**

    NA
:   **all**

    NA
:   **extracellular region part**

    Any constituent part of the extracellular region, the space external to the outermost structure of a cell. For cells without external protective or external encapsulating structures this refers to space outside of the plasma membrane. This term covers constituent parts of the host cell environment outside an intracellular parasite.
:   **proteinaceous extracellular matrix**

    A layer consisting mainly of proteins (especially collagen) and glycosaminoglycans (mostly as proteoglycans) that forms a sheet underlying or overlying cells such as endothelial and epithelial cells. The proteins are secreted by cells in the vicinity. An example of this component is found in Mus musculus.
:   **extracellular matrix**

    A structure lying external to one or more cells, which provides structural support for cells or tissues; may be completely external to the cell (as in animals and bacteria) or be part of the cell (as in plants).
:   **proteinaceous extracellular matrix**

    A layer consisting mainly of proteins (especially collagen) and glycosaminoglycans (mostly as proteoglycans) that forms a sheet underlying or overlying cells such as endothelial and epithelial cells. The proteins are secreted by cells in the vicinity. An example of this component is found in Mus musculus.

## Help | Hide | Top Help | Show | Top The GO tree — Molecular Function

### HELP

This is one of three sections showing Gene Ontology enrichment of the
current module: in this case for **molecular function**.

The graph shows the hierarchy of the GO categories, their enrichment
for the current module is color coded, and the blue number beside the
category is the minus log ten p-value of the enrichment. (Calculated
using the standard hypergeometric test.) The color of the arrows code
«is a» (cyan) and «part of» relationships.

The tree was built the following way. First all GO terms with more
significant enrichment p-value than 0.05 were collected. Then all
paths from these terms to the root node of the GO tree were included
too. If a GO term is included more than once in the tree, then the
green numbers show 1) the id of the node, this makes it easier to find
other appereances of the term, and 2) the number of appearences.

Note that the same GO category might show up on the graph many
times. This is because the GO was «straightened» for this
graph, i.e. if there are more paths from a GO term to the root node of
the tree, all of them are included. The green numbers

Move the mouse cursor over the terms to get their definition. Clicking
on them takes you to the corresponding Gene Ontology web page.

If you cannot see a graph here at all, that means that there were no
significantly enriched GO categories, at the 0.05 level.

— Click on the *Help* button again to close this help window.

## Help | Hide | Top Help | Show | Top GO BP test for over-representation

### HELP

List of all enriched GO categories (biological processes), at the 0.05
p-value level.

The columns:

- **ExpCount** is the expected count of genes in the
  module annotated with the given GO term, just by chance.
- **Count**
  is the number of genes in the module annotated with the given GO
  term.
- **Size** is the total number of genes (in our universe)
  annotated with the GO term.

Clicking on **Count** shows the genes that drive the
enrichment. You can also click on the individual numbers in
the **Count** column, to show the driving genes for that individual
GO category.

Clicking on the GO identifiers takes you to the Gene Ontology web
pages.

— Click on the *Help* button again to close this help window.

| Id | Pvalue | ExpCount | Count | Size | Term |
| --- | --- | --- | --- | --- | --- |
| GO:0022617 | 3.356e-02 | 0.1576 | 4 Ddr1, Dpp4, Fgfr4, Noxo1 | 12 | extracellular matrix disassembly |

## Help | Hide | Top Help | Show | Top GO CC test for over-representation

### HELP

List of all enriched GO categories (cellular components), at the 0.05
p-value level.

The columns:

- **ExpCount** is the expected count of genes in the
  module annotated with the given GO term, just by chance.
- **Count**
  is the number of genes in the module annotated with the given GO
  term.
- **Size** is the total number of genes (in our universe)
  annotated with the GO term.

Clicking on **Count** shows the genes that drive the
enrichment. You can also click on the individual numbers in
the **Count** column, to show the driving genes for that individual
GO category.

Clicking on the GO identifiers takes you to the Gene Ontology web
pages.

— Click on the *Help* button again to close this help window.

| Id | Pvalue | ExpCount | Count | Size | Term |
| --- | --- | --- | --- | --- | --- |
| GO:0005578 | 3.690e-06 | 3.867 | 19 Clec3b, Col11a2, Col13a1, Col19a1, Col5a1, Col5a2, Col6a2, Col7a1, Col9a3, Efemp2, Fbln1, Frem2, Lgals1, Mfap5, Mmp24, Ogn, Rpsa, Tgfbr3, Thbs2 | 293 | proteinaceous extracellular matrix |
| GO:0005576 | 2.884e-05 | 17.67 | 42 Acpp, Ccl17, Ccl5, Ccl8, Clec3b, Col11a2, Col13a1, Col19a1, Col5a1, Col5a2, Col6a2, Col7a1, Col9a3, Ctf1, Dpp4, Efemp2, Fbln1, Frem2, Fst, Fstl5, Gcnt1, Hhipl2, Igfbpl1, Il1rap, Itm2b, Lgals1, Lrp2, Mfap5, Mmp24, Nog, Ogn, Retn, Rpsa, Saa2, Sct, Sdf2, Serpina1e, Tgfbr3, Thbd, Thbs2, Ucn, Wfikkn2 | 1339 | extracellular region |
| GO:0044421 | 2.923e-05 | 11.34 | 32 Ccl17, Ccl5, Ccl8, Clec3b, Col11a2, Col13a1, Col19a1, Col5a1, Col5a2, Col6a2, Col7a1, Col9a3, Ctf1, Dpp4, Efemp2, Fbln1, Frem2, Gcnt1, Itm2b, Lgals1, Lrp2, Mfap5, Mmp24, Nog, Ogn, Retn, Rpsa, Saa2, Serpina1e, Tgfbr3, Thbd, Thbs2 | 859 | extracellular region part |
| GO:0031012 | 6.399e-05 | 4.632 | 19 Clec3b, Col11a2, Col13a1, Col19a1, Col5a1, Col5a2, Col6a2, Col7a1, Col9a3, Efemp2, Fbln1, Frem2, Lgals1, Mfap5, Mmp24, Ogn, Rpsa, Tgfbr3, Thbs2 | 351 | extracellular matrix |
| GO:0044420 | 1.160e-04 | 2.23 | 13 Col11a2, Col13a1, Col19a1, Col5a1, Col5a2, Col6a2, Col7a1, Col9a3, Fbln1, Frem2, Mfap5, Rpsa, Thbs2 | 169 | extracellular matrix part |
| GO:0005581 | 2.599e-03 | 1.029 | 8 Col11a2, Col13a1, Col19a1, Col5a1, Col5a2, Col6a2, Col7a1, Col9a3 | 78 | collagen |

## Help | Hide | Top Help | Show | Top GO MF test for over-representation

### HELP

List of all enriched GO categories (molecular function), at the 0.05
p-value level.

The columns:

- **ExpCount** is the expected count of genes in the
  module annotated with the given GO term, just by chance.
- **Count**
  is the number of genes in the module annotated with the given GO
  term.
- **Size** is the total number of genes (in our universe)
  annotated with the GO term.

Clicking on **Count** shows the genes that drive the
enrichment. You can also click on the individual numbers in
the **Count** column, to show the driving genes for that individual
GO category.

Clicking on the GO identifiers takes you to the Gene Ontology web
pages.

— Click on the *Help* button again to close this help window.

| Id | Pvalue | ExpCount | Count | Size | Term |
| --- | --- | --- | --- | --- | --- |
| GO:1901681 | 1.329e-02 | 1.939 | 10 Ccl5, Ccl8, Clec3b, Col13a1, Col5a1, Fgfr4, Fst, Ilvbl, Tgfbr3, Thbs2 | 149 | sulfur compound binding |
| GO:0008201 | 4.067e-02 | 1.392 | 8 Ccl5, Ccl8, Clec3b, Col13a1, Col5a1, Fgfr4, Tgfbr3, Thbs2 | 107 | heparin binding |

## Help | Hide | Top Help | Show | Top KEGG Pathway test for over-representation

### HELP

List of all enriched KEGG pathways, at the 0.05
p-value level.

The columns:

- **ExpCount** is the expected count of genes in the
  module annotated with the given KEGG pathway, just by chance.
- **Count**
  is the number of genes in the module annotated with the given KEGG
  pathway.
- **Size** is the total number of genes (in our universe)
  annotated with the KEGG pathway.

Clicking on **Count** shows the genes that drive the
enrichment. You can also click on the individual numbers in
the **Count** column, to show the driving genes for that individual
KEGG pathway.

Clicking on the KEGG identifiers takes you to the KEGG web site.

— Click on the *Help* button again to close this help window.

| Id | Pvalue | ExpCount | Count | Size | Term |
| --- | --- | --- | --- | --- | --- |
| 04974 | 7.697e-03 | 1.026 | 7 Col11a2, Col5a1, Col5a2, Col6a2, Col9a3, Dpp4, Slc1a1 | 61 | Protein digestion and absorption |


### HELP

List of all enriched miRNA families, at the 0.05
p-value level.

The columns:

- **ExpCount** is the expected count of genes in the
  module regulated by the given miRNA family, just by chance.
- **Count**
  is the number of genes in the module regulated by the given miRNA
  family.
- **Size** is the total number of genes (in our universe)
  regulated with the given miRNA family.

Clicking on **Count** shows the genes that drive the
enrichment. You can also click on the individual numbers in
the **Count** column, to show the driving genes for that individual
miRNA family.

The miRNA regulation data was taken from the

Top


### HELP

p-value level.

The columns:

- **ExpCount** is the expected number of genes in the- **Count**- **Size** is the total number of genes (in our universe)

Clicking on **Count** shows the genes that drive the
enrichment. You can also click on the individual numbers in
the **Count** column, to show the driving genes for that individual

— Click on the *Help* button again to close this help window.

## Help | Hide | Top Help | Show | Top Genes

### HELP

A list of all genes in the current module, in alphabetical order. The
size of the text corresponds to the gene scores.

Note that some gene symbols may show up more than once, if many
probes match the same Entrez gene.

Genes with no Entrez mapping are given separately, with their
Affymetrics probe ID.

— Click on the *Help* button again to close this help window.

### Genes Symbol

, score:

Actn3Unknown, score: 0.63
Adcy6Unknown, score: 0.66
AgaUnknown, score: 0.66
Ddr1Unknown, score: 0.74
CanxUnknown, score: 0.67
Cd33Unknown, score: 0.93
Cd4Unknown, score: 0.76
Col11a2Unknown, score: 0.74
Col13a1Unknown, score: 0.86
Col19a1Unknown, score: 0.65
Col5a1Unknown, score: 0.78
Col5a2Unknown, score: 0.64
Col6a2Unknown, score: 0.62
Col7a1Unknown, score: 0.82
Col9a3Unknown, score: 0.65
Atf6bUnknown, score: 0.69
CrklUnknown, score: 0.84
Csf2raUnknown, score: 0.65
Csf2rbUnknown, score: 0.67
Dnajc5Unknown, score: 0.8
Ctf1Unknown, score: 0.78
CtseUnknown, score: 0.66
Dad1Unknown, score: 0.68
DbtUnknown, score: 0.75
Dscr3Unknown, score: 0.96
DffbUnknown, score: 0.92
Dpp4Unknown, score: 0.71
Ercc3Unknown, score: 0.77
Fbln1Unknown, score: 0.76
Fgfr4Unknown, score: 0.65
Akr1b8Unknown, score: 0.84
FhitUnknown, score: 0.69
Aff2Unknown, score: 0.85
FstUnknown, score: 0.91
Gcnt1Unknown, score: 0.68
Gfpt1Unknown, score: 0.83
Lrp2Unknown, score: 0.81
Grk4Unknown, score: 0.88
Ica1Unknown, score: 0.61
Il1rapUnknown, score: 0.79
ItgavUnknown, score: 0.63
Itm2bUnknown, score: 0.68
KhsrpUnknown, score: 0.71
Kif5aUnknown, score: 0.64
Krt14Unknown, score: 0.65
RpsaUnknown, score: 0.66
Lgals1Unknown, score: 0.72
Ltb4r1Unknown, score: 0.84
MagohUnknown, score: 0.61
Man1a2Unknown, score: 0.62
MatkUnknown, score: 0.61
Men1Unknown, score: 0.64
Mmp24Unknown, score: 0.63
Gbp4Unknown, score: 0.86
Mrc1Unknown, score: 0.8
Polr2kUnknown, score: 0.65
NogUnknown, score: 0.81
OgnUnknown, score: 0.75
OmpUnknown, score: 0.62
Pip5k1bUnknown, score: 0.74
PrkdcUnknown, score: 0.66
PrkxUnknown, score: 0.77
PtgirUnknown, score: 0.68
Rfx1Unknown, score: 0.64
S100a10Unknown, score: 0.79
Saa2Unknown, score: 0.65
SctUnknown, score: 0.68
Ccl17Unknown, score: 0.66
Ccl5Unknown, score: 0.64
Ccl8Unknown, score: 0.69
Sdf2Unknown, score: 0.73
Sel1lUnknown, score: 0.66
Spi1Unknown, score: 0.76
Itsn2Unknown, score: 0.79
SkiUnknown, score: 0.68
Slc16a2Unknown, score: 0.65
Slc1a1Unknown, score: 0.66
Sntb2Unknown, score: 0.66
Serpina1eUnknown, score: 0.68
Spta1Unknown, score: 0.67
SpopUnknown, score: 0.77
SprUnknown, score: 0.73
Stat2Unknown, score: 0.63
Tgfbr3Unknown, score: 0.68
ThbdUnknown, score: 0.69
Thbs2Unknown, score: 0.72
Timm44Unknown, score: 1
Clec3bUnknown, score: 0.95
Tnfaip3Unknown, score: 0.61
Tubb2aUnknown, score: 0.62
UcnUnknown, score: 0.66
UgcgUnknown, score: 0.74
VillUnknown, score: 0.66
Slc30a4Unknown, score: 0.64
Ggt5Unknown, score: 0.88
Cul1Unknown, score: 0.62
Hacd1Unknown, score: 0.69
Mrps7Unknown, score: 0.97
Mfap5Unknown, score: 0.99
Rgs3Unknown, score: 0.91
Klhl7Unknown, score: 0.73
Nudcd2Unknown, score: 0.61
Plrg1Unknown, score: 0.69
Pla2g6Unknown, score: 0.71
Rfx5Unknown, score: 0.8
Rpp30Unknown, score: 0.69
Pdzrn3Unknown, score: 0.69
Fmo2Unknown, score: 0.7
Gps2Unknown, score: 0.65
AcppUnknown, score: 0.84
Clec4eUnknown, score: 0.68
RetnUnknown, score: 0.68
Zfp318Unknown, score: 0.66
Rab37Unknown, score: 0.62
Efemp2Unknown, score: 0.71
Dctn5Unknown, score: 0.73
CubnUnknown, score: 0.74
Uqcrfs1Unknown, score: 0.87
Peli1Unknown, score: 0.64
Bend5Unknown, score: 0.73
MgarpUnknown, score: 0.62
Pold3Unknown, score: 0.68
Vps53Unknown, score: 0.62
Samm50Unknown, score: 0.82
Zfand2bUnknown, score: 0.66
Ccdc23Unknown, score: 0.64
1700029J07RikUnknown, score: 0.63
Dnase1l1Unknown, score: 0.63
Bag5Unknown, score: 0.65
Taf15Unknown, score: 0.68
UvssaUnknown, score: 0.65
Otud1Unknown, score: 0.64
Dlg5Unknown, score: 0.62
Vps11Unknown, score: 0.61
Noxo1Unknown, score: 0.73
CdaUnknown, score: 0.72
Lsm11Unknown, score: 0.74
Mvb12aUnknown, score: 0.62
4632428N05RikUnknown, score: 0.62
Syvn1Unknown, score: 0.66
Clvs1Unknown, score: 0.7
CmipUnknown, score: 0.72
Cep128Unknown, score: 0.71
Tti1Unknown, score: 0.79
Igfbpl1Unknown, score: 0.73
Wdr64Unknown, score: 0.64
GptUnknown, score: 0.92
TgdsUnknown, score: 0.62
1700015E13RikUnknown, score: 0.64
Bcas1Unknown, score: 0.81
Hhipl2Unknown, score: 0.63
MispUnknown, score: 0.66
LrbaUnknown, score: 0.72
Ntng1Unknown, score: 0.61
Jdp2Unknown, score: 0.65
Tmem40Unknown, score: 0.67
Stard7Unknown, score: 0.77
Golga2Unknown, score: 0.73
Mycbp2Unknown, score: 0.64
Nt5c3Unknown, score: 0.71
Ddb2Unknown, score: 0.75
Ap4e1Unknown, score: 0.61
Crip3Unknown, score: 0.62
Kcnn3Unknown, score: 0.62
Ppargc1bUnknown, score: 0.78
Bcas3Unknown, score: 0.7
Hdac4Unknown, score: 0.71
Depdc7Unknown, score: 0.62
Plekhh1Unknown, score: 0.64
Fstl5Unknown, score: 0.72
Parp16Unknown, score: 0.83
IlvblUnknown, score: 0.79
Rufy1Unknown, score: 0.88
G2e3Unknown, score: 0.72
Hmbox1Unknown, score: 0.75
GgactUnknown, score: 0.76
Cep76Unknown, score: 0.7
Hectd2Unknown, score: 0.64
FicdUnknown, score: 0.72
Frmd4bUnknown, score: 0.68
Msantd2Unknown, score: 0.62
Snx33Unknown, score: 0.73
Ric8bUnknown, score: 0.62
Sec31bUnknown, score: 0.64
Mcmdc2Unknown, score: 0.8
Dusp27Unknown, score: 0.74
Frem2Unknown, score: 0.76
Fbxl5Unknown, score: 0.66
Sbno1Unknown, score: 0.63
Ankk1Unknown, score: 0.63
Nat14Unknown, score: 0.63
Marveld1Unknown, score: 0.79
Wfikkn2Unknown, score: 0.78
A230050P20RikUnknown, score: 0.66
C5ar2Unknown, score: 0.67
Nceh1Unknown, score: 0.69
Alg6Unknown, score: 0.67
Dennd5bUnknown, score: 0.64
D5Ertd579eUnknown, score: 0.66
Trim65Unknown, score: 0.62
Mrs2Unknown, score: 0.75
Greb1lUnknown, score: 0.68
Pdcd5Unknown, score: 0.62
Cfap43Unknown, score: 0.72
Ccdc13Unknown, score: 0.79
100862012Unknown, score: 0.67

## Help | Hide | Top Help | Show | Top Conditions

### HELP

Conditions in the module, given in the same order as on the expression
plot above. Red color means over-expression, green under-expression in
the given condition.

The barplot below shows the condition (sample) scores. A separate bar
is shown for each sample, its height is the corresponding score of the
sample in the module. The red and green numbers on the bars are the
sample scores expressed in percents, i.e. 100% is 1.0.

The red and green lines show the module thresholds, samples above
the red line and below the green line are included in the module.

The different experiments that were part of the study, are separated
by dashed vertical lines.

— Click on the *Help* button again to close this help window.

Id

LP\_J-ATE\_105

A\_J-ATE\_117

LP\_J-ATE\_98

FVB\_NJ-ATE\_60

FVB\_NJ-ATE\_53

NOD\_ShiLtJ-ATE\_23

FVB\_NJ-CTR\_51

FVB\_NJ-ATE\_57

NOD\_ShiLtJ-ATE\_22

LP\_J-CTR\_104

FVB\_NJ-CTR\_52

A\_J-ATE\_116

NOD\_ShiLtJ-ATE\_27

SJL\_J-ATE\_18

LP\_J-ATE\_101

SWR\_J-ATE\_135

LP\_J-CTR\_97

C57BL\_6J-ATE\_109

NOD\_ShiLtJ-CTR\_21

SWR\_J-ATE\_140

LP\_J-CTR\_100

C57BL\_6J-ATE\_95

A\_J-CTR\_120

FVB\_NJ-CTR\_56

A\_J-CTR\_119

SWR\_J-ATE\_139

NOD\_ShiLtJ-CTR\_20

SJL\_J-ATE\_17

SJL\_J-CTR\_10

C58\_J-ATE\_29

SWR\_J-CTR\_138

C58\_J-CTR\_32

C58\_J-ATE\_36

NOD\_ShiLtJ-CTR\_19

PL\_J-ATE\_76

SJL\_J-CTR\_15

NZB\_BLNJ-CTR\_92

C58\_J-ATE\_30

C58\_J-CTR\_34

SJL\_J-CTR\_16

A\_J-ATE\_122

BALB\_cByJ-ATE\_157

C57BL\_6J-CTR\_39

NZB\_BLNJ-ATE\_94

SJL\_J-ATE\_11

C57BL\_6J-CTR\_38

PL\_J-ATE\_75

BALB\_cByJ-ATE\_160

C57BLKS\_J-ATE\_108

C3H\_HeJ-ATE\_61

SWR\_J-CTR\_134

DBA\_2J-CTR\_1

LP\_J-ISO\_102

FVB\_NJ-ISO\_55

SWR\_J-ISO\_136

C58\_J-ISO\_37

C58\_J-ISO\_31

C57BL\_6J-ISO\_50

SWR\_J-ISO\_141

A\_J-ISO\_123

PL\_J-ISO\_77

A\_J-ISO\_124

NZB\_BLNJ-ISO\_96

A\_J-ISO\_118

PL\_J-ISO\_78

C57BL\_6J-ISO\_59

NZB\_BLNJ-ISO\_90

PL\_J-ISO\_73

NZB\_BLNJ-ISO\_91

BALB\_cByJ-ISO\_145

C57BLKS\_J-ISO\_114

I\_LnJ-ISO\_155

I\_LnJ-ISO\_152

BALB\_cByJ-ISO\_159

CBA\_J-ISO\_129

NOD\_ShiLtJ-ISO\_24

NOD\_ShiLtJ-ISO\_26

C3H\_HeJ-ISO\_68

CBA\_J-ISO\_128

C3H\_HeJ-ISO\_69

CBA\_J-ISO\_133

BALB\_cByJ-ISO\_156

NOD\_ShiLtJ-ISO\_25

C58\_J-ISO\_33

BALB\_cJ-ISO\_84

SM\_J-ISO\_45

SM\_J-ISO\_44

DBA\_2J-ISO\_4

DBA\_2J-ISO\_7

SM\_J-ISO\_46

BALB\_cJ-ISO\_83

C57BLKS\_J-ISO\_115

BALB\_cJ-ISO\_81

I\_LnJ-ISO\_149

C3H\_HeJ-ISO\_62

DBA\_2J-ISO\_8

© 2015 Computational Biology Group, Department of Medical Genetics,
University of Lausanne, Switzerland
