# Supplementary material for: RNAseq analysis of heart tissue from mice treated with atenolol and isoproterenol reveals a reciprocal transcriptional response
Source: BMC Genomics. 2016 Sep 7;17(1):717. doi: 10.1186/s12864-016-3059-6 (PMC5015234; doi:10.1186/s12864-016-3059-6)
Supplement: Additional file 4: — Supplementary data. Compressed HTML files of 98 expression modules annotated for genes, strains and GO or KEGG terms (see Additional file 3 for navigation details). (GZ 11006 kb) [file 12864_2016_3059_MOESM4_ESM.gz › modules.html/index.html]

Modules of counter-expressed genes

# Modules of counter-expressed genes

## All modules

|  |  |  |  |  |  |  |  |
| --- | --- | --- | --- | --- | --- | --- | --- |
| # | Thr. | #G | #C | GO BP | GO CC | GO MF | KEGG |
| 1 | 3.5/0.875 | 38 | 72 | lysosomal lumen acidification  (4.7e-02/1.2e-02/ 2/ 5) | intracellular organelle part  (3.3e-02/9.4e+00/ 20/3924) |  |  |
| 2 | 3.5/0.875 | 48 | 81 |  |  |  |  |
| 3 | 3.5/0.875 | 43 | 85 |  |  | enzyme binding  (4.6e-02/3.2e+00/ 11/1175) |  |
| 4 | 3.5/0.875 | 25 | 33 | antigen processing and presentation of peptide antigen  (1.5e-02/5.8e-02/ 3/ 38) | MHC protein complex  (4.8e-04/3.4e-02/ 3/ 23) | beta-2-microglobulin binding  (6.8e-08/1.4e-02/ 4/ 9) | Allograft rejection  (2.6e-06/ 0.1218/ 5/ 38) |
| 5 | 3.5/0.875 | 51 | 30 |  |  |  |  |
| 6 | 3.5/0.875 | 40 | 81 |  |  |  |  |
| 7 | 3.5/0.875 | 34 | 79 | lysosomal lumen acidification  (3.8e-02/1.1e-02/ 2/ 5) | organelle part  (2.2e-02/8.7e+00/ 19/4038) |  |  |
| 8 | 3.5/0.875 | 40 | 66 |  |  |  |  |
| 9 | 3.5/0.875 | 49 | 86 |  |  |  |  |
| 10 | 3.5/0.875 | 154 | 39 | immune response  (1.5e-12/6.0e+00/ 33/ 638) | MHC protein complex  (5.4e-04/2.2e-01/ 5/ 23) | beta-2-microglobulin binding  (3.9e-04/8.5e-02/ 4/ 9) | Antigen processing and presentation  (7.3e-10/ 0.7799/12/ 66) |
| 11 | 3.5/0.875 | 34 | 65 |  |  |  |  |
| 12 | 3.5/0.875 | 39 | 84 |  |  |  |  |
| 13 | 3.5/0.875 | 61 | 26 |  |  |  | Osteoclast differentiation  (1.5e-02/ 0.4110/ 4/114) |
| 14 | 3.5/0.875 | 28 | 69 |  | extracellular region  (3.5e-02/2.4e+00/ 9/1339) |  |  |
| 15 | 3.5/0.875 | 18 | 95 |  |  |  |  |
| 16 | 3.5/0.875 | 23 | 61 |  |  |  |  |
| 17 | 3.5/0.875 | 47 | 9 |  |  |  |  |
| 18 | 3.5/0.875 | 31 | 78 |  | organelle part  (3.4e-03/7.9e+00/ 19/4038) |  |  |
| 19 | 3.5/0.875 | 29 | 74 |  | extracellular region  (4.8e-02/2.4e+00/ 9/1339) |  |  |
| 20 | 3.5/0.875 | 13 | 67 |  |  |  | Bile secretion  (4.3e-02/ 0.0611/ 2/ 61) |
| 21 | 3.5/0.875 | 24 | 71 |  |  |  |  |
| 22 | 3.5/0.875 | 29 | 49 | positive regulation of leukocyte differentiation  (3.3e-02/1.8e-01/ 4/ 99) | external side of plasma membrane  (3.1e-04/4.1e-01/ 6/ 226) | peptide antigen binding  (2.0e-02/1.8e-02/ 2/ 10) |  |
| 23 | 3.5/0.875 | 62 | 9 |  |  |  |  |
| 24 | 3.5/0.875 | 13 | 71 |  |  |  |  |
| 25 | 3/0.75 | 59 | 48 |  | MHC protein complex  (1.2e-02/8.4e-02/ 3/ 23) | beta-2-microglobulin binding  (5.1e-06/3.3e-02/ 4/ 9) | Allograft rejection  (4.9e-05/ 0.1979/ 5/ 38) |
| 26 | 3/0.75 | 98 | 26 |  |  |  |  |
| 27 | 3/0.75 | 121 | 97 |  |  |  |  |
| 28 | 3/0.75 | 89 | 52 |  |  |  |  |
| 29 | 3/0.75 | 72 | 96 |  |  |  |  |
| 30 | 3/0.75 | 76 | 54 |  |  |  |  |
| 31 | 3/0.75 | 36 | 76 |  |  |  | Protein export  (4.3e-02/ 0.0601/ 2/ 25) |
| 32 | 3/0.75 | 50 | 50 |  | MHC protein complex  (6.6e-03/7.0e-02/ 3/ 23) | beta-2-microglobulin binding  (2.3e-06/2.8e-02/ 4/ 9) | Allograft rejection  (1.0e-06/ 0.1903/ 6/ 38) |
| 33 | 3/0.75 | 44 | 78 |  |  |  |  |
| 34 | 3/0.75 | 74 | 68 |  |  |  |  |
| 35 | 3/0.75 | 63 | 54 |  |  |  |  |
| 36 | 3/0.75 | 56 | 91 |  |  |  |  |
| 37 | 3/0.75 | 84 | 61 |  |  |  |  |
| 38 | 3/0.75 | 73 | 92 |  |  |  |  |
| 39 | 3/0.75 | 54 | 81 |  |  |  |  |
| 40 | 3/0.75 | 90 | 87 |  |  |  |  |
| 41 | 3/0.75 | 55 | 68 |  | organelle membrane  (2.6e-02/4.1e+00/ 13/1225) |  | Bile secretion  (4.8e-02/ 0.2321/ 3/ 61) |
| 42 | 3/0.75 | 44 | 82 |  |  |  |  |
| 43 | 3/0.75 | 75 | 78 |  |  |  |  |
| 44 | 3/0.75 | 82 | 86 | extracellular matrix organization  (2.1e-02/7.9e-01/ 7/ 148) | proteinaceous extracellular matrix  (5.1e-04/1.5e+00/ 10/ 293) | sulfur compound binding  (4.1e-02/7.9e-01/ 6/ 149) |  |
| 45 | 3/0.75 | 60 | 78 |  |  |  |  |
| 46 | 3/0.75 | 90 | 41 |  |  |  |  |
| 47 | 3/0.75 | 55 | 58 |  |  | peptide binding  (4.0e-02/5.3e-01/ 5/ 166) |  |
| 48 | 3/0.75 | 75 | 58 |  |  |  | Antigen processing and presentation  (1.4e-02/ 0.3305/ 4/ 66) |
| 49 | 3/0.75 | 42 | 95 |  |  |  |  |
| 50 | 3/0.75 | 78 | 85 | renal absorption  (6.4e-03/3.5e-02/ 3/ 7) |  | water channel activity  (5.0e-03/5.1e-02/ 3/ 10) |  |
| 51 | 3/0.75 | 63 | 51 |  | MHC protein complex  (1.4e-02/8.7e-02/ 3/ 23) | beta-2-microglobulin binding  (6.4e-06/3.4e-02/ 4/ 9) | Allograft rejection  (4.7e-05/ 0.1979/ 5/ 38) |
| 52 | 3/0.75 | 103 | 31 |  |  |  |  |
| 53 | 2.5/0.625 | 201 | 66 |  |  |  |  |
| 54 | 2.5/0.625 | 228 | 53 |  |  |  |  |
| 55 | 2.5/0.625 | 254 | 111 |  |  |  |  |
| 56 | 2.5/0.625 | 211 | 108 | extracellular matrix disassembly  (3.7e-02/1.6e-01/ 4/ 12) | extracellular region  (3.0e-03/1.8e+01/ 38/1339) | sulfur compound binding  (1.7e-02/2.0e+00/ 10/ 149) |  |
| 57 | 2.5/0.625 | 193 | 96 | extracellular matrix disassembly  (2.6e-02/1.5e-01/ 4/ 12) | extracellular region  (5.4e-03/1.7e+01/ 35/1339) |  |  |
| 58 | 2.5/0.625 | 174 | 85 |  |  |  |  |
| 59 | 2.5/0.625 | 160 | 87 |  |  |  |  |
| 60 | 2.5/0.625 | 172 | 83 |  |  |  |  |
| 61 | 2.5/0.625 | 184 | 96 |  |  |  |  |
| 62 | 2.5/0.625 | 178 | 82 |  |  |  | Terpenoid backbone biosynthesis  (1.9e-02/ 0.1198/ 3/ 13) |
| 63 | 2.5/0.625 | 171 | 84 |  |  |  |  |
| 64 | 2.5/0.625 | 173 | 88 |  |  |  |  |
| 65 | 2.5/0.625 | 188 | 86 |  |  |  |  |
| 66 | 2.5/0.625 | 146 | 68 |  |  |  |  |
| 67 | 2.5/0.625 | 185 | 87 |  |  |  |  |
| 68 | 2.5/0.625 | 144 | 84 |  |  |  |  |
| 69 | 2.5/0.625 | 219 | 41 |  |  |  |  |
| 70 | 2.5/0.625 | 150 | 86 |  |  |  |  |
| 71 | 2.5/0.625 | 150 | 71 | defense response  (6.7e-06/6.3e+00/ 25/ 676) |  |  | Leukocyte transendothelial migration  (4.6e-03/ 1.2996/ 8/103) |
| 72 | 2.5/0.625 | 148 | 81 |  |  |  |  |
| 73 | 2.5/0.625 | 184 | 75 |  |  |  | Terpenoid backbone biosynthesis  (7.0e-03/ 0.1015/ 3/ 13) |
| 74 | 2.5/0.625 | 253 | 67 | defense response  (7.4e-10/1.1e+01/ 40/ 676) | MHC protein complex  (8.6e-03/3.6e-01/ 5/ 23) | immunoglobulin binding  (2.0e-02/2.0e-01/ 4/ 13) | Antigen processing and presentation  (2.1e-06/ 1.1897/11/ 66) |
| 75 | 2.5/0.625 | 147 | 62 |  |  | beta-2-microglobulin binding  (2.7e-04/7.9e-02/ 4/ 9) | Allograft rejection  (3.9e-03/ 0.4110/ 5/ 38) |
| 76 | 2.5/0.625 | 206 | 96 | extracellular matrix disassembly  (3.4e-02/1.6e-01/ 4/ 12) | proteinaceous extracellular matrix  (3.7e-06/3.9e+00/ 19/ 293) | sulfur compound binding  (1.3e-02/1.9e+00/ 10/ 149) | Protein digestion and absorption  (7.7e-03/ 1.0262/ 7/ 61) |
| 77 | 2/0.5 | 548 | 129 |  | extracellular region part  (4.3e-02/3.0e+01/ 52/ 859) |  |  |
| 78 | 2/0.5 | 418 | 93 |  |  |  | Ribosome  (4.7e-02/ 2.1979/ 9/ 93) |
| 79 | 2/0.5 | 459 | 82 | defense response  (2.8e-08/1.9e+01/ 54/ 676) | external side of plasma membrane  (4.4e-02/6.5e+00/ 18/ 226) |  | Allograft rejection  (4.4e-05/ 1.2710/10/ 38) |
| 80 | 2/0.5 | 433 | 96 |  |  |  |  |
| 81 | 2/0.5 | 468 | 82 | defense response  (3.8e-07/2.0e+01/ 52/ 676) |  |  | Allograft rejection  (3.9e-03/ 1.2558/ 8/ 38) |
| 82 | 2/0.5 | 463 | 85 | defense response  (3.1e-08/1.9e+01/ 54/ 676) |  |  | Allograft rejection  (4.4e-04/ 1.2634/ 9/ 38) |
| 83 | 2/0.5 | 426 | 93 |  |  |  | Ribosome  (4.6e-02/ 2.1979/ 9/ 93) |
| 84 | 2/0.5 | 382 | 68 |  |  |  |  |
| 85 | 2/0.5 | 396 | 102 |  |  | GABA-A receptor activity  (4.3e-02/2.4e-01/ 4/ 10) |  |
| 86 | 1.5/0.375 | 1101 | 136 | regulation of signaling  (3.0e-02/1.3e+02/180/1895) | extracellular region  (7.4e-04/9.4e+01/139/1339) | chemokine activity  (3.6e-03/2.1e+00/ 11/ 30) | Chemokine signaling pathway  (2.1e-03/13.7212/31/170) |
| 87 | 1.5/0.375 | 1158 | 105 | defense response  (9.8e-05/4.8e+01/ 88/ 676) | cytosolic ribosome  (1.2e-02/5.9e+00/ 18/ 83) |  | Antigen processing and presentation  (8.8e-04/ 4.9702/17/ 66) |
| 88 | 1.5/0.375 | 1101 | 103 |  |  |  |  |
| 89 | 1.5/0.375 | 1016 | 106 | monocarboxylic acid metabolic process  (2.3e-03/2.2e+01/ 48/ 351) | cytoplasmic part  (4.9e-02/3.3e+02/383/5182) | tetrapyrrole binding  (3.8e-05/6.7e+00/ 24/ 105) | PPAR signaling pathway  (5.5e-04/ 4.7677/17/ 69) |
| 90 | 1.5/0.375 | 1036 | 92 |  |  |  | Ribosome  (5.2e-03/ 5.5506/17/ 93) |
| 91 | 1/0.25 | 2278 | 144 | cell communication  (1.3e-04/5.2e+02/627/3637) | extracellular region  (3.2e-05/1.9e+02/262/1339) | protease binding  (6.3e-04/9.9e+00/ 27/ 69) | Hematopoietic cell lineage  (1.9e-02/11.6639/25/ 74) |
| 92 | 1/0.25 | 2423 | 133 | regulation of localization  (3.0e-02/2.1e+02/274/1409) | proteinaceous extracellular matrix  (2.2e-03/4.5e+01/ 75/ 293) | chemokine activity  (1.5e-02/4.6e+00/ 15/ 30) |  |
| 93 | 1/0.25 | 2385 | 121 |  |  | oxidoreductase activity, acting on paired donors, with incorporation or reduction of molecular oxygen, reduced flavin or flavoprotein as one donor, and incorporation of one atom of oxygen  (1.4e-02/3.6e+00/ 13/ 24) |  |
| 94 | 0.5/0.125 | 4697 | 145 | cellular component movement  (1.6e-04/3.0e+02/384/1031) | extracellular region  (3.7e-03/4.0e+02/468/1339) | protease binding  (1.7e-02/2.0e+01/ 38/ 69) | Leishmaniasis  (1.6e-03/18.3978/35/ 60) |
| 95 | 0/0 | 7837 | 160 |  | cytosolic part  (2.5e-03/8.4e+01/114/ 172) |  | Ribosome  (1.9e-02/45.6898/64/ 93) |
| 96 | 0/0 | 7946 | 160 |  |  |  |  |
| 97 | 0/0 | 8063 | 160 |  | ribosomal subunit  (1.8e-02/6.3e+01/ 86/ 125) | structural constituent of ribosome  (1.8e-02/5.9e+01/ 83/ 118) |  |
| 98 | 0/0 | 8010 | 160 |  |  |  |  |

© 2015 Computational Biology Group, Department of Medical Genetics,
University of Lausanne, Switzerland
