# Supplementary material for: RNAseq analysis of heart tissue from mice treated with atenolol and isoproterenol reveals a reciprocal transcriptional response
Source: BMC Genomics. 2016 Sep 7;17(1):717. doi: 10.1186/s12864-016-3059-6 (PMC5015234; doi:10.1186/s12864-016-3059-6)
Supplement: Additional file 4: — Supplementary data. Compressed HTML files of 98 expression modules annotated for genes, strains and GO or KEGG terms (see Additional file 3 for navigation details). (GZ 11006 kb) [file 12864_2016_3059_MOESM4_ESM.gz › modules.html/mainlattice.html.in]

<!-- title -->


Annotation:

Gene Ontology, biological processes enrichment
Gene Ontology, cellular components enrichment
Gene Ontology, molecular function enrichment
KEGG pathways enrichment
miRNA families enrichment
Distinguished experiments
— See more options below the figure

|  |  |
| --- | --- |
| Color code: |  |

**Loading...**

|  |  |  |  |  |  |
| --- | --- | --- | --- | --- | --- |
| Tool tips: | Genes Conditions GO, biological process GO, cellular components GO, molecular function KEGG enriched pathways miRNA enrichment | Color code: | GO BP smallest p-value GO CC smallest p-value GO MF smallest p-value KEGG smallest p-value miRNA smallest p-value Distinguished experiments |  |  |
| Top left: | Module number Module size (genes and conditions) Empty | Bottom: | GO BP, most significant GO CC, most significant GO MF, most significant KEGG, most significant miRNA, most significant Experiments Empty | Top right: | GO BP P-value GO CC P-value GO MF P-value KEGG P-value miRNA P-value Distinguished experiments Empty |


© 2008 Computational Biology Group, Department of Medical Genetics,
University of Lausanne, Switzerland
