# Supplementary material for: RNAseq analysis of heart tissue from mice treated with atenolol and isoproterenol reveals a reciprocal transcriptional response
Source: BMC Genomics. 2016 Sep 7;17(1):717. doi: 10.1186/s12864-016-3059-6 (PMC5015234; doi:10.1186/s12864-016-3059-6)
Supplement: Additional file 4: — Supplementary data. Compressed HTML files of 98 expression modules annotated for genes, strains and GO or KEGG terms (see Additional file 3 for navigation details). (GZ 11006 kb) [file 12864_2016_3059_MOESM4_ESM.gz › modules.html/module-10.html]

Module #10, TG: 3.5, TC: 0.875, 154 probes, 154 Entrez genes, 39 conditions

# Previous module | Next module Module #10, TG: 3.5, TC: 0.875, 154 probes, 154 Entrez genes, 39 conditions

- Module tree/table

- Expression data

- The BP GO tree
- The CC GO tree
- The MF GO tree

- GO BP enrichment
- GO CC enrichment
- GO MF enrichment
- KEGG enrichment
- miRNA enrichment

- Genes
- Conditions

## Help | Hide | Top Help | Show | Top Expression data

### HELP

The image plot shows the color-coded level of gene expression, for the
genes and conditions in a given transcription module. The genes are on
the horizontal, the conditions on the vertical axis.

The genes are ordered according to their ISA gene scores, similarly
the conditions are ordered according to their condition scores. The
score of a gene means the «degree of inclusion» in
the module: a high score gene is essential in the module.

Condition scores can also be negative, that means that the genes of
the module are all down-regulated in the condition. Here the absolute
value of the score gives the «degree of inclusion».

The plots above and beside the expression matrix show the gene scores
and condition scores, respectively.

Note that the plot is interactive, you can see the name of the gene
and condition under the mouse cursor.

The expression matrix was normalized to have mean zero and standard
deviation one for every gene separately across all conditions
(i.e. not just for the conditions in the module).

— Click on the *Help* button again to close this help window.

Gene:   
Condition:


Under-expression is coded with green,
over-expression with red color.

## Help | Hide | Top Help | Show | Top The GO tree — Biological processes

### HELP

This is one of three sections showing Gene Ontology enrichment of the
current module: in this case for **biological processes**.

The graph shows the hierarchy of the GO categories, their enrichment
for the current module is color coded, and the blue number beside the
category is the minus log ten p-value of the enrichment. (Calculated
using the standard hypergeometric test.) The color of the arrows code
«is a» (cyan) and «part of» relationships.

The tree was built the following way. First all GO terms with more
significant enrichment p-value than 0.05 were collected. Then all
paths from these terms to the root node of the GO tree were included
too. If a GO term is included more than once in the tree, then the
green numbers show 1) the id of the node, this makes it easier to find
other appereances of the term, and 2) the number of appearences.

Note that the same GO category might show up on the graph many
times. This is because the GO was «straightened» for this
graph, i.e. if there are more paths from a GO term to the root node of
the tree, all of them are included. The green numbers

Move the mouse cursor over the terms to get their definition. Clicking
on them takes you to the corresponding Gene Ontology web page.

If you cannot see a graph here at all, that means that there were no
significantly enriched GO categories, at the 0.05 level.

— Click on the *Help* button again to close this help window.

:   **immune effector process**

    Any process of the immune system that can potentially contribute to an immune response.
:   **immune system process**

    Any process involved in the development or functioning of the immune system, an organismal system for calibrated responses to potential internal or invasive threats.
:   **leukocyte mediated immunity**

    Any process involved in the carrying out of an immune response by a leukocyte.
:   **lymphocyte mediated immunity**

    Any process involved in the carrying out of an immune response by a lymphocyte.
:   **humoral immune response mediated by circulating immunoglobulin**

    An immune response dependent upon secreted immunoglobulin. An example of this process is found in Mus musculus.
:   **adaptive immune response based on somatic recombination of immune receptors built from immunoglobulin superfamily domains**

    An immune response based on directed amplification of specific receptors for antigen produced through a somatic diversification process that includes somatic recombination of germline gene segments encoding immunoglobulin superfamily domains, and allowing for enhanced responses upon subsequent exposures to the same antigen (immunological memory). Recombined receptors for antigen encoded by immunoglobulin superfamily domains include T cell receptors and immunoglobulins (antibodies). An example of this is the adaptive immune response found in Mus musculus.
:   **antigen processing and presentation of exogenous peptide antigen**

    The process in which an antigen-presenting cell expresses a peptide antigen of exogenous origin on its cell surface in association with an MHC protein complex. The peptide is typically a fragment of a larger exogenous protein which has been degraded within the cell.
:   **regulation of immune system process**

    Any process that modulates the frequency, rate, or extent of an immune system process.
:   **positive regulation of immune system process**

    Any process that activates or increases the frequency, rate, or extent of an immune system process.
:   **regulation of adaptive immune response**

    Any process that modulates the frequency, rate, or extent of an adaptive immune response.
:   **positive regulation of adaptive immune response**

    Any process that activates or increases the frequency, rate, or extent of an adaptive immune response.
:   **response to stress**

    Any process that results in a change in state or activity of a cell or an organism (in terms of movement, secretion, enzyme production, gene expression, etc.) as a result of a disturbance in organismal or cellular homeostasis, usually, but not necessarily, exogenous (e.g. temperature, humidity, ionizing radiation).
:   **defense response**

    Reactions, triggered in response to the presence of a foreign body or the occurrence of an injury, which result in restriction of damage to the organism attacked or prevention/recovery from the infection caused by the attack.
:   **immune response**

    Any immune system process that functions in the calibrated response of an organism to a potential internal or invasive threat.
:   **humoral immune response**

    An immune response mediated through a body fluid.
:   **biological\_process**

    Any process specifically pertinent to the functioning of integrated living units: cells, tissues, organs, and organisms. A process is a collection of molecular events with a defined beginning and end.
:   **cellular process**

    Any process that is carried out at the cellular level, but not necessarily restricted to a single cell. For example, cell communication occurs among more than one cell, but occurs at the cellular level.
:   **response to organic substance**

    Any process that results in a change in state or activity of a cell or an organism (in terms of movement, secretion, enzyme production, gene expression, etc.) as a result of an organic substance stimulus.
:   **immunoglobulin mediated immune response**

    An immune response mediated by immunoglobulins, whether cell-bound or in solution.
:   **B cell mediated immunity**

    Any process involved with the carrying out of an immune response by a B cell, through, for instance, the production of antibodies or cytokines, or antigen presentation to T cells.
:   **antigen processing and presentation**

    The process in which an antigen-presenting cell expresses antigen (peptide or lipid) on its cell surface in association with an MHC protein complex.
:   **antigen processing and presentation of exogenous antigen**

    The process in which an antigen-presenting cell expresses antigen (peptide or lipid) of exogenous origin on its cell surface in association with an MHC protein complex.
:   **response to cytokine stimulus**

    Any process that results in a change in state or activity of a cell or an organism (in terms of movement, secretion, enzyme production, gene expression, etc.) as a result of a cytokine stimulus.
:   **response to interferon-beta**

    Any process that results in a change in state or activity of a cell or an organism (in terms of movement, secretion, enzyme production, gene expression, etc.) as a result of an interferon-beta stimulus. Interferon-beta is a type I interferon.
:   **cellular response to interferon-beta**

    Any process that results in a change in state or activity of a cell (in terms of movement, secretion, enzyme production, gene expression, etc.) as a result of an interferon-beta stimulus. Interferon-beta is a type I interferon.
:   **response to chemical stimulus**

    Any process that results in a change in state or activity of a cell or an organism (in terms of movement, secretion, enzyme production, gene expression, etc.) as a result of a chemical stimulus.
:   **single-organism process**

    A biological process that involves only one organism.
:   **single-organism cellular process**

    Any process that is carried out at the cellular level, occurring within a single organism.
:   **innate immune response**

    Innate immune responses are defense responses mediated by germline encoded components that directly recognize components of potential pathogens.
:   **antigen processing and presentation of peptide antigen**

    The process in which an antigen-presenting cell expresses peptide antigen in association with an MHC protein complex on its cell surface, including proteolysis and transport steps for the peptide antigen both prior to and following assembly with the MHC protein complex. The peptide antigen is typically, but not always, processed from an endogenous or exogenous protein.
:   **positive regulation of biological process**

    Any process that activates or increases the frequency, rate or extent of a biological process. Biological processes are regulated by many means; examples include the control of gene expression, protein modification or interaction with a protein or substrate molecule.
:   **regulation of response to stimulus**

    Any process that modulates the frequency, rate or extent of a response to a stimulus. Response to stimulus is a change in state or activity of a cell or an organism (in terms of movement, secretion, enzyme production, gene expression, etc.) as a result of a stimulus.
:   **positive regulation of response to stimulus**

    Any process that activates, maintains or increases the rate of a response to a stimulus. Response to stimulus is a change in state or activity of a cell or an organism (in terms of movement, secretion, enzyme production, gene expression, etc.) as a result of a stimulus.
:   **regulation of immune response**

    Any process that modulates the frequency, rate or extent of the immune response, the immunological reaction of an organism to an immunogenic stimulus.
:   **positive regulation of immune response**

    Any process that activates or increases the frequency, rate or extent of the immune response, the immunological reaction of an organism to an immunogenic stimulus.
:   **regulation of biological process**

    Any process that modulates the frequency, rate or extent of a biological process. Biological processes are regulated by many means; examples include the control of gene expression, protein modification or interaction with a protein or substrate molecule.
:   **response to stimulus**

    Any process that results in a change in state or activity of a cell or an organism (in terms of movement, secretion, enzyme production, gene expression, etc.) as a result of a stimulus. The process begins with detection of the stimulus and ends with a change in state or activity or the cell or organism.
:   **cellular response to stimulus**

    Any process that results in a change in state or activity of a cell (in terms of movement, secretion, enzyme production, gene expression, etc.) as a result of a stimulus. The process begins with detection of the stimulus by a cell and ends with a change in state or activity or the cell.
:   **biological regulation**

    Any process that modulates a measurable attribute of any biological process, quality or function.
:   **cellular response to chemical stimulus**

    Any process that results in a change in state or activity of a cell (in terms of movement, secretion, enzyme production, gene expression, etc.) as a result of a chemical stimulus.
:   **cellular response to organic substance**

    Any process that results in a change in state or activity of a cell (in terms of movement, secretion, enzyme production, gene expression, etc.) as a result of an organic substance stimulus.
:   **cellular response to cytokine stimulus**

    Any process that results in a change in state or activity of a cell (in terms of movement, secretion, enzyme production, gene expression, etc.) as a result of a cytokine stimulus.
:   **all**

    NA
:   **NA**

    NA
:   **innate immune response**

    Innate immune responses are defense responses mediated by germline encoded components that directly recognize components of potential pathogens.
:   **regulation of adaptive immune response**

    Any process that modulates the frequency, rate, or extent of an adaptive immune response.
:   **positive regulation of immune system process**

    Any process that activates or increases the frequency, rate, or extent of an immune system process.
:   **regulation of response to stimulus**

    Any process that modulates the frequency, rate or extent of a response to a stimulus. Response to stimulus is a change in state or activity of a cell or an organism (in terms of movement, secretion, enzyme production, gene expression, etc.) as a result of a stimulus.
:   **humoral immune response**

    An immune response mediated through a body fluid.
:   **positive regulation of response to stimulus**

    Any process that activates, maintains or increases the rate of a response to a stimulus. Response to stimulus is a change in state or activity of a cell or an organism (in terms of movement, secretion, enzyme production, gene expression, etc.) as a result of a stimulus.
:   **regulation of immune response**

    Any process that modulates the frequency, rate or extent of the immune response, the immunological reaction of an organism to an immunogenic stimulus.
:   **response to stimulus**

    Any process that results in a change in state or activity of a cell or an organism (in terms of movement, secretion, enzyme production, gene expression, etc.) as a result of a stimulus. The process begins with detection of the stimulus and ends with a change in state or activity or the cell or organism.
:   **regulation of adaptive immune response**

    Any process that modulates the frequency, rate, or extent of an adaptive immune response.
:   **positive regulation of immune response**

    Any process that activates or increases the frequency, rate or extent of the immune response, the immunological reaction of an organism to an immunogenic stimulus.
:   **regulation of biological process**

    Any process that modulates the frequency, rate or extent of a biological process. Biological processes are regulated by many means; examples include the control of gene expression, protein modification or interaction with a protein or substrate molecule.
:   **biological regulation**

    Any process that modulates a measurable attribute of any biological process, quality or function.
:   **regulation of biological process**

    Any process that modulates the frequency, rate or extent of a biological process. Biological processes are regulated by many means; examples include the control of gene expression, protein modification or interaction with a protein or substrate molecule.
:   **regulation of immune response**

    Any process that modulates the frequency, rate or extent of the immune response, the immunological reaction of an organism to an immunogenic stimulus.
:   **positive regulation of immune response**

    Any process that activates or increases the frequency, rate or extent of the immune response, the immunological reaction of an organism to an immunogenic stimulus.
:   **cellular response to organic substance**

    Any process that results in a change in state or activity of a cell (in terms of movement, secretion, enzyme production, gene expression, etc.) as a result of an organic substance stimulus.
:   **regulation of biological process**

    Any process that modulates the frequency, rate or extent of a biological process. Biological processes are regulated by many means; examples include the control of gene expression, protein modification or interaction with a protein or substrate molecule.
:   **positive regulation of adaptive immune response**

    Any process that activates or increases the frequency, rate, or extent of an adaptive immune response.
:   **response to stress**

    Any process that results in a change in state or activity of a cell or an organism (in terms of movement, secretion, enzyme production, gene expression, etc.) as a result of a disturbance in organismal or cellular homeostasis, usually, but not necessarily, exogenous (e.g. temperature, humidity, ionizing radiation).
:   **regulation of immune system process**

    Any process that modulates the frequency, rate, or extent of an immune system process.
:   **antigen processing and presentation of peptide antigen**

    The process in which an antigen-presenting cell expresses peptide antigen in association with an MHC protein complex on its cell surface, including proteolysis and transport steps for the peptide antigen both prior to and following assembly with the MHC protein complex. The peptide antigen is typically, but not always, processed from an endogenous or exogenous protein.
:   **cellular response to cytokine stimulus**

    Any process that results in a change in state or activity of a cell (in terms of movement, secretion, enzyme production, gene expression, etc.) as a result of a cytokine stimulus.
:   **response to stress**

    Any process that results in a change in state or activity of a cell or an organism (in terms of movement, secretion, enzyme production, gene expression, etc.) as a result of a disturbance in organismal or cellular homeostasis, usually, but not necessarily, exogenous (e.g. temperature, humidity, ionizing radiation).
:   **antigen processing and presentation**

    The process in which an antigen-presenting cell expresses antigen (peptide or lipid) on its cell surface in association with an MHC protein complex.
:   **all**

    NA
:   **response to chemical stimulus**

    Any process that results in a change in state or activity of a cell or an organism (in terms of movement, secretion, enzyme production, gene expression, etc.) as a result of a chemical stimulus.
:   **adaptive immune response based on somatic recombination of immune receptors built from immunoglobulin superfamily domains**

    An immune response based on directed amplification of specific receptors for antigen produced through a somatic diversification process that includes somatic recombination of germline gene segments encoding immunoglobulin superfamily domains, and allowing for enhanced responses upon subsequent exposures to the same antigen (immunological memory). Recombined receptors for antigen encoded by immunoglobulin superfamily domains include T cell receptors and immunoglobulins (antibodies). An example of this is the adaptive immune response found in Mus musculus.

## Help | Hide | Top Help | Show | Top The GO tree — Cellular Components

### HELP

This is one of three sections showing Gene Ontology enrichment of the
current module: in this case for **cellular components**.

The graph shows the hierarchy of the GO categories, their enrichment
for the current module is color coded, and the blue number beside the
category is the minus log ten p-value of the enrichment. (Calculated
using the standard hypergeometric test.) The color of the arrows code
«is a» (cyan) and «part of» relationships.

The tree was built the following way. First all GO terms with more
significant enrichment p-value than 0.05 were collected. Then all
paths from these terms to the root node of the GO tree were included
too. If a GO term is included more than once in the tree, then the
green numbers show 1) the id of the node, this makes it easier to find
other appereances of the term, and 2) the number of appearences.

Note that the same GO category might show up on the graph many
times. This is because the GO was «straightened» for this
graph, i.e. if there are more paths from a GO term to the root node of
the tree, all of them are included. The green numbers

Move the mouse cursor over the terms to get their definition. Clicking
on them takes you to the corresponding Gene Ontology web page.

If you cannot see a graph here at all, that means that there were no
significantly enriched GO categories, at the 0.05 level.

— Click on the *Help* button again to close this help window.

:   **cellular\_component**

    The part of a cell or its extracellular environment in which a gene product is located. A gene product may be located in one or more parts of a cell and its location may be as specific as a particular macromolecular complex, that is, a stable, persistent association of macromolecules that function together.
:   **membrane**

    Double layer of lipid molecules that encloses all cells, and, in eukaryotes, many organelles; may be a single or double lipid bilayer; also includes associated proteins.
:   **cell**

    The basic structural and functional unit of all organisms. Includes the plasma membrane and any external encapsulating structures such as the cell wall and cell envelope.
:   **macromolecular complex**

    A stable assembly of two or more macromolecules, i.e. proteins, nucleic acids, carbohydrates or lipids, in which the constituent parts function together.
:   **MHC protein complex**

    A transmembrane protein complex composed of an MHC alpha chain and, in most cases, either an MHC class II beta chain or an invariant beta2-microglobin chain, and with or without a bound peptide, lipid, or polysaccharide antigen.
:   **protein complex**

    Any macromolecular complex composed of two or more polypeptide subunits, which may or may not be identical. Protein complexes may have other associated non-protein prosthetic groups, such as nucleotides, metal ions or other small molecules.
:   **membrane part**

    Any constituent part of a membrane, a double layer of lipid molecules that encloses all cells, and, in eukaryotes, many organelles; may be a single or double lipid bilayer; also includes associated proteins.
:   **plasma membrane part**

    Any constituent part of the plasma membrane, the membrane surrounding a cell that separates the cell from its external environment. It consists of a phospholipid bilayer and associated proteins.
:   **cell part**

    Any constituent part of a cell, the basic structural and functional unit of all organisms.
:   **cell periphery**

    The part of a cell encompassing the cell cortex, the plasma membrane, and any external encapsulating structures.
:   **all**

    NA
:   **NA**

    NA
:   **plasma membrane part**

    Any constituent part of the plasma membrane, the membrane surrounding a cell that separates the cell from its external environment. It consists of a phospholipid bilayer and associated proteins.
:   **cell periphery**

    The part of a cell encompassing the cell cortex, the plasma membrane, and any external encapsulating structures.
:   **cellular\_component**

    The part of a cell or its extracellular environment in which a gene product is located. A gene product may be located in one or more parts of a cell and its location may be as specific as a particular macromolecular complex, that is, a stable, persistent association of macromolecules that function together.
:   **cell part**

    Any constituent part of a cell, the basic structural and functional unit of all organisms.
:   **cell part**

    Any constituent part of a cell, the basic structural and functional unit of all organisms.
:   **protein complex**

    Any macromolecular complex composed of two or more polypeptide subunits, which may or may not be identical. Protein complexes may have other associated non-protein prosthetic groups, such as nucleotides, metal ions or other small molecules.
:   **cellular\_component**

    The part of a cell or its extracellular environment in which a gene product is located. A gene product may be located in one or more parts of a cell and its location may be as specific as a particular macromolecular complex, that is, a stable, persistent association of macromolecules that function together.

## Help | Hide | Top Help | Show | Top The GO tree — Molecular Function

### HELP

This is one of three sections showing Gene Ontology enrichment of the
current module: in this case for **molecular function**.

The graph shows the hierarchy of the GO categories, their enrichment
for the current module is color coded, and the blue number beside the
category is the minus log ten p-value of the enrichment. (Calculated
using the standard hypergeometric test.) The color of the arrows code
«is a» (cyan) and «part of» relationships.

The tree was built the following way. First all GO terms with more
significant enrichment p-value than 0.05 were collected. Then all
paths from these terms to the root node of the GO tree were included
too. If a GO term is included more than once in the tree, then the
green numbers show 1) the id of the node, this makes it easier to find
other appereances of the term, and 2) the number of appearences.

Note that the same GO category might show up on the graph many
times. This is because the GO was «straightened» for this
graph, i.e. if there are more paths from a GO term to the root node of
the tree, all of them are included. The green numbers

Move the mouse cursor over the terms to get their definition. Clicking
on them takes you to the corresponding Gene Ontology web page.

If you cannot see a graph here at all, that means that there were no
significantly enriched GO categories, at the 0.05 level.

— Click on the *Help* button again to close this help window.

:   **molecular\_function**

    Elemental activities, such as catalysis or binding, describing the actions of a gene product at the molecular level. A given gene product may exhibit one or more molecular functions.
:   **binding**

    The selective, non-covalent, often stoichiometric, interaction of a molecule with one or more specific sites on another molecule.
:   **beta-2-microglobulin binding**

    Interacting selectively and non-covalently with beta-2-microglobulin.
:   **amide binding**

    Interacting selectively and non-covalently with an amide, any derivative of an oxoacid in which an acidic hydroxy group has been replaced by an amino or substituted amino group.
:   **peptide binding**

    Interacting selectively and non-covalently with peptides, any of a group of organic compounds comprising two or more amino acids linked by peptide bonds.
:   **all**

    NA
:   **NA**

    NA

## Help | Hide | Top Help | Show | Top GO BP test for over-representation

### HELP

List of all enriched GO categories (biological processes), at the 0.05
p-value level.

The columns:

- **ExpCount** is the expected count of genes in the
  module annotated with the given GO term, just by chance.
- **Count**
  is the number of genes in the module annotated with the given GO
  term.
- **Size** is the total number of genes (in our universe)
  annotated with the GO term.

Clicking on **Count** shows the genes that drive the
enrichment. You can also click on the individual numbers in
the **Count** column, to show the driving genes for that individual
GO category.

Clicking on the GO identifiers takes you to the Gene Ontology web
pages.

— Click on the *Help* button again to close this help window.

| Id | Pvalue | ExpCount | Count | Size | Term |
| --- | --- | --- | --- | --- | --- |
| GO:0006955 | 1.494e-12 | 6.023 | 33 C1qa, C1qbp, C2, C5ar1, Ccl7, Ccr4, Csf1, Cyba, Fcer2a, Gbp2b, Gbp5, H2-Aa, H2-D1, H2-DMa, H2-Eb1, H2-Q7, H60b, Hrg, Ifit1, Ifit2, Igj, Irgm1, Mif, Oas1b, Oas3, Plekha1, Rbck1, Serping1, Spon2, Stat1, Tap1, Tap2, Traf6 | 638 | immune response |
| GO:0045087 | 7.242e-10 | 2.709 | 21 C1qa, C1qbp, C2, Csf1, Cyba, Gbp2b, Gbp5, H2-Aa, H2-Eb1, H60b, Ifit1, Ifit2, Irgm1, Mif, Oas1b, Oas3, Serping1, Spon2, Stat1, Tap1, Tap2 | 287 | innate immune response |
| GO:0006952 | 2.102e-09 | 6.382 | 30 Ahsg, C1qa, C1qbp, C2, C5ar1, Ccl7, Ccr4, Csf1, Cyba, Ddit4, Gbp2b, Gbp5, H2-Aa, H2-Eb1, H60b, Hrg, Ifi47, Ifit1, Ifit2, Irgm1, Mif, Mvk, Oas1b, Oas3, Selp, Serping1, Spon2, Stat1, Tap1, Tap2 | 676 | defense response |
| GO:0002376 | 9.285e-09 | 11.93 | 40 C1qa, C1qbp, C2, C5ar1, Ccl7, Ccr4, Csf1, Cyba, Ddit4, Fcer2a, Gbp2b, Gbp5, H2-Aa, H2-D1, H2-DMa, H2-Eb1, H2-Q7, H60b, Hrg, Ifit1, Ifit2, Igj, Ihh, Irgm1, Lmo4, Mif, Oas1b, Oas3, Plekha1, Rac1, Rbck1, Selp, Serping1, Slfn1, Sp1, Spon2, Stat1, Tap1, Tap2, Traf6 | 1264 | immune system process |
| GO:0048002 | 4.633e-06 | 0.3587 | 8 H2-Aa, H2-D1, H2-DMa, H2-Eb1, H2-Q7, Tap1, Tap2, Traf6 | 38 | antigen processing and presentation of peptide antigen |
| GO:0002252 | 6.771e-06 | 3.578 | 19 C1qa, C1qbp, C2, Ddit4, Fcer2a, Gbp2b, H2-D1, H2-DMa, H2-Eb1, H60b, Ifit1, Ifit2, Mif, Oas1b, Oas3, Serping1, Tap1, Tap2, Traf6 | 379 | immune effector process |
| GO:0002449 | 2.822e-05 | 1.369 | 12 C1qa, C1qbp, C2, Fcer2a, H2-D1, H2-DMa, H2-Eb1, H60b, Serping1, Tap1, Tap2, Traf6 | 145 | lymphocyte mediated immunity |
| GO:0002684 | 5.250e-05 | 4.059 | 19 C1qa, C1qbp, C2, C5ar1, Ccl7, Fcer2a, H2-Aa, H2-D1, H2-DMa, Hrg, Ihh, Mif, Plekha1, Rbck1, Selp, Serping1, Tap1, Tap2, Traf6 | 430 | positive regulation of immune system process |
| GO:0050778 | 9.575e-05 | 2.558 | 15 C1qa, C1qbp, C2, C5ar1, Fcer2a, H2-D1, H2-DMa, Hrg, Mif, Plekha1, Rbck1, Serping1, Tap1, Tap2, Traf6 | 271 | positive regulation of immune response |
| GO:0002250 | 1.899e-04 | 1.624 | 12 C1qa, C1qbp, C2, Fcer2a, H2-D1, H2-DMa, H2-Eb1, Mif, Serping1, Tap1, Tap2, Traf6 | 172 | adaptive immune response |
| GO:0019882 | 2.030e-04 | 0.5664 | 8 H2-Aa, H2-D1, H2-DMa, H2-Eb1, H2-Q7, Tap1, Tap2, Traf6 | 60 | antigen processing and presentation |
| GO:0002443 | 4.214e-04 | 1.746 | 12 C1qa, C1qbp, C2, Fcer2a, H2-D1, H2-DMa, H2-Eb1, H60b, Serping1, Tap1, Tap2, Traf6 | 185 | leukocyte mediated immunity |
| GO:0006959 | 6.913e-04 | 0.6608 | 8 C1qa, C1qbp, C2, Fcer2a, H2-DMa, H2-Eb1, Igj, Serping1 | 70 | humoral immune response |
| GO:0002460 | 7.790e-04 | 1.51 | 11 C1qa, C1qbp, C2, Fcer2a, H2-D1, H2-DMa, H2-Eb1, Serping1, Tap1, Tap2, Traf6 | 160 | adaptive immune response based on somatic recombination of immune receptors built from immunoglobulin superfamily domains |
| GO:0002682 | 9.990e-04 | 6.448 | 22 C1qa, C1qbp, C2, C5ar1, Ccl7, Csf1, Fcer2a, H2-Aa, H2-D1, H2-DMa, Hrg, Ifit1, Ihh, Mif, Plekha1, Rbck1, Selp, Serping1, Slfn1, Tap1, Tap2, Traf6 | 683 | regulation of immune system process |
| GO:0002821 | 2.681e-03 | 0.5475 | 7 Fcer2a, H2-D1, H2-DMa, Mif, Tap1, Tap2, Traf6 | 58 | positive regulation of adaptive immune response |
| GO:0002455 | 2.690e-03 | 0.3493 | 6 C1qa, C1qbp, C2, Fcer2a, H2-Eb1, Serping1 | 37 | humoral immune response mediated by circulating immunoglobulin |
| GO:0002478 | 2.909e-03 | 0.1982 | 5 H2-Aa, H2-DMa, H2-Eb1, Tap2, Traf6 | 21 | antigen processing and presentation of exogenous peptide antigen |
| GO:0050776 | 4.328e-03 | 3.446 | 15 C1qa, C1qbp, C2, C5ar1, Fcer2a, H2-D1, H2-DMa, Hrg, Mif, Plekha1, Rbck1, Serping1, Tap1, Tap2, Traf6 | 365 | regulation of immune response |
| GO:0035458 | 4.734e-03 | 0.2171 | 5 Gbp2b, Gm4951, Ifi204, Ifit1, Stat1 | 23 | cellular response to interferon-beta |
| GO:0019884 | 9.041e-03 | 0.2454 | 5 H2-Aa, H2-DMa, H2-Eb1, Tap2, Traf6 | 26 | antigen processing and presentation of exogenous antigen |
| GO:0006950 | 9.853e-03 | 18.69 | 39 Ahsg, C1qa, C1qbp, C2, C5ar1, Cask, Ccl7, Ccr4, Csf1, Cyba, Ddit4, Gbp2b, Gbp5, H2-Aa, H2-Eb1, H60b, Hrg, Ifi204, Ifi47, Ifit1, Ifit2, Irgm1, Mif, Mvk, Nabp1, Oas1b, Oas3, Plekha1, Prmt6, Rac1, Selp, Serpine2, Serping1, Spon2, Stat1, Tap1, Tap2, Traf6, Trim28 | 1980 | response to stress |
| GO:0034097 | 1.015e-02 | 3.229 | 14 Ccr4, Gbp2b, Gbp5, Gm4951, H2-Aa, H2-Eb1, Ifi204, Ifit1, Ifit2, Rqcd1, Selp, Star, Stat1, Traf6 | 342 | response to cytokine stimulus |
| GO:0035456 | 1.329e-02 | 0.2643 | 5 Gbp2b, Gm4951, Ifi204, Ifit1, Stat1 | 28 | response to interferon-beta |
| GO:0019886 | 1.631e-02 | 0.1322 | 4 H2-Aa, H2-DMa, H2-Eb1, Traf6 | 14 | antigen processing and presentation of exogenous peptide antigen via MHC class II |
| GO:0019885 | 1.870e-02 | 0.0472 | 3 H2-D1, Tap1, Tap2 | 5 | antigen processing and presentation of endogenous peptide antigen via MHC class I |
| GO:0071345 | 2.138e-02 | 2.539 | 12 Ccr4, Gbp2b, Gbp5, Gm4951, Ifi204, Ifit1, Ifit2, Rqcd1, Selp, Star, Stat1, Traf6 | 269 | cellular response to cytokine stimulus |
| GO:0002824 | 2.335e-02 | 0.5003 | 6 Fcer2a, H2-D1, H2-DMa, Tap1, Tap2, Traf6 | 53 | positive regulation of adaptive immune response based on somatic recombination of immune receptors built from immunoglobulin superfamily domains |
| GO:0016064 | 2.574e-02 | 0.7647 | 7 C1qa, C1qbp, C2, Fcer2a, H2-DMa, H2-Eb1, Serping1 | 81 | immunoglobulin mediated immune response |
| GO:0019724 | 3.024e-02 | 0.7835 | 7 C1qa, C1qbp, C2, Fcer2a, H2-DMa, H2-Eb1, Serping1 | 83 | B cell mediated immunity |
| GO:0051704 | 3.710e-02 | 5.721 | 18 C1qbp, C5ar1, Ddit4, Fcer2a, Gbp2b, Hcfc1, Hrg, Ifit1, Ifit2, Mif, Oas1b, Oas3, Serpine2, Sp1, Stat1, Tap1, Tap2, Traf6 | 606 | multi-organism process |
| GO:0002495 | 3.782e-02 | 0.1605 | 4 H2-Aa, H2-DMa, H2-Eb1, Traf6 | 17 | antigen processing and presentation of peptide antigen via MHC class II |

## Help | Hide | Top Help | Show | Top GO CC test for over-representation

### HELP

List of all enriched GO categories (cellular components), at the 0.05
p-value level.

The columns:

- **ExpCount** is the expected count of genes in the
  module annotated with the given GO term, just by chance.
- **Count**
  is the number of genes in the module annotated with the given GO
  term.
- **Size** is the total number of genes (in our universe)
  annotated with the GO term.

Clicking on **Count** shows the genes that drive the
enrichment. You can also click on the individual numbers in
the **Count** column, to show the driving genes for that individual
GO category.

Clicking on the GO identifiers takes you to the Gene Ontology web
pages.

— Click on the *Help* button again to close this help window.

| Id | Pvalue | ExpCount | Count | Size | Term |
| --- | --- | --- | --- | --- | --- |
| GO:0042611 | 5.402e-04 | 0.2168 | 5 H2-Aa, H2-D1, H2-DMa, H2-Eb1, H2-Q7 | 23 | MHC protein complex |
| GO:0042613 | 2.476e-02 | 0.09427 | 3 H2-Aa, H2-DMa, H2-Eb1 | 10 | MHC class II protein complex |

## Help | Hide | Top Help | Show | Top GO MF test for over-representation

### HELP

List of all enriched GO categories (molecular function), at the 0.05
p-value level.

The columns:

- **ExpCount** is the expected count of genes in the
  module annotated with the given GO term, just by chance.
- **Count**
  is the number of genes in the module annotated with the given GO
  term.
- **Size** is the total number of genes (in our universe)
  annotated with the GO term.

Clicking on **Count** shows the genes that drive the
enrichment. You can also click on the individual numbers in
the **Count** column, to show the driving genes for that individual
GO category.

Clicking on the GO identifiers takes you to the Gene Ontology web
pages.

— Click on the *Help* button again to close this help window.

| Id | Pvalue | ExpCount | Count | Size | Term |
| --- | --- | --- | --- | --- | --- |
| GO:0030881 | 3.897e-04 | 0.08469 | 4 H2-D1, H2-Q4, H2-Q6, H2-Q7 | 9 | beta-2-microglobulin binding |
| GO:0042277 | 1.605e-03 | 1.562 | 10 Anpep, Ctsl, H2-Aa, H2-D1, H2-Q4, H2-Q6, H2-Q7, Ppic, Tap1, Tap2 | 166 | peptide binding |
| GO:0033218 | 2.199e-03 | 1.619 | 10 Anpep, Ctsl, H2-Aa, H2-D1, H2-Q4, H2-Q6, H2-Q7, Ppic, Tap1, Tap2 | 172 | amide binding |
| GO:0003823 | 1.684e-02 | 0.1976 | 4 H2-Aa, Igj, Tap1, Tap2 | 21 | antigen binding |
| GO:0042605 | 3.945e-02 | 0.0941 | 3 H2-Aa, Tap1, Tap2 | 10 | peptide antigen binding |

## Help | Hide | Top Help | Show | Top KEGG Pathway test for over-representation

### HELP

List of all enriched KEGG pathways, at the 0.05
p-value level.

The columns:

- **ExpCount** is the expected count of genes in the
  module annotated with the given KEGG pathway, just by chance.
- **Count**
  is the number of genes in the module annotated with the given KEGG
  pathway.
- **Size** is the total number of genes (in our universe)
  annotated with the KEGG pathway.

Clicking on **Count** shows the genes that drive the
enrichment. You can also click on the individual numbers in
the **Count** column, to show the driving genes for that individual
KEGG pathway.

Clicking on the KEGG identifiers takes you to the KEGG web site.

— Click on the *Help* button again to close this help window.

| Id | Pvalue | ExpCount | Count | Size | Term |
| --- | --- | --- | --- | --- | --- |
| 04612 | 7.283e-10 | 0.7799 | 12 Ctsl, H2-Aa, H2-D1, H2-DMa, H2-Eb1, H2-Q6, H2-Q7, H2-T22, H2-T3, Rfxap, Tap1, Tap2 | 66 | Antigen processing and presentation |
| 05330 | 8.998e-07 | 0.449 | 8 H2-Aa, H2-D1, H2-DMa, H2-Eb1, H2-Q6, H2-Q7, H2-T22, H2-T3 | 38 | Allograft rejection |
| 04145 | 1.127e-06 | 1.761 | 13 Ctsl, Cyba, H2-Aa, H2-D1, H2-DMa, H2-Eb1, H2-Q6, H2-Q7, H2-T22, H2-T3, Rac1, Tap1, Tap2 | 149 | Phagosome |
| 05416 | 1.127e-06 | 0.8862 | 10 H2-Aa, H2-D1, H2-DMa, H2-Eb1, H2-Q6, H2-Q7, H2-T22, H2-T3, Myh15, Rac1 | 75 | Viral myocarditis |
| 05320 | 1.349e-06 | 0.4727 | 8 H2-Aa, H2-D1, H2-DMa, H2-Eb1, H2-Q6, H2-Q7, H2-T22, H2-T3 | 40 | Autoimmune thyroid disease |
| 05332 | 1.645e-06 | 0.4845 | 8 H2-Aa, H2-D1, H2-DMa, H2-Eb1, H2-Q6, H2-Q7, H2-T22, H2-T3 | 41 | Graft-versus-host disease |
| 04940 | 2.426e-06 | 0.5081 | 8 H2-Aa, H2-D1, H2-DMa, H2-Eb1, H2-Q6, H2-Q7, H2-T22, H2-T3 | 43 | Type I diabetes mellitus |
| 05150 | 7.005e-05 | 0.5317 | 7 C1qa, C2, C5ar1, H2-Aa, H2-DMa, H2-Eb1, Selp | 45 | Staphylococcus aureus infection |
| 04514 | 1.076e-03 | 1.453 | 9 H2-Aa, H2-D1, H2-DMa, H2-Eb1, H2-Q6, H2-Q7, H2-T22, H2-T3, Selp | 123 | Cell adhesion molecules (CAMs) |
| 05140 | 6.160e-03 | 0.709 | 6 Cyba, H2-Aa, H2-DMa, H2-Eb1, Stat1, Traf6 | 60 | Leishmaniasis |


### HELP

List of all enriched miRNA families, at the 0.05
p-value level.

The columns:

- **ExpCount** is the expected count of genes in the
  module regulated by the given miRNA family, just by chance.
- **Count**
  is the number of genes in the module regulated by the given miRNA
  family.
- **Size** is the total number of genes (in our universe)
  regulated with the given miRNA family.

Clicking on **Count** shows the genes that drive the
enrichment. You can also click on the individual numbers in
the **Count** column, to show the driving genes for that individual
miRNA family.

The miRNA regulation data was taken from the

Top


### HELP

p-value level.

The columns:

- **ExpCount** is the expected number of genes in the- **Count**- **Size** is the total number of genes (in our universe)

Clicking on **Count** shows the genes that drive the
enrichment. You can also click on the individual numbers in
the **Count** column, to show the driving genes for that individual

— Click on the *Help* button again to close this help window.

## Help | Hide | Top Help | Show | Top Genes

### HELP

A list of all genes in the current module, in alphabetical order. The
size of the text corresponds to the gene scores.

Note that some gene symbols may show up more than once, if many
probes match the same Entrez gene.

Genes with no Entrez mapping are given separately, with their
Affymetrics probe ID.

— Click on the *Help* button again to close this help window.

### Genes Symbol

, score:

AhsgUnknown, score: 0.64
Arhgap6Unknown, score: 0.52
Cep131Unknown, score: 0.7
Serping1Unknown, score: 0.56
C1qaUnknown, score: 0.62
C1qbpUnknown, score: 0.54
C2Unknown, score: 0.64
C5ar1Unknown, score: 0.52
CaskUnknown, score: 0.62
Ccr4Unknown, score: 0.55
Csf1Unknown, score: 0.54
CtslUnknown, score: 0.6
CybaUnknown, score: 0.63
Fcer2aUnknown, score: 0.63
Gbp2bUnknown, score: 0.85
Hist1h1dUnknown, score: 0.53
H2-AaUnknown, score: 0.57
H2-D1Unknown, score: 0.59
H2-Eb1Unknown, score: 0.73
H2-DMaUnknown, score: 0.63
H2-Q4Unknown, score: 0.57
H2-Q7Unknown, score: 0.61
H2-T3Unknown, score: 0.63
H2-T22Unknown, score: 0.73
H2-T3Unknown, score: 0.63
Hao1Unknown, score: 0.54
Hcfc1Unknown, score: 0.56
Idh3gUnknown, score: 0.62
Ier5Unknown, score: 0.95
Irgm1Unknown, score: 0.64
Ifi204Unknown, score: 0.87
Ifi47Unknown, score: 0.77
Ifit1Unknown, score: 0.76
Ifit2Unknown, score: 0.84
IgjUnknown, score: 0.94
IhhUnknown, score: 0.57
IppUnknown, score: 0.75
Irx3Unknown, score: 0.83
Klra10Unknown, score: 0.63
AnpepUnknown, score: 0.56
Lmo4Unknown, score: 0.8
MifUnknown, score: 0.56
Cd200Unknown, score: 0.82
MvkUnknown, score: 0.55
Olfr33Unknown, score: 0.54
PpicUnknown, score: 0.71
Psmb1Unknown, score: 0.7
PtprbUnknown, score: 0.62
Rac1Unknown, score: 0.58
Rps8Unknown, score: 0.67
Ccl7Unknown, score: 0.61
SelpUnknown, score: 0.54
Slc9a1Unknown, score: 0.75
Slfn1Unknown, score: 0.61
Sumo3Unknown, score: 0.69
Sp1Unknown, score: 0.67
Serpine2Unknown, score: 0.62
SrprbUnknown, score: 0.66
StarUnknown, score: 0.98
Stat1Unknown, score: 0.96
TankUnknown, score: 0.88
Tap1Unknown, score: 0.52
Tap2Unknown, score: 0.64
Ift88Unknown, score: 0.8
Trim28Unknown, score: 0.53
Traf6Unknown, score: 0.61
Txn1Unknown, score: 0.69
WapUnknown, score: 0.61
Oas1gUnknown, score: 0.67
Oas1bUnknown, score: 0.87
Rbck1Unknown, score: 0.76
Ubl3Unknown, score: 0.7
UevldUnknown, score: 0.8
Crlf3Unknown, score: 0.97
Hes6Unknown, score: 0.91
RalaUnknown, score: 0.81
Noa1Unknown, score: 0.78
Supt20Unknown, score: 0.59
Rqcd1Unknown, score: 0.62
NelfbUnknown, score: 0.96
Doc2gUnknown, score: 0.8
WtapUnknown, score: 0.93
Gpr88Unknown, score: 0.72
Kbtbd4Unknown, score: 0.72
Loh12cr1Unknown, score: 0.84
2310009B15RikUnknown, score: 0.63
1810026J23RikUnknown, score: 0.6
Gdpd2Unknown, score: 0.76
Lypd6bUnknown, score: 0.78
Cybrd1Unknown, score: 0.65
Stra6lUnknown, score: 0.91
Samd4Unknown, score: 0.86
Ddit4Unknown, score: 0.53
Pcgf5Unknown, score: 0.73
Tusc3Unknown, score: 0.53
GhdcUnknown, score: 0.6
Gpr63Unknown, score: 0.65
Vmn1r65Unknown, score: 0.79
Cacng7Unknown, score: 0.65
Rbm5Unknown, score: 0.72
GlrxUnknown, score: 0.67
Trim33Unknown, score: 0.55
HrgUnknown, score: 0.55
Prmt6Unknown, score: 0.88
Spon2Unknown, score: 0.79
Plekha1Unknown, score: 0.53
Mal2Unknown, score: 0.52
Nabp1Unknown, score: 0.64
Acat1Unknown, score: 0.57
Amhr2Unknown, score: 0.61
H2-Q6Unknown, score: 0.57
RfxapUnknown, score: 0.71
Gipc3Unknown, score: 0.58
Clic6Unknown, score: 0.75
Rnf26Unknown, score: 0.7
Zfp609Unknown, score: 0.56
Rundc1Unknown, score: 0.54
Ska3Unknown, score: 0.62
Phf11aUnknown, score: 0.69
Zfp7Unknown, score: 0.78
A730017C20RikUnknown, score: 0.86
Tstd1Unknown, score: 0.56
Rap1gds1Unknown, score: 0.83
Gbp5Unknown, score: 0.85
Coq3Unknown, score: 0.72
Sesn2Unknown, score: 0.69
Myo1hUnknown, score: 0.57
Cbr4Unknown, score: 0.82
5730507C01RikUnknown, score: 0.59
PhgdhUnknown, score: 0.79
Btbd7Unknown, score: 0.74
Adamts19Unknown, score: 0.82
Gm4951Unknown, score: 0.9
BivmUnknown, score: 0.69
Rhobtb2Unknown, score: 0.72
Oas3Unknown, score: 0.61
Oas1hUnknown, score: 0.61
GphnUnknown, score: 0.57
Lhfpl4Unknown, score: 0.53
Pitpnm3Unknown, score: 0.69
Adcy1Unknown, score: 0.62
Zc3h12bUnknown, score: 1
620750Unknown, score: 0.89
622531Unknown, score: 0.74
H60bUnknown, score: 0.82
Myh15Unknown, score: 0.73
671242Unknown, score: 0.55
Zglp1Unknown, score: 0.54
Cep85lUnknown, score: 0.66
Ildr2Unknown, score: 0.8
Eif3j2Unknown, score: 0.71
100503235Unknown, score: 0.61
101056659Unknown, score: 0.61

## Help | Hide | Top Help | Show | Top Conditions

### HELP

Conditions in the module, given in the same order as on the expression
plot above. Red color means over-expression, green under-expression in
the given condition.

The barplot below shows the condition (sample) scores. A separate bar
is shown for each sample, its height is the corresponding score of the
sample in the module. The red and green numbers on the bars are the
sample scores expressed in percents, i.e. 100% is 1.0.

The red and green lines show the module thresholds, samples above
the red line and below the green line are included in the module.

The different experiments that were part of the study, are separated
by dashed vertical lines.

— Click on the *Help* button again to close this help window.

Id

SWR\_J-ATE\_135

A\_J-ISO\_123

PL\_J-ISO\_77

SM\_J-ISO\_44

SWR\_J-CTR\_134

NOD\_ShiLtJ-ATE\_23

NOD\_ShiLtJ-CTR\_19

SWR\_J-ATE\_139

FVB\_NJ-CTR\_52

CBA\_J-ISO\_128

A\_J-CTR\_120

NZB\_BLNJ-ATE\_88

SM\_J-ISO\_46

PL\_J-ISO\_78

NOD\_ShiLtJ-ATE\_22

FVB\_NJ-CTR\_51

NZB\_BLNJ-ISO\_90

NOD\_ShiLtJ-CTR\_21

SWR\_J-CTR\_137

A\_J-ATE\_117

DBA\_2J-ISO\_7

FVB\_NJ-CTR\_56

FVB\_NJ-ATE\_57

LP\_J-ISO\_99

DBA\_2J-CTR\_2

I\_LnJ-ATE\_154

DBA\_2J-ISO\_8

C3H\_HeJ-ATE\_61

I\_LnJ-CTR\_153

I\_LnJ-CTR\_150

SM\_J-CTR\_41

SM\_J-ATE\_43

FVB\_NJ-ISO\_58

SM\_J-ATE\_49

BALB\_cByJ-ISO\_145

C57BL\_6J-ISO\_50

I\_LnJ-ATE\_146

SM\_J-CTR\_48

SM\_J-ATE\_42

© 2015 Computational Biology Group, Department of Medical Genetics,
University of Lausanne, Switzerland
