# Supplementary material for: RNAseq analysis of heart tissue from mice treated with atenolol and isoproterenol reveals a reciprocal transcriptional response
Source: BMC Genomics. 2016 Sep 7;17(1):717. doi: 10.1186/s12864-016-3059-6 (PMC5015234; doi:10.1186/s12864-016-3059-6)
Supplement: Additional file 4: — Supplementary data. Compressed HTML files of 98 expression modules annotated for genes, strains and GO or KEGG terms (see Additional file 3 for navigation details). (GZ 11006 kb) [file 12864_2016_3059_MOESM4_ESM.gz › modules.html/module-86.html]

Module #86, TG: 1.5, TC: 0.375, 1101 probes, 1101 Entrez genes, 136 conditions

# Previous module | Next module Module #86, TG: 1.5, TC: 0.375, 1101 probes, 1101 Entrez genes, 136 conditions

- Module tree/table

- Expression data

- The BP GO tree
- The CC GO tree
- The MF GO tree

- GO BP enrichment
- GO CC enrichment
- GO MF enrichment
- KEGG enrichment
- miRNA enrichment

- Genes
- Conditions

## Help | Hide | Top Help | Show | Top Expression data

### HELP

The image plot shows the color-coded level of gene expression, for the
genes and conditions in a given transcription module. The genes are on
the horizontal, the conditions on the vertical axis.

The genes are ordered according to their ISA gene scores, similarly
the conditions are ordered according to their condition scores. The
score of a gene means the «degree of inclusion» in
the module: a high score gene is essential in the module.

Condition scores can also be negative, that means that the genes of
the module are all down-regulated in the condition. Here the absolute
value of the score gives the «degree of inclusion».

The plots above and beside the expression matrix show the gene scores
and condition scores, respectively.

Note that the plot is interactive, you can see the name of the gene
and condition under the mouse cursor.

The expression matrix was normalized to have mean zero and standard
deviation one for every gene separately across all conditions
(i.e. not just for the conditions in the module).

— Click on the *Help* button again to close this help window.

Gene:   
Condition:


Under-expression is coded with green,
over-expression with red color.

## Help | Hide | Top Help | Show | Top The GO tree — Biological processes

### HELP

This is one of three sections showing Gene Ontology enrichment of the
current module: in this case for **biological processes**.

The graph shows the hierarchy of the GO categories, their enrichment
for the current module is color coded, and the blue number beside the
category is the minus log ten p-value of the enrichment. (Calculated
using the standard hypergeometric test.) The color of the arrows code
«is a» (cyan) and «part of» relationships.

The tree was built the following way. First all GO terms with more
significant enrichment p-value than 0.05 were collected. Then all
paths from these terms to the root node of the GO tree were included
too. If a GO term is included more than once in the tree, then the
green numbers show 1) the id of the node, this makes it easier to find
other appereances of the term, and 2) the number of appearences.

Note that the same GO category might show up on the graph many
times. This is because the GO was «straightened» for this
graph, i.e. if there are more paths from a GO term to the root node of
the tree, all of them are included. The green numbers

Move the mouse cursor over the terms to get their definition. Clicking
on them takes you to the corresponding Gene Ontology web page.

If you cannot see a graph here at all, that means that there were no
significantly enriched GO categories, at the 0.05 level.

— Click on the *Help* button again to close this help window.

## Help | Hide | Top Help | Show | Top The GO tree — Cellular Components

### HELP

This is one of three sections showing Gene Ontology enrichment of the
current module: in this case for **cellular components**.

The graph shows the hierarchy of the GO categories, their enrichment
for the current module is color coded, and the blue number beside the
category is the minus log ten p-value of the enrichment. (Calculated
using the standard hypergeometric test.) The color of the arrows code
«is a» (cyan) and «part of» relationships.

The tree was built the following way. First all GO terms with more
significant enrichment p-value than 0.05 were collected. Then all
paths from these terms to the root node of the GO tree were included
too. If a GO term is included more than once in the tree, then the
green numbers show 1) the id of the node, this makes it easier to find
other appereances of the term, and 2) the number of appearences.

Note that the same GO category might show up on the graph many
times. This is because the GO was «straightened» for this
graph, i.e. if there are more paths from a GO term to the root node of
the tree, all of them are included. The green numbers

Move the mouse cursor over the terms to get their definition. Clicking
on them takes you to the corresponding Gene Ontology web page.

If you cannot see a graph here at all, that means that there were no
significantly enriched GO categories, at the 0.05 level.

— Click on the *Help* button again to close this help window.

:   **extracellular region**

    The space external to the outermost structure of a cell. For cells without external protective or external encapsulating structures this refers to space outside of the plasma membrane. This term covers the host cell environment outside an intracellular parasite.
:   **extracellular region part**

    Any constituent part of the extracellular region, the space external to the outermost structure of a cell. For cells without external protective or external encapsulating structures this refers to space outside of the plasma membrane. This term covers constituent parts of the host cell environment outside an intracellular parasite.
:   **all**

    NA
:   **NA**

    NA
:   **all**

    NA

## Help | Hide | Top Help | Show | Top The GO tree — Molecular Function

### HELP

This is one of three sections showing Gene Ontology enrichment of the
current module: in this case for **molecular function**.

The graph shows the hierarchy of the GO categories, their enrichment
for the current module is color coded, and the blue number beside the
category is the minus log ten p-value of the enrichment. (Calculated
using the standard hypergeometric test.) The color of the arrows code
«is a» (cyan) and «part of» relationships.

The tree was built the following way. First all GO terms with more
significant enrichment p-value than 0.05 were collected. Then all
paths from these terms to the root node of the GO tree were included
too. If a GO term is included more than once in the tree, then the
green numbers show 1) the id of the node, this makes it easier to find
other appereances of the term, and 2) the number of appearences.

Note that the same GO category might show up on the graph many
times. This is because the GO was «straightened» for this
graph, i.e. if there are more paths from a GO term to the root node of
the tree, all of them are included. The green numbers

Move the mouse cursor over the terms to get their definition. Clicking
on them takes you to the corresponding Gene Ontology web page.

If you cannot see a graph here at all, that means that there were no
significantly enriched GO categories, at the 0.05 level.

— Click on the *Help* button again to close this help window.

:   **G-protein coupled receptor binding**

    Interacting selectively and non-covalently with a G-protein coupled receptor.
:   **molecular\_function**

    Elemental activities, such as catalysis or binding, describing the actions of a gene product at the molecular level. A given gene product may exhibit one or more molecular functions.
:   **receptor binding**

    Interacting selectively and non-covalently with one or more specific sites on a receptor molecule, a macromolecule that undergoes combination with a hormone, neurotransmitter, drug or intracellular messenger to initiate a change in cell function.
:   **cytokine activity**

    Functions to control the survival, growth, differentiation and effector function of tissues and cells.
:   **cytokine receptor binding**

    Interacting selectively and non-covalently with a cytokine receptor.
:   **binding**

    The selective, non-covalent, often stoichiometric, interaction of a molecule with one or more specific sites on another molecule.
:   **chemokine activity**

    The function of a family of chemotactic pro-inflammatory activation-inducible cytokines acting primarily upon hemopoietic cells in immunoregulatory processes; all chemokines possess a number of conserved cysteine residues involved in intramolecular disulfide bond formation.
:   **chemokine receptor binding**

    Interacting selectively and non-covalently with any chemokine receptor.
:   **all**

    NA
:   **NA**

    NA
:   **all**

    NA
:   **chemokine receptor binding**

    Interacting selectively and non-covalently with any chemokine receptor.

## Help | Hide | Top Help | Show | Top GO BP test for over-representation

### HELP

List of all enriched GO categories (biological processes), at the 0.05
p-value level.

The columns:

- **ExpCount** is the expected count of genes in the
  module annotated with the given GO term, just by chance.
- **Count**
  is the number of genes in the module annotated with the given GO
  term.
- **Size** is the total number of genes (in our universe)
  annotated with the GO term.

Clicking on **Count** shows the genes that drive the
enrichment. You can also click on the individual numbers in
the **Count** column, to show the driving genes for that individual
GO category.

Clicking on the GO identifiers takes you to the Gene Ontology web
pages.

— Click on the *Help* button again to close this help window.

| Id | Pvalue | ExpCount | Count | Size | Term |
| --- | --- | --- | --- | --- | --- |
| GO:0023051 | 3.023e-02 | 132 | 180 Adora2a, Akt1s1, Amer1, Ankrd26, Apoc3, Arap3, Arhgap15, Arhgef1, Bnip2, Cacna1b, Card6, Casp1, Casp2, Ccl11, Ccl2, Ccl5, Ccny, Cd38, Cd4, Cdc42ep2, Cdk14, Cflar, Cish, Col1a1, Cspg5, Cx3cl1, Cxcl13, Cxcl5, Cxxc4, Dixdc1, Dkk3, Dut, Dvl3, Edn3, Egr1, Ei24, Ern1, F7, Fam110c, Fbxo9, Fgd5, Fgf10, Fgf20, Fgfr2, Fgfr4, Fkbp8, Frzb, Fst, Fxn, Fyn, G2e3, Gata6, Gdf6, Gdnf, Gli1, Gli2, Glis2, Gnai2, Gpld1, Gpr183, Gpr55, Gpr65, Gps2, Gpx1, Grik5, Grin2a, Grk4, Grk5, Hdac6, Hey2, Hipk3, Hmgn3, Hnf1a, Ica1, Igf1, Igfbp4, Igfbp5, Il1b, Inha, Intu, Itch, Itga1, Itga8, Itgal, Itgav, Itgb1bp1, Itsn2, Jun, Kcnc4, Kcnn2, Kcnn4, Kif7, Lgals1, Lyn, Mal, Mapk1, Men1, Mfng, Myc, Myocd, Ncdn, Nfkbib, Nlgn2, Nlrx1, Nog, Nrg1, Nup62, Onecut2, Osm, Pak1, Palm, Pdcd11, Pde1c, Pde4a, Pde8b, Pdgfb, Pecam1, Peli1, Pfdn5, Pgap2, Pip5k1a, Pla2g6, Pla2r1, Plekhg6, Postn, Pparg, Ppargc1b, Ppfia3, Prex1, Psd4, Psme3, Ptgir, Ptgs2, Ptk2b, Ptpre, Ralbp1, Rasal1, Retn, Rgn, Rgs16, Rgs19, Rgs3, Rptor, Runx2, Scai, Scrib, Serpine1, Sfrp1, Sfrp2, Shank3, Sigirr, Ski, Smad6, Smo, Socs6, Sod2, Sstr4, Stmn3, Strn3, Sulf1, Syvn1, Tbc1d15, Tbc1d8, Tgfbr3, Tle2, Tlr1, Tmem204, Tnfaip3, Tnik, Trio, Trp53, Tspyl5, Tti1, Ucn, Usp6nl, Vwc2, Wnt1, Wnt11, Wnt4, Zc3hc1 | 1895 | regulation of signaling |
| GO:0010646 | 3.444e-02 | 132.3 | 180 Adora2a, Akt1s1, Amer1, Ankrd26, Apoc3, Arap3, Arhgap15, Arhgef1, Bnip2, Cacna1b, Card6, Casp1, Casp2, Ccl11, Ccl2, Ccl5, Ccny, Cd38, Cd4, Cdc42ep2, Cdk14, Cflar, Cish, Col1a1, Cspg5, Cx3cl1, Cxcl13, Cxcl5, Cxxc4, Dixdc1, Dkk3, Dut, Dvl3, Edn3, Egr1, Ei24, Ern1, F7, Fam110c, Fbxo9, Fgd5, Fgf10, Fgf20, Fgfr2, Fgfr4, Fkbp8, Frzb, Fst, Fxn, Fyn, G2e3, Gata6, Gdf6, Gdnf, Gli1, Gli2, Glis2, Gnai2, Gpld1, Gpr183, Gpr55, Gpr65, Gps2, Gpx1, Grik5, Grin2a, Grk4, Grk5, Hdac6, Hey2, Hipk3, Hmgn3, Hnf1a, Ica1, Igf1, Igfbp4, Igfbp5, Il1b, Inha, Intu, Itch, Itga1, Itga8, Itgal, Itgav, Itgb1bp1, Itsn2, Jun, Kcnc4, Kcnn2, Kcnn4, Kif7, Lgals1, Lyn, Mal, Mapk1, Men1, Mfng, Myc, Myocd, Ncdn, Nfkbib, Nlgn2, Nlrx1, Nog, Nrg1, Nup62, Onecut2, Osm, Pak1, Palm, Pdcd11, Pde1c, Pde4a, Pde8b, Pdgfb, Pecam1, Peli1, Pfdn5, Pgap2, Pip5k1a, Pla2g6, Pla2r1, Plekhg6, Postn, Pparg, Ppargc1b, Ppfia3, Prex1, Psd4, Psme3, Ptgir, Ptgs2, Ptk2b, Ptpre, Ralbp1, Rasal1, Retn, Rgn, Rgs16, Rgs19, Rgs3, Rptor, Runx2, Scai, Scrib, Serpine1, Sfrp1, Sfrp2, Shank3, Sigirr, Ski, Smad6, Smo, Socs6, Sod2, Sstr4, Stmn3, Strn3, Sulf1, Syvn1, Tbc1d15, Tbc1d8, Tgfbr3, Tle2, Tlr1, Tmem204, Tnfaip3, Tnik, Trio, Trp53, Tspyl5, Tti1, Ucn, Usp6nl, Vwc2, Wnt1, Wnt11, Wnt4, Zc3hc1 | 1899 | regulation of cell communication |
| GO:0048583 | 4.708e-02 | 147.9 | 197 Adora2a, Agrp, Akap1, Akt1s1, Amer1, Ankrd26, Apoc3, Arap3, Arhgap15, Arhgef1, Bnip2, C1s, C5ar2, Cadm1, Card6, Casp1, Casp2, Ccl11, Ccl2, Ccl5, Ccny, Cd300a, Cd38, Cd4, Cd79a, Cdc42ep2, Cdk14, Cflar, Cish, Clec2d, Col1a1, Ctla2a, Cx3cl1, Cxcl13, Cxcl5, Cxcr4, Cxxc4, Dixdc1, Dkk3, Dpp4, Dut, Dvl3, Edn3, Egr1, Ei24, Ern1, F7, Fam110c, Fbxo9, Fcgr1, Fcgr2b, Fgd5, Fgf10, Fgf20, Fgfr2, Fgfr4, Fkbp8, Frzb, Fst, Fxn, Fyn, G2e3, Gata6, Gbp4, Gdf6, Gdnf, Gli1, Gli2, Glis2, Gnai2, Gpld1, Gpr183, Gpr55, Gpr65, Gps2, Gpx1, Grin3a, Grk4, Grk5, Hdac4, Hdac6, Hey2, Hipk3, Hmgn3, Hnf1a, Igf1, Igfbp4, Igfbp5, Il1b, Il1rl2, Intu, Itch, Itga1, Itga8, Itgal, Itgav, Itgb1bp1, Itk, Itsn2, Jun, Kcnn4, Kif7, Lgals1, Lyn, Mal, Mapk1, Mapkapk2, Men1, Mfng, Myc, Myo1f, Myocd, Nfkbib, Nlgn2, Nlrx1, Nog, Nrg1, Nup62, Onecut2, Osm, Pak1, Palm, Pdcd11, Pde4a, Pdgfb, Pdpn, Pecam1, Peli1, Pfdn5, Pgap2, Pip5k1a, Pla2g6, Pla2r1, Plekhg6, Pnp, Postn, Ppara, Pparg, Ppargc1b, Prex1, Psd4, Psme3, Ptger3, Ptgir, Ptgs2, Ptk2b, Ptpre, Ralbp1, Rasal1, Retn, Rgn, Rgs16, Rgs19, Rgs3, Rptor, Rtn4, Runx2, Scai, Scrib, Sell, Serpinc1, Serpine1, Sfrp1, Sfrp2, Shank3, Sigirr, Ski, Slc11a1, Smad6, Smo, Socs6, Sod2, Sox15, Sstr4, Stmn3, Strn3, Sulf1, Syvn1, Tbc1d15, Tbc1d8, Tgfbr3, Tle2, Tlr1, Tmem204, Tnfaip3, Tnik, Trio, Trp53, Tspyl5, Tti1, Ucn, Usp6nl, Vwc2, Wnt1, Wnt11, Wnt4, Zc3hc1 | 2124 | regulation of response to stimulus |

## Help | Hide | Top Help | Show | Top GO CC test for over-representation

### HELP

List of all enriched GO categories (cellular components), at the 0.05
p-value level.

The columns:

- **ExpCount** is the expected count of genes in the
  module annotated with the given GO term, just by chance.
- **Count**
  is the number of genes in the module annotated with the given GO
  term.
- **Size** is the total number of genes (in our universe)
  annotated with the GO term.

Clicking on **Count** shows the genes that drive the
enrichment. You can also click on the individual numbers in
the **Count** column, to show the driving genes for that individual
GO category.

Clicking on the GO identifiers takes you to the Gene Ontology web
pages.

— Click on the *Help* button again to close this help window.

| Id | Pvalue | ExpCount | Count | Size | Term |
| --- | --- | --- | --- | --- | --- |
| GO:0005576 | 7.440e-04 | 94.01 | 139 1110058L19Rik, Acpp, Adamts12, Adamts3, Agrp, Aimp1, Ambp, Angptl2, Apoc3, C1s, Casp1, Ccl11, Ccl17, Ccl2, Ccl5, Ccl6, Ccl8, Ccl9, Cd5l, Chgb, Clec11a, Clec3b, Col11a2, Col13a1, Col19a1, Col1a1, Col20a1, Col4a4, Col5a1, Col5a2, Col6a2, Col7a1, Col9a3, Cpa1, Ctf1, Ctla2a, Ctsk, Cx3cl1, Cxcl13, Cxcl2, Cxcl5, Cyb5d2, Dcn, Dkk3, Dpp4, Edn3, Efemp2, Ephx3, F7, Fap, Fbln1, Fbln2, Fcgr2b, Fgf10, Fgf20, Fgfr2, Frem2, Frzb, Fst, Fstl5, Gcnt1, Gdf6, Gdnf, Glb1l2, Gpld1, Hhipl2, Hspa8, Hyal3, Igf1, Igfbp4, Igfbp5, Igfbpl1, Il1b, Il1rap, Inha, Isg15, Itm2b, Kif23, Klk13, Lad1, Lgals1, Loxl3, Lrp2, Ltbp2, Ltbp3, Lyz2, Mfap5, Mmp13, Mmp24, Msn, Muc19, Muc6, Nog, Nyx, Ogn, Osm, Otogl, Pcnt, Pcsk2, Pdgfb, Pecam1, Pigr, Pla2g2d, Pla2r1, Pnp, Pon2, Pon3, Postn, Prrg2, Retn, Rpsa, Saa2, Sbspon, Sct, Sdf2, Serpina1e, Serpina3k, Serpinb6a, Serpinc1, Serpine1, Sfrp1, Sfrp2, Sparc, Sulf1, Tbc1d15, Tgfbr3, Thbd, Thbs2, Thbs3, Timp1, Tnfrsf18, Trem2, Ucn, Vwc2, Wfikkn2, Wnt1, Wnt11, Wnt4, Wnt6 | 1339 | extracellular region |
| GO:0044421 | 1.078e-03 | 60.31 | 97 Adamts12, Agrp, Aimp1, Ambp, Apoc3, Ccl11, Ccl17, Ccl2, Ccl5, Ccl6, Ccl8, Ccl9, Clec3b, Col11a2, Col13a1, Col19a1, Col1a1, Col20a1, Col4a4, Col5a1, Col5a2, Col6a2, Col7a1, Col9a3, Cpa1, Ctf1, Ctsk, Cx3cl1, Cxcl13, Cxcl2, Cxcl5, Dcn, Dkk3, Dpp4, Edn3, Efemp2, F7, Fap, Fbln1, Fbln2, Fgf10, Frem2, Frzb, Gcnt1, Gdf6, Gdnf, Gpld1, Hspa8, Igf1, Igfbp4, Igfbp5, Il1b, Inha, Itm2b, Kif23, Klk13, Lad1, Lgals1, Lrp2, Ltbp2, Ltbp3, Mfap5, Mmp13, Mmp24, Msn, Nog, Nyx, Ogn, Osm, Pcnt, Pcsk2, Pdgfb, Pecam1, Pon3, Postn, Retn, Rpsa, Saa2, Sbspon, Serpina1e, Serpina3k, Serpinb6a, Serpinc1, Serpine1, Sfrp1, Sfrp2, Sparc, Sulf1, Tgfbr3, Thbd, Thbs2, Timp1, Vwc2, Wnt1, Wnt11, Wnt4, Wnt6 | 859 | extracellular region part |
| GO:0005578 | 1.082e-02 | 20.57 | 41 Adamts12, Clec3b, Col11a2, Col13a1, Col19a1, Col1a1, Col20a1, Col4a4, Col5a1, Col5a2, Col6a2, Col7a1, Col9a3, Dcn, Efemp2, Fbln1, Fbln2, Frem2, Gpld1, Igf1, Lad1, Lgals1, Ltbp3, Mfap5, Mmp13, Mmp24, Nyx, Ogn, Postn, Rpsa, Sbspon, Sfrp1, Sparc, Tgfbr3, Thbs2, Timp1, Vwc2, Wnt1, Wnt11, Wnt4, Wnt6 | 293 | proteinaceous extracellular matrix |
| GO:0031012 | 1.869e-02 | 24.64 | 46 Adamts12, Clec3b, Col11a2, Col13a1, Col19a1, Col1a1, Col20a1, Col4a4, Col5a1, Col5a2, Col6a2, Col7a1, Col9a3, Dcn, Efemp2, Fbln1, Fbln2, Fgf10, Fgfr2, Frem2, Gpld1, Igf1, Lad1, Lgals1, Ltbp2, Ltbp3, Mfap5, Mmp13, Mmp16, Mmp24, Nyx, Ogn, Postn, Rpsa, Sbspon, Serpine1, Sfrp1, Sparc, Tgfbr3, Thbs2, Timp1, Vwc2, Wnt1, Wnt11, Wnt4, Wnt6 | 351 | extracellular matrix |
| GO:0005615 | 3.668e-02 | 42.97 | 69 Agrp, Aimp1, Ambp, Apoc3, Ccl11, Ccl17, Ccl2, Ccl5, Ccl6, Ccl8, Ccl9, Clec3b, Col1a1, Col6a2, Cpa1, Ctf1, Ctsk, Cx3cl1, Cxcl13, Cxcl2, Cxcl5, Dcn, Dkk3, Edn3, F7, Fap, Fbln1, Fgf10, Frzb, Gcnt1, Gdf6, Gdnf, Gpld1, Igf1, Igfbp4, Igfbp5, Il1b, Inha, Itm2b, Klk13, Lgals1, Lrp2, Ltbp2, Mmp13, Nog, Ogn, Osm, Pcsk2, Pdgfb, Pecam1, Pon3, Retn, Saa2, Serpina1e, Serpina3k, Serpinb6a, Serpinc1, Serpine1, Sfrp1, Sfrp2, Sparc, Sulf1, Tgfbr3, Thbd, Vwc2, Wnt1, Wnt11, Wnt4, Wnt6 | 612 | extracellular space |

## Help | Hide | Top Help | Show | Top GO MF test for over-representation

### HELP

List of all enriched GO categories (molecular function), at the 0.05
p-value level.

The columns:

- **ExpCount** is the expected count of genes in the
  module annotated with the given GO term, just by chance.
- **Count**
  is the number of genes in the module annotated with the given GO
  term.
- **Size** is the total number of genes (in our universe)
  annotated with the GO term.

Clicking on **Count** shows the genes that drive the
enrichment. You can also click on the individual numbers in
the **Count** column, to show the driving genes for that individual
GO category.

Clicking on the GO identifiers takes you to the Gene Ontology web
pages.

— Click on the *Help* button again to close this help window.

| Id | Pvalue | ExpCount | Count | Size | Term |
| --- | --- | --- | --- | --- | --- |
| GO:0008009 | 3.580e-03 | 2.092 | 11 Ccl11, Ccl17, Ccl2, Ccl5, Ccl6, Ccl8, Ccl9, Cx3cl1, Cxcl13, Cxcl2, Cxcl5 | 30 | chemokine activity |
| GO:0042379 | 7.689e-03 | 2.65 | 12 Ccl11, Ccl17, Ccl2, Ccl5, Ccl6, Ccl8, Ccl9, Cx3cl1, Cxcl13, Cxcl2, Cxcl5, Itch | 38 | chemokine receptor binding |

## Help | Hide | Top Help | Show | Top KEGG Pathway test for over-representation

### HELP

List of all enriched KEGG pathways, at the 0.05
p-value level.

The columns:

- **ExpCount** is the expected count of genes in the
  module annotated with the given KEGG pathway, just by chance.
- **Count**
  is the number of genes in the module annotated with the given KEGG
  pathway.
- **Size** is the total number of genes (in our universe)
  annotated with the KEGG pathway.

Clicking on **Count** shows the genes that drive the
enrichment. You can also click on the individual numbers in
the **Count** column, to show the driving genes for that individual
KEGG pathway.

Clicking on the KEGG identifiers takes you to the KEGG web site.

— Click on the *Help* button again to close this help window.

| Id | Pvalue | ExpCount | Count | Size | Term |
| --- | --- | --- | --- | --- | --- |
| 04062 | 2.080e-03 | 13.72 | 31 Adcy6, Ccl11, Ccl17, Ccl2, Ccl27a, Ccl5, Ccl6, Ccl8, Ccl9, Crkl, Cx3cl1, Cxcl13, Cxcl2, Cxcl5, Cxcr4, Gnai2, Gng5, Gng7, Grk4, Grk5, Itk, Lyn, Mapk1, Nfkbib, Pak1, Plcb3, Prex1, Prkx, Ptk2b, Rock1, Stat2 | 170 | Chemokine signaling pathway |


### HELP

List of all enriched miRNA families, at the 0.05
p-value level.

The columns:

- **ExpCount** is the expected count of genes in the
  module regulated by the given miRNA family, just by chance.
- **Count**
  is the number of genes in the module regulated by the given miRNA
  family.
- **Size** is the total number of genes (in our universe)
  regulated with the given miRNA family.

Clicking on **Count** shows the genes that drive the
enrichment. You can also click on the individual numbers in
the **Count** column, to show the driving genes for that individual
miRNA family.

The miRNA regulation data was taken from the

Top


### HELP

p-value level.

The columns:

- **ExpCount** is the expected number of genes in the- **Count**- **Size** is the total number of genes (in our universe)

Clicking on **Count** shows the genes that drive the
enrichment. You can also click on the individual numbers in
the **Count** column, to show the driving genes for that individual

— Click on the *Help* button again to close this help window.

## Help | Hide | Top Help | Show | Top Genes

### HELP

A list of all genes in the current module, in alphabetical order. The
size of the text corresponds to the gene scores.

Note that some gene symbols may show up more than once, if many
probes match the same Entrez gene.

Genes with no Entrez mapping are given separately, with their
Affymetrics probe ID.

— Click on the *Help* button again to close this help window.

### Genes Symbol

, score:

AatkUnknown, score: 0.55
Abca2Unknown, score: 0.5
Acp1Unknown, score: 0.66
Actn3Unknown, score: 0.66
Acta2Unknown, score: 0.79
Adam11Unknown, score: 0.41
Adam19Unknown, score: 0.41
Adam5Unknown, score: 0.7
Adcy6Unknown, score: 0.5
Adora2aUnknown, score: 0.37
AdslUnknown, score: 0.38
AvilUnknown, score: 0.44
AgaUnknown, score: 0.62
AgrpUnknown, score: 0.47
Akap1Unknown, score: 0.4
Akap2Unknown, score: 0.4
AmbpUnknown, score: 0.48
Anxa11Unknown, score: 0.49
Anxa7Unknown, score: 0.67
Cd5lUnknown, score: 0.38
Apoc3Unknown, score: 0.58
Aqp2Unknown, score: 0.69
Serpinc1Unknown, score: 0.39
Atf2Unknown, score: 0.48
Atp9aUnknown, score: 0.47
Atp6v0cUnknown, score: 0.55
Avpr2Unknown, score: 0.55
Bcl2l2Unknown, score: 0.39
Bnip2Unknown, score: 0.39
Commd3Unknown, score: 0.43
CiitaUnknown, score: 0.42
Cacna1bUnknown, score: 0.38
Cacnb2Unknown, score: 0.46
Ddr1Unknown, score: 0.78
CanxUnknown, score: 0.39
Cap1Unknown, score: 0.56
Casp1Unknown, score: 0.47
Casp2Unknown, score: 0.7
Runx2Unknown, score: 0.46
Cbx4Unknown, score: 0.64
Cct8Unknown, score: 0.45
Cd33Unknown, score: 0.82
Cd38Unknown, score: 0.5
Cd4Unknown, score: 0.72
Cd5Unknown, score: 0.41
Cd79aUnknown, score: 0.57
Cd83Unknown, score: 0.47
Cd8b1Unknown, score: 0.47
Cdk7Unknown, score: 0.5
Cdkn2cUnknown, score: 0.86
CflarUnknown, score: 0.46
ChgbUnknown, score: 0.41
CishUnknown, score: 0.59
Cited1Unknown, score: 0.53
Clcn4-2Unknown, score: 0.44
Cxcr4Unknown, score: 0.46
Plk3Unknown, score: 0.48
Col11a2Unknown, score: 0.63
Col13a1Unknown, score: 0.75
Col19a1Unknown, score: 0.5
Col4a4Unknown, score: 0.37
Col5a1Unknown, score: 0.69
Col5a2Unknown, score: 0.44
Col6a2Unknown, score: 0.48
Col7a1Unknown, score: 0.7
Col9a3Unknown, score: 0.52
Col1a1Unknown, score: 0.45
Atf6bUnknown, score: 0.59
CrklUnknown, score: 0.77
Csf2raUnknown, score: 0.63
Csf2rbUnknown, score: 0.59
Csf3rUnknown, score: 0.39
Dnajc5Unknown, score: 0.72
Ctf1Unknown, score: 0.78
Ctla2aUnknown, score: 0.54
CtseUnknown, score: 0.55
CtskUnknown, score: 0.44
CtslUnknown, score: 0.42
CtswUnknown, score: 0.43
Cyp7a1Unknown, score: 0.43
Dad1Unknown, score: 0.64
DbtUnknown, score: 0.73
DcnUnknown, score: 0.49
Dscr3Unknown, score: 0.91
DffaUnknown, score: 0.38
DffbUnknown, score: 0.91
Dlx4Unknown, score: 0.43
Dync1i2Unknown, score: 0.48
Dpp4Unknown, score: 0.86
Dvl3Unknown, score: 0.4
EbpUnknown, score: 0.53
Edn3Unknown, score: 0.65
Efnb3Unknown, score: 0.39
Egr1Unknown, score: 0.44
Ei24Unknown, score: 0.41
EllUnknown, score: 0.55
Aimp1Unknown, score: 0.45
Mark2Unknown, score: 0.57
En2Unknown, score: 0.39
EndogUnknown, score: 0.48
Epas1Unknown, score: 0.37
Ercc3Unknown, score: 0.78
Khdrbs3Unknown, score: 0.51
F7Unknown, score: 0.6
FapUnknown, score: 0.43
Fbln1Unknown, score: 0.82
Fbln2Unknown, score: 0.58
Fcgr1Unknown, score: 0.53
Fcgr2bUnknown, score: 0.5
Fgf10Unknown, score: 0.38
Fgfr2Unknown, score: 0.39
Fgfr4Unknown, score: 0.7
Akr1b8Unknown, score: 0.94
FhitUnknown, score: 0.85
Fkbp8Unknown, score: 0.38
Fmr1Unknown, score: 0.41
Aff2Unknown, score: 0.84
Folr2Unknown, score: 0.44
Fosl1Unknown, score: 0.67
Fpr2Unknown, score: 0.57
FxnUnknown, score: 0.39
FstUnknown, score: 0.83
FynUnknown, score: 0.47
GalcUnknown, score: 0.49
GartUnknown, score: 0.55
Gata6Unknown, score: 0.43
Gcnt1Unknown, score: 0.76
Gcnt2Unknown, score: 0.59
Gdi2Unknown, score: 0.58
GdnfUnknown, score: 0.54
Gfpt1Unknown, score: 0.83
Gli1Unknown, score: 0.44
Gli2Unknown, score: 0.47
Gnai2Unknown, score: 0.52
Gng5Unknown, score: 0.58
Gng7Unknown, score: 0.45
Lrp2Unknown, score: 0.64
PdpnUnknown, score: 0.5
Gp49aUnknown, score: 0.62
Gpr65Unknown, score: 0.44
Gpld1Unknown, score: 0.4
Grk4Unknown, score: 0.79
Grk5Unknown, score: 0.44
Gpx1Unknown, score: 0.38
Cdca3Unknown, score: 0.42
Grik5Unknown, score: 0.52
Grin2aUnknown, score: 0.7
HalUnknown, score: 0.65
HarsUnknown, score: 0.58
Has1Unknown, score: 0.56
Hdac6Unknown, score: 0.39
Hey2Unknown, score: 0.54
HgdUnknown, score: 0.45
Hipk3Unknown, score: 0.48
H2afxUnknown, score: 0.41
HmmrUnknown, score: 0.43
Hmox2Unknown, score: 0.68
Hoxd9Unknown, score: 0.45
Hrsp12Unknown, score: 0.44
Hspa8Unknown, score: 0.44
Hspa1bUnknown, score: 0.42
Ica1Unknown, score: 0.58
Igf1Unknown, score: 0.4
Igfbp4Unknown, score: 0.53
Igfbp5Unknown, score: 0.41
Il1bUnknown, score: 0.5
Il1rapUnknown, score: 0.78
Il3raUnknown, score: 0.48
Il5raUnknown, score: 0.43
Lrig1Unknown, score: 0.62
InhaUnknown, score: 0.67
ItchUnknown, score: 0.48
ItgalUnknown, score: 0.61
ItgavUnknown, score: 0.57
Itgb1bp1Unknown, score: 0.46
Itgb2Unknown, score: 0.5
ItkUnknown, score: 0.44
Itm2bUnknown, score: 0.76
JunUnknown, score: 0.37
Kcnc1Unknown, score: 0.6
Kcne1Unknown, score: 0.41
Kcnj16Unknown, score: 0.41
Kcnu1Unknown, score: 0.37
Kcnn4Unknown, score: 0.48
KhsrpUnknown, score: 0.6
Kif17Unknown, score: 0.4
Kif5aUnknown, score: 0.69
Kif7Unknown, score: 0.38
Krt14Unknown, score: 0.63
Krt18Unknown, score: 0.52
Lad1Unknown, score: 0.53
RpsaUnknown, score: 0.6
Arhgef1Unknown, score: 0.42
Lgals1Unknown, score: 0.73
GzmmUnknown, score: 0.61
LorUnknown, score: 0.59
Loxl3Unknown, score: 0.54
LrmpUnknown, score: 0.37
Lrrn1Unknown, score: 0.39
Lrrn3Unknown, score: 0.56
Ltb4r1Unknown, score: 0.86
Ltbp2Unknown, score: 0.51
Ltbp3Unknown, score: 0.43
LtkUnknown, score: 0.47
LynUnknown, score: 0.43
LystUnknown, score: 0.38
Lyz2Unknown, score: 0.38
Smad6Unknown, score: 0.5
MagohUnknown, score: 0.52
MalUnknown, score: 0.77
Man1a2Unknown, score: 0.45
MaoaUnknown, score: 0.45
Mapkapk2Unknown, score: 0.59
MatkUnknown, score: 0.47
Men1Unknown, score: 0.49
MfngUnknown, score: 0.5
Mllt4Unknown, score: 0.38
MmeUnknown, score: 0.56
Mmp13Unknown, score: 0.55
Mmp16Unknown, score: 0.57
Mmp24Unknown, score: 0.41
Gbp4Unknown, score: 0.92
MpdzUnknown, score: 0.38
MpzUnknown, score: 0.42
MrasUnknown, score: 0.44
Mrc1Unknown, score: 0.73
Msi1Unknown, score: 0.75
MsnUnknown, score: 0.5
Grpel1Unknown, score: 0.48
mt-Nd6Unknown, score: 0.59
Polr2kUnknown, score: 0.71
Map1aUnknown, score: 0.47
MycUnknown, score: 0.64
Myo1fUnknown, score: 0.47
Ppp1r12aUnknown, score: 0.39
Nat2Unknown, score: 0.72
NclUnknown, score: 0.4
Ndufv1Unknown, score: 0.48
Neu1Unknown, score: 0.49
NfkbibUnknown, score: 0.38
Nqo1Unknown, score: 0.46
NogUnknown, score: 0.91
Slc11a1Unknown, score: 0.38
Nup62Unknown, score: 0.62
OgnUnknown, score: 0.68
OmpUnknown, score: 0.63
OsmUnknown, score: 0.53
PahUnknown, score: 0.41
Pak1Unknown, score: 0.68
PalmUnknown, score: 0.64
PcntUnknown, score: 0.41
Pcsk2Unknown, score: 0.49
Pdcd11Unknown, score: 0.51
Pde1cUnknown, score: 0.51
Pde4aUnknown, score: 0.64
Pde8aUnknown, score: 0.54
PdgfbUnknown, score: 0.43
Pecam1Unknown, score: 0.76
Pex7Unknown, score: 0.54
Cdk14Unknown, score: 0.39
Phf2Unknown, score: 0.59
Phka1Unknown, score: 0.78
PigrUnknown, score: 0.46
Pip5k1bUnknown, score: 0.85
Pip5k1aUnknown, score: 0.49
18722Unknown, score: 0.49
Gm14548Unknown, score: 0.48
Gm15448Unknown, score: 0.49
Gm14548Unknown, score: 0.49
Gm14548Unknown, score: 0.49
Pla2r1Unknown, score: 0.51
Pla2g2dUnknown, score: 0.47
Serpine1Unknown, score: 0.37
PapolaUnknown, score: 0.42
Plcb3Unknown, score: 0.47
PnpUnknown, score: 0.49
PparaUnknown, score: 0.58
PpargUnknown, score: 0.37
Prkar2aUnknown, score: 0.38
PrkdcUnknown, score: 0.6
PrkxUnknown, score: 0.64
Psme3Unknown, score: 0.48
Ptger3Unknown, score: 0.51
PtgirUnknown, score: 0.68
Ptgs1Unknown, score: 0.52
Ptgs2Unknown, score: 0.41
Pth1rUnknown, score: 0.43
Ptk2bUnknown, score: 0.47
Ptp4a3Unknown, score: 0.38
Ptpn18Unknown, score: 0.38
PtpreUnknown, score: 0.38
PtprkUnknown, score: 0.49
PtprrUnknown, score: 0.37
PtsUnknown, score: 0.62
Rai1Unknown, score: 0.61
RapsnUnknown, score: 0.59
Rasal1Unknown, score: 0.49
Rbmxl1Unknown, score: 0.4
Bex1Unknown, score: 0.38
Rfx1Unknown, score: 0.83
RgnUnknown, score: 0.4
Rgs16Unknown, score: 0.42
Ralbp1Unknown, score: 0.37
Rnps1Unknown, score: 0.52
Rock1Unknown, score: 0.66
Rom1Unknown, score: 0.44
RorcUnknown, score: 0.73
Ryr1Unknown, score: 0.7
S100a1Unknown, score: 0.48
S100a10Unknown, score: 0.59
S100a3Unknown, score: 0.48
Saa2Unknown, score: 0.72
Atxn2Unknown, score: 0.45
Scd1Unknown, score: 0.5
Clec11aUnknown, score: 0.38
Stmn3Unknown, score: 0.48
Scn10aUnknown, score: 0.4
Scn3aUnknown, score: 0.46
SctUnknown, score: 0.66
Ccl11Unknown, score: 0.39
Ccl17Unknown, score: 0.63
Ccl2Unknown, score: 0.58
Ccl27aUnknown, score: 0.44
Ccl5Unknown, score: 0.54
Ccl6Unknown, score: 0.42
Ccl8Unknown, score: 0.64
Ccl9Unknown, score: 0.43
Cxcl2Unknown, score: 0.56
Cxcl5Unknown, score: 0.48
Cx3cl1Unknown, score: 0.42
Sdf2Unknown, score: 0.58
Sfrp2Unknown, score: 0.37
Sec22bUnknown, score: 0.53
Sel1lUnknown, score: 0.62
SellUnknown, score: 0.53
Spi1Unknown, score: 0.7
Sfrp1Unknown, score: 0.41
FrzbUnknown, score: 0.41
SgceUnknown, score: 0.49
Itsn2Unknown, score: 0.85
Siah1bUnknown, score: 0.69
ClpbUnknown, score: 0.44
SkiUnknown, score: 0.75
Slc10a1Unknown, score: 0.38
Slc16a1Unknown, score: 0.47
Slc16a2Unknown, score: 0.74
Slc1a1Unknown, score: 0.63
Slc1a2Unknown, score: 0.4
Slc1a5Unknown, score: 0.45
Slc6a2Unknown, score: 0.53
Slfn3Unknown, score: 0.39
Sstr2Unknown, score: 0.48
Sstr4Unknown, score: 0.56
SnrpeUnknown, score: 0.75
Sntb1Unknown, score: 0.37
Sntb2Unknown, score: 0.55
Sod2Unknown, score: 0.43
Sort1Unknown, score: 0.55
Sox15Unknown, score: 0.45
Sp4Unknown, score: 0.4
SparcUnknown, score: 0.48
Serpina1eUnknown, score: 0.65
Serpina3kUnknown, score: 0.74
Serpinb6aUnknown, score: 0.64
Spta1Unknown, score: 0.61
SpopUnknown, score: 0.78
SprUnknown, score: 0.72
Scarb1Unknown, score: 0.38
StamUnknown, score: 0.52
Stat2Unknown, score: 0.63
Stk10Unknown, score: 0.52
Stra13Unknown, score: 0.47
Bhlhe40Unknown, score: 0.63
Taf6Unknown, score: 0.44
TaglnUnknown, score: 0.54
Tbx3Unknown, score: 0.51
Hnf1aUnknown, score: 0.47
TfamUnknown, score: 0.55
Tsc22d1Unknown, score: 0.53
Tgfbr3Unknown, score: 0.58
ThbdUnknown, score: 0.67
Thbs2Unknown, score: 0.8
Thbs3Unknown, score: 0.64
Timm44Unknown, score: 1
Timp1Unknown, score: 0.5
Tle2Unknown, score: 0.51
Tlr1Unknown, score: 0.55
Clec3bUnknown, score: 0.94
Tnfaip3Unknown, score: 0.7
Tnfrsf18Unknown, score: 0.46
Tmem165Unknown, score: 0.41
Trp53Unknown, score: 0.47
Psmd3Unknown, score: 0.51
Tubb2aUnknown, score: 0.51
TyrUnknown, score: 0.39
UcnUnknown, score: 0.59
UgcgUnknown, score: 0.74
Ugt2b5Unknown, score: 0.38
Usp4Unknown, score: 0.52
VillUnknown, score: 0.67
Wnt1Unknown, score: 0.4
Wnt11Unknown, score: 0.41
Wnt4Unknown, score: 0.5
Wnt6Unknown, score: 0.39
Dctn6Unknown, score: 0.57
Yy1Unknown, score: 0.38
Slc30a4Unknown, score: 0.68
Coro1bUnknown, score: 0.45
AplnrUnknown, score: 0.42
Ggt5Unknown, score: 0.82
Nlrp5Unknown, score: 0.55
Pcbp1Unknown, score: 0.54
SigirrUnknown, score: 0.62
Zfp68Unknown, score: 0.42
Angptl2Unknown, score: 0.48
Mapk1Unknown, score: 0.6
Nr5a2Unknown, score: 0.41
Nubp2Unknown, score: 0.44
Plod2Unknown, score: 0.61
PoliUnknown, score: 0.39
NcdnUnknown, score: 0.42
Zfp346Unknown, score: 0.7
Cul1Unknown, score: 0.45
Sec23bUnknown, score: 0.37
Fkbp9Unknown, score: 0.43
Srp9Unknown, score: 0.49
PdhxUnknown, score: 0.47
Hip1rUnknown, score: 0.41
Stau2Unknown, score: 0.66
Cspg5Unknown, score: 0.41
Hacd1Unknown, score: 0.61
Mrps7Unknown, score: 0.9
Mfap5Unknown, score: 0.93
PostnUnknown, score: 0.44
Polg2Unknown, score: 0.46
Rgs3Unknown, score: 0.86
Dkk3Unknown, score: 0.56
Hs6st1Unknown, score: 0.66
Tmod3Unknown, score: 0.39
PrebUnknown, score: 0.43
C1s1Unknown, score: 0.37
Irx4Unknown, score: 0.42
Fubp1Unknown, score: 0.72
R3hcc1lUnknown, score: 0.53
Klhl7Unknown, score: 0.45
Stbd1Unknown, score: 0.49
Angel2Unknown, score: 0.4
Nudcd2Unknown, score: 0.62
D19Bwg1357eUnknown, score: 0.51
Plrg1Unknown, score: 0.78
Pla2g6Unknown, score: 0.81
Txnl1Unknown, score: 0.39
Rfx5Unknown, score: 0.7
Rpp30Unknown, score: 0.92
Nme6Unknown, score: 0.39
Mtmr7Unknown, score: 0.38
Stk19Unknown, score: 0.7
TollipUnknown, score: 0.45
Nup210Unknown, score: 0.44
Socs6Unknown, score: 0.46
Tbc1d8Unknown, score: 0.43
Atp8b2Unknown, score: 0.57
Cadm1Unknown, score: 0.39
Pdzrn3Unknown, score: 0.63
Cxcl13Unknown, score: 0.43
Fmo2Unknown, score: 0.75
Stard10Unknown, score: 0.38
PigpUnknown, score: 0.47
TroUnknown, score: 0.45
Tspan5Unknown, score: 0.45
Actr8Unknown, score: 0.69
Rbm14Unknown, score: 0.45
Arl6Unknown, score: 0.51
Metap2Unknown, score: 0.41
Gps2Unknown, score: 0.59
AcppUnknown, score: 0.78
Mettl3Unknown, score: 0.52
Pdlim5Unknown, score: 0.56
Ykt6Unknown, score: 0.4
Stub1Unknown, score: 0.41
Mbtps1Unknown, score: 0.44
Rgs19Unknown, score: 0.42
Nxt1Unknown, score: 0.37
Ruvbl1Unknown, score: 0.51
Pfdn5Unknown, score: 0.38
Mgst1Unknown, score: 0.49
Clec4eUnknown, score: 0.76
PigoUnknown, score: 0.44
Tdo2Unknown, score: 0.51
Necab3Unknown, score: 0.51
Sap30bpUnknown, score: 0.42
RetnUnknown, score: 0.76
B4galt3Unknown, score: 0.46
Piwil2Unknown, score: 0.54
Tk2Unknown, score: 0.48
Zfp318Unknown, score: 0.73
Rab37Unknown, score: 0.69
Fam184bUnknown, score: 0.42
Shank3Unknown, score: 0.4
CrbnUnknown, score: 0.43
Akr1a1Unknown, score: 0.39
Efemp2Unknown, score: 0.83
Pcbp4Unknown, score: 0.45
Nek7Unknown, score: 0.47
Dctn5Unknown, score: 0.7
Clstn2Unknown, score: 0.58
Sdf2l1Unknown, score: 0.39
RalbUnknown, score: 0.61
Suv39h2Unknown, score: 0.43
Rtn4rUnknown, score: 0.73
Dap3Unknown, score: 0.4
Prrg2Unknown, score: 0.38
CubnUnknown, score: 0.66
Zdhhc12Unknown, score: 0.64
Pno1Unknown, score: 0.42
Blzf1Unknown, score: 0.67
Pcp4l1Unknown, score: 0.39
Uba5Unknown, score: 0.44
Tmem41aUnknown, score: 0.38
Tbc1d15Unknown, score: 0.38
Uqcrfs1Unknown, score: 0.77
Henmt1Unknown, score: 0.55
Emc2Unknown, score: 0.42
Lztr1Unknown, score: 0.51
Lonp2Unknown, score: 0.63
Yae1d1Unknown, score: 0.5
PaicsUnknown, score: 0.46
PgpUnknown, score: 0.57
D16Ertd472eUnknown, score: 0.52
Gemin6Unknown, score: 0.7
Peli1Unknown, score: 0.48
Rpl39Unknown, score: 0.39
Gprasp1Unknown, score: 0.39
Zc2hc1aUnknown, score: 0.47
SrprUnknown, score: 0.61
Nudcd1Unknown, score: 0.42
Pnpla8Unknown, score: 0.45
IkbipUnknown, score: 0.7
Ccar1Unknown, score: 0.51
GstcdUnknown, score: 0.37
NarflUnknown, score: 0.44
Akt1s1Unknown, score: 0.51
Nob1Unknown, score: 0.79
Bend5Unknown, score: 0.45
Spc24Unknown, score: 0.56
MgarpUnknown, score: 0.72
Slc35a4Unknown, score: 0.41
Echdc3Unknown, score: 0.52
RprmUnknown, score: 0.55
SarafUnknown, score: 0.52
Ppa1Unknown, score: 0.51
Pold3Unknown, score: 0.68
CcnyUnknown, score: 0.66
TrabdUnknown, score: 0.43
Tmx3Unknown, score: 0.47
Fam134cUnknown, score: 0.41
Sdhaf4Unknown, score: 0.48
3010026O09RikUnknown, score: 0.63
Etaa1Unknown, score: 0.54
Ndufa5Unknown, score: 0.45
Trmt12Unknown, score: 0.58
Toe1Unknown, score: 0.62
Ddx39Unknown, score: 0.51
Vps53Unknown, score: 0.49
Zfyve21Unknown, score: 0.51
Rtn4Unknown, score: 0.47
Samm50Unknown, score: 0.76
Rnf166Unknown, score: 0.6
Trp53inp2Unknown, score: 0.55
Phtf2Unknown, score: 0.63
Tmem214Unknown, score: 0.55
Zfand2bUnknown, score: 0.72
Rnf208Unknown, score: 0.49
Fam58bUnknown, score: 0.5
Tmem110Unknown, score: 0.64
Ccdc23Unknown, score: 0.52
3300002I08RikUnknown, score: 0.39
Slc16a13Unknown, score: 0.69
PacrgUnknown, score: 0.71
Cdhr4Unknown, score: 0.53
1700029J07RikUnknown, score: 0.58
Dnase1l1Unknown, score: 0.55
Cd164l2Unknown, score: 0.45
1810026J23RikUnknown, score: 0.4
Polr2fUnknown, score: 0.44
Chn2Unknown, score: 0.53
Vps36Unknown, score: 0.41
Kcnip1Unknown, score: 0.42
Bag5Unknown, score: 0.5
Ica1lUnknown, score: 0.46
Stk26Unknown, score: 0.39
Taf15Unknown, score: 0.55
Slc35d2Unknown, score: 0.38
DdoUnknown, score: 0.43
Btf3l4Unknown, score: 0.55
Gpr137cUnknown, score: 0.7
CgnUnknown, score: 0.44
Ankib1Unknown, score: 0.48
Hmgxb4Unknown, score: 0.59
UvssaUnknown, score: 0.57
Clec12bUnknown, score: 0.41
Otud1Unknown, score: 0.83
Dlg5Unknown, score: 0.89
Alpk1Unknown, score: 0.46
GrapUnknown, score: 0.43
Fbxo9Unknown, score: 0.48
Mus81Unknown, score: 0.45
Vps11Unknown, score: 0.63
Ankle2Unknown, score: 0.41
Kif23Unknown, score: 0.41
Osgin1Unknown, score: 0.59
Noxo1Unknown, score: 0.91
Ppapdc1bUnknown, score: 0.63
Ephx3Unknown, score: 0.49
Tom1l1Unknown, score: 0.48
Cotl1Unknown, score: 0.71
Phf10Unknown, score: 0.58
Trub1Unknown, score: 0.55
Fbxl20Unknown, score: 0.39
CdaUnknown, score: 0.71
Smim24Unknown, score: 0.51
Lsm11Unknown, score: 0.7
Vps9d1Unknown, score: 0.65
Amer1Unknown, score: 0.45
Atxn7l2Unknown, score: 0.38
Pgam5Unknown, score: 0.45
Hspa12bUnknown, score: 0.42
2700094K13RikUnknown, score: 0.39
PhykplUnknown, score: 0.49
Prr36Unknown, score: 0.65
Tgfbrap1Unknown, score: 0.55
Col20a1Unknown, score: 0.47
Ndufaf7Unknown, score: 0.46
Gucy2gUnknown, score: 0.44
Mvb12aUnknown, score: 0.73
Man2c1Unknown, score: 0.41
Arhgap18Unknown, score: 0.38
4632428N05RikUnknown, score: 0.48
NeblUnknown, score: 0.39
Rbm19Unknown, score: 0.43
CrotUnknown, score: 0.58
AcoxlUnknown, score: 0.38
Syvn1Unknown, score: 0.72
Tm9sf1Unknown, score: 0.38
Slc35f5Unknown, score: 0.4
Zdhhc16Unknown, score: 0.68
Xpo6Unknown, score: 0.42
HnrnprUnknown, score: 0.47
RptorUnknown, score: 0.56
Clvs1Unknown, score: 0.62
CmipUnknown, score: 0.73
Kbtbd12Unknown, score: 0.41
Scrn3Unknown, score: 0.39
Ptchd3Unknown, score: 0.37
Rab3il1Unknown, score: 0.59
Fam63aUnknown, score: 0.45
4930505A04RikUnknown, score: 0.41
Ccdc146Unknown, score: 0.47
Cep128Unknown, score: 0.88
Tti1Unknown, score: 0.72
Igfbpl1Unknown, score: 0.87
Ttc32Unknown, score: 0.44
Lyrm7Unknown, score: 0.73
Morc4Unknown, score: 0.44
Wdr64Unknown, score: 0.64
Fam184aUnknown, score: 0.39
Ccdc125Unknown, score: 0.4
Arhgap15Unknown, score: 0.42
Atp6v0e2Unknown, score: 0.47
Slc38a3Unknown, score: 0.38
GptUnknown, score: 0.69
Asb5Unknown, score: 0.37
TgdsUnknown, score: 0.72
1700019B03RikUnknown, score: 0.41
Ppp1r18Unknown, score: 0.56
Msrb2Unknown, score: 0.38
Wdr38Unknown, score: 0.51
Ppfia3Unknown, score: 0.46
NubplUnknown, score: 0.47
Catsper3Unknown, score: 0.4
1700015E13RikUnknown, score: 0.48
Bcas1Unknown, score: 0.98
Zfp934Unknown, score: 0.63
C330018D20RikUnknown, score: 0.47
Zfp266Unknown, score: 0.57
Sbf1Unknown, score: 0.38
Mccc2Unknown, score: 0.46
Cox7b2Unknown, score: 0.42
Nxpe2Unknown, score: 0.51
TroapUnknown, score: 0.57
Galnt15Unknown, score: 0.72
Efcab11Unknown, score: 0.57
Hhipl2Unknown, score: 0.85
Stpg1Unknown, score: 0.5
Cnot10Unknown, score: 0.45
Pus7lUnknown, score: 0.43
MispUnknown, score: 0.81
Srd5a1Unknown, score: 0.49
Ern1Unknown, score: 0.47
Pacsin3Unknown, score: 0.39
Fgf20Unknown, score: 0.55
LrbaUnknown, score: 0.75
Ntng1Unknown, score: 0.74
LactbUnknown, score: 0.81
Gpr84Unknown, score: 0.41
Jdp2Unknown, score: 0.57
Sorcs2Unknown, score: 0.47
Sp6Unknown, score: 0.49
Glis2Unknown, score: 0.39
Trem2Unknown, score: 0.46
Slc25a2Unknown, score: 0.56
Gpr137bUnknown, score: 0.53
Rnf123Unknown, score: 0.49
Tnfrsf25Unknown, score: 0.58
Clec2dUnknown, score: 0.56
Pcdhb8Unknown, score: 0.41
Pcdhb13Unknown, score: 0.43
Pcdhb15Unknown, score: 0.43
Pcdhb18Unknown, score: 0.39
Pcdhb19Unknown, score: 0.38
Strn3Unknown, score: 0.39
Adarb2Unknown, score: 0.48
S1pr5Unknown, score: 0.43
PrccUnknown, score: 0.45
Tmem40Unknown, score: 0.59
Hmgn3Unknown, score: 0.66
TprnUnknown, score: 0.53
C78339Unknown, score: 0.5
D2hgdhUnknown, score: 0.39
Ehd4Unknown, score: 0.4
Usp6nlUnknown, score: 0.54
Mrps26Unknown, score: 0.39
Stard7Unknown, score: 0.6
Golga2Unknown, score: 0.59
Wdr47Unknown, score: 0.38
Usp53Unknown, score: 0.5
Kcnc4Unknown, score: 0.38
Ifi44Unknown, score: 0.42
Tyw1Unknown, score: 0.39
Fbxl14Unknown, score: 0.39
Phrf1Unknown, score: 0.59
Fam192aUnknown, score: 0.43
Lars2Unknown, score: 0.42
Snx19Unknown, score: 0.76
Rpp25Unknown, score: 0.49
Chchd10Unknown, score: 0.49
Nup107Unknown, score: 0.49
Slc25a41Unknown, score: 0.51
Ces1dUnknown, score: 0.4
Cdc42ep2Unknown, score: 0.61
Vipas39Unknown, score: 0.38
Tmem179Unknown, score: 0.45
Fam110cUnknown, score: 0.41
IarsUnknown, score: 0.63
Ankrd28Unknown, score: 0.49
Mycbp2Unknown, score: 0.54
ScribUnknown, score: 0.38
SrlUnknown, score: 0.72
Arap3Unknown, score: 0.4
Guca1bUnknown, score: 0.42
Il1rl2Unknown, score: 0.39
Nt5c3Unknown, score: 0.67
Mrpl30Unknown, score: 0.41
Ddb2Unknown, score: 0.73
Gtpbp6Unknown, score: 0.62
Ap4e1Unknown, score: 0.69
Skiv2lUnknown, score: 0.49
Prkag2Unknown, score: 0.47
Galnt7Unknown, score: 0.51
Dnajc9Unknown, score: 0.43
Rarres1Unknown, score: 0.61
Orai1Unknown, score: 0.57
Hyal3Unknown, score: 0.43
Cpa1Unknown, score: 0.38
Itga1Unknown, score: 0.39
MaobUnknown, score: 0.65
Art3Unknown, score: 0.52
DutUnknown, score: 0.51
MsraUnknown, score: 0.54
Grik4Unknown, score: 0.4
Egln2Unknown, score: 0.5
Zbtb21Unknown, score: 0.6
Crip3Unknown, score: 0.53
Derl2Unknown, score: 0.52
Gjc2Unknown, score: 0.39
Kcnn2Unknown, score: 0.46
Kcnn3Unknown, score: 0.56
Atp6v0a4Unknown, score: 0.53
Ubox5Unknown, score: 0.48
Olfr78Unknown, score: 0.54
KirrelUnknown, score: 0.57
Hdac10Unknown, score: 0.54
Ppargc1bUnknown, score: 0.81
Ntng2Unknown, score: 0.4
Timd2Unknown, score: 0.38
Havcr2Unknown, score: 0.39
Cd99l2Unknown, score: 0.43
MvdUnknown, score: 0.69
Stab2Unknown, score: 0.45
Bcas3Unknown, score: 0.88
Rapgef6Unknown, score: 0.54
Cyb5d2Unknown, score: 0.62
Fam120cUnknown, score: 0.49
Dtx4Unknown, score: 0.38
Gramd1cUnknown, score: 0.38
Cyp4f17Unknown, score: 0.4
Shroom4Unknown, score: 0.46
Creb3l2Unknown, score: 0.49
Hdac4Unknown, score: 0.58
Daam1Unknown, score: 0.6
Taf3Unknown, score: 0.51
MtrrUnknown, score: 0.38
Dock10Unknown, score: 0.37
Mettl14Unknown, score: 0.65
Coq10aUnknown, score: 0.66
Nrg1Unknown, score: 0.43
Exoc3Unknown, score: 0.38
Depdc7Unknown, score: 0.53
Plekhh1Unknown, score: 0.57
Zswim4Unknown, score: 0.41
Klhl32Unknown, score: 0.39
TifabUnknown, score: 0.57
Tmem71Unknown, score: 0.46
Fstl5Unknown, score: 0.82
Mylk3Unknown, score: 0.81
Plekhg6Unknown, score: 0.48
9530068E07RikUnknown, score: 0.67
Casd1Unknown, score: 0.48
Pak6Unknown, score: 0.39
CrygnUnknown, score: 0.45
MyocdUnknown, score: 0.39
Parp16Unknown, score: 0.76
Tmprss4Unknown, score: 0.63
Zfp879Unknown, score: 0.43
Neurl3Unknown, score: 0.39
Slc39a2Unknown, score: 0.58
Trim50Unknown, score: 0.69
Psd4Unknown, score: 0.46
Ctnna3Unknown, score: 0.46
Ube2d1Unknown, score: 0.53
IlvblUnknown, score: 0.74
Rufy1Unknown, score: 0.78
Tom1l2Unknown, score: 0.5
CntrobUnknown, score: 0.47
Wrap53Unknown, score: 0.39
Nlgn2Unknown, score: 0.43
Wscd1Unknown, score: 0.5
Arl5cUnknown, score: 0.53
Cd300aUnknown, score: 0.59
UnkUnknown, score: 0.4
Tnrc6cUnknown, score: 0.44
Trib2Unknown, score: 0.56
G2e3Unknown, score: 0.75
Mlh3Unknown, score: 0.72
Cdc42bpbUnknown, score: 0.43
Serinc5Unknown, score: 0.39
Pde8bUnknown, score: 0.4
Zfp503Unknown, score: 0.48
Mtmr6Unknown, score: 0.5
Arl11Unknown, score: 0.54
Hmbox1Unknown, score: 0.74
Pcdh20Unknown, score: 0.4
GgactUnknown, score: 0.61
TrioUnknown, score: 0.4
NaprtUnknown, score: 0.41
Gramd4Unknown, score: 0.75
Brd1Unknown, score: 0.4
Slc7a4Unknown, score: 0.37
D17Wsu92eUnknown, score: 0.67
Ankrd29Unknown, score: 0.39
Fam13bUnknown, score: 0.47
Onecut2Unknown, score: 0.5
Cep76Unknown, score: 0.58
Pla2g16Unknown, score: 0.39
Trpm3Unknown, score: 0.44
Hectd2Unknown, score: 0.55
Fam160b1Unknown, score: 0.66
Dyrk3Unknown, score: 0.41
Zfp281Unknown, score: 0.5
Aph1aUnknown, score: 0.38
Ppp2r5aUnknown, score: 0.46
SbsponUnknown, score: 0.43
Gpr55Unknown, score: 0.5
Prrc2bUnknown, score: 0.47
Kif18aUnknown, score: 0.53
Arhgap11aUnknown, score: 0.38
6820408C15RikUnknown, score: 0.49
D930015E06RikUnknown, score: 0.46
Isg20l2Unknown, score: 0.47
Gpr61Unknown, score: 0.48
Clcc1Unknown, score: 0.41
Frrs1lUnknown, score: 0.38
Sh3tc1Unknown, score: 0.41
Slc10a4Unknown, score: 0.44
AasdhUnknown, score: 0.52
FicdUnknown, score: 0.68
MepceUnknown, score: 0.42
Ap5z1Unknown, score: 0.39
Plekha8Unknown, score: 0.4
Fgd5Unknown, score: 0.59
Frmd4bUnknown, score: 0.55
Ankrd26Unknown, score: 0.44
Gys2Unknown, score: 0.65
Amn1Unknown, score: 0.49
Zc3hc1Unknown, score: 0.41
Tmc3Unknown, score: 0.41
Pgap2Unknown, score: 0.47
Thumpd1Unknown, score: 0.47
Gtf3c1Unknown, score: 0.53
Prr14Unknown, score: 0.56
Naf1Unknown, score: 0.5
Gatad2aUnknown, score: 0.48
Klhl36Unknown, score: 0.51
Ccdc67Unknown, score: 0.46
Msantd2Unknown, score: 0.62
Tmem136Unknown, score: 0.38
DlatUnknown, score: 0.41
Dmxl2Unknown, score: 0.44
HykkUnknown, score: 0.38
Snx33Unknown, score: 0.77
Usp3Unknown, score: 0.46
Gk5Unknown, score: 0.38
NyxUnknown, score: 0.61
Mmgt1Unknown, score: 0.5
Pcyt1bUnknown, score: 0.63
Gnl3lUnknown, score: 0.48
Ric8bUnknown, score: 0.58
Slfn9Unknown, score: 0.56
Slc24a4Unknown, score: 0.54
Serpinb6dUnknown, score: 0.53
Ppwd1Unknown, score: 0.63
HomezUnknown, score: 0.4
Slitrk6Unknown, score: 0.41
Card6Unknown, score: 0.55
Adamts12Unknown, score: 0.4
Tspyl5Unknown, score: 0.4
Fam83aUnknown, score: 0.38
239611Unknown, score: 0.68
Dip2bUnknown, score: 0.38
Neurl1bUnknown, score: 0.44
Fem1cUnknown, score: 0.49
Dmxl1Unknown, score: 0.61
F830016B08RikUnknown, score: 0.44
Sec31bUnknown, score: 0.78
Mcmdc2Unknown, score: 0.83
Sulf1Unknown, score: 0.53
Klhl12Unknown, score: 0.53
Zbtb37Unknown, score: 0.45
Scyl3Unknown, score: 0.42
Dusp27Unknown, score: 0.83
Lrrc52Unknown, score: 0.39
Itga8Unknown, score: 0.54
St8sia6Unknown, score: 0.43
Xirp2Unknown, score: 0.61
Cers6Unknown, score: 0.49
241621Unknown, score: 0.38
Wdr76Unknown, score: 0.45
Frem2Unknown, score: 0.6
Gdf6Unknown, score: 0.42
Gabbr2Unknown, score: 0.42
Grin3aUnknown, score: 0.47
Frmd3Unknown, score: 0.42
Rsbn1lUnknown, score: 0.43
Fbxl5Unknown, score: 0.65
Sbno1Unknown, score: 0.5
Grip2Unknown, score: 0.52
Kbtbd8Unknown, score: 0.43
GgnUnknown, score: 0.64
Nars2Unknown, score: 0.4
Trim30bUnknown, score: 0.44
Tox3Unknown, score: 0.38
Kdm4dUnknown, score: 0.51
Glb1l2Unknown, score: 0.38
Ankk1Unknown, score: 0.54
Dcaf12l1Unknown, score: 0.61
Kir3dl1Unknown, score: 0.37
Mum1l1Unknown, score: 0.45
Amdhd2Unknown, score: 0.41
RttnUnknown, score: 0.38
BivmUnknown, score: 0.38
Prokr2Unknown, score: 0.41
Obox6Unknown, score: 0.39
Tpcn1Unknown, score: 0.45
Ehd2Unknown, score: 0.41
Gga3Unknown, score: 0.4
Cdk8Unknown, score: 0.58
Zbtb24Unknown, score: 0.42
MpgUnknown, score: 0.42
Bahcc1Unknown, score: 0.42
Nup54Unknown, score: 0.56
Nbeal1Unknown, score: 0.41
Rtn4rl2Unknown, score: 0.41
VcpUnknown, score: 0.47
Gpr157Unknown, score: 0.41
Pank4Unknown, score: 0.4
Zfp384Unknown, score: 0.49
Pon3Unknown, score: 0.41
Tspan12Unknown, score: 0.41
Nat14Unknown, score: 0.69
Rccd1Unknown, score: 0.44
Nup98Unknown, score: 0.4
GcdhUnknown, score: 0.42
Nlrx1Unknown, score: 0.39
Klhl18Unknown, score: 0.37
Eif5aUnknown, score: 0.37
Marveld1Unknown, score: 0.59
Prex1Unknown, score: 0.44
Vstm2lUnknown, score: 0.45
Fam131cUnknown, score: 0.48
Wfikkn2Unknown, score: 0.93
Kctd19Unknown, score: 0.4
Pcdh19Unknown, score: 0.4
Gpr4Unknown, score: 0.41
A230050P20RikUnknown, score: 0.8
C5ar2Unknown, score: 0.57
Lrtm1Unknown, score: 0.63
Cxxc4Unknown, score: 0.5
SmoUnknown, score: 0.37
Dock6Unknown, score: 0.52
Vwc2Unknown, score: 0.39
Nceh1Unknown, score: 0.78
Exph5Unknown, score: 0.71
Zmat4Unknown, score: 0.6
Hook3Unknown, score: 0.46
ScaiUnknown, score: 0.4
Tmem251Unknown, score: 0.54
FryUnknown, score: 0.47
CenptUnknown, score: 0.42
Alg6Unknown, score: 0.54
P4ha3Unknown, score: 0.53
Sycp2Unknown, score: 0.39
Dennd5bUnknown, score: 0.56
Tmem88bUnknown, score: 0.42
Phf8Unknown, score: 0.38
D5Ertd579eUnknown, score: 0.56
Tmem117Unknown, score: 0.42
Zhx3Unknown, score: 0.47
Cdh10Unknown, score: 0.41
Mical2Unknown, score: 0.4
Wscd2Unknown, score: 0.46
Gpr183Unknown, score: 0.52
Cdv3Unknown, score: 0.39
Trmt61aUnknown, score: 0.41
Aknad1Unknown, score: 0.42
Adamts3Unknown, score: 0.38
Gal3st4Unknown, score: 0.52
Sdk1Unknown, score: 0.51
Pon2Unknown, score: 0.41
Ctxn1Unknown, score: 0.57
Zfp866Unknown, score: 0.38
Dixdc1Unknown, score: 0.85
Rgag4Unknown, score: 0.47
Tfap2eUnknown, score: 0.49
Trim65Unknown, score: 0.6
Sfmbt2Unknown, score: 0.57
353328Unknown, score: 0.41
Hist1h3aUnknown, score: 0.4
Speer4dUnknown, score: 0.42
IntuUnknown, score: 0.58
Mrs2Unknown, score: 0.58
Lyrm4Unknown, score: 0.49
Gm21981Unknown, score: 0.54
Kif14Unknown, score: 0.55
Zfp408Unknown, score: 0.63
Rbm33Unknown, score: 0.37
Zcwpw1Unknown, score: 0.77
Foxr1Unknown, score: 0.46
Snx22Unknown, score: 0.38
Zkscan7Unknown, score: 0.42
Ankrd63Unknown, score: 0.58
Scn4bUnknown, score: 0.52
Plcxd1Unknown, score: 0.6
Tmem204Unknown, score: 0.51
Ociad2Unknown, score: 0.47
Armcx5Unknown, score: 0.43
Fam186bUnknown, score: 0.5
545487Unknown, score: 0.44
Gramd2Unknown, score: 0.4
Zfp827Unknown, score: 0.38
Klk13Unknown, score: 0.54
Lipo4Unknown, score: 0.48
OtoglUnknown, score: 0.45
640370Unknown, score: 0.41
Gcnt7Unknown, score: 0.4
TnikUnknown, score: 0.49
666448Unknown, score: 0.62
667739Unknown, score: 0.42
Isg15Unknown, score: 0.49
100039794Unknown, score: 0.46
Pdcd5Unknown, score: 0.52
100043217Unknown, score: 0.4
100047518Unknown, score: 0.39
100047632Unknown, score: 0.43
Cfap43Unknown, score: 0.75
Ddx43Unknown, score: 0.42
Ccdc13Unknown, score: 0.59
Ccdc149Unknown, score: 0.42
TmppeUnknown, score: 0.77
100862012Unknown, score: 0.59
Gm21698Unknown, score: 0.63
100862570Unknown, score: 0.39

## Help | Hide | Top Help | Show | Top Conditions

### HELP

Conditions in the module, given in the same order as on the expression
plot above. Red color means over-expression, green under-expression in
the given condition.

The barplot below shows the condition (sample) scores. A separate bar
is shown for each sample, its height is the corresponding score of the
sample in the module. The red and green numbers on the bars are the
sample scores expressed in percents, i.e. 100% is 1.0.

The red and green lines show the module thresholds, samples above
the red line and below the green line are included in the module.

The different experiments that were part of the study, are separated
by dashed vertical lines.

— Click on the *Help* button again to close this help window.

Id

C57BL\_6J-ATE\_95

BALB\_cByJ-ATE\_157

NOD\_ShiLtJ-ATE\_23

A\_J-ATE\_117

C57BL\_6J-ATE\_109

FVB\_NJ-ATE\_60

LP\_J-ATE\_98

FVB\_NJ-ATE\_57

LP\_J-ATE\_105

NOD\_ShiLtJ-ATE\_27

FVB\_NJ-CTR\_51

FVB\_NJ-ATE\_53

SJL\_J-ATE\_18

PL\_J-ATE\_76

A\_J-ATE\_116

LP\_J-ATE\_101

NOD\_ShiLtJ-ATE\_22

FVB\_NJ-CTR\_52

C57BLKS\_J-ATE\_112

BALB\_cByJ-ATE\_160

PL\_J-ATE\_75

A\_J-ATE\_122

C57BLKS\_J-ATE\_113

C58\_J-ATE\_36

LP\_J-CTR\_104

C58\_J-ATE\_29

C57BL\_6J-CTR\_39

SWR\_J-ATE\_135

SJL\_J-ATE\_17

LP\_J-CTR\_100

C58\_J-ATE\_30

NOD\_ShiLtJ-CTR\_21

DBA\_2J-ATE\_6

SWR\_J-ATE\_140

C57BL\_6J-ATE\_106

C57BLKS\_J-ATE\_108

NZB\_BLNJ-ATE\_94

BALB\_cByJ-ATE\_151

A\_J-CTR\_120

LP\_J-CTR\_97

PL\_J-ATE\_71

BALB\_cByJ-CTR\_158

C57BL\_6J-CTR\_38

SM\_J-ATE\_43

A\_J-CTR\_119

SM\_J-CTR\_48

SM\_J-ATE\_49

PL\_J-CTR\_70

SWR\_J-CTR\_138

C3H\_HeJ-ATE\_61

C3H\_HeJ-ATE\_67

SJL\_J-CTR\_16

C57BL\_6J-CTR\_35

C58\_J-CTR\_32

C57BLKS\_J-CTR\_110

SM\_J-CTR\_40

NOD\_ShiLtJ-CTR\_20

CBA\_J-ATE\_132

SJL\_J-ATE\_11

PL\_J-CTR\_74

SWR\_J-ATE\_139

FVB\_NJ-CTR\_56

SJL\_J-CTR\_10

SJL\_J-CTR\_15

C58\_J-CTR\_34

NZB\_BLNJ-CTR\_92

NZB\_BLNJ-ATE\_89

C57BLKS\_J-CTR\_107

NOD\_ShiLtJ-CTR\_19

NZB\_BLNJ-CTR\_93

PL\_J-CTR\_72

CBA\_J-ATE\_127

I\_LnJ-CTR\_150

SM\_J-CTR\_41

C57BLKS\_J-CTR\_111

NZB\_BLNJ-ATE\_88

BALB\_cJ-ATE\_80

C58\_J-CTR\_28

SM\_J-ATE\_42

I\_LnJ-ATE\_154

CBA\_J-ATE\_131

BALB\_cByJ-CTR\_143

C3H\_HeJ-ATE\_66

C57BL\_6J-ISO\_47

DBA\_2J-CTR\_1

C57BL\_6J-ISO\_59

PL\_J-ISO\_77

SJL\_J-ISO\_12

LP\_J-ISO\_102

PL\_J-ISO\_78

LP\_J-ISO\_103

SJL\_J-ISO\_14

SJL\_J-ISO\_13

FVB\_NJ-ISO\_54

C57BL\_6J-ISO\_50

FVB\_NJ-ISO\_58

A\_J-ISO\_123

C58\_J-ISO\_37

C58\_J-ISO\_31

SWR\_J-ISO\_142

BALB\_cByJ-ISO\_145

PL\_J-ISO\_73

C57BLKS\_J-ISO\_114

A\_J-ISO\_124

SWR\_J-ISO\_141

FVB\_NJ-ISO\_55

NZB\_BLNJ-ISO\_90

A\_J-ISO\_118

NZB\_BLNJ-ISO\_96

BALB\_cByJ-ISO\_159

SWR\_J-ISO\_136

I\_LnJ-ISO\_152

NZB\_BLNJ-ISO\_91

I\_LnJ-ISO\_155

SM\_J-ISO\_44

CBA\_J-ISO\_128

SM\_J-ISO\_45

SM\_J-ISO\_46

CBA\_J-ISO\_133

CBA\_J-ISO\_129

C58\_J-ISO\_33

C3H\_HeJ-ISO\_68

C57BLKS\_J-ISO\_115

BALB\_cByJ-ISO\_156

C3H\_HeJ-ISO\_69

BALB\_cJ-ISO\_84

BALB\_cJ-ISO\_83

NOD\_ShiLtJ-ISO\_26

I\_LnJ-ISO\_149

DBA\_2J-ISO\_7

DBA\_2J-ISO\_4

NOD\_ShiLtJ-ISO\_25

NOD\_ShiLtJ-ISO\_24

BALB\_cJ-ISO\_81

C3H\_HeJ-ISO\_62

DBA\_2J-ISO\_8

© 2015 Computational Biology Group, Department of Medical Genetics,
University of Lausanne, Switzerland
