# Supplementary material for: RNAseq analysis of heart tissue from mice treated with atenolol and isoproterenol reveals a reciprocal transcriptional response
Source: BMC Genomics. 2016 Sep 7;17(1):717. doi: 10.1186/s12864-016-3059-6 (PMC5015234; doi:10.1186/s12864-016-3059-6)
Supplement: Additional file 4: — Supplementary data. Compressed HTML files of 98 expression modules annotated for genes, strains and GO or KEGG terms (see Additional file 3 for navigation details). (GZ 11006 kb) [file 12864_2016_3059_MOESM4_ESM.gz › modules.html/module-89.html]

Module #89, TG: 1.5, TC: 0.375, 1016 probes, 1016 Entrez genes, 106 conditions

# Previous module | Next module Module #89, TG: 1.5, TC: 0.375, 1016 probes, 1016 Entrez genes, 106 conditions

- Module tree/table

- Expression data

- The BP GO tree
- The CC GO tree
- The MF GO tree

- GO BP enrichment
- GO CC enrichment
- GO MF enrichment
- KEGG enrichment
- miRNA enrichment

- Genes
- Conditions

## Help | Hide | Top Help | Show | Top Expression data

### HELP

The image plot shows the color-coded level of gene expression, for the
genes and conditions in a given transcription module. The genes are on
the horizontal, the conditions on the vertical axis.

The genes are ordered according to their ISA gene scores, similarly
the conditions are ordered according to their condition scores. The
score of a gene means the «degree of inclusion» in
the module: a high score gene is essential in the module.

Condition scores can also be negative, that means that the genes of
the module are all down-regulated in the condition. Here the absolute
value of the score gives the «degree of inclusion».

The plots above and beside the expression matrix show the gene scores
and condition scores, respectively.

Note that the plot is interactive, you can see the name of the gene
and condition under the mouse cursor.

The expression matrix was normalized to have mean zero and standard
deviation one for every gene separately across all conditions
(i.e. not just for the conditions in the module).

— Click on the *Help* button again to close this help window.

Gene:   
Condition:


Under-expression is coded with green,
over-expression with red color.

## Help | Hide | Top Help | Show | Top The GO tree — Biological processes

### HELP

This is one of three sections showing Gene Ontology enrichment of the
current module: in this case for **biological processes**.

The graph shows the hierarchy of the GO categories, their enrichment
for the current module is color coded, and the blue number beside the
category is the minus log ten p-value of the enrichment. (Calculated
using the standard hypergeometric test.) The color of the arrows code
«is a» (cyan) and «part of» relationships.

The tree was built the following way. First all GO terms with more
significant enrichment p-value than 0.05 were collected. Then all
paths from these terms to the root node of the GO tree were included
too. If a GO term is included more than once in the tree, then the
green numbers show 1) the id of the node, this makes it easier to find
other appereances of the term, and 2) the number of appearences.

Note that the same GO category might show up on the graph many
times. This is because the GO was «straightened» for this
graph, i.e. if there are more paths from a GO term to the root node of
the tree, all of them are included. The green numbers

Move the mouse cursor over the terms to get their definition. Clicking
on them takes you to the corresponding Gene Ontology web page.

If you cannot see a graph here at all, that means that there were no
significantly enriched GO categories, at the 0.05 level.

— Click on the *Help* button again to close this help window.

:   **biological\_process**

    Any process specifically pertinent to the functioning of integrated living units: cells, tissues, organs, and organisms. A process is a collection of molecular events with a defined beginning and end.
:   **metabolic process**

    The chemical reactions and pathways, including anabolism and catabolism, by which living organisms transform chemical substances. Metabolic processes typically transform small molecules, but also include macromolecular processes such as DNA repair and replication, and protein synthesis and degradation.
:   **cellular process**

    Any process that is carried out at the cellular level, but not necessarily restricted to a single cell. For example, cell communication occurs among more than one cell, but occurs at the cellular level.
:   **carboxylic acid metabolic process**

    The chemical reactions and pathways involving carboxylic acids, any organic acid containing one or more carboxyl (COOH) groups or anions (COO-).
:   **monocarboxylic acid metabolic process**

    The chemical reactions and pathways involving monocarboxylic acids, any organic acid containing one carboxyl (COOH) group or anion (COO-).
:   **oxoacid metabolic process**

    The chemical reactions and pathways involving any oxoacid; an oxoacid is a compound which contains oxygen, at least one other element, and at least one hydrogen bound to oxygen, and which produces a conjugate base by loss of positive hydrogen ion(s) (hydrons).
:   **cellular metabolic process**

    The chemical reactions and pathways by which individual cells transform chemical substances.
:   **small molecule metabolic process**

    The chemical reactions and pathways involving small molecules, any low molecular weight, monomeric, non-encoded molecule.
:   **single-organism metabolic process**

    A metabolic process - chemical reactions and pathways, including anabolism and catabolism, by which living organisms transform chemical substances - which involves a single organism.
:   **organic substance metabolic process**

    The chemical reactions and pathways involving an organic substance, any molecular entity containing carbon.
:   **all**

    NA
:   **NA**

    NA
:   **small molecule metabolic process**

    The chemical reactions and pathways involving small molecules, any low molecular weight, monomeric, non-encoded molecule.
:   **biological\_process**

    Any process specifically pertinent to the functioning of integrated living units: cells, tissues, organs, and organisms. A process is a collection of molecular events with a defined beginning and end.
:   **biological\_process**

    Any process specifically pertinent to the functioning of integrated living units: cells, tissues, organs, and organisms. A process is a collection of molecular events with a defined beginning and end.

## Help | Hide | Top Help | Show | Top The GO tree — Cellular Components

### HELP

This is one of three sections showing Gene Ontology enrichment of the
current module: in this case for **cellular components**.

The graph shows the hierarchy of the GO categories, their enrichment
for the current module is color coded, and the blue number beside the
category is the minus log ten p-value of the enrichment. (Calculated
using the standard hypergeometric test.) The color of the arrows code
«is a» (cyan) and «part of» relationships.

The tree was built the following way. First all GO terms with more
significant enrichment p-value than 0.05 were collected. Then all
paths from these terms to the root node of the GO tree were included
too. If a GO term is included more than once in the tree, then the
green numbers show 1) the id of the node, this makes it easier to find
other appereances of the term, and 2) the number of appearences.

Note that the same GO category might show up on the graph many
times. This is because the GO was «straightened» for this
graph, i.e. if there are more paths from a GO term to the root node of
the tree, all of them are included. The green numbers

Move the mouse cursor over the terms to get their definition. Clicking
on them takes you to the corresponding Gene Ontology web page.

If you cannot see a graph here at all, that means that there were no
significantly enriched GO categories, at the 0.05 level.

— Click on the *Help* button again to close this help window.

## Help | Hide | Top Help | Show | Top The GO tree — Molecular Function

### HELP

This is one of three sections showing Gene Ontology enrichment of the
current module: in this case for **molecular function**.

The graph shows the hierarchy of the GO categories, their enrichment
for the current module is color coded, and the blue number beside the
category is the minus log ten p-value of the enrichment. (Calculated
using the standard hypergeometric test.) The color of the arrows code
«is a» (cyan) and «part of» relationships.

The tree was built the following way. First all GO terms with more
significant enrichment p-value than 0.05 were collected. Then all
paths from these terms to the root node of the GO tree were included
too. If a GO term is included more than once in the tree, then the
green numbers show 1) the id of the node, this makes it easier to find
other appereances of the term, and 2) the number of appearences.

Note that the same GO category might show up on the graph many
times. This is because the GO was «straightened» for this
graph, i.e. if there are more paths from a GO term to the root node of
the tree, all of them are included. The green numbers

Move the mouse cursor over the terms to get their definition. Clicking
on them takes you to the corresponding Gene Ontology web page.

If you cannot see a graph here at all, that means that there were no
significantly enriched GO categories, at the 0.05 level.

— Click on the *Help* button again to close this help window.

:   **molecular\_function**

    Elemental activities, such as catalysis or binding, describing the actions of a gene product at the molecular level. A given gene product may exhibit one or more molecular functions.
:   **catalytic activity**

    Catalysis of a biochemical reaction at physiological temperatures. In biologically catalyzed reactions, the reactants are known as substrates, and the catalysts are naturally occurring macromolecular substances known as enzymes. Enzymes possess specific binding sites for substrates, and are usually composed wholly or largely of protein, but RNA that has catalytic activity (ribozyme) is often also regarded as enzymatic.
:   **monooxygenase activity**

    Catalysis of the incorporation of one atom from molecular oxygen into a compound and the reduction of the other atom of oxygen to water.
:   **binding**

    The selective, non-covalent, often stoichiometric, interaction of a molecule with one or more specific sites on another molecule.
:   **metal ion binding**

    Interacting selectively and non-covalently with any metal ion.
:   **iron ion binding**

    Interacting selectively and non-covalently with iron (Fe) ions.
:   **oxidoreductase activity**

    Catalysis of an oxidation-reduction (redox) reaction, a reversible chemical reaction in which the oxidation state of an atom or atoms within a molecule is altered. One substrate acts as a hydrogen or electron donor and becomes oxidized, while the other acts as hydrogen or electron acceptor and becomes reduced.
:   **oxidoreductase activity, acting on paired donors, with incorporation or reduction of molecular oxygen**

    Catalysis of an oxidation-reduction (redox) reaction in which hydrogen or electrons are transferred from each of two donors, and molecular oxygen is reduced or incorporated into a donor.
:   **oxidoreductase activity, acting on paired donors, with incorporation or reduction of molecular oxygen, reduced flavin or flavoprotein as one donor, and incorporation of one atom of oxygen**

    Catalysis of an oxidation-reduction (redox) reaction in which hydrogen or electrons are transferred from reduced flavin or flavoprotein and one other donor, and one atom of oxygen is incorporated into one donor.
:   **heme binding**

    Interacting selectively and non-covalently with heme, any compound of iron complexed in a porphyrin (tetrapyrrole) ring.
:   **ion binding**

    Interacting selectively and non-covalently with ions, charged atoms or groups of atoms.
:   **cation binding**

    Interacting selectively and non-covalently with cations, charged atoms or groups of atoms with a net positive charge.
:   **tetrapyrrole binding**

    Interacting selectively and non-covalently with a tetrapyrrole, a compound containing four pyrrole nuclei variously substituted and linked to each other through carbons at the alpha position.
:   **transition metal ion binding**

    Interacting selectively and non-covalently with a transition metal ions; a transition metal is an element whose atom has an incomplete d-subshell of extranuclear electrons, or which gives rise to a cation or cations with an incomplete d-subshell. Transition metals often have more than one valency state. Biologically relevant transition metals include vanadium, manganese, iron, copper, cobalt, nickel, molybdenum and silver.
:   **aromatase activity**

    Catalysis of the reduction of an aliphatic ring to yield an aromatic ring.
:   **organic cyclic compound binding**

    Interacting selectively and non-covalently with an organic cyclic compound, any molecular entity that contains carbon arranged in a cyclic molecular structure.
:   **heterocyclic compound binding**

    Interacting selectively and non-covalently with heterocyclic compound.
:   **all**

    NA
:   **NA**

    NA
:   **transition metal ion binding**

    Interacting selectively and non-covalently with a transition metal ions; a transition metal is an element whose atom has an incomplete d-subshell of extranuclear electrons, or which gives rise to a cation or cations with an incomplete d-subshell. Transition metals often have more than one valency state. Biologically relevant transition metals include vanadium, manganese, iron, copper, cobalt, nickel, molybdenum and silver.
:   **heme binding**

    Interacting selectively and non-covalently with heme, any compound of iron complexed in a porphyrin (tetrapyrrole) ring.

## Help | Hide | Top Help | Show | Top GO BP test for over-representation

### HELP

List of all enriched GO categories (biological processes), at the 0.05
p-value level.

The columns:

- **ExpCount** is the expected count of genes in the
  module annotated with the given GO term, just by chance.
- **Count**
  is the number of genes in the module annotated with the given GO
  term.
- **Size** is the total number of genes (in our universe)
  annotated with the GO term.

Clicking on **Count** shows the genes that drive the
enrichment. You can also click on the individual numbers in
the **Count** column, to show the driving genes for that individual
GO category.

Clicking on the GO identifiers takes you to the Gene Ontology web
pages.

— Click on the *Help* button again to close this help window.

| Id | Pvalue | ExpCount | Count | Size | Term |
| --- | --- | --- | --- | --- | --- |
| GO:0032787 | 2.286e-03 | 22.41 | 48 Abcd1, Abhd5, Acaa2, Acot3, Acot4, Acox2, Acsl1, Acsl4, Adh7, Adipoq, Agt, Akr1d1, Apoa2, Apoa5, Apoc2, Arv1, Cpt1a, Cpt1b, Cyp1a1, Cyp26a1, Cyp26b1, Cyp2j6, Cyp4a10, Cyp4f14, Echs1, Edn1, Elovl1, Elovl3, Fabp1, Hadh, Hnf4a, Htt, Insig1, Ldha, Mgll, Mid1ip1, Nr1h2, Pkm, Prkaa2, Ptplad1, Rdh9, Sc5d, Sirt4, Slc27a1, Slc27a2, Sphk1, Tmlhe, Ucp3 | 351 | monocarboxylic acid metabolic process |
| GO:0006629 | 1.047e-02 | 55.79 | 91 Abcd1, Abhd5, Acaa2, Acot3, Acot4, Acox2, Acsl1, Acsl4, Adh7, Adipoq, Adm, Adora1, Adora2b, Agt, Akr1d1, Aplp2, Apoa1, Apoa2, Apoa5, Apobr, Apoc2, Apoe, Arv1, Asah1, B4galt6, Colgalt2, Cpt1a, Cpt1b, Cyp1a1, Cyp26a1, Cyp26b1, Cyp27a1, Cyp2b10, Cyp2e1, Cyp2j6, Cyp46a1, Cyp4a10, Cyp4f14, Cyp51, Ddhd2, Dgkd, Echs1, Edn1, Elovl1, Elovl3, Fabp1, Fabp3, Fdxr, Gm2a, Hadh, Hmgcr, Hnf4a, Hsd17b1, Hsd3b2, Hsd3b3, Impa2, Insig1, Ip6k1, Ipmk, Lamtor1, Map7, Mgll, Mid1ip1, Nr1h2, Ocrl, Opa3, Pdss1, Pigf, Pigyl, Pik3r6, Plbd1, Plbd2, Plin1, Ppap2b, Ppt1, Prkaa2, Ptplad1, Rarres2, Rdh12, Rdh9, Sc5d, Sirt4, Slc27a1, Slc27a2, Smpd1, Sphk1, Spns2, St6galnac1, Sult1a1, Tecrl, Ucp3 | 874 | lipid metabolic process |
| GO:0006631 | 1.643e-02 | 17.17 | 38 Abcd1, Abhd5, Acaa2, Acot3, Acot4, Acox2, Acsl1, Acsl4, Adh7, Adipoq, Agt, Apoa2, Apoa5, Apoc2, Cpt1a, Cpt1b, Cyp2j6, Cyp4a10, Cyp4f14, Echs1, Edn1, Elovl1, Elovl3, Fabp1, Hadh, Hnf4a, Insig1, Mgll, Mid1ip1, Nr1h2, Prkaa2, Ptplad1, Sc5d, Sirt4, Slc27a1, Slc27a2, Sphk1, Ucp3 | 269 | fatty acid metabolic process |
| GO:0006805 | 1.692e-02 | 1.724 | 10 Acsl1, Ahrr, Cyp1a1, Cyp2b10, Cyp2e1, Cyp2f2, Cyp46a1, Gstm5, Hnf4a, Sult1a1 | 27 | xenobiotic metabolic process |
| GO:0071466 | 1.692e-02 | 1.724 | 10 Acsl1, Ahrr, Cyp1a1, Cyp2b10, Cyp2e1, Cyp2f2, Cyp46a1, Gstm5, Hnf4a, Sult1a1 | 27 | cellular response to xenobiotic stimulus |
| GO:0006869 | 2.005e-02 | 11.55 | 29 Abcd1, Acsl1, Acsl4, Adipoq, Apoa1, Apoa2, Apoa5, Apobr, Apoc2, Apoe, Arv1, Atp10a, Bdkrb2, Cpt1b, Fabp1, Fabp3, Ghrl, Gltp, Got2, Lamtor1, Msr1, Nr1h2, Osbpl7, Repin1, Runx1, Slc27a1, Slc27a2, Spns2, Vps4b | 181 | lipid transport |

## Help | Hide | Top Help | Show | Top GO CC test for over-representation

### HELP

List of all enriched GO categories (cellular components), at the 0.05
p-value level.

The columns:

- **ExpCount** is the expected count of genes in the
  module annotated with the given GO term, just by chance.
- **Count**
  is the number of genes in the module annotated with the given GO
  term.
- **Size** is the total number of genes (in our universe)
  annotated with the GO term.

Clicking on **Count** shows the genes that drive the
enrichment. You can also click on the individual numbers in
the **Count** column, to show the driving genes for that individual
GO category.

Clicking on the GO identifiers takes you to the Gene Ontology web
pages.

— Click on the *Help* button again to close this help window.

| Id | Pvalue | ExpCount | Count | Size | Term |
| --- | --- | --- | --- | --- | --- |
| GO:0044444 | 4.905e-02 | 328.1 | 383 5033411D12Rik, Abcd1, Abcf2, Abhd11, Abhd5, Abl2, Acaa2, Ache, Acot13, Acot3, Acot4, Acox2, Acsl1, Acsl4, Adipoq, Adora1, Agl, Agt, Agtrap, Ak3, Ak4, Akap11, Akap13, Akap7, Akr1d1, Alas2, Ankrd1, Anxa9, Ap4m1, Apba3, Apoa1, Apoe, Appl2, Aqp5, Aqp6, Arcn1, Arsg, Asah1, Aspscr1, Astn1, Atl1, Atp10a, Atp6v1c1, Aurka, Axin2, B3gat2, B4galnt3, B4galt5, B4galt6, Bace1, Bckdhb, Bcl2l13, Bdkrb2, Bgn, Bloc1s3, Bloc1s4, Bok, Cabp4, Cacna1g, Cad, Caly, Car3, Cars, Casp9, Cbr2, Ccdc33, Cd300lh, Cd3eap, Cdk16, Cdk5, Cdkn1b, Cep192, Ces1e, Chchd3, Chmp1b, Chmp2a, Chst1, Chst8, Chsy1, Chuk, Cideb, Clasp2, Clec2g, Cln5, Cnot1, Cntf, Cog4, Colgalt2, Cox18, Cox6a1, Cox7c, Cpe, Cpox, Cpt1a, Cpt1b, Creb3l4, Ctso, Cul2, Cx3cr1, Cyfip2, Cyp1a1, Cyp26a1, Cyp26b1, Cyp27a1, Cyp2a12, Cyp2b10, Cyp2c29, Cyp2c37, Cyp2c50, Cyp2d9, Cyp2e1, Cyp2f2, Cyp2j5, Cyp2j6, Cyp46a1, Cyp4a10, Cyp4f14, Cyp51, Dact1, Ddhd2, Dgkd, Dhodh, Dmtn, Dnaic2, Dnajc1, Dus2l, Dync1li1, Dzip1l, Echs1, Edem3, Eif4e, Elovl1, Elovl3, Emc1, Ephx1, Epm2aip1, Epn2, Eral1, Esd, Fabp1, Fabp3, Faf1, Fahd2a, Fam109a, Fcho2, Fdxr, Fgd1, Fgfr1, Fkbp2, Flot1, Fmnl1, Fndc5, Furin, Fzd8, Fzd9, Gal3st3, Galnt12, Ganab, Gcsh, Gga1, Glrx3, Glyctk, Gm2a, Gnb2l1, Gnptg, Gorasp1, Gorasp2, Got2, Gpr161, Gstp1, Gstt2, Hadh, Hagh, Hbb-b2, Hebp1, Hectd3, Higd2a, Hmgb2, Hmgcr, Hnrnpu, Hpd, Hprt, Hs2st1, Hsd17b1, Hsd3b2, Hsd3b3, Htt, Ifngr1, Ift57, Igfbp2, Insig1, Isoc2b, Jagn1, Jup, Kank2, Kcna5, Ktn1, Ky, Lace1, Lamtor1, Lcp1, Ldha, Leprot, Lgals3, Lgr5, Manba, Maneal, Map1lc3a, Marc1, March5, Mars, Med15, Mff, Mgp, Mib2, Mid1ip1, Mmaa, Mnf1, Mocs2, Mpv17l2, Mrpl22, Mrpl50, Mrps10, Mrps6, Msr1, Mtfr1, Mthfr, Mul1, Myl4, Myl9, Myo1a, Myod1, Myof, Myom1, Mypn, ND3, ND5, Ndfip2, Ndufa6, Ndufaf4, Ndufaf5, Ndufb10, Ndufb11, Ndufb3, Ndufb6, Nipsnap3b, Nit1, Nos1, Ntm, Ocln, Ocrl, Ofd1, Opa3, Osbpl7, Pank1, Papolb, Park2, Pcca, Pdp1, Pdp2, Pdss1, Pfkm, Pgm2, Pgpep1, Piezo1, Pigf, Pigyl, Pik3r6, Pin4, Pkm, Pkp4, Plbd2, Plin1, Plk2, Plod1, Poc1a, Ppap2b, Ppp1r16a, Ppp1r3b, Ppp1r3c, Ppp2r2b, Ppt1, Pptc7, Prdm2, Prnp, Prosc, Prrc1, Psma6, Psmc2, Ptn, Ptplad1, Ptprm, Qtrt1, Rab11b, Rab13, Rab14, Rab23, Rab2a, Rangap1, Rapgef1, Rcbtb2, Repin1, Rer1, Rgs13, Rilp, Rnf144a, Rpl18, Rpl18a, Rps14, Rps16, Rpusd4, Rrp1b, Rsph9, Ryr2, Ryr3, S100a16, Sc5d, Scyl1, Sdhaf2, Sdhb, Sec61a2, Sec63, Sema4c, Sept8, Serp1, Sgip1, Shroom2, Sirt4, Sirt5, Slc22a18, Slc25a21, Slc25a47, Slc27a1, Slc27a2, Slc35g2, Slc39a6, Slirp, Smpd1, Sox10, Sphk1, St6galnac1, Stmn2, Sult1a1, Suox, Svip, Syn1, Syndig1, Syt11, Syt17, Tmc8, Tmed3, Tmed5, Tmed9, Tmem106b, Tmem33, Tmem38a, Tmlhe, Tnni3, Tomm5, Tprkb, Trak1, Trap1, Trappc2l, Tubg1, Ubash3a, Ucp2, Ucp3, Ugdh, Upk2, Uqcr10, Usp8, Uxt, Vdac3, Vegfa, Vps41, Vps4b, Wipf1, Xrcc3, Xrn1, Yrdc, Zap70, Zdhhc3, Zmiz2 | 5182 | cytoplasmic part |

## Help | Hide | Top Help | Show | Top GO MF test for over-representation

### HELP

List of all enriched GO categories (molecular function), at the 0.05
p-value level.

The columns:

- **ExpCount** is the expected count of genes in the
  module annotated with the given GO term, just by chance.
- **Count**
  is the number of genes in the module annotated with the given GO
  term.
- **Size** is the total number of genes (in our universe)
  annotated with the GO term.

Clicking on **Count** shows the genes that drive the
enrichment. You can also click on the individual numbers in
the **Count** column, to show the driving genes for that individual
GO category.

Clicking on the GO identifiers takes you to the Gene Ontology web
pages.

— Click on the *Help* button again to close this help window.

| Id | Pvalue | ExpCount | Count | Size | Term |
| --- | --- | --- | --- | --- | --- |
| GO:0046906 | 3.770e-05 | 6.685 | 24 Cyp1a1, Cyp26a1, Cyp26b1, Cyp27a1, Cyp2a12, Cyp2b10, Cyp2c29, Cyp2c37, Cyp2c50, Cyp2d9, Cyp2e1, Cyp2f2, Cyp2j5, Cyp2j6, Cyp46a1, Cyp4a10, Cyp4f14, Cyp51, Hbb-b2, Hebp1, Mtr, Nos1, Pgrmc2, Suox | 105 | tetrapyrrole binding |
| GO:0020037 | 4.296e-05 | 6.239 | 23 Cyp1a1, Cyp26a1, Cyp26b1, Cyp27a1, Cyp2a12, Cyp2b10, Cyp2c29, Cyp2c37, Cyp2c50, Cyp2d9, Cyp2e1, Cyp2f2, Cyp2j5, Cyp2j6, Cyp46a1, Cyp4a10, Cyp4f14, Cyp51, Hbb-b2, Hebp1, Nos1, Pgrmc2, Suox | 98 | heme binding |
| GO:0016712 | 9.204e-05 | 1.528 | 11 Cyp1a1, Cyp2a12, Cyp2b10, Cyp2c29, Cyp2c37, Cyp2c50, Cyp2d9, Cyp2e1, Cyp2f2, Cyp2j5, Cyp2j6 | 24 | oxidoreductase activity, acting on paired donors, with incorporation or reduction of molecular oxygen, reduced flavin or flavoprotein as one donor, and incorporation of one atom of oxygen |
| GO:0005506 | 2.498e-04 | 8.404 | 26 1300017J02Rik, Cyp1a1, Cyp26a1, Cyp26b1, Cyp27a1, Cyp2a12, Cyp2b10, Cyp2c29, Cyp2c37, Cyp2c50, Cyp2d9, Cyp2e1, Cyp2f2, Cyp2j5, Cyp2j6, Cyp46a1, Cyp4a10, Cyp4f14, Cyp51, Hbb-b2, Nos1, Ogfod2, Plod1, Ppef2, Sc5d, Tmlhe | 132 | iron ion binding |
| GO:0009055 | 4.092e-04 | 5.985 | 21 Cyp1a1, Cyp26a1, Cyp26b1, Cyp27a1, Cyp2a12, Cyp2b10, Cyp2c29, Cyp2c37, Cyp2c50, Cyp2d9, Cyp2e1, Cyp2f2, Cyp2j5, Cyp2j6, Cyp46a1, Cyp4a10, Cyp4f14, Cyp51, Glrx3, Sdhb, Suox | 94 | electron carrier activity |
| GO:0070330 | 4.663e-03 | 1.401 | 9 Cyp1a1, Cyp2a12, Cyp2b10, Cyp2c29, Cyp2c37, Cyp2c50, Cyp2d9, Cyp2j5, Cyp2j6 | 22 | aromatase activity |
| GO:0004497 | 6.063e-03 | 5.921 | 19 Cyp1a1, Cyp26a1, Cyp26b1, Cyp27a1, Cyp2a12, Cyp2b10, Cyp2c29, Cyp2c37, Cyp2c50, Cyp2d9, Cyp2e1, Cyp2f2, Cyp2j5, Cyp2j6, Cyp46a1, Cyp4a10, Cyp4f14, Cyp51, Nos1 | 93 | monooxygenase activity |

## Help | Hide | Top Help | Show | Top KEGG Pathway test for over-representation

### HELP

List of all enriched KEGG pathways, at the 0.05
p-value level.

The columns:

- **ExpCount** is the expected count of genes in the
  module annotated with the given KEGG pathway, just by chance.
- **Count**
  is the number of genes in the module annotated with the given KEGG
  pathway.
- **Size** is the total number of genes (in our universe)
  annotated with the KEGG pathway.

Clicking on **Count** shows the genes that drive the
enrichment. You can also click on the individual numbers in
the **Count** column, to show the driving genes for that individual
KEGG pathway.

Clicking on the KEGG identifiers takes you to the KEGG web site.

— Click on the *Help* button again to close this help window.

| Id | Pvalue | ExpCount | Count | Size | Term |
| --- | --- | --- | --- | --- | --- |
| 03320 | 5.491e-04 | 4.768 | 17 Acox2, Acsl1, Acsl4, Adipoq, Apoa1, Apoa2, Apoa5, Aqp7, Cpt1a, Cpt1b, Cyp27a1, Cyp4a10, Fabp1, Fabp3, Plin1, Slc27a1, Slc27a2 | 69 | PPAR signaling pathway |


### HELP

List of all enriched miRNA families, at the 0.05
p-value level.

The columns:

- **ExpCount** is the expected count of genes in the
  module regulated by the given miRNA family, just by chance.
- **Count**
  is the number of genes in the module regulated by the given miRNA
  family.
- **Size** is the total number of genes (in our universe)
  regulated with the given miRNA family.

Clicking on **Count** shows the genes that drive the
enrichment. You can also click on the individual numbers in
the **Count** column, to show the driving genes for that individual
miRNA family.

The miRNA regulation data was taken from the

Top


### HELP

p-value level.

The columns:

- **ExpCount** is the expected number of genes in the- **Count**- **Size** is the total number of genes (in our universe)

Clicking on **Count** shows the genes that drive the
enrichment. You can also click on the individual numbers in
the **Count** column, to show the driving genes for that individual

— Click on the *Help* button again to close this help window.

## Help | Hide | Top Help | Show | Top Genes

### HELP

A list of all genes in the current module, in alphabetical order. The
size of the text corresponds to the gene scores.

Note that some gene symbols may show up more than once, if many
probes match the same Entrez gene.

Genes with no Entrez mapping are given separately, with their
Affymetrics probe ID.

— Click on the *Help* button again to close this help window.

### Genes Symbol

, score:

Abl2Unknown, score: 0.45
AcheUnknown, score: 0.58
AdipoqUnknown, score: 0.62
Adh7Unknown, score: 0.34
AdmUnknown, score: 0.49
Adora1Unknown, score: 0.4
Adora2bUnknown, score: 0.49
Adra1bUnknown, score: 0.4
AgtUnknown, score: 0.39
AgtrapUnknown, score: 0.49
AhrrUnknown, score: 0.48
Ak4Unknown, score: 0.42
Alas2Unknown, score: 0.48
Abcd1Unknown, score: 0.46
Alx4Unknown, score: 0.58
Ampd3Unknown, score: 0.65
Ap4m1Unknown, score: 0.35
Aplp2Unknown, score: 0.72
Apoa1Unknown, score: 0.56
Apoa2Unknown, score: 0.51
Apobec2Unknown, score: 0.46
Apoc2Unknown, score: 0.53
ApoeUnknown, score: 0.46
AprtUnknown, score: 0.61
Aqp5Unknown, score: 0.43
Aqp6Unknown, score: 0.51
Aqp7Unknown, score: 0.48
ArtnUnknown, score: 0.57
Asah1Unknown, score: 0.56
Astn1Unknown, score: 0.58
Fxyd2Unknown, score: 0.42
Atp10aUnknown, score: 0.49
Axin2Unknown, score: 0.5
BckdhbUnknown, score: 0.35
Bcl7bUnknown, score: 0.38
Bdkrb2Unknown, score: 0.61
BgnUnknown, score: 0.61
Prdm1Unknown, score: 0.43
C1qbUnknown, score: 0.34
C4bUnknown, score: 0.51
C6Unknown, score: 0.59
Cacna1gUnknown, score: 0.66
AspmUnknown, score: 0.45
Car3Unknown, score: 0.41
Car7Unknown, score: 0.39
Casp9Unknown, score: 0.36
Runx1Unknown, score: 0.42
CbfbUnknown, score: 0.52
Cbr2Unknown, score: 0.48
Entpd2Unknown, score: 0.48
Cdk11bUnknown, score: 0.36
Cdk5Unknown, score: 0.48
Cdkn1bUnknown, score: 0.41
Chek1Unknown, score: 0.63
ChukUnknown, score: 0.36
CidebUnknown, score: 0.38
Elovl3Unknown, score: 0.4
Clca3a1Unknown, score: 0.53
ClockUnknown, score: 0.35
Cxcr3Unknown, score: 0.34
CntfUnknown, score: 0.58
Col12a1Unknown, score: 0.38
CompUnknown, score: 0.55
Cox6a1Unknown, score: 0.46
Cox7cUnknown, score: 0.36
CpeUnknown, score: 0.4
CpoxUnknown, score: 0.59
Cpt1aUnknown, score: 0.68
Cpt1bUnknown, score: 0.43
CraddUnknown, score: 0.38
Pcdha11Unknown, score: 0.4
Cx3cr1Unknown, score: 0.51
Cyp1a1Unknown, score: 0.58
Cyp26a1Unknown, score: 0.63
Cyp2a12Unknown, score: 0.42
Cyp2b10Unknown, score: 0.66
Cyp2c29Unknown, score: 0.53
Cyp2c37Unknown, score: 0.61
Cyp2d9Unknown, score: 0.37
Cyp2e1Unknown, score: 0.53
Cyp2f2Unknown, score: 0.65
Cyp2j5Unknown, score: 0.41
Cyp2j6Unknown, score: 0.66
Cyp46a1Unknown, score: 0.64
Cyp4a10Unknown, score: 0.38
Cyp51Unknown, score: 0.39
DbpUnknown, score: 0.37
Dclk1Unknown, score: 0.72
Dnajc1Unknown, score: 0.38
Dpep1Unknown, score: 0.34
Edn1Unknown, score: 0.56
Eef2kUnknown, score: 0.6
Eif4a2Unknown, score: 0.67
Eif4eUnknown, score: 0.4
DmtnUnknown, score: 0.65
StomUnknown, score: 0.55
Ephb2Unknown, score: 0.36
Ephx1Unknown, score: 0.61
Epn2Unknown, score: 0.35
ErhUnknown, score: 0.63
EsdUnknown, score: 0.5
Ces1eUnknown, score: 0.6
F10Unknown, score: 0.5
F13bUnknown, score: 0.57
Fabp3Unknown, score: 0.62
Fabp1Unknown, score: 0.36
Acsl1Unknown, score: 0.57
Faf1Unknown, score: 0.37
Fbn1Unknown, score: 0.39
Fbn2Unknown, score: 0.6
Fbp2Unknown, score: 0.54
FdxrUnknown, score: 0.72
Lgr5Unknown, score: 0.67
Fgd1Unknown, score: 0.49
Fgf5Unknown, score: 0.47
Fgfr1Unknown, score: 0.5
Fkbp2Unknown, score: 0.39
Flot1Unknown, score: 0.39
Fzd8Unknown, score: 0.49
Fzd9Unknown, score: 0.39
GanabUnknown, score: 0.45
Gabpb1Unknown, score: 0.34
Gbx2Unknown, score: 0.65
Gjc1Unknown, score: 0.62
HaghUnknown, score: 0.48
Gm2aUnknown, score: 0.37
Gnb2l1Unknown, score: 0.84
Gngt2Unknown, score: 0.47
Got2Unknown, score: 0.34
Emg1Unknown, score: 0.43
Gsta3Unknown, score: 0.37
Gstm5Unknown, score: 0.36
Gstp1Unknown, score: 0.48
Gstt2Unknown, score: 0.35
H3f3aUnknown, score: 0.38
HadhUnknown, score: 0.47
Hand2Unknown, score: 0.46
Hbb-bsUnknown, score: 0.38
Hcn1Unknown, score: 0.46
HttUnknown, score: 0.57
Hebp1Unknown, score: 0.49
HgfUnknown, score: 0.36
Hic1Unknown, score: 0.38
HmgcrUnknown, score: 0.82
Hn1Unknown, score: 0.58
Hnf4aUnknown, score: 0.36
Onecut1Unknown, score: 0.45
HpcaUnknown, score: 0.61
HpdUnknown, score: 0.55
HprtUnknown, score: 0.38
Hsd17b1Unknown, score: 0.57
Hsd3b2Unknown, score: 0.51
Hsd3b3Unknown, score: 0.57
Ifngr1Unknown, score: 0.47
IgfalsUnknown, score: 0.75
Igfbp2Unknown, score: 0.65
Irx2Unknown, score: 0.67
Itga5Unknown, score: 0.5
Cd47Unknown, score: 0.6
Itih2Unknown, score: 0.37
F11rUnknown, score: 0.53
JupUnknown, score: 0.55
Kcna3Unknown, score: 0.58
Kcna5Unknown, score: 0.45
Kifc5bUnknown, score: 0.58
Klrd1Unknown, score: 0.63
Ktn1Unknown, score: 0.34
KyUnknown, score: 0.41
Lbx2Unknown, score: 0.35
LdhaUnknown, score: 0.35
Lgals3Unknown, score: 0.42
Lmnb2Unknown, score: 0.75
Lrp8Unknown, score: 0.36
Lrrc23Unknown, score: 0.35
LumUnknown, score: 0.39
Ly96Unknown, score: 0.55
Mef2dUnknown, score: 0.45
MetUnknown, score: 0.36
MgpUnknown, score: 0.37
Bhlha15Unknown, score: 0.34
Mocs2Unknown, score: 0.37
mt-Nd3Unknown, score: 0.34
mt-Nd5Unknown, score: 0.4
Map7Unknown, score: 0.67
MthfrUnknown, score: 0.54
Mast2Unknown, score: 0.64
17831Unknown, score: 0.78
Mug1Unknown, score: 0.6
Mup1Unknown, score: 0.68
Myl4Unknown, score: 0.47
Myo7bUnknown, score: 0.38
Myod1Unknown, score: 0.43
Myom1Unknown, score: 0.89
Nfatc1Unknown, score: 0.46
Nos1Unknown, score: 0.35
Npas1Unknown, score: 0.43
Npm3Unknown, score: 0.48
Ntan1Unknown, score: 0.43
Ddr2Unknown, score: 0.62
Nxph1Unknown, score: 0.39
OclnUnknown, score: 0.69
Slc22a18Unknown, score: 0.48
Hspa4lUnknown, score: 0.43
Pbx3Unknown, score: 0.42
Pcp2Unknown, score: 0.46
FurinUnknown, score: 0.35
Cdk16Unknown, score: 0.58
Per3Unknown, score: 0.51
CfpUnknown, score: 0.42
PfkmUnknown, score: 0.55
PigfUnknown, score: 0.53
PkmUnknown, score: 0.38
PkiaUnknown, score: 0.47
PlaaUnknown, score: 0.39
Pa2g4Unknown, score: 0.63
PlgUnknown, score: 0.46
Plod1Unknown, score: 0.49
Lcp1Unknown, score: 0.38
Pold2Unknown, score: 0.44
Ppef2Unknown, score: 0.68
Ppt1Unknown, score: 0.7
PrnpUnknown, score: 0.59
Psmc2Unknown, score: 0.52
PtnUnknown, score: 0.39
PtprdUnknown, score: 0.38
PtprmUnknown, score: 0.72
PtprtUnknown, score: 0.35
Pvrl2Unknown, score: 0.36
Rab11bUnknown, score: 0.55
Rab19Unknown, score: 0.53
Rab23Unknown, score: 0.36
Rangap1Unknown, score: 0.38
RdxUnknown, score: 0.61
Rev3lUnknown, score: 0.6
RfxankUnknown, score: 0.49
RhdUnknown, score: 0.44
RoraUnknown, score: 0.41
Rp2hUnknown, score: 0.47
RpiaUnknown, score: 0.57
Rpl18Unknown, score: 0.41
Rps14Unknown, score: 0.41
Rps16Unknown, score: 0.54
Rps6ka2Unknown, score: 0.37
Ryr2Unknown, score: 0.59
Ryr3Unknown, score: 0.46
Saa3Unknown, score: 0.67
Khdrbs1Unknown, score: 0.62
Stmn2Unknown, score: 0.56
Msr1Unknown, score: 0.37
Sema4cUnknown, score: 0.58
Sema5bUnknown, score: 0.36
Sept8Unknown, score: 0.49
Shfm1Unknown, score: 0.37
St6galnac1Unknown, score: 0.43
Vps4bUnknown, score: 0.37
Slc20a1Unknown, score: 0.37
Slc22a3Unknown, score: 0.37
Smpd1Unknown, score: 0.46
Plk2Unknown, score: 0.35
Sos2Unknown, score: 0.49
Sox10Unknown, score: 0.39
Sphk1Unknown, score: 0.41
Serpina1bUnknown, score: 0.64
Serpina1cUnknown, score: 0.41
Serpina1aUnknown, score: 0.37
Serpinb9bUnknown, score: 0.6
Serpini1Unknown, score: 0.66
AurkaUnknown, score: 0.57
Sult1a1Unknown, score: 0.37
Syn1Unknown, score: 0.34
Tacr3Unknown, score: 0.48
Tcf7l1Unknown, score: 0.35
Phf1Unknown, score: 0.71
Terf1Unknown, score: 0.47
Tex261Unknown, score: 0.4
Klf10Unknown, score: 0.42
Tnni3Unknown, score: 0.66
Tpp2Unknown, score: 0.41
Nr2c2Unknown, score: 0.41
Ucp2Unknown, score: 0.55
Ucp3Unknown, score: 0.35
UgdhUnknown, score: 0.59
Nr1h2Unknown, score: 0.61
Upk2Unknown, score: 0.77
UxtUnknown, score: 0.67
Vdac3Unknown, score: 0.54
VegfaUnknown, score: 0.35
Vil1Unknown, score: 0.4
VipUnknown, score: 0.39
WasUnknown, score: 0.36
Wnt5bUnknown, score: 0.39
XpaUnknown, score: 0.49
Zap70Unknown, score: 0.43
Zfp27Unknown, score: 0.36
Zfp28Unknown, score: 0.49
Zfp64Unknown, score: 0.36
Ikzf3Unknown, score: 0.35
Arih2Unknown, score: 0.34
Bace1Unknown, score: 0.49
Cdc6Unknown, score: 0.41
Gmcl1Unknown, score: 0.36
Gpr34Unknown, score: 0.44
Hs2st1Unknown, score: 0.37
KelUnknown, score: 0.5
Ly6hUnknown, score: 0.46
MgllUnknown, score: 0.51
Nr0b2Unknown, score: 0.45
Slc26a4Unknown, score: 0.72
Psmd13Unknown, score: 0.48
Tiam2Unknown, score: 0.38
IkUnknown, score: 0.4
Sh3bp2Unknown, score: 0.45
Spry1Unknown, score: 0.34
Spry2Unknown, score: 0.49
NelfaUnknown, score: 0.41
Xrn1Unknown, score: 0.36
Abcg2Unknown, score: 0.45
EsrraUnknown, score: 0.45
Mapk13Unknown, score: 0.47
Psma6Unknown, score: 0.36
Sema4gUnknown, score: 0.4
Slc27a1Unknown, score: 0.61
Slc27a2Unknown, score: 0.4
PcloUnknown, score: 0.45
Cops6Unknown, score: 0.42
Med14Unknown, score: 0.74
Nit1Unknown, score: 0.4
CarsUnknown, score: 0.39
Insl6Unknown, score: 0.46
Csnk1eUnknown, score: 0.68
Ip6k1Unknown, score: 0.51
Abcf2Unknown, score: 0.41
Peg12Unknown, score: 0.52
NagluUnknown, score: 0.5
Tada1Unknown, score: 0.62
Mrpl50Unknown, score: 0.51
Tinf2Unknown, score: 0.43
Serp1Unknown, score: 0.48
Reep3Unknown, score: 0.34
Dcaf11Unknown, score: 0.55
Trpm2Unknown, score: 0.61
Slco1a4Unknown, score: 0.42
Mlf2Unknown, score: 0.4
Slamf6Unknown, score: 0.56
Glrx3Unknown, score: 0.78
Slc46a2Unknown, score: 0.59
Cbx8Unknown, score: 0.75
Ebi3Unknown, score: 0.38
Hist1h1cUnknown, score: 0.36
Acsl4Unknown, score: 0.43
Park2Unknown, score: 0.47
BokUnknown, score: 0.85
HnrnpuUnknown, score: 0.45
Ppp1r10Unknown, score: 0.44
Rhpn2Unknown, score: 0.45
Ddx56Unknown, score: 0.38
Acaa2Unknown, score: 0.41
SgtaUnknown, score: 0.4
Setd3Unknown, score: 0.34
ZwintUnknown, score: 0.39
Ccdc43Unknown, score: 0.57
Tspyl2Unknown, score: 0.78
Rbfox3Unknown, score: 0.35
Zmiz2Unknown, score: 0.36
Ppp1r3cUnknown, score: 0.42
Stk39Unknown, score: 0.44
Tuba8Unknown, score: 0.41
Col5a3Unknown, score: 0.39
Cdkl2Unknown, score: 0.5
Irf6Unknown, score: 0.47
Spag5Unknown, score: 0.68
Cyhr1Unknown, score: 0.38
Elovl1Unknown, score: 0.4
Elp5Unknown, score: 0.45
Ftsj1Unknown, score: 0.36
Msh4Unknown, score: 0.42
Pdss1Unknown, score: 0.36
NagkUnknown, score: 0.59
EspnUnknown, score: 0.36
Ak3Unknown, score: 0.36
MycbpUnknown, score: 0.4
B4galt5Unknown, score: 0.58
Net1Unknown, score: 0.45
GltpUnknown, score: 0.44
1110004F10RikUnknown, score: 0.61
Arpc3Unknown, score: 0.43
B4galt6Unknown, score: 0.64
DptUnknown, score: 0.4
Ybx3Unknown, score: 0.48
Tbk1Unknown, score: 0.37
PapolbUnknown, score: 0.47
Mpp6Unknown, score: 0.45
Rgs17Unknown, score: 0.38
Hspb3Unknown, score: 0.41
Clec7aUnknown, score: 0.7
DhodhUnknown, score: 0.49
Ccl28Unknown, score: 0.58
Brd4Unknown, score: 0.42
Apba3Unknown, score: 0.36
NelfcdUnknown, score: 0.39
Gigyf1Unknown, score: 0.42
Sec61a2Unknown, score: 0.44
Fmnl1Unknown, score: 0.48
CdonUnknown, score: 0.4
TescUnknown, score: 0.36
Eral1Unknown, score: 0.46
Hacd3Unknown, score: 0.69
Sorcs1Unknown, score: 0.45
Sall1Unknown, score: 0.56
Repin1Unknown, score: 0.54
GhrlUnknown, score: 0.5
Trappc2lUnknown, score: 0.51
Rab2aUnknown, score: 0.41
Dact1Unknown, score: 0.45
Rbm8aUnknown, score: 0.58
CenpkUnknown, score: 0.8
Qtrt1Unknown, score: 0.38
Foxj2Unknown, score: 0.76
Smoc2Unknown, score: 0.45
Mllt1Unknown, score: 0.35
Cyp4f14Unknown, score: 0.34
Mrps10Unknown, score: 0.36
NmiUnknown, score: 0.53
Dpysl5Unknown, score: 0.63
Sdhaf2Unknown, score: 0.44
Chchd3Unknown, score: 0.49
Tmed3Unknown, score: 0.51
Marc1Unknown, score: 0.59
Apoa5Unknown, score: 0.49
Uqcr10Unknown, score: 0.35
Med11Unknown, score: 0.44
PigylUnknown, score: 0.49
Atp6v1c1Unknown, score: 0.46
Snw1Unknown, score: 0.35
RtcaUnknown, score: 0.53
Dus2Unknown, score: 0.37
Ppp1r7Unknown, score: 0.44
2410004B18RikUnknown, score: 0.46
Hamp2Unknown, score: 0.35
Exosc7Unknown, score: 0.44
Polr2lUnknown, score: 0.47
Ndufb3Unknown, score: 0.46
Lamtor1Unknown, score: 0.5
Pgpep1Unknown, score: 0.52
Nipsnap3bUnknown, score: 0.4
NtpcrUnknown, score: 0.34
Esf1Unknown, score: 0.35
Ogfod2Unknown, score: 0.41
Sike1Unknown, score: 0.46
Hspbap1Unknown, score: 0.48
Map1lc3aUnknown, score: 0.45
Acot13Unknown, score: 0.48
Ppp1r2Unknown, score: 0.52
Plbd1Unknown, score: 0.48
Nhlrc2Unknown, score: 0.55
Mfsd1Unknown, score: 0.44
Naa16Unknown, score: 0.38
MtapUnknown, score: 0.5
Tmem107Unknown, score: 0.51
Pqlc1Unknown, score: 0.36
Edem3Unknown, score: 0.4
Thap4Unknown, score: 0.36
Higd2aUnknown, score: 0.44
Chd1Unknown, score: 0.48
2610002M06RikUnknown, score: 0.56
Trak1Unknown, score: 0.48
Ndufa6Unknown, score: 0.46
Fam103a1Unknown, score: 0.51
Pdzk1ip1Unknown, score: 0.41
Gatad1Unknown, score: 0.49
CinpUnknown, score: 0.35
Uqcc2Unknown, score: 0.4
Eri1Unknown, score: 0.36
Brd3Unknown, score: 0.41
Eps8l1Unknown, score: 0.35
Ccdc127Unknown, score: 0.44
Isoc2bUnknown, score: 0.52
NvlUnknown, score: 0.76
Gpalpp1Unknown, score: 0.36
Abhd5Unknown, score: 0.41
Mtfr1Unknown, score: 0.62
Dhx40Unknown, score: 0.63
Tmed9Unknown, score: 0.9
Ube2r2Unknown, score: 0.36
Lrp2bpUnknown, score: 0.38
SdhbUnknown, score: 0.35
Pcnxl4Unknown, score: 0.47
Jagn1Unknown, score: 0.48
Snrnp48Unknown, score: 0.5
Atp1b4Unknown, score: 0.53
Rer1Unknown, score: 0.34
Tmem39aUnknown, score: 0.42
S100a16Unknown, score: 0.4
Enoph1Unknown, score: 0.4
Tmem33Unknown, score: 0.45
Camsap2Unknown, score: 0.36
Ppap2bUnknown, score: 0.37
ZwilchUnknown, score: 0.34
Trap1Unknown, score: 0.45
Mid1ip1Unknown, score: 0.41
Fahd2aUnknown, score: 0.45
GcshUnknown, score: 0.43
Upf3bUnknown, score: 0.37
Stx19Unknown, score: 0.43
Bcas2Unknown, score: 0.46
Zmym1Unknown, score: 0.53
Lypd2Unknown, score: 0.53
Rab13Unknown, score: 0.41
Ndufb10Unknown, score: 0.41
Sirt5Unknown, score: 0.4
Mul1Unknown, score: 0.41
AspdhUnknown, score: 0.38
Rab14Unknown, score: 0.36
Ndufaf4Unknown, score: 0.4
Tomm5Unknown, score: 0.5
Col6a4Unknown, score: 0.42
CalyUnknown, score: 0.46
MocosUnknown, score: 0.38
Tmem216Unknown, score: 0.35
Ifitm1Unknown, score: 0.45
Angel1Unknown, score: 0.46
Rreb1Unknown, score: 0.45
Abhd11Unknown, score: 0.46
MypnUnknown, score: 0.34
Arv1Unknown, score: 0.39
1110065P20RikUnknown, score: 0.78
Rpap1Unknown, score: 0.39
Mospd3Unknown, score: 0.37
Aspscr1Unknown, score: 0.77
Chst8Unknown, score: 0.45
Chmp2aUnknown, score: 0.56
Paqr6Unknown, score: 0.36
Zdhhc3Unknown, score: 0.35
March5Unknown, score: 0.39
Dnajc17Unknown, score: 0.58
2300009A05RikUnknown, score: 0.48
Ndufaf5Unknown, score: 0.43
Klk10Unknown, score: 0.47
Wdr5bUnknown, score: 0.36
Klhdc2Unknown, score: 0.46
Mfsd3Unknown, score: 0.46
Ddx51Unknown, score: 0.36
Pin4Unknown, score: 0.63
IpmkUnknown, score: 0.39
CadUnknown, score: 0.34
Fbxo7Unknown, score: 0.73
Ms4a6bUnknown, score: 0.44
Smap2Unknown, score: 0.38
TprkbUnknown, score: 0.36
Glod5Unknown, score: 0.43
Pcgf1Unknown, score: 0.36
Ttc39bUnknown, score: 0.4
Coa7Unknown, score: 0.64
Zfp715Unknown, score: 0.44
Smc4Unknown, score: 0.53
Lrch3Unknown, score: 0.38
Gorasp2Unknown, score: 0.36
Poc1aUnknown, score: 0.43
Mkrn2osUnknown, score: 0.39
Cd3eapUnknown, score: 0.82
Arhgap17Unknown, score: 0.64
Ankrd24Unknown, score: 0.45
Cep192Unknown, score: 0.5
Pgrmc2Unknown, score: 0.39
Clec2gUnknown, score: 0.41
Prss41Unknown, score: 0.52
Osbpl7Unknown, score: 0.4
KaznUnknown, score: 0.39
Rarres2Unknown, score: 0.61
Tmem248Unknown, score: 0.42
Cul2Unknown, score: 0.51
Gm29609Unknown, score: 0.38
VwceUnknown, score: 0.39
Plbd2Unknown, score: 0.65
1300017J02RikUnknown, score: 0.37
Anxa9Unknown, score: 0.36
Tmem106bUnknown, score: 0.39
Rpusd4Unknown, score: 0.47
Cnot2Unknown, score: 0.43
Ush1cUnknown, score: 0.57
Ddhd2Unknown, score: 0.36
Dennd2dUnknown, score: 0.63
Pgm2Unknown, score: 0.37
Ccpg1Unknown, score: 0.4
Sh2d4aUnknown, score: 0.49
Faim2Unknown, score: 0.34
Rrp1bUnknown, score: 0.69
Tmem87bUnknown, score: 0.42
Dzip1lUnknown, score: 0.36
Zfp655Unknown, score: 0.43
Mex3aUnknown, score: 0.44
Rspo3Unknown, score: 0.43
Kctd17Unknown, score: 0.45
Cxx1cUnknown, score: 0.53
Macrod2Unknown, score: 0.34
Ppp2r2bUnknown, score: 0.34
Ppp1r16aUnknown, score: 0.35
Sgip1Unknown, score: 0.46
Tmed5Unknown, score: 0.45
Prrc1Unknown, score: 0.34
Gpbp1Unknown, score: 0.41
Ddit4lUnknown, score: 0.42
Dcbld2Unknown, score: 0.34
Tex35Unknown, score: 0.38
1700001O22RikUnknown, score: 0.41
Cabp4Unknown, score: 0.38
2410004P03RikUnknown, score: 0.37
Rbm20Unknown, score: 0.38
Kif2cUnknown, score: 0.35
Tmem198bUnknown, score: 0.51
Ift57Unknown, score: 0.36
Atl1Unknown, score: 0.47
ArsgUnknown, score: 0.53
Krt80Unknown, score: 0.58
F13a1Unknown, score: 0.59
Tmem38aUnknown, score: 0.36
Tubgcp2Unknown, score: 0.67
Rnf145Unknown, score: 0.4
Xrcc3Unknown, score: 0.44
Crtc2Unknown, score: 0.36
Ttc25Unknown, score: 0.45
Gorasp1Unknown, score: 0.7
Lmbr1lUnknown, score: 0.35
Rprd2Unknown, score: 0.38
Ccdc83Unknown, score: 0.39
Sirt4Unknown, score: 0.4
Akap13Unknown, score: 0.81
Rsph9Unknown, score: 0.45
Higd1bUnknown, score: 0.67
MffUnknown, score: 0.54
Pank1Unknown, score: 0.37
SvipUnknown, score: 0.52
Ipo4Unknown, score: 0.38
Abca6Unknown, score: 0.39
76224Unknown, score: 0.34
Ttc8Unknown, score: 0.35
Ndfip2Unknown, score: 0.53
Mfap4Unknown, score: 0.67
Gid8Unknown, score: 0.64
Pcolce2Unknown, score: 0.74
Ppp1r3gUnknown, score: 0.41
Clasp2Unknown, score: 0.49
Mib2Unknown, score: 0.35
Hectd3Unknown, score: 0.45
Rpl18aUnknown, score: 0.34
Cyfip2Unknown, score: 0.46
Jade2Unknown, score: 0.38
Lrg1Unknown, score: 0.37
Nt5c2Unknown, score: 0.37
Chst1Unknown, score: 0.62
2810007J24RikUnknown, score: 0.79
Arxes2Unknown, score: 0.64
Hdhd2Unknown, score: 0.39
Arid2Unknown, score: 0.47
Tmem181aUnknown, score: 0.37
AglUnknown, score: 0.47
Epm2aip1Unknown, score: 0.37
Lrrc42Unknown, score: 0.55
6030458C11RikUnknown, score: 0.49
Rdh12Unknown, score: 0.54
Prr15Unknown, score: 0.43
Creb3l4Unknown, score: 0.46
B230219D22RikUnknown, score: 0.42
Scyl1Unknown, score: 0.47
Kcnip4Unknown, score: 0.58
Senp3Unknown, score: 0.61
PolhUnknown, score: 0.54
MrgprhUnknown, score: 0.43
Vmn1r58Unknown, score: 0.39
Dnajb1Unknown, score: 0.49
SrrtUnknown, score: 0.55
Usp8Unknown, score: 0.38
Cd163Unknown, score: 0.56
Ear6Unknown, score: 0.54
Acox2Unknown, score: 0.36
Echs1Unknown, score: 0.51
Klra4Unknown, score: 0.34
Bcl2l13Unknown, score: 0.49
Med15Unknown, score: 0.65
Col4a6Unknown, score: 0.35
Lrp1bUnknown, score: 0.34
Hmgb2Unknown, score: 0.51
Nol12Unknown, score: 0.5
Cdc123Unknown, score: 0.37
Eps8l2Unknown, score: 0.4
Myl9Unknown, score: 0.36
Olfml3Unknown, score: 0.6
Papd4Unknown, score: 0.5
Grwd1Unknown, score: 0.41
Champ1Unknown, score: 0.61
Ces2aUnknown, score: 0.5
Cog4Unknown, score: 0.53
Lrrc49Unknown, score: 0.38
Rdh9Unknown, score: 0.52
Traf3ip2Unknown, score: 0.64
Zc3h10Unknown, score: 0.36
Tubg1Unknown, score: 0.79
Cuedc1Unknown, score: 0.34
Plin1Unknown, score: 0.36
Cyp27a1Unknown, score: 0.49
Ndufb11Unknown, score: 0.85
Cabin1Unknown, score: 0.41
Isl2Unknown, score: 0.55
Meig1Unknown, score: 0.48
Tsr1Unknown, score: 0.53
Slc16a6Unknown, score: 0.46
Pik3r6Unknown, score: 0.45
Tdp1Unknown, score: 0.43
Slc25a47Unknown, score: 0.37
Cdk20Unknown, score: 0.46
Rcbtb2Unknown, score: 0.45
Card10Unknown, score: 0.34
Gga1Unknown, score: 0.36
Wdr90Unknown, score: 0.4
VmacUnknown, score: 0.45
Slc39a6Unknown, score: 0.39
LarsUnknown, score: 0.42
Rrp12Unknown, score: 0.44
Cyp2c50Unknown, score: 0.4
LpxnUnknown, score: 0.37
Rapgef1Unknown, score: 0.51
Lgals2Unknown, score: 0.35
Ankrd1Unknown, score: 0.34
Usp9yUnknown, score: 0.38
Prkaa2Unknown, score: 0.34
Rnf144aUnknown, score: 0.66
Ak6Unknown, score: 0.38
Sft2d2Unknown, score: 0.46
Oxsr1Unknown, score: 0.53
Rnf169Unknown, score: 0.43
Uhrf2Unknown, score: 0.5
MmaaUnknown, score: 0.42
Tspan9Unknown, score: 0.35
Prr5Unknown, score: 0.38
Art4Unknown, score: 0.64
Syt17Unknown, score: 0.36
MpiUnknown, score: 0.69
ManbaUnknown, score: 0.57
DgkgUnknown, score: 0.53
Shroom2Unknown, score: 0.57
Prdm2Unknown, score: 0.57
Rps6ka3Unknown, score: 0.42
Cse1lUnknown, score: 0.44
PccaUnknown, score: 0.35
Slc9a4Unknown, score: 0.7
Hmga1-rs1Unknown, score: 0.39
Ly6g6dUnknown, score: 0.45
Impa2Unknown, score: 0.45
Spred1Unknown, score: 0.43
ProscUnknown, score: 0.39
Dph1Unknown, score: 0.56
Ube3bUnknown, score: 0.51
Bloc1s4Unknown, score: 0.45
Klf16Unknown, score: 0.34
Mrps6Unknown, score: 0.6
Sec63Unknown, score: 0.53
Bmp2kUnknown, score: 0.39
Recql5Unknown, score: 0.56
Smco4Unknown, score: 0.45
Asic3Unknown, score: 0.38
Acot3Unknown, score: 0.46
Acot4Unknown, score: 0.56
ApobrUnknown, score: 0.52
SugctUnknown, score: 0.48
Prpf8Unknown, score: 0.39
Pcdha12Unknown, score: 0.5
TmlheUnknown, score: 0.39
Rassf3Unknown, score: 0.57
BC049762Unknown, score: 0.65
Mtmr11Unknown, score: 0.53
NhsUnknown, score: 0.43
Zfp871Unknown, score: 0.68
Tspan10Unknown, score: 0.37
Fam20aUnknown, score: 0.58
Akr1d1Unknown, score: 0.45
Ulk4Unknown, score: 0.5
Samd9lUnknown, score: 0.39
Brcc3Unknown, score: 0.34
D130040H23RikUnknown, score: 0.44
Cln5Unknown, score: 0.72
SuoxUnknown, score: 0.4
Fancd2Unknown, score: 0.34
Vstm2aUnknown, score: 0.4
Scfd2Unknown, score: 0.5
Evi5lUnknown, score: 0.44
Wdr19Unknown, score: 0.45
Arcn1Unknown, score: 0.39
Tnrc6bUnknown, score: 0.73
GnptgUnknown, score: 0.4
4930486L24RikUnknown, score: 0.35
ManealUnknown, score: 0.62
DiexfUnknown, score: 0.4
Wipf1Unknown, score: 0.45
Mfsd6lUnknown, score: 0.69
Adgrg6Unknown, score: 0.43
Fam26fUnknown, score: 0.33
Lace1Unknown, score: 0.37
Zfp365Unknown, score: 0.53
Appl2Unknown, score: 0.64
Tcp11l2Unknown, score: 0.34
MarsUnknown, score: 0.53
Efemp1Unknown, score: 0.34
Mrpl22Unknown, score: 0.45
Spns2Unknown, score: 0.39
Proca1Unknown, score: 0.45
Prr15lUnknown, score: 0.37
Tmc8Unknown, score: 0.43
Trappc12Unknown, score: 0.35
Slc25a21Unknown, score: 0.36
CipcUnknown, score: 0.44
Nrde2Unknown, score: 0.35
Ifi27l2bUnknown, score: 0.5
Vps41Unknown, score: 0.35
Zfp322aUnknown, score: 0.42
Fcho2Unknown, score: 0.43
Akap11Unknown, score: 0.64
Itgbl1Unknown, score: 0.56
Arhgap39Unknown, score: 0.41
Zdhhc14Unknown, score: 0.39
Enpp4Unknown, score: 0.54
Treml4Unknown, score: 0.4
Fbxo11Unknown, score: 0.34
Lims2Unknown, score: 0.43
Cd226Unknown, score: 0.38
Eml3Unknown, score: 0.45
MyofUnknown, score: 0.61
Taf5Unknown, score: 0.71
Zbtb41Unknown, score: 0.38
TiprlUnknown, score: 0.44
Ccnyl1Unknown, score: 0.56
DgkdUnknown, score: 0.39
Cdh19Unknown, score: 0.35
Uap1l1Unknown, score: 0.42
Sh3glb2Unknown, score: 0.47
Ccdc148Unknown, score: 0.85
Pkp4Unknown, score: 0.36
Phf20Unknown, score: 0.59
CtsoUnknown, score: 0.45
Syt11Unknown, score: 0.43
Clca2Unknown, score: 0.42
Ndufb6Unknown, score: 0.36
Nol6Unknown, score: 0.36
Car9Unknown, score: 0.46
Galnt12Unknown, score: 0.47
Zfp189Unknown, score: 0.63
LeprotUnknown, score: 0.67
Tesk2Unknown, score: 0.47
Nt5c1aUnknown, score: 0.36
YrdcUnknown, score: 0.37
Fam167bUnknown, score: 0.35
Emc1Unknown, score: 0.43
Vps13dUnknown, score: 0.39
Insig1Unknown, score: 0.43
Tnip2Unknown, score: 0.34
Tapt1Unknown, score: 0.44
Ythdc1Unknown, score: 0.65
Cox18Unknown, score: 0.46
Fras1Unknown, score: 0.44
Plac8Unknown, score: 0.76
Vsig10Unknown, score: 0.55
Fam109aUnknown, score: 0.4
Mob1aUnknown, score: 0.74
Cyp26b1Unknown, score: 0.34
Hdac11Unknown, score: 0.38
Pyroxd1Unknown, score: 0.44
Zfp212Unknown, score: 0.48
Bloc1s3Unknown, score: 0.44
Luzp2Unknown, score: 0.41
P2ry6Unknown, score: 0.48
BC030336Unknown, score: 0.42
Zfp764Unknown, score: 0.4
Pcid2Unknown, score: 0.4
Neil3Unknown, score: 0.51
Zfp868Unknown, score: 0.44
Klhl26Unknown, score: 0.38
Mpv17l2Unknown, score: 0.34
Heatr3Unknown, score: 0.47
Cnot1Unknown, score: 0.41
Txnl4bUnknown, score: 0.55
Piezo1Unknown, score: 0.43
9230110C19RikUnknown, score: 0.49
Kank2Unknown, score: 0.42
Zfp599Unknown, score: 0.49
NtmUnknown, score: 0.38
Sc5dUnknown, score: 0.8
GlyctkUnknown, score: 0.36
Dync1li1Unknown, score: 0.47
Adgrg2Unknown, score: 0.44
Ofd1Unknown, score: 0.65
MyrflUnknown, score: 0.43
HexdcUnknown, score: 0.35
BC068281Unknown, score: 0.62
MtrUnknown, score: 0.57
Cdc20bUnknown, score: 0.37
Apol8Unknown, score: 0.35
Zfp760Unknown, score: 0.37
Fsd1Unknown, score: 0.36
Gpr161Unknown, score: 0.37
Fam78aUnknown, score: 0.52
Rbm45Unknown, score: 0.36
Phc3Unknown, score: 0.41
Pabpc4lUnknown, score: 0.41
1700024P16RikUnknown, score: 0.39
Ifnlr1Unknown, score: 0.46
Cct8l1Unknown, score: 0.59
TecrlUnknown, score: 0.34
H1fxUnknown, score: 0.46
Ccdc37Unknown, score: 0.69
SiglecgUnknown, score: 0.37
Olfml1Unknown, score: 0.71
Ppp1r3bUnknown, score: 0.41
Slc35g2Unknown, score: 0.34
Tbc1d8bUnknown, score: 0.63
Rbm47Unknown, score: 0.53
Fcgr4Unknown, score: 0.34
Rgs13Unknown, score: 0.47
Olfr39Unknown, score: 0.49
Nav3Unknown, score: 0.5
Ttc26Unknown, score: 0.79
Rapgefl1Unknown, score: 0.39
Tox4Unknown, score: 0.78
Wdfy2Unknown, score: 0.57
Stk32aUnknown, score: 0.47
Colgalt2Unknown, score: 0.42
Chsy1Unknown, score: 0.54
Prr11Unknown, score: 0.38
5031414D18RikUnknown, score: 0.4
Tmem169Unknown, score: 0.62
Tstd2Unknown, score: 0.53
Irf2bp1Unknown, score: 0.43
Gm694Unknown, score: 0.68
RilpUnknown, score: 0.42
B3gat2Unknown, score: 0.46
Adam1bUnknown, score: 0.35
Hist2h2bbUnknown, score: 0.37
C130050O18RikUnknown, score: 0.41
Tns3Unknown, score: 0.49
Cc2d1bUnknown, score: 0.45
Klri2Unknown, score: 0.41
Tmem215Unknown, score: 0.46
Fam169aUnknown, score: 0.36
OcrlUnknown, score: 0.35
Pptc7Unknown, score: 0.47
Vstm4Unknown, score: 0.36
Amigo3Unknown, score: 0.41
Mtap7d3Unknown, score: 0.37
Ubtd2Unknown, score: 0.48
Ubash3aUnknown, score: 0.37
Nol4lUnknown, score: 0.39
Zfp786Unknown, score: 0.64
B4galnt3Unknown, score: 0.42
Htra4Unknown, score: 0.38
KcpUnknown, score: 0.64
Cngb1Unknown, score: 0.43
Map3k9Unknown, score: 1
Slc2a12Unknown, score: 0.41
Hcfc1r1Unknown, score: 0.35
Bpifb3Unknown, score: 0.45
Tagap1Unknown, score: 0.34
Atxn7l1Unknown, score: 0.68
SlirpUnknown, score: 0.51
Rsl1Unknown, score: 0.71
Pdp1Unknown, score: 0.62
Mup20Unknown, score: 0.56
Tatdn2Unknown, score: 0.43
Apold1Unknown, score: 0.37
Ano8Unknown, score: 0.72
Urb2Unknown, score: 0.51
Pdp2Unknown, score: 0.63
Ccdc33Unknown, score: 0.4
Cd300lhUnknown, score: 0.4
Mtx3Unknown, score: 0.69
Fndc5Unknown, score: 0.75
Zfp174Unknown, score: 0.39
Opa3Unknown, score: 0.44
Iqgap3Unknown, score: 0.38
Panx2Unknown, score: 0.35
Gja6Unknown, score: 0.34
Zfp457Unknown, score: 0.46
Akap7Unknown, score: 0.38
Myo1aUnknown, score: 0.68
Dnaic2Unknown, score: 0.36
432768Unknown, score: 0.45
Syndig1Unknown, score: 0.41
Chchd2Unknown, score: 0.56
Ccdc8Unknown, score: 0.34
Zfp560Unknown, score: 0.4
Gal3st3Unknown, score: 0.66
545423Unknown, score: 0.64
545531Unknown, score: 0.49
545600Unknown, score: 0.45
Ccdc17Unknown, score: 0.45
631287Unknown, score: 0.54
Clec2lUnknown, score: 0.41
668936Unknown, score: 0.64
Rnf213Unknown, score: 0.45
Sirpb1cUnknown, score: 0.52
Gm13305Unknown, score: 0.64
1810037I17RikUnknown, score: 0.53
Plac9aUnknown, score: 0.52
100040961Unknown, score: 0.46
ErmardUnknown, score: 0.62
100044398Unknown, score: 0.45
100044729Unknown, score: 0.39
100045326Unknown, score: 0.41
100046151Unknown, score: 0.41
100046899Unknown, score: 0.39
100048447Unknown, score: 0.36
100048759Unknown, score: 0.62
Gm3194Unknown, score: 0.52
Gm3411Unknown, score: 0.52
101055794Unknown, score: 0.46
101056452Unknown, score: 0.53
101056544Unknown, score: 0.42
101056688Unknown, score: 0.34

## Help | Hide | Top Help | Show | Top Conditions

### HELP

Conditions in the module, given in the same order as on the expression
plot above. Red color means over-expression, green under-expression in
the given condition.

The barplot below shows the condition (sample) scores. A separate bar
is shown for each sample, its height is the corresponding score of the
sample in the module. The red and green numbers on the bars are the
sample scores expressed in percents, i.e. 100% is 1.0.

The red and green lines show the module thresholds, samples above
the red line and below the green line are included in the module.

The different experiments that were part of the study, are separated
by dashed vertical lines.

— Click on the *Help* button again to close this help window.

Id

DBA\_2J-ISO\_8

SWR\_J-ISO\_141

SWR\_J-ISO\_142

SWR\_J-ISO\_136

NOD\_ShiLtJ-ISO\_25

SJL\_J-ISO\_12

NOD\_ShiLtJ-ISO\_24

BALB\_cJ-ISO\_81

FVB\_NJ-ISO\_54

DBA\_2J-ISO\_4

NZB\_BLNJ-ISO\_91

FVB\_NJ-ISO\_55

NOD\_ShiLtJ-ISO\_26

BALB\_cJ-ISO\_83

C3H\_HeJ-ISO\_68

C57BL\_6J-ISO\_50

NZB\_BLNJ-ISO\_96

I\_LnJ-ISO\_149

NZB\_BLNJ-ISO\_90

C3H\_HeJ-ISO\_62

I\_LnJ-ISO\_152

C58\_J-ISO\_33

FVB\_NJ-ISO\_58

SJL\_J-ISO\_13

C3H\_HeJ-ISO\_69

BALB\_cJ-ISO\_84

SM\_J-ISO\_46

SM\_J-ISO\_45

SJL\_J-ISO\_14

C57BLKS\_J-ISO\_115

SM\_J-ISO\_44

LP\_J-ISO\_103

LP\_J-ISO\_102

C58\_J-ISO\_37

C57BL\_6J-ISO\_59

C58\_J-ISO\_31

PL\_J-ISO\_78

I\_LnJ-ISO\_155

PL\_J-ISO\_73

DBA\_2J-ISO\_7

C57BL\_6J-ISO\_47

LP\_J-ISO\_99

BALB\_cByJ-ISO\_159

BALB\_cByJ-ISO\_145

PL\_J-ISO\_77

C57BLKS\_J-ISO\_114

A\_J-ISO\_118

BALB\_cByJ-ISO\_156

SWR\_J-CTR\_137

FVB\_NJ-ATE\_53

BALB\_cByJ-CTR\_144

PL\_J-CTR\_70

FVB\_NJ-ATE\_57

PL\_J-ATE\_71

C57BLKS\_J-CTR\_111

C58\_J-ATE\_36

C57BL\_6J-ATE\_106

C58\_J-ATE\_30

C57BLKS\_J-ATE\_108

LP\_J-ATE\_105

DBA\_2J-ATE\_3

LP\_J-ATE\_101

C58\_J-ATE\_29

C3H\_HeJ-ATE\_66

C58\_J-CTR\_34

NZB\_BLNJ-ATE\_94

PL\_J-ATE\_75

DBA\_2J-ATE\_5

NOD\_ShiLtJ-ATE\_27

PL\_J-ATE\_76

C57BLKS\_J-CTR\_110

C57BL\_6J-ATE\_95

NOD\_ShiLtJ-CTR\_20

C57BL\_6J-ATE\_109

C3H\_HeJ-ATE\_61

FVB\_NJ-ATE\_60

C57BLKS\_J-ATE\_113

NOD\_ShiLtJ-ATE\_22

C57BLKS\_J-ATE\_112

NOD\_ShiLtJ-CTR\_21

DBA\_2J-ATE\_6

BALB\_cJ-ATE\_80

SWR\_J-ATE\_135

BALB\_cByJ-ATE\_160

NOD\_ShiLtJ-CTR\_19

A\_J-CTR\_119

A\_J-CTR\_121

BALB\_cByJ-CTR\_158

CBA\_J-ISO\_129

A\_J-CTR\_120

A\_J-ATE\_122

C3H\_HeJ-ATE\_67

LP\_J-ATE\_98

A\_J-ATE\_117

BALB\_cByJ-ATE\_151

CBA\_J-CTR\_125

A\_J-ATE\_116

LP\_J-CTR\_97

CBA\_J-ATE\_131

CBA\_J-ATE\_132

CBA\_J-ISO\_133

CBA\_J-CTR\_126

CBA\_J-ATE\_127

NOD\_ShiLtJ-ATE\_23

BALB\_cByJ-ATE\_157

CBA\_J-CTR\_130

© 2015 Computational Biology Group, Department of Medical Genetics,
University of Lausanne, Switzerland
