# Supplementary material for: RNAseq analysis of heart tissue from mice treated with atenolol and isoproterenol reveals a reciprocal transcriptional response
Source: BMC Genomics. 2016 Sep 7;17(1):717. doi: 10.1186/s12864-016-3059-6 (PMC5015234; doi:10.1186/s12864-016-3059-6)
Supplement: Additional file 4: — Supplementary data. Compressed HTML files of 98 expression modules annotated for genes, strains and GO or KEGG terms (see Additional file 3 for navigation details). (GZ 11006 kb) [file 12864_2016_3059_MOESM4_ESM.gz › modules.html/module-98.html]

Module #98, TG: 0, TC: 0, 8010 probes, 8010 Entrez genes, 160 conditions

# Previous module | Next module Module #98, TG: 0, TC: 0, 8010 probes, 8010 Entrez genes, 160 conditions

- Module tree/table

- Expression data

- The BP GO tree
- The CC GO tree
- The MF GO tree

- GO BP enrichment
- GO CC enrichment
- GO MF enrichment
- KEGG enrichment
- miRNA enrichment

- Genes
- Conditions

## Help | Hide | Top Help | Show | Top Expression data

### HELP

The image plot shows the color-coded level of gene expression, for the
genes and conditions in a given transcription module. The genes are on
the horizontal, the conditions on the vertical axis.

The genes are ordered according to their ISA gene scores, similarly
the conditions are ordered according to their condition scores. The
score of a gene means the «degree of inclusion» in
the module: a high score gene is essential in the module.

Condition scores can also be negative, that means that the genes of
the module are all down-regulated in the condition. Here the absolute
value of the score gives the «degree of inclusion».

The plots above and beside the expression matrix show the gene scores
and condition scores, respectively.

Note that the plot is interactive, you can see the name of the gene
and condition under the mouse cursor.

The expression matrix was normalized to have mean zero and standard
deviation one for every gene separately across all conditions
(i.e. not just for the conditions in the module).

— Click on the *Help* button again to close this help window.

Gene:   
Condition:


Under-expression is coded with green,
over-expression with red color.

## Help | Hide | Top Help | Show | Top The GO tree — Biological processes

### HELP

This is one of three sections showing Gene Ontology enrichment of the
current module: in this case for **biological processes**.

The graph shows the hierarchy of the GO categories, their enrichment
for the current module is color coded, and the blue number beside the
category is the minus log ten p-value of the enrichment. (Calculated
using the standard hypergeometric test.) The color of the arrows code
«is a» (cyan) and «part of» relationships.

The tree was built the following way. First all GO terms with more
significant enrichment p-value than 0.05 were collected. Then all
paths from these terms to the root node of the GO tree were included
too. If a GO term is included more than once in the tree, then the
green numbers show 1) the id of the node, this makes it easier to find
other appereances of the term, and 2) the number of appearences.

Note that the same GO category might show up on the graph many
times. This is because the GO was «straightened» for this
graph, i.e. if there are more paths from a GO term to the root node of
the tree, all of them are included. The green numbers

Move the mouse cursor over the terms to get their definition. Clicking
on them takes you to the corresponding Gene Ontology web page.

If you cannot see a graph here at all, that means that there were no
significantly enriched GO categories, at the 0.05 level.

— Click on the *Help* button again to close this help window.

## Help | Hide | Top Help | Show | Top The GO tree — Cellular Components

### HELP

This is one of three sections showing Gene Ontology enrichment of the
current module: in this case for **cellular components**.

The graph shows the hierarchy of the GO categories, their enrichment
for the current module is color coded, and the blue number beside the
category is the minus log ten p-value of the enrichment. (Calculated
using the standard hypergeometric test.) The color of the arrows code
«is a» (cyan) and «part of» relationships.

The tree was built the following way. First all GO terms with more
significant enrichment p-value than 0.05 were collected. Then all
paths from these terms to the root node of the GO tree were included
too. If a GO term is included more than once in the tree, then the
green numbers show 1) the id of the node, this makes it easier to find
other appereances of the term, and 2) the number of appearences.

Note that the same GO category might show up on the graph many
times. This is because the GO was «straightened» for this
graph, i.e. if there are more paths from a GO term to the root node of
the tree, all of them are included. The green numbers

Move the mouse cursor over the terms to get their definition. Clicking
on them takes you to the corresponding Gene Ontology web page.

If you cannot see a graph here at all, that means that there were no
significantly enriched GO categories, at the 0.05 level.

— Click on the *Help* button again to close this help window.

## Help | Hide | Top Help | Show | Top The GO tree — Molecular Function

### HELP

This is one of three sections showing Gene Ontology enrichment of the
current module: in this case for **molecular function**.

The graph shows the hierarchy of the GO categories, their enrichment
for the current module is color coded, and the blue number beside the
category is the minus log ten p-value of the enrichment. (Calculated
using the standard hypergeometric test.) The color of the arrows code
«is a» (cyan) and «part of» relationships.

The tree was built the following way. First all GO terms with more
significant enrichment p-value than 0.05 were collected. Then all
paths from these terms to the root node of the GO tree were included
too. If a GO term is included more than once in the tree, then the
green numbers show 1) the id of the node, this makes it easier to find
other appereances of the term, and 2) the number of appearences.

Note that the same GO category might show up on the graph many
times. This is because the GO was «straightened» for this
graph, i.e. if there are more paths from a GO term to the root node of
the tree, all of them are included. The green numbers

Move the mouse cursor over the terms to get their definition. Clicking
on them takes you to the corresponding Gene Ontology web page.

If you cannot see a graph here at all, that means that there were no
significantly enriched GO categories, at the 0.05 level.

— Click on the *Help* button again to close this help window.

## Help | Hide | Top Help | Show | Top GO BP test for over-representation

### HELP

List of all enriched GO categories (biological processes), at the 0.05
p-value level.

The columns:

- **ExpCount** is the expected count of genes in the
  module annotated with the given GO term, just by chance.
- **Count**
  is the number of genes in the module annotated with the given GO
  term.
- **Size** is the total number of genes (in our universe)
  annotated with the GO term.

Clicking on **Count** shows the genes that drive the
enrichment. You can also click on the individual numbers in
the **Count** column, to show the driving genes for that individual
GO category.

Clicking on the GO identifiers takes you to the Gene Ontology web
pages.

— Click on the *Help* button again to close this help window.

No enriched terms

## Help | Hide | Top Help | Show | Top GO CC test for over-representation

### HELP

List of all enriched GO categories (cellular components), at the 0.05
p-value level.

The columns:

- **ExpCount** is the expected count of genes in the
  module annotated with the given GO term, just by chance.
- **Count**
  is the number of genes in the module annotated with the given GO
  term.
- **Size** is the total number of genes (in our universe)
  annotated with the GO term.

Clicking on **Count** shows the genes that drive the
enrichment. You can also click on the individual numbers in
the **Count** column, to show the driving genes for that individual
GO category.

Clicking on the GO identifiers takes you to the Gene Ontology web
pages.

— Click on the *Help* button again to close this help window.

No enriched terms

## Help | Hide | Top Help | Show | Top GO MF test for over-representation

### HELP

List of all enriched GO categories (molecular function), at the 0.05
p-value level.

The columns:

- **ExpCount** is the expected count of genes in the
  module annotated with the given GO term, just by chance.
- **Count**
  is the number of genes in the module annotated with the given GO
  term.
- **Size** is the total number of genes (in our universe)
  annotated with the GO term.

Clicking on **Count** shows the genes that drive the
enrichment. You can also click on the individual numbers in
the **Count** column, to show the driving genes for that individual
GO category.

Clicking on the GO identifiers takes you to the Gene Ontology web
pages.

— Click on the *Help* button again to close this help window.

No enriched terms

## Help | Hide | Top Help | Show | Top KEGG Pathway test for over-representation

### HELP

List of all enriched KEGG pathways, at the 0.05
p-value level.

The columns:

- **ExpCount** is the expected count of genes in the
  module annotated with the given KEGG pathway, just by chance.
- **Count**
  is the number of genes in the module annotated with the given KEGG
  pathway.
- **Size** is the total number of genes (in our universe)
  annotated with the KEGG pathway.

Clicking on **Count** shows the genes that drive the
enrichment. You can also click on the individual numbers in
the **Count** column, to show the driving genes for that individual
KEGG pathway.

Clicking on the KEGG identifiers takes you to the KEGG web site.

— Click on the *Help* button again to close this help window.

No enriched terms


### HELP

List of all enriched miRNA families, at the 0.05
p-value level.

The columns:

- **ExpCount** is the expected count of genes in the
  module regulated by the given miRNA family, just by chance.
- **Count**
  is the number of genes in the module regulated by the given miRNA
  family.
- **Size** is the total number of genes (in our universe)
  regulated with the given miRNA family.

Clicking on **Count** shows the genes that drive the
enrichment. You can also click on the individual numbers in
the **Count** column, to show the driving genes for that individual
miRNA family.

The miRNA regulation data was taken from the

Top


### HELP

p-value level.

The columns:

- **ExpCount** is the expected number of genes in the- **Count**- **Size** is the total number of genes (in our universe)

Clicking on **Count** shows the genes that drive the
enrichment. You can also click on the individual numbers in
the **Count** column, to show the driving genes for that individual

— Click on the *Help* button again to close this help window.

## Help | Hide | Top Help | Show | Top Genes

### HELP

A list of all genes in the current module, in alphabetical order. The
size of the text corresponds to the gene scores.

Note that some gene symbols may show up more than once, if many
probes match the same Entrez gene.

Genes with no Entrez mapping are given separately, with their
Affymetrics probe ID.

— Click on the *Help* button again to close this help window.

### Genes Symbol

, score:

PzpUnknown, score: 0.08
AanatUnknown, score: 0.08
Abca1Unknown, score: 0.22
Abcg1Unknown, score: 0.34
Abl2Unknown, score: 0.35
AcadlUnknown, score: 0.08
Slc33a1Unknown, score: 0.09
AcheUnknown, score: 0.32
Aco2Unknown, score: 0.09
AdipoqUnknown, score: 0.72
Acta1Unknown, score: 0.13
Actg2Unknown, score: -0.01
Actl7aUnknown, score: 0.04
Actn3Unknown, score: 0.23
Acvr1Unknown, score: 0.07
Acvr1bUnknown, score: 0.48
Acvrl1Unknown, score: 0.28
AdaUnknown, score: 0.19
Adam10Unknown, score: 0.2
Adam11Unknown, score: 0.17
Adam17Unknown, score: 0.2
Adam19Unknown, score: 0.32
Adam3Unknown, score: 0.01
Adam4Unknown, score: 0.31
Adam9Unknown, score: 0.01
Adcy6Unknown, score: 0.03
Adcy7Unknown, score: 0.36
Adcy9Unknown, score: 0.16
Add2Unknown, score: 0.31
Plin2Unknown, score: 0.07
Adh1Unknown, score: 0.04
Adh7Unknown, score: 0.3
Adh5Unknown, score: 0.35
AdmUnknown, score: 0.32
Adora2bUnknown, score: 0.24
AdprhUnknown, score: 0
Parp1Unknown, score: 0.23
Adra1bUnknown, score: 0.21
Adra1dUnknown, score: 0.11
Adrb3Unknown, score: -0.01
Adssl1Unknown, score: 0.27
Aebp1Unknown, score: 0.11
AcanUnknown, score: 0.07
AgerUnknown, score: 0.11
AgrpUnknown, score: 0.02
Agtr1aUnknown, score: 0.29
Agtr2Unknown, score: 0.01
AgtrapUnknown, score: 0.3
AhrUnknown, score: 0.31
AhrrUnknown, score: 0.4
AhsgUnknown, score: 0.14
Aif1Unknown, score: 0.12
Ak1Unknown, score: 0.07
Ak4Unknown, score: 0.17
Alas2Unknown, score: 0.34
AlbUnknown, score: 0.03
Abcd1Unknown, score: 0.59
Aldh2Unknown, score: 0.04
Alox8Unknown, score: 0.22
Alx4Unknown, score: 0.38
AmhUnknown, score: 0.1
Ampd3Unknown, score: 0.44
Mat1aUnknown, score: 0.1
Amy1Unknown, score: 0.09
AnkUnknown, score: 0.2
Ankfy1Unknown, score: 0.26
Anxa3Unknown, score: 0.17
Anxa4Unknown, score: 0.35
Anxa5Unknown, score: 0.16
Anxa6Unknown, score: 0.35
Prdx3Unknown, score: 0.08
Prdx6Unknown, score: 0.28
Ap1s1Unknown, score: 0.16
Ap2m1Unknown, score: 0.42
Ap3d1Unknown, score: 0.17
Ap4m1Unknown, score: 0.6
Ap4s1Unknown, score: 0.04
Apaf1Unknown, score: 0.29
Apbb1Unknown, score: 0.02
Apbb2Unknown, score: 0.09
Apex1Unknown, score: 0.21
Atg5Unknown, score: 0.12
Birc3Unknown, score: 0.45
Birc2Unknown, score: 0.13
XiapUnknown, score: 0.12
Api5Unknown, score: 0.13
Aplp1Unknown, score: 0.28
Aplp2Unknown, score: 0.33
Apoa1Unknown, score: 0.48
Apoa2Unknown, score: 0.27
Apoa4Unknown, score: 0.22
Apobec1Unknown, score: 0.11
Apobec2Unknown, score: 0.19
Apoc1Unknown, score: 0.32
Apoc2Unknown, score: 0.28
ApodUnknown, score: 0.05
ApoeUnknown, score: 0.18
ApohUnknown, score: 0.31
Nr2f2Unknown, score: -0.01
Aqp1Unknown, score: 0.02
Aqp4Unknown, score: 0.55
Aqp5Unknown, score: 0.81
Aqp6Unknown, score: 0.33
Aqp7Unknown, score: 0.32
Aqp8Unknown, score: 0.11
AqrUnknown, score: 0.4
Rplp0Unknown, score: 0
Arf3Unknown, score: 0.11
Arf5Unknown, score: 0.29
Arf6Unknown, score: 0.03
Arg2Unknown, score: 0.25
RhoaUnknown, score: 0.03
RhobUnknown, score: 0.13
RhodUnknown, score: 0.36
Arhgap5Unknown, score: 0.18
Arhgap6Unknown, score: 0.06
Phox2aUnknown, score: 0.03
Arl4aUnknown, score: 0.11
Arpc1bUnknown, score: 0.01
ArtnUnknown, score: 0.04
AC084822.3Unknown, score: 0.3
ArxUnknown, score: 0.33
ArsaUnknown, score: 0.14
Asah1Unknown, score: 0.3
Asgr2Unknown, score: 0.04
Rab27aUnknown, score: 0.07
Astn1Unknown, score: 0.28
Serpinc1Unknown, score: 0.24
Atf1Unknown, score: 0.31
Atf3Unknown, score: 0.02
Atf4Unknown, score: 0.01
Atp1b1Unknown, score: 0.2
Atp1b3Unknown, score: 0.15
Fxyd2Unknown, score: 0.13
Atp2a1Unknown, score: 0.01
Atp2a2Unknown, score: 0.2
Atp5bUnknown, score: 0.07
Atp5c1Unknown, score: 0.15
Atp5g1Unknown, score: 0.01
Atp6v0d1Unknown, score: 0.02
Atp6v0eUnknown, score: 0.2
Atp7bUnknown, score: 0.17
Atp10aUnknown, score: 0.44
AtrnUnknown, score: 0.57
Aup1Unknown, score: 0.1
AvpUnknown, score: 0.19
Axin2Unknown, score: 0.31
Cep131Unknown, score: 0.1
B2mUnknown, score: 0.04
Bach1Unknown, score: 0.08
BadUnknown, score: 0.18
Bak1Unknown, score: 0.06
BaxUnknown, score: 0.18
BcanUnknown, score: 0.4
Bcap29Unknown, score: 0.14
Bcl10Unknown, score: 0.65
Bcl2l1Unknown, score: 0.03
Bcl6Unknown, score: 0.12
Bcl7bUnknown, score: 0.21
Bcl7cUnknown, score: 0.04
Opn1swUnknown, score: 0.01
Bdkrb2Unknown, score: 0.58
BdnfUnknown, score: 0.28
Bet1Unknown, score: 0.01
Ngfrap1Unknown, score: 0.08
Glb1Unknown, score: 0.07
BgnUnknown, score: 0.39
BhmtUnknown, score: 0.65
BidUnknown, score: 0.01
Bcl2l11Unknown, score: 0.05
Fabp7Unknown, score: 0.11
Prdm1Unknown, score: 0.35
BlkUnknown, score: 0.21
BlmUnknown, score: 0.03
Cxcr5Unknown, score: 0.21
Bmp1Unknown, score: 0.08
Bmp2Unknown, score: 0.04
Bmp4Unknown, score: 0.15
Bmp7Unknown, score: 0.29
Bmpr1aUnknown, score: -0.01
Bmpr2Unknown, score: 0.49
Bnc1Unknown, score: 0.13
Bnip3Unknown, score: 0.09
Bnip3lUnknown, score: 0.04
Bop1Unknown, score: 0.17
BpgmUnknown, score: 0.22
Brca1Unknown, score: 0.28
Zfp36l2Unknown, score: 0.09
Birc6Unknown, score: 0.56
BsgUnknown, score: 0.05
BsnUnknown, score: 0.34
BtcUnknown, score: 0.03
Btg1Unknown, score: 0.15
Btg2Unknown, score: 0.66
Btg3Unknown, score: 0
BtrcUnknown, score: 0.28
Bub1Unknown, score: 0.18
Bub3Unknown, score: 0.26
C1qbUnknown, score: 0.07
C2Unknown, score: 0.13
CiitaUnknown, score: 0.13
C3Unknown, score: 0.14
C4bUnknown, score: 0.27
C6Unknown, score: 0.29
Cab39Unknown, score: 0.14
Cacna1aUnknown, score: 0.09
Cacna1cUnknown, score: 0.25
Cacna1gUnknown, score: 0.69
Cacna1sUnknown, score: 0.35
Cacna2d3Unknown, score: 0.24
Cacnb3Unknown, score: 0.11
Cacnb4Unknown, score: 0.11
Cacng1Unknown, score: 0.31
CacybpUnknown, score: 0.24
Calm3Unknown, score: 0.16
AspmUnknown, score: 0.29
Car8Unknown, score: 0.3
CaluUnknown, score: -0.01
Camk2aUnknown, score: 0.04
Camk2bUnknown, score: 0.23
Camk2gUnknown, score: 0.09
Camk4Unknown, score: 0.14
CamlUnknown, score: 0.06
CanxUnknown, score: 0.2
Capn1Unknown, score: 0.08
Capn5Unknown, score: 0.32
Capn6Unknown, score: 0.1
Capza1Unknown, score: 0.54
Capza2Unknown, score: 0.19
Car2Unknown, score: -0.01
Car3Unknown, score: 0.41
Car5aUnknown, score: 0.06
Car7Unknown, score: 0.32
Nr1i3Unknown, score: 0.2
CatUnknown, score: 0.07
CaskUnknown, score: 0.46
Casp1Unknown, score: 0.14
Casp4Unknown, score: 0.49
Casp3Unknown, score: 0.01
Casp6Unknown, score: 0.03
Casp7Unknown, score: 0.39
Casp9Unknown, score: 0.13
CasrUnknown, score: 0.15
Ctnnb1Unknown, score: 0.09
Ctnnd1Unknown, score: 0.04
Cav1Unknown, score: 0.25
Cav3Unknown, score: 0.27
Runx1Unknown, score: 0.08
Runx3Unknown, score: 0.47
CbfbUnknown, score: 0.32
Serpinh1Unknown, score: 0.35
Cbr2Unknown, score: 0
CbsUnknown, score: 0.25
Cbx2Unknown, score: 0.23
Cbx5Unknown, score: 0.38
Rb1cc1Unknown, score: 0.11
CckUnknown, score: 0.3
Ccna2Unknown, score: 0.14
Ccnb2Unknown, score: 0.2
Ccnd3Unknown, score: 0
CcnfUnknown, score: 0.35
Ccng1Unknown, score: 0.17
Ccng2Unknown, score: 0.15
CcnkUnknown, score: 0.03
Ccnt1Unknown, score: 0.2
Ccrn4lUnknown, score: 0
CcsUnknown, score: 0.15
Cct2Unknown, score: 0.13
Cct3Unknown, score: 0.19
Cct6aUnknown, score: 0.08
Cct6bUnknown, score: 0.09
Cct7Unknown, score: 0.1
Ctla4Unknown, score: 0.12
Cd19Unknown, score: 0.11
Cd1d2Unknown, score: -0.01
Cd2Unknown, score: 0.11
Cd28Unknown, score: 0.54
Cd33Unknown, score: 0.12
Cd34Unknown, score: 0.37
Cd36Unknown, score: 0.45
Cd37Unknown, score: 0.33
Cd38Unknown, score: 0.04
Entpd2Unknown, score: 0.31
Entpd6Unknown, score: 0.11
Cd3gUnknown, score: 0.04
Cd44Unknown, score: 0.05
Cd5Unknown, score: 0.01
Cd7Unknown, score: 0.37
Cd82Unknown, score: 0.38
Cd83Unknown, score: 0.04
Cd84Unknown, score: 0.28
Cd9Unknown, score: 0.49
Cdc25bUnknown, score: 0.04
Cdc25cUnknown, score: 0.11
Cdk1Unknown, score: 0.48
Cdk11bUnknown, score: 0.12
Cdc37Unknown, score: 0.46
Cdc42Unknown, score: 0.19
Cdc45Unknown, score: 0.26
Cdc7Unknown, score: 0.06
Cdh1Unknown, score: 0
Cdh3Unknown, score: 0.32
Cdh4Unknown, score: 0.02
Cdk5Unknown, score: 0.15
Cdkn1aUnknown, score: 0.33
Cdkn1bUnknown, score: 0.23
Cdkn1cUnknown, score: -0.01
Cdr2Unknown, score: 0.44
MiaUnknown, score: 0.23
CebpaUnknown, score: 0.76
CebpbUnknown, score: 0.19
CenpbUnknown, score: 0.18
Cenpc1Unknown, score: 0.11
Ces1gUnknown, score: 0.17
Cetn3Unknown, score: 0.08
CfiUnknown, score: 0.01
Cfl2Unknown, score: 0.28
ChadUnknown, score: 0.47
Chd1Unknown, score: 0.08
Chek1Unknown, score: 0.43
Chil3Unknown, score: 0.14
Ovgp1Unknown, score: 0.03
ChkaUnknown, score: 0.42
Chl1Unknown, score: 0.69
ChukUnknown, score: 0.22
CideaUnknown, score: 0.19
CidebUnknown, score: 0.08
Elovl3Unknown, score: 0.22
CitUnknown, score: 0
CkmUnknown, score: 0.06
Coro1aUnknown, score: 0.28
Clcn3Unknown, score: 0.08
ClcnkaUnknown, score: 0.17
Cldn1Unknown, score: 0.22
Cldn2Unknown, score: 0.06
Cldn3Unknown, score: 0.11
Cldn5Unknown, score: 0.29
Clk1Unknown, score: 0.16
Tpp1Unknown, score: 0.01
ClockUnknown, score: 0.22
CluUnknown, score: 0.17
CmahUnknown, score: 0.49
Cxcr3Unknown, score: 0.38
Ccr9Unknown, score: 0.23
Ccr1l1Unknown, score: 0.09
Ccr2Unknown, score: 0.29
Ccr4Unknown, score: 0.26
Ackr3Unknown, score: 0.24
Abcc2Unknown, score: 0.35
CnbpUnknown, score: 0.01
Cnga3Unknown, score: 0.16
Cnih2Unknown, score: 0.02
Cnn1Unknown, score: 0.18
Cnn2Unknown, score: 0.05
Cnr1Unknown, score: 0.05
CntfUnknown, score: 0.07
CntfrUnknown, score: 0.37
Cntn1Unknown, score: 0.35
Hps3Unknown, score: 0.06
CoblUnknown, score: -0.01
Col11a1Unknown, score: 0.12
Col12a1Unknown, score: 0.03
Col13a1Unknown, score: 0.11
Col14a1Unknown, score: 0.03
Col3a1Unknown, score: 0.04
Col4a1Unknown, score: 0.1
Col4a2Unknown, score: 0.33
Col4a3Unknown, score: 0.35
Col4a5Unknown, score: 0.03
Col5a1Unknown, score: 0.05
Col5a2Unknown, score: 0.07
Col6a1Unknown, score: 0.09
Col6a2Unknown, score: 0.08
Col7a1Unknown, score: 0.29
Col8a1Unknown, score: 0.07
Col9a3Unknown, score: -0.01
Col1a1Unknown, score: 0
Col1a2Unknown, score: 0.17
ComtUnknown, score: 0.06
CopaUnknown, score: 0.14
Coq7Unknown, score: 0.11
Cox5bUnknown, score: 0
CpUnknown, score: 0.1
CpeUnknown, score: 0.54
Cpeb1Unknown, score: 0.09
Cplx1Unknown, score: 0.15
CpoxUnknown, score: 0.36
Cpt1aUnknown, score: 0
Cpt1bUnknown, score: 0.07
Cpt2Unknown, score: 0.15
Crabp2Unknown, score: 0.04
CratUnknown, score: 0.04
CrcpUnknown, score: 0.32
Creb1Unknown, score: 0.18
Atf6bUnknown, score: 0.17
Crhr2Unknown, score: 0.01
Crmp1Unknown, score: 0.09
Pcdha11Unknown, score: 0.02
CrpUnknown, score: 0.23
Cr1lUnknown, score: 0.01
Hapln1Unknown, score: 0.06
Crybb3Unknown, score: 0.15
Csf1rUnknown, score: 0.03
Csf3rUnknown, score: 0.08
CskUnknown, score: 0.16
Csnk2a1Unknown, score: 0.22
Dnajc5Unknown, score: 0.05
VcanUnknown, score: 0.35
Smc3Unknown, score: 0.23
Ctbp1Unknown, score: 0.02
Ctbp2Unknown, score: 0
CtsbUnknown, score: 0.26
CtsdUnknown, score: 0.11
CtshUnknown, score: 0.23
CtskUnknown, score: 0.04
Cux2Unknown, score: 0.05
CybaUnknown, score: 0.35
CybbUnknown, score: 0.13
Cyp11a1Unknown, score: 0.27
Cyp1a1Unknown, score: 0.34
Cyp21a1Unknown, score: 0.04
Cyp26a1Unknown, score: 0.35
Cyp2a12Unknown, score: 0.29
Cyp2b10Unknown, score: 0.29
Cyp2c29Unknown, score: 0.36
Cyp2c37Unknown, score: 0.32
Cyp2d9Unknown, score: 0.17
Cyp2e1Unknown, score: 0.21
Cyp2f2Unknown, score: 0.38
Cyp2j5Unknown, score: 0.48
Cyp2j6Unknown, score: 0.37
Cyp3a13Unknown, score: 0.02
Cyp46a1Unknown, score: 0.32
Cyp4a10Unknown, score: 0.21
Cyp4b1Unknown, score: 0.13
Cyp51Unknown, score: 0.16
Cyp8b1Unknown, score: 0.22
Dab2Unknown, score: 0.52
Dad1Unknown, score: 0.28
Dag1Unknown, score: 0.04
DgkaUnknown, score: 0.04
Dapk3Unknown, score: 0.06
DbhUnknown, score: 0.19
DbiUnknown, score: 0.08
Dbil5Unknown, score: 0
DbpUnknown, score: 0.2
Dclk1Unknown, score: 0.09
Eci1Unknown, score: 0.17
DckUnknown, score: 0.21
DcnUnknown, score: 0.27
Dscr3Unknown, score: 0.03
Ddb1Unknown, score: 0.13
DdcUnknown, score: 0.07
Asap1Unknown, score: -0.01
DdtUnknown, score: 0.21
Ddx5Unknown, score: 0.09
Dhx9Unknown, score: 0.06
Degs1Unknown, score: 0.17
DffaUnknown, score: 0.08
Dhcr7Unknown, score: 0.24
DffbUnknown, score: 0.02
Dio2Unknown, score: 0.14
Dlg4Unknown, score: 0.13
Dll1Unknown, score: 0.06
Dlx1Unknown, score: 0.11
Dlx4Unknown, score: -0.01
DmwdUnknown, score: 0.31
Dmc1Unknown, score: 0.36
Dmp1Unknown, score: 0.18
Dnah11Unknown, score: 0.49
Dnah8Unknown, score: 0.34
Dync1h1Unknown, score: 0.04
Dync1i1Unknown, score: 0.25
Dnmt1Unknown, score: 0.17
Trdmt1Unknown, score: -0.01
Doc2bUnknown, score: 0.05
Dpep1Unknown, score: 0.4
Dpm2Unknown, score: 0.13
Dpp6Unknown, score: 0.16
Slc26a3Unknown, score: 0.06
Drd1Unknown, score: 0.2
Drd2Unknown, score: 0.23
Arid3aUnknown, score: 0.01
Atn1Unknown, score: 0.03
Dsc2Unknown, score: 0.03
DscamUnknown, score: 0.15
Dsg2Unknown, score: 0.15
Dsg3Unknown, score: 0
EpycUnknown, score: 0.09
DstUnknown, score: 0.17
Adam28Unknown, score: 0.25
DtnbUnknown, score: 0.07
Dvl1Unknown, score: 0.07
E2f1Unknown, score: 0.31
E2f3Unknown, score: 0.39
E4f1Unknown, score: 0.22
Ear2Unknown, score: 0.34
Lefty1Unknown, score: 0.19
Ebf1Unknown, score: 0.39
Ebf2Unknown, score: 0.26
Ecm1Unknown, score: 0.43
Opn3Unknown, score: 0.17
EdarUnknown, score: 0.04
Edn1Unknown, score: 0.27
EdnraUnknown, score: 0.52
EdnrbUnknown, score: 0.13
EedUnknown, score: 0.17
Eef2kUnknown, score: 0.17
Efna1Unknown, score: 0.28
Efnb1Unknown, score: 0.38
EfsUnknown, score: 0.13
EgfUnknown, score: 0.08
Klk1b26Unknown, score: 0.19
Rhbdf1Unknown, score: 0.07
Ehd1Unknown, score: 0.09
EhfUnknown, score: 0.17
Ei24Unknown, score: 0.16
Eif1aUnknown, score: 0.11
Eif2s1Unknown, score: 0.05
Eif2ak3Unknown, score: 0.03
Eif2b4Unknown, score: 0.39
Eif3aUnknown, score: 0.1
Eif4a1Unknown, score: 0.19
Eif4a2Unknown, score: 0.66
Eif4eUnknown, score: 0.07
Eif4ebp2Unknown, score: 0.05
Eif4g2Unknown, score: 0.13
Elf1Unknown, score: 0.1
Elk1Unknown, score: 0.22
Elk3Unknown, score: 0.3
ElnUnknown, score: 0.17
Aimp1Unknown, score: 0.05
EmbUnknown, score: 0.02
Mark2Unknown, score: 0.19
En2Unknown, score: 0.33
Eno1Unknown, score: 0.03
EnpepUnknown, score: 0.16
EomesUnknown, score: 0.02
Epb4.1l1Unknown, score: 0.42
Epb4.1l2Unknown, score: 0.4
Epb4.1l3Unknown, score: 0.26
Epb4.1l4aUnknown, score: 0.02
DmtnUnknown, score: 0.57
StomUnknown, score: 0.26
Epha4Unknown, score: 0.51
Epha5Unknown, score: 0.2
Epha6Unknown, score: 0.22
Epha7Unknown, score: 0.17
Ephb6Unknown, score: 0.02
Ephx1Unknown, score: 0.4
EporUnknown, score: 0.19
Eps15l1Unknown, score: 0.27
Nr2f6Unknown, score: 0.01
Ercc1Unknown, score: 0.04
Ercc2Unknown, score: 0.14
ErfUnknown, score: 0.18
ErgUnknown, score: 0.11
ErhUnknown, score: 0.27
Ces1cUnknown, score: 0.34
EsdUnknown, score: 0.13
Ces1eUnknown, score: 0.25
Ces3bUnknown, score: 0.14
Gnb1lUnknown, score: 0.12
Esr1Unknown, score: 0.06
Fgd6Unknown, score: 0.1
Gm14288Unknown, score: 0.04
Chchd2Unknown, score: 0.22
Celf2Unknown, score: 0.13
Etv1Unknown, score: 0.07
Etv6Unknown, score: 0.29
Mpzl2Unknown, score: 0.41
MecomUnknown, score: 0.05
Evi2aUnknown, score: 0.41
Evi5Unknown, score: 0.54
EvlUnknown, score: 0.07
Wfdc18Unknown, score: -0.01
Ext2Unknown, score: 0.38
Eya4Unknown, score: 0.02
Ezh1Unknown, score: 0.35
Ezh2Unknown, score: 0.02
F10Unknown, score: 0.33
F13bUnknown, score: 0.31
F2Unknown, score: 0.16
F2rUnknown, score: 0.12
F2rl2Unknown, score: -0.01
F3Unknown, score: 0.02
F5Unknown, score: 0.04
F8aUnknown, score: 0.1
F9Unknown, score: -0.01
FaahUnknown, score: 0.07
Fabp3Unknown, score: 0.31
Fabp1Unknown, score: 0.21
Acsl1Unknown, score: 0.17
FaddUnknown, score: 0.09
Ptk2Unknown, score: 0.24
FahUnknown, score: 0.19
FanccUnknown, score: 0.33
FapUnknown, score: -0.01
FasnUnknown, score: 0.12
Foxh1Unknown, score: 0.15
Fbn1Unknown, score: 0.08
Fbn2Unknown, score: 0.48
Fbp2Unknown, score: 0.41
FbrsUnknown, score: 0.01
Fcer1gUnknown, score: 0.03
Fcer2aUnknown, score: 0.01
Fcgr1Unknown, score: 0.16
Fcgr2bUnknown, score: 0.08
Fcgr3Unknown, score: 0.12
FdxrUnknown, score: 0.39
Fem1aUnknown, score: 0.23
Fen1Unknown, score: 0.12
Lgr5Unknown, score: 0.34
Fgd1Unknown, score: 0.58
Fgf10Unknown, score: 0.07
Fgf11Unknown, score: 0.25
Fgf12Unknown, score: 0.2
Fgf13Unknown, score: 0.25
Fgf14Unknown, score: 0.25
Fgf17Unknown, score: 0.2
Fgf2Unknown, score: 0.25
Fgf5Unknown, score: 0.54
Fgf6Unknown, score: 0.74
Fgf7Unknown, score: 0.2
Fgf9Unknown, score: 0.15
Fgfr1Unknown, score: 0.38
FgrUnknown, score: 0.06
Fh1Unknown, score: 0.4
Fhl2Unknown, score: 0.09
Fhl3Unknown, score: 0.3
Fhl4Unknown, score: 0
Il4i1Unknown, score: 0.46
FigfUnknown, score: 0.18
Ppm1gUnknown, score: 0.36
Fjx1Unknown, score: 0.12
Fkbp1aUnknown, score: 0.15
Fkbp1bUnknown, score: 0.24
Fkbp5Unknown, score: -0.01
Fkbp10Unknown, score: 0.42
Foxc2Unknown, score: 0.27
Foxm1Unknown, score: 0.33
Foxf2Unknown, score: 0.08
Lpin1Unknown, score: 0.21
Flot1Unknown, score: 0.12
Flot2Unknown, score: 0
Flt1Unknown, score: 0.28
Flt3lUnknown, score: 0.01
Flt4Unknown, score: 0.07
Fmo3Unknown, score: 0.02
Fmo5Unknown, score: 0.12
FmodUnknown, score: 0.35
Fn1Unknown, score: 0.05
Fnbp1Unknown, score: 0.56
Srgap2Unknown, score: 0.06
FntaUnknown, score: 0.22
Ncs1Unknown, score: 0.43
Frg1Unknown, score: 0.27
Brd2Unknown, score: 0.16
Fstl1Unknown, score: 0.03
Fth1Unknown, score: 0.11
Ftl1Unknown, score: 0.08
AktipUnknown, score: 0.01
Fut2Unknown, score: 0.05
Fut7Unknown, score: 0.37
Dtx1Unknown, score: 0.17
Fzd4Unknown, score: 0.05
Fzd5Unknown, score: 0.2
Fzd6Unknown, score: 0.15
Fzd8Unknown, score: 0.01
Fzd9Unknown, score: 0.05
G0s2Unknown, score: 0.48
Xrcc6Unknown, score: 0.03
GanabUnknown, score: 0.29
Gab1Unknown, score: 0.09
Gab2Unknown, score: 0.05
GabpaUnknown, score: 0.08
Gabpb1Unknown, score: 0.24
Gabra1Unknown, score: 0.72
Gabra3Unknown, score: 0.47
Gabrb2Unknown, score: 0.1
GabrdUnknown, score: 0.28
Gabrg3Unknown, score: 0.27
Gabrr2Unknown, score: 0.02
Gad2Unknown, score: 0.13
B4galnt2Unknown, score: 0.32
Galnt3Unknown, score: 0.18
GamtUnknown, score: -0.01
Gas1Unknown, score: 0.16
Gas6Unknown, score: 0.12
Gata3Unknown, score: 0.09
Gata4Unknown, score: 0.03
GbaUnknown, score: -0.01
GbasUnknown, score: 0.31
Gbx2Unknown, score: 0.32
GcUnknown, score: 0.19
Usp15Unknown, score: 0.28
MtpnUnknown, score: 0.19
Gch1Unknown, score: 0.48
Gcnt1Unknown, score: 0.03
Gcnt2Unknown, score: 0.18
GdaUnknown, score: 0.1
Gdap1Unknown, score: 0.2
Gdap2Unknown, score: 0.28
Mrps33Unknown, score: 0.01
Gpd1Unknown, score: 0.16
Gdf10Unknown, score: 0.06
Gdf3Unknown, score: 0.09
Gdi1Unknown, score: 0.34
ArhgdigUnknown, score: 0.23
Gpd2Unknown, score: 0.16
GemUnknown, score: 0.08
GfapUnknown, score: 0.08
Gfra1Unknown, score: 0.3
Gfra4Unknown, score: 0.19
GghUnknown, score: 0.29
Ggps1Unknown, score: 0.35
B4galt1Unknown, score: -0.01
Gpr83Unknown, score: 0.01
Gja4Unknown, score: 0.05
Gja5Unknown, score: 0.06
Gjc1Unknown, score: 0.34
Gjb1Unknown, score: 0.11
Gjb2Unknown, score: 0
Gjb5Unknown, score: 0.4
Ostm1Unknown, score: 0.19
GclcUnknown, score: 0.31
GclmUnknown, score: 0.11
Gli2Unknown, score: 0.28
Galk1Unknown, score: 0.47
GlulUnknown, score: 0.19
HaghUnknown, score: 0.45
GlrbUnknown, score: 0.32
GlsUnknown, score: 0.22
Slc6a9Unknown, score: 0.24
Gm2aUnknown, score: 0.46
Gna14Unknown, score: 0.01
Gna15Unknown, score: 0.21
Gnai2Unknown, score: 0.23
GnalUnknown, score: 0.14
Gnao1Unknown, score: 0.13
GnazUnknown, score: 0.11
Gnb2l1Unknown, score: 0.34
Gnb3Unknown, score: 0.22
Gnb5Unknown, score: 0.04
Gng10Unknown, score: 0.14
Gng12Unknown, score: 0.35
Bscl2Unknown, score: 0.11
Gng8Unknown, score: 0.2
GnmtUnknown, score: 0.04
Got2Unknown, score: 0.35
Gp1bbUnknown, score: 0.1
Lrp2Unknown, score: 0.57
PdpnUnknown, score: 0.07
Gpaa1Unknown, score: 0.13
GpamUnknown, score: 0.06
Gpc3Unknown, score: 0.15
Gpc4Unknown, score: 0.09
S1pr2Unknown, score: 0.02
Lpar1Unknown, score: 0.02
Cmklr1Unknown, score: 0.17
PigqUnknown, score: -0.01
Gpld1Unknown, score: 0.4
Gpr27Unknown, score: 0.21
Adgrg1Unknown, score: 0.09
Nmur1Unknown, score: 0.04
Lancl1Unknown, score: 0.05
Grk4Unknown, score: 0.04
Gpx3Unknown, score: 0.05
GsrUnknown, score: 0
Grb10Unknown, score: 0.01
Grb2Unknown, score: 0.05
Rhpn1Unknown, score: 0.05
Emg1Unknown, score: 0.21
AesUnknown, score: -0.01
Gria1Unknown, score: 0.38
Gria4Unknown, score: 0.14
Grid1Unknown, score: 0.24
Grik2Unknown, score: 0.13
Grin2cUnknown, score: 0.02
Grin2dUnknown, score: 0.26
Cxcl1Unknown, score: 0.25
Pdia3Unknown, score: 0.18
Hspa5Unknown, score: 0.29
Gsg2Unknown, score: 0.11
Gspt1Unknown, score: 0.19
Gsta1Unknown, score: 0.06
Gsta2Unknown, score: 0.29
Gsta3Unknown, score: 0.33
Gstm3Unknown, score: 0.25
Gstm4Unknown, score: 0.02
Gstm5Unknown, score: 0.37
Gstm6Unknown, score: 0.35
Gstp1Unknown, score: 0.02
Gsto1Unknown, score: 0.25
Gstz1Unknown, score: 0.31
Cfap20Unknown, score: 0.07
Gtpbp1Unknown, score: 0.42
Nkx6-2Unknown, score: 0.2
Guca1aUnknown, score: 0.08
Guca2bUnknown, score: 0.02
Gucy2dUnknown, score: 0
Guk1Unknown, score: 0.07
Magi1Unknown, score: 0.04
Gys1Unknown, score: 0.49
GzmaUnknown, score: 0.04
GzmbUnknown, score: 0.01
Hist1h1dUnknown, score: 0.22
CfbUnknown, score: 0.01
H2-D1Unknown, score: 0.43
H2-K1Unknown, score: 0.35
H2-DMaUnknown, score: 0.19
H2-DMb1Unknown, score: 0.12
H2-DMb2Unknown, score: 0
H2-OaUnknown, score: 0.06
H2-ObUnknown, score: 0.1
H2-Q10Unknown, score: 0.1
H2-Q2Unknown, score: 0.17
H2-T3Unknown, score: 0.61
H2-T22Unknown, score: 0.58
H2-T23Unknown, score: 0.05
H2-T3Unknown, score: 0.61
H2-T22Unknown, score: 0.26
15061Unknown, score: 0.47
Mr1Unknown, score: 0.34
H3f3aUnknown, score: 0.28
HadhUnknown, score: 0.06
Hand2Unknown, score: 0.25
Hao1Unknown, score: 0.03
Has2Unknown, score: 0.24
Has3Unknown, score: 0.1
Hbb-bsUnknown, score: 0.28
HcUnknown, score: 0.05
HccsUnknown, score: 0.21
HckUnknown, score: 0.12
Hcls1Unknown, score: 0.01
Hcn1Unknown, score: -0.01
Hcn3Unknown, score: 0.14
Ptpn6Unknown, score: 0.3
Hdac2Unknown, score: 0.08
HdcUnknown, score: 0.36
HttUnknown, score: 0.02
Hebp1Unknown, score: 0.1
HbegfUnknown, score: 0.18
HellsUnknown, score: 0
Herc2Unknown, score: 0.17
Hes1Unknown, score: 0.01
Hes5Unknown, score: 0.3
Hey1Unknown, score: 0.01
HfeUnknown, score: 0.01
Foxd3Unknown, score: 0.08
Foxf1Unknown, score: 0.16
HgdUnknown, score: 0.01
HgfUnknown, score: 0.19
HgsUnknown, score: 0.34
Hic1Unknown, score: 0.42
Hk2Unknown, score: 0.27
HmbsUnknown, score: 0.05
Hmg20bUnknown, score: 0
HmgclUnknown, score: 0.08
HmgcrUnknown, score: 0.45
Hmga2Unknown, score: 0.19
Hmox1Unknown, score: 0.07
Hn1Unknown, score: 0.35
Hnf4aUnknown, score: 0.17
Onecut1Unknown, score: 0.83
HnrnpcUnknown, score: 0.03
Hoxa1Unknown, score: 0.21
Hoxa4Unknown, score: 0.08
Hoxa6Unknown, score: 0.47
Hoxa7Unknown, score: 0.17
Hoxb6Unknown, score: 0.11
Hoxd4Unknown, score: 0.06
HpUnknown, score: 0.01
Hp1bp3Unknown, score: 0.07
HpcaUnknown, score: 0.21
HpdUnknown, score: 0.45
HpgdUnknown, score: 0.06
LipcUnknown, score: 0.23
HpnUnknown, score: 0.72
HprtUnknown, score: 0.14
HrasUnknown, score: 0.09
Agfg1Unknown, score: 0.19
Hrh2Unknown, score: 0.28
Prmt2Unknown, score: 0.01
Hs3st1Unknown, score: 0.26
Hs3st3a1Unknown, score: 0.2
Hspa8Unknown, score: 0.04
Hspa1lUnknown, score: 0.03
Hsd11b2Unknown, score: 0
Hsd17b1Unknown, score: 0.28
Hsd17b2Unknown, score: 0.27
Hsd17b4Unknown, score: 0.78
Hsd17b7Unknown, score: 0.17
Hsd3b2Unknown, score: 0.54
Hsd3b3Unknown, score: 0.36
Hsf1Unknown, score: 0.11
Hsf2Unknown, score: 0.28
Dnaja1Unknown, score: 0.09
Dnajb3Unknown, score: 0.31
Hsph1Unknown, score: 0.11
Hspb1Unknown, score: 0.13
Hspa1bUnknown, score: -0.01
Hspa2Unknown, score: 0.08
Hsp90ab1Unknown, score: 0.36
Hsp90aa1Unknown, score: 0.14
Hspa4Unknown, score: 0.14
Hspe1Unknown, score: 0.17
Trmt2aUnknown, score: -0.01
Htr2bUnknown, score: -0.01
Slc6a4Unknown, score: 0.13
Elavl3Unknown, score: -0.01
Hyal2Unknown, score: 0.1
Id4Unknown, score: 0.02
IdeUnknown, score: 0.05
Ido1Unknown, score: 0.1
Ier3Unknown, score: 0.04
Ier5Unknown, score: 0.12
Irgm1Unknown, score: 0.01
Cxcl10Unknown, score: 0.36
Ifi203Unknown, score: 0.28
Ifi204Unknown, score: 0.2
Ifi47Unknown, score: 0.15
Ifit1Unknown, score: 0.45
Ifit2Unknown, score: 0.06
Ifit3Unknown, score: 0.04
Ifnar2Unknown, score: 0.08
Ifngr1Unknown, score: 0.13
Ifrd1Unknown, score: 0
Ifrd2Unknown, score: 0.05
Cd79bUnknown, score: 0.16
IgfalsUnknown, score: 0.29
Cyr61Unknown, score: 0.19
Igfbp2Unknown, score: 0.37
Igfbp4Unknown, score: 0
Il18bpUnknown, score: 0.14
IhhUnknown, score: 0.1
IkbkbUnknown, score: 0.02
Il10raUnknown, score: 0.09
Il10rbUnknown, score: 0.11
Il12rb1Unknown, score: 0.1
Il12rb2Unknown, score: 0.26
Il15Unknown, score: 0.05
Il15raUnknown, score: 0.28
Il17raUnknown, score: 0
Il1aUnknown, score: 0.01
Il1bUnknown, score: 0.31
Irak1Unknown, score: 0.13
Il1rnUnknown, score: 0.28
Il18r1Unknown, score: 0.33
Il2rbUnknown, score: 0.1
Il6Unknown, score: 0.04
Il6stUnknown, score: -0.01
Ilf3Unknown, score: 0.17
Lrig1Unknown, score: 0.16
ImpactUnknown, score: 0.12
Kpnb1Unknown, score: 0.33
InhbbUnknown, score: 0.1
Cep250Unknown, score: 0.04
Insl3Unknown, score: 0.24
InsrUnknown, score: 0.37
Irf1Unknown, score: 0.13
Irf2Unknown, score: 0.25
Irx2Unknown, score: 0.18
Irf9Unknown, score: 0.13
Itga3Unknown, score: 0.27
Itga5Unknown, score: 0.66
ItgamUnknown, score: 0.29
ItgaxUnknown, score: 0.18
Itgb1Unknown, score: 0
Itgb5Unknown, score: 0.17
Cd47Unknown, score: 0.25
Itih1Unknown, score: 0.17
Itih2Unknown, score: 0.29
Itih3Unknown, score: 0.02
ItkUnknown, score: 0.21
Stt3aUnknown, score: 0.25
Itm2aUnknown, score: 0.11
Itm2bUnknown, score: 0.11
ItpaUnknown, score: 0.02
Itpr1Unknown, score: 0.08
Jag2Unknown, score: 0.26
Jak1Unknown, score: 0.14
F11rUnknown, score: 0.33
JrkUnknown, score: 0.2
JupUnknown, score: 0.26
Kcna1Unknown, score: 0.07
Kcna3Unknown, score: 0.32
Kcna4Unknown, score: 0.05
Kcna5Unknown, score: 0.35
Kcna6Unknown, score: 0.23
Kcnab2Unknown, score: 0.09
Kcnd1Unknown, score: 0.25
Kcnd2Unknown, score: 0.33
Kcnj12Unknown, score: 0.37
Kcnj15Unknown, score: 0.08
Kcnj2Unknown, score: 0.17
Kcnj5Unknown, score: 0.23
Kcnj6Unknown, score: 0.1
Kcnj8Unknown, score: 0.27
Kcnk1Unknown, score: 0.44
Kcnk4Unknown, score: 0.1
Kcnk7Unknown, score: 0.02
Kcnma1Unknown, score: 0.14
Kcnu1Unknown, score: 0.17
NapsaUnknown, score: 0.1
KdrUnknown, score: 0.4
KhsrpUnknown, score: 0.08
Kif13bUnknown, score: 0.09
Kif1cUnknown, score: 0.32
Kif2aUnknown, score: 0.2
Kif21bUnknown, score: 0.64
Kif3bUnknown, score: 0.05
Kif3cUnknown, score: 0.16
Kif4Unknown, score: 0.29
Kif5aUnknown, score: 0.07
Kif5bUnknown, score: 0.22
Kif9Unknown, score: 0.2
Kifc3Unknown, score: 0.39
KinUnknown, score: 0.32
Uhmk1Unknown, score: 0.4
Klf1Unknown, score: 0.11
Klf2Unknown, score: 0.17
Klk1b26Unknown, score: 0.19
Klkb1Unknown, score: 0.15
Klra4Unknown, score: 0.04
CU424478.2Unknown, score: 0.12
Klrc2Unknown, score: 0.16
Klrd1Unknown, score: 0.36
Kng1Unknown, score: 0.05
Kpna2Unknown, score: 0.22
Kpna3Unknown, score: 0.26
Kpna6Unknown, score: 0.29
SspnUnknown, score: 0.13
Krt10Unknown, score: 0.12
Krt19Unknown, score: 0.05
Ksr1Unknown, score: 0.37
Ktn1Unknown, score: 0.03
KyUnknown, score: 0.29
Lag3Unknown, score: 0.18
Dsg4Unknown, score: 0.04
Lama3Unknown, score: 0.07
Lama5Unknown, score: 0.17
Lamp1Unknown, score: 0.34
RpsaUnknown, score: 0.01
AnpepUnknown, score: -0.01
Laptm5Unknown, score: 0.04
LargeUnknown, score: 0.01
LatUnknown, score: 0.24
LbpUnknown, score: 0.44
Lbx2Unknown, score: 0.04
Ldb1Unknown, score: 0.2
LdhaUnknown, score: 0.1
LdlrUnknown, score: 0.11
Lect1Unknown, score: 0.21
LepUnknown, score: 0.05
LfngUnknown, score: 0
Lgals3Unknown, score: 0.36
Eif2dUnknown, score: 0.05
LhcgrUnknown, score: 0.01
Lhx2Unknown, score: 0.19
LifrUnknown, score: 0.18
Limk1Unknown, score: 0.04
LipeUnknown, score: 0.09
LmnaUnknown, score: 0.29
Lmnb2Unknown, score: 0.66
Lmo2Unknown, score: 0.28
Lmo4Unknown, score: 0.05
Lmx1bUnknown, score: 0.07
PhyhUnknown, score: 0.15
Sh2b3Unknown, score: 0.22
Loxl1Unknown, score: 0.05
Loxl3Unknown, score: 0.2
Anxa1Unknown, score: 0.25
LplUnknown, score: 0.1
Lrp5Unknown, score: 0.08
Lrp8Unknown, score: 0.07
Lrrc23Unknown, score: 0.54
Lrrfip1Unknown, score: 0.17
Lrrn2Unknown, score: 0.21
Lst1Unknown, score: 0.05
LtkUnknown, score: 0.13
LumUnknown, score: 0.05
AladUnknown, score: 0.18
LxnUnknown, score: 0.07
BlnkUnknown, score: 0.01
Muc13Unknown, score: 0.19
Cd180Unknown, score: 0.37
Tmed1Unknown, score: 0.31
Ly9Unknown, score: 0.16
Ncr1Unknown, score: 0.25
Ly96Unknown, score: 0.76
Lyl1Unknown, score: 0.12
LystUnknown, score: 0.07
Lyz2Unknown, score: 0.11
Tm4sf1Unknown, score: 0.18
M6prUnknown, score: 0.05
AmacrUnknown, score: 0.38
Madcam1Unknown, score: 0.17
Smad1Unknown, score: 0.19
Smad4Unknown, score: 0.04
Smad7Unknown, score: 0.63
MafUnknown, score: -0.01
MafkUnknown, score: 0.06
Mfap2Unknown, score: 0.26
Man1aUnknown, score: 0.03
Man1a2Unknown, score: 0.4
Man2b2Unknown, score: 0.42
Mas1Unknown, score: 0.05
Masp2Unknown, score: 0.14
MatkUnknown, score: 0.31
Matn2Unknown, score: 0.02
Matn4Unknown, score: 0.44
Matr3Unknown, score: 0.02
MaxUnknown, score: 0.02
MazUnknown, score: 0.19
MbUnknown, score: 0.23
Mbd1Unknown, score: 0.16
Mcl1Unknown, score: 0.31
Mcm3Unknown, score: 0.15
Mcm2Unknown, score: 0.01
Mcm4Unknown, score: 0.02
Mcm7Unknown, score: -0.01
Anapc1Unknown, score: 0.16
Mcpt2Unknown, score: 0.24
Mcpt4Unknown, score: 0.1
Cma1Unknown, score: 0.03
Mgrn1Unknown, score: 0.09
MdfiUnknown, score: 0.25
MdkUnknown, score: 0.09
Mdm1Unknown, score: 0.05
Abcc1Unknown, score: 0.1
Mea1Unknown, score: 0.1
Mecp2Unknown, score: 0.02
Mef2bUnknown, score: 0.25
Mef2dUnknown, score: 0.29
Meis1Unknown, score: 0.26
Rab8aUnknown, score: 0.16
MelkUnknown, score: 0.02
Men1Unknown, score: 0.09
Meox1Unknown, score: 0.17
MertkUnknown, score: 0.48
MestUnknown, score: 0.39
MetUnknown, score: 0.08
Mgat3Unknown, score: 0.16
MgpUnknown, score: 0.18
MifUnknown, score: 0
MipUnknown, score: 0.06
Bhlha15Unknown, score: 0.33
MitfUnknown, score: 0.26
Pias2Unknown, score: 0.36
Mki67Unknown, score: 0.05
Mlf1Unknown, score: 0.08
Aff1Unknown, score: 0.32
Marcksl1Unknown, score: 0.29
Trpm1Unknown, score: 0.38
Mmp11Unknown, score: 0.03
Mmp15Unknown, score: 0.29
Mmp2Unknown, score: 0.16
Mmp24Unknown, score: 0.49
Mmp9Unknown, score: 0.3
Foxk1Unknown, score: 0.54
Mocs2Unknown, score: 0.43
Mov10Unknown, score: 0.21
Psmd7Unknown, score: 0.19
Cd200Unknown, score: 0.13
Clec4dUnknown, score: 0.16
MplUnknown, score: 0.16
Mpp1Unknown, score: 0.06
Gm29609Unknown, score: 0.36
MpzUnknown, score: 0.11
MrasUnknown, score: 0.06
Mre11aUnknown, score: 0.12
Meis3Unknown, score: 0.1
Cited2Unknown, score: 0.07
Msh2Unknown, score: 0.14
Msh3Unknown, score: 0.09
Msh5Unknown, score: 0.08
Msh6Unknown, score: 0.02
Msl3Unknown, score: 0.3
Msx1Unknown, score: 0
Msx2Unknown, score: 0.56
mt-Atp6Unknown, score: 0.46
mt-Atp8Unknown, score: 0.29
mt-Co1Unknown, score: 0.23
mt-Co2Unknown, score: 0.32
mt-Co3Unknown, score: 0.3
Grpel1Unknown, score: 0.08
Grpel2Unknown, score: 0.39
mt-Nd1Unknown, score: 0.24
mt-Nd2Unknown, score: 0.39
mt-Nd3Unknown, score: 0.35
mt-Nd4Unknown, score: 0.47
mt-Nd5Unknown, score: 0.6
Mt2Unknown, score: 0.13
Map1aUnknown, score: 0.16
Map1bUnknown, score: 0.36
Map4Unknown, score: 0.13
Map6Unknown, score: 0.28
Map7Unknown, score: 0.16
MaptUnknown, score: 0.29
Mtf1Unknown, score: 0.08
MthfrUnknown, score: 0.33
Mtl5Unknown, score: 0.14
Mast2Unknown, score: 0.66
Bloc1s5Unknown, score: 0.02
17831Unknown, score: 0.44
Mug1Unknown, score: 0.39
Mup1Unknown, score: 0.41
Mup3Unknown, score: 0.1
Commd1Unknown, score: 0.15
Usp34Unknown, score: 0.12
MybUnknown, score: 0.58
Mybpc3Unknown, score: 0.11
Myd88Unknown, score: 0.05
Myef2Unknown, score: 0.15
Myh11Unknown, score: 0.09
Myh3Unknown, score: 0.18
Myh8Unknown, score: 0.03
Myh9Unknown, score: 0.06
Myh6Unknown, score: 0.12
Myl4Unknown, score: 0.04
Myl7Unknown, score: 0.4
Myl6Unknown, score: 0.25
Myl2Unknown, score: 0.02
Myo15Unknown, score: 0.1
Myo1fUnknown, score: 0.3
Myo5aUnknown, score: 0.45
Myo5bUnknown, score: 0.23
Myo7bUnknown, score: 0.15
MyocUnknown, score: -0.01
Myod1Unknown, score: 0.26
Myom1Unknown, score: 0.33
Myom2Unknown, score: 0.36
Ppp1r12aUnknown, score: 0.16
Nab1Unknown, score: 0.1
Naip2Unknown, score: 0
Naip6Unknown, score: 0.17
Nap1l2Unknown, score: 0.01
Nap1l4Unknown, score: 0.37
NapbUnknown, score: 0.11
Nat1Unknown, score: 0.28
Nbl1Unknown, score: 0.13
Nbr1Unknown, score: 0.06
Ncam1Unknown, score: 0.03
Ncam2Unknown, score: 0.01
Ncf1Unknown, score: 0.26
Ncf4Unknown, score: 0.18
Nck2Unknown, score: 0.33
Nedd1Unknown, score: 0.12
Nedd4Unknown, score: 0.2
Nedd9Unknown, score: 0.28
Nek1Unknown, score: 0.22
Neo1Unknown, score: 0.07
Neurl1aUnknown, score: 0.06
Nf1Unknown, score: 0.15
Nfe2l1Unknown, score: 0.09
Nfe2l2Unknown, score: 0.25
Nfe2l3Unknown, score: 0.22
NfiaUnknown, score: 0.29
NfkbibUnknown, score: 0.04
NeflUnknown, score: 0.07
NfyaUnknown, score: 0.18
NfycUnknown, score: 0.12
NgpUnknown, score: 0.16
Nid2Unknown, score: 0.42
Ninj1Unknown, score: 0.18
Nipsnap1Unknown, score: 0.04
Nkx2-5Unknown, score: 0.13
NmbrUnknown, score: -0.01
Cd244Unknown, score: 0.11
Nmt1Unknown, score: 0.31
MycnUnknown, score: 0.08
NnatUnknown, score: 0.02
NnmtUnknown, score: 0.06
Emc8Unknown, score: 0.15
NodalUnknown, score: 0.04
Mrpl49Unknown, score: 0.08
Nr4a3Unknown, score: 0.06
Nos1Unknown, score: 0.19
Notch2Unknown, score: 0.3
Notch3Unknown, score: 0.18
NovUnknown, score: 0.3
ZfmlUnknown, score: 0.03
Npas1Unknown, score: 0.04
Npdc1Unknown, score: 0.39
Npm3Unknown, score: 0.62
Npr1Unknown, score: 0.34
Npr3Unknown, score: 0.02
Npy1rUnknown, score: 0.19
NrapUnknown, score: 0.3
Nrf1Unknown, score: 0.2
Nrp1Unknown, score: 0.23
NrtnUnknown, score: 0.21
Nrxn2Unknown, score: 0.11
Nsd1Unknown, score: 0.21
Nsg2Unknown, score: 0.07
MuskUnknown, score: 0.04
NsmafUnknown, score: 0.38
Ntan1Unknown, score: 0.18
Ntn3Unknown, score: 0
Dusp8Unknown, score: 0.03
NudcUnknown, score: 0.28
Nr4a2Unknown, score: 0.02
NxnUnknown, score: 0.61
Nxph1Unknown, score: 0.17
Oaz1Unknown, score: 0.02
OclnUnknown, score: 0.39
Odc1Unknown, score: 0.01
Ogg1Unknown, score: 0.47
Olfr18Unknown, score: 0.03
Olfr33Unknown, score: 0.06
Oprd1Unknown, score: 0.17
Sigmar1Unknown, score: 0
Orc1Unknown, score: 0.07
Slc22a18Unknown, score: 0.21
Orm3Unknown, score: 0.08
Slc25a15Unknown, score: 0.02
OsmUnknown, score: 0.2
OsmrUnknown, score: 0.21
Hspa4lUnknown, score: 0.28
Cldn11Unknown, score: 0.01
OtogUnknown, score: 0.23
Ovol1Unknown, score: 0.15
P2rx6Unknown, score: -0.01
P2ry2Unknown, score: 0.21
P4ha2Unknown, score: 0.27
P4hbUnknown, score: 0.37
Bloc1s6Unknown, score: 0.43
Pafah1b1Unknown, score: 0.14
Pafah1b3Unknown, score: 0.01
Prdx1Unknown, score: 0.34
PamUnknown, score: 0.08
Cntn3Unknown, score: 0.04
PappaUnknown, score: 0.08
Pax3Unknown, score: 0.15
Pax5Unknown, score: 0.24
Pbx2Unknown, score: 0.17
Pbx3Unknown, score: 0.52
Igbp1Unknown, score: 0.09
Pcbp2Unknown, score: 0.29
Pck1Unknown, score: 0.3
Pcmt1Unknown, score: 0.05
PcntUnknown, score: 0.17
PcolceUnknown, score: 0.05
Pcp2Unknown, score: 0.37
Cdk16Unknown, score: 0.03
Cdk18Unknown, score: 0.02
Pdcd11Unknown, score: 0.05
Pde1aUnknown, score: 0.02
Pde1bUnknown, score: 0.08
Pde4bUnknown, score: 0.14
Pde6dUnknown, score: 0.4
Pde7aUnknown, score: 0.12
Pde8aUnknown, score: 0.28
Pde9aUnknown, score: 0.03
Pde6bUnknown, score: 0.29
PdgfbUnknown, score: 0.04
PdgfrbUnknown, score: 0.03
Padi2Unknown, score: 0.06
Padi3Unknown, score: 0.24
Pdk2Unknown, score: 0.17
Pdpk1Unknown, score: 0.19
Pea15aUnknown, score: 0.23
Etv4Unknown, score: 0.05
PemtUnknown, score: 0.03
Per3Unknown, score: 0.37
Pex11aUnknown, score: 0.05
CfpUnknown, score: 0.31
Pfdn2Unknown, score: 0.18
Pfkfb1Unknown, score: 0.14
PfkmUnknown, score: 0.21
Pfn1Unknown, score: 0.12
Pfn2Unknown, score: 0.13
Prf1Unknown, score: 0.19
Slc25a3Unknown, score: 0.17
Phkg1Unknown, score: 0.26
Pick1Unknown, score: 0.02
PigaUnknown, score: 0.12
PigfUnknown, score: 0.55
PigrUnknown, score: 0.22
Pik3c2aUnknown, score: 0.11
Pik3c2gUnknown, score: 0.19
Pip5k1cUnknown, score: 0.27
PirbUnknown, score: 0.01
Pou1f1Unknown, score: 0.16
Pitpnm1Unknown, score: 0.1
PkmUnknown, score: -0.01
PrkacaUnknown, score: 0.29
PrkacbUnknown, score: 0.3
PrkcaUnknown, score: 0.01
PrkcdUnknown, score: 0.09
PrkceUnknown, score: 0.17
PrkchUnknown, score: 0.38
PrkciUnknown, score: 0.13
Prkd1Unknown, score: 0.48
Pkd1Unknown, score: 0.12
PkdrejUnknown, score: 0.22
PkiaUnknown, score: 0.64
PkigUnknown, score: 0.27
Pknox1Unknown, score: 0.24
Lypla1Unknown, score: 0.24
Pla2g2cUnknown, score: 0.01
Pla2g2dUnknown, score: 0.22
Pla2g5Unknown, score: 0.17
PlaaUnknown, score: 0.29
Serpinb2Unknown, score: 0.15
PapolaUnknown, score: 0.08
PlatUnknown, score: 0.32
Plcb3Unknown, score: 0.45
Plcd1Unknown, score: 0.07
Plcg1Unknown, score: 0.08
PlecUnknown, score: 0.41
Pa2g4Unknown, score: 0.34
PlgUnknown, score: 0.29
Plk1Unknown, score: 0.06
PlnUnknown, score: 0.01
Plod1Unknown, score: 0.63
Plp1Unknown, score: 0.09
Plp2Unknown, score: 0.18
Lcp1Unknown, score: 0.31
Plscr2Unknown, score: 0.02
Plxna1Unknown, score: 0.12
Pms2Unknown, score: 0.16
Prrx1Unknown, score: 0.28
Phox2bUnknown, score: 0.13
Ppp1r14bUnknown, score: 0.31
PnmtUnknown, score: 0.58
PnpUnknown, score: 0.03
Sept4Unknown, score: 0.01
Pola1Unknown, score: 0.06
Pola2Unknown, score: 0.15
Pold1Unknown, score: -0.01
Pold2Unknown, score: 0.35
PoleUnknown, score: 0.1
PomcUnknown, score: -0.01
Pon1Unknown, score: 0.69
Cnot7Unknown, score: 0.19
PorUnknown, score: 0.1
Pou2f1Unknown, score: 0.16
Pou2f2Unknown, score: 0
Pou6f1Unknown, score: 0.38
PpardUnknown, score: 0.39
PpargUnknown, score: 0.13
Ppef2Unknown, score: 0.35
CtsaUnknown, score: 0.2
SyplUnknown, score: 0.02
PpibUnknown, score: 0.09
PplUnknown, score: 0.11
Ppp1r1bUnknown, score: 0.16
Ppp2caUnknown, score: 0.31
Ppp2cbUnknown, score: 0.39
Ppp3caUnknown, score: 0.16
Ppp3cbUnknown, score: 0.34
Ppp3ccUnknown, score: 0.07
Inpp5kUnknown, score: 0.36
Ppt1Unknown, score: 0.7
Npy4rUnknown, score: 0.27
Nup88Unknown, score: 0.27
Mob4Unknown, score: 0.11
PrepUnknown, score: 0.23
SrgnUnknown, score: 0.19
Prim1Unknown, score: 0.12
Prim2Unknown, score: -0.01
Prkar2bUnknown, score: 0.07
PrkcshUnknown, score: 0.07
Prkg1Unknown, score: 0.01
Prkg2Unknown, score: 0.25
Eif2ak2Unknown, score: 0.42
PrkxUnknown, score: 0.46
PrlrUnknown, score: 0.02
PrnpUnknown, score: 0.3
ProcUnknown, score: 0
Prox1Unknown, score: 0.06
PrphUnknown, score: 0.65
Prpf4bUnknown, score: 0.04
LgmnUnknown, score: 0.01
St14Unknown, score: 0.14
Cyth2Unknown, score: 0.05
Cyth3Unknown, score: 0.18
Psen2Unknown, score: 0.15
Psma2Unknown, score: 0.11
Psmb1Unknown, score: 0.08
Psmb6Unknown, score: 0.02
Psmb7Unknown, score: 0.21
Psmc1Unknown, score: 0.25
Psmc2Unknown, score: 0.14
Pstpip1Unknown, score: 0.09
Pstpip2Unknown, score: 0
PtafrUnknown, score: -0.01
Ptch2Unknown, score: 0.32
Ptdss1Unknown, score: 0.12
PtenUnknown, score: 0.39
PterUnknown, score: 0.03
Ptger2Unknown, score: 0.01
PtgfrnUnknown, score: 0.14
Ptgs1Unknown, score: 0.1
Ptk2bUnknown, score: 0.25
Twf1Unknown, score: 0.3
PtmaUnknown, score: 0.36
Tmsb10Unknown, score: 0.22
Tmsb4xUnknown, score: 0.48
PtnUnknown, score: 0.61
Ptp4a1Unknown, score: 0.2
Ptpn11Unknown, score: 0.17
Ptpn18Unknown, score: 0.36
Ptpn2Unknown, score: 0.39
Ptpn4Unknown, score: 0.13
SirpaUnknown, score: 0.34
PtpraUnknown, score: 0.04
PtprbUnknown, score: 0.15
PtprdUnknown, score: 0.33
PtpreUnknown, score: 0.22
PtprfUnknown, score: 0.21
PtprjUnknown, score: 0.2
PtpruUnknown, score: 0.1
PtprmUnknown, score: 0.08
PtprnUnknown, score: 0.26
PtprsUnknown, score: 0.44
PtprtUnknown, score: 0.47
PtrfUnknown, score: 0.4
Ptx3Unknown, score: 0.07
Igdcc3Unknown, score: 0.26
PuraUnknown, score: 0.1
Pvrl2Unknown, score: 0.29
Pex19Unknown, score: 0.26
PygmUnknown, score: 0.17
QkUnknown, score: 0.21
Rab1Unknown, score: 0.19
Rab10Unknown, score: 0.03
Rab11bUnknown, score: 0.61
Rab12Unknown, score: 0.11
Rab17Unknown, score: 0.26
Rab20Unknown, score: 0.02
Rab22aUnknown, score: 0.06
Rab24Unknown, score: 0.17
Rab33aUnknown, score: 0.36
Rab3aUnknown, score: 0.33
Rab4bUnknown, score: 0.04
Rab5cUnknown, score: 0.12
Rab6aUnknown, score: 0.22
Dennd5aUnknown, score: 0.41
Kif20aUnknown, score: 0.19
Rab7Unknown, score: 0.03
RabggtbUnknown, score: 0.37
Rad1Unknown, score: 0.32
Rad17Unknown, score: 0
Rad21Unknown, score: 0.09
Rad23bUnknown, score: 0.44
Rad50Unknown, score: 0.31
Rad51Unknown, score: 0.29
Rad51bUnknown, score: 0.2
Rad52Unknown, score: 0.17
Rad9aUnknown, score: 0.1
Raet1dUnknown, score: 0.1
Raet1dUnknown, score: 0.12
RanUnknown, score: 0.29
Ranbp1Unknown, score: 0.1
Rangap1Unknown, score: 0.15
RargUnknown, score: 0.32
Rasd1Unknown, score: 0.02
Rasgrp1Unknown, score: 0.1
Rasl2-9Unknown, score: 0.27
Rb1Unknown, score: 0.1
Rbbp4Unknown, score: 0.26
Rbbp6Unknown, score: 0.11
Robo3Unknown, score: 0.49
Rbl1Unknown, score: 0.14
Rbl2Unknown, score: 0.07
Rbm3Unknown, score: 0.17
Rbm6Unknown, score: 0.09
Rbmxl1Unknown, score: 0.39
Rbp4Unknown, score: 0.13
Rce1Unknown, score: 0.27
Rdh5Unknown, score: 0.35
Rdh16Unknown, score: 0.04
RdxUnknown, score: 0.64
Rfc1Unknown, score: 0.06
RecqlUnknown, score: 0.15
RelbUnknown, score: 0.15
RelnUnknown, score: 0
Rem1Unknown, score: 0
Upf1Unknown, score: 0.16
Reps1Unknown, score: 0.02
RetUnknown, score: 0.05
Rev3lUnknown, score: 0.84
RfngUnknown, score: 0.13
Rfx3Unknown, score: 0.06
RfxankUnknown, score: 0.12
Rgl2Unknown, score: 0.02
Rgs4Unknown, score: 0.31
RhagUnknown, score: 0.02
RhebUnknown, score: 0.12
RhdUnknown, score: 0.26
Ring1Unknown, score: 0.01
Rit1Unknown, score: 0.21
Xpr1Unknown, score: 0.29
Rnaseh1Unknown, score: 0.24
RlimUnknown, score: 0.32
Rnf2Unknown, score: 0.19
Rnf4Unknown, score: 0.09
Robo1Unknown, score: 0.32
Rock1Unknown, score: 0.1
Rock2Unknown, score: 0.21
Mst1rUnknown, score: 0.27
Rp1Unknown, score: 0.22
Rp2hUnknown, score: 0.08
Rpa2Unknown, score: 0.17
Rpl10aUnknown, score: 0.05
Rpl18Unknown, score: 0.19
Rpl21Unknown, score: 0.03
Mrpl23Unknown, score: 0.11
Rpl30Unknown, score: 0.04
Rpl6Unknown, score: 0.03
Polr1bUnknown, score: 0.17
Polr2aUnknown, score: 0.05
Polr2jUnknown, score: 0.78
Rps14Unknown, score: -0.01
Rps16Unknown, score: 0.05
Rps17Unknown, score: 0.21
Rps19Unknown, score: 0.08
Rps3a1Unknown, score: 0.01
Rps5Unknown, score: 0.34
Rps6ka2Unknown, score: 0.09
Rps7Unknown, score: 0.17
Rps8Unknown, score: 0.2
Trim30aUnknown, score: 0.06
RrasUnknown, score: 0
Rs1Unknown, score: 0.08
Dhrs3Unknown, score: 0.83
Rtn2Unknown, score: 0.05
Hps6Unknown, score: 0.04
RxrgUnknown, score: 0.17
Uimc1Unknown, score: 0.33
Ncor1Unknown, score: 0.15
RykUnknown, score: 0.03
Ryr2Unknown, score: 0.57
Ryr3Unknown, score: 0.32
S100a10Unknown, score: 0.09
S100a9Unknown, score: 0.23
Prrx2Unknown, score: 0.06
Saa1Unknown, score: 0
Saa3Unknown, score: 0.43
SagUnknown, score: 0.09
Khdrbs1Unknown, score: 0.33
ApcsUnknown, score: 0.25
Sf3a2Unknown, score: 0.05
Sar1aUnknown, score: 0.16
Sart1Unknown, score: 0.08
Scd2Unknown, score: 0.04
Scg2Unknown, score: 0.03
Scg3Unknown, score: 0.35
Stmn2Unknown, score: 0.29
Scn10aUnknown, score: 0.14
Scn5aUnknown, score: 0.26
Scnn1bUnknown, score: 0.18
Scp2Unknown, score: 0.08
Ccl22Unknown, score: 0.08
Ccl4Unknown, score: 0.23
Ccl5Unknown, score: 0.02
Sdf2Unknown, score: 0.15
Serpinf1Unknown, score: 0.09
NptnUnknown, score: 0.02
SordUnknown, score: 0.14
SdprUnknown, score: 0.04
Sec22bUnknown, score: 0.03
Sec23aUnknown, score: -0.01
Sec61gUnknown, score: 0.21
Exoc4Unknown, score: 0.02
Selenbp2Unknown, score: 0.07
SelplgUnknown, score: 0.39
Sema3bUnknown, score: 0.27
Sema3cUnknown, score: 0.27
Sema4dUnknown, score: 0.05
Sema4fUnknown, score: 0.08
Sema5aUnknown, score: 0.35
Sema5bUnknown, score: 0.41
Sept8Unknown, score: 0.29
Sepw1Unknown, score: 0.26
Serf1Unknown, score: 0.31
Spi1Unknown, score: 0.09
Sfrp1Unknown, score: 0.12
Srsf3Unknown, score: 0.04
Srsf5Unknown, score: 0.2
SgcaUnknown, score: 0.32
Sgk1Unknown, score: 0.15
Scg5Unknown, score: 0.1
Zfp106Unknown, score: 0.32
Itsn2Unknown, score: 0.01
Sh3gl1Unknown, score: 0.14
20408Unknown, score: 0.08
Shfm1Unknown, score: 0.4
Shmt1Unknown, score: 0.26
Siah2Unknown, score: 0.27
St6galnac1Unknown, score: 0.34
St8sia1Unknown, score: 0.24
St8sia2Unknown, score: 0.4
Ptk6Unknown, score: 0
StilUnknown, score: 0.03
Cox7a2lUnknown, score: 0.04
Six3Unknown, score: 0.18
Six5Unknown, score: 0.04
Six6Unknown, score: 0
SkiUnknown, score: 0.03
SlaUnknown, score: 0.02
SlbpUnknown, score: 0.17
Slc10a1Unknown, score: 0.09
Slc12a3Unknown, score: 0
Slc16a2Unknown, score: 0.04
Slc19a1Unknown, score: 0.09
Slc1a1Unknown, score: 0.05
Slc1a2Unknown, score: 0.29
Slc1a3Unknown, score: 0
Slc20a2Unknown, score: 0.14
Slc22a1Unknown, score: 0.08
Slc22a3Unknown, score: 0
Slc22a12Unknown, score: 0.02
Slc23a1Unknown, score: 0.04
Slc25a14Unknown, score: 0.33
Slc2a1Unknown, score: 0.11
Slc2a4Unknown, score: 0.03
Slc31a2Unknown, score: 0.12
Slc4a1Unknown, score: 0.15
Slc5a1Unknown, score: 0.01
Slc9a1Unknown, score: 0.03
Slfn1Unknown, score: 0.03
Slfn2Unknown, score: 0
Slfn3Unknown, score: 0.08
Slit1Unknown, score: 0.33
SlpiUnknown, score: 0.27
Snai2Unknown, score: 0.26
HltfUnknown, score: 0.14
Smarca4Unknown, score: 0
Smarcb1Unknown, score: 0.24
Kdm5cUnknown, score: 0.35
Kdm5dUnknown, score: 0.17
Ncor2Unknown, score: 0.17
SmsUnknown, score: 0.13
Sstr1Unknown, score: 0
SnapinUnknown, score: 0.26
SncgUnknown, score: 0.78
Snap23Unknown, score: 0.39
Plk2Unknown, score: 0.18
SnnUnknown, score: 0.06
SnrpbUnknown, score: 0.37
Snrpb2Unknown, score: 0.26
Snta1Unknown, score: 0.61
Sntb1Unknown, score: 0.01
Sntb2Unknown, score: 0.11
Sod3Unknown, score: 0.01
Sorl1Unknown, score: 0.05
Sort1Unknown, score: 0.32
Sos1Unknown, score: 0.18
Sos2Unknown, score: 0.07
Sox10Unknown, score: 0.04
Sox13Unknown, score: 0.09
Sox18Unknown, score: 0
Sox2Unknown, score: 0.14
Sox4Unknown, score: 0.36
Sox8Unknown, score: 0.01
Sox9Unknown, score: 0.13
Sp1Unknown, score: 0.33
Sp100Unknown, score: 0.34
Spa17Unknown, score: 0.33
Sp3Unknown, score: 0.12
Sp4Unknown, score: 0.18
Sphk1Unknown, score: 0.19
Serpina1aUnknown, score: 0.13
Serpina1bUnknown, score: 0.32
Serpina1cUnknown, score: 0.19
Serpina1aUnknown, score: 0.52
Serpina1eUnknown, score: 0.06
Serpinb9bUnknown, score: 0.51
Serpinb6bUnknown, score: 0.01
Serpinb9fUnknown, score: 0
Serpinb9eUnknown, score: 0.19
Serpini1Unknown, score: 0.39
Serpina3nUnknown, score: 0.15
Serpine2Unknown, score: 0.44
Serpinb9Unknown, score: 0.09
SpicUnknown, score: 0.2
SpnUnknown, score: 0.3
Spta1Unknown, score: 0.05
SptbUnknown, score: 0
Sptbn1Unknown, score: 0.02
StrbpUnknown, score: 0.13
TmieUnknown, score: 0.12
Srp14Unknown, score: 0.01
Srpk1Unknown, score: 0.25
SrprbUnknown, score: 0.17
Trim21Unknown, score: 0.21
Trove2Unknown, score: 0.33
Ssr4Unknown, score: 0.08
StacUnknown, score: 0.23
Zfp143Unknown, score: 0.04
Stag1Unknown, score: 0.6
Stag2Unknown, score: 0.39
StarUnknown, score: 0.03
Stat6Unknown, score: 0.07
Stau1Unknown, score: 0.04
Stc1Unknown, score: 0.15
Stim1Unknown, score: 0.22
Stk11Unknown, score: 0.03
Stk16Unknown, score: 0.18
AC146980.1Unknown, score: 0.18
AurkbUnknown, score: 0.16
AurkaUnknown, score: 0.24
Sult1a1Unknown, score: 0.15
Stra6Unknown, score: 0.36
StrapUnknown, score: -0.01
Stx1aUnknown, score: 0.2
Stx3Unknown, score: 0.03
Stxbp2Unknown, score: 0.17
Stxbp4Unknown, score: 0.39
Sucla2Unknown, score: 0
Abcc8Unknown, score: 0.38
Surf1Unknown, score: 0.27
Surf2Unknown, score: 0.02
Surf6Unknown, score: 0.11
Suv39h1Unknown, score: 0.04
Swap70Unknown, score: 0.27
Vamp7Unknown, score: 0.23
Sycp1Unknown, score: 0.2
Syn1Unknown, score: 0.2
Sdc3Unknown, score: 0.06
Sdc4Unknown, score: 0.01
Syngr3Unknown, score: 0.19
Tac1Unknown, score: 0.42
Tacr3Unknown, score: 0.37
Taf1aUnknown, score: 0.18
Tagln2Unknown, score: 0.02
Taldo1Unknown, score: 0.15
Cntn2Unknown, score: 0.2
Tbl1xUnknown, score: 0.4
Tbrg4Unknown, score: 0.04
Tbx1Unknown, score: 0.44
Tbxa2rUnknown, score: 0.01
TcapUnknown, score: 0.13
Tcea2Unknown, score: 0.27
Tcea3Unknown, score: 0.06
Skp1aUnknown, score: 0.05
Hnf1aUnknown, score: 0.17
Tcf15Unknown, score: 0.31
Tcf21Unknown, score: 0.3
Tcf7Unknown, score: 0.07
Tcf7l1Unknown, score: 0.21
Tcf7l2Unknown, score: 0.03
Zeb1Unknown, score: 0.06
Tfap2aUnknown, score: 0.12
Tfap2bUnknown, score: 0.02
Tfap2cUnknown, score: 0
Vps72Unknown, score: 0.18
MlxUnknown, score: 0.18
Tcn2Unknown, score: 0.01
Tcof1Unknown, score: 0.1
Tcp1Unknown, score: 0.03
Tcte2Unknown, score: 0.44
Phf1Unknown, score: 0.3
Phlda1Unknown, score: 0.1
TdgUnknown, score: 0.35
Tead1Unknown, score: 0.01
Tead4Unknown, score: 0.14
TectaUnknown, score: 0.25
TectbUnknown, score: 0.02
TefUnknown, score: 0.09
Tep1Unknown, score: 0.33
Terf1Unknown, score: 0.56
Tesk1Unknown, score: 0.32
Psmd2Unknown, score: 0.11
Tex261Unknown, score: 0.04
Zfand3Unknown, score: 0.12
Cirh1aUnknown, score: 0
Tex9Unknown, score: 0.43
Tfdp1Unknown, score: 0.09
TfpiUnknown, score: 0.08
Tgfb1Unknown, score: 0.04
Tgfb1i1Unknown, score: 0.05
Tsc22d1Unknown, score: 0.05
Tgfb2Unknown, score: 0.14
TgfbiUnknown, score: 0.25
Tgfbr2Unknown, score: 0.08
Tgfbr3Unknown, score: 0.28
Tgm3Unknown, score: 0.14
TgUnknown, score: 0.3
Ift88Unknown, score: 0.45
Thbs3Unknown, score: 0.28
ThraUnknown, score: 0
Thy1Unknown, score: 0
Tia1Unknown, score: 0.29
Tial1Unknown, score: 0.17
Tie1Unknown, score: 0.26
Klf10Unknown, score: 0.69
Trim24Unknown, score: 0.02
Timp1Unknown, score: 0.07
Timp2Unknown, score: 0
Timp3Unknown, score: 0.21
Nkx2-1Unknown, score: 0.02
Atp6v0a2Unknown, score: 0.07
Tjp1Unknown, score: 0.21
Tjp2Unknown, score: 0.06
Tle3Unknown, score: 0.23
Tle4Unknown, score: 0.18
Tlr1Unknown, score: 0.15
Tlr4Unknown, score: 0.05
Tlx1Unknown, score: 0.17
Tspan7Unknown, score: 0.09
Tmod1Unknown, score: 0.13
TmpoUnknown, score: 0.01
TncUnknown, score: 0.05
Tnnc1Unknown, score: 0.15
Tnnc2Unknown, score: 0.02
TnfUnknown, score: 0.09
Tnfaip1Unknown, score: 0.08
Tnfaip2Unknown, score: 0
Tnfaip6Unknown, score: 0.16
Tnfrsf10bUnknown, score: 0.21
Tnfrsf1aUnknown, score: 0.2
Cd27Unknown, score: 0.12
Tnfrsf8Unknown, score: 0.01
Tnfsf11Unknown, score: 0.11
Tnfsf9Unknown, score: 0.06
TnksUnknown, score: 0.18
Tnni3Unknown, score: 0.45
Tnnt1Unknown, score: 0.06
Tnnt2Unknown, score: 0
Tom1Unknown, score: 0.16
Top1Unknown, score: 0.33
Top2aUnknown, score: 0.19
Top2bUnknown, score: 0.08
Top3bUnknown, score: 0.12
Tpd52Unknown, score: 0.5
Tpi1Unknown, score: 0.11
Tpm1Unknown, score: 0.07
Tpm2Unknown, score: 0.05
TpmtUnknown, score: 0.33
Tpp2Unknown, score: 0.54
Tpst2Unknown, score: 0.24
Nr2c2Unknown, score: 0.43
Traf4Unknown, score: 0.1
Traf6Unknown, score: 0.22
Tnfsf10Unknown, score: 0.18
Plscr1Unknown, score: 0.15
Trex1Unknown, score: 0.2
Tob1Unknown, score: 0.1
Trp53Unknown, score: 0.21
Trp63Unknown, score: 0.07
Trpc2Unknown, score: 0.02
Trpc4Unknown, score: -0.01
Tpt1Unknown, score: 0.27
Tsc2Unknown, score: 0.06
Tsg101Unknown, score: 0.13
TshrUnknown, score: 0.1
TsnUnknown, score: 0
Tssk2Unknown, score: 0.02
Psmd3Unknown, score: 0.22
Ttc3Unknown, score: 0.17
Tgoln1Unknown, score: 0.22
TtnUnknown, score: 0.05
Tuba1bUnknown, score: 0.05
Tuba1cUnknown, score: 0.05
Tubb5Unknown, score: 0.22
Tuft1Unknown, score: 0.07
Tulp1Unknown, score: 0.02
Tulp3Unknown, score: 0.11
TxkUnknown, score: 0.04
Txn1Unknown, score: 0.1
Cmpk2Unknown, score: 0.23
TymsUnknown, score: 0.25
TyrobpUnknown, score: 0.36
Zrsr1Unknown, score: 0.07
Zrsr2Unknown, score: 0.04
Ube2e3Unknown, score: 0.15
Ube2e1Unknown, score: 0.39
Ube2l3Unknown, score: 0.21
Ube2iUnknown, score: 0.19
Ube2hUnknown, score: 0.63
Usp12Unknown, score: 0.44
Sumo1Unknown, score: 0.07
Ubp1Unknown, score: 0.12
Usp10Unknown, score: 0.04
Ucp2Unknown, score: 0.43
Ucp3Unknown, score: 0.23
Slc35a2Unknown, score: 0.67
UgcgUnknown, score: 0.26
UgdhUnknown, score: 0.29
Ugt2b5Unknown, score: 0.34
Unc13bUnknown, score: 0.26
UngUnknown, score: 0.07
Nr1h2Unknown, score: 0.4
UoxUnknown, score: 0.08
Upk1bUnknown, score: 0.06
Upk2Unknown, score: 0.44
UrodUnknown, score: 0.13
Usf2Unknown, score: 0.03
Ush2aUnknown, score: 0.01
Scgb1a1Unknown, score: 0.18
UtrnUnknown, score: 0.1
Kdm6aUnknown, score: 0.05
Slc45a2Unknown, score: 0.06
Cdh23Unknown, score: 0.23
Vamp1Unknown, score: 0
Vamp2Unknown, score: 0.07
VarsUnknown, score: 0.15
Vav1Unknown, score: 0.03
Vav2Unknown, score: 0.13
Vbp1Unknown, score: 0.14
Vdac1Unknown, score: 0.01
Vdac2Unknown, score: 0.02
Vdac3Unknown, score: -0.01
VdrUnknown, score: 0.19
VegfbUnknown, score: 0.26
Lin7bUnknown, score: 0.18
VhlUnknown, score: 0.12
Vil1Unknown, score: 0.15
VimUnknown, score: 0.01
VipUnknown, score: 0.55
Vipr2Unknown, score: 0.27
Vnn1Unknown, score: 0
Vpreb3Unknown, score: 0.25
VwfUnknown, score: 0.25
Wbp2Unknown, score: 0.05
Fmnl3Unknown, score: 0.46
Wbp4Unknown, score: 0.35
Eif4hUnknown, score: 0.34
Wisp1Unknown, score: 0.04
Wnt2bUnknown, score: 0.06
Wnt5bUnknown, score: -0.01
Wnt7aUnknown, score: 0.15
Wnt8bUnknown, score: 0.24
WrnUnknown, score: 0.18
Wt1Unknown, score: 0.02
XdhUnknown, score: 0
Xirp1Unknown, score: 0.2
XkUnknown, score: 0.63
Xlr3aUnknown, score: 0.04
Xlr3cUnknown, score: 0.2
AtrxUnknown, score: 0.56
XpaUnknown, score: 0.15
Xrcc1Unknown, score: 0.35
Yap1Unknown, score: 0.2
Ybx1Unknown, score: 0.45
Yes1Unknown, score: 0.15
Slc23a3Unknown, score: 0.26
YwhagUnknown, score: 0.07
YwhahUnknown, score: 0.24
YwhaqUnknown, score: 0.18
Zap70Unknown, score: 0.05
Zbtb17Unknown, score: 0.47
Zfp101Unknown, score: 0.05
Rnf103Unknown, score: 0.1
Zfp105Unknown, score: 0.09
Pcgf2Unknown, score: 0.04
Zfp148Unknown, score: 0.16
Sf1Unknown, score: 0.02
Trim26Unknown, score: 0.04
Zfp185Unknown, score: 0.41
Zfp2Unknown, score: 0.17
Zfp207Unknown, score: 0.14
Zfp239Unknown, score: 0.03
Zpr1Unknown, score: 0.19
Zfp26Unknown, score: 0.01
Zfp27Unknown, score: 0.31
Zfp28Unknown, score: 0.02
Zscan2Unknown, score: 0
Zfp30Unknown, score: 0.34
Zfp36Unknown, score: 0.41
Zscan21Unknown, score: 0.03
Zfp39Unknown, score: 0.19
Zfp46Unknown, score: 0.31
Zfp51Unknown, score: 0.13
Zfp52Unknown, score: 0.02
Zfp54Unknown, score: 0.14
Zfp57Unknown, score: 0.29
Zfp59Unknown, score: 0.37
Zfp60Unknown, score: -0.01
Zfp61Unknown, score: 0.22
Zbtb7bUnknown, score: 0.5
Zfp85Unknown, score: 0.28
Zfp9Unknown, score: 0.2
Zfp90Unknown, score: 0.01
Zkscan5Unknown, score: 0.06
Zfpm2Unknown, score: 0.05
ZfrUnknown, score: 0.15
ZfxUnknown, score: 0.56
Zik1Unknown, score: 0.14
Zim1Unknown, score: 0.03
Ikzf1Unknown, score: 0.31
Ikzf2Unknown, score: 0.18
Ikzf3Unknown, score: 0.17
Slc30a3Unknown, score: 0.1
Zp2Unknown, score: 0.56
Adam23Unknown, score: 0.26
Adamts5Unknown, score: 0.09
AplnrUnknown, score: 0.23
Apc2Unknown, score: 0.24
Arih2Unknown, score: 0.47
Bace1Unknown, score: 0.11
Bpnt1Unknown, score: 0.09
Car14Unknown, score: 0.25
Cd52Unknown, score: 0.12
Cdh20Unknown, score: 0.5
Cfdp1Unknown, score: 0.1
Clec5aUnknown, score: 0.22
Dido1Unknown, score: 0.16
Dand5Unknown, score: 0.1
Ets2Unknown, score: 0.21
FaimUnknown, score: 0.25
Fxr2Unknown, score: 0.16
Gadd45gUnknown, score: 0.23
Gmcl1Unknown, score: 0.36
Gpc6Unknown, score: 0.19
Gpr34Unknown, score: 0.21
Grem2Unknown, score: 0.29
Gtf2h2Unknown, score: 0.03
HcstUnknown, score: 0.1
Hs2st1Unknown, score: 0.09
Impdh2Unknown, score: 0.27
JtbUnknown, score: 0.24
AadatUnknown, score: 0.23
KelUnknown, score: 0.07
Ly6hUnknown, score: 0.12
Lynx1Unknown, score: 0.17
Mab21l2Unknown, score: 0.07
Mapk7Unknown, score: 0.05
MgllUnknown, score: 0.41
Mmp17Unknown, score: 0.34
Dnajb6Unknown, score: 0.13
Nek4Unknown, score: 0.37
Nr0b2Unknown, score: 0.05
Nt5eUnknown, score: -0.01
Oas1bUnknown, score: 0.12
Oasl2Unknown, score: 0.23
Tenm1Unknown, score: 0.27
Tenm3Unknown, score: 0.02
Osr1Unknown, score: 0.25
Nlrp5Unknown, score: 0.31
Papss1Unknown, score: 0.38
Slc26a4Unknown, score: 0.29
Klk7Unknown, score: 0.04
Psmc4Unknown, score: 0.07
Psmd13Unknown, score: 0.21
Tiam2Unknown, score: 0.09
Rai2Unknown, score: 0.41
IkUnknown, score: 0.37
Rgs7Unknown, score: 0.11
Grk1Unknown, score: 0.45
RnaselUnknown, score: 0.3
Abce1Unknown, score: 0.46
Rnf13Unknown, score: 0.19
Ccl19Unknown, score: 0.03
Sept3Unknown, score: 0.08
Sh3bp2Unknown, score: 0.33
Sh3bp5Unknown, score: 0.11
Slco2a1Unknown, score: 0.16
Slc35a1Unknown, score: 0.26
Smc1aUnknown, score: 0.32
Spry1Unknown, score: 0.01
Spry2Unknown, score: 0.23
Spry4Unknown, score: 0.38
Srp54aUnknown, score: 0.02
Mpdu1Unknown, score: 0.34
Synj2bpUnknown, score: 0.33
Natd1Unknown, score: 0.15
Tekt2Unknown, score: 0.36
Tlk2Unknown, score: 0.19
Tll2Unknown, score: -0.01
Rbck1Unknown, score: 0
Ubl3Unknown, score: 0.13
NelfaUnknown, score: 0.4
Xrn1Unknown, score: 0.61
Xrn2Unknown, score: 0.04
Ldb3Unknown, score: 0.21
Zfp53Unknown, score: 0.44
Zfp68Unknown, score: 0.04
Zeb2Unknown, score: 0.11
Ing1Unknown, score: 0.18
Abcg2Unknown, score: 0.36
BtdUnknown, score: 0
Adgre5Unknown, score: 0.26
Clcn7Unknown, score: 0.32
FtoUnknown, score: 0
Hsf4Unknown, score: 0
Map2k3Unknown, score: 0.03
Map2k7Unknown, score: 0.17
Map3k11Unknown, score: 0.17
Map3k12Unknown, score: 0.23
Map3k4Unknown, score: 0.16
Map3k5Unknown, score: 0.14
Map3k7Unknown, score: -0.01
Map3k8Unknown, score: 0.23
Map4k2Unknown, score: 0
Mapk13Unknown, score: 0.25
Mapk3Unknown, score: 0.18
Mapk8Unknown, score: 0.15
Mapk9Unknown, score: 0.07
NbeaUnknown, score: 0
Nr5a1Unknown, score: 0.2
Creb3l1Unknown, score: 0.21
Orc4Unknown, score: 0.34
PargUnknown, score: 0.01
Git2Unknown, score: 0.65
Plod3Unknown, score: 0.37
PrndUnknown, score: 0.41
Psg16Unknown, score: 0.24
Psma1Unknown, score: 0.4
Psma4Unknown, score: 0.1
Psma5Unknown, score: 0.2
Psma6Unknown, score: 0.11
Psmb3Unknown, score: 0.05
MokUnknown, score: 0.47
Rbbp9Unknown, score: 0.4
Rpl27aUnknown, score: 0.32
Slc27a1Unknown, score: 0.36
Slc27a2Unknown, score: 0.17
Zfp146Unknown, score: 0.36
Itgb1bp2Unknown, score: 0.26
Homer1Unknown, score: 0.01
Homer3Unknown, score: 0.14
HunkUnknown, score: 0.05
Cops3Unknown, score: 0.12
Rcn2Unknown, score: 0.01
Abcd2Unknown, score: 0.32
PcloUnknown, score: 0.23
Adh4Unknown, score: 0.06
B3galt1Unknown, score: 0.2
B3galnt1Unknown, score: 0.24
Casp8ap2Unknown, score: 0.15
Chst4Unknown, score: 0.16
Clec4a2Unknown, score: 0.12
Cln8Unknown, score: 0.22
Cops6Unknown, score: 0.14
Cops7bUnknown, score: 0.02
Med14Unknown, score: 0.45
Acot1Unknown, score: 0.04
DysfUnknown, score: 0.23
Eif2s3xUnknown, score: 0.31
Eif2s3yUnknown, score: 0.12
GcatUnknown, score: 0.26
CntrlUnknown, score: 0.03
Aifm1Unknown, score: -0.01
Ppp2r5cUnknown, score: 0.18
Ppp2r5eUnknown, score: 0.2
MpripUnknown, score: 0.04
EcsitUnknown, score: 0.19
Slc9a3r1Unknown, score: 0.19
Spag1Unknown, score: 0.09
Rpl8Unknown, score: 0.17
Cul1Unknown, score: 0.16
Pla2g2fUnknown, score: 0.05
Spo11Unknown, score: 0.11
Eif4e2Unknown, score: 0.15
Brd7Unknown, score: 0.16
Micall1Unknown, score: 0.19
Tspan32Unknown, score: 0.05
SgshUnknown, score: 0.24
Nit1Unknown, score: 0.43
OmdUnknown, score: 0.03
AsnsUnknown, score: 0
Sec23bUnknown, score: 0.05
Ncoa4Unknown, score: -0.01
Srp9Unknown, score: 0.13
Tcirg1Unknown, score: 0.02
Bcap31Unknown, score: 0.08
B9d1Unknown, score: 0.53
Rpl7aUnknown, score: 0.4
PodxlUnknown, score: 0.47
NrkUnknown, score: 0.14
Rps11Unknown, score: 0.35
Dbf4Unknown, score: 0.06
Slamf1Unknown, score: 0.2
Chaf1aUnknown, score: 0.18
Atp1a4Unknown, score: 0.04
Trp53bp1Unknown, score: 0.04
CarsUnknown, score: 0.5
Pdk4Unknown, score: 0.36
Zfp354bUnknown, score: 0.37
Nufip1Unknown, score: 0.18
Plekhb1Unknown, score: 0.02
Golga5Unknown, score: 0.28
Tnfrsf12aUnknown, score: 0.09
Phlda3Unknown, score: 0.38
HraslsUnknown, score: 0.4
Pald1Unknown, score: 0.12
Insl6Unknown, score: 0.37
GygUnknown, score: 0.18
Add3Unknown, score: 0.13
Msrb1Unknown, score: 0.36
SrrUnknown, score: 0.27
Txnl4aUnknown, score: 0.04
Sh2d2aUnknown, score: 0.11
Csnk1eUnknown, score: 0.49
Slc25a10Unknown, score: 0.42
Yme1l1Unknown, score: 0.25
Mmel1Unknown, score: 0.06
PignUnknown, score: 0.18
Ip6k1Unknown, score: 0.24
Hsd17b6Unknown, score: 0.05
Skp2Unknown, score: 0.06
Abca7Unknown, score: 0.46
Abcf2Unknown, score: 0.15
Abca3Unknown, score: 0.03
Peg12Unknown, score: 0.37
SergefUnknown, score: 0.12
Abcc5Unknown, score: 0.1
NagluUnknown, score: 0.28
Shroom3Unknown, score: 0.54
AmotUnknown, score: 0.33
NrepUnknown, score: 0.2
NelfeUnknown, score: 0.11
Vwa7Unknown, score: 0.52
Tada1Unknown, score: 0.05
Eif3bUnknown, score: 0.06
Exosc5Unknown, score: 0.09
Prpf19Unknown, score: 0.24
Fam21Unknown, score: 0
MiipUnknown, score: 0.29
Ubfd1Unknown, score: 0.13
Mrpl50Unknown, score: 0.37
Gfm1Unknown, score: 0.35
Usp39Unknown, score: 0.43
Larp7Unknown, score: 0.18
Yipf3Unknown, score: 0.65
TwistnbUnknown, score: 0.02
Med10Unknown, score: 0.07
Fam104aUnknown, score: 0.19
MydgfUnknown, score: 0.17
Tinf2Unknown, score: 0.26
Nsun2Unknown, score: 0.19
Nop16Unknown, score: 0.39
Cep63Unknown, score: 0.1
Serp1Unknown, score: 0.01
Agpat3Unknown, score: 0.18
Tomm70aUnknown, score: 0.46
Reep3Unknown, score: 0.26
Dcaf11Unknown, score: 0.2
Trpm2Unknown, score: 0.36
Slco1a1Unknown, score: 0.36
Slco1a4Unknown, score: 0.17
Limd1Unknown, score: 0.23
Tpk1Unknown, score: 0.11
MgaUnknown, score: 0.09
Zfp385aUnknown, score: 0.3
Hip1rUnknown, score: 0.14
Hspb7Unknown, score: 0.31
Pmm1Unknown, score: 0.14
Sult4a1Unknown, score: 0.05
Pde7bUnknown, score: 0.01
Cabp1Unknown, score: 0.04
Ulk2Unknown, score: 0.04
Gtse1Unknown, score: 0.15
Scmh1Unknown, score: 0.19
Iqgap1Unknown, score: 0.24
Clic4Unknown, score: 0.22
Opn4Unknown, score: 0.2
Dnajc12Unknown, score: 0.2
Zfp292Unknown, score: 0.13
Pcsk1nUnknown, score: 0.17
Timm9Unknown, score: 0.03
Timm8bUnknown, score: 0.16
Cttnbp2Unknown, score: 0.01
Pdlim4Unknown, score: 0.49
Slc22a4Unknown, score: 0.01
Mlf2Unknown, score: 0.49
Gnl3Unknown, score: 0.28
AplnUnknown, score: 0.42
Angptl3Unknown, score: 0.02
Slamf6Unknown, score: 0.83
Glrx3Unknown, score: 0.1
Snai3Unknown, score: 0.12
Zbtb18Unknown, score: 0.4
Vps26aUnknown, score: 0
Tor1aUnknown, score: 0.05
Tor1bUnknown, score: 0.02
Slc46a2Unknown, score: 0.33
Fgd3Unknown, score: 0.17
Pttg1Unknown, score: 0.13
Usp21Unknown, score: 0.46
Zfp354cUnknown, score: 0.11
Abt1Unknown, score: 0.17
Adat1Unknown, score: 0.46
Bin1Unknown, score: 0.2
Cbx8Unknown, score: 0.4
Siva1Unknown, score: 0.13
Mapk8ip3Unknown, score: 0.06
Hacd1Unknown, score: 0.21
E2f6Unknown, score: 0.04
Ebi3Unknown, score: 0.47
Sall2Unknown, score: 0.01
Spag6Unknown, score: 0.3
Tmprss2Unknown, score: 0.1
Mfap5Unknown, score: 0.14
Hist1h1cUnknown, score: 0.38
Hist1h1eUnknown, score: -0.01
IcoslUnknown, score: -0.01
Fbxl17Unknown, score: 0.41
Fbxo6Unknown, score: 0.13
Atp9bUnknown, score: 0.14
Rgs1Unknown, score: 0.29
Rgs3Unknown, score: 0.01
Fbxl8Unknown, score: 0.17
Fbxl3Unknown, score: 0.21
Acsl4Unknown, score: 0.3
Magi2Unknown, score: 0.35
Klf13Unknown, score: 0.27
Sh3bgrUnknown, score: 0.18
GneUnknown, score: 0.11
Rnf10Unknown, score: 0.2
Park2Unknown, score: 0.19
Tmod3Unknown, score: 0.3
Stag3Unknown, score: 0.11
SclyUnknown, score: 0.37
Chek2Unknown, score: 0.47
Nckap1Unknown, score: 0.34
Il17rbUnknown, score: 0.18
C1raUnknown, score: 0.49
Exosc10Unknown, score: 0.16
MyadmUnknown, score: 0.02
HnrnpdlUnknown, score: 0.39
NaspUnknown, score: 0.18
Tnfsf14Unknown, score: 0.26
Mink1Unknown, score: 0.35
Uba2Unknown, score: 0.21
Pdcd7Unknown, score: 0.04
Mpp2Unknown, score: 0.01
SrpxUnknown, score: 0.25
CtpsUnknown, score: 0.18
BokUnknown, score: 0.36
Ramp1Unknown, score: 0.06
HnrnpuUnknown, score: 0.4
Mcrs1Unknown, score: 0.28
CcncUnknown, score: 0.05
Rif1Unknown, score: 0.15
Tubgcp4Unknown, score: 0.18
Atg13Unknown, score: 0.3
Rnf24Unknown, score: 0.34
Ccdc39Unknown, score: 0.28
Cdk2ap2Unknown, score: 0.21
Hn1lUnknown, score: 0.11
R3hcc1lUnknown, score: 0.14
Nus1Unknown, score: 0.42
Pibf1Unknown, score: 0.07
Ppp6r3Unknown, score: 0.13
Coq5Unknown, score: 0.1
Mfhas1Unknown, score: 0.06
Tmem38bUnknown, score: 0.08
HgsnatUnknown, score: 0.49
Kcnk6Unknown, score: 0.23
Tmem222Unknown, score: 0.54
Odf2lUnknown, score: 0.05
RragdUnknown, score: 0.1
Rbm34Unknown, score: 0.14
Commd2Unknown, score: 0.01
Reep1Unknown, score: 0.23
Cdca8Unknown, score: 0.08
Klhl7Unknown, score: 0.65
Atxn1lUnknown, score: 0.13
D6Ertd527eUnknown, score: 0.04
Adgra1Unknown, score: 0.09
D1Ertd622eUnknown, score: 0.31
Echdc2Unknown, score: 0.26
Ppp2r2dUnknown, score: 0.35
Tax1bp1Unknown, score: 0.26
Carhsp1Unknown, score: 0.12
CenpoUnknown, score: 0
Ddx56Unknown, score: 0.27
Zfp622Unknown, score: 0.08
Mettl17Unknown, score: 0.1
SgtaUnknown, score: 0.05
Ccdc69Unknown, score: 0.05
Trmt10cUnknown, score: -0.01
Tspan14Unknown, score: 0.04
NcaldUnknown, score: 0.14
Adgre4Unknown, score: 0.17
Suz12Unknown, score: 0.28
Cdkn2aipnlUnknown, score: 0.1
Nit2Unknown, score: 0.02
Cisd1Unknown, score: 0.17
Wipi1Unknown, score: 0.22
Cpsf4lUnknown, score: 0.28
Ncaph2Unknown, score: 0.07
Setd3Unknown, score: 0.4
ZwintUnknown, score: 0.73
Txndc17Unknown, score: 0.55
Krr1Unknown, score: 0
Slc52a2Unknown, score: 0.03
Zkscan6Unknown, score: 0.58
Ccdc59Unknown, score: 0.42
Ccdc43Unknown, score: 0.29
Anapc16Unknown, score: 0.08
Tspyl2Unknown, score: 0.12
Rufy3Unknown, score: 0.14
Lurap1lUnknown, score: 0.76
DnlzUnknown, score: 0.11
Sgsm1Unknown, score: 0.17
Mtg2Unknown, score: 0.09
CdiptUnknown, score: 0.09
Slx4Unknown, score: 0.66
D19Bwg1357eUnknown, score: 0.32
Rgs7bpUnknown, score: 0.07
Sco1Unknown, score: 0.09
Rbfox3Unknown, score: 0.18
RnasekUnknown, score: 0.08
Zmiz2Unknown, score: 0.3
Dlg3Unknown, score: 0
MybphUnknown, score: 0.18
Cntnap1Unknown, score: 0.25
Nucb2Unknown, score: 0.05
BanpUnknown, score: 0.04
Pgrmc1Unknown, score: 0.43
Tomm40Unknown, score: 0.21
Gosr1Unknown, score: 0.04
Usp2Unknown, score: 0.23
SdcbpUnknown, score: 0.18
Hnrnpa2b1Unknown, score: 0.36
Prdx4Unknown, score: 0.27
Ppp1r3cUnknown, score: 0.43
ByslUnknown, score: 0.12
Dctn3Unknown, score: 0
Timm23Unknown, score: 0.21
Pcdh12Unknown, score: 0.05
TslpUnknown, score: 0.19
ZpbpUnknown, score: 0.33
ClasrpUnknown, score: 0.02
Vti1bUnknown, score: 0.09
ReckUnknown, score: 0.08
Vamp5Unknown, score: 0.48
Cnot4Unknown, score: 0.59
B3gnt2Unknown, score: 0.02
Ddx39bUnknown, score: 0.15
Tuba8Unknown, score: 0.18
Sept9Unknown, score: 0.62
Zranb2Unknown, score: 0
Col5a3Unknown, score: 0.44
Rab11aUnknown, score: 0.31
Pkd2l2Unknown, score: 0.09
Ear6Unknown, score: 0.22
Naip6Unknown, score: 0.12
Nphp1Unknown, score: 0.18
Sart3Unknown, score: 0.27
Ppm1dUnknown, score: 0.23
Nudt5Unknown, score: 0.05
Gal3st1Unknown, score: 0.09
Rcan3Unknown, score: 0.36
Slc40a1Unknown, score: 0.18
Rfx5Unknown, score: 0.05
NgefUnknown, score: 0.28
Ddx20Unknown, score: 0.11
Lpar2Unknown, score: 0.13
Gipc2Unknown, score: 0.21
PolmUnknown, score: 0.06
Rps28Unknown, score: -0.01
Pmm2Unknown, score: 0.1
AcrbpUnknown, score: 0.32
Atxn10Unknown, score: 0.14
Irf6Unknown, score: 0.37
Avpr1aUnknown, score: 0.26
Spag5Unknown, score: 0.38
Rasa4Unknown, score: 0.12
Copg2Unknown, score: 0.09
IcosUnknown, score: 0
RragcUnknown, score: 0.18
Cpsf4Unknown, score: 0.22
Rabep1Unknown, score: 0.21
Akap8lUnknown, score: 0.23
Gucy1b3Unknown, score: 0.19
Rnf5Unknown, score: 0.16
Sept1Unknown, score: 0.19
Arl6ip1Unknown, score: 0.14
Cd320Unknown, score: 0.15
Elovl1Unknown, score: 0.35
Slc23a2Unknown, score: 0.4
Skap2Unknown, score: 0.14
Rassf5Unknown, score: 0.1
Epb4.1l4bUnknown, score: 0.43
Ctnnal1Unknown, score: 0.22
Nme6Unknown, score: 0.08
Chst2Unknown, score: 0.21
Azin1Unknown, score: 0.15
Cacng4Unknown, score: 0.23
Smarcal1Unknown, score: 0.35
Phc2Unknown, score: 0.02
Sit1Unknown, score: 0.15
RfkUnknown, score: 0.12
NcapgUnknown, score: 0.14
Gabbr1Unknown, score: -0.01
Bet1lUnknown, score: 0.08
Atp6ap1Unknown, score: 0.03
Nfat5Unknown, score: 0.41
Mkrn1Unknown, score: 0.07
Dll4Unknown, score: 0.28
HpgdsUnknown, score: 0.34
Syt7Unknown, score: 0.39
Nup210Unknown, score: 0.27
PcnxUnknown, score: 0.14
Ubqln2Unknown, score: 0
Sfrp5Unknown, score: 0.2
NpffUnknown, score: 0.02
Extl3Unknown, score: 0.18
Prickle3Unknown, score: 0.39
Nphs1Unknown, score: 0.01
Ftsj1Unknown, score: 0.41
MagixUnknown, score: 0.56
Ccdc22Unknown, score: 0.22
Otud5Unknown, score: 0.16
Sfmbt1Unknown, score: 0.06
Atp8b2Unknown, score: 0.24
Atp8b1Unknown, score: -0.01
Adgrg3Unknown, score: 0.42
Sh3glb1Unknown, score: 0.13
Zfp108Unknown, score: 0.05
Prdx5Unknown, score: 0.02
Plagl2Unknown, score: 0.18
Plxnc1Unknown, score: 0.25
Tyk2Unknown, score: 0.55
Tfip11Unknown, score: 0.21
Hes6Unknown, score: 0.31
Gbp3Unknown, score: 0.33
Rp9Unknown, score: 0
Fnbp4Unknown, score: 0.14
Ctps2Unknown, score: 0.09
Sertad1Unknown, score: 0.41
Stx8Unknown, score: 0.01
Eif3dUnknown, score: 0.4
Mpc1Unknown, score: 0.31
Slc1a4Unknown, score: 0.2
Paxip1Unknown, score: 0.22
Cxcl13Unknown, score: 0.24
Snx12Unknown, score: 0.46
Nop58Unknown, score: 0.04
Trim3Unknown, score: 0.05
Msh4Unknown, score: 0.35
Smad9Unknown, score: 0.01
Alyref2Unknown, score: 0.19
Srcin1Unknown, score: 0.3
Tmem131Unknown, score: 0.07
Nprl2Unknown, score: 0.07
Rplp1Unknown, score: 0.29
Uso1Unknown, score: 0.45
Akr1e1Unknown, score: 0.42
RalaUnknown, score: 0.33
Samhd1Unknown, score: 0.03
MslnUnknown, score: 0.33
Lgals8Unknown, score: 0.35
Gtpbp2Unknown, score: 0.16
Ammecr1Unknown, score: 0.2
Pdss1Unknown, score: 0.08
DgkeUnknown, score: -0.01
Car5bUnknown, score: 0.08
Astn2Unknown, score: 0.04
Ubqln1Unknown, score: 0.35
Dnah10Unknown, score: 0.04
Ramp3Unknown, score: 0.3
GraspUnknown, score: 0.2
Mad2l1Unknown, score: 0.07
NagkUnknown, score: 0.51
Bace2Unknown, score: 0.07
RabggtaUnknown, score: 0.08
Fxyd1Unknown, score: 0.14
Rbm38Unknown, score: 0.28
TroUnknown, score: 0.04
PlekUnknown, score: 0.41
Prpf40aUnknown, score: 0.55
Ptbp2Unknown, score: 0.45
Tdp2Unknown, score: 0.09
HeylUnknown, score: 0
Abcb10Unknown, score: 0.11
Ddx21Unknown, score: 0.02
EnsaUnknown, score: 0.65
Becn1Unknown, score: 0.23
Gde1Unknown, score: -0.01
Scamp4Unknown, score: 0.14
Stx1bUnknown, score: 0.29
Extl1Unknown, score: 0.18
Cited4Unknown, score: 0.74
EspnUnknown, score: 0.65
Hdac7Unknown, score: 0.1
Ak3Unknown, score: 0.07
Hnrnph2Unknown, score: 0.11
Pex14Unknown, score: 0.3
Tmem45aUnknown, score: 0.45
Mrpl12Unknown, score: 0.13
Naa10Unknown, score: 0.06
Slc35g3Unknown, score: 0.09
Higd1aUnknown, score: 0.46
Arl6Unknown, score: 0.03
Atl2Unknown, score: 0.26
FkbplUnknown, score: 0.19
PitpnbUnknown, score: 0.2
Fam60aUnknown, score: 0.09
Metap2Unknown, score: 0.14
MycbpUnknown, score: 0.05
Gps2Unknown, score: 0.06
Nupr1Unknown, score: 0.26
Dbn1Unknown, score: 0.27
Dnajb5Unknown, score: 0.1
Arl2Unknown, score: 0.02
Pdcd5Unknown, score: 0.02
Amotl2Unknown, score: 0.15
Tmed2Unknown, score: 0.23
B4galt5Unknown, score: 0.39
TxnipUnknown, score: 0.06
Eif3cUnknown, score: 0.06
Hsd17b12Unknown, score: 0.16
Net1Unknown, score: 0.37
Arl3Unknown, score: 0.1
Ptges3Unknown, score: 0.15
RybpUnknown, score: 0.32
Copz2Unknown, score: 0.23
Zmym3Unknown, score: 0.34
ClcnkbUnknown, score: 0.65
Cyb561d2Unknown, score: -0.01
ApipUnknown, score: 0.21
1110004F10RikUnknown, score: 0.39
Cpb2Unknown, score: 0.1
B4galt4Unknown, score: 0.19
Pdlim5Unknown, score: 0
Arpc3Unknown, score: 0.18
SpenUnknown, score: 0.54
Rab9Unknown, score: 0.18
Letm1Unknown, score: 0.11
B4galt6Unknown, score: 0.34
Sssca1Unknown, score: 0.11
Tmem115Unknown, score: -0.01
Chp1Unknown, score: 0
P3h1Unknown, score: 0.32
SyncripUnknown, score: 0.16
Trip4Unknown, score: 0.05
Ykt6Unknown, score: 0.17
PfkpUnknown, score: 0.09
Stub1Unknown, score: 0.63
Pdcd10Unknown, score: 0.34
Mtch2Unknown, score: 0.15
DptUnknown, score: 0.51
Clip1Unknown, score: 0.33
DstnUnknown, score: 0.36
Vps29Unknown, score: 0.24
Nat6Unknown, score: 0.23
Arpc1aUnknown, score: 0.63
Actr10Unknown, score: 0.18
Cyp2d22Unknown, score: 0.15
Suclg1Unknown, score: 0.08
Aldh18a1Unknown, score: 0.05
Clptm1Unknown, score: 0.28
Foxo1Unknown, score: 0.16
Mtch1Unknown, score: 0.03
Snd1Unknown, score: 0.17
Fads2Unknown, score: 0.32
Tbk1Unknown, score: 0.06
Foxo3Unknown, score: 0.13
Asna1Unknown, score: 0.44
Tspan6Unknown, score: 0.38
Elf4Unknown, score: 0.16
Srpk3Unknown, score: 0.01
Cib2Unknown, score: 0.37
Pard6aUnknown, score: 0.45
Slc22a21Unknown, score: 0.13
Nme4Unknown, score: 0.08
PapolbUnknown, score: 0.82
Zfp235Unknown, score: 0.1
Mast1Unknown, score: 0.37
Sec11aUnknown, score: 0.09
Cnpy2Unknown, score: 0.03
Ripk3Unknown, score: 0.8
Rgs17Unknown, score: 0.32
Hspb3Unknown, score: 0.2
Klk11Unknown, score: 0.01
Habp4Unknown, score: 0.06
Kcnd3Unknown, score: 0.11
Vmn2r1Unknown, score: 0.09
Sec1Unknown, score: 0.2
Txn2Unknown, score: 0.1
Raet1dUnknown, score: 0.02
Rps6ka4Unknown, score: 0.02
Clec4nUnknown, score: 0.14
PollUnknown, score: 0.19
Dnase2bUnknown, score: 0.04
Sphk2Unknown, score: 0.12
Fgf21Unknown, score: 0.07
Gsk3bUnknown, score: 0.05
Ankrd2Unknown, score: 0.44
Clec7aUnknown, score: 0.2
MlycdUnknown, score: 0.07
Gpr132Unknown, score: 0.03
Akap10Unknown, score: 0.49
GlmpUnknown, score: 0.06
PigoUnknown, score: 0.3
Ranbp9Unknown, score: 0.35
Ccnl1Unknown, score: 0.17
Dnajb12Unknown, score: 0.16
Brinp1Unknown, score: 0.08
Plag1Unknown, score: 0.07
MtorUnknown, score: 0.47
Tdo2Unknown, score: 0
LitafUnknown, score: 0.24
CriptUnknown, score: 0.49
Krt71Unknown, score: 0.01
Rnf14Unknown, score: 0.03
Mocs1Unknown, score: 0.05
Rec8Unknown, score: 0.22
Pf4Unknown, score: 0.05
DhodhUnknown, score: 0.33
Aldh9a1Unknown, score: -0.01
Clec1bUnknown, score: 0.32
Mllt11Unknown, score: 0.16
Ralgapa1Unknown, score: 0.05
Tmem9bUnknown, score: 0.32
Supt20Unknown, score: 0.23
Ube2l6Unknown, score: 0.1
Stap1Unknown, score: 0.15
Arl10Unknown, score: 0.01
Cacna2d2Unknown, score: 0.12
Ccl28Unknown, score: 0.41
Lgi1Unknown, score: 0.13
Necab3Unknown, score: 0.14
Zfp109Unknown, score: 0.05
Lmbr1Unknown, score: 0.2
NsmfUnknown, score: 0.37
Dolpp1Unknown, score: 0.16
Zfp276Unknown, score: 0.16
Ly6iUnknown, score: 0.15
GabrqUnknown, score: 0.33
Vav3Unknown, score: 0.22
Brd4Unknown, score: 0.05
RetnUnknown, score: 0.03
LenepUnknown, score: 0.09
BcamUnknown, score: 0.07
Psmd8Unknown, score: 0.02
NelfcdUnknown, score: 0.07
Park7Unknown, score: 0.06
Gigyf1Unknown, score: 0.26
ParvaUnknown, score: 0.39
PpbpUnknown, score: 0.1
Cramp1lUnknown, score: 0.03
Srd5a3Unknown, score: 0.08
Akip1Unknown, score: 0.2
MogsUnknown, score: 0.01
P2ry4Unknown, score: 0.05
Dnajc4Unknown, score: 0.34
Plin4Unknown, score: 0.21
Gabarapl1Unknown, score: 0.31
Golga7Unknown, score: 0.35
March7Unknown, score: 0.01
Tmem183aUnknown, score: 0.05
Ehd3Unknown, score: 0.29
GmnnUnknown, score: 0.01
Isg20Unknown, score: 0.05
Slc15a2Unknown, score: 0.01
Zfp112Unknown, score: 0.16
JmyUnknown, score: 0.09
Wdr12Unknown, score: 0.1
Rnf25Unknown, score: 0.42
Tacc2Unknown, score: 0.33
Ntn4Unknown, score: 0.03
Wdr4Unknown, score: 0.27
Fmnl1Unknown, score: 0.05
Fxyd7Unknown, score: 0.2
Cd200r1Unknown, score: 0
RangrfUnknown, score: 0.14
Kcne4Unknown, score: 0.14
Spata5Unknown, score: 0.19
TescUnknown, score: -0.01
Eral1Unknown, score: 0.29
Hacd3Unknown, score: 0.59
Ccdc126Unknown, score: 0.33
Krcc1Unknown, score: 0.14
Isy1Unknown, score: 0.01
GsdmaUnknown, score: 0.02
Crlf2Unknown, score: 0.21
Tnfrsf13bUnknown, score: 0.15
Sertad2Unknown, score: 0.08
Rgs20Unknown, score: 0.12
RhbgUnknown, score: 0.22
Sorcs1Unknown, score: 0.12
Rqcd1Unknown, score: 0.17
Rsad2Unknown, score: 0.44
Vstm2bUnknown, score: 0.01
Extl2Unknown, score: 0.28
Sall1Unknown, score: 0.06
NelfbUnknown, score: 0
Zbtb32Unknown, score: 0.04
Stk4Unknown, score: 0.05
Nudt11Unknown, score: 0.22
Gpr180Unknown, score: 0.01
1700123O20RikUnknown, score: 0.15
FibpUnknown, score: 0.31
Chst11Unknown, score: 0.07
BC100451Unknown, score: 0.17
0610007P14RikUnknown, score: 0.45
Trim54Unknown, score: 0.22
Elp2Unknown, score: 0.22
CrbnUnknown, score: 0.1
Trpm7Unknown, score: 0.02
1700027J19RikUnknown, score: 0
Slco1c1Unknown, score: 0.13
Cysltr1Unknown, score: 0.15
Pex5lUnknown, score: 0.14
Repin1Unknown, score: 0.41
Fam13aUnknown, score: 0.06
MyotUnknown, score: 0.04
Rps6kb2Unknown, score: 0.01
GhrlUnknown, score: 0.27
Arhgap23Unknown, score: 0.04
Pvrl3Unknown, score: 0.24
Pias4Unknown, score: -0.01
Trappc2lUnknown, score: 0.4
Sh3rf1Unknown, score: -0.01
Myoz1Unknown, score: 0.07
Moxd1Unknown, score: 0.21
Thap11Unknown, score: 0.12
Rab2aUnknown, score: 0.04
Med12Unknown, score: 0.11
Usp14Unknown, score: 0.12
Huwe1Unknown, score: 0.17
Rcl1Unknown, score: 0.23
Psmd14Unknown, score: 0.04
Chst12Unknown, score: 0.15
Carm1Unknown, score: 0.01
Dact1Unknown, score: 0.25
CopeUnknown, score: 0
Wsb2Unknown, score: 0.33
Stard3Unknown, score: 0.38
PnkpUnknown, score: 0.28
Slc22a17Unknown, score: 0.48
Nsa2Unknown, score: 0.34
Zfp191Unknown, score: 0
Tpm3Unknown, score: 0.22
Pcbp3Unknown, score: 0.1
Fxyd6Unknown, score: 0.27
NcstnUnknown, score: 0.3
Dctn5Unknown, score: 0.04
Ackr2Unknown, score: 0.11
Myg1Unknown, score: 0.12
Nrip2Unknown, score: 0.36
Ms4a4bUnknown, score: 0.07
Cldn15Unknown, score: 0.08
DonsonUnknown, score: 0.41
Rbm8aUnknown, score: 0.3
Trappc4Unknown, score: 0.22
CenpkUnknown, score: 0.52
Doc2gUnknown, score: 0.06
Iigp1Unknown, score: -0.01
Acss2Unknown, score: 0.14
Fads3Unknown, score: 0.11
Fignl1Unknown, score: 0.42
WtapUnknown, score: 0.18
Actn4Unknown, score: 0
Gucy1a3Unknown, score: 0.11
Mapk8ip2Unknown, score: -0.01
Trp53inp1Unknown, score: 0.05
Foxj2Unknown, score: 0.34
Bco1Unknown, score: 0.01
Trpv4Unknown, score: 0.3
Cables1Unknown, score: 0.1
Slc29a1Unknown, score: 0.18
GmfbUnknown, score: 0.07
GmfgUnknown, score: 0.09
Aqp9Unknown, score: 0.01
Syne1Unknown, score: 0.28
NrgnUnknown, score: 0.12
PerpUnknown, score: 0.36
Smoc2Unknown, score: 0.3
Smoc1Unknown, score: 0.15
Popdc2Unknown, score: 0.21
Mllt1Unknown, score: 0.32
St7Unknown, score: 0.28
Stk32bUnknown, score: 0.07
Abhd8Unknown, score: 0.2
Gprc5bUnknown, score: 0.01
Ms4a4cUnknown, score: 0.12
Sirt3Unknown, score: 0.31
Cyp4f14Unknown, score: 0.15
Inpp5eUnknown, score: 0.09
Tspan4Unknown, score: 0.17
NischUnknown, score: 0.13
Mrps22Unknown, score: 0.07
Mrps23Unknown, score: 0.21
Mrps10Unknown, score: 0.19
Mrps24Unknown, score: 0.38
NmiUnknown, score: 0.26
Htra2Unknown, score: 0.14
Scube1Unknown, score: 0.43
Suv39h2Unknown, score: 0.19
Lpin3Unknown, score: 0.26
Bhmt2Unknown, score: 0.04
Izumo1rUnknown, score: 0.1
Ap3m2Unknown, score: 0.09
Pes1Unknown, score: 0.11
Cldn12Unknown, score: 0.38
Rpl23Unknown, score: 0.24
Zfp110Unknown, score: 0.07
Lpar3Unknown, score: 0.09
Zfand6Unknown, score: 0.09
Arl6ip4Unknown, score: 0.12
Ndfip1Unknown, score: 0.25
Slc15a3Unknown, score: 0.09
Dpysl5Unknown, score: 0.23
Twsg1Unknown, score: 0.8
Utp3Unknown, score: 0.01
EefsecUnknown, score: 0.03
CubnUnknown, score: 0.2
Lima1Unknown, score: 0.11
Ifi30Unknown, score: 0.18
AsphUnknown, score: 0.13
Tmem254aUnknown, score: 0.07
Atp5dUnknown, score: 0.02
Dtd1Unknown, score: 0.07
Cndp2Unknown, score: 0.66
Zfp524Unknown, score: 0.11
Tctex1d2Unknown, score: 0.41
Gng11Unknown, score: 0.44
Gtpbp8Unknown, score: 0.17
SnupnUnknown, score: 0.4
Ethe1Unknown, score: 0.26
Sdhaf2Unknown, score: 0.45
Txndc12Unknown, score: 0.14
Tmem167Unknown, score: 0.33
Chchd3Unknown, score: 0.3
Abhd6Unknown, score: 0.3
Setd6Unknown, score: 0.19
Rmnd1Unknown, score: 0.02
FopnlUnknown, score: 0.15
GhitmUnknown, score: 0.21
Lamtor4Unknown, score: 0.17
Chchd6Unknown, score: 0.25
PpihUnknown, score: 0.04
Cxcl16Unknown, score: 0.33
Ube2d3Unknown, score: 0.05
Ndufa9Unknown, score: 0.14
Tmed3Unknown, score: 0.22
Marc1Unknown, score: 0.29
Apoa5Unknown, score: 0.07
Dnajc30Unknown, score: 0.27
1110001J03RikUnknown, score: 0.57
Sf3b5Unknown, score: 0.01
Mrps36Unknown, score: 0.39
Aaed1Unknown, score: 0.37
TipinUnknown, score: 0.32
Wbscr22Unknown, score: 0.17
Ska2Unknown, score: 0.01
Cox7bUnknown, score: 0.3
Atp6v1fUnknown, score: 0.21
Tmem57Unknown, score: 0.07
Dnajc15Unknown, score: 0.47
Uqcr10Unknown, score: 0.06
Cxx1aUnknown, score: 0.06
Mrpl4Unknown, score: 0.07
Nip7Unknown, score: 0.36
BccipUnknown, score: 0.7
Med11Unknown, score: 0.25
P3h4Unknown, score: 0.2
Nop10Unknown, score: 0.2
1110037F02RikUnknown, score: 0.28
Ier3ip1Unknown, score: 0.08
Lage3Unknown, score: 0.14
Myo19Unknown, score: 0.05
Commd4Unknown, score: 0.26
Commd6Unknown, score: 0.29
Vta1Unknown, score: 0.32
1110059G10RikUnknown, score: 0.13
Cd302Unknown, score: 0.11
NenfUnknown, score: 0.2
Sec61bUnknown, score: 0.71
Mrpl35Unknown, score: 0.27
LlphUnknown, score: 0.25
Mrps28Unknown, score: 0.62
Dmap1Unknown, score: 0.1
Eif1axUnknown, score: 0
Mrps16Unknown, score: 0.35
OsgepUnknown, score: 0.25
Alg5Unknown, score: 0.29
Pno1Unknown, score: 0.11
Arfgap3Unknown, score: 0.37
Aig1Unknown, score: 0.26
Dimt1Unknown, score: 0.1
Mrps17Unknown, score: 0.07
Camk2n1Unknown, score: 0.13
Tm4sf20Unknown, score: 0.03
Ccdc28bUnknown, score: 0.19
PigylUnknown, score: 0.32
Lyrm9Unknown, score: 0.18
Klf15Unknown, score: 0.07
Smim20Unknown, score: 0.11
Tmem218Unknown, score: 0.34
Tma16Unknown, score: 0.33
Atp6v1g1Unknown, score: 0.24
Fam3aUnknown, score: 0.16
Inafm1Unknown, score: 0.42
Fam53cUnknown, score: 0.16
Isoc1Unknown, score: 0.04
Tmem128Unknown, score: 0.17
Smurf2Unknown, score: 0.14
Tpd52l2Unknown, score: 0.41
Senp7Unknown, score: 0.13
Atp6v1c1Unknown, score: 0.61
CenppUnknown, score: 0.15
Cdrt4Unknown, score: 0.05
Atp5slUnknown, score: 0.03
Pla2g12aUnknown, score: 0.41
Riiad1Unknown, score: 0.6
Snw1Unknown, score: 0.06
GmprUnknown, score: 0.12
Knop1Unknown, score: 0
AdprmUnknown, score: 0.46
Cox20Unknown, score: 0.19
Exosc3Unknown, score: 0.35
Ergic3Unknown, score: 0.09
RtcaUnknown, score: 0.15
Dus2Unknown, score: 0.39
Chmp4cUnknown, score: 0.03
IscuUnknown, score: 0.4
Srp19Unknown, score: 0.23
CutcUnknown, score: 0
AhnakUnknown, score: 0.06
Ccdc82Unknown, score: 0.4
SlnUnknown, score: 0.21
Asf1aUnknown, score: 0.33
Sac3d1Unknown, score: 0.14
AptxUnknown, score: 0.54
Rsl1d1Unknown, score: 0.18
TbcbUnknown, score: 0.07
Arrdc4Unknown, score: 0.21
Psmd6Unknown, score: 0.18
Ndufa7Unknown, score: 0.05
2410004B18RikUnknown, score: 0.02
Oxld1Unknown, score: 0.12
Slc7a6osUnknown, score: 0.01
Chchd7Unknown, score: 0
Fis1Unknown, score: 0.05
Hamp2Unknown, score: 0.28
2010012O05RikUnknown, score: 0.22
Cdc26Unknown, score: 0.32
MagohbUnknown, score: 0.28
Spc25Unknown, score: 0.11
Nmnat1Unknown, score: 0.15
Cnpy4Unknown, score: 0.33
2810428I15RikUnknown, score: 0.24
Taf12Unknown, score: 0.3
Gtf2h5Unknown, score: 0.25
Ska1Unknown, score: 0.05
Fam213bUnknown, score: 0.21
Anp32eUnknown, score: 0.09
Rpl36alUnknown, score: 0.2
Polr2lUnknown, score: 0.59
Dda1Unknown, score: 0.08
Slc30a7Unknown, score: 0.15
Lamtor1Unknown, score: 0.53
Rnf181Unknown, score: 0.09
Asrgl1Unknown, score: 0.09
Cul7Unknown, score: 0.47
Pgpep1Unknown, score: 0.54
Timm50Unknown, score: 0.23
Ubxn6Unknown, score: 0.1
Rep15Unknown, score: 0.49
Nipsnap3bUnknown, score: 0.35
Rps19bp1Unknown, score: 0.02
Fam107bUnknown, score: 0.13
Aggf1Unknown, score: 0.29
Sppl2aUnknown, score: 0.19
Drap1Unknown, score: 0.14
NtpcrUnknown, score: 0.75
Rwdd3Unknown, score: 0.25
Gdpd1Unknown, score: 0.11
CenpmUnknown, score: 0.08
UqcrhUnknown, score: 0.14
Mis18aUnknown, score: 0.74
Esf1Unknown, score: 0.06
Snrnp40Unknown, score: 0.32
Crls1Unknown, score: 0.03
FastkUnknown, score: 0.26
FarsaUnknown, score: 0.02
Mad2l1bpUnknown, score: 0.01
Gtf3aUnknown, score: 0.06
Trim13Unknown, score: 0.44
Tmigd1Unknown, score: 0.03
Gemin2Unknown, score: 0.56
Lrrc57Unknown, score: 0.36
Ms4a4dUnknown, score: 0.2
Abi3Unknown, score: 0.01
Ormdl3Unknown, score: 0.51
Atg4bUnknown, score: 0.07
Ntmt1Unknown, score: 0.28
Ubr7Unknown, score: 0.35
Spcs2Unknown, score: 0.13
Ogfod2Unknown, score: 0.41
Dph6Unknown, score: 0.12
Tsen15Unknown, score: 0.01
Sike1Unknown, score: 0.15
Lix1Unknown, score: 0.14
Pspc1Unknown, score: 0.28
Ndnl2Unknown, score: 0.29
Acp6Unknown, score: 0.8
SltmUnknown, score: 0.16
Msantd3Unknown, score: 0.3
Hspbap1Unknown, score: 0.62
Spryd7Unknown, score: 0.33
Pgm1Unknown, score: 0.04
Trappc5Unknown, score: 0.62
Dcbld1Unknown, score: 0.11
Tmem186Unknown, score: 0.14
Uqcrfs1Unknown, score: 0.39
AspnUnknown, score: 0.18
Chmp3Unknown, score: 0.06
Ndufaf3Unknown, score: 0.27
NkaplUnknown, score: 0.6
SbdsUnknown, score: 0.21
Actr2Unknown, score: 0.24
4921524J17RikUnknown, score: 0.22
Ccdc96Unknown, score: 0.09
Klhl10Unknown, score: 0.02
Tab3Unknown, score: 0.18
Map1lc3aUnknown, score: 0.52
Rnf220Unknown, score: 0.16
Erich2Unknown, score: 0.45
Erlec1Unknown, score: 0.22
Tmem239Unknown, score: 0.23
PacrglUnknown, score: -0.01
Gid4Unknown, score: 0.03
Hacd4Unknown, score: 0.12
Grtp1Unknown, score: 0.05
Atg10Unknown, score: 0.48
Ube2wUnknown, score: 0.03
9030624G23RikUnknown, score: 0.18
Krt20Unknown, score: 0.15
Duoxa2Unknown, score: 0.04
PpcdcUnknown, score: 0.47
Ccdc109bUnknown, score: 0.09
PycardUnknown, score: 0
TazUnknown, score: 0.16
Rsph3aUnknown, score: 0.39
Acot13Unknown, score: 0.11
0610009O20RikUnknown, score: 0.18
Wdr45bUnknown, score: 0.29
Ormdl2Unknown, score: 0.11
Mrpl33Unknown, score: 0
Hint3Unknown, score: 0.26
Fuca2Unknown, score: 0
Trim35Unknown, score: 0.1
Plbd1Unknown, score: 0.34
Slc16a9Unknown, score: 0.11
Tanc1Unknown, score: 0.08
Lztr1Unknown, score: 0.15
Clec14aUnknown, score: 0.05
Nhlrc2Unknown, score: 0.28
Mfsd1Unknown, score: 0.31
Zfp869Unknown, score: 0.2
Serbp1Unknown, score: 0.15
TrilUnknown, score: 0.15
1200014J11RikUnknown, score: 0.32
Swt1Unknown, score: 0.13
Crnkl1Unknown, score: 0.13
Riok3Unknown, score: 0.18
Pcyox1Unknown, score: 0.15
Bzw1Unknown, score: 0.03
Appbp2Unknown, score: 0.08
AcadsbUnknown, score: 0.08
Pxdc1Unknown, score: 0.24
Naa16Unknown, score: 0.56
Baiap2l1Unknown, score: 0.6
ProzUnknown, score: 0.56
Tmem107Unknown, score: 0.01
Ndufb7Unknown, score: 0.07
Chordc1Unknown, score: 0.37
Prpf38bUnknown, score: 0.07
Rras2Unknown, score: 0.19
SdhdUnknown, score: 0.47
Rexo1Unknown, score: 0.11
Dsn1Unknown, score: 0.48
Shisa5Unknown, score: 0.14
SdhaUnknown, score: 0.07
Acad8Unknown, score: 0.17
Tmem206Unknown, score: 0
2310030G06RikUnknown, score: 0.21
Cdca7Unknown, score: 0.09
Tmx2Unknown, score: 0.03
Dusp26Unknown, score: 0.76
Swsap1Unknown, score: 0
Ctu2Unknown, score: 0.28
Trit1Unknown, score: 0.1
Edem3Unknown, score: 0.04
Plin5Unknown, score: 0.26
Ssbp2Unknown, score: 0.09
Mrps18bUnknown, score: 0.02
Trappc13Unknown, score: 0.44
Nuf2Unknown, score: 0.24
Pole4Unknown, score: 0.16
Zdhhc6Unknown, score: 0.41
Rassf7Unknown, score: 0.38
Zcchc18Unknown, score: 0.28
Psmd12Unknown, score: 0.16
Med28Unknown, score: 0.06
Cisd2Unknown, score: 0.54
Yae1d1Unknown, score: 0.01
Ttc23Unknown, score: 0.23
Rbm7Unknown, score: 0.21
Oma1Unknown, score: 0.02
MinaUnknown, score: 0
Tbc1d2bUnknown, score: 0.07
Fam210bUnknown, score: 0.19
Mkrn2Unknown, score: 0.19
2610002M06RikUnknown, score: 0.25
FanclUnknown, score: 0.27
Upf3aUnknown, score: 0.48
Dnajb4Unknown, score: 0.02
Pmf1Unknown, score: 0.05
2010109I03RikUnknown, score: 0.78
Oxct1Unknown, score: 0.03
Ift27Unknown, score: 0.38
Syap1Unknown, score: 0.17
Higd2aUnknown, score: 0.42
Tbc1d7Unknown, score: 0.13
Vma21Unknown, score: 0.11
Pus3Unknown, score: 0.34
NkapUnknown, score: 0.26
Rpp14Unknown, score: 0.01
PaicsUnknown, score: 0.25
Yaf2Unknown, score: 0.55
Tmem246Unknown, score: 0.19
2610002M06RikUnknown, score: 0.29
Dynlrb1Unknown, score: 0.2
Rps6ka6Unknown, score: 0.02
Cdc37l1Unknown, score: 0.07
Mon2Unknown, score: 0.22
Magt1Unknown, score: 0.11
PgpUnknown, score: 0.02
1700019D03RikUnknown, score: 0.15
Cand2Unknown, score: 0.1
Trappc6aUnknown, score: 0.02
Trak1Unknown, score: 0.28
Rps10Unknown, score: 0.04
D16Ertd472eUnknown, score: 0.05
Ptgr1Unknown, score: 0.25
Rpl14Unknown, score: 0.09
Cuedc2Unknown, score: 0.22
BfarUnknown, score: 0.13
Ccdc159Unknown, score: 0.37
Ttc14Unknown, score: 0.11
MastlUnknown, score: 0.39
NrarpUnknown, score: 0.15
Atp5eUnknown, score: 0.71
Ndufa6Unknown, score: 0.24
Acbd4Unknown, score: 0.15
Kbtbd4Unknown, score: 0.29
Herc6Unknown, score: 0.1
Mis12Unknown, score: 0.13
Fbxo5Unknown, score: 0.12
Ikzf5Unknown, score: 0.14
Fam103a1Unknown, score: 0.84
Psmd9Unknown, score: 0.17
Rnaseh2bUnknown, score: 0.4
MtdhUnknown, score: 0.37
Smarca2Unknown, score: 0.05
2610301B20RikUnknown, score: 0.18
Sft2d3Unknown, score: 0.13
Eef1gUnknown, score: 0.26
Sclt1Unknown, score: 0.21
Lipt2Unknown, score: 0.51
Arl8bUnknown, score: 0.12
NraddUnknown, score: 0.58
Cdt1Unknown, score: 0.15
Zmat5Unknown, score: 0.18
Ccdc25Unknown, score: 0
Yipf5Unknown, score: 0.23
Ctdnep1Unknown, score: 0.01
Ube2tUnknown, score: 0.35
Zcrb1Unknown, score: 0.11
Pfdn1Unknown, score: 0.25
Ccdc77Unknown, score: 0.3
Nde1Unknown, score: 0.82
Eif2s2Unknown, score: 0.61
Utp11lUnknown, score: 0.11
Lsm1Unknown, score: 0.16
Gatad1Unknown, score: 0.27
Mboat2Unknown, score: 0.06
Med18Unknown, score: 0.22
Plekho1Unknown, score: 0.07
Srfbp1Unknown, score: 0.08
Med29Unknown, score: 0.43
Rnpc3Unknown, score: 0.02
Dph7Unknown, score: 0.6
Prpf18Unknown, score: 0.29
Zkscan14Unknown, score: 0.25
CinpUnknown, score: 0.82
Rpf2Unknown, score: 0.46
Peli1Unknown, score: 0.52
Rpl39Unknown, score: 0.39
Tbc1d19Unknown, score: 0.03
Cap2Unknown, score: 0.51
Cers4Unknown, score: 0.3
Uqcc2Unknown, score: 0.22
Agtpbp1Unknown, score: 0.07
Mrpl42Unknown, score: 0.03
Cmtm5Unknown, score: 0.09
Ndufa10Unknown, score: 0.33
Eri1Unknown, score: 0.28
Rpl37Unknown, score: 0.25
Slc25a19Unknown, score: 0.51
Ift22Unknown, score: 0.24
Parp6Unknown, score: 0.32
3110040N11RikUnknown, score: 0.22
PigcUnknown, score: 0.25
Rab3cUnknown, score: 0.49
Socs4Unknown, score: 0.22
Gprasp1Unknown, score: 0.07
Dock7Unknown, score: 0.1
CltcUnknown, score: 0.56
Gpx7Unknown, score: 0.12
Pbld2Unknown, score: 0.07
Mrpl46Unknown, score: 0.17
NanpUnknown, score: 0.05
1700037H04RikUnknown, score: 0.28
Stk35Unknown, score: 0.1
Herc4Unknown, score: 0.03
QpctlUnknown, score: 0.21
Zfp606Unknown, score: 0.14
Gtf3c6Unknown, score: 0.48
Jam2Unknown, score: 0.06
QprtUnknown, score: 0.26
Dedd2Unknown, score: 0.26
Brd3Unknown, score: 0.25
Rnmtl1Unknown, score: 0.03
Erp29Unknown, score: 0.06
NtsUnknown, score: 0.01
Mfn1Unknown, score: 0.03
Ears2Unknown, score: 0.14
3632451O06RikUnknown, score: 0.24
DhddsUnknown, score: 0.14
Rps20Unknown, score: 0.11
4921536K21RikUnknown, score: 0.04
Hoga1Unknown, score: 0.35
Ccdc127Unknown, score: 0.05
Ankrd33bUnknown, score: 0.44
Xab2Unknown, score: 0.28
Isoc2bUnknown, score: 0.1
RetsatUnknown, score: 0.01
Map1lc3bUnknown, score: 0.14
Dusp28Unknown, score: 0.14
Plxdc2Unknown, score: 0.27
Pkp2Unknown, score: 0.19
Pnpla8Unknown, score: 0.44
Slc25a46Unknown, score: 0.2
IkbipUnknown, score: 0
Klhl13Unknown, score: 0.44
Ergic1Unknown, score: 0.32
NvlUnknown, score: 0.58
Sf3a1Unknown, score: 0.15
PdclUnknown, score: 0.1
Gpalpp1Unknown, score: 0.24
MmdUnknown, score: 0.05
Abhd5Unknown, score: 0.62
Mtfr1Unknown, score: 0.43
Slc47a1Unknown, score: 0.42
Snap29Unknown, score: 0.19
Abhd15Unknown, score: 0.16
Cwc25Unknown, score: 0.1
Eepd1Unknown, score: 0.59
Polr3gUnknown, score: 0.18
Dhx40Unknown, score: 0.28
Calcoco1Unknown, score: 0.1
Ap4b1Unknown, score: 0.16
Zfand4Unknown, score: 0.14
Mettl16Unknown, score: 0.41
Tmem167bUnknown, score: 0.16
Ccar1Unknown, score: 0.18
Ccdc50Unknown, score: 0.23
Tmed9Unknown, score: 0.45
2610002J02RikUnknown, score: 0.23
Ttc33Unknown, score: 0.18
Trim62Unknown, score: 0.38
Nudt7Unknown, score: 0.22
Fgfr1op2Unknown, score: 0.24
Mfap1aUnknown, score: 0.21
Ppfibp1Unknown, score: 0.18
Ttll4Unknown, score: 0.17
Cog6Unknown, score: 0.24
Fam120bUnknown, score: 0.1
Slc39a8Unknown, score: 0.16
Gpr89Unknown, score: 0.02
Slc25a30Unknown, score: 0.13
PigmUnknown, score: 0.02
Loxl4Unknown, score: 0.17
Alg13Unknown, score: 0.25
Cpeb4Unknown, score: 0.19
Lrrc18Unknown, score: 0.26
Rnf41Unknown, score: 0.04
Tespa1Unknown, score: 0.11
Akt1s1Unknown, score: 0.05
FibinUnknown, score: 0.15
NarfUnknown, score: 0.23
4930453N24RikUnknown, score: 0.44
Rspry1Unknown, score: 0.09
Ube2r2Unknown, score: 0.62
Lrp2bpUnknown, score: 0.39
Bend5Unknown, score: 0.45
Tm7sf3Unknown, score: 0.59
Spc24Unknown, score: 0.22
Samd8Unknown, score: 0.37
Lyrm5Unknown, score: 0.37
4930523C07RikUnknown, score: 0.1
Ctdp1Unknown, score: 0.16
4930548H24RikUnknown, score: -0.01
Ift172Unknown, score: 0.01
Rnf125Unknown, score: 0.38
Dctn4Unknown, score: 0.04
Hapln3Unknown, score: 0.2
Alkbh8Unknown, score: 0.33
Rpp21Unknown, score: 0
SdhbUnknown, score: 0.1
Mrpl18Unknown, score: 0.25
Pbdc1Unknown, score: 0.46
Aldh3b1Unknown, score: 0.06
HypkUnknown, score: 0.51
Fam174aUnknown, score: 0.29
Kirrel3Unknown, score: 0.05
Taf6lUnknown, score: 0.22
Mrpl24Unknown, score: 0.17
Pcnxl4Unknown, score: 0.1
Polr2gUnknown, score: 0.12
Nsmce1Unknown, score: 0.18
Slc25a37Unknown, score: 0.12
Dnajc19Unknown, score: 0.05
Pop1Unknown, score: 0.13
Nudt13Unknown, score: 0.05
Fam114a2Unknown, score: 0.4
Iah1Unknown, score: 0.16
Itgb3bpUnknown, score: 0.33
Ccdc130Unknown, score: 0.19
Ttc39dUnknown, score: 0.8
Slc48a1Unknown, score: 0.14
Ribc2Unknown, score: 0.18
Ppp1r32Unknown, score: 0.02
EqtnUnknown, score: 0.09
Ddx47Unknown, score: 0.32
PlgrktUnknown, score: 0
Jagn1Unknown, score: 0.67
N6amt1Unknown, score: 0
Gpatch2Unknown, score: 0.58
Arpc5Unknown, score: 0.05
Kat8Unknown, score: 0.13
Loh12cr1Unknown, score: 0.18
Rtp4Unknown, score: 0.01
Vwa5aUnknown, score: 0.23
Zfp639Unknown, score: 0.24
Plxnd1Unknown, score: 0.17
Zmym4Unknown, score: 0.39
Dalrd3Unknown, score: 0
RnlsUnknown, score: 0.04
Snrnp48Unknown, score: 0.17
PllpUnknown, score: 0.23
Limd2Unknown, score: 0.33
TprglUnknown, score: 0.4
Poldip2Unknown, score: 0.09
Ubxn4Unknown, score: 0.17
Derl1Unknown, score: 0.24
Atp1b4Unknown, score: 0.34
Rer1Unknown, score: 0.85
Brix1Unknown, score: 0.02
Wdr83Unknown, score: 0.2
Mrpl57Unknown, score: 0.71
Slc35a4Unknown, score: 0.17
Rab32Unknown, score: 0.05
Rnf115Unknown, score: 0.08
Tmem39aUnknown, score: 0.51
SncaipUnknown, score: 0.03
Ddx55Unknown, score: 0.14
Cdca5Unknown, score: 0.09
Echdc3Unknown, score: 0.3
S100a16Unknown, score: 0.43
Akr1b10Unknown, score: 0.29
Slc25a11Unknown, score: 0.27
Yipf4Unknown, score: 0.43
Lrrc28Unknown, score: 0.4
Enoph1Unknown, score: 0.19
Mri1Unknown, score: 0.1
Naa20Unknown, score: 0.2
DcxrUnknown, score: 0.05
1810043G02RikUnknown, score: 0.37
Camsap2Unknown, score: 0.03
SarafUnknown, score: 0.04
Tmem100Unknown, score: 0.09
Rbm18Unknown, score: 0.54
Ufm1Unknown, score: 0.24
Tmem86aUnknown, score: 0.53
Ccdc80Unknown, score: 0.12
Pef1Unknown, score: 0.16
Zfp169Unknown, score: 0.24
Coq9Unknown, score: 0.23
Ppap2bUnknown, score: 0.12
Zcchc3Unknown, score: 0.29
Mak16Unknown, score: 0.1
Hcfc2Unknown, score: 0.33
Wdr55Unknown, score: 0.24
Myl12bUnknown, score: 0.06
Prorsd1Unknown, score: 0.19
Rpl41Unknown, score: 0.3
Fbxo28Unknown, score: 0.31
Tubb6Unknown, score: 0.22
Sugt1Unknown, score: 0.34
Setd8Unknown, score: 0.37
Puf60Unknown, score: 0.04
Npc2Unknown, score: 0.24
Zcchc10Unknown, score: 0.47
Tppp3Unknown, score: 0.16
Atp2b1Unknown, score: 0.41
Mphosph10Unknown, score: 0
67978Unknown, score: 0.2
Gnpda2Unknown, score: 0.22
Pdzd9Unknown, score: 0.44
Nacc2Unknown, score: 0.15
Srsf6Unknown, score: 0.12
Fam134cUnknown, score: 0.08
SnrpgUnknown, score: 0.34
ZwilchUnknown, score: 0.1
Trap1Unknown, score: 0.56
Ftsj2Unknown, score: 0.12
BphlUnknown, score: 0.01
Hist1h2bcUnknown, score: 0.32
Rpl22l1Unknown, score: 0.37
Emc4Unknown, score: 0.05
Cox19Unknown, score: 0.52
Chid1Unknown, score: 0.14
Mid1ip1Unknown, score: 0.04
Chac2Unknown, score: 0.25
Akirin1Unknown, score: 0.28
Rps13Unknown, score: 0.31
Ubxn2bUnknown, score: 0.24
Serpina12Unknown, score: 0.29
Atp5sUnknown, score: 0.2
Tm9sf2Unknown, score: 0.11
Slc25a39Unknown, score: 0
Pdzd2Unknown, score: 0.06
Gpn3Unknown, score: 0.14
Dusp19Unknown, score: 0.04
Pak1ip1Unknown, score: 0
Arpc4Unknown, score: 0.02
Yif1aUnknown, score: 0.39
Ncbp2Unknown, score: 0.02
Smarcc2Unknown, score: 0.35
Dynll2Unknown, score: 0.11
Rchy1Unknown, score: 0.51
Sdccag3Unknown, score: -0.01
Mum1Unknown, score: 0.29
Atg101Unknown, score: 0.09
Cmtm3Unknown, score: 0.64
Fahd2aUnknown, score: 0.74
GcshUnknown, score: 0.36
Upf3bUnknown, score: 0.18
Kdelr1Unknown, score: 0.27
Ino80Unknown, score: 0.47
Etaa1Unknown, score: 0.08
Arl13bUnknown, score: 0.19
WlsUnknown, score: 0.19
Fam133bUnknown, score: 0.37
Stx19Unknown, score: 0.39
Fdx1lUnknown, score: 0.22
Spire1Unknown, score: 0.25
NdnfUnknown, score: 0.53
Rpl39lUnknown, score: 0.03
Fam212aUnknown, score: 0.31
EbplUnknown, score: 0.3
HyiUnknown, score: 0.2
Bcas2Unknown, score: 0.51
DenrUnknown, score: 0.16
Coa4Unknown, score: 0.29
Leprotl1Unknown, score: 0.05
Ndufb4Unknown, score: 0.37
Ndufb2Unknown, score: 0.63
Ccdc34Unknown, score: 0.13
Diras2Unknown, score: 0.21
Gsto2Unknown, score: 0.65
Nudt21Unknown, score: 0.24
AI846148Unknown, score: 0.02
MturnUnknown, score: 0.07
A930018P22RikUnknown, score: 0.22
Tmem86bUnknown, score: 0.19
Ift80Unknown, score: 0.17
Agpat4Unknown, score: 0.25
PdhbUnknown, score: 0.04
Slc25a22Unknown, score: 0.16
Zdhhc21Unknown, score: 0.17
Rbm28Unknown, score: 0.04
Mcoln2Unknown, score: 0.42
Gm12666Unknown, score: 0.24
9530077C05RikUnknown, score: 0.06
Mto1Unknown, score: 0.19
Ncapd2Unknown, score: 0.15
Vps53Unknown, score: 0.31
Fam114a1Unknown, score: 0.45
Kdelc2Unknown, score: 0.26
Gstm7Unknown, score: 0.08
ApooUnknown, score: 0.09
Tsr3Unknown, score: 0
Rab13Unknown, score: 0.23
Crip2Unknown, score: 0.21
Ccdc88cUnknown, score: 0.02
Ndufb10Unknown, score: 0.04
AspdhUnknown, score: 0.53
Tmem129Unknown, score: 0.13
Mogat1Unknown, score: 0.1
SycnUnknown, score: 0.1
Fbxl15Unknown, score: 0.1
Rpl34Unknown, score: 0.08
Tbc1d10bUnknown, score: 0.3
Ppp1r14aUnknown, score: 0.1
Mrpl14Unknown, score: 0.35
Adipor2Unknown, score: 0.29
Tmem126bUnknown, score: 0.22
Mpzl1Unknown, score: 0.06
Zfp579Unknown, score: 0.22
Ndufaf4Unknown, score: 0.13
Mrpl53Unknown, score: 0.08
Vps51Unknown, score: 0.13
Ppfia4Unknown, score: 0.07
Dcdc2cUnknown, score: 0.3
Tomm5Unknown, score: 0.71
Evc2Unknown, score: 0.43
Gpr155Unknown, score: 0.55
Mphosph6Unknown, score: 0.17
2310036O22RikUnknown, score: 0.05
EcscrUnknown, score: 0.48
Sgol2aUnknown, score: 0.12
TefmUnknown, score: 0.08
Smim14Unknown, score: 0.11
Col6a4Unknown, score: 0.44
Uckl1Unknown, score: 0.02
Dpm3Unknown, score: 0.13
CalyUnknown, score: 0.34
Tmed10Unknown, score: 0.12
Rtn4Unknown, score: 0.07
Cthrc1Unknown, score: 0.22
MocosUnknown, score: 0.32
Syf2Unknown, score: 0.05
Ccdc167Unknown, score: 0.34
Mtcl1Unknown, score: 0.17
Fbxw9Unknown, score: 0.2
Cryl1Unknown, score: 0.14
Tm2d3Unknown, score: 0.28
Nadk2Unknown, score: 0.14
Fndc1Unknown, score: 0.31
Pcyt2Unknown, score: 0.29
Slc44a2Unknown, score: 0.65
Kansl1lUnknown, score: 0.34
RereUnknown, score: 0.12
Gtf2f2Unknown, score: 0.39
1110032A03RikUnknown, score: 0.44
Arl8aUnknown, score: 0.12
Trp53inp2Unknown, score: 0.13
Lrrc16aUnknown, score: 0.55
Mrps18cUnknown, score: 0.34
Angel1Unknown, score: 0.17
Tmem219Unknown, score: 0.42
Rreb1Unknown, score: 0.35
Abhd11Unknown, score: 0.09
Phtf2Unknown, score: 0.05
Ms4a6dUnknown, score: 0.02
Tmem214Unknown, score: 0.04
PdgfrlUnknown, score: -0.01
Elovl5Unknown, score: 0.01
MypnUnknown, score: 0.64
Dock5Unknown, score: 0.12
Zfand2bUnknown, score: 0.05
SyncUnknown, score: 0.1
1110057K04RikUnknown, score: 0.45
Ankrd46Unknown, score: 0.14
Tulp4Unknown, score: 0.06
Rnf208Unknown, score: 0.53
Lrrn4clUnknown, score: 0.02
Asb11Unknown, score: 0.11
Dtwd2Unknown, score: 0.36
Arv1Unknown, score: 0.02
Maf1Unknown, score: 0.17
Prpf6Unknown, score: 0.04
Vars2Unknown, score: 0.39
1110065P20RikUnknown, score: 0.57
Rpap1Unknown, score: 0.01
Aspscr1Unknown, score: 0.25
Rasl11bUnknown, score: 0.44
Pink1Unknown, score: -0.01
Chst8Unknown, score: 0.32
Fam57bUnknown, score: 0.03
Chmp2aUnknown, score: 0.54
Ctc1Unknown, score: 0.09
Dcaf12Unknown, score: 0.1
Tamm41Unknown, score: 0.22
HaghlUnknown, score: 0.15
Nol11Unknown, score: 0.19
Snrpa1Unknown, score: 0.16
Prpf31Unknown, score: 0.04
Zfp580Unknown, score: 0.48
Mcts1Unknown, score: 0.35
Anapc10Unknown, score: 0.24
Prrt2Unknown, score: 0.02
Snx15Unknown, score: 0.34
Mitd1Unknown, score: 0.14
Smdt1Unknown, score: 0.1
Lyzl4Unknown, score: 0.09
Zdhhc3Unknown, score: 0.05
Tmem258Unknown, score: 0.02
Isca1Unknown, score: 0.21
Pycr2Unknown, score: -0.01
Chac1Unknown, score: 0.02
1810011H11RikUnknown, score: 0.09
Tmem97Unknown, score: 0.13
Ebna1bp2Unknown, score: 0.1
Triap1Unknown, score: 0.23
GmppaUnknown, score: 0.43
Zc3h15Unknown, score: -0.01
Zcchc9Unknown, score: -0.01
Ascc1Unknown, score: 0.06
YdjcUnknown, score: 0.1
March5Unknown, score: 0.24
Fam58bUnknown, score: 0.03
Cnot8Unknown, score: 0.37
Pex11gUnknown, score: 0.15
Cdk12Unknown, score: 0.2
2200002J24RikUnknown, score: 0.18
Kbtbd3Unknown, score: 0.27
LzicUnknown, score: 0.07
Bola1Unknown, score: 0.07
C1qtnf2Unknown, score: 0.23
Tmem256Unknown, score: -0.01
Erp27Unknown, score: 0.18
DymUnknown, score: 0.05
Dhx16Unknown, score: -0.01
PtmsUnknown, score: 0.29
Srsf11Unknown, score: 0.03
Sat2Unknown, score: 0.39
Plekha4Unknown, score: 0.01
CarkdUnknown, score: 0
Snx24Unknown, score: 0.17
SeltUnknown, score: 0.07
Zfp688Unknown, score: 0.67
Kctd5Unknown, score: 0.15
Ing2Unknown, score: 0.05
Rfc3Unknown, score: 0.17
Gins1Unknown, score: 0.19
CtdsplUnknown, score: 0.23
Sec62Unknown, score: 0.11
Lrrc46Unknown, score: 0.14
TesclUnknown, score: 0.06
DcpsUnknown, score: 0.14
Ppp1r42Unknown, score: 0.09
1700008O03RikUnknown, score: 0.29
Necab1Unknown, score: 0.03
Slc38a4Unknown, score: 0.04
Lrrc51Unknown, score: 0.25
Wdfy1Unknown, score: 0.27
Dnajb13Unknown, score: 0.01
Plac8l1Unknown, score: 0.36
Dnajc17Unknown, score: 0.23
Clic3Unknown, score: 0.43
Commd10Unknown, score: 0.17
Ubl7Unknown, score: 0.07
Tmco2Unknown, score: 0.16
2300009A05RikUnknown, score: 0.33
Ttc9Unknown, score: 0.03
Nup35Unknown, score: 0.03
Ndufaf5Unknown, score: 0.11
Tsr2Unknown, score: 0.18
Zfp932Unknown, score: 0.05
Rwdd2aUnknown, score: 0.03
Mrps9Unknown, score: 0.18
Dnase1l1Unknown, score: 0.09
Klk10Unknown, score: 0.55
Wdr5bUnknown, score: 0.27
Mapk1ip1Unknown, score: 0.13
Fam65cUnknown, score: 0.33
Klhdc2Unknown, score: 0.09
Bod1Unknown, score: 0.11
Nmrk2Unknown, score: 0.06
Mfsd3Unknown, score: 0.28
Hfe2Unknown, score: 0.02
Pcgf3Unknown, score: 0.54
Ap5s1Unknown, score: 0.08
Dab2ipUnknown, score: 0.02
LnpUnknown, score: 0.3
Kansl2Unknown, score: 0.24
ClyblUnknown, score: 0.16
Exosc8Unknown, score: -0.01
Wdr20Unknown, score: 0.31
Dctn2Unknown, score: 0.16
Cd164l2Unknown, score: 0.07
PirUnknown, score: -0.01
Psmg4Unknown, score: 0.2
Tmem52Unknown, score: -0.01
Mif4gdUnknown, score: 0.03
Emc10Unknown, score: 0.11
Hddc2Unknown, score: 0.08
Camsap3Unknown, score: 0.36
Slc52a3Unknown, score: 0.3
Col22a1Unknown, score: 0.32
Ndufaf1Unknown, score: 0.1
Arap1Unknown, score: 0.1
Pin4Unknown, score: 0.22
TfptUnknown, score: 0.34
Trip13Unknown, score: 0.09
IpmkUnknown, score: 0.08
CadUnknown, score: 0.41
Nkiras1Unknown, score: 0.07
Rnaseh2aUnknown, score: 0.07
Smyd3Unknown, score: 0.03
Gemin7Unknown, score: 0.18
Nup37Unknown, score: 0.05
TtlUnknown, score: 0.25
Aldh16a1Unknown, score: 0.03
Fbxo7Unknown, score: 0.59
Leng1Unknown, score: 0.24
Tnfaip8l2Unknown, score: 0.14
1810026J23RikUnknown, score: 0.09
Ms4a6bUnknown, score: 0.35
Smap2Unknown, score: 0.46
1500009L16RikUnknown, score: 0.17
TprkbUnknown, score: 0.23
Med30Unknown, score: 0.12
Trim32Unknown, score: 0.16
Mzb1Unknown, score: -0.01
Mterf4Unknown, score: 0.26
Glod5Unknown, score: 0.16
Rab43Unknown, score: 0.09
Pla2g12bUnknown, score: 0.07
Pcgf1Unknown, score: 0.06
Ttc39bUnknown, score: 0.24
A1cfUnknown, score: 0.41
SnrpfUnknown, score: 0.4
Vwa9Unknown, score: 0.1
Coa7Unknown, score: 0.47
Mfsd11Unknown, score: 0.16
Rasip1Unknown, score: 0.08
Rab3bUnknown, score: 0.21
Nup43Unknown, score: 0.31
Polr2iUnknown, score: 0.47
Vrk2Unknown, score: 0.09
Dnah17Unknown, score: 0.06
Apitd1Unknown, score: 0.17
Zfp715Unknown, score: 0.52
Trmt10bUnknown, score: 0.16
Exoc1Unknown, score: 0.01
Rnf113a1Unknown, score: 0.08
Fars2Unknown, score: 0.14
Rpp25lUnknown, score: 0.29
Galk2Unknown, score: 0.36
1700026L06RikUnknown, score: 0.31
Ino80bUnknown, score: 0.1
Nt5dc2Unknown, score: 0
Tut1Unknown, score: 0.19
Trnt1Unknown, score: 0.12
Prpf4Unknown, score: 0.18
Degs2Unknown, score: 0.15
Nol7Unknown, score: 0.04
Igsf23Unknown, score: 0.28
Meaf6Unknown, score: 0.19
Ube2q1Unknown, score: 0.12
Sash1Unknown, score: 0.69
Smc4Unknown, score: 0.14
Odf3bUnknown, score: 0.02
Yars2Unknown, score: 0.23
Dpf3Unknown, score: 0.03
2210011C24RikUnknown, score: 0.16
Lrch3Unknown, score: 0.21
Mettl7a1Unknown, score: 0.12
2210016F16RikUnknown, score: 0.54
Ogfrl1Unknown, score: 0.51
Tmem143Unknown, score: 0.01
Kif18bUnknown, score: 0.18
NarsUnknown, score: 0.03
Ppil3Unknown, score: 0.06
Gorasp2Unknown, score: 0.16
Cd2bp2Unknown, score: 0.2
Poc1aUnknown, score: 0.56
Bhlhb9Unknown, score: 0.19
Ufsp1Unknown, score: 0.37
2010107E04RikUnknown, score: 0.13
Ccbl1Unknown, score: 0.37
Mkrn2osUnknown, score: 0.45
Tbc1d13Unknown, score: 0.39
Rabep2Unknown, score: 0.1
Ndufab1Unknown, score: 0.1
Arl16Unknown, score: 0.23
PigwUnknown, score: 0.45
Cd3eapUnknown, score: 0.3
Ube2cbpUnknown, score: 0.24
Basp1Unknown, score: 0.07
Ppp4r1Unknown, score: 0.13
Secisbp2lUnknown, score: 0.06
St13Unknown, score: 0.24
Kcnip1Unknown, score: 0.12
Gtpbp3Unknown, score: 0.06
Bag5Unknown, score: 0.19
Derl3Unknown, score: 0.36
Mospd1Unknown, score: 0.29
Tecpr1Unknown, score: 0.04
Spdl1Unknown, score: 0.03
Ttc9cUnknown, score: 0.2
KptnUnknown, score: 0
Asnsd1Unknown, score: 0.01
Calml3Unknown, score: 0.21
Polr3fUnknown, score: 0.23
Stk26Unknown, score: 0.2
Megf10Unknown, score: 0.02
2810408A11RikUnknown, score: 0.07
2610034B18RikUnknown, score: 0.02
Ints2Unknown, score: 0.17
Tspan15Unknown, score: 0.39
Mier2Unknown, score: 0.18
Polr3bUnknown, score: 0.07
TbceUnknown, score: 0.38
Inf2Unknown, score: 0.35
Cd248Unknown, score: 0.14
Dhrs13Unknown, score: 0.24
Mpc2Unknown, score: -0.01
Crtc3Unknown, score: 0.01
Wdr77Unknown, score: 0.2
Rprd1bUnknown, score: 0.52
Pnma1Unknown, score: 0.06
Slc35d2Unknown, score: 0.03
Arhgap17Unknown, score: 0.35
DdoUnknown, score: 0.34
BbxUnknown, score: 0
Eef2kmtUnknown, score: 0.23
Lrfn2Unknown, score: 0.31
QpctUnknown, score: 0.02
Tmem242Unknown, score: 0.33
Slc25a33Unknown, score: 0.17
Wars2Unknown, score: 0.38
Txndc16Unknown, score: 0.27
Fam213aUnknown, score: 0.26
Fra10ac1Unknown, score: 0.19
Gfod2Unknown, score: 0.46
Pak4Unknown, score: 0.08
5730455P16RikUnknown, score: 0.08
Ssfa2Unknown, score: 0.17
MutyhUnknown, score: 0.32
Dnajb14Unknown, score: 0.27
Zdhhc24Unknown, score: 0.06
Ankrd24Unknown, score: 0.33
Sugp1Unknown, score: 0.24
5730508B09RikUnknown, score: 0.4
Med26Unknown, score: 0.07
Dcp2Unknown, score: 0.15
Oip5Unknown, score: 0.12
Naa30Unknown, score: 0.14
Zcchc8Unknown, score: 0.21
Prdm16Unknown, score: 0.09
Vcpip1Unknown, score: 0.55
Gulp1Unknown, score: 0.3
Dusp16Unknown, score: 0.45
Adgra3Unknown, score: 0
Gpr137cUnknown, score: 0.02
Angptl6Unknown, score: 0
Rasgef1aUnknown, score: 0.21
6330409D20RikUnknown, score: 0.19
CgnUnknown, score: 0.38
Tspan2Unknown, score: -0.01
KdsrUnknown, score: 0.21
Hacd2Unknown, score: 0.13
Dclk2Unknown, score: 0.29
Prpf3Unknown, score: 0.18
Nolc1Unknown, score: 0.02
Ggnbp1Unknown, score: 0.34
Prdm5Unknown, score: 0.23
Rasl12Unknown, score: 0.04
Klhl30Unknown, score: 0.13
KynuUnknown, score: 0.02
Zdhhc1Unknown, score: 0.1
Ankib1Unknown, score: 0.18
Cep192Unknown, score: 0.5
Clec2gUnknown, score: 0.46
Krt25Unknown, score: 0.12
4921507P07RikUnknown, score: 0.18
Ccdc93Unknown, score: 0.02
Spag9Unknown, score: 0.25
Ttll7Unknown, score: 0.04
Lpcat2bUnknown, score: 0.05
Nsun7Unknown, score: 0.04
Cdkn2aipUnknown, score: -0.01
Trim69Unknown, score: 0.44
Nol8Unknown, score: 0.25
4921539E11RikUnknown, score: 0.31
Mmrn1Unknown, score: 0.58
Eva1cUnknown, score: 0.25
Pgm2l1Unknown, score: 0.46
Spef1Unknown, score: 0.39
Phf6Unknown, score: 0.16
Prss41Unknown, score: 0.45
D7Ertd443eUnknown, score: 0.08
Prss55Unknown, score: 0.11
Hexim2Unknown, score: 0.25
Zfp597Unknown, score: 0.23
Stox2Unknown, score: 0
Arhgap19Unknown, score: 0.19
Atoh8Unknown, score: 0.19
Tssk4Unknown, score: 0.25
UvssaUnknown, score: 0.17
OxsmUnknown, score: 0.43
4933413G19RikUnknown, score: 0.02
Eri2Unknown, score: 0.07
NbasUnknown, score: 0.18
AsunUnknown, score: 0.44
Clec12bUnknown, score: 0
Nudt4Unknown, score: 0
Gpr15Unknown, score: 0.01
EnkurUnknown, score: 0.3
Osbpl7Unknown, score: 0.38
Naif1Unknown, score: 0.29
Lrrfip2Unknown, score: 0.2
Noxred1Unknown, score: 0.03
Arhgap26Unknown, score: 0.34
Rassf8Unknown, score: 0.08
Tchhl1Unknown, score: 0.02
Treml1Unknown, score: 0.17
RbksUnknown, score: 0.57
Riok1Unknown, score: 0.67
Wdr31Unknown, score: 0.15
Col24a1Unknown, score: 0.01
Arid5bUnknown, score: 0.05
Pex1Unknown, score: 0.06
Chd6Unknown, score: 0.18
Kctd6Unknown, score: 0.16
5430419D17RikUnknown, score: 0.23
5430427O19RikUnknown, score: 0.42
Fmnl2Unknown, score: 0.1
AmtnUnknown, score: 0.01
Arhgap21Unknown, score: 0.06
Tmem80Unknown, score: 0.01
Mettl13Unknown, score: 0.08
Ppp6r2Unknown, score: 0.36
Alpk1Unknown, score: 0.31
Bbs7Unknown, score: 0.55
Zfp935Unknown, score: 0.14
SfpqUnknown, score: 0.03
Cyp2u1Unknown, score: 0.23
GrapUnknown, score: 0.12
KaznUnknown, score: 0.47
Fbxo9Unknown, score: 0.07
Arhgap42Unknown, score: 0.21
ClmpUnknown, score: 0.2
9130008F23RikUnknown, score: 0.04
Gdpd2Unknown, score: 0.26
Ifih1Unknown, score: 0.02
PogkUnknown, score: 0.1
Senp8Unknown, score: 0.41
Zfp949Unknown, score: 0.38
Zgrf1Unknown, score: 0.58
OptnUnknown, score: 0
Rarres2Unknown, score: 0.3
Fuca1Unknown, score: 0.04
Tmem248Unknown, score: 0.06
Rnf215Unknown, score: 0.13
0610010F05RikUnknown, score: 0.44
BroxUnknown, score: 0.01
GypcUnknown, score: 0.39
Tmem25Unknown, score: 0.54
Slc41a3Unknown, score: 0.15
Pnpt1Unknown, score: 0.48
Cdc5lUnknown, score: 0.02
Armcx3Unknown, score: 0.04
Ubiad1Unknown, score: 0.11
Syde1Unknown, score: 0.3
Mus81Unknown, score: 0.06
Dram1Unknown, score: 0.18
Osbpl3Unknown, score: 0.12
Susd2Unknown, score: 0
Lrwd1Unknown, score: 0.1
CoasyUnknown, score: 0.17
R3hdm2Unknown, score: -0.01
Map3k13Unknown, score: 0.15
Gm29609Unknown, score: 0.26
Tmprss6Unknown, score: 0.37
Cpn2Unknown, score: 0.08
EtnpplUnknown, score: 0.09
VwceUnknown, score: 0.23
Plbd2Unknown, score: 0.39
Ugt2b1Unknown, score: 0.02
Shroom1Unknown, score: 0.14
Tha1Unknown, score: 0.43
Ing3Unknown, score: 0.06
Slc16a14Unknown, score: 0.09
Ankle2Unknown, score: 0.07
Trnau1apUnknown, score: 0.16
Anxa9Unknown, score: 0.28
Ints12Unknown, score: 0.13
Ptcd1Unknown, score: 0.02
Slc25a18Unknown, score: 0.2
Mtfr2Unknown, score: 0.18
Ranbp3Unknown, score: 0.17
Tmem50aUnknown, score: 0.35
Ddi1Unknown, score: 0.25
Zbtb43Unknown, score: 0.13
Lancl2Unknown, score: 0.16
1700003E16RikUnknown, score: 0.17
1700008I05RikUnknown, score: 0.01
R3hcc1Unknown, score: 0
Syce2Unknown, score: 0.11
Dpep3Unknown, score: 0.16
Cfap52Unknown, score: 0.33
Gpr160Unknown, score: 0.09
Fam217aUnknown, score: 0.31
Fbxo30Unknown, score: 0.05
Cfap45Unknown, score: 0.27
CenpuUnknown, score: 0.17
2310003H01RikUnknown, score: 0.4
Epn3Unknown, score: 0.13
Mad2l2Unknown, score: 0.05
Cdadc1Unknown, score: 0.01
Paqr7Unknown, score: 0.03
Serpina9Unknown, score: 0.05
Haus5Unknown, score: 0.13
Bdh1Unknown, score: -0.01
Antxr2Unknown, score: -0.01
Zcchc24Unknown, score: 0.34
Rpap3Unknown, score: 0.08
2310047M10RikUnknown, score: 0.06
Itfg1Unknown, score: 0.2
Tmem123Unknown, score: 0.09
Cars2Unknown, score: 0.23
Tom1l1Unknown, score: 0.08
Suds3Unknown, score: 0.15
Ist1Unknown, score: 0.05
Gatsl3Unknown, score: 0.18
Cdca4Unknown, score: 0.19
Wdr73Unknown, score: 0.12
Rbpms2Unknown, score: 0.06
Prmt3Unknown, score: 0.26
Ppp2r2aUnknown, score: 0.33
Tdrd12Unknown, score: 0.31
Ddx28Unknown, score: 0.36
Esco2Unknown, score: 0.02
Cnn3Unknown, score: -0.01
Smg9Unknown, score: 0.09
Slc25a35Unknown, score: 0.06
1600002H07RikUnknown, score: -0.01
Cyb5r1Unknown, score: 0.01
Fundc1Unknown, score: 0.49
Slc39a4Unknown, score: 0.11
Cnpy3Unknown, score: 0.46
Tsc22d2Unknown, score: 0.27
Mccc1Unknown, score: 0.12
Sulf2Unknown, score: 0.02
UrgcpUnknown, score: 0.45
Ddx42Unknown, score: 0.02
1810055G02RikUnknown, score: 0.19
2010111I01RikUnknown, score: 0.42
Cnot2Unknown, score: 0.27
OgfrUnknown, score: 0.08
Gcnt3Unknown, score: 0.22
PigxUnknown, score: 0.3
Osgepl1Unknown, score: 0.13
Ush1cUnknown, score: 0.37
Ugt2a3Unknown, score: 0.2
2010300C02RikUnknown, score: 0.24
Dusp11Unknown, score: 0.22
Jmjd8Unknown, score: 0
Dscc1Unknown, score: 0.02
Adck1Unknown, score: 0.24
Naa50Unknown, score: 0.31
Tpx2Unknown, score: 0.1
Dennd2dUnknown, score: 0.55
Ccdc71lUnknown, score: 0.15
Seh1lUnknown, score: 0.49
2610008E11RikUnknown, score: 0.15
Pex13Unknown, score: 0.29
Chst14Unknown, score: 0.03
Zbtb46Unknown, score: 0.38
StradaUnknown, score: 0.39
Rfc5Unknown, score: 0.02
Zfp157Unknown, score: 0.13
CenpnUnknown, score: 0.05
Pgm2Unknown, score: 0.15
Tmem163Unknown, score: 0.41
Dhx36Unknown, score: 0.14
Chchd4Unknown, score: 0.04
Shq1Unknown, score: 0.18
Mfsd8Unknown, score: 0.14
Zfp661Unknown, score: 0.16
Nsun4Unknown, score: 0.41
Dbndd1Unknown, score: 0.12
2510009E07RikUnknown, score: 0.15
Mms19Unknown, score: 0.16
1700030K09RikUnknown, score: 0.27
Tram1Unknown, score: 0.03
Lrrc8eUnknown, score: 0.02
Ccpg1Unknown, score: 0.18
Sh2d4aUnknown, score: 0.26
Lsm11Unknown, score: 0.03
Rusc1Unknown, score: 0
Brf1Unknown, score: 0.02
Tmem158Unknown, score: 0.05
Cyth4Unknown, score: 0.04
Asb6Unknown, score: -0.01
PalldUnknown, score: 0.46
Amer1Unknown, score: 0.02
Cdpf1Unknown, score: 0.04
2210016L21RikUnknown, score: 0.07
2310045N01RikUnknown, score: 0.12
2210408I21RikUnknown, score: 0.16
Faim2Unknown, score: 0.33
Rbm12b1Unknown, score: 0.03
BrapUnknown, score: 0.37
Pinx1Unknown, score: 0.19
Slc43a1Unknown, score: 0.49
Wdr44Unknown, score: 0.02
Kcnmb2Unknown, score: 0.31
Sgol1Unknown, score: 0.13
Ttc30bUnknown, score: 0.53
Katnbl1Unknown, score: 0.12
Dnajc25Unknown, score: -0.01
Ccdc71Unknown, score: 0.34
Htatsf1Unknown, score: 0.22
PrcpUnknown, score: 0.14
Ssbp3Unknown, score: 0.31
Tmem87bUnknown, score: 0.35
Ier5lUnknown, score: 0.09
2610507B11RikUnknown, score: 0.03
Dzip1lUnknown, score: 1
Rps6kb1Unknown, score: 0.02
Tmem173Unknown, score: 0.3
Fgfbp3Unknown, score: 0.23
Wdr43Unknown, score: 0.13
Aldh1b1Unknown, score: 0.17
Hsdl1Unknown, score: 0.28
Utp14aUnknown, score: 0.38
Zfp566Unknown, score: 0.21
Pcbd2Unknown, score: 0.39
UacaUnknown, score: 0.14
Bclaf1Unknown, score: 0.36
Lin9Unknown, score: 0.38
Bbs5Unknown, score: 0.08
Spats2Unknown, score: 0.24
ZufspUnknown, score: 0.34
Cul4bUnknown, score: 0.39
Lypd1Unknown, score: 0.17
Pdia5Unknown, score: 0.02
Usp13Unknown, score: 0.28
Zfp655Unknown, score: 0.59
2700029M09RikUnknown, score: 0.11
Anks3Unknown, score: 0.1
Hspa12bUnknown, score: 0.07
TdrkhUnknown, score: 0.07
LinsUnknown, score: 0.18
Mex3aUnknown, score: 0.41
Tmem209Unknown, score: 0.26
2810006K23RikUnknown, score: 0.15
Ccdc12Unknown, score: 0.2
Serp2Unknown, score: 0.13
Skida1Unknown, score: 0.37
Adipor1Unknown, score: 0.23
Calhm2Unknown, score: 0.05
Lime1Unknown, score: -0.01
Angptl1Unknown, score: 0.27
Fam98aUnknown, score: 0.27
B3gat3Unknown, score: 0
Cdc42se2Unknown, score: 0.13
Tmx1Unknown, score: 0.35
Zkscan3Unknown, score: 0.13
TonslUnknown, score: 0.06
72775Unknown, score: 0.32
Dnajc22Unknown, score: 0.32
Rspo3Unknown, score: 0.32
Ndc1Unknown, score: 0.18
Veph1Unknown, score: 0.1
Zfp839Unknown, score: 0.45
Zfp429Unknown, score: 0.03
Scn2bUnknown, score: 0.08
Fam76bUnknown, score: 0.37
Dhx30Unknown, score: 0.14
Crtac1Unknown, score: 0.11
AC182748.2Unknown, score: 0.05
Kctd17Unknown, score: 0.63
Mblac2Unknown, score: 0.23
Cxx1cUnknown, score: 0.52
Xlr4bUnknown, score: 0.19
Macrod2Unknown, score: 0.16
Ndufv2Unknown, score: 0.16
Ddx41Unknown, score: 0.44
TpppUnknown, score: 0.22
Ccnt2Unknown, score: 0.43
TympUnknown, score: 0.1
Ccser2Unknown, score: 0.06
Fbxo47Unknown, score: 0.2
Tmem138Unknown, score: 0.61
Appl1Unknown, score: 0.01
Kremen2Unknown, score: 0.04
Tmem192Unknown, score: 0.09
Ppil6Unknown, score: 0.18
Rps6ka5Unknown, score: 0.48
Prss57Unknown, score: 0.55
Fam101aUnknown, score: 0.14
Tgfbrap1Unknown, score: 0.21
Golim4Unknown, score: 0.21
Tmed5Unknown, score: 0.05
Prrc1Unknown, score: 0.08
Clec4a3Unknown, score: 0.15
Arhgap8Unknown, score: 0.12
Exo5Unknown, score: 0.37
Tbkbp1Unknown, score: 0.24
Pear1Unknown, score: 0.2
XpotUnknown, score: 0.14
Fam118aUnknown, score: 0.18
3110052M02RikUnknown, score: 0.25
BmperUnknown, score: 0.52
Atat1Unknown, score: 0.11
MrgbpUnknown, score: 0.14
Setd7Unknown, score: 0.17
1700037C18RikUnknown, score: 0.3
Gpbp1Unknown, score: 0.15
Ddit4lUnknown, score: 0.46
Ccdc103Unknown, score: 0.07
Calr3Unknown, score: 0.02
Ube2d2bUnknown, score: 0.16
Pradc1Unknown, score: 0.31
Ccdc30Unknown, score: 0.14
Slc25a31Unknown, score: 0.12
Arhgef6Unknown, score: 0.1
Col20a1Unknown, score: 0.01
Dcbld2Unknown, score: 0.15
TeppUnknown, score: 0.05
Nme8Unknown, score: 0.11
1700052N19RikUnknown, score: 0.29
Prox2Unknown, score: 0.31
Tex35Unknown, score: 0.2
1700066B19RikUnknown, score: 0.38
Izumo1Unknown, score: 0.18
1700055N04RikUnknown, score: 0.27
1700066M21RikUnknown, score: 0.06
Iws1Unknown, score: 0.08
Mipol1Unknown, score: 0.09
4933434E20RikUnknown, score: 0.1
Vgll3Unknown, score: 0.02
1700001O22RikUnknown, score: 0.12
Cybrd1Unknown, score: 0.23
Spns1Unknown, score: 0.09
Cabp4Unknown, score: 0.17
Thoc3Unknown, score: 0.03
2410004P03RikUnknown, score: 0.26
Ttc21bUnknown, score: 0.07
Rec114Unknown, score: 0.44
Wdr75Unknown, score: 0.07
Psma8Unknown, score: 0.07
Zbtb8aUnknown, score: 0.06
Bloc1s2Unknown, score: 0.02
Glipr1Unknown, score: 0.1
2410089E03RikUnknown, score: 0.06
Rbm20Unknown, score: 0.19
Cst6Unknown, score: 0.01
Sh3bgrl3Unknown, score: 0.02
PsdUnknown, score: 0.28
1110008P14RikUnknown, score: 0.37
WhrnUnknown, score: -0.01
Thap1Unknown, score: 0.03
Kif2cUnknown, score: 0.18
Mfsd12Unknown, score: 0.13
Poldip3Unknown, score: 0.05
Tmem198bUnknown, score: 0.17
Dcaf4Unknown, score: 0.41
Fam98cUnknown, score: 0.11
Slc35b2Unknown, score: 0
Ankrd45Unknown, score: 0.03
Fam110aUnknown, score: 0.49
4930415F15RikUnknown, score: 0.04
4930415O20RikUnknown, score: 0.25
Zdbf2Unknown, score: 0.06
Psmb11Unknown, score: 0.64
Irak3Unknown, score: 0.28
Ift57Unknown, score: 0.08
Fam151bUnknown, score: 0.16
Otud4Unknown, score: 0.14
Atl1Unknown, score: 0.15
Psd2Unknown, score: 0.03
74004Unknown, score: 0.24
ArsgUnknown, score: 0.38
Slc25a27Unknown, score: 0.35
Rap2bUnknown, score: 0.36
Fcho1Unknown, score: 0.04
Phf19Unknown, score: 0.3
Als2Unknown, score: 0.24
Traf3ip1Unknown, score: 0.12
Nphp3Unknown, score: 0.1
Msl1Unknown, score: 0.36
Rin2Unknown, score: 0.01
Nfam1Unknown, score: 0.13
4632428N05RikUnknown, score: 0.35
Steap2Unknown, score: 0.18
4931406B18RikUnknown, score: 0.02
Plce1Unknown, score: 0.16
Serpina3aUnknown, score: 0.27
Nmnat3Unknown, score: 0.19
Slc7a13Unknown, score: 0.15
Them7Unknown, score: 0.07
Tjap1Unknown, score: 0.22
Pop7Unknown, score: 0.14
Arpp21Unknown, score: 0.26
Slc35a5Unknown, score: 0.06
Dcaf6Unknown, score: 0.23
Cep55Unknown, score: 0.16
Rbm19Unknown, score: 0.61
Usp16Unknown, score: 0.26
Actr3Unknown, score: 0.06
Krt80Unknown, score: 0.3
Smg8Unknown, score: 0.08
Lonp1Unknown, score: 0.18
F13a1Unknown, score: 0.29
CluhUnknown, score: 0.53
Slc35f5Unknown, score: 0.03
Errfi1Unknown, score: 0.13
Josd1Unknown, score: 0.3
Nfx1Unknown, score: 0
Fbxl22Unknown, score: -0.01
Tmem38aUnknown, score: 0.09
Stk40Unknown, score: 0.16
Muc5bUnknown, score: 0.05
Exoc3l4Unknown, score: 0.02
P2ry13Unknown, score: 0.02
Arpc5lUnknown, score: 0.47
Elp3Unknown, score: 0.43
Ttc27Unknown, score: 0.25
Dtx2Unknown, score: 0.3
VitUnknown, score: 0.16
2810403A07RikUnknown, score: 0.33
Fblim1Unknown, score: 0.02
Xpo6Unknown, score: 0.07
1700017B05RikUnknown, score: 0.43
Paqr8Unknown, score: 0.22
Tubgcp2Unknown, score: 0.77
ChpfUnknown, score: 0.13
Atg7Unknown, score: 0.06
GaleUnknown, score: 0.22
Ankrd9Unknown, score: 0.28
Armc1Unknown, score: 0.02
Gpn1Unknown, score: 0.16
Smu1Unknown, score: 0.12
Tspan17Unknown, score: 0.07
Usp20Unknown, score: 0.09
Kcmf1Unknown, score: 0.09
Spem1Unknown, score: 0.01
Mtmr3Unknown, score: 0.1
Rnf145Unknown, score: 0.46
Mettl23Unknown, score: 0.04
Cxxc1Unknown, score: 0.18
CltbUnknown, score: 0.36
Xrcc3Unknown, score: 0.29
Crtc2Unknown, score: 0.32
Fam160a2Unknown, score: 0.13
Ddx23Unknown, score: 0
Zfp84Unknown, score: 0.12
LrgukUnknown, score: 0.22
4931428F04RikUnknown, score: -0.01
4931414P19RikUnknown, score: 0.34
Ubap2lUnknown, score: 0.15
Ap5m1Unknown, score: 0.51
Rmi1Unknown, score: 0.02
Map10Unknown, score: 0
Efhc2Unknown, score: 0.12
Ttll11Unknown, score: 0.29
Tc2nUnknown, score: 0.18
4930452B06RikUnknown, score: 0.18
Lrriq3Unknown, score: 0.29
Arl13aUnknown, score: 0.02
Cfap53Unknown, score: 0.27
Nsun6Unknown, score: 0.05
Zswim5Unknown, score: 0.12
4933427G17RikUnknown, score: 0.49
Pus10Unknown, score: 0.26
4933433C11RikUnknown, score: 0.06
MamstrUnknown, score: 0.12
Gorasp1Unknown, score: 0.37
Fam53aUnknown, score: 0.1
Lrrc17Unknown, score: 0.36
Neto2Unknown, score: 0.26
Cyp2j9Unknown, score: 0.02
Ppp4r4Unknown, score: 0.03
Mgme1Unknown, score: 0.1
Rasgef1cUnknown, score: 0.22
Ttc17Unknown, score: 0.09
Glb1lUnknown, score: 0.2
Cds1Unknown, score: 0.13
Mrpl47Unknown, score: 0.17
Abcb8Unknown, score: 0.05
OcstampUnknown, score: 0.42
Scrn3Unknown, score: 0.34
Scpep1Unknown, score: -0.01
Tmem81Unknown, score: 0.11
Spsb1Unknown, score: 0.17
PomkUnknown, score: 0.05
Lrrc48Unknown, score: 0.1
4930432K21RikUnknown, score: 0.12
Zfp943Unknown, score: 0.01
74684Unknown, score: 0.04
Tbc1d30Unknown, score: 0.36
Spata17Unknown, score: 0.33
Snx16Unknown, score: 0.13
Trim14Unknown, score: 0.38
Pcf11Unknown, score: 0.09
Ddit4Unknown, score: 0
5830415F09RikUnknown, score: 0.13
Mdga1Unknown, score: 0.07
Lmbr1lUnknown, score: 0.16
Ppa2Unknown, score: 0.13
Sepn1Unknown, score: 0.19
Wipi2Unknown, score: 0.09
Glt8d2Unknown, score: 0.37
Naa15Unknown, score: 0.2
ManfUnknown, score: -0.01
Mss51Unknown, score: 0.15
Fam228aUnknown, score: 0
Tmem65Unknown, score: 0.24
Armc4Unknown, score: 0.15
Usp47Unknown, score: 0.26
Rnase10Unknown, score: 0.18
PurgUnknown, score: 0.03
Kif27Unknown, score: 0.36
Ccdc173Unknown, score: 0.17
Usp50Unknown, score: 0.04
4930505A04RikUnknown, score: 0
Uhrf1bp1lUnknown, score: 0.06
Lysmd4Unknown, score: 0.2
Mmd2Unknown, score: -0.01
Nxnl2Unknown, score: 0.19
75136Unknown, score: 0.03
4930538K18RikUnknown, score: 0.18
Spaca6Unknown, score: 0.38
Prr3Unknown, score: 0.03
Rnf121Unknown, score: 0.23
Bcdin3dUnknown, score: 0.02
Zbtb3Unknown, score: 0.28
Fgfr1opUnknown, score: -0.01
4930550C14RikUnknown, score: 0.51
Taf1dUnknown, score: 0.18
ParpbpUnknown, score: 0.28
Etnk1Unknown, score: 0.41
4930563D23RikUnknown, score: 0.1
Ccdc83Unknown, score: 0.7
Mphosph8Unknown, score: 0.05
Slamf7Unknown, score: 0.06
Sirt4Unknown, score: 0.17
Spp2Unknown, score: 0.36
Mrpl32Unknown, score: 0.42
Ndufs7Unknown, score: 0.49
Kmt2bUnknown, score: 0.23
Arhgap12Unknown, score: 0.3
Mettl5Unknown, score: 0.22
Arl5aUnknown, score: 0.35
Zfp820Unknown, score: 0.11
Fam183bUnknown, score: 0.2
CklfUnknown, score: 0.18
Dynlrb2Unknown, score: 0.38
Fabp12Unknown, score: 0.12
Tex29Unknown, score: 0.47
Nme5Unknown, score: 0.09
Fam71e1Unknown, score: 0.06
Akap13Unknown, score: 0.22
Dnali1Unknown, score: 0
Rsph9Unknown, score: 0.12
75570Unknown, score: 0.04
Acyp2Unknown, score: 0.19
Malsu1Unknown, score: 0.19
Calml4Unknown, score: 0.01
Tm4sf5Unknown, score: 0.2
Wnk2Unknown, score: 0.04
Chmp4bUnknown, score: 0.04
Med25Unknown, score: 0.48
Smim15Unknown, score: 0.36
Rps25Unknown, score: 0.46
Spaca3Unknown, score: 0.13
Tex30Unknown, score: 0.05
Mageh1Unknown, score: 0.4
IppkUnknown, score: 0.05
Higd1bUnknown, score: 0.14
Anks6Unknown, score: 0.35
Nr2c2apUnknown, score: 0.18
C2cd4bUnknown, score: 0.22
Tmem14aUnknown, score: 0.1
Amotl1Unknown, score: 0.35
Phf14Unknown, score: 0.17
Fam227aUnknown, score: 0
IdnkUnknown, score: 0
MffUnknown, score: 0.35
Egfem1Unknown, score: 0.07
SvipUnknown, score: 0.62
Slc10a6Unknown, score: 0.21
Ipo4Unknown, score: 0.14
9130401M01RikUnknown, score: 0.25
DcstampUnknown, score: 0
Lppr5Unknown, score: 0.03
Brsk2Unknown, score: 0.03
Pnpla5Unknown, score: 0.07
Them4Unknown, score: 0.09
Lca5Unknown, score: 0.08
Klhl24Unknown, score: 0
Cdyl2Unknown, score: 0.57
4930447C04RikUnknown, score: 0.45
NlnUnknown, score: 0.05
Tasp1Unknown, score: 0.1
Wdr64Unknown, score: 0.3
Fam227bUnknown, score: 0.05
Senp2Unknown, score: 0.28
Hormad2Unknown, score: 0.14
Rnf139Unknown, score: 0.33
Clec4gUnknown, score: 0.24
Zfp821Unknown, score: 0.1
AdalUnknown, score: 0.03
4930578C19RikUnknown, score: 0.42
Fam184aUnknown, score: 0.25
Vmp1Unknown, score: 0.1
Exoc6bUnknown, score: 0.2
4930579G24RikUnknown, score: 0.01
Srrm2Unknown, score: 0.09
Trappc8Unknown, score: 0.23
Zdhhc20Unknown, score: 0.14
75973Unknown, score: 0.26
Dock11Unknown, score: 0.15
Rab30Unknown, score: 0.13
Zmym2Unknown, score: 0.02
Cant1Unknown, score: 0.27
Ccdc125Unknown, score: 0.02
Rnf183Unknown, score: 0.31
Pcgf5Unknown, score: 0.13
Gbp8Unknown, score: 0.18
TtpalUnknown, score: 0.42
Rap2aUnknown, score: 0.1
Arhgap15Unknown, score: 0.31
Gpsm2Unknown, score: 0.24
Las1lUnknown, score: 0.23
Depdc1aUnknown, score: 0.18
FaxcUnknown, score: 0
Mcur1Unknown, score: 0.12
Fam131bUnknown, score: 0.09
Slc35d3Unknown, score: 0.04
Usp31Unknown, score: 0.2
Abca6Unknown, score: 0.23
Adhfe1Unknown, score: 0.11
Jakmip2Unknown, score: 0.15
Arxes1Unknown, score: 0.4
76224Unknown, score: 0.04
Dnttip1Unknown, score: 0.17
GrhprUnknown, score: 0.11
Rtf1Unknown, score: 0.11
Ercc6l2Unknown, score: 0.26
Slc38a3Unknown, score: 0.34
Ttc8Unknown, score: 0.31
Tsen54Unknown, score: 0.5
Ndfip2Unknown, score: 0.36
GptUnknown, score: 0.19
Mfap4Unknown, score: 0.22
Asb5Unknown, score: 0.12
Atp11bUnknown, score: 0.5
Erp44Unknown, score: 0.07
PcnpUnknown, score: 0.19
OsbpUnknown, score: 0.18
Rab2bUnknown, score: 0.11
Tbx18Unknown, score: 0.18
Det1Unknown, score: 0.11
Slc24a2Unknown, score: 0.32
Cep112Unknown, score: 0.26
Abcc3Unknown, score: 0.26
Ift43Unknown, score: 0.78
Znrd1asUnknown, score: 0.2
Gid8Unknown, score: 0.32
Rftn1Unknown, score: 0.09
Daam2Unknown, score: 0.07
Ccdc134Unknown, score: 0.11
Casc5Unknown, score: 0.17
Cmya5Unknown, score: 0.04
Pcolce2Unknown, score: 0.47
Haus8Unknown, score: 0.54
Lmf1Unknown, score: 0.33
Ly6kUnknown, score: 0.26
Ppp1r3gUnknown, score: 0.15
Abhd14bUnknown, score: 0.25
Paqr4Unknown, score: -0.01
Clasp2Unknown, score: 0.29
Il34Unknown, score: -0.01
Atg2bUnknown, score: 0.36
Snx7Unknown, score: 0.12
Qrsl1Unknown, score: 0.03
Fam101bUnknown, score: 0.04
Mfsd2aUnknown, score: 0.03
Mib2Unknown, score: 0.53
Dnajc18Unknown, score: 0
Hectd3Unknown, score: 0.3
Lrrc27Unknown, score: 0.14
ImmtUnknown, score: 0.18
Msi2Unknown, score: 0.22
Stambpl1Unknown, score: 0.42
1700112E06RikUnknown, score: 0.26
1700113H08RikUnknown, score: 0.08
Wdr38Unknown, score: 0
Srxn1Unknown, score: 0.03
Cfap70Unknown, score: 0.14
Clasp1Unknown, score: 0.15
Ckmt2Unknown, score: -0.01
Creld2Unknown, score: 0.2
Snx27Unknown, score: 0.04
Dapl1Unknown, score: 0.42
TrdnUnknown, score: 0.49
Wdyhv1Unknown, score: 0.06
Slc10a7Unknown, score: 0.13
Cluap1Unknown, score: 0.02
Mettl4Unknown, score: 0.56
Klhdc10Unknown, score: 0.03
2410131K14RikUnknown, score: -0.01
Tbc1d9bUnknown, score: 0.27
Tmem234Unknown, score: 0.13
Usp42Unknown, score: 0.23
Calcoco2Unknown, score: 0.3
Sdccag8Unknown, score: 0.23
Fam49aUnknown, score: 0.02
Mtfr1lUnknown, score: 0.09
Dok5Unknown, score: 0.41
Rps9Unknown, score: -0.01
Gper1Unknown, score: 0.17
Catsper3Unknown, score: 0
Dcun1d5Unknown, score: 0.23
Morn1Unknown, score: -0.01
Ccdc116Unknown, score: 0
Rab36Unknown, score: 0.39
Cyfip2Unknown, score: 0.45
Adck4Unknown, score: 0.05
Memo1Unknown, score: 0.18
Cers2Unknown, score: 0.14
Bicd2Unknown, score: 0.14
Ssbp4Unknown, score: 0.03
Lrg1Unknown, score: 0.41
Mnd1Unknown, score: 0.06
Timmdc1Unknown, score: 0.02
Arrdc5Unknown, score: 0.26
1700015E13RikUnknown, score: 0.21
HnrnpmUnknown, score: 0.2
Nt5c2Unknown, score: 0
St5Unknown, score: 0.12
Chmp5Unknown, score: 0.16
Chst1Unknown, score: 0.36
2810007J24RikUnknown, score: 0.45
UrahUnknown, score: 0.15
Arxes2Unknown, score: 0.39
Ube2ql1Unknown, score: 0.02
3110035E14RikUnknown, score: 0.02
Hdhd2Unknown, score: 0.27
Ddrgk1Unknown, score: 0.06
Mpped2Unknown, score: 0.14
Slc9a8Unknown, score: 0.23
2510039O18RikUnknown, score: 0.26
Kdm8Unknown, score: 0.14
1700109H08RikUnknown, score: 0.02
Atg16l1Unknown, score: 0.19
Bcl7aUnknown, score: 0.33
Ston1Unknown, score: 0.25
Ints7Unknown, score: 0.05
Tmem181aUnknown, score: 0.27
Mtmr2Unknown, score: 0.33
Zfp934Unknown, score: 0.12
Il33Unknown, score: 0.07
Hnrnpa0Unknown, score: 0.17
Ptgr2Unknown, score: -0.01
Tmem200aUnknown, score: 0.2
Zfp142Unknown, score: 0.22
Wdr82Unknown, score: 0.31
Ankrd55Unknown, score: 0.07
Esrp2Unknown, score: 0.24
Tmem116Unknown, score: 0.17
Kidins220Unknown, score: 0.11
Anks1bUnknown, score: 0.17
JrklUnknown, score: 0.24
AglUnknown, score: 0.45
Vps33aUnknown, score: 0.02
Tcaf1Unknown, score: 0.02
Bcl9Unknown, score: 0.03
NotumUnknown, score: 0.28
Chst15Unknown, score: 0.57
Ddx10Unknown, score: 0.29
Usp45Unknown, score: 0.06
Adgrf1Unknown, score: 0.65
Ccdc151Unknown, score: 0.06
Prss36Unknown, score: 0.53
Prelid2Unknown, score: 0.3
Prdm8Unknown, score: 0.52
Snapc3Unknown, score: 0.09
Mrps5Unknown, score: 0.2
Adamtsl1Unknown, score: 0.47
BoraUnknown, score: 0.05
Elp4Unknown, score: 0.01
Dcst1Unknown, score: 0.17
Ulbp1Unknown, score: 0
Epm2aip1Unknown, score: 0.5
PolqUnknown, score: 0.19
Adamtsl2Unknown, score: 0.06
Sla2Unknown, score: 0.32
Krba1Unknown, score: -0.01
TchpUnknown, score: 0.19
MlanaUnknown, score: 0.41
Msl2Unknown, score: 0.21
Thyn1Unknown, score: 0.09
Ypel2Unknown, score: 0.34
6030458C11RikUnknown, score: 0.26
Ube2sUnknown, score: 0.6
Fam53bUnknown, score: 0.07
Rpgrip1Unknown, score: 0.15
Cyp20a1Unknown, score: 0.28
Rdh12Unknown, score: 0.28
Tmem50bUnknown, score: 0.08
CutalUnknown, score: 0.03
Prr15Unknown, score: 0.33
Ccdc150Unknown, score: 0.03
Cpt1cUnknown, score: 0.08
SowahbUnknown, score: 0.38
Msantd4Unknown, score: 0.39
Cox7b2Unknown, score: 0.25
NinlUnknown, score: 0.04
9230112D13RikUnknown, score: 0.5
Dnajc21Unknown, score: 0.04
Acbd7Unknown, score: 0.11
Phf23Unknown, score: 0.52
Adgrf4Unknown, score: 0.34
Zfp712Unknown, score: 0.09
Klhdc8bUnknown, score: 0.34
Map7d2Unknown, score: 0.11
Creb3l4Unknown, score: 0.07
Nav2Unknown, score: 0.05
Rps27aUnknown, score: 0.05
Fam219bUnknown, score: 0
Ndufv3Unknown, score: 0.08
Snrnp25Unknown, score: 0.08
MvpUnknown, score: 0.34
Ddx52Unknown, score: 0.21
3110062M04RikUnknown, score: 0.21
Rnase6Unknown, score: 0.08
Pgpep1lUnknown, score: 0.04
HelzUnknown, score: 0.27
Skap1Unknown, score: 0.38
Micu3Unknown, score: 0.2
Arhgap10Unknown, score: 0.02
B230219D22RikUnknown, score: 0.29
Adgra2Unknown, score: 0.24
Utp23Unknown, score: 0.17
UvragUnknown, score: 0.15
CstadUnknown, score: 0.21
Acap2Unknown, score: 0.05
1700061G19RikUnknown, score: 0.41
Lsm6Unknown, score: 0.3
Bola3Unknown, score: 0.24
Eif3j1Unknown, score: 0.25
Brd8Unknown, score: 0.13
Naa35Unknown, score: 0
Pus7Unknown, score: 0.12
Filip1lUnknown, score: 0.29
Zc3h6Unknown, score: 0.29
Csgalnact2Unknown, score: 0.3
Galnt15Unknown, score: 0.03
RictorUnknown, score: 0.2
Mctp1Unknown, score: 0.1
Spata2lUnknown, score: 0.4
Armc9Unknown, score: 0.26
Ndor1Unknown, score: 0.23
Ak7Unknown, score: 0.01
Ttc30a1Unknown, score: 0.04
GmipUnknown, score: 0.16
Desi2Unknown, score: 0
Slc25a12Unknown, score: 0.12
Cacul1Unknown, score: 0.2
Gins3Unknown, score: 0.22
Coro7Unknown, score: 0.16
Sfi1Unknown, score: 0.32
Trmt44Unknown, score: -0.01
Scyl1Unknown, score: 0.45
AacsUnknown, score: 0.32
Pus7lUnknown, score: 0.01
Wrnip1Unknown, score: 0.02
Sp2Unknown, score: 0.25
Ltn1Unknown, score: -0.01
Nadsyn1Unknown, score: 0.13
Fndc8Unknown, score: 0.08
9130019O22RikUnknown, score: 0.17
Chsy3Unknown, score: 0.15
PigtUnknown, score: 0
Polr3hUnknown, score: 0
Agbl4Unknown, score: 0.08
Saal1Unknown, score: 0.25
Ern1Unknown, score: 0.2
Tnfrsf22Unknown, score: 0.17
LratUnknown, score: 0.11
Trim39Unknown, score: 0.25
Krit1Unknown, score: 0.26
LiasUnknown, score: 0.13
CptpUnknown, score: 0.08
BC005537Unknown, score: 0.34
Ublcp1Unknown, score: 0.12
Sh3bp5lUnknown, score: 0.06
Smim12Unknown, score: 0.02
Tusc3Unknown, score: 0.11
Bcl9lUnknown, score: 0.15
Sptbn4Unknown, score: 0.14
Kcnip4Unknown, score: 0
Med8Unknown, score: 0.23
Herpud2Unknown, score: 0.02
Pacsin3Unknown, score: 0.48
Rab27bUnknown, score: 0.23
Pbx4Unknown, score: 0.06
Slc19a3Unknown, score: 0.03
Vps16Unknown, score: 0.31
Cwc22Unknown, score: 0.14
N4bp1Unknown, score: 0.14
CblcUnknown, score: 0.14
SelkUnknown, score: 0.16
Clca3a2Unknown, score: 0.18
Hist1h1aUnknown, score: 0.13
NfkbizUnknown, score: 0.71
Senp3Unknown, score: 0.31
Mesdc1Unknown, score: 0.15
Tmprss5Unknown, score: -0.01
Cxcr6Unknown, score: 0.17
Zfp202Unknown, score: 0.13
PolhUnknown, score: 0.06
Pum1Unknown, score: 0.12
CemipUnknown, score: 0.14
Trim44Unknown, score: 0.26
Rad54l2Unknown, score: 0.34
Tbl1xr1Unknown, score: 0.19
Gpr63Unknown, score: 0.1
Vmn1r65Unknown, score: 0.21
Vmn1r58Unknown, score: 0.04
Dnajb1Unknown, score: 0.06
Sil1Unknown, score: 0.15
Egfl8Unknown, score: 0.06
Jdp2Unknown, score: 0.18
Tfcp2l1Unknown, score: 0.24
Cacng7Unknown, score: 0.42
Cyp4x1Unknown, score: 0.02
Zfpl1Unknown, score: 0.42
Tfap4Unknown, score: 0.18
Glis2Unknown, score: 0.11
Akap12Unknown, score: 0.02
Lamtor2Unknown, score: 0.39
Il23aUnknown, score: 0
Ndel1Unknown, score: 0
Trem2Unknown, score: 0.29
Plekha3Unknown, score: 0.04
Plekha2Unknown, score: 0.09
NgrnUnknown, score: 0.33
Rbm5Unknown, score: 0.15
Pik3ap1Unknown, score: 0.3
Lin28aUnknown, score: 0.14
Tex11Unknown, score: 0.09
Tex14Unknown, score: 0.37
Gtf2a1Unknown, score: 0.1
Wdr6Unknown, score: 0.16
Sytl2Unknown, score: 0.17
Cnnm1Unknown, score: 0.07
SrrtUnknown, score: 0.29
Akr1c6Unknown, score: 0.24
Smarcd2Unknown, score: 0.16
Smarcd1Unknown, score: 0.03
Tnk1Unknown, score: 0.41
Nedd4lUnknown, score: 0.46
Slc25a2Unknown, score: 0.24
Tmem2Unknown, score: 0.16
Gpr137bUnknown, score: 0.14
Dnaja3Unknown, score: 0.49
Nrg4Unknown, score: 0.09
Btbd1Unknown, score: 0.1
Jam3Unknown, score: 0.33
Tssk6Unknown, score: 0.39
Kcnn1Unknown, score: 0.22
Usp8Unknown, score: 0.3
Ptov1Unknown, score: 0.11
Setdb1Unknown, score: 0.15
Fam126aUnknown, score: 0.09
Pla1aUnknown, score: 0.01
Tac4Unknown, score: 0.17
Cd163Unknown, score: 0.32
Clec2iUnknown, score: 0.31
Trim8Unknown, score: 0.05
GlceUnknown, score: 0.61
Sep15Unknown, score: 0.22
Rbfox2Unknown, score: 0.04
Csnk1a1Unknown, score: 0.15
Lmod1Unknown, score: 0.21
GlrxUnknown, score: 0.73
Ear6Unknown, score: 0.3
Pcdhga12Unknown, score: 0.04
Rnase2aUnknown, score: 0.52
Lztfl1Unknown, score: 0.26
Acox2Unknown, score: 0.26
Pard6gUnknown, score: 0.14
Gabarapl2Unknown, score: 0.03
Echs1Unknown, score: 0.3
Immp2lUnknown, score: 0.18
Arid1aUnknown, score: 0.19
Smarca1Unknown, score: 0.11
Smarca5Unknown, score: 0.14
Nipa2Unknown, score: 0.02
Rnf111Unknown, score: 0.51
Dqx1Unknown, score: 0.24
Igsf9Unknown, score: 0.13
PnckUnknown, score: 0.02
Pcdhb3Unknown, score: 0.11
Pcdhb4Unknown, score: 0.21
Pcdhb8Unknown, score: 0.24
Pcdhb11Unknown, score: 0.25
Pcdhb12Unknown, score: 0.3
Pcdhb13Unknown, score: 0.4
Pcdhb14Unknown, score: 0.1
Pcdhb15Unknown, score: 0.26
Pcdhb16Unknown, score: 0.39
Pcdhb17Unknown, score: 0.42
Pcdhb18Unknown, score: 0.3
Pcdhb19Unknown, score: 0.2
Pcdhb21Unknown, score: 0.33
Klra4Unknown, score: 0.31
Bcl2l13Unknown, score: 0.24
P2rx5Unknown, score: 0.39
Mrpl1Unknown, score: 0.07
Mrpl43Unknown, score: 0.07
Trim7Unknown, score: 0.22
Trim11Unknown, score: 0.2
Trim16Unknown, score: 0.23
Trim33Unknown, score: 0.07
Trim34aUnknown, score: 0.08
Med15Unknown, score: 0.36
Acsbg1Unknown, score: 0.03
Tnfrsf21Unknown, score: 0.26
Zfp423Unknown, score: 0.23
Ophn1Unknown, score: 0.05
C1galt1Unknown, score: 0.15
Pag1Unknown, score: 0.27
Lrp1bUnknown, score: 0.03
Cnnm3Unknown, score: 0.12
Cnnm4Unknown, score: 0.43
S1pr5Unknown, score: 0.12
Tinagl1Unknown, score: 0.01
Fkbp6Unknown, score: 0.06
Dtnbp1Unknown, score: 0.13
Slc24a3Unknown, score: 0.17
Maged1Unknown, score: 0.38
Sfxn3Unknown, score: 0.5
Ugt1a6aUnknown, score: 0.03
Cadm3Unknown, score: 0.02
Tmem40Unknown, score: 0.05
Loxl2Unknown, score: 0.24
Prg4Unknown, score: 0.05
Susd4Unknown, score: 0.27
TprnUnknown, score: 0.38
Wwtr1Unknown, score: 0.05
Slc9b2Unknown, score: 0.48
Nmd3Unknown, score: 0.34
Hist2h4Unknown, score: 0.11
A430005L14RikUnknown, score: 0.63
Hmgb2Unknown, score: 0.28
HadhaUnknown, score: 0.01
Naa11Unknown, score: 0
Mtmr14Unknown, score: 0.07
Cog8Unknown, score: 0.12
Sgsm2Unknown, score: 0.03
4833439L19RikUnknown, score: 0.01
Exd2Unknown, score: 0.13
B3galnt2Unknown, score: 0.17
Nlrp4fUnknown, score: 0.07
Nol12Unknown, score: 0.46
DeptorUnknown, score: 0.04
Dcaf8Unknown, score: 0.22
Lrrc59Unknown, score: 0.15
KmoUnknown, score: 0.77
D2hgdhUnknown, score: 0.31
Slamf9Unknown, score: 0.54
Smap1Unknown, score: 0.32
Slc41a1Unknown, score: 0.09
Sh3bp4Unknown, score: 0.24
Zfp451Unknown, score: 0.37
AI597479Unknown, score: 0.34
Nucks1Unknown, score: 0.28
Phlpp1Unknown, score: 0.11
Gtf3c3Unknown, score: 0.06
Pid1Unknown, score: 0.2
Mfsd6Unknown, score: 0.29
RabifUnknown, score: 0.11
Obsl1Unknown, score: 0.1
Ubac1Unknown, score: 0.24
Eps8l2Unknown, score: 0.19
Usp6nlUnknown, score: 0.24
Fam102aUnknown, score: 0.12
Nat10Unknown, score: 0.31
Anapc2Unknown, score: 0.76
Ssx2ipUnknown, score: 0.02
Commd7Unknown, score: 0.09
Garnl3Unknown, score: 0.2
Zscan29Unknown, score: 0.09
Arfgef2Unknown, score: 0.04
Golga2Unknown, score: 0.09
Duox1Unknown, score: 0.01
Magi3Unknown, score: 0.15
Dnttip2Unknown, score: 0.19
Usp53Unknown, score: 0.06
Olfml3Unknown, score: 0.35
FggUnknown, score: 0.15
Ankrd50Unknown, score: 0.26
Cept1Unknown, score: 0.22
Taf13Unknown, score: 0.23
Arfip1Unknown, score: 0.09
Prmt6Unknown, score: 0.37
Kdm1aUnknown, score: 0.01
Ldlrap1Unknown, score: 0.12
Mdn1Unknown, score: 0.26
Gpr153Unknown, score: 0.04
Phactr4Unknown, score: 0.49
H6pdUnknown, score: 0.06
Stx12Unknown, score: 0.1
Ppp1r8Unknown, score: 0.25
Smpdl3bUnknown, score: 0.11
Fam46bUnknown, score: 0.07
Bsdc1Unknown, score: 0.31
Slc44a1Unknown, score: 0
Lao1Unknown, score: 0.02
Zfand2aUnknown, score: 0.39
Zfp518bUnknown, score: 0.07
Nsun5Unknown, score: 0.15
Upk3bUnknown, score: 0.21
Pds5bUnknown, score: 0.19
Papd4Unknown, score: 0.3
Ugt2b34Unknown, score: 0.15
Mapre3Unknown, score: 0.02
Rita1Unknown, score: 0.06
Tbc1d14Unknown, score: 0.12
Chpf2Unknown, score: 0
Emilin1Unknown, score: 0.01
Rab28Unknown, score: 0.11
Nfxl1Unknown, score: 0.19
Zfp282Unknown, score: 0.02
Snx21Unknown, score: 0.16
Rpusd3Unknown, score: 0.07
Itfg2Unknown, score: 0.13
B630005N14RikUnknown, score: 0.27
Pot1aUnknown, score: 0.19
Parp11Unknown, score: 0.11
Zfp956Unknown, score: 0.21
Hepacam2Unknown, score: 0.14
Tada3Unknown, score: 0.2
Wdr91Unknown, score: 0.17
Brk1Unknown, score: 0.11
Prrt4Unknown, score: 0.27
Adamts9Unknown, score: 0.2
Plekha1Unknown, score: 0.41
Slco2b1Unknown, score: 0.01
Inpp5fUnknown, score: -0.01
Zfp575Unknown, score: 0.06
Vrk3Unknown, score: 0.24
Grwd1Unknown, score: 0.16
Pwwp2bUnknown, score: 0.18
Spty2d1Unknown, score: 0.47
Numa1Unknown, score: 0.44
Psip1Unknown, score: 0
Spred3Unknown, score: 0.3
C230052I12RikUnknown, score: 0.2
AW146154Unknown, score: 0.11
Unc45aUnknown, score: 0
Igflr1Unknown, score: 0.08
Champ1Unknown, score: 0.41
Ces2aUnknown, score: 0.3
Plekhg4Unknown, score: 0.31
DohhUnknown, score: 0.53
Enkd1Unknown, score: 0.1
Zdhhc7Unknown, score: 0.02
Agpat6Unknown, score: 0.35
Cpne7Unknown, score: 0.47
Cyp4v3Unknown, score: 0.01
Ankrd10Unknown, score: 0.03
Cog4Unknown, score: 0.11
MyzapUnknown, score: 0.09
Lars2Unknown, score: 0.19
Imp3Unknown, score: 0.06
Cmtm7Unknown, score: 0
Phldb1Unknown, score: 0.14
Lrrc49Unknown, score: 0.4
Pls3Unknown, score: 0.05
D330045A20RikUnknown, score: 0.21
Nudt10Unknown, score: 0.14
Sept10Unknown, score: 0.09
Pan2Unknown, score: 0.13
Gstt3Unknown, score: 0.1
Rdh9Unknown, score: 0.18
Upb1Unknown, score: 0.12
Traf3ip2Unknown, score: 0.32
Csnk1g2Unknown, score: 0.22
Tmem263Unknown, score: 0.25
NclnUnknown, score: 0.22
Nt5dc3Unknown, score: 0.04
Nup107Unknown, score: 0.36
Mgat4bUnknown, score: 0.28
Psme4Unknown, score: 0.34
Fbxw11Unknown, score: 0.06
Slc35e4Unknown, score: 0.02
Tubg1Unknown, score: 0.33
Pex12Unknown, score: 0
Mien1Unknown, score: 0.41
Tmem98Unknown, score: 0.26
Tmem17Unknown, score: 0.42
Slc25a41Unknown, score: 0.03
Cuedc1Unknown, score: 0.34
Rpn1Unknown, score: 0.16
Plin1Unknown, score: 0.29
GckUnknown, score: 0.17
Rtn1Unknown, score: 0.05
SynpoUnknown, score: 0.26
Wdr7Unknown, score: 0.2
Cyp27a1Unknown, score: 0.39
Itga9Unknown, score: 0.05
Ndufb11Unknown, score: 0.32
Sbk1Unknown, score: 0.57
BlmhUnknown, score: 0.07
Cabin1Unknown, score: 0.09
Tex15Unknown, score: 0.32
Csnk1dUnknown, score: 0.22
Gas8Unknown, score: 0.3
Zfp120Unknown, score: 0.36
Isl2Unknown, score: 0.48
Rhox9Unknown, score: 0.14
DgkzUnknown, score: 0.34
0610010K14RikUnknown, score: 0.29
Ccdc117Unknown, score: 0.27
RprmlUnknown, score: 0.11
MycbpapUnknown, score: 0.41
Cnot6Unknown, score: 0.27
Tsr1Unknown, score: 0.17
Slc16a6Unknown, score: 0.23
Pik3r6Unknown, score: 0.25
Ttc7bUnknown, score: 0.08
Pld4Unknown, score: 0.25
JkampUnknown, score: 0
Vipas39Unknown, score: 0.2
Ptpn23Unknown, score: 0.11
Tecpr2Unknown, score: 0.05
Spata7Unknown, score: 0.19
Tdp1Unknown, score: 0.33
Tmem179Unknown, score: 0
Slc25a47Unknown, score: 0.37
PeloUnknown, score: 0.14
IarsUnknown, score: 0.04
Nhlrc1Unknown, score: 0.12
Rnf44Unknown, score: 0.49
Slc9a3Unknown, score: 0.02
Txndc5Unknown, score: 0.4
Cdk20Unknown, score: 0.48
Epdr1Unknown, score: 0.2
Utp15Unknown, score: 0.38
Ankrd32Unknown, score: 0.12
Fam149bUnknown, score: 0.13
Slain1Unknown, score: 0.07
Kctd9Unknown, score: 0.08
Dock9Unknown, score: 0.13
Mmrn2Unknown, score: 0.08
Abhd4Unknown, score: 0.19
Exoc5Unknown, score: 0.02
Ankrd28Unknown, score: 0.13
Zfp957Unknown, score: 0.15
Rcbtb2Unknown, score: 0.06
PpifUnknown, score: 0.13
Mycbp2Unknown, score: 0.19
Kdelr3Unknown, score: 0.23
Amigo2Unknown, score: -0.01
Sgsm3Unknown, score: 0.34
Card10Unknown, score: 0.04
Lmf2Unknown, score: 0.25
Csdc2Unknown, score: 0.16
Fam19a5Unknown, score: 0.17
Gga1Unknown, score: 0.02
Fbxo4Unknown, score: 0.18
Mfsd5Unknown, score: 0.16
Txndc11Unknown, score: 0.35
Zc3h7aUnknown, score: 0.16
Qtrtd1Unknown, score: 0.27
Rrn3Unknown, score: 0.12
Nsun3Unknown, score: 0.51
Rfc4Unknown, score: 0
Ildr1Unknown, score: 0.15
Ypel1Unknown, score: 0.14
Slc51aUnknown, score: 0.05
Sft2d1Unknown, score: 0.24
Stk38Unknown, score: 0.13
TecrUnknown, score: 0.18
Scaf8Unknown, score: 0.56
Wdr90Unknown, score: 0.27
VmacUnknown, score: 0.23
G6bUnknown, score: 0.09
CatsperdUnknown, score: 0.1
Stap2Unknown, score: 0.03
Dhx57Unknown, score: 0.13
Tcf19Unknown, score: 0.17
Unc119bUnknown, score: 0.28
Abhd3Unknown, score: 0.15
Afap1l1Unknown, score: 0.55
Slc39a3Unknown, score: 0.05
Arap3Unknown, score: 0.05
Slc39a6Unknown, score: 0.3
Gramd3Unknown, score: 0.22
Me2Unknown, score: 0.11
LarsUnknown, score: 0.37
Psmg2Unknown, score: 0.03
Rrp12Unknown, score: 0.3
Cyp2c50Unknown, score: 0.19
Gpr137Unknown, score: 0.19
Kazald1Unknown, score: 0.09
YarsUnknown, score: 0.27
Gbf1Unknown, score: 0.12
Kank1Unknown, score: 0.14
Tm9sf3Unknown, score: 0.13
Pdzd8Unknown, score: 0.12
Exoc6Unknown, score: 0.25
Brms1Unknown, score: 0.3
AcacaUnknown, score: 0.38
Atf5Unknown, score: 0.48
Lgr4Unknown, score: -0.01
Ece2Unknown, score: 0.46
Arl2bpUnknown, score: 0.49
Wwp1Unknown, score: 0
Col16a1Unknown, score: 0.06
MylkUnknown, score: 0.03
Nod1Unknown, score: 0
Pi4kbUnknown, score: 0.11
Mrpl10Unknown, score: 0.37
Mrpl41Unknown, score: 0.09
Rapgef1Unknown, score: 0.07
Aldh1l1Unknown, score: 0.14
Prrxl1Unknown, score: 0.17
Lgals2Unknown, score: 0.59
Ankrd1Unknown, score: 0.22
HaaoUnknown, score: -0.01
Tm6sf2Unknown, score: 0.28
BmycUnknown, score: 0.36
Jmjd6Unknown, score: 0.01
Whsc1Unknown, score: 0.17
Usp9yUnknown, score: 0.21
Mgat5Unknown, score: 0.02
Pacs1Unknown, score: 0.04
Ddb2Unknown, score: 0.11
CenpfUnknown, score: 0.07
Ap4e1Unknown, score: 0.23
Ap1s2Unknown, score: 0.38
Srsf9Unknown, score: 0.32
Shmt2Unknown, score: 0.37
Slc14a1Unknown, score: 0.37
Camk2dUnknown, score: 0.18
Cstf2Unknown, score: 0.11
Olr1Unknown, score: 0.1
Prkaa2Unknown, score: 0.14
Pip4k2bUnknown, score: 0.08
Rnf216Unknown, score: 0.47
Rnf144aUnknown, score: 0.68
Prkab2Unknown, score: 0.01
Fermt3Unknown, score: 0.17
U2af1Unknown, score: 0.32
NapaUnknown, score: 0.38
Xrcc4Unknown, score: 0.58
Ak6Unknown, score: 0.14
Galnt2Unknown, score: 0.53
Galnt7Unknown, score: 0.56
Adamts6Unknown, score: 0.17
Mthfd1Unknown, score: 0.3
Ubxn8Unknown, score: 0.26
Slc35b3Unknown, score: 0.03
108660Unknown, score: 0.01
Epsti1Unknown, score: 0.18
Dnajc9Unknown, score: 0.11
Zdhhc15Unknown, score: 0.2
Gpt2Unknown, score: 0.49
Obfc1Unknown, score: 0.32
Chn1Unknown, score: 0.13
Card11Unknown, score: 0.02
Sft2d2Unknown, score: 0.57
Oxsr1Unknown, score: 0.08
Galnt16Unknown, score: 0.1
Ston2Unknown, score: 0
Calr4Unknown, score: 0.1
Ccdc122Unknown, score: 0.09
Mtrf1lUnknown, score: 0.32
Atad3aUnknown, score: 0.03
2700081O15RikUnknown, score: 0.22
TbcdUnknown, score: 0.41
Nusap1Unknown, score: 0.28
Rcc2Unknown, score: 0.21
Cdca2Unknown, score: 0.2
LhfpUnknown, score: 0.19
Rnf169Unknown, score: 0.38
Ppp1r15bUnknown, score: 0.06
Fam73bUnknown, score: 0.07
E2f8Unknown, score: 0.16
Ciapin1Unknown, score: 0.56
Sp110Unknown, score: 0.17
Fbxw17Unknown, score: 0.73
Rbm15bUnknown, score: 0.03
Slc30a9Unknown, score: 0.12
Uhrf2Unknown, score: 0.35
Supt3Unknown, score: 0.29
MmaaUnknown, score: 0.5
Chd9Unknown, score: 0.05
MlecUnknown, score: 0.05
Ube2q2Unknown, score: 0.27
Atl3Unknown, score: 0.19
Trip11Unknown, score: 0.17
SobpUnknown, score: 0.34
Ms4a7Unknown, score: 0.09
SccpdhUnknown, score: 0.03
Kif24Unknown, score: 0.3
Tspan9Unknown, score: 0.12
AdtrpUnknown, score: 0.35
Ssc4dUnknown, score: 0.04
Prr5Unknown, score: 0.26
R3hdm4Unknown, score: 0.4
Orai1Unknown, score: 0.25
C1qtnf7Unknown, score: 0.23
Rnf20Unknown, score: 0.22
Cdcp1Unknown, score: 0.29
Fam163bUnknown, score: 0.13
Fam175bUnknown, score: 0.58
SriUnknown, score: 0.01
Lmo1Unknown, score: 0.04
DspUnknown, score: 0.2
TxlnaUnknown, score: 0.04
CtrlUnknown, score: -0.01
Ampd2Unknown, score: 0.01
Cpa1Unknown, score: 0.49
Itga1Unknown, score: 0.1
Cyb5r3Unknown, score: 0.2
BlvraUnknown, score: 0.42
Pgm3Unknown, score: 0.5
Cbr3Unknown, score: 0.24
Mzf1Unknown, score: 0.04
Cela1Unknown, score: 0.28
Mcf2Unknown, score: 0.5
Rap1aUnknown, score: 0.24
AbrUnknown, score: 0.45
Art4Unknown, score: 0.42
Tpgs1Unknown, score: 0.2
Kif22Unknown, score: 0.29
DekUnknown, score: 0.09
Syt17Unknown, score: -0.01
Bmp3Unknown, score: 0.37
PygbUnknown, score: 0.05
110083Unknown, score: 0.17
Phka2Unknown, score: 0.6
MpiUnknown, score: 0.43
FgbUnknown, score: 0
Raf1Unknown, score: 0.04
Gpr18Unknown, score: 0.22
Slc35b1Unknown, score: 0.29
ManbaUnknown, score: 0.35
TriobpUnknown, score: 0.1
MsraUnknown, score: 0
Cox6b1Unknown, score: 0.06
Tas1r1Unknown, score: 0.23
Pp2d1Unknown, score: 0.02
Sec13Unknown, score: 0.07
Shroom2Unknown, score: 0.36
PighUnknown, score: 0.06
110558Unknown, score: 0.05
Prdm2Unknown, score: 0.41
Timp4Unknown, score: -0.01
HdlbpUnknown, score: 0.11
Grik4Unknown, score: 0.31
Prps2Unknown, score: 0.27
Rps6ka3Unknown, score: 0.19
Aldh7a1Unknown, score: 0.02
Chaf1bUnknown, score: 0
Adgrv1Unknown, score: 0.01
Pwp2Unknown, score: 0.17
PccaUnknown, score: 0.03
Ppp2r4Unknown, score: 0
Scn2a1Unknown, score: 0.01
Slc18a1Unknown, score: 0.19
Slc8a3Unknown, score: 0.05
Slc9a4Unknown, score: 0.71
Cds2Unknown, score: 0.18
M1apUnknown, score: 0.43
Mbd6Unknown, score: 0.11
Egln3Unknown, score: 0.02
1700102P08RikUnknown, score: 0.21
2010002M12RikUnknown, score: 0.39
Acaa1aUnknown, score: 0.17
Laptm4bUnknown, score: 0.22
Foxp2Unknown, score: 0
Atp6v0bUnknown, score: 0.07
NpntUnknown, score: 0.17
Slc5a5Unknown, score: 0.17
CsprsUnknown, score: 0.07
D17H6S53EUnknown, score: 0.06
Prdm15Unknown, score: 0
Tle6Unknown, score: 0.07
Rpl31Unknown, score: 0.02
Ly6g6dUnknown, score: 0.17
Impa2Unknown, score: 0.52
Hsd17b11Unknown, score: 0
SelmUnknown, score: 0.34
Rad51cUnknown, score: 0.51
Spred1Unknown, score: 0.17
Spred2Unknown, score: 0.43
Supt16Unknown, score: 0.14
PawrUnknown, score: 0.05
ProscUnknown, score: 0.24
CygbUnknown, score: 0.85
Pcdha1Unknown, score: 0.15
Tsga13Unknown, score: 0.08
Lsm10Unknown, score: 0.18
Rims2Unknown, score: 0.15
Mta3Unknown, score: 0.33
Stim2Unknown, score: 0.23
Alpk3Unknown, score: 0.03
Dph1Unknown, score: 0.32
Pnpla3Unknown, score: 0.32
Fam57aUnknown, score: 0.01
Ube3bUnknown, score: 0.27
Bloc1s4Unknown, score: 0.34
Ivns1abpUnknown, score: 0.16
A1bgUnknown, score: 0.09
Asb10Unknown, score: 0.24
B3galt6Unknown, score: 0.04
Klf16Unknown, score: 0.37
Synpo2Unknown, score: 0.18
Mrps6Unknown, score: 0.51
Zfp358Unknown, score: 0.07
Igf2bp3Unknown, score: 0.14
AF251705Unknown, score: 0.09
Rxfp2Unknown, score: 0.26
Elmo2Unknown, score: 0.03
Cacng5Unknown, score: 0.29
Sesn1Unknown, score: 0.16
Bmp2kUnknown, score: 0.14
Myh7Unknown, score: 0.46
Colec12Unknown, score: 0.17
P2ry14Unknown, score: 0.08
Il25Unknown, score: 0.03
Lnx2Unknown, score: 0
Zcchc14Unknown, score: 0.08
Asb14Unknown, score: 0.2
Asb13Unknown, score: 0.13
Tlr3Unknown, score: 0.1
Elovl6Unknown, score: 0.03
Slc2a10Unknown, score: 0.16
Stard4Unknown, score: 0.24
Recql5Unknown, score: 0.06
Grin3bUnknown, score: 0.13
Sp7Unknown, score: 0.12
Ubn1Unknown, score: 0.28
Idh3bUnknown, score: 0.12
PaplnUnknown, score: 0.09
Znrf1Unknown, score: 0.31
Kcnh7Unknown, score: 0.11
Zfp287Unknown, score: 0.02
Pilrb1Unknown, score: 0.08
Sertad3Unknown, score: 0.06
Smco4Unknown, score: 0.05
Mtmr4Unknown, score: 0.1
Xpnpep1Unknown, score: 0.05
Zfp704Unknown, score: 0.07
Slc8b1Unknown, score: 0.26
Atp13a1Unknown, score: 0.66
Acbd3Unknown, score: 0.21
Pdzd3Unknown, score: -0.01
Nup155Unknown, score: 0.11
Glcci1Unknown, score: 0.15
Cd209dUnknown, score: 0.24
Acot8Unknown, score: 0.28
Rbm39Unknown, score: 0.19
Rtkn2Unknown, score: 0.13
Usp33Unknown, score: 0.56
GlmnUnknown, score: 0.01
Inpp5jUnknown, score: 0.16
Zfp369Unknown, score: 0.27
Zfp617Unknown, score: 0.16
Myoz3Unknown, score: 0.07
Acer1Unknown, score: 0.23
Asic3Unknown, score: 0.3
Galnt10Unknown, score: 0.25
Acot3Unknown, score: 0.32
Acot4Unknown, score: 0.32
Havcr2Unknown, score: 0.04
Slc12a8Unknown, score: 0.03
Cd99l2Unknown, score: 0.04
ApobrUnknown, score: 0.01
BmfUnknown, score: 0.14
Nme7Unknown, score: 0.22
Mical1Unknown, score: 0.03
HelqUnknown, score: 0.01
Dicer1Unknown, score: 0
BspryUnknown, score: 0.19
Prpf8Unknown, score: 0.07
Pcdha12Unknown, score: -0.01
SardhUnknown, score: 0.28
Stab1Unknown, score: 0.25
Stab2Unknown, score: 0.06
Shkbp1Unknown, score: 0.08
Edem1Unknown, score: 0.01
Ash1lUnknown, score: 0.18
Lrrc4Unknown, score: 0.02
Prom2Unknown, score: 0.13
Hps4Unknown, score: 0.02
Hps1Unknown, score: 0.15
Phf21aUnknown, score: 0.12
Slc25a36Unknown, score: 0.21
Cabp7Unknown, score: 0.35
Pla2g15Unknown, score: 0.19
Ripk2Unknown, score: 0.48
Ell2Unknown, score: 0
Rassf3Unknown, score: 0.27
Lrrc75bUnknown, score: 0.47
Kcnh6Unknown, score: 0.04
Dhrs11Unknown, score: 0.12
Lrrc75aUnknown, score: 0.02
Trpv1Unknown, score: 0.07
Zfp3Unknown, score: 0.45
BC049762Unknown, score: 0.36
Zbtb12Unknown, score: 0.15
Mcfd2Unknown, score: 0.03
Eme2Unknown, score: 0.27
Mtmr11Unknown, score: 0.22
Cnksr1Unknown, score: 0.46
Tet3Unknown, score: 0.04
Mical3Unknown, score: 0.2
Reps2Unknown, score: 0
Klf11Unknown, score: 0.02
195209Unknown, score: 0.13
Zfp691Unknown, score: 0.44
Gm13152Unknown, score: 0.14
Skint3Unknown, score: 0.61
NhsUnknown, score: 0.32
BptfUnknown, score: 0.28
Cetn4Unknown, score: 0.22
Arhgef17Unknown, score: 0.26
Larp4Unknown, score: 0.07
Zbtb7cUnknown, score: 0.32
Fchsd2Unknown, score: 0.07
Hectd1Unknown, score: 0.04
Elfn2Unknown, score: 0.17
Wdr11Unknown, score: 0.53
Baiap2l2Unknown, score: 0.25
Igsf11Unknown, score: 0.12
Cfap69Unknown, score: 0.11
C2cd2Unknown, score: 0.05
BC034090Unknown, score: -0.01
Gm608Unknown, score: 0.06
SmagpUnknown, score: 0.06
Klhl25Unknown, score: 0.31
Alg11Unknown, score: 0.61
Pknox2Unknown, score: 0.15
Ubap1lUnknown, score: 0.2
MlxipUnknown, score: 0.18
9830147E19RikUnknown, score: 0.44
Aph1bUnknown, score: 0.04
Dhx37Unknown, score: 0.02
Yeats2Unknown, score: 0.01
BtlaUnknown, score: 0.16
Fam180aUnknown, score: 0.68
Btbd2Unknown, score: 0.15
Zfp871Unknown, score: 0.73
Dip2cUnknown, score: 0.17
Mterf1bUnknown, score: 0.53
Rsrc2Unknown, score: 0.25
Alg3Unknown, score: 0.15
Kntc1Unknown, score: 0.09
Tspan10Unknown, score: 0.09
Slc25a38Unknown, score: 0.19
Eif4g1Unknown, score: 0.17
Creb3l2Unknown, score: -0.01
Fam20aUnknown, score: 0.28
Akr1d1Unknown, score: 0.2
Creb3l3Unknown, score: 0.47
Eif5a2Unknown, score: 0.02
Hdac4Unknown, score: 0.18
Prrg3Unknown, score: 0.13
Tmem63aUnknown, score: 0.11
FanciUnknown, score: 0.15
Dock3Unknown, score: 0.06
Zdhhc9Unknown, score: 0.07
Slc26a7Unknown, score: 0.06
Unc13cUnknown, score: 0.1
Ccdc62Unknown, score: 0.04
Adamts18Unknown, score: 0.17
Thnsl1Unknown, score: 0.28
Zfp280cUnknown, score: 0.09
NpbUnknown, score: 0.33
Ulk4Unknown, score: 0.07
Pycr1Unknown, score: 0.24
Gipc3Unknown, score: 0.39
Samd9lUnknown, score: 0.29
Snx30Unknown, score: 0.07
Clic6Unknown, score: 0.23
Dtx3lUnknown, score: 0.3
Osgin2Unknown, score: 0.2
GanUnknown, score: 0.28
Gps1Unknown, score: 0.07
Gen1Unknown, score: 0.15
Gtf2h3Unknown, score: 0.54
Taf3Unknown, score: 0.05
Trim30dUnknown, score: 0.25
Trp53bp2Unknown, score: 0.29
Hace1Unknown, score: 0.16
Tmem164Unknown, score: 0.01
Rgag1Unknown, score: 0.17
Enpp3Unknown, score: 0.07
Erich3Unknown, score: 0.02
Dhtkd1Unknown, score: 0.1
LcorlUnknown, score: 0.25
Kif15Unknown, score: 0.07
Tbc1d25Unknown, score: 0.34
Pgbd5Unknown, score: 0
MetrnlUnknown, score: 0.14
Iglon5Unknown, score: 0.15
Zfp658Unknown, score: 0.11
D130043K22RikUnknown, score: 0.06
LppUnknown, score: 0.05
Shank2Unknown, score: 0.15
Dock10Unknown, score: 0.36
Thsd7bUnknown, score: 0.04
P3h2Unknown, score: 0.16
Prrt3Unknown, score: 0.2
ThemisUnknown, score: 0.04
Ppp1r36Unknown, score: 0.11
Brcc3Unknown, score: 0.15
Tbc1d4Unknown, score: 0.14
Lacc1Unknown, score: 0.25
Zfp947Unknown, score: 0.04
Ints9Unknown, score: 0.15
4931408C20RikUnknown, score: 0.05
Gltscr1lUnknown, score: 0.11
Lpcat1Unknown, score: 0.03
Trim41Unknown, score: 0.01
Lzts1Unknown, score: 0.24
D130040H23RikUnknown, score: 0.17
March11Unknown, score: 0.11
Churc1Unknown, score: 0.29
Lrtm2Unknown, score: 0.22
Cpne9Unknown, score: 0.16
Mtrf1Unknown, score: 0.09
Kbtbd7Unknown, score: 0.19
Cln5Unknown, score: 0.62
Nrg1Unknown, score: 0.34
Ncoa7Unknown, score: 0.22
SuoxUnknown, score: 0.1
Exoc3Unknown, score: 0.15
Kcnj14Unknown, score: 0.13
EfhbUnknown, score: 0.44
AdoUnknown, score: 0.09
Tmem87aUnknown, score: -0.01
Nomo1Unknown, score: 0.57
TifaUnknown, score: 0.04
Plac9aUnknown, score: 0.06
Fancd2Unknown, score: 0.36
Wwc1Unknown, score: 0.59
Cspp1Unknown, score: 0.14
Mgst2Unknown, score: 0.18
Arfgef1Unknown, score: 0.21
Vstm2aUnknown, score: 0.02
Trib1Unknown, score: 0.2
Asap2Unknown, score: 0.44
Spsb4Unknown, score: 0.13
Tmem60Unknown, score: 0
Cc2d1aUnknown, score: 0.15
8030462N17RikUnknown, score: 0.2
Wdr25Unknown, score: 0.33
Zfp729aUnknown, score: 0.22
Arap2Unknown, score: 0.23
Mapre2Unknown, score: 0.28
Fam149aUnknown, score: 0.12
Mms22lUnknown, score: 0.39
Frat2Unknown, score: 0.38
AA986860Unknown, score: 0.4
Lactb2Unknown, score: 0.32
Mtg1Unknown, score: 0.09
Spice1Unknown, score: 0.18
Trmt1Unknown, score: 0.17
Sh3bgrl2Unknown, score: 0.19
RhoUnknown, score: 0.42
Pqlc2Unknown, score: 0.5
Zfp273Unknown, score: 0.23
Prpsap2Unknown, score: 0.1
Aldh4a1Unknown, score: 0.19
Mars2Unknown, score: 0.12
Gm17296Unknown, score: 0.1
Ccdc64bUnknown, score: 0.2
Arl14epUnknown, score: 0.51
Chpt1Unknown, score: 0.25
Rsph4aUnknown, score: 0.11
DseUnknown, score: 0.28
Kctd7Unknown, score: 0.33
Slc45a3Unknown, score: 0.11
Scfd2Unknown, score: 0.17
Tnpo2Unknown, score: 0.05
Ifitm6Unknown, score: 0.04
Mfsd4Unknown, score: 0.28
Evi5lUnknown, score: 0.46
Slc39a14Unknown, score: 0.05
Gabpb2Unknown, score: 0.15
Wdr19Unknown, score: 0.24
Cdkl3Unknown, score: 0.2
Phf3Unknown, score: 0.08
213119Unknown, score: 0.07
Rnf26Unknown, score: 0.01
Fbxl21Unknown, score: 0.1
Pddc1Unknown, score: 0.07
Rassf4Unknown, score: 0.04
8430408G22RikUnknown, score: 0.42
Zcchc5Unknown, score: 0.03
A630033H20RikUnknown, score: 0.11
DstykUnknown, score: -0.01
Lgi3Unknown, score: 0.09
Arhgef11Unknown, score: 0.03
Ythdf2Unknown, score: 0.02
Dis3lUnknown, score: 0.12
Cracr2bUnknown, score: 0.17
Dync2li1Unknown, score: 0.37
Slc44a3Unknown, score: 0.36
Arhgef19Unknown, score: 0.05
Zfp598Unknown, score: 0.03
Tbl3Unknown, score: 0.25
Plekhg1Unknown, score: 0.37
Casd1Unknown, score: 0.21
Arcn1Unknown, score: 0.49
Bms1Unknown, score: 0.38
Fbxw10Unknown, score: 0
Tnrc6bUnknown, score: 0.16
Agap3Unknown, score: 0.67
Ccdc186Unknown, score: 0.48
Larp1bUnknown, score: 0.45
Dnajc16Unknown, score: 0
Slc18a2Unknown, score: 0.23
4933430I17RikUnknown, score: 0.08
Slc24a1Unknown, score: 0.21
Tet2Unknown, score: 0.24
Arhgap29Unknown, score: 0.19
Ago3Unknown, score: 0.33
Disp2Unknown, score: 0.23
Etnk2Unknown, score: 0.04
Nudt15Unknown, score: 0.3
Zcchc6Unknown, score: 0.02
Hhipl1Unknown, score: -0.01
Gm4787Unknown, score: 0.16
Parp16Unknown, score: 0.01
Cdk5rap2Unknown, score: 0.07
Fam168bUnknown, score: 0.71
Cdc73Unknown, score: 0.02
GnptgUnknown, score: 0.31
Tmprss13Unknown, score: 0.14
SheUnknown, score: 0.01
PstkUnknown, score: 0.06
Spg11Unknown, score: 0.04
4930486L24RikUnknown, score: 0.61
ChadlUnknown, score: 0.13
IqubUnknown, score: 0.11
Rcor3Unknown, score: 0.4
Mb21d1Unknown, score: 0.15
Mmp21Unknown, score: 0.19
Zfp879Unknown, score: 0.2
Zfp609Unknown, score: 0.07
Arid5aUnknown, score: 0.15
Lman2lUnknown, score: 0.29
Csnk1g1Unknown, score: 0.32
Kdm5aUnknown, score: 0.19
Fam173aUnknown, score: 0.05
Cecr5Unknown, score: 0.22
Wfikkn1Unknown, score: 0.17
Vgll2Unknown, score: 0.17
Slc35f1Unknown, score: 0.21
ManealUnknown, score: 0.2
Slc43a2Unknown, score: 0.15
DiexfUnknown, score: 0.1
Kri1Unknown, score: 0.15
Il1f9Unknown, score: 0.19
Wipf1Unknown, score: 0.13
Slc36a1Unknown, score: 0.12
Brinp3Unknown, score: 0.4
Csrnp1Unknown, score: 0.21
Rab11fip3Unknown, score: 0.08
Entpd3Unknown, score: 0.02
Rap1bUnknown, score: 0.14
Rassf2Unknown, score: 0.1
Nav1Unknown, score: 0.4
Ccdc92Unknown, score: 0.02
Mfsd6lUnknown, score: 0.28
Cnksr3Unknown, score: 0.23
Ginm1Unknown, score: 0.06
AdgbUnknown, score: 0.07
Phactr2Unknown, score: 0.39
Adgrg6Unknown, score: 0.23
Arfgef3Unknown, score: 0.06
BC021785Unknown, score: 0.18
AI317395Unknown, score: 0.13
Lace1Unknown, score: 0.3
Lrrc20Unknown, score: 0.09
Ctnna3Unknown, score: 0.11
Zfp365Unknown, score: 0.53
Trappc10Unknown, score: 0.03
IlvblUnknown, score: 0.04
Abhd17aUnknown, score: 0.07
AU041133Unknown, score: 0.17
Appl2Unknown, score: 0.58
Tcp11l2Unknown, score: 0.22
Slc17a8Unknown, score: 0.08
Socs2Unknown, score: 0.14
Eea1Unknown, score: 0.34
Cep290Unknown, score: 0.34
Rab21Unknown, score: 0.35
Zfc3h1Unknown, score: 0.47
Tspan8Unknown, score: 0.01
Rab3ipUnknown, score: 0.4
Agap2Unknown, score: 0.15
MarsUnknown, score: 0.38
Myl6bUnknown, score: 0.03
Cep68Unknown, score: 0.03
LgalslUnknown, score: 0.59
Ugp2Unknown, score: 0.2
WdpcpUnknown, score: 0.13
Ehbp1Unknown, score: 0.31
PapolgUnknown, score: 0.28
Efemp1Unknown, score: 0.62
Hbq1aUnknown, score: 0.34
Clint1Unknown, score: 0.16
Adamts2Unknown, score: 0.17
Acsl6Unknown, score: 0.14
Gemin5Unknown, score: 0.09
Wnt9aUnknown, score: -0.01
Tmem11Unknown, score: 0.14
Usp22Unknown, score: 0.25
CntrobUnknown, score: 0.17
Kctd11Unknown, score: 0.01
Neurl4Unknown, score: 0.01
Mgl2Unknown, score: 0.08
Arrb2Unknown, score: 0.07
Gltpd2Unknown, score: 0.11
Spns2Unknown, score: 0.09
Trp53i13Unknown, score: 0.2
Fam222bUnknown, score: 0.48
Proca1Unknown, score: 0.19
216976Unknown, score: 0.12
Utp6Unknown, score: 0.03
Mrm1Unknown, score: 0.16
Ggnbp2Unknown, score: 0.07
Ptrh2Unknown, score: 0.2
Gm525Unknown, score: 0.28
HlfUnknown, score: 0.13
Xylt2Unknown, score: 0.16
Ppp1r9bUnknown, score: -0.01
Samd14Unknown, score: -0.01
Kat7Unknown, score: 0.01
Scrn2Unknown, score: 0.09
Nr1d1Unknown, score: 0.11
Rundc1Unknown, score: 0.01
Tmem106aUnknown, score: -0.01
Dhx8Unknown, score: 0.15
BC030867Unknown, score: 0.02
Fam171a2Unknown, score: 0.25
Cdc27Unknown, score: 0.22
Abca8aUnknown, score: 0.14
Abca9Unknown, score: 0.07
Abca5Unknown, score: 0.17
Cd300eUnknown, score: 0.03
Rnf157Unknown, score: 0.13
Rhbdf2Unknown, score: 0.06
Tmc8Unknown, score: 0.03
Rab40bUnknown, score: 0.15
Dnajc27Unknown, score: 0.11
Trib2Unknown, score: 0.28
Trappc12Unknown, score: 0.43
Snx13Unknown, score: 0.43
DgkbUnknown, score: 0.14
Stxbp6Unknown, score: 0.42
G2e3Unknown, score: 0.33
Baz1aUnknown, score: 0.2
MbipUnknown, score: 0.03
Slc25a21Unknown, score: 0.64
Ctage5Unknown, score: 0.37
Gm527Unknown, score: 0.38
Mis18bp1Unknown, score: 0.06
Susd6Unknown, score: 0.07
Zfyve1Unknown, score: 0.37
Acot6Unknown, score: 0.29
Coq6Unknown, score: 0.41
Eif2b2Unknown, score: 0.12
Nek9Unknown, score: -0.01
CipcUnknown, score: 0.13
Pomt2Unknown, score: 0.09
Ahsa1Unknown, score: 0.08
Ism2Unknown, score: 0.17
Itpk1Unknown, score: 0.22
Ifi27l2bUnknown, score: 0.33
Serpina10Unknown, score: 0
Cdc42bpbUnknown, score: 0.5
BC022687Unknown, score: 0.16
Pacs2Unknown, score: 0.2
Wdr60Unknown, score: -0.01
Rapgef5Unknown, score: 0.08
Cdca7lUnknown, score: 0.23
Larp4bUnknown, score: 0.12
Heatr1Unknown, score: 0.53
Vps41Unknown, score: 0.54
AmphUnknown, score: 0.43
Zfp322aUnknown, score: 0.23
Slc17a2Unknown, score: 0.2
Mboat1Unknown, score: 0.09
MylipUnknown, score: 0.06
Nup153Unknown, score: 0.18
Ptpdc1Unknown, score: 0.04
B4galt7Unknown, score: 0.28
Cdc14bUnknown, score: 0.4
Zfp455Unknown, score: 0.17
Zfp595Unknown, score: 0.14
Ice1Unknown, score: 0.01
Clptm1lUnknown, score: 0.03
RfesdUnknown, score: 0.01
Ttc37Unknown, score: 0.18
Rasa1Unknown, score: 0.1
Zfyve16Unknown, score: 0.14
Serinc5Unknown, score: -0.01
Lhfpl2Unknown, score: 0.45
Gcnt4Unknown, score: 0.45
Btf3Unknown, score: 0.38
Fcho2Unknown, score: 0.11
Mrps27Unknown, score: 0.08
Marveld2Unknown, score: 0.54
Srek1Unknown, score: 0.24
SgtbUnknown, score: 0.02
Depdc1bUnknown, score: 0.44
Mier3Unknown, score: 0.07
CcnoUnknown, score: 0.52
Arl15Unknown, score: 0
Paip1Unknown, score: 0.29
PxkUnknown, score: 0.51
3830406C13RikUnknown, score: 0.39
Polr3aUnknown, score: 0.44
Fam208aUnknown, score: 0.25
Oxnad1Unknown, score: 0.29
WapalUnknown, score: 0.17
Fermt2Unknown, score: 0.08
Wdhd1Unknown, score: 0.16
Mapk1ip1lUnknown, score: 0.2
KhnynUnknown, score: 0.46
CenpjUnknown, score: 0.68
Zmym5Unknown, score: 0.21
Phf11aUnknown, score: 0.22
Shisa2Unknown, score: 0.09
Mtmr6Unknown, score: 0.25
Fam167aUnknown, score: 0.16
Ccar2Unknown, score: 0.15
Akap11Unknown, score: 0.45
Vwa8Unknown, score: 0.18
Pcdh17Unknown, score: 0.48
Tdrd3Unknown, score: 0.08
Farp1Unknown, score: 0.17
GgactUnknown, score: 0.63
Itgbl1Unknown, score: 0.36
Ugt3a2Unknown, score: 0.04
DapUnknown, score: 0
March6Unknown, score: 0.13
Nipal2Unknown, score: 0.3
AbraUnknown, score: 0.09
E430025E21RikUnknown, score: 0.33
Lrrc24Unknown, score: 0.07
Arhgap39Unknown, score: 0.15
Tmem184bUnknown, score: 0.08
McatUnknown, score: 0.01
Tbc1d22aUnknown, score: 0.01
SeloUnknown, score: 0.14
Gxylt1Unknown, score: 0.39
Pphln1Unknown, score: 0.29
Krt79Unknown, score: 0.1
Soat2Unknown, score: 0.05
AaasUnknown, score: 0.06
Marf1Unknown, score: 0.22
SpidrUnknown, score: 0.31
Scarf2Unknown, score: 0.01
Eif2b5Unknown, score: 0.17
Atp13a4Unknown, score: 0.1
Tmem44Unknown, score: 0.23
Fam43aUnknown, score: 0.18
NrrosUnknown, score: 0.21
Adcy5Unknown, score: 0.05
Dirc2Unknown, score: 0.42
Golgb1Unknown, score: 0.01
Poglut1Unknown, score: 0.08
Dzip3Unknown, score: 0.33
C330027C09RikUnknown, score: 0.05
Impg2Unknown, score: 0.18
Cldnd1Unknown, score: 0.05
Rbm11Unknown, score: 0.4
Cyyr1Unknown, score: 0.1
Map3k7clUnknown, score: 0.11
Setd4Unknown, score: 0.11
Zdhhc14Unknown, score: 0.37
Tfb1mUnknown, score: 0.01
Acat3Unknown, score: 0.17
Zfp160Unknown, score: 0.16
Slc37a1Unknown, score: 0.23
Zfp472Unknown, score: 0.53
Bag6Unknown, score: 0.07
Adgrf5Unknown, score: 0.26
Enpp4Unknown, score: 0.17
Abcc10Unknown, score: 0.09
Rrp36Unknown, score: 0.07
Pex6Unknown, score: 0.04
224829Unknown, score: -0.01
AI661453Unknown, score: 0.21
Treml4Unknown, score: 0.08
Plcl2Unknown, score: 0.55
Dpp9Unknown, score: 0.07
Crb3Unknown, score: 0.12
Dlgap1Unknown, score: 0.3
Fez2Unknown, score: 0.34
Srsf7Unknown, score: 0.31
Ttc7Unknown, score: 0.38
Fbxo11Unknown, score: 0.2
SvilUnknown, score: 0.2
WacUnknown, score: 0.33
Thoc1Unknown, score: 0.11
Mib1Unknown, score: 0.34
Rbbp8Unknown, score: 0.07
Zfp521Unknown, score: 0.34
Rsl24d1Unknown, score: 0.55
Ino80cUnknown, score: 0.2
Fhod3Unknown, score: 0.26
AW554918Unknown, score: 0.34
Ammecr1lUnknown, score: 0.09
Lims2Unknown, score: 0.5
Wdr36Unknown, score: 0.25
Reep2Unknown, score: 0.19
Rell2Unknown, score: 0.42
Pggt1bUnknown, score: -0.01
Prdm6Unknown, score: 0.19
Cep120Unknown, score: 0.41
Slc27a6Unknown, score: 0.2
Gm4841Unknown, score: 0.42
Sh3tc2Unknown, score: 0.4
Cep76Unknown, score: 0.09
Mapk4Unknown, score: 0.05
Rnf165Unknown, score: 0.26
Haus1Unknown, score: 0.1
Cd226Unknown, score: 0.35
Ndufs8Unknown, score: 0
Taf6lUnknown, score: 0.17
Eml3Unknown, score: 0.4
Cyb561a3Unknown, score: 0
Patl1Unknown, score: 0
D030056L22RikUnknown, score: 0
Trpm6Unknown, score: 0.53
RorbUnknown, score: 0.29
Trpm3Unknown, score: 0.64
Pgm5Unknown, score: 0.08
Glis3Unknown, score: 0.08
Ric1Unknown, score: 0.12
Ermp1Unknown, score: 0.54
Hectd2Unknown, score: 0.05
MyofUnknown, score: 0.38
Ubtd1Unknown, score: 0.05
Cox15Unknown, score: 0.05
Cyp2c44Unknown, score: 0.06
Erlin1Unknown, score: 0.1
Lzts2Unknown, score: 0.04
Wbp1lUnknown, score: 0.23
InaUnknown, score: 0.07
Taf5Unknown, score: 0.28
Afap1l2Unknown, score: 0.28
Ablim1Unknown, score: 0.35
Atrnl1Unknown, score: 0.03
Eno4Unknown, score: 0
Rab3gap1Unknown, score: 0
LctUnknown, score: 0.23
Yod1Unknown, score: 0.06
Rab29Unknown, score: 0.17
Ipo9Unknown, score: 0.14
Zfp281Unknown, score: 0.03
Zbtb41Unknown, score: 0.68
Lamc1Unknown, score: 0.03
Aph1aUnknown, score: 0.2
SucoUnknown, score: 0.08
Fmo4Unknown, score: 0.04
TiprlUnknown, score: 0.19
Rcsd1Unknown, score: 0.16
Atf6Unknown, score: -0.01
Ndufs2Unknown, score: 0.03
Tstd1Unknown, score: 0.12
AI607873Unknown, score: 0.28
CnstUnknown, score: 0
Ahctf1Unknown, score: 0.04
Cdc42bpaUnknown, score: 0.05
Wdr26Unknown, score: 0.05
Mark1Unknown, score: 0.2
Kctd3Unknown, score: 0.01
Mfsd7bUnknown, score: 0.1
HhatUnknown, score: 0.01
Kansl3Unknown, score: -0.01
Actr1bUnknown, score: 0.57
Eif5bUnknown, score: 0.47
Slc9a2Unknown, score: 0.12
Dnah7bUnknown, score: 0.14
Slc39a10Unknown, score: 0.16
HibchUnknown, score: 0.33
Pms1Unknown, score: -0.01
Ormdl1Unknown, score: 0.23
Plcl1Unknown, score: 0.04
StradbUnknown, score: 0.18
Ino80dUnknown, score: 0.09
Ndufs1Unknown, score: 0.14
Ccnyl1Unknown, score: 0.36
Cps1Unknown, score: 0.06
Fam134aUnknown, score: 0.07
DnerUnknown, score: -0.01
B3gnt7Unknown, score: 0.14
DgkdUnknown, score: 0.73
Usp40Unknown, score: 0.13
Fam132bUnknown, score: 0.05
2310035C23RikUnknown, score: 0.19
Zcchc2Unknown, score: 0.64
Cdh19Unknown, score: 0.5
Rpp38Unknown, score: 0.37
CdnfUnknown, score: 0.24
Man1b1Unknown, score: 0.15
Uap1l1Unknown, score: 0.04
BC029214Unknown, score: 0.04
Rabl6Unknown, score: 0.29
Sec16aUnknown, score: 0.13
Sh3glb2Unknown, score: 0.65
BC005624Unknown, score: 0.06
GsnUnknown, score: 0.08
Dennd1aUnknown, score: 0.32
Gtdc1Unknown, score: 0.23
CytipUnknown, score: 0.25
Ccdc148Unknown, score: 0.69
Pkp4Unknown, score: 0.23
GcaUnknown, score: 0.04
Klhl41Unknown, score: 0.71
PpigUnknown, score: 0.11
Tlk1Unknown, score: 0.36
Mettl8Unknown, score: 0.35
Atp5g3Unknown, score: 0.23
Sestd1Unknown, score: 0.09
CerklUnknown, score: 0.14
Zdhhc5Unknown, score: 0.04
P2rx3Unknown, score: 0.04
Slc35c1Unknown, score: 0.26
Kif18aUnknown, score: 0.03
Ano3Unknown, score: 0.28
Arhgap11aUnknown, score: 0.19
Bahd1Unknown, score: 0.04
ItpkaUnknown, score: 0
Frmd5Unknown, score: 0.32
MallUnknown, score: 0.49
SmoxUnknown, score: 0
Gm561Unknown, score: 0.18
Sdcbp2Unknown, score: 0.14
PiguUnknown, score: 0.07
Phf20Unknown, score: 0.36
Dlgap4Unknown, score: 0.1
Tgif2Unknown, score: 0.12
Ppp1r16bUnknown, score: 0.02
Slc35c2Unknown, score: 0.17
Zmynd8Unknown, score: 0.22
Ddx27Unknown, score: 0.17
Zfp217Unknown, score: 0.16
Npepl1Unknown, score: -0.01
Ppp1r3dUnknown, score: 0.07
Samd10Unknown, score: 0.35
Zbtb10Unknown, score: 0.11
Ythdf3Unknown, score: 0.16
Acad9Unknown, score: 0.13
QrfprUnknown, score: 0.02
4932438A13RikUnknown, score: 0.44
Stoml3Unknown, score: 0.01
Spg20Unknown, score: 0.11
Tm4sf4Unknown, score: 0.35
Eif2aUnknown, score: 0.16
Clrn1Unknown, score: -0.01
Gpr171Unknown, score: 0.08
GmpsUnknown, score: 0.12
CtsoUnknown, score: 0.08
Fhdc1Unknown, score: 0.22
Fcrl1Unknown, score: 0.23
Rrnad1Unknown, score: 0.19
Syt11Unknown, score: 0.23
PruneUnknown, score: 0.2
CiartUnknown, score: 0.12
BC028528Unknown, score: 0.12
Otud7bUnknown, score: 0.06
Trim45Unknown, score: 0
Vangl1Unknown, score: 0.32
Bcl2l15Unknown, score: 0.08
Rsbn1Unknown, score: 0.28
CymUnknown, score: 0.37
Rbm15Unknown, score: 0.23
Ahcyl1Unknown, score: 0.11
Amigo1Unknown, score: 0.2
5330417C22RikUnknown, score: 0.04
Clcc1Unknown, score: 0.22
Slc25a24Unknown, score: 0.25
Cdc14aUnknown, score: 0.11
Trmt13Unknown, score: 0.28
Slc35a3Unknown, score: 0.46
Lppr4Unknown, score: 0.15
Tram1l1Unknown, score: 0.4
CenpeUnknown, score: 0.36
Rap1gds1Unknown, score: 0.08
Gbp5Unknown, score: 0.04
Gbp7Unknown, score: 0.18
Ccbl2Unknown, score: 0.07
Gtf2bUnknown, score: 0.21
Clca2Unknown, score: 0.57
Ak5Unknown, score: 0.27
Coq3Unknown, score: 0.24
Nol6Unknown, score: 0.16
Fam214bUnknown, score: 0.09
Car9Unknown, score: 0.01
Galnt12Unknown, score: 0.49
Tmeff1Unknown, score: 0.24
Zfp189Unknown, score: 0.08
AldobUnknown, score: 0.24
IkbkapUnknown, score: 0.25
Frrs1lUnknown, score: 0.12
AI314180Unknown, score: 0.15
Ptbp3Unknown, score: 0.01
6330416G13RikUnknown, score: 0.22
Megf9Unknown, score: 0.28
FocadUnknown, score: 0.67
LeprotUnknown, score: 0.36
C8aUnknown, score: 0.03
Glis1Unknown, score: 0.11
Zcchc11Unknown, score: 0.21
Prpf38aUnknown, score: 0.07
Nrd1Unknown, score: 0.26
Ttc39aUnknown, score: 0.08
Lrrc41Unknown, score: 0.25
Szt2Unknown, score: 0.19
Tmem125Unknown, score: 0.03
AU022252Unknown, score: 0.04
Foxj3Unknown, score: 0.56
Nt5c1aUnknown, score: 0.09
YrdcUnknown, score: 0.28
Zc3h12aUnknown, score: 0.24
Eva1bUnknown, score: 0.2
Fam167bUnknown, score: 0.15
Sesn2Unknown, score: 0.18
Aim1lUnknown, score: 0.2
Pdik1lUnknown, score: 0.41
Slc30a2Unknown, score: 0.17
Ece1Unknown, score: 0.02
Emc1Unknown, score: 0.28
Igsf21Unknown, score: 0.13
Vps13dUnknown, score: 0.14
NppaUnknown, score: 0.07
Fbxo44Unknown, score: 0.11
Tmem201Unknown, score: -0.01
Phf13Unknown, score: 0.12
B930041F14RikUnknown, score: 0.19
Klhl17Unknown, score: 0.15
9330182L06RikUnknown, score: 0.05
Nupl2Unknown, score: 0.11
Gbx1Unknown, score: 0.14
Agbl5Unknown, score: 0.53
Zfyve28Unknown, score: 0.44
Cytl1Unknown, score: 0.29
AF366264Unknown, score: 0.07
Cc2d2aUnknown, score: 0.2
Tapt1Unknown, score: 0.09
Sel1l3Unknown, score: 0.55
Slc10a4Unknown, score: 0.1
PpatUnknown, score: 0.06
Polr2bUnknown, score: 0.29
Uba6Unknown, score: 0.13
Ythdc1Unknown, score: 0.33
Grsf1Unknown, score: 0.16
Cox18Unknown, score: 0.51
Parm1Unknown, score: 0.22
Sdad1Unknown, score: 0.21
Cnot6lUnknown, score: 0.03
Fras1Unknown, score: 0.13
Paqr3Unknown, score: 0.07
Tmem150cUnknown, score: 0.26
Plac8Unknown, score: 0.5
Agpat9Unknown, score: 0.3
Arhgap24Unknown, score: 0.19
Lrrc8dUnknown, score: 0.32
A830010M20RikUnknown, score: 0.04
ChfrUnknown, score: 0.14
Tmem119Unknown, score: 0.05
Alkbh2Unknown, score: 0.03
Myo1hUnknown, score: 0.05
Gcn1l1Unknown, score: 0.06
Vsig10Unknown, score: 0.13
Trafd1Unknown, score: 0.22
Fam109aUnknown, score: 0.18
B3gnt4Unknown, score: 0.27
Rimbp2Unknown, score: 0.02
BC037034Unknown, score: 0.51
Micall2Unknown, score: 0.16
E130309D02RikUnknown, score: 0.01
DaglbUnknown, score: 0.04
Aimp2Unknown, score: 0.31
Lmtk2Unknown, score: 0.25
Pdap1Unknown, score: 0.24
Bud31Unknown, score: 0.02
Katnal1Unknown, score: 0.14
Uspl1Unknown, score: 0.23
Gimap6Unknown, score: 0.16
Fam221aUnknown, score: 0.27
Jazf1Unknown, score: 0.19
Fkbp14Unknown, score: 0.11
Vopp1Unknown, score: 0.39
Ccser1Unknown, score: 0.14
Thnsl2Unknown, score: 0.03
Tmem150aUnknown, score: 0.14
Mat2aUnknown, score: 0.07
Eva1aUnknown, score: 0.36
Mob1aUnknown, score: 0.31
Cyp26b1Unknown, score: 0.31
C87436Unknown, score: 0.31
HmcesUnknown, score: 0.14
Hdac11Unknown, score: 0.73
Fgd5Unknown, score: 0.05
Tmf1Unknown, score: 0.17
Frmd4bUnknown, score: 0.26
Gxylt2Unknown, score: 0.65
Zfp637Unknown, score: 0.39
A2mUnknown, score: 0.12
C1rlUnknown, score: 0.26
BC035044Unknown, score: 0.13
Clec2eUnknown, score: 0.52
Clec12aUnknown, score: 0.22
DeraUnknown, score: 0.15
Pyroxd1Unknown, score: 0.13
Klhl42Unknown, score: 0.34
Caprin2Unknown, score: 0.31
Ccdc136Unknown, score: 0.14
Zfp212Unknown, score: 0.3
Leng8Unknown, score: 0.4
Suv420h2Unknown, score: 0.07
Zfp954Unknown, score: 0.12
Zfp418Unknown, score: 0.1
Zfp772Unknown, score: 0.04
Zscan18Unknown, score: 0.12
Zbtb45Unknown, score: 0.14
Hnrnpul1Unknown, score: 0.04
Zfp940Unknown, score: 0.34
Zfp382Unknown, score: 0.03
Kctd15Unknown, score: 0.26
Dpy19l3Unknown, score: 0.35
233147Unknown, score: 0.34
AI987944Unknown, score: 0.1
Ctu1Unknown, score: 0.22
Mrgprb1Unknown, score: 0.47
Ano5Unknown, score: 0.39
Luzp2Unknown, score: 0.29
SiglechUnknown, score: 0.14
SynmUnknown, score: 0.02
Vps33bUnknown, score: 0.32
Tmc3Unknown, score: 0.41
PicalmUnknown, score: 0.17
Kctd14Unknown, score: 0.65
2210018M11RikUnknown, score: 0.43
P2ry6Unknown, score: 0.33
Pgap2Unknown, score: 0.25
Tmem41bUnknown, score: 0.27
Galnt18Unknown, score: 0.2
Spon1Unknown, score: 0.16
Xylt1Unknown, score: 0.06
Thumpd1Unknown, score: 0.31
Dcun1d3Unknown, score: 0.13
Tmem159Unknown, score: 0.02
Abca16Unknown, score: 0.18
BC030336Unknown, score: 0.03
Cog7Unknown, score: 0.08
Palb2Unknown, score: 0.09
Tnrc6aUnknown, score: 0.22
Atxn2lUnknown, score: 0.09
Ino80eUnknown, score: 0.24
Sez6l2Unknown, score: 0.04
Asphd1Unknown, score: 0.07
Zfp764Unknown, score: 0.11
Gm166Unknown, score: 0.13
BC017158Unknown, score: 0.34
Gpr26Unknown, score: 0.1
Tpcn2Unknown, score: 0.54
Zfp958Unknown, score: 0.48
Arglu1Unknown, score: 0.11
Pcid2Unknown, score: 0.34
Adprhl1Unknown, score: 0.12
Tmco3Unknown, score: 0.34
Erich1Unknown, score: 0.09
Arhgef10Unknown, score: 0
Tti2Unknown, score: 0.21
Mboat4Unknown, score: 0.28
234159Unknown, score: 0.21
Fgl1Unknown, score: 0.18
HeltUnknown, score: 0.3
Neil3Unknown, score: 0.56
Gpm6aUnknown, score: 0.13
Cbr4Unknown, score: 0.26
234311Unknown, score: 0.16
Psd3Unknown, score: 0.21
Csgalnact1Unknown, score: 0.18
Zfp930Unknown, score: 0.05
Klhl26Unknown, score: 0
Ccdc124Unknown, score: 0.2
Ushbp1Unknown, score: 0.01
Ankle1Unknown, score: 0.04
Nxnl1Unknown, score: 0.02
Colgalt1Unknown, score: 0.4
Zfp961Unknown, score: 0.15
Cib3Unknown, score: 0.4
Tmem184cUnknown, score: 0.06
Inpp4bUnknown, score: 0.2
Heatr3Unknown, score: 0.11
Cpne2Unknown, score: 0.05
Slc38a7Unknown, score: 0.11
Dync1li2Unknown, score: 0.7
Nae1Unknown, score: 0.44
Txnl4bUnknown, score: 0.33
TatUnknown, score: 0.15
AtminUnknown, score: 0.2
MthfsdUnknown, score: 0.38
Klhdc4Unknown, score: 0.01
Piezo1Unknown, score: 0.12
Nup133Unknown, score: 0.34
BC021891Unknown, score: 0.03
9230110C19RikUnknown, score: 0.73
Cep126Unknown, score: 0.18
Mbd3l2Unknown, score: 0.45
PpanUnknown, score: 0
Kank2Unknown, score: 0.08
Lppr2Unknown, score: 0.29
Zfp599Unknown, score: 0.31
Zfp810Unknown, score: 0.31
Igsf9bUnknown, score: 0.31
NtmUnknown, score: 0.14
Adamts15Unknown, score: 0.11
Tmem45bUnknown, score: 0.14
Fez1Unknown, score: 0.31
Sc5dUnknown, score: 0.58
Tmem136Unknown, score: 0.08
C1qtnf5Unknown, score: 0.19
Zbtb16Unknown, score: 0.02
Ttc12Unknown, score: 0.25
GldnUnknown, score: 0.25
Dmxl2Unknown, score: 0.31
Lingo1Unknown, score: 0.05
Snx33Unknown, score: 0.05
Coro2bUnknown, score: 0.18
Herc1Unknown, score: 0.1
Usp3Unknown, score: 0.36
Gtf2a2Unknown, score: 0.02
Fam63bUnknown, score: 0.68
Zfp280dUnknown, score: 0.12
Fam214aUnknown, score: 0.16
Slc17a5Unknown, score: 0.34
Cd109Unknown, score: 0.16
Plscr4Unknown, score: 0.15
Pxylp1Unknown, score: 0.19
Ppp2r3aUnknown, score: 0.19
Topbp1Unknown, score: 0.1
Dnajc13Unknown, score: 0.4
Dusp7Unknown, score: 0.27
Parp3Unknown, score: 0.02
ScapUnknown, score: 0.3
Als2clUnknown, score: 0.28
Rtp3Unknown, score: 0.05
Dync1li1Unknown, score: 0.58
Acaa1bUnknown, score: 0.3
Zfp445Unknown, score: 0.36
Zfp65Unknown, score: 0.37
DHRSXUnknown, score: 0.26
Zfp709Unknown, score: 0.11
Pyhin1Unknown, score: 0.28
5730507C01RikUnknown, score: 0.13
PhgdhUnknown, score: 0.1
Spry3Unknown, score: -0.01
Sytl5Unknown, score: 0.14
Usp11Unknown, score: 0.02
Gpr119Unknown, score: 0.17
Ddx26bUnknown, score: 0.22
Slc9a6Unknown, score: 0.17
BC023829Unknown, score: 0
Pdk3Unknown, score: 0.2
Klhl15Unknown, score: 0.6
Stard8Unknown, score: 0.27
Ercc6lUnknown, score: 0.73
Klhl4Unknown, score: 0.16
Nox1Unknown, score: 0.3
Tceal1Unknown, score: 0.37
Rbm41Unknown, score: 0.19
Adgrg2Unknown, score: 0.52
FancbUnknown, score: 0.12
Gemin8Unknown, score: 0.56
Ofd1Unknown, score: 0.17
Zc3h12dUnknown, score: 0.05
Il22ra2Unknown, score: 0.25
Aldh8a1Unknown, score: 0.12
Lingo3Unknown, score: 0.16
Ric8bUnknown, score: 0.19
Gas2l3Unknown, score: 0.23
Cdk17Unknown, score: 0.26
Ccdc38Unknown, score: 0.21
Osbpl8Unknown, score: 0.16
MyrflUnknown, score: 0.02
Gpr75Unknown, score: 0.75
Btnl9Unknown, score: 0.24
Zfp454Unknown, score: 0.04
SowahaUnknown, score: 0.32
PfasUnknown, score: 0.07
Slc13a5Unknown, score: 0.14
Rtn4rl1Unknown, score: 0.14
Tusc5Unknown, score: 0.67
Sarm1Unknown, score: 0.04
PhbUnknown, score: 0.17
Slfn9Unknown, score: 0.05
Usp32Unknown, score: 0.14
HexdcUnknown, score: 0.23
Fn3krpUnknown, score: 0.15
BC068281Unknown, score: 0.32
Cog5Unknown, score: 0.13
Dock4Unknown, score: 0.04
Akap6Unknown, score: 0.67
Gpr135Unknown, score: 0.11
Tmem30bUnknown, score: 0.39
Rps6kl1Unknown, score: 0.35
Zdhhc22Unknown, score: 0.11
MtrUnknown, score: 0.43
Mylk4Unknown, score: 0.29
Zfp367Unknown, score: 0.26
Zfp458Unknown, score: 0.52
Zfp874aUnknown, score: 0.17
Zfp58Unknown, score: 0.35
Zfp72Unknown, score: 0.01
Pde4dUnknown, score: 0.2
GaptUnknown, score: 0.18
Cdc20bUnknown, score: 0.21
Erc2Unknown, score: 0.38
Arhgap22Unknown, score: 0.18
Tlr11Unknown, score: 0.14
Cdh24Unknown, score: 0.1
Il17dUnknown, score: 0.11
Setdb2Unknown, score: 0.08
Pnma2Unknown, score: 0.14
Enox1Unknown, score: 0.4
Slitrk6Unknown, score: 0.12
Plcxd3Unknown, score: 0.17
Adamts12Unknown, score: 0.01
Rspo2Unknown, score: 0.26
Colec10Unknown, score: 0.12
Zfp647Unknown, score: 0.06
Apol8Unknown, score: 0.06
Cacna1iUnknown, score: 0
Ttc38Unknown, score: 0.09
Slc2a13Unknown, score: 0.25
Ccdc184Unknown, score: 0.12
Zfp641Unknown, score: 0.19
Mkl2Unknown, score: 0.09
Rimbp3Unknown, score: 0.4
Klhl6Unknown, score: -0.01
OstnUnknown, score: 0.26
PigzUnknown, score: 0.16
LmlnUnknown, score: 0.17
Gpr156Unknown, score: 0.17
Cd200r4Unknown, score: 0.29
LnpepUnknown, score: 0.51
Zfp760Unknown, score: 0.56
Zfp811Unknown, score: 0.04
Zfp952Unknown, score: 0.07
Zfp563Unknown, score: 0.19
H2-M5Unknown, score: 0.37
Fsd1Unknown, score: 0.19
Lrrc30Unknown, score: 0.2
9430020K01RikUnknown, score: 0.09
Gpr151Unknown, score: 0.05
Ythdc2Unknown, score: 0.43
Ccdc112Unknown, score: 0.17
Fem1cUnknown, score: 0.29
Dmxl1Unknown, score: 0.07
Adamts19Unknown, score: 0.21
Gm4951Unknown, score: 0.19
Slc6a7Unknown, score: 0.04
Pcyox1lUnknown, score: 0.12
Malt1Unknown, score: 0.33
Kcng2Unknown, score: 0.22
Kcnv2Unknown, score: 0.04
9930021J03RikUnknown, score: 0.06
Ranbp6Unknown, score: 0.16
Slc16a12Unknown, score: 0.42
Kif20bUnknown, score: 0.01
Slc35g1Unknown, score: -0.01
St18Unknown, score: 0.17
Mcmdc2Unknown, score: -0.01
Sulf1Unknown, score: 0.03
Plekha6Unknown, score: 0.07
Lax1Unknown, score: 0.05
Klhl12Unknown, score: 0.29
Brinp2Unknown, score: 0.02
Tnfsf18Unknown, score: 0.27
Gpr161Unknown, score: 0.22
Vsig8Unknown, score: 0.07
Pgap1Unknown, score: 0.23
CatipUnknown, score: 0.21
Ccdc108Unknown, score: 0.06
Asic4Unknown, score: 0.18
Fam124bUnknown, score: 0.03
Gpr158Unknown, score: 0
Fam78aUnknown, score: 0.37
Ralgps1Unknown, score: 0.15
Zbtb34Unknown, score: 0.26
Crb2Unknown, score: 0.21
Olfml2aUnknown, score: 0.1
Galnt5Unknown, score: 0.23
Xirp2Unknown, score: 0.02
Rbm45Unknown, score: 0.48
Zfp385bUnknown, score: 0.2
Ypel4Unknown, score: 0.38
Lrrc55Unknown, score: 0.09
Harbi1Unknown, score: 0.11
Tspan18Unknown, score: 0.14
Lrrc4cUnknown, score: 0.38
Ldlrad3Unknown, score: 0.35
Slc5a12Unknown, score: 0.4
Exd1Unknown, score: 0.17
Wdr76Unknown, score: 0.01
Lzts3Unknown, score: 0.29
Pak7Unknown, score: 0.15
Dzank1Unknown, score: 0
SnphUnknown, score: 0.06
Phc3Unknown, score: 0.27
Slc7a14Unknown, score: 0.17
Ccdc144bUnknown, score: 0.1
D3Ertd254eUnknown, score: 0.26
Bbs12Unknown, score: 0.35
Pabpc4lUnknown, score: 0.05
Frem2Unknown, score: 0.22
Igsf10Unknown, score: 0.03
Ppm1lUnknown, score: 0.05
Rxfp4Unknown, score: 0.62
Zfp697Unknown, score: 0.17
Slc22a15Unknown, score: 0.13
Pde5aUnknown, score: 0.17
Bank1Unknown, score: 0.12
Wdr63Unknown, score: 0.2
Slc44a5Unknown, score: 0.1
Gdf6Unknown, score: 0.16
Atp6v0d2Unknown, score: 0.39
Rgp1Unknown, score: 0.51
Tmem8bUnknown, score: 0.02
Grin3aUnknown, score: 0
Tmem245Unknown, score: 0.26
D630039A03RikUnknown, score: 0.14
RasefUnknown, score: 0.03
Frmd3Unknown, score: 0.19
Kank4Unknown, score: 0.54
Atg4cUnknown, score: 0.1
Raver2Unknown, score: 0.25
Wdr78Unknown, score: 0.26
Slc35d1Unknown, score: 0.04
1700024P16RikUnknown, score: 0.03
Rims3Unknown, score: 0
Dlgap3Unknown, score: 0.1
Azin2Unknown, score: 0.04
Ifnlr1Unknown, score: 0.04
E2f2Unknown, score: 0.45
Pramef8Unknown, score: 0.37
Lrrd1Unknown, score: 0.23
Gnat3Unknown, score: 0.14
Rsbn1lUnknown, score: 0.22
NapepldUnknown, score: 0
Cct8l1Unknown, score: 0.15
GaremlUnknown, score: 0.35
CpzUnknown, score: 0.06
Kctd8Unknown, score: 0.37
TecrlUnknown, score: 0.3
2900026A02RikUnknown, score: 0.03
Hcar1Unknown, score: 0.02
Nyap1Unknown, score: 0.36
243302Unknown, score: 0.11
Slc29a4Unknown, score: 0.41
Zfp775Unknown, score: 0.62
H1fxUnknown, score: 0.29
Uroc1Unknown, score: 0.18
Ccdc37Unknown, score: 0.22
Grip2Unknown, score: 0.09
Prickle2Unknown, score: 0.12
Iqsec3Unknown, score: 0.24
Klre1Unknown, score: 0.19
Styk1Unknown, score: 0.49
2010107G12RikUnknown, score: 0.11
Slc13a4Unknown, score: 0
Parp12Unknown, score: 0.05
Ppp6r1Unknown, score: 0.32
Zfp14Unknown, score: 0.02
Rgs9bpUnknown, score: 0.15
SiglecgUnknown, score: -0.01
RgmaUnknown, score: 0.2
Fsd2Unknown, score: 0.48
Trim30bUnknown, score: 0.03
Olfml1Unknown, score: 0.58
Dlgap2Unknown, score: 0.42
Mcph1Unknown, score: 0.14
Kat6aUnknown, score: 0.22
Erlin2Unknown, score: 0.24
D8Ertd82eUnknown, score: -0.01
Lonrf1Unknown, score: 0.13
Wdr17Unknown, score: 0.15
Elmod2Unknown, score: 0.05
Rpgrip1lUnknown, score: 0.24
Pskh1Unknown, score: 0.02
SprtnUnknown, score: 0.08
Disc1Unknown, score: -0.01
Sipa1l2Unknown, score: 0.87
Cntn5Unknown, score: 0
Kdm4dUnknown, score: 0.03
Zfp317Unknown, score: 0.18
Olfm2Unknown, score: 0.25
AW551984Unknown, score: -0.01
Nxpe4Unknown, score: 0.28
Ankk1Unknown, score: 0.29
LaynUnknown, score: 0
Zc3h12cUnknown, score: 0.25
NpatUnknown, score: 0.01
AI118078Unknown, score: 0.37
ScaperUnknown, score: 0.11
C2cd4aUnknown, score: 0.07
Klhl31Unknown, score: 0.06
Mrap2Unknown, score: 0.2
Snx14Unknown, score: 0.14
Slc35g2Unknown, score: 0.35
Col6a6Unknown, score: 0.36
MyripUnknown, score: 0.17
Fam198aUnknown, score: 0.36
RetnlgUnknown, score: 0.58
Zfp300Unknown, score: 0.37
Slitrk4Unknown, score: 0.08
Slitrk2Unknown, score: -0.01
Pdzd4Unknown, score: 0.43
Dkc1Unknown, score: 0.39
Zc4h2Unknown, score: 0.17
Eda2rUnknown, score: 0.12
Awat2Unknown, score: 0.05
Gm614Unknown, score: 0.33
C77370Unknown, score: 0.47
Pcdh11xUnknown, score: 0.01
Zfp711Unknown, score: 0.09
HdxUnknown, score: 0.37
Kir3dl1Unknown, score: 0.08
Fam199xUnknown, score: 0.4
Mum1l1Unknown, score: 0.11
Tbc1d8bUnknown, score: 0.47
Iqsec2Unknown, score: 0.29
Klhl34Unknown, score: 0.14
TceancUnknown, score: 0.24
Trappc1Unknown, score: 0.18
Spag4Unknown, score: 0.21
Ift52Unknown, score: 0.34
Ankrd27Unknown, score: 0.46
Myo1gUnknown, score: 0.24
OtoaUnknown, score: 0.19
Vwa1Unknown, score: 0.25
BivmUnknown, score: 0.08
Fcgr4Unknown, score: 0.42
CsadUnknown, score: 0.13
Klhl8Unknown, score: 0.16
Lgi2Unknown, score: 0.37
Neto1Unknown, score: 0.31
Slc25a28Unknown, score: 0.08
Defb19Unknown, score: 0.03
Cd300lfUnknown, score: 0.03
Il27Unknown, score: 0.21
Atpaf2Unknown, score: 0.12
Obox6Unknown, score: 0.21
ToxUnknown, score: 0
Usp7Unknown, score: 0.29
MiosUnknown, score: 0.22
Gin1Unknown, score: 0.48
Cables2Unknown, score: 0.04
Ropn1lUnknown, score: 0.16
Tpcn1Unknown, score: 0.15
Olfr544Unknown, score: 0
Olfr212Unknown, score: 0.1
Olfr1034Unknown, score: 0.13
Olfr877Unknown, score: -0.01
Olfr1392Unknown, score: 0.18
Olfr873Unknown, score: 0.11
Olfr1033Unknown, score: 0.09
Olfr39Unknown, score: 0.32
Olfr457Unknown, score: 0.05
Olfr691Unknown, score: 0.36
Olfr520Unknown, score: 0.35
Olfr558Unknown, score: 0.41
Olfr613Unknown, score: 0.2
Olfr550Unknown, score: 0.25
Klk8Unknown, score: 0.13
Tubgcp3Unknown, score: 0.04
Ehd2Unknown, score: 0.03
FevUnknown, score: 0.02
Cadm4Unknown, score: 0.05
Nphp4Unknown, score: 0.11
Nav3Unknown, score: 0.36
Cdc42ep3Unknown, score: 0.34
Plekhg3Unknown, score: 0.32
Spata2Unknown, score: 0.16
Ttc26Unknown, score: 0.59
Acsf2Unknown, score: -0.01
Ly6g5bUnknown, score: 0.17
AcmsdUnknown, score: 0.12
Snx17Unknown, score: 0.07
Rps15aUnknown, score: 0.33
ShprhUnknown, score: 0.16
Zbtb24Unknown, score: 0.12
Kcnc2Unknown, score: 0.4
Fam19a2Unknown, score: 0.35
PpiaUnknown, score: 0.4
Ankrd13bUnknown, score: 0.06
Phf12Unknown, score: 0.02
Rab11fip4Unknown, score: 0.03
Eme1Unknown, score: 0.06
Zfp652Unknown, score: 0.15
Rapgefl1Unknown, score: 0.17
Lsm12Unknown, score: 0.18
Gm1564Unknown, score: 0.3
Sntg2Unknown, score: 0.34
Zbtb1Unknown, score: 0.28
GphnUnknown, score: 0.27
Tmem229bUnknown, score: 0.03
Sptlc1Unknown, score: 0.33
Zfp759Unknown, score: 0.18
Ccnb1Unknown, score: 0.34
Tox4Unknown, score: 0.41
Wdfy2Unknown, score: 0.27
GuloUnknown, score: 0.19
9930012K11RikUnknown, score: 0.13
EgflamUnknown, score: 0.07
Adck5Unknown, score: 0.29
Nlrc3Unknown, score: 0.23
Rbfox1Unknown, score: 0.13
Xxylt1Unknown, score: 0.18
Stfa2l1Unknown, score: 0.11
Robo2Unknown, score: 0.42
Capn11Unknown, score: 0.11
Arhgap28Unknown, score: 0
Ltbp1Unknown, score: 0.15
StrnUnknown, score: 0.28
Sh3rf2Unknown, score: 0.26
Stk32aUnknown, score: 0.49
DaglaUnknown, score: 0.21
Cpsf7Unknown, score: 0.48
Colgalt2Unknown, score: 0.25
Inpp4aUnknown, score: 0.13
Mgat4aUnknown, score: 0.31
PaskUnknown, score: -0.01
Gtf3c4Unknown, score: 0.08
SetxUnknown, score: 0.1
Muc15Unknown, score: 0.25
Ccdc32Unknown, score: 0.41
Ell3Unknown, score: 0.22
Slc4a11Unknown, score: 0.05
AhcyUnknown, score: 0.07
3110057O12RikUnknown, score: 0.21
Plch1Unknown, score: 0.1
Wdr3Unknown, score: 0.04
Lrig2Unknown, score: 0.12
Fbxo10Unknown, score: 0.26
Tex10Unknown, score: 0.26
Plekhg5Unknown, score: 0.05
Plch2Unknown, score: 0.32
Ppp2r2cUnknown, score: 0.16
Gm15800Unknown, score: 0.54
269701Unknown, score: 0.23
Mphosph9Unknown, score: 0.06
Lhfpl4Unknown, score: 0.04
Zfp384Unknown, score: 0.26
Pon3Unknown, score: 0.69
Vmn2r57Unknown, score: 0.25
Gdpgp1Unknown, score: 0.08
Orai3Unknown, score: 0.13
Lpcat2Unknown, score: 0.13
Ogfod1Unknown, score: 0.25
Mon1bUnknown, score: 0.1
Rpl13Unknown, score: 0.36
Irf2bp2Unknown, score: 0.36
Maml2Unknown, score: 0.19
Amica1Unknown, score: 0.11
Rab39Unknown, score: 0
Elmod1Unknown, score: 0.35
Myo9aUnknown, score: -0.01
Mbtps2Unknown, score: 0.34
Tmem132eUnknown, score: 0.24
Klhdc1Unknown, score: 0.43
Ankdd1bUnknown, score: 0.1
5031414D18RikUnknown, score: 0.07
Phf21bUnknown, score: 0.13
Zbtb11Unknown, score: 0.06
Ip6k3Unknown, score: 0.2
Rab5aUnknown, score: 0.23
Vps13aUnknown, score: 0.08
Adcy10Unknown, score: 0.5
Cdk15Unknown, score: 0.05
Tmem169Unknown, score: 0.34
C2cd4dUnknown, score: 0.01
TbckUnknown, score: 0.13
Tstd2Unknown, score: 0.42
SpibUnknown, score: 0.06
Tarsl2Unknown, score: 0
B3gnt6Unknown, score: 0.1
Tango6Unknown, score: 0.24
TbcelUnknown, score: 0.07
PigsUnknown, score: 0.1
Gemin4Unknown, score: 0.2
Ccdc42Unknown, score: 0.02
Marveld1Unknown, score: 0.09
NynrinUnknown, score: 0.02
Trpa1Unknown, score: 0.08
Klhl23Unknown, score: 0.15
Gm694Unknown, score: 0.36
Cyp4a12aUnknown, score: 0.2
C2cd3Unknown, score: 0.06
Exoc3lUnknown, score: 0.19
Vsig4Unknown, score: -0.01
Spaca5Unknown, score: 0.06
Spin2cUnknown, score: 0.18
Tmtc2Unknown, score: 0.03
Lrrc10bUnknown, score: 0.42
Adamts13Unknown, score: 0.1
Tlr13Unknown, score: 0.32
Rhbdd3Unknown, score: 0.04
RilpUnknown, score: 0.52
Lix1lUnknown, score: 0.07
Emilin3Unknown, score: 0.11
B3gat2Unknown, score: 0.3
AfmUnknown, score: 0.32
Adam1bUnknown, score: 0.12
Kif19aUnknown, score: 0.15
Klk14Unknown, score: 0.08
Sec22aUnknown, score: 0.1
Slc24a5Unknown, score: 0.2
Gimap5Unknown, score: 0.56
Gimap9Unknown, score: 0.34
Hist1h3cUnknown, score: 0.34
Hist1h3dUnknown, score: 0.34
Hist1h3bUnknown, score: 0.34
Hist1h3eUnknown, score: 0.07
Hist1h3hUnknown, score: 0.07
Hist1h3iUnknown, score: 0.34
Hist1h4nUnknown, score: 0.55
Hist1h2bbUnknown, score: 0.01
Hist1h2bfUnknown, score: 0.01
Hist1h2bnUnknown, score: 0.02
Hist2h2bbUnknown, score: 0.5
Gpr4Unknown, score: 0.06
SctrUnknown, score: 0.68
Fchsd1Unknown, score: 0.36
A130010J15RikUnknown, score: 0.01
A230046K03RikUnknown, score: 0.17
Sf3b2Unknown, score: 0.37
Ubald2Unknown, score: 0.34
Dpep2Unknown, score: 0.28
Fndc3aUnknown, score: 0.02
Pld5Unknown, score: 0.07
Zfp672Unknown, score: 0.12
6030419C18RikUnknown, score: 0.36
Itga11Unknown, score: 0.03
A430078G23RikUnknown, score: 0.06
Zfp750Unknown, score: 0.22
Zfp182Unknown, score: 0.24
B230216G23RikUnknown, score: 0.16
Idi1Unknown, score: 0.04
Syne2Unknown, score: 0.13
Xkr5Unknown, score: 0.12
6430573F11RikUnknown, score: 0.3
Hif1anUnknown, score: -0.01
Zfp653Unknown, score: 0.08
SybuUnknown, score: 0.21
Dcp1bUnknown, score: 0.18
GalmUnknown, score: 0.15
Efcab5Unknown, score: 0.06
Fsd1lUnknown, score: 0.08
Nt5dc1Unknown, score: 0.19
Rab9bUnknown, score: 0.15
Usp37Unknown, score: 0.27
Podxl2Unknown, score: 0.1
Eml5Unknown, score: 0.16
Ablim3Unknown, score: 0.02
Simc1Unknown, score: 0.05
Zfyve27Unknown, score: 0.09
SmoUnknown, score: 0.1
Rfx7Unknown, score: 0.19
C130050O18RikUnknown, score: 0.23
9630033F20RikUnknown, score: 0.2
Bbs9Unknown, score: 0.17
Tmcc3Unknown, score: 0.2
Dock6Unknown, score: 0.32
DselUnknown, score: 0.14
Ism1Unknown, score: 0.13
Apba1Unknown, score: 0.12
Tns3Unknown, score: 0.14
A530016L24RikUnknown, score: -0.01
Ttll1Unknown, score: 0.1
Cc2d1bUnknown, score: 0.17
319974Unknown, score: 0.04
Kif6Unknown, score: 0.55
Casc4Unknown, score: 0.07
Sidt1Unknown, score: 0.14
Uggt1Unknown, score: 0.08
Ccdc79Unknown, score: 0.38
Nceh1Unknown, score: 0.05
Zbtb39Unknown, score: 0.05
Ano4Unknown, score: 0.12
ReltUnknown, score: 0.12
Slc38a11Unknown, score: 0.25
Prr18Unknown, score: 0.03
Fndc9Unknown, score: 0.12
Fbxl13Unknown, score: 0.01
Adrbk2Unknown, score: 0.14
Fam179aUnknown, score: 0.61
Cep95Unknown, score: 0.12
Fndc7Unknown, score: 0.19
Msrb3Unknown, score: 0.02
Lrrc58Unknown, score: 0.39
Hook3Unknown, score: 0.37
Senp5Unknown, score: -0.01
Maats1Unknown, score: 0.03
Ccdc171Unknown, score: 0.69
Ccdc66Unknown, score: 0.2
Ttll5Unknown, score: 0.12
March3Unknown, score: 0
Fubp3Unknown, score: 0.3
Rasgef1bUnknown, score: 0.28
Glt28d2Unknown, score: 0.03
Tmem251Unknown, score: 0.15
Bcorl1Unknown, score: 0.25
CenptUnknown, score: 0.03
Cadps2Unknown, score: 0.28
Trank1Unknown, score: 0.16
RinlUnknown, score: 0.39
Alg6Unknown, score: 0.23
P4ha3Unknown, score: 0.22
Ppm1eUnknown, score: 0.28
Rasal3Unknown, score: 0.31
Heatr5aUnknown, score: 0.33
A830018L16RikUnknown, score: 0.1
Ipcef1Unknown, score: 0.44
Tmem215Unknown, score: 0.37
Lmod3Unknown, score: 0.08
Tmem104Unknown, score: 0.11
Ubn2Unknown, score: 0.07
Tcp11l1Unknown, score: 0.27
Fam169aUnknown, score: 0.57
Sycp2Unknown, score: 0.35
Dennd5bUnknown, score: 0.02
Islr2Unknown, score: 0.07
Snrnp200Unknown, score: 0.21
Zbtb26Unknown, score: 0.04
OcrlUnknown, score: 0.33
D5Ertd579eUnknown, score: 0.1
Zfp629Unknown, score: 0.56
DctdUnknown, score: 0.17
Ccdc158Unknown, score: 0.03
Soga1Unknown, score: 0.15
Tmem117Unknown, score: 0.26
Pptc7Unknown, score: 0.16
Slc26a9Unknown, score: 0.17
Fastkd1Unknown, score: 0.04
A330050F15RikUnknown, score: 0.09
Vstm4Unknown, score: 0.1
Tmem154Unknown, score: 0.01
Pkn1Unknown, score: 0
Dcaf5Unknown, score: 0.16
Ankrd16Unknown, score: -0.01
Atad2bUnknown, score: 0.19
C530008M17RikUnknown, score: 0.53
Amigo3Unknown, score: 0.2
Cdh10Unknown, score: 0.09
Mical2Unknown, score: 0.32
Itgb8Unknown, score: 0.14
Wscd2Unknown, score: 0.18
Mtap7d3Unknown, score: 0.12
Ccbe1Unknown, score: 0.12
Tnpo3Unknown, score: 0.27
Atp11cUnknown, score: 0.22
PisdUnknown, score: -0.01
Lrrn4Unknown, score: 0.24
Enpp6Unknown, score: 0.26
Arl4cUnknown, score: 0.42
Xpnpep3Unknown, score: 0.12
VprbpUnknown, score: 0.04
Serac1Unknown, score: 0.05
Gpr183Unknown, score: 0.15
Cdv3Unknown, score: 0.1
Hist1h4bUnknown, score: 0.3
Dna2Unknown, score: 0.05
Tmem26Unknown, score: 0.14
Ubtd2Unknown, score: 0.2
PiglUnknown, score: 0.04
Pitpnm3Unknown, score: 0.16
Xaf1Unknown, score: 0
Slfn5Unknown, score: 0.15
Fads6Unknown, score: 0.17
Dtd2Unknown, score: 0.31
Fam179bUnknown, score: 0.57
Gfod1Unknown, score: 0.08
Zfp459Unknown, score: 0.02
Rft1Unknown, score: 0.03
Sh2d4bUnknown, score: 0.33
Npm2Unknown, score: 0.01
Vwa5b2Unknown, score: 0.17
MccUnknown, score: 0.35
Zfp532Unknown, score: 0.18
Zfp516Unknown, score: 0.01
Atg2aUnknown, score: 0.03
Pkd2l1Unknown, score: 0.33
Scd4Unknown, score: 0.26
Hecw2Unknown, score: 0.24
Ankrd44Unknown, score: 0.14
Unc80Unknown, score: 0.1
Ppp1r12bUnknown, score: 0.2
Lgr6Unknown, score: 0.32
Ptrh1Unknown, score: 0.29
AccsUnknown, score: 0.27
1810024B03RikUnknown, score: 0.14
Nol4lUnknown, score: 0.4
Med12lUnknown, score: 0.21
E130311K13RikUnknown, score: 0.04
Dennd2cUnknown, score: 0.28
Fam19a3Unknown, score: 0.15
Aknad1Unknown, score: 0.28
Tmem67Unknown, score: -0.01
Fam166bUnknown, score: 0.2
Dennd4cUnknown, score: 0.12
Foxo6Unknown, score: 0.19
Catsper4Unknown, score: 0.23
Fhad1Unknown, score: 0.21
Slc5a6Unknown, score: 0.65
Shisa3Unknown, score: 0.02
Cxcl3Unknown, score: 0.12
Kctd10Unknown, score: 0.27
Taok3Unknown, score: 0.41
Sdk1Unknown, score: 0.15
D630045J12RikUnknown, score: 0.11
Zfp786Unknown, score: 0.19
Fam188bUnknown, score: 0.23
Dnah6Unknown, score: 0.32
Gcfc2Unknown, score: 0.27
B4galnt3Unknown, score: 0.26
Gm766Unknown, score: 0.08
Far2Unknown, score: 0.1
Tmem150bUnknown, score: 0.32
Fan1Unknown, score: 0.13
Prss53Unknown, score: 0.06
Btbd16Unknown, score: 0.13
Htra4Unknown, score: 0.16
Slc7a6Unknown, score: 0.32
Trim67Unknown, score: 0.02
Piwil4Unknown, score: 0.56
OpcmlUnknown, score: 0.17
Hcn4Unknown, score: 0.09
GmppbUnknown, score: -0.01
Tgm4Unknown, score: 0.01
331374Unknown, score: 0.42
Thoc2Unknown, score: 0.02
XkrxUnknown, score: 0.41
Mapk15Unknown, score: 0.16
Card9Unknown, score: 0.08
KcpUnknown, score: 0.23
Cox6b2Unknown, score: 0
Cngb1Unknown, score: 0.52
Ppp1r13lUnknown, score: 0.06
N4bp2Unknown, score: 0.4
Cd59bUnknown, score: 0.48
Ttc16Unknown, score: 0.3
Acad12Unknown, score: 0.17
Akap17bUnknown, score: 0.06
Zfp780bUnknown, score: 0.16
Supv3l1Unknown, score: 0.09
UstUnknown, score: 0.18
Tmem241Unknown, score: 0.06
Slc41a2Unknown, score: 0.25
338366Unknown, score: 0.06
Fam109bUnknown, score: 0.15
Tmem220Unknown, score: 0.3
EndovUnknown, score: 0
Map3k9Unknown, score: 0.66
Scgb1c1Unknown, score: 0.22
Morc3Unknown, score: 0.18
Fa2hUnknown, score: 0
Agap1Unknown, score: 0.62
Egfl7Unknown, score: 0.16
Slc2a12Unknown, score: 0.77
GarsUnknown, score: 0.08
Nr1d2Unknown, score: 0.06
Edc3Unknown, score: 0.42
Zfp931Unknown, score: 0.24
Pcdhac2Unknown, score: 0.09
Mrpl21Unknown, score: 0.24
Ltv1Unknown, score: 0.13
Zfp703Unknown, score: 0.31
Rtl1Unknown, score: 0.19
Gpr141Unknown, score: 0.26
Myo18aUnknown, score: 0.07
Trim46Unknown, score: 0.17
Speer4dUnknown, score: 0.11
Nlrp12Unknown, score: 0.02
Nanos2Unknown, score: 0.39
Pram1Unknown, score: 0.51
Lrrc24Unknown, score: 0.09
Tagap1Unknown, score: 0.17
Lemd3Unknown, score: 0
Lin28bUnknown, score: 0.01
NefhUnknown, score: 0.28
Slc47a2Unknown, score: 0.1
Tmem102Unknown, score: 0.25
Rap1gap2Unknown, score: 0.3
Rph3alUnknown, score: 0.09
Mks1Unknown, score: 0.02
Tssc1Unknown, score: 0.23
Atxn7l1Unknown, score: 0.29
SlirpUnknown, score: 0.33
Serpina11Unknown, score: -0.01
Mrs2Unknown, score: 0.45
Serpinb1cUnknown, score: -0.01
Stmnd1Unknown, score: 0.21
Gm5141Unknown, score: 0.31
Rsl1Unknown, score: 0.44
Lrch1Unknown, score: 0.23
DgkhUnknown, score: 0
Olfm4Unknown, score: 0.02
Alg10bUnknown, score: 0.31
ParlUnknown, score: 0.21
Ccdc58Unknown, score: 0.11
ErmardUnknown, score: 0.25
Zfp948Unknown, score: 0.32
Dnph1Unknown, score: 0.2
Tmem232Unknown, score: 0.48
Rmdn2Unknown, score: 0.04
Arhgef33Unknown, score: 0.08
GaremUnknown, score: 0.32
Prob1Unknown, score: 0.07
Greb1lUnknown, score: 0.63
Naaladl1Unknown, score: 0.22
Fam189a2Unknown, score: 0.15
Cfap58Unknown, score: 0.3
Lipo1Unknown, score: 0.29
Gm973Unknown, score: 0.23
MregUnknown, score: 0.38
Atp2b4Unknown, score: 0.01
Rc3h1Unknown, score: 0.12
BC055324Unknown, score: 0.18
MndaUnknown, score: 0.06
6330403A02RikUnknown, score: 0.17
Iars2Unknown, score: 0.12
Batf3Unknown, score: 0.11
Fam178bUnknown, score: 0.18
Lonrf2Unknown, score: 0.07
Cacfd1Unknown, score: 0.28
Med19Unknown, score: 0.16
Bpifb4Unknown, score: 0.07
2810408M09RikUnknown, score: 0.19
Cdh26Unknown, score: 0
Gpr176Unknown, score: 0.03
Gm14085Unknown, score: 0.11
Trim55Unknown, score: 0.27
S100a7aUnknown, score: 0.17
Dpy19l4Unknown, score: 0.29
Pdp1Unknown, score: 0.3
Mup20Unknown, score: 0.35
Mup21Unknown, score: 0.29
Ube2uUnknown, score: 0.08
Ccdc24Unknown, score: 0.53
Zfp69Unknown, score: 0.13
Tbc1d2Unknown, score: 0.06
Rbm33Unknown, score: 0.1
Adgrf3Unknown, score: 0.15
VgfUnknown, score: 0.39
Wdr95Unknown, score: 0.45
B3glctUnknown, score: 0.11
Drc1Unknown, score: 0.45
Ssbp1Unknown, score: 0.08
Tatdn2Unknown, score: 0.32
Lpar5Unknown, score: 0.18
Cracr2aUnknown, score: 0.56
2700089E24RikUnknown, score: 0.04
Brsk1Unknown, score: 0.02
Ano8Unknown, score: 0.2
Unc13aUnknown, score: 0.07
Zfp882Unknown, score: 0.22
Pabpn1lUnknown, score: 0
Urb2Unknown, score: 0.18
Pdp2Unknown, score: 0.34
AB124611Unknown, score: 0.23
Ccdc84Unknown, score: 0.07
Ccdc33Unknown, score: 0.31
Snx22Unknown, score: 0.32
Cep162Unknown, score: 0.06
Susd5Unknown, score: 0.03
TcaimUnknown, score: 0.31
Fdxacb1Unknown, score: 0.2
Jade3Unknown, score: 0.06
8030474K03RikUnknown, score: 0.34
Tmem29Unknown, score: 0.47
Cdkl5Unknown, score: 0.17
Poc1bUnknown, score: 0.13
Atxn7l3bUnknown, score: 0.06
Best3Unknown, score: 0.05
382522Unknown, score: 0.07
Cd300lhUnknown, score: 0.34
Pfn4Unknown, score: 0.02
Zbtb42Unknown, score: 0.14
Mtx3Unknown, score: 0.31
ColqUnknown, score: 0.3
Zfp488Unknown, score: 0.17
Neil2Unknown, score: 0.21
Rrm2bUnknown, score: 0.07
Tvp23aUnknown, score: 0.32
Ypel5Unknown, score: 0.09
Gpr25Unknown, score: 0.14
Tlr12Unknown, score: 0.34
Fndc5Unknown, score: 0.36
Slc25a34Unknown, score: 0.05
Ephx4Unknown, score: 0.12
GatcUnknown, score: 0.31
Trim56Unknown, score: 0
Cyp2t4Unknown, score: 0.29
Irs2Unknown, score: 0.29
Frmd7Unknown, score: 0.21
Nxpe3Unknown, score: 0.37
Lca5lUnknown, score: 0.13
Zfp174Unknown, score: 0.2
Thoc6Unknown, score: 0.16
Nsfl1cUnknown, score: 0.03
Eid2Unknown, score: 0.12
Tas2r135Unknown, score: 0.27
Mettl7a2Unknown, score: 0.08
Ugt1a6bUnknown, score: 0.15
Flrt1Unknown, score: 0.06
Map4k5Unknown, score: 0.54
Btbd6Unknown, score: 0.49
BC052040Unknown, score: 0.01
Tmsb15lUnknown, score: 0.22
Ccdc87Unknown, score: 0.3
Opa3Unknown, score: 0.29
H2afy2Unknown, score: 0.14
Iqgap3Unknown, score: 0.39
Panx2Unknown, score: 0.37
Ndufs6Unknown, score: 0.06
Ndufa4l2Unknown, score: 0.77
Ecm2Unknown, score: 0.35
Znrf3Unknown, score: 0.18
Tmem204Unknown, score: 0.27
PrimpolUnknown, score: 0.08
BC049352Unknown, score: 0.16
Zfp873Unknown, score: 0.16
Zfp456Unknown, score: 0.22
Zfp874bUnknown, score: 0.11
Tnip3Unknown, score: 0
Gja6Unknown, score: 0.01
Cfap43Unknown, score: 0.17
Neurl2Unknown, score: 0.19
Zfp457Unknown, score: 0.52
Akap7Unknown, score: 0.17
GnptabUnknown, score: 0.38
Myo1aUnknown, score: 0.4
Adcy1Unknown, score: 0.16
Gm5431Unknown, score: 0.1
E130309D14RikUnknown, score: 0.1
Dnaic2Unknown, score: 0.1
432725Unknown, score: 0.11
432768Unknown, score: 0.64
Gm28557Unknown, score: 0.41
Lrrc14bUnknown, score: 0.4
432798Unknown, score: 0.13
432800Unknown, score: 0.1
Gm5464Unknown, score: 0.17
432879Unknown, score: 0.31
Rpl30Unknown, score: 0.05
Plcxd2Unknown, score: 0.25
Vmn2r96Unknown, score: 0.19
Pnpla1Unknown, score: 0.03
Eno1bUnknown, score: 0.34
Rbm10Unknown, score: 0.06
Sgpp2Unknown, score: 0.12
Teddm1bUnknown, score: 0.14
AA467197Unknown, score: 0.37
I830077J02RikUnknown, score: 0.07
Ankrd13cUnknown, score: 0.05
Akirin2Unknown, score: 0.03
Ncbp1Unknown, score: 0.04
433745Unknown, score: 0.27
Trim63Unknown, score: 0.33
Minos1Unknown, score: 0.41
Gm13251Unknown, score: 0.34
Pusl1Unknown, score: 0.18
433874Unknown, score: 0.31
PiggUnknown, score: 0.02
Dnaaf5Unknown, score: 0.04
Ccdc8Unknown, score: 0.02
Eid2bUnknown, score: 0.18
434175Unknown, score: 0.57
434219Unknown, score: 0.02
IqckUnknown, score: 0.13
Tmem221Unknown, score: 0.08
AmtUnknown, score: 0.23
Sp140Unknown, score: 0.11
Gm10220Unknown, score: 0.16
Akap14Unknown, score: 0.2
Rhox8Unknown, score: 0.11
434782Unknown, score: 0.01
Ldoc1Unknown, score: 0.12
Xlr4aUnknown, score: 0.19
434843Unknown, score: 0.05
Atp6ap1lUnknown, score: 0.18
Adgrf2Unknown, score: 0.08
ShfUnknown, score: 0.21
Nup85Unknown, score: 0.11
Xrra1Unknown, score: 0.05
Cbx6Unknown, score: 0.23
Apcdd1Unknown, score: 0.09
Zdhhc18Unknown, score: 0.22
Cfap74Unknown, score: 0.4
Hbq1bUnknown, score: 0.06
Zkscan4Unknown, score: 0.23
544944Unknown, score: 0.26
Iqgap2Unknown, score: 0.18
Wdfy4Unknown, score: 0.06
Wdr70Unknown, score: 0.03
Hnrnpa1Unknown, score: 0.1
Gal3st3Unknown, score: 0.37
Cfhr2Unknown, score: 0.13
Hmcn1Unknown, score: 0.15
BC094916Unknown, score: 0.37
545423Unknown, score: 0.35
545459Unknown, score: 0.06
Tubb1Unknown, score: 0.4
545531Unknown, score: 0.22
Ankrd34aUnknown, score: 0.39
545578Unknown, score: 0.58
545600Unknown, score: 0.31
Ptpn3Unknown, score: 0.21
Gm13290Unknown, score: 0.21
Gm13289Unknown, score: 0.21
Gm13277Unknown, score: 0.21
Gm13276Unknown, score: 0.21
Gm13279Unknown, score: 0.07
Fam159aUnknown, score: 0.1
Mansc4Unknown, score: 0.15
Zfp607Unknown, score: 0.15
CrxosUnknown, score: 0.07
C330021F23RikUnknown, score: 0.4
Mast3Unknown, score: 0.12
Wdr72Unknown, score: 0.47
546663Unknown, score: 0.03
Trim43aUnknown, score: 0.06
Zc3h12bUnknown, score: 0.17
Cxx1bUnknown, score: 0.04
Fam196bUnknown, score: 0.18
Xlr3bUnknown, score: 0.17
Gsk3aUnknown, score: 0.12
Rfx8Unknown, score: 0.13
Tmem253Unknown, score: 0.04
Tnfsfm13Unknown, score: 0.1
Rpl34Unknown, score: 0.23
Gm6086Unknown, score: 0.84
Zcchc17Unknown, score: -0.01
Rpl30Unknown, score: 0.05
619973Unknown, score: 0.06
620499Unknown, score: 0.04
Tmem28Unknown, score: 0.08
Ttc30a2Unknown, score: 0.02
620750Unknown, score: 0.01
Gm12185Unknown, score: 0.22
AI429214Unknown, score: 0.09
AC087559.2Unknown, score: 0.18
Aldh3b2Unknown, score: 0.04
Cldn20Unknown, score: 0.04
Psme2Unknown, score: 0.28
Tmem170bUnknown, score: 0.25
Rhox2hUnknown, score: 0.03
Kctd21Unknown, score: 0.55
622335Unknown, score: 0
Ccdc107Unknown, score: 0.48
1700123I01RikUnknown, score: 0.05
Ccdc17Unknown, score: 0.21
Gm6377Unknown, score: 0.14
Pydc4Unknown, score: 0.1
623286Unknown, score: 0.25
Lipt1Unknown, score: 0.1
Gm6484Unknown, score: 0.05
Speer4eUnknown, score: 0.24
Cks1brtUnknown, score: 0.35
Lekr1Unknown, score: 0.35
Slc38a6Unknown, score: 0.07
Rpl30Unknown, score: 0.01
Wdr93Unknown, score: 0.08
Rgs22Unknown, score: 0.06
Gm14322Unknown, score: 0.62
Etohi1Unknown, score: 0.09
Gm11992Unknown, score: 0.19
Fam196aUnknown, score: 0.32
627375Unknown, score: 0.08
627901Unknown, score: 0.1
628061Unknown, score: 0.02
Fbxo39Unknown, score: 0.1
Gm14420Unknown, score: 0.23
Gm6904Unknown, score: 0.2
Phf11cUnknown, score: 0.15
628746Unknown, score: 0.04
Hs3st4Unknown, score: 0.23
Serpina3iUnknown, score: 0.18
Fam124aUnknown, score: 0
Ctxn3Unknown, score: 0.21
Mroh8Unknown, score: 0.36
629595Unknown, score: 0.41
Cd101Unknown, score: 0.39
2010315B03RikUnknown, score: 0.1
630855Unknown, score: 0.01
631287Unknown, score: 0.45
631966Unknown, score: 0.23
632329Unknown, score: 0.09
632465Unknown, score: 0.12
Erich4Unknown, score: 0.21
RerglUnknown, score: 0.27
Rbm46Unknown, score: 0.28
634339Unknown, score: 0.18
634555Unknown, score: 0.09
Usp51Unknown, score: 0.4
635470Unknown, score: 0.1
Gm7168Unknown, score: 0.1
635999Unknown, score: 0.48
Vmn2r3Unknown, score: -0.01
637553Unknown, score: 0.07
637657Unknown, score: 0.05
639606Unknown, score: 0.14
Skint7Unknown, score: 0.16
639905Unknown, score: 0.09
640524Unknown, score: 0.1
640611Unknown, score: 0.25
640972Unknown, score: 0.22
641387Unknown, score: 0.29
Zfp784Unknown, score: 0.34
Smco3Unknown, score: 0.12
Ankrd37Unknown, score: 0.27
CtcflUnknown, score: 0.1
Gm7356Unknown, score: 0.1
Gm7356Unknown, score: 0
Rpl30Unknown, score: 0.36
Isoc2aUnknown, score: 0.53
Gm14326Unknown, score: 0.03
Col6a5Unknown, score: 0.11
Sec14l5Unknown, score: 0.38
Clec2lUnknown, score: 0.04
665181Unknown, score: 0.53
Plb1Unknown, score: 0.17
665562Unknown, score: 0.24
665611Unknown, score: 0.25
Trabd2bUnknown, score: 0.04
Frmpd1Unknown, score: 0.15
Vps13bUnknown, score: 0.05
Zscan25Unknown, score: 0.16
Apol7eUnknown, score: 0.3
Tomm5Unknown, score: 0.02
666448Unknown, score: 0.18
Atg4aUnknown, score: 0.02
666501Unknown, score: 0.12
666513Unknown, score: 0.37
Gm13139Unknown, score: 0.02
Rpl29Unknown, score: 0.4
666648Unknown, score: 0.14
Samd1Unknown, score: 0.15
Trim43cUnknown, score: 0.02
Trim43bUnknown, score: 0.02
Rbm24Unknown, score: 0.03
Gm8369Unknown, score: 0.46
667035Unknown, score: 0.07
667253Unknown, score: 0.08
H60bUnknown, score: 0.16
I830012O16RikUnknown, score: 0.16
Gm14446Unknown, score: 0.03
667384Unknown, score: 0.09
Hist1h2alUnknown, score: 0.34
667739Unknown, score: 0.01
Piezo2Unknown, score: 0.39
Myh15Unknown, score: 0.58
667952Unknown, score: 0.37
Gm8909Unknown, score: 0.7
Gm14305Unknown, score: 0.1
Mettl7a3Unknown, score: 0.21
Gm13288Unknown, score: 0.2
Kif26aUnknown, score: 0.16
668459Unknown, score: 0.06
Zfp507Unknown, score: 0.35
668548Unknown, score: 0.08
2410002F23RikUnknown, score: 0.28
668894Unknown, score: 0.37
671641Unknown, score: 0.17
SmsUnknown, score: 0.02
Rnf213Unknown, score: 0.74
674321Unknown, score: 0.2
675521Unknown, score: -0.01
675851Unknown, score: 0.05
676710Unknown, score: 0.08
Cyp4f37Unknown, score: 0.08
Sap25Unknown, score: 0.14
TomtUnknown, score: 0
Zglp1Unknown, score: 0.24
Mfap1bUnknown, score: 0.01
Tmsb15b2Unknown, score: 0.32
Sirpb1cUnknown, score: 0.29
Gm13305Unknown, score: 0.31
Ccl19Unknown, score: 0.1
Gm2026Unknown, score: 0.05
1810037I17RikUnknown, score: 0.36
Plac9aUnknown, score: 0.29
Plac9bUnknown, score: 0.47
Tmem254bUnknown, score: 0.14
100039503Unknown, score: 0.14
Hrct1Unknown, score: 0.23
100039794Unknown, score: -0.01
Ildr2Unknown, score: 0.45
Tgtp2Unknown, score: 0.04
100039826Unknown, score: 0.1
Gm12942Unknown, score: 0.07
100040052Unknown, score: 0.17
100040260Unknown, score: 0.17
100040416Unknown, score: 0.35
MndalUnknown, score: 0.14
100040500Unknown, score: 0.44
Dynlt1fUnknown, score: 0.44
Gm15319Unknown, score: 0.17
FancfUnknown, score: 0.19
Gm2897Unknown, score: 0.34
100040697Unknown, score: 0.06
Mroh2aUnknown, score: 0.19
Cyp4a32Unknown, score: 0.21
Rpl17Unknown, score: 0.39
100040961Unknown, score: 0.69
Tceal7Unknown, score: 0.24
Gm3194Unknown, score: 0.23
G630090E17RikUnknown, score: 0.7
Gm15448Unknown, score: 0.69
Gm3194Unknown, score: 0.01
Hist1h4mUnknown, score: 0.05
Supt4aUnknown, score: 0.17
Gm13242Unknown, score: 0.03
Gm13247Unknown, score: 0.02
100041478Unknown, score: 0.24
Ly6c2Unknown, score: 0.12
AC087559.2Unknown, score: 0.15
Amd2Unknown, score: 0.37
ErmardUnknown, score: 0.44
Mup7Unknown, score: 0.26
100041680Unknown, score: 0.21
Gm10408Unknown, score: 0.31
Gm3558Unknown, score: 0.17
CsprsUnknown, score: 0.25
100042069Unknown, score: 0.06
Nrg2Unknown, score: 0.38
BC005561Unknown, score: 0.27
Gm10639Unknown, score: 0.07
100042335Unknown, score: 0.39
Ube2l3Unknown, score: 0.02
Pdcd5Unknown, score: 0.05
Nhsl2Unknown, score: 0.05
Gm13305Unknown, score: -0.01
100042561Unknown, score: 0.35
100042773Unknown, score: 0.07
Eif3j2Unknown, score: 0.17
100042880Unknown, score: 0.27
100043002Unknown, score: -0.01
Gm11711Unknown, score: 0.02
100043184Unknown, score: 0.14
Zbed4Unknown, score: 0.07
100043257Unknown, score: 0
TigitUnknown, score: 0.09
Rpl21Unknown, score: 0.1
Gm14308Unknown, score: 0.7
Gng5Unknown, score: 0.1
100043718Unknown, score: 0.05
100043872Unknown, score: 0.1
Rpl34Unknown, score: 0.26
100043882Unknown, score: 0.1
Gm4724Unknown, score: 0
Ifi202bUnknown, score: 0.14
100044324Unknown, score: 0.24
100044391Unknown, score: 0.09
Tgfbr3lUnknown, score: 0.38
Rpl23Unknown, score: 0.03
100044729Unknown, score: 0.61
100044829Unknown, score: 0.21
100044900Unknown, score: 0.18
100045191Unknown, score: 0.05
100045326Unknown, score: 0.19
Ect2lUnknown, score: 0.24
100045848Unknown, score: 0.39
100045901Unknown, score: 0.2
100045999Unknown, score: 0.14
100046079Unknown, score: 0.14
100046151Unknown, score: -0.01
100046297Unknown, score: 0.39
100046899Unknown, score: 0.08
100047082Unknown, score: 0.27
100047252Unknown, score: 0.07
100047429Unknown, score: 0.04
100047577Unknown, score: 0.02
100047632Unknown, score: 0.5
100047658Unknown, score: 0.37
100048117Unknown, score: 0.1
100048119Unknown, score: 0.06
100048410Unknown, score: 0.61
100048499Unknown, score: 0.23
Cfap43Unknown, score: 0.33
Ddx43Unknown, score: 0.13
100048759Unknown, score: 0.44
Mup17Unknown, score: 0.12
Adat3Unknown, score: 0.16
100137011Unknown, score: 0.11
Ubl4Unknown, score: 0.24
Gm3194Unknown, score: 0.29
Mup19Unknown, score: 0.2
AK010878Unknown, score: 0.05
Gm17455Unknown, score: 0.37
Gm14431Unknown, score: 0.44
Dynlt1aUnknown, score: 0
Il4i1Unknown, score: 0.39
Zfp729bUnknown, score: 0.39
1700021K19RikUnknown, score: 0.05
Ccdc13Unknown, score: 0.09
100502897Unknown, score: 0.32
Btbd8Unknown, score: 0.1
100503217Unknown, score: 0.15
100503254Unknown, score: 0.1
Gm14440Unknown, score: 0.04
100503428Unknown, score: 0.17
CsprsUnknown, score: 0.09
Gm10354Unknown, score: 0.13
DosUnknown, score: 0.37
Rpl5Unknown, score: 0.12
Gm5901Unknown, score: 0.15
Ccdc149Unknown, score: 0.11
100503895Unknown, score: 0.17
CsprsUnknown, score: 0.2
Cmtm1Unknown, score: 0.3
100504174Unknown, score: 0.54
Ccdc170Unknown, score: 0.36
Dcdc2bUnknown, score: 0.01
100504500Unknown, score: 0.14
Mettl21bUnknown, score: 0.06
100504821Unknown, score: 0.03
100504863Unknown, score: 0.04
100505089Unknown, score: 0.16
100505154Unknown, score: 0.15
Schip1Unknown, score: 0.25
Gm20604Unknown, score: 0.04
Gm3411Unknown, score: 0.29
Gm13305Unknown, score: 0.01
100862012Unknown, score: 0.05
Loxl2Unknown, score: 0.15
AC087559.2Unknown, score: 0.05
Hmgb1Unknown, score: 0.06
Entpd4Unknown, score: 0.06
100862437Unknown, score: 0.11
Ftl1Unknown, score: 0.38
Gm5616Unknown, score: 0.36
100862473Unknown, score: 0.21
100862531Unknown, score: 0.12
100862558Unknown, score: 0.2
100862563Unknown, score: 0.02
100862578Unknown, score: 0.03
100862584Unknown, score: 0.28
100862587Unknown, score: 0.06
100862594Unknown, score: 0.14
100862597Unknown, score: 0.23
100862618Unknown, score: 0.35
100862620Unknown, score: 0.16
101055707Unknown, score: 0.14
101055716Unknown, score: 0.17
Sytl3Unknown, score: 0.09
101055738Unknown, score: 0.17
Sytl3Unknown, score: 0.7
101055764Unknown, score: -0.01
101055794Unknown, score: 0.46
101055802Unknown, score: 0.07
101055820Unknown, score: 0.01
101055854Unknown, score: 0.17
101055915Unknown, score: 0.1
101055925Unknown, score: 0.13
101055956Unknown, score: -0.01
101055995Unknown, score: 0.05
101056016Unknown, score: 0.11
101056061Unknown, score: 0.45
101056167Unknown, score: 0.16
101056341Unknown, score: 0
101056362Unknown, score: 0.08
101056365Unknown, score: 0.13
101056381Unknown, score: 0.11
101056386Unknown, score: 0.15
101056392Unknown, score: 0
101056447Unknown, score: 0.23
101056452Unknown, score: 0.36
101056460Unknown, score: 0.26
101056482Unknown, score: 0.3
101056500Unknown, score: 0.01
101056523Unknown, score: 0.09
101056544Unknown, score: 0.37
101056547Unknown, score: 0.01
101056558Unknown, score: 0.1
101056574Unknown, score: 0.16
101056577Unknown, score: 0.39
101056584Unknown, score: 0.18
101056603Unknown, score: 0
101056616Unknown, score: 0.17
101056619Unknown, score: 0.16
101056627Unknown, score: 0.27
101056654Unknown, score: 0.05
101056690Unknown, score: 0.11

## Help | Hide | Top Help | Show | Top Conditions

### HELP

Conditions in the module, given in the same order as on the expression
plot above. Red color means over-expression, green under-expression in
the given condition.

The barplot below shows the condition (sample) scores. A separate bar
is shown for each sample, its height is the corresponding score of the
sample in the module. The red and green numbers on the bars are the
sample scores expressed in percents, i.e. 100% is 1.0.

The red and green lines show the module thresholds, samples above
the red line and below the green line are included in the module.

The different experiments that were part of the study, are separated
by dashed vertical lines.

— Click on the *Help* button again to close this help window.

Id

SWR\_J-ISO\_136

NZB\_BLNJ-ISO\_91

NZB\_BLNJ-ISO\_90

NZB\_BLNJ-ISO\_96

LP\_J-ISO\_102

FVB\_NJ-ISO\_55

SJL\_J-ISO\_13

SWR\_J-CTR\_134

SJL\_J-ISO\_14

SWR\_J-ISO\_141

SWR\_J-ISO\_142

FVB\_NJ-ISO\_58

NOD\_ShiLtJ-ISO\_26

SWR\_J-CTR\_137

PL\_J-ISO\_73

SWR\_J-CTR\_138

NZB\_BLNJ-CTR\_92

LP\_J-ISO\_99

FVB\_NJ-CTR\_56

NZB\_BLNJ-ATE\_89

NOD\_ShiLtJ-ISO\_24

SWR\_J-ATE\_135

SJL\_J-ISO\_12

LP\_J-ISO\_103

PL\_J-ISO\_78

SWR\_J-ATE\_140

NZB\_BLNJ-CTR\_93

NZB\_BLNJ-ATE\_94

SJL\_J-CTR\_15

SJL\_J-ATE\_11

NZB\_BLNJ-CTR\_87

NZB\_BLNJ-ATE\_88

SWR\_J-ATE\_139

NOD\_ShiLtJ-ISO\_25

PL\_J-ISO\_77

SJL\_J-ATE\_17

PL\_J-CTR\_74

FVB\_NJ-ISO\_54

SJL\_J-CTR\_10

SJL\_J-ATE\_18

C57BL\_6J-ISO\_50

A\_J-ISO\_118

DBA\_2J-ISO\_4

LP\_J-ATE\_105

C57BLKS\_J-ISO\_115

SJL\_J-CTR\_16

PL\_J-CTR\_72

FVB\_NJ-ATE\_57

FVB\_NJ-ATE\_60

C57BL\_6J-ISO\_47

PL\_J-ATE\_71

C57BLKS\_J-ISO\_114

I\_LnJ-ISO\_149

C57BL\_6J-ISO\_59

C3H\_HeJ-ISO\_62

SM\_J-ISO\_44

NOD\_ShiLtJ-CTR\_19

C57BL\_6J-CTR\_35

LP\_J-CTR\_100

A\_J-ISO\_123

C58\_J-ISO\_31

C57BL\_6J-CTR\_38

I\_LnJ-ISO\_152

I\_LnJ-ISO\_155

C57BLKS\_J-CTR\_107

PL\_J-ATE\_76

LP\_J-ATE\_98

SM\_J-ISO\_45

BALB\_cJ-ISO\_81

C3H\_HeJ-ISO\_68

C58\_J-ISO\_33

A\_J-ISO\_124

LP\_J-CTR\_97

LP\_J-ATE\_101

LP\_J-CTR\_104

C58\_J-CTR\_28

C58\_J-ISO\_37

NOD\_ShiLtJ-ATE\_27

PL\_J-ATE\_75

SM\_J-ISO\_46

FVB\_NJ-CTR\_52

PL\_J-CTR\_70

C3H\_HeJ-ISO\_69

FVB\_NJ-CTR\_51

NOD\_ShiLtJ-CTR\_21

FVB\_NJ-ATE\_53

C58\_J-ATE\_29

I\_LnJ-CTR\_150

NOD\_ShiLtJ-CTR\_20

A\_J-CTR\_119

C58\_J-ATE\_30

C57BLKS\_J-CTR\_111

A\_J-CTR\_121

BALB\_cByJ-ISO\_156

DBA\_2J-ATE\_3

I\_LnJ-CTR\_147

NOD\_ShiLtJ-ATE\_22

BALB\_cByJ-ISO\_145

C3H\_HeJ-CTR\_65

A\_J-CTR\_120

I\_LnJ-ATE\_146

C57BL\_6J-CTR\_39

A\_J-ATE\_122

C57BLKS\_J-ATE\_112

C57BL\_6J-ATE\_109

A\_J-ATE\_116

A\_J-ATE\_117

SM\_J-CTR\_48

I\_LnJ-ATE\_148

NOD\_ShiLtJ-ATE\_23

C3H\_HeJ-CTR\_64

DBA\_2J-ISO\_7

I\_LnJ-CTR\_153

SM\_J-ATE\_49

C58\_J-CTR\_32

C3H\_HeJ-CTR\_63

SM\_J-ATE\_42

C3H\_HeJ-ATE\_66

SM\_J-CTR\_41

C58\_J-CTR\_34

C58\_J-ATE\_36

DBA\_2J-ISO\_8

C3H\_HeJ-ATE\_61

BALB\_cByJ-ISO\_159

SM\_J-ATE\_43

C57BLKS\_J-ATE\_108

I\_LnJ-ATE\_154

C57BLKS\_J-CTR\_110

C57BLKS\_J-ATE\_113

BALB\_cJ-CTR\_79

BALB\_cByJ-ATE\_160

BALB\_cJ-ATE\_80

SM\_J-CTR\_40

CBA\_J-ISO\_128

DBA\_2J-CTR\_2

C3H\_HeJ-ATE\_67

DBA\_2J-CTR\_1

CBA\_J-ISO\_129

DBA\_2J-CTR\_9

DBA\_2J-ATE\_5

DBA\_2J-ATE\_6

BALB\_cByJ-ATE\_151

BALB\_cByJ-CTR\_158

CBA\_J-CTR\_125

BALB\_cByJ-CTR\_143

CBA\_J-ATE\_131

CBA\_J-CTR\_126

CBA\_J-ISO\_133

CBA\_J-ATE\_127

CBA\_J-ATE\_132

C57BL\_6J-ATE\_106

BALB\_cJ-ISO\_83

BALB\_cByJ-ATE\_157

BALB\_cJ-ISO\_84

CBA\_J-CTR\_130

C57BL\_6J-ATE\_95

BALB\_cJ-ATE\_82

BALB\_cJ-CTR\_85

BALB\_cJ-ATE\_86

BALB\_cByJ-CTR\_144

© 2015 Computational Biology Group, Department of Medical Genetics,
University of Lausanne, Switzerland
