# Supplementary material for: RNAseq analysis of heart tissue from mice treated with atenolol and isoproterenol reveals a reciprocal transcriptional response
Source: BMC Genomics. 2016 Sep 7;17(1):717. doi: 10.1186/s12864-016-3059-6 (PMC5015234; doi:10.1186/s12864-016-3059-6)
Supplement: Additional file 4: — Supplementary data. Compressed HTML files of 98 expression modules annotated for genes, strains and GO or KEGG terms (see Additional file 3 for navigation details). (GZ 11006 kb) [file 12864_2016_3059_MOESM4_ESM.gz › modules.html/module-47.html]

Module #47, TG: 3, TC: 0.75, 55 probes, 55 Entrez genes, 58 conditions

# Previous module | Next module Module #47, TG: 3, TC: 0.75, 55 probes, 55 Entrez genes, 58 conditions

- Module tree/table

- Expression data

- The BP GO tree
- The CC GO tree
- The MF GO tree

- GO BP enrichment
- GO CC enrichment
- GO MF enrichment
- KEGG enrichment
- miRNA enrichment

- Genes
- Conditions

## Help | Hide | Top Help | Show | Top Expression data

### HELP

The image plot shows the color-coded level of gene expression, for the
genes and conditions in a given transcription module. The genes are on
the horizontal, the conditions on the vertical axis.

The genes are ordered according to their ISA gene scores, similarly
the conditions are ordered according to their condition scores. The
score of a gene means the «degree of inclusion» in
the module: a high score gene is essential in the module.

Condition scores can also be negative, that means that the genes of
the module are all down-regulated in the condition. Here the absolute
value of the score gives the «degree of inclusion».

The plots above and beside the expression matrix show the gene scores
and condition scores, respectively.

Note that the plot is interactive, you can see the name of the gene
and condition under the mouse cursor.

The expression matrix was normalized to have mean zero and standard
deviation one for every gene separately across all conditions
(i.e. not just for the conditions in the module).

— Click on the *Help* button again to close this help window.

Gene:   
Condition:


Under-expression is coded with green,
over-expression with red color.

## Help | Hide | Top Help | Show | Top The GO tree — Biological processes

### HELP

This is one of three sections showing Gene Ontology enrichment of the
current module: in this case for **biological processes**.

The graph shows the hierarchy of the GO categories, their enrichment
for the current module is color coded, and the blue number beside the
category is the minus log ten p-value of the enrichment. (Calculated
using the standard hypergeometric test.) The color of the arrows code
«is a» (cyan) and «part of» relationships.

The tree was built the following way. First all GO terms with more
significant enrichment p-value than 0.05 were collected. Then all
paths from these terms to the root node of the GO tree were included
too. If a GO term is included more than once in the tree, then the
green numbers show 1) the id of the node, this makes it easier to find
other appereances of the term, and 2) the number of appearences.

Note that the same GO category might show up on the graph many
times. This is because the GO was «straightened» for this
graph, i.e. if there are more paths from a GO term to the root node of
the tree, all of them are included. The green numbers

Move the mouse cursor over the terms to get their definition. Clicking
on them takes you to the corresponding Gene Ontology web page.

If you cannot see a graph here at all, that means that there were no
significantly enriched GO categories, at the 0.05 level.

— Click on the *Help* button again to close this help window.

## Help | Hide | Top Help | Show | Top The GO tree — Cellular Components

### HELP

This is one of three sections showing Gene Ontology enrichment of the
current module: in this case for **cellular components**.

The graph shows the hierarchy of the GO categories, their enrichment
for the current module is color coded, and the blue number beside the
category is the minus log ten p-value of the enrichment. (Calculated
using the standard hypergeometric test.) The color of the arrows code
«is a» (cyan) and «part of» relationships.

The tree was built the following way. First all GO terms with more
significant enrichment p-value than 0.05 were collected. Then all
paths from these terms to the root node of the GO tree were included
too. If a GO term is included more than once in the tree, then the
green numbers show 1) the id of the node, this makes it easier to find
other appereances of the term, and 2) the number of appearences.

Note that the same GO category might show up on the graph many
times. This is because the GO was «straightened» for this
graph, i.e. if there are more paths from a GO term to the root node of
the tree, all of them are included. The green numbers

Move the mouse cursor over the terms to get their definition. Clicking
on them takes you to the corresponding Gene Ontology web page.

If you cannot see a graph here at all, that means that there were no
significantly enriched GO categories, at the 0.05 level.

— Click on the *Help* button again to close this help window.

## Help | Hide | Top Help | Show | Top The GO tree — Molecular Function

### HELP

This is one of three sections showing Gene Ontology enrichment of the
current module: in this case for **molecular function**.

The graph shows the hierarchy of the GO categories, their enrichment
for the current module is color coded, and the blue number beside the
category is the minus log ten p-value of the enrichment. (Calculated
using the standard hypergeometric test.) The color of the arrows code
«is a» (cyan) and «part of» relationships.

The tree was built the following way. First all GO terms with more
significant enrichment p-value than 0.05 were collected. Then all
paths from these terms to the root node of the GO tree were included
too. If a GO term is included more than once in the tree, then the
green numbers show 1) the id of the node, this makes it easier to find
other appereances of the term, and 2) the number of appearences.

Note that the same GO category might show up on the graph many
times. This is because the GO was «straightened» for this
graph, i.e. if there are more paths from a GO term to the root node of
the tree, all of them are included. The green numbers

Move the mouse cursor over the terms to get their definition. Clicking
on them takes you to the corresponding Gene Ontology web page.

If you cannot see a graph here at all, that means that there were no
significantly enriched GO categories, at the 0.05 level.

— Click on the *Help* button again to close this help window.

## Help | Hide | Top Help | Show | Top GO BP test for over-representation

### HELP

List of all enriched GO categories (biological processes), at the 0.05
p-value level.

The columns:

- **ExpCount** is the expected count of genes in the
  module annotated with the given GO term, just by chance.
- **Count**
  is the number of genes in the module annotated with the given GO
  term.
- **Size** is the total number of genes (in our universe)
  annotated with the GO term.

Clicking on **Count** shows the genes that drive the
enrichment. You can also click on the individual numbers in
the **Count** column, to show the driving genes for that individual
GO category.

Clicking on the GO identifiers takes you to the Gene Ontology web
pages.

— Click on the *Help* button again to close this help window.

No enriched terms

## Help | Hide | Top Help | Show | Top GO CC test for over-representation

### HELP

List of all enriched GO categories (cellular components), at the 0.05
p-value level.

The columns:

- **ExpCount** is the expected count of genes in the
  module annotated with the given GO term, just by chance.
- **Count**
  is the number of genes in the module annotated with the given GO
  term.
- **Size** is the total number of genes (in our universe)
  annotated with the GO term.

Clicking on **Count** shows the genes that drive the
enrichment. You can also click on the individual numbers in
the **Count** column, to show the driving genes for that individual
GO category.

Clicking on the GO identifiers takes you to the Gene Ontology web
pages.

— Click on the *Help* button again to close this help window.

No enriched terms

## Help | Hide | Top Help | Show | Top GO MF test for over-representation

### HELP

List of all enriched GO categories (molecular function), at the 0.05
p-value level.

The columns:

- **ExpCount** is the expected count of genes in the
  module annotated with the given GO term, just by chance.
- **Count**
  is the number of genes in the module annotated with the given GO
  term.
- **Size** is the total number of genes (in our universe)
  annotated with the GO term.

Clicking on **Count** shows the genes that drive the
enrichment. You can also click on the individual numbers in
the **Count** column, to show the driving genes for that individual
GO category.

Clicking on the GO identifiers takes you to the Gene Ontology web
pages.

— Click on the *Help* button again to close this help window.

| Id | Pvalue | ExpCount | Count | Size | Term |
| --- | --- | --- | --- | --- | --- |
| GO:0042277 | 4.009e-02 | 0.5318 | 5 Ace2, Calr, Hcrtr1, Itm2c, Nfkbia | 166 | peptide binding |
| GO:0033218 | 4.708e-02 | 0.551 | 5 Ace2, Calr, Hcrtr1, Itm2c, Nfkbia | 172 | amide binding |

## Help | Hide | Top Help | Show | Top KEGG Pathway test for over-representation

### HELP

List of all enriched KEGG pathways, at the 0.05
p-value level.

The columns:

- **ExpCount** is the expected count of genes in the
  module annotated with the given KEGG pathway, just by chance.
- **Count**
  is the number of genes in the module annotated with the given KEGG
  pathway.
- **Size** is the total number of genes (in our universe)
  annotated with the KEGG pathway.

Clicking on **Count** shows the genes that drive the
enrichment. You can also click on the individual numbers in
the **Count** column, to show the driving genes for that individual
KEGG pathway.

Clicking on the KEGG identifiers takes you to the KEGG web site.

— Click on the *Help* button again to close this help window.

No enriched terms


### HELP

List of all enriched miRNA families, at the 0.05
p-value level.

The columns:

- **ExpCount** is the expected count of genes in the
  module regulated by the given miRNA family, just by chance.
- **Count**
  is the number of genes in the module regulated by the given miRNA
  family.
- **Size** is the total number of genes (in our universe)
  regulated with the given miRNA family.

Clicking on **Count** shows the genes that drive the
enrichment. You can also click on the individual numbers in
the **Count** column, to show the driving genes for that individual
miRNA family.

The miRNA regulation data was taken from the

Top


### HELP

p-value level.

The columns:

- **ExpCount** is the expected number of genes in the- **Count**- **Size** is the total number of genes (in our universe)

Clicking on **Count** shows the genes that drive the
enrichment. You can also click on the individual numbers in
the **Count** column, to show the driving genes for that individual

— Click on the *Help* button again to close this help window.

## Help | Hide | Top Help | Show | Top Genes

### HELP

A list of all genes in the current module, in alphabetical order. The
size of the text corresponds to the gene scores.

Note that some gene symbols may show up more than once, if many
probes match the same Entrez gene.

Genes with no Entrez mapping are given separately, with their
Affymetrics probe ID.

— Click on the *Help* button again to close this help window.

### Genes Symbol

, score:

Bag1Unknown, score: 0.83
CalrUnknown, score: 0.7
Clca3a1Unknown, score: 0.87
Dyrk1bUnknown, score: 0.76
Slc6a12Unknown, score: 0.71
Gap43Unknown, score: 0.94
GnalUnknown, score: 0.88
Il13ra1Unknown, score: 0.86
NfkbiaUnknown, score: 0.8
Sipa1Unknown, score: 0.92
Cntn6Unknown, score: 0.85
Dclre1aUnknown, score: 0.77
Sumf1Unknown, score: 0.8
Itm2cUnknown, score: 0.98
Cxx1aUnknown, score: 0.89
Fis1Unknown, score: 1
Slc25a23Unknown, score: 0.8
Thap4Unknown, score: 0.75
Kirrel3Unknown, score: 0.94
EqtnUnknown, score: 0.86
Nsmce4aUnknown, score: 0.84
Rnf122Unknown, score: 0.73
Rpap1Unknown, score: 0.76
Nup43Unknown, score: 0.76
Ace2Unknown, score: 0.93
P2ry12Unknown, score: 0.96
Ccpg1Unknown, score: 0.74
1110017D15RikUnknown, score: 0.76
Wdr54Unknown, score: 0.87
Ppp1r11Unknown, score: 0.74
Lsm8Unknown, score: 0.75
Arid2Unknown, score: 0.71
Setdb1Unknown, score: 0.84
Qsox1Unknown, score: 0.73
Gpsm3Unknown, score: 0.78
PkdccUnknown, score: 0.71
Psat1Unknown, score: 0.72
Utp18Unknown, score: 0.93
IqccUnknown, score: 0.73
Hcrtr1Unknown, score: 0.75
Grhl3Unknown, score: 0.72
Ssh1Unknown, score: 0.79
H2afjUnknown, score: 0.86
Sorbs2Unknown, score: 0.94
Wdr24Unknown, score: 0.83
Slc4a11Unknown, score: 0.88
Nup98Unknown, score: 0.8
433748Unknown, score: 0.72
Fam222aUnknown, score: 0.71
Gm2026Unknown, score: 0.94
100042023Unknown, score: 0.94
100048005Unknown, score: 0.94
Gm3194Unknown, score: 0.76
101056294Unknown, score: 0.94
101056654Unknown, score: 0.88

## Help | Hide | Top Help | Show | Top Conditions

### HELP

Conditions in the module, given in the same order as on the expression
plot above. Red color means over-expression, green under-expression in
the given condition.

The barplot below shows the condition (sample) scores. A separate bar
is shown for each sample, its height is the corresponding score of the
sample in the module. The red and green numbers on the bars are the
sample scores expressed in percents, i.e. 100% is 1.0.

The red and green lines show the module thresholds, samples above
the red line and below the green line are included in the module.

The different experiments that were part of the study, are separated
by dashed vertical lines.

— Click on the *Help* button again to close this help window.

Id

BALB\_cJ-ISO\_83

BALB\_cJ-ISO\_84

LP\_J-CTR\_100

BALB\_cJ-CTR\_85

BALB\_cJ-ATE\_82

BALB\_cJ-CTR\_79

NOD\_ShiLtJ-ISO\_24

LP\_J-ISO\_99

BALB\_cJ-ATE\_86

BALB\_cByJ-ISO\_145

BALB\_cByJ-CTR\_144

LP\_J-CTR\_97

LP\_J-ISO\_103

BALB\_cJ-ATE\_80

LP\_J-CTR\_104

BALB\_cByJ-CTR\_143

BALB\_cJ-ISO\_81

LP\_J-ATE\_105

LP\_J-ISO\_102

SJL\_J-ISO\_12

BALB\_cByJ-ISO\_159

SJL\_J-ISO\_14

BALB\_cByJ-ATE\_151

NOD\_ShiLtJ-ISO\_26

NOD\_ShiLtJ-ISO\_25

NOD\_ShiLtJ-CTR\_20

SJL\_J-ISO\_13

NOD\_ShiLtJ-CTR\_21

SJL\_J-CTR\_16

NOD\_ShiLtJ-ATE\_22

BALB\_cByJ-ISO\_156

C3H\_HeJ-ISO\_68

SWR\_J-ATE\_139

LP\_J-ATE\_101

DBA\_2J-ATE\_6

C57BL\_6J-CTR\_39

DBA\_2J-CTR\_9

C57BL\_6J-ATE\_109

C57BL\_6J-CTR\_38

C57BL\_6J-CTR\_35

SM\_J-ATE\_49

SM\_J-ISO\_44

SM\_J-ISO\_45

SM\_J-ATE\_43

SM\_J-ISO\_46

SM\_J-CTR\_40

SM\_J-ATE\_42

SM\_J-CTR\_48

SM\_J-CTR\_41

NZB\_BLNJ-ISO\_91

NZB\_BLNJ-ATE\_89

NZB\_BLNJ-ISO\_90

NZB\_BLNJ-ISO\_96

NZB\_BLNJ-ATE\_88

NZB\_BLNJ-CTR\_87

NZB\_BLNJ-CTR\_92

NZB\_BLNJ-ATE\_94

NZB\_BLNJ-CTR\_93

© 2015 Computational Biology Group, Department of Medical Genetics,
University of Lausanne, Switzerland
